# Projected health workforce requirements and shortage for addressing the disease burden in the WHO Africa Region, 2022–2030: a needs-based modelling study

**DOI:** 10.1136/bmjgh-2024-015972

**Published:** 2024-10-22

**Authors:** James Avoka Asamani, Kouadjo San Boris Bediakon, Mathieu Boniol, Joseph Kyalo Munga’tu, Francis Abande Akugri, Learnmore Lisa Muvango, Esther Diana Zziwa Bayiga, Christmal Dela Christmals, Sunny Okoroafor, Maritza Titus, Regina Titi-Ofei, Benard Gotora, Bernard Nkala, Adwoa Twumwaah Twum-Barimah, Jean Bernard Moussound, Richmond Sowah, Hillary Kipruto, Solyana Ngusbrhan Kidane, Benson Droti, Geoffrey Bisorborwa, Adam Ahmat, Ogochukwu Chukwujekwu, Joseph Waogodo Cabore, Kasonde Mwinga, The Expert Working Group on Health Workforce Needs Analysis, Joël Makadi Kombe

**Affiliations:** 1 Health Workforce Unit, Universal Health Coverage Life - Course Cluster, World Health Organization Regional Office for Africa, Brazzaville, Congo; 2 Centre for Health Professions Education, North-West University - Potchefstroom, Potchefstroom, South Africa; 3 Health Workforce, World Health Organization, Geneva, Switzerland; 4 Acurial Science Department, Jomo Kenyatta University of Agriculture and Technology, Nairobi, Kenya; 5 School of Nursing and Midwifery, University of Development Studies, Tamale, Ghana; 6 Internal Medicine, United Bulawayo Hospitals, Bulawayo, Zimbabwe; 7 School of Public Health, Makerere University, Kampala, Uganda; 8 Health Finance Department, The Global Fund to Fight AIDS Tuberculosis and Malaria, Grand-Saconnex, Switzerland; 9 Health Service Commission, Zimbabwe, Harare, Zimbabwe; 10 Ghana Country Office, World Health Organization, Accra, Ghana; 11 Human Resource Development Division, Ghana Health Service, Accra, Ghana; 12 Health Information Systems, World Health Organization Regional Office for Africa, Brazzaville, Congo; 13 Data Analytics and Knowledge Management, World Health Organization Regional Office for Africa, Brazzaville, Congo; 14 Child and Adolescent Health, World Health Organization Regional Office for Africa, Brazzaville, Congo; 15 Health Financing and Investment Unit, Universal Health Coverage - Life Course, World Health Organization Regional Office for Africa, Brazzaville, Congo; 16 World Health Organization Regional Office for Africa, Brazzaville, Congo; 17 Universal Health Coverage - Life Course, World Health Organization Regional Office for Africa, Brazzaville, Congo

**Keywords:** Health policies and all other topics, Health economics, Health services research, Health systems, Public Health

## Abstract

**ABSTRACT:**

**Introduction:**

An adequate health workforce (HWF) is essential to achieving the targets of the Sustainable Development Goals (SDG), including universal health coverage. However, weak HWF planning and constrained fiscal space for health, among other factors in the WHO Africa Region, has consistently resulted in underinvestment in HWF development, shortages of the HWF at the frontlines of service delivery and unemployment of qualified and trained health workers. This is further compounded by the ever-evolving disease burden and reduced access to essential health services along the continuum of health promotion, disease prevention, diagnostics, curative care, rehabilitation and palliative care.

**Methods:**

A stock and flow model based on HWF stock in 2022, age structure, graduation and migration was conducted to project the available stock by 2030. To estimate the gap between the projected stock and the need, a population needs-based modelling was conducted to forecast the HWF needs by 2030. These estimations were conducted for all 47 countries in the WHO African Region. Combining the stock projection and needs-based estimation, the modelling framework included the stock of health workers, the population’s need for health services, the need for health workers and gap analysis expressed as a needs-based shortage of health workers.

**Results:**

The needs-based requirement for health workers in Africa was estimated to be 9.75 million in 2022, with an expected 21% increase to 11.8 million by 2030. The available health workers in 2022 covered 43% of the needs-based requirements and are anticipated to improve to 49% by 2030 if the current trajectory of training and education outputs is maintained. An increase of at least 40% in the stock of health workers between 2022 and 2030 is anticipated, but this increase would still leave a needs-based shortage of 6.1 million workers by 2030. Considering only the SDG 3.c.1 tracer occupations (medical doctors, nurses, midwives, pharmacists and dentists), the projected needs-based shortage is 5.3 million by 2030. In sensitivity analysis, the needs-based shortage is most amenable to the prevalence of diseases/risk factors and professional standards for service delivery

**Conclusions:**

The WHO African Region would need to more than double its 2022 HWF stock if the growing population’s health needs are to be adequately addressed. The present analysis offers new prospects to better plan HWF efforts considering country-specific HWF structure, and the burden of disease.

**WHAT IS ALREADY KNOWN ON THIS TOPIC**

- An estimated 2.5 million avertable deaths occur every year in Africa, of which almost 48% are due to poor quality of healthcare while 52% are attributable to lack of access to health services.
- Quantitative shortfalls in the number of health workers and qualitative gaps in the competencies of health workers have been identified as some of the major challenges alongside inadequate infrastructure, equipment, medical products and financing, among others.
- The African Region is known to face the largest share of the global disease burden but has the smallest share of the global health workforce (HWF).
- Various studies estimated the HWF requirements for the African Region, but their inexplicit link with disease burden, population dynamics and essential packages of health service limited their acceptability and use as planning targets by countries.

**WHAT THIS STUDY ADDS**

- Using a needs-based approach, this paper modelled country-specific needs for health workers, taking into account disease burden, population along their life course and essential service package for service delivery.
- The needs-based requirements were compared with projected stock up to 2030.
- The needs-based requirement for health workers in the African Region was estimated to be 9.75 million in 2022, which is due to increase to 11.8 million by 2030.
- Communicable diseases contribute 47% of the need for health workers (on a downward trajectory), and non-communicable diseases contribute 37% (on an upward trajectory), while injuries contribute 16% (on an upward trajectory).
- The needs-based shortage is anticipated to be approximately 6.1 million by 2030 of which 87% or 5.3 million of the shortage will be for doctors, nurses, midwives, pharmacists and dentists.
- The available health workers in 2022 covered less than 43% of the needs-based requirements in 2022, which is anticipated to improve to 49% by 2030 if the current trajectory of training and education outputs are maintained.

**HOW THIS STUDY MIGHT AFFECT RESEARCH, PRACTICE OR POLICY**

- Countries in the WHO African Region would need to more than double their 2022 HWF stock to maximise the prospects of halving the shortage by 2030 and addressing the growing population’s health needs.
- However, countries are not on the same trajectory or at the same pace.
- Hence, nuanced adaptation at the country level is important and should be underpinned by national policy dialogues to engender policy action and investments.
- This study lays a solid foundation for further research in advancing a wider use of the needs-based approach to HWF planning and its integration into health labour market analysis and HWF planning.

## Introduction

 The 2021 Universal Health Coverage (UHC) monitoring report,^1^ revealed substantial progress in the global UHC Service Coverage Index (SCI) from 45 (on a scale from 0 to 100) in 2000 to 68 in 2021. These gains have, however, not been the same across all the Regions. The average score in 2021 was highest in the European Region and Region of the Americas, which were 80 and above, followed by the Western Pacific Region at 79, with the African Region being the lowest at 48. The African Region’s low UHC SCI suggests that only a fraction of the health needs of the population are being met with services of good quality and without an impoverishing effect on those who use them. Indeed, every year, an estimated 2.5 million avertable deaths occur in Africa, of which almost 48% are due to poor quality of healthcare while 52% are attributable to lack of access to health services.^2^ Quantitative shortfalls in the number of health workers and qualitative gaps in the competencies of health workers are some of the major challenges alongside inadequate infrastructure, equipment and medical products, among others.

Over the last two decades, the African Region made noticeable progress in health workforce (HWF) development with a remarkable increase in investments towards training and education infrastructure, increasing the number of health professions education institutions and/or programmes to more than 4000 in 2018 from less than 1000 in 2005.^3 4^ More than 400 institutions are now training doctors and dentists compared with 168 institutions in 2005.^5^ Similarly, there are more than 2200 nursing and midwifery schools compared with less than 1000 two decades ago.^6^ Consequently, the Region can train at least 255 000 health workers annually, with the annual training capacity for nurses and midwives increasing from 26 000 in 2005 to at least 151 300 in 2022. Furthermore, the annual training capacity of doctors also increased from about 6000 to almost 39 850 per year. Thus, between 2013 and 2020, access to the HWF increased from an average of 11 doctors, nurses, midwives, dentists and pharmacists per 10 000 people in 2013 to almost 27 per 10 000 by 2022.^7^,^10^

Nevertheless, the African Region continues to face a critical shortage of health workers, but the magnitude of the shortage has varied from one estimate to another depending on the methodology and quality of data used. A series of analyses covering the African Region used a threshold density of health workers below which a shortage is estimated. Depending on the occupations included and threshold used, these estimations varied: 5.3 million health workers by 2030,^9^ 6.1 million doctors, nurses, and midwives by 2030,^11 12^ to an estimation of 9.2 million doctors, nurses, midwives, dentists and pharmacists in 2019.^13^ The latter uses occupation-specific thresholds and a target of UHC ‘effective coverage’ of 80.

These approaches have been justified due to data limitations and the lack of tools to directly link population health needs and service delivery interventions to determine HWF needs-based requirements.^14^,^16^ However, these approaches consider a unique standard density as a reference and do not account for country-specific population health needs. As such, these ignore the tenets of universal health coverage, such as addressing disease burden and stratifying the population by life course demographics.

To address these limitations, a population needs-based approach to HWF planning has been increasingly used across the world.^17^,^23^ These models require more data and have been refined, adapted and used in Africa.^24^,^32^ The needs-based approach adopts a ‘right to health’ approach with the fundamental assumption being that whether a need is manifestly expressed by the population or not, any given population requires some health services (which is a means to regain or remain healthy). This approach has been applied in the health labour market analyses conducted in Ethiopia, Eswatini, Lesotho, Ghana, Kenya, Mozambique, Uganda, Zambia and Zimbabwe and has been demonstrated to be impactful in stimulating policy decisions and HWF investments in recent times.^2628 29 31^,^37^ The present study reports results from a needs-based modelling of the number and mix of health workers required to adequately address Africa’s disease burden in 2022 and by 2030, considering country-specific disease burden, population dynamics along the life course, package of services and incorporating expert opinion on professional standards of service delivery.

## Methods

### Conceptual and empirical framework

The population’s need for health services is estimated using the population’s size and demography, the prevalence of diseases and risk factors, and the type and frequency of health interventions planned or necessary to address the identified diseases, conditions and risk factors. The population’s need for health services can then be translated to HWF requirements if the professional standards of delivering the desired interventions can be reasonably agreed on, including workload division among health workers with overlapping or similar skills.^17 19 38 39^

The needs-based HWF requirement takes into consideration four main groups of parameters:

1. *Population (along their life course)*: this parameter considers the size of the population and its distribution along the life course (age cohorts), gender and geography (rural vs urban and by sub-national levels).
2. *Disease burden and level of health*: this parameter considers the prevalence/incidence of diseases, conditions, injuries and risk factors that account for at least 95% of mortalities and morbidities or the need for health service use, including the essential public health functions.
3. *Interdisciplinary health interventions along the life course*: interventions that are planned or are otherwise proven to be necessary to address the health needs (diseases, conditions, injuries and risk factors) along the life course and across the continuum of health promotion, disease prevention, diagnosis/detection, treatment/curative, rehabilitation and palliation.
4. *Interdisciplinary professional standards for service delivery and productivity*: the acceptable standard of delivering effective interventions, including with the aid of technology. This covers the frequency of service, the tools and medicines needed and the acceptable time it would take a reasonably trained health worker to deliver the intervention to professional standards.

**Figure 1:**
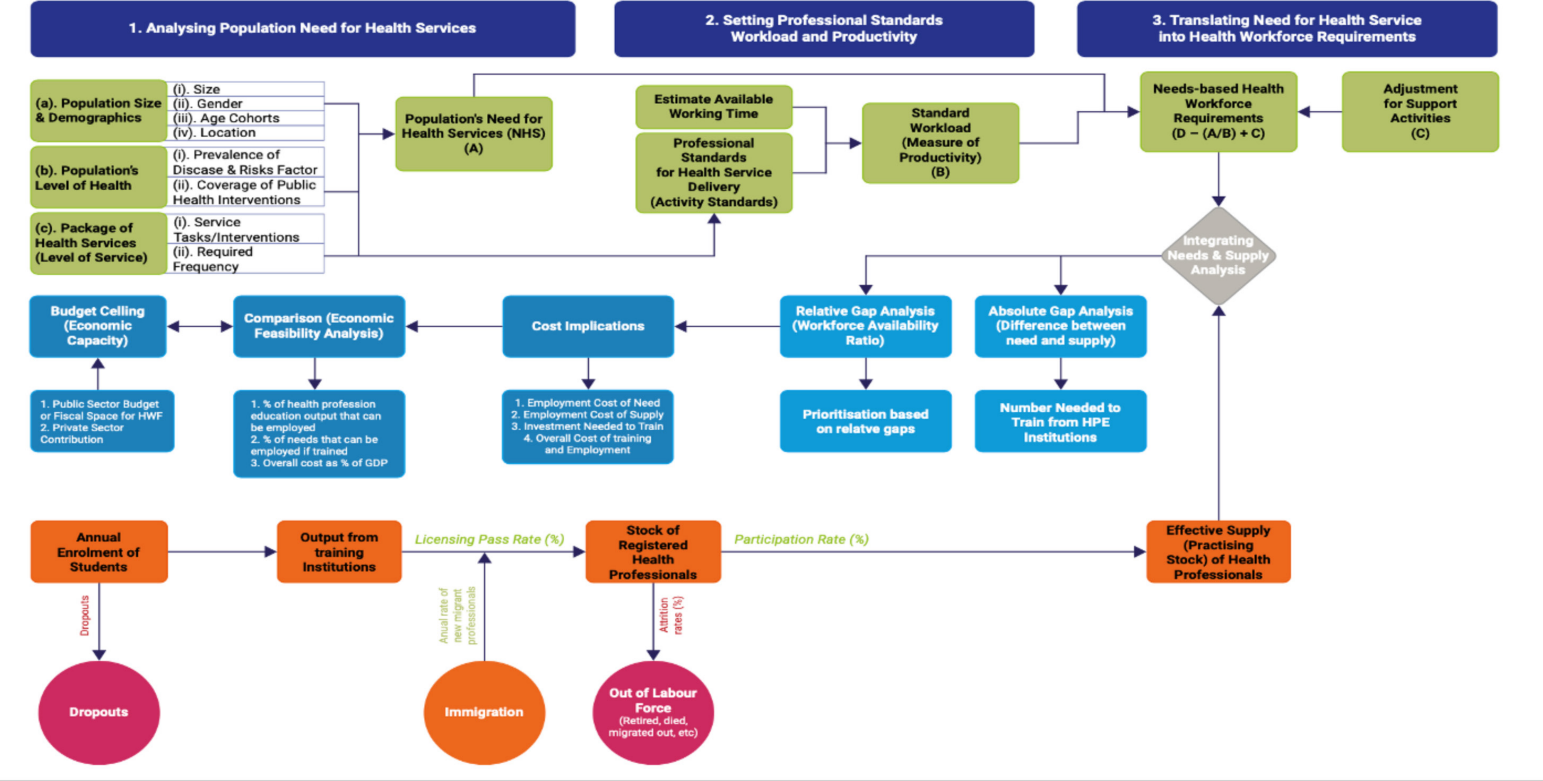
Framework for Need-based Health Workforce Planning.

The conceptual and analytical framework for the needs-based HWF requirement estimation presented in figure 1 has been described in detail in previous publications.^14 33 38 39^ In the context of health labour market analyses, this approach has been applied in nine countries in Africa,^24^,^2628 29 31 32 34^ and the concept has underpinned several HWF planning exercises in Australia, Canada, Jamaica, Saudi Arabia and the UK.^1516 19^,^22 40^ The framework comprises four inter-related estimations: (a) the supply of health workers, (b) the population’s need for health services, (c) the need for health workers and (d) gap analysis.

### Modelling population health needs for health professionals

#### Analysing the population’s need for health services (estimating the needs-based service requirements)

Based on previous work,^14 17 19 39 41^ three broad parameters are considered in estimating the need for health services, NHS: (a) the population (size, gender, age distribution along the life course and geographical location); (b) the level of health or health status (disease prevalence/incidence and risk factors) and (c) the services planned (or are otherwise necessary) to address the health deficits or to maintain optimal health. The relationship between these parameters determining the need for health services is adopted from Asamani *et al*,^38^ which is mathematically expressed as:


(1) $$NHS_{t}=\sum P_{i,j,t,g}\times \left[H_{h,i,j,t-1}\times \left(1+R_{h}\right)\right]\times L_{y,h,i,j,t}$$

Where:

- NHS_*t*_ represents the ‘Needed Health Services’ by a given over a period of time *t*.
- *P_i_*_,_*_j_*_,_*_g_*_,_*_t_* represents the size of the given population of age cohort *i*, gender *j* in location (rural or urban) *g* at time *t* in a given jurisdiction (this represents the population and its demographic characteristics).
- *H_h_*_,_*_i_*_,_*_j_*_,_*_g_*_,_*_t_* represents the proportion of the given population with health status *h*, of age cohort *i*, gender *j* in location *g* at time *t* (this represents the level of health of the population).
- *L_y_*_,_*_h_*_,_*_i_*_,_*_j_*_,_*_g_*_,_*_t_* represents the frequency of health services of type *y* planned or otherwise required (per unit of disease—being one individual of a particular age group, gender, location with the health status), under a specified service model, to address the needs of individuals of health status *h* among age cohort *i*, gender *j* in location *g* over time *t* (this represents the level of service required by the population).
- *R*_*h*_ is the instantaneous rate of change of the health status, *h*. This represents the rate of change in the prevalence rates of disease or coverage rates (as applicable).

#### Translating the evidence-based health service requirements into HWF requirements

To enable the conversion of need for health services to the corresponding needed number of health workers, explicit assumptions are warranted on a ‘workload standard’ or ‘measure of productivity’.^17^ This was done by leaning on the concept of standard workload (SW), as defined in the widely used and well-documented Workload Indicators of Staffing Need (WISN).^42^,^45^ The function encompasses two components: (a) the Service Standard, which is the average time that a well-trained and motivated health worker will spend to perform the service delivery activity to acceptable professional standards in the appropriate context and (b) the available working time (AWT)—the time a health worker has available in 1 year to do his/her work, taking into account all absences. Equations 2 and 3 illustrate these relationships.


(2) $${\text{SW}}_{n,y}=\frac{{\text{AWT}}_{n}}{{\text{SS}}_{y,n}}$$

Where:

- SW*_n_*_,_*_y_* is the standard workload for health workers of category *n* when performing health service activity *y*.
- AWT*_n_* is the available working time of the health worker of category *n.*
- SS*_y_*_,_*_n_* is the Service Standard or the time it takes a well-trained health worker of category *n* to deliver the service activity, *y*.

(3) $${AWT}_{n}=\left[A-\left(B+C+D+E\right)\right]\times F$$

- AWT*_n_* is the total available working time in a year for a health worker of category *n.*
- *A* is the number of possible working days in a year.
- *B* is the number of public holidays in a year.
- *C* is the number of annual leave days in a year.
- *D* is the average number of sick leave days a health worker takes in a year.
- *E* is the number of days off due to other leave, such as training in a year.
- *F* is the number of working hours in 1 day.

The model accounts for instances where an intervention can be performed by more than one type of health worker, known as the work division.^15^ The estimated need for health services derived in equation 1 is adjusted for the proportion of work division (which can be represented by *W*) to get the number of service activities *y* to be performed by a health worker of category *n* for individuals of health status *h*, age group *i*, gender *j* at location *g* over time *t*. The workload division adjusted need for health services can then be divided by the standard workload (defined in equation 2), as illustrated in equation 4.


(4) $$N_{n,t}=\sum \frac{\sum \left(P_{i,j,g,t}\times \left[H_{h,i,j,t-1}\times \left(1+R_{h}\right)\right]\times L_{y,i,j,t}\right)\times W_{y,n,h,i,j,t}}{{SW}_{n,y}}$$

- *N_n_*_,_*_t_* is the number of health workers of category *n* required to deliver a given service model *L_y_*_,_*_i_*_,_*_j_*, to a given population over a period of time *t.*
- *W_y_*_,_*_n_*_,_*_h_*_,_*_i_*_,_*_j_*_,_*_t_* is the proportion of services of type *y* to be performed by a health worker of category *n* for individuals of health status *h*, age cohort *i* and gender *j* at time *t*.

#### Adjusting the HWF requirements for catalytic or support activities of health workers

Aside from direct patient or direct person care, health workers perform other catalytic or indirect activities which are necessary for the quality and continuity of direct care interventions. Borrowing from WISN methodology,^46^ a Support Allowance Standard (SAS) is defined as the proportion of a health worker’s time that is spent on support activities. When the total SAS (in a proportion of total time) is subtracted from the whole, the difference represents a proportion of the health worker’s AWT that is devoted to direct patient/person services.^46^

### Modelling the stock and supply of the HWF

Computationally, the stock was modelled using a stock and flow process. For each occupation group, the projection of health workers up to 2030 used a simple stock and flow model over 8 years (between 2023 and 2030), which factors in the production and attrition of health workers. This modelling approach was initially applied in the State of the World’s Nursing 2020 and to a global estimation of shortage conducted in 2022.^9^ In this paper, the model was adapted to factor in out-migration for medical doctors and nurses as follows:


(5) $$\begin{array}{l} Health\;workforce\;stock_{2030} \end{array}\approx \begin{array}{l} \left({health\;workforce\;stock}_{2022}\times \;(1-\%\;{age}_{65+})\right)\\ \times \;(1-0.1\times \%\;{age}_{55-64}{)}^{\left(8-1\right)}+\;8\times 0.7\\ \times \;number\;of\;graduates\;annually-1/20\\ \times \;number\;of\;workers\;in\;a\;foreign\\ country\;(outmigration) \end{array}$$

To estimate individual year projections from 2023 to 2030, the value ‘8’ used in the formula was adapted to be 1 for 2023, 2 for 2024, … and 8 for 2030.

The use of the percentage of health workers older than 65 years was applied in the first projection year (2023), and later, the share of those aged 55–64 in the following years (2024–2030) was used to factor in attrition from 2023 to 2030. Since the age of retirement is commonly 65 years in many countries,^47^ the share of those aged 65 and above was considered as attrition on an annual basis. One-tenth of those aged 55–64 were considered as moving to the category 65+ in the preceding years after 2023.

Health systems’ absorption capacity of new graduates from health professional education was also factored into the modelling by adding in a fixed 70% absorption rate of the annual graduates.^9^ Data from 10 countries in the African Region that have conducted health labour market analyses between 2019 and 2023 show that 27% of graduates are unemployed, implying an average absorption capacity of about 70%. This estimate is similar to Organisation for Economic Cooperation and Development Stat,^48^ data by activity level, which shows an average ratio between practising and licensed to-practice health workers of around 70% for physicians (74%), nurses (70%), dentists (70%) and pharmacists (66%). The fixed 70% absorption capacity considered across all country income groups comes with the assumption that in each country, the education market would self-regulate relative to the availability of positions in the health labour market.

The out-migration factor used the National Health Workforce Accounts (NHWA) data on the number of medical doctors and nursing personnel reported as working in a foreign country. For medical doctors, these data correspond to the sum of foreign workers from each country of origin reported by 69 countries, and for nursing personnel, the sum reported by 63 countries. These data cover mostly high-income countries, including large economies in the African Region. However, some countries did not report the number of foreign workers by country of origin. Consequently, this number is an under-reported count of workers in a foreign country. Because this figure corresponds to the total number of workers, irrespective of the time of arrival in a foreign country, a factor of 1/20 was used to mimic the annual outflow from the country of origin. This factor was arrived at after the assumption that the out-migrated will work in the foreign country for a period of 20 years, on average.

A sensitivity analysis was conducted assuming a complete absorption capacity of countries to 100% of graduates. And another sensitivity analysis also projected health workers’ stock in the absence of migration patterns.

### Analysing mismatches between the need for and stock/supply of the HWF

A gap expressed as the absolute number of health workers missing was estimated for each occupation category and each period by subtracting the needs-based requirement *N*_*n*,*t*_ from the supply *S*_*n*,*t*_. Another metric was also used to assess the share of needs covered by the supply, dividing the supply *S*_*n*,*t*_ by the Need *N*_*n*,*t*_.

### Practical application of the needs-based framework

In implementing the above-mentioned framework for estimating the need for health workers, we applied the model using five steps.

#### Defining the essential diseases, conditions, injuries and risk factors for countries

From the WHO Health Observatory,^49^ and Global Burden of Disease Study,^50^ comprehensive data was extracted on diseases, risk factors and injuries and their contribution to disability-adjusted life years (DALYs) and mortalities in Africa. These were ranked based on the risk factors and their contribution to the DALYs. Diseases and risk factors that account for at least 95% of the DALYs in the countries were selected, and others that are of regional priority but not necessarily among the top contributors to DALYs were added. Comparable data on the prevalence of each disease, condition, injury type and risk factors were extracted for all the countries (online supplemental material 1). Comparing 2010 and 2019 data enabled estimation of the rate of change of disease burden.

#### Quantifying the population in need of health services

All the diseases, conditions, injuries, and risk factors were mapped to the most affected life course cohort(s), gender and geographical distribution (urban or rural). Using the prevalence rates and disaggregated population data from the UN population dataset (2022 revision),^51^ for all countries, the quantification of the population in need of health service was then computed using equation 1.

#### Defining essential interventions by occupation and certain professional standards for delivering the interventions

The WHO’s Regional Office for Africa (WHO AFRO) convened an expert group of 25 members known as the Technical Working Group on Health Workforce Need Estimation. The expert groups had representation across different expertise, including internal medicine, surgery, obstetrics and gynaecology, general practitioners, laboratory scientists, nurses, midwives, physiotherapy and rehabilitation, dentistry, pharmacy, non-communicable diseases, infectious diseases, injuries, maternal and child health and public health, and nutrition as well as statistics, and epidemiology.

##### Identification of essential interventions

The expert group reviewed data and reports from previous needs-based analyses conducted by countries, the WHO African Region’s Package of Essential Health Services, and the WHO UHC Compendium and integrated their expert experiences from the field in the African Region (see table 1 for data sources). A total of 450 interventions along the continuum of care were identified and defined by the expert working group—these are the context-appropriate interventions to address various diseases and risk factors in line with the training and competencies of various occupations (see online supplemental material 2).

**Table 1:** Summary of parameters and data sources

Dimension for model application	Parameter(s)	Data source(s)
Population	The size of the population Gender distribution Age composition (age cohorts) The distribution of the population in terms of regions, rural and urban locations	UN Population (2023) Health Labour Market Analysis Reports Country HRH profiles National HRH Strategic Plans Annual Reports of Professional Regulatory Councils
Level of health (or disease burden)	Prevalence or incidence of diseases and risk factors that constitute 95% of the burden of mortalities, outpatient attendance and hospital admissions Coverage rates of essential public health interventions	Institute of Health Metrics and Evaluation (IHME), 2023: The 2019 Global Burden of Disease Study—Prevalence data. Retrieved on 3 November 2023 from https://vizhub.healthdata.org/gbd-results/ WHO Global Health Observatory (2022). https://www.who.int/data/gho/data/indicators
Level of service	Essential Interventions to address the identified conditions, risk factors and injuries (accounting for 95% of DALYs)	WHO Africa Region Essential Health Package Tool Kit. *Integrated African Health Observatory (iAHO)—Database of Indicators*. 4 November 2023, from: https://aho.afro.who.int/essential-health-intervention/af WHO UHC Compendium (2023). https://www.who.int/universal-health-coverage/compendium WHO AFRO 2023: Output of Expert Working Group on Need Analysis (Unpublished data)
Professional standards for delivering the identified interventions	The main health interventions undertaken by health workers to address the disease burden The standard workload health worker per year is the quantity or volume of work (within one health service intervention) that one worker could perform in a year, assuming that worker is fully dedicating their working time to performing that intervention	Ahmat *et al*, 2022: https://doi.org/10.1136/bmjgh-2022-008456 Asamani *et al*, 2021: https://doi.org/10.3390/healthcare9030332 Asamani *et al*, 2022: https://doi.org/10.1371/journal.pone.0257957 Kunjamen, Okech *et al*, 2022: https://doi.org/10.1186/s12960-021-00671-3 WHO AFRO 2023: Output of Expert Working Group on Need Analysis (Unpublished data)
Workload division	Based on previous recommendations, the workload division of 70% for professional nurses (registered general nurses) and 30% for auxiliary nurses (enrolled nurses) were adopted. The clinical functions of midwives, community health nurses, nutritionists and dieticians are often not shared with other health workers, and so no assumptions were made about their workload division	Expert opinion from the Expert Working Group on Health Workforce Need Estimation (EWG HWF-NE) established the workload divisions based on a consensus process informed by their practical experiences, eight country applications, WISN reports and literature
Stock data	Current stock (2022) as reported through the National Health Workforce Account and WHO/AFRO Regional report on the State of Health Workforce in Africa Training pipeline output from the State of Health Workforce in Africa report Attrition rate from the State of Health Workforce in Africa report	National Health Workforce Account (NHWA) State of Health Workforce in Africa report^4^ HLMA country reports (2017–2023)

DALYs, disability-adjusted life years; HLMA, Health Labour Market Analysis; UHC, Universal Health Coverage; WISN, Workload Indicators of Staffing Need.

##### Setting professionally acceptable time standards for delivering the interventions

**Figure 2:**
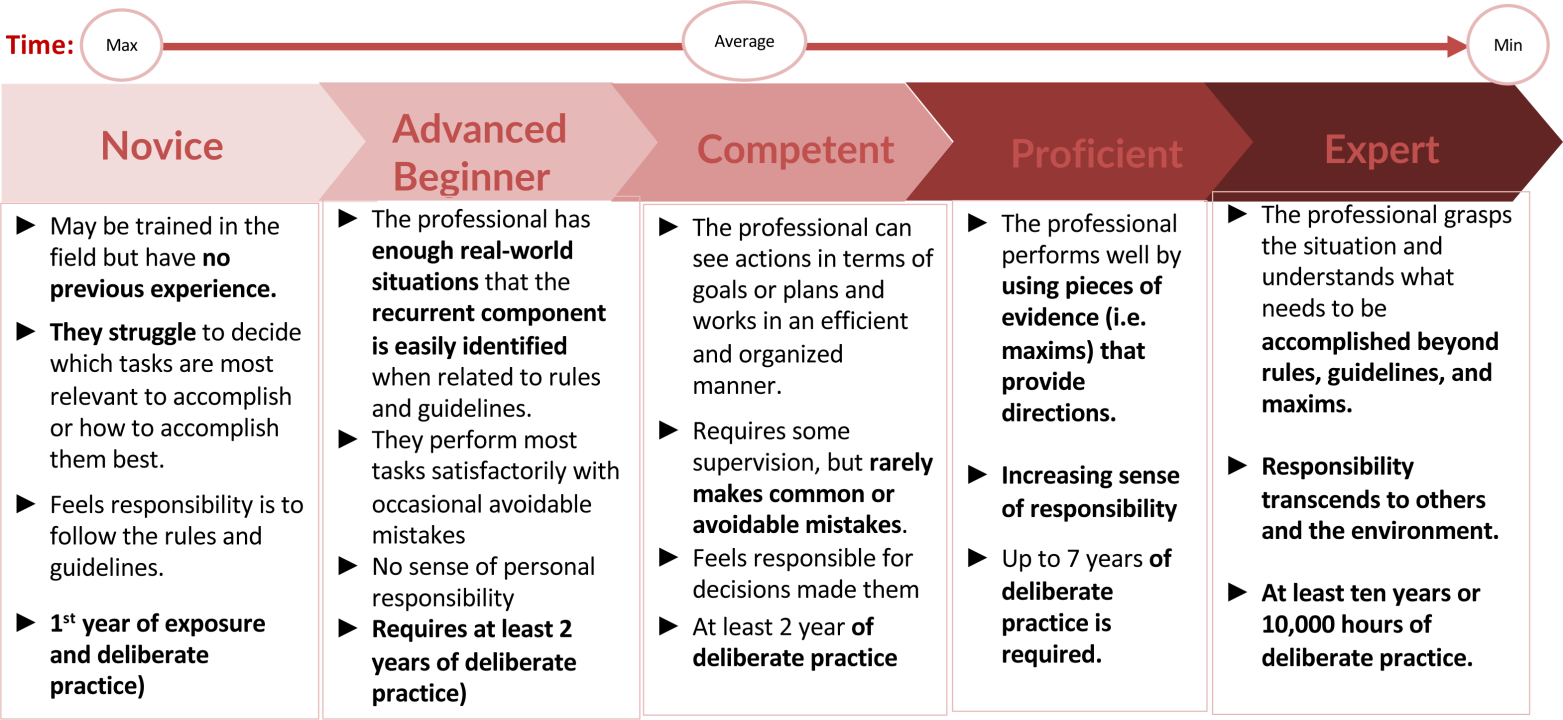
Application of the Benner's *From Novice to Expert* model for setting professional standards of health interventions.

For each of the interventions, the expert group built consensus on a plausible time range necessary to undertake the intervention to acceptable professional levels by applying Benner’s From Novice to Expert model.^52 53^ Expert-level health workers who are deemed more competent were assigned the minimum amount of time required to undertake an intervention. The maximum time was presumed to be the amount of time that it may take a novice practitioner to accomplish the same intervention successfully (as shown in figure 2). The average time was then assumed for the reasonably competent health worker (similar to the WISN methodology).

The expert group triangulated data from various sources, such as published literature on WISN activity standards,^54^,^56^ and the professional standards defined some of the country applications conducted between 2020 and 2022.^26 28 29 31 32 34 36^ The compiled interventions and professional standards were also mapped to the continuum of care, namely, health promotion, disease prevention, detection and diagnostics, curative services and disease management, rehabilitation and palliative care.

### Matching the essential interventions with the population in need to quantify the annual need for health interventions

For each of the diseases/risk factors or injuries, all the appropriate health worker professionals were selected across the continuum of care and all their relevant interventions for that particular disease/condition/risk factor/injury were listed and matched. Where the intervention to be performed is shared among different occupations, a workload division was made among the different health worker categories by assigning percentages. Also, the percentage of the affected population group who will need the intervention and the frequency they would require the intervention per year were assigned—drawn from guidelines or the expert opinion of the expert working group. The matching process yielded 2602 unique permutations between diseases/risk factors/injuries with occupation and their essential interventions.

### Estimating the need for health workers and sensitivity/scenario analysis

Computations of the needs-based HWF requirements were undertaken using an Excel-based analytic tool developed by WHO Regional Office for Africa.^57^ The tool produced estimates and scenarios of analysis per country, which were then aggregated and analysed to draw a regional picture. Two uncertain parameters were examined and varied simultaneously in scenario analyses (prevalence rates of the diseases, risk factors, and injuries, and the professional standards for delivering the interventions). From this, three main scenarios were analysed and reported:

1. *Base case scenario (medium estimate)*: the rate of change in the prevalence of diseases, risk factors and injuries between 2010 and 2019 was assumed to continue until 2030. This scenario uses the average prevalence rate of diseases, conditions, risk factors and injuries and applies an annual adjustment based on the observed rate of change. The average professional standards are also assumed in the base-case scenario.
2. *Minimum scenario*: in this scenario, the rate of change in the prevalence of diseases, conditions, risk factors and injuries between 2010 and 2019 was assumed to continue to 2030 and the lower confidence limits of the prevalence rate of diseases, risk factors and injuries with annual adjustments based on observed rate of change. The expert level of care that requires minimum time to deliver interventions is also assumed. As such, the minimum scenario reflects the situation where all the applicable variables take their lowest values.
3. *Maximum case scenario*: this scenario uses the combination of the upper confidence limits of the prevalence rate of diseases, conditions, risk factors and injuries with annual adjustments based on the observed rate of change and the novice level of care that requires a maximum amount of time to deliver the intervention. As such, the maximum scenario reflects the situation where all the applicable variables take their highest values.

### Triangulating data on the stock of health workers

Data were systematically combined from multiple sources, including the NHWA, Health Labour Market Analysis (HLMA), Country HRH profiles, HRH Strategic Plans and Annual Reports of Professional Regulatory Councils. The NHWA Dataset (2023 release) was downloaded, covering the period from 2012 to 2022. The dataset was assessed for completeness and pattern of trends, and it was concluded that there were several data gaps and breaks in trends. Hence, cross-sectional comparisons of 2018 and 2022 were used for the descriptive part of the analysis.

For 14 countries (30% of the WHO member states in the African Region), there were HLMA reports between 2019 and 2023, as well as HRH profiles or annual statistics reports from professional regulatory bodies. For these countries, wherever the NHWA data shows a break in trend or missing data points, the data from these other sources were used as a substitute.

### Data sources and assumptions for applying the model

Data were triangulated from multiple sources to apply the model. Table 1 provides details of the sources from which data was obtained and inputted into the Microsoft Excel-based model.^38 39^ Other assumptions underlying the model are also described in the separate publication.^39^

## Results

### Regional needs-based requirements for health workers by occupational group

The base estimate shows that considering the burden of disease, an essential package of health service interventions and professional standards for health service delivery, the overall need for health workers in Africa was estimated to be 9.7 million in 2022 and projected to increase by 21% to 11.8 million by 2030 (table 2). Of the estimated needs-based requirement, about 8 million (83%) are expected to be medical doctors, nurses, midwives, and community health workers.

**Table 2:** Estimates of needs-based health workforce requirements in the Africa Region, 2022–2030

Occupations	Base estimate			Minimum scenario			Maximum scenario		
	2022	2026	2030	2022	2026	2030	2022	2026	2030
Audiologists and speech therapists	16 583	18 359	20 411	11 391	12 604	14 002	23 138	25 618	28 462
Community health workers*	1 063 537	1 158 928	1 261 868	716 888	777 288	844 766	1 692 087	1 838 648	1 982 955
Dentists	83 099	92 344	103 858	53 890	59 885	67 365	141 124	156 805	176 316
Dieticians and nutritionists	163 569	172 819	181 833	123 260	130 090	136 730	237 504	251 046	264 247
Environmental and occupational health and hygiene workers	94 322	104 102	115 029	51 062	56 532	62 697	145 658	160 475	176 941
Generalist medical practitioners	485 407	535 842	592 278	330 272	363 848	401 464	683 188	754 167	832 788
Medical and dental prosthetic technicians	225 536	252 347	282 713	146 080	163 307	182 840	328 585	367 758	412 022
Medical and pathology laboratory scientists	149 959	166 809	187 327	102 113	113 114	126 622	215 414	239 479	268 278
Medical and pathology laboratory technicians	192 428	212 285	235 843	127 208	139 207	154 453	299 202	327 744	358 144
Medical imaging and therapeutic equipment technicians	89 420	101 007	115 371	60 794	68 538	78 104	135 797	153 689	176 091
Midwifery personnel	878 039	958 395	1 048 335	629 199	687 989	754 109	1 257 208	1 368 540	1 494 845
Nursing associate professionals	1 430 103	1 577 416	1 741 763	1 078 297	1 187 684	1 309 767	1 956 824	2 159 270	2 384 378
Nursing professionals	3 667 003	4 033 695	4 460 768	2 623 424	2 889 386	3 199 095	5 566 851	6 112 001	6 750 496
Optometrists and ophthalmic opticians	19 134	21 614	24 413	11 465	12 975	14 690	30 526	34 499	38 989
Paramedical practitioners	210 198	227 528	248 726	131 314	141 777	155 113	328 533	353 603	382 204
Pharmaceutical technicians and assistants	127 995	140 879	155 334	90 469	99 498	109 587	200 673	221 028	243 965
Pharmacists	160 052	171 780	185 286	108 003	115 792	124 653	232 801	249 945	269 951
Physiotherapists and physiotherapy assistants	30 139	33 509	37 403	20 749	23 062	25 717	43 377	48 269	53 946
Psychologists	76 365	83 876	92 643	54 795	60 142	66 364	97 484	107 149	118 459
Social workers	44 899	47 323	50 204	32 054	33 637	35 362	60 824	64 485	69 178
Specialist medical practitioners	540 817	605 867	683 566	358 023	400 396	450 718	786 886	883 071	998 539
Grand total	9 748 602	10 716 722	11 824 973	6 860 750	7 536 751	8 314 218	14 463 684	15 877 291	17 481 195

* The analysis assumed full-time work community health workers (CHWs). However, in practice, CHWs in most countries do part-time work. This should be taken into account or adjusted for when using the results to make decisions on CHWs.

In 2022, the Region required about 1 million medical doctors, and slightly less than half of them (47%, n=485 400) were needed to be generalist medical practitioners and the rest of the 53% (n=540 800) were expected to be specialist medical practitioners. The required number of doctors is projected to increase by 24% to 1.3 million by 2030, with 46% being generalist medical practitioners and 54% being specialist medical practitioners. Nearly 160 000 pharmacists were needed in 2022, and this number is projected to increase by 16% (n=185 000) in 2030. About 127 995 pharmaceutical technicians and assistants were needed to complement the work of the pharmacists, and their need is anticipated to increase by 21.4% to 155 334 by 2030. In addition, the Region needed at least 5.1 million nursing personnel, 880 000 midwifery personnel and 343 000 laboratory personnel in 2022. Online supplemental material 3 provides detailed country-by-country estimates for all health occupations considered in the analysis. The tool used in the need analysis is contained in supplementary material 4.

In scenario and sensitivity analysis, the minimum scenario, where the lower bounds of disease prevalence and minimum time requirements from health workers were assumed, the Region required 6.9 million health workers in 2022, which could increase to 8.3 million by 2030. The minimum scenario generally estimated an average of 42% lower requirements compared with the best estimates. In the maximum scenario, where escalated disease prevalence and maximum time requirement from health workers (assuming low competencies and/or lack of logistics to work), the Region required about 14.5 million health workers in 2022, which could increase to 17.5 million by 2030. Compared with the best estimate, this extreme scenario yields an average of 48% higher need for health workers.

**Figure 3:**
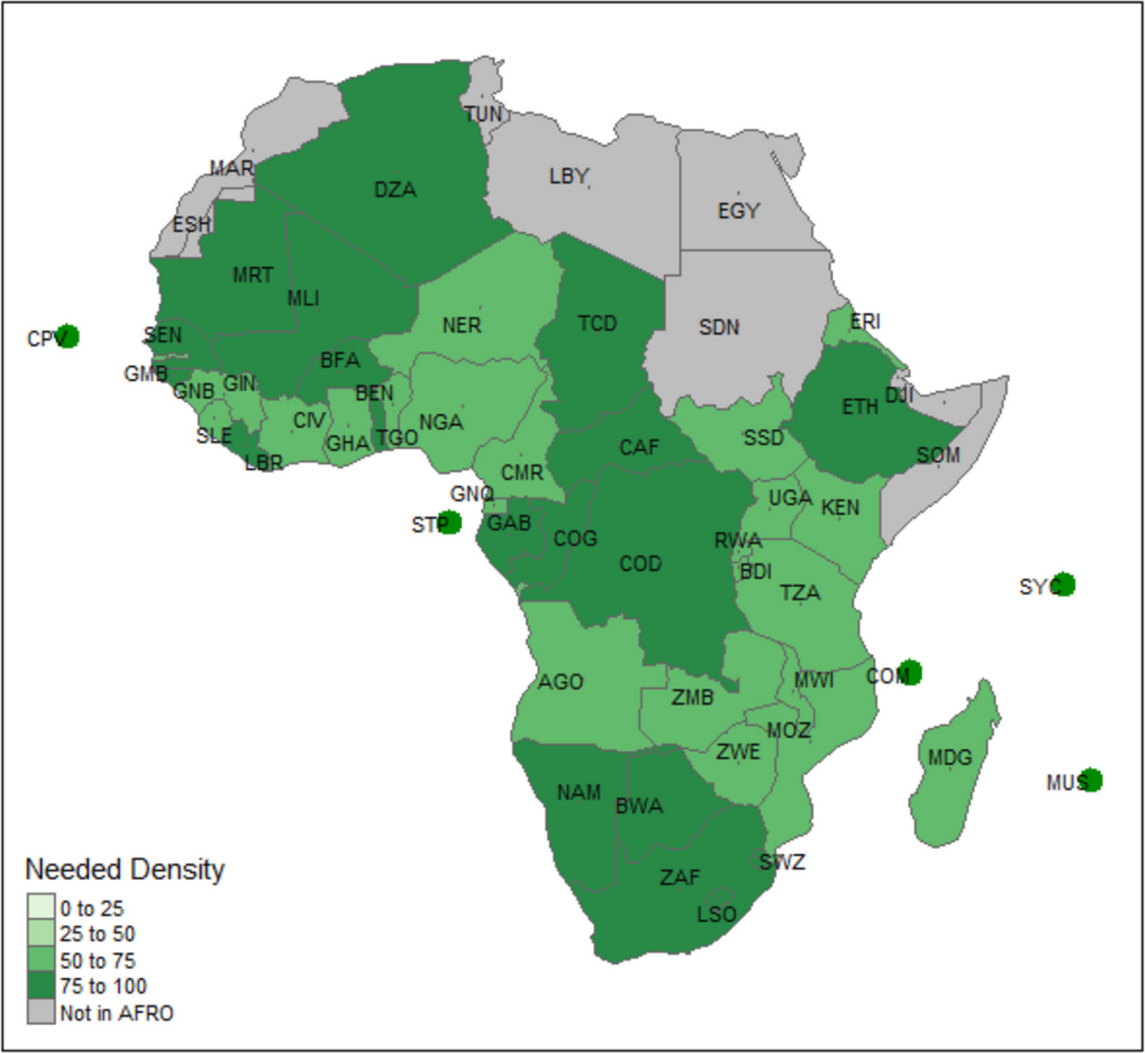
Density of Estimated HWF based on the Need-based Requirements (per 10,000 population).

Based on the disease burden and model of care, on average, countries needed 5.4 general medical practitioners per 10 000 population, ranging from 4.4 in Eritrea to 7.3 in Mauritius. In addition, an average of 6.45 medical specialists are required per 10 000 population, but this number ranges from 5.46 per 10 000 population in Chad to 12.36 per 10 000 population in Mauritius (figure 3 and table 3). Also, about 41 (range: 32–87) nursing professionals per 10 000 population are needed, alongside 9.2 (range: 7–14) midwifery personnel per 10 000 population and 1.6 (range: 1–4) pharmacists per 10 000 population. Furthermore, at least 4.5 laboratory personnel were required per 10 000 population.

**Table 3:** Estimated densities per 10 000 population of needed health workforce for selected occupations by country

Country	Dentists	Generalist medical practitioners	Medical and pathology laboratory scientists	Medical and pathology laboratory technicians	Midwifery personnel	Nursing associate professionals	Nursing professionals	Pharmacists	Specialist medical practitioners
Algeria	1.05	4.93	2.17	1.56	7.69	15.24	59.26	1.47	8.70
Angola	0.86	5.29	1.72	2.10	9.06	16.48	35.23	1.57	5.73
Benin	0.91	5.14	1.68	3.25	9.33	15.16	36.93	1.10	5.81
Botswana	1.15	5.68	2.38	1.86	8.20	17.45	46.33	1.63	7.45
Burkina Faso	0.82	5.01	1.55	2.80	13.25	17.10	35.97	1.14	6.25
Burundi	0.95	5.59	2.15	6.04	8.90	15.04	34.62	1.21	5.04
Cabo Verde	1.03	5.03	1.85	1.50	8.35	15.24	47.96	1.30	7.82
Cameroon	1.03	5.16	1.69	2.67	9.18	15.25	36.92	1.10	6.34
Central African Republic	1.02	5.79	1.93	2.32	10.73	17.37	44.75	2.16	5.99
Chad	0.88	4.86	1.36	1.57	10.00	15.17	44.83	1.14	5.46
Comoros	1.11	4.73	1.54	1.13	8.11	14.72	42.91	1.58	6.76
Congo	0.93	5.77	2.10	3.09	8.58	17.15	37.09	1.66	6.47
Côte d'Ivoire	0.95	5.08	1.52	1.75	8.85	15.20	36.90	1.43	6.02
Democratic Republic of the Congo	0.96	5.36	1.57	1.81	9.55	16.77	37.55	1.67	5.90
Equatorial Guinea	1.09	6.30	2.44	3.82	8.70	16.69	34.64	1.59	6.20
Eritrea	1.23	4.35	1.27	1.04	8.40	13.44	37.90	1.24	5.71
Eswatini	1.32	5.46	2.48	2.01	8.05	16.92	40.79	1.44	6.48
Ethiopia	0.90	5.15	1.31	1.33	8.57	13.90	52.40	2.55	5.20
Gabon	0.94	6.30	2.32	3.10	8.13	18.06	41.34	2.26	7.01
Gambia	1.02	4.69	1.27	1.28	9.65	13.58	33.69	1.00	5.73
Ghana	0.84	5.11	1.61	1.42	8.44	15.02	37.84	1.54	6.53
Guinea	0.91	4.96	1.58	2.76	10.67	13.44	32.26	1.20	5.69
Guinea-Bissau	1.10	4.96	1.35	1.13	9.03	14.81	45.43	1.52	5.68
Kenya	0.83	5.44	1.66	2.01	8.56	14.09	35.79	2.07	5.89
Lesotho	1.12	5.67	2.79	2.15	8.00	17.60	39.75	1.51	6.88
Liberia	1.05	6.32	2.12	5.42	10.36	15.98	36.36	2.18	6.11
Madagascar	1.23	4.63	1.40	1.39	8.70	12.98	31.74	1.34	6.03
Malawi	1.11	4.67	1.67	1.51	8.88	14.73	35.68	1.07	5.93
Mali	0.91	4.89	1.33	2.12	12.81	14.49	39.18	1.07	5.61
Mauritania	1.05	6.09	2.14	4.96	9.10	17.15	42.53	1.12	6.83
Mauritius	0.72	7.34	3.86	2.95	6.88	18.51	86.94	3.94	12.36
Mozambique	1.00	5.11	2.10	3.14	10.36	15.91	33.10	1.22	5.55
Namibia	1.04	6.89	2.78	5.46	8.27	17.47	39.80	2.23	6.93
Niger	0.89	5.09	1.41	2.01	9.97	15.47	36.73	1.14	5.59
Nigeria	0.78	5.41	1.42	1.78	9.28	15.03	35.09	1.74	5.78
Rwanda	1.06	5.72	2.19	5.24	9.23	14.30	33.72	1.35	6.58
Sao Tome and Principe	1.00	4.81	1.38	2.78	14.10	15.01	39.75	1.17	6.83
Senegal	1.14	4.76	1.34	1.39	10.09	18.77	46.56	1.14	5.87
Seychelles	0.68	6.51	3.18	2.15	7.02	16.85	68.38	2.61	10.49
Sierra Leone	1.02	5.04	1.59	2.55	9.46	14.66	34.76	1.26	5.86
South Africa	0.93	6.81	3.02	2.46	7.85	19.22	51.43	2.62	8.51
South Sudan	0.88	4.64	1.47	1.59	8.18	14.13	40.72	1.53	5.51
Togo	1.03	5.30	1.71	2.85	9.16	15.58	43.02	1.13	6.39
Uganda	0.84	5.16	1.76	3.04	8.61	16.76	33.06	1.63	5.55
United Republic of Tanzania	1.05	4.39	1.38	2.17	11.97	14.20	34.88	0.94	5.98
Zambia	0.94	5.13	1.86	2.05	8.75	16.59	36.24	1.21	5.77
Zimbabwe	0.95	4.74	1.84	1.56	9.03	15.91	36.76	0.99	6.39
**Regional average**	**0.98**	**5.35**	**1.88**	**2.47**	**9.23**	**15.76**	**40.97**	**1.55**	**6.45**

### Impact of evolving disease burden on needs-based HWF requirements

**Figure 4:**
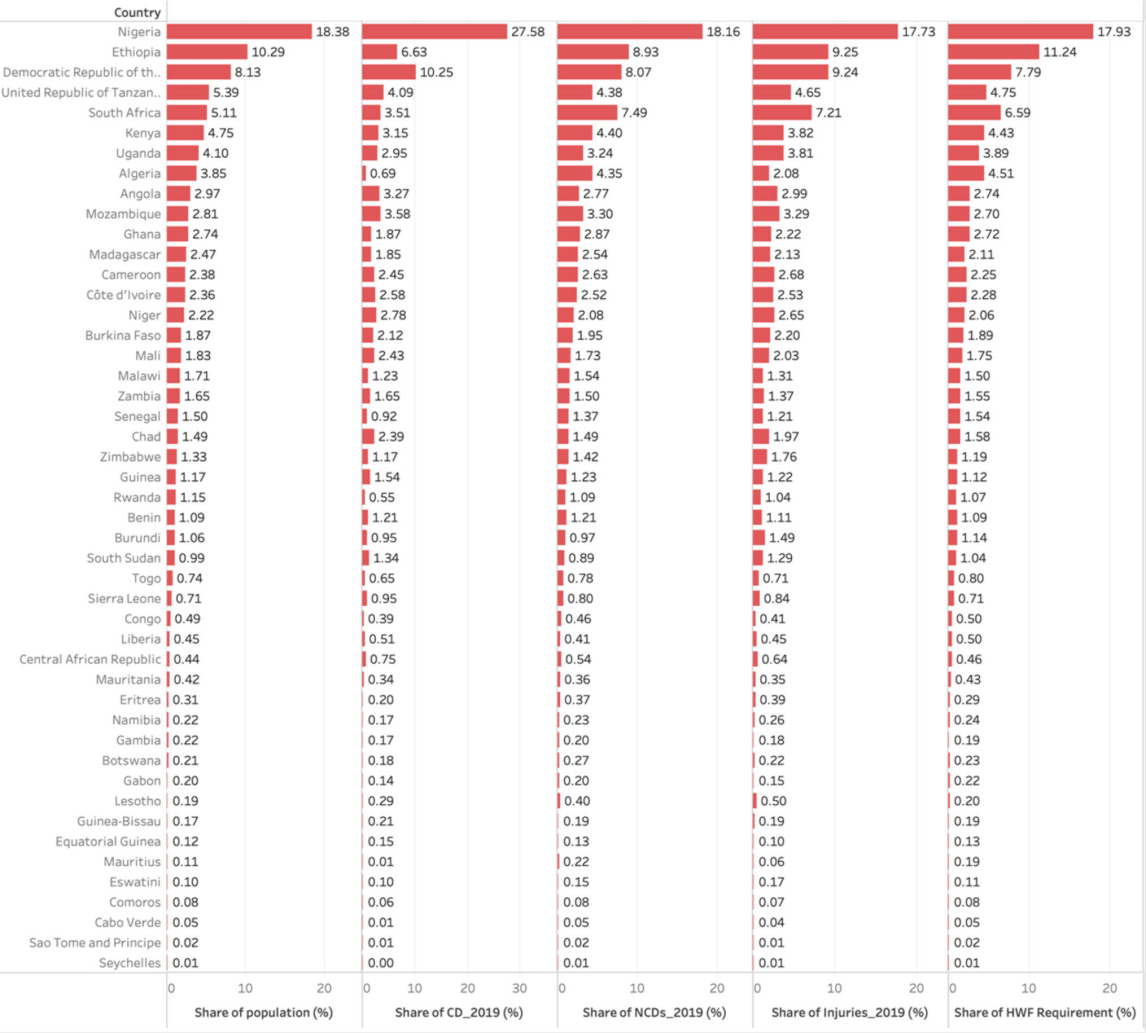
Share of Disease Burden and Population compared to share of HWF need-based requirements. CD, communicable disease; HWF, health workforce; NCD, non-communicable disease.

On average, the need for health workers is increasing at a 20% faster rate than the population growth rate due to the effect of the evolving disease burden. In a multiple linear regression analysis (see online supplemental material 5), using disease burden as measured by DALYs,^50^ non-communicable diseases, communicable diseases and injuries collectively account for 99.2% of the variations in the ‘need for health workers’ across the countries. If the other groups of diseases influencing the need were held constant, communicable diseases contributed to 47% of the requirements for health workers, but a trend that tended to decrease over time. Also, non-communicable diseases contributed 37% of the requirements for health workers if the other factors were held constant, a trend that tended to increase over time. Similarly, injuries contributed 16% of the requirements for health workers, and the trend tended to increase over time. Figure 4 illustrates the population growth rate compared with the anticipated increase in needs-based HWF requirements.

### Stock of the HWF in the Africa region

In the year 2022, there were 5.1 million practising health workers of any kind in the African Region, a significant improvement from 4.3 million in 2018 (table 4). Of the reported stock in 2022, nurses account for 33.3%, 16.7% are community health workers and 7.2% are medical doctors (table 4).

**Table 4:** Stock of health workers in the WHO African Region, 2018 and 2022

Occupation	Stock in 2018	Stock in 2022	Share of 2022 stock (including CHWs) (%)
Audiologists and speech therapists	3611	1247	0.02
Community health workers	681 651	850 462	16.7
Dental assistants and therapists	27 391	22 367	0.4
Dentists	37 007	34 404	0.7
Dieticians and nutritionists	10 593	28 074	0.6
Environmental and occupational health and hygiene workers	51 667	60 028	1.2
Managerial staff	31 336	65 901	1.3
Medical and dental prosthetic technicians	12 613	6484	0.1
Medical and pathology laboratory scientists	82 242	100 765	2.0
Medical and pathology laboratory technicians	377 113	125 216	2.5
Medical doctors	337 942	369 145	7.2
Generalist medical practitioners	244 831	274 393	5.4
Specialist medical practitioners	93 111	94 752	1.9
Medical imaging and therapeutic equipment technicians	30 579	43 796	0.9
Medical records and health information technicians	51 189	49 709	1.0
Midwifery personnel	219 661	334 530	6.6
Nursing personnel	1 254 023	1 698 828	33.3
Nursing associate professionals	474 406	636 953	12.5
Nursing professionals	779 617	1 061 876	20.8
Optometrists and ophthalmic opticians	13 026	10 866	0.2
Other non-medical professional staff	386 282	296 957	5.8
Other non-medical support staff	155 106	181 593	3.6
Paramedical practitioners	78 871	271 132	5.3
Medical assistants	42 017	50 851	1.0
Personal care workers in health service	124 980	126 433	2.5
Pharmaceutical technicians and assistants	31 717	48 644	1.0
Pharmacists	91 152	101 401	2.0
Physiotherapists and physiotherapy assistants	35 404	24 929	0.5
Psychologists	14 391	8004	0.2
Social workers	29 344	26 398	0.5
Traditional and complementary medicine practitioners	100 716	160 113	3.1
Total	4 311 624	5 098 278	100

Source: WHO AFRO (2024), National Health Workforce Accounts 2023 Release.

CHWs, community health workers.

For only occupations included in the needs-based projection, their stock of the HWF in the African Region is projected to increase by nearly 1.7 million from about 4.17 million in 2022 to almost 5.77 million by 2030, representing a 38% improvement in the headcount of practising stock (table 5). For the occupations tracked in the Sustainable Development Goals (SDG) 3.c.1 (medical doctors, nurses, midwives, dentists and pharmacists), the Region’s baseline stock of 2.5 million in 2022 is anticipated to increase by 18.2% to at least 3 million in 2026 and then to almost 3.5 million by 2030. Thus, an overall improvement of nearly 1 million doctors, nurses, midwives, pharmacists and dentists is anticipated, or a 37% increase in 8 years (see online supplemental material 6 for details of the projected stock).

**Table 5:** Projected practicing stock of health workers

Occupational category	Baseline (2022)	Projected 2026 stock	Projected 2030 stock	Anticipated % change
Medical doctors	369 145	437 935	515 890	40
Nursing personnel	1 698 828	1 951 363	2 212 905	30
Midwifery personnel	334 530	421 408	506 796	51
Pharmacist	101 401	147 485	187 436	85
Dentist	34 405	46 667	53 054	54
Other health workers*	1 628 411	1 935 201	2 290 343	41
**Overall**	**4 166 720**	**4 940 057**	**5 766 424**	**38**

* Seven other health occupations were not included in the need estimates.

Four countries, namely Seychelles, South Africa, Liberia and Kenya, are projected to experience reduced stock of practising health workers if business remains as usual, particularly for nurses and midwives. Furthermore, 13 countries are projected to increase their stock by less than 30% between 2022 and 2030, compared with an anticipated regional average of 38%. A total of 30 countries are on a relatively fast pace in their HWF growth, which are expected to increase by more than 30% between 2022 and 2030. In contrast, the growth rate for nurses is on a downward trajectory for six countries and slower than expected in 20 countries. Six countries appear to also be on a downward trajectory for doctors, 20 countries (43%) are on a slower-than-expected growth rate and 21 countries (45%) are anticipated to increase production by at least 40%.

### Needs-based HWF shortage in the African Region

As presented in table 6, there was a 5.6 million shortage of health workers in 2022 when the population health needs-based requirement of 9.75 million was compared with the reported stock of 4.2 million health workers for the occupations that were considered. The available stock of health workers covered approximately 43% of the need for health workers. However, when all uncertainties in disease burden and variations in professional practices and technologies are considered, the available stock covered only 29% of the maximum scenario of need and 61% if the minimum scenario of need is considered (table 7).

**Table 6:** Projected stock of health workers compared with needs-based requirements, 2022 and 2030

Occupation	Estimates for 2022				Estimates for 2030			
	Reported stock (a)	Estimated need (b)	Shortage (b–a)	HWF NAR (a/b×100) (%)	Projected stock (a)	Projected need (b)	Shortage (b–a)	HWF NAR (a/b×100) (%)
Medical doctors	369 145	1 026 224	657 079	36	515 890	1 275 843	759 953	40
Nursing personnel	1 698 828	5 097 106	3 398 278	33	2 212 905	6 202 531	3 989 626	36
Midwifery personnel	334 530	878 039	543 509	38	506 796	1 048 335	541 539	48
Pharmacist	101 401	160 052	58 650	63	187 436	185 286		
Dentist	34 404	83 099	48 695	41	53 054	103 858	50 804	51
Other health workers*	1 628 411	2 504 083	875 672	65	2 290 343	3 009 120	718 777	76
**Total**	4 166 720	9 748 602	5 581 882	**43**	5 766 424	11 824 973	6 060 699	**49**
**Doctors, nurses, midwives, dentists, pharmacists**	2 538 309	7 244 520	4 706 210	**35**	3 476 081	8 815 853	5 341 922	**39**

HWF NAR (calculated as the stock of health workers divided by the estimated need multiplied by 100).

* Seven other health occupations (with a reported headcount of 931 557, 18% of the entire workforce) were excluded from the stock projections because they were not included in the need estimates.

HWF NAR, health workforce need-availability ratio.

**Table 7:** Scenario analysis for tackling the needs-based shortage of health workers

Scenario	Needs-based shortage (all occupations)	% change in shortage from the base scenario	Absolute difference	Implication
70% absorption and maintain current training outputs (base case scenario)	6 060 699			
100% absorption of trained health workers—maintain current training outputs	5 219 267	−14	841 431	Ensuring all trained health workers are absorbed but maintaining current levels of training outputs would likely reduce the needs-based shortage in 2030 by 0.84 million or 14%
100% absorption and 50% increase in retention	5 158 134	−15	902 564	Ensuring 100% absorption and focusing on addressing migration without expanding training output will likely cut the 2030 needs-based shortage by 0.9 million
90% absorption and increase education output by 20%	5 003 986	−17	1 056 712	90% absorption and eliminating the drop-out rate of 20% will likely have a positive impact by reducing 1 million of the needs-based shortage
90% absorption and increase education output by 30%	4 756 107	−22	1 304 591	90% absorption and eliminating the drop-out rate of 20%, increasing the additional capacity of 10% will likely have a positive impact by reducing 1.3 million of the needs-based shortage
90% absorption and increase education output by 40%	4 508 228	−26	1 552 471	90% absorption and eliminating the drop-out rate of 20%, increasing the additional capacity of 20% will likely have a positive impact by reducing 1.5 million of the needs-based shortage
80% absorption and increase education output by 50%	4 678 537	−23	1 382 162	80% absorption and eliminating the drop-out rate of 20%, increasing the additional capacity of 30% will likely have a positive impact by reducing 1.4 million of the needs-based shortage
90% absorption and double education output	3 639 180	−40	2 421 518	Doubling the training output (by reducing losses and expanding capacity) and improving the rate of absorption of trained health to at least 90% from the current 70% can reduce the needs-based shortage by at least 40%

Specifically for specialist medical practitioners, the available stock in 2022 covered approximately 11.5% of the needs-based requirements while the stock of psychologists covered only 10.5%; nursing professionals covered 24.1%. The stock of midwives in 2022 covered approximately 38.1%; dentists covered 31.5% of the need for health workers to enable the provision of desired levels of health service interventions.

With a projected stock of 5.7 million by 2030 (of the occupations included in the stock and need projections) compared with the projected need for 11.8 million, the projected shortage of health workers is likely to be around 6.1 million by 2030. Thus, the projected stock would likely cover about 48% of the needs-based requirements in 2030.

Considering only the five tracer occupations in the SDG 3.c.1 monitoring indicator, just 35% of the need for medical doctors, nurses, midwives, dentists and pharmacists were covered in 2022 (2.52 million stock vs 7.25 million needs-based requirements). It is projected that by 2030, the stock of doctors, nurses, midwives, dentists and pharmacists will likely reach 3.48 million compared with a need for 8.82 million, leaving a needs-based shortage of 5.3 million by 2030. Thus, the projected stock would likely cover 39% of the need in 2030, but it varies from 36% for nurses to 51% for dentists.

### Scenario analysis for addressing the needs-based HWF shortage in 2030

Overall, any scenario involving increasing the absorption of all trained health workers to at least 90%, reducing losses from the training outputs and increasing training capacity by at least 20% would contribute to reducing but not eliminating the projected needs-based shortage by 2030 (table 7). In a scenario where all trained health workers are absorbed, but the current training outputs are maintained, it would likely reduce the needs-based shortage in 2030 by 0.84 million or 14%. In a scenario where all trained health workers are absorbed, out-migration is reduced by half, but the current training outputs are maintained; it would likely cut the 2030 needs-based shortage by 0.9 million (15%). Reducing the needs-based shortage by at least one quarter combines a situation where 90% of all trained health workers are absorbed, and education output losses of 20% are removed, increasing the additional capacity of 20%. This scenario would likely cut the needs-based shortage in 2030 by 1.5 million (26%). Most scenarios targeting higher production capacity without improving absorption tend to have a relatively low impact in reducing the shortage of health workers.

### Validating the ‘need for HWF’: relationship between the coverage of needs-based requirement and universal health coverage

To explore how sensitive the needs-based estimate of HWF will support the attainment of UHC, we compared the proportion of HWF needs (doctors, nurses, midwives, dentists and pharmacists) that are covered by existing stock in 2022 to the countries’ respective UHC SCI score in 2021.

**Figure 5:**
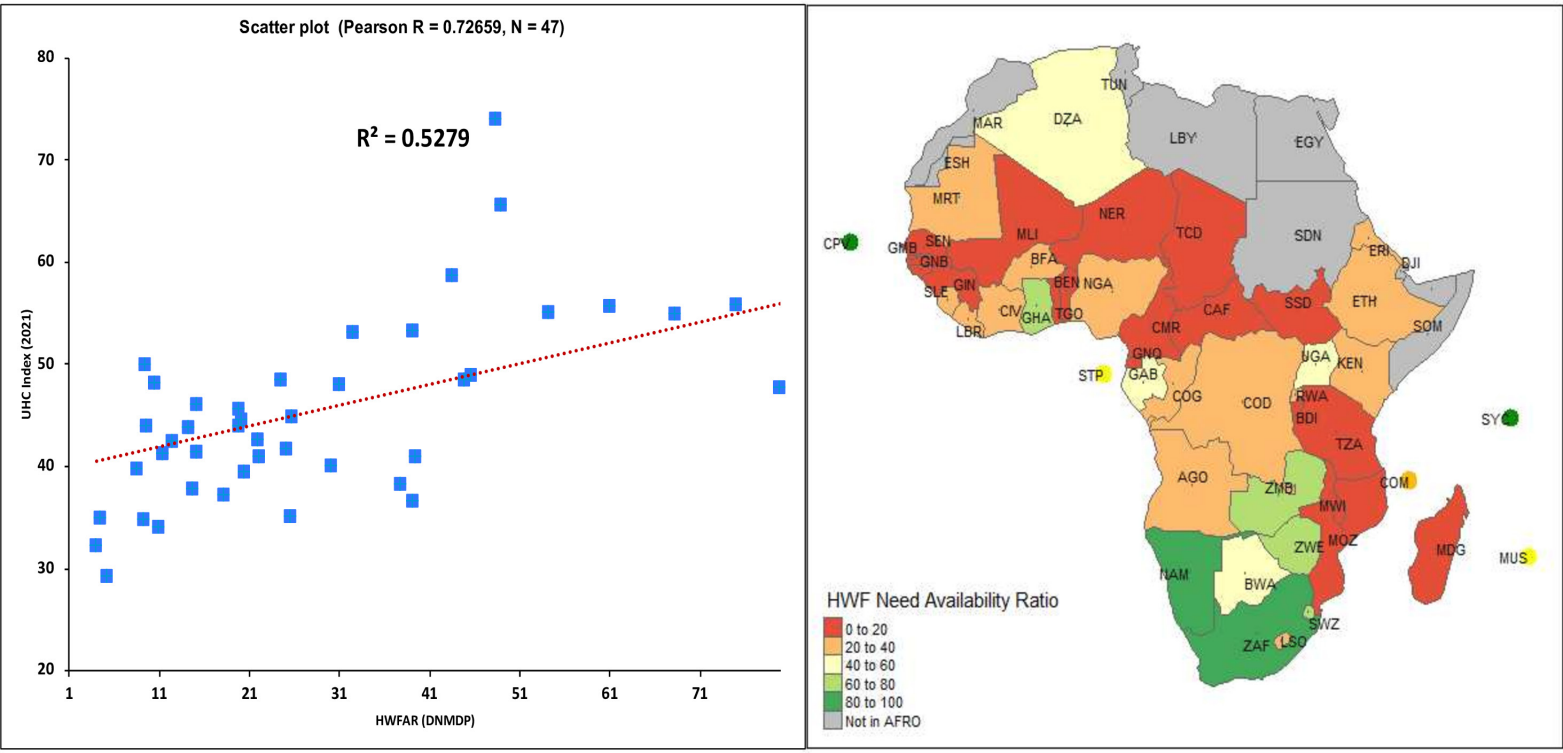
Relationship between HWF Need Availability Ratio (NAR) and UHC Service Coverage Score. HWF, health workforce.

The analysis revealed that the HWF need availability ratio was highly correlated with UHC SCI attainment (r=0.73) and explained about 53% of the variations in UHC SCI scores observed across countries in the African Region in 2021 (figure 5).

## Discussion

This study estimates that the WHO Africa Region needed 9.75 million health workers in 2022 to appropriately address disease burden and population health needs. With about 4.17 million health workers being available, the needs-based shortage was estimated to be approximately 5.6 million. The current estimate is in the same order of magnitude as recent WHO global estimates of HWF shortages.^9^ In addition, the Global Burden of Disease Collaborative,^13^ estimated shortage in sub-Saharan Africa for selected occupations draws a similar conclusion to the current estimates, with differences in the magnitude of shortfall obviously due to differences in methodological approaches and underpinning data sources. Much earlier estimates using thresholds or benchmarks also warned of these shortages.^11 58 59^ For example, using SDG index methodology, it was estimated at the onset of the SDG era that the Region would need a total of nearly 6.5 million health workers by 2030.^11^ With the nuanced needs-based method incorporating disease burden, essential health interventions, expert opinions on professional standards and a comprehensive coverage of health worker occupations, the current study shows that the ‘true’ need of health workers is about double that of the previous estimate. The present study enabled a direct linkage to essential service packages and has more granularity in terms of occupation level needs that are contextualised to the countries’ situation on disease burden, demographic changes and the countries’ specific skill mix. Also, with more nuance methods that incorporate disease burden, essential health interventions, expert opinions on professional standards, and comprehensive coverage of health worker occupations, this result estimates that the true need for health workers is more than double the previous estimates.

This analysis found that 99% of the needs-based requirements for health workers can be explained by the evolving disease burden, which is consistent with the conceptual notion that the population’s level of health ought to be the foundation of determining the need for health workers.^41 60^ Empirical studies have established a clear relationship between population health status and the propensity to use health services, which creates a workload that requires health workers.^61 62^ When the assumption of changing disease burden is relaxed, the need for health workers increases significantly by 14% in the base estimate, 6% in the minimum estimate and as much as 30% in the maximum scenario. Thus, the model is very sensitive to the trajectory and rate of change in the burden and prevalence of diseases, risk factors and injuries.

Nevertheless, the prevalence estimates may reflect the currently detected burden of diseases and risk factors, which in turn is a function of the health system’s capacity to detect and report. Low-income countries tend to have a large undetected burden of disease, especially for non-communicable diseases, in part due to a shortage of detection services. This could lead to an underestimation of health needs, hence the HWF requirement derived therefrom.

Also, the size of the ageing population is contributing to the needs-based HWF requirements. For example, five countries (South Africa, Mauritania, Namibia, Seychelles and Mauritius) with the highest estimated density of needed health workers have an average of 6.6% (range: 3.2%–12.5%) of the population being 65 years or older. In contrast, the rest of the 42 countries have an average of 3.1% of the population being 65 years or older (range: 2%–6.7%) (see online supplemental material 7).

This analysis estimates that the African Region faces a potential needs-based shortage of 6.1 million health workers by 2030 if the disease burden and population health needs are considered. The available health workers covered 43% of the needs-based requirements in 2022 and is anticipated to improve to 48% by 2030 if the current trajectory of training and education outputs are maintained. This estimate corroborates current global knowledge,^9 13 63^ and for specific country contexts such as Ghana, Guinea, Kenya, Lesotho, Namibia, Niger, Rwanda and Zimbabwe,^2831 33 35 39 64^,^67^ in terms of the direction of the evidence but with variations stemming from the nuanced and comprehensive approach employed in this analysis. Some researchers,^13^ suggested that global density-based estimates by WHO potentially underestimated the true scale of HWF requirements. However, the approach employed in this analysis provides more granular estimates, but the findings are consistent with the previous WHO global estimates.

The increasing paradoxical surplus of health workers is compounding the crisis and must be tackled alongside expanding education and training. One key threat is the rising paradoxical unemployment of health workers,^68 69^—estimated to be 27% on average and becoming widespread,^70^ which is increasing unsalaried/unpaid precarious work,^71^ and agitations.^72^ Thus, the solution to the dire needs-based shortage of health workers is not just about training more health workers but also ensuring their employment, equitable distribution and retention.^12 73 74^ The budgetary implications of addressing the HWF crisis must be at the centre of any policy and strategic action. In sensitivity analysis, the authors ran a scenario that assumed that all the trained health workers would be employed. This scenario would likely add almost 0.84 million health workers by 2030, which will reduce the 2030 needs-based shortage of those occupations by 14% from 6.1 million to 5.2 million.

### Strengths and limitations

To the best of our knowledge, this analysis represents the first attempt at using nuanced and country-specific data for all countries in the WHO African Region encompassing the known drivers of the need to assess HWF requirements: (a) the population’s size and demography together with the (b) prevalence of diseases and risk factors, and (c) type and frequency of health interventions planned or is otherwise necessary to address the identified diseases, conditions and risk factors. This approach also allows the inclusion of many health worker cadres and incorporates the expert inputs of health workers, health systems and policy practitioners who are working in the Region and are conversant with the models of care. Despite using the same sources of data for all countries, the specific strength of this methodological approach is that it produces specific needs-based requirements and densities for countries, making it possible for the estimates to be used for dialogue and planning at the country level without necessarily expecting rich and poorer countries to use one threshold for planning.

However, some limitations must be taken into account. First, the model is data intensive, and we did not have countries’ owned data on over 300 diseases, risk factors and injuries that were matched to 455 interventions. We, therefore, relied on only publicly available global datasets, some of which may be modelled data—hence, this study indirectly inherits the assumptions underpinning those modelled data.

Second, the use of expert opinion in obtaining data on SW or productivity measure, workload proportions distributed among the different occupations matched with interventions to a particular disease, as well as the proportion of patients who would likely need those interventions and the frequency they would need it per year, came with subjectivity.

Third, the SW was defined for all countries and did not consider country-specific variations in time to deliver interventions due to variations in health settings and distances between population and healthcare providers. As expert-based, without field validation, these could represent an aspirational workload in an ideal situation. Also, obtaining the plausible range of time necessary to undertake the interventions to acceptable professional levels, considering Benner’s novice to an expert model, produced a wide range of practice variations, from minimum to maximum time. Unsurprisingly, this led to a wide range of uncertainty in the estimates, with a large range between the minimum and maximum estimates.

Finally, in the stock modelling, a 70% absorption capacity was assumed based on data derived from literature and a regional average based on a sample of countries that have conducted health labour market analysis. However, absorption capacity is not constant across countries. Consequently, scenario analysis was conducted to explore the impact of varying absorption capacity on the projected shortage of health workers by 2030.

## Conclusion

This study presented needs-based modelling of the number and mix of health workers needed to adequately address Africa’s disease burden, considering the population dynamics and package of services and incorporating expert opinion on professional standards of service delivery. The findings provide contemporary evidence and valuable insights to shape health dialogues, policies and investments. Our approach has proven to be valid and highly correlates with UHC, explaining 53% of variations in UHC scores among countries. The African Region would need to more than double its 2022 HWF stock if the disease burden and growing population health needs are to be adequately addressed.

## Supplementary material

10.1136/bmjgh-2024-015972 online supplemental material 1

10.1136/bmjgh-2024-015972 online supplemental material 2

10.1136/bmjgh-2024-015972 online supplemental material 3

10.1136/bmjgh-2024-015972 online supplemental material 4

10.1136/bmjgh-2024-015972 online supplemental material 5

10.1136/bmjgh-2024-015972 online supplemental material 6

## Data Availability

Data are available in a public, open access repository. All data relevant to the study are included in the article or uploaded as supplementary information.
