# Supplementary material for: Projected health workforce requirements and shortage for addressing the disease burden in the WHO Africa Region, 2022–2030: a needs-based modelling study
Source: BMJ Glob Health. 2024 Oct 22;7(Suppl 1):e015972. doi: 10.1136/bmjgh-2024-015972 (PMC11789529; doi:10.1136/bmjgh-2024-015972)
Supplement: online supplemental material 1 [file bmjgh-7-Suppl_1-s001.pdf]

Supplementary material 1: List of diseases, conditions, risk factors and injuries

No	Program Area (Please, select)	Disease, Risk Factor, Public Health Intervention	Measure
1	Non-communicable Conditions	Acne vulgaris	Prevalence
2	Non-communicable Conditions	Acute glomerulonephritis	Prevalence
3	Communicable Conditions	Acute hepatitis A	Prevalence
4	Communicable Conditions	Acute hepatitis B	Prevalence
5	Communicable Conditions	Acute hepatitis C	Prevalence
6	Communicable Conditions	Acute hepatitis E	Prevalence
7	Cancer	Acute lymphoid leukemia	Prevalence
8	Cancer	Acute myeloid leukemia	Prevalence
9	Non-communicable Conditions	Adverse effects of medical treatment	Prevalence
10	Neglected, Tropical and Vector borne Diseases	African trypanosomiasis	Prevalence
11	Non-communicable Conditions	Age-related and other hearing loss	Prevalence
12	Non-communicable Conditions	Age-related macular degeneration	Prevalence
13	Non-communicable Conditions	Alcohol use disorders	Prevalence
14	Non-communicable Conditions	Alcoholic cardiomyopathy	Prevalence
15	Non-communicable Conditions	Alopecia areata	Prevalence
16	Non-communicable Conditions	Alzheimer's disease and other dementias	Prevalence
17	Non-communicable Conditions	Amphetamine use disorders	Prevalence
18	Mental Health	Anorexia nervosa	Prevalence
19	Mental Health	Anxiety disorders	Prevalence
20	Non-communicable Conditions	Aortic aneurysm	Prevalence
21	Non-communicable Conditions	Appendicitis	Prevalence

**22**	Non-communicable Conditions	Asbestosis	Prevalence
**23**	Non-communicable Conditions	Ascariasis	Prevalence
**24**	Non-communicable Conditions	Asthma	Prevalence
**25**	Communicable Conditions	Atopic dermatitis	Prevalence
**26**	Non-communicable Conditions	Atrial fibrillation and flutter	Prevalence
**27**	Mental Health	Attention-deficit/hyperactivity disorder	Prevalence
**28**	Child and adolescent Health	Autism spectrum disorders	Prevalence
**29**	Cancer	Benign and in situ cervical and uterine neoplasms	Prevalence
**30**	Cancer	Benign and in situ intestinal neoplasms	Prevalence
**31**	Cancer	Benign prostatic hyperplasia	Prevalence
**32**	Mental Health	Bipolar disorder	Prevalence
**33**	Cancer	Bladder cancer	Prevalence
**34**	Cancer	Brain and central nervous system cancer	Prevalence
**35**	Cancer	Breast cancer	Prevalence
**36**	Mental Health	Bulimia nervosa	Prevalence
**37**	Non-communicable Conditions	Cannabis use disorders	Prevalence
**38**	Oral Health	Caries of deciduous teeth	Prevalence
**39**	Oral Health	Caries of permanent teeth	Prevalence
**40**	Opthalmic and ENT Services	Cataract	Prevalence
**41**	Non-communicable Conditions	Cellulitis	Prevalence
**42**	Cancer	Cervical cancer	Prevalence
**43**	Neglected, Tropical and Vector borne Diseases	Chagas disease	Prevalence
**44**	Communicable Conditions	Chlamydial infection	Prevalence
**45**	Non-communicable Conditions	Chronic kidney disease due to diabetes mellitus type 1	Prevalence
**46**	Non-communicable Conditions	Chronic kidney disease due to diabetes mellitus type 2	Prevalence
**47**	Non-communicable Conditions	Chronic kidney disease due to glomerulonephritis	Prevalence

**48**	Non-communicable Conditions	Chronic kidney disease due to hypertension	Prevalence
**49**	Non-communicable Conditions	Chronic kidney disease due to other and unspecified causes	Prevalence
**50**	Cancer	Chronic lymphoid leukemia	Prevalence
**51**	Cancer	Chronic myeloid leukemia	Prevalence
**52**	Non-communicable Conditions	Chronic obstructive pulmonary disease	Prevalence
**53**	Non-communicable Conditions	Cirrhosis and other chronic liver diseases due to alcohol use	Prevalence
**54**	Non-communicable Conditions	Cirrhosis and other chronic liver diseases due to hepatitis B	Prevalence
**55**	Non-communicable Conditions	Cirrhosis and other chronic liver diseases due to hepatitis C	Prevalence
**56**	Non-communicable Conditions	Cirrhosis and other chronic liver diseases due to NAFLD	Prevalence
**57**	Non-communicable Conditions	Cirrhosis and other chronic liver diseases due to other causes	Prevalence
**58**	Non-communicable Conditions	Coal workers pneumoconiosis	Prevalence
**59**	Non-communicable Conditions	Cocaine use disorders	Prevalence
**60**	Cancer	Colon and rectum cancer	Prevalence
**61**	Mental Health	Conduct disorder	Prevalence
**62**	Mental Health	Conflict and terrorism	Prevalence
**63**	Reproductive, Maternal and Neonatal Health	Congenital heart anomalies	Prevalence
**64**	Reproductive, Maternal and Neonatal Health	Congenital musculoskeletal and limb anomalies	Prevalence
**65**	Communicable Conditions	Contact dermatitis	Prevalence
**66**	Neglected, Tropical and Vector borne Diseases	Cutaneous and mucocutaneous leishmaniasis	Prevalence
**67**	Non-communicable Conditions	Cyclist road injuries	Prevalence
**68**	Neglected, Tropical and Vector borne Diseases	Cystic echinococcosis	Prevalence
**69**	Neglected, Tropical and Vector borne Diseases	Cysticercosis	Prevalence

**70**	Neglected, Tropical and Vector borne Diseases	Decubitus ulcer	Prevalence
**71**	Neglected, Tropical and Vector borne Diseases	Dengue	Prevalence
**72**	Non-communicable Conditions	Diabetes mellitus type 1	Prevalence
**73**	Non-communicable Conditions	Diabetes mellitus type 2	Prevalence
**74**	Non-communicable Conditions	Diarrheal diseases	Prevalence
**75**	Nutrition	Dietary iron deficiency	Prevalence
**76**	Child and adolescent Health	Digestive congenital anomalies	Prevalence
**77**	Communicable Conditions	Diphtheria	Prevalence
**78**	Non-communicable Conditions	Down syndrome	Prevalence
**79**	Non-communicable Conditions	Drowning	Prevalence
**80**	Communicable Conditions	Drug-susceptible tuberculosis	Prevalence
**81**	Mental Health	Dysthymia	Prevalence
**82**	Communicable Conditions	Ebola	Prevalence
**83**	Reproductive, Maternal and Neonatal Health	Ectopic pregnancy	Prevalence
**84**	Oral Health	Edentulism	Prevalence
**85**	Non-communicable Conditions	Encephalitis	Prevalence
**86**	Non-communicable Conditions	Endocarditis	Prevalence
**87**	Non-communicable Conditions	Endocrine, metabolic, blood, and immune disorders	Prevalence
**88**	Reproductive, Maternal and Neonatal Health	Endometriosis	Prevalence
**89**	Non-communicable Conditions	Environmental heat and cold exposure	Prevalence
**90**	Cancer	Esophageal cancer	Prevalence
**91**	Mental Health	Executions and police conflict	Prevalence
**92**	Mental Health	Exposure to forces of nature	Prevalence
**93**	Communicable Conditions	Extensively drug-resistant tuberculosis	Prevalence

**94**	Non-communicable Conditions	Falls	Prevalence
**95**	Reproductive, Maternal and Neonatal Health	Female infertility	Prevalence
**96**	Communicable Conditions	Fire, heat, and hot substances	Prevalence
**97**	Non-communicable Conditions	Food-borne trematodiases	Prevalence
**98**	Opthalmic and ENT Services	Foreign body in eyes	Prevalence
**99**	Non-communicable Conditions	Foreign body in other body part	Prevalence
**100**	Communicable Conditions	Fungal skin diseases	Prevalence
**101**	Non-communicable Conditions	G6PD deficiency	Prevalence
**102**	Non-communicable Conditions	G6PD trait	Prevalence
**103**	Non-communicable Conditions	Gallbladder and biliary diseases	Prevalence
**104**	Cancer	Gallbladder and biliary tract cancer	Prevalence
**105**	Non-communicable Conditions	Gastritis and duodenitis	Prevalence
**106**	Non-communicable Conditions	Gastroesophageal reflux disease	Prevalence
**107**	Communicable Conditions	Genital herpes	Prevalence
**108**	Reproductive, Maternal and Neonatal Health	Genital prolapse	Prevalence
**109**	Opthalmic and ENT Services	Glaucoma	Prevalence
**110**	Communicable Conditions	Gonococcal infection	Prevalence
**111**	Non-communicable Conditions	Gout	Prevalence
**112**	Neglected, Tropical and Vector borne Diseases	Guinea worm disease	Prevalence
**113**	Reproductive, Maternal and Neonatal Health	Hemolytic disease and other neonatal jaundice	Prevalence
**114**	Communicable Conditions	HIV/AIDS - Drug-susceptible Tuberculosis	Prevalence
**115**	Communicable Conditions	HIV/AIDS - Extensively drug-resistant Tuberculosis	Prevalence
**116**	Communicable Conditions	HIV/AIDS - Multidrug-resistant Tuberculosis without extensive drug resistance	Prevalence

**117**	Communicable Conditions	HIV/AIDS resulting in other diseases	Prevalence
**118**	Non-communicable Conditions	Hodgkin lymphoma	Prevalence
**119**	Neglected, Tropical and Vector borne Diseases	Hookworm disease	Prevalence
**120**	Non-communicable Conditions	Hypertensive heart disease	Prevalence
**121**	Communicable Conditions	Idiopathic developmental intellectual disability	Prevalence
**122**	Non-communicable Conditions	Idiopathic epilepsy	Prevalence
**123**	Reproductive, Maternal and Neonatal Health	Indirect maternal deaths	Prevalence
**124**	Non-communicable Conditions	Inflammatory bowel disease	Prevalence
**125**	Non-communicable Conditions	Inguinal, femoral, and abdominal hernia	Prevalence
**126**	Non-communicable Conditions	Interstitial lung disease and pulmonary sarcoidosis	Prevalence
**127**	Non-communicable Conditions	Intracerebral hemorrhage	Prevalence
**128**	Non-communicable Conditions	Invasive Non-typhoidal Salmonella (iNTS)	Prevalence
**129**	Non-communicable Conditions	Iodine deficiency	Prevalence
**130**	Non-communicable Conditions	Ischemic heart disease	Prevalence
**131**	Non-communicable Conditions	Ischemic stroke	Prevalence
**132**	Cancer	Kidney cancer	Prevalence
**133**	Non-communicable Conditions	Klinefelter syndrome	Prevalence
**134**	Cancer	Larynx cancer	Prevalence
**135**	Reproductive, Maternal and Neonatal Health	Late maternal deaths	Prevalence
**136**	Communicable Conditions	Latent tuberculosis infection	Prevalence
**137**	Neglected, Tropical and Vector borne Diseases	Leprosy	Prevalence
**138**	Cancer	Lip and oral cavity cancer	Prevalence
**139**	Cancer	Liver cancer due to alcohol use	Prevalence
**140**	Cancer	Liver cancer due to hepatitis B	Prevalence

**141**	Cancer	Liver cancer due to hepatitis C	Prevalence
**142**	Cancer	Liver cancer due to NASH	Prevalence
**143**	Cancer	Liver cancer due to other causes	Prevalence
**144**	Non-communicable Conditions	Low back pain	Prevalence
**145**	Communicable Conditions	Lower respiratory infections	Prevalence
**146**	Neglected, Tropical and Vector borne Diseases	Lymphatic filariasis	Prevalence
**147**	Mental Health	Major depressive disorder	Prevalence
**148**	Non-communicable Conditions	Malaria	Prevalence
**149**	Non-communicable Conditions	Male infertility	Prevalence
**150**	Cancer	Malignant skin melanoma	Prevalence
**151**	Reproductive, Maternal and Neonatal Health	Maternal abortion and miscarriage	Prevalence
**152**	Reproductive, Maternal and Neonatal Health	Maternal deaths aggravated by HIV/AIDS	Prevalence
**153**	Reproductive, Maternal and Neonatal Health	Maternal hemorrhage	Prevalence
**154**	Reproductive, Maternal and Neonatal Health	Maternal hypertensive disorders	Prevalence
**155**	Reproductive, Maternal and Neonatal Health	Maternal obstructed labor and uterine rupture	Prevalence
**156**	Reproductive, Maternal and Neonatal Health	Maternal sepsis and other maternal infections	Prevalence
**157**	Communicable Conditions	Measles	Prevalence
**158**	Communicable Conditions	Meningitis	Prevalence
**159**	Cancer	Mesothelioma	Prevalence
**160**	Non-communicable Conditions	Migraine	Prevalence
**161**	Non-communicable Conditions	Motor neuron disease	Prevalence

**162**	Non-communicable Conditions	Motor vehicle road injuries	Prevalence
**163**	Non-communicable Conditions	Motorcyclist road injuries	Prevalence
**164**	Communicable Conditions	Multidrug-resistant tuberculosis without extensive drug resistance	Prevalence
**165**	Cancer	Multiple myeloma	Prevalence
**166**	Non-communicable Conditions	Multiple sclerosis	Prevalence
**167**	Cancer	Myelodysplastic, myeloproliferative, and other hematopoietic neoplasms	Prevalence
**168**	Non-communicable Conditions	Myocarditis	Prevalence
**169**	Cancer	Nasopharynx cancer	Prevalence
**170**	Non-communicable Conditions	Near vision loss	Prevalence
**171**	Non-communicable Conditions	Neck pain	Prevalence
**172**	Reproductive, Maternal and Neonatal Health	Neonatal encephalopathy due to birth asphyxia and trauma	Prevalence
**173**	Reproductive, Maternal and Neonatal Health	Neonatal preterm birth	Prevalence
**174**	Reproductive, Maternal and Neonatal Health	Neonatal sepsis and other neonatal infections	Prevalence
**175**	Reproductive, Maternal and Neonatal Health	Neural tube defects	Prevalence
**176**	Cancer	Non-Hodgkin lymphoma	Prevalence
**177**	Cancer	Non-melanoma skin cancer (basal-cell carcinoma)	Prevalence
**178**	Cancer	Non-melanoma skin cancer (squamous-cell carcinoma)	Prevalence
**179**	Non-communicable Conditions	Non-rheumatic calcific aortic valve disease	Prevalence
**180**	Non-communicable Conditions	Non-rheumatic degenerative mitral valve disease	Prevalence
**181**	Non-communicable Conditions	Non-venomous animal contact	Prevalence
**182**	Neglected, Tropical and Vector borne Diseases	Onchocerciasis	Prevalence

**183**	Mental Health	Opioid use disorders	Prevalence
**184**	Non-communicable Conditions	Orofacial clefts	Prevalence
**185**	Non-communicable Conditions	Osteoarthritis hand	Prevalence
**186**	Non-communicable Conditions	Osteoarthritis hip	Prevalence
**187**	Non-communicable Conditions	Osteoarthritis knee	Prevalence
**188**	Non-communicable Conditions	Osteoarthritis other	Prevalence
**189**	Non-communicable Conditions	Other benign and in situ neoplasms	Prevalence
**190**	Non-communicable Conditions	Other cardiomyopathy	Prevalence
**191**	Non-communicable Conditions	Other cardiovascular and circulatory diseases	Prevalence
**192**	Non-communicable Conditions	Other chromosomal abnormalities	Prevalence
**193**	Non-communicable Conditions	Other chronic respiratory diseases	Prevalence
**194**	Non-communicable Conditions	Other congenital birth defects	Prevalence
**195**	Non-communicable Conditions	Other digestive diseases	Prevalence
**196**	Non-communicable Conditions	Other drug use disorders	Prevalence
**197**	Non-communicable Conditions	Other exposure to mechanical forces	Prevalence
**198**	Reproductive, Maternal and Neonatal Health	Other gynecological diseases	Prevalence
**199**	Non-communicable Conditions	Other hemoglobinopathies and hemolytic anemias	Prevalence
**200**	Communicable Conditions	Other intestinal infectious diseases	Prevalence
**201**	Cancer	Other leukemia	Prevalence
**202**	Cancer	Other malignant neoplasms	Prevalence
**203**	Reproductive, Maternal and Neonatal Health	Other maternal disorders	Prevalence
**204**	Mental Health	Other mental disorders	Prevalence
**205**	Non-communicable Conditions	Other musculoskeletal disorders	Prevalence
**206**	Neglected, Tropical and Vector borne Diseases	Other neglected tropical diseases	Prevalence
**207**	Non-communicable Conditions	Other neonatal disorders	Prevalence

**208**	Non-communicable Conditions	Other neurological disorders	Prevalence
**209**	Non-communicable Conditions	Other non-rheumatic valve diseases	Prevalence
**210**	Nutrition	Other nutritional deficiencies	Prevalence
**211**	Oral Health	Other oral disorders	Prevalence
**212**	Cancer	Other pharynx cancer	Prevalence
**213**	Non-communicable Conditions	Other pneumoconiosis	Prevalence
**214**	Non-communicable Conditions	Other road injuries	Prevalence
**215**	Non-communicable Conditions	Other sense organ diseases	Prevalence
**216**	Communicable Conditions	Other sexually transmitted infections	Prevalence
**217**	Non-communicable Conditions	Other skin and subcutaneous diseases	Prevalence
**218**	Non-communicable Conditions	Other transport injuries	Prevalence
**219**	Non-communicable Conditions	Other unintentional injuries	Prevalence
**220**	Communicable Conditions	Other unspecified infectious diseases	Prevalence
**221**	Non-communicable Conditions	Other urinary diseases	Prevalence
**222**	Non-communicable Conditions	Other vision loss	Prevalence
**223**	Opthalmic and ENT Services	Otitis media	Prevalence
**224**	Cancer	Ovarian cancer	Prevalence
**225**	Cancer	Pancreatic cancer	Prevalence
**226**	Non-communicable Conditions	Pancreatitis	Prevalence
**227**	Non-communicable Conditions	Paralytic ileus and intestinal obstruction	Prevalence
**228**	Non-communicable Conditions	Paratyphoid fever	Prevalence
**229**	Non-communicable Conditions	Parkinson's disease	Prevalence
**230**	Non-communicable Conditions	Pedestrian road injuries	Prevalence
**231**	Non-communicable Conditions	Peptic ulcer disease	Prevalence
**232**	Non-communicable Conditions	Periodontal diseases	Prevalence
**233**	Non-communicable Conditions	Peripheral artery disease	Prevalence
**234**	Non-communicable Conditions	Physical violence by firearm	Prevalence

**235**	Non-communicable Conditions	Physical violence by other means	Prevalence
**236**	Non-communicable Conditions	Physical violence by sharp object	Prevalence
**237**	Non-communicable Conditions	Poisoning by carbon monoxide	Prevalence
**238**	Non-communicable Conditions	Poisoning by other means	Prevalence
**239**	Reproductive, Maternal and Neonatal Health	Polycystic ovarian syndrome	Prevalence
**240**	Reproductive, Maternal and Neonatal Health	Premenstrual syndrome	Prevalence
**241**	Cancer	Prostate cancer	Prevalence
**242**	Non-communicable Conditions	Protein-energy malnutrition	Prevalence
**243**	Non-communicable Conditions	Pruritus	Prevalence
**244**	Non-communicable Conditions	Psoriasis	Prevalence
**245**	Non-communicable Conditions	Pulmonary aspiration and foreign body in airway	Prevalence
**246**	Non-communicable Conditions	Pyoderma	Prevalence
**247**	Community Health	Rabies	Prevalence
**248**	Non-communicable Conditions	Refraction disorders	Prevalence
**249**	Non-communicable Conditions	Rheumatic heart disease	Prevalence
**250**	Non-communicable Conditions	Rheumatoid arthritis	Prevalence
**251**	Neglected, Tropical and Vector borne Diseases	Scabies	Prevalence
**252**	Neglected, Tropical and Vector borne Diseases	Schistosomiasis	Prevalence
**253**	Mental Health	Schizophrenia	Prevalence
**254**	Non-communicable Conditions	Seborrhoeic dermatitis	Prevalence
**255**	Non-communicable Conditions	Self-harm by firearm	Prevalence
**256**	Non-communicable Conditions	Self-harm by other specified means	Prevalence
**257**	Non-communicable Conditions	Sexual violence	Prevalence
**258**	Non-communicable Conditions	Sickle cell disorders	Prevalence

**259**	Non-communicable Conditions	Sickle cell trait	Prevalence
**260**	Opthalmic and ENT Services	Silicosis	Prevalence
**261**	Cancer	Stomach cancer	Prevalence
**262**	Non-communicable Conditions	Subarachnoid hemorrhage	Prevalence
**263**	Child and adolescent Health	Sudden infant death syndrome	Prevalence
**264**	Communicable Conditions	Syphilis	Prevalence
**265**	Non-communicable Conditions	Tension-type headache	Prevalence
**266**	Cancer	Testicular cancer	Prevalence
**267**	Non-communicable Conditions	Tetanus	Prevalence
**268**	Non-communicable Conditions	Thalassemias	Prevalence
**269**	Non-communicable Conditions	Thalassemias trait	Prevalence
**270**	Cancer	Thyroid cancer	Prevalence
**271**	Communicable Conditions	Total burden related to hepatitis B	Prevalence
**272**	Communicable Conditions	Total burden related to hepatitis C	Prevalence
**273**	Non-communicable Conditions	Total burden related to Non-alcoholic fatty liver disease (NAFLD)	Prevalence
**274**	Cancer	Total cancers	Prevalence
**275**	Cancer	Tracheal, bronchus, and lung cancer	Prevalence
**276**	Non-communicable Conditions	Trachoma	Prevalence
**277**	Neglected, Tropical and Vector borne Diseases	Trichomoniasis	Prevalence
**278**	Neglected, Tropical and Vector borne Diseases	Trichuriasis	Prevalence
**279**	Reproductive, Maternal and Neonatal Health	Turner syndrome	Prevalence
**280**	Neglected, Tropical and Vector borne Diseases	Typhoid fever	Prevalence
**281**	Non-communicable Conditions	Unintentional firearm injuries	Prevalence

**282**	Non-communicable Conditions	Upper respiratory infections	Prevalence
**283**	Communicable Conditions	Urinary tract infections and interstitial nephritis	Prevalence
**284**	Non-communicable Conditions	Urogenital congenital anomalies	Prevalence
**285**	Non-communicable Conditions	Urolithiasis	Prevalence
**286**	Non-communicable Conditions	Urticaria	Prevalence
**287**	Cancer	Uterine cancer	Prevalence
**288**	Reproductive, Maternal and Neonatal Health	Uterine fibroids	Prevalence
**289**	Non-communicable Conditions	Varicella and herpes zoster	Prevalence
**290**	Non-communicable Conditions	Vascular intestinal disorders	Prevalence
**291**	Non-communicable Conditions	Venomous animal contact	Prevalence
**292**	Non-communicable Conditions	Viral skin diseases	Prevalence
**293**	Non-communicable Conditions	Visceral leishmaniasis	Prevalence
**294**	Non-communicable Conditions	Vitamin A deficiency	Prevalence
**295**	Non-communicable Conditions	Whooping cough	Prevalence
**296**	Communicable Conditions	Yellow fever	Prevalence
**297**	Communicable Conditions	Zika virus	Prevalence
**298**	Communicable Conditions	COVID-19	Prevalence
**299**	Nutrition	Stunting	Prevalence
**300**	Non-communicable Conditions	Obesity	Prevalence
**301**	Reproductive, Maternal and Neonatal Health	Antenatal care	Coverage
**302**	Reproductive, Maternal and Neonatal Health	Postnatal care	Coverage
**303**	Communicable Conditions	Immunization	Coverage
**304**	Reproductive, Maternal and Neonatal Health	Skilled Birth Attendance	Coverage

**305**	Community Health	Family-targeted interventions (Number of local families in the country)	Coverage
**306**	Community Health	Community-targeted interventions (Number of local communities in the country)	Coverage
**307**	Community Health	Persons with disability	Prevalence
**308**	Reproductive, Maternal and Neonatal Health	Family Planning	Coverage
