# Supplementary material for: Projected health workforce requirements and shortage for addressing the disease burden in the WHO Africa Region, 2022–2030: a needs-based modelling study
Source: BMJ Glob Health. 2024 Oct 22;7(Suppl 1):e015972. doi: 10.1136/bmjgh-2024-015972 (PMC11789529; doi:10.1136/bmjgh-2024-015972)
Supplement: online supplemental material 2 [file bmjgh-7-Suppl_1-s002.pdf]

Supplementary material 2: Interventions by Health Occupations

ISCO-08 Classification	Occupation	Public Health Function (Along the Continuum of Care)	Essential Intervention	Activity Type	Unit of Measurement	Professional Standard (Average Time)	Minimum Professional Standard (Time)	Maximum Professional Standard (Time)
**2212 - Specialist medical practitioners**	Anaesthesiologist	Detection/Diagnosis	Preoperative assessment of patient	Direct_Patient_Care	Minutes per patient	20	15	30
**2212 - Specialist medical practitioners**	Anaesthesiologist	Disease Management/Curative Services	Postoperative assessment of patient	Direct_Patient_Care	Minutes per patient	20	15	30
**2212 - Specialist medical practitioners**	Anaesthesiologist	Disease Management/Curative Services	Intraoperative Anaesthesia minor surgery includes Minor surgery includes Incision and Drainage, lacerations	Direct_Patient_Care	Minutes per patient	40	30	50
**2212 - Specialist medical practitioners**	Anaesthesiologist	Disease Management/Curative Services	Intraoperative Anaesthesia major surgery such as Major surgery includes laparotomies,head and neck	Direct_Patient_Care	Minutes per patient	150	120	180
**2212 - Specialist medical practitioners**	Anaesthesiologist	Disease Management/Curative Services	Critical patient management and Hemodynamic support, central and arterial lines, intubations, Intensive Care Unit ward rounds	Direct_Patient_Care	Minutes per patient	80	60	90
**2212 - Specialist medical practitioners**	Anaesthesiologist	Disease Management/Curative Services	Outpatient Pain management includes regional techniques in the outpatients	Direct_Patient_Care	Minutes per patient	40	30	50

**2212 - Specialist medical practitioners**	Anaesthesiologist	Disease Management/Curative Services	Critical Care Unit Admission and airway management / Patient on life support	Direct_Patient_Care	Minutes per patient	75	60	90
**2212 - Specialist medical practitioners**	Anaesthesiologist	Disease Management/Curative Services	Critical Care Unit Patient Transfer and Patient on life support	Direct_Patient_Care	Minutes per patient	75	60	90
**2212 - Specialist medical practitioners**	Anaesthesiologist	Disease Management/Curative Services	Sedation outside of Theatre includes Radiology departments,general ward	Direct_Patient_Care	Minutes per patient	40	30	50
**2212 - Specialist medical practitioners**	Anaesthesiologist	Disease Management/Curative Services	Clinical meetings	Support_Activity	Hours per day	1	1	1
**2212 - Specialist medical practitioners**	Cardiologist	Disease Management/Curative Services	Inpatient care (daily ward rounds, prescriptions,counseling, bedside procedures, documentation)	Direct_Patient_Care	Minutes per patient	20	10	30
**2212 - Specialist medical practitioners**	Cardiologist	Disease Management/Curative Services	Outpatient care (reviews, follow up of patients, patient education)	Direct_Patient_Care	Minutes per patient	20	15	25
**2212 - Specialist medical practitioners**	Cardiologist	Detection/Diagnosis	Echo cardiography	Direct_Patient_Care	Minutes per patient	30	15	35
**2212 - Specialist medical practitioners**	Cardiothoracic Surgeon	Disease Management/Curative Services	Bedside cardiothoracic procedures (includes: chest drain insertion, hemodialysis catheter insertion and removal)	Direct_Patient_Care	Minutes per patient	50	40	60
**2212 - Specialist medical practitioners**	Cardiothoracic Surgeon	Disease Management/Curative Services	Minor cardiothoracic surgery (includes: esophagoscopy, thoracic biopsy, gastrostomy tube insertion, rib resection, saphenous vein harvesting, varicose vein stripping) bronchoscopy, pleurodesis, AV fistula creation	Direct_Patient_Care	Minutes per patient	40	30	50

**2212 - Specialist medical practitioners**	Cardiothoracic Surgeon	Disease Management/Curative Services	Major cardiothoracic surgery (includes: Bronchopleural fistula closure/ lung abscess surgery, decortication, exploratory thoracotomy, chest or mediastinal tumor resection) diaphragm repair, heart surgery, pulmonectomy/ lung surgery , aneurysm repair	Direct_Patient_Care	Minutes per patient	150	120	180
**2212 - Specialist medical practitioners**	Cardiothoracic Surgeon	Disease Management/Curative Services	Inpatient care (includes ward rounds, prescriptions, bedside procedures like chest drain insertion and pleuroscentesis))	Direct_Patient_Care	Minutes per inpatient day	25	20	30
**2212 - Specialist medical practitioners**	Cardiothoracic Surgeon	Disease Management/Curative Services	Outpatient consultations (reviews, prescriptions,diagnostic procedures for example pleuroscentesis)	Direct_Patient_Care	Minutes per patient	25	20	40
**2212 - Specialist medical practitioners**	Cardiothoracic Surgeon	Disease Management/Curative Services	Multidisciplinary meetings	Direct_Patient_Care	Hours per day	1	1	1
**2261 - Dentists**	Dentist	Disease Management/Curative Services	Dental Out patients care (consultations, age estimation ,assessment of injuries , report generation , Oral health education)	Direct_Patient_Care	Minutes per patient	25	20	30
**2261 - Dentists**	Dentist	Disease Management/Curative Services	Dental major procedures as Surgical extraction , incision and drainage , management of fractures	Direct_Patient_Care	Minutes per patient	100	90	120

**2261 - Dentists**	Dentist	Disease Management/Curative Services	Dental minor procedures includes : Fitting of prosthetics , Fissure sealing ,tooth extraction ,scaling and polishing s, occlusal equilibartion ,biopsies ,implant placement.	Direct_Patient_Care	Minutes per patient	40	30	60
**2261 - Dentists**	Dentist	Disease Management/Curative Services	Dental inpatient care Ward rounds , in patient care ,prescribing , referrals	Direct_Patient_Care	Minutes per patient	20	15	25
**2261 - Dentists**	Dentist	Disease Management/Curative Services	Clinical meetings	Support_Activity	Hours per week	2	1	2
**2261 - Dentists**	Dentist	Health Promotion	Community outreach	Support_Activity	Hours per month	32	30	40
**2212 - Specialist medical practitioners**	Dermatologist	Disease Management/Curative Services	Inpatient care (Includes ward rounds , bedside procedures , prescriptions)	Direct_Patient_Care	Minutes per inpatient day	20	15	25
**2212 - Specialist medical practitioners**	Dermatologist	Disease Management/Curative Services	Outpatients care (consultations , referrals , follow up, prescriptions)	Direct_Patient_Care	Minutes per patient	25	20	30
**2212 - Specialist medical practitioners**	Dermatologist	Detection/Diagnosis	Bedside procedures includes : Skin biopsy …	Direct_Patient_Care	Minutes per task	25	20	30
**2212 - Specialist medical practitioners**	Endocrinologist	Disease Management/Curative Services	Outpatient consultations as Assessment, diagnosis, treatment	Direct_Patient_Care	Minutes per patient	25	20	30
**2212 - Specialist medical practitioners**	Endocrinologist	Disease Management/Curative Services	Clinical meetings	Support_Activity	Hours per day	1	1	1
**2212 - Specialist medical practitioners**	Endocrinologist	Disease Management/Curative Services	Inpatient care ward rounds , in patient care ,prescribing , referrals	Direct_Patient_Care	Minutes per inpatient day	20	15	25

**2212 - Specialist medical practitioners**	ENT Surgeon	Disease Management/Curative Services	ENT minor surgery (Includes :Adenotonsillectomy, Foreign body removal, Pharyngoscopy)	Direct_Patient_Care	Minutes per patient	50	40	60
**2212 - Specialist medical practitioners**	ENT Surgeon	Disease Management/Curative Services	Outpatient consultations (consultations , referrals , follow up etc)	Direct_Patient_Care	Minutes per patient	25	20	30
**2212 - Specialist medical practitioners**	ENT Surgeon	Disease Management/Curative Services	Inpatient care (Ward rounds , in patient care ,prescribing , referrals)	Direct_Patient_Care	Minutes per inpatient day	20	15	30
**2212 - Specialist medical practitioners**	ENT Surgeon	Disease Management/Curative Services	ENT major surgery (Head and Neck tumour excision)	Direct_Patient_Care	Minutes per patient	100	90	120
**2212 - Specialist medical practitioners**	ENT Surgeon	Disease Management/Curative Services	Clinical meetings	Support_Activity	Hours per day	1	1	1
**2212 - Specialist medical practitioners**	Gastroenterologist	Disease Management/Curative Services	Clinical meeting	Support_Activity	Hours per day	1	1	1
**2212 - Specialist medical practitioners**	Gastroenterologist	Disease Management/Curative Services	Outpatients consultations, referral of patient , follow up	Direct_Patient_Care	Minutes per patient (Old)	25	20	30
**2212 - Specialist medical practitioners**	Gastroenterologist	Disease Management/Curative Services	Inpatient care (Ward rounds , in patient care ,prescribing , referral of patient)	Direct_Patient_Care	Minutes per inpatient day	20	15	25
**2212 - Specialist medical practitioners**	Gastroenterologist	Detection/Diagnosis	Endoscopy as Upper gastrointestinal endoscopy, Diagnostic Colonoscopy	Direct_Patient_Care	Minutes per patient	35	30	40
**2211 - Generalist medical practitioners**	General Medical Practitioner (Generalist Doctor)	Disease Management/Curative Services	Outpatient Consultations as Assessment, diagnosis, treatment	Direct_Patient_Care	Minutes per patient	25	20	30

**2211 - Generalist medical practitioners**	General Medical Practitioner (Generalist Doctor)	Disease Management/Curative Services	Inpatient Care including ward rounds	Direct_Patient_Care	Minutes per inpatient day	20	15	25
**2211 - Generalist medical practitioners**	General Medical Practitioner (Generalist Doctor)	Disease Management/Curative Services	Bedside procedures as nasogastric tube insertion, lumbar puncture, catheterisation, paracentesis, chest tube insertion	Direct_Patient_Care	Minutes per patient	30	25	40
**2211 - Generalist medical practitioners**	General Medical Practitioner (Generalist Doctor)	Disease Management/Curative Services	Minor surgical procedures such suturing lacerations, biopsies, male circumcision, reduction of fractures,	Direct_Patient_Care	Minutes per patient	50	40	60
**2211 - Generalist medical practitioners**	General Medical Practitioner (Generalist Doctor)	Disease Management/Curative Services	Major surgical procedures such caesarean section	Direct_Patient_Care	Minutes per patient	60	45	120
**2211 - Generalist medical practitioners**	General Medical Practitioner (Generalist Doctor)	Disease Management/Curative Services	Clinical meetings	Support_Activity	Hours per week	2	1	2
**2212 - Specialist medical practitioners**	General Surgeon	Disease Management/Curative Services	Outpatient Consultations as Assessment, diagnosis, treatment	Direct_Patient_Care	Minutes per patient	25	20	30
**2212 - Specialist medical practitioners**	General Surgeon	Disease Management/Curative Services	Inpatient Care including ward rounds	Direct_Patient_Care	Minutes per inpatient day	20	15	25
**2212 - Specialist medical practitioners**	General Surgeon	Disease Management/Curative Services	Bedside surgical procedures as urethral catheterisation, cutdown IV access, etc	Direct_Patient_Care	Minutes per patient	25	20	30
**2212 - Specialist medical practitioners**	General Surgeon	Disease Management/Curative Services	Minor surgical procedures like breast lumpectomy, I&D, herniorrhaphy, etc	Direct_Patient_Care	Minutes per patient	40	30	50

**2212 - Specialist medical practitioners**	General Surgeon	Disease Management/Curative Services	Major surgical procedures like intestinal resection and anastomoses, mastectomy, cholecystectomy	Direct_Patient_Care	Minutes per patient	120	100	150
**2212 - Specialist medical practitioners**	General Surgeon	Disease Management/Curative Services	Clinical meetings	Support_Activity	Hours per day	1	1	1
**2212 - Specialist medical practitioners**	Haematologist	Disease Management/Curative Services	Inpatient care including ward rounds	Direct_Patient_Care	Minutes per inpatient day	20	15	25
**2212 - Specialist medical practitioners**	Haematologist	Disease Management/Curative Services	Outpatient consultations as Assessment, diagnosis, treatment	Direct_Patient_Care	Minutes per patient	25	20	30
**2212 - Specialist medical practitioners**	Haematologist	Detection/Diagnosis	Bone marrow aspiration	Direct_Patient_Care	Minutes per patient	25	20	30
**2212 - Specialist medical practitioners**	Haematologist	Disease Management/Curative Services	Clinical meetings as Continuing medical education (CME) meetings	Support_Activity	Hours per day	1	1	1
**2212 - Specialist medical practitioners**	Infectious Diseases Specialist	Disease Management/Curative Services	Outpatients Consultations as Assessment, diagnosis, treatment	Direct_Patient_Care	Minutes per patient	25	20	30
**2212 - Specialist medical practitioners**	Infectious Diseases Specialist	Disease Management/Curative Services	Inpatient care as including ward rounds	Direct_Patient_Care	Minutes per inpatient day	20	15	25
**2212 - Specialist medical practitioners**	Infectious Diseases Specialist	Disease Prevention	Infection prevention control	Support_Activity	Hours per task	1	2	3
**2212 - Specialist medical practitioners**	Infectious Diseases Specialist	Disease Management/Curative Services	Clinical meetings	Support_Activity	Hours per day	1	1	1
**2212 - Specialist medical practitioners**	Nephrologist	Disease Management/Curative Services	Outpatients Consultations as Assessment, diagnosis, treatment	Direct_Patient_Care	Minutes per patient (Old)	25	20	30

**2212 - Specialist medical practitioners**	Nephrologist	Disease Management/Curative Services	Inpatient care including ward rounds	Direct_Patient_Care	Minutes per inpatient day	20	15	25
**2212 - Specialist medical practitioners**	Nephrologist	Disease Management/Curative Services	Dialysis vein cannulation includes : femoral, subclavian, jugular	Direct_Patient_Care	Minutes per patient (New)	50	40	60
**2212 - Specialist medical practitioners**	Nephrologist	Disease Management/Curative Services	Clinical meetings	Support_Activity	Hours per day	1	1	1
**2212 - Specialist medical practitioners**	Neuro-Surgeon	Disease Management/Curative Services	Outpatient Consultation like Assessment, diagnosis, treatment	Direct_Patient_Care	Minutes per patient	25	20	30
**2212 - Specialist medical practitioners**	Neuro-Surgeon	Disease Management/Curative Services	Inpatient care including ward rounds	Direct_Patient_Care	Minutes per inpatient day	20	15	25
**2212 - Specialist medical practitioners**	Neuro-Surgeon	Disease Management/Curative Services	Major surgical procedures such as brain tumour excision, spinal decompression	Direct_Patient_Care	Minutes per patient	260	240	300
**2212 - Specialist medical practitioners**	Neuro-Surgeon	Disease Management/Curative Services	Bedside procedures as lumbar puncture, ventricular tap	Direct_Patient_Care	Minutes per task	25	20	30
**2212 - Specialist medical practitioners**	Neuro-Surgeon	Disease Management/Curative Services	Minor surgical procedures such as burr hole, ventriculostomy	Direct_Patient_Care	Minutes per task	80	90	120
**2212 - Specialist medical practitioners**	Neuro-Surgeon	Disease Management/Curative Services	Clinical meetings	Support_Activity	Hours per day	1	1	1
**2212 - Specialist medical practitioners**	Obstetrician & Gynaecologist	Disease Management/Curative Services	Outpatient and Antenatal Consultation as Assessment, diagnosis, treatment	Direct_Patient_Care	Minutes per patient	25	20	30

**2212 - Specialist medical practitioners**	Obstetrician & Gynaecologist	Disease Management/Curative Services	Inpatient Care including ward rounds	Direct_Patient_Care	Minutes per inpatient day	20	15	25
**2212 - Specialist medical practitioners**	Obstetrician & Gynaecologist	Disease Management/Curative Services	Bedside obstetric procedures such as pelvic ultrasound scan, cardiotocography, etc	Direct_Patient_Care	Minutes per patient	25	20	30
**2212 - Specialist medical practitioners**	Obstetrician & Gynaecologist	Disease Management/Curative Services	Bedside gynaecological procedures like insertion of intrauterine device, colposcopy, etc	Direct_Patient_Care	Minutes per patient	25	20	30
**2212 - Specialist medical practitioners**	Obstetrician & Gynaecologist	Disease Management/Curative Services	Minor obstetric surgery such as cervical cerclage, instrumental vaginal deliveries, repair of genital lacerations	Direct_Patient_Care	Minutes per patient	40	30	50
**2212 - Specialist medical practitioners**	Obstetrician & Gynaecologist	Disease Management/Curative Services	Minor gynaecological surgery such as marsupialisation	Direct_Patient_Care	Minutes per patient	40	30	50
**2212 - Specialist medical practitioners**	Obstetrician & Gynaecologist	Disease Management/Curative Services	Major obstetric surgery such as caesarean section, laparotomy for ruptured uterus	Direct_Patient_Care	Minutes per patient	50	45	60
**2212 - Specialist medical practitioners**	Obstetrician & Gynaecologist	Disease Management/Curative Services	Major gynaecological surgery such as myomectomy, hysterectomy, laparotomy for ectopic pregnancy, therapeutic lapararoscopy	Direct_Patient_Care	Minutes per patient	50	45	60
**2212 - Specialist medical practitioners**	Obstetrician & Gynaecologist	Disease Management/Curative Services	Clinical meetings	Support_Activity	Hours per week	1	1	1
**2212 - Specialist medical practitioners**	Ophthalmologist	Disease Management/Curative Services	Bedside procedures such as Refraction, Eye examination, Intravitreal injection	Direct_Patient_Care	Minutes per patient	25	20	30

**2212 - Specialist medical practitioners**	Ophthalmologist	Disease Management/Curative Services	Minor Opthalmic Surgery like Corneal scrapping,excisional-incisional biopsy,incision/curretage chalazion, Evisceration,anterior chamber washout	Direct_Patient_Care	Minutes per patient	40	30	50
**2212 - Specialist medical practitioners**	Ophthalmologist	Disease Management/Curative Services	Major Opthalmic Surgery like Cataracts,trabeculectomy, photocoagulation, keratoplasty, Reconstruction, orbototomy, pas planar vetrectomy, retinal detachment repair	Direct_Patient_Care	Minutes per patient	100	90	120
**2212 - Specialist medical practitioners**	Ophthalmologist	Detection/Diagnosis	Disability Assessment	Direct_Patient_Care	Minutes per patient	60	45	75
**2212 - Specialist medical practitioners**	Ophthalmologist	Disease Management/Curative Services	Outpatients consultations includes disability assessment, reviews, follow up	Direct_Patient_Care	Minutes per patient	25	20	30
**2212 - Specialist medical practitioners**	Orthopaedic Surgeon	Disease Management/Curative Services	Bedside Orthpaedic Procedures such as Plaster of paris application and removal,pin removal, Manipulation under anaesthesia	Direct_Patient_Care	Minutes per patient	25	20	30
**2212 - Specialist medical practitioners**	Orthopaedic Surgeon	Disease Management/Curative Services	Minor Orthopaedic surgery like Removal of foreign body, closed reduction, Decompression of an infection,Arthroscopy	Direct_Patient_Care	Minutes per patient	40	30	50

**2212 - Specialist medical practitioners**	Orthopaedic Surgeon	Disease Management/Curative Services	Major Orthopaedic surgery such as Amputation, Fasciotomy, Debridement, Arthrodesis, Nail Insertion,Knee/hip replacements, Tendon surgery, corrective osteotomies	Direct_Patient_Care	Minutes per patient	100	90	120
**2212 - Specialist medical practitioners**	Orthopaedic Surgeon	Disease Management/Curative Services	Inpatient Care like Ward rounds, Discharge of patients	Direct_Patient_Care	Minutes per inpatient day	20	15	25
**2212 - Specialist medical practitioners**	Orthopaedic Surgeon	Disease Management/Curative Services	Outpatient Consultations	Direct_Patient_Care	Minutes per patient	25	20	30
**2212 - Specialist medical practitioners**	Paediatric Surgeon	Disease Management/Curative Services	Bedside procedures such as Venepuncture, cannula insertion, Naso gastric tube insertion, Biopsy, CVP insertion, Intubation of patients	Direct_Patient_Care	Minutes per patient	25	20	30
**2212 - Specialist medical practitioners**	Paediatric Surgeon	Disease Management/Curative Services	Minor Paediatric Surgery like Umbilical and inguinal hernia repair	Direct_Patient_Care	Minutes per patient	40	30	50
**2212 - Specialist medical practitioners**	Paediatric Surgeon	Disease Management/Curative Services	Major Paediatric Surgery like Appendicectomy, Colonoscopy, laparotomy, Nephrectomy, Rectal Pull through	Direct_Patient_Care	Minutes per patient	100	90	120
**2212 - Specialist medical practitioners**	Paediatric Surgeon	Disease Management/Curative Services	In patient Care like Ward rounds, Discharges	Direct_Patient_Care	Minutes per patient	20	15	25
**2212 - Specialist medical practitioners**	Paediatric Surgeon	Disease Management/Curative Services	Outpatient consultations	Direct_Patient_Care	Minutes per patient	25	20	30

**2212 - Specialist medical practitioners**	Paediatrician	Disease Management/Curative Services	Bedside Paediatric Procedures like Venepuncture, Cannula. Biopsy, urinary-umbilical catherisation,pleurocentesis, peritoniocentesis, NGT insertion, Lumbar puncture	Direct_Patient_Care	Minutes per patient	25	20	30
**2212 - Specialist medical practitioners**	Paediatrician	Disease Management/Curative Services	Outpatient paediatric consultations as initial clerking,	Direct_Patient_Care	Minutes per patient	25	20	30
**2212 - Specialist medical practitioners**	Paediatrician	Disease Management/Curative Services	Exchange blood transfusions	Direct_Patient_Care	Hours per task	2	2	3
**2212 - Specialist medical practitioners**	Paediatrician	Disease Management/Curative Services	Paediatric critical care like Bone marrow, ward rounds, counselling	Direct_Patient_Care	Minutes per patient	75	60	90
**2212 - Specialist medical practitioners**	Paediatrician	Disease Management/Curative Services	Inpatient paediatric care like Dialysis,Resuscitation, Intubation, Bone marrow, ward rounds, counselling	Direct_Patient_Care	Minutes per inpatient day	20	15	25
**2212 - Specialist medical practitioners**	Paediatrician	Disease Management/Curative Services	Multidisciplinary Clinical Meeting	Support_Activity	Hours per week	1	1	1
**2212 - Specialist medical practitioners**	Pathologist	Detection/Diagnosis	Forensic pathology includes Attendance,Toxicology, DNA analysis,legal activities,forensic autopsies	Direct_Patient_Care	Hours per task	6	4	8

**2212 - Specialist medical practitioners**	Pathologist	Detection/Diagnosis	Laboratory diagnostic evaluation as Cytology, Immunohistochemistry, virology, histopathology, resection, microscopy, DNA testing, Bacteriology, Mycology, Parasitology, clinical Chemistry	Direct_Patient_Care	Minutes per patient	30	25	40
**2212 - Specialist medical practitioners**	Pathologist	Disease Management/Curative Services	General medical management	Direct_Patient_Care	Minutes per patient	70	60	80
**2212 - Specialist medical practitioners**	Pathologist	Disease Management/Curative Services	Multiple Disciplinary Team Meetings	Support_Activity	Hours per day	1	1	1
**2212 - Specialist medical practitioners**	Physician	Disease Management/Curative Services	Bed side procedures includes: Peritoniocentesis, lumbar punctures, biopsy, thoracocentesis, pericardiocentesis, chest drain insertion, femoral cathetor insertion	Direct_Patient_Care	Minutes per patient	25	20	30
**2212 - Specialist medical practitioners**	Physician	Disease Management/Curative Services	In patient care and ward rounds includes: clinical reviews and physical examination, management and discharge plan, consultations from other specialists	Direct_Patient_Care	Minutes per patient	20	15	25
**2212 - Specialist medical practitioners**	Physician	Disease Management/Curative Services	Outpatient Clinic and Follow Up	Direct_Patient_Care	Minutes per patient	25	20	30
**2212 - Specialist medical practitioners**	Physician	Disease Management/Curative Services	Clinical meetings	Support_Activity	Hours per week	1	1	1
**2212 - Specialist medical practitioners**	Plastic Surgeon	Disease Management/Curative Services	Consultations from outpatients	Direct_Patient_Care	Minutes per patient	25	20	30

**2212 - Specialist medical practitioners**	Plastic Surgeon	Disease Management/Curative Services	Inpatient care	Direct_Patient_Care	Minutes per inpatient day	20	15	25
**2212 - Specialist medical practitioners**	Plastic Surgeon	Disease Management/Curative Services	Bedside procedures as Venous access, Escharotomy, Wound dressing, Extradigit excision	Direct_Patient_Care	Minutes per patient	25	20	30
**2212 - Specialist medical practitioners**	Plastic Surgeon	Disease Management/Curative Services	Minor plastic surgery such as Wound debridement, Keloid excision, cleft lip repair, scar revision	Direct_Patient_Care	Minutes per patient	40	30	50
**2212 - Specialist medical practitioners**	Plastic Surgeon	Disease Management/Curative Services	Major plastic surgery such as Breast reduction, Abdominoplasty, Syndactyl release, Flap reconstruction, Contracture release, Tendon repair, Cleft palate repair	Direct_Patient_Care	Minutes per patient	100	90	120
**2212 - Specialist medical practitioners**	Plastic Surgeon	Disease Management/Curative Services	Clinical meeting	Support_Activity	Hours per month	1	1	1
**2212 - Specialist medical practitioners**	Psychiatrist	Health Promotion	Community outreach such as outreach for children, adoloscents and adults	Support_Activity	Hours per week	3	2	4
**2212 - Specialist medical practitioners**	Psychiatrist	Disease Management/Curative Services	Outpatient Consultation includes children, adoloscents and adults, clerking new patients	Direct_Patient_Care	Minutes per day	50	40	60
**2212 - Specialist medical practitioners**	Psychiatrist	Health Promotion	Caregiver psycho-education includes pschoeducation for children and adoloscents, adults and care givers	Support_Activity	Hours per week	2	2	2

**2212 - Specialist medical practitioners**	Psychiatrist	Disease Management/Curative Services	Inpatient care includes child, adoloscents, adults and correctional service in patients, medical liaison services	Direct_Patient_Care	Minutes per inpatient day	45	35	50
**2212 - Specialist medical practitioners**	Psychiatrist	Disease Management/Curative Services	Electroconvulsive therapy	Direct_Patient_Care	Minutes per patient	50	40	60
**2212 - Specialist medical practitioners**	Psychiatrist	Disease Management/Curative Services	Psychotherapy intervention	Direct_Patient_Care	Minutes per patient	50	40	60
**2212 - Specialist medical practitioners**	Psychiatrist	Disease Management/Curative Services	Neurodevelopmental and psychometric assessments / includes griffiths	Direct_Patient_Care	Minutes per patient	100	90	120
**2212 - Specialist medical practitioners**	Psychiatrist	Detection/Diagnosis	Forensic evaluation, report writing and legal proceedings / includes: evaluation of children/adolscents/ adults and court appearance	Direct_Patient_Care	Minutes per patient	100	90	120
**2212 - Specialist medical practitioners**	Radiation Oncologist	Disease Management/Curative Services	Administration of Chemotherapy	Direct_Patient_Care	Minutes per patient	60	50	80
**2212 - Specialist medical practitioners**	Radiation Oncologist	Disease Management/Curative Services	Outpatient Consultation(reviews, prescriptions, followup)	Direct_Patient_Care	Minutes per patient	35	30	40
**2212 - Specialist medical practitioners**	Radiation Oncologist	Disease Management/Curative Services	Brachy therapy includes radiotherapist oncologist	Direct_Patient_Care	Minutes per patient	60	50	80
**2212 - Specialist medical practitioners**	Radiation Oncologist	Disease Management/Curative Services	Inpatient Consultation such as Ward rounds and follow up (includes: radiotherapist oncologist)	Direct_Patient_Care	Minutes per inpatient day	20	15	25
**2212 - Specialist medical practitioners**	Radiation Oncologist	Disease Management/Curative Services	Radiotherapy includes radiotherapist oncologist	Direct_Patient_Care	minutes per patient	60	50	80

**2212 - Specialist medical practitioners**	Radiation Oncologist	Disease Management/Curative Services	Bedside procedure such as Paracentesis-lung/abdomen (includes: radiotherapist oncologist)	Direct_Patient_Care	minutes per patient	25	20	30
**3211 - Medical imaging and therapeutic equipment technicians**	Radiographer (Diagnostics and Therapy)	Detection/Diagnosis	X rays includes contrast based imaging	Direct_Patient_Care	Minutes per patient	25	20	30
**3211 - Medical imaging and therapeutic equipment technicians**	Radiographer (Diagnostics and Therapy)	Detection/Diagnosis	Mammography	Direct_Patient_Care	Minutes per patient	25	15	30
**3211 - Medical imaging and therapeutic equipment technicians**	Radiographer (Diagnostics and Therapy)	Detection/Diagnosis	Dexa Scans and Bone density scans	Direct_Patient_Care	Minutes per patient	25	15	30
**3211 - Medical imaging and therapeutic equipment technicians**	Radiographer (Diagnostics and Therapy)	Detection/Diagnosis	Ultrasound	Direct_Patient_Care	Minutes per patient	30	25	40
**3211 - Medical imaging and therapeutic equipment technicians**	Radiographer (Diagnostics and Therapy)	Detection/Diagnosis	Assisting in Echocardiography	Direct_Patient_Care	Minutes per patient	30	25	40
**3211 - Medical imaging and therapeutic equipment technicians**	Radiographer (Diagnostics and Therapy)	Detection/Diagnosis	CT Scan includes contrast based imaging	Direct_Patient_Care	Minutes per patient	50	40	60
**3211 - Medical imaging and therapeutic equipment technicians**	Radiographer (Diagnostics and Therapy)	Detection/Diagnosis	MRI Studies includes contrast based imaging	Direct_Patient_Care	Minutes per patient	50	40	60

**3211 - Medical imaging and therapeutic equipment technicians**	Radiographer (Diagnostics and Therapy)	Detection/Diagnosis	Fluroscopy	Direct_Patient_Care	Minutes per patient	50	40	60
**3211 - Medical imaging and therapeutic equipment technicians**	Radiographer (Diagnostics and Therapy)	Disease Management/Curative Services	Clinical meetings	Support_Activity	Hours per week	1	1	1
**3211 - Medical imaging and therapeutic equipment technicians**	Radiographer (Diagnostics and Therapy)	Disease Management/Curative Services	External Beam Radiotherapy and Administering radiotherapy	Direct_Patient_Care	Minutes per patient	50	40	60
**2212 - Specialist medical practitioners**	Radiologist	Disease Management/Curative Services	Consultations-Clinical Referrals of patients	Direct_Patient_Care	Minutes per task	25	20	30
**2212 - Specialist medical practitioners**	Radiologist	Detection/Diagnosis	Interpretation of radiological evaluations and reporting on images including CT scan, X-rays, MRI, ultrasound, fluoroscopy	Direct_Patient_Care	Minutes per patient	15	10	20
**2212 - Specialist medical practitioners**	Radiologist	Detection/Diagnosis	Ultrasound Examination	Direct_Patient_Care	Minutes per patient	25	20	30
**2212 - Specialist medical practitioners**	Radiologist	Detection/Diagnosis	Fluoroscopy Examination	Direct_Patient_Care	Minutes per patient	25	20	30
**2212 - Specialist medical practitioners**	Radiologist	Detection/Diagnosis	Radiology guided Procedures includng CT guided, ultrasound, fluoroscopy	Direct_Patient_Care	Minutes per patient	30	25	40
**2212 - Specialist medical practitioners**	Radiologist	Disease Management/Curative Services	Multidisciplinary Team meetings	Support_Activity	Hours per week	1	1	1
**2212 - Specialist medical practitioners**	Respiratory Physician	Disease Management/Curative Services	Outpatients care as assessment, Diagnosis ,treatment, referrals	Direct_Patient_Care	Minutes per patient (Old)	25	20	30

**2212 - Specialist medical practitioners**	Respiratory Physician	Disease Management/Curative Services	Inpatient care include ward rounds , bedside procedures , referrals	Direct_Patient_Care	Minutes per inpatient day	20	15	25
**2212 - Specialist medical practitioners**	Respiratory Physician	Disease Management/Curative Services	Clinical meetings	Direct_Patient_Care	Hours per week	1	1	1
**2212 - Specialist medical practitioners**	Respiratory Physician	Detection/Diagnosis	Bronchoscopy	Direct_Patient_Care	Minutes per patient (New)	50	40	60
**2212 - Specialist medical practitioners**	Rheumatologist	Disease Management/Curative Services	Outpatients consultations (reviews, prescriptions, follow up)	Direct_Patient_Care	Minutes per patient	40	30	50
**2212 - Specialist medical practitioners**	Rheumatologist	Disease Management/Curative Services	Inpatient care (ward rounds, prescriptions, bedside procedures)	Direct_Patient_Care	Minutes per inpatient day	20	15	25
**2212 - Specialist medical practitioners**	Rheumatologist	Disease Management/Curative Services	Clinical meetings	Direct_Patient_Care	Hours per week	1	1	1
**2212 - Specialist medical practitioners**	Urologist	Disease Management/Curative Services	Bedside procedures like Cystostomy, urethral catheterisation	Direct_Patient_Care	Minutes per patient	25	20	30
**2212 - Specialist medical practitioners**	Urologist	Disease Management/Curative Services	Inpatient care (Ward rounds, discharge of patient)	Direct_Patient_Care	Minutes per inpatient day	20	15	25
**2212 - Specialist medical practitioners**	Urologist	Disease Management/Curative Services	Outpatient consultation	Direct_Patient_Care	Minutes per patient	25	20	30
**2212 - Specialist medical practitioners**	Urologist	Disease Management/Curative Services	Minor urological surgery like Urethrotomy, Cystoscopy and biopsy, Hydrocelectomy, orchidectomy/orchidoplexy	Direct_Patient_Care	Minutes per patient	40	30	50

**2212 - Specialist medical practitioners**	Urologist	Disease Management/Curative Services	Major urological surgery such as TURP, Prostatectomy,Nephrectomy, Urethroplasty	Direct_Patient_Care	Minutes per patient	100	90	120
**2212 - Specialist medical practitioners**	Urologist	Disease Management/Curative Services	Clinical meetings	Support_Activity	Hours per week	1	1	1
**2262 - Pharmacists**	Clinical Pharmacist	Disease Management/Curative Services	Drug therapy (management,education, information)	Direct_Patient_Care	Minutes per patient	20	15	25
**2262 - Pharmacists**	Clinical Pharmacist	Disease Management/Curative Services	Pharmacovigilance(identification Adverse drug reactions and feedback)	Direct_Patient_Care	minutes per case	60	48	72
**2262 - Pharmacists**	Clinical Pharmacist	Disease Management/Curative Services	Clinical meetings(evaluation of patient management)	Direct_Patient_Care	Hours per week	60	60	120
**3221 - Nursing associate professionals**	Community Health Nurse	Disease Prevention	Monitoring disease patterns &Investigation of disease outbreaks	Support_Activity	Hours per week	10	9	12
**3221 - Nursing associate professionals**	Community Health Nurse	Health Promotion	Growth monitoring and Nutrition assesment	Direct_Patient_Care	Minutes per patient	12	10	15
**3221 - Nursing associate professionals**	Community Health Nurse	Disease Management/Curative Services	Out-patient care(consultation, Antenatal care,family planning, and management of minor ailments)	Direct_Patient_Care	Minutes per patient	12	10	15
**3221 - Nursing associate professionals**	Community Health Nurse	Health Promotion	Outreach services(family planning,immunisation)	Support_Activity	Days per week	1	1	2
**3221 - Nursing associate professionals**	Community Health Nurse	Health Promotion	Home visits	Support_Activity	Hours per month	10	8	14
**3221 - Nursing associate professionals**	Community Health Nurse	Disease Management/Curative Services	Documentation and coordination of care(daily and monthly reports, handover and take over)	Support_Activity	Hours per day	1	1	2

**3213 - Pharmaceutical technicians and assistants**	Pharmacy Technician	Disease Management/Curative Services	Assisting in house keeping duties(collect and maintain patient information,prepacking of medicines,restocking shelves)	Support_Activity	Hours per task	360	240	480
**3213 - Pharmaceutical technicians and assistants**	Pharmacy Technician	Health Promotion	Environmental cleaning	Support_Activity	Minutes per day	45	30	60
**3221 - Nursing associate professionals**	Enrolled Nurse	Disease Management/Curative Services	In-patient care and management(assisting RGNs,independent patient care)	Direct_Patient_Care	Minutes per patient	15	12	20
**3221 - Nursing associate professionals**	Enrolled Nurse	Disease Management/Curative Services	Out patient care and management(assisting RGNs,independent care)	Direct_Patient_Care	Minutes per patient	16	14	17
**3221 - Nursing associate professionals**	Enrolled Nurse	Disease Management/Curative Services	Assistive Documentation and coordination of care(daily and monthly reports, handover and take over)	Support_Activity	Hours per month	70	60	90
**2221 - Nursing professionals**	Intensive Care Nurse	Disease Management/Curative Services	Patient assessment and admission	Direct_Patient_Care	Minutes per patient	60	50	90
**2221 - Nursing professionals**	Intensive Care Nurse	Disease Management/Curative Services	Continuous inpatient care(patient management ,documentation and reporting)	Direct_Patient_Care	Minutes per inpatient day	60	50	80
**2221 - Nursing professionals**	Intensive Care Nurse	Disease Management/Curative Services	Equipment inventory(ventilator management, emergency trolley assessment and restocking)	Support_Activity	Minutes per day	60	50	80
**2221 - Nursing professionals**	Intensive Care Nurse	Disease Management/Curative Services	Patient and family education	Direct_Patient_Care	Minutes per day	60	60	90

**2221 - Nursing professionals**	Mental Health Nurse	Disease Management/Curative Services	Inpatient care(medication, ward rounds,wound dressing)	Direct_Patient_Care	Minutes per inpatient day	30	20	45
**2221 - Nursing professionals**	Mental Health Nurse	Health Promotion	Patient and family Health Education	Direct_Patient_Care	Minutes per task	45	30	60
**2221 - Nursing professionals**	Mental Health Nurse	Disease Management/Curative Services	Outpatient care(reviews,consultation, counselling)	Direct_Patient_Care	Minutes per patient	25	20	30
**2221 - Nursing professionals**	Mental Health Nurse	Disease Management/Curative Services	Mental Health Assessment(Pyschometric Examination)	Direct_Patient_Care	Minutes per patient	60	45	60
**2222 - Midwifery professionals**	Midwife	Health Promotion	Family planning services	Direct_Patient_Care	Minutes per patient	40	30	60
**2222 - Midwifery professionals**	Midwife	Health Promotion	Pre-conception care of patient	Direct_Patient_Care	Minutes per patient	30	20	40
**2222 - Midwifery professionals**	Midwife	Disease Management/Curative Services	Postnatal care (PNC) services	Direct_Patient_Care	Minutes per patient	30	25	40
**2222 - Midwifery professionals**	Midwife	Disease Management/Curative Services	Antenatal care (ANC) services	Direct_Patient_Care	Minutes per patient	50	40	60
**2222 - Midwifery professionals**	Midwife	Disease Management/Curative Services	Labor and delivery care	Direct_Patient_Care	Minutes per patient	180	120	230
**2222 - Midwifery professionals**	Midwife	Disease Management/Curative Services	Post partum care	Direct_Patient_Care	Minutes per patient	45	30	50
**2222 - Midwifery professionals**	Midwife	Disease Management/Curative Services	Care of the newborn	Direct_Patient_Care	Minutes per patient	30	20	40
**2222 - Midwifery professionals**	Midwife	Disease Management/Curative Services	Clinical Meetings(audit,unit conference)	Support_Activity	Hours per week	2	1	2

**2222 - Midwifery professionals**	Midwife	Disease Management/Curative Services	Documentation and coordination of care(daily and monthly reports, handover and take over)	Direct_Patient_Care	Hours per day	2	2	3
**2222 - Midwifery professionals**	Midwife	Disease Management/Curative Services	Emergency obstetric and neonatal care(basic and comprehensive)	Direct_Patient_Care	Minutes per task	40	35	45
**3221 - Nursing associate professionals**	Associate Nurse/Enrolled Nurse/Nursing Assistant	Disease Management/Curative Services	Inpatient management(grooming,bed pans,bedmaking,feeding)	Direct_Patient_Care	Minutes per inpatient day	135	120	160
**3221 - Nursing associate professionals**	Associate Nurse/Enrolled Nurse/Nursing Assistant	Disease Management/Curative Services	Assistive patient care(direct patient care under supervisions,vital observations, damp dusting)	Direct_Patient_Care	Minutes per patient	12	10	15
**3221 - Nursing associate professionals**	Associate Nurse/Enrolled Nurse/Nursing Assistant	Disease Management/Curative Services	Hospital Errands	Support_Activity	Hours per day	2	1	3
**2221 - Nursing professionals**	Nurse Anaesthetist	Disease Management/Curative Services	Peri operative management [major surgery]	Direct_Patient_Care	Minutes per patient	150	120	180
**2221 - Nursing professionals**	Nurse Anaesthetist	Disease Management/Curative Services	Peri operative management [minor surgery]	Direct_Patient_Care	Minutes per patient	60	45	90
**2221 - Nursing professionals**	Nurse Anaesthetist	Disease Management/Curative Services	Assesment of anaesthetic machine and equipment	Support_Activity	Minutes per day	90	60	120
**2221 - Nursing professionals**	Nurse Anaesthetist	Disease Management/Curative Services	Transfer of critically ill patients(with support from anaesthesiologists)	Direct_Patient_Care	Minutes per patient	25	20	40

**2221 - Nursing professionals**	Oncology Nurse	Disease Management/Curative Services	Patient assessment (diagnostic,history taking, examination)	Direct_Patient_Care	Minutes per patient	20	15	30
**2221 - Nursing professionals**	Oncology Nurse	Disease Management/Curative Services	Inpatient management(grooming,feeds,medicine administration,tests)	Direct_Patient_Care	Minutes per inpatient day	25	20	30
**2221 - Nursing professionals**	Oncology Nurse	Disease Management/Curative Services	Outpatients clinics(reviews)	Direct_Patient_Care	Minutes per patient	20	15	30
**2221 - Nursing professionals**	Oncology Nurse	Health Promotion	Patient and family Health education and counselling	Direct_Patient_Care	Hours per week	120	60	180
**2221 - Nursing professionals**	Oncology Nurse	Disease Management/Curative Services	Preparation of Environment and working Equipment	Support_Activity	Minutes per day	45	30	60
**2221 - Nursing professionals**	Operating Theatre Nurse	Disease Management/Curative Services	Peri operative management [minor surgery-Incision and drainage, suturing,debridement]	Direct_Patient_Care	Minutes per patient	90	60	120
**2221 - Nursing professionals**	Operating Theatre Nurse	Disease Management/Curative Services	Peri operative [major surgery-caesarian section, laparotomy]	Direct_Patient_Care	Minutes per patient	200	180	240
**2221 - Nursing professionals**	Operating Theatre Nurse	Disease Prevention	Preparation of working Environment and Equipment	Support_Activity	Minutes per day	45	40	60
**2221 - Nursing professionals**	Ophthalmic Nurse	Disease Management/Curative Services	Patient assessment (history taking,examination)	Direct_Patient_Care	Minutes per patient	20	15	25
**2221 - Nursing professionals**	Ophthalmic Nurse	Disease Management/Curative Services	Conducting diagnostic and therauptic procedures(eye vision testing ,eye irrigation,eye care,foreign body removal)	Direct_Patient_Care	Minutes per patient	20	15	25

**2221 - Nursing professionals**	Ophthalmic Nurse	Disease Management/Curative Services	Inpatient care(medication, ward rounds,wound dressing)	Direct_Patient_Care	Minutes per inpatient day	20	15	20
**2221 - Nursing professionals**	Ophthalmic Nurse	Disease Management/Curative Services	Peri operative care(pre,post Operative management)	Direct_Patient_Care	Minutes per patient	30	20	45
**2221 - Nursing professionals**	Ophthalmic Nurse	Health Promotion	Health Education and Promotion	Direct_Patient_Care	Minutes per patient	45	60	90
**2221 - Nursing professionals**	Ophthalmic Nurse	Disease Management/Curative Services	Preparation of Working environment and working equipment	Support_Activity	Minutes per day	60	30	120
**2221 - Nursing professionals**	Orthopaedic Nurse	Disease Management/Curative Services	Inpatient care(drug administration, ward rounds,traction management)	Direct_Patient_Care	Minutes per inpatient day	10	5	12
**2221 - Nursing professionals**	Orthopaedic Nurse	Disease Management/Curative Services	Counselling of patients and guardians on admission and discharge	Direct_Patient_Care	Minutes per patient	20	10	25
**2221 - Nursing professionals**	Orthopaedic Nurse	Disease Management/Curative Services	Outpatients review clinics(rehabilitation,Plaster of Paris application and removal)	Direct_Patient_Care	Minutes per patient	35	15	60
**2221 - Nursing professionals**	Orthopaedic Nurse	Disease Management/Curative Services	Peri operative care(pre,intra ,post Operative management)	Direct_Patient_Care	Minutes per patient	120	60	180
**2221 - Nursing professionals**	Orthopaedic Nurse	Disease Prevention	Preparation of working enviroment and equipment	Support_Activity	Minutes per day	90	60	120
**2221 - Nursing professionals**	Paediatric Nurse	Disease Management/Curative Services	Patient assessment(history taking, examination, decision)	Direct_Patient_Care	Minutes per patient	20	15	25
**2221 - Nursing professionals**	Paediatric Nurse	Disease Management/Curative Services	In patient management sick and well children (medication, ward rounds,vitals, transfusion)	Direct_Patient_Care	Minutes per task	30	20	45

**2221 - Nursing professionals**	Paediatric Nurse	Disease Management/Curative Services	Nutrition assessment and growth monitoring(weighing,height,milestones)	Direct_Patient_Care	Minutes per day	30	20	35
**2221 - Nursing professionals**	Paediatric Nurse	Disease Management/Curative Services	Documentation and coordination of care(daily and monthly reports, handover and take over)	Direct_Patient_Care	Hours per day	120	90	150
**2221 - Nursing professionals**	Paediatric Nurse	Disease Management/Curative Services	Clinical meetings(evaluation of patient management)	Support_Activity	Hours per week	1	1	2
**2221 - Nursing professionals**	Paediatric Nurse	Health Promotion	Family health education	Direct_Patient_Care	Minutes per patient	25	20	30
**2262 - Pharmacists**	Pharmacist	Disease Management/Curative Services	Prescription auditing and dispensing	Direct_Patient_Care	Minutes per patient	10	8	12
**2262 - Pharmacists**	Pharmacist	Health Promotion	Patient adherence counselling and education	Direct_Patient_Care	Minutes per patient	12	10	15
**2262 - Pharmacists**	Pharmacist	Health Promotion	Monitoring and Rational Medicines use	Direct_Patient_Care	Minutes per patient	5	3	8
**2262 - Pharmacists**	Pharmacist	Health Promotion	Clinical Meetings	Support_Activity	Hours per week	1	1	2
**2262 - Pharmacists**	Pharmacist	Disease Management/Curative Services	Supply Chain Management	Support_Activity	Hours per month	18	12	24
**2262 - Pharmacists**	Pharmacist	Disease Management/Curative Services	Pharmaco-vigilance	Support_Activity	Hours per week	4	3	6
**3213 - Pharmaceutical technicians and assistants**	Pharmacy Technician	Disease Management/Curative Services	Dispensing of Medicines	Direct_Patient_Care	Minutes per patient	6	5	8
**3213 - Pharmaceutical**	Pharmacy Technician	Disease Management/Curative Services	Odering and delivery of medicines to facilities	Support_Activity	Hours per month	18	12	24

**technicians and assistants**								
**3213 - Pharmaceutical technicians and assistants**	Pharmacy Technician	Disease Management/Curative Services	Prescription filing & Maintenance of inventory	Support_Activity	Hours per week	15	12	18
**3213 - Pharmaceutical technicians and assistants**	Pharmacy Technician	Disease Management/Curative Services	Medicine preparations(reconstitution and mixing)	Direct_Patient_Care	Minutes per patient	8	6	10
**3221 - Nursing associate professionals**	Primary Care Nurse	Disease Management/Curative Services	Inpatient care(medication, wound dressing)	Direct_Patient_Care	Minutes per inpatient day	60	40	90
**3221 - Nursing associate professionals**	Primary Care Nurse	Disease Management/Curative Services	Outpatient care (daily dressings, reviews, follow up)	Direct_Patient_Care	Minutes per patient	20	15	25
**3221 - Nursing associate professionals**	Primary Care Nurse	Health Promotion	Health Education and promotion(family planning, Voluntary medical male circumcision (VMMC)	Direct_Patient_Care	Minutes per patient	15	10	20
**3221 - Nursing associate professionals**	Primary Care Nurse	Disease Management/Curative Services	Stabilisation of patients and referral	Direct_Patient_Care	Minutes per patient	30	20	40
**3221 - Nursing associate professionals**	Primary Care Nurse	Health Promotion	Antenatal and post natal care services(immunisation, growth monitoring)	Direct_Patient_Care	Minutes per patient	30	25	40
**3221 - Nursing associate professionals**	Primary Care Nurse	Disease Management/Curative Services	Domicilliary and home visits(home nursing,geriatric)	Direct_Patient_Care	Minutes per patient	75	60	90
**2221 - Nursing professionals**	Registered General Nurse / State Certified Nurse	Disease Management/Curative Services	Patient assessment and diagnosis(physical examination,sample collection)	Direct_Patient_Care	Minutes per patient	15	12	20

**2221 - Nursing professionals**	Registered General Nurse / State Certified Nurse	Disease Management/Curative Services	Inpatient management(assessment, planning, implementation, evaluation, intervention)	Direct_Patient_Care	Minutes per inpatient day	90	80	120
**2221 - Nursing professionals**	Registered General Nurse / State Certified Nurse	Disease Management/Curative Services	Outpatient management(consultations,minor conditions)	Direct_Patient_Care	Minutes per patient	25	15	20
**2221 - Nursing professionals**	Registered General Nurse / State Certified Nurse	Disease Prevention	Immunisation/Vaccination(growth monitoring, Antenatal and postnatal services)	Direct_Patient_Care	Minutes per patient	15	10	20
**2221 - Nursing professionals**	Registered General Nurse / State Certified Nurse	Disease Management/Curative Services	Health education and promotion(family planning, Voluntary medical male circumcision)	Direct_Patient_Care	Minutes per patient	15	12	20
**2221 - Nursing professionals**	registered General Nurse / State Certified Nurse	Disease Management/Curative Services	Documentation and coordination of care(daily and monthly reports, handover and take over)	Direct_Patient_Care	Hours per day	2	1	3
**2221 - Nursing professionals**	Registered General Nurse / State Certified Nurse	Disease Management/Curative Services	Emergency care(basic life support,disaster and trauma nursing, first aide)	Direct_Patient_Care	Minutes per patient	30	25	35
**2221 - Nursing professionals**	Registered General Nurse / State Certified Nurse	Disease Management/Curative Services	Multidisciplinary clinical and coordination meetings(unit meetings,peer reviews,nursing audit)	Support_Activity	Hours per week	2	1	3
**2221 - Nursing professionals**	Renal Nurse	Disease Management/Curative Services	Pre- dialysis care (patient assessment, stabilisation)	Direct_Patient_Care	Minutes per patient	20	15	30
**2221 - Nursing professionals**	Renal Nurse	Disease Management/Curative Services	Haemodialysis (Pre-dialysis, intradialysis and post-dialysis care and monitoring)	Direct_Patient_Care	Minutes per patient	30	20	60

**2221 - Nursing professionals**	Renal Nurse	Disease Management/Curative Services	Post dialysis care(acutely) (assessment and stabilisation, medication administration)	Direct_Patient_Care	Minutes per patient	20	15	30
**2221 - Nursing professionals**	Renal Nurse	Disease Management/Curative Services	Renal clinic management(reviews, monitoring, admission)	Direct_Patient_Care	Minutes per patient	30	25	40
**2221 - Nursing professionals**	Renal Nurse	Disease Management/Curative Services	Documentation and coordination of care(daily and monthly reports, handover and take over)	Support_Activity	Hours per day	2	1	2
**2221 - Nursing professionals**	Renal Nurse	Disease Management/Curative Services	Assessment of dialysis machine and equipment	Support_Activity	Minutes per day	60	50	80
**2262 - Pharmacists**	Specialist Pharmacist (Radiopharmacist)	Disease Management/Curative Services	Inpatient care(ward rounds)	Direct_Patient_Care	Minutes per inpatient day	12	10	15
**2262 - Pharmacists**	Specialist Pharmacist (Radiopharmacist)	Disease Management/Curative Services	Outpatient care(reviews)	Direct_Patient_Care	Minutes per patient	15	12	18
**2262 - Pharmacists**	Specialist Pharmacist (Radiopharmacist)	Health Promotion	Health education,Counselling/adherence counselling	Direct_Patient_Care	Minutes per patient	12	10	15
**3253 - Community health workers**	Community health worker/Village health worker	Health Promotion	Coordination of community health activities(health education,community mobilisation,data collection)	Support_Activity	Days per week	1	1	2
**3253 - Community health workers**	Community health worker/Village health worker	Disease Prevention	Prevention infectious diseases(first aide, deworming ,distribution of prophylactic equipment)	Direct_Patient_Care	Minutes per task	25	20	30

**3253 - Community health workers**	Community health worker/Village health worker	Health Promotion	Home visits(register children under 5,report outbreaks,identify special children,mapping)	Direct_Patient_Care	Minutes per task	60	45	90
**3253 - Community health workers**	Community health worker/Village health worker	Disease Prevention	Environmental Health(environmental assessment)	Support_Activity	Hours per week	8	7	10
**3253 - Community health workers**	Community health worker/Village health worker	Health Promotion	Documentation and coordination of care(daily and monthly reports,referrals)	Support_Activity	Hours per month	20	18	22
**3253 - Community health workers**	Community health worker/Village health worker	Disease Prevention	Community dialogue(community leaders, health days)	Direct_Patient_Care	Hours per task	2	2	2
**3253 - Community health workers**	Community health worker/Village health worker	Disease Management/Curative Services	Screening and referral of patients to formal health care system	Direct_Patient_Care	Minutes per patient	60	45	70
**3259 - Health associate professionals not elsewhere classified**	Medical Laboratory Assistant	Clinical support	Workstation Preparation such as Disinfection/Sterilization (cleaning of laboratory hardware, housekeeping)	Support_Activity	Minutes per task	25	20	30
**3259 - Health associate professionals not elsewhere classified**	Medical Laboratory Assistant	Detection/Diagnosis	Preparation and patient instruction	Direct_Patient_Care	Minutes per patient	5	4	6
**3259 - Health associate professionals not elsewhere classified**	Medical Laboratory Assistant	Clinical support	Specimen Storage and Retention, Disposal of specimen	Support_Activity	Minutes per day	30	25	40

**3259 - Health associate professionals not elsewhere classified**	Medical Laboratory Assistant	Clinical support	Specimen Transport and Reception, result distribution	Support_Activity	Hours per day	2	1.5	3
**3212 - Medical and pathology laboratory technicians**	Medical Laboratory Scientist	Detection/Diagnosis	Patient Instruction and Preparation	Direct_Patient_Care	Minutes per patient	7	5	10
**3212 - Medical and pathology laboratory technicians**	Medical Laboratory Scientist	detection/Diagnosis	Hematology (bone marrow test, coagulation studies, Electrophoresis) including Interpretation)	Direct_Patient_Care	Minutes per task	60	45	90
**3212 - Medical and pathology laboratory technicians**	Medical Laboratory Scientist	detection/Diagnosis	Transfusion Science (Compatibility Testing/ Blood component separation, Investigation of Transfusion Reaction)	Direct_Patient_Care	Minutes per task	75	60	90
**3212 - Medical and pathology laboratory technicians**	Medical Laboratory Scientist	Clinical support	Expert Witness (paternity testing)	Direct_Patient_Care	Hours per month	4	3	6
**3212 - Medical and pathology laboratory technicians**	Medical Laboratory Scientist	detection/Diagnosis	Immunology (Serology) Interpretation	Direct_Patient_Care	Minutes per task	35	20	45
**3212 - Medical and pathology laboratory technicians**	Medical Laboratory Scientist	detection/Diagnosis	General and special Biochemistry (CSF analysis) Interpretation	Direct_Patient_Care	Minutes per task	40	30	50
**3212 - Medical and pathology laboratory technicians**	Medical Laboratory Scientist	detection/Diagnosis	Bacteriology, Parasitology, Virology, Mycology and Interpretation	Direct_Patient_Care	Minutes per task	100	90	120
**3212 - Medical and pathology laboratory technicians**	Medical Laboratory Scientist	detection/Diagnosis	Immunochemistry (Molecular/DNA/PCR Assay including Interpretation	Direct_Patient_Care	Minutes per task	40	30	45
**3212 - Medical and pathology laboratory technicians**	Medical Laboratory Scientist	detection/Diagnosis	Histology& Cytology including Interpretation/Report Validation & Writing	Direct_Patient_Care	Minutes per task	135	120	150

**3212 - Medical and pathology laboratory technicians**	Medical Laboratory Scientist	Clinical support	Specimen Storage and Retention/Disposal of spceimen	support_Activity	Minutes per task	12	10	15
**3212 - Medical and pathology laboratory technicians**	Medical Laboratory Technician	clinical support	Workstation Preparationand Disinfection/Sterilization (including equipment calibration)	support_Activity	Minutes per task	25	20	30
**3212 - Medical and pathology laboratory technicians**	Medical Laboratory Technician	detection/Diagnosis	Specimen Transport and Reception, Preparation, patient instruction	Direct_Patient_Care	Minutes per task	5	4	6
**3212 - Medical and pathology laboratory technicians**	Medical Laboratory Technician	detection/Diagnosis	Phlebotomy	Direct_Patient_Care	Minutes per patient	8	7	9
**3212 - Medical and pathology laboratory technicians**	Medical Laboratory Technician	detection/Diagnosis	Hematology (Full Blood Count, ESR, Sickling Film Comment, blood film for parasite, Electophoresis	Direct_Patient_Care	Minutes per patient	50	45	55
**3212 - Medical and pathology laboratory technicians**	Medical Laboratory Technician	detection/Diagnosis	Transfusion Science - Donor Testing/Compartibility Testing, Blood Component Separation including Interpretation	Direct_Patient_Care	Minutes per task	65	60	70
**3212 - Medical and pathology laboratory technicians**	Medical Laboratory Technician	detection/Diagnosis	Immunology (Serology) including Interpretation, Report Validation & Writing	Direct_Patient_Care	Minutes per task	22	30	35
**3212 - Medical and pathology laboratory technicians**	Medical Laboratory Technician	detection/Diagnosis	General Biochemistry (CSF Analysis) including Interpretation	Direct_Patient_Care	Minutes per task	40	30	50
**3212 - Medical and pathology laboratory technicians**	Medical Laboratory Technician	detection/Diagnosis	Bacteriology,Parasitology,Virology, Mycology including Interpretation	Direct_Patient_Care	Minutes per task	100	90	120
**3212 - Medical and pathology laboratory technicians**	Medical Laboratory Technician	detection/Diagnosis	Histology& Cytology including Interpretation	Direct_Patient_Care	Minutes per task	135	120	150

**3212 - Medical and pathology laboratory technicians**	Medical Laboratory Technician	clinical support	Specimen Storage and Retention/Disposal of spceimen	support_Activity	Minutes per task	5	4	6
**2265 - Dieticians and nutritionists**	Dietitian	Health Promotion	Maternal Nutrition (pre-pregancy, antenatal & postnatal care, breastfeeding)	Support_Activity	Minutes per task	30	15	45
**2265 - Dieticians and nutritionists**	Dietitian	Disease Management/Curative Services	Feeding in special circumstances and nutrition support in critical illness,parenteral and enteral nutrition support	Direct_Patient_Care	Minutes per inpatient day	25	20	35
**2265 - Dieticians and nutritionists**	Dietitian	Disease Management/Curative Services	Management of non communicable disease,nutrition support of communicable disease	Direct_Patient_Care	Minutes per patient	15	10	30
**2263 - Environmental and occupational health and hygiene professionals**	Environmental Health Officer	Health Promotion	Planning and evaluation for environmental health activities	Support_Activity	Hours per task	1	1	2
**2263 - Environmental and occupational health and hygiene professionals**	Environmental Health Officer	Health Promotion	Water and sanitation management (Carry out water sampling, Collection of water samples)	Support_Activity	Hours per task	6	4	8
**2263 - Environmental and occupational health and hygiene professionals**	Environmental Health Officer	Health Promotion	Educate communities on disease prevention	Direct_Patient_Care	Hours per task	2	1	3

**2263 - Environmental and occupational health and hygiene professionals**	Environmental Health Officer	Health Promotion	Inspection of trading premises and non trading premises and Prosecution of public health offenders	Support_Activity	Hours per week	3	2	4
**2263 - Environmental and occupational health and hygiene professionals**	Environmental Health Officer	Disease Prevention	Food inspection (Carry out food sampling, Meat inspection)	Support_Activity	Hours per week	3	2	4
**2263 - Environmental and occupational health and hygiene professionals**	Environmental Health Officer	Disease Prevention	Inspection of shipment consignments at ports of entry	Support_Activity	Hours per task	3	4	5
**2263 - Environmental and occupational health and hygiene professionals**	Environmental Health Officer	Disease Prevention	Examination of building plans for approval	Support_Activity	Hours per task	2	1	3
**2263 - Environmental and occupational health and hygiene professionals**	Environmental Health Officer	Disease Prevention	Attending weekly disease surveillance meetings and Community diagnosis	Support_Activity	Hours per week	1.5	1	2
**2263 - Environmental and occupational health and hygiene professionals**	Environmental Health Officer	Disease Prevention	Training public on health requirements for food handlers, latrine builders etc)	Support_Activity	Hours per month	4	3	6
**2263 - Environmental and occupational health and**	Environmental Health Officer	Disease Prevention	Parasites and pests control/Vector control and mapping	Support_Activity	Hours per task	4	3	5

**hygiene professionals**								
**2263 - Environmental and occupational health and hygiene professionals**	Environmental Health Technician	Health Promotion	Planning and evaluation for environmental health activities	Support_Activity	Hours per task	1	1	2
**2263 - Environmental and occupational health and hygiene professionals**	Environmental Health Technician	Health Promotion	Water and sanitation management (Carry out water sampling, Collection of water samples)	Support_Activity	Hours per task	6	4	8
**2263 - Environmental and occupational health and hygiene professionals**	Environmental Health Technician	Disease Prevention	Educate communities on disease prevention	Support_Activity	Hours per task	2	1	3
**2263 - Environmental and occupational health and hygiene professionals**	Environmental Health Technician	Health Promotion	Inspection of trading premises and non trading premises and Prosecution of public health offenders	Support_Activity	Hours per day	3	2	4
**2263 - Environmental and occupational health and hygiene professionals**	Environmental Health Technician	Disease Prevention	Food inspection (Carry out food sampling, Meat inspection)	Support_Activity	Hours per week	3	2	4
**2263 - Environmental and occupational health and hygiene professionals**	Environmental Health Technician	Disease Prevention	Inspection of shipment consignments at ports of entry	Support_Activity	Hours per task	3	4	5

**2263 - Environmental and occupational health and hygiene professionals**	Environmental Health Technician	Disease Prevention	Examination of building plans for approval	Support_Activity	Hours per task	2	1	3
**2263 - Environmental and occupational health and hygiene professionals**	Environmental Health Technician	Disease Prevention	Attending weekly disease surveillance meetings and Community diagnosis	Support_Activity	Hours per week	1.5	1	2
**2263 - Environmental and occupational health and hygiene professionals**	Environmental Health Technician	Disease Prevention	Training public on health requirements for food handlers, latrine builders etc)	Support_Activity	Hours per month	4	3	6
**2263 - Environmental and occupational health and hygiene professionals**	Environmental Health Technician	Disease Prevention	Parasites and pests control/Vector control and mapping	Support_Activity	Hours per task	4	3	5
**3252 - Medical records and health information technicians**	Health Information Officer	Clinical support	Disease surveillance including Daily reporting of outbreak and campaign data, Coordinate weekly data reporting from Districts,Weekly data analysis for detection of outbreaks, Data entry or various reports, Monthly DHIS2 report production, data verification)	Support_Activity	Hours per day	4	3	5
**3252 - Medical records and health information technicians**	Health Information Officer	Clinical support	Conduct Trainings	Support_Activity	Hours per task	1	1	1

**3252 - Medical records and health information technicians**	Health Information Officer	Clinical support	Weekly, Monthly and Quarterly surveillance meetings	Support_Activity	Hours per week	1	1	1
**2269 - Health professionals not elsewhere classified**	Health Promoter/Health Educator	Health Promotion	Communtiy health promotion activities including community mobilization, assessing knowledge gaps, managing programmes, implementing of health promotion programmes, conducting health campaigns)	Support_Activity	Hours per task	2	1	3
**2269 - Health professionals not elsewhere classified**	Health Promoter/Health Educator	Health Promotion	Health education including development and distribution of IEC materials	Support_Activity	Hours per task	1	1	1
**2269 - Health professionals not elsewhere classified**	Health Promoter/Health Educator	Health Promotion	Training health workers on health promotion	Support_Activity	Hours per task	1	1	1
**2269 - Health professionals not elsewhere classified**	Health Promoter/Health Educator	Health Promotion	Meetings including conducting advocacy meetings	Support_Activity	Hours per week	1	1	1
**2265 - Dieticians and nutritionists**	Nutritionist	Detection/Diagnosis	Nutrition screening and assessment	Direct_Patient_Care	Minutes per patient	7	5	10
**2265 - Dieticians and nutritionists**	Nutritionist	Disease Management/Curative Services	Management of severe acute malnutrition	Direct_Patient_Care	Minutes per patient	60	50	80
**2265 - Dieticians and nutritionists**	Nutritionist	Disease Prevention	Infant and young children feeding counselling, nutrition counselling/education/management of micronutrient deficiencies	Direct_Patient_Care	Minutes per task	30	15	45

**2265 - Dieticians and nutritionists**	Nutritionist	Clinical support	Nutrition outreaches	Support_Activity	Hours per task	4	3	5
**2266 - Audiologists and speech therapists**	Audiologist	Disease Prevention	Health education	Direct_Patient_Care	Minutes per patient	7	5	10
**2266 - Audiologists and speech therapists**	Audiologist	Detection/Diagnosis	Patient Screening	Support_Activity	Hours per month	8	6	10
**2266 - Audiologists and speech therapists**	Audiologist	Disease Management/Curative Services	Referral of patients	Direct_Patient_Care	Minutes per patient	10	5	15
**2266 - Audiologists and speech therapists**	Audiologist	Detection/Diagnosis	Hearing impairement assessment and hearing testing	Direct_Patient_Care	Minutes per patient	20	15	30
**2266 - Audiologists and speech therapists**	Audiologist	Rehabilitaion	Hearing aid device prescription and fitting	Direct_Patient_Care	Minutes per patient	20	15	25
**2266 - Audiologists and speech therapists**	Audiologist	Rehabilitaion	Family counselling	Direct_Patient_Care	Minutes per patient	20	15	30
**2266 - Audiologists and speech therapists**	Audiologist	Disease Management/Curative Services	Treatment plan of patients	Direct_Patient_Care	Minutes per patient	20	15	25
**2149 - Engineering professionals not elsewhere classified**	Biomedical Equipment Technician	Clinical support	Perform quartely Medical Equipment Inventory	Support_Activity	Hours per task	12	8	16
**2149 - Engineering professionals not elsewhere classified**	Biomedical Equipment Technician	Clinical support	Visit clinical departments to check status of Biomedical equipment/ Perform electrical safety inspections on Biomedical Equipment	Support_Activity	Minutes per day	30	15	45

**2149 - Engineering professionals not elsewhere classified**	Biomedical Equipment Technician	Clinical support	Installation, maintenance and repair of Biomedical equipment	Support_Activity	Minutes per task	60	45	90
**2149 - Engineering professionals not elsewhere classified**	Biomedical Equipment Technician	Clinical support	Calibration of Biomedical equipment	Support_Activity	Minutes per task	20	10	30
**2149 - Engineering professionals not elsewhere classified**	Biomedical Equipment Technician	Clinical support	Assisting and training end-users on the use of Biomedical equipment	Support_Activity	Minutes per task	45	30	60
**2149 - Engineering professionals not elsewhere classified**	Biomedical Equipment Technician	Clinical support	Acceptance testing of Biomedical equipment	Support_Activity	Minutes per task	45	30	60
**3256 - Medical assistants**	Clinical Officer/Physician Assistant	Disease Management/Curative Services	Minor surgeries e.g incision and drainage, suturing lacerations	Direct_Patient_Care	Minutes per patient	40	30	50
**3256 - Medical assistants**	Clinical Officer/Physician Assistant	Disease Management/Curative Services	Bedside procedures (e.g Naso-gastric tube insertion, Joint tapping, pleuroscentesis, paracentesis, Lumbar puncture, Catheter insertion and removal,gastric lavage ,(transurethral suprapubic)	Direct_Patient_Care	Minutes per patient	25	20	30
**3256 - Medical assistants**	Clinical Officer/Physician Assistant	Disease Management/Curative Services	Out patient consultation	Direct_Patient_Care	Minutes per patient	25	20	30

**3256 - Medical assistants**	Clinical Officer/Physician Assistant	Disease Management/Curative Services	Inpatient care (including ward rounds, admission, discharge, referral, notification of death, rescuscitation)	Direct_Patient_Care	Minutes per inpatient day	20	15	25
**3256 - Medical assistants**	Clinical Officer/Physician Assistant	Disease Management/Curative Services	Other non-complex surgical procedures	Direct_Patient_Care	Minutes per patient	60	45	120
**3256 - Medical assistants**	Clinical Officer/Physician Assistant	Disease Management/Curative Services	Clinical meetings	Support_Activity	Hours per week	1	1	2
**2634 - Psychologists**	Clinical Psychologist	Detection/Diagnosis	Psycho-assessment	Direct_Patient_Care	Minutes per patient	8	7	10
**2634 - Psychologists**	Clinical Psychologist	Disease Management/Curative Services	Treatment plan for patient	Direct_Patient_Care	Minutes per patient	10	8	12
**2634 - Psychologists**	Clinical Psychologist	Detection/Diagnosis	Psychometric testing of patients	Direct_Patient_Care	Minutes per patient	15	10	18
**2634 - Psychologists**	Clinical Psychologist	Disease Management/Curative Services	Monitoring and stabilising	Direct_Patient_Care	Minutes per patient	13	10	15
**2634 - Psychologists**	Clinical Psychologist	Disease Management/Curative Services	Psychotherapy	Direct_Patient_Care	Minutes per patient	70	60	75
**2634 - Psychologists**	Clinical Psychologist	Disease Management/Curative Services	Counselling of patients and guardians on admission and discharge	Direct_Patient_Care	Minutes per patient	70	60	75
**2634 - Psychologists**	Clinical Psychologist	Detection/Diagnosis	Psycho Forensic assessments	Direct_Patient_Care	Minutes per patient	75	60	90
**2634 - Psychologists**	Clinical Psychologist	Health Promotion	Patient education	Support_Activity	Minutes per day	20	30	40

**2265 - Dieticians and nutritionists**	Dental Surgery Assistant	Clinical support	Charting and documentation	Direct_Patient_Care	Minutes per patient	8	6	10
**3251 - Dental assistants and therapists**	Dental Surgery Assistant	Clinical support	Exposing and processing radiographs	Direct_Patient_Care	Minutes per task	8	6	10
**3251 - Dental assistants and therapists**	Dental Surgery Assistant	Clinical support	Assisting dental operator	Direct_Patient_Care	Minutes per patient	38	30	45
**3251 - Dental assistants and therapists**	Dental Surgery Assistant	Clinical support	Sitting and preparation of patients	Direct_Patient_Care	Minutes per patient	20	15	25
**3251 - Dental assistants and therapists**	Dental Surgery Assistant	Clinical support	Education and instruction	Direct_Patient_Care	Minutes per day	20	15	30
**3251 - Dental assistants and therapists**	Dental Surgery Assistant	Clinical support	Sterilisation of instruments (includes scrubbing and packing for sterilization)	Support_Activity	Minutes per task	53	45	60
**3251 - Dental assistants and therapists**	Dental Surgery Assistant	Clinical support	Disinfection of working environment	Support_Activity	Hours per day	2.5	2	3
**3251 - Dental assistants and therapists**	Dental Technician	Clinical support	Developing study models	Support_Activity	Minutes per task	15	10	20
**3251 - Dental assistants and therapists**	Dental Technician	Clinical support	Making mouth guards + Bleaching trays	Direct_Patient_Care	Minutes per task	25	20	30
**3251 - Dental assistants and therapists**	Dental Technician	Clinical support	Denture tooth addition and denture repair	Direct_Patient_Care	Minutes per task	25	20	30
**3251 - Dental assistants and therapists**	Dental Technician	Clinical support	Fabrication of Orthodontic Appliances (including obturators)	Direct_Patient_Care	Hours per task	3.5	3	4
**3251 - Dental assistants and therapists**	Dental Technician	Clinical support	Denture making and fabrication - (Acrylic + Flexible/valplast + Chrome Cobalt)	Direct_Patient_Care	Minutes per task	180	120	240

**3251 - Dental assistants and therapists**	Dental Technician	Clinical support	Fabrication of Implant supported dentures	Direct_Patient_Care	Minutes per task	225	150	300
**3251 - Dental assistants and therapists**	Dental Technician	Clinical support	Fabrication of Dental Crowns and Bridges	Direct_Patient_Care	Minutes per task	240	180	300
**3251 - Dental assistants and therapists**	Dental Therapist	Disease Management/Curative Services	Prescribing medication	Direct_Patient_Care	Minutes per patient	3.5	3	5
**3251 - Dental assistants and therapists**	Dental Therapist	Disease Management/Curative Services	Referral of patients	Direct_Patient_Care	Minutes per patient	15	10	20
**3251 - Dental assistants and therapists**	Dental Therapist	Detection/Diagnosis	Oral health assessment, screening, check up	Direct_Patient_Care	Minutes per patient	17	15	20
**3251 - Dental assistants and therapists**	Dental Therapist	Disease Prevention	Oral health education	Direct_Patient_Care	Minutes per patient	17	15	20
**3251 - Dental assistants and therapists**	Dental Therapist	Disease Prevention	Preventive management of dental caries and periodontal disease e.g. Fissure sealant, topical fluoride, oral prophylaxis, polishing	Direct_Patient_Care	Minutes per patient	12	10	15
**3251 - Dental assistants and therapists**	Dental Therapist	Detection/Diagnosis	Taking and developing dental radiograph	Direct_Patient_Care	Minutes per patient	8	5	10
**3251 - Dental assistants and therapists**	Dental Therapist	Disease Management/Curative Services	Therapeutic management of dental caries e.g. extraction, fillings, incision and drainage, dry socket management	Direct_Patient_Care	Minutes per patient	23	15	30
**3251 - Dental assistants and therapists**	Dental Therapist	Disease Management/Curative Services	Therapeutic management of periodontal diseases e.g. Scaling, root planning	Direct_Patient_Care	Minutes per patient	38	30	45
**3251 - Dental assistants and therapists**	Dental Therapist	Disease Management/Curative Services	Management of minor emergencies e.g. tooth fracture, tooth avulsion,	Direct_Patient_Care	Minutes per patient	25	20	30

**3251 - Dental assistants and therapists**	Dental Therapist	Health Promotion	Conducting Health promotion and outreach activities	Support_Activity	Days per week	1	1	3
**1344 - Social welfare managers**	Medical Social Worker	Disease Management/Curative Services	Ward rounds	Direct_Patient_Care	Minutes per inpatient day	17	15	20
**1344 - Social welfare managers**	Medical Social Worker	Detection/Diagnosis	Patient assessment (inpatient and out patient)	Direct_Patient_Care	Minutes per patient	45	30	60
**1344 - Social welfare managers**	Medical Social Worker	Health Promotion	Health education	Direct_Patient_Care	Minutes per task	50	40	60
**1344 - Social welfare managers**	Medical Social Worker	Rehabilitaion	Counselling patients and relatives (includes supporting vulnerable groups/individuals)	Direct_Patient_Care	Minutes per patient	50	40	60
**1344 - Social welfare managers**	Medical Social Worker	Rehabilitaion	Placement of individuals from vulnerable families (include adults, older persons, children, refugees)	Support_Activity	Minutes per task	120	100	150
**1344 - Social welfare managers**	Medical Social Worker	Rehabilitaion	Conducting home visits	Support_Activity	Minutes per patient	50	40	60
**1344 - Social welfare managers**	Medical Social Worker	Rehabilitaion	Linking and referring patients to resources (include coordination of patient discharge)	Support_Activity	Minutes per task	15	10	20
**1344 - Social welfare managers**	Medical Social Worker	Clinical support	Tracing of relatives	Support_Activity	Minutes per task	40	30	50
**2230 - Traditional and complementary medicine professionals**	Natural Therapist	Detection/Diagnosis	Patient Assessment and treatment (e.g. homeopathy, Ayuvedic, Moxibustion, Naturolpathic, Acupuncture, Cupping)	Direct_Patient_Care	Minutes per patient	10	8	15

**2230 - Traditional and complementary medicine professionals**	Natural Therapist	Disease Management/Curative Services	Refer patients to other health professionals	Direct_Patient_Care	Minutes per patient	10	8	15
**2230 - Traditional and complementary medicine professionals**	Natural Therapist	Disease Management/Curative Services	Preparation and Administration of herbal medicine	Direct_Patient_Care	Minutes per patient	15	10	17
**2230 - Traditional and complementary medicine professionals**	Natural Therapist	Disease Management/Curative Services	Health Education	Direct_Patient_Care	Minutes per patient	20	15	25
**2269 - Health professionals not elsewhere classified**	Occupational Therapist	Detection/Diagnosis	Assessment of patients	Direct_Patient_Care	Minutes per patient	15	10	20
**2269 - Health professionals not elsewhere classified**	Occupational Therapist	Disease Management/Curative Services	Discharge and referral of patients	Direct_Patient_Care	Minutes per patient	10	8	15
**2269 - Health professionals not elsewhere classified**	Occupational Therapist	Detection/Diagnosis	Screening of patients	Support_Activity	Hours per month	8	6	10
**2269 - Health professionals not elsewhere classified**	Occupational Therapist	Disease Management/Curative Services	Inpatient treatment including counselling	Direct_Patient_Care	Minutes per patient	50	45	60
**2269 - Health professionals not elsewhere classified**	Occupational Therapist	Rehabilitaion	Wheel chair prescription and fitting, training	Direct_Patient_Care	Minutes per patient	75	60	90
**2269 - Health professionals not elsewhere classified**	Occupational Therapist	Rehabilitaion	Environment modification	Support_Activity	Minutes per task	60	50	70
**2269 - Health professionals not elsewhere classified**	Occupational Therapist	Health Promotion	Health education	Direct_Patient_Care	Minutes per task	45	40	60

**2267 - Optometrists and ophthalmic opticians**	Optometrist	Detection/Diagnosis	Screening (including in-office and community outreach to examine the eyes for assessment and diagnosis)	Support_Activity	Hours per month	8	6	10
**2267 - Optometrists and ophthalmic opticians**	Optometrist	Rehabilitaion	Prescription of visual aids	Direct_Patient_Care	Minutes per patient	30	24	36
**2267 - Optometrists and ophthalmic opticians**	Optometrist	Disease Management/Curative Services	Counselling of patients and guardians on admission and discharge	Direct_Patient_Care	Minutes per patient	10	8	15
**2267 - Optometrists and ophthalmic opticians**	Optometrist	Health Promotion	Health education (including group education)	Direct_Patient_Care	Minutes per task	50	45	60
**3214 - Medical and dental prosthetic technicians**	Orthopaedic Assistant	Rehabilitaion	Assist in fabrication (including design , finishing, fitting) of prosthetics products and corrective orthopedic shoes	Direct_Patient_Care	Hours per task	72	48	96
**3214 - Medical and dental prosthetic technicians**	Orthopaedic Assistant	Rehabilitaion	Assist in fabrication (including design , finishing and fitting of assistive orthotics products	Direct_Patient_Care	Minutes per task	72	48	96
**3214 - Medical and dental prosthetic technicians**	Orthopaedic Assistant	Rehabilitaion	Assist in repairs of assistive products (prosthetics, orthotics, walking aids, shoes)	Direct_Patient_Care	Hours per day	2	2	3
**3214 - Medical and dental prosthetic technicians**	Orthopaedic Assistant	Rehabilitaion	Fabrication of walking aids such as crutches, walking frame and quadipod	Direct_Patient_Care	Days per week	1	1	2
**3214 - Medical and dental prosthetic technicians**	Orthopaedic Technologist	Rehabilitaion	Fabrication (including design ,finishing, fitting) and training to use of prosthetics products and corrective orthopedic shoes	Direct_Patient_Care	Minutes per task	72	48	96

**3214 - Medical and dental prosthetic technicians**	Orthopaedic Technologist	Rehabilitaion	Consultations (assessment, prescription of assistive products, follow up reviews, referrals)	Direct_Patient_Care	Minutes per patient	30	25	40
**3214 - Medical and dental prosthetic technicians**	Orthopaedic Technologist	Rehabilitaion	Fabrication (including design , finishing and fitting) and training on the use of orthotics products	Direct_Patient_Care	Minutes per patient	72	48	96
**3214 - Medical and dental prosthetic technicians**	Orthopaedic Technologist	Rehabilitaion	Repairs of assistive products (prosthetics, orthotics, walking aids, shoes)	Direct_Patient_Care	Hours per task	2	2	3
**2264 - Physiotherapists**	Physiotherapist	Rehabilitaion	Assessment and treatment of out-patient, including gait training, electrophysical agents, exercises	Direct_Patient_Care	Minutes per patient	45	30	60
**2264 - Physiotherapists**	Physiotherapist	Rehabilitaion	Discharge and referral of patients	Direct_Patient_Care	Minutes per patient	10	8	15
**2264 - Physiotherapists**	Physiotherapist	Rehabilitaion	Screening (such as outreach)	Support_Activity	Hours per month	8	6	10
**2264 - Physiotherapists**	Physiotherapist	Rehabilitaion	Measurement, prescription, fitting and training on use of assistive products	Direct_Patient_Care	Minutes per patient	30	20	40
**2264 - Physiotherapists**	Physiotherapist	Rehabilitaion	In-patient treatment, including assessment, exercises, gait training and transfer	Direct_Patient_Care	Minutes per patient	30	25	40
**2264 - Physiotherapists**	Physiotherapist	Rehabilitaion	Home visits	Direct_Patient_Care	Minutes per patient	60	45	75
**2264 - Physiotherapists**	Physiotherapist	Disease Management/Curative Services	Health education	Direct_Patient_Care	Minutes per patient	20	15	30

**3214 - Medical and dental prosthetic technicians**	Rehabilitation Technician	Detection/Diagnosis	Assessment of patients	Direct_Patient_Care	Minutes per patient	10	15	20
**3214 - Medical and dental prosthetic technicians**	Rehabilitation Technician	Disease Management/Curative Services	Discharge and referral	Direct_Patient_Care	Minutes per patient	10	8	15
**3214 - Medical and dental prosthetic technicians**	Rehabilitation Technician	Rehabilitaion	Treatment plan	Direct_Patient_Care	Minutes per patient	45	30	60
**3214 - Medical and dental prosthetic technicians**	Rehabilitation Technician	Detection/Diagnosis	Patient Screening	Support_Activity	Hours per month	8	6	10
**3214 - Medical and dental prosthetic technicians**	Rehabilitation Technician	Rehabilitaion	Home visits	Direct_Patient_Care	Minutes per patient	60	50	75
**3214 - Medical and dental prosthetic technicians**	Rehabilitation Technician	Health Promotion	Health education	Direct_Patient_Care	Minutes per task	60	50	75
**2266 - Audiologists and speech therapists**	Speech Therapist	Detection/Diagnosis	Patient Screening	Support_Activity	Hours per month	8	6	10
**2266 - Audiologists and speech therapists**	Speech Therapist	Disease Management/Curative Services	Health Education	Direct_Patient_Care	Minutes per patient	20	15	25
**2266 - Audiologists and speech therapists**	Speech Therapist	Detection/Diagnosis	Patient Assessment	Direct_Patient_Care	Minutes per patient	40	30	50
**2266 - Audiologists and speech therapists**	Speech Therapist	Rehabilitaion	In patient Treatment	Direct_Patient_Care	Minutes per patient	30	20	40
**2266 - Audiologists and speech therapists**	Speech Therapist	Rehabilitaion	Out patient treatment	Direct_Patient_Care	Minutes per patient	40	30	45
**2266 - Audiologists and speech therapists**	Speech Therapist	Rehabilitaion	Follow up of patients	Direct_Patient_Care	Minutes per patient	45	30	45

**2269 - Health professionals not elsewhere classified**	Occupational Therapist	Disease Management/Curative Services	Out patient treatment including counselling	Direct_Patient_Care	Minutes per patient	50	45	60
**2269 - Health professionals not elsewhere classified**	Occupational Therapist	Rehabilitaion	Fabrication of hand orthotics	Direct_Patient_Care	Minutes per task	120	100	130
**2267 - Optometrists and ophthalmic opticians**	Optometrist	Disease Management/Curative Services	Follow up/review and monitoring	Direct_Patient_Care	Minutes per patient	10	8	15
**2267 - Optometrists and ophthalmic opticians**	Optometrist	Disease Management/Curative Services	Patient Referral of patients	Direct_Patient_Care	Minutes per patient	10	8	15
**2262 - Pharmacists**	Clinical Pharmacist	Disease Management/Curative Services	Prescription audit and dispensing medicines	Direct_Patient_Care	Minutes per patient	15	10	20
**2264 - Physiotherapists**	Physiotherapist	Health Promotion	Outreach - facility health based and non-health facility based	Support_Activity	Hours per month	3	2	4
**2266 - Audiologists and speech therapists**	Speech Therapist	Disease Management/Curative Services	Referral of patients	Direct_Patient_Care	Minutes per patient	10	8	15
**2266 - Audiologists and speech therapists**	Speech Therapist	Rehabilitaion	Prescription of corrective device including training on use	Direct_Patient_Care	Minutes per task	10	8	15
**2221 - Nursing professionals**	Registered General Nurse / State Certified Nurse	Disease Management/Curative Services	Haemodialysis (Pre-dialysis, intradialysis and post-dialysis care and monitoring)	Direct_Patient_Care	Minutes per task	80	70	90
**2222 - Midwifery professionals**	Midwife	Disease Management/Curative Services	Routine Inpatient Care for sick pregnant women	Direct_Patient_Care	Minutes per inpatient day	70	60	90
**2221 - Nursing professionals**	Community Health Nurse	Disease Prevention	Immunisation/Vaccination(growth monitoring, Antenatal and Postnatal services,)	Direct_Patient_Care	Minutes per patient	15	10	20

**3221 - Nursing associate professionals**	Associate Nurse/Enrolled Nurse/Nursing Assistant	Disease Management/Curative Services	Outpatient management(consultations,minor conditions)	Direct_Patient_Care	Minutes per patient	15	10	20
**3221 - Nursing associate professionals**	Associate Nurse/Enrolled Nurse/Nursing Assistant	Disease Management/Curative Services	Patient assessment and diagnosis(physical examination,sample collection)	Direct_Patient_Care	Minutes per patient	15	10	20
**2212 - Specialist medical practitioners**	Cardiologist	Disease Management/Curative Services	Limited invasive procedures(pacemaker insertion, defribrillator implants)	Direct_Patient_Care	Hours per task	2.5	2	3
**2212 - Specialist medical practitioners**	Cardiologist	Disease Management/Curative Services	Cardiac catheterisation	Direct_Patient_Care	Hours per task	2.5	2	3
**2212 - Specialist medical practitioners**	Cardiologist	Detection/Diagnosis	Cardiac investigations and interpretation(Electrocardiogram, angiography)	Direct_Patient_Care	Minutes per task	90	60	100
**2212 - Specialist medical practitioners**	Cardiologist	Clinical Support	Multidisciplinary clinical meetings	Support_Activity	Hours per week	2	2	2
**2212 - Specialist medical practitioners**	Cardiologist	Disease Management/Curative Services	Percutaneous coronary interventions	Direct_Patient_Care	Hours per task	2	1	3
**2212 - Specialist medical practitioners**	Cardiologist	Disease Management/Curative Services	Invasive surgery (heart valve surgery, ablation)	Direct_Patient_Care	Minutes per patient	180	150	200
**2212 - Specialist medical practitioners**	Cardiologist	Detection/Diagnosis	Cardiac electrophysiology	Direct_Patient_Care	Hours per task	3	2	4

**2212 - Specialist medical practitioners**	Dermatologist	Disease Management/Curative Services	Minor invasive procedures (dermabrasion, mole and wart excision chemical peels)	Direct_Patient_Care	Minutes per patient	90	100	120
**2212 - Specialist medical practitioners**	Dermatologist	Disease Management/Curative Services	Invasive procedures(liposuction,cyrosurgery, sclerotherapy)	Direct_Patient_Care	Minutes per patient	150	120	180
**2212 - Specialist medical practitioners**	Dermatologist	Detection/Diagnosis	Patient screening and skin analysis	Direct_Patient_Care	Minutes per patient	40	30	60
**2212 - Specialist medical practitioners**	Haematologist	Detection/Diagnosis	Interpretation of Investigations (peripheral smears, bone marrow aspirate samples)	Direct_Patient_Care	Minutes per task	40	30	50
**2212 - Specialist medical practitioners**	Haematologist	Detection/Diagnosis	Therapeutic procedures (plasmapheresis, exchange transfusion)	Direct_Patient_Care	Minutes per patient	180	160	200
**2212 - Specialist medical practitioners**	Rheumatologist	Disease Management/Curative Services	Minimal invasive procedures (joint injections, and aspiration)	Direct_Patient_Care	Minutes per patient	45	40	50
**2212 - Specialist medical practitioners**	Rheumatologist	Detection/Diagnosis	Diagnostic procedures (biopsy, joint ultrasound, synovial fluid analysis)	Direct_Patient_Care	Minutes per patient	45	40	50
**2212 - Specialist medical practitioners**	Rheumatologist	Clinical Support	Multidisciplinary clinical meetings	Support_Activity	Hours per week	1	1	1
**2212 - Specialist medical practitioners**	Ophthalmologist	Disease Management/Curative Services	Inpatient care (ward rounds, prescriptions, counselling)	Direct_Patient_Care	Minutes per patient	20	15	25
**2221 - Nursing professionals**	Intensive Care Nurse	Disease Management/Curative Services	Emergency care(including resuscitation, airway support, defibrillator support)	Direct_Patient_Care	Minutes per patient	40	30	50
**3213 - Pharmaceutical technicians and assistants**	Pharmacy Technician	Disease Management/Curative Services	Dispensing of Medicines	Direct_Patient_Care	Minutes per patient	10	8	15

**3213 - Pharmaceutical technicians and assistants**	Pharmacy Technician	Disease Management/Curative Services	Reconstitution and mixing medicines	Direct_Patient_Care	Minutes per patient	15	12	18
**3213 - Pharmaceutical technicians and assistants**	Pharmacy Technician	Health Promotion	Patient education and counselling	Direct_Patient_Care	Minutes per patient	5	4	6
**2212 - Specialist medical practitioners**	Radiologist	Detection/Diagnosis	Echocardiography	Direct_Patient_Care	Minutes per patient	30	25	40
