# Supplementary material for: Projected health workforce requirements and shortage for addressing the disease burden in the WHO Africa Region, 2022–2030: a needs-based modelling study
Source: BMJ Glob Health. 2024 Oct 22;7(Suppl 1):e015972. doi: 10.1136/bmjgh-2024-015972 (PMC11789529; doi:10.1136/bmjgh-2024-015972)
Supplement: online supplemental material 3 [file bmjgh-7-Suppl_1-s003.pdf]

S/N	Health Professionals	2022	2026	2030	Density per 10,000 population	Required Population ratio (1 professional is to xxx population)	Name of Country	Modelling Scenario	ISCO-08 Match	Income Group Classification	Sub-Regional Grouping	SDG 3c Occupation
1	Anaesthesiologist	612	669	729	0.17	60,265	Algeria	Base Estimate	2212 - Specialist medical practitioners	High Income and Upper Middle Income	West	Medical Doctors
2	Associate Nurse/Enrolled Nurse/Nursing Assistant	57,868	62,899	67,146	15.24	656	Algeria	Base Estimate	3221 - Nursing associate professionals	High Income and Upper Middle Income	West	Nursing Personnel
3	Audiologist	166	181	195	0.04	227,567	Algeria	Base Estimate	2266 - Audiologists and speech therapists	High Income and Upper Middle Income	West	Other Health Workers
4	Cardiologist	662	766	881	0.20	50,142	Algeria	Base Estimate	2212 - Specialist medical practitioners	High Income and Upper Middle Income	West	Medical Doctors
5	Cardiothoracic Surgeon	63	67	70	0.02	631,443	Algeria	Base Estimate	2212 - Specialist medical practitioners	High Income and Upper Middle Income	West	Medical Doctors
6	Clinical Officer/Physician Assistant	4,710	5,321	5,881	1.34	7,438	Algeria	Base Estimate	3256 - Medical assistants	High Income and Upper Middle Income	West	Other Health Workers
7	Clinical Pharmacist	1,950	2,178	2,381	0.54	18,422	Algeria	Base Estimate	2262 - Pharmacists	High Income and Upper Middle Income	West	Pharmacist
8	Clinical Psychologist	3,376	3,553	3,670	0.83	11,995	Algeria	Base Estimate	2634 - Psychologists	High Income and Upper Middle Income	West	Other Health Workers
9	Community health worker/Village health worker	22,200	23,083	23,352	5.30	1,886	Algeria	Base Estimate	3253 - Community health workers	High Income and Upper Middle Income	West	Other Health Workers
10	Dental Surgery Assistant	5,075	5,530	5,866	1.33	7,519	Algeria	Base Estimate	3251 - Dental assistants and therapists	High Income and Upper Middle Income	West	Other Health Workers
11	Dental Therapist	3,282	3,577	3,796	0.86	11,620	Algeria	Base Estimate	3251 - Dental assistants and therapists	High Income and Upper Middle Income	West	Other Health Workers
12	Dentist	4,270	4,517	4,603	1.05	9,543	Algeria	Base Estimate	2261 - Dentists	High Income and Upper Middle Income	West	Dentist
13	Dermatologist	184	196	207	0.05	213,759	Algeria	Base Estimate	2212 - Specialist medical practitioners	High Income and Upper Middle Income	West	Medical Doctors
14	Endocrinologist	823	1,020	1,215	0.28	35,800	Algeria	Base Estimate	2212 - Specialist medical practitioners	High Income and Upper Middle Income	West	Medical Doctors
15	ENT Surgeon	624	694	788	0.18	55,680	Algeria	Base Estimate	2212 - Specialist medical practitioners	High Income and Upper Middle Income	West	Medical Doctors
16	Environmental Health Officer	2,178	2,309	2,420	0.55	18,279	Algeria	Base Estimate	2263 - Environmental and occupational health and hygiene professionals	High Income and Upper Middle Income	West	Other Health Workers
17	Gastroenterologist	341	354	355	0.08	122,885	Algeria	Base Estimate	2212 - Specialist medical practitioners	High Income and Upper Middle Income	West	Medical Doctors
18	General Medical Practitioner (Generalist Doctor)	18,761	20,368	21,725	4.93	2,029	Algeria	Base Estimate	2211 - Generalist medical practitioners	High Income and Upper Middle Income	West	Medical Doctors
19	General Surgeon	765	905	1,046	0.24	41,932	Algeria	Base Estimate	2212 - Specialist medical practitioners	High Income and Upper Middle Income	West	Medical Doctors
20	Haematologist	102	112	121	0.03	362,065	Algeria	Base Estimate	2212 - Specialist medical practitioners	High Income and Upper Middle Income	West	Medical Doctors
21	Health Promoter/Health Educator	373	404	431	0.10	101,638	Algeria	Base Estimate	2269 - Health professionals not elsewhere classified	High Income and Upper Middle Income	West	Other Health Workers
22	Infectious Diseases Specialist	48	50	52	0.01	838,529	Algeria	Base Estimate	2212 - Specialist medical practitioners	High Income and Upper Middle Income	West	Medical Doctors
23	Intensive Care Nurse	1,616	1,731	1,843	0.42	23,960	Algeria	Base Estimate	2221 - Nursing professionals	High Income and Upper Middle Income	West	Nursing Personnel
24	Medical Laboratory Scientist	7,390	8,487	9,499	2.17	4,609	Algeria	Base Estimate	3212 - Medical and pathology laboratory technicians	High Income and Upper Middle Income	West	Other Health Workers
25	Medical Laboratory Technician	5,657	6,283	6,849	1.56	6,409	Algeria	Base Estimate	3212 - Medical and pathology laboratory technicians	High Income and Upper Middle Income	West	Other Health Workers
26	Medical Social Worker	1,413	1,477	1,486	0.34	29,590	Algeria	Base Estimate	1344 - Social welfare managers	High Income and Upper Middle Income	West	Other Health Workers
27	Mental Health Nurse	4,366	4,626	4,742	1.08	9,227	Algeria	Base Estimate	2221 - Nursing professionals	High Income and Upper Middle Income	West	Nursing Personnel
28	Midwife	31,642	33,290	33,867	7.69	1,300	Algeria	Base Estimate	2222 - Midwifery professionals	High Income and Upper Middle Income	West	Midwifery Personnel
29	Nephrologist	1,472	1,714	1,916	0.44	22,842	Algeria	Base Estimate	2212 - Specialist medical practitioners	High Income and Upper Middle Income	West	Medical Doctors
30	Neuro-Surgeon	309	345	419	0.10	103,029	Algeria	Base Estimate	2212 - Specialist medical practitioners	High Income and Upper Middle Income	West	Medical Doctors
31	Nurse Anaesthetist	2,193	2,619	3,091	0.70	14,244	Algeria	Base Estimate	2221 - Nursing professionals	High Income and Upper Middle Income	West	Nursing Personnel
32	Nutritionist	5,267	5,510	5,665	1.28	7,796	Algeria	Base Estimate	2265 - Dieticians and nutritionists	High Income and Upper Middle Income	West	Other Health Workers
33	Obstetrician & Gynaecologist	3,063	3,276	3,457	0.78	12,768	Algeria	Base Estimate	2212 - Specialist medical practitioners	High Income and Upper Middle Income	West	Medical Doctors
34	Occupational Therapist	1,399	1,519	1,629	0.37	26,972	Algeria	Base Estimate	2269 - Health professionals not elsewhere classified	High Income and Upper Middle Income	West	Other Health Workers
35	Oncology Nurse	1,108	1,306	1,495	0.34	29,334	Algeria	Base Estimate	2221 - Nursing professionals	High Income and Upper Middle Income	West	Nursing Personnel
36	Operating Theatre Nurse	9,725	10,743	11,724	2.69	3,723	Algeria	Base Estimate	2221 - Nursing professionals	High Income and Upper Middle Income	West	Nursing Personnel
37	Ophthalmic Nurse	1,805	2,067	2,323	0.53	18,883	Algeria	Base Estimate	2221 - Nursing professionals	High Income and Upper Middle Income	West	Nursing Personnel
38	Ophthalmologist	440	527	622	0.14	69,980	Algeria	Base Estimate	2212 - Specialist medical practitioners	High Income and Upper Middle Income	West	Medical Doctors
39	Optometrist	968	1,157	1,347	0.31	32,528	Algeria	Base Estimate	2267 - Optometrists and ophthalmic opticians	High Income and Upper Middle Income	West	Other Health Workers
40	Orthopaedic Nurse	1,269	1,362	1,459	0.33	30,231	Algeria	Base Estimate	2221 - Nursing professionals	High Income and Upper Middle Income	West	Nursing Personnel
41	Orthopaedic Surgeon	3,619	4,101	4,585	1.04	9,623	Algeria	Base Estimate	2212 - Specialist medical practitioners	High Income and Upper Middle Income	West	Medical Doctors
42	Orthopaedic Technologist	1,479	1,575	1,666	0.38	26,521	Algeria	Base Estimate	3214 - Medical and dental prosthetic technicians	High Income and Upper Middle Income	West	Other Health Workers
43	Paediatric Nurse	4,051	4,264	4,271	0.97	10,322	Algeria	Base Estimate	2221 - Nursing professionals	High Income and Upper Middle Income	West	Nursing Personnel
44	Paediatric Surgeon	174	185	189	0.04	234,079	Algeria	Base Estimate	2212 - Specialist medical practitioners	High Income and Upper Middle Income	West	Medical Doctors
45	Paediatrician	2,685	3,027	3,322	0.76	13,243	Algeria	Base Estimate	2212 - Specialist medical practitioners	High Income and Upper Middle Income	West	Medical Doctors
46	Pathologist	381	407	421	0.10	103,845	Algeria	Base Estimate	2212 - Specialist medical practitioners	High Income and Upper Middle Income	West	Medical Doctors
47	Pharmacist	3,368	3,729	4,069	0.93	10,758	Algeria	Base Estimate	2262 - Pharmacists	High Income and Upper Middle Income	West	Pharmacist
48	Pharmacy Technician	5,374	5,809	6,189	1.40	7,118	Algeria	Base Estimate	3213 - Pharmaceutical technicians and assistants	High Income and Upper Middle Income	West	Other Health Workers
49	Physician	7,596	8,527	9,370	2.13	4,686	Algeria	Base Estimate	2212 - Specialist medical practitioners	High Income and Upper Middle Income	West	Medical Doctors
50	Physiotherapist	1,674	1,817	2,002	0.46	21,881	Algeria	Base Estimate	2264 - Physiotherapists	High Income and Upper Middle Income	West	Other Health Workers
51	Plastic Surgeon	286	305	322	0.07	137,531	Algeria	Base Estimate	2212 - Specialist medical practitioners	High Income and Upper Middle Income	West	Medical Doctors
52	Psychiatrist	3,784	3,997	4,109	0.94	10,662	Algeria	Base Estimate	2212 - Specialist medical practitioners	High Income and Upper Middle Income	West	Medical Doctors
53	Radiation Oncologist	333	408	490	0.11	89,478	Algeria	Base Estimate	2212 - Specialist medical practitioners	High Income and Upper Middle Income	West	Medical Doctors
54	Radiographer (Diagnostics and Therapy)	6,682	7,532	8,264	1.88	5,307	Algeria	Base Estimate	3211 - Medical imaging and therapeutic equipment technicians	High Income and Upper Middle Income	West	Other Health Workers
55	Radiologist	1,988	2,252	2,510	0.57	17,497	Algeria	Base Estimate	2212 - Specialist medical practitioners	High Income and Upper Middle Income	West	Medical Doctors
56	Registered General Nurse / State Certified Nurse	164,936	186,050	203,460	46.32	216	Algeria	Base Estimate	2221 - Nursing professionals	High Income and Upper Middle Income	West	Nursing Personnel
57	Renal Nurse	19,772	23,022	25,752	5.88	1,699	Algeria	Base Estimate	2221 - Nursing professionals	High Income and Upper Middle Income	West	Nursing Personnel
58	Respiratory Physician	463	523	581	0.13	76,088	Algeria	Base Estimate	2212 - Specialist medical practitioners	High Income and Upper Middle Income	West	Medical Doctors
59	Rheumatologist	197	218	240	0.05	183,520	Algeria	Base Estimate	2212 - Specialist medical practitioners	High Income and Upper Middle Income	West	Medical Doctors
60	Speech Therapist	802	873	998	0.23	43,649	Algeria	Base Estimate	2266 - Audiologists and speech therapists	High Income and Upper Middle Income	West	Other Health Workers
61	Urologist	145	171	196	0.04	222,410	Algeria	Base Estimate	2212 - Specialist medical practitioners	High Income and Upper Middle Income	West	Medical Doctors
1	Anaesthesiologist	297	337	382	0.12	86,727	Angola	Base Estimate	2212 - Specialist medical practitioners	Lower-middle Income	Southern	Medical Doctors
2	Associate Nurse/Enrolled Nurse/Nursing Assistant	42,954	48,443	54,545	16.48	607	Angola	Base Estimate	3221 - Nursing associate professionals	Lower-middle Income	Southern	Nursing Personnel
3	Audiologist	112	127	142	0.04	233,567	Angola	Base Estimate	2266 - Audiologists and speech therapists	Lower-middle Income	Southern	Other Health Workers
4	Cardiologist	238	278	323	0.10	102,385	Angola	Base Estimate	2212 - Specialist medical practitioners	Lower-middle Income	Southern	Medical Doctors
5	Cardiothoracic Surgeon	43	47	52	0.02	641,959	Angola	Base Estimate	2212 - Specialist medical practitioners	Lower-middle Income	Southern	Medical Doctors
6	Clinical Officer/Physician Assistant	5,911	6,528	7,270	2.21	4,530	Angola	Base Estimate	3256 - Medical assistants	Lower-middle Income	Southern	Other Health Workers
7	Clinical Pharmacist	1,027	1,143	1,281	0.39	25,792	Angola	Base Estimate	2262 - Pharmacists	Lower-middle Income	Southern	Pharmacist
8	Clinical Psychologist	2,624	2,930	3,279	0.99	10,087	Angola	Base Estimate	2634 - Psychologists	Lower-middle Income	Southern	Other Health Workers
9	Community health worker/Village health worker	30,058	33,795	37,923	11.47	872	Angola	Base Estimate	3253 - Community health workers	Lower-middle Income	Southern	Other Health Workers
10	Dental Surgery Assistant	3,201	3,643	4,150	1.25	7,979	Angola	Base Estimate	3251 - Dental assistants and therapists	Lower-middle Income	Southern	Other Health Workers
11	Dental Therapist	2,077	2,363	2,691	0.81	12,304	Angola	Base Estimate	3251 - Dental assistants and therapists	Lower-middle Income	Southern	Other Health Workers
12	Dentist	2,183	2,475	2,844	0.86	11,621	Angola	Base Estimate	2261 - Dentists	Lower-middle Income	Southern	Dentist
13	Dermatologist	124	139	156	0.05	212,960	Angola	Base Estimate	2212 - Specialist medical practitioners	Lower-middle Income	Southern	Medical Doctors
14	Endocrinologist	129	154	187	0.06	175,133	Angola	Base Estimate	2212 - Specialist medical practitioners	Lower-middle Income	Southern	Medical Doctors
15	ENT Surgeon	307	351	400	0.12	82,708	Angola	Base Estimate	2212 - Specialist medical practitioners	Lower-middle Income	Southern	Medical Doctors
16	Environmental Health Officer	1,632	1,837	2,056	0.62	16,138	Angola	Base Estimate	2263 - Environmental and occupational health and hygiene professionals	Lower-middle Income	Southern	Other Health Workers
17	Gastroenterologist	559	582	616	0.19	53,428	Angola	Base Estimate	2212 - Specialist medical practitioners	Lower-middle Income	Southern	Medical Doctors
18	General Medical Practitioner (Generalist Doctor)	13,751	15,494	17,471	5.29	1,891	Angola	Base Estimate	2211 - Generalist medical practitioners	Lower-middle Income	Southern	Medical Doctors
19	General Surgeon	292	345	412	0.13	79,679	Angola	Base Estimate	2212 - Specialist medical practitioners	Lower-middle Income	Southern	Medical Doctors
20	Haematologist	106	118	130	0.04	254,441	Angola	Base Estimate	2212 - Specialist medical practitioners	Lower-middle Income	Southern	Medical Doctors
21	Health Promoter/Health Educator	466	506	554	0.17	59,525	Angola	Base Estimate	2269 - Health professionals not elsewhere classified	Lower-middle Income	Southern	Other Health Workers
22	Infectious Diseases Specialist	54	59	64	0.02	516,796	Angola	Base Estimate	2212 - Specialist medical practitioners	Lower-middle Income	Southern	Medical Doctors
23	Intensive Care Nurse	1,102	1,220	1,344	0.41	24,660	Angola	Base Estimate	2221 - Nursing professionals	Lower-middle Income	Southern	Nursing Personnel

S/N	Health Professionals	2022	2026	2030	Density per 10,000 population	Required Population ratio (1 professional is to xxx population)	Name of Country	Modelling Scenario	ISCO-08 Match	Income Group Classification	Sub-Regional Grouping	SDG 3c Occupation
24	Medical Laboratory Scientist	4,379	4,971	5,677	1.72	5,809	Angola	Base Estimate	3212 - Medical and pathology laboratory technicians	Lower-middle Income	Southern	Other Health Workers
25	Medical Laboratory Technician	5,214	5,985	6,907	2.10	4,769	Angola	Base Estimate	3212 - Medical and pathology laboratory technicians	Lower-middle Income	Southern	Other Health Workers
26	Medical Social Worker	1,464	1,537	1,617	0.49	20,408	Angola	Base Estimate	1344 - Social welfare managers	Lower-middle Income	Southern	Other Health Workers
27	Mental Health Nurse	1,961	2,211	2,532	0.77	13,013	Angola	Base Estimate	2221 - Nursing professionals	Lower-middle Income	Southern	Nursing Personnel
28	Midwife	24,312	26,936	29,975	9.06	1,103	Angola	Base Estimate	2222 - Midwifery professionals	Lower-middle Income	Southern	Midwifery Personnel
29	Nephrologist	257	300	355	0.11	92,356	Angola	Base Estimate	2212 - Specialist medical practitioners	Lower-middle Income	Southern	Medical Doctors
30	Neuro-Surgeon	112	125	140	0.04	235,744	Angola	Base Estimate	2212 - Specialist medical practitioners	Lower-middle Income	Southern	Medical Doctors
31	Nurse Anaesthetist	609	702	806	0.24	41,082	Angola	Base Estimate	2221 - Nursing professionals	Lower-middle Income	Southern	Nursing Personnel
32	Nutritionist	3,760	3,943	4,112	1.24	8,047	Angola	Base Estimate	2265 - Dieticians and nutritionists	Lower-middle Income	Southern	Other Health Workers
33	Obstetrician & Gynaecologist	2,398	2,715	3,069	0.93	10,792	Angola	Base Estimate	2212 - Specialist medical practitioners	Lower-middle Income	Southern	Medical Doctors
34	Occupational Therapist	573	657	753	0.23	43,931	Angola	Base Estimate	2269 - Health professionals not elsewhere classified	Lower-middle Income	Southern	Other Health Workers
35	Oncology Nurse	202	235	276	0.08	119,176	Angola	Base Estimate	2221 - Nursing professionals	Lower-middle Income	Southern	Nursing Personnel
36	Operating Theatre Nurse	3,111	3,565	4,128	1.25	7,978	Angola	Base Estimate	2221 - Nursing professionals	Lower-middle Income	Southern	Nursing Personnel
37	Ophthalmic Nurse	512	588	677	0.20	48,839	Angola	Base Estimate	2221 - Nursing professionals	Lower-middle Income	Southern	Nursing Personnel
38	Ophthalmologist	106	123	143	0.04	230,192	Angola	Base Estimate	2212 - Specialist medical practitioners	Lower-middle Income	Southern	Medical Doctors
39	Optometrist	401	462	531	0.16	62,260	Angola	Base Estimate	2267 - Optometrists and ophthalmic opticians	Lower-middle Income	Southern	Other Health Workers
40	Orthopaedic Nurse	568	650	739	0.22	44,877	Angola	Base Estimate	2221 - Nursing professionals	Lower-middle Income	Southern	Nursing Personnel
41	Orthopaedic Surgeon	1,008	1,285	1,647	0.51	19,737	Angola	Base Estimate	2212 - Specialist medical practitioners	Lower-middle Income	Southern	Medical Doctors
42	Orthopaedic Technologist	939	1,058	1,186	0.36	27,977	Angola	Base Estimate	3214 - Medical and dental prosthetic technicians	Lower-middle Income	Southern	Other Health Workers
43	Paediatric Nurse	3,679	4,054	4,416	1.33	7,510	Angola	Base Estimate	2221 - Nursing professionals	Lower-middle Income	Southern	Nursing Personnel
44	Paediatric Surgeon	132	145	157	0.05	211,638	Angola	Base Estimate	2212 - Specialist medical practitioners	Lower-middle Income	Southern	Medical Doctors
45	Paediatrician	1,396	1,544	1,706	0.52	19,394	Angola	Base Estimate	2212 - Specialist medical practitioners	Lower-middle Income	Southern	Medical Doctors
46	Pathologist	267	303	350	0.11	93,992	Angola	Base Estimate	2212 - Specialist medical practitioners	Lower-middle Income	Southern	Medical Doctors
47	Pharmacist	3,508	3,702	3,909	1.18	8,452	Angola	Base Estimate	2262 - Pharmacists	Lower-middle Income	Southern	Pharmacist
48	Pharmacy Technician	3,606	3,998	4,434	1.34	7,463	Angola	Base Estimate	3213 - Pharmaceutical technicians and assistants	Lower-middle Income	Southern	Other Health Workers
49	Physician	3,429	3,858	4,354	1.32	7,589	Angola	Base Estimate	2212 - Specialist medical practitioners	Lower-middle Income	Southern	Medical Doctors
50	Physiotherapist	831	939	1,060	0.32	31,256	Angola	Base Estimate	2264 - Physiotherapists	Lower-middle Income	Southern	Other Health Workers
51	Plastic Surgeon	167	188	210	0.06	157,742	Angola	Base Estimate	2212 - Specialist medical practitioners	Lower-middle Income	Southern	Medical Doctors
52	Psychiatrist	1,793	2,008	2,280	0.69	14,463	Angola	Base Estimate	2212 - Specialist medical practitioners	Lower-middle Income	Southern	Medical Doctors
53	Radiation Oncologist	69	81	96	0.03	341,598	Angola	Base Estimate	2212 - Specialist medical practitioners	Lower-middle Income	Southern	Medical Doctors
54	Radiographer (Diagnostics and Therapy)	2,368	2,718	3,150	0.96	10,456	Angola	Base Estimate	3211 - Medical imaging and therapeutic equipment technicians	Lower-middle Income	Southern	Other Health Workers
55	Radiologist	897	1,030	1,193	0.36	27,614	Angola	Base Estimate	2212 - Specialist medical practitioners	Lower-middle Income	Southern	Medical Doctors
56	Registered General Nurse / State Certified Nurse	79,879	87,736	96,646	29.29	341	Angola	Base Estimate	2221 - Nursing professionals	Lower-middle Income	Southern	Nursing Personnel
57	Renal Nurse	3,390	3,957	4,692	1.43	6,997	Angola	Base Estimate	2221 - Nursing professionals	Lower-middle Income	Southern	Nursing Personnel
58	Respiratory Physician	254	277	302	0.09	109,774	Angola	Base Estimate	2212 - Specialist medical practitioners	Lower-middle Income	Southern	Medical Doctors
59	Rheumatologist	97	111	127	0.04	261,099	Angola	Base Estimate	2212 - Specialist medical practitioners	Lower-middle Income	Southern	Medical Doctors
60	Speech Therapist	318	358	402	0.12	82,342	Angola	Base Estimate	2266 - Audiologists and speech therapists	Lower-middle Income	Southern	Other Health Workers
61	Urologist	30	35	41	0.01	809,752	Angola	Base Estimate	2212 - Specialist medical practitioners	Lower-middle Income	Southern	Medical Doctors
1	Anaesthesiologist	139	157	177	0.14	69,012	Benin	Base Estimate	2212 - Specialist medical practitioners	Low Income	West	Medical Doctors
2	Associate Nurse/Enrolled Nurse/Nursing Assistant	14,926	16,632	18,518	15.16	660	Benin	Base Estimate	3221 - Nursing associate professionals	Low Income	West	Nursing Personnel
3	Audiologist	46	52	58	0.05	210,932	Benin	Base Estimate	2266 - Audiologists and speech therapists	Low Income	West	Other Health Workers
4	Cardiologist	86	98	110	0.09	110,659	Benin	Base Estimate	2212 - Specialist medical practitioners	Low Income	West	Medical Doctors
5	Cardiothoracic Surgeon	20	23	26	0.02	478,952	Benin	Base Estimate	2212 - Specialist medical practitioners	Low Income	West	Medical Doctors
6	Clinical Officer/Physician Assistant	2,201	2,426	2,685	2.20	4,539	Benin	Base Estimate	3256 - Medical assistants	Low Income	West	Other Health Workers
7	Clinical Pharmacist	381	422	472	0.39	25,844	Benin	Base Estimate	2262 - Pharmacists	Low Income	West	Pharmacist
8	Clinical Psychologist	739	815	904	0.74	13,496	Benin	Base Estimate	2634 - Psychologists	Low Income	West	Other Health Workers
9	Community health worker/Village health worker	15,217	16,875	18,657	15.27	655	Benin	Base Estimate	3253 - Community health workers	Low Income	West	Other Health Workers
10	Dental Surgery Assistant	1,326	1,488	1,669	1.37	7,324	Benin	Base Estimate	3251 - Dental assistants and therapists	Low Income	West	Other Health Workers
11	Dental Therapist	860	965	1,082	0.89	11,295	Benin	Base Estimate	3251 - Dental assistants and therapists	Low Income	West	Other Health Workers
12	Dentist	889	990	1,112	0.91	10,981	Benin	Base Estimate	2261 - Dentists	Low Income	West	Dentist
13	Dermatologist	45	50	56	0.05	219,944	Benin	Base Estimate	2212 - Specialist medical practitioners	Low Income	West	Medical Doctors
14	Endocrinologist	41	48	58	0.05	210,521	Benin	Base Estimate	2212 - Specialist medical practitioners	Low Income	West	Medical Doctors
15	ENT Surgeon	128	145	163	0.13	74,807	Benin	Base Estimate	2212 - Specialist medical practitioners	Low Income	West	Medical Doctors
16	Environmental Health Officer	602	668	738	0.60	16,571	Benin	Base Estimate	2263 - Environmental and occupational health and hygiene professionals	Low Income	West	Other Health Workers
17	Gastroenterologist	149	150	155	0.13	78,223	Benin	Base Estimate	2212 - Specialist medical practitioners	Low Income	West	Medical Doctors
18	General Medical Practitioner (Generalist Doctor)	5,119	5,672	6,278	5.14	1,945	Benin	Base Estimate	2211 - Generalist medical practitioners	Low Income	West	Medical Doctors
19	General Surgeon	110	126	146	0.12	83,659	Benin	Base Estimate	2212 - Specialist medical practitioners	Low Income	West	Medical Doctors
20	Haematologist	46	50	55	0.04	223,767	Benin	Base Estimate	2212 - Specialist medical practitioners	Low Income	West	Medical Doctors
21	Health Promoter/Health Educator	175	191	210	0.17	57,862	Benin	Base Estimate	2269 - Health professionals not elsewhere classified	Low Income	West	Other Health Workers
22	Infectious Diseases Specialist	17	18	20	0.02	599,685	Benin	Base Estimate	2212 - Specialist medical practitioners	Low Income	West	Medical Doctors
23	Intensive Care Nurse	381	425	472	0.39	25,890	Benin	Base Estimate	2221 - Nursing professionals	Low Income	West	Nursing Personnel
24	Medical Laboratory Scientist	1,636	1,822	2,045	1.68	5,958	Benin	Base Estimate	3212 - Medical and pathology laboratory technicians	Low Income	West	Other Health Workers
25	Medical Laboratory Technician	3,101	3,511	3,972	3.25	3,074	Benin	Base Estimate	3212 - Medical and pathology laboratory technicians	Low Income	West	Other Health Workers
26	Medical Social Worker	555	587	625	0.51	19,441	Benin	Base Estimate	1344 - Social welfare managers	Low Income	West	Other Health Workers
27	Mental Health Nurse	693	778	887	0.73	13,729	Benin	Base Estimate	2221 - Nursing professionals	Low Income	West	Nursing Personnel
28	Midwife	9,394	10,352	11,404	9.33	1,072	Benin	Base Estimate	2222 - Midwifery professionals	Low Income	West	Midwifery Personnel
29	Nephrologist	115	132	154	0.13	78,928	Benin	Base Estimate	2212 - Specialist medical practitioners	Low Income	West	Medical Doctors
30	Neuro-Surgeon	50	56	63	0.05	194,312	Benin	Base Estimate	2212 - Specialist medical practitioners	Low Income	West	Medical Doctors
31	Nurse Anaesthetist	230	262	297	0.24	41,112	Benin	Base Estimate	2221 - Nursing professionals	Low Income	West	Nursing Personnel
32	Nutritionist	2,006	2,139	2,269	1.86	5,387	Benin	Base Estimate	2265 - Dieticians and nutritionists	Low Income	West	Other Health Workers
33	Obstetrician & Gynaecologist	804	898	1,005	0.82	12,134	Benin	Base Estimate	2212 - Specialist medical practitioners	Low Income	West	Medical Doctors
34	Occupational Therapist	263	301	345	0.28	35,420	Benin	Base Estimate	2269 - Health professionals not elsewhere classified	Low Income	West	Other Health Workers
35	Oncology Nurse	82	93	108	0.09	113,054	Benin	Base Estimate	2221 - Nursing professionals	Low Income	West	Nursing Personnel
36	Operating Theatre Nurse	1,252	1,410	1,611	1.32	7,562	Benin	Base Estimate	2221 - Nursing professionals	Low Income	West	Nursing Personnel
37	Ophthalmic Nurse	151	172	197	0.16	61,945	Benin	Base Estimate	2221 - Nursing professionals	Low Income	West	Nursing Personnel
38	Ophthalmologist	42	47	55	0.04	223,304	Benin	Base Estimate	2212 - Specialist medical practitioners	Low Income	West	Medical Doctors
39	Optometrist	161	181	205	0.17	59,734	Benin	Base Estimate	2267 - Optometrists and ophthalmic opticians	Low Income	West	Other Health Workers
40	Orthopaedic Nurse	246	286	331	0.27	36,929	Benin	Base Estimate	2221 - Nursing professionals	Low Income	West	Nursing Personnel
41	Orthopaedic Surgeon	391	450	518	0.42	23,556	Benin	Base Estimate	2212 - Specialist medical practitioners	Low Income	West	Medical Doctors
42	Orthopaedic Technologist	365	411	461	0.38	26,538	Benin	Base Estimate	3214 - Medical and dental prosthetic technicians	Low Income	West	Other Health Workers
43	Paediatric Nurse	1,526	1,663	1,793	1.47	6,824	Benin	Base Estimate	2221 - Nursing professionals	Low Income	West	Nursing Personnel
44	Paediatric Surgeon	48	53	58	0.05	210,622	Benin	Base Estimate	2212 - Specialist medical practitioners	Low Income	West	Medical Doctors
45	Paediatrician	593	662	739	0.61	16,525	Benin	Base Estimate	2212 - Specialist medical practitioners	Low Income	West	Medical Doctors
46	Pathologist	66	73	81	0.07	150,468	Benin	Base Estimate	2212 - Specialist medical practitioners	Low Income	West	Medical Doctors

S/N	Health Professionals	2022	2026	2030	Density per 10,000 population	Required Population ratio (1 professional is to xxx population)	Name of Country	Modelling Scenario	ISCO-08 Match	Income Group Classification	Sub-Regional Grouping	SDG 3c Occupation
47	Pharmacist	792	828	870	0.72	13,961	Benin	Base Estimate	2262 - Pharmacists	Low Income	West	Pharmacist
48	Pharmacy Technician	1,276	1,414	1,568	1.28	7,787	Benin	Base Estimate	3213 - Pharmaceutical technicians and assistants	Low Income	West	Other Health Workers
49	Physician	1,466	1,636	1,830	1.50	6,673	Benin	Base Estimate	2212 - Specialist medical practitioners	Low Income	West	Medical Doctors
50	Physiotherapist	324	363	408	0.33	29,961	Benin	Base Estimate	2264 - Physiotherapists	Low Income	West	Other Health Workers
51	Plastic Surgeon	82	94	107	0.09	114,001	Benin	Base Estimate	2212 - Specialist medical practitioners	Low Income	West	Medical Doctors
52	Psychiatrist	697	773	869	0.71	14,039	Benin	Base Estimate	2212 - Specialist medical practitioners	Low Income	West	Medical Doctors
53	Radiation Oncologist	22	25	28	0.02	429,230	Benin	Base Estimate	2212 - Specialist medical practitioners	Low Income	West	Medical Doctors
54	Radiographer (Diagnostics and Therapy)	927	1,044	1,189	0.98	10,237	Benin	Base Estimate	3211 - Medical imaging and therapeutic equipment technicians	Low Income	West	Other Health Workers
55	Radiologist	333	378	435	0.36	27,844	Benin	Base Estimate	2212 - Specialist medical practitioners	Low Income	West	Medical Doctors
56	Registered General Nurse / State Certified Nurse	31,600	34,245	37,282	30.60	327	Benin	Base Estimate	2221 - Nursing professionals	Low Income	West	Nursing Personnel
57	Renal Nurse	1,508	1,726	2,018	1.66	6,027	Benin	Base Estimate	2221 - Nursing professionals	Low Income	West	Nursing Personnel
58	Respiratory Physician	91	103	117	0.10	104,777	Benin	Base Estimate	2212 - Specialist medical practitioners	Low Income	West	Medical Doctors
59	Rheumatologist	34	39	43	0.04	283,435	Benin	Base Estimate	2212 - Specialist medical practitioners	Low Income	West	Medical Doctors
60	Speech Therapist	127	141	157	0.13	77,671	Benin	Base Estimate	2266 - Audiologists and speech therapists	Low Income	West	Other Health Workers
61	Urologist	15	18	22	0.02	539,368	Benin	Base Estimate	2212 - Specialist medical practitioners	Low Income	West	Medical Doctors
1	Anaesthesiologist	35	39	43	0.18	55,021	Botswana	Base Estimate	2212 - Specialist medical practitioners	High Income and Upper Middle Income	Southern	Medical Doctors
2	Associate Nurse/Enrolled Nurse/Nursing Assistant	3,544	3,827	4,130	17.45	573	Botswana	Base Estimate	3221 - Nursing associate professionals	High Income and Upper Middle Income	Southern	Nursing Personnel
3	Audiologist	10	11	11	0.05	206,363	Botswana	Base Estimate	2266 - Audiologists and speech therapists	High Income and Upper Middle Income	Southern	Other Health Workers
4	Cardiologist	27	31	35	0.15	67,785	Botswana	Base Estimate	2212 - Specialist medical practitioners	High Income and Upper Middle Income	Southern	Medical Doctors
5	Cardiothoracic Surgeon	5	5	6	0.02	401,181	Botswana	Base Estimate	2212 - Specialist medical practitioners	High Income and Upper Middle Income	Southern	Medical Doctors
6	Clinical Officer/Physician Assistant	491	524	564	2.39	4,185	Botswana	Base Estimate	3256 - Medical assistants	High Income and Upper Middle Income	Southern	Other Health Workers
7	Clinical Pharmacist	85	94	105	0.45	22,434	Botswana	Base Estimate	2262 - Pharmacists	High Income and Upper Middle Income	Southern	Pharmacist
8	Clinical Psychologist	160	173	187	0.79	12,632	Botswana	Base Estimate	2634 - Psychologists	High Income and Upper Middle Income	Southern	Other Health Workers
9	Community health worker/Village health worker	1,528	1,590	1,637	6.93	1,443	Botswana	Base Estimate	3253 - Community health workers	High Income and Upper Middle Income	Southern	Other Health Workers
10	Dental Surgery Assistant	342	379	418	1.76	5,679	Botswana	Base Estimate	3251 - Dental assistants and therapists	High Income and Upper Middle Income	Southern	Other Health Workers
11	Dental Therapist	221	245	271	1.14	8,766	Botswana	Base Estimate	3251 - Dental assistants and therapists	High Income and Upper Middle Income	Southern	Other Health Workers
12	Dentist	233	252	272	1.15	8,725	Botswana	Base Estimate	2261 - Dentists	High Income and Upper Middle Income	Southern	Dentist
13	Dermatologist	10	11	12	0.05	205,856	Botswana	Base Estimate	2212 - Specialist medical practitioners	High Income and Upper Middle Income	Southern	Medical Doctors
14	Endocrinologist	22	26	31	0.13	74,446	Botswana	Base Estimate	2212 - Specialist medical practitioners	High Income and Upper Middle Income	Southern	Medical Doctors
15	ENT Surgeon	37	40	46	0.19	51,946	Botswana	Base Estimate	2212 - Specialist medical practitioners	High Income and Upper Middle Income	Southern	Medical Doctors
16	Environmental Health Officer	117	125	132	0.56	17,957	Botswana	Base Estimate	2263 - Environmental and occupational health and hygiene professionals	High Income and Upper Middle Income	Southern	Other Health Workers
17	Gastroenterologist	21	21	22	0.09	106,933	Botswana	Base Estimate	2212 - Specialist medical practitioners	High Income and Upper Middle Income	Southern	Medical Doctors
18	General Medical Practitioner (Generalist Doctor)	1,159	1,250	1,344	5.68	1,762	Botswana	Base Estimate	2211 - Generalist medical practitioners	High Income and Upper Middle Income	Southern	Medical Doctors
19	General Surgeon	43	48	54	0.23	43,370	Botswana	Base Estimate	2212 - Specialist medical practitioners	High Income and Upper Middle Income	Southern	Medical Doctors
20	Haematologist	7	7	8	0.03	295,052	Botswana	Base Estimate	2212 - Specialist medical practitioners	High Income and Upper Middle Income	Southern	Medical Doctors
21	Health Promoter/Health Educator	36	38	40	0.17	59,138	Botswana	Base Estimate	2269 - Health professionals not elsewhere classified	High Income and Upper Middle Income	Southern	Other Health Workers
22	Infectious Diseases Specialist	4	4	5	0.02	520,973	Botswana	Base Estimate	2212 - Specialist medical practitioners	High Income and Upper Middle Income	Southern	Medical Doctors
23	Intensive Care Nurse	69	74	80	0.34	29,642	Botswana	Base Estimate	2221 - Nursing professionals	High Income and Upper Middle Income	Southern	Nursing Personnel
24	Medical Laboratory Scientist	471	512	562	2.38	4,196	Botswana	Base Estimate	3212 - Medical and pathology laboratory technicians	High Income and Upper Middle Income	Southern	Other Health Workers
25	Medical Laboratory Technician	367	401	439	1.86	5,376	Botswana	Base Estimate	3212 - Medical and pathology laboratory technicians	High Income and Upper Middle Income	Southern	Other Health Workers
26	Medical Social Worker	67	69	71	0.30	33,338	Botswana	Base Estimate	1344 - Social welfare managers	High Income and Upper Middle Income	Southern	Other Health Workers
27	Mental Health Nurse	177	192	211	0.89	11,178	Botswana	Base Estimate	2221 - Nursing professionals	High Income and Upper Middle Income	Southern	Nursing Personnel
28	Midwife	1,713	1,834	1,945	8.20	1,220	Botswana	Base Estimate	2222 - Midwifery professionals	High Income and Upper Middle Income	Southern	Midwifery Personnel
29	Nephrologist	47	54	65	0.27	36,401	Botswana	Base Estimate	2212 - Specialist medical practitioners	High Income and Upper Middle Income	Southern	Medical Doctors
30	Neuro-Surgeon	14	16	18	0.08	129,598	Botswana	Base Estimate	2212 - Specialist medical practitioners	High Income and Upper Middle Income	Southern	Medical Doctors
31	Nurse Anaesthetist	135	161	193	0.82	12,190	Botswana	Base Estimate	2221 - Nursing professionals	High Income and Upper Middle Income	Southern	Nursing Personnel
32	Nutritionist	198	203	206	0.87	11,483	Botswana	Base Estimate	2265 - Dieticians and nutritionists	High Income and Upper Middle Income	Southern	Other Health Workers
33	Obstetrician & Gynaecologist	214	229	245	1.03	9,666	Botswana	Base Estimate	2212 - Specialist medical practitioners	High Income and Upper Middle Income	Southern	Medical Doctors
34	Occupational Therapist	63	73	85	0.36	27,786	Botswana	Base Estimate	2269 - Health professionals not elsewhere classified	High Income and Upper Middle Income	Southern	Other Health Workers
35	Oncology Nurse	43	51	61	0.26	38,494	Botswana	Base Estimate	2221 - Nursing professionals	High Income and Upper Middle Income	Southern	Nursing Personnel
36	Operating Theatre Nurse	404	450	512	2.18	4,596	Botswana	Base Estimate	2221 - Nursing professionals	High Income and Upper Middle Income	Southern	Nursing Personnel
37	Ophthalmic Nurse	49	56	64	0.27	36,705	Botswana	Base Estimate	2221 - Nursing professionals	High Income and Upper Middle Income	Southern	Nursing Personnel
38	Ophthalmologist	19	22	25	0.11	92,871	Botswana	Base Estimate	2212 - Specialist medical practitioners	High Income and Upper Middle Income	Southern	Medical Doctors
39	Optometrist	93	107	124	0.52	19,090	Botswana	Base Estimate	2267 - Optometrists and ophthalmic opticians	High Income and Upper Middle Income	Southern	Other Health Workers
40	Orthopaedic Nurse	60	70	83	0.35	28,652	Botswana	Base Estimate	2221 - Nursing professionals	High Income and Upper Middle Income	Southern	Nursing Personnel
41	Orthopaedic Surgeon	133	152	174	0.73	13,619	Botswana	Base Estimate	2212 - Specialist medical practitioners	High Income and Upper Middle Income	Southern	Medical Doctors
42	Orthopaedic Technologist	77	85	94	0.39	25,321	Botswana	Base Estimate	3214 - Medical and dental prosthetic technicians	High Income and Upper Middle Income	Southern	Other Health Workers
43	Paediatric Nurse	202	212	217	0.92	10,909	Botswana	Base Estimate	2221 - Nursing professionals	High Income and Upper Middle Income	Southern	Nursing Personnel
44	Paediatric Surgeon	6	6	6	0.03	376,076	Botswana	Base Estimate	2212 - Specialist medical practitioners	High Income and Upper Middle Income	Southern	Medical Doctors
45	Paediatrician	107	120	134	0.57	17,567	Botswana	Base Estimate	2212 - Specialist medical practitioners	High Income and Upper Middle Income	Southern	Medical Doctors
46	Pathologist	35	38	41	0.18	56,840	Botswana	Base Estimate	2212 - Specialist medical practitioners	High Income and Upper Middle Income	Southern	Medical Doctors
47	Pharmacist	249	263	280	1.19	8,414	Botswana	Base Estimate	2262 - Pharmacists	High Income and Upper Middle Income	Southern	Pharmacist
48	Pharmacy Technician	303	330	359	1.52	6,596	Botswana	Base Estimate	3213 - Pharmaceutical technicians and assistants	High Income and Upper Middle Income	Southern	Other Health Workers
49	Physician	307	342	382	1.62	6,182	Botswana	Base Estimate	2212 - Specialist medical practitioners	High Income and Upper Middle Income	Southern	Medical Doctors
50	Physiotherapist	75	82	92	0.39	25,582	Botswana	Base Estimate	2264 - Physiotherapists	High Income and Upper Middle Income	Southern	Other Health Workers
51	Plastic Surgeon	27	30	33	0.14	70,979	Botswana	Base Estimate	2212 - Specialist medical practitioners	High Income and Upper Middle Income	Southern	Medical Doctors
52	Psychiatrist	149	160	175	0.74	13,467	Botswana	Base Estimate	2212 - Specialist medical practitioners	High Income and Upper Middle Income	Southern	Medical Doctors
53	Radiation Oncologist	19	24	29	0.13	79,611	Botswana	Base Estimate	2212 - Specialist medical practitioners	High Income and Upper Middle Income	Southern	Medical Doctors
54	Radiographer (Diagnostics and Therapy)	276	309	348	1.48	6,772	Botswana	Base Estimate	3211 - Medical imaging and therapeutic equipment technicians	High Income and Upper Middle Income	Southern	Other Health Workers
55	Radiologist	98	110	123	0.52	19,265	Botswana	Base Estimate	2212 - Specialist medical practitioners	High Income and Upper Middle Income	Southern	Medical Doctors
56	Registered General Nurse / State Certified Nurse	6,953	7,717	8,628	36.62	273	Botswana	Base Estimate	2221 - Nursing professionals	High Income and Upper Middle Income	Southern	Nursing Personnel
57	Renal Nurse	625	728	864	3.68	2,717	Botswana	Base Estimate	2221 - Nursing professionals	High Income and Upper Middle Income	Southern	Nursing Personnel
58	Respiratory Physician	19	21	24	0.10	98,549	Botswana	Base Estimate	2212 - Specialist medical practitioners	High Income and Upper Middle Income	Southern	Medical Doctors
59	Rheumatologist	10	11	12	0.05	200,935	Botswana	Base Estimate	2212 - Specialist medical practitioners	High Income and Upper Middle Income	Southern	Medical Doctors
60	Speech Therapist	28	30	34	0.14	70,313	Botswana	Base Estimate	2266 - Audiologists and speech therapists	High Income and Upper Middle Income	Southern	Other Health Workers
61	Urologist	7	9	10	0.04	226,028	Botswana	Base Estimate	2212 - Specialist medical practitioners	High Income and Upper Middle Income	Southern	Medical Doctors
1	Anaesthesiologist	260	303	352	0.17	59,749	Burkina Faso	Base Estimate	2212 - Specialist medical practitioners	Low Income	West	Medical Doctors
2	Associate Nurse/Enrolled Nurse/Nursing Assistant	29,482	32,567	36,002	17.10	585	Burkina Faso	Base Estimate	3221 - Nursing associate professionals	Low Income	West	Nursing Personnel
3	Audiologist	79	88	97	0.05	217,107	Burkina Faso	Base Estimate	2266 - Audiologists and speech therapists	Low Income	West	Other Health Workers
4	Cardiologist	126	139	154	0.07	137,236	Burkina Faso	Base Estimate	2212 - Specialist medical practitioners	Low Income	West	Medical Doctors
5	Cardiothoracic Surgeon	40	46	54	0.03	390,145	Burkina Faso	Base Estimate	2212 - Specialist medical practitioners	Low Income	West	Medical Doctors
6	Clinical Officer/Physician Assistant	2,473	2,471	2,512	1.21	8,275	Burkina Faso	Base Estimate	3256 - Medical assistants	Low Income	West	Other Health Workers
7	Clinical Pharmacist	680	748	836	0.40	25,043	Burkina Faso	Base Estimate	2262 - Pharmacists	Low Income	West	Pharmacist
8	Clinical Psychologist	1,139	1,251	1,392	0.66	15,083	Burkina Faso	Base Estimate	2634 - Psychologists	Low Income	West	Other Health Workers

S/N	Health Professionals	2022	2026	2030	Density per 10,000 population	Required Population ratio (1 professional is to xxx population)	Name of Country	Modelling Scenario	ISCO-08 Match	Income Group Classification	Sub-Regional Grouping	SDG 3c Occupation
9	Community health worker/Village health worker	22,883	24,146	25,641	12.25	816	Burkina Faso	Base Estimate	3253 - Community health workers	Low Income	West	Other Health Workers
10	Dental Surgery Assistant	2,185	2,432	2,718	1.29	7,753	Burkina Faso	Base Estimate	3251 - Dental assistants and therapists	Low Income	West	Other Health Workers
11	Dental Therapist	1,418	1,578	1,763	0.84	11,951	Burkina Faso	Base Estimate	3251 - Dental assistants and therapists	Low Income	West	Other Health Workers
12	Dentist	1,396	1,542	1,732	0.82	12,161	Burkina Faso	Base Estimate	2261 - Dentists	Low Income	West	Dentist
13	Dermatologist	77	86	95	0.05	221,070	Burkina Faso	Base Estimate	2212 - Specialist medical practitioners	Low Income	West	Medical Doctors
14	Endocrinologist	65	76	92	0.04	227,815	Burkina Faso	Base Estimate	2212 - Specialist medical practitioners	Low Income	West	Medical Doctors
15	ENT Surgeon	209	235	263	0.12	80,097	Burkina Faso	Base Estimate	2212 - Specialist medical practitioners	Low Income	West	Medical Doctors
16	Environmental Health Officer	1,038	1,146	1,259	0.60	16,757	Burkina Faso	Base Estimate	2263 - Environmental and occupational health and hygiene professionals	Low Income	West	Other Health Workers
17	Gastroenterologist	351	354	366	0.18	56,969	Burkina Faso	Base Estimate	2212 - Specialist medical practitioners	Low Income	West	Medical Doctors
18	General Medical Practitioner (Generalist Doctor)	8,818	9,620	10,530	5.01	1,996	Burkina Faso	Base Estimate	2211 - Generalist medical practitioners	Low Income	West	Medical Doctors
19	General Surgeon	203	230	265	0.13	79,364	Burkina Faso	Base Estimate	2212 - Specialist medical practitioners	Low Income	West	Medical Doctors
20	Haematologist	124	144	167	0.08	125,713	Burkina Faso	Base Estimate	2212 - Specialist medical practitioners	Low Income	West	Medical Doctors
21	Health Promoter/Health Educator	217	220	228	0.11	91,254	Burkina Faso	Base Estimate	2269 - Health professionals not elsewhere classified	Low Income	West	Other Health Workers
22	Infectious Diseases Specialist	56	60	64	0.03	327,635	Burkina Faso	Base Estimate	2212 - Specialist medical practitioners	Low Income	West	Medical Doctors
23	Intensive Care Nurse	757	835	920	0.44	22,870	Burkina Faso	Base Estimate	2221 - Nursing professionals	Low Income	West	Nursing Personnel
24	Medical Laboratory Scientist	2,731	2,949	3,240	1.55	6,448	Burkina Faso	Base Estimate	3212 - Medical and pathology laboratory technicians	Low Income	West	Other Health Workers
25	Medical Laboratory Technician	4,788	5,230	5,829	2.80	3,568	Burkina Faso	Base Estimate	3212 - Medical and pathology laboratory technicians	Low Income	West	Other Health Workers
26	Medical Social Worker	1,252	1,297	1,358	0.65	15,360	Burkina Faso	Base Estimate	1344 - Social welfare managers	Low Income	West	Other Health Workers
27	Mental Health Nurse	1,155	1,274	1,438	0.69	14,585	Burkina Faso	Base Estimate	2221 - Nursing professionals	Low Income	West	Nursing Personnel
28	Midwife	20,982	24,121	27,837	13.25	755	Burkina Faso	Base Estimate	2222 - Midwifery professionals	Low Income	West	Midwifery Personnel
29	Nephrologist	179	203	236	0.11	88,786	Burkina Faso	Base Estimate	2212 - Specialist medical practitioners	Low Income	West	Medical Doctors
30	Neuro-Surgeon	70	83	97	0.05	215,612	Burkina Faso	Base Estimate	2212 - Specialist medical practitioners	Low Income	West	Medical Doctors
31	Nurse Anaesthetist	467	552	651	0.31	32,271	Burkina Faso	Base Estimate	2221 - Nursing professionals	Low Income	West	Nursing Personnel
32	Nutritionist	4,734	4,966	5,196	2.47	4,046	Burkina Faso	Base Estimate	2265 - Dieticians and nutritionists	Low Income	West	Other Health Workers
33	Obstetrician & Gynaecologist	1,370	1,534	1,726	0.82	12,149	Burkina Faso	Base Estimate	2212 - Specialist medical practitioners	Low Income	West	Medical Doctors
34	Occupational Therapist	462	541	636	0.30	33,014	Burkina Faso	Base Estimate	2269 - Health professionals not elsewhere classified	Low Income	West	Other Health Workers
35	Oncology Nurse	142	163	191	0.09	109,802	Burkina Faso	Base Estimate	2221 - Nursing professionals	Low Income	West	Nursing Personnel
36	Operating Theatre Nurse	2,097	2,378	2,747	1.31	7,627	Burkina Faso	Base Estimate	2221 - Nursing professionals	Low Income	West	Nursing Personnel
37	Ophthalmic Nurse	250	280	316	0.15	66,633	Burkina Faso	Base Estimate	2221 - Nursing professionals	Low Income	West	Nursing Personnel
38	Ophthalmologist	70	79	90	0.04	232,592	Burkina Faso	Base Estimate	2212 - Specialist medical practitioners	Low Income	West	Medical Doctors
39	Optometrist	292	326	364	0.17	57,862	Burkina Faso	Base Estimate	2267 - Optometrists and ophthalmic opticians	Low Income	West	Other Health Workers
40	Orthopaedic Nurse	397	472	559	0.27	37,592	Burkina Faso	Base Estimate	2221 - Nursing professionals	Low Income	West	Nursing Personnel
41	Orthopaedic Surgeon	706	810	931	0.44	22,590	Burkina Faso	Base Estimate	2212 - Specialist medical practitioners	Low Income	West	Medical Doctors
42	Orthopaedic Technologist	626	707	795	0.38	26,497	Burkina Faso	Base Estimate	3214 - Medical and dental prosthetic technicians	Low Income	West	Other Health Workers
43	Paediatric Nurse	3,431	3,811	4,209	2.00	5,001	Burkina Faso	Base Estimate	2221 - Nursing professionals	Low Income	West	Nursing Personnel
44	Paediatric Surgeon	93	104	114	0.05	185,616	Burkina Faso	Base Estimate	2212 - Specialist medical practitioners	Low Income	West	Medical Doctors
45	Paediatrician	1,026	1,141	1,274	0.61	16,484	Burkina Faso	Base Estimate	2212 - Specialist medical practitioners	Low Income	West	Medical Doctors
46	Pathologist	466	502	544	0.26	38,680	Burkina Faso	Base Estimate	2212 - Specialist medical practitioners	Low Income	West	Medical Doctors
47	Pharmacist	1,427	1,484	1,553	0.74	13,478	Burkina Faso	Base Estimate	2262 - Pharmacists	Low Income	West	Pharmacist
48	Pharmacy Technician	2,259	2,488	2,750	1.31	7,645	Burkina Faso	Base Estimate	3213 - Pharmaceutical technicians and assistants	Low Income	West	Other Health Workers
49	Physician	2,724	3,024	3,380	1.61	6,212	Burkina Faso	Base Estimate	2212 - Specialist medical practitioners	Low Income	West	Medical Doctors
50	Physiotherapist	543	609	684	0.32	30,796	Burkina Faso	Base Estimate	2264 - Physiotherapists	Low Income	West	Other Health Workers
51	Plastic Surgeon	142	165	191	0.09	110,439	Burkina Faso	Base Estimate	2212 - Specialist medical practitioners	Low Income	West	Medical Doctors
52	Psychiatrist	1,250	1,360	1,503	0.72	13,967	Burkina Faso	Base Estimate	2212 - Specialist medical practitioners	Low Income	West	Medical Doctors
53	Radiation Oncologist	45	53	64	0.03	327,860	Burkina Faso	Base Estimate	2212 - Specialist medical practitioners	Low Income	West	Medical Doctors
54	Radiographer (Diagnostics and Therapy)	1,605	1,795	2,038	0.97	10,275	Burkina Faso	Base Estimate	3211 - Medical imaging and therapeutic equipment technicians	Low Income	West	Other Health Workers
55	Radiologist	602	683	789	0.38	26,332	Burkina Faso	Base Estimate	2212 - Specialist medical practitioners	Low Income	West	Medical Doctors
56	Registered General Nurse / State Certified Nurse	49,262	54,963	61,491	29.24	342	Burkina Faso	Base Estimate	2221 - Nursing professionals	Low Income	West	Nursing Personnel
57	Renal Nurse	2,353	2,661	3,093	1.48	6,764	Burkina Faso	Base Estimate	2221 - Nursing professionals	Low Income	West	Nursing Personnel
58	Respiratory Physician	153	173	196	0.09	107,479	Burkina Faso	Base Estimate	2212 - Specialist medical practitioners	Low Income	West	Medical Doctors
59	Rheumatologist	58	65	72	0.03	290,905	Burkina Faso	Base Estimate	2212 - Specialist medical practitioners	Low Income	West	Medical Doctors
60	Speech Therapist	205	227	251	0.12	83,788	Burkina Faso	Base Estimate	2266 - Audiologists and speech therapists	Low Income	West	Other Health Workers
61	Urologist	22	27	33	0.02	630,434	Burkina Faso	Base Estimate	2212 - Specialist medical practitioners	Low Income	West	Medical Doctors
1	Anaesthesiologist	108	122	137	0.11	87,525	Burundi	Base Estimate	2212 - Specialist medical practitioners	Low Income	Central	Medical Doctors
2	Associate Nurse/Enrolled Nurse/Nursing Assistant	14,470	16,592	18,098	15.04	665	Burundi	Base Estimate	3221 - Nursing associate professionals	Low Income	Central	Nursing Personnel
3	Audiologist	47	53	58	0.05	205,069	Burundi	Base Estimate	2266 - Audiologists and speech therapists	Low Income	Central	Other Health Workers
4	Cardiologist	71	80	90	0.08	133,318	Burundi	Base Estimate	2212 - Specialist medical practitioners	Low Income	Central	Medical Doctors
5	Cardiothoracic Surgeon	19	21	23	0.02	514,551	Burundi	Base Estimate	2212 - Specialist medical practitioners	Low Income	Central	Medical Doctors
6	Clinical Officer/Physician Assistant	3,502	4,869	5,322	4.33	2,310	Burundi	Base Estimate	3256 - Medical assistants	Low Income	Central	Other Health Workers
7	Clinical Pharmacist	297	330	369	0.31	32,072	Burundi	Base Estimate	2262 - Pharmacists	Low Income	Central	Pharmacist
8	Clinical Psychologist	755	839	930	0.78	12,849	Burundi	Base Estimate	2634 - Psychologists	Low Income	Central	Other Health Workers
9	Community health worker/Village health worker	21,417	29,619	32,390	26.33	380	Burundi	Base Estimate	3253 - Community health workers	Low Income	Central	Other Health Workers
10	Dental Surgery Assistant	1,127	1,260	1,414	1.18	8,461	Burundi	Base Estimate	3251 - Dental assistants and therapists	Low Income	Central	Other Health Workers
11	Dental Therapist	730	816	915	0.77	13,072	Burundi	Base Estimate	3251 - Dental assistants and therapists	Low Income	Central	Other Health Workers
12	Dentist	902	1,002	1,134	0.95	10,521	Burundi	Base Estimate	2261 - Dentists	Low Income	Central	Dentist
13	Dermatologist	40	44	49	0.04	244,354	Burundi	Base Estimate	2212 - Specialist medical practitioners	Low Income	Central	Medical Doctors
14	Endocrinologist	31	37	43	0.04	274,876	Burundi	Base Estimate	2212 - Specialist medical practitioners	Low Income	Central	Medical Doctors
15	ENT Surgeon	110	125	143	0.12	84,078	Burundi	Base Estimate	2212 - Specialist medical practitioners	Low Income	Central	Medical Doctors
16	Environmental Health Officer	591	655	723	0.60	16,583	Burundi	Base Estimate	2263 - Environmental and occupational health and hygiene professionals	Low Income	Central	Other Health Workers
17	Gastroenterologist	70	73	77	0.06	154,094	Burundi	Base Estimate	2212 - Specialist medical practitioners	Low Income	Central	Medical Doctors
18	General Medical Practitioner (Generalist Doctor)	5,072	6,156	6,763	5.59	1,789	Burundi	Base Estimate	2211 - Generalist medical practitioners	Low Income	Central	Medical Doctors
19	General Surgeon	83	96	113	0.10	105,135	Burundi	Base Estimate	2212 - Specialist medical practitioners	Low Income	Central	Medical Doctors
20	Haematologist	32	34	37	0.03	321,072	Burundi	Base Estimate	2212 - Specialist medical practitioners	Low Income	Central	Medical Doctors
21	Health Promoter/Health Educator	249	335	366	0.30	33,349	Burundi	Base Estimate	2269 - Health professionals not elsewhere classified	Low Income	Central	Other Health Workers
22	Infectious Diseases Specialist	11	12	13	0.01	953,538	Burundi	Base Estimate	2212 - Specialist medical practitioners	Low Income	Central	Medical Doctors
23	Intensive Care Nurse	337	377	419	0.35	28,605	Burundi	Base Estimate	2221 - Nursing professionals	Low Income	Central	Nursing Personnel
24	Medical Laboratory Scientist	1,794	2,350	2,612	2.15	4,644	Burundi	Base Estimate	3212 - Medical and pathology laboratory technicians	Low Income	Central	Other Health Workers
25	Medical Laboratory Technician	4,578	6,779	7,457	6.04	1,657	Burundi	Base Estimate	3212 - Medical and pathology laboratory technicians	Low Income	Central	Other Health Workers
26	Medical Social Worker	422	463	513	0.44	22,718	Burundi	Base Estimate	1344 - Social welfare managers	Low Income	Central	Other Health Workers
27	Mental Health Nurse	676	756	851	0.72	13,946	Burundi	Base Estimate	2221 - Nursing professionals	Low Income	Central	Nursing Personnel
28	Midwife	8,834	9,660	10,628	8.90	1,124	Burundi	Base Estimate	2222 - Midwifery professionals	Low Income	Central	Midwifery Personnel
29	Nephrologist	88	100	115	0.10	103,048	Burundi	Base Estimate	2212 - Specialist medical practitioners	Low Income	Central	Medical Doctors
30	Neuro-Surgeon	33	37	43	0.04	279,741	Burundi	Base Estimate	2212 - Specialist medical practitioners	Low Income	Central	Medical Doctors
31	Nurse Anaesthetist	219	246	274	0.23	43,614	Burundi	Base Estimate	2221 - Nursing professionals	Low Income	Central	Nursing Personnel

S/N	Health Professionals	2022	2026	2030	Density per 10,000 population	Required Population ratio (1 professional is to xxx population)	Name of Country	Modelling Scenario	ISCO-08 Match	Income Group Classification	Sub-Regional Grouping	SDG 3c Occupation
32	Nutritionist	1,831	1,992	2,151	1.79	5,573	Burundi	Base Estimate	2265 - Dieticians and nutritionists	Low Income	Central	Other Health Workers
33	Obstetrician & Gynaecologist	812	912	1,032	0.87	11,445	Burundi	Base Estimate	2212 - Specialist medical practitioners	Low Income	Central	Medical Doctors
34	Occupational Therapist	192	217	244	0.20	48,983	Burundi	Base Estimate	2269 - Health professionals not elsewhere classified	Low Income	Central	Other Health Workers
35	Oncology Nurse	72	81	92	0.08	129,176	Burundi	Base Estimate	2221 - Nursing professionals	Low Income	Central	Nursing Personnel
36	Operating Theatre Nurse	1,059	1,194	1,356	1.14	8,753	Burundi	Base Estimate	2221 - Nursing professionals	Low Income	Central	Nursing Personnel
37	Ophthalmic Nurse	88	99	112	0.09	106,807	Burundi	Base Estimate	2221 - Nursing professionals	Low Income	Central	Nursing Personnel
38	Ophthalmologist	36	41	47	0.04	253,879	Burundi	Base Estimate	2212 - Specialist medical practitioners	Low Income	Central	Medical Doctors
39	Optometrist	168	191	215	0.18	55,531	Burundi	Base Estimate	2267 - Optometrists and ophthalmic opticians	Low Income	Central	Other Health Workers
40	Orthopaedic Nurse	179	202	227	0.19	52,753	Burundi	Base Estimate	2221 - Nursing professionals	Low Income	Central	Nursing Personnel
41	Orthopaedic Surgeon	171	191	213	0.18	55,881	Burundi	Base Estimate	2212 - Specialist medical practitioners	Low Income	Central	Medical Doctors
42	Orthopaedic Technologist	339	378	419	0.35	28,644	Burundi	Base Estimate	3214 - Medical and dental prosthetic technicians	Low Income	Central	Other Health Workers
43	Paediatric Nurse	1,418	1,553	1,667	1.39	7,194	Burundi	Base Estimate	2221 - Nursing professionals	Low Income	Central	Nursing Personnel
44	Paediatric Surgeon	50	56	61	0.05	196,711	Burundi	Base Estimate	2212 - Specialist medical practitioners	Low Income	Central	Medical Doctors
45	Paediatrician	535	579	624	0.52	19,141	Burundi	Base Estimate	2212 - Specialist medical practitioners	Low Income	Central	Medical Doctors
46	Pathologist	85	95	108	0.09	109,802	Burundi	Base Estimate	2212 - Specialist medical practitioners	Low Income	Central	Medical Doctors
47	Pharmacist	940	1,003	1,071	0.90	11,116	Burundi	Base Estimate	2262 - Pharmacists	Low Income	Central	Pharmacist
48	Pharmacy Technician	1,038	1,146	1,265	1.06	9,440	Burundi	Base Estimate	3213 - Pharmaceutical technicians and assistants	Low Income	Central	Other Health Workers
49	Physician	1,196	1,411	1,558	1.29	7,731	Burundi	Base Estimate	2212 - Specialist medical practitioners	Low Income	Central	Medical Doctors
50	Physiotherapist	299	333	371	0.31	32,247	Burundi	Base Estimate	2264 - Physiotherapists	Low Income	Central	Other Health Workers
51	Plastic Surgeon	126	143	162	0.13	74,100	Burundi	Base Estimate	2212 - Specialist medical practitioners	Low Income	Central	Medical Doctors
52	Psychiatrist	631	700	780	0.66	15,251	Burundi	Base Estimate	2212 - Specialist medical practitioners	Low Income	Central	Medical Doctors
53	Radiation Oncologist	24	27	30	0.03	398,964	Burundi	Base Estimate	2212 - Specialist medical practitioners	Low Income	Central	Medical Doctors
54	Radiographer (Diagnostics and Therapy)	638	722	827	0.70	14,221	Burundi	Base Estimate	3211 - Medical imaging and therapeutic equipment technicians	Low Income	Central	Other Health Workers
55	Radiologist	220	255	307	0.27	36,877	Burundi	Base Estimate	2212 - Specialist medical practitioners	Low Income	Central	Medical Doctors
56	Registered General Nurse / State Certified Nurse	30,454	32,550	34,703	29.14	343	Burundi	Base Estimate	2221 - Nursing professionals	Low Income	Central	Nursing Personnel
57	Renal Nurse	1,169	1,330	1,521	1.29	7,777	Burundi	Base Estimate	2221 - Nursing professionals	Low Income	Central	Nursing Personnel
58	Respiratory Physician	118	126	134	0.11	89,457	Burundi	Base Estimate	2212 - Specialist medical practitioners	Low Income	Central	Medical Doctors
59	Rheumatologist	32	37	41	0.03	292,700	Burundi	Base Estimate	2212 - Specialist medical practitioners	Low Income	Central	Medical Doctors
60	Speech Therapist	121	135	152	0.13	79,139	Burundi	Base Estimate	2266 - Audiologists and speech therapists	Low Income	Central	Other Health Workers
61	Urologist	11	13	16	0.01	717,201	Burundi	Base Estimate	2212 - Specialist medical practitioners	Low Income	Central	Medical Doctors
1	Anaesthesiologist	9	10	11	0.20	50,525	Cabo Verde	Base Estimate	2212 - Specialist medical practitioners	Lower-middle Income	West	Medical Doctors
2	Associate Nurse/Enrolled Nurse/Nursing Assistant	749	796	852	15.24	656	Cabo Verde	Base Estimate	3221 - Nursing associate professionals	Lower-middle Income	West	Nursing Personnel
3	Audiologist	2	3	3	0.05	200,348	Cabo Verde	Base Estimate	2266 - Audiologists and speech therapists	Lower-middle Income	West	Other Health Workers
4	Cardiologist	9	9	10	0.18	55,444	Cabo Verde	Base Estimate	2212 - Specialist medical practitioners	Lower-middle Income	West	Medical Doctors
5	Cardiothoracic Surgeon	1	2	2	0.04	276,395	Cabo Verde	Base Estimate	2212 - Specialist medical practitioners	Lower-middle Income	West	Medical Doctors
6	Clinical Officer/Physician Assistant	64	69	76	1.37	7,325	Cabo Verde	Base Estimate	3256 - Medical assistants	Lower-middle Income	West	Other Health Workers
7	Clinical Pharmacist	24	26	28	0.51	19,745	Cabo Verde	Base Estimate	2262 - Pharmacists	Lower-middle Income	West	Pharmacist
8	Clinical Psychologist	44	46	49	0.88	11,332	Cabo Verde	Base Estimate	2634 - Psychologists	Lower-middle Income	West	Other Health Workers
9	Community health worker/Village health worker	307	314	321	5.80	1,725	Cabo Verde	Base Estimate	3253 - Community health workers	Lower-middle Income	West	Other Health Workers
10	Dental Surgery Assistant	91	99	107	1.90	5,259	Cabo Verde	Base Estimate	3251 - Dental assistants and therapists	Lower-middle Income	West	Other Health Workers
11	Dental Therapist	59	64	69	1.23	8,104	Cabo Verde	Base Estimate	3251 - Dental assistants and therapists	Lower-middle Income	West	Other Health Workers
12	Dentist	53	55	58	1.03	9,741	Cabo Verde	Base Estimate	2261 - Dentists	Lower-middle Income	West	Dentist
13	Dermatologist	2	3	3	0.05	200,871	Cabo Verde	Base Estimate	2212 - Specialist medical practitioners	Lower-middle Income	West	Medical Doctors
14	Endocrinologist	6	7	8	0.14	70,624	Cabo Verde	Base Estimate	2212 - Specialist medical practitioners	Lower-middle Income	West	Medical Doctors
15	ENT Surgeon	9	10	12	0.21	47,644	Cabo Verde	Base Estimate	2212 - Specialist medical practitioners	Lower-middle Income	West	Medical Doctors
16	Environmental Health Officer	28	29	30	0.53	18,795	Cabo Verde	Base Estimate	2263 - Environmental and occupational health and hygiene professionals	Lower-middle Income	West	Other Health Workers
17	Gastroenterologist	9	8	9	0.15	64,519	Cabo Verde	Base Estimate	2212 - Specialist medical practitioners	Lower-middle Income	West	Medical Doctors
18	General Medical Practitioner (Generalist Doctor)	248	263	281	5.03	1,986	Cabo Verde	Base Estimate	2211 - Generalist medical practitioners	Lower-middle Income	West	Medical Doctors
19	General Surgeon	10	12	14	0.25	40,235	Cabo Verde	Base Estimate	2212 - Specialist medical practitioners	Lower-middle Income	West	Medical Doctors
20	Haematologist	2	2	2	0.04	225,830	Cabo Verde	Base Estimate	2212 - Specialist medical practitioners	Lower-middle Income	West	Medical Doctors
21	Health Promoter/Health Educator	5	5	5	0.09	105,484	Cabo Verde	Base Estimate	2269 - Health professionals not elsewhere classified	Lower-middle Income	West	Other Health Workers
22	Infectious Diseases Specialist	1	1	1	0.02	452,437	Cabo Verde	Base Estimate	2212 - Specialist medical practitioners	Lower-middle Income	West	Medical Doctors
23	Intensive Care Nurse	16	17	18	0.33	30,259	Cabo Verde	Base Estimate	2221 - Nursing professionals	Lower-middle Income	West	Nursing Personnel
24	Medical Laboratory Scientist	87	94	103	1.85	5,399	Cabo Verde	Base Estimate	3212 - Medical and pathology laboratory technicians	Lower-middle Income	West	Other Health Workers
25	Medical Laboratory Technician	70	76	83	1.50	6,670	Cabo Verde	Base Estimate	3212 - Medical and pathology laboratory technicians	Lower-middle Income	West	Other Health Workers
26	Medical Social Worker	20	20	20	0.36	27,677	Cabo Verde	Base Estimate	1344 - Social welfare managers	Lower-middle Income	West	Other Health Workers
27	Mental Health Nurse	45	48	51	0.91	10,940	Cabo Verde	Base Estimate	2221 - Nursing professionals	Lower-middle Income	West	Nursing Personnel
28	Midwife	437	454	467	8.35	1,197	Cabo Verde	Base Estimate	2222 - Midwifery professionals	Lower-middle Income	West	Midwifery Personnel
29	Nephrologist	12	14	16	0.28	35,127	Cabo Verde	Base Estimate	2212 - Specialist medical practitioners	Lower-middle Income	West	Medical Doctors
30	Neuro-Surgeon	4	4	5	0.09	114,442	Cabo Verde	Base Estimate	2212 - Specialist medical practitioners	Lower-middle Income	West	Medical Doctors
31	Nurse Anaesthetist	33	40	50	0.91	10,980	Cabo Verde	Base Estimate	2221 - Nursing professionals	Lower-middle Income	West	Nursing Personnel
32	Nutritionist	46	47	47	0.85	11,805	Cabo Verde	Base Estimate	2265 - Dieticians and nutritionists	Lower-middle Income	West	Other Health Workers
33	Obstetrician & Gynaecologist	45	48	52	0.92	10,857	Cabo Verde	Base Estimate	2212 - Specialist medical practitioners	Lower-middle Income	West	Medical Doctors
34	Occupational Therapist	17	20	22	0.40	24,907	Cabo Verde	Base Estimate	2269 - Health professionals not elsewhere classified	Lower-middle Income	West	Other Health Workers
35	Oncology Nurse	11	13	16	0.29	34,278	Cabo Verde	Base Estimate	2221 - Nursing professionals	Lower-middle Income	West	Nursing Personnel
36	Operating Theatre Nurse	110	121	137	2.46	4,064	Cabo Verde	Base Estimate	2221 - Nursing professionals	Lower-middle Income	West	Nursing Personnel
37	Ophthalmic Nurse	11	12	14	0.24	40,888	Cabo Verde	Base Estimate	2221 - Nursing professionals	Lower-middle Income	West	Nursing Personnel
38	Ophthalmologist	4	5	5	0.10	103,686	Cabo Verde	Base Estimate	2212 - Specialist medical practitioners	Lower-middle Income	West	Medical Doctors
39	Optometrist	16	18	20	0.35	28,221	Cabo Verde	Base Estimate	2267 - Optometrists and ophthalmic opticians	Lower-middle Income	West	Other Health Workers
40	Orthopaedic Nurse	13	15	17	0.30	32,977	Cabo Verde	Base Estimate	2221 - Nursing professionals	Lower-middle Income	West	Nursing Personnel
41	Orthopaedic Surgeon	34	38	43	0.77	13,037	Cabo Verde	Base Estimate	2212 - Specialist medical practitioners	Lower-middle Income	West	Medical Doctors
42	Orthopaedic Technologist	18	20	21	0.38	26,496	Cabo Verde	Base Estimate	3214 - Medical and dental prosthetic technicians	Lower-middle Income	West	Other Health Workers
43	Paediatric Nurse	50	51	52	0.92	10,812	Cabo Verde	Base Estimate	2221 - Nursing professionals	Lower-middle Income	West	Nursing Personnel
44	Paediatric Surgeon	1	1	1	0.02	423,643	Cabo Verde	Base Estimate	2212 - Specialist medical practitioners	Lower-middle Income	West	Medical Doctors
45	Paediatrician	27	29	31	0.55	18,144	Cabo Verde	Base Estimate	2212 - Specialist medical practitioners	Lower-middle Income	West	Medical Doctors
46	Pathologist	7	8	9	0.16	62,740	Cabo Verde	Base Estimate	2212 - Specialist medical practitioners	Lower-middle Income	West	Medical Doctors
47	Pharmacist	41	42	44	0.79	12,596	Cabo Verde	Base Estimate	2262 - Pharmacists	Lower-middle Income	West	Pharmacist
48	Pharmacy Technician	71	76	81	1.46	6,870	Cabo Verde	Base Estimate	3213 - Pharmaceutical technicians and assistants	Lower-middle Income	West	Other Health Workers
49	Physician	86	93	101	1.81	5,523	Cabo Verde	Base Estimate	2212 - Specialist medical practitioners	Lower-middle Income	West	Medical Doctors
50	Physiotherapist	18	20	22	0.39	25,531	Cabo Verde	Base Estimate	2264 - Physiotherapists	Lower-middle Income	West	Other Health Workers
51	Plastic Surgeon	6	7	7	0.13	76,932	Cabo Verde	Base Estimate	2212 - Specialist medical practitioners	Lower-middle Income	West	Medical Doctors
52	Psychiatrist	40	42	45	0.81	12,377	Cabo Verde	Base Estimate	2212 - Specialist medical practitioners	Lower-middle Income	West	Medical Doctors
53	Radiation Oncologist	5	7	9	0.16	61,997	Cabo Verde	Base Estimate	2212 - Specialist medical practitioners	Lower-middle Income	West	Medical Doctors
54	Radiographer (Diagnostics and Therapy)	73	79	88	1.56	6,393	Cabo Verde	Base Estimate	3211 - Medical imaging and therapeutic equipment technicians	Lower-middle Income	West	Other Health Workers

S/N	Health Professionals	2022	2026	2030	Density per 10,000 population	Required Population ratio (1 professional is to xxx population)	Name of Country	Modelling Scenario	ISCO-08 Match	Income Group Classification	Sub-Regional Grouping	SDG 3c Occupation
55	Radiologist	25	27	30	0.54	18,589	Cabo Verde	Base Estimate	2212 - Specialist medical practitioners	Lower-middle Income	West	Medical Doctors
56	Registered General Nurse / State Certified Nurse	1,740	1,903	2,112	37.75	265	Cabo Verde	Base Estimate	2221 - Nursing professionals	Lower-middle Income	West	Nursing Personnel
57	Renal Nurse	168	187	215	3.83	2,612	Cabo Verde	Base Estimate	2221 - Nursing professionals	Lower-middle Income	West	Nursing Personnel
58	Respiratory Physician	4	5	5	0.09	108,228	Cabo Verde	Base Estimate	2212 - Specialist medical practitioners	Lower-middle Income	West	Medical Doctors
59	Rheumatologist	2	3	3	0.05	203,176	Cabo Verde	Base Estimate	2212 - Specialist medical practitioners	Lower-middle Income	West	Medical Doctors
60	Speech Therapist	7	7	8	0.14	70,516	Cabo Verde	Base Estimate	2266 - Audiologists and speech therapists	Lower-middle Income	West	Other Health Workers
61	Urologist	2	2	3	0.05	190,186	Cabo Verde	Base Estimate	2212 - Specialist medical practitioners	Lower-middle Income	West	Medical Doctors
1	Anaesthesiologist	385	435	489	0.18	54,691	Cameroon	Base Estimate	2212 - Specialist medical practitioners	Lower-middle Income	Central	Medical Doctors
2	Associate Nurse/Enrolled Nurse/Nursing Assistant	32,659	36,470	40,760	15.25	656	Cameroon	Base Estimate	3221 - Nursing associate professionals	Lower-middle Income	Central	Nursing Personnel
3	Audiologist	103	116	128	0.05	208,502	Cameroon	Base Estimate	2266 - Audiologists and speech therapists	Lower-middle Income	Central	Other Health Workers
4	Cardiologist	186	211	238	0.09	112,669	Cameroon	Base Estimate	2212 - Specialist medical practitioners	Lower-middle Income	Central	Medical Doctors
5	Cardiothoracic Surgeon	50	57	64	0.02	420,530	Cameroon	Base Estimate	2212 - Specialist medical practitioners	Lower-middle Income	Central	Medical Doctors
6	Clinical Officer/Physician Assistant	4,068	4,507	5,052	1.90	5,269	Cameroon	Base Estimate	3256 - Medical assistants	Lower-middle Income	Central	Other Health Workers
7	Clinical Pharmacist	841	951	1,091	0.41	24,314	Cameroon	Base Estimate	2262 - Pharmacists	Lower-middle Income	Central	Pharmacist
8	Clinical Psychologist	1,947	2,184	2,458	0.92	10,874	Cameroon	Base Estimate	2634 - Psychologists	Lower-middle Income	Central	Other Health Workers
9	Community health worker/Village health worker	25,550	28,228	31,121	11.64	859	Cameroon	Base Estimate	3253 - Community health workers	Lower-middle Income	Central	Other Health Workers
10	Dental Surgery Assistant	3,238	3,670	4,168	1.56	6,420	Cameroon	Base Estimate	3251 - Dental assistants and therapists	Lower-middle Income	Central	Other Health Workers
11	Dental Therapist	2,099	2,379	2,701	1.01	9,906	Cameroon	Base Estimate	3251 - Dental assistants and therapists	Lower-middle Income	Central	Other Health Workers
12	Dentist	2,143	2,410	2,749	1.03	9,725	Cameroon	Base Estimate	2261 - Dentists	Lower-middle Income	Central	Dentist
13	Dermatologist	103	116	130	0.05	205,480	Cameroon	Base Estimate	2212 - Specialist medical practitioners	Lower-middle Income	Central	Medical Doctors
14	Endocrinologist	93	111	134	0.05	196,905	Cameroon	Base Estimate	2212 - Specialist medical practitioners	Lower-middle Income	Central	Medical Doctors
15	ENT Surgeon	291	329	372	0.14	71,932	Cameroon	Base Estimate	2212 - Specialist medical practitioners	Lower-middle Income	Central	Medical Doctors
16	Environmental Health Officer	1,319	1,459	1,607	0.60	16,679	Cameroon	Base Estimate	2263 - Environmental and occupational health and hygiene professionals	Lower-middle Income	Central	Other Health Workers
17	Gastroenterologist	234	242	257	0.10	103,251	Cameroon	Base Estimate	2212 - Specialist medical practitioners	Lower-middle Income	Central	Medical Doctors
18	General Medical Practitioner (Generalist Doctor)	11,084	12,347	13,778	5.16	1,938	Cameroon	Base Estimate	2211 - Generalist medical practitioners	Lower-middle Income	Central	Medical Doctors
19	General Surgeon	274	315	368	0.14	72,532	Cameroon	Base Estimate	2212 - Specialist medical practitioners	Lower-middle Income	Central	Medical Doctors
20	Haematologist	81	88	96	0.04	279,628	Cameroon	Base Estimate	2212 - Specialist medical practitioners	Lower-middle Income	Central	Medical Doctors
21	Health Promoter/Health Educator	318	352	397	0.15	66,416	Cameroon	Base Estimate	2269 - Health professionals not elsewhere classified	Lower-middle Income	Central	Other Health Workers
22	Infectious Diseases Specialist	34	37	40	0.01	668,019	Cameroon	Base Estimate	2212 - Specialist medical practitioners	Lower-middle Income	Central	Medical Doctors
23	Intensive Care Nurse	840	916	995	0.37	26,899	Cameroon	Base Estimate	2221 - Nursing professionals	Lower-middle Income	Central	Nursing Personnel
24	Medical Laboratory Scientist	3,494	3,920	4,470	1.69	5,925	Cameroon	Base Estimate	3212 - Medical and pathology laboratory technicians	Lower-middle Income	Central	Other Health Workers
25	Medical Laboratory Technician	5,531	6,250	7,099	2.67	3,750	Cameroon	Base Estimate	3212 - Medical and pathology laboratory technicians	Lower-middle Income	Central	Other Health Workers
26	Medical Social Worker	853	936	1,063	0.41	24,361	Cameroon	Base Estimate	1344 - Social welfare managers	Lower-middle Income	Central	Other Health Workers
27	Mental Health Nurse	1,534	1,726	1,988	0.75	13,382	Cameroon	Base Estimate	2221 - Nursing professionals	Lower-middle Income	Central	Nursing Personnel
28	Midwife	20,456	22,404	24,580	9.18	1,089	Cameroon	Base Estimate	2222 - Midwifery professionals	Lower-middle Income	Central	Midwifery Personnel
29	Nephrologist	334	388	464	0.17	57,180	Cameroon	Base Estimate	2212 - Specialist medical practitioners	Lower-middle Income	Central	Medical Doctors
30	Neuro-Surgeon	135	151	168	0.06	159,020	Cameroon	Base Estimate	2212 - Specialist medical practitioners	Lower-middle Income	Central	Medical Doctors
31	Nurse Anaesthetist	683	798	931	0.35	28,687	Cameroon	Base Estimate	2221 - Nursing professionals	Lower-middle Income	Central	Nursing Personnel
32	Nutritionist	2,675	2,787	2,888	1.08	9,254	Cameroon	Base Estimate	2265 - Dieticians and nutritionists	Lower-middle Income	Central	Other Health Workers
33	Obstetrician & Gynaecologist	1,991	2,275	2,635	1.00	10,014	Cameroon	Base Estimate	2212 - Specialist medical practitioners	Lower-middle Income	Central	Medical Doctors
34	Occupational Therapist	787	891	1,011	0.38	26,430	Cameroon	Base Estimate	2269 - Health professionals not elsewhere classified	Lower-middle Income	Central	Other Health Workers
35	Oncology Nurse	238	278	331	0.12	80,211	Cameroon	Base Estimate	2221 - Nursing professionals	Lower-middle Income	Central	Nursing Personnel
36	Operating Theatre Nurse	3,238	3,675	4,246	1.60	6,268	Cameroon	Base Estimate	2221 - Nursing professionals	Lower-middle Income	Central	Nursing Personnel
37	Ophthalmic Nurse	288	332	386	0.14	69,072	Cameroon	Base Estimate	2221 - Nursing professionals	Lower-middle Income	Central	Nursing Personnel
38	Ophthalmologist	93	106	123	0.05	217,044	Cameroon	Base Estimate	2212 - Specialist medical practitioners	Lower-middle Income	Central	Medical Doctors
39	Optometrist	362	410	464	0.17	57,606	Cameroon	Base Estimate	2267 - Optometrists and ophthalmic opticians	Lower-middle Income	Central	Other Health Workers
40	Orthopaedic Nurse	781	892	1,014	0.38	26,398	Cameroon	Base Estimate	2221 - Nursing professionals	Lower-middle Income	Central	Nursing Personnel
41	Orthopaedic Surgeon	1,121	1,297	1,505	0.56	17,738	Cameroon	Base Estimate	2212 - Specialist medical practitioners	Lower-middle Income	Central	Medical Doctors
42	Orthopaedic Technologist	885	992	1,107	0.41	24,190	Cameroon	Base Estimate	3214 - Medical and dental prosthetic technicians	Lower-middle Income	Central	Other Health Workers
43	Paediatric Nurse	2,720	2,949	3,156	1.18	8,484	Cameroon	Base Estimate	2221 - Nursing professionals	Lower-middle Income	Central	Nursing Personnel
44	Paediatric Surgeon	96	104	111	0.04	241,192	Cameroon	Base Estimate	2212 - Specialist medical practitioners	Lower-middle Income	Central	Medical Doctors
45	Paediatrician	1,118	1,264	1,436	0.54	18,591	Cameroon	Base Estimate	2212 - Specialist medical practitioners	Lower-middle Income	Central	Medical Doctors
46	Pathologist	234	266	311	0.12	85,455	Cameroon	Base Estimate	2212 - Specialist medical practitioners	Lower-middle Income	Central	Medical Doctors
47	Pharmacist	1,649	1,717	1,819	0.69	14,491	Cameroon	Base Estimate	2262 - Pharmacists	Lower-middle Income	Central	Pharmacist
48	Pharmacy Technician	2,952	3,296	3,691	1.38	7,229	Cameroon	Base Estimate	3213 - Pharmaceutical technicians and assistants	Lower-middle Income	Central	Other Health Workers
49	Physician	3,140	3,543	4,014	1.50	6,654	Cameroon	Base Estimate	2212 - Specialist medical practitioners	Lower-middle Income	Central	Medical Doctors
50	Physiotherapist	765	857	959	0.36	27,901	Cameroon	Base Estimate	2264 - Physiotherapists	Lower-middle Income	Central	Other Health Workers
51	Plastic Surgeon	203	231	263	0.10	101,897	Cameroon	Base Estimate	2212 - Specialist medical practitioners	Lower-middle Income	Central	Medical Doctors
52	Psychiatrist	1,452	1,620	1,843	0.69	14,457	Cameroon	Base Estimate	2212 - Specialist medical practitioners	Lower-middle Income	Central	Medical Doctors
53	Radiation Oncologist	70	82	98	0.04	270,642	Cameroon	Base Estimate	2212 - Specialist medical practitioners	Lower-middle Income	Central	Medical Doctors
54	Radiographer (Diagnostics and Therapy)	2,292	2,647	3,112	1.18	8,499	Cameroon	Base Estimate	3211 - Medical imaging and therapeutic equipment technicians	Lower-middle Income	Central	Other Health Workers
55	Radiologist	836	1,002	1,240	0.48	20,828	Cameroon	Base Estimate	2212 - Specialist medical practitioners	Lower-middle Income	Central	Medical Doctors
56	Registered General Nurse / State Certified Nurse	63,865	70,817	79,228	29.75	336	Cameroon	Base Estimate	2221 - Nursing professionals	Lower-middle Income	Central	Nursing Personnel
57	Renal Nurse	4,343	5,053	6,036	2.28	4,394	Cameroon	Base Estimate	2221 - Nursing professionals	Lower-middle Income	Central	Nursing Personnel
58	Respiratory Physician	187	214	245	0.09	109,008	Cameroon	Base Estimate	2212 - Specialist medical practitioners	Lower-middle Income	Central	Medical Doctors
59	Rheumatologist	80	90	101	0.04	265,543	Cameroon	Base Estimate	2212 - Specialist medical practitioners	Lower-middle Income	Central	Medical Doctors
60	Speech Therapist	267	296	327	0.12	81,887	Cameroon	Base Estimate	2266 - Audiologists and speech therapists	Lower-middle Income	Central	Other Health Workers
61	Urologist	52	71	101	0.04	250,059	Cameroon	Base Estimate	2212 - Specialist medical practitioners	Lower-middle Income	Central	Medical Doctors
1	Anaesthesiologist	45	52	59	0.12	82,191	Central African Rep	Base Estimate	2212 - Specialist medical practitioners	Low Income	Central	Medical Doctors
2	Associate Nurse/Enrolled Nurse/Nursing Assistant	6,634	7,438	8,446	17.37	576	Central African Rep	Base Estimate	3221 - Nursing associate professionals	Low Income	Central	Nursing Personnel
3	Audiologist	20	23	26	0.05	185,980	Central African Rep	Base Estimate	2266 - Audiologists and speech therapists	Low Income	Central	Other Health Workers
4	Cardiologist	31	36	42	0.09	116,846	Central African Rep	Base Estimate	2212 - Specialist medical practitioners	Low Income	Central	Medical Doctors
5	Cardiothoracic Surgeon	7	8	9	0.02	565,353	Central African Rep	Base Estimate	2212 - Specialist medical practitioners	Low Income	Central	Medical Doctors
6	Clinical Officer/Physician Assistant	1,141	1,203	1,303	2.70	3,708	Central African Rep	Base Estimate	3256 - Medical assistants	Low Income	Central	Other Health Workers
7	Clinical Pharmacist	158	177	206	0.43	23,500	Central African Rep	Base Estimate	2262 - Pharmacists	Low Income	Central	Pharmacist
8	Clinical Psychologist	362	407	469	0.97	10,347	Central African Rep	Base Estimate	2634 - Psychologists	Low Income	Central	Other Health Workers
9	Community health worker/Village health worker	5,323	5,729	6,240	12.85	778	Central African Rep	Base Estimate	3253 - Community health workers	Low Income	Central	Other Health Workers
10	Dental Surgery Assistant	474	538	626	1.29	7,772	Central African Rep	Base Estimate	3251 - Dental assistants and therapists	Low Income	Central	Other Health Workers
11	Dental Therapist	307	348	405	0.83	12,009	Central African Rep	Base Estimate	3251 - Dental assistants and therapists	Low Income	Central	Other Health Workers
12	Dentist	367	417	496	1.02	9,783	Central African Rep	Base Estimate	2261 - Dentists	Low Income	Central	Dentist
13	Dermatologist	20	22	25	0.05	192,899	Central African Rep	Base Estimate	2212 - Specialist medical practitioners	Low Income	Central	Medical Doctors
14	Endocrinologist	23	28	36	0.07	134,396	Central African Rep	Base Estimate	2212 - Specialist medical practitioners	Low Income	Central	Medical Doctors
15	ENT Surgeon	48	55	63	0.13	76,898	Central African Rep	Base Estimate	2212 - Specialist medical practitioners	Low Income	Central	Medical Doctors
16	Environmental Health Officer	240	270	304	0.62	16,016	Central African Rep	Base Estimate	2263 - Environmental and occupational health and hygiene professionals	Low Income	Central	Other Health Workers

S/N	Health Professionals	2022	2026	2030	Density per 10,000 population	Required Population ratio (1 professional is to xxx population)	Name of Country	Modelling Scenario	ISCO-08 Match	Income Group Classification	Sub-Regional Grouping	SDG 3c Occupation
17	Gastroenterologist	78	85	97	0.20	49,756	Central African Rep	Base Estimate	2212 - Specialist medical practitioners	Low Income	Central	Medical Doctors
18	General Medical Practitioner (Generalist Doctor)	2,251	2,497	2,813	5.79	1,727	Central African Rep	Base Estimate	2211 - Generalist medical practitioners	Low Income	Central	Medical Doctors
19	General Surgeon	49	56	66	0.14	74,013	Central African Rep	Base Estimate	2212 - Specialist medical practitioners	Low Income	Central	Medical Doctors
20	Haematologist	20	23	26	0.05	185,244	Central African Rep	Base Estimate	2212 - Specialist medical practitioners	Low Income	Central	Medical Doctors
21	Health Promoter/Health Educator	89	95	104	0.21	46,647	Central African Rep	Base Estimate	2269 - Health professionals not elsewhere classified	Low Income	Central	Other Health Workers
22	Infectious Diseases Specialist	9	10	11	0.02	447,288	Central African Rep	Base Estimate	2212 - Specialist medical practitioners	Low Income	Central	Medical Doctors
23	Intensive Care Nurse	180	200	224	0.46	21,762	Central African Rep	Base Estimate	2221 - Nursing professionals	Low Income	Central	Nursing Personnel
24	Medical Laboratory Scientist	733	815	936	1.93	5,169	Central African Rep	Base Estimate	3212 - Medical and pathology laboratory technicians	Low Income	Central	Other Health Workers
25	Medical Laboratory Technician	1,004	1,054	1,125	2.32	4,306	Central African Rep	Base Estimate	3212 - Medical and pathology laboratory technicians	Low Income	Central	Other Health Workers
26	Medical Social Worker	221	240	270	0.56	17,973	Central African Rep	Base Estimate	1344 - Social welfare managers	Low Income	Central	Other Health Workers
27	Mental Health Nurse	334	380	457	0.95	10,575	Central African Rep	Base Estimate	2221 - Nursing professionals	Low Income	Central	Nursing Personnel
28	Midwife	4,130	4,593	5,225	10.73	932	Central African Rep	Base Estimate	2222 - Midwifery professionals	Low Income	Central	Midwifery Personnel
29	Nephrologist	38	44	55	0.11	87,503	Central African Rep	Base Estimate	2212 - Specialist medical practitioners	Low Income	Central	Medical Doctors
30	Neuro-Surgeon	16	18	20	0.04	242,965	Central African Rep	Base Estimate	2212 - Specialist medical practitioners	Low Income	Central	Medical Doctors
31	Nurse Anaesthetist	83	94	108	0.22	45,088	Central African Rep	Base Estimate	2221 - Nursing professionals	Low Income	Central	Nursing Personnel
32	Nutritionist	654	714	783	1.61	6,218	Central African Rep	Base Estimate	2265 - Dieticians and nutritionists	Low Income	Central	Other Health Workers
33	Obstetrician & Gynaecologist	358	405	465	0.96	10,449	Central African Rep	Base Estimate	2212 - Specialist medical practitioners	Low Income	Central	Medical Doctors
34	Occupational Therapist	77	88	103	0.21	47,253	Central African Rep	Base Estimate	2269 - Health professionals not elsewhere classified	Low Income	Central	Other Health Workers
35	Oncology Nurse	29	34	41	0.08	118,881	Central African Rep	Base Estimate	2221 - Nursing professionals	Low Income	Central	Nursing Personnel
36	Operating Theatre Nurse	480	550	659	1.37	7,318	Central African Rep	Base Estimate	2221 - Nursing professionals	Low Income	Central	Nursing Personnel
37	Ophthalmic Nurse	64	75	89	0.18	54,279	Central African Rep	Base Estimate	2221 - Nursing professionals	Low Income	Central	Nursing Personnel
38	Ophthalmologist	18	21	26	0.05	187,740	Central African Rep	Base Estimate	2212 - Specialist medical practitioners	Low Income	Central	Medical Doctors
39	Optometrist	72	85	101	0.21	47,809	Central African Rep	Base Estimate	2267 - Optometrists and ophthalmic opticians	Low Income	Central	Other Health Workers
40	Orthopaedic Nurse	58	66	75	0.15	64,686	Central African Rep	Base Estimate	2221 - Nursing professionals	Low Income	Central	Nursing Personnel
41	Orthopaedic Surgeon	119	131	148	0.31	32,720	Central African Rep	Base Estimate	2212 - Specialist medical practitioners	Low Income	Central	Medical Doctors
42	Orthopaedic Technologist	129	146	165	0.34	29,554	Central African Rep	Base Estimate	3214 - Medical and dental prosthetic technicians	Low Income	Central	Other Health Workers
43	Paediatric Nurse	595	668	755	1.55	6,461	Central African Rep	Base Estimate	2221 - Nursing professionals	Low Income	Central	Nursing Personnel
44	Paediatric Surgeon	20	23	25	0.05	193,598	Central African Rep	Base Estimate	2212 - Specialist medical practitioners	Low Income	Central	Medical Doctors
45	Paediatrician	217	243	276	0.57	17,624	Central African Rep	Base Estimate	2212 - Specialist medical practitioners	Low Income	Central	Medical Doctors
46	Pathologist	46	52	63	0.13	76,333	Central African Rep	Base Estimate	2212 - Specialist medical practitioners	Low Income	Central	Medical Doctors
47	Pharmacist	702	764	842	1.73	5,767	Central African Rep	Base Estimate	2262 - Pharmacists	Low Income	Central	Pharmacist
48	Pharmacy Technician	579	646	732	1.51	6,639	Central African Rep	Base Estimate	3213 - Pharmaceutical technicians and assistants	Low Income	Central	Other Health Workers
49	Physician	557	625	717	1.48	6,758	Central African Rep	Base Estimate	2212 - Specialist medical practitioners	Low Income	Central	Medical Doctors
50	Physiotherapist	119	135	154	0.32	31,645	Central African Rep	Base Estimate	2264 - Physiotherapists	Low Income	Central	Other Health Workers
51	Plastic Surgeon	25	29	33	0.07	147,058	Central African Rep	Base Estimate	2212 - Specialist medical practitioners	Low Income	Central	Medical Doctors
52	Psychiatrist	290	327	384	0.79	12,610	Central African Rep	Base Estimate	2212 - Specialist medical practitioners	Low Income	Central	Medical Doctors
53	Radiation Oncologist	9	10	12	0.02	418,760	Central African Rep	Base Estimate	2212 - Specialist medical practitioners	Low Income	Central	Medical Doctors
54	Radiographer (Diagnostics and Therapy)	356	405	479	0.99	10,087	Central African Rep	Base Estimate	3211 - Medical imaging and therapeutic equipment technicians	Low Income	Central	Other Health Workers
55	Radiologist	132	150	174	0.36	27,792	Central African Rep	Base Estimate	2212 - Specialist medical practitioners	Low Income	Central	Medical Doctors
56	Registered General Nurse / State Certified Nurse	14,497	16,270	18,586	38.26	261	Central African Rep	Base Estimate	2221 - Nursing professionals	Low Income	Central	Nursing Personnel
57	Renal Nurse	508	591	731	1.52	6,575	Central African Rep	Base Estimate	2221 - Nursing professionals	Low Income	Central	Nursing Personnel
58	Respiratory Physician	40	44	49	0.10	99,220	Central African Rep	Base Estimate	2212 - Specialist medical practitioners	Low Income	Central	Medical Doctors
59	Rheumatologist	14	16	18	0.04	269,844	Central African Rep	Base Estimate	2212 - Specialist medical practitioners	Low Income	Central	Medical Doctors
60	Speech Therapist	48	54	61	0.13	79,621	Central African Rep	Base Estimate	2266 - Audiologists and speech therapists	Low Income	Central	Other Health Workers
61	Urologist	5	6	7	0.01	687,780	Central African Rep	Base Estimate	2212 - Specialist medical practitioners	Low Income	Central	Medical Doctors
1	Anaesthesiologist	147	166	186	0.11	89,176	Chad	Base Estimate	2212 - Specialist medical practitioners	Low Income	West	Medical Doctors
2	Associate Nurse/Enrolled Nurse/Nursing Assistant	20,183	22,528	25,114	15.17	659	Chad	Base Estimate	3221 - Nursing associate professionals	Low Income	West	Nursing Personnel
3	Audiologist	61	69	77	0.05	216,280	Chad	Base Estimate	2266 - Audiologists and speech therapists	Low Income	West	Other Health Workers
4	Cardiologist	86	96	107	0.06	154,491	Chad	Base Estimate	2212 - Specialist medical practitioners	Low Income	West	Medical Doctors
5	Cardiothoracic Surgeon	25	28	31	0.02	531,470	Chad	Base Estimate	2212 - Specialist medical practitioners	Low Income	West	Medical Doctors
6	Clinical Officer/Physician Assistant	2,177	2,250	2,346	1.43	7,010	Chad	Base Estimate	3256 - Medical assistants	Low Income	West	Other Health Workers
7	Clinical Pharmacist	497	554	624	0.38	26,463	Chad	Base Estimate	2262 - Pharmacists	Low Income	West	Pharmacist
8	Clinical Psychologist	1,067	1,193	1,347	0.82	12,264	Chad	Base Estimate	2634 - Psychologists	Low Income	West	Other Health Workers
9	Community health worker/Village health worker	18,037	19,701	21,423	12.94	773	Chad	Base Estimate	3253 - Community health workers	Low Income	West	Other Health Workers
10	Dental Surgery Assistant	1,609	1,818	2,061	1.24	8,037	Chad	Base Estimate	3251 - Dental assistants and therapists	Low Income	West	Other Health Workers
11	Dental Therapist	1,043	1,179	1,336	0.81	12,399	Chad	Base Estimate	3251 - Dental assistants and therapists	Low Income	West	Other Health Workers
12	Dentist	1,120	1,267	1,460	0.88	11,318	Chad	Base Estimate	2261 - Dentists	Low Income	West	Dentist
13	Dermatologist	58	66	74	0.04	224,854	Chad	Base Estimate	2212 - Specialist medical practitioners	Low Income	West	Medical Doctors
14	Endocrinologist	39	45	53	0.03	309,553	Chad	Base Estimate	2212 - Specialist medical practitioners	Low Income	West	Medical Doctors
15	ENT Surgeon	155	176	198	0.12	83,771	Chad	Base Estimate	2212 - Specialist medical practitioners	Low Income	West	Medical Doctors
16	Environmental Health Officer	816	920	1,032	0.62	16,071	Chad	Base Estimate	2263 - Environmental and occupational health and hygiene professionals	Low Income	West	Other Health Workers
17	Gastroenterologist	295	314	345	0.21	47,627	Chad	Base Estimate	2212 - Specialist medical practitioners	Low Income	West	Medical Doctors
18	General Medical Practitioner (Generalist Doctor)	6,512	7,239	8,041	4.86	2,058	Chad	Base Estimate	2211 - Generalist medical practitioners	Low Income	West	Medical Doctors
19	General Surgeon	131	148	170	0.10	97,296	Chad	Base Estimate	2212 - Specialist medical practitioners	Low Income	West	Medical Doctors
20	Haematologist	70	80	90	0.05	184,671	Chad	Base Estimate	2212 - Specialist medical practitioners	Low Income	West	Medical Doctors
21	Health Promoter/Health Educator	214	222	232	0.14	71,068	Chad	Base Estimate	2269 - Health professionals not elsewhere classified	Low Income	West	Other Health Workers
22	Infectious Diseases Specialist	24	26	29	0.02	570,064	Chad	Base Estimate	2212 - Specialist medical practitioners	Low Income	West	Medical Doctors
23	Intensive Care Nurse	620	691	767	0.46	21,621	Chad	Base Estimate	2221 - Nursing professionals	Low Income	West	Nursing Personnel
24	Medical Laboratory Scientist	1,814	2,010	2,250	1.36	7,336	Chad	Base Estimate	3212 - Medical and pathology laboratory technicians	Low Income	West	Other Health Workers
25	Medical Laboratory Technician	2,243	2,410	2,593	1.57	6,376	Chad	Base Estimate	3212 - Medical and pathology laboratory technicians	Low Income	West	Other Health Workers
26	Medical Social Worker	1,130	1,229	1,343	0.81	12,316	Chad	Base Estimate	1344 - Social welfare managers	Low Income	West	Other Health Workers
27	Mental Health Nurse	947	1,073	1,243	0.75	13,251	Chad	Base Estimate	2221 - Nursing professionals	Low Income	West	Nursing Personnel
28	Midwife	13,402	14,865	16,550	10.00	1,000	Chad	Base Estimate	2222 - Midwifery professionals	Low Income	West	Midwifery Personnel
29	Nephrologist	115	131	155	0.09	106,031	Chad	Base Estimate	2212 - Specialist medical practitioners	Low Income	West	Medical Doctors
30	Neuro-Surgeon	37	42	48	0.03	347,894	Chad	Base Estimate	2212 - Specialist medical practitioners	Low Income	West	Medical Doctors
31	Nurse Anaesthetist	250	283	320	0.19	51,772	Chad	Base Estimate	2221 - Nursing professionals	Low Income	West	Nursing Personnel
32	Nutritionist	4,368	4,814	5,269	3.18	3,148	Chad	Base Estimate	2265 - Dieticians and nutritionists	Low Income	West	Other Health Workers
33	Obstetrician & Gynaecologist	1,026	1,159	1,308	0.79	12,661	Chad	Base Estimate	2212 - Specialist medical practitioners	Low Income	West	Medical Doctors
34	Occupational Therapist	272	307	347	0.21	47,734	Chad	Base Estimate	2269 - Health professionals not elsewhere classified	Low Income	West	Other Health Workers
35	Oncology Nurse	83	94	110	0.07	149,739	Chad	Base Estimate	2221 - Nursing professionals	Low Income	West	Nursing Personnel
36	Operating Theatre Nurse	1,371	1,533	1,750	1.06	9,421	Chad	Base Estimate	2221 - Nursing professionals	Low Income	West	Nursing Personnel
37	Ophthalmic Nurse	161	180	203	0.12	81,611	Chad	Base Estimate	2221 - Nursing professionals	Low Income	West	Nursing Personnel
38	Ophthalmologist	45	51	58	0.03	287,052	Chad	Base Estimate	2212 - Specialist medical practitioners	Low Income	West	Medical Doctors
39	Optometrist	182	201	222	0.13	74,621	Chad	Base Estimate	2267 - Optometrists and ophthalmic opticians	Low Income	West	Other Health Workers

S/N	Health Professionals	2022	2026	2030	Density per 10,000 population	Required Population ratio (1 professional is to xxx population)	Name of Country	Modelling Scenario	ISCO-08 Match	Income Group Classification	Sub-Regional Grouping	SDG 3c Occupation
40	Orthopaedic Nurse	269	307	347	0.21	47,839	Chad	Base Estimate	2221 - Nursing professionals	Low Income	West	Nursing Personnel
41	Orthopaedic Surgeon	404	468	545	0.33	30,317	Chad	Base Estimate	2212 - Specialist medical practitioners	Low Income	West	Medical Doctors
42	Orthopaedic Technologist	470	531	597	0.36	27,776	Chad	Base Estimate	3214 - Medical and dental prosthetic technicians	Low Income	West	Other Health Workers
43	Paediatric Nurse	2,620	2,941	3,261	1.96	5,090	Chad	Base Estimate	2221 - Nursing professionals	Low Income	West	Nursing Personnel
44	Paediatric Surgeon	77	88	99	0.06	168,055	Chad	Base Estimate	2212 - Specialist medical practitioners	Low Income	West	Medical Doctors
45	Paediatrician	634	726	834	0.50	19,843	Chad	Base Estimate	2212 - Specialist medical practitioners	Low Income	West	Medical Doctors
46	Pathologist	125	137	153	0.09	107,761	Chad	Base Estimate	2212 - Specialist medical practitioners	Low Income	West	Medical Doctors
47	Pharmacist	1,171	1,208	1,250	0.76	13,169	Chad	Base Estimate	2262 - Pharmacists	Low Income	West	Pharmacist
48	Pharmacy Technician	1,678	1,863	2,071	1.25	7,988	Chad	Base Estimate	3213 - Pharmaceutical technicians and assistants	Low Income	West	Other Health Workers
49	Physician	1,847	2,065	2,316	1.40	7,144	Chad	Base Estimate	2212 - Specialist medical practitioners	Low Income	West	Medical Doctors
50	Physiotherapist	408	460	517	0.31	32,019	Chad	Base Estimate	2264 - Physiotherapists	Low Income	West	Other Health Workers
51	Plastic Surgeon	99	112	127	0.08	131,066	Chad	Base Estimate	2212 - Specialist medical practitioners	Low Income	West	Medical Doctors
52	Psychiatrist	1,045	1,172	1,332	0.81	12,392	Chad	Base Estimate	2212 - Specialist medical practitioners	Low Income	West	Medical Doctors
53	Radiation Oncologist	24	27	31	0.02	539,200	Chad	Base Estimate	2212 - Specialist medical practitioners	Low Income	West	Medical Doctors
54	Radiographer (Diagnostics and Therapy)	1,105	1,247	1,427	0.86	11,569	Chad	Base Estimate	3211 - Medical imaging and therapeutic equipment technicians	Low Income	West	Other Health Workers
55	Radiologist	416	468	530	0.32	31,175	Chad	Base Estimate	2212 - Specialist medical practitioners	Low Income	West	Medical Doctors
56	Registered General Nurse / State Certified Nurse	56,975	60,264	63,961	38.78	258	Chad	Base Estimate	2221 - Nursing professionals	Low Income	West	Nursing Personnel
57	Renal Nurse	1,483	1,695	1,999	1.22	8,216	Chad	Base Estimate	2221 - Nursing professionals	Low Income	West	Nursing Personnel
58	Respiratory Physician	104	121	140	0.08	118,314	Chad	Base Estimate	2212 - Specialist medical practitioners	Low Income	West	Medical Doctors
59	Rheumatologist	43	49	55	0.03	302,039	Chad	Base Estimate	2212 - Specialist medical practitioners	Low Income	West	Medical Doctors
60	Speech Therapist	164	186	209	0.13	79,335	Chad	Base Estimate	2266 - Audiologists and speech therapists	Low Income	West	Other Health Workers
61	Urologist	17	19	22	0.01	762,290	Chad	Base Estimate	2212 - Specialist medical practitioners	Low Income	West	Medical Doctors
1	Anaesthesiologist	16	19	22	0.25	40,187	Comoros	Base Estimate	2212 - Specialist medical practitioners	Lower-middle Income	Southern	Medical Doctors
2	Associate Nurse/Enrolled Nurse/Nursing Assistant	1,102	1,191	1,288	14.72	679	Comoros	Base Estimate	3221 - Nursing associate professionals	Lower-middle Income	Southern	Nursing Personnel
3	Audiologist	4	4	5	0.06	177,978	Comoros	Base Estimate	2266 - Audiologists and speech therapists	Lower-middle Income	Southern	Other Health Workers
4	Cardiologist	10	12	14	0.16	64,215	Comoros	Base Estimate	2212 - Specialist medical practitioners	Lower-middle Income	Southern	Medical Doctors
5	Cardiothoracic Surgeon	3	3	4	0.04	240,896	Comoros	Base Estimate	2212 - Specialist medical practitioners	Lower-middle Income	Southern	Medical Doctors
6	Clinical Officer/Physician Assistant	149	159	170	1.95	5,128	Comoros	Base Estimate	3256 - Medical assistants	Lower-middle Income	Southern	Other Health Workers
7	Clinical Pharmacist	29	33	37	0.42	23,835	Comoros	Base Estimate	2262 - Pharmacists	Lower-middle Income	Southern	Pharmacist
8	Clinical Psychologist	52	57	64	0.73	13,724	Comoros	Base Estimate	2634 - Psychologists	Lower-middle Income	Southern	Other Health Workers
9	Community health worker/Village health worker	624	657	687	7.85	1,273	Comoros	Base Estimate	3253 - Community health workers	Lower-middle Income	Southern	Other Health Workers
10	Dental Surgery Assistant	110	122	135	1.54	6,473	Comoros	Base Estimate	3251 - Dental assistants and therapists	Lower-middle Income	Southern	Other Health Workers
11	Dental Therapist	71	79	88	1.00	9,998	Comoros	Base Estimate	3251 - Dental assistants and therapists	Lower-middle Income	Southern	Other Health Workers
12	Dentist	80	88	97	1.11	8,988	Comoros	Base Estimate	2261 - Dentists	Lower-middle Income	Southern	Dentist
13	Dermatologist	3	4	4	0.05	210,963	Comoros	Base Estimate	2212 - Specialist medical practitioners	Lower-middle Income	Southern	Medical Doctors
14	Endocrinologist	4	5	6	0.06	156,680	Comoros	Base Estimate	2212 - Specialist medical practitioners	Lower-middle Income	Southern	Medical Doctors
15	ENT Surgeon	12	14	16	0.18	55,146	Comoros	Base Estimate	2212 - Specialist medical practitioners	Lower-middle Income	Southern	Medical Doctors
16	Environmental Health Officer	43	46	50	0.56	17,711	Comoros	Base Estimate	2263 - Environmental and occupational health and hygiene professionals	Lower-middle Income	Southern	Other Health Workers
17	Gastroenterologist	8	8	9	0.10	96,658	Comoros	Base Estimate	2212 - Specialist medical practitioners	Lower-middle Income	Southern	Medical Doctors
18	General Medical Practitioner (Generalist Doctor)	351	382	414	4.73	2,113	Comoros	Base Estimate	2211 - Generalist medical practitioners	Lower-middle Income	Southern	Medical Doctors
19	General Surgeon	10	11	13	0.15	68,648	Comoros	Base Estimate	2212 - Specialist medical practitioners	Lower-middle Income	Southern	Medical Doctors
20	Haematologist	4	5	5	0.06	171,716	Comoros	Base Estimate	2212 - Specialist medical practitioners	Lower-middle Income	Southern	Medical Doctors
21	Health Promoter/Health Educator	9	10	10	0.12	83,723	Comoros	Base Estimate	2269 - Health professionals not elsewhere classified	Lower-middle Income	Southern	Other Health Workers
22	Infectious Diseases Specialist	1	1	1	0.01	1,007,873	Comoros	Base Estimate	2212 - Specialist medical practitioners	Lower-middle Income	Southern	Medical Doctors
23	Intensive Care Nurse	31	33	36	0.41	24,223	Comoros	Base Estimate	2221 - Nursing professionals	Lower-middle Income	Southern	Nursing Personnel
24	Medical Laboratory Scientist	109	121	134	1.54	6,496	Comoros	Base Estimate	3212 - Medical and pathology laboratory technicians	Lower-middle Income	Southern	Other Health Workers
25	Medical Laboratory Technician	82	90	99	1.13	8,821	Comoros	Base Estimate	3212 - Medical and pathology laboratory technicians	Lower-middle Income	Southern	Other Health Workers
26	Medical Social Worker	30	31	32	0.37	27,247	Comoros	Base Estimate	1344 - Social welfare managers	Lower-middle Income	Southern	Other Health Workers
27	Mental Health Nurse	62	68	77	0.88	11,322	Comoros	Base Estimate	2221 - Nursing professionals	Lower-middle Income	Southern	Nursing Personnel
28	Midwife	621	665	710	8.11	1,233	Comoros	Base Estimate	2222 - Midwifery professionals	Lower-middle Income	Southern	Midwifery Personnel
29	Nephrologist	12	13	16	0.18	55,752	Comoros	Base Estimate	2212 - Specialist medical practitioners	Lower-middle Income	Southern	Medical Doctors
30	Neuro-Surgeon	5	6	8	0.09	115,771	Comoros	Base Estimate	2212 - Specialist medical practitioners	Lower-middle Income	Southern	Medical Doctors
31	Nurse Anaesthetist	35	41	49	0.56	17,785	Comoros	Base Estimate	2221 - Nursing professionals	Lower-middle Income	Southern	Nursing Personnel
32	Nutritionist	102	103	104	1.19	8,393	Comoros	Base Estimate	2265 - Dieticians and nutritionists	Lower-middle Income	Southern	Other Health Workers
33	Obstetrician & Gynaecologist	65	72	79	0.90	11,126	Comoros	Base Estimate	2212 - Specialist medical practitioners	Lower-middle Income	Southern	Medical Doctors
34	Occupational Therapist	39	45	52	0.60	16,722	Comoros	Base Estimate	2269 - Health professionals not elsewhere classified	Lower-middle Income	Southern	Other Health Workers
35	Oncology Nurse	10	12	14	0.16	62,490	Comoros	Base Estimate	2221 - Nursing professionals	Lower-middle Income	Southern	Nursing Personnel
36	Operating Theatre Nurse	137	155	178	2.04	4,906	Comoros	Base Estimate	2221 - Nursing professionals	Lower-middle Income	Southern	Nursing Personnel
37	Ophthalmic Nurse	13	14	16	0.18	54,537	Comoros	Base Estimate	2221 - Nursing professionals	Lower-middle Income	Southern	Nursing Personnel
38	Ophthalmologist	5	5	6	0.07	147,171	Comoros	Base Estimate	2212 - Specialist medical practitioners	Lower-middle Income	Southern	Medical Doctors
39	Optometrist	21	24	28	0.31	31,751	Comoros	Base Estimate	2267 - Optometrists and ophthalmic opticians	Lower-middle Income	Southern	Other Health Workers
40	Orthopaedic Nurse	46	54	63	0.72	13,961	Comoros	Base Estimate	2221 - Nursing professionals	Lower-middle Income	Southern	Nursing Personnel
41	Orthopaedic Surgeon	44	50	58	0.66	15,057	Comoros	Base Estimate	2212 - Specialist medical practitioners	Lower-middle Income	Southern	Medical Doctors
42	Orthopaedic Technologist	38	43	49	0.55	18,040	Comoros	Base Estimate	3214 - Medical and dental prosthetic technicians	Lower-middle Income	Southern	Other Health Workers
43	Paediatric Nurse	106	112	118	1.34	7,449	Comoros	Base Estimate	2221 - Nursing professionals	Lower-middle Income	Southern	Nursing Personnel
44	Paediatric Surgeon	2	2	2	0.03	359,284	Comoros	Base Estimate	2212 - Specialist medical practitioners	Lower-middle Income	Southern	Medical Doctors
45	Paediatrician	48	52	57	0.65	15,333	Comoros	Base Estimate	2212 - Specialist medical practitioners	Lower-middle Income	Southern	Medical Doctors
46	Pathologist	10	11	12	0.14	70,299	Comoros	Base Estimate	2212 - Specialist medical practitioners	Lower-middle Income	Southern	Medical Doctors
47	Pharmacist	92	96	101	1.16	8,618	Comoros	Base Estimate	2262 - Pharmacists	Lower-middle Income	Southern	Pharmacist
48	Pharmacy Technician	107	118	130	1.49	6,721	Comoros	Base Estimate	3213 - Pharmaceutical technicians and assistants	Lower-middle Income	Southern	Other Health Workers
49	Physician	93	103	114	1.30	7,702	Comoros	Base Estimate	2212 - Specialist medical practitioners	Lower-middle Income	Southern	Medical Doctors
50	Physiotherapist	32	36	40	0.46	21,636	Comoros	Base Estimate	2264 - Physiotherapists	Lower-middle Income	Southern	Other Health Workers
51	Plastic Surgeon	19	21	24	0.27	36,827	Comoros	Base Estimate	2212 - Specialist medical practitioners	Lower-middle Income	Southern	Medical Doctors
52	Psychiatrist	52	57	63	0.72	13,853	Comoros	Base Estimate	2212 - Specialist medical practitioners	Lower-middle Income	Southern	Medical Doctors
53	Radiation Oncologist	4	5	6	0.06	156,687	Comoros	Base Estimate	2212 - Specialist medical practitioners	Lower-middle Income	Southern	Medical Doctors
54	Radiographer (Diagnostics and Therapy)	75	84	96	1.10	9,102	Comoros	Base Estimate	3211 - Medical imaging and therapeutic equipment technicians	Lower-middle Income	Southern	Other Health Workers
55	Radiologist	28	32	37	0.43	23,458	Comoros	Base Estimate	2212 - Specialist medical practitioners	Lower-middle Income	Southern	Medical Doctors
56	Registered General Nurse / State Certified Nurse	2,454	2,700	2,988	34.21	292	Comoros	Base Estimate	2221 - Nursing professionals	Lower-middle Income	Southern	Nursing Personnel
57	Renal Nurse	159	181	210	2.40	4,160	Comoros	Base Estimate	2221 - Nursing professionals	Lower-middle Income	Southern	Nursing Personnel
58	Respiratory Physician	10	10	11	0.13	77,923	Comoros	Base Estimate	2212 - Specialist medical practitioners	Lower-middle Income	Southern	Medical Doctors
59	Rheumatologist	3	3	4	0.04	226,859	Comoros	Base Estimate	2212 - Specialist medical practitioners	Lower-middle Income	Southern	Medical Doctors
60	Speech Therapist	10	11	12	0.14	73,556	Comoros	Base Estimate	2266 - Audiologists and speech therapists	Lower-middle Income	Southern	Other Health Workers
61	Urologist	2	2	2	0.03	350,928	Comoros	Base Estimate	2212 - Specialist medical practitioners	Lower-middle Income	Southern	Medical Doctors
1	Anaesthesiologist	57	64	72	0.13	76,741	Congo	Base Estimate	2212 - Specialist medical practitioners	Lower-middle Income	Central	Medical Doctors

S/N	Health Professionals	2022	2026	2030	Density per 10,000 population	Required Population ratio (1 professional is to xxx population)	Name of Country	Modelling Scenario	ISCO-08 Match	Income Group Classification	Sub-Regional Grouping	SDG 3c Occupation
2	Associate Nurse/Enrolled Nurse/Nursing Assistant	7,779	8,593	9,511	17.15	583	Congo	Base Estimate	3221 - Nursing associate professionals	Lower-middle Income	Central	Nursing Personnel
3	Audiologist	21	23	26	0.05	216,090	Congo	Base Estimate	2266 - Audiologists and speech therapists	Lower-middle Income	Central	Other Health Workers
4	Cardiologist	49	56	64	0.12	86,221	Congo	Base Estimate	2212 - Specialist medical practitioners	Lower-middle Income	Central	Medical Doctors
5	Cardiothoracic Surgeon	8	9	10	0.02	550,571	Congo	Base Estimate	2212 - Specialist medical practitioners	Lower-middle Income	Central	Medical Doctors
6	Clinical Officer/Physician Assistant	1,155	1,319	1,526	2.77	3,610	Congo	Base Estimate	3256 - Medical assistants	Lower-middle Income	Central	Other Health Workers
7	Clinical Pharmacist	191	210	234	0.42	23,607	Congo	Base Estimate	2262 - Pharmacists	Lower-middle Income	Central	Pharmacist
8	Clinical Psychologist	458	500	548	0.99	10,127	Congo	Base Estimate	2634 - Psychologists	Lower-middle Income	Central	Other Health Workers
9	Community health worker/Village health worker	5,890	6,747	7,766	14.07	711	Congo	Base Estimate	3253 - Community health workers	Lower-middle Income	Central	Other Health Workers
10	Dental Surgery Assistant	641	716	800	1.44	6,939	Congo	Base Estimate	3251 - Dental assistants and therapists	Lower-middle Income	Central	Other Health Workers
11	Dental Therapist	416	464	519	0.93	10,701	Congo	Base Estimate	3251 - Dental assistants and therapists	Lower-middle Income	Central	Other Health Workers
12	Dentist	419	462	514	0.93	10,789	Congo	Base Estimate	2261 - Dentists	Lower-middle Income	Central	Dentist
13	Dermatologist	22	24	27	0.05	208,031	Congo	Base Estimate	2212 - Specialist medical practitioners	Lower-middle Income	Central	Medical Doctors
14	Endocrinologist	36	43	52	0.09	105,845	Congo	Base Estimate	2212 - Specialist medical practitioners	Lower-middle Income	Central	Medical Doctors
15	ENT Surgeon	63	72	82	0.15	67,663	Congo	Base Estimate	2212 - Specialist medical practitioners	Lower-middle Income	Central	Medical Doctors
16	Environmental Health Officer	274	299	326	0.59	17,040	Congo	Base Estimate	2263 - Environmental and occupational health and hygiene professionals	Lower-middle Income	Central	Other Health Workers
17	Gastroenterologist	73	74	78	0.14	70,641	Congo	Base Estimate	2212 - Specialist medical practitioners	Lower-middle Income	Central	Medical Doctors
18	General Medical Practitioner (Generalist Doctor)	2,580	2,866	3,193	5.77	1,735	Congo	Base Estimate	2211 - Generalist medical practitioners	Lower-middle Income	Central	Medical Doctors
19	General Surgeon	64	74	87	0.16	63,696	Congo	Base Estimate	2212 - Specialist medical practitioners	Lower-middle Income	Central	Medical Doctors
20	Haematologist	22	24	26	0.05	217,141	Congo	Base Estimate	2212 - Specialist medical practitioners	Lower-middle Income	Central	Medical Doctors
21	Health Promoter/Health Educator	84	94	107	0.19	51,466	Congo	Base Estimate	2269 - Health professionals not elsewhere classified	Lower-middle Income	Central	Other Health Workers
22	Infectious Diseases Specialist	8	9	10	0.02	581,865	Congo	Base Estimate	2212 - Specialist medical practitioners	Lower-middle Income	Central	Medical Doctors
23	Intensive Care Nurse	172	188	204	0.37	27,166	Congo	Base Estimate	2221 - Nursing professionals	Lower-middle Income	Central	Nursing Personnel
24	Medical Laboratory Scientist	899	1,016	1,161	2.10	4,754	Congo	Base Estimate	3212 - Medical and pathology laboratory technicians	Lower-middle Income	Central	Other Health Workers
25	Medical Laboratory Technician	1,188	1,418	1,702	3.09	3,236	Congo	Base Estimate	3212 - Medical and pathology laboratory technicians	Lower-middle Income	Central	Other Health Workers
26	Medical Social Worker	191	200	211	0.38	26,106	Congo	Base Estimate	1344 - Social welfare managers	Lower-middle Income	Central	Other Health Workers
27	Mental Health Nurse	372	409	457	0.83	12,096	Congo	Base Estimate	2221 - Nursing professionals	Lower-middle Income	Central	Nursing Personnel
28	Midwife	3,995	4,364	4,761	8.58	1,166	Congo	Base Estimate	2222 - Midwifery professionals	Lower-middle Income	Central	Midwifery Personnel
29	Nephrologist	63	72	84	0.15	66,001	Congo	Base Estimate	2212 - Specialist medical practitioners	Lower-middle Income	Central	Medical Doctors
30	Neuro-Surgeon	22	24	27	0.05	203,624	Congo	Base Estimate	2212 - Specialist medical practitioners	Lower-middle Income	Central	Medical Doctors
31	Nurse Anaesthetist	148	171	198	0.36	28,027	Congo	Base Estimate	2221 - Nursing professionals	Lower-middle Income	Central	Nursing Personnel
32	Nutritionist	484	500	515	0.93	10,756	Congo	Base Estimate	2265 - Dieticians and nutritionists	Lower-middle Income	Central	Other Health Workers
33	Obstetrician & Gynaecologist	440	489	544	0.98	10,180	Congo	Base Estimate	2212 - Specialist medical practitioners	Lower-middle Income	Central	Medical Doctors
34	Occupational Therapist	116	131	150	0.27	36,903	Congo	Base Estimate	2269 - Health professionals not elsewhere classified	Lower-middle Income	Central	Other Health Workers
35	Oncology Nurse	51	59	69	0.13	79,885	Congo	Base Estimate	2221 - Nursing professionals	Lower-middle Income	Central	Nursing Personnel
36	Operating Theatre Nurse	703	798	922	1.67	5,980	Congo	Base Estimate	2221 - Nursing professionals	Lower-middle Income	Central	Nursing Personnel
37	Ophthalmic Nurse	121	139	159	0.29	34,711	Congo	Base Estimate	2221 - Nursing professionals	Lower-middle Income	Central	Nursing Personnel
38	Ophthalmologist	25	29	34	0.06	160,035	Congo	Base Estimate	2212 - Specialist medical practitioners	Lower-middle Income	Central	Medical Doctors
39	Optometrist	94	109	127	0.23	43,646	Congo	Base Estimate	2267 - Optometrists and ophthalmic opticians	Lower-middle Income	Central	Other Health Workers
40	Orthopaedic Nurse	108	122	137	0.25	40,419	Congo	Base Estimate	2221 - Nursing professionals	Lower-middle Income	Central	Nursing Personnel
41	Orthopaedic Surgeon	227	266	314	0.57	17,634	Congo	Base Estimate	2212 - Specialist medical practitioners	Lower-middle Income	Central	Medical Doctors
42	Orthopaedic Technologist	164	181	199	0.36	27,960	Congo	Base Estimate	3214 - Medical and dental prosthetic technicians	Lower-middle Income	Central	Other Health Workers
43	Paediatric Nurse	601	647	691	1.25	8,027	Congo	Base Estimate	2221 - Nursing professionals	Lower-middle Income	Central	Nursing Personnel
44	Paediatric Surgeon	18	19	20	0.04	278,746	Congo	Base Estimate	2212 - Specialist medical practitioners	Lower-middle Income	Central	Medical Doctors
45	Paediatrician	278	307	341	0.62	16,257	Congo	Base Estimate	2212 - Specialist medical practitioners	Lower-middle Income	Central	Medical Doctors
46	Pathologist	61	68	78	0.14	71,027	Congo	Base Estimate	2212 - Specialist medical practitioners	Lower-middle Income	Central	Medical Doctors
47	Pharmacist	609	644	685	1.24	8,069	Congo	Base Estimate	2262 - Pharmacists	Lower-middle Income	Central	Pharmacist
48	Pharmacy Technician	643	703	772	1.39	7,186	Congo	Base Estimate	3213 - Pharmaceutical technicians and assistants	Lower-middle Income	Central	Other Health Workers
49	Physician	661	742	838	1.51	6,607	Congo	Base Estimate	2212 - Specialist medical practitioners	Lower-middle Income	Central	Medical Doctors
50	Physiotherapist	153	169	189	0.34	29,378	Congo	Base Estimate	2264 - Physiotherapists	Lower-middle Income	Central	Other Health Workers
51	Plastic Surgeon	33	37	42	0.08	132,090	Congo	Base Estimate	2212 - Specialist medical practitioners	Lower-middle Income	Central	Medical Doctors
52	Psychiatrist	321	350	389	0.70	14,239	Congo	Base Estimate	2212 - Specialist medical practitioners	Lower-middle Income	Central	Medical Doctors
53	Radiation Oncologist	19	22	27	0.05	207,738	Congo	Base Estimate	2212 - Specialist medical practitioners	Lower-middle Income	Central	Medical Doctors
54	Radiographer (Diagnostics and Therapy)	486	549	627	1.14	8,807	Congo	Base Estimate	3211 - Medical imaging and therapeutic equipment technicians	Lower-middle Income	Central	Other Health Workers
55	Radiologist	177	203	234	0.42	23,548	Congo	Base Estimate	2212 - Specialist medical practitioners	Lower-middle Income	Central	Medical Doctors
56	Registered General Nurse / State Certified Nurse	13,413	14,880	16,572	29.93	334	Congo	Base Estimate	2221 - Nursing professionals	Lower-middle Income	Central	Nursing Personnel
57	Renal Nurse	839	960	1,118	2.03	4,929	Congo	Base Estimate	2221 - Nursing professionals	Lower-middle Income	Central	Nursing Personnel
58	Respiratory Physician	55	61	68	0.12	81,773	Congo	Base Estimate	2212 - Specialist medical practitioners	Lower-middle Income	Central	Medical Doctors
59	Rheumatologist	19	21	23	0.04	237,775	Congo	Base Estimate	2212 - Specialist medical practitioners	Lower-middle Income	Central	Medical Doctors
60	Speech Therapist	56	62	69	0.12	80,519	Congo	Base Estimate	2266 - Audiologists and speech therapists	Lower-middle Income	Central	Other Health Workers
61	Urologist	8	10	12	0.02	440,845	Congo	Base Estimate	2212 - Specialist medical practitioners	Lower-middle Income	Central	Medical Doctors
1	Anaesthesiologist	314	360	413	0.16	64,245	Côte d'Ivoire	Base Estimate	2212 - Specialist medical practitioners	Lower-middle Income	West	Medical Doctors
2	Associate Nurse/Enrolled Nurse/Nursing Assistant	32,794	36,351	40,371	15.20	658	Côte d'Ivoire	Base Estimate	3221 - Nursing associate professionals	Lower-middle Income	West	Nursing Personnel
3	Audiologist	100	112	124	0.05	213,582	Côte d'Ivoire	Base Estimate	2266 - Audiologists and speech therapists	Lower-middle Income	West	Other Health Workers
4	Cardiologist	195	225	258	0.10	102,779	Côte d'Ivoire	Base Estimate	2212 - Specialist medical practitioners	Lower-middle Income	West	Medical Doctors
5	Cardiothoracic Surgeon	48	55	62	0.02	425,343	Côte d'Ivoire	Base Estimate	2212 - Specialist medical practitioners	Lower-middle Income	West	Medical Doctors
6	Clinical Officer/Physician Assistant	5,433	5,474	5,567	2.11	4,732	Côte d'Ivoire	Base Estimate	3256 - Medical assistants	Lower-middle Income	West	Other Health Workers
7	Clinical Pharmacist	820	917	1,036	0.39	25,583	Côte d'Ivoire	Base Estimate	2262 - Pharmacists	Lower-middle Income	West	Pharmacist
8	Clinical Psychologist	1,702	1,888	2,108	0.79	12,594	Côte d'Ivoire	Base Estimate	2634 - Psychologists	Lower-middle Income	West	Other Health Workers
9	Community health worker/Village health worker	26,967	28,138	29,459	11.14	898	Côte d'Ivoire	Base Estimate	3253 - Community health workers	Lower-middle Income	West	Other Health Workers
10	Dental Surgery Assistant	3,191	3,588	4,034	1.52	6,589	Côte d'Ivoire	Base Estimate	3251 - Dental assistants and therapists	Lower-middle Income	West	Other Health Workers
11	Dental Therapist	2,070	2,327	2,617	0.98	10,160	Côte d'Ivoire	Base Estimate	3251 - Dental assistants and therapists	Lower-middle Income	West	Other Health Workers
12	Dentist	2,034	2,256	2,521	0.95	10,540	Côte d'Ivoire	Base Estimate	2261 - Dentists	Lower-middle Income	West	Dentist
13	Dermatologist	101	113	125	0.05	212,130	Côte d'Ivoire	Base Estimate	2212 - Specialist medical practitioners	Lower-middle Income	West	Medical Doctors
14	Endocrinologist	102	123	151	0.06	175,038	Côte d'Ivoire	Base Estimate	2212 - Specialist medical practitioners	Lower-middle Income	West	Medical Doctors
15	ENT Surgeon	295	336	382	0.14	69,624	Côte d'Ivoire	Base Estimate	2212 - Specialist medical practitioners	Lower-middle Income	West	Medical Doctors
16	Environmental Health Officer	1,310	1,445	1,589	0.60	16,740	Côte d'Ivoire	Base Estimate	2263 - Environmental and occupational health and hygiene professionals	Lower-middle Income	West	Other Health Workers
17	Gastroenterologist	281	281	288	0.11	91,621	Côte d'Ivoire	Base Estimate	2212 - Specialist medical practitioners	Lower-middle Income	West	Medical Doctors
18	General Medical Practitioner (Generalist Doctor)	11,260	12,297	13,469	5.08	1,970	Côte d'Ivoire	Base Estimate	2211 - Generalist medical practitioners	Lower-middle Income	West	Medical Doctors
19	General Surgeon	270	310	359	0.14	73,892	Côte d'Ivoire	Base Estimate	2212 - Specialist medical practitioners	Lower-middle Income	West	Medical Doctors
20	Haematologist	119	138	158	0.06	168,106	Côte d'Ivoire	Base Estimate	2212 - Specialist medical practitioners	Lower-middle Income	West	Medical Doctors
21	Health Promoter/Health Educator	337	342	351	0.13	75,009	Côte d'Ivoire	Base Estimate	2269 - Health professionals not elsewhere classified	Lower-middle Income	West	Other Health Workers
22	Infectious Diseases Specialist	36	39	43	0.02	619,531	Côte d'Ivoire	Base Estimate	2212 - Specialist medical practitioners	Lower-middle Income	West	Medical Doctors
23	Intensive Care Nurse	820	907	1,001	0.38	26,563	Côte d'Ivoire	Base Estimate	2221 - Nursing professionals	Lower-middle Income	West	Nursing Personnel
24	Medical Laboratory Scientist	3,344	3,639	4,021	1.52	6,573	Côte d'Ivoire	Base Estimate	3212 - Medical and pathology laboratory technicians	Lower-middle Income	West	Other Health Workers

S/N	Health Professionals	2022	2026	2030	Density per 10,000 population	Required Population ratio (1 professional is to xxx population)	Name of Country	Modelling Scenario	ISCO-08 Match	Income Group Classification	Sub-Regional Grouping	SDG 3c Occupation
25	Medical Laboratory Technician	4,422	4,490	4,613	1.75	5,704	Côte d'Ivoire	Base Estimate	3212 - Medical and pathology laboratory technicians	Lower-middle Income	West	Other Health Workers
26	Medical Social Worker	1,029	1,080	1,143	0.43	23,141	Côte d'Ivoire	Base Estimate	1344 - Social welfare managers	Lower-middle Income	West	Other Health Workers
27	Mental Health Nurse	1,493	1,671	1,904	0.72	13,926	Côte d'Ivoire	Base Estimate	2221 - Nursing professionals	Lower-middle Income	West	Nursing Personnel
28	Midwife	19,581	21,463	23,532	8.85	1,130	Côte d'Ivoire	Base Estimate	2222 - Midwifery professionals	Lower-middle Income	West	Midwifery Personnel
29	Nephrologist	289	331	388	0.15	68,289	Côte d'Ivoire	Base Estimate	2212 - Specialist medical practitioners	Lower-middle Income	West	Medical Doctors
30	Neuro-Surgeon	107	122	140	0.05	190,238	Côte d'Ivoire	Base Estimate	2212 - Specialist medical practitioners	Lower-middle Income	West	Medical Doctors
31	Nurse Anaesthetist	561	652	757	0.29	35,051	Côte d'Ivoire	Base Estimate	2221 - Nursing professionals	Lower-middle Income	West	Nursing Personnel
32	Nutritionist	3,738	3,986	4,237	1.59	6,270	Côte d'Ivoire	Base Estimate	2265 - Dieticians and nutritionists	Lower-middle Income	West	Other Health Workers
33	Obstetrician & Gynaecologist	1,832	2,055	2,312	0.87	11,480	Côte d'Ivoire	Base Estimate	2212 - Specialist medical practitioners	Lower-middle Income	West	Medical Doctors
34	Occupational Therapist	613	709	823	0.31	32,208	Côte d'Ivoire	Base Estimate	2269 - Health professionals not elsewhere classified	Lower-middle Income	West	Other Health Workers
35	Oncology Nurse	204	235	275	0.10	96,310	Côte d'Ivoire	Base Estimate	2221 - Nursing professionals	Lower-middle Income	West	Nursing Personnel
36	Operating Theatre Nurse	3,042	3,469	4,021	1.52	6,593	Côte d'Ivoire	Base Estimate	2221 - Nursing professionals	Lower-middle Income	West	Nursing Personnel
37	Ophthalmic Nurse	355	405	465	0.18	57,104	Côte d'Ivoire	Base Estimate	2221 - Nursing professionals	Lower-middle Income	West	Nursing Personnel
38	Ophthalmologist	101	117	137	0.05	193,998	Côte d'Ivoire	Base Estimate	2212 - Specialist medical practitioners	Lower-middle Income	West	Medical Doctors
39	Optometrist	398	460	533	0.20	49,746	Côte d'Ivoire	Base Estimate	2267 - Optometrists and ophthalmic opticians	Lower-middle Income	West	Other Health Workers
40	Orthopaedic Nurse	599	702	818	0.31	32,434	Côte d'Ivoire	Base Estimate	2221 - Nursing professionals	Lower-middle Income	West	Nursing Personnel
41	Orthopaedic Surgeon	991	1,149	1,336	0.50	19,866	Côte d'Ivoire	Base Estimate	2212 - Specialist medical practitioners	Lower-middle Income	West	Medical Doctors
42	Orthopaedic Technologist	831	934	1,047	0.39	25,384	Côte d'Ivoire	Base Estimate	3214 - Medical and dental prosthetic technicians	Lower-middle Income	West	Other Health Workers
43	Paediatric Nurse	3,428	3,805	4,191	1.58	6,349	Côte d'Ivoire	Base Estimate	2221 - Nursing professionals	Lower-middle Income	West	Nursing Personnel
44	Paediatric Surgeon	99	108	117	0.04	227,987	Côte d'Ivoire	Base Estimate	2212 - Specialist medical practitioners	Lower-middle Income	West	Medical Doctors
45	Paediatrician	1,263	1,427	1,617	0.61	16,426	Côte d'Ivoire	Base Estimate	2212 - Specialist medical practitioners	Lower-middle Income	West	Medical Doctors
46	Pathologist	180	204	236	0.09	112,469	Côte d'Ivoire	Base Estimate	2212 - Specialist medical practitioners	Lower-middle Income	West	Medical Doctors
47	Pharmacist	2,554	2,638	2,736	1.04	9,657	Côte d'Ivoire	Base Estimate	2262 - Pharmacists	Lower-middle Income	West	Pharmacist
48	Pharmacy Technician	2,934	3,244	3,594	1.35	7,386	Côte d'Ivoire	Base Estimate	3213 - Pharmaceutical technicians and assistants	Lower-middle Income	West	Other Health Workers
49	Physician	3,084	3,421	3,816	1.44	6,954	Côte d'Ivoire	Base Estimate	2212 - Specialist medical practitioners	Lower-middle Income	West	Medical Doctors
50	Physiotherapist	733	823	925	0.35	28,736	Côte d'Ivoire	Base Estimate	2264 - Physiotherapists	Lower-middle Income	West	Other Health Workers
51	Plastic Surgeon	192	220	252	0.09	105,518	Côte d'Ivoire	Base Estimate	2212 - Specialist medical practitioners	Lower-middle Income	West	Medical Doctors
52	Psychiatrist	1,454	1,612	1,811	0.68	14,644	Côte d'Ivoire	Base Estimate	2212 - Specialist medical practitioners	Lower-middle Income	West	Medical Doctors
53	Radiation Oncologist	56	66	78	0.03	340,822	Côte d'Ivoire	Base Estimate	2212 - Specialist medical practitioners	Lower-middle Income	West	Medical Doctors
54	Radiographer (Diagnostics and Therapy)	2,175	2,461	2,817	1.06	9,408	Côte d'Ivoire	Base Estimate	3211 - Medical imaging and therapeutic equipment technicians	Lower-middle Income	West	Other Health Workers
55	Radiologist	782	894	1,033	0.39	25,593	Côte d'Ivoire	Base Estimate	2212 - Specialist medical practitioners	Lower-middle Income	West	Medical Doctors
56	Registered General Nurse / State Certified Nurse	64,402	71,160	79,291	29.91	334	Côte d'Ivoire	Base Estimate	2221 - Nursing professionals	Lower-middle Income	West	Nursing Personnel
57	Renal Nurse	3,798	4,357	5,115	1.93	5,178	Côte d'Ivoire	Base Estimate	2221 - Nursing professionals	Lower-middle Income	West	Nursing Personnel
58	Respiratory Physician	223	251	283	0.11	93,783	Côte d'Ivoire	Base Estimate	2212 - Specialist medical practitioners	Lower-middle Income	West	Medical Doctors
59	Rheumatologist	81	92	104	0.04	256,651	Côte d'Ivoire	Base Estimate	2212 - Specialist medical practitioners	Lower-middle Income	West	Medical Doctors
60	Speech Therapist	275	304	337	0.13	78,919	Côte d'Ivoire	Base Estimate	2266 - Audiologists and speech therapists	Lower-middle Income	West	Other Health Workers
61	Urologist	46	56	70	0.03	375,718	Côte d'Ivoire	Base Estimate	2212 - Specialist medical practitioners	Lower-middle Income	West	Medical Doctors
1	Anaesthesiologist	780	915	1,071	0.12	84,056	Democratic Republi	Base Estimate	2212 - Specialist medical practitioners	Low Income	Central	Medical Doctors
2	Associate Nurse/Enrolled Nurse/Nursing Assistant	117,889	133,420	151,144	16.77	596	Democratic Republi	Base Estimate	3221 - Nursing associate professionals	Low Income	Central	Nursing Personnel
3	Audiologist	346	391	439	0.05	205,728	Democratic Republi	Base Estimate	2266 - Audiologists and speech therapists	Low Income	Central	Other Health Workers
4	Cardiologist	572	663	766	0.08	117,765	Democratic Republi	Base Estimate	2212 - Specialist medical practitioners	Low Income	Central	Medical Doctors
5	Cardiothoracic Surgeon	128	149	173	0.02	521,888	Democratic Republi	Base Estimate	2212 - Specialist medical practitioners	Low Income	Central	Medical Doctors
6	Clinical Officer/Physician Assistant	16,028	16,776	17,819	1.99	5,016	Democratic Republi	Base Estimate	3256 - Medical assistants	Low Income	Central	Other Health Workers
7	Clinical Pharmacist	2,727	3,099	3,547	0.39	25,342	Democratic Republi	Base Estimate	2262 - Pharmacists	Low Income	Central	Pharmacist
8	Clinical Psychologist	7,143	8,006	8,994	1.00	10,021	Democratic Republi	Base Estimate	2634 - Psychologists	Low Income	Central	Other Health Workers
9	Community health worker/Village health worker	87,988	92,824	98,039	10.90	917	Democratic Republi	Base Estimate	3253 - Community health workers	Low Income	Central	Other Health Workers
10	Dental Surgery Assistant	8,819	10,247	11,959	1.33	7,521	Democratic Republi	Base Estimate	3251 - Dental assistants and therapists	Low Income	Central	Other Health Workers
11	Dental Therapist	5,715	6,641	7,750	0.86	11,607	Democratic Republi	Base Estimate	3251 - Dental assistants and therapists	Low Income	Central	Other Health Workers
12	Dentist	6,454	7,410	8,652	0.96	10,369	Democratic Republi	Base Estimate	2261 - Dentists	Low Income	Central	Dentist
13	Dermatologist	348	396	449	0.05	200,984	Democratic Republi	Base Estimate	2212 - Specialist medical practitioners	Low Income	Central	Medical Doctors
14	Endocrinologist	373	453	560	0.06	158,611	Democratic Republi	Base Estimate	2212 - Specialist medical practitioners	Low Income	Central	Medical Doctors
15	ENT Surgeon	901	1,041	1,202	0.13	74,993	Democratic Republi	Base Estimate	2212 - Specialist medical practitioners	Low Income	Central	Medical Doctors
16	Environmental Health Officer	4,449	5,051	5,714	0.63	15,808	Democratic Republi	Base Estimate	2263 - Environmental and occupational health and hygiene professionals	Low Income	Central	Other Health Workers
17	Gastroenterologist	752	793	852	0.10	104,986	Democratic Republi	Base Estimate	2212 - Specialist medical practitioners	Low Income	Central	Medical Doctors
18	General Medical Practitioner (Generalist Doctor)	38,200	42,903	48,284	5.36	1,865	Democratic Republi	Base Estimate	2211 - Generalist medical practitioners	Low Income	Central	Medical Doctors
19	General Surgeon	903	1,053	1,243	0.14	72,155	Democratic Republi	Base Estimate	2212 - Specialist medical practitioners	Low Income	Central	Medical Doctors
20	Haematologist	334	375	420	0.05	215,184	Democratic Republi	Base Estimate	2212 - Specialist medical practitioners	Low Income	Central	Medical Doctors
21	Health Promoter/Health Educator	1,232	1,289	1,362	0.15	65,772	Democratic Republi	Base Estimate	2269 - Health professionals not elsewhere classified	Low Income	Central	Other Health Workers
22	Infectious Diseases Specialist	133	150	170	0.02	529,996	Democratic Republi	Base Estimate	2212 - Specialist medical practitioners	Low Income	Central	Medical Doctors
23	Intensive Care Nurse	3,082	3,483	3,932	0.44	22,945	Democratic Republi	Base Estimate	2221 - Nursing professionals	Low Income	Central	Nursing Personnel
24	Medical Laboratory Scientist	11,219	12,497	14,059	1.57	6,384	Democratic Republi	Base Estimate	3212 - Medical and pathology laboratory technicians	Low Income	Central	Other Health Workers
25	Medical Laboratory Technician	15,380	15,664	16,141	1.81	5,532	Democratic Republi	Base Estimate	3212 - Medical and pathology laboratory technicians	Low Income	Central	Other Health Workers
26	Medical Social Worker	3,353	3,573	3,800	0.42	23,688	Democratic Republi	Base Estimate	1344 - Social welfare managers	Low Income	Central	Other Health Workers
27	Mental Health Nurse	5,573	6,408	7,498	0.84	11,934	Democratic Republi	Base Estimate	2221 - Nursing professionals	Low Income	Central	Nursing Personnel
28	Midwife	70,033	77,244	85,855	9.55	1,047	Democratic Republi	Base Estimate	2222 - Midwifery professionals	Low Income	Central	Midwifery Personnel
29	Nephrologist	720	852	1,026	0.12	86,903	Democratic Republi	Base Estimate	2212 - Specialist medical practitioners	Low Income	Central	Medical Doctors
30	Neuro-Surgeon	300	352	413	0.05	217,475	Democratic Republi	Base Estimate	2212 - Specialist medical practitioners	Low Income	Central	Medical Doctors
31	Nurse Anaesthetist	1,643	1,965	2,346	0.26	38,322	Democratic Republi	Base Estimate	2221 - Nursing professionals	Low Income	Central	Nursing Personnel
32	Nutritionist	13,092	13,863	14,618	1.62	6,172	Democratic Republi	Base Estimate	2265 - Dieticians and nutritionists	Low Income	Central	Other Health Workers
33	Obstetrician & Gynaecologist	6,596	7,544	8,640	0.96	10,429	Democratic Republi	Base Estimate	2212 - Specialist medical practitioners	Low Income	Central	Medical Doctors
34	Occupational Therapist	1,519	1,788	2,111	0.23	42,564	Democratic Republi	Base Estimate	2269 - Health professionals not elsewhere classified	Low Income	Central	Other Health Workers
35	Oncology Nurse	559	664	799	0.09	111,920	Democratic Republi	Base Estimate	2221 - Nursing professionals	Low Income	Central	Nursing Personnel
36	Operating Theatre Nurse	8,737	10,173	11,995	1.34	7,463	Democratic Republi	Base Estimate	2221 - Nursing professionals	Low Income	Central	Nursing Personnel
37	Ophthalmic Nurse	1,048	1,220	1,428	0.16	62,853	Democratic Republi	Base Estimate	2221 - Nursing professionals	Low Income	Central	Nursing Personnel
38	Ophthalmologist	307	361	428	0.05	209,618	Democratic Republi	Base Estimate	2212 - Specialist medical practitioners	Low Income	Central	Medical Doctors
39	Optometrist	1,194	1,381	1,601	0.18	56,198	Democratic Republi	Base Estimate	2267 - Optometrists and ophthalmic opticians	Low Income	Central	Other Health Workers
40	Orthopaedic Nurse	1,501	1,786	2,116	0.24	42,550	Democratic Republi	Base Estimate	2221 - Nursing professionals	Low Income	Central	Nursing Personnel
41	Orthopaedic Surgeon	2,861	3,454	4,179	0.47	21,456	Democratic Republi	Base Estimate	2212 - Specialist medical practitioners	Low Income	Central	Medical Doctors
42	Orthopaedic Technologist	2,578	2,965	3,401	0.38	26,535	Democratic Republi	Base Estimate	3214 - Medical and dental prosthetic technicians	Low Income	Central	Other Health Workers
43	Paediatric Nurse	11,018	12,220	13,384	1.48	6,752	Democratic Republi	Base Estimate	2221 - Nursing professionals	Low Income	Central	Nursing Personnel
44	Paediatric Surgeon	333	367	399	0.04	226,782	Democratic Republi	Base Estimate	2212 - Specialist medical practitioners	Low Income	Central	Medical Doctors
45	Paediatrician	3,976	4,525	5,151	0.57	17,490	Democratic Republi	Base Estimate	2212 - Specialist medical practitioners	Low Income	Central	Medical Doctors
46	Pathologist	818	948	1,115	0.12	80,185	Democratic Republi	Base Estimate	2212 - Specialist medical practitioners	Low Income	Central	Medical Doctors
47	Pharmacist	9,727	10,559	11,497	1.28	7,833	Democratic Republi	Base Estimate	2262 - Pharmacists	Low Income	Central	Pharmacist

S/N	Health Professionals	2022	2026	2030	Density per 10,000 population	Required Population ratio (1 professional is to xxx population)	Name of Country	Modelling Scenario	ISCO-08 Match	Income Group Classification	Sub-Regional Grouping	SDG 3c Occupation
48	Pharmacy Technician	9,642	10,946	12,450	1.38	7,237	Democratic Republi	Base Estimate	3213 - Pharmaceutical technicians and assistants	Low Income	Central	Other Health Workers
49	Physician	9,701	10,979	12,504	1.39	7,187	Democratic Republi	Base Estimate	2212 - Specialist medical practitioners	Low Income	Central	Medical Doctors
50	Physiotherapist	2,295	2,637	3,029	0.34	29,763	Democratic Republi	Base Estimate	2264 - Physiotherapists	Low Income	Central	Other Health Workers
51	Plastic Surgeon	490	580	684	0.08	131,784	Democratic Republi	Base Estimate	2212 - Specialist medical practitioners	Low Income	Central	Medical Doctors
52	Psychiatrist	5,208	5,913	6,816	0.76	13,150	Democratic Republi	Base Estimate	2212 - Specialist medical practitioners	Low Income	Central	Medical Doctors
53	Radiation Oncologist	176	214	261	0.03	343,066	Democratic Republi	Base Estimate	2212 - Specialist medical practitioners	Low Income	Central	Medical Doctors
54	Radiographer (Diagnostics and Therapy)	6,165	7,198	8,481	0.95	10,572	Democratic Republi	Base Estimate	3211 - Medical imaging and therapeutic equipment technicians	Low Income	Central	Other Health Workers
55	Radiologist	2,253	2,635	3,100	0.35	28,954	Democratic Republi	Base Estimate	2212 - Specialist medical practitioners	Low Income	Central	Medical Doctors
56	Registered General Nurse / State Certified Nurse	233,016	253,913	279,392	31.19	321	Democratic Republi	Base Estimate	2221 - Nursing professionals	Low Income	Central	Nursing Personnel
57	Renal Nurse	9,553	11,301	13,612	1.53	6,552	Democratic Republi	Base Estimate	2221 - Nursing professionals	Low Income	Central	Nursing Personnel
58	Respiratory Physician	710	812	928	0.10	97,172	Democratic Republi	Base Estimate	2212 - Specialist medical practitioners	Low Income	Central	Medical Doctors
59	Rheumatologist	255	294	338	0.04	266,658	Democratic Republi	Base Estimate	2212 - Specialist medical practitioners	Low Income	Central	Medical Doctors
60	Speech Therapist	881	1,004	1,142	0.13	78,973	Democratic Republi	Base Estimate	2266 - Audiologists and speech therapists	Low Income	Central	Other Health Workers
61	Urologist	87	104	128	0.01	696,133	Democratic Republi	Base Estimate	2212 - Specialist medical practitioners	Low Income	Central	Medical Doctors
1	Anaesthesiologist	13	14	16	0.11	89,515	Equatorial Guinea	Base Estimate	2212 - Specialist medical practitioners	High Income and Upper Middle Income	West	Medical Doctors
2	Associate Nurse/Enrolled Nurse/Nursing Assistant	1,864	2,097	2,354	16.69	599	Equatorial Guinea	Base Estimate	3221 - Nursing associate professionals	High Income and Upper Middle Income	West	Nursing Personnel
3	Audiologist	5	5	6	0.04	248,855	Equatorial Guinea	Base Estimate	2266 - Audiologists and speech therapists	High Income and Upper Middle Income	West	Other Health Workers
4	Cardiologist	9	10	11	0.08	127,477	Equatorial Guinea	Base Estimate	2212 - Specialist medical practitioners	High Income and Upper Middle Income	West	Medical Doctors
5	Cardiothoracic Surgeon	2	2	2	0.01	736,208	Equatorial Guinea	Base Estimate	2212 - Specialist medical practitioners	High Income and Upper Middle Income	West	Medical Doctors
6	Clinical Officer/Physician Assistant	380	436	500	3.55	2,815	Equatorial Guinea	Base Estimate	3256 - Medical assistants	High Income and Upper Middle Income	West	Other Health Workers
7	Clinical Pharmacist	44	50	55	0.39	25,526	Equatorial Guinea	Base Estimate	2262 - Pharmacists	High Income and Upper Middle Income	West	Pharmacist
8	Clinical Psychologist	111	125	141	0.99	10,056	Equatorial Guinea	Base Estimate	2634 - Psychologists	High Income and Upper Middle Income	West	Other Health Workers
9	Community health worker/Village health worker	1,750	1,968	2,210	15.66	639	Equatorial Guinea	Base Estimate	3253 - Community health workers	High Income and Upper Middle Income	West	Other Health Workers
10	Dental Surgery Assistant	165	188	216	1.53	6,557	Equatorial Guinea	Base Estimate	3251 - Dental assistants and therapists	High Income and Upper Middle Income	West	Other Health Workers
11	Dental Therapist	107	122	140	0.99	10,129	Equatorial Guinea	Base Estimate	3251 - Dental assistants and therapists	High Income and Upper Middle Income	West	Other Health Workers
12	Dentist	118	135	155	1.09	9,137	Equatorial Guinea	Base Estimate	2261 - Dentists	High Income and Upper Middle Income	West	Dentist
13	Dermatologist	5	6	7	0.05	214,337	Equatorial Guinea	Base Estimate	2212 - Specialist medical practitioners	High Income and Upper Middle Income	West	Medical Doctors
14	Endocrinologist	8	9	11	0.08	128,747	Equatorial Guinea	Base Estimate	2212 - Specialist medical practitioners	High Income and Upper Middle Income	West	Medical Doctors
15	ENT Surgeon	14	16	18	0.13	79,998	Equatorial Guinea	Base Estimate	2212 - Specialist medical practitioners	High Income and Upper Middle Income	West	Medical Doctors
16	Environmental Health Officer	70	76	83	0.59	17,039	Equatorial Guinea	Base Estimate	2263 - Environmental and occupational health and hygiene professionals	High Income and Upper Middle Income	West	Other Health Workers
17	Gastroenterologist	8	9	9	0.06	156,808	Equatorial Guinea	Base Estimate	2212 - Specialist medical practitioners	High Income and Upper Middle Income	West	Medical Doctors
18	General Medical Practitioner (Generalist Doctor)	697	788	888	6.30	1,588	Equatorial Guinea	Base Estimate	2211 - Generalist medical practitioners	High Income and Upper Middle Income	West	Medical Doctors
19	General Surgeon	16	18	21	0.15	68,695	Equatorial Guinea	Base Estimate	2212 - Specialist medical practitioners	High Income and Upper Middle Income	West	Medical Doctors
20	Haematologist	4	5	5	0.03	294,134	Equatorial Guinea	Base Estimate	2212 - Specialist medical practitioners	High Income and Upper Middle Income	West	Medical Doctors
21	Health Promoter/Health Educator	26	29	33	0.24	42,156	Equatorial Guinea	Base Estimate	2269 - Health professionals not elsewhere classified	High Income and Upper Middle Income	West	Other Health Workers
22	Infectious Diseases Specialist	2	2	2	0.01	693,451	Equatorial Guinea	Base Estimate	2212 - Specialist medical practitioners	High Income and Upper Middle Income	West	Medical Doctors
23	Intensive Care Nurse	43	47	51	0.36	27,878	Equatorial Guinea	Base Estimate	2221 - Nursing professionals	High Income and Upper Middle Income	West	Nursing Personnel
24	Medical Laboratory Scientist	253	294	342	2.44	4,103	Equatorial Guinea	Base Estimate	3212 - Medical and pathology laboratory technicians	High Income and Upper Middle Income	West	Other Health Workers
25	Medical Laboratory Technician	400	464	538	3.82	2,618	Equatorial Guinea	Base Estimate	3212 - Medical and pathology laboratory technicians	High Income and Upper Middle Income	West	Other Health Workers
26	Medical Social Worker	33	34	35	0.25	39,929	Equatorial Guinea	Base Estimate	1344 - Social welfare managers	High Income and Upper Middle Income	West	Other Health Workers
27	Mental Health Nurse	115	131	148	1.05	9,517	Equatorial Guinea	Base Estimate	2221 - Nursing professionals	High Income and Upper Middle Income	West	Nursing Personnel
28	Midwife	1,036	1,129	1,232	8.70	1,149	Equatorial Guinea	Base Estimate	2222 - Midwifery professionals	High Income and Upper Middle Income	West	Midwifery Personnel
29	Nephrologist	16	18	21	0.15	66,068	Equatorial Guinea	Base Estimate	2212 - Specialist medical practitioners	High Income and Upper Middle Income	West	Medical Doctors
30	Neuro-Surgeon	5	5	6	0.04	250,382	Equatorial Guinea	Base Estimate	2212 - Specialist medical practitioners	High Income and Upper Middle Income	West	Medical Doctors
31	Nurse Anaesthetist	29	33	38	0.27	37,117	Equatorial Guinea	Base Estimate	2221 - Nursing professionals	High Income and Upper Middle Income	West	Nursing Personnel
32	Nutritionist	115	115	114	0.81	12,303	Equatorial Guinea	Base Estimate	2265 - Dieticians and nutritionists	High Income and Upper Middle Income	West	Other Health Workers
33	Obstetrician & Gynaecologist	109	121	134	0.95	10,574	Equatorial Guinea	Base Estimate	2212 - Specialist medical practitioners	High Income and Upper Middle Income	West	Medical Doctors
34	Occupational Therapist	26	30	34	0.24	41,980	Equatorial Guinea	Base Estimate	2269 - Health professionals not elsewhere classified	High Income and Upper Middle Income	West	Other Health Workers
35	Oncology Nurse	12	14	16	0.11	89,576	Equatorial Guinea	Base Estimate	2221 - Nursing professionals	High Income and Upper Middle Income	West	Nursing Personnel
36	Operating Theatre Nurse	168	191	215	1.53	6,547	Equatorial Guinea	Base Estimate	2221 - Nursing professionals	High Income and Upper Middle Income	West	Nursing Personnel
37	Ophthalmic Nurse	22	25	28	0.20	50,774	Equatorial Guinea	Base Estimate	2221 - Nursing professionals	High Income and Upper Middle Income	West	Nursing Personnel
38	Ophthalmologist	5	6	7	0.05	201,504	Equatorial Guinea	Base Estimate	2212 - Specialist medical practitioners	High Income and Upper Middle Income	West	Medical Doctors
39	Optometrist	16	18	20	0.14	69,806	Equatorial Guinea	Base Estimate	2267 - Optometrists and ophthalmic opticians	High Income and Upper Middle Income	West	Other Health Workers
40	Orthopaedic Nurse	24	27	30	0.21	47,293	Equatorial Guinea	Base Estimate	2221 - Nursing professionals	High Income and Upper Middle Income	West	Nursing Personnel
41	Orthopaedic Surgeon	60	71	83	0.58	17,096	Equatorial Guinea	Base Estimate	2212 - Specialist medical practitioners	High Income and Upper Middle Income	West	Medical Doctors
42	Orthopaedic Technologist	39	44	48	0.34	29,683	Equatorial Guinea	Base Estimate	3214 - Medical and dental prosthetic technicians	High Income and Upper Middle Income	West	Other Health Workers
43	Paediatric Nurse	138	146	152	1.08	9,288	Equatorial Guinea	Base Estimate	2221 - Nursing professionals	High Income and Upper Middle Income	West	Nursing Personnel
44	Paediatric Surgeon	4	4	4	0.03	314,496	Equatorial Guinea	Base Estimate	2212 - Specialist medical practitioners	High Income and Upper Middle Income	West	Medical Doctors
45	Paediatrician	67	73	80	0.56	17,747	Equatorial Guinea	Base Estimate	2212 - Specialist medical practitioners	High Income and Upper Middle Income	West	Medical Doctors
46	Pathologist	16	18	20	0.14	69,916	Equatorial Guinea	Base Estimate	2212 - Specialist medical practitioners	High Income and Upper Middle Income	West	Medical Doctors
47	Pharmacist	148	159	170	1.20	8,326	Equatorial Guinea	Base Estimate	2262 - Pharmacists	High Income and Upper Middle Income	West	Pharmacist
48	Pharmacy Technician	160	178	198	1.40	7,147	Equatorial Guinea	Base Estimate	3213 - Pharmaceutical technicians and assistants	High Income and Upper Middle Income	West	Other Health Workers
49	Physician	164	185	209	1.48	6,762	Equatorial Guinea	Base Estimate	2212 - Specialist medical practitioners	High Income and Upper Middle Income	West	Medical Doctors
50	Physiotherapist	36	40	44	0.31	32,084	Equatorial Guinea	Base Estimate	2264 - Physiotherapists	High Income and Upper Middle Income	West	Other Health Workers
51	Plastic Surgeon	8	9	11	0.08	130,368	Equatorial Guinea	Base Estimate	2212 - Specialist medical practitioners	High Income and Upper Middle Income	West	Medical Doctors
52	Psychiatrist	98	110	123	0.87	11,433	Equatorial Guinea	Base Estimate	2212 - Specialist medical practitioners	High Income and Upper Middle Income	West	Medical Doctors
53	Radiation Oncologist	4	4	5	0.04	272,005	Equatorial Guinea	Base Estimate	2212 - Specialist medical practitioners	High Income and Upper Middle Income	West	Medical Doctors
54	Radiographer (Diagnostics and Therapy)	114	130	147	1.04	9,573	Equatorial Guinea	Base Estimate	3211 - Medical imaging and therapeutic equipment technicians	High Income and Upper Middle Income	West	Other Health Workers
55	Radiologist	40	45	51	0.36	27,571	Equatorial Guinea	Base Estimate	2212 - Specialist medical practitioners	High Income and Upper Middle Income	West	Medical Doctors
56	Registered General Nurse / State Certified Nurse	3,150	3,525	3,922	27.83	359	Equatorial Guinea	Base Estimate	2221 - Nursing professionals	High Income and Upper Middle Income	West	Nursing Personnel
57	Renal Nurse	211	245	282	2.01	4,980	Equatorial Guinea	Base Estimate	2221 - Nursing professionals	High Income and Upper Middle Income	West	Nursing Personnel
58	Respiratory Physician	11	13	14	0.10	102,790	Equatorial Guinea	Base Estimate	2212 - Specialist medical practitioners	High Income and Upper Middle Income	West	Medical Doctors
59	Rheumatologist	4	5	5	0.04	269,628	Equatorial Guinea	Base Estimate	2212 - Specialist medical practitioners	High Income and Upper Middle Income	West	Medical Doctors
60	Speech Therapist	13	15	16	0.11	89,807	Equatorial Guinea	Base Estimate	2266 - Audiologists and speech therapists	High Income and Upper Middle Income	West	Other Health Workers
61	Urologist	2	2	2	0.02	567,771	Equatorial Guinea	Base Estimate	2212 - Specialist medical practitioners	High Income and Upper Middle Income	West	Medical Doctors
1	Anaesthesiologist	39	43	48	0.14	73,954	Eritrea	Base Estimate	2212 - Specialist medical practitioners	Low Income	East	Medical Doctors
2	Associate Nurse/Enrolled Nurse/Nursing Assistant	4,128	4,407	4,768	13.44	744	Eritrea	Base Estimate	3221 - Nursing associate professionals	Low Income	East	Nursing Personnel
3	Audiologist	15	16	18	0.05	201,062	Eritrea	Base Estimate	2266 - Audiologists and speech therapists	Low Income	East	Other Health Workers
4	Cardiologist	24	27	31	0.09	116,104	Eritrea	Base Estimate	2212 - Specialist medical practitioners	Low Income	East	Medical Doctors
5	Cardiothoracic Surgeon	7	8	9	0.02	413,873	Eritrea	Base Estimate	2212 - Specialist medical practitioners	Low Income	East	Medical Doctors
6	Clinical Officer/Physician Assistant	423	439	469	1.33	7,492	Eritrea	Base Estimate	3256 - Medical assistants	Low Income	East	Other Health Workers
7	Clinical Pharmacist	107	117	131	0.37	26,969	Eritrea	Base Estimate	2262 - Pharmacists	Low Income	East	Pharmacist
8	Clinical Psychologist	264	289	322	0.91	11,019	Eritrea	Base Estimate	2634 - Psychologists	Low Income	East	Other Health Workers
9	Community health worker/Village health worker	2,782	2,876	3,001	8.45	1,183	Eritrea	Base Estimate	3253 - Community health workers	Low Income	East	Other Health Workers

S/N	Health Professionals	2022	2026	2030	Density per 10,000 population	Required Population ratio (1 professional is to xxx population)	Name of Country	Modelling Scenario	ISCO-08 Match	Income Group Classification	Sub-Regional Grouping	SDG 3c Occupation
10	Dental Surgery Assistant	410	456	523	1.47	6,809	Eritrea	Base Estimate	3251 - Dental assistants and therapists	Low Income	East	Other Health Workers
11	Dental Therapist	265	295	338	0.95	10,539	Eritrea	Base Estimate	3251 - Dental assistants and therapists	Low Income	East	Other Health Workers
12	Dentist	338	374	438	1.23	8,120	Eritrea	Base Estimate	2261 - Dentists	Low Income	East	Dentist
13	Dermatologist	13	14	16	0.04	228,848	Eritrea	Base Estimate	2212 - Specialist medical practitioners	Low Income	East	Medical Doctors
14	Endocrinologist	12	15	18	0.05	195,644	Eritrea	Base Estimate	2212 - Specialist medical practitioners	Low Income	East	Medical Doctors
15	ENT Surgeon	43	48	53	0.15	67,223	Eritrea	Base Estimate	2212 - Specialist medical practitioners	Low Income	East	Medical Doctors
16	Environmental Health Officer	176	189	204	0.57	17,456	Eritrea	Base Estimate	2263 - Environmental and occupational health and hygiene professionals	Low Income	East	Other Health Workers
17	Gastroenterologist	27	27	28	0.08	122,429	Eritrea	Base Estimate	2212 - Specialist medical practitioners	Low Income	East	Medical Doctors
18	General Medical Practitioner (Generalist Doctor)	1,294	1,399	1,539	4.35	2,297	Eritrea	Base Estimate	2211 - Generalist medical practitioners	Low Income	East	Medical Doctors
19	General Surgeon	27	32	40	0.11	88,067	Eritrea	Base Estimate	2212 - Specialist medical practitioners	Low Income	East	Medical Doctors
20	Haematologist	15	16	17	0.05	212,140	Eritrea	Base Estimate	2212 - Specialist medical practitioners	Low Income	East	Medical Doctors
21	Health Promoter/Health Educator	38	39	41	0.12	86,258	Eritrea	Base Estimate	2269 - Health professionals not elsewhere classified	Low Income	East	Other Health Workers
22	Infectious Diseases Specialist	4	4	5	0.01	743,247	Eritrea	Base Estimate	2212 - Specialist medical practitioners	Low Income	East	Medical Doctors
23	Intensive Care Nurse	116	123	131	0.37	27,263	Eritrea	Base Estimate	2221 - Nursing professionals	Low Income	East	Nursing Personnel
24	Medical Laboratory Scientist	371	403	449	1.27	7,849	Eritrea	Base Estimate	3212 - Medical and pathology laboratory technicians	Low Income	East	Other Health Workers
25	Medical Laboratory Technician	308	333	366	1.04	9,659	Eritrea	Base Estimate	3212 - Medical and pathology laboratory technicians	Low Income	East	Other Health Workers
26	Medical Social Worker	131	132	135	0.38	26,100	Eritrea	Base Estimate	1344 - Social welfare managers	Low Income	East	Other Health Workers
27	Mental Health Nurse	238	261	300	0.86	11,682	Eritrea	Base Estimate	2221 - Nursing professionals	Low Income	East	Nursing Personnel
28	Midwife	2,546	2,724	2,991	8.40	1,190	Eritrea	Base Estimate	2222 - Midwifery professionals	Low Income	East	Midwifery Personnel
29	Nephrologist	33	38	45	0.13	77,380	Eritrea	Base Estimate	2212 - Specialist medical practitioners	Low Income	East	Medical Doctors
30	Neuro-Surgeon	13	15	16	0.05	216,433	Eritrea	Base Estimate	2212 - Specialist medical practitioners	Low Income	East	Medical Doctors
31	Nurse Anaesthetist	90	104	121	0.34	29,405	Eritrea	Base Estimate	2221 - Nursing professionals	Low Income	East	Nursing Personnel
32	Nutritionist	548	548	551	1.56	6,430	Eritrea	Base Estimate	2265 - Dieticians and nutritionists	Low Income	East	Other Health Workers
33	Obstetrician & Gynaecologist	256	278	307	0.86	11,564	Eritrea	Base Estimate	2212 - Specialist medical practitioners	Low Income	East	Medical Doctors
34	Occupational Therapist	69	78	89	0.25	39,895	Eritrea	Base Estimate	2269 - Health professionals not elsewhere classified	Low Income	East	Other Health Workers
35	Oncology Nurse	28	32	38	0.11	91,714	Eritrea	Base Estimate	2221 - Nursing professionals	Low Income	East	Nursing Personnel
36	Operating Theatre Nurse	358	402	466	1.33	7,525	Eritrea	Base Estimate	2221 - Nursing professionals	Low Income	East	Nursing Personnel
37	Ophthalmic Nurse	45	50	56	0.16	63,162	Eritrea	Base Estimate	2221 - Nursing professionals	Low Income	East	Nursing Personnel
38	Ophthalmologist	14	16	18	0.05	197,624	Eritrea	Base Estimate	2212 - Specialist medical practitioners	Low Income	East	Medical Doctors
39	Optometrist	57	65	74	0.21	47,864	Eritrea	Base Estimate	2267 - Optometrists and ophthalmic opticians	Low Income	East	Other Health Workers
40	Orthopaedic Nurse	67	75	85	0.24	41,878	Eritrea	Base Estimate	2221 - Nursing professionals	Low Income	East	Nursing Personnel
41	Orthopaedic Surgeon	82	108	149	0.44	22,563	Eritrea	Base Estimate	2212 - Specialist medical practitioners	Low Income	East	Medical Doctors
42	Orthopaedic Technologist	110	119	131	0.37	27,223	Eritrea	Base Estimate	3214 - Medical and dental prosthetic technicians	Low Income	East	Other Health Workers
43	Paediatric Nurse	445	466	488	1.37	7,280	Eritrea	Base Estimate	2221 - Nursing professionals	Low Income	East	Nursing Personnel
44	Paediatric Surgeon	12	12	13	0.04	282,500	Eritrea	Base Estimate	2212 - Specialist medical practitioners	Low Income	East	Medical Doctors
45	Paediatrician	186	201	220	0.62	16,118	Eritrea	Base Estimate	2212 - Specialist medical practitioners	Low Income	East	Medical Doctors
46	Pathologist	31	34	39	0.11	89,954	Eritrea	Base Estimate	2212 - Specialist medical practitioners	Low Income	East	Medical Doctors
47	Pharmacist	290	297	309	0.87	11,455	Eritrea	Base Estimate	2262 - Pharmacists	Low Income	East	Pharmacist
48	Pharmacy Technician	352	381	418	1.18	8,495	Eritrea	Base Estimate	3213 - Pharmaceutical technicians and assistants	Low Income	East	Other Health Workers
49	Physician	345	377	419	1.18	8,455	Eritrea	Base Estimate	2212 - Specialist medical practitioners	Low Income	East	Medical Doctors
50	Physiotherapist	97	106	117	0.33	30,505	Eritrea	Base Estimate	2264 - Physiotherapists	Low Income	East	Other Health Workers
51	Plastic Surgeon	58	64	71	0.20	50,469	Eritrea	Base Estimate	2212 - Specialist medical practitioners	Low Income	East	Medical Doctors
52	Psychiatrist	213	230	258	0.73	13,631	Eritrea	Base Estimate	2212 - Specialist medical practitioners	Low Income	East	Medical Doctors
53	Radiation Oncologist	10	12	14	0.04	245,024	Eritrea	Base Estimate	2212 - Specialist medical practitioners	Low Income	East	Medical Doctors
54	Radiographer (Diagnostics and Therapy)	236	266	312	0.89	11,234	Eritrea	Base Estimate	3211 - Medical imaging and therapeutic equipment technicians	Low Income	East	Other Health Workers
55	Radiologist	85	96	113	0.32	31,145	Eritrea	Base Estimate	2212 - Specialist medical practitioners	Low Income	East	Medical Doctors
56	Registered General Nurse / State Certified Nurse	9,922	10,379	11,063	31.42	318	Eritrea	Base Estimate	2221 - Nursing professionals	Low Income	East	Nursing Personnel
57	Renal Nurse	436	499	594	1.71	5,848	Eritrea	Base Estimate	2221 - Nursing professionals	Low Income	East	Nursing Personnel
58	Respiratory Physician	40	43	47	0.13	75,089	Eritrea	Base Estimate	2212 - Specialist medical practitioners	Low Income	East	Medical Doctors
59	Rheumatologist	11	12	13	0.04	274,048	Eritrea	Base Estimate	2212 - Specialist medical practitioners	Low Income	East	Medical Doctors
60	Speech Therapist	39	42	45	0.13	78,413	Eritrea	Base Estimate	2266 - Audiologists and speech therapists	Low Income	East	Other Health Workers
61	Urologist	4	4	5	0.02	655,395	Eritrea	Base Estimate	2212 - Specialist medical practitioners	Low Income	East	Medical Doctors
1	Anaesthesiologist	15	16	18	0.16	64,291	Eswatini	Base Estimate	2212 - Specialist medical practitioners	Lower-middle Income	Southern	Medical Doctors
2	Associate Nurse/Enrolled Nurse/Nursing Assistant	1,742	1,829	1,956	16.92	591	Eswatini	Base Estimate	3221 - Nursing associate professionals	Lower-middle Income	Southern	Nursing Personnel
3	Audiologist	5	5	5	0.05	214,101	Eswatini	Base Estimate	2266 - Audiologists and speech therapists	Lower-middle Income	Southern	Other Health Workers
4	Cardiologist	12	13	15	0.13	78,449	Eswatini	Base Estimate	2212 - Specialist medical practitioners	Lower-middle Income	Southern	Medical Doctors
5	Cardiothoracic Surgeon	2	2	2	0.02	537,457	Eswatini	Base Estimate	2212 - Specialist medical practitioners	Lower-middle Income	Southern	Medical Doctors
6	Clinical Officer/Physician Assistant	242	250	267	2.32	4,303	Eswatini	Base Estimate	3256 - Medical assistants	Lower-middle Income	Southern	Other Health Workers
7	Clinical Pharmacist	37	39	44	0.38	26,330	Eswatini	Base Estimate	2262 - Pharmacists	Lower-middle Income	Southern	Pharmacist
8	Clinical Psychologist	72	76	81	0.70	14,223	Eswatini	Base Estimate	2634 - Psychologists	Lower-middle Income	Southern	Other Health Workers
9	Community health worker/Village health worker	803	814	836	7.25	1,380	Eswatini	Base Estimate	3253 - Community health workers	Lower-middle Income	Southern	Other Health Workers
10	Dental Surgery Assistant	164	176	193	1.67	6,006	Eswatini	Base Estimate	3251 - Dental assistants and therapists	Lower-middle Income	Southern	Other Health Workers
11	Dental Therapist	106	114	125	1.08	9,293	Eswatini	Base Estimate	3251 - Dental assistants and therapists	Lower-middle Income	Southern	Other Health Workers
12	Dentist	132	139	153	1.32	7,603	Eswatini	Base Estimate	2261 - Dentists	Lower-middle Income	Southern	Dentist
13	Dermatologist	5	5	5	0.05	221,342	Eswatini	Base Estimate	2212 - Specialist medical practitioners	Lower-middle Income	Southern	Medical Doctors
14	Endocrinologist	8	8	10	0.08	118,782	Eswatini	Base Estimate	2212 - Specialist medical practitioners	Lower-middle Income	Southern	Medical Doctors
15	ENT Surgeon	16	17	19	0.16	62,762	Eswatini	Base Estimate	2212 - Specialist medical practitioners	Lower-middle Income	Southern	Medical Doctors
16	Environmental Health Officer	58	60	62	0.54	18,571	Eswatini	Base Estimate	2263 - Environmental and occupational health and hygiene professionals	Lower-middle Income	Southern	Other Health Workers
17	Gastroenterologist	10	9	10	0.08	119,327	Eswatini	Base Estimate	2212 - Specialist medical practitioners	Lower-middle Income	Southern	Medical Doctors
18	General Medical Practitioner (Generalist Doctor)	564	591	631	5.46	1,830	Eswatini	Base Estimate	2211 - Generalist medical practitioners	Lower-middle Income	Southern	Medical Doctors
19	General Surgeon	17	18	20	0.17	57,819	Eswatini	Base Estimate	2212 - Specialist medical practitioners	Lower-middle Income	Southern	Medical Doctors
20	Haematologist	3	3	3	0.02	418,460	Eswatini	Base Estimate	2212 - Specialist medical practitioners	Lower-middle Income	Southern	Medical Doctors
21	Health Promoter/Health Educator	18	18	19	0.17	59,857	Eswatini	Base Estimate	2269 - Health professionals not elsewhere classified	Lower-middle Income	Southern	Other Health Workers
22	Infectious Diseases Specialist	2	2	2	0.02	625,003	Eswatini	Base Estimate	2212 - Specialist medical practitioners	Lower-middle Income	Southern	Medical Doctors
23	Intensive Care Nurse	30	31	33	0.29	35,015	Eswatini	Base Estimate	2221 - Nursing professionals	Lower-middle Income	Southern	Nursing Personnel
24	Medical Laboratory Scientist	244	260	285	2.48	4,032	Eswatini	Base Estimate	3212 - Medical and pathology laboratory technicians	Lower-middle Income	Southern	Other Health Workers
25	Medical Laboratory Technician	198	211	231	2.01	4,982	Eswatini	Base Estimate	3212 - Medical and pathology laboratory technicians	Lower-middle Income	Southern	Other Health Workers
26	Medical Social Worker	27	27	30	0.27	37,496	Eswatini	Base Estimate	1344 - Social welfare managers	Lower-middle Income	Southern	Other Health Workers
27	Mental Health Nurse	76	80	90	0.78	12,787	Eswatini	Base Estimate	2221 - Nursing professionals	Lower-middle Income	Southern	Nursing Personnel
28	Midwife	853	883	935	8.05	1,242	Eswatini	Base Estimate	2222 - Midwifery professionals	Lower-middle Income	Southern	Midwifery Personnel
29	Nephrologist	19	22	26	0.23	43,355	Eswatini	Base Estimate	2212 - Specialist medical practitioners	Lower-middle Income	Southern	Medical Doctors
30	Neuro-Surgeon	5	6	6	0.05	183,144	Eswatini	Base Estimate	2212 - Specialist medical practitioners	Lower-middle Income	Southern	Medical Doctors
31	Nurse Anaesthetist	41	45	51	0.44	22,573	Eswatini	Base Estimate	2221 - Nursing professionals	Lower-middle Income	Southern	Nursing Personnel
32	Nutritionist	65	65	66	0.57	17,602	Eswatini	Base Estimate	2265 - Dieticians and nutritionists	Lower-middle Income	Southern	Other Health Workers

S/N	Health Professionals	2022	2026	2030	Density per 10,000 population	Required Population ratio (1 professional is to xxx population)	Name of Country	Modelling Scenario	ISCO-08 Match	Income Group Classification	Sub-Regional Grouping	SDG 3c Occupation
33	Obstetrician & Gynaecologist	102	109	121	1.05	9,523	Eswatini	Base Estimate	2212 - Specialist medical practitioners	Lower-middle Income	Southern	Medical Doctors
34	Occupational Therapist	24	27	30	0.26	38,260	Eswatini	Base Estimate	2269 - Health professionals not elsewhere classified	Lower-middle Income	Southern	Other Health Workers
35	Oncology Nurse	15	17	20	0.17	58,385	Eswatini	Base Estimate	2221 - Nursing professionals	Lower-middle Income	Southern	Nursing Personnel
36	Operating Theatre Nurse	160	176	201	1.75	5,715	Eswatini	Base Estimate	2221 - Nursing professionals	Lower-middle Income	Southern	Nursing Personnel
37	Ophthalmic Nurse	21	23	25	0.22	46,262	Eswatini	Base Estimate	2221 - Nursing professionals	Lower-middle Income	Southern	Nursing Personnel
38	Ophthalmologist	7	8	9	0.08	127,714	Eswatini	Base Estimate	2212 - Specialist medical practitioners	Lower-middle Income	Southern	Medical Doctors
39	Optometrist	37	41	46	0.40	25,193	Eswatini	Base Estimate	2267 - Optometrists and ophthalmic opticians	Lower-middle Income	Southern	Other Health Workers
40	Orthopaedic Nurse	22	24	27	0.23	43,116	Eswatini	Base Estimate	2221 - Nursing professionals	Lower-middle Income	Southern	Nursing Personnel
41	Orthopaedic Surgeon	53	59	67	0.58	17,240	Eswatini	Base Estimate	2212 - Specialist medical practitioners	Lower-middle Income	Southern	Medical Doctors
42	Orthopaedic Technologist	35	37	39	0.33	29,867	Eswatini	Base Estimate	3214 - Medical and dental prosthetic technicians	Lower-middle Income	Southern	Other Health Workers
43	Paediatric Nurse	95	97	98	0.85	11,762	Eswatini	Base Estimate	2221 - Nursing professionals	Lower-middle Income	Southern	Nursing Personnel
44	Paediatric Surgeon	3	3	3	0.03	335,452	Eswatini	Base Estimate	2212 - Specialist medical practitioners	Lower-middle Income	Southern	Medical Doctors
45	Paediatrician	59	61	66	0.57	17,437	Eswatini	Base Estimate	2212 - Specialist medical practitioners	Lower-middle Income	Southern	Medical Doctors
46	Pathologist	15	16	19	0.16	61,546	Eswatini	Base Estimate	2212 - Specialist medical practitioners	Lower-middle Income	Southern	Medical Doctors
47	Pharmacist	121	120	122	1.06	9,393	Eswatini	Base Estimate	2262 - Pharmacists	Lower-middle Income	Southern	Pharmacist
48	Pharmacy Technician	138	144	153	1.33	7,539	Eswatini	Base Estimate	3213 - Pharmaceutical technicians and assistants	Lower-middle Income	Southern	Other Health Workers
49	Physician	135	144	158	1.37	7,291	Eswatini	Base Estimate	2212 - Specialist medical practitioners	Lower-middle Income	Southern	Medical Doctors
50	Physiotherapist	33	34	37	0.32	31,267	Eswatini	Base Estimate	2264 - Physiotherapists	Lower-middle Income	Southern	Other Health Workers
51	Plastic Surgeon	11	12	12	0.11	93,751	Eswatini	Base Estimate	2212 - Specialist medical practitioners	Lower-middle Income	Southern	Medical Doctors
52	Psychiatrist	64	67	75	0.65	15,404	Eswatini	Base Estimate	2212 - Specialist medical practitioners	Lower-middle Income	Southern	Medical Doctors
53	Radiation Oncologist	5	6	7	0.06	174,129	Eswatini	Base Estimate	2212 - Specialist medical practitioners	Lower-middle Income	Southern	Medical Doctors
54	Radiographer (Diagnostics and Therapy)	117	128	145	1.27	7,877	Eswatini	Base Estimate	3211 - Medical imaging and therapeutic equipment technicians	Lower-middle Income	Southern	Other Health Workers
55	Radiologist	41	46	53	0.47	21,412	Eswatini	Base Estimate	2212 - Specialist medical practitioners	Lower-middle Income	Southern	Medical Doctors
56	Registered General Nurse / State Certified Nurse	3,281	3,459	3,785	33.02	303	Eswatini	Base Estimate	2221 - Nursing professionals	Lower-middle Income	Southern	Nursing Personnel
57	Renal Nurse	252	286	346	3.04	3,295	Eswatini	Base Estimate	2221 - Nursing professionals	Lower-middle Income	Southern	Nursing Personnel
58	Respiratory Physician	12	13	13	0.12	86,847	Eswatini	Base Estimate	2212 - Specialist medical practitioners	Lower-middle Income	Southern	Medical Doctors
59	Rheumatologist	4	4	5	0.04	240,998	Eswatini	Base Estimate	2212 - Specialist medical practitioners	Lower-middle Income	Southern	Medical Doctors
60	Speech Therapist	13	13	14	0.12	83,267	Eswatini	Base Estimate	2266 - Audiologists and speech therapists	Lower-middle Income	Southern	Other Health Workers
61	Urologist	2	2	3	0.02	403,423	Eswatini	Base Estimate	2212 - Specialist medical practitioners	Lower-middle Income	Southern	Medical Doctors
1	Anaesthesiologist	940	1,062	1,200	0.10	96,680	Ethiopia	Base Estimate	2212 - Specialist medical practitioners	Low Income	East	Medical Doctors
2	Associate Nurse/Enrolled Nurse/Nursing Assistant	132,642	146,179	161,257	13.90	719	Ethiopia	Base Estimate	3221 - Nursing associate professionals	Low Income	East	Nursing Personnel
3	Audiologist	488	541	597	0.05	194,320	Ethiopia	Base Estimate	2266 - Audiologists and speech therapists	Low Income	East	Other Health Workers
4	Cardiologist	715	820	935	0.08	124,113	Ethiopia	Base Estimate	2212 - Specialist medical practitioners	Low Income	East	Medical Doctors
5	Cardiothoracic Surgeon	120	131	142	0.01	818,044	Ethiopia	Base Estimate	2212 - Specialist medical practitioners	Low Income	East	Medical Doctors
6	Clinical Officer/Physician Assistant	30,381	34,463	39,296	3.40	2,944	Ethiopia	Base Estimate	3256 - Medical assistants	Low Income	East	Other Health Workers
7	Clinical Pharmacist	2,863	3,178	3,580	0.31	32,339	Ethiopia	Base Estimate	2262 - Pharmacists	Low Income	East	Pharmacist
8	Clinical Psychologist	7,038	7,759	8,630	0.74	13,437	Ethiopia	Base Estimate	2634 - Psychologists	Low Income	East	Other Health Workers
9	Community health worker/Village health worker	109,829	120,625	131,809	11.36	880	Ethiopia	Base Estimate	3253 - Community health workers	Low Income	East	Other Health Workers
10	Dental Surgery Assistant	10,905	12,327	13,945	1.20	8,319	Ethiopia	Base Estimate	3251 - Dental assistants and therapists	Low Income	East	Other Health Workers
11	Dental Therapist	7,065	7,987	9,035	0.78	12,841	Ethiopia	Base Estimate	3251 - Dental assistants and therapists	Low Income	East	Other Health Workers
12	Dentist	8,330	9,254	10,401	0.90	11,141	Ethiopia	Base Estimate	2261 - Dentists	Low Income	East	Dentist
13	Dermatologist	406	454	505	0.04	229,997	Ethiopia	Base Estimate	2212 - Specialist medical practitioners	Low Income	East	Medical Doctors
14	Endocrinologist	269	312	377	0.03	305,966	Ethiopia	Base Estimate	2212 - Specialist medical practitioners	Low Income	East	Medical Doctors
15	ENT Surgeon	1,376	1,570	1,790	0.15	64,801	Ethiopia	Base Estimate	2212 - Specialist medical practitioners	Low Income	East	Medical Doctors
16	Environmental Health Officer	5,710	6,299	6,905	0.59	16,826	Ethiopia	Base Estimate	2263 - Environmental and occupational health and hygiene professionals	Low Income	East	Other Health Workers
17	Gastroenterologist	864	912	1,001	0.09	115,523	Ethiopia	Base Estimate	2212 - Specialist medical practitioners	Low Income	East	Medical Doctors
18	General Medical Practitioner (Generalist Doctor)	47,288	53,121	59,715	5.15	1,941	Ethiopia	Base Estimate	2211 - Generalist medical practitioners	Low Income	East	Medical Doctors
19	General Surgeon	774	883	1,022	0.09	113,011	Ethiopia	Base Estimate	2212 - Specialist medical practitioners	Low Income	East	Medical Doctors
20	Haematologist	297	321	345	0.03	336,315	Ethiopia	Base Estimate	2212 - Specialist medical practitioners	Low Income	East	Medical Doctors
21	Health Promoter/Health Educator	2,655	2,960	3,298	0.28	35,175	Ethiopia	Base Estimate	2269 - Health professionals not elsewhere classified	Low Income	East	Other Health Workers
22	Infectious Diseases Specialist	99	106	115	0.01	1,005,167	Ethiopia	Base Estimate	2212 - Specialist medical practitioners	Low Income	East	Medical Doctors
23	Intensive Care Nurse	3,264	3,540	3,818	0.33	30,402	Ethiopia	Base Estimate	2221 - Nursing professionals	Low Income	East	Nursing Personnel
24	Medical Laboratory Scientist	11,648	13,178	15,160	1.31	7,620	Ethiopia	Base Estimate	3212 - Medical and pathology laboratory technicians	Low Income	East	Other Health Workers
25	Medical Laboratory Technician	11,759	13,395	15,404	1.33	7,498	Ethiopia	Base Estimate	3212 - Medical and pathology laboratory technicians	Low Income	East	Other Health Workers
26	Medical Social Worker	4,564	4,866	5,214	0.45	22,201	Ethiopia	Base Estimate	1344 - Social welfare managers	Low Income	East	Other Health Workers
27	Mental Health Nurse	7,337	8,264	9,620	0.83	12,013	Ethiopia	Base Estimate	2221 - Nursing professionals	Low Income	East	Nursing Personnel
28	Midwife	82,439	90,498	99,418	8.57	1,167	Ethiopia	Base Estimate	2222 - Midwifery professionals	Low Income	East	Midwifery Personnel
29	Nephrologist	1,013	1,174	1,419	0.12	81,288	Ethiopia	Base Estimate	2212 - Specialist medical practitioners	Low Income	East	Medical Doctors
30	Neuro-Surgeon	279	324	382	0.03	301,614	Ethiopia	Base Estimate	2212 - Specialist medical practitioners	Low Income	East	Medical Doctors
31	Nurse Anaesthetist	2,041	2,344	2,692	0.23	43,086	Ethiopia	Base Estimate	2221 - Nursing professionals	Low Income	East	Nursing Personnel
32	Nutritionist	18,821	19,735	20,550	1.77	5,642	Ethiopia	Base Estimate	2265 - Dieticians and nutritionists	Low Income	East	Other Health Workers
33	Obstetrician & Gynaecologist	8,008	9,119	10,466	0.91	11,037	Ethiopia	Base Estimate	2212 - Specialist medical practitioners	Low Income	East	Medical Doctors
34	Occupational Therapist	1,665	1,902	2,202	0.19	52,590	Ethiopia	Base Estimate	2269 - Health professionals not elsewhere classified	Low Income	East	Other Health Workers
35	Oncology Nurse	802	931	1,112	0.10	103,921	Ethiopia	Base Estimate	2221 - Nursing professionals	Low Income	East	Nursing Personnel
36	Operating Theatre Nurse	11,166	12,816	15,207	1.32	7,595	Ethiopia	Base Estimate	2221 - Nursing professionals	Low Income	East	Nursing Personnel
37	Ophthalmic Nurse	1,539	1,715	1,919	0.17	60,469	Ethiopia	Base Estimate	2221 - Nursing professionals	Low Income	East	Nursing Personnel
38	Ophthalmologist	380	429	491	0.04	235,845	Ethiopia	Base Estimate	2212 - Specialist medical practitioners	Low Income	East	Medical Doctors
39	Optometrist	1,158	1,302	1,466	0.13	79,142	Ethiopia	Base Estimate	2267 - Optometrists and ophthalmic opticians	Low Income	East	Other Health Workers
40	Orthopaedic Nurse	1,280	1,475	1,691	0.15	68,595	Ethiopia	Base Estimate	2221 - Nursing professionals	Low Income	East	Nursing Personnel
41	Orthopaedic Surgeon	3,316	3,997	4,857	0.42	23,744	Ethiopia	Base Estimate	2212 - Specialist medical practitioners	Low Income	East	Medical Doctors
42	Orthopaedic Technologist	3,027	3,360	3,707	0.32	31,335	Ethiopia	Base Estimate	3214 - Medical and dental prosthetic technicians	Low Income	East	Other Health Workers
43	Paediatric Nurse	13,174	14,224	15,137	1.30	7,684	Ethiopia	Base Estimate	2221 - Nursing professionals	Low Income	East	Nursing Personnel
44	Paediatric Surgeon	412	451	488	0.04	238,421	Ethiopia	Base Estimate	2212 - Specialist medical practitioners	Low Income	East	Medical Doctors
45	Paediatrician	4,647	5,171	5,794	0.50	20,018	Ethiopia	Base Estimate	2212 - Specialist medical practitioners	Low Income	East	Medical Doctors
46	Pathologist	866	1,086	1,422	0.12	80,038	Ethiopia	Base Estimate	2212 - Specialist medical practitioners	Low Income	East	Medical Doctors
47	Pharmacist	20,963	23,427	26,072	2.25	4,454	Ethiopia	Base Estimate	2262 - Pharmacists	Low Income	East	Pharmacist
48	Pharmacy Technician	13,539	15,144	16,908	1.46	6,866	Ethiopia	Base Estimate	3213 - Pharmaceutical technicians and assistants	Low Income	East	Other Health Workers
49	Physician	9,286	10,354	11,638	1.00	9,958	Ethiopia	Base Estimate	2212 - Specialist medical practitioners	Low Income	East	Medical Doctors
50	Physiotherapist	2,826	3,149	3,515	0.30	33,009	Ethiopia	Base Estimate	2264 - Physiotherapists	Low Income	East	Other Health Workers
51	Plastic Surgeon	1,195	1,369	1,558	0.13	74,550	Ethiopia	Base Estimate	2212 - Specialist medical practitioners	Low Income	East	Medical Doctors
52	Psychiatrist	6,869	7,611	8,646	0.75	13,377	Ethiopia	Base Estimate	2212 - Specialist medical practitioners	Low Income	East	Medical Doctors
53	Radiation Oncologist	244	288	344	0.03	335,457	Ethiopia	Base Estimate	2212 - Specialist medical practitioners	Low Income	East	Medical Doctors
54	Radiographer (Diagnostics and Therapy)	7,101	8,149	9,547	0.83	12,106	Ethiopia	Base Estimate	3211 - Medical imaging and therapeutic equipment technicians	Low Income	East	Other Health Workers
55	Radiologist	2,554	2,964	3,484	0.30	33,086	Ethiopia	Base Estimate	2212 - Specialist medical practitioners	Low Income	East	Medical Doctors

S/N	Health Professionals	2022	2026	2030	Density per 10,000 population	Required Population ratio (1 professional is to xxx population)	Name of Country	Modelling Scenario	ISCO-08 Match	Income Group Classification	Sub-Regional Grouping	SDG 3c Occupation
56	Registered General Nurse / State Certified Nurse	449,824	491,334	537,673	46.36	216	Ethiopia	Base Estimate	2221 - Nursing professionals	Low Income	East	Nursing Personnel
57	Renal Nurse	13,331	15,457	18,701	1.62	6,169	Ethiopia	Base Estimate	2221 - Nursing professionals	Low Income	East	Nursing Personnel
58	Respiratory Physician	900	1,004	1,118	0.10	103,919	Ethiopia	Base Estimate	2212 - Specialist medical practitioners	Low Income	East	Medical Doctors
59	Rheumatologist	333	377	426	0.04	272,766	Ethiopia	Base Estimate	2212 - Specialist medical practitioners	Low Income	East	Medical Doctors
60	Speech Therapist	1,193	1,328	1,477	0.13	78,598	Ethiopia	Base Estimate	2266 - Audiologists and speech therapists	Low Income	East	Other Health Workers
61	Urologist	141	168	208	0.02	549,898	Ethiopia	Base Estimate	2212 - Specialist medical practitioners	Low Income	East	Medical Doctors
1	Anaesthesiologist	29	32	35	0.16	63,292	Gabon	Base Estimate	2212 - Specialist medical practitioners	High Income and Upper Middle Income	Central	Medical Doctors
2	Associate Nurse/Enrolled Nurse/Nursing Assistant	3,385	3,709	4,045	18.06	554	Gabon	Base Estimate	3221 - Nursing associate professionals	High Income and Upper Middle Income	Central	Nursing Personnel
3	Audiologist	8	9	10	0.04	224,686	Gabon	Base Estimate	2266 - Audiologists and speech therapists	High Income and Upper Middle Income	Central	Other Health Workers
4	Cardiologist	23	26	29	0.13	76,557	Gabon	Base Estimate	2212 - Specialist medical practitioners	High Income and Upper Middle Income	Central	Medical Doctors
5	Cardiothoracic Surgeon	4	5	5	0.02	456,916	Gabon	Base Estimate	2212 - Specialist medical practitioners	High Income and Upper Middle Income	Central	Medical Doctors
6	Clinical Officer/Physician Assistant	673	744	823	3.69	2,707	Gabon	Base Estimate	3256 - Medical assistants	High Income and Upper Middle Income	Central	Other Health Workers
7	Clinical Pharmacist	85	93	103	0.46	21,781	Gabon	Base Estimate	2262 - Pharmacists	High Income and Upper Middle Income	Central	Pharmacist
8	Clinical Psychologist	194	212	231	1.03	9,678	Gabon	Base Estimate	2634 - Psychologists	High Income and Upper Middle Income	Central	Other Health Workers
9	Community health worker/Village health worker	2,361	2,666	3,005	13.48	742	Gabon	Base Estimate	3253 - Community health workers	High Income and Upper Middle Income	Central	Other Health Workers
10	Dental Surgery Assistant	283	314	344	1.54	6,507	Gabon	Base Estimate	3251 - Dental assistants and therapists	High Income and Upper Middle Income	Central	Other Health Workers
11	Dental Therapist	183	204	223	1.00	10,032	Gabon	Base Estimate	3251 - Dental assistants and therapists	High Income and Upper Middle Income	Central	Other Health Workers
12	Dentist	179	196	210	0.94	10,620	Gabon	Base Estimate	2261 - Dentists	High Income and Upper Middle Income	Central	Dentist
13	Dermatologist	9	10	11	0.05	209,001	Gabon	Base Estimate	2212 - Specialist medical practitioners	High Income and Upper Middle Income	Central	Medical Doctors
14	Endocrinologist	19	23	26	0.12	85,183	Gabon	Base Estimate	2212 - Specialist medical practitioners	High Income and Upper Middle Income	Central	Medical Doctors
15	ENT Surgeon	28	32	35	0.16	63,100	Gabon	Base Estimate	2212 - Specialist medical practitioners	High Income and Upper Middle Income	Central	Medical Doctors
16	Environmental Health Officer	111	119	128	0.57	17,482	Gabon	Base Estimate	2263 - Environmental and occupational health and hygiene professionals	High Income and Upper Middle Income	Central	Other Health Workers
17	Gastroenterologist	27	28	28	0.13	79,614	Gabon	Base Estimate	2212 - Specialist medical practitioners	High Income and Upper Middle Income	Central	Medical Doctors
18	General Medical Practitioner (Generalist Doctor)	1,181	1,294	1,410	6.30	1,587	Gabon	Base Estimate	2211 - Generalist medical practitioners	High Income and Upper Middle Income	Central	Medical Doctors
19	General Surgeon	31	35	40	0.18	56,104	Gabon	Base Estimate	2212 - Specialist medical practitioners	High Income and Upper Middle Income	Central	Medical Doctors
20	Haematologist	10	11	11	0.05	200,782	Gabon	Base Estimate	2212 - Specialist medical practitioners	High Income and Upper Middle Income	Central	Medical Doctors
21	Health Promoter/Health Educator	49	53	58	0.26	38,321	Gabon	Base Estimate	2269 - Health professionals not elsewhere classified	High Income and Upper Middle Income	Central	Other Health Workers
22	Infectious Diseases Specialist	4	4	4	0.02	562,390	Gabon	Base Estimate	2212 - Specialist medical practitioners	High Income and Upper Middle Income	Central	Medical Doctors
23	Intensive Care Nurse	73	79	84	0.38	26,590	Gabon	Base Estimate	2221 - Nursing professionals	High Income and Upper Middle Income	Central	Nursing Personnel
24	Medical Laboratory Scientist	413	463	517	2.32	4,314	Gabon	Base Estimate	3212 - Medical and pathology laboratory technicians	High Income and Upper Middle Income	Central	Other Health Workers
25	Medical Laboratory Technician	499	587	691	3.10	3,225	Gabon	Base Estimate	3212 - Medical and pathology laboratory technicians	High Income and Upper Middle Income	Central	Other Health Workers
26	Medical Social Worker	72	76	78	0.35	28,411	Gabon	Base Estimate	1344 - Social welfare managers	High Income and Upper Middle Income	Central	Other Health Workers
27	Mental Health Nurse	180	198	217	0.97	10,261	Gabon	Base Estimate	2221 - Nursing professionals	High Income and Upper Middle Income	Central	Nursing Personnel
28	Midwife	1,601	1,725	1,817	8.13	1,230	Gabon	Base Estimate	2222 - Midwifery professionals	High Income and Upper Middle Income	Central	Midwifery Personnel
29	Nephrologist	33	38	43	0.19	51,471	Gabon	Base Estimate	2212 - Specialist medical practitioners	High Income and Upper Middle Income	Central	Medical Doctors
30	Neuro-Surgeon	11	13	14	0.06	161,096	Gabon	Base Estimate	2212 - Specialist medical practitioners	High Income and Upper Middle Income	Central	Medical Doctors
31	Nurse Anaesthetist	78	91	104	0.47	21,427	Gabon	Base Estimate	2221 - Nursing professionals	High Income and Upper Middle Income	Central	Nursing Personnel
32	Nutritionist	171	178	183	0.82	12,214	Gabon	Base Estimate	2265 - Dieticians and nutritionists	High Income and Upper Middle Income	Central	Other Health Workers
33	Obstetrician & Gynaecologist	190	210	232	1.04	9,654	Gabon	Base Estimate	2212 - Specialist medical practitioners	High Income and Upper Middle Income	Central	Medical Doctors
34	Occupational Therapist	59	66	74	0.33	30,261	Gabon	Base Estimate	2269 - Health professionals not elsewhere classified	High Income and Upper Middle Income	Central	Other Health Workers
35	Oncology Nurse	28	32	36	0.16	61,141	Gabon	Base Estimate	2221 - Nursing professionals	High Income and Upper Middle Income	Central	Nursing Personnel
36	Operating Theatre Nurse	337	378	422	1.90	5,275	Gabon	Base Estimate	2221 - Nursing professionals	High Income and Upper Middle Income	Central	Nursing Personnel
37	Ophthalmic Nurse	55	62	69	0.31	32,370	Gabon	Base Estimate	2221 - Nursing professionals	High Income and Upper Middle Income	Central	Nursing Personnel
38	Ophthalmologist	12	14	16	0.07	140,625	Gabon	Base Estimate	2212 - Specialist medical practitioners	High Income and Upper Middle Income	Central	Medical Doctors
39	Optometrist	44	50	57	0.25	39,403	Gabon	Base Estimate	2267 - Optometrists and ophthalmic opticians	High Income and Upper Middle Income	Central	Other Health Workers
40	Orthopaedic Nurse	55	61	68	0.30	32,846	Gabon	Base Estimate	2221 - Nursing professionals	High Income and Upper Middle Income	Central	Nursing Personnel
41	Orthopaedic Surgeon	120	137	156	0.70	14,322	Gabon	Base Estimate	2212 - Specialist medical practitioners	High Income and Upper Middle Income	Central	Medical Doctors
42	Orthopaedic Technologist	71	78	84	0.38	26,639	Gabon	Base Estimate	3214 - Medical and dental prosthetic technicians	High Income and Upper Middle Income	Central	Other Health Workers
43	Paediatric Nurse	238	252	259	1.16	8,638	Gabon	Base Estimate	2221 - Nursing professionals	High Income and Upper Middle Income	Central	Nursing Personnel
44	Paediatric Surgeon	7	7	7	0.03	321,001	Gabon	Base Estimate	2212 - Specialist medical practitioners	High Income and Upper Middle Income	Central	Medical Doctors
45	Paediatrician	108	117	127	0.57	17,630	Gabon	Base Estimate	2212 - Specialist medical practitioners	High Income and Upper Middle Income	Central	Medical Doctors
46	Pathologist	30	34	38	0.17	58,896	Gabon	Base Estimate	2212 - Specialist medical practitioners	High Income and Upper Middle Income	Central	Medical Doctors
47	Pharmacist	372	388	402	1.80	5,550	Gabon	Base Estimate	2262 - Pharmacists	High Income and Upper Middle Income	Central	Pharmacist
48	Pharmacy Technician	327	352	377	1.69	5,934	Gabon	Base Estimate	3213 - Pharmaceutical technicians and assistants	High Income and Upper Middle Income	Central	Other Health Workers
49	Physician	286	318	353	1.58	6,328	Gabon	Base Estimate	2212 - Specialist medical practitioners	High Income and Upper Middle Income	Central	Medical Doctors
50	Physiotherapist	67	73	80	0.36	27,869	Gabon	Base Estimate	2264 - Physiotherapists	High Income and Upper Middle Income	Central	Other Health Workers
51	Plastic Surgeon	16	18	20	0.09	113,637	Gabon	Base Estimate	2212 - Specialist medical practitioners	High Income and Upper Middle Income	Central	Medical Doctors
52	Psychiatrist	146	160	174	0.78	12,782	Gabon	Base Estimate	2212 - Specialist medical practitioners	High Income and Upper Middle Income	Central	Medical Doctors
53	Radiation Oncologist	11	13	15	0.07	146,226	Gabon	Base Estimate	2212 - Specialist medical practitioners	High Income and Upper Middle Income	Central	Medical Doctors
54	Radiographer (Diagnostics and Therapy)	230	259	289	1.30	7,715	Gabon	Base Estimate	3211 - Medical imaging and therapeutic equipment technicians	High Income and Upper Middle Income	Central	Other Health Workers
55	Radiologist	84	96	109	0.49	20,555	Gabon	Base Estimate	2212 - Specialist medical practitioners	High Income and Upper Middle Income	Central	Medical Doctors
56	Registered General Nurse / State Certified Nurse	6,171	6,787	7,392	33.09	302	Gabon	Base Estimate	2221 - Nursing professionals	High Income and Upper Middle Income	Central	Nursing Personnel
57	Renal Nurse	447	511	580	2.60	3,841	Gabon	Base Estimate	2221 - Nursing professionals	High Income and Upper Middle Income	Central	Nursing Personnel
58	Respiratory Physician	17	19	21	0.09	107,606	Gabon	Base Estimate	2212 - Specialist medical practitioners	High Income and Upper Middle Income	Central	Medical Doctors
59	Rheumatologist	8	9	10	0.05	221,922	Gabon	Base Estimate	2212 - Specialist medical practitioners	High Income and Upper Middle Income	Central	Medical Doctors
60	Speech Therapist	24	26	28	0.12	80,439	Gabon	Base Estimate	2266 - Audiologists and speech therapists	High Income and Upper Middle Income	Central	Other Health Workers
61	Urologist	5	6	7	0.03	307,903	Gabon	Base Estimate	2212 - Specialist medical practitioners	High Income and Upper Middle Income	Central	Medical Doctors
1	Anaesthesiologist	25	28	31	0.13	78,201	Gambia	Base Estimate	2212 - Specialist medical practitioners	Low Income	West	Medical Doctors
2	Associate Nurse/Enrolled Nurse/Nursing Assistant	2,707	2,996	3,309	13.58	736	Gambia	Base Estimate	3221 - Nursing associate professionals	Low Income	West	Nursing Personnel
3	Audiologist	10	11	12	0.05	199,375	Gambia	Base Estimate	2266 - Audiologists and speech therapists	Low Income	West	Other Health Workers
4	Cardiologist	19	21	23	0.10	104,662	Gambia	Base Estimate	2212 - Specialist medical practitioners	Low Income	West	Medical Doctors
5	Cardiothoracic Surgeon	3	4	4	0.02	561,364	Gambia	Base Estimate	2212 - Specialist medical practitioners	Low Income	West	Medical Doctors
6	Clinical Officer/Physician Assistant	190	202	217	0.90	11,132	Gambia	Base Estimate	3256 - Medical assistants	Low Income	West	Other Health Workers
7	Clinical Pharmacist	68	77	86	0.35	28,236	Gambia	Base Estimate	2262 - Pharmacists	Low Income	West	Pharmacist
8	Clinical Psychologist	115	130	146	0.60	16,617	Gambia	Base Estimate	2634 - Psychologists	Low Income	West	Other Health Workers
9	Community health worker/Village health worker	1,883	2,026	2,169	8.90	1,123	Gambia	Base Estimate	3253 - Community health workers	Low Income	West	Other Health Workers
10	Dental Surgery Assistant	319	359	403	1.65	6,046	Gambia	Base Estimate	3251 - Dental assistants and therapists	Low Income	West	Other Health Workers
11	Dental Therapist	207	233	261	1.07	9,324	Gambia	Base Estimate	3251 - Dental assistants and therapists	Low Income	West	Other Health Workers
12	Dentist	197	220	248	1.02	9,789	Gambia	Base Estimate	2261 - Dentists	Low Income	West	Dentist
13	Dermatologist	9	11	12	0.05	207,114	Gambia	Base Estimate	2212 - Specialist medical practitioners	Low Income	West	Medical Doctors
14	Endocrinologist	8	10	12	0.05	207,770	Gambia	Base Estimate	2212 - Specialist medical practitioners	Low Income	West	Medical Doctors
15	ENT Surgeon	27	30	33	0.14	73,060	Gambia	Base Estimate	2212 - Specialist medical practitioners	Low Income	West	Medical Doctors
16	Environmental Health Officer	120	132	145	0.59	16,872	Gambia	Base Estimate	2263 - Environmental and occupational health and hygiene professionals	Low Income	West	Other Health Workers
17	Gastroenterologist	17	18	19	0.08	129,180	Gambia	Base Estimate	2212 - Specialist medical practitioners	Low Income	West	Medical Doctors

S/N	Health Professionals	2022	2026	2030	Density per 10,000 population	Required Population ratio (1 professional is to xxx population)	Name of Country	Modelling Scenario	ISCO-08 Match	Income Group Classification	Sub-Regional Grouping	SDG 3c Occupation
18	General Medical Practitioner (Generalist Doctor)	938	1,037	1,143	4.69	2,130	Gambia	Base Estimate	2211 - Generalist medical practitioners	Low Income	West	Medical Doctors
19	General Surgeon	22	25	29	0.12	83,491	Gambia	Base Estimate	2212 - Specialist medical practitioners	Low Income	West	Medical Doctors
20	Haematologist	12	14	15	0.06	158,467	Gambia	Base Estimate	2212 - Specialist medical practitioners	Low Income	West	Medical Doctors
21	Health Promoter/Health Educator	17	18	19	0.08	126,266	Gambia	Base Estimate	2269 - Health professionals not elsewhere classified	Low Income	West	Other Health Workers
22	Infectious Diseases Specialist	2	2	2	0.01	1,095,094	Gambia	Base Estimate	2212 - Specialist medical practitioners	Low Income	West	Medical Doctors
23	Intensive Care Nurse	62	68	74	0.31	32,745	Gambia	Base Estimate	2221 - Nursing professionals	Low Income	West	Nursing Personnel
24	Medical Laboratory Scientist	247	275	308	1.27	7,875	Gambia	Base Estimate	3212 - Medical and pathology laboratory technicians	Low Income	West	Other Health Workers
25	Medical Laboratory Technician	249	278	311	1.28	7,828	Gambia	Base Estimate	3212 - Medical and pathology laboratory technicians	Low Income	West	Other Health Workers
26	Medical Social Worker	87	91	96	0.39	25,434	Gambia	Base Estimate	1344 - Social welfare managers	Low Income	West	Other Health Workers
27	Mental Health Nurse	154	172	195	0.80	12,433	Gambia	Base Estimate	2221 - Nursing professionals	Low Income	West	Nursing Personnel
28	Midwife	1,927	2,132	2,352	9.65	1,036	Gambia	Base Estimate	2222 - Midwifery professionals	Low Income	West	Midwifery Personnel
29	Nephrologist	25	28	33	0.14	73,524	Gambia	Base Estimate	2212 - Specialist medical practitioners	Low Income	West	Medical Doctors
30	Neuro-Surgeon	6	7	7	0.03	325,747	Gambia	Base Estimate	2212 - Specialist medical practitioners	Low Income	West	Medical Doctors
31	Nurse Anaesthetist	45	53	61	0.25	39,971	Gambia	Base Estimate	2221 - Nursing professionals	Low Income	West	Nursing Personnel
32	Nutritionist	382	398	412	1.69	5,910	Gambia	Base Estimate	2265 - Dieticians and nutritionists	Low Income	West	Other Health Workers
33	Obstetrician & Gynaecologist	161	180	200	0.82	12,167	Gambia	Base Estimate	2212 - Specialist medical practitioners	Low Income	West	Medical Doctors
34	Occupational Therapist	46	51	58	0.24	41,842	Gambia	Base Estimate	2269 - Health professionals not elsewhere classified	Low Income	West	Other Health Workers
35	Oncology Nurse	17	20	23	0.10	104,819	Gambia	Base Estimate	2221 - Nursing professionals	Low Income	West	Nursing Personnel
36	Operating Theatre Nurse	247	277	315	1.30	7,709	Gambia	Base Estimate	2221 - Nursing professionals	Low Income	West	Nursing Personnel
37	Ophthalmic Nurse	31	35	39	0.16	62,943	Gambia	Base Estimate	2221 - Nursing professionals	Low Income	West	Nursing Personnel
38	Ophthalmologist	9	10	11	0.05	217,025	Gambia	Base Estimate	2212 - Specialist medical practitioners	Low Income	West	Medical Doctors
39	Optometrist	34	38	42	0.17	57,784	Gambia	Base Estimate	2267 - Optometrists and ophthalmic opticians	Low Income	West	Other Health Workers
40	Orthopaedic Nurse	41	47	53	0.22	46,084	Gambia	Base Estimate	2221 - Nursing professionals	Low Income	West	Nursing Personnel
41	Orthopaedic Surgeon	81	93	107	0.44	22,772	Gambia	Base Estimate	2212 - Specialist medical practitioners	Low Income	West	Medical Doctors
42	Orthopaedic Technologist	70	78	86	0.35	28,348	Gambia	Base Estimate	3214 - Medical and dental prosthetic technicians	Low Income	West	Other Health Workers
43	Paediatric Nurse	344	375	403	1.65	6,058	Gambia	Base Estimate	2221 - Nursing professionals	Low Income	West	Nursing Personnel
44	Paediatric Surgeon	8	9	9	0.04	260,198	Gambia	Base Estimate	2212 - Specialist medical practitioners	Low Income	West	Medical Doctors
45	Paediatrician	111	125	141	0.58	17,299	Gambia	Base Estimate	2212 - Specialist medical practitioners	Low Income	West	Medical Doctors
46	Pathologist	14	16	18	0.08	131,530	Gambia	Base Estimate	2212 - Specialist medical practitioners	Low Income	West	Medical Doctors
47	Pharmacist	145	151	157	0.65	15,394	Gambia	Base Estimate	2262 - Pharmacists	Low Income	West	Pharmacist
48	Pharmacy Technician	250	277	307	1.26	7,923	Gambia	Base Estimate	3213 - Pharmaceutical technicians and assistants	Low Income	West	Other Health Workers
49	Physician	276	309	347	1.43	7,015	Gambia	Base Estimate	2212 - Specialist medical practitioners	Low Income	West	Medical Doctors
50	Physiotherapist	63	70	77	0.32	31,586	Gambia	Base Estimate	2264 - Physiotherapists	Low Income	West	Other Health Workers
51	Plastic Surgeon	17	20	23	0.09	107,419	Gambia	Base Estimate	2212 - Specialist medical practitioners	Low Income	West	Medical Doctors
52	Psychiatrist	150	166	185	0.76	13,117	Gambia	Base Estimate	2212 - Specialist medical practitioners	Low Income	West	Medical Doctors
53	Radiation Oncologist	4	5	6	0.03	398,973	Gambia	Base Estimate	2212 - Specialist medical practitioners	Low Income	West	Medical Doctors
54	Radiographer (Diagnostics and Therapy)	191	217	248	1.02	9,804	Gambia	Base Estimate	3211 - Medical imaging and therapeutic equipment technicians	Low Income	West	Other Health Workers
55	Radiologist	66	76	87	0.36	28,070	Gambia	Base Estimate	2212 - Specialist medical practitioners	Low Income	West	Medical Doctors
56	Registered General Nurse / State Certified Nurse	5,440	5,984	6,595	27.13	369	Gambia	Base Estimate	2221 - Nursing professionals	Low Income	West	Nursing Personnel
57	Renal Nurse	323	370	432	1.78	5,611	Gambia	Base Estimate	2221 - Nursing professionals	Low Income	West	Nursing Personnel
58	Respiratory Physician	19	21	24	0.10	100,295	Gambia	Base Estimate	2212 - Specialist medical practitioners	Low Income	West	Medical Doctors
59	Rheumatologist	7	8	9	0.04	279,778	Gambia	Base Estimate	2212 - Specialist medical practitioners	Low Income	West	Medical Doctors
60	Speech Therapist	24	27	29	0.12	82,487	Gambia	Base Estimate	2266 - Audiologists and speech therapists	Low Income	West	Other Health Workers
61	Urologist	3	4	4	0.02	551,071	Gambia	Base Estimate	2212 - Specialist medical practitioners	Low Income	West	Medical Doctors
1	Anaesthesiologist	469	539	618	0.20	50,618	Ghana	Base Estimate	2212 - Specialist medical practitioners	Lower-middle Income	West	Medical Doctors
2	Associate Nurse/Enrolled Nurse/Nursing Assistant	39,937	43,273	46,956	15.02	666	Ghana	Base Estimate	3221 - Nursing associate professionals	Lower-middle Income	West	Nursing Personnel
3	Audiologist	123	135	147	0.05	213,010	Ghana	Base Estimate	2266 - Audiologists and speech therapists	Lower-middle Income	West	Other Health Workers
4	Cardiologist	274	308	344	0.11	90,905	Ghana	Base Estimate	2212 - Specialist medical practitioners	Lower-middle Income	West	Medical Doctors
5	Cardiothoracic Surgeon	83	96	111	0.04	282,200	Ghana	Base Estimate	2212 - Specialist medical practitioners	Lower-middle Income	West	Medical Doctors
6	Clinical Officer/Physician Assistant	5,298	5,463	5,789	1.88	5,322	Ghana	Base Estimate	3256 - Medical assistants	Lower-middle Income	West	Other Health Workers
7	Clinical Pharmacist	1,183	1,298	1,436	0.46	21,742	Ghana	Base Estimate	2262 - Pharmacists	Lower-middle Income	West	Pharmacist
8	Clinical Psychologist	2,519	2,747	3,011	0.96	10,383	Ghana	Base Estimate	2634 - Psychologists	Lower-middle Income	West	Other Health Workers
9	Community health worker/Village health worker	25,391	26,081	26,937	8.67	1,153	Ghana	Base Estimate	3253 - Community health workers	Lower-middle Income	West	Other Health Workers
10	Dental Surgery Assistant	3,929	4,369	4,841	1.55	6,465	Ghana	Base Estimate	3251 - Dental assistants and therapists	Lower-middle Income	West	Other Health Workers
11	Dental Therapist	2,551	2,837	3,144	1.00	9,955	Ghana	Base Estimate	3251 - Dental assistants and therapists	Lower-middle Income	West	Other Health Workers
12	Dentist	2,222	2,420	2,631	0.84	11,873	Ghana	Base Estimate	2261 - Dentists	Lower-middle Income	West	Dentist
13	Dermatologist	133	146	160	0.05	196,309	Ghana	Base Estimate	2212 - Specialist medical practitioners	Lower-middle Income	West	Medical Doctors
14	Endocrinologist	162	182	208	0.07	150,073	Ghana	Base Estimate	2212 - Specialist medical practitioners	Lower-middle Income	West	Medical Doctors
15	ENT Surgeon	387	430	485	0.16	64,205	Ghana	Base Estimate	2212 - Specialist medical practitioners	Lower-middle Income	West	Medical Doctors
16	Environmental Health Officer	1,543	1,664	1,787	0.57	17,530	Ghana	Base Estimate	2263 - Environmental and occupational health and hygiene professionals	Lower-middle Income	West	Other Health Workers
17	Gastroenterologist	504	497	498	0.16	62,263	Ghana	Base Estimate	2212 - Specialist medical practitioners	Lower-middle Income	West	Medical Doctors
18	General Medical Practitioner (Generalist Doctor)	13,794	14,814	15,956	5.11	1,956	Ghana	Base Estimate	2211 - Generalist medical practitioners	Lower-middle Income	West	Medical Doctors
19	General Surgeon	365	414	472	0.15	66,190	Ghana	Base Estimate	2212 - Specialist medical practitioners	Lower-middle Income	West	Medical Doctors
20	Haematologist	146	165	185	0.06	169,246	Ghana	Base Estimate	2212 - Specialist medical practitioners	Lower-middle Income	West	Medical Doctors
21	Health Promoter/Health Educator	406	405	413	0.13	74,579	Ghana	Base Estimate	2269 - Health professionals not elsewhere classified	Lower-middle Income	West	Other Health Workers
22	Infectious Diseases Specialist	44	47	49	0.02	632,584	Ghana	Base Estimate	2212 - Specialist medical practitioners	Lower-middle Income	West	Medical Doctors
23	Intensive Care Nurse	919	997	1,082	0.35	28,901	Ghana	Base Estimate	2221 - Nursing professionals	Lower-middle Income	West	Nursing Personnel
24	Medical Laboratory Scientist	4,296	4,612	5,013	1.61	6,215	Ghana	Base Estimate	3212 - Medical and pathology laboratory technicians	Lower-middle Income	West	Other Health Workers
25	Medical Laboratory Technician	4,196	4,258	4,398	1.42	7,031	Ghana	Base Estimate	3212 - Medical and pathology laboratory technicians	Lower-middle Income	West	Other Health Workers
26	Medical Social Worker	1,332	1,348	1,375	0.44	22,549	Ghana	Base Estimate	1344 - Social welfare managers	Lower-middle Income	West	Other Health Workers
27	Mental Health Nurse	1,985	2,178	2,421	0.77	12,908	Ghana	Base Estimate	2221 - Nursing professionals	Lower-middle Income	West	Nursing Personnel
28	Midwife	23,082	24,753	26,386	8.44	1,185	Ghana	Base Estimate	2222 - Midwifery professionals	Lower-middle Income	West	Midwifery Personnel
29	Nephrologist	423	483	558	0.18	55,919	Ghana	Base Estimate	2212 - Specialist medical practitioners	Lower-middle Income	West	Medical Doctors
30	Neuro-Surgeon	154	175	202	0.07	153,501	Ghana	Base Estimate	2212 - Specialist medical practitioners	Lower-middle Income	West	Medical Doctors
31	Nurse Anaesthetist	1,088	1,276	1,492	0.48	20,938	Ghana	Base Estimate	2221 - Nursing professionals	Lower-middle Income	West	Nursing Personnel
32	Nutritionist	3,717	3,793	3,858	1.24	8,078	Ghana	Base Estimate	2265 - Dieticians and nutritionists	Lower-middle Income	West	Other Health Workers
33	Obstetrician & Gynaecologist	2,348	2,588	2,858	0.91	10,936	Ghana	Base Estimate	2212 - Specialist medical practitioners	Lower-middle Income	West	Medical Doctors
34	Occupational Therapist	922	1,064	1,234	0.39	25,327	Ghana	Base Estimate	2269 - Health professionals not elsewhere classified	Lower-middle Income	West	Other Health Workers
35	Oncology Nurse	335	387	451	0.14	69,300	Ghana	Base Estimate	2221 - Nursing professionals	Lower-middle Income	West	Nursing Personnel
36	Operating Theatre Nurse	3,928	4,434	5,081	1.63	6,144	Ghana	Base Estimate	2221 - Nursing professionals	Lower-middle Income	West	Nursing Personnel
37	Ophthalmic Nurse	560	612	670	0.21	46,733	Ghana	Base Estimate	2221 - Nursing professionals	Lower-middle Income	West	Nursing Personnel
38	Ophthalmologist	138	153	171	0.05	181,909	Ghana	Base Estimate	2212 - Specialist medical practitioners	Lower-middle Income	West	Medical Doctors
39	Optometrist	487	533	583	0.19	53,720	Ghana	Base Estimate	2267 - Optometrists and ophthalmic opticians	Lower-middle Income	West	Other Health Workers
40	Orthopaedic Nurse	992	1,159	1,350	0.43	23,151	Ghana	Base Estimate	2221 - Nursing professionals	Lower-middle Income	West	Nursing Personnel

S/N	Health Professionals	2022	2026	2030	Density per 10,000 population	Required Population ratio (1 professional is to xxx population)	Name of Country	Modelling Scenario	ISCO-08 Match	Income Group Classification	Sub-Regional Grouping	SDG 3c Occupation
41	Orthopaedic Surgeon	1,422	1,634	1,878	0.60	16,654	Ghana	Base Estimate	2212 - Specialist medical practitioners	Lower-middle Income	West	Medical Doctors
42	Orthopaedic Technologist	1,163	1,299	1,449	0.46	21,599	Ghana	Base Estimate	3214 - Medical and dental prosthetic technicians	Lower-middle Income	West	Other Health Workers
43	Paediatric Nurse	3,635	3,925	4,194	1.34	7,464	Ghana	Base Estimate	2221 - Nursing professionals	Lower-middle Income	West	Nursing Personnel
44	Paediatric Surgeon	95	101	105	0.03	298,386	Ghana	Base Estimate	2212 - Specialist medical practitioners	Lower-middle Income	West	Medical Doctors
45	Paediatrician	1,421	1,575	1,750	0.56	17,879	Ghana	Base Estimate	2212 - Specialist medical practitioners	Lower-middle Income	West	Medical Doctors
46	Pathologist	283	316	358	0.11	87,197	Ghana	Base Estimate	2212 - Specialist medical practitioners	Lower-middle Income	West	Medical Doctors
47	Pharmacist	3,174	3,226	3,336	1.08	9,255	Ghana	Base Estimate	2262 - Pharmacists	Lower-middle Income	West	Pharmacist
48	Pharmacy Technician	4,011	4,330	4,697	1.51	6,642	Ghana	Base Estimate	3213 - Pharmaceutical technicians and assistants	Lower-middle Income	West	Other Health Workers
49	Physician	3,960	4,321	4,734	1.51	6,605	Ghana	Base Estimate	2212 - Specialist medical practitioners	Lower-middle Income	West	Medical Doctors
50	Physiotherapist	982	1,089	1,218	0.39	25,643	Ghana	Base Estimate	2264 - Physiotherapists	Lower-middle Income	West	Other Health Workers
51	Plastic Surgeon	266	304	347	0.11	90,249	Ghana	Base Estimate	2212 - Specialist medical practitioners	Lower-middle Income	West	Medical Doctors
52	Psychiatrist	1,889	2,052	2,253	0.72	13,864	Ghana	Base Estimate	2212 - Specialist medical practitioners	Lower-middle Income	West	Medical Doctors
53	Radiation Oncologist	110	130	155	0.05	201,217	Ghana	Base Estimate	2212 - Specialist medical practitioners	Lower-middle Income	West	Medical Doctors
54	Radiographer (Diagnostics and Therapy)	3,141	3,503	3,938	1.26	7,930	Ghana	Base Estimate	3211 - Medical imaging and therapeutic equipment technicians	Lower-middle Income	West	Other Health Workers
55	Radiologist	1,127	1,268	1,437	0.46	21,658	Ghana	Base Estimate	2212 - Specialist medical practitioners	Lower-middle Income	West	Medical Doctors
56	Registered General Nurse / State Certified Nurse	79,780	86,075	93,806	30.11	332	Ghana	Base Estimate	2221 - Nursing professionals	Lower-middle Income	West	Nursing Personnel
57	Renal Nurse	5,621	6,413	7,425	2.38	4,206	Ghana	Base Estimate	2221 - Nursing professionals	Lower-middle Income	West	Nursing Personnel
58	Respiratory Physician	206	231	260	0.08	120,506	Ghana	Base Estimate	2212 - Specialist medical practitioners	Lower-middle Income	West	Medical Doctors
59	Rheumatologist	104	116	128	0.04	244,368	Ghana	Base Estimate	2212 - Specialist medical practitioners	Lower-middle Income	West	Medical Doctors
60	Speech Therapist	343	373	414	0.13	74,973	Ghana	Base Estimate	2266 - Audiologists and speech therapists	Lower-middle Income	West	Other Health Workers
61	Urologist	61	72	88	0.03	352,158	Ghana	Base Estimate	2212 - Specialist medical practitioners	Lower-middle Income	West	Medical Doctors
1	Anaesthesiologist	146	162	179	0.13	74,145	Guinea	Base Estimate	2212 - Specialist medical practitioners	Low Income	West	Medical Doctors
2	Associate Nurse/Enrolled Nurse/Nursing Assistant	14,836	16,233	17,795	13.44	744	Guinea	Base Estimate	3221 - Nursing associate professionals	Low Income	West	Nursing Personnel
3	Audiologist	50	55	59	0.04	223,096	Guinea	Base Estimate	2266 - Audiologists and speech therapists	Low Income	West	Other Health Workers
4	Cardiologist	83	88	94	0.07	141,246	Guinea	Base Estimate	2212 - Specialist medical practitioners	Low Income	West	Medical Doctors
5	Cardiothoracic Surgeon	23	25	28	0.02	477,868	Guinea	Base Estimate	2212 - Specialist medical practitioners	Low Income	West	Medical Doctors
6	Clinical Officer/Physician Assistant	2,193	2,238	2,314	1.76	5,676	Guinea	Base Estimate	3256 - Medical assistants	Low Income	West	Other Health Workers
7	Clinical Pharmacist	427	466	516	0.39	25,644	Guinea	Base Estimate	2262 - Pharmacists	Low Income	West	Pharmacist
8	Clinical Psychologist	821	893	986	0.75	13,409	Guinea	Base Estimate	2634 - Psychologists	Low Income	West	Other Health Workers
9	Community health worker/Village health worker	14,760	15,682	16,615	12.56	796	Guinea	Base Estimate	3253 - Community health workers	Low Income	West	Other Health Workers
10	Dental Surgery Assistant	1,425	1,571	1,744	1.32	7,604	Guinea	Base Estimate	3251 - Dental assistants and therapists	Low Income	West	Other Health Workers
11	Dental Therapist	924	1,018	1,130	0.85	11,733	Guinea	Base Estimate	3251 - Dental assistants and therapists	Low Income	West	Other Health Workers
12	Dentist	975	1,073	1,211	0.91	10,945	Guinea	Base Estimate	2261 - Dentists	Low Income	West	Dentist
13	Dermatologist	48	53	58	0.04	227,510	Guinea	Base Estimate	2212 - Specialist medical practitioners	Low Income	West	Medical Doctors
14	Endocrinologist	40	46	56	0.04	235,963	Guinea	Base Estimate	2212 - Specialist medical practitioners	Low Income	West	Medical Doctors
15	ENT Surgeon	135	147	160	0.12	82,912	Guinea	Base Estimate	2212 - Specialist medical practitioners	Low Income	West	Medical Doctors
16	Environmental Health Officer	652	716	782	0.59	16,964	Guinea	Base Estimate	2263 - Environmental and occupational health and hygiene professionals	Low Income	West	Other Health Workers
17	Gastroenterologist	228	232	247	0.19	53,408	Guinea	Base Estimate	2212 - Specialist medical practitioners	Low Income	West	Medical Doctors
18	General Medical Practitioner (Generalist Doctor)	5,582	6,040	6,557	4.96	2,018	Guinea	Base Estimate	2211 - Generalist medical practitioners	Low Income	West	Medical Doctors
19	General Surgeon	118	131	148	0.11	89,403	Guinea	Base Estimate	2212 - Specialist medical practitioners	Low Income	West	Medical Doctors
20	Haematologist	58	65	72	0.05	184,102	Guinea	Base Estimate	2212 - Specialist medical practitioners	Low Income	West	Medical Doctors
21	Health Promoter/Health Educator	178	182	188	0.14	70,022	Guinea	Base Estimate	2269 - Health professionals not elsewhere classified	Low Income	West	Other Health Workers
22	Infectious Diseases Specialist	17	20	24	0.02	536,866	Guinea	Base Estimate	2212 - Specialist medical practitioners	Low Income	West	Medical Doctors
23	Intensive Care Nurse	385	421	459	0.35	28,857	Guinea	Base Estimate	2221 - Nursing professionals	Low Income	West	Nursing Personnel
24	Medical Laboratory Scientist	1,763	1,901	2,082	1.58	6,338	Guinea	Base Estimate	3212 - Medical and pathology laboratory technicians	Low Income	West	Other Health Workers
25	Medical Laboratory Technician	3,242	3,437	3,647	2.76	3,625	Guinea	Base Estimate	3212 - Medical and pathology laboratory technicians	Low Income	West	Other Health Workers
26	Medical Social Worker	653	685	730	0.55	18,107	Guinea	Base Estimate	1344 - Social welfare managers	Low Income	West	Other Health Workers
27	Mental Health Nurse	709	782	890	0.67	14,835	Guinea	Base Estimate	2221 - Nursing professionals	Low Income	West	Nursing Personnel
28	Midwife	11,731	12,848	14,146	10.67	938	Guinea	Base Estimate	2222 - Midwifery professionals	Low Income	West	Midwifery Personnel
29	Nephrologist	115	127	145	0.11	90,758	Guinea	Base Estimate	2212 - Specialist medical practitioners	Low Income	West	Medical Doctors
30	Neuro-Surgeon	38	42	47	0.04	283,367	Guinea	Base Estimate	2212 - Specialist medical practitioners	Low Income	West	Medical Doctors
31	Nurse Anaesthetist	288	318	351	0.26	37,779	Guinea	Base Estimate	2221 - Nursing professionals	Low Income	West	Nursing Personnel
32	Nutritionist	1,974	2,112	2,248	1.70	5,894	Guinea	Base Estimate	2265 - Dieticians and nutritionists	Low Income	West	Other Health Workers
33	Obstetrician & Gynaecologist	873	966	1,072	0.81	12,357	Guinea	Base Estimate	2212 - Specialist medical practitioners	Low Income	West	Medical Doctors
34	Occupational Therapist	273	302	335	0.25	39,476	Guinea	Base Estimate	2269 - Health professionals not elsewhere classified	Low Income	West	Other Health Workers
35	Oncology Nurse	89	98	111	0.08	119,042	Guinea	Base Estimate	2221 - Nursing professionals	Low Income	West	Nursing Personnel
36	Operating Theatre Nurse	1,277	1,400	1,579	1.19	8,370	Guinea	Base Estimate	2221 - Nursing professionals	Low Income	West	Nursing Personnel
37	Ophthalmic Nurse	160	176	195	0.15	67,964	Guinea	Base Estimate	2221 - Nursing professionals	Low Income	West	Nursing Personnel
38	Ophthalmologist	43	47	53	0.04	251,369	Guinea	Base Estimate	2212 - Specialist medical practitioners	Low Income	West	Medical Doctors
39	Optometrist	170	182	195	0.15	67,828	Guinea	Base Estimate	2267 - Optometrists and ophthalmic opticians	Low Income	West	Other Health Workers
40	Orthopaedic Nurse	271	302	336	0.25	39,505	Guinea	Base Estimate	2221 - Nursing professionals	Low Income	West	Nursing Personnel
41	Orthopaedic Surgeon	403	450	506	0.38	26,184	Guinea	Base Estimate	2212 - Specialist medical practitioners	Low Income	West	Medical Doctors
42	Orthopaedic Technologist	403	444	487	0.37	27,213	Guinea	Base Estimate	3214 - Medical and dental prosthetic technicians	Low Income	West	Other Health Workers
43	Paediatric Nurse	1,727	1,897	2,065	1.56	6,430	Guinea	Base Estimate	2221 - Nursing professionals	Low Income	West	Nursing Personnel
44	Paediatric Surgeon	53	58	62	0.05	214,834	Guinea	Base Estimate	2212 - Specialist medical practitioners	Low Income	West	Medical Doctors
45	Paediatrician	663	735	817	0.62	16,218	Guinea	Base Estimate	2212 - Specialist medical practitioners	Low Income	West	Medical Doctors
46	Pathologist	82	91	103	0.08	127,473	Guinea	Base Estimate	2212 - Specialist medical practitioners	Low Income	West	Medical Doctors
47	Pharmacist	1,046	1,045	1,055	0.81	12,418	Guinea	Base Estimate	2262 - Pharmacists	Low Income	West	Pharmacist
48	Pharmacy Technician	1,446	1,561	1,694	1.28	7,803	Guinea	Base Estimate	3213 - Pharmaceutical technicians and assistants	Low Income	West	Other Health Workers
49	Physician	1,626	1,782	1,964	1.48	6,737	Guinea	Base Estimate	2212 - Specialist medical practitioners	Low Income	West	Medical Doctors
50	Physiotherapist	348	383	420	0.32	31,517	Guinea	Base Estimate	2264 - Physiotherapists	Low Income	West	Other Health Workers
51	Plastic Surgeon	92	103	114	0.09	116,447	Guinea	Base Estimate	2212 - Specialist medical practitioners	Low Income	West	Medical Doctors
52	Psychiatrist	706	774	870	0.66	15,198	Guinea	Base Estimate	2212 - Specialist medical practitioners	Low Income	West	Medical Doctors
53	Radiation Oncologist	28	30	33	0.03	397,649	Guinea	Base Estimate	2212 - Specialist medical practitioners	Low Income	West	Medical Doctors
54	Radiographer (Diagnostics and Therapy)	1,023	1,128	1,269	0.96	10,414	Guinea	Base Estimate	3211 - Medical imaging and therapeutic equipment technicians	Low Income	West	Other Health Workers
55	Radiologist	379	420	471	0.36	28,041	Guinea	Base Estimate	2212 - Specialist medical practitioners	Low Income	West	Medical Doctors
56	Registered General Nurse / State Certified Nurse	29,616	31,900	34,754	26.30	380	Guinea	Base Estimate	2221 - Nursing professionals	Low Income	West	Nursing Personnel
57	Renal Nurse	1,504	1,654	1,898	1.44	6,953	Guinea	Base Estimate	2221 - Nursing professionals	Low Income	West	Nursing Personnel
58	Respiratory Physician	118	131	146	0.11	91,076	Guinea	Base Estimate	2212 - Specialist medical practitioners	Low Income	West	Medical Doctors
59	Rheumatologist	36	40	43	0.03	306,606	Guinea	Base Estimate	2212 - Specialist medical practitioners	Low Income	West	Medical Doctors
60	Speech Therapist	134	147	161	0.12	82,324	Guinea	Base Estimate	2266 - Audiologists and speech therapists	Low Income	West	Other Health Workers
61	Urologist	18	20	24	0.02	548,920	Guinea	Base Estimate	2212 - Specialist medical practitioners	Low Income	West	Medical Doctors
1	Anaesthesiologist	25	28	31	0.16	63,850	Guinea-Bissau	Base Estimate	2212 - Specialist medical practitioners	Low Income	West	Medical Doctors
2	Associate Nurse/Enrolled Nurse/Nursing Assistant	2,486	2,698	2,933	14.81	675	Guinea-Bissau	Base Estimate	3221 - Nursing associate professionals	Low Income	West	Nursing Personnel

S/N	Health Professionals	2022	2026	2030	Density per 10,000 population	Required Population ratio (1 professional is to xxx population)	Name of Country	Modelling Scenario	ISCO-08 Match	Income Group Classification	Sub-Regional Grouping	SDG 3c Occupation
3	Audiologist	8	8	9	0.05	215,013	Guinea-Bissau	Base Estimate	2266 - Audiologists and speech therapists	Low Income	West	Other Health Workers
4	Cardiologist	11	12	14	0.07	143,655	Guinea-Bissau	Base Estimate	2212 - Specialist medical practitioners	Low Income	West	Medical Doctors
5	Cardiothoracic Surgeon	4	5	5	0.03	385,522	Guinea-Bissau	Base Estimate	2212 - Specialist medical practitioners	Low Income	West	Medical Doctors
6	Clinical Officer/Physician Assistant	339	342	349	1.78	5,616	Guinea-Bissau	Base Estimate	3256 - Medical assistants	Low Income	West	Other Health Workers
7	Clinical Pharmacist	64	70	77	0.39	25,798	Guinea-Bissau	Base Estimate	2262 - Pharmacists	Low Income	West	Pharmacist
8	Clinical Psychologist	133	145	158	0.80	12,497	Guinea-Bissau	Base Estimate	2634 - Psychologists	Low Income	West	Other Health Workers
9	Community health worker/Village health worker	1,664	1,733	1,803	9.13	1,096	Guinea-Bissau	Base Estimate	3253 - Community health workers	Low Income	West	Other Health Workers
10	Dental Surgery Assistant	244	271	302	1.52	6,561	Guinea-Bissau	Base Estimate	3251 - Dental assistants and therapists	Low Income	West	Other Health Workers
11	Dental Therapist	158	175	196	0.99	10,135	Guinea-Bissau	Base Estimate	3251 - Dental assistants and therapists	Low Income	West	Other Health Workers
12	Dentist	177	195	218	1.10	9,069	Guinea-Bissau	Base Estimate	2261 - Dentists	Low Income	West	Dentist
13	Dermatologist	8	8	9	0.05	216,197	Guinea-Bissau	Base Estimate	2212 - Specialist medical practitioners	Low Income	West	Medical Doctors
14	Endocrinologist	7	8	10	0.05	195,363	Guinea-Bissau	Base Estimate	2212 - Specialist medical practitioners	Low Income	West	Medical Doctors
15	ENT Surgeon	21	23	25	0.13	78,858	Guinea-Bissau	Base Estimate	2212 - Specialist medical practitioners	Low Income	West	Medical Doctors
16	Environmental Health Officer	98	106	115	0.58	17,224	Guinea-Bissau	Base Estimate	2263 - Environmental and occupational health and hygiene professionals	Low Income	West	Other Health Workers
17	Gastroenterologist	30	30	31	0.16	62,581	Guinea-Bissau	Base Estimate	2212 - Specialist medical practitioners	Low Income	West	Medical Doctors
18	General Medical Practitioner (Generalist Doctor)	841	909	983	4.96	2,014	Guinea-Bissau	Base Estimate	2211 - Generalist medical practitioners	Low Income	West	Medical Doctors
19	General Surgeon	19	21	24	0.12	82,613	Guinea-Bissau	Base Estimate	2212 - Specialist medical practitioners	Low Income	West	Medical Doctors
20	Haematologist	8	9	10	0.05	203,617	Guinea-Bissau	Base Estimate	2212 - Specialist medical practitioners	Low Income	West	Medical Doctors
21	Health Promoter/Health Educator	31	31	32	0.16	62,259	Guinea-Bissau	Base Estimate	2269 - Health professionals not elsewhere classified	Low Income	West	Other Health Workers
22	Infectious Diseases Specialist	2	2	3	0.01	769,847	Guinea-Bissau	Base Estimate	2212 - Specialist medical practitioners	Low Income	West	Medical Doctors
23	Intensive Care Nurse	57	62	67	0.34	29,800	Guinea-Bissau	Base Estimate	2221 - Nursing professionals	Low Income	West	Nursing Personnel
24	Medical Laboratory Scientist	224	243	266	1.35	7,406	Guinea-Bissau	Base Estimate	3212 - Medical and pathology laboratory technicians	Low Income	West	Other Health Workers
25	Medical Laboratory Technician	200	211	223	1.13	8,826	Guinea-Bissau	Base Estimate	3212 - Medical and pathology laboratory technicians	Low Income	West	Other Health Workers
26	Medical Social Worker	87	89	91	0.46	21,614	Guinea-Bissau	Base Estimate	1344 - Social welfare managers	Low Income	West	Other Health Workers
27	Mental Health Nurse	111	122	137	0.70	14,356	Guinea-Bissau	Base Estimate	2221 - Nursing professionals	Low Income	West	Nursing Personnel
28	Midwife	1,538	1,656	1,788	9.03	1,108	Guinea-Bissau	Base Estimate	2222 - Midwifery professionals	Low Income	West	Midwifery Personnel
29	Nephrologist	20	23	26	0.13	74,469	Guinea-Bissau	Base Estimate	2212 - Specialist medical practitioners	Low Income	West	Medical Doctors
30	Neuro-Surgeon	7	8	9	0.04	224,892	Guinea-Bissau	Base Estimate	2212 - Specialist medical practitioners	Low Income	West	Medical Doctors
31	Nurse Anaesthetist	46	51	56	0.28	35,179	Guinea-Bissau	Base Estimate	2221 - Nursing professionals	Low Income	West	Nursing Personnel
32	Nutritionist	268	280	290	1.47	6,825	Guinea-Bissau	Base Estimate	2265 - Dieticians and nutritionists	Low Income	West	Other Health Workers
33	Obstetrician & Gynaecologist	137	150	166	0.84	11,963	Guinea-Bissau	Base Estimate	2212 - Specialist medical practitioners	Low Income	West	Medical Doctors
34	Occupational Therapist	49	54	61	0.31	32,657	Guinea-Bissau	Base Estimate	2269 - Health professionals not elsewhere classified	Low Income	West	Other Health Workers
35	Oncology Nurse	15	17	19	0.10	104,570	Guinea-Bissau	Base Estimate	2221 - Nursing professionals	Low Income	West	Nursing Personnel
36	Operating Theatre Nurse	211	235	266	1.35	7,417	Guinea-Bissau	Base Estimate	2221 - Nursing professionals	Low Income	West	Nursing Personnel
37	Ophthalmic Nurse	25	28	31	0.16	63,047	Guinea-Bissau	Base Estimate	2221 - Nursing professionals	Low Income	West	Nursing Personnel
38	Ophthalmologist	7	8	9	0.04	222,809	Guinea-Bissau	Base Estimate	2212 - Specialist medical practitioners	Low Income	West	Medical Doctors
39	Optometrist	27	30	33	0.17	59,324	Guinea-Bissau	Base Estimate	2267 - Optometrists and ophthalmic opticians	Low Income	West	Other Health Workers
40	Orthopaedic Nurse	50	56	63	0.32	31,460	Guinea-Bissau	Base Estimate	2221 - Nursing professionals	Low Income	West	Nursing Personnel
41	Orthopaedic Surgeon	70	80	91	0.46	21,732	Guinea-Bissau	Base Estimate	2212 - Specialist medical practitioners	Low Income	West	Medical Doctors
42	Orthopaedic Technologist	65	72	79	0.40	25,200	Guinea-Bissau	Base Estimate	3214 - Medical and dental prosthetic technicians	Low Income	West	Other Health Workers
43	Paediatric Nurse	247	267	284	1.43	6,985	Guinea-Bissau	Base Estimate	2221 - Nursing professionals	Low Income	West	Nursing Personnel
44	Paediatric Surgeon	7	8	8	0.04	247,016	Guinea-Bissau	Base Estimate	2212 - Specialist medical practitioners	Low Income	West	Medical Doctors
45	Paediatrician	86	95	105	0.53	18,868	Guinea-Bissau	Base Estimate	2212 - Specialist medical practitioners	Low Income	West	Medical Doctors
46	Pathologist	13	15	17	0.09	113,560	Guinea-Bissau	Base Estimate	2212 - Specialist medical practitioners	Low Income	West	Medical Doctors
47	Pharmacist	213	217	222	1.13	8,870	Guinea-Bissau	Base Estimate	2262 - Pharmacists	Low Income	West	Pharmacist
48	Pharmacy Technician	247	267	289	1.46	6,846	Guinea-Bissau	Base Estimate	3213 - Pharmaceutical technicians and assistants	Low Income	West	Other Health Workers
49	Physician	226	248	274	1.38	7,220	Guinea-Bissau	Base Estimate	2212 - Specialist medical practitioners	Low Income	West	Medical Doctors
50	Physiotherapist	56	61	67	0.34	29,589	Guinea-Bissau	Base Estimate	2264 - Physiotherapists	Low Income	West	Other Health Workers
51	Plastic Surgeon	14	16	17	0.09	113,985	Guinea-Bissau	Base Estimate	2212 - Specialist medical practitioners	Low Income	West	Medical Doctors
52	Psychiatrist	109	119	132	0.67	14,920	Guinea-Bissau	Base Estimate	2212 - Specialist medical practitioners	Low Income	West	Medical Doctors
53	Radiation Oncologist	4	5	5	0.03	376,715	Guinea-Bissau	Base Estimate	2212 - Specialist medical practitioners	Low Income	West	Medical Doctors
54	Radiographer (Diagnostics and Therapy)	165	183	206	1.04	9,600	Guinea-Bissau	Base Estimate	3211 - Medical imaging and therapeutic equipment technicians	Low Income	West	Other Health Workers
55	Radiologist	59	65	73	0.37	27,276	Guinea-Bissau	Base Estimate	2212 - Specialist medical practitioners	Low Income	West	Medical Doctors
56	Registered General Nurse / State Certified Nurse	7,220	7,407	7,663	39.00	256	Guinea-Bissau	Base Estimate	2221 - Nursing professionals	Low Income	West	Nursing Personnel
57	Renal Nurse	265	300	347	1.77	5,665	Guinea-Bissau	Base Estimate	2221 - Nursing professionals	Low Income	West	Nursing Personnel
58	Respiratory Physician	16	18	20	0.10	100,655	Guinea-Bissau	Base Estimate	2212 - Specialist medical practitioners	Low Income	West	Medical Doctors
59	Rheumatologist	6	6	7	0.03	292,163	Guinea-Bissau	Base Estimate	2212 - Specialist medical practitioners	Low Income	West	Medical Doctors
60	Speech Therapist	20	22	24	0.12	83,617	Guinea-Bissau	Base Estimate	2266 - Audiologists and speech therapists	Low Income	West	Other Health Workers
61	Urologist	2	3	3	0.02	663,461	Guinea-Bissau	Base Estimate	2212 - Specialist medical practitioners	Low Income	West	Medical Doctors
1	Anaesthesiologist	530	610	709	0.13	76,108	Kenya	Base Estimate	2212 - Specialist medical practitioners	Lower-middle Income	East	Medical Doctors
2	Associate Nurse/Enrolled Nurse/Nursing Assistant	64,441	69,738	76,165	14.09	709	Kenya	Base Estimate	3221 - Nursing associate professionals	Lower-middle Income	East	Nursing Personnel
3	Audiologist	235	259	284	0.05	190,587	Kenya	Base Estimate	2266 - Audiologists and speech therapists	Lower-middle Income	East	Other Health Workers
4	Cardiologist	461	526	600	0.11	90,301	Kenya	Base Estimate	2212 - Specialist medical practitioners	Lower-middle Income	East	Medical Doctors
5	Cardiothoracic Surgeon	80	90	101	0.02	533,331	Kenya	Base Estimate	2212 - Specialist medical practitioners	Lower-middle Income	East	Medical Doctors
6	Clinical Officer/Physician Assistant	11,515	12,638	14,134	2.63	3,807	Kenya	Base Estimate	3256 - Medical assistants	Lower-middle Income	East	Other Health Workers
7	Clinical Pharmacist	1,571	1,733	1,954	0.36	27,585	Kenya	Base Estimate	2262 - Pharmacists	Lower-middle Income	East	Pharmacist
8	Clinical Psychologist	3,831	4,145	4,539	0.84	11,916	Kenya	Base Estimate	2634 - Psychologists	Lower-middle Income	East	Other Health Workers
9	Community health worker/Village health worker	44,263	49,244	55,242	10.25	976	Kenya	Base Estimate	3253 - Community health workers	Lower-middle Income	East	Other Health Workers
10	Dental Surgery Assistant	5,453	6,076	6,827	1.26	7,950	Kenya	Base Estimate	3251 - Dental assistants and therapists	Lower-middle Income	East	Other Health Workers
11	Dental Therapist	3,537	3,942	4,429	0.82	12,254	Kenya	Base Estimate	3251 - Dental assistants and therapists	Lower-middle Income	East	Other Health Workers
12	Dentist	3,707	4,035	4,492	0.83	12,110	Kenya	Base Estimate	2261 - Dentists	Lower-middle Income	East	Dentist
13	Dermatologist	202	224	247	0.05	219,659	Kenya	Base Estimate	2212 - Specialist medical practitioners	Lower-middle Income	East	Medical Doctors
14	Endocrinologist	188	223	277	0.05	193,160	Kenya	Base Estimate	2212 - Specialist medical practitioners	Lower-middle Income	East	Medical Doctors
15	ENT Surgeon	711	799	917	0.17	58,859	Kenya	Base Estimate	2212 - Specialist medical practitioners	Lower-middle Income	East	Medical Doctors
16	Environmental Health Officer	2,671	2,888	3,116	0.57	17,396	Kenya	Base Estimate	2263 - Environmental and occupational health and hygiene professionals	Lower-middle Income	East	Other Health Workers
17	Gastroenterologist	343	357	391	0.07	137,265	Kenya	Base Estimate	2212 - Specialist medical practitioners	Lower-middle Income	East	Medical Doctors
18	General Medical Practitioner (Generalist Doctor)	24,223	26,610	29,446	5.44	1,837	Kenya	Base Estimate	2211 - Generalist medical practitioners	Lower-middle Income	East	Medical Doctors
19	General Surgeon	466	533	624	0.12	86,579	Kenya	Base Estimate	2212 - Specialist medical practitioners	Lower-middle Income	East	Medical Doctors
20	Haematologist	151	162	174	0.03	311,669	Kenya	Base Estimate	2212 - Specialist medical practitioners	Lower-middle Income	East	Medical Doctors
21	Health Promoter/Health Educator	936	1,018	1,123	0.21	47,911	Kenya	Base Estimate	2269 - Health professionals not elsewhere classified	Lower-middle Income	East	Other Health Workers
22	Infectious Diseases Specialist	76	83	93	0.02	578,275	Kenya	Base Estimate	2212 - Specialist medical practitioners	Lower-middle Income	East	Medical Doctors
23	Intensive Care Nurse	1,966	2,119	2,285	0.42	23,695	Kenya	Base Estimate	2221 - Nursing professionals	Lower-middle Income	East	Nursing Personnel
24	Medical Laboratory Scientist	7,026	7,836	8,937	1.66	6,020	Kenya	Base Estimate	3212 - Medical and pathology laboratory technicians	Lower-middle Income	East	Other Health Workers
25	Medical Laboratory Technician	8,012	9,214	10,780	2.01	4,975	Kenya	Base Estimate	3212 - Medical and pathology laboratory technicians	Lower-middle Income	East	Other Health Workers

S/N	Health Professionals	2022	2026	2030	Density per 10,000 population	Required Population ratio (1 professional is to xxx population)	Name of Country	Modelling Scenario	ISCO-08 Match	Income Group Classification	Sub-Regional Grouping	SDG 3c Occupation
26	Medical Social Worker	1,731	1,848	2,012	0.38	26,656	Kenya	Base Estimate	1344 - Social welfare managers	Lower-middle Income	East	Other Health Workers
27	Mental Health Nurse	3,637	4,017	4,623	0.86	11,668	Kenya	Base Estimate	2221 - Nursing professionals	Lower-middle Income	East	Nursing Personnel
28	Midwife	39,893	42,873	46,511	8.56	1,168	Kenya	Base Estimate	2222 - Midwifery professionals	Lower-middle Income	East	Midwifery Personnel
29	Nephrologist	579	665	801	0.15	67,037	Kenya	Base Estimate	2212 - Specialist medical practitioners	Lower-middle Income	East	Medical Doctors
30	Neuro-Surgeon	152	177	215	0.04	249,090	Kenya	Base Estimate	2212 - Specialist medical practitioners	Lower-middle Income	East	Medical Doctors
31	Nurse Anaesthetist	1,202	1,405	1,653	0.31	32,672	Kenya	Base Estimate	2221 - Nursing professionals	Lower-middle Income	East	Nursing Personnel
32	Nutritionist	6,789	7,057	7,325	1.35	7,386	Kenya	Base Estimate	2265 - Dieticians and nutritionists	Lower-middle Income	East	Other Health Workers
33	Obstetrician & Gynaecologist	4,074	4,499	5,035	0.93	10,718	Kenya	Base Estimate	2212 - Specialist medical practitioners	Lower-middle Income	East	Medical Doctors
34	Occupational Therapist	955	1,104	1,308	0.24	41,145	Kenya	Base Estimate	2269 - Health professionals not elsewhere classified	Lower-middle Income	East	Other Health Workers
35	Oncology Nurse	444	514	616	0.11	87,262	Kenya	Base Estimate	2221 - Nursing professionals	Lower-middle Income	East	Nursing Personnel
36	Operating Theatre Nurse	6,399	7,254	8,591	1.60	6,257	Kenya	Base Estimate	2221 - Nursing professionals	Lower-middle Income	East	Nursing Personnel
37	Ophthalmic Nurse	583	651	741	0.14	72,825	Kenya	Base Estimate	2221 - Nursing professionals	Lower-middle Income	East	Nursing Personnel
38	Ophthalmologist	209	238	280	0.05	192,437	Kenya	Base Estimate	2212 - Specialist medical practitioners	Lower-middle Income	East	Medical Doctors
39	Optometrist	690	787	904	0.17	59,785	Kenya	Base Estimate	2267 - Optometrists and ophthalmic opticians	Lower-middle Income	East	Other Health Workers
40	Orthopaedic Nurse	731	845	978	0.18	55,292	Kenya	Base Estimate	2221 - Nursing professionals	Lower-middle Income	East	Nursing Personnel
41	Orthopaedic Surgeon	2,066	2,391	2,802	0.52	19,268	Kenya	Base Estimate	2212 - Specialist medical practitioners	Lower-middle Income	East	Medical Doctors
42	Orthopaedic Technologist	1,511	1,657	1,818	0.34	29,809	Kenya	Base Estimate	3214 - Medical and dental prosthetic technicians	Lower-middle Income	East	Other Health Workers
43	Paediatric Nurse	5,439	5,787	6,140	1.13	8,832	Kenya	Base Estimate	2221 - Nursing professionals	Lower-middle Income	East	Nursing Personnel
44	Paediatric Surgeon	163	170	176	0.03	306,928	Kenya	Base Estimate	2212 - Specialist medical practitioners	Lower-middle Income	East	Medical Doctors
45	Paediatrician	2,379	2,601	2,885	0.53	18,729	Kenya	Base Estimate	2212 - Specialist medical practitioners	Lower-middle Income	East	Medical Doctors
46	Pathologist	549	618	725	0.13	74,296	Kenya	Base Estimate	2212 - Specialist medical practitioners	Lower-middle Income	East	Medical Doctors
47	Pharmacist	7,965	8,541	9,209	1.70	5,868	Kenya	Base Estimate	2262 - Pharmacists	Lower-middle Income	East	Pharmacist
48	Pharmacy Technician	6,557	7,156	7,857	1.45	6,884	Kenya	Base Estimate	3213 - Pharmaceutical technicians and assistants	Lower-middle Income	East	Other Health Workers
49	Physician	5,468	6,085	6,862	1.27	7,871	Kenya	Base Estimate	2212 - Specialist medical practitioners	Lower-middle Income	East	Medical Doctors
50	Physiotherapist	1,412	1,554	1,736	0.32	31,114	Kenya	Base Estimate	2264 - Physiotherapists	Lower-middle Income	East	Other Health Workers
51	Plastic Surgeon	544	635	740	0.14	73,147	Kenya	Base Estimate	2212 - Specialist medical practitioners	Lower-middle Income	East	Medical Doctors
52	Psychiatrist	3,296	3,599	4,067	0.75	13,281	Kenya	Base Estimate	2212 - Specialist medical practitioners	Lower-middle Income	East	Medical Doctors
53	Radiation Oncologist	141	167	201	0.04	268,233	Kenya	Base Estimate	2212 - Specialist medical practitioners	Lower-middle Income	East	Medical Doctors
54	Radiographer (Diagnostics and Therapy)	4,091	4,653	5,440	1.01	9,902	Kenya	Base Estimate	3211 - Medical imaging and therapeutic equipment technicians	Lower-middle Income	East	Other Health Workers
55	Radiologist	1,454	1,687	2,004	0.37	26,727	Kenya	Base Estimate	2212 - Specialist medical practitioners	Lower-middle Income	East	Medical Doctors
56	Registered General Nurse / State Certified Nurse	125,392	138,727	156,648	29.06	344	Kenya	Base Estimate	2221 - Nursing professionals	Lower-middle Income	East	Nursing Personnel
57	Renal Nurse	7,662	8,814	10,629	1.98	5,053	Kenya	Base Estimate	2221 - Nursing professionals	Lower-middle Income	East	Nursing Personnel
58	Respiratory Physician	424	467	517	0.10	104,736	Kenya	Base Estimate	2212 - Specialist medical practitioners	Lower-middle Income	East	Medical Doctors
59	Rheumatologist	172	191	214	0.04	253,018	Kenya	Base Estimate	2212 - Specialist medical practitioners	Lower-middle Income	East	Medical Doctors
60	Speech Therapist	550	597	661	0.12	81,566	Kenya	Base Estimate	2266 - Audiologists and speech therapists	Lower-middle Income	East	Other Health Workers
61	Urologist	86	105	134	0.03	395,311	Kenya	Base Estimate	2212 - Specialist medical practitioners	Lower-middle Income	East	Medical Doctors
1	Anaesthesiologist	31	35	38	0.18	56,618	Lesotho	Base Estimate	2212 - Specialist medical practitioners	Lower-middle Income	Southern	Medical Doctors
2	Associate Nurse/Enrolled Nurse/Nursing Assistant	3,362	3,566	3,792	17.60	568	Lesotho	Base Estimate	3221 - Nursing associate professionals	Lower-middle Income	Southern	Nursing Personnel
3	Audiologist	9	10	10	0.05	206,978	Lesotho	Base Estimate	2266 - Audiologists and speech therapists	Lower-middle Income	Southern	Other Health Workers
4	Cardiologist	23	24	25	0.12	84,884	Lesotho	Base Estimate	2212 - Specialist medical practitioners	Lower-middle Income	Southern	Medical Doctors
5	Cardiothoracic Surgeon	4	5	5	0.02	400,736	Lesotho	Base Estimate	2212 - Specialist medical practitioners	Lower-middle Income	Southern	Medical Doctors
6	Clinical Officer/Physician Assistant	430	455	487	2.26	4,424	Lesotho	Base Estimate	3256 - Medical assistants	Lower-middle Income	Southern	Other Health Workers
7	Clinical Pharmacist	82	88	96	0.45	22,224	Lesotho	Base Estimate	2262 - Pharmacists	Lower-middle Income	Southern	Pharmacist
8	Clinical Psychologist	150	157	166	0.77	12,974	Lesotho	Base Estimate	2634 - Psychologists	Lower-middle Income	Southern	Other Health Workers
9	Community health worker/Village health worker	1,637	1,692	1,741	8.10	1,235	Lesotho	Base Estimate	3253 - Community health workers	Lower-middle Income	Southern	Other Health Workers
10	Dental Surgery Assistant	284	305	325	1.51	6,642	Lesotho	Base Estimate	3251 - Dental assistants and therapists	Lower-middle Income	Southern	Other Health Workers
11	Dental Therapist	184	197	210	0.97	10,265	Lesotho	Base Estimate	3251 - Dental assistants and therapists	Lower-middle Income	Southern	Other Health Workers
12	Dentist	218	230	241	1.12	8,962	Lesotho	Base Estimate	2261 - Dentists	Lower-middle Income	Southern	Dentist
13	Dermatologist	9	10	10	0.05	215,281	Lesotho	Base Estimate	2212 - Specialist medical practitioners	Lower-middle Income	Southern	Medical Doctors
14	Endocrinologist	16	18	21	0.10	103,464	Lesotho	Base Estimate	2212 - Specialist medical practitioners	Lower-middle Income	Southern	Medical Doctors
15	ENT Surgeon	32	34	37	0.17	58,488	Lesotho	Base Estimate	2212 - Specialist medical practitioners	Lower-middle Income	Southern	Medical Doctors
16	Environmental Health Officer	106	111	115	0.54	18,668	Lesotho	Base Estimate	2263 - Environmental and occupational health and hygiene professionals	Lower-middle Income	Southern	Other Health Workers
17	Gastroenterologist	28	27	27	0.13	78,102	Lesotho	Base Estimate	2212 - Specialist medical practitioners	Lower-middle Income	Southern	Medical Doctors
18	General Medical Practitioner (Generalist Doctor)	1,084	1,148	1,219	5.67	1,765	Lesotho	Base Estimate	2211 - Generalist medical practitioners	Lower-middle Income	Southern	Medical Doctors
19	General Surgeon	37	40	43	0.20	50,265	Lesotho	Base Estimate	2212 - Specialist medical practitioners	Lower-middle Income	Southern	Medical Doctors
20	Haematologist	6	6	7	0.03	327,156	Lesotho	Base Estimate	2212 - Specialist medical practitioners	Lower-middle Income	Southern	Medical Doctors
21	Health Promoter/Health Educator	31	33	36	0.17	59,000	Lesotho	Base Estimate	2269 - Health professionals not elsewhere classified	Lower-middle Income	Southern	Other Health Workers
22	Infectious Diseases Specialist	4	4	4	0.02	494,784	Lesotho	Base Estimate	2212 - Specialist medical practitioners	Lower-middle Income	Southern	Medical Doctors
23	Intensive Care Nurse	66	70	73	0.34	29,367	Lesotho	Base Estimate	2221 - Nursing professionals	Lower-middle Income	Southern	Nursing Personnel
24	Medical Laboratory Scientist	503	545	598	2.79	3,583	Lesotho	Base Estimate	3212 - Medical and pathology laboratory technicians	Lower-middle Income	Southern	Other Health Workers
25	Medical Laboratory Technician	393	424	462	2.15	4,644	Lesotho	Base Estimate	3212 - Medical and pathology laboratory technicians	Lower-middle Income	Southern	Other Health Workers
26	Medical Social Worker	79	83	92	0.45	22,160	Lesotho	Base Estimate	1344 - Social welfare managers	Lower-middle Income	Southern	Other Health Workers
27	Mental Health Nurse	178	189	204	0.95	10,573	Lesotho	Base Estimate	2221 - Nursing professionals	Lower-middle Income	Southern	Nursing Personnel
28	Midwife	1,609	1,672	1,724	8.00	1,251	Lesotho	Base Estimate	2222 - Midwifery professionals	Lower-middle Income	Southern	Midwifery Personnel
29	Nephrologist	38	42	48	0.22	44,924	Lesotho	Base Estimate	2212 - Specialist medical practitioners	Lower-middle Income	Southern	Medical Doctors
30	Neuro-Surgeon	12	13	15	0.07	142,783	Lesotho	Base Estimate	2212 - Specialist medical practitioners	Lower-middle Income	Southern	Medical Doctors
31	Nurse Anaesthetist	87	98	111	0.51	19,419	Lesotho	Base Estimate	2221 - Nursing professionals	Lower-middle Income	Southern	Nursing Personnel
32	Nutritionist	195	192	189	0.88	11,331	Lesotho	Base Estimate	2265 - Dieticians and nutritionists	Lower-middle Income	Southern	Other Health Workers
33	Obstetrician & Gynaecologist	199	217	242	1.14	8,761	Lesotho	Base Estimate	2212 - Specialist medical practitioners	Lower-middle Income	Southern	Medical Doctors
34	Occupational Therapist	53	59	67	0.31	32,215	Lesotho	Base Estimate	2269 - Health professionals not elsewhere classified	Lower-middle Income	Southern	Other Health Workers
35	Oncology Nurse	30	34	38	0.18	56,177	Lesotho	Base Estimate	2221 - Nursing professionals	Lower-middle Income	Southern	Nursing Personnel
36	Operating Theatre Nurse	339	368	407	1.89	5,302	Lesotho	Base Estimate	2221 - Nursing professionals	Lower-middle Income	Southern	Nursing Personnel
37	Ophthalmic Nurse	43	46	50	0.23	43,325	Lesotho	Base Estimate	2221 - Nursing professionals	Lower-middle Income	Southern	Nursing Personnel
38	Ophthalmologist	15	17	18	0.08	118,299	Lesotho	Base Estimate	2212 - Specialist medical practitioners	Lower-middle Income	Southern	Medical Doctors
39	Optometrist	77	83	89	0.41	24,270	Lesotho	Base Estimate	2267 - Optometrists and ophthalmic opticians	Lower-middle Income	Southern	Other Health Workers
40	Orthopaedic Nurse	47	55	63	0.29	34,355	Lesotho	Base Estimate	2221 - Nursing professionals	Lower-middle Income	Southern	Nursing Personnel
41	Orthopaedic Surgeon	100	110	122	0.57	17,652	Lesotho	Base Estimate	2212 - Specialist medical practitioners	Lower-middle Income	Southern	Medical Doctors
42	Orthopaedic Technologist	68	73	79	0.37	27,274	Lesotho	Base Estimate	3214 - Medical and dental prosthetic technicians	Lower-middle Income	Southern	Other Health Workers
43	Paediatric Nurse	188	193	194	0.90	11,075	Lesotho	Base Estimate	2221 - Nursing professionals	Lower-middle Income	Southern	Nursing Personnel
44	Paediatric Surgeon	6	6	6	0.03	353,963	Lesotho	Base Estimate	2212 - Specialist medical practitioners	Lower-middle Income	Southern	Medical Doctors
45	Paediatrician	77	83	90	0.42	24,009	Lesotho	Base Estimate	2212 - Specialist medical practitioners	Lower-middle Income	Southern	Medical Doctors
46	Pathologist	31	34	38	0.17	57,272	Lesotho	Base Estimate	2212 - Specialist medical practitioners	Lower-middle Income	Southern	Medical Doctors
47	Pharmacist	219	220	225	1.06	9,449	Lesotho	Base Estimate	2262 - Pharmacists	Lower-middle Income	Southern	Pharmacist
48	Pharmacy Technician	259	274	292	1.36	7,351	Lesotho	Base Estimate	3213 - Pharmaceutical technicians and assistants	Lower-middle Income	Southern	Other Health Workers

S/N	Health Professionals	2022	2026	2030	Density per 10,000 population	Required Population ratio (1 professional is to xxx population)	Name of Country	Modelling Scenario	ISCO-08 Match	Income Group Classification	Sub-Regional Grouping	SDG 3c Occupation
49	Physician	268	286	308	1.43	7,003	Lesotho	Base Estimate	2212 - Specialist medical practitioners	Lower-middle Income	Southern	Medical Doctors
50	Physiotherapist	65	69	75	0.35	28,828	Lesotho	Base Estimate	2264 - Physiotherapists	Lower-middle Income	Southern	Other Health Workers
51	Plastic Surgeon	26	28	31	0.14	70,751	Lesotho	Base Estimate	2212 - Specialist medical practitioners	Lower-middle Income	Southern	Medical Doctors
52	Psychiatrist	151	159	170	0.79	12,679	Lesotho	Base Estimate	2212 - Specialist medical practitioners	Lower-middle Income	Southern	Medical Doctors
53	Radiation Oncologist	11	12	14	0.07	150,988	Lesotho	Base Estimate	2212 - Specialist medical practitioners	Lower-middle Income	Southern	Medical Doctors
54	Radiographer (Diagnostics and Therapy)	239	262	294	1.38	7,262	Lesotho	Base Estimate	3211 - Medical imaging and therapeutic equipment technicians	Lower-middle Income	Southern	Other Health Workers
55	Radiologist	88	102	123	0.59	16,829	Lesotho	Base Estimate	2212 - Specialist medical practitioners	Lower-middle Income	Southern	Medical Doctors
56	Registered General Nurse / State Certified Nurse	5,809	6,258	6,795	31.51	317	Lesotho	Base Estimate	2221 - Nursing professionals	Lower-middle Income	Southern	Nursing Personnel
57	Renal Nurse	500	558	637	2.95	3,395	Lesotho	Base Estimate	2221 - Nursing professionals	Lower-middle Income	Southern	Nursing Personnel
58	Respiratory Physician	12	13	14	0.06	154,174	Lesotho	Base Estimate	2212 - Specialist medical practitioners	Lower-middle Income	Southern	Medical Doctors
59	Rheumatologist	8	8	9	0.04	244,951	Lesotho	Base Estimate	2212 - Specialist medical practitioners	Lower-middle Income	Southern	Medical Doctors
60	Speech Therapist	25	26	28	0.13	78,108	Lesotho	Base Estimate	2266 - Audiologists and speech therapists	Lower-middle Income	Southern	Other Health Workers
61	Urologist	5	6	7	0.04	280,799	Lesotho	Base Estimate	2212 - Specialist medical practitioners	Lower-middle Income	Southern	Medical Doctors
1	Anaesthesiologist	53	62	71	0.14	71,593	Liberia	Base Estimate	2212 - Specialist medical practitioners	Low Income	West	Medical Doctors
2	Associate Nurse/Enrolled Nurse/Nursing Assistant	6,530	7,260	8,121	15.98	626	Liberia	Base Estimate	3221 - Nursing associate professionals	Low Income	West	Nursing Personnel
3	Audiologist	21	23	25	0.05	202,884	Liberia	Base Estimate	2266 - Audiologists and speech therapists	Low Income	West	Other Health Workers
4	Cardiologist	39	45	51	0.10	100,283	Liberia	Base Estimate	2212 - Specialist medical practitioners	Low Income	West	Medical Doctors
5	Cardiothoracic Surgeon	7	8	10	0.02	526,025	Liberia	Base Estimate	2212 - Specialist medical practitioners	Low Income	West	Medical Doctors
6	Clinical Officer/Physician Assistant	1,954	2,263	2,664	5.29	1,890	Liberia	Base Estimate	3256 - Medical assistants	Low Income	West	Other Health Workers
7	Clinical Pharmacist	161	177	197	0.39	25,817	Liberia	Base Estimate	2262 - Pharmacists	Low Income	West	Pharmacist
8	Clinical Psychologist	397	431	473	0.93	10,765	Liberia	Base Estimate	2634 - Psychologists	Low Income	West	Other Health Workers
9	Community health worker/Village health worker	7,155	8,502	10,194	20.22	494	Liberia	Base Estimate	3253 - Community health workers	Low Income	West	Other Health Workers
10	Dental Surgery Assistant	631	709	801	1.57	6,365	Liberia	Base Estimate	3251 - Dental assistants and therapists	Low Income	West	Other Health Workers
11	Dental Therapist	409	459	519	1.02	9,825	Liberia	Base Estimate	3251 - Dental assistants and therapists	Low Income	West	Other Health Workers
12	Dentist	431	477	538	1.05	9,488	Liberia	Base Estimate	2261 - Dentists	Low Income	West	Dentist
13	Dermatologist	20	22	25	0.05	207,576	Liberia	Base Estimate	2212 - Specialist medical practitioners	Low Income	West	Medical Doctors
14	Endocrinologist	24	27	33	0.07	152,770	Liberia	Base Estimate	2212 - Specialist medical practitioners	Low Income	West	Medical Doctors
15	ENT Surgeon	58	65	74	0.15	68,920	Liberia	Base Estimate	2212 - Specialist medical practitioners	Low Income	West	Medical Doctors
16	Environmental Health Officer	251	274	297	0.58	17,156	Liberia	Base Estimate	2263 - Environmental and occupational health and hygiene professionals	Low Income	West	Other Health Workers
17	Gastroenterologist	76	76	79	0.16	64,267	Liberia	Base Estimate	2212 - Specialist medical practitioners	Low Income	West	Medical Doctors
18	General Medical Practitioner (Generalist Doctor)	2,554	2,848	3,205	6.32	1,582	Liberia	Base Estimate	2211 - Generalist medical practitioners	Low Income	West	Medical Doctors
19	General Surgeon	48	54	63	0.12	80,553	Liberia	Base Estimate	2212 - Specialist medical practitioners	Low Income	West	Medical Doctors
20	Haematologist	12	13	14	0.03	373,805	Liberia	Base Estimate	2212 - Specialist medical practitioners	Low Income	West	Medical Doctors
21	Health Promoter/Health Educator	132	147	167	0.33	30,210	Liberia	Base Estimate	2269 - Health professionals not elsewhere classified	Low Income	West	Other Health Workers
22	Infectious Diseases Specialist	8	8	9	0.02	573,016	Liberia	Base Estimate	2212 - Specialist medical practitioners	Low Income	West	Medical Doctors
23	Intensive Care Nurse	136	147	159	0.31	31,956	Liberia	Base Estimate	2221 - Nursing professionals	Low Income	West	Nursing Personnel
24	Medical Laboratory Scientist	796	915	1,072	2.12	4,715	Liberia	Base Estimate	3212 - Medical and pathology laboratory technicians	Low Income	West	Other Health Workers
25	Medical Laboratory Technician	1,861	2,246	2,733	5.42	1,843	Liberia	Base Estimate	3212 - Medical and pathology laboratory technicians	Low Income	West	Other Health Workers
26	Medical Social Worker	195	199	207	0.41	24,398	Liberia	Base Estimate	1344 - Social welfare managers	Low Income	West	Other Health Workers
27	Mental Health Nurse	310	342	388	0.77	13,068	Liberia	Base Estimate	2221 - Nursing professionals	Low Income	West	Nursing Personnel
28	Midwife	4,557	4,897	5,284	10.36	965	Liberia	Base Estimate	2222 - Midwifery professionals	Low Income	West	Midwifery Personnel
29	Nephrologist	56	64	75	0.15	67,542	Liberia	Base Estimate	2212 - Specialist medical practitioners	Low Income	West	Medical Doctors
30	Neuro-Surgeon	20	22	26	0.05	197,087	Liberia	Base Estimate	2212 - Specialist medical practitioners	Low Income	West	Medical Doctors
31	Nurse Anaesthetist	103	119	138	0.27	37,013	Liberia	Base Estimate	2221 - Nursing professionals	Low Income	West	Nursing Personnel
32	Nutritionist	464	466	468	0.92	10,839	Liberia	Base Estimate	2265 - Dieticians and nutritionists	Low Income	West	Other Health Workers
33	Obstetrician & Gynaecologist	366	407	454	0.89	11,195	Liberia	Base Estimate	2212 - Specialist medical practitioners	Low Income	West	Medical Doctors
34	Occupational Therapist	103	119	139	0.27	36,545	Liberia	Base Estimate	2269 - Health professionals not elsewhere classified	Low Income	West	Other Health Workers
35	Oncology Nurse	40	46	53	0.11	95,193	Liberia	Base Estimate	2221 - Nursing professionals	Low Income	West	Nursing Personnel
36	Operating Theatre Nurse	595	677	790	1.56	6,417	Liberia	Base Estimate	2221 - Nursing professionals	Low Income	West	Nursing Personnel
37	Ophthalmic Nurse	68	77	87	0.17	58,221	Liberia	Base Estimate	2221 - Nursing professionals	Low Income	West	Nursing Personnel
38	Ophthalmologist	21	24	28	0.06	180,572	Liberia	Base Estimate	2212 - Specialist medical practitioners	Low Income	West	Medical Doctors
39	Optometrist	83	95	109	0.21	46,766	Liberia	Base Estimate	2267 - Optometrists and ophthalmic opticians	Low Income	West	Other Health Workers
40	Orthopaedic Nurse	90	105	122	0.24	41,645	Liberia	Base Estimate	2221 - Nursing professionals	Low Income	West	Nursing Personnel
41	Orthopaedic Surgeon	169	204	247	0.49	20,484	Liberia	Base Estimate	2212 - Specialist medical practitioners	Low Income	West	Medical Doctors
42	Orthopaedic Technologist	148	164	182	0.36	27,928	Liberia	Base Estimate	3214 - Medical and dental prosthetic technicians	Low Income	West	Other Health Workers
43	Paediatric Nurse	474	500	526	1.03	9,697	Liberia	Base Estimate	2221 - Nursing professionals	Low Income	West	Nursing Personnel
44	Paediatric Surgeon	15	16	16	0.03	314,896	Liberia	Base Estimate	2212 - Specialist medical practitioners	Low Income	West	Medical Doctors
45	Paediatrician	193	213	237	0.47	21,468	Liberia	Base Estimate	2212 - Specialist medical practitioners	Low Income	West	Medical Doctors
46	Pathologist	37	42	50	0.10	102,260	Liberia	Base Estimate	2212 - Specialist medical practitioners	Low Income	West	Medical Doctors
47	Pharmacist	824	858	905	1.79	5,581	Liberia	Base Estimate	2262 - Pharmacists	Low Income	West	Pharmacist
48	Pharmacy Technician	702	754	816	1.61	6,222	Liberia	Base Estimate	3213 - Pharmaceutical technicians and assistants	Low Income	West	Other Health Workers
49	Physician	683	765	865	1.70	5,869	Liberia	Base Estimate	2212 - Specialist medical practitioners	Low Income	West	Medical Doctors
50	Physiotherapist	136	151	169	0.33	30,080	Liberia	Base Estimate	2264 - Physiotherapists	Low Income	West	Other Health Workers
51	Plastic Surgeon	37	43	50	0.10	101,862	Liberia	Base Estimate	2212 - Specialist medical practitioners	Low Income	West	Medical Doctors
52	Psychiatrist	275	299	333	0.66	15,241	Liberia	Base Estimate	2212 - Specialist medical practitioners	Low Income	West	Medical Doctors
53	Radiation Oncologist	10	12	14	0.03	365,830	Liberia	Base Estimate	2212 - Specialist medical practitioners	Low Income	West	Medical Doctors
54	Radiographer (Diagnostics and Therapy)	445	502	578	1.14	8,784	Liberia	Base Estimate	3211 - Medical imaging and therapeutic equipment technicians	Low Income	West	Other Health Workers
55	Radiologist	157	179	208	0.41	24,371	Liberia	Base Estimate	2212 - Specialist medical practitioners	Low Income	West	Medical Doctors
56	Registered General Nurse / State Certified Nurse	12,560	13,693	15,177	29.94	334	Liberia	Base Estimate	2221 - Nursing professionals	Low Income	West	Nursing Personnel
57	Renal Nurse	741	845	996	1.97	5,088	Liberia	Base Estimate	2221 - Nursing professionals	Low Income	West	Nursing Personnel
58	Respiratory Physician	34	38	42	0.08	121,384	Liberia	Base Estimate	2212 - Specialist medical practitioners	Low Income	West	Medical Doctors
59	Rheumatologist	15	17	19	0.04	269,961	Liberia	Base Estimate	2212 - Specialist medical practitioners	Low Income	West	Medical Doctors
60	Speech Therapist	52	56	62	0.12	82,094	Liberia	Base Estimate	2266 - Audiologists and speech therapists	Low Income	West	Other Health Workers
61	Urologist	9	10	13	0.03	388,413	Liberia	Base Estimate	2212 - Specialist medical practitioners	Low Income	West	Medical Doctors
1	Anaesthesiologist	277	314	355	0.13	78,548	Madagascar	Base Estimate	2212 - Specialist medical practitioners	Low Income	Southern	Medical Doctors
2	Associate Nurse/Enrolled Nurse/Nursing Assistant	30,648	33,252	36,194	12.98	770	Madagascar	Base Estimate	3221 - Nursing associate professionals	Low Income	Southern	Nursing Personnel
3	Audiologist	118	132	147	0.05	190,191	Madagascar	Base Estimate	2266 - Audiologists and speech therapists	Low Income	Southern	Other Health Workers
4	Cardiologist	221	254	290	0.10	96,290	Madagascar	Base Estimate	2212 - Specialist medical practitioners	Low Income	Southern	Medical Doctors
5	Cardiothoracic Surgeon	48	55	61	0.02	455,803	Madagascar	Base Estimate	2212 - Specialist medical practitioners	Low Income	Southern	Medical Doctors
6	Clinical Officer/Physician Assistant	3,458	3,755	4,124	1.48	6,751	Madagascar	Base Estimate	3256 - Medical assistants	Low Income	Southern	Other Health Workers
7	Clinical Pharmacist	849	946	1,064	0.38	26,151	Madagascar	Base Estimate	2262 - Pharmacists	Low Income	Southern	Pharmacist
8	Clinical Psychologist	2,028	2,266	2,542	0.91	10,978	Madagascar	Base Estimate	2634 - Psychologists	Low Income	Southern	Other Health Workers
9	Community health worker/Village health worker	22,328	24,059	25,844	9.25	1,081	Madagascar	Base Estimate	3253 - Community health workers	Low Income	Southern	Other Health Workers
10	Dental Surgery Assistant	3,238	3,635	4,081	1.46	6,851	Madagascar	Base Estimate	3251 - Dental assistants and therapists	Low Income	Southern	Other Health Workers

S/N	Health Professionals	2022	2026	2030	Density per 10,000 population	Required Population ratio (1 professional is to xxx population)	Name of Country	Modelling Scenario	ISCO-08 Match	Income Group Classification	Sub-Regional Grouping	SDG 3c Occupation
11	Dental Therapist	2,093	2,349	2,637	0.94	10,603	Madagascar	Base Estimate	3251 - Dental assistants and therapists	Low Income	Southern	Other Health Workers
12	Dentist	2,741	3,062	3,439	1.23	8,132	Madagascar	Base Estimate	2261 - Dentists	Low Income	Southern	Dentist
13	Dermatologist	99	111	124	0.04	225,066	Madagascar	Base Estimate	2212 - Specialist medical practitioners	Low Income	Southern	Medical Doctors
14	Endocrinologist	90	108	131	0.05	211,274	Madagascar	Base Estimate	2212 - Specialist medical practitioners	Low Income	Southern	Medical Doctors
15	ENT Surgeon	324	368	421	0.15	66,111	Madagascar	Base Estimate	2212 - Specialist medical practitioners	Low Income	Southern	Medical Doctors
16	Environmental Health Officer	1,375	1,511	1,652	0.59	16,919	Madagascar	Base Estimate	2263 - Environmental and occupational health and hygiene professionals	Low Income	Southern	Other Health Workers
17	Gastroenterologist	207	213	226	0.08	122,413	Madagascar	Base Estimate	2212 - Specialist medical practitioners	Low Income	Southern	Medical Doctors
18	General Medical Practitioner (Generalist Doctor)	10,597	11,692	12,915	4.63	2,161	Madagascar	Base Estimate	2211 - Generalist medical practitioners	Low Income	Southern	Medical Doctors
19	General Surgeon	223	259	303	0.11	92,019	Madagascar	Base Estimate	2212 - Specialist medical practitioners	Low Income	Southern	Medical Doctors
20	Haematologist	111	124	137	0.05	203,274	Madagascar	Base Estimate	2212 - Specialist medical practitioners	Low Income	Southern	Medical Doctors
21	Health Promoter/Health Educator	273	296	325	0.12	85,568	Madagascar	Base Estimate	2269 - Health professionals not elsewhere classified	Low Income	Southern	Other Health Workers
22	Infectious Diseases Specialist	30	33	37	0.01	750,145	Madagascar	Base Estimate	2212 - Specialist medical practitioners	Low Income	Southern	Medical Doctors
23	Intensive Care Nurse	900	988	1,085	0.39	25,712	Madagascar	Base Estimate	2221 - Nursing professionals	Low Income	Southern	Nursing Personnel
24	Medical Laboratory Scientist	3,036	3,418	3,891	1.40	7,148	Madagascar	Base Estimate	3212 - Medical and pathology laboratory technicians	Low Income	Southern	Other Health Workers
25	Medical Laboratory Technician	3,150	3,480	3,868	1.39	7,196	Madagascar	Base Estimate	3212 - Medical and pathology laboratory technicians	Low Income	Southern	Other Health Workers
26	Medical Social Worker	1,027	1,073	1,136	0.41	24,274	Madagascar	Base Estimate	1344 - Social welfare managers	Low Income	Southern	Other Health Workers
27	Mental Health Nurse	1,951	2,188	2,506	0.90	11,115	Madagascar	Base Estimate	2221 - Nursing professionals	Low Income	Southern	Nursing Personnel
28	Midwife	20,487	22,327	24,316	8.70	1,150	Madagascar	Base Estimate	2222 - Midwifery professionals	Low Income	Southern	Midwifery Personnel
29	Nephrologist	255	293	345	0.12	80,696	Madagascar	Base Estimate	2212 - Specialist medical practitioners	Low Income	Southern	Medical Doctors
30	Neuro-Surgeon	84	96	112	0.04	246,225	Madagascar	Base Estimate	2212 - Specialist medical practitioners	Low Income	Southern	Medical Doctors
31	Nurse Anaesthetist	579	665	762	0.27	36,620	Madagascar	Base Estimate	2221 - Nursing professionals	Low Income	Southern	Nursing Personnel
32	Nutritionist	4,246	4,321	4,383	1.58	6,344	Madagascar	Base Estimate	2265 - Dieticians and nutritionists	Low Income	Southern	Other Health Workers
33	Obstetrician & Gynaecologist	2,025	2,269	2,558	0.92	10,876	Madagascar	Base Estimate	2212 - Specialist medical practitioners	Low Income	Southern	Medical Doctors
34	Occupational Therapist	511	580	664	0.24	41,999	Madagascar	Base Estimate	2269 - Health professionals not elsewhere classified	Low Income	Southern	Other Health Workers
35	Oncology Nurse	199	228	267	0.10	104,203	Madagascar	Base Estimate	2221 - Nursing professionals	Low Income	Southern	Nursing Personnel
36	Operating Theatre Nurse	2,950	3,339	3,868	1.39	7,196	Madagascar	Base Estimate	2221 - Nursing professionals	Low Income	Southern	Nursing Personnel
37	Ophthalmic Nurse	340	386	440	0.16	63,388	Madagascar	Base Estimate	2221 - Nursing professionals	Low Income	Southern	Nursing Personnel
38	Ophthalmologist	100	115	134	0.05	208,626	Madagascar	Base Estimate	2212 - Specialist medical practitioners	Low Income	Southern	Medical Doctors
39	Optometrist	432	497	572	0.20	48,801	Madagascar	Base Estimate	2267 - Optometrists and ophthalmic opticians	Low Income	Southern	Other Health Workers
40	Orthopaedic Nurse	469	534	607	0.22	45,996	Madagascar	Base Estimate	2221 - Nursing professionals	Low Income	Southern	Nursing Personnel
41	Orthopaedic Surgeon	897	1,035	1,199	0.43	23,263	Madagascar	Base Estimate	2212 - Specialist medical practitioners	Low Income	Southern	Medical Doctors
42	Orthopaedic Technologist	830	921	1,020	0.37	27,396	Madagascar	Base Estimate	3214 - Medical and dental prosthetic technicians	Low Income	Southern	Other Health Workers
43	Paediatric Nurse	3,618	3,884	4,140	1.48	6,753	Madagascar	Base Estimate	2221 - Nursing professionals	Low Income	Southern	Nursing Personnel
44	Paediatric Surgeon	93	101	108	0.04	260,036	Madagascar	Base Estimate	2212 - Specialist medical practitioners	Low Income	Southern	Medical Doctors
45	Paediatrician	1,794	1,937	2,095	0.75	13,323	Madagascar	Base Estimate	2212 - Specialist medical practitioners	Low Income	Southern	Medical Doctors
46	Pathologist	244	277	322	0.12	86,618	Madagascar	Base Estimate	2212 - Specialist medical practitioners	Low Income	Southern	Medical Doctors
47	Pharmacist	2,376	2,515	2,672	0.96	10,407	Madagascar	Base Estimate	2262 - Pharmacists	Low Income	Southern	Pharmacist
48	Pharmacy Technician	2,770	3,063	3,393	1.22	8,220	Madagascar	Base Estimate	3213 - Pharmaceutical technicians and assistants	Low Income	Southern	Other Health Workers
49	Physician	2,791	3,091	3,443	1.23	8,098	Madagascar	Base Estimate	2212 - Specialist medical practitioners	Low Income	Southern	Medical Doctors
50	Physiotherapist	749	832	931	0.33	29,979	Madagascar	Base Estimate	2264 - Physiotherapists	Low Income	Southern	Other Health Workers
51	Plastic Surgeon	470	543	626	0.22	44,615	Madagascar	Base Estimate	2212 - Specialist medical practitioners	Low Income	Southern	Medical Doctors
52	Psychiatrist	1,708	1,879	2,102	0.75	13,251	Madagascar	Base Estimate	2212 - Specialist medical practitioners	Low Income	Southern	Medical Doctors
53	Radiation Oncologist	62	72	84	0.03	329,858	Madagascar	Base Estimate	2212 - Specialist medical practitioners	Low Income	Southern	Medical Doctors
54	Radiographer (Diagnostics and Therapy)	1,922	2,193	2,539	0.91	10,947	Madagascar	Base Estimate	3211 - Medical imaging and therapeutic equipment technicians	Low Income	Southern	Other Health Workers
55	Radiologist	690	803	949	0.34	29,005	Madagascar	Base Estimate	2212 - Specialist medical practitioners	Low Income	Southern	Medical Doctors
56	Registered General Nurse / State Certified Nurse	56,295	62,660	70,227	25.19	397	Madagascar	Base Estimate	2221 - Nursing professionals	Low Income	Southern	Nursing Personnel
57	Renal Nurse	3,380	3,887	4,582	1.65	6,073	Madagascar	Base Estimate	2221 - Nursing professionals	Low Income	Southern	Nursing Personnel
58	Respiratory Physician	421	449	478	0.17	58,341	Madagascar	Base Estimate	2212 - Specialist medical practitioners	Low Income	Southern	Medical Doctors
59	Rheumatologist	82	93	104	0.04	267,691	Madagascar	Base Estimate	2212 - Specialist medical practitioners	Low Income	Southern	Medical Doctors
60	Speech Therapist	301	332	371	0.13	75,125	Madagascar	Base Estimate	2266 - Audiologists and speech therapists	Low Income	Southern	Other Health Workers
61	Urologist	33	41	52	0.02	527,694	Madagascar	Base Estimate	2212 - Specialist medical practitioners	Low Income	Southern	Medical Doctors
1	Anaesthesiologist	164	186	211	0.11	91,551	Malawi	Base Estimate	2212 - Specialist medical practitioners	Low Income	Southern	Medical Doctors
2	Associate Nurse/Enrolled Nurse/Nursing Assistant	23,151	25,545	28,370	14.73	679	Malawi	Base Estimate	3221 - Nursing associate professionals	Low Income	Southern	Nursing Personnel
3	Audiologist	80	90	101	0.05	191,301	Malawi	Base Estimate	2266 - Audiologists and speech therapists	Low Income	Southern	Other Health Workers
4	Cardiologist	137	155	176	0.09	109,561	Malawi	Base Estimate	2212 - Specialist medical practitioners	Low Income	Southern	Medical Doctors
5	Cardiothoracic Surgeon	28	31	34	0.02	562,801	Malawi	Base Estimate	2212 - Specialist medical practitioners	Low Income	Southern	Medical Doctors
6	Clinical Officer/Physician Assistant	2,273	2,368	2,535	1.33	7,534	Malawi	Base Estimate	3256 - Medical assistants	Low Income	Southern	Other Health Workers
7	Clinical Pharmacist	609	682	781	0.41	24,563	Malawi	Base Estimate	2262 - Pharmacists	Low Income	Southern	Pharmacist
8	Clinical Psychologist	1,743	1,944	2,189	1.14	8,796	Malawi	Base Estimate	2634 - Psychologists	Low Income	Southern	Other Health Workers
9	Community health worker/Village health worker	15,062	16,098	17,336	9.01	1,110	Malawi	Base Estimate	3253 - Community health workers	Low Income	Southern	Other Health Workers
10	Dental Surgery Assistant	1,947	2,201	2,529	1.31	7,638	Malawi	Base Estimate	3251 - Dental assistants and therapists	Low Income	Southern	Other Health Workers
11	Dental Therapist	1,259	1,423	1,635	0.85	11,817	Malawi	Base Estimate	3251 - Dental assistants and therapists	Low Income	Southern	Other Health Workers
12	Dentist	1,627	1,842	2,155	1.11	8,969	Malawi	Base Estimate	2261 - Dentists	Low Income	Southern	Dentist
13	Dermatologist	68	77	87	0.05	221,837	Malawi	Base Estimate	2212 - Specialist medical practitioners	Low Income	Southern	Medical Doctors
14	Endocrinologist	64	74	89	0.05	214,077	Malawi	Base Estimate	2212 - Specialist medical practitioners	Low Income	Southern	Medical Doctors
15	ENT Surgeon	209	238	271	0.14	71,081	Malawi	Base Estimate	2212 - Specialist medical practitioners	Low Income	Southern	Medical Doctors
16	Environmental Health Officer	950	1,051	1,159	0.60	16,656	Malawi	Base Estimate	2263 - Environmental and occupational health and hygiene professionals	Low Income	Southern	Other Health Workers
17	Gastroenterologist	212	215	228	0.12	83,627	Malawi	Base Estimate	2212 - Specialist medical practitioners	Low Income	Southern	Medical Doctors
18	General Medical Practitioner (Generalist Doctor)	7,220	8,022	8,986	4.67	2,143	Malawi	Base Estimate	2211 - Generalist medical practitioners	Low Income	Southern	Medical Doctors
19	General Surgeon	162	189	226	0.12	85,062	Malawi	Base Estimate	2212 - Specialist medical practitioners	Low Income	Southern	Medical Doctors
20	Haematologist	85	97	110	0.06	175,821	Malawi	Base Estimate	2212 - Specialist medical practitioners	Low Income	Southern	Medical Doctors
21	Health Promoter/Health Educator	177	188	206	0.11	92,780	Malawi	Base Estimate	2269 - Health professionals not elsewhere classified	Low Income	Southern	Other Health Workers
22	Infectious Diseases Specialist	21	23	24	0.01	789,106	Malawi	Base Estimate	2212 - Specialist medical practitioners	Low Income	Southern	Medical Doctors
23	Intensive Care Nurse	581	646	717	0.37	26,907	Malawi	Base Estimate	2221 - Nursing professionals	Low Income	Southern	Nursing Personnel
24	Medical Laboratory Scientist	2,581	2,842	3,202	1.67	5,978	Malawi	Base Estimate	3212 - Medical and pathology laboratory technicians	Low Income	Southern	Other Health Workers
25	Medical Laboratory Technician	2,545	2,677	2,885	1.51	6,609	Malawi	Base Estimate	3212 - Medical and pathology laboratory technicians	Low Income	Southern	Other Health Workers
26	Medical Social Worker	665	712	786	0.41	24,148	Malawi	Base Estimate	1344 - Social welfare managers	Low Income	Southern	Other Health Workers
27	Mental Health Nurse	1,159	1,325	1,578	0.82	12,126	Malawi	Base Estimate	2221 - Nursing professionals	Low Income	Southern	Nursing Personnel
28	Midwife	14,016	15,410	17,174	8.88	1,126	Malawi	Base Estimate	2222 - Midwifery professionals	Low Income	Southern	Midwifery Personnel
29	Nephrologist	242	279	335	0.18	56,887	Malawi	Base Estimate	2212 - Specialist medical practitioners	Low Income	Southern	Medical Doctors
30	Neuro-Surgeon	48	54	61	0.03	315,181	Malawi	Base Estimate	2212 - Specialist medical practitioners	Low Income	Southern	Medical Doctors
31	Nurse Anaesthetist	409	467	533	0.28	36,146	Malawi	Base Estimate	2221 - Nursing professionals	Low Income	Southern	Nursing Personnel
32	Nutritionist	2,133	2,295	2,466	1.28	7,825	Malawi	Base Estimate	2265 - Dieticians and nutritionists	Low Income	Southern	Other Health Workers
33	Obstetrician & Gynaecologist	1,433	1,611	1,833	0.96	10,453	Malawi	Base Estimate	2212 - Specialist medical practitioners	Low Income	Southern	Medical Doctors

S/N	Health Professionals	2022	2026	2030	Density per 10,000 population	Required Population ratio (1 professional is to xxx population)	Name of Country	Modelling Scenario	ISCO-08 Match	Income Group Classification	Sub-Regional Grouping	SDG 3c Occupation
34	Occupational Therapist	281	318	367	0.19	52,376	Malawi	Base Estimate	2269 - Health professionals not elsewhere classified	Low Income	Southern	Other Health Workers
35	Oncology Nurse	181	208	247	0.13	77,468	Malawi	Base Estimate	2221 - Nursing professionals	Low Income	Southern	Nursing Personnel
36	Operating Theatre Nurse	1,812	2,065	2,434	1.27	7,860	Malawi	Base Estimate	2221 - Nursing professionals	Low Income	Southern	Nursing Personnel
37	Ophthalmic Nurse	254	285	323	0.17	59,660	Malawi	Base Estimate	2221 - Nursing professionals	Low Income	Southern	Nursing Personnel
38	Ophthalmologist	67	76	88	0.05	218,445	Malawi	Base Estimate	2212 - Specialist medical practitioners	Low Income	Southern	Medical Doctors
39	Optometrist	277	313	356	0.18	54,174	Malawi	Base Estimate	2267 - Optometrists and ophthalmic opticians	Low Income	Southern	Other Health Workers
40	Orthopaedic Nurse	232	263	298	0.15	64,776	Malawi	Base Estimate	2221 - Nursing professionals	Low Income	Southern	Nursing Personnel
41	Orthopaedic Surgeon	574	665	776	0.40	24,760	Malawi	Base Estimate	2212 - Specialist medical practitioners	Low Income	Southern	Medical Doctors
42	Orthopaedic Technologist	521	579	643	0.33	30,030	Malawi	Base Estimate	3214 - Medical and dental prosthetic technicians	Low Income	Southern	Other Health Workers
43	Paediatric Nurse	2,347	2,607	2,890	1.49	6,692	Malawi	Base Estimate	2221 - Nursing professionals	Low Income	Southern	Nursing Personnel
44	Paediatric Surgeon	66	71	75	0.04	257,347	Malawi	Base Estimate	2212 - Specialist medical practitioners	Low Income	Southern	Medical Doctors
45	Paediatrician	1,045	1,188	1,363	0.71	14,119	Malawi	Base Estimate	2212 - Specialist medical practitioners	Low Income	Southern	Medical Doctors
46	Pathologist	166	185	214	0.11	89,418	Malawi	Base Estimate	2212 - Specialist medical practitioners	Low Income	Southern	Medical Doctors
47	Pharmacist	1,096	1,168	1,261	0.66	15,188	Malawi	Base Estimate	2262 - Pharmacists	Low Income	Southern	Pharmacist
48	Pharmacy Technician	1,709	1,915	2,162	1.12	8,904	Malawi	Base Estimate	3213 - Pharmaceutical technicians and assistants	Low Income	Southern	Other Health Workers
49	Physician	1,888	2,122	2,415	1.26	7,960	Malawi	Base Estimate	2212 - Specialist medical practitioners	Low Income	Southern	Medical Doctors
50	Physiotherapist	474	528	591	0.31	32,613	Malawi	Base Estimate	2264 - Physiotherapists	Low Income	Southern	Other Health Workers
51	Plastic Surgeon	277	325	380	0.20	50,639	Malawi	Base Estimate	2212 - Specialist medical practitioners	Low Income	Southern	Medical Doctors
52	Psychiatrist	989	1,117	1,307	0.68	14,674	Malawi	Base Estimate	2212 - Specialist medical practitioners	Low Income	Southern	Medical Doctors
53	Radiation Oncologist	48	55	63	0.03	304,620	Malawi	Base Estimate	2212 - Specialist medical practitioners	Low Income	Southern	Medical Doctors
54	Radiographer (Diagnostics and Therapy)	1,389	1,591	1,877	0.98	10,179	Malawi	Base Estimate	3211 - Medical imaging and therapeutic equipment technicians	Low Income	Southern	Other Health Workers
55	Radiologist	466	544	657	0.35	28,702	Malawi	Base Estimate	2212 - Specialist medical practitioners	Low Income	Southern	Medical Doctors
56	Registered General Nurse / State Certified Nurse	43,102	48,311	55,010	28.62	349	Malawi	Base Estimate	2221 - Nursing professionals	Low Income	Southern	Nursing Personnel
57	Renal Nurse	3,255	3,747	4,512	2.36	4,229	Malawi	Base Estimate	2221 - Nursing professionals	Low Income	Southern	Nursing Personnel
58	Respiratory Physician	199	225	255	0.13	75,567	Malawi	Base Estimate	2212 - Specialist medical practitioners	Low Income	Southern	Medical Doctors
59	Rheumatologist	54	61	69	0.04	278,935	Malawi	Base Estimate	2212 - Specialist medical practitioners	Low Income	Southern	Medical Doctors
60	Speech Therapist	198	220	244	0.13	79,114	Malawi	Base Estimate	2266 - Audiologists and speech therapists	Low Income	Southern	Other Health Workers
61	Urologist	20	24	31	0.02	606,163	Malawi	Base Estimate	2212 - Specialist medical practitioners	Low Income	Southern	Medical Doctors
1	Anaesthesiologist	181	207	236	0.12	86,470	Mali	Base Estimate	2212 - Specialist medical practitioners	Low Income	West	Medical Doctors
2	Associate Nurse/Enrolled Nurse/Nursing Assistant	23,090	26,100	29,534	14.49	690	Mali	Base Estimate	3221 - Nursing associate professionals	Low Income	West	Nursing Personnel
3	Audiologist	79	90	101	0.05	201,773	Mali	Base Estimate	2266 - Audiologists and speech therapists	Low Income	West	Other Health Workers
4	Cardiologist	123	139	155	0.08	131,554	Mali	Base Estimate	2212 - Specialist medical practitioners	Low Income	West	Medical Doctors
5	Cardiothoracic Surgeon	27	31	35	0.02	591,670	Mali	Base Estimate	2212 - Specialist medical practitioners	Low Income	West	Medical Doctors
6	Clinical Officer/Physician Assistant	1,749	1,826	1,945	0.96	10,382	Mali	Base Estimate	3256 - Medical assistants	Low Income	West	Other Health Workers
7	Clinical Pharmacist	590	669	766	0.38	26,554	Mali	Base Estimate	2262 - Pharmacists	Low Income	West	Pharmacist
8	Clinical Psychologist	1,305	1,453	1,627	0.80	12,518	Mali	Base Estimate	2634 - Psychologists	Low Income	West	Other Health Workers
9	Community health worker/Village health worker	19,281	21,551	24,154	11.87	843	Mali	Base Estimate	3253 - Community health workers	Low Income	West	Other Health Workers
10	Dental Surgery Assistant	2,093	2,369	2,695	1.32	7,570	Mali	Base Estimate	3251 - Dental assistants and therapists	Low Income	West	Other Health Workers
11	Dental Therapist	1,357	1,536	1,747	0.86	11,677	Mali	Base Estimate	3251 - Dental assistants and therapists	Low Income	West	Other Health Workers
12	Dentist	1,418	1,607	1,858	0.91	10,962	Mali	Base Estimate	2261 - Dentists	Low Income	West	Dentist
13	Dermatologist	71	81	91	0.04	223,778	Mali	Base Estimate	2212 - Specialist medical practitioners	Low Income	West	Medical Doctors
14	Endocrinologist	52	62	76	0.04	265,518	Mali	Base Estimate	2212 - Specialist medical practitioners	Low Income	West	Medical Doctors
15	ENT Surgeon	204	231	261	0.13	78,181	Mali	Base Estimate	2212 - Specialist medical practitioners	Low Income	West	Medical Doctors
16	Environmental Health Officer	1,006	1,134	1,275	0.62	16,022	Mali	Base Estimate	2263 - Environmental and occupational health and hygiene professionals	Low Income	West	Other Health Workers
17	Gastroenterologist	193	209	231	0.11	87,407	Mali	Base Estimate	2212 - Specialist medical practitioners	Low Income	West	Medical Doctors
18	General Medical Practitioner (Generalist Doctor)	7,846	8,834	9,967	4.89	2,044	Mali	Base Estimate	2211 - Generalist medical practitioners	Low Income	West	Medical Doctors
19	General Surgeon	185	214	254	0.13	79,975	Mali	Base Estimate	2212 - Specialist medical practitioners	Low Income	West	Medical Doctors
20	Haematologist	123	146	173	0.08	117,886	Mali	Base Estimate	2212 - Specialist medical practitioners	Low Income	West	Medical Doctors
21	Health Promoter/Health Educator	168	176	187	0.09	108,348	Mali	Base Estimate	2269 - Health professionals not elsewhere classified	Low Income	West	Other Health Workers
22	Infectious Diseases Specialist	19	22	25	0.01	818,066	Mali	Base Estimate	2212 - Specialist medical practitioners	Low Income	West	Medical Doctors
23	Intensive Care Nurse	660	745	839	0.41	24,318	Mali	Base Estimate	2221 - Nursing professionals	Low Income	West	Nursing Personnel
24	Medical Laboratory Scientist	2,094	2,361	2,696	1.33	7,527	Mali	Base Estimate	3212 - Medical and pathology laboratory technicians	Low Income	West	Other Health Workers
25	Medical Laboratory Technician	3,319	3,771	4,315	2.12	4,708	Mali	Base Estimate	3212 - Medical and pathology laboratory technicians	Low Income	West	Other Health Workers
26	Medical Social Worker	1,016	1,116	1,228	0.60	16,586	Mali	Base Estimate	1344 - Social welfare managers	Low Income	West	Other Health Workers
27	Mental Health Nurse	967	1,105	1,291	0.64	15,674	Mali	Base Estimate	2221 - Nursing professionals	Low Income	West	Nursing Personnel
28	Midwife	19,900	22,765	26,122	12.81	781	Mali	Base Estimate	2222 - Midwifery professionals	Low Income	West	Midwifery Personnel
29	Nephrologist	153	175	206	0.10	98,036	Mali	Base Estimate	2212 - Specialist medical practitioners	Low Income	West	Medical Doctors
30	Neuro-Surgeon	45	51	59	0.03	344,756	Mali	Base Estimate	2212 - Specialist medical practitioners	Low Income	West	Medical Doctors
31	Nurse Anaesthetist	334	382	437	0.21	46,687	Mali	Base Estimate	2221 - Nursing professionals	Low Income	West	Nursing Personnel
32	Nutritionist	4,638	5,144	5,687	2.78	3,591	Mali	Base Estimate	2265 - Dieticians and nutritionists	Low Income	West	Other Health Workers
33	Obstetrician & Gynaecologist	1,338	1,513	1,714	0.84	11,893	Mali	Base Estimate	2212 - Specialist medical practitioners	Low Income	West	Medical Doctors
34	Occupational Therapist	338	390	451	0.22	45,042	Mali	Base Estimate	2269 - Health professionals not elsewhere classified	Low Income	West	Other Health Workers
35	Oncology Nurse	113	130	151	0.07	133,952	Mali	Base Estimate	2221 - Nursing professionals	Low Income	West	Nursing Personnel
36	Operating Theatre Nurse	1,673	1,922	2,252	1.11	8,981	Mali	Base Estimate	2221 - Nursing professionals	Low Income	West	Nursing Personnel
37	Ophthalmic Nurse	217	246	281	0.14	72,363	Mali	Base Estimate	2221 - Nursing professionals	Low Income	West	Nursing Personnel
38	Ophthalmologist	62	71	81	0.04	251,142	Mali	Base Estimate	2212 - Specialist medical practitioners	Low Income	West	Medical Doctors
39	Optometrist	253	281	313	0.15	65,075	Mali	Base Estimate	2267 - Optometrists and ophthalmic opticians	Low Income	West	Other Health Workers
40	Orthopaedic Nurse	341	394	453	0.22	45,040	Mali	Base Estimate	2221 - Nursing professionals	Low Income	West	Nursing Personnel
41	Orthopaedic Surgeon	510	593	691	0.34	29,416	Mali	Base Estimate	2212 - Specialist medical practitioners	Low Income	West	Medical Doctors
42	Orthopaedic Technologist	578	655	739	0.36	27,641	Mali	Base Estimate	3214 - Medical and dental prosthetic technicians	Low Income	West	Other Health Workers
43	Paediatric Nurse	3,415	3,918	4,466	2.19	4,569	Mali	Base Estimate	2221 - Nursing professionals	Low Income	West	Nursing Personnel
44	Paediatric Surgeon	90	102	115	0.06	178,277	Mali	Base Estimate	2212 - Specialist medical practitioners	Low Income	West	Medical Doctors
45	Paediatrician	863	1,005	1,173	0.58	17,337	Mali	Base Estimate	2212 - Specialist medical practitioners	Low Income	West	Medical Doctors
46	Pathologist	131	146	168	0.08	120,686	Mali	Base Estimate	2212 - Specialist medical practitioners	Low Income	West	Medical Doctors
47	Pharmacist	1,278	1,340	1,414	0.70	14,356	Mali	Base Estimate	2262 - Pharmacists	Low Income	West	Pharmacist
48	Pharmacy Technician	1,993	2,241	2,525	1.24	8,072	Mali	Base Estimate	3213 - Pharmaceutical technicians and assistants	Low Income	West	Other Health Workers
49	Physician	2,367	2,697	3,083	1.51	6,605	Mali	Base Estimate	2212 - Specialist medical practitioners	Low Income	West	Medical Doctors
50	Physiotherapist	503	570	645	0.32	31,626	Mali	Base Estimate	2264 - Physiotherapists	Low Income	West	Other Health Workers
51	Plastic Surgeon	128	147	168	0.08	121,689	Mali	Base Estimate	2212 - Specialist medical practitioners	Low Income	West	Medical Doctors
52	Psychiatrist	1,107	1,249	1,434	0.71	14,163	Mali	Base Estimate	2212 - Specialist medical practitioners	Low Income	West	Medical Doctors
53	Radiation Oncologist	33	38	44	0.02	466,454	Mali	Base Estimate	2212 - Specialist medical practitioners	Low Income	West	Medical Doctors
54	Radiographer (Diagnostics and Therapy)	1,351	1,542	1,782	0.88	11,397	Mali	Base Estimate	3211 - Medical imaging and therapeutic equipment technicians	Low Income	West	Other Health Workers
55	Radiologist	491	563	650	0.32	31,279	Mali	Base Estimate	2212 - Specialist medical practitioners	Low Income	West	Medical Doctors
56	Registered General Nurse / State Certified Nurse	54,454	60,123	66,764	32.85	304	Mali	Base Estimate	2221 - Nursing professionals	Low Income	West	Nursing Personnel

S/N	Health Professionals	2022	2026	2030	Density per 10,000 population	Required Population ratio (1 professional is to xxx population)	Name of Country	Modelling Scenario	ISCO-08 Match	Income Group Classification	Sub-Regional Grouping	SDG 3c Occupation
57	Renal Nurse	1,998	2,280	2,679	1.33	7,532	Mali	Base Estimate	2221 - Nursing professionals	Low Income	West	Nursing Personnel
58	Respiratory Physician	131	154	180	0.09	112,812	Mali	Base Estimate	2212 - Specialist medical practitioners	Low Income	West	Medical Doctors
59	Rheumatologist	55	62	70	0.03	291,125	Mali	Base Estimate	2212 - Specialist medical practitioners	Low Income	West	Medical Doctors
60	Speech Therapist	201	227	256	0.13	79,881	Mali	Base Estimate	2266 - Audiologists and speech therapists	Low Income	West	Other Health Workers
61	Urologist	25	29	34	0.02	587,548	Mali	Base Estimate	2212 - Specialist medical practitioners	Low Income	West	Medical Doctors
1	Anaesthesiologist	65	73	82	0.18	56,858	Mauritania	Base Estimate	2212 - Specialist medical practitioners	Lower-middle Income	West	Medical Doctors
2	Associate Nurse/Enrolled Nurse/Nursing Assistant	6,025	6,901	8,104	17.15	583	Mauritania	Base Estimate	3221 - Nursing associate professionals	Lower-middle Income	West	Nursing Personnel
3	Audiologist	18	21	23	0.05	200,673	Mauritania	Base Estimate	2266 - Audiologists and speech therapists	Lower-middle Income	West	Other Health Workers
4	Cardiologist	43	49	57	0.12	82,793	Mauritania	Base Estimate	2212 - Specialist medical practitioners	Lower-middle Income	West	Medical Doctors
5	Cardiothoracic Surgeon	10	11	12	0.03	387,572	Mauritania	Base Estimate	2212 - Specialist medical practitioners	Lower-middle Income	West	Medical Doctors
6	Clinical Officer/Physician Assistant	672	988	1,648	3.31	3,017	Mauritania	Base Estimate	3256 - Medical assistants	Lower-middle Income	West	Other Health Workers
7	Clinical Pharmacist	162	179	201	0.43	23,297	Mauritania	Base Estimate	2262 - Pharmacists	Lower-middle Income	West	Pharmacist
8	Clinical Psychologist	333	369	411	0.88	11,392	Mauritania	Base Estimate	2634 - Psychologists	Lower-middle Income	West	Other Health Workers
9	Community health worker/Village health worker	4,662	6,563	10,427	21.02	476	Mauritania	Base Estimate	3253 - Community health workers	Lower-middle Income	West	Other Health Workers
10	Dental Surgery Assistant	574	650	736	1.57	6,370	Mauritania	Base Estimate	3251 - Dental assistants and therapists	Lower-middle Income	West	Other Health Workers
11	Dental Therapist	372	421	477	1.02	9,829	Mauritania	Base Estimate	3251 - Dental assistants and therapists	Lower-middle Income	West	Other Health Workers
12	Dentist	383	433	490	1.05	9,559	Mauritania	Base Estimate	2261 - Dentists	Lower-middle Income	West	Dentist
13	Dermatologist	19	21	24	0.05	198,691	Mauritania	Base Estimate	2212 - Specialist medical practitioners	Lower-middle Income	West	Medical Doctors
14	Endocrinologist	16	19	24	0.05	193,707	Mauritania	Base Estimate	2212 - Specialist medical practitioners	Lower-middle Income	West	Medical Doctors
15	ENT Surgeon	56	64	73	0.16	63,628	Mauritania	Base Estimate	2212 - Specialist medical practitioners	Lower-middle Income	West	Medical Doctors
16	Environmental Health Officer	231	256	284	0.60	16,534	Mauritania	Base Estimate	2263 - Environmental and occupational health and hygiene professionals	Lower-middle Income	West	Other Health Workers
17	Gastroenterologist	85	87	90	0.19	51,609	Mauritania	Base Estimate	2212 - Specialist medical practitioners	Lower-middle Income	West	Medical Doctors
18	General Medical Practitioner (Generalist Doctor)	1,985	2,342	2,906	6.09	1,642	Mauritania	Base Estimate	2211 - Generalist medical practitioners	Lower-middle Income	West	Medical Doctors
19	General Surgeon	49	56	66	0.14	71,113	Mauritania	Base Estimate	2212 - Specialist medical practitioners	Lower-middle Income	West	Medical Doctors
20	Haematologist	17	18	19	0.04	247,965	Mauritania	Base Estimate	2212 - Specialist medical practitioners	Lower-middle Income	West	Medical Doctors
21	Health Promoter/Health Educator	56	76	117	0.24	42,138	Mauritania	Base Estimate	2269 - Health professionals not elsewhere classified	Lower-middle Income	West	Other Health Workers
22	Infectious Diseases Specialist	5	6	6	0.01	727,582	Mauritania	Base Estimate	2212 - Specialist medical practitioners	Lower-middle Income	West	Medical Doctors
23	Intensive Care Nurse	146	160	176	0.37	26,692	Mauritania	Base Estimate	2221 - Nursing professionals	Lower-middle Income	West	Nursing Personnel
24	Medical Laboratory Scientist	598	752	1,035	2.14	4,673	Mauritania	Base Estimate	3212 - Medical and pathology laboratory technicians	Lower-middle Income	West	Other Health Workers
25	Medical Laboratory Technician	856	1,390	2,491	4.96	2,015	Mauritania	Base Estimate	3212 - Medical and pathology laboratory technicians	Lower-middle Income	West	Other Health Workers
26	Medical Social Worker	234	242	252	0.54	18,540	Mauritania	Base Estimate	1344 - Social welfare managers	Lower-middle Income	West	Other Health Workers
27	Mental Health Nurse	262	296	340	0.73	13,727	Mauritania	Base Estimate	2221 - Nursing professionals	Lower-middle Income	West	Nursing Personnel
28	Midwife	3,512	3,878	4,268	9.10	1,099	Mauritania	Base Estimate	2222 - Midwifery professionals	Lower-middle Income	West	Midwifery Personnel
29	Nephrologist	61	71	83	0.18	56,021	Mauritania	Base Estimate	2212 - Specialist medical practitioners	Lower-middle Income	West	Medical Doctors
30	Neuro-Surgeon	19	22	25	0.05	190,149	Mauritania	Base Estimate	2212 - Specialist medical practitioners	Lower-middle Income	West	Medical Doctors
31	Nurse Anaesthetist	127	147	171	0.37	27,295	Mauritania	Base Estimate	2221 - Nursing professionals	Lower-middle Income	West	Nursing Personnel
32	Nutritionist	691	719	746	1.59	6,275	Mauritania	Base Estimate	2265 - Dieticians and nutritionists	Lower-middle Income	West	Other Health Workers
33	Obstetrician & Gynaecologist	326	365	408	0.87	11,478	Mauritania	Base Estimate	2212 - Specialist medical practitioners	Lower-middle Income	West	Medical Doctors
34	Occupational Therapist	145	163	184	0.39	25,479	Mauritania	Base Estimate	2269 - Health professionals not elsewhere classified	Lower-middle Income	West	Other Health Workers
35	Oncology Nurse	44	51	61	0.13	76,849	Mauritania	Base Estimate	2221 - Nursing professionals	Lower-middle Income	West	Nursing Personnel
36	Operating Theatre Nurse	587	666	765	1.64	6,104	Mauritania	Base Estimate	2221 - Nursing professionals	Lower-middle Income	West	Nursing Personnel
37	Ophthalmic Nurse	64	73	84	0.18	55,674	Mauritania	Base Estimate	2221 - Nursing professionals	Lower-middle Income	West	Nursing Personnel
38	Ophthalmologist	18	21	25	0.05	189,553	Mauritania	Base Estimate	2212 - Specialist medical practitioners	Lower-middle Income	West	Medical Doctors
39	Optometrist	74	85	98	0.21	47,914	Mauritania	Base Estimate	2267 - Optometrists and ophthalmic opticians	Lower-middle Income	West	Other Health Workers
40	Orthopaedic Nurse	161	181	203	0.43	23,143	Mauritania	Base Estimate	2221 - Nursing professionals	Lower-middle Income	West	Nursing Personnel
41	Orthopaedic Surgeon	205	237	273	0.58	17,118	Mauritania	Base Estimate	2212 - Specialist medical practitioners	Lower-middle Income	West	Medical Doctors
42	Orthopaedic Technologist	169	188	209	0.45	22,420	Mauritania	Base Estimate	3214 - Medical and dental prosthetic technicians	Lower-middle Income	West	Other Health Workers
43	Paediatric Nurse	580	625	667	1.42	7,033	Mauritania	Base Estimate	2221 - Nursing professionals	Lower-middle Income	West	Nursing Personnel
44	Paediatric Surgeon	15	16	17	0.04	271,580	Mauritania	Base Estimate	2212 - Specialist medical practitioners	Lower-middle Income	West	Medical Doctors
45	Paediatrician	265	297	335	0.72	13,980	Mauritania	Base Estimate	2212 - Specialist medical practitioners	Lower-middle Income	West	Medical Doctors
46	Pathologist	35	39	45	0.10	103,426	Mauritania	Base Estimate	2212 - Specialist medical practitioners	Lower-middle Income	West	Medical Doctors
47	Pharmacist	297	307	320	0.69	14,490	Mauritania	Base Estimate	2262 - Pharmacists	Lower-middle Income	West	Pharmacist
48	Pharmacy Technician	553	613	682	1.46	6,860	Mauritania	Base Estimate	3213 - Pharmaceutical technicians and assistants	Lower-middle Income	West	Other Health Workers
49	Physician	598	695	832	1.76	5,681	Mauritania	Base Estimate	2212 - Specialist medical practitioners	Lower-middle Income	West	Medical Doctors
50	Physiotherapist	145	162	182	0.39	25,736	Mauritania	Base Estimate	2264 - Physiotherapists	Lower-middle Income	West	Other Health Workers
51	Plastic Surgeon	37	43	49	0.11	95,073	Mauritania	Base Estimate	2212 - Specialist medical practitioners	Lower-middle Income	West	Medical Doctors
52	Psychiatrist	269	300	339	0.73	13,780	Mauritania	Base Estimate	2212 - Specialist medical practitioners	Lower-middle Income	West	Medical Doctors
53	Radiation Oncologist	13	15	19	0.04	251,451	Mauritania	Base Estimate	2212 - Specialist medical practitioners	Lower-middle Income	West	Medical Doctors
54	Radiographer (Diagnostics and Therapy)	432	490	561	1.20	8,329	Mauritania	Base Estimate	3211 - Medical imaging and therapeutic equipment technicians	Lower-middle Income	West	Other Health Workers
55	Radiologist	154	174	197	0.42	23,783	Mauritania	Base Estimate	2212 - Specialist medical practitioners	Lower-middle Income	West	Medical Doctors
56	Registered General Nurse / State Certified Nurse	13,496	14,770	16,292	34.91	286	Mauritania	Base Estimate	2221 - Nursing professionals	Lower-middle Income	West	Nursing Personnel
57	Renal Nurse	799	928	1,095	2.35	4,258	Mauritania	Base Estimate	2221 - Nursing professionals	Lower-middle Income	West	Nursing Personnel
58	Respiratory Physician	56	64	72	0.15	64,867	Mauritania	Base Estimate	2212 - Specialist medical practitioners	Lower-middle Income	West	Medical Doctors
59	Rheumatologist	15	17	19	0.04	242,677	Mauritania	Base Estimate	2212 - Specialist medical practitioners	Lower-middle Income	West	Medical Doctors
60	Speech Therapist	49	55	61	0.13	76,056	Mauritania	Base Estimate	2266 - Audiologists and speech therapists	Lower-middle Income	West	Other Health Workers
61	Urologist	8	10	12	0.02	405,110	Mauritania	Base Estimate	2212 - Specialist medical practitioners	Lower-middle Income	West	Medical Doctors
1	Anaesthesiologist	42	47	53	0.42	23,989	Mauritius	Base Estimate	2212 - Specialist medical practitioners	High Income and Upper Middle Income	Southern	Medical Doctors
2	Associate Nurse/Enrolled Nurse/Nursing Assistant	2,162	2,259	2,372	18.51	540	Mauritius	Base Estimate	3221 - Nursing associate professionals	High Income and Upper Middle Income	Southern	Nursing Personnel
3	Audiologist	5	6	6	0.05	210,348	Mauritius	Base Estimate	2266 - Audiologists and speech therapists	High Income and Upper Middle Income	Southern	Other Health Workers
4	Cardiologist	48	55	63	0.49	20,396	Mauritius	Base Estimate	2212 - Specialist medical practitioners	High Income and Upper Middle Income	Southern	Medical Doctors
5	Cardiothoracic Surgeon	4	4	4	0.03	302,750	Mauritius	Base Estimate	2212 - Specialist medical practitioners	High Income and Upper Middle Income	Southern	Medical Doctors
6	Clinical Officer/Physician Assistant	476	496	518	4.04	2,477	Mauritius	Base Estimate	3256 - Medical assistants	High Income and Upper Middle Income	Southern	Other Health Workers
7	Clinical Pharmacist	85	91	98	0.76	13,181	Mauritius	Base Estimate	2262 - Pharmacists	High Income and Upper Middle Income	Southern	Pharmacist
8	Clinical Psychologist	90	91	92	0.72	13,868	Mauritius	Base Estimate	2634 - Psychologists	High Income and Upper Middle Income	Southern	Other Health Workers
9	Community health worker/Village health worker	810	810	807	6.33	1,580	Mauritius	Base Estimate	3253 - Community health workers	High Income and Upper Middle Income	Southern	Other Health Workers
10	Dental Surgery Assistant	115	120	124	0.96	10,411	Mauritius	Base Estimate	3251 - Dental assistants and therapists	High Income and Upper Middle Income	Southern	Other Health Workers
11	Dental Therapist	75	78	80	0.62	16,059	Mauritius	Base Estimate	3251 - Dental assistants and therapists	High Income and Upper Middle Income	Southern	Other Health Workers
12	Dentist	95	95	93	0.72	13,823	Mauritius	Base Estimate	2261 - Dentists	High Income and Upper Middle Income	Southern	Dentist
13	Dermatologist	5	6	6	0.05	219,621	Mauritius	Base Estimate	2212 - Specialist medical practitioners	High Income and Upper Middle Income	Southern	Medical Doctors
14	Endocrinologist	49	55	61	0.47	21,370	Mauritius	Base Estimate	2212 - Specialist medical practitioners	High Income and Upper Middle Income	Southern	Medical Doctors
15	ENT Surgeon	39	42	50	0.40	25,303	Mauritius	Base Estimate	2212 - Specialist medical practitioners	High Income and Upper Middle Income	Southern	Medical Doctors
16	Environmental Health Officer	63	63	63	0.50	20,082	Mauritius	Base Estimate	2263 - Environmental and occupational health and hygiene professionals	High Income and Upper Middle Income	Southern	Other Health Workers
17	Gastroenterologist	10	10	10	0.08	133,104	Mauritius	Base Estimate	2212 - Specialist medical practitioners	High Income and Upper Middle Income	Southern	Medical Doctors
18	General Medical Practitioner (Generalist Doctor)	869	904	940	7.34	1,362	Mauritius	Base Estimate	2211 - Generalist medical practitioners	High Income and Upper Middle Income	Southern	Medical Doctors

S/N	Health Professionals	2022	2026	2030	Density per 10,000 population	Required Population ratio (1 professional is to xxx population)	Name of Country	Modelling Scenario	ISCO-08 Match	Income Group Classification	Sub-Regional Grouping	SDG 3c Occupation
19	General Surgeon	40	45	50	0.39	25,783	Mauritius	Base Estimate	2212 - Specialist medical practitioners	High Income and Upper Middle Income	Southern	Medical Doctors
20	Haematologist	5	5	5	0.04	236,927	Mauritius	Base Estimate	2212 - Specialist medical practitioners	High Income and Upper Middle Income	Southern	Medical Doctors
21	Health Promoter/Health Educator	38	39	40	0.32	31,718	Mauritius	Base Estimate	2269 - Health professionals not elsewhere classified	High Income and Upper Middle Income	Southern	Other Health Workers
22	Infectious Diseases Specialist	1	1	1	0.01	887,955	Mauritius	Base Estimate	2212 - Specialist medical practitioners	High Income and Upper Middle Income	Southern	Medical Doctors
23	Intensive Care Nurse	64	67	73	0.58	17,391	Mauritius	Base Estimate	2221 - Nursing professionals	High Income and Upper Middle Income	Southern	Nursing Personnel
24	Medical Laboratory Scientist	415	454	498	3.86	2,589	Mauritius	Base Estimate	3212 - Medical and pathology laboratory technicians	High Income and Upper Middle Income	Southern	Other Health Workers
25	Medical Laboratory Technician	318	347	379	2.95	3,393	Mauritius	Base Estimate	3212 - Medical and pathology laboratory technicians	High Income and Upper Middle Income	Southern	Other Health Workers
26	Medical Social Worker	43	42	40	0.32	31,541	Mauritius	Base Estimate	1344 - Social welfare managers	High Income and Upper Middle Income	Southern	Other Health Workers
27	Mental Health Nurse	124	124	124	0.96	10,368	Mauritius	Base Estimate	2221 - Nursing professionals	High Income and Upper Middle Income	Southern	Nursing Personnel
28	Midwife	896	894	883	6.88	1,452	Mauritius	Base Estimate	2222 - Midwifery professionals	High Income and Upper Middle Income	Southern	Midwifery Personnel
29	Nephrologist	89	98	108	0.84	11,937	Mauritius	Base Estimate	2212 - Specialist medical practitioners	High Income and Upper Middle Income	Southern	Medical Doctors
30	Neuro-Surgeon	19	21	26	0.20	49,095	Mauritius	Base Estimate	2212 - Specialist medical practitioners	High Income and Upper Middle Income	Southern	Medical Doctors
31	Nurse Anaesthetist	159	184	212	1.65	6,048	Mauritius	Base Estimate	2221 - Nursing professionals	High Income and Upper Middle Income	Southern	Nursing Personnel
32	Nutritionist	172	167	162	1.28	7,817	Mauritius	Base Estimate	2265 - Dieticians and nutritionists	High Income and Upper Middle Income	Southern	Other Health Workers
33	Obstetrician & Gynaecologist	98	99	100	0.78	12,807	Mauritius	Base Estimate	2212 - Specialist medical practitioners	High Income and Upper Middle Income	Southern	Medical Doctors
34	Occupational Therapist	61	66	71	0.55	18,115	Mauritius	Base Estimate	2269 - Health professionals not elsewhere classified	High Income and Upper Middle Income	Southern	Other Health Workers
35	Oncology Nurse	71	80	90	0.70	14,337	Mauritius	Base Estimate	2221 - Nursing professionals	High Income and Upper Middle Income	Southern	Nursing Personnel
36	Operating Theatre Nurse	434	470	522	4.08	2,454	Mauritius	Base Estimate	2221 - Nursing professionals	High Income and Upper Middle Income	Southern	Nursing Personnel
37	Ophthalmic Nurse	79	85	93	0.72	13,890	Mauritius	Base Estimate	2221 - Nursing professionals	High Income and Upper Middle Income	Southern	Nursing Personnel
38	Ophthalmologist	27	30	35	0.27	36,927	Mauritius	Base Estimate	2212 - Specialist medical practitioners	High Income and Upper Middle Income	Southern	Medical Doctors
39	Optometrist	66	73	82	0.63	15,791	Mauritius	Base Estimate	2267 - Optometrists and ophthalmic opticians	High Income and Upper Middle Income	Southern	Other Health Workers
40	Orthopaedic Nurse	31	32	34	0.27	36,835	Mauritius	Base Estimate	2221 - Nursing professionals	High Income and Upper Middle Income	Southern	Nursing Personnel
41	Orthopaedic Surgeon	153	164	178	1.38	7,221	Mauritius	Base Estimate	2212 - Specialist medical practitioners	High Income and Upper Middle Income	Southern	Medical Doctors
42	Orthopaedic Technologist	41	41	42	0.33	29,969	Mauritius	Base Estimate	3214 - Medical and dental prosthetic technicians	High Income and Upper Middle Income	Southern	Other Health Workers
43	Paediatric Nurse	109	107	104	0.82	12,253	Mauritius	Base Estimate	2221 - Nursing professionals	High Income and Upper Middle Income	Southern	Nursing Personnel
44	Paediatric Surgeon	2	1	1	0.01	900,661	Mauritius	Base Estimate	2212 - Specialist medical practitioners	High Income and Upper Middle Income	Southern	Medical Doctors
45	Paediatrician	106	113	120	0.93	10,709	Mauritius	Base Estimate	2212 - Specialist medical practitioners	High Income and Upper Middle Income	Southern	Medical Doctors
46	Pathologist	13	14	14	0.11	90,226	Mauritius	Base Estimate	2212 - Specialist medical practitioners	High Income and Upper Middle Income	Southern	Medical Doctors
47	Pharmacist	377	392	407	3.18	3,142	Mauritius	Base Estimate	2262 - Pharmacists	High Income and Upper Middle Income	Southern	Pharmacist
48	Pharmacy Technician	282	292	302	2.36	4,231	Mauritius	Base Estimate	3213 - Pharmaceutical technicians and assistants	High Income and Upper Middle Income	Southern	Other Health Workers
49	Physician	341	364	389	3.02	3,310	Mauritius	Base Estimate	2212 - Specialist medical practitioners	High Income and Upper Middle Income	Southern	Medical Doctors
50	Physiotherapist	62	65	72	0.57	17,578	Mauritius	Base Estimate	2264 - Physiotherapists	High Income and Upper Middle Income	Southern	Other Health Workers
51	Plastic Surgeon	24	25	25	0.19	51,696	Mauritius	Base Estimate	2212 - Specialist medical practitioners	High Income and Upper Middle Income	Southern	Medical Doctors
52	Psychiatrist	103	102	101	0.79	12,695	Mauritius	Base Estimate	2212 - Specialist medical practitioners	High Income and Upper Middle Income	Southern	Medical Doctors
53	Radiation Oncologist	29	34	40	0.31	32,033	Mauritius	Base Estimate	2212 - Specialist medical practitioners	High Income and Upper Middle Income	Southern	Medical Doctors
54	Radiographer (Diagnostics and Therapy)	292	316	343	2.66	3,760	Mauritius	Base Estimate	3211 - Medical imaging and therapeutic equipment technicians	High Income and Upper Middle Income	Southern	Other Health Workers
55	Radiologist	85	91	97	0.76	13,181	Mauritius	Base Estimate	2212 - Specialist medical practitioners	High Income and Upper Middle Income	Southern	Medical Doctors
56	Registered General Nurse / State Certified Nurse	7,122	7,752	8,463	65.71	152	Mauritius	Base Estimate	2221 - Nursing professionals	High Income and Upper Middle Income	Southern	Nursing Personnel
57	Renal Nurse	1,211	1,341	1,483	11.46	873	Mauritius	Base Estimate	2221 - Nursing professionals	High Income and Upper Middle Income	Southern	Nursing Personnel
58	Respiratory Physician	12	12	12	0.09	107,331	Mauritius	Base Estimate	2212 - Specialist medical practitioners	High Income and Upper Middle Income	Southern	Medical Doctors
59	Rheumatologist	7	7	8	0.06	165,219	Mauritius	Base Estimate	2212 - Specialist medical practitioners	High Income and Upper Middle Income	Southern	Medical Doctors
60	Speech Therapist	28	29	35	0.28	36,065	Mauritius	Base Estimate	2266 - Audiologists and speech therapists	High Income and Upper Middle Income	Southern	Other Health Workers
61	Urologist	21	25	30	0.23	42,998	Mauritius	Base Estimate	2212 - Specialist medical practitioners	High Income and Upper Middle Income	Southern	Medical Doctors
1	Anaesthesiologist	330	373	421	0.13	74,988	Mozambique	Base Estimate	2212 - Specialist medical practitioners	Low Income	Southern	Medical Doctors
2	Associate Nurse/Enrolled Nurse/Nursing Assistant	40,155	44,814	50,107	15.91	628	Mozambique	Base Estimate	3221 - Nursing associate professionals	Low Income	Southern	Nursing Personnel
3	Audiologist	124	139	154	0.05	204,362	Mozambique	Base Estimate	2266 - Audiologists and speech therapists	Low Income	Southern	Other Health Workers
4	Cardiologist	195	218	241	0.08	130,852	Mozambique	Base Estimate	2212 - Specialist medical practitioners	Low Income	Southern	Medical Doctors
5	Cardiothoracic Surgeon	64	72	81	0.03	388,959	Mozambique	Base Estimate	2212 - Specialist medical practitioners	Low Income	Southern	Medical Doctors
6	Clinical Officer/Physician Assistant	6,443	6,985	7,680	2.45	4,078	Mozambique	Base Estimate	3256 - Medical assistants	Low Income	Southern	Other Health Workers
7	Clinical Pharmacist	898	999	1,127	0.36	27,877	Mozambique	Base Estimate	2262 - Pharmacists	Low Income	Southern	Pharmacist
8	Clinical Psychologist	2,163	2,403	2,689	0.85	11,709	Mozambique	Base Estimate	2634 - Psychologists	Low Income	Southern	Other Health Workers
9	Community health worker/Village health worker	35,885	39,289	42,971	13.64	733	Mozambique	Base Estimate	3253 - Community health workers	Low Income	Southern	Other Health Workers
10	Dental Surgery Assistant	2,960	3,294	3,689	1.17	8,553	Mozambique	Base Estimate	3251 - Dental assistants and therapists	Low Income	Southern	Other Health Workers
11	Dental Therapist	1,915	2,131	2,386	0.76	13,226	Mozambique	Base Estimate	3251 - Dental assistants and therapists	Low Income	Southern	Other Health Workers
12	Dentist	2,496	2,780	3,153	1.00	9,995	Mozambique	Base Estimate	2261 - Dentists	Low Income	Southern	Dentist
13	Dermatologist	103	116	129	0.04	244,589	Mozambique	Base Estimate	2212 - Specialist medical practitioners	Low Income	Southern	Medical Doctors
14	Endocrinologist	93	109	132	0.04	237,525	Mozambique	Base Estimate	2212 - Specialist medical practitioners	Low Income	Southern	Medical Doctors
15	ENT Surgeon	314	355	400	0.13	78,867	Mozambique	Base Estimate	2212 - Specialist medical practitioners	Low Income	Southern	Medical Doctors
16	Environmental Health Officer	1,552	1,732	1,923	0.61	16,412	Mozambique	Base Estimate	2263 - Environmental and occupational health and hygiene professionals	Low Income	Southern	Other Health Workers
17	Gastroenterologist	312	314	328	0.10	95,306	Mozambique	Base Estimate	2212 - Specialist medical practitioners	Low Income	Southern	Medical Doctors
18	General Medical Practitioner (Generalist Doctor)	12,776	14,305	16,081	5.11	1,956	Mozambique	Base Estimate	2211 - Generalist medical practitioners	Low Income	Southern	Medical Doctors
19	General Surgeon	240	280	331	0.11	94,703	Mozambique	Base Estimate	2212 - Specialist medical practitioners	Low Income	Southern	Medical Doctors
20	Haematologist	115	127	138	0.04	228,297	Mozambique	Base Estimate	2212 - Specialist medical practitioners	Low Income	Southern	Medical Doctors
21	Health Promoter/Health Educator	477	517	567	0.18	55,228	Mozambique	Base Estimate	2269 - Health professionals not elsewhere classified	Low Income	Southern	Other Health Workers
22	Infectious Diseases Specialist	34	37	41	0.01	766,229	Mozambique	Base Estimate	2212 - Specialist medical practitioners	Low Income	Southern	Medical Doctors
23	Intensive Care Nurse	811	916	1,032	0.33	30,542	Mozambique	Base Estimate	2221 - Nursing professionals	Low Income	Southern	Nursing Personnel
24	Medical Laboratory Scientist	4,984	5,700	6,588	2.10	4,760	Mozambique	Base Estimate	3212 - Medical and pathology laboratory technicians	Low Income	Southern	Other Health Workers
25	Medical Laboratory Technician	8,029	8,885	9,860	3.14	3,189	Mozambique	Base Estimate	3212 - Medical and pathology laboratory technicians	Low Income	Southern	Other Health Workers
26	Medical Social Worker	1,033	1,098	1,189	0.38	26,283	Mozambique	Base Estimate	1344 - Social welfare managers	Low Income	Southern	Other Health Workers
27	Mental Health Nurse	1,786	2,016	2,342	0.75	13,397	Mozambique	Base Estimate	2221 - Nursing professionals	Low Income	Southern	Nursing Personnel
28	Midwife	27,002	29,627	32,656	10.36	966	Mozambique	Base Estimate	2222 - Midwifery professionals	Low Income	Southern	Midwifery Personnel
29	Nephrologist	235	267	314	0.10	99,694	Mozambique	Base Estimate	2212 - Specialist medical practitioners	Low Income	Southern	Medical Doctors
30	Neuro-Surgeon	105	120	136	0.04	231,519	Mozambique	Base Estimate	2212 - Specialist medical practitioners	Low Income	Southern	Medical Doctors
31	Nurse Anaesthetist	698	805	926	0.29	34,031	Mozambique	Base Estimate	2221 - Nursing professionals	Low Income	Southern	Nursing Personnel
32	Nutritionist	3,183	3,372	3,556	1.13	8,865	Mozambique	Base Estimate	2265 - Dieticians and nutritionists	Low Income	Southern	Other Health Workers
33	Obstetrician & Gynaecologist	2,241	2,528	2,867	0.91	10,964	Mozambique	Base Estimate	2212 - Specialist medical practitioners	Low Income	Southern	Medical Doctors
34	Occupational Therapist	525	596	681	0.22	46,231	Mozambique	Base Estimate	2269 - Health professionals not elsewhere classified	Low Income	Southern	Other Health Workers
35	Oncology Nurse	200	229	267	0.09	117,543	Mozambique	Base Estimate	2221 - Nursing professionals	Low Income	Southern	Nursing Personnel
36	Operating Theatre Nurse	2,783	3,131	3,598	1.15	8,725	Mozambique	Base Estimate	2221 - Nursing professionals	Low Income	Southern	Nursing Personnel
37	Ophthalmic Nurse	330	369	416	0.13	75,589	Mozambique	Base Estimate	2221 - Nursing professionals	Low Income	Southern	Nursing Personnel
38	Ophthalmologist	98	110	126	0.04	250,416	Mozambique	Base Estimate	2212 - Specialist medical practitioners	Low Income	Southern	Medical Doctors
39	Optometrist	399	440	487	0.15	64,727	Mozambique	Base Estimate	2267 - Optometrists and ophthalmic opticians	Low Income	Southern	Other Health Workers
40	Orthopaedic Nurse	463	534	612	0.19	51,522	Mozambique	Base Estimate	2221 - Nursing professionals	Low Income	Southern	Nursing Personnel
41	Orthopaedic Surgeon	779	937	1,137	0.36	27,513	Mozambique	Base Estimate	2212 - Specialist medical practitioners	Low Income	Southern	Medical Doctors

S/N	Health Professionals	2022	2026	2030	Density per 10,000 population	Required Population ratio (1 professional is to xxx population)	Name of Country	Modelling Scenario	ISCO-08 Match	Income Group Classification	Sub-Regional Grouping	SDG 3c Occupation
42	Orthopaedic Technologist	934	1,050	1,176	0.37	26,826	Mozambique	Base Estimate	3214 - Medical and dental prosthetic technicians	Low Income	Southern	Other Health Workers
43	Paediatric Nurse	3,697	4,068	4,438	1.40	7,121	Mozambique	Base Estimate	2221 - Nursing professionals	Low Income	Southern	Nursing Personnel
44	Paediatric Surgeon	126	140	154	0.05	205,354	Mozambique	Base Estimate	2212 - Specialist medical practitioners	Low Income	Southern	Medical Doctors
45	Paediatrician	1,638	1,855	2,105	0.67	14,968	Mozambique	Base Estimate	2212 - Specialist medical practitioners	Low Income	Southern	Medical Doctors
46	Pathologist	240	270	312	0.10	100,463	Mozambique	Base Estimate	2212 - Specialist medical practitioners	Low Income	Southern	Medical Doctors
47	Pharmacist	2,480	2,570	2,689	0.86	11,636	Mozambique	Base Estimate	2262 - Pharmacists	Low Income	Southern	Pharmacist
48	Pharmacy Technician	2,820	3,111	3,449	1.10	9,122	Mozambique	Base Estimate	3213 - Pharmaceutical technicians and assistants	Low Income	Southern	Other Health Workers
49	Physician	3,005	3,340	3,733	1.19	8,434	Mozambique	Base Estimate	2212 - Specialist medical practitioners	Low Income	Southern	Medical Doctors
50	Physiotherapist	809	908	1,016	0.32	31,022	Mozambique	Base Estimate	2264 - Physiotherapists	Low Income	Southern	Other Health Workers
51	Plastic Surgeon	489	558	634	0.20	49,769	Mozambique	Base Estimate	2212 - Specialist medical practitioners	Low Income	Southern	Medical Doctors
52	Psychiatrist	1,529	1,709	1,957	0.62	16,048	Mozambique	Base Estimate	2212 - Specialist medical practitioners	Low Income	Southern	Medical Doctors
53	Radiation Oncologist	68	80	94	0.03	334,582	Mozambique	Base Estimate	2212 - Specialist medical practitioners	Low Income	Southern	Medical Doctors
54	Radiographer (Diagnostics and Therapy)	1,900	2,160	2,503	0.80	12,522	Mozambique	Base Estimate	3211 - Medical imaging and therapeutic equipment technicians	Low Income	Southern	Other Health Workers
55	Radiologist	727	847	1,005	0.32	30,977	Mozambique	Base Estimate	2212 - Specialist medical practitioners	Low Income	Southern	Medical Doctors
56	Registered General Nurse / State Certified Nurse	74,002	79,473	86,175	27.45	364	Mozambique	Base Estimate	2221 - Nursing professionals	Low Income	Southern	Nursing Personnel
57	Renal Nurse	3,082	3,504	4,118	1.31	7,610	Mozambique	Base Estimate	2221 - Nursing professionals	Low Income	Southern	Nursing Personnel
58	Respiratory Physician	368	420	479	0.15	65,869	Mozambique	Base Estimate	2212 - Specialist medical practitioners	Low Income	Southern	Medical Doctors
59	Rheumatologist	84	94	106	0.03	298,736	Mozambique	Base Estimate	2212 - Specialist medical practitioners	Low Income	Southern	Medical Doctors
60	Speech Therapist	333	372	412	0.13	76,524	Mozambique	Base Estimate	2266 - Audiologists and speech therapists	Low Income	Southern	Other Health Workers
61	Urologist	29	34	43	0.01	723,851	Mozambique	Base Estimate	2212 - Specialist medical practitioners	Low Income	Southern	Medical Doctors
1	Anaesthesiologist	34	38	42	0.16	60,946	Namibia	Base Estimate	2212 - Specialist medical practitioners	High Income and Upper Middle Income	Southern	Medical Doctors
2	Associate Nurse/Enrolled Nurse/Nursing Assistant	3,564	4,036	4,571	17.47	572	Namibia	Base Estimate	3221 - Nursing associate professionals	High Income and Upper Middle Income	Southern	Nursing Personnel
3	Audiologist	11	12	13	0.05	202,614	Namibia	Base Estimate	2266 - Audiologists and speech therapists	High Income and Upper Middle Income	Southern	Other Health Workers
4	Cardiologist	27	30	33	0.13	77,021	Namibia	Base Estimate	2212 - Specialist medical practitioners	High Income and Upper Middle Income	Southern	Medical Doctors
5	Cardiothoracic Surgeon	4	5	5	0.02	512,406	Namibia	Base Estimate	2212 - Specialist medical practitioners	High Income and Upper Middle Income	Southern	Medical Doctors
6	Clinical Officer/Physician Assistant	747	1,098	1,494	5.24	1,909	Namibia	Base Estimate	3256 - Medical assistants	High Income and Upper Middle Income	Southern	Other Health Workers
7	Clinical Pharmacist	78	85	97	0.39	25,560	Namibia	Base Estimate	2262 - Pharmacists	High Income and Upper Middle Income	Southern	Pharmacist
8	Clinical Psychologist	140	151	164	0.64	15,596	Namibia	Base Estimate	2634 - Psychologists	High Income and Upper Middle Income	Southern	Other Health Workers
9	Community health worker/Village health worker	2,241	4,127	6,228	20.79	481	Namibia	Base Estimate	3253 - Community health workers	High Income and Upper Middle Income	Southern	Other Health Workers
10	Dental Surgery Assistant	319	348	381	1.49	6,700	Namibia	Base Estimate	3251 - Dental assistants and therapists	High Income and Upper Middle Income	Southern	Other Health Workers
11	Dental Therapist	206	226	247	0.97	10,345	Namibia	Base Estimate	3251 - Dental assistants and therapists	High Income and Upper Middle Income	Southern	Other Health Workers
12	Dentist	230	246	266	1.04	9,594	Namibia	Base Estimate	2261 - Dentists	High Income and Upper Middle Income	Southern	Dentist
13	Dermatologist	10	11	12	0.05	221,102	Namibia	Base Estimate	2212 - Specialist medical practitioners	High Income and Upper Middle Income	Southern	Medical Doctors
14	Endocrinologist	16	18	20	0.08	125,432	Namibia	Base Estimate	2212 - Specialist medical practitioners	High Income and Upper Middle Income	Southern	Medical Doctors
15	ENT Surgeon	36	40	44	0.17	58,156	Namibia	Base Estimate	2212 - Specialist medical practitioners	High Income and Upper Middle Income	Southern	Medical Doctors
16	Environmental Health Officer	126	134	143	0.56	17,920	Namibia	Base Estimate	2263 - Environmental and occupational health and hygiene professionals	High Income and Upper Middle Income	Southern	Other Health Workers
17	Gastroenterologist	20	20	20	0.08	128,162	Namibia	Base Estimate	2212 - Specialist medical practitioners	High Income and Upper Middle Income	Southern	Medical Doctors
18	General Medical Practitioner (Generalist Doctor)	1,292	1,549	1,841	6.89	1,451	Namibia	Base Estimate	2211 - Generalist medical practitioners	High Income and Upper Middle Income	Southern	Medical Doctors
19	General Surgeon	40	44	50	0.20	51,006	Namibia	Base Estimate	2212 - Specialist medical practitioners	High Income and Upper Middle Income	Southern	Medical Doctors
20	Haematologist	6	7	7	0.03	345,602	Namibia	Base Estimate	2212 - Specialist medical practitioners	High Income and Upper Middle Income	Southern	Medical Doctors
21	Health Promoter/Health Educator	58	80	106	0.38	26,104	Namibia	Base Estimate	2269 - Health professionals not elsewhere classified	High Income and Upper Middle Income	Southern	Other Health Workers
22	Infectious Diseases Specialist	4	4	5	0.02	558,548	Namibia	Base Estimate	2212 - Specialist medical practitioners	High Income and Upper Middle Income	Southern	Medical Doctors
23	Intensive Care Nurse	79	84	90	0.35	28,313	Namibia	Base Estimate	2221 - Nursing professionals	High Income and Upper Middle Income	Southern	Nursing Personnel
24	Medical Laboratory Scientist	436	580	751	2.78	3,601	Namibia	Base Estimate	3212 - Medical and pathology laboratory technicians	High Income and Upper Middle Income	Southern	Other Health Workers
25	Medical Laboratory Technician	473	1,026	1,651	5.46	1,830	Namibia	Base Estimate	3212 - Medical and pathology laboratory technicians	High Income and Upper Middle Income	Southern	Other Health Workers
26	Medical Social Worker	83	90	107	0.47	21,259	Namibia	Base Estimate	1344 - Social welfare managers	High Income and Upper Middle Income	Southern	Other Health Workers
27	Mental Health Nurse	170	183	200	0.79	12,695	Namibia	Base Estimate	2221 - Nursing professionals	High Income and Upper Middle Income	Southern	Nursing Personnel
28	Midwife	1,875	1,991	2,110	8.27	1,210	Namibia	Base Estimate	2222 - Midwifery professionals	High Income and Upper Middle Income	Southern	Midwifery Personnel
29	Nephrologist	40	46	53	0.21	48,144	Namibia	Base Estimate	2212 - Specialist medical practitioners	High Income and Upper Middle Income	Southern	Medical Doctors
30	Neuro-Surgeon	12	13	15	0.06	173,353	Namibia	Base Estimate	2212 - Specialist medical practitioners	High Income and Upper Middle Income	Southern	Medical Doctors
31	Nurse Anaesthetist	109	131	158	0.62	16,131	Namibia	Base Estimate	2221 - Nursing professionals	High Income and Upper Middle Income	Southern	Nursing Personnel
32	Nutritionist	296	301	306	1.20	8,329	Namibia	Base Estimate	2265 - Dieticians and nutritionists	High Income and Upper Middle Income	Southern	Other Health Workers
33	Obstetrician & Gynaecologist	224	248	286	1.17	8,541	Namibia	Base Estimate	2212 - Specialist medical practitioners	High Income and Upper Middle Income	Southern	Medical Doctors
34	Occupational Therapist	61	69	77	0.30	33,001	Namibia	Base Estimate	2269 - Health professionals not elsewhere classified	High Income and Upper Middle Income	Southern	Other Health Workers
35	Oncology Nurse	35	41	49	0.19	52,184	Namibia	Base Estimate	2221 - Nursing professionals	High Income and Upper Middle Income	Southern	Nursing Personnel
36	Operating Theatre Nurse	389	432	487	1.91	5,226	Namibia	Base Estimate	2221 - Nursing professionals	High Income and Upper Middle Income	Southern	Nursing Personnel
37	Ophthalmic Nurse	46	51	56	0.22	45,858	Namibia	Base Estimate	2221 - Nursing professionals	High Income and Upper Middle Income	Southern	Nursing Personnel
38	Ophthalmologist	16	18	20	0.08	126,731	Namibia	Base Estimate	2212 - Specialist medical practitioners	High Income and Upper Middle Income	Southern	Medical Doctors
39	Optometrist	85	95	106	0.41	24,249	Namibia	Base Estimate	2267 - Optometrists and ophthalmic opticians	High Income and Upper Middle Income	Southern	Other Health Workers
40	Orthopaedic Nurse	58	65	73	0.29	35,004	Namibia	Base Estimate	2221 - Nursing professionals	High Income and Upper Middle Income	Southern	Nursing Personnel
41	Orthopaedic Surgeon	123	141	163	0.64	15,696	Namibia	Base Estimate	2212 - Specialist medical practitioners	High Income and Upper Middle Income	Southern	Medical Doctors
42	Orthopaedic Technologist	80	87	94	0.37	27,125	Namibia	Base Estimate	3214 - Medical and dental prosthetic technicians	High Income and Upper Middle Income	Southern	Other Health Workers
43	Paediatric Nurse	237	246	252	0.99	10,125	Namibia	Base Estimate	2221 - Nursing professionals	High Income and Upper Middle Income	Southern	Nursing Personnel
44	Paediatric Surgeon	8	8	8	0.03	303,559	Namibia	Base Estimate	2212 - Specialist medical practitioners	High Income and Upper Middle Income	Southern	Medical Doctors
45	Paediatrician	95	104	113	0.44	22,531	Namibia	Base Estimate	2212 - Specialist medical practitioners	High Income and Upper Middle Income	Southern	Medical Doctors
46	Pathologist	32	36	40	0.16	63,823	Namibia	Base Estimate	2212 - Specialist medical practitioners	High Income and Upper Middle Income	Southern	Medical Doctors
47	Pharmacist	419	438	464	1.84	5,443	Namibia	Base Estimate	2262 - Pharmacists	High Income and Upper Middle Income	Southern	Pharmacist
48	Pharmacy Technician	369	395	428	1.69	5,932	Namibia	Base Estimate	3213 - Pharmaceutical technicians and assistants	High Income and Upper Middle Income	Southern	Other Health Workers
49	Physician	300	351	410	1.55	6,437	Namibia	Base Estimate	2212 - Specialist medical practitioners	High Income and Upper Middle Income	Southern	Medical Doctors
50	Physiotherapist	75	81	89	0.35	28,817	Namibia	Base Estimate	2264 - Physiotherapists	High Income and Upper Middle Income	Southern	Other Health Workers
51	Plastic Surgeon	25	27	30	0.12	86,523	Namibia	Base Estimate	2212 - Specialist medical practitioners	High Income and Upper Middle Income	Southern	Medical Doctors
52	Psychiatrist	149	160	173	0.68	14,683	Namibia	Base Estimate	2212 - Specialist medical practitioners	High Income and Upper Middle Income	Southern	Medical Doctors
53	Radiation Oncologist	13	16	19	0.08	130,644	Namibia	Base Estimate	2212 - Specialist medical practitioners	High Income and Upper Middle Income	Southern	Medical Doctors
54	Radiographer (Diagnostics and Therapy)	257	288	333	1.34	7,439	Namibia	Base Estimate	3211 - Medical imaging and therapeutic equipment technicians	High Income and Upper Middle Income	Southern	Other Health Workers
55	Radiologist	95	113	145	0.63	15,819	Namibia	Base Estimate	2212 - Specialist medical practitioners	High Income and Upper Middle Income	Southern	Medical Doctors
56	Registered General Nurse / State Certified Nurse	7,061	7,489	8,034	31.68	316	Namibia	Base Estimate	2221 - Nursing professionals	High Income and Upper Middle Income	Southern	Nursing Personnel
57	Renal Nurse	533	608	701	2.76	3,619	Namibia	Base Estimate	2221 - Nursing professionals	High Income and Upper Middle Income	Southern	Nursing Personnel
58	Respiratory Physician	16	17	19	0.07	134,561	Namibia	Base Estimate	2212 - Specialist medical practitioners	High Income and Upper Middle Income	Southern	Medical Doctors
59	Rheumatologist	10	10	11	0.04	222,961	Namibia	Base Estimate	2212 - Specialist medical practitioners	High Income and Upper Middle Income	Southern	Medical Doctors
60	Speech Therapist	27	29	31	0.12	81,537	Namibia	Base Estimate	2266 - Audiologists and speech therapists	High Income and Upper Middle Income	Southern	Other Health Workers
61	Urologist	5	6	8	0.03	324,247	Namibia	Base Estimate	2212 - Specialist medical practitioners	High Income and Upper Middle Income	Southern	Medical Doctors
1	Anaesthesiologist	191	223	260	0.11	93,788	Niger	Base Estimate	2212 - Specialist medical practitioners	Low Income	West	Medical Doctors
2	Associate Nurse/Enrolled Nurse/Nursing Assistant	28,518	32,773	37,686	15.47	646	Niger	Base Estimate	3221 - Nursing associate professionals	Low Income	West	Nursing Personnel
3	Audiologist	95	109	126	0.05	193,130	Niger	Base Estimate	2266 - Audiologists and speech therapists	Low Income	West	Other Health Workers

S/N	Health Professionals	2022	2026	2030	Density per 10,000 population	Required Population ratio (1 professional is to xxx population)	Name of Country	Modelling Scenario	ISCO-08 Match	Income Group Classification	Sub-Regional Grouping	SDG 3c Occupation
4	Cardiologist	140	165	193	0.08	126,457	Niger	Base Estimate	2212 - Specialist medical practitioners	Low Income	West	Medical Doctors
5	Cardiothoracic Surgeon	32	38	44	0.02	552,148	Niger	Base Estimate	2212 - Specialist medical practitioners	Low Income	West	Medical Doctors
6	Clinical Officer/Physician Assistant	3,096	3,279	3,503	1.44	6,921	Niger	Base Estimate	3256 - Medical assistants	Low Income	West	Other Health Workers
7	Clinical Pharmacist	663	764	889	0.37	27,291	Niger	Base Estimate	2262 - Pharmacists	Low Income	West	Pharmacist
8	Clinical Psychologist	1,253	1,433	1,650	0.68	14,733	Niger	Base Estimate	2634 - Psychologists	Low Income	West	Other Health Workers
9	Community health worker/Village health worker	28,556	31,581	34,927	14.33	698	Niger	Base Estimate	3253 - Community health workers	Low Income	West	Other Health Workers
10	Dental Surgery Assistant	2,269	2,620	3,039	1.25	8,009	Niger	Base Estimate	3251 - Dental assistants and therapists	Low Income	West	Other Health Workers
11	Dental Therapist	1,472	1,699	1,971	0.81	12,351	Niger	Base Estimate	3251 - Dental assistants and therapists	Low Income	West	Other Health Workers
12	Dentist	1,569	1,825	2,161	0.89	11,217	Niger	Base Estimate	2261 - Dentists	Low Income	West	Dentist
13	Dermatologist	86	100	117	0.05	208,820	Niger	Base Estimate	2212 - Specialist medical practitioners	Low Income	West	Medical Doctors
14	Endocrinologist	48	66	92	0.04	252,096	Niger	Base Estimate	2212 - Specialist medical practitioners	Low Income	West	Medical Doctors
15	ENT Surgeon	229	267	309	0.13	78,867	Niger	Base Estimate	2212 - Specialist medical practitioners	Low Income	West	Medical Doctors
16	Environmental Health Officer	1,202	1,392	1,608	0.66	15,165	Niger	Base Estimate	2263 - Environmental and occupational health and hygiene professionals	Low Income	West	Other Health Workers
17	Gastroenterologist	274	290	312	0.13	77,690	Niger	Base Estimate	2212 - Specialist medical practitioners	Low Income	West	Medical Doctors
18	General Medical Practitioner (Generalist Doctor)	9,537	10,857	12,383	5.09	1,966	Niger	Base Estimate	2211 - Generalist medical practitioners	Low Income	West	Medical Doctors
19	General Surgeon	204	240	286	0.12	84,510	Niger	Base Estimate	2212 - Specialist medical practitioners	Low Income	West	Medical Doctors
20	Haematologist	110	129	150	0.06	162,388	Niger	Base Estimate	2212 - Specialist medical practitioners	Low Income	West	Medical Doctors
21	Health Promoter/Health Educator	274	293	315	0.13	77,015	Niger	Base Estimate	2269 - Health professionals not elsewhere classified	Low Income	West	Other Health Workers
22	Infectious Diseases Specialist	27	31	35	0.01	695,814	Niger	Base Estimate	2212 - Specialist medical practitioners	Low Income	West	Medical Doctors
23	Intensive Care Nurse	856	989	1,140	0.47	21,382	Niger	Base Estimate	2221 - Nursing professionals	Low Income	West	Nursing Personnel
24	Medical Laboratory Scientist	2,620	2,975	3,414	1.41	7,093	Niger	Base Estimate	3212 - Medical and pathology laboratory technicians	Low Income	West	Other Health Workers
25	Medical Laboratory Technician	4,295	4,564	4,875	2.01	4,983	Niger	Base Estimate	3212 - Medical and pathology laboratory technicians	Low Income	West	Other Health Workers
26	Medical Social Worker	1,462	1,630	1,820	0.75	13,380	Niger	Base Estimate	1344 - Social welfare managers	Low Income	West	Other Health Workers
27	Mental Health Nurse	1,203	1,406	1,663	0.69	14,565	Niger	Base Estimate	2221 - Nursing professionals	Low Income	West	Nursing Personnel
28	Midwife	18,744	21,229	24,234	9.97	1,003	Niger	Base Estimate	2222 - Midwifery professionals	Low Income	West	Midwifery Personnel
29	Nephrologist	179	211	253	0.10	95,299	Niger	Base Estimate	2212 - Specialist medical practitioners	Low Income	West	Medical Doctors
30	Neuro-Surgeon	44	52	61	0.03	396,835	Niger	Base Estimate	2212 - Specialist medical practitioners	Low Income	West	Medical Doctors
31	Nurse Anaesthetist	334	398	473	0.19	51,447	Niger	Base Estimate	2221 - Nursing professionals	Low Income	West	Nursing Personnel
32	Nutritionist	6,531	7,378	8,309	3.40	2,939	Niger	Base Estimate	2265 - Dieticians and nutritionists	Low Income	West	Other Health Workers
33	Obstetrician & Gynaecologist	1,474	1,711	1,988	0.82	12,242	Niger	Base Estimate	2212 - Specialist medical practitioners	Low Income	West	Medical Doctors
34	Occupational Therapist	344	403	472	0.19	51,526	Niger	Base Estimate	2269 - Health professionals not elsewhere classified	Low Income	West	Other Health Workers
35	Oncology Nurse	124	147	176	0.07	137,713	Niger	Base Estimate	2221 - Nursing professionals	Low Income	West	Nursing Personnel
36	Operating Theatre Nurse	1,776	2,058	2,409	0.99	10,067	Niger	Base Estimate	2221 - Nursing professionals	Low Income	West	Nursing Personnel
37	Ophthalmic Nurse	261	315	385	0.16	62,509	Niger	Base Estimate	2221 - Nursing professionals	Low Income	West	Nursing Personnel
38	Ophthalmologist	77	92	112	0.05	215,342	Niger	Base Estimate	2212 - Specialist medical practitioners	Low Income	West	Medical Doctors
39	Optometrist	400	462	536	0.22	45,330	Niger	Base Estimate	2267 - Optometrists and ophthalmic opticians	Low Income	West	Other Health Workers
40	Orthopaedic Nurse	330	391	460	0.19	52,935	Niger	Base Estimate	2221 - Nursing professionals	Low Income	West	Nursing Personnel
41	Orthopaedic Surgeon	532	620	723	0.30	33,630	Niger	Base Estimate	2212 - Specialist medical practitioners	Low Income	West	Medical Doctors
42	Orthopaedic Technologist	659	766	889	0.36	27,420	Niger	Base Estimate	3214 - Medical and dental prosthetic technicians	Low Income	West	Other Health Workers
43	Paediatric Nurse	4,005	4,607	5,260	2.15	4,641	Niger	Base Estimate	2221 - Nursing professionals	Low Income	West	Nursing Personnel
44	Paediatric Surgeon	121	142	166	0.07	147,407	Niger	Base Estimate	2212 - Specialist medical practitioners	Low Income	West	Medical Doctors
45	Paediatrician	1,089	1,269	1,478	0.61	16,471	Niger	Base Estimate	2212 - Specialist medical practitioners	Low Income	West	Medical Doctors
46	Pathologist	127	143	163	0.07	148,400	Niger	Base Estimate	2212 - Specialist medical practitioners	Low Income	West	Medical Doctors
47	Pharmacist	1,584	1,718	1,875	0.77	12,943	Niger	Base Estimate	2262 - Pharmacists	Low Income	West	Pharmacist
48	Pharmacy Technician	2,320	2,665	3,070	1.26	7,924	Niger	Base Estimate	3213 - Pharmaceutical technicians and assistants	Low Income	West	Other Health Workers
49	Physician	2,689	3,090	3,569	1.47	6,804	Niger	Base Estimate	2212 - Specialist medical practitioners	Low Income	West	Medical Doctors
50	Physiotherapist	577	670	777	0.32	31,359	Niger	Base Estimate	2264 - Physiotherapists	Low Income	West	Other Health Workers
51	Plastic Surgeon	138	162	191	0.08	127,785	Niger	Base Estimate	2212 - Specialist medical practitioners	Low Income	West	Medical Doctors
52	Psychiatrist	1,419	1,637	1,905	0.78	12,752	Niger	Base Estimate	2212 - Specialist medical practitioners	Low Income	West	Medical Doctors
53	Radiation Oncologist	32	39	47	0.02	517,265	Niger	Base Estimate	2212 - Specialist medical practitioners	Low Income	West	Medical Doctors
54	Radiographer (Diagnostics and Therapy)	1,484	1,731	2,040	0.84	11,871	Niger	Base Estimate	3211 - Medical imaging and therapeutic equipment technicians	Low Income	West	Other Health Workers
55	Radiologist	545	639	758	0.31	31,874	Niger	Base Estimate	2212 - Specialist medical practitioners	Low Income	West	Medical Doctors
56	Registered General Nurse / State Certified Nurse	59,596	66,250	74,035	30.47	328	Niger	Base Estimate	2221 - Nursing professionals	Low Income	West	Nursing Personnel
57	Renal Nurse	2,288	2,701	3,241	1.34	7,451	Niger	Base Estimate	2221 - Nursing professionals	Low Income	West	Nursing Personnel
58	Respiratory Physician	174	202	235	0.10	103,656	Niger	Base Estimate	2212 - Specialist medical practitioners	Low Income	West	Medical Doctors
59	Rheumatologist	62	73	85	0.03	288,380	Niger	Base Estimate	2212 - Specialist medical practitioners	Low Income	West	Medical Doctors
60	Speech Therapist	237	276	320	0.13	76,158	Niger	Base Estimate	2266 - Audiologists and speech therapists	Low Income	West	Other Health Workers
61	Urologist	23	29	36	0.02	656,374	Niger	Base Estimate	2212 - Specialist medical practitioners	Low Income	West	Medical Doctors
1	Anaesthesiologist	2,019	2,268	2,539	0.12	81,857	Nigeria	Base Estimate	2212 - Specialist medical practitioners	Lower-middle Income	West	Medical Doctors
2	Associate Nurse/Enrolled Nurse/Nursing Assistant	265,381	287,904	312,097	15.03	665	Nigeria	Base Estimate	3221 - Nursing associate professionals	Lower-middle Income	West	Nursing Personnel
3	Audiologist	845	935	1,028	0.05	202,352	Nigeria	Base Estimate	2266 - Audiologists and speech therapists	Lower-middle Income	West	Other Health Workers
4	Cardiologist	1,594	1,787	1,995	0.10	104,263	Nigeria	Base Estimate	2212 - Specialist medical practitioners	Lower-middle Income	West	Medical Doctors
5	Cardiothoracic Surgeon	264	297	332	0.02	627,227	Nigeria	Base Estimate	2212 - Specialist medical practitioners	Lower-middle Income	West	Medical Doctors
6	Clinical Officer/Physician Assistant	39,403	40,583	41,952	2.03	4,926	Nigeria	Base Estimate	3256 - Medical assistants	Lower-middle Income	West	Other Health Workers
7	Clinical Pharmacist	6,703	7,329	8,055	0.39	25,740	Nigeria	Base Estimate	2262 - Pharmacists	Lower-middle Income	West	Pharmacist
8	Clinical Psychologist	12,243	13,457	14,873	0.72	13,940	Nigeria	Base Estimate	2634 - Psychologists	Lower-middle Income	West	Other Health Workers
9	Community health worker/Village health worker	202,202	213,016	224,230	10.82	924	Nigeria	Base Estimate	3253 - Community health workers	Lower-middle Income	West	Other Health Workers
10	Dental Surgery Assistant	20,050	22,268	24,725	1.19	8,404	Nigeria	Base Estimate	3251 - Dental assistants and therapists	Lower-middle Income	West	Other Health Workers
11	Dental Therapist	13,016	14,454	16,044	0.77	12,951	Nigeria	Base Estimate	3251 - Dental assistants and therapists	Lower-middle Income	West	Other Health Workers
12	Dentist	12,816	14,346	16,201	0.78	12,801	Nigeria	Base Estimate	2261 - Dentists	Lower-middle Income	West	Dentist
13	Dermatologist	793	871	953	0.05	218,206	Nigeria	Base Estimate	2212 - Specialist medical practitioners	Lower-middle Income	West	Medical Doctors
14	Endocrinologist	486	566	670	0.03	307,455	Nigeria	Base Estimate	2212 - Specialist medical practitioners	Lower-middle Income	West	Medical Doctors
15	ENT Surgeon	2,527	2,820	3,140	0.15	66,161	Nigeria	Base Estimate	2212 - Specialist medical practitioners	Lower-middle Income	West	Medical Doctors
16	Environmental Health Officer	10,239	11,240	12,290	0.59	16,926	Nigeria	Base Estimate	2263 - Environmental and occupational health and hygiene professionals	Lower-middle Income	West	Other Health Workers
17	Gastroenterologist	3,038	3,079	3,168	0.15	65,061	Nigeria	Base Estimate	2212 - Specialist medical practitioners	Lower-middle Income	West	Medical Doctors
18	General Medical Practitioner (Generalist Doctor)	94,866	103,227	112,263	5.41	1,849	Nigeria	Base Estimate	2211 - Generalist medical practitioners	Lower-middle Income	West	Medical Doctors
19	General Surgeon	1,668	1,840	2,048	0.10	101,216	Nigeria	Base Estimate	2212 - Specialist medical practitioners	Lower-middle Income	West	Medical Doctors
20	Haematologist	1,120	1,257	1,406	0.07	147,927	Nigeria	Base Estimate	2212 - Specialist medical practitioners	Lower-middle Income	West	Medical Doctors
21	Health Promoter/Health Educator	3,257	3,378	3,513	0.17	58,895	Nigeria	Base Estimate	2269 - Health professionals not elsewhere classified	Lower-middle Income	West	Other Health Workers
22	Infectious Diseases Specialist	230	249	270	0.01	766,022	Nigeria	Base Estimate	2212 - Specialist medical practitioners	Lower-middle Income	West	Medical Doctors
23	Intensive Care Nurse	6,546	7,134	7,753	0.37	26,810	Nigeria	Base Estimate	2221 - Nursing professionals	Lower-middle Income	West	Nursing Personnel
24	Medical Laboratory Scientist	25,118	27,038	29,310	1.42	7,063	Nigeria	Base Estimate	3212 - Medical and pathology laboratory technicians	Lower-middle Income	West	Other Health Workers
25	Medical Laboratory Technician	35,369	35,880	36,614	1.78	5,628	Nigeria	Base Estimate	3212 - Medical and pathology laboratory technicians	Lower-middle Income	West	Other Health Workers
26	Medical Social Worker	10,704	11,108	11,529	0.56	17,964	Nigeria	Base Estimate	1344 - Social welfare managers	Lower-middle Income	West	Other Health Workers

S/N	Health Professionals	2022	2026	2030	Density per 10,000 population	Required Population ratio (1 professional is to xxx population)	Name of Country	Modelling Scenario	ISCO-08 Match	Income Group Classification	Sub-Regional Grouping	SDG 3c Occupation
27	Mental Health Nurse	10,239	10,991	11,954	0.58	17,303	Nigeria	Base Estimate	2221 - Nursing professionals	Lower-middle Income	West	Nursing Personnel
28	Midwife	166,431	178,797	192,392	9.28	1,078	Nigeria	Base Estimate	2222 - Midwifery professionals	Lower-middle Income	West	Midwifery Personnel
29	Nephrologist	1,950	2,180	2,477	0.12	83,455	Nigeria	Base Estimate	2212 - Specialist medical practitioners	Lower-middle Income	West	Medical Doctors
30	Neuro-Surgeon	510	573	644	0.03	322,104	Nigeria	Base Estimate	2212 - Specialist medical practitioners	Lower-middle Income	West	Medical Doctors
31	Nurse Anaesthetist	3,988	4,528	5,132	0.25	40,487	Nigeria	Base Estimate	2221 - Nursing professionals	Lower-middle Income	West	Nursing Personnel
32	Nutritionist	38,929	41,125	43,260	2.08	4,800	Nigeria	Base Estimate	2265 - Dieticians and nutritionists	Lower-middle Income	West	Other Health Workers
33	Obstetrician & Gynaecologist	13,769	15,220	16,814	0.81	12,359	Nigeria	Base Estimate	2212 - Specialist medical practitioners	Lower-middle Income	West	Medical Doctors
34	Occupational Therapist	3,849	4,315	4,853	0.23	42,742	Nigeria	Base Estimate	2269 - Health professionals not elsewhere classified	Lower-middle Income	West	Other Health Workers
35	Oncology Nurse	1,434	1,611	1,829	0.09	113,140	Nigeria	Base Estimate	2221 - Nursing professionals	Lower-middle Income	West	Nursing Personnel
36	Operating Theatre Nurse	23,669	26,250	29,455	1.42	7,026	Nigeria	Base Estimate	2221 - Nursing professionals	Lower-middle Income	West	Nursing Personnel
37	Ophthalmic Nurse	2,555	2,827	3,133	0.15	66,235	Nigeria	Base Estimate	2221 - Nursing professionals	Lower-middle Income	West	Nursing Personnel
38	Ophthalmologist	774	861	961	0.05	215,726	Nigeria	Base Estimate	2212 - Specialist medical practitioners	Lower-middle Income	West	Medical Doctors
39	Optometrist	3,006	3,329	3,680	0.18	56,465	Nigeria	Base Estimate	2267 - Optometrists and ophthalmic opticians	Lower-middle Income	West	Other Health Workers
40	Orthopaedic Nurse	3,177	3,591	4,041	0.19	51,453	Nigeria	Base Estimate	2221 - Nursing professionals	Lower-middle Income	West	Nursing Personnel
41	Orthopaedic Surgeon	7,231	8,114	9,118	0.44	22,771	Nigeria	Base Estimate	2212 - Specialist medical practitioners	Lower-middle Income	West	Medical Doctors
42	Orthopaedic Technologist	5,786	6,405	7,063	0.34	29,447	Nigeria	Base Estimate	3214 - Medical and dental prosthetic technicians	Lower-middle Income	West	Other Health Workers
43	Paediatric Nurse	30,934	33,839	36,674	1.76	5,670	Nigeria	Base Estimate	2221 - Nursing professionals	Lower-middle Income	West	Nursing Personnel
44	Paediatric Surgeon	832	910	983	0.05	211,762	Nigeria	Base Estimate	2212 - Specialist medical practitioners	Lower-middle Income	West	Medical Doctors
45	Paediatrician	11,734	12,953	14,288	0.69	14,536	Nigeria	Base Estimate	2212 - Specialist medical practitioners	Lower-middle Income	West	Medical Doctors
46	Pathologist	1,256	1,397	1,577	0.08	131,065	Nigeria	Base Estimate	2212 - Specialist medical practitioners	Lower-middle Income	West	Medical Doctors
47	Pharmacist	25,765	26,837	27,926	1.35	7,420	Nigeria	Base Estimate	2262 - Pharmacists	Lower-middle Income	West	Pharmacist
48	Pharmacy Technician	26,808	29,234	31,867	1.53	6,517	Nigeria	Base Estimate	3213 - Pharmaceutical technicians and assistants	Lower-middle Income	West	Other Health Workers
49	Physician	25,646	28,029	30,704	1.48	6,757	Nigeria	Base Estimate	2212 - Specialist medical practitioners	Lower-middle Income	West	Medical Doctors
50	Physiotherapist	5,320	5,875	6,485	0.31	32,033	Nigeria	Base Estimate	2264 - Physiotherapists	Lower-middle Income	West	Other Health Workers
51	Plastic Surgeon	1,239	1,410	1,598	0.08	130,060	Nigeria	Base Estimate	2212 - Specialist medical practitioners	Lower-middle Income	West	Medical Doctors
52	Psychiatrist	11,288	12,134	13,166	0.64	15,727	Nigeria	Base Estimate	2212 - Specialist medical practitioners	Lower-middle Income	West	Medical Doctors
53	Radiation Oncologist	395	448	511	0.02	405,662	Nigeria	Base Estimate	2212 - Specialist medical practitioners	Lower-middle Income	West	Medical Doctors
54	Radiographer (Diagnostics and Therapy)	16,543	18,344	20,477	0.99	10,123	Nigeria	Base Estimate	3211 - Medical imaging and therapeutic equipment technicians	Lower-middle Income	West	Other Health Workers
55	Radiologist	6,006	6,637	7,360	0.35	28,192	Nigeria	Base Estimate	2212 - Specialist medical practitioners	Lower-middle Income	West	Medical Doctors
56	Registered General Nurse / State Certified Nurse	493,802	542,754	596,014	28.71	348	Nigeria	Base Estimate	2221 - Nursing professionals	Lower-middle Income	West	Nursing Personnel
57	Renal Nurse	25,402	28,412	32,289	1.56	6,401	Nigeria	Base Estimate	2221 - Nursing professionals	Lower-middle Income	West	Nursing Personnel
58	Respiratory Physician	1,822	1,989	2,166	0.10	95,964	Nigeria	Base Estimate	2212 - Specialist medical practitioners	Lower-middle Income	West	Medical Doctors
59	Rheumatologist	612	679	751	0.04	276,796	Nigeria	Base Estimate	2212 - Specialist medical practitioners	Lower-middle Income	West	Medical Doctors
60	Speech Therapist	2,079	2,295	2,528	0.12	82,167	Nigeria	Base Estimate	2266 - Audiologists and speech therapists	Lower-middle Income	West	Other Health Workers
61	Urologist	240	260	286	0.01	724,543	Nigeria	Base Estimate	2212 - Specialist medical practitioners	Lower-middle Income	West	Medical Doctors
1	Anaesthesiologist	168	191	218	0.17	59,864	Rwanda	Base Estimate	2212 - Specialist medical practitioners	Low Income	East	Medical Doctors
2	Associate Nurse/Enrolled Nurse/Nursing Assistant	14,273	16,286	19,078	14.30	699	Rwanda	Base Estimate	3221 - Nursing associate professionals	Low Income	East	Nursing Personnel
3	Audiologist	54	60	66	0.05	197,999	Rwanda	Base Estimate	2266 - Audiologists and speech therapists	Low Income	East	Other Health Workers
4	Cardiologist	116	138	164	0.13	79,454	Rwanda	Base Estimate	2212 - Specialist medical practitioners	Low Income	East	Medical Doctors
5	Cardiothoracic Surgeon	27	31	35	0.03	377,809	Rwanda	Base Estimate	2212 - Specialist medical practitioners	Low Income	East	Medical Doctors
6	Clinical Officer/Physician Assistant	2,657	3,650	5,530	3.78	2,649	Rwanda	Base Estimate	3256 - Medical assistants	Low Income	East	Other Health Workers
7	Clinical Pharmacist	392	433	487	0.38	26,260	Rwanda	Base Estimate	2262 - Pharmacists	Low Income	East	Pharmacist
8	Clinical Psychologist	867	930	1,001	0.77	13,017	Rwanda	Base Estimate	2634 - Psychologists	Low Income	East	Other Health Workers
9	Community health worker/Village health worker	13,339	19,295	30,179	20.33	492	Rwanda	Base Estimate	3253 - Community health workers	Low Income	East	Other Health Workers
10	Dental Surgery Assistant	1,488	1,685	1,913	1.46	6,830	Rwanda	Base Estimate	3251 - Dental assistants and therapists	Low Income	East	Other Health Workers
11	Dental Therapist	963	1,091	1,239	0.95	10,548	Rwanda	Base Estimate	3251 - Dental assistants and therapists	Low Income	East	Other Health Workers
12	Dentist	1,114	1,233	1,385	1.06	9,445	Rwanda	Base Estimate	2261 - Dentists	Low Income	East	Dentist
13	Dermatologist	48	53	59	0.05	219,890	Rwanda	Base Estimate	2212 - Specialist medical practitioners	Low Income	East	Medical Doctors
14	Endocrinologist	47	57	71	0.05	182,348	Rwanda	Base Estimate	2212 - Specialist medical practitioners	Low Income	East	Medical Doctors
15	ENT Surgeon	158	182	212	0.16	61,118	Rwanda	Base Estimate	2212 - Specialist medical practitioners	Low Income	East	Medical Doctors
16	Environmental Health Officer	643	703	764	0.58	17,104	Rwanda	Base Estimate	2263 - Environmental and occupational health and hygiene professionals	Low Income	East	Other Health Workers
17	Gastroenterologist	94	96	100	0.08	128,889	Rwanda	Base Estimate	2212 - Specialist medical practitioners	Low Income	East	Medical Doctors
18	General Medical Practitioner (Generalist Doctor)	5,282	6,249	7,760	5.72	1,748	Rwanda	Base Estimate	2211 - Generalist medical practitioners	Low Income	East	Medical Doctors
19	General Surgeon	115	139	170	0.13	75,728	Rwanda	Base Estimate	2212 - Specialist medical practitioners	Low Income	East	Medical Doctors
20	Haematologist	35	37	40	0.03	326,015	Rwanda	Base Estimate	2212 - Specialist medical practitioners	Low Income	East	Medical Doctors
21	Health Promoter/Health Educator	193	254	372	0.26	38,541	Rwanda	Base Estimate	2269 - Health professionals not elsewhere classified	Low Income	East	Other Health Workers
22	Infectious Diseases Specialist	13	14	15	0.01	841,412	Rwanda	Base Estimate	2212 - Specialist medical practitioners	Low Income	East	Medical Doctors
23	Intensive Care Nurse	368	404	442	0.34	29,568	Rwanda	Base Estimate	2221 - Nursing professionals	Low Income	East	Nursing Personnel
24	Medical Laboratory Scientist	1,727	2,207	3,030	2.19	4,569	Rwanda	Base Estimate	3212 - Medical and pathology laboratory technicians	Low Income	East	Other Health Workers
25	Medical Laboratory Technician	3,029	4,715	7,853	5.24	1,907	Rwanda	Base Estimate	3212 - Medical and pathology laboratory technicians	Low Income	East	Other Health Workers
26	Medical Social Worker	340	366	413	0.35	28,677	Rwanda	Base Estimate	1344 - Social welfare managers	Low Income	East	Other Health Workers
27	Mental Health Nurse	913	1,030	1,181	0.91	11,012	Rwanda	Base Estimate	2221 - Nursing professionals	Low Income	East	Nursing Personnel
28	Midwife	10,384	11,184	12,054	9.23	1,084	Rwanda	Base Estimate	2222 - Midwifery professionals	Low Income	East	Midwifery Personnel
29	Nephrologist	140	163	194	0.15	66,779	Rwanda	Base Estimate	2212 - Specialist medical practitioners	Low Income	East	Medical Doctors
30	Neuro-Surgeon	50	57	68	0.05	189,127	Rwanda	Base Estimate	2212 - Specialist medical practitioners	Low Income	East	Medical Doctors
31	Nurse Anaesthetist	382	457	546	0.42	23,818	Rwanda	Base Estimate	2221 - Nursing professionals	Low Income	East	Nursing Personnel
32	Nutritionist	1,098	1,134	1,162	0.89	11,211	Rwanda	Base Estimate	2265 - Dieticians and nutritionists	Low Income	East	Other Health Workers
33	Obstetrician & Gynaecologist	969	1,078	1,223	0.97	10,319	Rwanda	Base Estimate	2212 - Specialist medical practitioners	Low Income	East	Medical Doctors
34	Occupational Therapist	276	318	368	0.28	35,321	Rwanda	Base Estimate	2269 - Health professionals not elsewhere classified	Low Income	East	Other Health Workers
35	Oncology Nurse	120	143	172	0.13	75,182	Rwanda	Base Estimate	2221 - Nursing professionals	Low Income	East	Nursing Personnel
36	Operating Theatre Nurse	1,658	1,909	2,244	1.73	5,771	Rwanda	Base Estimate	2221 - Nursing professionals	Low Income	East	Nursing Personnel
37	Ophthalmic Nurse	143	164	189	0.15	68,707	Rwanda	Base Estimate	2221 - Nursing professionals	Low Income	East	Nursing Personnel
38	Ophthalmologist	52	61	72	0.06	181,256	Rwanda	Base Estimate	2212 - Specialist medical practitioners	Low Income	East	Medical Doctors
39	Optometrist	238	279	326	0.25	39,991	Rwanda	Base Estimate	2267 - Optometrists and ophthalmic opticians	Low Income	East	Other Health Workers
40	Orthopaedic Nurse	174	203	238	0.18	54,773	Rwanda	Base Estimate	2221 - Nursing professionals	Low Income	East	Nursing Personnel
41	Orthopaedic Surgeon	379	498	671	0.54	18,631	Rwanda	Base Estimate	2212 - Specialist medical practitioners	Low Income	East	Medical Doctors
42	Orthopaedic Technologist	376	417	461	0.35	28,311	Rwanda	Base Estimate	3214 - Medical and dental prosthetic technicians	Low Income	East	Other Health Workers
43	Paediatric Nurse	1,321	1,392	1,436	1.10	9,093	Rwanda	Base Estimate	2221 - Nursing professionals	Low Income	East	Nursing Personnel
44	Paediatric Surgeon	42	45	47	0.04	280,389	Rwanda	Base Estimate	2212 - Specialist medical practitioners	Low Income	East	Medical Doctors
45	Paediatrician	863	900	939	0.72	13,842	Rwanda	Base Estimate	2212 - Specialist medical practitioners	Low Income	East	Medical Doctors
46	Pathologist	120	134	152	0.12	85,664	Rwanda	Base Estimate	2212 - Specialist medical practitioners	Low Income	East	Medical Doctors
47	Pharmacist	1,198	1,205	1,234	0.97	10,345	Rwanda	Base Estimate	2262 - Pharmacists	Low Income	East	Pharmacist
48	Pharmacy Technician	1,347	1,464	1,604	1.24	8,066	Rwanda	Base Estimate	3213 - Pharmaceutical technicians and assistants	Low Income	East	Other Health Workers
49	Physician	1,400	1,623	1,941	1.45	6,897	Rwanda	Base Estimate	2212 - Specialist medical practitioners	Low Income	East	Medical Doctors

S/N	Health Professionals	2022	2026	2030	Density per 10,000 population	Required Population ratio (1 professional is to xxx population)	Name of Country	Modelling Scenario	ISCO-08 Match	Income Group Classification	Sub-Regional Grouping	SDG 3c Occupation
50	Physiotherapist	348	388	435	0.33	29,891	Rwanda	Base Estimate	2264 - Physiotherapists	Low Income	East	Other Health Workers
51	Plastic Surgeon	208	240	275	0.21	47,531	Rwanda	Base Estimate	2212 - Specialist medical practitioners	Low Income	East	Medical Doctors
52	Psychiatrist	756	843	955	0.73	13,635	Rwanda	Base Estimate	2212 - Specialist medical practitioners	Low Income	East	Medical Doctors
53	Radiation Oncologist	45	56	69	0.05	186,426	Rwanda	Base Estimate	2212 - Specialist medical practitioners	Low Income	East	Medical Doctors
54	Radiographer (Diagnostics and Therapy)	949	1,103	1,317	1.04	9,612	Rwanda	Base Estimate	3211 - Medical imaging and therapeutic equipment technicians	Low Income	East	Other Health Workers
55	Radiologist	317	384	497	0.42	23,574	Rwanda	Base Estimate	2212 - Specialist medical practitioners	Low Income	East	Medical Doctors
56	Registered General Nurse / State Certified Nurse	28,402	31,317	34,731	26.76	374	Rwanda	Base Estimate	2221 - Nursing professionals	Low Income	East	Nursing Personnel
57	Renal Nurse	1,867	2,180	2,593	2.00	4,998	Rwanda	Base Estimate	2221 - Nursing professionals	Low Income	East	Nursing Personnel
58	Respiratory Physician	216	221	225	0.17	57,765	Rwanda	Base Estimate	2212 - Specialist medical practitioners	Low Income	East	Medical Doctors
59	Rheumatologist	42	48	55	0.04	237,698	Rwanda	Base Estimate	2212 - Specialist medical practitioners	Low Income	East	Medical Doctors
60	Speech Therapist	136	150	169	0.13	76,828	Rwanda	Base Estimate	2266 - Audiologists and speech therapists	Low Income	East	Other Health Workers
61	Urologist	17	22	28	0.02	453,346	Rwanda	Base Estimate	2212 - Specialist medical practitioners	Low Income	East	Medical Doctors
1	Anaesthesiologist	3	3	4	0.18	57,049	Sao Tome and Princ	Base Estimate	2212 - Specialist medical practitioners	Lower-middle Income	West	Medical Doctors
2	Associate Nurse/Enrolled Nurse/Nursing Assistant	272	299	330	15.01	666	Sao Tome and Princ	Base Estimate	3221 - Nursing associate professionals	Lower-middle Income	West	Nursing Personnel
3	Audiologist	1	1	1	0.05	209,673	Sao Tome and Princ	Base Estimate	2266 - Audiologists and speech therapists	Lower-middle Income	West	Other Health Workers
4	Cardiologist	2	3	3	0.14	73,967	Sao Tome and Princ	Base Estimate	2212 - Specialist medical practitioners	Lower-middle Income	West	Medical Doctors
5	Cardiothoracic Surgeon	0	1	1	0.03	370,355	Sao Tome and Princ	Base Estimate	2212 - Specialist medical practitioners	Lower-middle Income	West	Medical Doctors
6	Clinical Officer/Physician Assistant	22	23	24	1.11	9,000	Sao Tome and Princ	Base Estimate	3256 - Medical assistants	Lower-middle Income	West	Other Health Workers
7	Clinical Pharmacist	8	8	9	0.43	23,292	Sao Tome and Princ	Base Estimate	2262 - Pharmacists	Lower-middle Income	West	Pharmacist
8	Clinical Psychologist	17	19	21	0.94	10,600	Sao Tome and Princ	Base Estimate	2634 - Psychologists	Lower-middle Income	West	Other Health Workers
9	Community health worker/Village health worker	133	138	144	6.54	1,530	Sao Tome and Princ	Base Estimate	3253 - Community health workers	Lower-middle Income	West	Other Health Workers
10	Dental Surgery Assistant	30	33	38	1.71	5,861	Sao Tome and Princ	Base Estimate	3251 - Dental assistants and therapists	Lower-middle Income	West	Other Health Workers
11	Dental Therapist	19	22	24	1.11	9,034	Sao Tome and Princ	Base Estimate	3251 - Dental assistants and therapists	Lower-middle Income	West	Other Health Workers
12	Dentist	18	20	22	1.00	9,971	Sao Tome and Princ	Base Estimate	2261 - Dentists	Lower-middle Income	West	Dentist
13	Dermatologist	1	1	1	0.05	200,406	Sao Tome and Princ	Base Estimate	2212 - Specialist medical practitioners	Lower-middle Income	West	Medical Doctors
14	Endocrinologist	1	1	1	0.06	154,389	Sao Tome and Princ	Base Estimate	2212 - Specialist medical practitioners	Lower-middle Income	West	Medical Doctors
15	ENT Surgeon	3	3	3	0.16	63,468	Sao Tome and Princ	Base Estimate	2212 - Specialist medical practitioners	Lower-middle Income	West	Medical Doctors
16	Environmental Health Officer	11	12	13	0.57	17,409	Sao Tome and Princ	Base Estimate	2263 - Environmental and occupational health and hygiene professionals	Lower-middle Income	West	Other Health Workers
17	Gastroenterologist	3	3	3	0.12	84,368	Sao Tome and Princ	Base Estimate	2212 - Specialist medical practitioners	Lower-middle Income	West	Medical Doctors
18	General Medical Practitioner (Generalist Doctor)	88	96	106	4.81	2,078	Sao Tome and Princ	Base Estimate	2211 - Generalist medical practitioners	Lower-middle Income	West	Medical Doctors
19	General Surgeon	2	3	3	0.15	68,789	Sao Tome and Princ	Base Estimate	2212 - Specialist medical practitioners	Lower-middle Income	West	Medical Doctors
20	Haematologist	1	1	1	0.05	220,628	Sao Tome and Princ	Base Estimate	2212 - Specialist medical practitioners	Lower-middle Income	West	Medical Doctors
21	Health Promoter/Health Educator	2	2	2	0.09	110,434	Sao Tome and Princ	Base Estimate	2269 - Health professionals not elsewhere classified	Lower-middle Income	West	Other Health Workers
22	Infectious Diseases Specialist	0	0	0	0.02	629,138	Sao Tome and Princ	Base Estimate	2212 - Specialist medical practitioners	Lower-middle Income	West	Medical Doctors
23	Intensive Care Nurse	6	6	7	0.31	32,029	Sao Tome and Princ	Base Estimate	2221 - Nursing professionals	Lower-middle Income	West	Nursing Personnel
24	Medical Laboratory Scientist	25	27	30	1.38	7,265	Sao Tome and Princ	Base Estimate	3212 - Medical and pathology laboratory technicians	Lower-middle Income	West	Other Health Workers
25	Medical Laboratory Technician	34	44	58	2.78	3,602	Sao Tome and Princ	Base Estimate	3212 - Medical and pathology laboratory technicians	Lower-middle Income	West	Other Health Workers
26	Medical Social Worker	7	7	7	0.34	29,484	Sao Tome and Princ	Base Estimate	1344 - Social welfare managers	Lower-middle Income	West	Other Health Workers
27	Mental Health Nurse	13	14	16	0.73	13,775	Sao Tome and Princ	Base Estimate	2221 - Nursing professionals	Lower-middle Income	West	Nursing Personnel
28	Midwife	203	244	302	14.10	709	Sao Tome and Princ	Base Estimate	2222 - Midwifery professionals	Lower-middle Income	West	Midwifery Personnel
29	Nephrologist	3	4	4	0.19	52,416	Sao Tome and Princ	Base Estimate	2212 - Specialist medical practitioners	Lower-middle Income	West	Medical Doctors
30	Neuro-Surgeon	1	1	2	0.07	143,195	Sao Tome and Princ	Base Estimate	2212 - Specialist medical practitioners	Lower-middle Income	West	Medical Doctors
31	Nurse Anaesthetist	8	9	11	0.48	20,851	Sao Tome and Princ	Base Estimate	2221 - Nursing professionals	Lower-middle Income	West	Nursing Personnel
32	Nutritionist	21	20	20	0.92	10,837	Sao Tome and Princ	Base Estimate	2265 - Dieticians and nutritionists	Lower-middle Income	West	Other Health Workers
33	Obstetrician & Gynaecologist	16	18	20	0.90	11,131	Sao Tome and Princ	Base Estimate	2212 - Specialist medical practitioners	Lower-middle Income	West	Medical Doctors
34	Occupational Therapist	6	7	8	0.36	27,721	Sao Tome and Princ	Base Estimate	2269 - Health professionals not elsewhere classified	Lower-middle Income	West	Other Health Workers
35	Oncology Nurse	2	3	3	0.16	63,887	Sao Tome and Princ	Base Estimate	2221 - Nursing professionals	Lower-middle Income	West	Nursing Personnel
36	Operating Theatre Nurse	27	31	36	1.67	5,988	Sao Tome and Princ	Base Estimate	2221 - Nursing professionals	Lower-middle Income	West	Nursing Personnel
37	Ophthalmic Nurse	3	4	4	0.19	52,389	Sao Tome and Princ	Base Estimate	2221 - Nursing professionals	Lower-middle Income	West	Nursing Personnel
38	Ophthalmologist	1	1	1	0.06	171,805	Sao Tome and Princ	Base Estimate	2212 - Specialist medical practitioners	Lower-middle Income	West	Medical Doctors
39	Optometrist	4	4	5	0.23	43,906	Sao Tome and Princ	Base Estimate	2267 - Optometrists and ophthalmic opticians	Lower-middle Income	West	Other Health Workers
40	Orthopaedic Nurse	6	7	8	0.38	26,552	Sao Tome and Princ	Base Estimate	2221 - Nursing professionals	Lower-middle Income	West	Nursing Personnel
41	Orthopaedic Surgeon	9	11	13	0.59	16,816	Sao Tome and Princ	Base Estimate	2212 - Specialist medical practitioners	Lower-middle Income	West	Medical Doctors
42	Orthopaedic Technologist	7	8	9	0.41	24,194	Sao Tome and Princ	Base Estimate	3214 - Medical and dental prosthetic technicians	Lower-middle Income	West	Other Health Workers
43	Paediatric Nurse	24	25	26	1.18	8,454	Sao Tome and Princ	Base Estimate	2221 - Nursing professionals	Lower-middle Income	West	Nursing Personnel
44	Paediatric Surgeon	1	1	1	0.03	327,821	Sao Tome and Princ	Base Estimate	2212 - Specialist medical practitioners	Lower-middle Income	West	Medical Doctors
45	Paediatrician	12	13	14	0.63	15,993	Sao Tome and Princ	Base Estimate	2212 - Specialist medical practitioners	Lower-middle Income	West	Medical Doctors
46	Pathologist	2	2	2	0.11	95,033	Sao Tome and Princ	Base Estimate	2212 - Specialist medical practitioners	Lower-middle Income	West	Medical Doctors
47	Pharmacist	15	16	16	0.74	13,479	Sao Tome and Princ	Base Estimate	2262 - Pharmacists	Lower-middle Income	West	Pharmacist
48	Pharmacy Technician	26	29	32	1.44	6,928	Sao Tome and Princ	Base Estimate	3213 - Pharmaceutical technicians and assistants	Lower-middle Income	West	Other Health Workers
49	Physician	30	34	40	1.83	5,478	Sao Tome and Princ	Base Estimate	2212 - Specialist medical practitioners	Lower-middle Income	West	Medical Doctors
50	Physiotherapist	7	7	8	0.37	26,833	Sao Tome and Princ	Base Estimate	2264 - Physiotherapists	Lower-middle Income	West	Other Health Workers
51	Plastic Surgeon	2	2	3	0.13	75,644	Sao Tome and Princ	Base Estimate	2212 - Specialist medical practitioners	Lower-middle Income	West	Medical Doctors
52	Psychiatrist	12	13	15	0.66	15,079	Sao Tome and Princ	Base Estimate	2212 - Specialist medical practitioners	Lower-middle Income	West	Medical Doctors
53	Radiation Oncologist	1	1	1	0.06	155,750	Sao Tome and Princ	Base Estimate	2212 - Specialist medical practitioners	Lower-middle Income	West	Medical Doctors
54	Radiographer (Diagnostics and Therapy)	21	24	28	1.26	7,956	Sao Tome and Princ	Base Estimate	3211 - Medical imaging and therapeutic equipment technicians	Lower-middle Income	West	Other Health Workers
55	Radiologist	7	8	10	0.44	22,550	Sao Tome and Princ	Base Estimate	2212 - Specialist medical practitioners	Lower-middle Income	West	Medical Doctors
56	Registered General Nurse / State Certified Nurse	558	622	704	32.14	311	Sao Tome and Princ	Base Estimate	2221 - Nursing professionals	Lower-middle Income	West	Nursing Personnel
57	Renal Nurse	41	47	55	2.52	3,974	Sao Tome and Princ	Base Estimate	2221 - Nursing professionals	Lower-middle Income	West	Nursing Personnel
58	Respiratory Physician	2	3	3	0.13	78,416	Sao Tome and Princ	Base Estimate	2212 - Specialist medical practitioners	Lower-middle Income	West	Medical Doctors
59	Rheumatologist	1	1	1	0.04	242,916	Sao Tome and Princ	Base Estimate	2212 - Specialist medical practitioners	Lower-middle Income	West	Medical Doctors
60	Speech Therapist	2	3	3	0.14	74,006	Sao Tome and Princ	Base Estimate	2266 - Audiologists and speech therapists	Lower-middle Income	West	Other Health Workers
61	Urologist	0	0	1	0.02	418,629	Sao Tome and Princ	Base Estimate	2212 - Specialist medical practitioners	Lower-middle Income	West	Medical Doctors
1	Anaesthesiologist	186	213	244	0.14	69,111	Senegal	Base Estimate	2212 - Specialist medical practitioners	Lower-middle Income	West	Medical Doctors
2	Associate Nurse/Enrolled Nurse/Nursing Assistant	23,461	27,129	31,492	18.77	533	Senegal	Base Estimate	3221 - Nursing associate professionals	Lower-middle Income	West	Nursing Personnel
3	Audiologist	66	74	82	0.05	204,549	Senegal	Base Estimate	2266 - Audiologists and speech therapists	Lower-middle Income	West	Other Health Workers
4	Cardiologist	140	159	179	0.11	94,027	Senegal	Base Estimate	2212 - Specialist medical practitioners	Lower-middle Income	West	Medical Doctors
5	Cardiothoracic Surgeon	28	32	35	0.02	475,059	Senegal	Base Estimate	2212 - Specialist medical practitioners	Lower-middle Income	West	Medical Doctors
6	Clinical Officer/Physician Assistant	1,754	1,830	1,937	1.16	8,635	Senegal	Base Estimate	3256 - Medical assistants	Lower-middle Income	West	Other Health Workers
7	Clinical Pharmacist	504	562	633	0.38	26,518	Senegal	Base Estimate	2262 - Pharmacists	Lower-middle Income	West	Pharmacist
8	Clinical Psychologist	974	1,082	1,210	0.72	13,898	Senegal	Base Estimate	2634 - Psychologists	Lower-middle Income	West	Other Health Workers
9	Community health worker/Village health worker	13,142	14,053	14,985	8.90	1,124	Senegal	Base Estimate	3253 - Community health workers	Lower-middle Income	West	Other Health Workers
10	Dental Surgery Assistant	2,134	2,374	2,661	1.58	6,326	Senegal	Base Estimate	3251 - Dental assistants and therapists	Lower-middle Income	West	Other Health Workers
11	Dental Therapist	1,381	1,537	1,722	1.02	9,774	Senegal	Base Estimate	3251 - Dental assistants and therapists	Lower-middle Income	West	Other Health Workers

S/N	Health Professionals	2022	2026	2030	Density per 10,000 population	Required Population ratio (1 professional is to xxx population)	Name of Country	Modelling Scenario	ISCO-08 Match	Income Group Classification	Sub-Regional Grouping	SDG 3c Occupation
12	Dentist	1,564	1,717	1,923	1.14	8,736	Senegal	Base Estimate	2261 - Dentists	Lower-middle Income	West	Dentist
13	Dermatologist	66	74	82	0.05	205,827	Senegal	Base Estimate	2212 - Specialist medical practitioners	Lower-middle Income	West	Medical Doctors
14	Endocrinologist	81	92	106	0.06	157,263	Senegal	Base Estimate	2212 - Specialist medical practitioners	Lower-middle Income	West	Medical Doctors
15	ENT Surgeon	193	218	247	0.15	68,155	Senegal	Base Estimate	2212 - Specialist medical practitioners	Lower-middle Income	West	Medical Doctors
16	Environmental Health Officer	832	919	1,013	0.60	16,666	Senegal	Base Estimate	2263 - Environmental and occupational health and hygiene professionals	Lower-middle Income	West	Other Health Workers
17	Gastroenterologist	156	158	163	0.10	102,011	Senegal	Base Estimate	2212 - Specialist medical practitioners	Lower-middle Income	West	Medical Doctors
18	General Medical Practitioner (Generalist Doctor)	6,590	7,260	8,008	4.76	2,103	Senegal	Base Estimate	2211 - Generalist medical practitioners	Lower-middle Income	West	Medical Doctors
19	General Surgeon	186	213	247	0.15	67,875	Senegal	Base Estimate	2212 - Specialist medical practitioners	Lower-middle Income	West	Medical Doctors
20	Haematologist	85	94	104	0.06	161,674	Senegal	Base Estimate	2212 - Specialist medical practitioners	Lower-middle Income	West	Medical Doctors
21	Health Promoter/Health Educator	158	165	174	0.10	95,985	Senegal	Base Estimate	2269 - Health professionals not elsewhere classified	Lower-middle Income	West	Other Health Workers
22	Infectious Diseases Specialist	16	17	19	0.01	875,661	Senegal	Base Estimate	2212 - Specialist medical practitioners	Lower-middle Income	West	Medical Doctors
23	Intensive Care Nurse	484	538	596	0.35	28,275	Senegal	Base Estimate	2221 - Nursing professionals	Lower-middle Income	West	Nursing Personnel
24	Medical Laboratory Scientist	1,834	2,020	2,251	1.34	7,455	Senegal	Base Estimate	3212 - Medical and pathology laboratory technicians	Lower-middle Income	West	Other Health Workers
25	Medical Laboratory Technician	2,023	2,162	2,327	1.39	7,215	Senegal	Base Estimate	3212 - Medical and pathology laboratory technicians	Lower-middle Income	West	Other Health Workers
26	Medical Social Worker	578	604	634	0.38	26,412	Senegal	Base Estimate	1344 - Social welfare managers	Lower-middle Income	West	Other Health Workers
27	Mental Health Nurse	885	994	1,142	0.68	14,647	Senegal	Base Estimate	2221 - Nursing professionals	Lower-middle Income	West	Nursing Personnel
28	Midwife	14,250	15,536	16,985	10.09	991	Senegal	Base Estimate	2222 - Midwifery professionals	Lower-middle Income	West	Midwifery Personnel
29	Nephrologist	173	198	231	0.14	72,398	Senegal	Base Estimate	2212 - Specialist medical practitioners	Lower-middle Income	West	Medical Doctors
30	Neuro-Surgeon	51	59	69	0.04	241,648	Senegal	Base Estimate	2212 - Specialist medical practitioners	Lower-middle Income	West	Medical Doctors
31	Nurse Anaesthetist	408	481	569	0.34	29,522	Senegal	Base Estimate	2221 - Nursing professionals	Lower-middle Income	West	Nursing Personnel
32	Nutritionist	2,151	2,265	2,375	1.41	7,092	Senegal	Base Estimate	2265 - Dieticians and nutritionists	Lower-middle Income	West	Other Health Workers
33	Obstetrician & Gynaecologist	1,146	1,280	1,433	0.85	11,755	Senegal	Base Estimate	2212 - Specialist medical practitioners	Lower-middle Income	West	Medical Doctors
34	Occupational Therapist	395	459	536	0.32	31,333	Senegal	Base Estimate	2269 - Health professionals not elsewhere classified	Lower-middle Income	West	Other Health Workers
35	Oncology Nurse	136	158	186	0.11	89,699	Senegal	Base Estimate	2221 - Nursing professionals	Lower-middle Income	West	Nursing Personnel
36	Operating Theatre Nurse	1,812	2,071	2,412	1.44	6,938	Senegal	Base Estimate	2221 - Nursing professionals	Lower-middle Income	West	Nursing Personnel
37	Ophthalmic Nurse	239	267	301	0.18	55,927	Senegal	Base Estimate	2221 - Nursing professionals	Lower-middle Income	West	Nursing Personnel
38	Ophthalmologist	71	80	91	0.05	183,662	Senegal	Base Estimate	2212 - Specialist medical practitioners	Lower-middle Income	West	Medical Doctors
39	Optometrist	285	322	363	0.22	46,400	Senegal	Base Estimate	2267 - Optometrists and ophthalmic opticians	Lower-middle Income	West	Other Health Workers
40	Orthopaedic Nurse	431	505	590	0.35	28,520	Senegal	Base Estimate	2221 - Nursing professionals	Lower-middle Income	West	Nursing Personnel
41	Orthopaedic Surgeon	604	699	812	0.48	20,702	Senegal	Base Estimate	2212 - Specialist medical practitioners	Lower-middle Income	West	Medical Doctors
42	Orthopaedic Technologist	541	608	683	0.40	24,707	Senegal	Base Estimate	3214 - Medical and dental prosthetic technicians	Lower-middle Income	West	Other Health Workers
43	Paediatric Nurse	2,153	2,351	2,538	1.50	6,645	Senegal	Base Estimate	2221 - Nursing professionals	Lower-middle Income	West	Nursing Personnel
44	Paediatric Surgeon	57	61	65	0.04	259,612	Senegal	Base Estimate	2212 - Specialist medical practitioners	Lower-middle Income	West	Medical Doctors
45	Paediatrician	714	809	919	0.55	18,305	Senegal	Base Estimate	2212 - Specialist medical practitioners	Lower-middle Income	West	Medical Doctors
46	Pathologist	101	115	132	0.08	126,285	Senegal	Base Estimate	2212 - Specialist medical practitioners	Lower-middle Income	West	Medical Doctors
47	Pharmacist	1,179	1,221	1,274	0.76	13,139	Senegal	Base Estimate	2262 - Pharmacists	Lower-middle Income	West	Pharmacist
48	Pharmacy Technician	1,830	2,031	2,260	1.34	7,447	Senegal	Base Estimate	3213 - Pharmaceutical technicians and assistants	Lower-middle Income	West	Other Health Workers
49	Physician	1,964	2,182	2,436	1.45	6,908	Senegal	Base Estimate	2212 - Specialist medical practitioners	Lower-middle Income	West	Medical Doctors
50	Physiotherapist	474	533	600	0.36	28,080	Senegal	Base Estimate	2264 - Physiotherapists	Lower-middle Income	West	Other Health Workers
51	Plastic Surgeon	125	144	165	0.10	102,001	Senegal	Base Estimate	2212 - Specialist medical practitioners	Lower-middle Income	West	Medical Doctors
52	Psychiatrist	887	987	1,119	0.67	14,973	Senegal	Base Estimate	2212 - Specialist medical practitioners	Lower-middle Income	West	Medical Doctors
53	Radiation Oncologist	45	54	66	0.04	255,294	Senegal	Base Estimate	2212 - Specialist medical practitioners	Lower-middle Income	West	Medical Doctors
54	Radiographer (Diagnostics and Therapy)	1,365	1,537	1,752	1.04	9,576	Senegal	Base Estimate	3211 - Medical imaging and therapeutic equipment technicians	Lower-middle Income	West	Other Health Workers
55	Radiologist	494	560	640	0.38	26,217	Senegal	Base Estimate	2212 - Specialist medical practitioners	Lower-middle Income	West	Medical Doctors
56	Registered General Nurse / State Certified Nurse	53,822	59,714	66,680	39.77	251	Senegal	Base Estimate	2221 - Nursing professionals	Lower-middle Income	West	Nursing Personnel
57	Renal Nurse	2,295	2,617	3,057	1.83	5,463	Senegal	Base Estimate	2221 - Nursing professionals	Lower-middle Income	West	Nursing Personnel
58	Respiratory Physician	116	134	155	0.09	108,393	Senegal	Base Estimate	2212 - Specialist medical practitioners	Lower-middle Income	West	Medical Doctors
59	Rheumatologist	52	58	65	0.04	257,495	Senegal	Base Estimate	2212 - Specialist medical practitioners	Lower-middle Income	West	Medical Doctors
60	Speech Therapist	175	194	215	0.13	78,286	Senegal	Base Estimate	2266 - Audiologists and speech therapists	Lower-middle Income	West	Other Health Workers
61	Urologist	28	33	41	0.02	409,414	Senegal	Base Estimate	2212 - Specialist medical practitioners	Lower-middle Income	West	Medical Doctors
1	Anaesthesiologist	2	3	3	0.31	32,173	Seychelles	Base Estimate	2212 - Specialist medical practitioners	High Income and Upper Middle Income	Southern	Medical Doctors
2	Associate Nurse/Enrolled Nurse/Nursing Assistant	147	155	164	16.85	593	Seychelles	Base Estimate	3221 - Nursing associate professionals	High Income and Upper Middle Income	Southern	Nursing Personnel
3	Audiologist	0	0	0	0.04	241,574	Seychelles	Base Estimate	2266 - Audiologists and speech therapists	High Income and Upper Middle Income	Southern	Other Health Workers
4	Cardiologist	2	3	3	0.29	34,047	Seychelles	Base Estimate	2212 - Specialist medical practitioners	High Income and Upper Middle Income	Southern	Medical Doctors
5	Cardiothoracic Surgeon	0	0	0	0.03	293,543	Seychelles	Base Estimate	2212 - Specialist medical practitioners	High Income and Upper Middle Income	Southern	Medical Doctors
6	Clinical Officer/Physician Assistant	21	23	24	2.46	4,059	Seychelles	Base Estimate	3256 - Medical assistants	High Income and Upper Middle Income	Southern	Other Health Workers
7	Clinical Pharmacist	6	6	7	0.66	15,141	Seychelles	Base Estimate	2262 - Pharmacists	High Income and Upper Middle Income	Southern	Pharmacist
8	Clinical Psychologist	7	7	7	0.72	13,896	Seychelles	Base Estimate	2634 - Psychologists	High Income and Upper Middle Income	Southern	Other Health Workers
9	Community health worker/Village health worker	64	66	67	6.74	1,484	Seychelles	Base Estimate	3253 - Community health workers	High Income and Upper Middle Income	Southern	Other Health Workers
10	Dental Surgery Assistant	9	9	9	0.94	10,636	Seychelles	Base Estimate	3251 - Dental assistants and therapists	High Income and Upper Middle Income	Southern	Other Health Workers
11	Dental Therapist	6	6	6	0.61	16,396	Seychelles	Base Estimate	3251 - Dental assistants and therapists	High Income and Upper Middle Income	Southern	Other Health Workers
12	Dentist	7	7	7	0.68	14,603	Seychelles	Base Estimate	2261 - Dentists	High Income and Upper Middle Income	Southern	Dentist
13	Dermatologist	0	0	0	0.04	240,257	Seychelles	Base Estimate	2212 - Specialist medical practitioners	High Income and Upper Middle Income	Southern	Medical Doctors
14	Endocrinologist	3	4	4	0.43	23,475	Seychelles	Base Estimate	2212 - Specialist medical practitioners	High Income and Upper Middle Income	Southern	Medical Doctors
15	ENT Surgeon	2	2	3	0.27	36,968	Seychelles	Base Estimate	2212 - Specialist medical practitioners	High Income and Upper Middle Income	Southern	Medical Doctors
16	Environmental Health Officer	5	5	5	0.51	19,451	Seychelles	Base Estimate	2263 - Environmental and occupational health and hygiene professionals	High Income and Upper Middle Income	Southern	Other Health Workers
17	Gastroenterologist	1	1	1	0.10	104,978	Seychelles	Base Estimate	2212 - Specialist medical practitioners	High Income and Upper Middle Income	Southern	Medical Doctors
18	General Medical Practitioner (Generalist Doctor)	56	60	64	6.51	1,537	Seychelles	Base Estimate	2211 - Generalist medical practitioners	High Income and Upper Middle Income	Southern	Medical Doctors
19	General Surgeon	3	3	4	0.37	26,974	Seychelles	Base Estimate	2212 - Specialist medical practitioners	High Income and Upper Middle Income	Southern	Medical Doctors
20	Haematologist	0	0	1	0.05	194,262	Seychelles	Base Estimate	2212 - Specialist medical practitioners	High Income and Upper Middle Income	Southern	Medical Doctors
21	Health Promoter/Health Educator	2	2	2	0.19	54,034	Seychelles	Base Estimate	2269 - Health professionals not elsewhere classified	High Income and Upper Middle Income	Southern	Other Health Workers
22	Infectious Diseases Specialist	0	0	0	0.01	988,741	Seychelles	Base Estimate	2212 - Specialist medical practitioners	High Income and Upper Middle Income	Southern	Medical Doctors
23	Intensive Care Nurse	4	5	5	0.49	20,353	Seychelles	Base Estimate	2221 - Nursing professionals	High Income and Upper Middle Income	Southern	Nursing Personnel
24	Medical Laboratory Scientist	26	29	31	3.18	3,144	Seychelles	Base Estimate	3212 - Medical and pathology laboratory technicians	High Income and Upper Middle Income	Southern	Other Health Workers
25	Medical Laboratory Technician	18	20	21	2.15	4,657	Seychelles	Base Estimate	3212 - Medical and pathology laboratory technicians	High Income and Upper Middle Income	Southern	Other Health Workers
26	Medical Social Worker	4	4	4	0.37	26,896	Seychelles	Base Estimate	1344 - Social welfare managers	High Income and Upper Middle Income	Southern	Other Health Workers
27	Mental Health Nurse	9	9	9	0.90	11,110	Seychelles	Base Estimate	2221 - Nursing professionals	High Income and Upper Middle Income	Southern	Nursing Personnel
28	Midwife	69	70	69	7.02	1,425	Seychelles	Base Estimate	2222 - Midwifery professionals	High Income and Upper Middle Income	Southern	Midwifery Personnel
29	Nephrologist	5	5	5	0.52	19,118	Seychelles	Base Estimate	2212 - Specialist medical practitioners	High Income and Upper Middle Income	Southern	Medical Doctors
30	Neuro-Surgeon	1	1	1	0.13	79,476	Seychelles	Base Estimate	2212 - Specialist medical practitioners	High Income and Upper Middle Income	Southern	Medical Doctors
31	Nurse Anaesthetist	12	14	16	1.66	6,019	Seychelles	Base Estimate	2221 - Nursing professionals	High Income and Upper Middle Income	Southern	Nursing Personnel
32	Nutritionist	13	14	14	1.42	7,033	Seychelles	Base Estimate	2265 - Dieticians and nutritionists	High Income and Upper Middle Income	Southern	Other Health Workers
33	Obstetrician & Gynaecologist	7	7	7	0.76	13,233	Seychelles	Base Estimate	2212 - Specialist medical practitioners	High Income and Upper Middle Income	Southern	Medical Doctors
34	Occupational Therapist	4	5	5	0.50	19,940	Seychelles	Base Estimate	2269 - Health professionals not elsewhere classified	High Income and Upper Middle Income	Southern	Other Health Workers

S/N	Health Professionals	2022	2026	2030	Density per 10,000 population	Required Population ratio (1 professional is to xxx population)	Name of Country	Modelling Scenario	ISCO-08 Match	Income Group Classification	Sub-Regional Grouping	SDG 3c Occupation
35	Oncology Nurse	4	5	5	0.55	18,145	Seychelles	Base Estimate	2221 - Nursing professionals	High Income and Upper Middle Income	Southern	Nursing Personnel
36	Operating Theatre Nurse	28	30	32	3.22	3,109	Seychelles	Base Estimate	2221 - Nursing professionals	High Income and Upper Middle Income	Southern	Nursing Personnel
37	Ophthalmic Nurse	5	6	6	0.64	15,534	Seychelles	Base Estimate	2221 - Nursing professionals	High Income and Upper Middle Income	Southern	Nursing Personnel
38	Ophthalmologist	2	2	2	0.20	48,879	Seychelles	Base Estimate	2212 - Specialist medical practitioners	High Income and Upper Middle Income	Southern	Medical Doctors
39	Optometrist	4	5	5	0.51	19,518	Seychelles	Base Estimate	2267 - Optometrists and ophthalmic opticians	High Income and Upper Middle Income	Southern	Other Health Workers
40	Orthopaedic Nurse	3	3	4	0.39	25,586	Seychelles	Base Estimate	2221 - Nursing professionals	High Income and Upper Middle Income	Southern	Nursing Personnel
41	Orthopaedic Surgeon	10	11	12	1.19	8,424	Seychelles	Base Estimate	2212 - Specialist medical practitioners	High Income and Upper Middle Income	Southern	Medical Doctors
42	Orthopaedic Technologist	3	4	4	0.39	25,843	Seychelles	Base Estimate	3214 - Medical and dental prosthetic technicians	High Income and Upper Middle Income	Southern	Other Health Workers
43	Paediatric Nurse	9	9	9	0.93	10,771	Seychelles	Base Estimate	2221 - Nursing professionals	High Income and Upper Middle Income	Southern	Nursing Personnel
44	Paediatric Surgeon	0	0	0	0.02	641,774	Seychelles	Base Estimate	2212 - Specialist medical practitioners	High Income and Upper Middle Income	Southern	Medical Doctors
45	Paediatrician	7	7	7	0.72	13,886	Seychelles	Base Estimate	2212 - Specialist medical practitioners	High Income and Upper Middle Income	Southern	Medical Doctors
46	Pathologist	1	1	1	0.10	98,913	Seychelles	Base Estimate	2212 - Specialist medical practitioners	High Income and Upper Middle Income	Southern	Medical Doctors
47	Pharmacist	16	18	19	1.95	5,126	Seychelles	Base Estimate	2262 - Pharmacists	High Income and Upper Middle Income	Southern	Pharmacist
48	Pharmacy Technician	16	17	18	1.88	5,331	Seychelles	Base Estimate	3213 - Pharmaceutical technicians and assistants	High Income and Upper Middle Income	Southern	Other Health Workers
49	Physician	22	24	26	2.63	3,804	Seychelles	Base Estimate	2212 - Specialist medical practitioners	High Income and Upper Middle Income	Southern	Medical Doctors
50	Physiotherapist	4	5	5	0.51	19,474	Seychelles	Base Estimate	2264 - Physiotherapists	High Income and Upper Middle Income	Southern	Other Health Workers
51	Plastic Surgeon	2	2	2	0.19	51,300	Seychelles	Base Estimate	2212 - Specialist medical practitioners	High Income and Upper Middle Income	Southern	Medical Doctors
52	Psychiatrist	7	7	7	0.75	13,273	Seychelles	Base Estimate	2212 - Specialist medical practitioners	High Income and Upper Middle Income	Southern	Medical Doctors
53	Radiation Oncologist	2	3	3	0.34	29,797	Seychelles	Base Estimate	2212 - Specialist medical practitioners	High Income and Upper Middle Income	Southern	Medical Doctors
54	Radiographer (Diagnostics and Therapy)	18	19	21	2.08	4,804	Seychelles	Base Estimate	3211 - Medical imaging and therapeutic equipment technicians	High Income and Upper Middle Income	Southern	Other Health Workers
55	Radiologist	6	7	7	0.73	13,749	Seychelles	Base Estimate	2212 - Specialist medical practitioners	High Income and Upper Middle Income	Southern	Medical Doctors
56	Registered General Nurse / State Certified Nurse	457	491	518	52.44	191	Seychelles	Base Estimate	2221 - Nursing professionals	High Income and Upper Middle Income	Southern	Nursing Personnel
57	Renal Nurse	63	68	71	7.16	1,397	Seychelles	Base Estimate	2221 - Nursing professionals	High Income and Upper Middle Income	Southern	Nursing Personnel
58	Respiratory Physician	1	1	1	0.10	97,507	Seychelles	Base Estimate	2212 - Specialist medical practitioners	High Income and Upper Middle Income	Southern	Medical Doctors
59	Rheumatologist	0	0	1	0.05	187,880	Seychelles	Base Estimate	2212 - Specialist medical practitioners	High Income and Upper Middle Income	Southern	Medical Doctors
60	Speech Therapist	2	2	2	0.21	46,661	Seychelles	Base Estimate	2266 - Audiologists and speech therapists	High Income and Upper Middle Income	Southern	Other Health Workers
61	Urologist	1	1	2	0.16	62,280	Seychelles	Base Estimate	2212 - Specialist medical practitioners	High Income and Upper Middle Income	Southern	Medical Doctors
1	Anaesthesiologist	98	109	121	0.15	66,471	Sierra Leone	Base Estimate	2212 - Specialist medical practitioners	Low Income	West	Medical Doctors
2	Associate Nurse/Enrolled Nurse/Nursing Assistant	9,958	10,827	11,784	14.66	682	Sierra Leone	Base Estimate	3221 - Nursing associate professionals	Low Income	West	Nursing Personnel
3	Audiologist	31	34	37	0.05	216,083	Sierra Leone	Base Estimate	2266 - Audiologists and speech therapists	Low Income	West	Other Health Workers
4	Cardiologist	64	71	78	0.10	103,505	Sierra Leone	Base Estimate	2212 - Specialist medical practitioners	Low Income	West	Medical Doctors
5	Cardiothoracic Surgeon	13	15	16	0.02	488,278	Sierra Leone	Base Estimate	2212 - Specialist medical practitioners	Low Income	West	Medical Doctors
6	Clinical Officer/Physician Assistant	1,605	1,661	1,731	2.17	4,618	Sierra Leone	Base Estimate	3256 - Medical assistants	Low Income	West	Other Health Workers
7	Clinical Pharmacist	264	287	317	0.40	25,263	Sierra Leone	Base Estimate	2262 - Pharmacists	Low Income	West	Pharmacist
8	Clinical Psychologist	553	605	670	0.83	11,976	Sierra Leone	Base Estimate	2634 - Psychologists	Low Income	West	Other Health Workers
9	Community health worker/Village health worker	9,273	9,628	9,963	12.42	805	Sierra Leone	Base Estimate	3253 - Community health workers	Low Income	West	Other Health Workers
10	Dental Surgery Assistant	685	755	838	1.04	9,599	Sierra Leone	Base Estimate	3251 - Dental assistants and therapists	Low Income	West	Other Health Workers
11	Dental Therapist	443	487	541	0.67	14,871	Sierra Leone	Base Estimate	3251 - Dental assistants and therapists	Low Income	West	Other Health Workers
12	Dentist	669	735	824	1.02	9,767	Sierra Leone	Base Estimate	2261 - Dentists	Low Income	West	Dentist
13	Dermatologist	31	34	37	0.05	216,732	Sierra Leone	Base Estimate	2212 - Specialist medical practitioners	Low Income	West	Medical Doctors
14	Endocrinologist	21	24	30	0.04	266,159	Sierra Leone	Base Estimate	2212 - Specialist medical practitioners	Low Income	West	Medical Doctors
15	ENT Surgeon	86	95	105	0.13	76,793	Sierra Leone	Base Estimate	2212 - Specialist medical practitioners	Low Income	West	Medical Doctors
16	Environmental Health Officer	396	431	465	0.58	17,313	Sierra Leone	Base Estimate	2263 - Environmental and occupational health and hygiene professionals	Low Income	West	Other Health Workers
17	Gastroenterologist	102	99	99	0.13	79,789	Sierra Leone	Base Estimate	2212 - Specialist medical practitioners	Low Income	West	Medical Doctors
18	General Medical Practitioner (Generalist Doctor)	3,468	3,745	4,048	5.04	1,984	Sierra Leone	Base Estimate	2211 - Generalist medical practitioners	Low Income	West	Medical Doctors
19	General Surgeon	87	100	117	0.15	68,709	Sierra Leone	Base Estimate	2212 - Specialist medical practitioners	Low Income	West	Medical Doctors
20	Haematologist	39	42	46	0.06	175,764	Sierra Leone	Base Estimate	2212 - Specialist medical practitioners	Low Income	West	Medical Doctors
21	Health Promoter/Health Educator	111	114	118	0.15	67,853	Sierra Leone	Base Estimate	2269 - Health professionals not elsewhere classified	Low Income	West	Other Health Workers
22	Infectious Diseases Specialist	7	8	9	0.01	909,938	Sierra Leone	Base Estimate	2212 - Specialist medical practitioners	Low Income	West	Medical Doctors
23	Intensive Care Nurse	226	246	267	0.33	30,166	Sierra Leone	Base Estimate	2221 - Nursing professionals	Low Income	West	Nursing Personnel
24	Medical Laboratory Scientist	1,094	1,172	1,273	1.59	6,287	Sierra Leone	Base Estimate	3212 - Medical and pathology laboratory technicians	Low Income	West	Other Health Workers
25	Medical Laboratory Technician	1,899	1,969	2,045	2.55	3,916	Sierra Leone	Base Estimate	3212 - Medical and pathology laboratory technicians	Low Income	West	Other Health Workers
26	Medical Social Worker	334	340	349	0.44	22,860	Sierra Leone	Base Estimate	1344 - Social welfare managers	Low Income	West	Other Health Workers
27	Mental Health Nurse	509	562	638	0.80	12,535	Sierra Leone	Base Estimate	2221 - Nursing professionals	Low Income	West	Nursing Personnel
28	Midwife	6,528	7,041	7,614	9.46	1,057	Sierra Leone	Base Estimate	2222 - Midwifery professionals	Low Income	West	Midwifery Personnel
29	Nephrologist	87	98	115	0.14	69,386	Sierra Leone	Base Estimate	2212 - Specialist medical practitioners	Low Income	West	Medical Doctors
30	Neuro-Surgeon	28	31	35	0.04	228,879	Sierra Leone	Base Estimate	2212 - Specialist medical practitioners	Low Income	West	Medical Doctors
31	Nurse Anaesthetist	175	199	227	0.28	35,436	Sierra Leone	Base Estimate	2221 - Nursing professionals	Low Income	West	Nursing Personnel
32	Nutritionist	1,131	1,177	1,218	1.52	6,595	Sierra Leone	Base Estimate	2265 - Dieticians and nutritionists	Low Income	West	Other Health Workers
33	Obstetrician & Gynaecologist	560	616	678	0.84	11,850	Sierra Leone	Base Estimate	2212 - Specialist medical practitioners	Low Income	West	Medical Doctors
34	Occupational Therapist	199	223	251	0.31	32,029	Sierra Leone	Base Estimate	2269 - Health professionals not elsewhere classified	Low Income	West	Other Health Workers
35	Oncology Nurse	63	71	83	0.10	96,705	Sierra Leone	Base Estimate	2221 - Nursing professionals	Low Income	West	Nursing Personnel
36	Operating Theatre Nurse	897	993	1,126	1.41	7,107	Sierra Leone	Base Estimate	2221 - Nursing professionals	Low Income	West	Nursing Personnel
37	Ophthalmic Nurse	95	105	117	0.15	68,675	Sierra Leone	Base Estimate	2221 - Nursing professionals	Low Income	West	Nursing Personnel
38	Ophthalmologist	26	29	33	0.04	245,934	Sierra Leone	Base Estimate	2212 - Specialist medical practitioners	Low Income	West	Medical Doctors
39	Optometrist	111	123	135	0.17	59,374	Sierra Leone	Base Estimate	2267 - Optometrists and ophthalmic opticians	Low Income	West	Other Health Workers
40	Orthopaedic Nurse	194	221	250	0.31	32,199	Sierra Leone	Base Estimate	2221 - Nursing professionals	Low Income	West	Nursing Personnel
41	Orthopaedic Surgeon	269	310	359	0.45	22,318	Sierra Leone	Base Estimate	2212 - Specialist medical practitioners	Low Income	West	Medical Doctors
42	Orthopaedic Technologist	251	277	304	0.38	26,450	Sierra Leone	Base Estimate	3214 - Medical and dental prosthetic technicians	Low Income	West	Other Health Workers
43	Paediatric Nurse	1,024	1,102	1,170	1.45	6,884	Sierra Leone	Base Estimate	2221 - Nursing professionals	Low Income	West	Nursing Personnel
44	Paediatric Surgeon	27	29	30	0.04	268,750	Sierra Leone	Base Estimate	2212 - Specialist medical practitioners	Low Income	West	Medical Doctors
45	Paediatrician	393	431	474	0.59	16,952	Sierra Leone	Base Estimate	2212 - Specialist medical practitioners	Low Income	West	Medical Doctors
46	Pathologist	53	59	68	0.09	117,331	Sierra Leone	Base Estimate	2212 - Specialist medical practitioners	Low Income	West	Medical Doctors
47	Pharmacist	652	669	690	0.86	11,582	Sierra Leone	Base Estimate	2262 - Pharmacists	Low Income	West	Pharmacist
48	Pharmacy Technician	905	985	1,074	1.34	7,483	Sierra Leone	Base Estimate	3213 - Pharmaceutical technicians and assistants	Low Income	West	Other Health Workers
49	Physician	981	1,071	1,174	1.46	6,837	Sierra Leone	Base Estimate	2212 - Specialist medical practitioners	Low Income	West	Medical Doctors
50	Physiotherapist	222	244	269	0.33	29,901	Sierra Leone	Base Estimate	2264 - Physiotherapists	Low Income	West	Other Health Workers
51	Plastic Surgeon	58	65	72	0.09	112,082	Sierra Leone	Base Estimate	2212 - Specialist medical practitioners	Low Income	West	Medical Doctors
52	Psychiatrist	470	513	573	0.71	13,987	Sierra Leone	Base Estimate	2212 - Specialist medical practitioners	Low Income	West	Medical Doctors
53	Radiation Oncologist	17	20	23	0.03	344,969	Sierra Leone	Base Estimate	2212 - Specialist medical practitioners	Low Income	West	Medical Doctors
54	Radiographer (Diagnostics and Therapy)	654	725	818	1.02	9,793	Sierra Leone	Base Estimate	3211 - Medical imaging and therapeutic equipment technicians	Low Income	West	Other Health Workers
55	Radiologist	230	256	288	0.36	27,817	Sierra Leone	Base Estimate	2212 - Specialist medical practitioners	Low Income	West	Medical Doctors
56	Registered General Nurse / State Certified Nurse	19,176	20,622	22,440	28.04	357	Sierra Leone	Base Estimate	2221 - Nursing professionals	Low Income	West	Nursing Personnel
57	Renal Nurse	1,135	1,285	1,506	1.89	5,300	Sierra Leone	Base Estimate	2221 - Nursing professionals	Low Income	West	Nursing Personnel

S/N	Health Professionals	2022	2026	2030	Density per 10,000 population	Required Population ratio (1 professional is to xxx population)	Name of Country	Modelling Scenario	ISCO-08 Match	Income Group Classification	Sub-Regional Grouping	SDG 3c Occupation
58	Respiratory Physician	61	67	74	0.09	107,983	Sierra Leone	Base Estimate	2212 - Specialist medical practitioners	Low Income	West	Medical Doctors
59	Rheumatologist	23	25	28	0.03	287,471	Sierra Leone	Base Estimate	2212 - Specialist medical practitioners	Low Income	West	Medical Doctors
60	Speech Therapist	83	91	98	0.12	81,547	Sierra Leone	Base Estimate	2266 - Audiologists and speech therapists	Low Income	West	Other Health Workers
61	Urologist	12	15	18	0.02	447,256	Sierra Leone	Base Estimate	2212 - Specialist medical practitioners	Low Income	West	Medical Doctors
1	Anaesthesiologist	949	1,009	1,079	0.18	55,244	South Africa	Base Estimate	2212 - Specialist medical practitioners	High Income and Upper Middle Income	Southern	Medical Doctors
2	Associate Nurse/Enrolled Nurse/Nursing Assistant	99,187	106,320	114,473	19.22	520	South Africa	Base Estimate	3221 - Nursing associate professionals	High Income and Upper Middle Income	Southern	Nursing Personnel
3	Audiologist	319	342	367	0.06	162,837	South Africa	Base Estimate	2266 - Audiologists and speech therapists	High Income and Upper Middle Income	Southern	Other Health Workers
4	Cardiologist	897	964	1,038	0.17	57,571	South Africa	Base Estimate	2212 - Specialist medical practitioners	High Income and Upper Middle Income	Southern	Medical Doctors
5	Cardiothoracic Surgeon	105	106	108	0.02	549,571	South Africa	Base Estimate	2212 - Specialist medical practitioners	High Income and Upper Middle Income	Southern	Medical Doctors
6	Clinical Officer/Physician Assistant	17,418	18,624	20,066	3.38	2,958	South Africa	Base Estimate	3256 - Medical assistants	High Income and Upper Middle Income	Southern	Other Health Workers
7	Clinical Pharmacist	2,341	2,538	2,778	0.47	21,381	South Africa	Base Estimate	2262 - Pharmacists	High Income and Upper Middle Income	Southern	Pharmacist
8	Clinical Psychologist	3,571	3,744	3,936	0.66	15,137	South Africa	Base Estimate	2634 - Psychologists	High Income and Upper Middle Income	Southern	Other Health Workers
9	Community health worker/Village health worker	47,394	49,759	52,530	8.87	1,128	South Africa	Base Estimate	3253 - Community health workers	High Income and Upper Middle Income	Southern	Other Health Workers
10	Dental Surgery Assistant	7,674	8,141	8,624	1.44	6,928	South Africa	Base Estimate	3251 - Dental assistants and therapists	High Income and Upper Middle Income	Southern	Other Health Workers
11	Dental Therapist	4,974	5,278	5,591	0.94	10,686	South Africa	Base Estimate	3251 - Dental assistants and therapists	High Income and Upper Middle Income	Southern	Other Health Workers
12	Dentist	5,055	5,300	5,551	0.93	10,754	South Africa	Base Estimate	2261 - Dentists	High Income and Upper Middle Income	Southern	Dentist
13	Dermatologist	267	282	297	0.05	200,854	South Africa	Base Estimate	2212 - Specialist medical practitioners	High Income and Upper Middle Income	Southern	Medical Doctors
14	Endocrinologist	695	781	882	0.15	67,394	South Africa	Base Estimate	2212 - Specialist medical practitioners	High Income and Upper Middle Income	Southern	Medical Doctors
15	ENT Surgeon	1,251	1,358	1,504	0.25	39,623	South Africa	Base Estimate	2212 - Specialist medical practitioners	High Income and Upper Middle Income	Southern	Medical Doctors
16	Environmental Health Officer	2,946	3,061	3,179	0.53	18,778	South Africa	Base Estimate	2263 - Environmental and occupational health and hygiene professionals	High Income and Upper Middle Income	Southern	Other Health Workers
17	Gastroenterologist	514	510	509	0.09	115,998	South Africa	Base Estimate	2212 - Specialist medical practitioners	High Income and Upper Middle Income	Southern	Medical Doctors
18	General Medical Practitioner (Generalist Doctor)	35,488	37,890	40,580	6.81	1,468	South Africa	Base Estimate	2211 - Generalist medical practitioners	High Income and Upper Middle Income	Southern	Medical Doctors
19	General Surgeon	1,047	1,124	1,217	0.20	48,940	South Africa	Base Estimate	2212 - Specialist medical practitioners	High Income and Upper Middle Income	Southern	Medical Doctors
20	Haematologist	139	143	147	0.02	404,020	South Africa	Base Estimate	2212 - Specialist medical practitioners	High Income and Upper Middle Income	Southern	Medical Doctors
21	Health Promoter/Health Educator	1,289	1,357	1,438	0.24	41,339	South Africa	Base Estimate	2269 - Health professionals not elsewhere classified	High Income and Upper Middle Income	Southern	Other Health Workers
22	Infectious Diseases Specialist	95	99	104	0.02	575,409	South Africa	Base Estimate	2212 - Specialist medical practitioners	High Income and Upper Middle Income	Southern	Medical Doctors
23	Intensive Care Nurse	1,723	1,780	1,845	0.31	32,306	South Africa	Base Estimate	2221 - Nursing professionals	High Income and Upper Middle Income	Southern	Nursing Personnel
24	Medical Laboratory Scientist	14,975	16,356	17,968	3.02	3,312	South Africa	Base Estimate	3212 - Medical and pathology laboratory technicians	High Income and Upper Middle Income	Southern	Other Health Workers
25	Medical Laboratory Technician	12,003	13,179	14,594	2.46	4,061	South Africa	Base Estimate	3212 - Medical and pathology laboratory technicians	High Income and Upper Middle Income	Southern	Other Health Workers
26	Medical Social Worker	1,682	1,695	1,699	0.29	34,928	South Africa	Base Estimate	1344 - Social welfare managers	High Income and Upper Middle Income	Southern	Other Health Workers
27	Mental Health Nurse	5,232	5,503	5,828	0.98	10,198	South Africa	Base Estimate	2221 - Nursing professionals	High Income and Upper Middle Income	Southern	Nursing Personnel
28	Midwife	43,590	45,288	46,837	7.85	1,274	South Africa	Base Estimate	2222 - Midwifery professionals	High Income and Upper Middle Income	Southern	Midwifery Personnel
29	Nephrologist	1,496	1,676	1,888	0.32	31,488	South Africa	Base Estimate	2212 - Specialist medical practitioners	High Income and Upper Middle Income	Southern	Medical Doctors
30	Neuro-Surgeon	343	366	402	0.07	147,988	South Africa	Base Estimate	2212 - Specialist medical practitioners	High Income and Upper Middle Income	Southern	Medical Doctors
31	Nurse Anaesthetist	2,911	3,206	3,536	0.59	16,888	South Africa	Base Estimate	2221 - Nursing professionals	High Income and Upper Middle Income	Southern	Nursing Personnel
32	Nutritionist	4,838	4,861	4,872	0.82	12,205	South Africa	Base Estimate	2265 - Dieticians and nutritionists	High Income and Upper Middle Income	Southern	Other Health Workers
33	Obstetrician & Gynaecologist	5,770	6,056	6,363	1.07	9,373	South Africa	Base Estimate	2212 - Specialist medical practitioners	High Income and Upper Middle Income	Southern	Medical Doctors
34	Occupational Therapist	1,697	1,797	1,934	0.33	30,735	South Africa	Base Estimate	2269 - Health professionals not elsewhere classified	High Income and Upper Middle Income	Southern	Other Health Workers
35	Oncology Nurse	1,157	1,295	1,455	0.24	40,916	South Africa	Base Estimate	2221 - Nursing professionals	High Income and Upper Middle Income	Southern	Nursing Personnel
36	Operating Theatre Nurse	11,695	12,590	13,704	2.30	4,340	South Africa	Base Estimate	2221 - Nursing professionals	High Income and Upper Middle Income	Southern	Nursing Personnel
37	Ophthalmic Nurse	1,446	1,579	1,729	0.29	34,478	South Africa	Base Estimate	2221 - Nursing professionals	High Income and Upper Middle Income	Southern	Nursing Personnel
38	Ophthalmologist	620	682	754	0.13	79,093	South Africa	Base Estimate	2212 - Specialist medical practitioners	High Income and Upper Middle Income	Southern	Medical Doctors
39	Optometrist	3,009	3,311	3,644	0.61	16,400	South Africa	Base Estimate	2267 - Optometrists and ophthalmic opticians	High Income and Upper Middle Income	Southern	Other Health Workers
40	Orthopaedic Nurse	1,346	1,386	1,431	0.24	41,643	South Africa	Base Estimate	2221 - Nursing professionals	High Income and Upper Middle Income	Southern	Nursing Personnel
41	Orthopaedic Surgeon	3,696	4,084	4,524	0.76	13,198	South Africa	Base Estimate	2212 - Specialist medical practitioners	High Income and Upper Middle Income	Southern	Medical Doctors
42	Orthopaedic Technologist	1,873	1,939	2,011	0.34	29,654	South Africa	Base Estimate	3214 - Medical and dental prosthetic technicians	High Income and Upper Middle Income	Southern	Other Health Workers
43	Paediatric Nurse	5,263	5,663	6,102	1.03	9,706	South Africa	Base Estimate	2221 - Nursing professionals	High Income and Upper Middle Income	Southern	Nursing Personnel
44	Paediatric Surgeon	152	157	161	0.03	370,477	South Africa	Base Estimate	2212 - Specialist medical practitioners	High Income and Upper Middle Income	Southern	Medical Doctors
45	Paediatrician	4,441	5,432	6,713	1.14	8,765	South Africa	Base Estimate	2212 - Specialist medical practitioners	High Income and Upper Middle Income	Southern	Medical Doctors
46	Pathologist	936	979	1,028	0.17	57,766	South Africa	Base Estimate	2212 - Specialist medical practitioners	High Income and Upper Middle Income	Southern	Medical Doctors
47	Pharmacist	11,620	12,180	12,813	2.15	4,646	South Africa	Base Estimate	2262 - Pharmacists	High Income and Upper Middle Income	Southern	Pharmacist
48	Pharmacy Technician	8,979	9,456	9,985	1.68	5,968	South Africa	Base Estimate	3213 - Pharmaceutical technicians and assistants	High Income and Upper Middle Income	Southern	Other Health Workers
49	Physician	8,915	9,717	10,660	1.79	5,577	South Africa	Base Estimate	2212 - Specialist medical practitioners	High Income and Upper Middle Income	Southern	Medical Doctors
50	Physiotherapist	1,980	2,081	2,224	0.37	26,758	South Africa	Base Estimate	2264 - Physiotherapists	High Income and Upper Middle Income	Southern	Other Health Workers
51	Plastic Surgeon	572	576	580	0.10	102,593	South Africa	Base Estimate	2212 - Specialist medical practitioners	High Income and Upper Middle Income	Southern	Medical Doctors
52	Psychiatrist	4,151	4,368	4,630	0.78	12,847	South Africa	Base Estimate	2212 - Specialist medical practitioners	High Income and Upper Middle Income	Southern	Medical Doctors
53	Radiation Oncologist	408	456	513	0.09	116,091	South Africa	Base Estimate	2212 - Specialist medical practitioners	High Income and Upper Middle Income	Southern	Medical Doctors
54	Radiographer (Diagnostics and Therapy)	7,943	8,602	9,355	1.57	6,366	South Africa	Base Estimate	3211 - Medical imaging and therapeutic equipment technicians	High Income and Upper Middle Income	Southern	Other Health Workers
55	Radiologist	2,749	2,957	3,192	0.54	18,684	South Africa	Base Estimate	2212 - Specialist medical practitioners	High Income and Upper Middle Income	Southern	Medical Doctors
56	Registered General Nurse / State Certified Nurse	207,612	224,950	244,927	41.18	243	South Africa	Base Estimate	2221 - Nursing professionals	High Income and Upper Middle Income	Southern	Nursing Personnel
57	Renal Nurse	20,066	22,500	25,353	4.26	2,345	South Africa	Base Estimate	2221 - Nursing professionals	High Income and Upper Middle Income	Southern	Nursing Personnel
58	Respiratory Physician	1,003	1,289	1,665	0.28	35,226	South Africa	Base Estimate	2212 - Specialist medical practitioners	High Income and Upper Middle Income	Southern	Medical Doctors
59	Rheumatologist	269	289	313	0.05	190,650	South Africa	Base Estimate	2212 - Specialist medical practitioners	High Income and Upper Middle Income	Southern	Medical Doctors
60	Speech Therapist	783	822	893	0.15	66,537	South Africa	Base Estimate	2266 - Audiologists and speech therapists	High Income and Upper Middle Income	Southern	Other Health Workers
61	Urologist	225	255	291	0.05	203,932	South Africa	Base Estimate	2212 - Specialist medical practitioners	High Income and Upper Middle Income	Southern	Medical Doctors
1	Anaesthesiologist	121	135	152	0.14	73,646	South Sudan	Base Estimate	2212 - Specialist medical practitioners	Low Income	East	Medical Doctors
2	Associate Nurse/Enrolled Nurse/Nursing Assistant	13,868	14,718	15,821	14.13	708	South Sudan	Base Estimate	3221 - Nursing associate professionals	Low Income	East	Nursing Personnel
3	Audiologist	42	46	50	0.04	224,476	South Sudan	Base Estimate	2266 - Audiologists and speech therapists	Low Income	East	Other Health Workers
4	Cardiologist	91	107	127	0.11	88,304	South Sudan	Base Estimate	2212 - Specialist medical practitioners	Low Income	East	Medical Doctors
5	Cardiothoracic Surgeon	21	23	26	0.02	435,721	South Sudan	Base Estimate	2212 - Specialist medical practitioners	Low Income	East	Medical Doctors
6	Clinical Officer/Physician Assistant	2,193	2,242	2,334	2.09	4,777	South Sudan	Base Estimate	3256 - Medical assistants	Low Income	East	Other Health Workers
7	Clinical Pharmacist	339	367	405	0.36	27,636	South Sudan	Base Estimate	2262 - Pharmacists	Low Income	East	Pharmacist
8	Clinical Psychologist	806	861	936	0.84	11,969	South Sudan	Base Estimate	2634 - Psychologists	Low Income	East	Other Health Workers
9	Community health worker/Village health worker	11,990	12,229	12,586	11.26	888	South Sudan	Base Estimate	3253 - Community health workers	Low Income	East	Other Health Workers
10	Dental Surgery Assistant	1,130	1,227	1,351	1.20	8,303	South Sudan	Base Estimate	3251 - Dental assistants and therapists	Low Income	East	Other Health Workers
11	Dental Therapist	732	795	875	0.78	12,817	South Sudan	Base Estimate	3251 - Dental assistants and therapists	Low Income	East	Other Health Workers
12	Dentist	821	888	985	0.88	11,378	South Sudan	Base Estimate	2261 - Dentists	Low Income	East	Dentist
13	Dermatologist	38	41	45	0.04	248,495	South Sudan	Base Estimate	2212 - Specialist medical practitioners	Low Income	East	Medical Doctors
14	Endocrinologist	37	44	54	0.05	206,065	South Sudan	Base Estimate	2212 - Specialist medical practitioners	Low Income	East	Medical Doctors
15	ENT Surgeon	128	142	159	0.14	70,475	South Sudan	Base Estimate	2212 - Specialist medical practitioners	Low Income	East	Medical Doctors
16	Environmental Health Officer	556	594	638	0.57	17,584	South Sudan	Base Estimate	2263 - Environmental and occupational health and hygiene professionals	Low Income	East	Other Health Workers
17	Gastroenterologist	118	119	125	0.11	89,004	South Sudan	Base Estimate	2212 - Specialist medical practitioners	Low Income	East	Medical Doctors
18	General Medical Practitioner (Generalist Doctor)	4,555	4,834	5,194	4.64	2,156	South Sudan	Base Estimate	2211 - Generalist medical practitioners	Low Income	East	Medical Doctors
19	General Surgeon	80	88	101	0.09	110,911	South Sudan	Base Estimate	2212 - Specialist medical practitioners	Low Income	East	Medical Doctors

S/N	Health Professionals	2022	2026	2030	Density per 10,000 population	Required Population ratio (1 professional is to xxx population)	Name of Country	Modelling Scenario	ISCO-08 Match	Income Group Classification	Sub-Regional Grouping	SDG 3c Occupation
20	Haematologist	46	49	53	0.05	210,165	South Sudan	Base Estimate	2212 - Specialist medical practitioners	Low Income	East	Medical Doctors
21	Health Promoter/Health Educator	174	176	180	0.16	61,786	South Sudan	Base Estimate	2269 - Health professionals not elsewhere classified	Low Income	East	Other Health Workers
22	Infectious Diseases Specialist	11	12	12	0.01	904,998	South Sudan	Base Estimate	2212 - Specialist medical practitioners	Low Income	East	Medical Doctors
23	Intensive Care Nurse	396	416	441	0.39	25,386	South Sudan	Base Estimate	2221 - Nursing professionals	Low Income	East	Nursing Personnel
24	Medical Laboratory Scientist	1,380	1,488	1,637	1.47	6,825	South Sudan	Base Estimate	3212 - Medical and pathology laboratory technicians	Low Income	East	Other Health Workers
25	Medical Laboratory Technician	1,608	1,674	1,771	1.59	6,308	South Sudan	Base Estimate	3212 - Medical and pathology laboratory technicians	Low Income	East	Other Health Workers
26	Medical Social Worker	527	529	537	0.48	20,774	South Sudan	Base Estimate	1344 - Social welfare managers	Low Income	East	Other Health Workers
27	Mental Health Nurse	845	912	1,018	0.91	10,968	South Sudan	Base Estimate	2221 - Nursing professionals	Low Income	East	Nursing Personnel
28	Midwife	7,981	8,500	9,178	8.18	1,222	South Sudan	Base Estimate	2222 - Midwifery professionals	Low Income	East	Midwifery Personnel
29	Nephrologist	96	108	125	0.11	89,447	South Sudan	Base Estimate	2212 - Specialist medical practitioners	Low Income	East	Medical Doctors
30	Neuro-Surgeon	32	37	42	0.04	265,095	South Sudan	Base Estimate	2212 - Specialist medical practitioners	Low Income	East	Medical Doctors
31	Nurse Anaesthetist	233	264	302	0.27	37,055	South Sudan	Base Estimate	2221 - Nursing professionals	Low Income	East	Nursing Personnel
32	Nutritionist	2,176	2,169	2,177	1.95	5,128	South Sudan	Base Estimate	2265 - Dieticians and nutritionists	Low Income	East	Other Health Workers
33	Obstetrician & Gynaecologist	781	841	917	0.82	12,226	South Sudan	Base Estimate	2212 - Specialist medical practitioners	Low Income	East	Medical Doctors
34	Occupational Therapist	221	248	284	0.25	39,429	South Sudan	Base Estimate	2269 - Health professionals not elsewhere classified	Low Income	East	Other Health Workers
35	Oncology Nurse	76	85	99	0.09	113,293	South Sudan	Base Estimate	2221 - Nursing professionals	Low Income	East	Nursing Personnel
36	Operating Theatre Nurse	1,183	1,321	1,518	1.36	7,347	South Sudan	Base Estimate	2221 - Nursing professionals	Low Income	East	Nursing Personnel
37	Ophthalmic Nurse	132	144	160	0.14	69,700	South Sudan	Base Estimate	2221 - Nursing professionals	Low Income	East	Nursing Personnel
38	Ophthalmologist	44	51	60	0.05	187,130	South Sudan	Base Estimate	2212 - Specialist medical practitioners	Low Income	East	Medical Doctors
39	Optometrist	193	227	269	0.24	41,581	South Sudan	Base Estimate	2267 - Optometrists and ophthalmic opticians	Low Income	East	Other Health Workers
40	Orthopaedic Nurse	204	229	259	0.23	43,247	South Sudan	Base Estimate	2221 - Nursing professionals	Low Income	East	Nursing Personnel
41	Orthopaedic Surgeon	275	306	345	0.31	32,460	South Sudan	Base Estimate	2212 - Specialist medical practitioners	Low Income	East	Medical Doctors
42	Orthopaedic Technologist	341	370	404	0.36	27,792	South Sudan	Base Estimate	3214 - Medical and dental prosthetic technicians	Low Income	East	Other Health Workers
43	Paediatric Nurse	1,554	1,630	1,715	1.53	6,544	South Sudan	Base Estimate	2221 - Nursing professionals	Low Income	East	Nursing Personnel
44	Paediatric Surgeon	41	44	46	0.04	242,829	South Sudan	Base Estimate	2212 - Specialist medical practitioners	Low Income	East	Medical Doctors
45	Paediatrician	594	647	714	0.64	15,698	South Sudan	Base Estimate	2212 - Specialist medical practitioners	Low Income	East	Medical Doctors
46	Pathologist	86	94	106	0.10	104,917	South Sudan	Base Estimate	2212 - Specialist medical practitioners	Low Income	East	Medical Doctors
47	Pharmacist	1,200	1,246	1,310	1.17	8,537	South Sudan	Base Estimate	2262 - Pharmacists	Low Income	East	Pharmacist
48	Pharmacy Technician	1,171	1,254	1,359	1.21	8,243	South Sudan	Base Estimate	3213 - Pharmaceutical technicians and assistants	Low Income	East	Other Health Workers
49	Physician	1,113	1,201	1,319	1.18	8,478	South Sudan	Base Estimate	2212 - Specialist medical practitioners	Low Income	East	Medical Doctors
50	Physiotherapist	306	333	367	0.33	30,536	South Sudan	Base Estimate	2264 - Physiotherapists	Low Income	East	Other Health Workers
51	Plastic Surgeon	183	204	228	0.20	49,334	South Sudan	Base Estimate	2212 - Specialist medical practitioners	Low Income	East	Medical Doctors
52	Psychiatrist	731	775	846	0.76	13,194	South Sudan	Base Estimate	2212 - Specialist medical practitioners	Low Income	East	Medical Doctors
53	Radiation Oncologist	25	29	34	0.03	324,193	South Sudan	Base Estimate	2212 - Specialist medical practitioners	Low Income	East	Medical Doctors
54	Radiographer (Diagnostics and Therapy)	722	794	896	0.80	12,475	South Sudan	Base Estimate	3211 - Medical imaging and therapeutic equipment technicians	Low Income	East	Other Health Workers
55	Radiologist	259	284	317	0.28	35,341	South Sudan	Base Estimate	2212 - Specialist medical practitioners	Low Income	East	Medical Doctors
56	Registered General Nurse / State Certified Nurse	35,515	36,442	38,186	34.31	291	South Sudan	Base Estimate	2221 - Nursing professionals	Low Income	East	Nursing Personnel
57	Renal Nurse	1,275	1,428	1,657	1.49	6,727	South Sudan	Base Estimate	2221 - Nursing professionals	Low Income	East	Nursing Personnel
58	Respiratory Physician	125	136	151	0.13	74,542	South Sudan	Base Estimate	2212 - Specialist medical practitioners	Low Income	East	Medical Doctors
59	Rheumatologist	36	39	44	0.04	255,312	South Sudan	Base Estimate	2212 - Specialist medical practitioners	Low Income	East	Medical Doctors
60	Speech Therapist	122	132	143	0.13	78,111	South Sudan	Base Estimate	2266 - Audiologists and speech therapists	Low Income	East	Other Health Workers
61	Urologist	13	15	18	0.02	621,845	South Sudan	Base Estimate	2212 - Specialist medical practitioners	Low Income	East	Medical Doctors
1	Anaesthesiologist	113	129	147	0.18	56,503	Togo	Base Estimate	2212 - Specialist medical practitioners	Low Income	West	Medical Doctors
2	Associate Nurse/Enrolled Nurse/Nursing Assistant	10,668	11,744	12,970	15.58	642	Togo	Base Estimate	3221 - Nursing associate professionals	Low Income	West	Nursing Personnel
3	Audiologist	34	38	42	0.05	198,283	Togo	Base Estimate	2266 - Audiologists and speech therapists	Low Income	West	Other Health Workers
4	Cardiologist	71	83	96	0.11	86,981	Togo	Base Estimate	2212 - Specialist medical practitioners	Low Income	West	Medical Doctors
5	Cardiothoracic Surgeon	17	19	22	0.03	381,065	Togo	Base Estimate	2212 - Specialist medical practitioners	Low Income	West	Medical Doctors
6	Clinical Officer/Physician Assistant	1,502	1,611	1,750	2.11	4,734	Togo	Base Estimate	3256 - Medical assistants	Low Income	West	Other Health Workers
7	Clinical Pharmacist	268	300	338	0.41	24,544	Togo	Base Estimate	2262 - Pharmacists	Low Income	West	Pharmacist
8	Clinical Psychologist	524	584	657	0.79	12,664	Togo	Base Estimate	2634 - Psychologists	Low Income	West	Other Health Workers
9	Community health worker/Village health worker	9,658	10,428	11,264	13.53	739	Togo	Base Estimate	3253 - Community health workers	Low Income	West	Other Health Workers
10	Dental Surgery Assistant	1,034	1,159	1,304	1.56	6,400	Togo	Base Estimate	3251 - Dental assistants and therapists	Low Income	West	Other Health Workers
11	Dental Therapist	670	751	845	1.01	9,875	Togo	Base Estimate	3251 - Dental assistants and therapists	Low Income	West	Other Health Workers
12	Dentist	687	763	858	1.03	9,738	Togo	Base Estimate	2261 - Dentists	Low Income	West	Dentist
13	Dermatologist	33	36	40	0.05	207,823	Togo	Base Estimate	2212 - Specialist medical practitioners	Low Income	West	Medical Doctors
14	Endocrinologist	26	33	41	0.05	197,629	Togo	Base Estimate	2212 - Specialist medical practitioners	Low Income	West	Medical Doctors
15	ENT Surgeon	97	110	125	0.15	66,650	Togo	Base Estimate	2212 - Specialist medical practitioners	Low Income	West	Medical Doctors
16	Environmental Health Officer	411	450	490	0.59	17,022	Togo	Base Estimate	2263 - Environmental and occupational health and hygiene professionals	Low Income	West	Other Health Workers
17	Gastroenterologist	79	80	84	0.10	97,967	Togo	Base Estimate	2212 - Specialist medical practitioners	Low Income	West	Medical Doctors
18	General Medical Practitioner (Generalist Doctor)	3,651	4,009	4,413	5.30	1,886	Togo	Base Estimate	2211 - Generalist medical practitioners	Low Income	West	Medical Doctors
19	General Surgeon	98	115	136	0.16	60,874	Togo	Base Estimate	2212 - Specialist medical practitioners	Low Income	West	Medical Doctors
20	Haematologist	37	41	45	0.05	184,680	Togo	Base Estimate	2212 - Specialist medical practitioners	Low Income	West	Medical Doctors
21	Health Promoter/Health Educator	117	124	133	0.16	62,484	Togo	Base Estimate	2269 - Health professionals not elsewhere classified	Low Income	West	Other Health Workers
22	Infectious Diseases Specialist	11	12	13	0.02	616,659	Togo	Base Estimate	2212 - Specialist medical practitioners	Low Income	West	Medical Doctors
23	Intensive Care Nurse	247	270	295	0.35	28,230	Togo	Base Estimate	2221 - Nursing professionals	Low Income	West	Nursing Personnel
24	Medical Laboratory Scientist	1,135	1,260	1,416	1.71	5,862	Togo	Base Estimate	3212 - Medical and pathology laboratory technicians	Low Income	West	Other Health Workers
25	Medical Laboratory Technician	1,920	2,131	2,371	2.85	3,510	Togo	Base Estimate	3212 - Medical and pathology laboratory technicians	Low Income	West	Other Health Workers
26	Medical Social Worker	274	284	297	0.36	27,817	Togo	Base Estimate	1344 - Social welfare managers	Low Income	West	Other Health Workers
27	Mental Health Nurse	516	573	654	0.79	12,688	Togo	Base Estimate	2221 - Nursing professionals	Low Income	West	Nursing Personnel
28	Midwife	6,476	7,027	7,644	9.16	1,092	Togo	Base Estimate	2222 - Midwifery professionals	Low Income	West	Midwifery Personnel
29	Nephrologist	98	113	135	0.16	61,490	Togo	Base Estimate	2212 - Specialist medical practitioners	Low Income	West	Medical Doctors
30	Neuro-Surgeon	41	46	52	0.06	158,503	Togo	Base Estimate	2212 - Specialist medical practitioners	Low Income	West	Medical Doctors
31	Nurse Anaesthetist	213	251	297	0.36	28,007	Togo	Base Estimate	2221 - Nursing professionals	Low Income	West	Nursing Personnel
32	Nutritionist	965	993	1,019	1.23	8,157	Togo	Base Estimate	2265 - Dieticians and nutritionists	Low Income	West	Other Health Workers
33	Obstetrician & Gynaecologist	593	661	738	0.89	11,275	Togo	Base Estimate	2212 - Specialist medical practitioners	Low Income	West	Medical Doctors
34	Occupational Therapist	222	255	295	0.36	28,148	Togo	Base Estimate	2269 - Health professionals not elsewhere classified	Low Income	West	Other Health Workers
35	Oncology Nurse	72	84	101	0.12	82,221	Togo	Base Estimate	2221 - Nursing professionals	Low Income	West	Nursing Personnel
36	Operating Theatre Nurse	1,012	1,148	1,333	1.61	6,221	Togo	Base Estimate	2221 - Nursing professionals	Low Income	West	Nursing Personnel
37	Ophthalmic Nurse	113	129	149	0.18	55,880	Togo	Base Estimate	2221 - Nursing professionals	Low Income	West	Nursing Personnel
38	Ophthalmologist	31	36	43	0.05	192,568	Togo	Base Estimate	2212 - Specialist medical practitioners	Low Income	West	Medical Doctors
39	Optometrist	133	153	177	0.21	46,847	Togo	Base Estimate	2267 - Optometrists and ophthalmic opticians	Low Income	West	Other Health Workers
40	Orthopaedic Nurse	216	251	290	0.35	28,719	Togo	Base Estimate	2221 - Nursing professionals	Low Income	West	Nursing Personnel
41	Orthopaedic Surgeon	341	394	458	0.55	18,163	Togo	Base Estimate	2212 - Specialist medical practitioners	Low Income	West	Medical Doctors
42	Orthopaedic Technologist	274	306	341	0.41	24,422	Togo	Base Estimate	3214 - Medical and dental prosthetic technicians	Low Income	West	Other Health Workers

S/N	Health Professionals	2022	2026	2030	Density per 10,000 population	Required Population ratio (1 professional is to xxx population)	Name of Country	Modelling Scenario	ISCO-08 Match	Income Group Classification	Sub-Regional Grouping	SDG 3c Occupation
43	Paediatric Nurse	1,014	1,095	1,174	1.41	7,098	Togo	Base Estimate	2221 - Nursing professionals	Low Income	West	Nursing Personnel
44	Paediatric Surgeon	26	28	29	0.03	288,218	Togo	Base Estimate	2212 - Specialist medical practitioners	Low Income	West	Medical Doctors
45	Paediatrician	453	505	565	0.68	14,726	Togo	Base Estimate	2212 - Specialist medical practitioners	Low Income	West	Medical Doctors
46	Pathologist	63	72	83	0.10	99,827	Togo	Base Estimate	2212 - Specialist medical practitioners	Low Income	West	Medical Doctors
47	Pharmacist	556	575	601	0.73	13,753	Togo	Base Estimate	2262 - Pharmacists	Low Income	West	Pharmacist
48	Pharmacy Technician	929	1,027	1,139	1.37	7,307	Togo	Base Estimate	3213 - Pharmaceutical technicians and assistants	Low Income	West	Other Health Workers
49	Physician	1,018	1,136	1,274	1.53	6,526	Togo	Base Estimate	2212 - Specialist medical practitioners	Low Income	West	Medical Doctors
50	Physiotherapist	240	268	300	0.36	27,713	Togo	Base Estimate	2264 - Physiotherapists	Low Income	West	Other Health Workers
51	Plastic Surgeon	64	74	84	0.10	98,866	Togo	Base Estimate	2212 - Specialist medical practitioners	Low Income	West	Medical Doctors
52	Psychiatrist	473	521	585	0.70	14,201	Togo	Base Estimate	2212 - Specialist medical practitioners	Low Income	West	Medical Doctors
53	Radiation Oncologist	21	26	31	0.04	266,701	Togo	Base Estimate	2212 - Specialist medical practitioners	Low Income	West	Medical Doctors
54	Radiographer (Diagnostics and Therapy)	717	813	935	1.13	8,879	Togo	Base Estimate	3211 - Medical imaging and therapeutic equipment technicians	Low Income	West	Other Health Workers
55	Radiologist	249	284	328	0.40	25,265	Togo	Base Estimate	2212 - Specialist medical practitioners	Low Income	West	Medical Doctors
56	Registered General Nurse / State Certified Nurse	26,058	27,511	29,511	35.71	280	Togo	Base Estimate	2221 - Nursing professionals	Low Income	West	Nursing Personnel
57	Renal Nurse	1,287	1,490	1,777	2.15	4,654	Togo	Base Estimate	2221 - Nursing professionals	Low Income	West	Nursing Personnel
58	Respiratory Physician	81	91	103	0.12	80,702	Togo	Base Estimate	2212 - Specialist medical practitioners	Low Income	West	Medical Doctors
59	Rheumatologist	26	29	32	0.04	257,785	Togo	Base Estimate	2212 - Specialist medical practitioners	Low Income	West	Medical Doctors
60	Speech Therapist	87	96	106	0.13	78,615	Togo	Base Estimate	2266 - Audiologists and speech therapists	Low Income	West	Other Health Workers
61	Urologist	11	14	17	0.02	489,162	Togo	Base Estimate	2212 - Specialist medical practitioners	Low Income	West	Medical Doctors
1	Anaesthesiologist	402	462	530	0.11	86,983	Uganda	Base Estimate	2212 - Specialist medical practitioners	Low Income	East	Medical Doctors
2	Associate Nurse/Enrolled Nurse/Nursing Assistant	60,815	68,522	77,289	16.76	596	Uganda	Base Estimate	3221 - Nursing associate professionals	Low Income	East	Nursing Personnel
3	Audiologist	178	201	225	0.05	204,948	Uganda	Base Estimate	2266 - Audiologists and speech therapists	Low Income	East	Other Health Workers
4	Cardiologist	274	317	365	0.08	126,427	Uganda	Base Estimate	2212 - Specialist medical practitioners	Low Income	East	Medical Doctors
5	Cardiothoracic Surgeon	67	77	88	0.02	525,920	Uganda	Base Estimate	2212 - Specialist medical practitioners	Low Income	East	Medical Doctors
6	Clinical Officer/Physician Assistant	10,512	11,391	12,431	2.70	3,705	Uganda	Base Estimate	3256 - Medical assistants	Low Income	East	Other Health Workers
7	Clinical Pharmacist	1,275	1,414	1,601	0.35	28,560	Uganda	Base Estimate	2262 - Pharmacists	Low Income	East	Pharmacist
8	Clinical Psychologist	3,806	4,098	4,445	0.96	10,364	Uganda	Base Estimate	2634 - Psychologists	Low Income	East	Other Health Workers
9	Community health worker/Village health worker	56,118	61,302	66,610	14.43	693	Uganda	Base Estimate	3253 - Community health workers	Low Income	East	Other Health Workers
10	Dental Surgery Assistant	3,974	4,543	5,227	1.13	8,813	Uganda	Base Estimate	3251 - Dental assistants and therapists	Low Income	East	Other Health Workers
11	Dental Therapist	2,577	2,946	3,389	0.74	13,596	Uganda	Base Estimate	3251 - Dental assistants and therapists	Low Income	East	Other Health Workers
12	Dentist	2,899	3,291	3,844	0.84	11,952	Uganda	Base Estimate	2261 - Dentists	Low Income	East	Dentist
13	Dermatologist	150	170	191	0.04	241,483	Uganda	Base Estimate	2212 - Specialist medical practitioners	Low Income	East	Medical Doctors
14	Endocrinologist	136	161	201	0.04	225,840	Uganda	Base Estimate	2212 - Specialist medical practitioners	Low Income	East	Medical Doctors
15	ENT Surgeon	451	519	598	0.13	77,093	Uganda	Base Estimate	2212 - Specialist medical practitioners	Low Income	East	Medical Doctors
16	Environmental Health Officer	2,272	2,531	2,804	0.61	16,487	Uganda	Base Estimate	2263 - Environmental and occupational health and hygiene professionals	Low Income	East	Other Health Workers
17	Gastroenterologist	281	274	281	0.06	161,861	Uganda	Base Estimate	2212 - Specialist medical practitioners	Low Income	East	Medical Doctors
18	General Medical Practitioner (Generalist Doctor)	19,329	21,419	23,771	5.16	1,938	Uganda	Base Estimate	2211 - Generalist medical practitioners	Low Income	East	Medical Doctors
19	General Surgeon	337	397	479	0.10	95,578	Uganda	Base Estimate	2212 - Specialist medical practitioners	Low Income	East	Medical Doctors
20	Haematologist	123	134	146	0.03	315,363	Uganda	Base Estimate	2212 - Specialist medical practitioners	Low Income	East	Medical Doctors
21	Health Promoter/Health Educator	803	870	952	0.21	48,175	Uganda	Base Estimate	2269 - Health professionals not elsewhere classified	Low Income	East	Other Health Workers
22	Infectious Diseases Specialist	51	55	60	0.01	768,734	Uganda	Base Estimate	2212 - Specialist medical practitioners	Low Income	East	Medical Doctors
23	Intensive Care Nurse	1,342	1,463	1,588	0.34	29,078	Uganda	Base Estimate	2221 - Nursing professionals	Low Income	East	Nursing Personnel
24	Medical Laboratory Scientist	6,475	7,168	8,055	1.76	5,689	Uganda	Base Estimate	3212 - Medical and pathology laboratory technicians	Low Income	East	Other Health Workers
25	Medical Laboratory Technician	11,249	12,538	13,992	3.04	3,294	Uganda	Base Estimate	3212 - Medical and pathology laboratory technicians	Low Income	East	Other Health Workers
26	Medical Social Worker	1,187	1,276	1,408	0.32	31,696	Uganda	Base Estimate	1344 - Social welfare managers	Low Income	East	Other Health Workers
27	Mental Health Nurse	2,787	3,134	3,691	0.81	12,389	Uganda	Base Estimate	2221 - Nursing professionals	Low Income	East	Nursing Personnel
28	Midwife	31,821	35,435	39,684	8.61	1,162	Uganda	Base Estimate	2222 - Midwifery professionals	Low Income	East	Midwifery Personnel
29	Nephrologist	319	372	455	0.10	100,085	Uganda	Base Estimate	2212 - Specialist medical practitioners	Low Income	East	Medical Doctors
30	Neuro-Surgeon	122	141	164	0.04	279,700	Uganda	Base Estimate	2212 - Specialist medical practitioners	Low Income	East	Medical Doctors
31	Nurse Anaesthetist	1,084	1,305	1,569	0.34	29,297	Uganda	Base Estimate	2221 - Nursing professionals	Low Income	East	Nursing Personnel
32	Nutritionist	4,081	4,322	4,538	0.98	10,169	Uganda	Base Estimate	2265 - Dieticians and nutritionists	Low Income	East	Other Health Workers
33	Obstetrician & Gynaecologist	3,251	3,644	4,138	0.91	11,032	Uganda	Base Estimate	2212 - Specialist medical practitioners	Low Income	East	Medical Doctors
34	Occupational Therapist	691	798	933	0.20	49,249	Uganda	Base Estimate	2269 - Health professionals not elsewhere classified	Low Income	East	Other Health Workers
35	Oncology Nurse	312	371	453	0.10	100,820	Uganda	Base Estimate	2221 - Nursing professionals	Low Income	East	Nursing Personnel
36	Operating Theatre Nurse	3,916	4,507	5,390	1.18	8,476	Uganda	Base Estimate	2221 - Nursing professionals	Low Income	East	Nursing Personnel
37	Ophthalmic Nurse	386	446	524	0.11	87,644	Uganda	Base Estimate	2221 - Nursing professionals	Low Income	East	Nursing Personnel
38	Ophthalmologist	143	165	194	0.04	236,374	Uganda	Base Estimate	2212 - Specialist medical practitioners	Low Income	East	Medical Doctors
39	Optometrist	585	675	781	0.17	59,026	Uganda	Base Estimate	2267 - Optometrists and ophthalmic opticians	Low Income	East	Other Health Workers
40	Orthopaedic Nurse	616	717	829	0.18	55,663	Uganda	Base Estimate	2221 - Nursing professionals	Low Income	East	Nursing Personnel
41	Orthopaedic Surgeon	1,242	1,517	1,870	0.41	24,447	Uganda	Base Estimate	2212 - Specialist medical practitioners	Low Income	East	Medical Doctors
42	Orthopaedic Technologist	1,263	1,422	1,594	0.35	28,984	Uganda	Base Estimate	3214 - Medical and dental prosthetic technicians	Low Income	East	Other Health Workers
43	Paediatric Nurse	4,785	5,225	5,575	1.21	8,282	Uganda	Base Estimate	2221 - Nursing professionals	Low Income	East	Nursing Personnel
44	Paediatric Surgeon	183	202	217	0.05	213,130	Uganda	Base Estimate	2212 - Specialist medical practitioners	Low Income	East	Medical Doctors
45	Paediatrician	2,503	2,806	3,153	0.68	14,622	Uganda	Base Estimate	2212 - Specialist medical practitioners	Low Income	East	Medical Doctors
46	Pathologist	329	358	407	0.09	112,379	Uganda	Base Estimate	2212 - Specialist medical practitioners	Low Income	East	Medical Doctors
47	Pharmacist	5,140	5,487	5,876	1.28	7,822	Uganda	Base Estimate	2262 - Pharmacists	Low Income	East	Pharmacist
48	Pharmacy Technician	4,401	4,898	5,472	1.19	8,412	Uganda	Base Estimate	3213 - Pharmaceutical technicians and assistants	Low Income	East	Other Health Workers
49	Physician	4,147	4,687	5,339	1.16	8,626	Uganda	Base Estimate	2212 - Specialist medical practitioners	Low Income	East	Medical Doctors
50	Physiotherapist	1,112	1,252	1,413	0.31	32,642	Uganda	Base Estimate	2264 - Physiotherapists	Low Income	East	Other Health Workers
51	Plastic Surgeon	620	739	876	0.19	52,620	Uganda	Base Estimate	2212 - Specialist medical practitioners	Low Income	East	Medical Doctors
52	Psychiatrist	2,362	2,625	3,033	0.66	15,109	Uganda	Base Estimate	2212 - Specialist medical practitioners	Low Income	East	Medical Doctors
53	Radiation Oncologist	134	163	202	0.04	226,657	Uganda	Base Estimate	2212 - Specialist medical practitioners	Low Income	East	Medical Doctors
54	Radiographer (Diagnostics and Therapy)	2,633	3,059	3,670	0.81	12,382	Uganda	Base Estimate	3211 - Medical imaging and therapeutic equipment technicians	Low Income	East	Other Health Workers
55	Radiologist	982	1,181	1,479	0.33	29,948	Uganda	Base Estimate	2212 - Specialist medical practitioners	Low Income	East	Medical Doctors
56	Registered General Nurse / State Certified Nurse	104,422	113,897	125,751	27.46	364	Uganda	Base Estimate	2221 - Nursing professionals	Low Income	East	Nursing Personnel
57	Renal Nurse	4,214	4,912	6,026	1.32	7,559	Uganda	Base Estimate	2221 - Nursing professionals	Low Income	East	Nursing Personnel
58	Respiratory Physician	589	662	741	0.16	62,328	Uganda	Base Estimate	2212 - Specialist medical practitioners	Low Income	East	Medical Doctors
59	Rheumatologist	126	143	162	0.04	284,512	Uganda	Base Estimate	2212 - Specialist medical practitioners	Low Income	East	Medical Doctors
60	Speech Therapist	452	505	563	0.12	81,907	Uganda	Base Estimate	2266 - Audiologists and speech therapists	Low Income	East	Other Health Workers
61	Urologist	39	48	65	0.01	685,601	Uganda	Base Estimate	2212 - Specialist medical practitioners	Low Income	East	Medical Doctors
1	Anaesthesiologist	578	671	777	0.13	77,375	United Republic of	Base Estimate	2212 - Specialist medical practitioners	Lower-middle Income	East	Medical Doctors
2	Associate Nurse/Enrolled Nurse/Nursing Assistant	69,281	76,987	85,461	14.20	704	United Republic of	Base Estimate	3221 - Nursing associate professionals	Lower-middle Income	East	Nursing Personnel
3	Audiologist	240	271	304	0.05	198,363	United Republic of	Base Estimate	2266 - Audiologists and speech therapists	Lower-middle Income	East	Other Health Workers
4	Cardiologist	479	560	651	0.11	92,485	United Republic of	Base Estimate	2212 - Specialist medical practitioners	Lower-middle Income	East	Medical Doctors

S/N	Health Professionals	2022	2026	2030	Density per 10,000 population	Required Population ratio (1 professional is to xxx population)	Name of Country	Modelling Scenario	ISCO-08 Match	Income Group Classification	Sub-Regional Grouping	SDG 3c Occupation
5	Cardiothoracic Surgeon	93	108	124	0.02	484,400	United Republic of	Base Estimate	2212 - Specialist medical practitioners	Lower-middle Income	East	Medical Doctors
6	Clinical Officer/Physician Assistant	5,813	6,171	6,639	1.11	9,009	United Republic of	Base Estimate	3256 - Medical assistants	Lower-middle Income	East	Other Health Workers
7	Clinical Pharmacist	1,705	1,917	2,167	0.36	27,739	United Republic of	Base Estimate	2262 - Pharmacists	Lower-middle Income	East	Pharmacist
8	Clinical Psychologist	4,021	4,453	4,939	0.82	12,183	United Republic of	Base Estimate	2634 - Psychologists	Lower-middle Income	East	Other Health Workers
9	Community health worker/Village health worker	45,557	49,638	53,932	8.96	1,116	United Republic of	Base Estimate	3253 - Community health workers	Lower-middle Income	East	Other Health Workers
10	Dental Surgery Assistant	6,160	7,038	8,060	1.34	7,476	United Republic of	Base Estimate	3251 - Dental assistants and therapists	Lower-middle Income	East	Other Health Workers
11	Dental Therapist	3,987	4,555	5,215	0.87	11,553	United Republic of	Base Estimate	3251 - Dental assistants and therapists	Lower-middle Income	East	Other Health Workers
12	Dentist	4,837	5,494	6,309	1.05	9,551	United Republic of	Base Estimate	2261 - Dentists	Lower-middle Income	East	Dentist
13	Dermatologist	205	233	263	0.04	229,442	United Republic of	Base Estimate	2212 - Specialist medical practitioners	Lower-middle Income	East	Medical Doctors
14	Endocrinologist	163	192	232	0.04	257,359	United Republic of	Base Estimate	2212 - Specialist medical practitioners	Lower-middle Income	East	Medical Doctors
15	ENT Surgeon	659	757	869	0.14	69,174	United Republic of	Base Estimate	2212 - Specialist medical practitioners	Lower-middle Income	East	Medical Doctors
16	Environmental Health Officer	2,967	3,325	3,703	0.61	16,282	United Republic of	Base Estimate	2263 - Environmental and occupational health and hygiene professionals	Lower-middle Income	East	Other Health Workers
17	Gastroenterologist	365	380	405	0.07	147,411	United Republic of	Base Estimate	2212 - Specialist medical practitioners	Lower-middle Income	East	Medical Doctors
18	General Medical Practitioner (Generalist Doctor)	21,099	23,613	26,399	4.39	2,280	United Republic of	Base Estimate	2211 - Generalist medical practitioners	Lower-middle Income	East	Medical Doctors
19	General Surgeon	475	554	651	0.11	92,228	United Republic of	Base Estimate	2212 - Specialist medical practitioners	Lower-middle Income	East	Medical Doctors
20	Haematologist	295	335	378	0.06	159,569	United Republic of	Base Estimate	2212 - Specialist medical practitioners	Lower-middle Income	East	Medical Doctors
21	Health Promoter/Health Educator	453	477	509	0.09	117,527	United Republic of	Base Estimate	2269 - Health professionals not elsewhere classified	Lower-middle Income	East	Other Health Workers
22	Infectious Diseases Specialist	62	69	77	0.01	779,083	United Republic of	Base Estimate	2212 - Specialist medical practitioners	Lower-middle Income	East	Medical Doctors
23	Intensive Care Nurse	1,774	2,003	2,251	0.37	26,764	United Republic of	Base Estimate	2221 - Nursing professionals	Lower-middle Income	East	Nursing Personnel
24	Medical Laboratory Scientist	6,590	7,370	8,292	1.38	7,242	United Republic of	Base Estimate	3212 - Medical and pathology laboratory technicians	Lower-middle Income	East	Other Health Workers
25	Medical Laboratory Technician	10,466	11,704	13,083	2.17	4,598	United Republic of	Base Estimate	3212 - Medical and pathology laboratory technicians	Lower-middle Income	East	Other Health Workers
26	Medical Social Worker	1,575	1,683	1,796	0.30	33,469	United Republic of	Base Estimate	1344 - Social welfare managers	Lower-middle Income	East	Other Health Workers
27	Mental Health Nurse	3,515	4,005	4,648	0.78	12,891	United Republic of	Base Estimate	2221 - Nursing professionals	Lower-middle Income	East	Nursing Personnel
28	Midwife	56,929	64,170	72,192	11.97	835	United Republic of	Base Estimate	2222 - Midwifery professionals	Lower-middle Income	East	Midwifery Personnel
29	Nephrologist	534	624	743	0.12	80,432	United Republic of	Base Estimate	2212 - Specialist medical practitioners	Lower-middle Income	East	Medical Doctors
30	Neuro-Surgeon	163	192	228	0.04	262,347	United Republic of	Base Estimate	2212 - Specialist medical practitioners	Lower-middle Income	East	Medical Doctors
31	Nurse Anaesthetist	1,449	1,711	2,014	0.34	29,843	United Republic of	Base Estimate	2221 - Nursing professionals	Lower-middle Income	East	Nursing Personnel
32	Nutritionist	6,133	6,613	7,089	1.18	8,498	United Republic of	Base Estimate	2265 - Dieticians and nutritionists	Lower-middle Income	East	Other Health Workers
33	Obstetrician & Gynaecologist	4,227	4,761	5,356	0.89	11,244	United Republic of	Base Estimate	2212 - Specialist medical practitioners	Lower-middle Income	East	Medical Doctors
34	Occupational Therapist	989	1,148	1,340	0.22	44,792	United Republic of	Base Estimate	2269 - Health professionals not elsewhere classified	Lower-middle Income	East	Other Health Workers
35	Oncology Nurse	466	547	649	0.11	92,210	United Republic of	Base Estimate	2221 - Nursing professionals	Lower-middle Income	East	Nursing Personnel
36	Operating Theatre Nurse	5,767	6,612	7,704	1.29	7,771	United Republic of	Base Estimate	2221 - Nursing professionals	Lower-middle Income	East	Nursing Personnel
37	Ophthalmic Nurse	649	734	832	0.14	72,249	United Republic of	Base Estimate	2221 - Nursing professionals	Lower-middle Income	East	Nursing Personnel
38	Ophthalmologist	271	312	360	0.06	166,968	United Republic of	Base Estimate	2212 - Specialist medical practitioners	Lower-middle Income	East	Medical Doctors
39	Optometrist	1,582	1,814	2,072	0.34	29,069	United Republic of	Base Estimate	2267 - Optometrists and ophthalmic opticians	Lower-middle Income	East	Other Health Workers
40	Orthopaedic Nurse	853	1,002	1,172	0.19	51,306	United Republic of	Base Estimate	2221 - Nursing professionals	Lower-middle Income	East	Nursing Personnel
41	Orthopaedic Surgeon	1,894	2,210	2,580	0.43	23,282	United Republic of	Base Estimate	2212 - Specialist medical practitioners	Lower-middle Income	East	Medical Doctors
42	Orthopaedic Technologist	1,691	1,918	2,165	0.36	27,828	United Republic of	Base Estimate	3214 - Medical and dental prosthetic technicians	Lower-middle Income	East	Other Health Workers
43	Paediatric Nurse	7,794	8,680	9,568	1.59	6,304	United Republic of	Base Estimate	2221 - Nursing professionals	Lower-middle Income	East	Nursing Personnel
44	Paediatric Surgeon	228	252	276	0.05	218,904	United Republic of	Base Estimate	2212 - Specialist medical practitioners	Lower-middle Income	East	Medical Doctors
45	Paediatrician	3,917	4,358	4,840	0.80	12,440	United Republic of	Base Estimate	2212 - Specialist medical practitioners	Lower-middle Income	East	Medical Doctors
46	Pathologist	438	498	577	0.10	103,786	United Republic of	Base Estimate	2212 - Specialist medical practitioners	Lower-middle Income	East	Medical Doctors
47	Pharmacist	3,097	3,276	3,486	0.58	17,181	United Republic of	Base Estimate	2262 - Pharmacists	Lower-middle Income	East	Pharmacist
48	Pharmacy Technician	5,119	5,772	6,508	1.08	9,246	United Republic of	Base Estimate	3213 - Pharmaceutical technicians and assistants	Lower-middle Income	East	Other Health Workers
49	Physician	6,072	6,894	7,829	1.30	7,682	United Republic of	Base Estimate	2212 - Specialist medical practitioners	Lower-middle Income	East	Medical Doctors
50	Physiotherapist	1,527	1,732	1,965	0.33	30,619	United Republic of	Base Estimate	2264 - Physiotherapists	Lower-middle Income	East	Other Health Workers
51	Plastic Surgeon	946	1,119	1,316	0.22	45,677	United Republic of	Base Estimate	2212 - Specialist medical practitioners	Lower-middle Income	East	Medical Doctors
52	Psychiatrist	3,023	3,422	3,933	0.66	15,256	United Republic of	Base Estimate	2212 - Specialist medical practitioners	Lower-middle Income	East	Medical Doctors
53	Radiation Oncologist	177	211	253	0.04	236,563	United Republic of	Base Estimate	2212 - Specialist medical practitioners	Lower-middle Income	East	Medical Doctors
54	Radiographer (Diagnostics and Therapy)	3,867	4,460	5,187	0.86	11,568	United Republic of	Base Estimate	3211 - Medical imaging and therapeutic equipment technicians	Lower-middle Income	East	Other Health Workers
55	Radiologist	1,387	1,597	1,845	0.31	32,571	United Republic of	Base Estimate	2212 - Specialist medical practitioners	Lower-middle Income	East	Medical Doctors
56	Registered General Nurse / State Certified Nurse	139,264	153,388	170,346	28.44	352	United Republic of	Base Estimate	2221 - Nursing professionals	Lower-middle Income	East	Nursing Personnel
57	Renal Nurse	7,054	8,237	9,811	1.64	6,090	United Republic of	Base Estimate	2221 - Nursing professionals	Lower-middle Income	East	Nursing Personnel
58	Respiratory Physician	871	959	1,051	0.17	57,334	United Republic of	Base Estimate	2212 - Specialist medical practitioners	Lower-middle Income	East	Medical Doctors
59	Rheumatologist	182	208	238	0.04	253,387	United Republic of	Base Estimate	2212 - Specialist medical practitioners	Lower-middle Income	East	Medical Doctors
60	Speech Therapist	635	717	811	0.13	74,075	United Republic of	Base Estimate	2266 - Audiologists and speech therapists	Lower-middle Income	East	Other Health Workers
61	Urologist	61	72	86	0.01	698,028	United Republic of	Base Estimate	2212 - Specialist medical practitioners	Lower-middle Income	East	Medical Doctors
1	Anaesthesiologist	144	166	190	0.10	97,151	Zambia	Base Estimate	2212 - Specialist medical practitioners	Lower-middle Income	Southern	Medical Doctors
2	Associate Nurse/Enrolled Nurse/Nursing Assistant	24,600	27,479	30,749	16.59	603	Zambia	Base Estimate	3221 - Nursing associate professionals	Lower-middle Income	Southern	Nursing Personnel
3	Audiologist	70	79	88	0.05	210,409	Zambia	Base Estimate	2266 - Audiologists and speech therapists	Lower-middle Income	Southern	Other Health Workers
4	Cardiologist	115	134	154	0.08	120,438	Zambia	Base Estimate	2212 - Specialist medical practitioners	Lower-middle Income	Southern	Medical Doctors
5	Cardiothoracic Surgeon	23	26	30	0.02	623,831	Zambia	Base Estimate	2212 - Specialist medical practitioners	Lower-middle Income	Southern	Medical Doctors
6	Clinical Officer/Physician Assistant	3,186	3,339	3,564	1.94	5,161	Zambia	Base Estimate	3256 - Medical assistants	Lower-middle Income	Southern	Other Health Workers
7	Clinical Pharmacist	563	641	742	0.40	24,853	Zambia	Base Estimate	2262 - Pharmacists	Lower-middle Income	Southern	Pharmacist
8	Clinical Psychologist	1,503	1,711	1,961	1.06	9,441	Zambia	Base Estimate	2634 - Psychologists	Lower-middle Income	Southern	Other Health Workers
9	Community health worker/Village health worker	18,307	20,150	22,159	11.95	837	Zambia	Base Estimate	3253 - Community health workers	Lower-middle Income	Southern	Other Health Workers
10	Dental Surgery Assistant	1,861	2,133	2,455	1.32	7,556	Zambia	Base Estimate	3251 - Dental assistants and therapists	Lower-middle Income	Southern	Other Health Workers
11	Dental Therapist	1,206	1,382	1,591	0.86	11,660	Zambia	Base Estimate	3251 - Dental assistants and therapists	Lower-middle Income	Southern	Other Health Workers
12	Dentist	1,350	1,524	1,750	0.94	10,609	Zambia	Base Estimate	2261 - Dentists	Lower-middle Income	Southern	Dentist
13	Dermatologist	63	71	80	0.04	231,767	Zambia	Base Estimate	2212 - Specialist medical practitioners	Lower-middle Income	Southern	Medical Doctors
14	Endocrinologist	56	67	83	0.05	220,239	Zambia	Base Estimate	2212 - Specialist medical practitioners	Lower-middle Income	Southern	Medical Doctors
15	ENT Surgeon	193	222	255	0.14	72,444	Zambia	Base Estimate	2212 - Specialist medical practitioners	Lower-middle Income	Southern	Medical Doctors
16	Environmental Health Officer	913	1,016	1,124	0.61	16,517	Zambia	Base Estimate	2263 - Environmental and occupational health and hygiene professionals	Lower-middle Income	Southern	Other Health Workers
17	Gastroenterologist	186	193	209	0.11	88,284	Zambia	Base Estimate	2212 - Specialist medical practitioners	Lower-middle Income	Southern	Medical Doctors
18	General Medical Practitioner (Generalist Doctor)	7,679	8,525	9,503	5.13	1,948	Zambia	Base Estimate	2211 - Generalist medical practitioners	Lower-middle Income	Southern	Medical Doctors
19	General Surgeon	146	170	202	0.11	91,462	Zambia	Base Estimate	2212 - Specialist medical practitioners	Lower-middle Income	Southern	Medical Doctors
20	Haematologist	110	124	138	0.07	134,627	Zambia	Base Estimate	2212 - Specialist medical practitioners	Lower-middle Income	Southern	Medical Doctors
21	Health Promoter/Health Educator	239	251	270	0.15	67,786	Zambia	Base Estimate	2269 - Health professionals not elsewhere classified	Lower-middle Income	Southern	Other Health Workers
22	Infectious Diseases Specialist	23	25	28	0.01	670,951	Zambia	Base Estimate	2212 - Specialist medical practitioners	Lower-middle Income	Southern	Medical Doctors
23	Intensive Care Nurse	570	635	704	0.38	26,360	Zambia	Base Estimate	2221 - Nursing professionals	Lower-middle Income	Southern	Nursing Personnel
24	Medical Laboratory Scientist	2,700	3,023	3,430	1.86	5,380	Zambia	Base Estimate	3212 - Medical and pathology laboratory technicians	Lower-middle Income	Southern	Other Health Workers
25	Medical Laboratory Technician	2,952	3,328	3,778	2.05	4,888	Zambia	Base Estimate	3212 - Medical and pathology laboratory technicians	Lower-middle Income	Southern	Other Health Workers
26	Medical Social Worker	619	672	748	0.41	24,386	Zambia	Base Estimate	1344 - Social welfare managers	Lower-middle Income	Southern	Other Health Workers
27	Mental Health Nurse	1,097	1,257	1,492	0.81	12,363	Zambia	Base Estimate	2221 - Nursing professionals	Lower-middle Income	Southern	Nursing Personnel

S/N	Health Professionals	2022	2026	2030	Density per 10,000 population	Required Population ratio (1 professional is to xxx population)	Name of Country	Modelling Scenario	ISCO-08 Match	Income Group Classification	Sub-Regional Grouping	SDG 3c Occupation
28	Midwife	13,234	14,619	16,245	8.75	1,143	Zambia	Base Estimate	2222 - Midwifery professionals	Lower-middle Income	Southern	Midwifery Personnel
29	Nephrologist	163	192	236	0.13	77,899	Zambia	Base Estimate	2212 - Specialist medical practitioners	Lower-middle Income	Southern	Medical Doctors
30	Neuro-Surgeon	41	47	54	0.03	336,589	Zambia	Base Estimate	2212 - Specialist medical practitioners	Lower-middle Income	Southern	Medical Doctors
31	Nurse Anaesthetist	436	520	620	0.34	29,829	Zambia	Base Estimate	2221 - Nursing professionals	Lower-middle Income	Southern	Nursing Personnel
32	Nutritionist	2,165	2,326	2,489	1.34	7,452	Zambia	Base Estimate	2265 - Dieticians and nutritionists	Lower-middle Income	Southern	Other Health Workers
33	Obstetrician & Gynaecologist	1,348	1,524	1,739	0.94	10,594	Zambia	Base Estimate	2212 - Specialist medical practitioners	Lower-middle Income	Southern	Medical Doctors
34	Occupational Therapist	235	273	322	0.17	57,361	Zambia	Base Estimate	2269 - Health professionals not elsewhere classified	Lower-middle Income	Southern	Other Health Workers
35	Oncology Nurse	144	172	209	0.11	88,072	Zambia	Base Estimate	2221 - Nursing professionals	Lower-middle Income	Southern	Nursing Personnel
36	Operating Theatre Nurse	1,702	1,986	2,395	1.30	7,686	Zambia	Base Estimate	2221 - Nursing professionals	Lower-middle Income	Southern	Nursing Personnel
37	Ophthalmic Nurse	178	203	233	0.13	79,248	Zambia	Base Estimate	2221 - Nursing professionals	Lower-middle Income	Southern	Nursing Personnel
38	Ophthalmologist	60	70	82	0.04	225,190	Zambia	Base Estimate	2212 - Specialist medical practitioners	Lower-middle Income	Southern	Medical Doctors
39	Optometrist	241	278	321	0.17	57,734	Zambia	Base Estimate	2267 - Optometrists and ophthalmic opticians	Lower-middle Income	Southern	Other Health Workers
40	Orthopaedic Nurse	181	211	245	0.13	75,623	Zambia	Base Estimate	2221 - Nursing professionals	Lower-middle Income	Southern	Nursing Personnel
41	Orthopaedic Surgeon	598	715	861	0.47	21,437	Zambia	Base Estimate	2212 - Specialist medical practitioners	Lower-middle Income	Southern	Medical Doctors
42	Orthopaedic Technologist	485	545	609	0.33	30,480	Zambia	Base Estimate	3214 - Medical and dental prosthetic technicians	Lower-middle Income	Southern	Other Health Workers
43	Paediatric Nurse	2,439	2,698	2,968	1.60	6,259	Zambia	Base Estimate	2221 - Nursing professionals	Lower-middle Income	Southern	Nursing Personnel
44	Paediatric Surgeon	63	68	72	0.04	256,117	Zambia	Base Estimate	2212 - Specialist medical practitioners	Lower-middle Income	Southern	Medical Doctors
45	Paediatrician	863	1,000	1,166	0.63	15,863	Zambia	Base Estimate	2212 - Specialist medical practitioners	Lower-middle Income	Southern	Medical Doctors
46	Pathologist	151	165	189	0.10	97,957	Zambia	Base Estimate	2212 - Specialist medical practitioners	Lower-middle Income	Southern	Medical Doctors
47	Pharmacist	1,443	1,450	1,481	0.81	12,339	Zambia	Base Estimate	2262 - Pharmacists	Lower-middle Income	Southern	Pharmacist
48	Pharmacy Technician	1,756	1,938	2,154	1.17	8,573	Zambia	Base Estimate	3213 - Pharmaceutical technicians and assistants	Lower-middle Income	Southern	Other Health Workers
49	Physician	1,745	1,985	2,277	1.23	8,127	Zambia	Base Estimate	2212 - Specialist medical practitioners	Lower-middle Income	Southern	Medical Doctors
50	Physiotherapist	441	497	561	0.30	33,012	Zambia	Base Estimate	2264 - Physiotherapists	Lower-middle Income	Southern	Other Health Workers
51	Plastic Surgeon	242	279	320	0.17	57,968	Zambia	Base Estimate	2212 - Specialist medical practitioners	Lower-middle Income	Southern	Medical Doctors
52	Psychiatrist	942	1,066	1,241	0.67	14,885	Zambia	Base Estimate	2212 - Specialist medical practitioners	Lower-middle Income	Southern	Medical Doctors
53	Radiation Oncologist	58	71	87	0.05	211,266	Zambia	Base Estimate	2212 - Specialist medical practitioners	Lower-middle Income	Southern	Medical Doctors
54	Radiographer (Diagnostics and Therapy)	1,243	1,447	1,724	0.94	10,661	Zambia	Base Estimate	3211 - Medical imaging and therapeutic equipment technicians	Lower-middle Income	Southern	Other Health Workers
55	Radiologist	451	536	655	0.36	27,633	Zambia	Base Estimate	2212 - Specialist medical practitioners	Lower-middle Income	Southern	Medical Doctors
56	Registered General Nurse / State Certified Nurse	44,955	49,249	54,866	29.76	336	Zambia	Base Estimate	2221 - Nursing professionals	Lower-middle Income	Southern	Nursing Personnel
57	Renal Nurse	2,139	2,523	3,100	1.69	5,931	Zambia	Base Estimate	2221 - Nursing professionals	Lower-middle Income	Southern	Nursing Personnel
58	Respiratory Physician	141	169	202	0.11	91,319	Zambia	Base Estimate	2212 - Specialist medical practitioners	Lower-middle Income	Southern	Medical Doctors
59	Rheumatologist	54	62	71	0.04	262,951	Zambia	Base Estimate	2212 - Specialist medical practitioners	Lower-middle Income	Southern	Medical Doctors
60	Speech Therapist	185	206	230	0.12	80,470	Zambia	Base Estimate	2266 - Audiologists and speech therapists	Lower-middle Income	Southern	Other Health Workers
61	Urologist	18	22	28	0.02	641,572	Zambia	Base Estimate	2212 - Specialist medical practitioners	Lower-middle Income	Southern	Medical Doctors
1	Anaesthesiologist	160	185	213	0.14	70,142	Zimbabwe	Base Estimate	2212 - Specialist medical practitioners	Lower-middle Income	Southern	Medical Doctors
2	Associate Nurse/Enrolled Nurse/Nursing Assistant	19,786	21,637	23,742	15.91	629	Zimbabwe	Base Estimate	3221 - Nursing associate professionals	Lower-middle Income	Southern	Nursing Personnel
3	Audiologist	67	74	82	0.05	182,976	Zimbabwe	Base Estimate	2266 - Audiologists and speech therapists	Lower-middle Income	Southern	Other Health Workers
4	Cardiologist	143	161	180	0.12	83,023	Zimbabwe	Base Estimate	2212 - Specialist medical practitioners	Lower-middle Income	Southern	Medical Doctors
5	Cardiothoracic Surgeon	18	21	24	0.02	619,891	Zimbabwe	Base Estimate	2212 - Specialist medical practitioners	Lower-middle Income	Southern	Medical Doctors
6	Clinical Officer/Physician Assistant	1,676	1,757	1,893	1.28	7,803	Zimbabwe	Base Estimate	3256 - Medical assistants	Lower-middle Income	Southern	Other Health Workers
7	Clinical Pharmacist	480	528	594	0.40	24,903	Zimbabwe	Base Estimate	2262 - Pharmacists	Lower-middle Income	Southern	Pharmacist
8	Clinical Psychologist	1,205	1,265	1,339	0.90	11,120	Zimbabwe	Base Estimate	2634 - Psychologists	Lower-middle Income	Southern	Other Health Workers
9	Community health worker/Village health worker	9,266	9,791	10,305	6.91	1,446	Zimbabwe	Base Estimate	3253 - Community health workers	Lower-middle Income	Southern	Other Health Workers
10	Dental Surgery Assistant	1,593	1,778	2,001	1.34	7,450	Zimbabwe	Base Estimate	3251 - Dental assistants and therapists	Lower-middle Income	Southern	Other Health Workers
11	Dental Therapist	1,032	1,152	1,297	0.87	11,497	Zimbabwe	Base Estimate	3251 - Dental assistants and therapists	Lower-middle Income	Southern	Other Health Workers
12	Dentist	1,135	1,246	1,408	0.95	10,534	Zimbabwe	Base Estimate	2261 - Dentists	Lower-middle Income	Southern	Dentist
13	Dermatologist	58	64	71	0.05	212,135	Zimbabwe	Base Estimate	2212 - Specialist medical practitioners	Lower-middle Income	Southern	Medical Doctors
14	Endocrinologist	90	105	128	0.09	114,614	Zimbabwe	Base Estimate	2212 - Specialist medical practitioners	Lower-middle Income	Southern	Medical Doctors
15	ENT Surgeon	191	214	241	0.16	61,973	Zimbabwe	Base Estimate	2212 - Specialist medical practitioners	Lower-middle Income	Southern	Medical Doctors
16	Environmental Health Officer	738	802	867	0.58	17,289	Zimbabwe	Base Estimate	2263 - Environmental and occupational health and hygiene professionals	Lower-middle Income	Southern	Other Health Workers
17	Gastroenterologist	169	167	173	0.12	84,817	Zimbabwe	Base Estimate	2212 - Specialist medical practitioners	Lower-middle Income	Southern	Medical Doctors
18	General Medical Practitioner (Generalist Doctor)	5,925	6,459	7,073	4.74	2,108	Zimbabwe	Base Estimate	2211 - Generalist medical practitioners	Lower-middle Income	Southern	Medical Doctors
19	General Surgeon	248	282	330	0.22	44,866	Zimbabwe	Base Estimate	2212 - Specialist medical practitioners	Lower-middle Income	Southern	Medical Doctors
20	Haematologist	47	51	56	0.04	267,598	Zimbabwe	Base Estimate	2212 - Specialist medical practitioners	Lower-middle Income	Southern	Medical Doctors
21	Health Promoter/Health Educator	125	132	142	0.10	103,404	Zimbabwe	Base Estimate	2269 - Health professionals not elsewhere classified	Lower-middle Income	Southern	Other Health Workers
22	Infectious Diseases Specialist	25	27	29	0.02	515,792	Zimbabwe	Base Estimate	2212 - Specialist medical practitioners	Lower-middle Income	Southern	Medical Doctors
23	Intensive Care Nurse	439	488	542	0.36	27,628	Zimbabwe	Base Estimate	2221 - Nursing professionals	Lower-middle Income	Southern	Nursing Personnel
24	Medical Laboratory Scientist	2,234	2,443	2,728	1.84	5,425	Zimbabwe	Base Estimate	3212 - Medical and pathology laboratory technicians	Lower-middle Income	Southern	Other Health Workers
25	Medical Laboratory Technician	1,930	2,102	2,318	1.56	6,407	Zimbabwe	Base Estimate	3212 - Medical and pathology laboratory technicians	Lower-middle Income	Southern	Other Health Workers
26	Medical Social Worker	511	537	577	0.40	25,311	Zimbabwe	Base Estimate	1344 - Social welfare managers	Lower-middle Income	Southern	Other Health Workers
27	Mental Health Nurse	781	859	979	0.66	15,084	Zimbabwe	Base Estimate	2221 - Nursing professionals	Lower-middle Income	Southern	Nursing Personnel
28	Midwife	11,368	12,330	13,452	9.03	1,108	Zimbabwe	Base Estimate	2222 - Midwifery professionals	Lower-middle Income	Southern	Midwifery Personnel
29	Nephrologist	202	237	290	0.20	50,538	Zimbabwe	Base Estimate	2212 - Specialist medical practitioners	Lower-middle Income	Southern	Medical Doctors
30	Neuro-Surgeon	53	60	68	0.05	218,511	Zimbabwe	Base Estimate	2212 - Specialist medical practitioners	Lower-middle Income	Southern	Medical Doctors
31	Nurse Anaesthetist	501	591	697	0.47	21,417	Zimbabwe	Base Estimate	2221 - Nursing professionals	Lower-middle Income	Southern	Nursing Personnel
32	Nutritionist	1,515	1,594	1,666	1.12	8,968	Zimbabwe	Base Estimate	2265 - Dieticians and nutritionists	Lower-middle Income	Southern	Other Health Workers
33	Obstetrician & Gynaecologist	1,284	1,417	1,582	1.06	9,390	Zimbabwe	Base Estimate	2212 - Specialist medical practitioners	Lower-middle Income	Southern	Medical Doctors
34	Occupational Therapist	233	270	320	0.22	46,290	Zimbabwe	Base Estimate	2269 - Health professionals not elsewhere classified	Lower-middle Income	Southern	Other Health Workers
35	Oncology Nurse	168	198	238	0.16	61,942	Zimbabwe	Base Estimate	2221 - Nursing professionals	Lower-middle Income	Southern	Nursing Personnel
36	Operating Theatre Nurse	1,995	2,272	2,657	1.80	5,565	Zimbabwe	Base Estimate	2221 - Nursing professionals	Lower-middle Income	Southern	Nursing Personnel
37	Ophthalmic Nurse	279	313	356	0.24	41,837	Zimbabwe	Base Estimate	2221 - Nursing professionals	Lower-middle Income	Southern	Nursing Personnel
38	Ophthalmologist	88	100	116	0.08	127,892	Zimbabwe	Base Estimate	2212 - Specialist medical practitioners	Lower-middle Income	Southern	Medical Doctors
39	Optometrist	452	514	586	0.39	25,484	Zimbabwe	Base Estimate	2267 - Optometrists and ophthalmic opticians	Lower-middle Income	Southern	Other Health Workers
40	Orthopaedic Nurse	158	190	226	0.15	66,023	Zimbabwe	Base Estimate	2221 - Nursing professionals	Lower-middle Income	Southern	Nursing Personnel
41	Orthopaedic Surgeon	591	682	791	0.53	18,833	Zimbabwe	Base Estimate	2212 - Specialist medical practitioners	Lower-middle Income	Southern	Medical Doctors
42	Orthopaedic Technologist	389	430	475	0.32	31,493	Zimbabwe	Base Estimate	3214 - Medical and dental prosthetic technicians	Lower-middle Income	Southern	Other Health Workers
43	Paediatric Nurse	1,461	1,573	1,660	1.11	8,974	Zimbabwe	Base Estimate	2221 - Nursing professionals	Lower-middle Income	Southern	Nursing Personnel
44	Paediatric Surgeon	51	56	59	0.04	253,403	Zimbabwe	Base Estimate	2212 - Specialist medical practitioners	Lower-middle Income	Southern	Medical Doctors
45	Paediatrician	559	635	729	0.49	20,368	Zimbabwe	Base Estimate	2212 - Specialist medical practitioners	Lower-middle Income	Southern	Medical Doctors
46	Pathologist	154	168	190	0.13	77,733	Zimbabwe	Base Estimate	2212 - Specialist medical practitioners	Lower-middle Income	Southern	Medical Doctors
47	Pharmacist	782	814	868	0.59	16,949	Zimbabwe	Base Estimate	2262 - Pharmacists	Lower-middle Income	Southern	Pharmacist
48	Pharmacy Technician	1,442	1,586	1,755	1.18	8,490	Zimbabwe	Base Estimate	3213 - Pharmaceutical technicians and assistants	Lower-middle Income	Southern	Other Health Workers
49	Physician	1,705	1,899	2,139	1.44	6,958	Zimbabwe	Base Estimate	2212 - Specialist medical practitioners	Lower-middle Income	Southern	Medical Doctors
50	Physiotherapist	376	416	464	0.31	32,197	Zimbabwe	Base Estimate	2264 - Physiotherapists	Lower-middle Income	Southern	Other Health Workers

S/N	Health Professionals	2022	2026	2030	Density per 10,000 population	Required Population ratio (1 professional is to xxx population)	Name of Country	Modelling Scenario	ISCO-08 Match	Income Group Classification	Sub-Regional Grouping	SDG 3c Occupation
51	Plastic Surgeon	131	147	164	0.11	91,444	Zimbabwe	Base Estimate	2212 - Specialist medical practitioners	Lower-middle Income	Southern	Medical Doctors
52	Psychiatrist	728	798	901	0.61	16,423	Zimbabwe	Base Estimate	2212 - Specialist medical practitioners	Lower-middle Income	Southern	Medical Doctors
53	Radiation Oncologist	65	77	92	0.06	160,743	Zimbabwe	Base Estimate	2212 - Specialist medical practitioners	Lower-middle Income	Southern	Medical Doctors
54	Radiographer (Diagnostics and Therapy)	1,404	1,595	1,856	1.26	7,958	Zimbabwe	Base Estimate	3211 - Medical imaging and therapeutic equipment technicians	Lower-middle Income	Southern	Other Health Workers
55	Radiologist	517	597	706	0.48	20,780	Zimbabwe	Base Estimate	2212 - Specialist medical practitioners	Lower-middle Income	Southern	Medical Doctors
56	Registered General Nurse / State Certified Nurse	34,633	38,492	43,311	29.22	342	Zimbabwe	Base Estimate	2221 - Nursing professionals	Lower-middle Income	Southern	Nursing Personnel
57	Renal Nurse	2,634	3,097	3,785	2.58	3,875	Zimbabwe	Base Estimate	2221 - Nursing professionals	Lower-middle Income	Southern	Nursing Personnel
58	Respiratory Physician	90	103	119	0.08	124,916	Zimbabwe	Base Estimate	2212 - Specialist medical practitioners	Lower-middle Income	Southern	Medical Doctors
59	Rheumatologist	49	55	61	0.04	246,554	Zimbabwe	Base Estimate	2212 - Specialist medical practitioners	Lower-middle Income	Southern	Medical Doctors
60	Speech Therapist	154	169	185	0.12	81,003	Zimbabwe	Base Estimate	2266 - Audiologists and speech therapists	Lower-middle Income	Southern	Other Health Workers
61	Urologist	23	27	33	0.02	440,372	Zimbabwe	Base Estimate	2212 - Specialist medical practitioners	Lower-middle Income	Southern	Medical Doctors
1	Anaesthesiologist	397	434	472	0.11	93,165	Algeria	Minimum Estimate	2212 - Specialist medical practitioners	High Income and Upper Middle Income	West	Medical Doctors
2	Associate Nurse/Enrolled Nurse/Nursing Assistant	43,142	46,867	50,006	11.35	881	Algeria	Minimum Estimate	3221 - Nursing associate professionals	High Income and Upper Middle Income	West	Nursing Personnel
3	Audiologist	104	113	122	0.03	363,730	Algeria	Minimum Estimate	2266 - Audiologists and speech therapists	High Income and Upper Middle Income	West	Other Health Workers
4	Cardiologist	283	328	377	0.09	117,317	Algeria	Minimum Estimate	2212 - Specialist medical practitioners	High Income and Upper Middle Income	West	Medical Doctors
5	Cardiothoracic Surgeon	45	48	51	0.01	871,184	Algeria	Minimum Estimate	2212 - Specialist medical practitioners	High Income and Upper Middle Income	West	Medical Doctors
6	Clinical Officer/Physician Assistant	3,149	3,565	3,948	0.90	11,078	Algeria	Minimum Estimate	3256 - Medical assistants	High Income and Upper Middle Income	West	Other Health Workers
7	Clinical Pharmacist	1,240	1,385	1,513	0.35	28,981	Algeria	Minimum Estimate	2262 - Pharmacists	High Income and Upper Middle Income	West	Pharmacist
8	Clinical Psychologist	2,397	2,523	2,607	0.59	16,890	Algeria	Minimum Estimate	2634 - Psychologists	High Income and Upper Middle Income	West	Other Health Workers
9	Community health worker/Village health worker	15,897	16,554	16,755	3.80	2,629	Algeria	Minimum Estimate	3253 - Community health workers	High Income and Upper Middle Income	West	Other Health Workers
10	Dental Surgery Assistant	3,173	3,447	3,642	0.83	12,104	Algeria	Minimum Estimate	3251 - Dental assistants and therapists	High Income and Upper Middle Income	West	Other Health Workers
11	Dental Therapist	1,960	2,134	2,262	0.51	19,500	Algeria	Minimum Estimate	3251 - Dental assistants and therapists	High Income and Upper Middle Income	West	Other Health Workers
12	Dentist	2,831	2,994	3,051	0.69	14,395	Algeria	Minimum Estimate	2261 - Dentists	High Income and Upper Middle Income	West	Dentist
13	Dermatologist	131	139	147	0.03	301,631	Algeria	Minimum Estimate	2212 - Specialist medical practitioners	High Income and Upper Middle Income	West	Medical Doctors
14	Endocrinologist	574	712	848	0.19	51,307	Algeria	Minimum Estimate	2212 - Specialist medical practitioners	High Income and Upper Middle Income	West	Medical Doctors
15	ENT Surgeon	424	471	536	0.12	81,793	Algeria	Minimum Estimate	2212 - Specialist medical practitioners	High Income and Upper Middle Income	West	Medical Doctors
16	Environmental Health Officer	1,089	1,154	1,210	0.27	36,557	Algeria	Minimum Estimate	2263 - Environmental and occupational health and hygiene professionals	High Income and Upper Middle Income	West	Other Health Workers
17	Gastroenterologist	207	214	214	0.05	203,373	Algeria	Minimum Estimate	2212 - Specialist medical practitioners	High Income and Upper Middle Income	West	Medical Doctors
18	General Medical Practitioner (Generalist Doctor)	12,820	13,905	14,803	3.36	2,977	Algeria	Minimum Estimate	2211 - Generalist medical practitioners	High Income and Upper Middle Income	West	Medical Doctors
19	General Surgeon	522	618	715	0.16	61,289	Algeria	Minimum Estimate	2212 - Specialist medical practitioners	High Income and Upper Middle Income	West	Medical Doctors
20	Haematologist	71	77	84	0.02	521,127	Algeria	Minimum Estimate	2212 - Specialist medical practitioners	High Income and Upper Middle Income	West	Medical Doctors
21	Health Promoter/Health Educator	157	170	182	0.04	241,165	Algeria	Minimum Estimate	2269 - Health professionals not elsewhere classified	High Income and Upper Middle Income	West	Other Health Workers
22	Infectious Diseases Specialist	28	30	32	0.01	1,391,208	Algeria	Minimum Estimate	2212 - Specialist medical practitioners	High Income and Upper Middle Income	West	Medical Doctors
23	Intensive Care Nurse	1,170	1,252	1,331	0.30	33,177	Algeria	Minimum Estimate	2221 - Nursing professionals	High Income and Upper Middle Income	West	Nursing Personnel
24	Medical Laboratory Scientist	4,983	5,717	6,394	1.46	6,848	Algeria	Minimum Estimate	3212 - Medical and pathology laboratory technicians	High Income and Upper Middle Income	West	Other Health Workers
25	Medical Laboratory Technician	3,811	4,240	4,627	1.05	9,490	Algeria	Minimum Estimate	3212 - Medical and pathology laboratory technicians	High Income and Upper Middle Income	West	Other Health Workers
26	Medical Social Worker	980	1,023	1,028	0.23	42,798	Algeria	Minimum Estimate	1344 - Social welfare managers	High Income and Upper Middle Income	West	Other Health Workers
27	Mental Health Nurse	2,524	2,676	2,746	0.63	15,935	Algeria	Minimum Estimate	2221 - Nursing professionals	High Income and Upper Middle Income	West	Nursing Personnel
28	Midwife	23,022	24,277	24,766	5.62	1,778	Algeria	Minimum Estimate	2222 - Midwifery professionals	High Income and Upper Middle Income	West	Midwifery Personnel
29	Nephrologist	1,013	1,179	1,319	0.30	33,191	Algeria	Minimum Estimate	2212 - Specialist medical practitioners	High Income and Upper Middle Income	West	Medical Doctors
30	Neuro-Surgeon	248	277	334	0.08	129,042	Algeria	Minimum Estimate	2212 - Specialist medical practitioners	High Income and Upper Middle Income	West	Medical Doctors
31	Nurse Anaesthetist	1,412	1,686	1,989	0.45	22,136	Algeria	Minimum Estimate	2221 - Nursing professionals	High Income and Upper Middle Income	West	Nursing Personnel
32	Nutritionist	3,828	4,005	4,121	0.93	10,717	Algeria	Minimum Estimate	2265 - Dieticians and nutritionists	High Income and Upper Middle Income	West	Other Health Workers
33	Obstetrician & Gynaecologist	2,265	2,420	2,551	0.58	17,304	Algeria	Minimum Estimate	2212 - Specialist medical practitioners	High Income and Upper Middle Income	West	Medical Doctors
34	Occupational Therapist	1,055	1,144	1,223	0.28	35,940	Algeria	Minimum Estimate	2269 - Health professionals not elsewhere classified	High Income and Upper Middle Income	West	Other Health Workers
35	Oncology Nurse	768	904	1,031	0.24	42,533	Algeria	Minimum Estimate	2221 - Nursing professionals	High Income and Upper Middle Income	West	Nursing Personnel
36	Operating Theatre Nurse	7,408	8,176	8,892	2.04	4,910	Algeria	Minimum Estimate	2221 - Nursing professionals	High Income and Upper Middle Income	West	Nursing Personnel
37	Ophthalmic Nurse	1,197	1,371	1,541	0.35	28,472	Algeria	Minimum Estimate	2221 - Nursing professionals	High Income and Upper Middle Income	West	Nursing Personnel
38	Ophthalmologist	317	382	454	0.10	95,730	Algeria	Minimum Estimate	2212 - Specialist medical practitioners	High Income and Upper Middle Income	West	Medical Doctors
39	Optometrist	621	746	872	0.20	50,202	Algeria	Minimum Estimate	2267 - Optometrists and ophthalmic opticians	High Income and Upper Middle Income	West	Other Health Workers
40	Orthopaedic Nurse	527	566	606	0.14	72,761	Algeria	Minimum Estimate	2221 - Nursing professionals	High Income and Upper Middle Income	West	Nursing Personnel
41	Orthopaedic Surgeon	2,598	2,945	3,293	0.75	13,399	Algeria	Minimum Estimate	2212 - Specialist medical practitioners	High Income and Upper Middle Income	West	Medical Doctors
42	Orthopaedic Technologist	1,239	1,319	1,393	0.32	31,721	Algeria	Minimum Estimate	3214 - Medical and dental prosthetic technicians	High Income and Upper Middle Income	West	Other Health Workers
43	Paediatric Nurse	2,767	2,909	2,913	0.66	15,129	Algeria	Minimum Estimate	2221 - Nursing professionals	High Income and Upper Middle Income	West	Nursing Personnel
44	Paediatric Surgeon	123	131	133	0.03	332,045	Algeria	Minimum Estimate	2212 - Specialist medical practitioners	High Income and Upper Middle Income	West	Medical Doctors
45	Paediatrician	1,857	2,098	2,305	0.52	19,085	Algeria	Minimum Estimate	2212 - Specialist medical practitioners	High Income and Upper Middle Income	West	Medical Doctors
46	Pathologist	239	259	271	0.06	161,582	Algeria	Minimum Estimate	2212 - Specialist medical practitioners	High Income and Upper Middle Income	West	Medical Doctors
47	Pharmacist	2,309	2,560	2,798	0.64	15,639	Algeria	Minimum Estimate	2262 - Pharmacists	High Income and Upper Middle Income	West	Pharmacist
48	Pharmacy Technician	3,823	4,133	4,406	1.00	9,999	Algeria	Minimum Estimate	3213 - Pharmaceutical technicians and assistants	High Income and Upper Middle Income	West	Other Health Workers
49	Physician	5,092	5,728	6,305	1.44	6,962	Algeria	Minimum Estimate	2212 - Specialist medical practitioners	High Income and Upper Middle Income	West	Medical Doctors
50	Physiotherapist	1,129	1,223	1,342	0.31	32,653	Algeria	Minimum Estimate	2264 - Physiotherapists	High Income and Upper Middle Income	West	Other Health Workers
51	Plastic Surgeon	194	207	218	0.05	202,764	Algeria	Minimum Estimate	2212 - Specialist medical practitioners	High Income and Upper Middle Income	West	Medical Doctors
52	Psychiatrist	2,404	2,541	2,616	0.60	16,753	Algeria	Minimum Estimate	2212 - Specialist medical practitioners	High Income and Upper Middle Income	West	Medical Doctors
53	Radiation Oncologist	201	246	296	0.07	147,837	Algeria	Minimum Estimate	2212 - Specialist medical practitioners	High Income and Upper Middle Income	West	Medical Doctors
54	Radiographer (Diagnostics and Therapy)	4,704	5,313	5,836	1.33	7,513	Algeria	Minimum Estimate	3211 - Medical imaging and therapeutic equipment technicians	High Income and Upper Middle Income	West	Other Health Workers
55	Radiologist	1,185	1,345	1,502	0.34	29,234	Algeria	Minimum Estimate	2212 - Specialist medical practitioners	High Income and Upper Middle Income	West	Medical Doctors
56	Registered General Nurse / State Certified Nurse	125,797	142,153	155,755	35.46	282	Algeria	Minimum Estimate	2221 - Nursing professionals	High Income and Upper Middle Income	West	Nursing Personnel
57	Renal Nurse	12,726	14,818	16,575	3.79	2,640	Algeria	Minimum Estimate	2221 - Nursing professionals	High Income and Upper Middle Income	West	Nursing Personnel
58	Respiratory Physician	293	330	367	0.08	120,290	Algeria	Minimum Estimate	2212 - Specialist medical practitioners	High Income and Upper Middle Income	West	Medical Doctors
59	Rheumatologist	141	156	171	0.04	257,576	Algeria	Minimum Estimate	2212 - Specialist medical practitioners	High Income and Upper Middle Income	West	Medical Doctors
60	Speech Therapist	552	601	686	0.16	63,531	Algeria	Minimum Estimate	2266 - Audiologists and speech therapists	High Income and Upper Middle Income	West	Other Health Workers
61	Urologist	89	105	121	0.03	362,304	Algeria	Minimum Estimate	2212 - Specialist medical practitioners	High Income and Upper Middle Income	West	Medical Doctors
1	Anaesthesiologist	193	220	249	0.08	133,093	Angola	Minimum Estimate	2212 - Specialist medical practitioners	Lower-middle Income	Southern	Medical Doctors
2	Associate Nurse/Enrolled Nurse/Nursing Assistant	31,805	35,833	40,296	12.17	822	Angola	Minimum Estimate	3221 - Nursing associate professionals	Lower-middle Income	Southern	Nursing Personnel
3	Audiologist	71	80	89	0.03	370,705	Angola	Minimum Estimate	2266 - Audiologists and speech therapists	Lower-middle Income	Southern	Other Health Workers
4	Cardiologist	97	114	133	0.04	249,567	Angola	Minimum Estimate	2212 - Specialist medical practitioners	Lower-middle Income	Southern	Medical Doctors
5	Cardiothoracic Surgeon	30	33	36	0.01	921,855	Angola	Minimum Estimate	2212 - Specialist medical practitioners	Lower-middle Income	Southern	Medical Doctors
6	Clinical Officer/Physician Assistant	3,620	4,011	4,480	1.36	7,354	Angola	Minimum Estimate	3256 - Medical assistants	Lower-middle Income	Southern	Other Health Workers
7	Clinical Pharmacist	649	722	809	0.24	40,851	Angola	Minimum Estimate	2262 - Pharmacists	Lower-middle Income	Southern	Pharmacist
8	Clinical Psychologist	1,877	2,094	2,342	0.71	14,127	Angola	Minimum Estimate	2634 - Psychologists	Lower-middle Income	Southern	Other Health Workers
9	Community health worker/Village health worker	20,507	23,003	25,740	7.78	1,285	Angola	Minimum Estimate	3253 - Community health workers	Lower-middle Income	Southern	Other Health Workers
10	Dental Surgery Assistant	1,997	2,273	2,591	0.78	12,780	Angola	Minimum Estimate	3251 - Dental assistants and therapists	Lower-middle Income	Southern	Other Health Workers
11	Dental Therapist	1,252	1,425	1,624	0.49	20,387	Angola	Minimum Estimate	3251 - Dental assistants and therapists	Lower-middle Income	Southern	Other Health Workers
12	Dentist	1,404	1,593	1,831	0.55	18,050	Angola	Minimum Estimate	2261 - Dentists	Lower-middle Income	Southern	Dentist

S/N	Health Professionals	2022	2026	2030	Density per 10,000 population	Required Population ratio (1 professional is to xxx population)	Name of Country	Modelling Scenario	ISCO-08 Match	Income Group Classification	Sub-Regional Grouping	SDG 3c Occupation
13	Dermatologist	89	100	111	0.03	298,026	Angola	Minimum Estimate	2212 - Specialist medical practitioners	Lower-middle Income	Southern	Medical Doctors
14	Endocrinologist	90	107	130	0.04	251,446	Angola	Minimum Estimate	2212 - Specialist medical practitioners	Lower-middle Income	Southern	Medical Doctors
15	ENT Surgeon	206	235	268	0.08	123,277	Angola	Minimum Estimate	2212 - Specialist medical practitioners	Lower-middle Income	Southern	Medical Doctors
16	Environmental Health Officer	816	919	1,028	0.31	32,275	Angola	Minimum Estimate	2263 - Environmental and occupational health and hygiene professionals	Lower-middle Income	Southern	Other Health Workers
17	Gastroenterologist	357	371	392	0.12	83,835	Angola	Minimum Estimate	2212 - Specialist medical practitioners	Lower-middle Income	Southern	Medical Doctors
18	General Medical Practitioner (Generalist Doctor)	9,201	10,334	11,608	3.51	2,848	Angola	Minimum Estimate	2211 - Generalist medical practitioners	Lower-middle Income	Southern	Medical Doctors
19	General Surgeon	188	220	261	0.08	126,148	Angola	Minimum Estimate	2212 - Specialist medical practitioners	Lower-middle Income	Southern	Medical Doctors
20	Haematologist	73	81	89	0.03	371,559	Angola	Minimum Estimate	2212 - Specialist medical practitioners	Lower-middle Income	Southern	Medical Doctors
21	Health Promoter/Health Educator	187	204	223	0.07	148,042	Angola	Minimum Estimate	2269 - Health professionals not elsewhere classified	Lower-middle Income	Southern	Other Health Workers
22	Infectious Diseases Specialist	34	37	40	0.01	825,229	Angola	Minimum Estimate	2212 - Specialist medical practitioners	Lower-middle Income	Southern	Medical Doctors
23	Intensive Care Nurse	804	888	978	0.29	33,901	Angola	Minimum Estimate	2221 - Nursing professionals	Lower-middle Income	Southern	Nursing Personnel
24	Medical Laboratory Scientist	2,972	3,364	3,829	1.16	8,616	Angola	Minimum Estimate	3212 - Medical and pathology laboratory technicians	Lower-middle Income	Southern	Other Health Workers
25	Medical Laboratory Technician	3,574	4,110	4,747	1.44	6,943	Angola	Minimum Estimate	3212 - Medical and pathology laboratory technicians	Lower-middle Income	Southern	Other Health Workers
26	Medical Social Worker	1,040	1,091	1,146	0.35	28,807	Angola	Minimum Estimate	1344 - Social welfare managers	Lower-middle Income	Southern	Other Health Workers
27	Mental Health Nurse	1,124	1,265	1,446	0.44	22,788	Angola	Minimum Estimate	2221 - Nursing professionals	Lower-middle Income	Southern	Nursing Personnel
28	Midwife	17,529	19,503	21,787	6.59	1,518	Angola	Minimum Estimate	2222 - Midwifery professionals	Lower-middle Income	Southern	Midwifery Personnel
29	Nephrologist	171	200	237	0.07	138,272	Angola	Minimum Estimate	2212 - Specialist medical practitioners	Lower-middle Income	Southern	Medical Doctors
30	Neuro-Surgeon	86	97	109	0.03	304,283	Angola	Minimum Estimate	2212 - Specialist medical practitioners	Lower-middle Income	Southern	Medical Doctors
31	Nurse Anaesthetist	377	434	498	0.15	66,532	Angola	Minimum Estimate	2221 - Nursing professionals	Lower-middle Income	Southern	Nursing Personnel
32	Nutritionist	2,820	2,951	3,072	0.93	10,773	Angola	Minimum Estimate	2265 - Dieticians and nutritionists	Lower-middle Income	Southern	Other Health Workers
33	Obstetrician & Gynaecologist	1,787	2,021	2,282	0.69	14,514	Angola	Minimum Estimate	2212 - Specialist medical practitioners	Lower-middle Income	Southern	Medical Doctors
34	Occupational Therapist	432	495	567	0.17	58,295	Angola	Minimum Estimate	2269 - Health professionals not elsewhere classified	Lower-middle Income	Southern	Other Health Workers
35	Oncology Nurse	135	157	185	0.06	177,967	Angola	Minimum Estimate	2221 - Nursing professionals	Lower-middle Income	Southern	Nursing Personnel
36	Operating Theatre Nurse	2,356	2,698	3,124	0.95	10,540	Angola	Minimum Estimate	2221 - Nursing professionals	Lower-middle Income	Southern	Nursing Personnel
37	Ophthalmic Nurse	338	389	448	0.14	73,844	Angola	Minimum Estimate	2221 - Nursing professionals	Lower-middle Income	Southern	Nursing Personnel
38	Ophthalmologist	66	77	91	0.03	362,680	Angola	Minimum Estimate	2212 - Specialist medical practitioners	Lower-middle Income	Southern	Medical Doctors
39	Optometrist	237	274	316	0.10	104,650	Angola	Minimum Estimate	2267 - Optometrists and ophthalmic opticians	Lower-middle Income	Southern	Other Health Workers
40	Orthopaedic Nurse	234	268	304	0.09	108,883	Angola	Minimum Estimate	2221 - Nursing professionals	Lower-middle Income	Southern	Nursing Personnel
41	Orthopaedic Surgeon	583	732	925	0.28	35,210	Angola	Minimum Estimate	2212 - Specialist medical practitioners	Lower-middle Income	Southern	Medical Doctors
42	Orthopaedic Technologist	801	902	1,010	0.30	32,833	Angola	Minimum Estimate	3214 - Medical and dental prosthetic technicians	Lower-middle Income	Southern	Other Health Workers
43	Paediatric Nurse	2,544	2,802	3,054	0.92	10,858	Angola	Minimum Estimate	2221 - Nursing professionals	Lower-middle Income	Southern	Nursing Personnel
44	Paediatric Surgeon	93	101	110	0.03	302,455	Angola	Minimum Estimate	2212 - Specialist medical practitioners	Lower-middle Income	Southern	Medical Doctors
45	Paediatrician	909	1,006	1,113	0.34	29,717	Angola	Minimum Estimate	2212 - Specialist medical practitioners	Lower-middle Income	Southern	Medical Doctors
46	Pathologist	190	216	250	0.08	131,850	Angola	Minimum Estimate	2212 - Specialist medical practitioners	Lower-middle Income	Southern	Medical Doctors
47	Pharmacist	2,310	2,444	2,585	0.78	12,784	Angola	Minimum Estimate	2262 - Pharmacists	Lower-middle Income	Southern	Pharmacist
48	Pharmacy Technician	2,507	2,782	3,087	0.93	10,722	Angola	Minimum Estimate	3213 - Pharmaceutical technicians and assistants	Lower-middle Income	Southern	Other Health Workers
49	Physician	2,209	2,488	2,810	0.85	11,758	Angola	Minimum Estimate	2212 - Specialist medical practitioners	Lower-middle Income	Southern	Medical Doctors
50	Physiotherapist	576	651	734	0.22	45,099	Angola	Minimum Estimate	2264 - Physiotherapists	Lower-middle Income	Southern	Other Health Workers
51	Plastic Surgeon	112	126	141	0.04	235,628	Angola	Minimum Estimate	2212 - Specialist medical practitioners	Lower-middle Income	Southern	Medical Doctors
52	Psychiatrist	1,135	1,267	1,434	0.43	23,003	Angola	Minimum Estimate	2212 - Specialist medical practitioners	Lower-middle Income	Southern	Medical Doctors
53	Radiation Oncologist	39	46	55	0.02	602,834	Angola	Minimum Estimate	2212 - Specialist medical practitioners	Lower-middle Income	Southern	Medical Doctors
54	Radiographer (Diagnostics and Therapy)	1,582	1,807	2,084	0.63	15,807	Angola	Minimum Estimate	3211 - Medical imaging and therapeutic equipment technicians	Lower-middle Income	Southern	Other Health Workers
55	Radiologist	517	588	675	0.20	48,841	Angola	Minimum Estimate	2212 - Specialist medical practitioners	Lower-middle Income	Southern	Medical Doctors
56	Registered General Nurse / State Certified Nurse	55,891	61,622	68,131	20.64	484	Angola	Minimum Estimate	2221 - Nursing professionals	Lower-middle Income	Southern	Nursing Personnel
57	Renal Nurse	2,133	2,490	2,952	0.90	11,122	Angola	Minimum Estimate	2221 - Nursing professionals	Lower-middle Income	Southern	Nursing Personnel
58	Respiratory Physician	154	168	183	0.06	181,169	Angola	Minimum Estimate	2212 - Specialist medical practitioners	Lower-middle Income	Southern	Medical Doctors
59	Rheumatologist	70	80	92	0.03	361,839	Angola	Minimum Estimate	2212 - Specialist medical practitioners	Lower-middle Income	Southern	Medical Doctors
60	Speech Therapist	228	256	288	0.09	114,891	Angola	Minimum Estimate	2266 - Audiologists and speech therapists	Lower-middle Income	Southern	Other Health Workers
61	Urologist	17	20	23	0.01	1,404,036	Angola	Minimum Estimate	2212 - Specialist medical practitioners	Lower-middle Income	Southern	Medical Doctors
1	Anaesthesiologist	91	103	116	0.10	105,057	Benin	Minimum Estimate	2212 - Specialist medical practitioners	Low Income	West	Medical Doctors
2	Associate Nurse/Enrolled Nurse/Nursing Assistant	11,180	12,461	13,876	11.36	881	Benin	Minimum Estimate	3221 - Nursing associate professionals	Low Income	West	Nursing Personnel
3	Audiologist	29	33	36	0.03	335,935	Benin	Minimum Estimate	2266 - Audiologists and speech therapists	Low Income	West	Other Health Workers
4	Cardiologist	35	40	45	0.04	270,970	Benin	Minimum Estimate	2212 - Specialist medical practitioners	Low Income	West	Medical Doctors
5	Cardiothoracic Surgeon	14	16	18	0.01	698,052	Benin	Minimum Estimate	2212 - Specialist medical practitioners	Low Income	West	Medical Doctors
6	Clinical Officer/Physician Assistant	1,483	1,638	1,818	1.49	6,708	Benin	Minimum Estimate	3256 - Medical assistants	Low Income	West	Other Health Workers
7	Clinical Pharmacist	239	265	295	0.24	41,382	Benin	Minimum Estimate	2262 - Pharmacists	Low Income	West	Pharmacist
8	Clinical Psychologist	519	573	635	0.52	19,215	Benin	Minimum Estimate	2634 - Psychologists	Low Income	West	Other Health Workers
9	Community health worker/Village health worker	10,755	11,937	13,206	10.81	925	Benin	Minimum Estimate	3253 - Community health workers	Low Income	West	Other Health Workers
10	Dental Surgery Assistant	836	938	1,053	0.86	11,605	Benin	Minimum Estimate	3251 - Dental assistants and therapists	Low Income	West	Other Health Workers
11	Dental Therapist	525	589	662	0.54	18,472	Benin	Minimum Estimate	3251 - Dental assistants and therapists	Low Income	West	Other Health Workers
12	Dentist	575	640	720	0.59	16,960	Benin	Minimum Estimate	2261 - Dentists	Low Income	West	Dentist
13	Dermatologist	32	35	39	0.03	309,739	Benin	Minimum Estimate	2212 - Specialist medical practitioners	Low Income	West	Medical Doctors
14	Endocrinologist	29	34	40	0.03	302,667	Benin	Minimum Estimate	2212 - Specialist medical practitioners	Low Income	West	Medical Doctors
15	ENT Surgeon	85	96	109	0.09	112,186	Benin	Minimum Estimate	2212 - Specialist medical practitioners	Low Income	West	Medical Doctors
16	Environmental Health Officer	301	334	369	0.30	33,143	Benin	Minimum Estimate	2263 - Environmental and occupational health and hygiene professionals	Low Income	West	Other Health Workers
17	Gastroenterologist	90	91	94	0.08	129,491	Benin	Minimum Estimate	2212 - Specialist medical practitioners	Low Income	West	Medical Doctors
18	General Medical Practitioner (Generalist Doctor)	3,503	3,879	4,291	3.51	2,847	Benin	Minimum Estimate	2211 - Generalist medical practitioners	Low Income	West	Medical Doctors
19	General Surgeon	75	86	99	0.08	123,167	Benin	Minimum Estimate	2212 - Specialist medical practitioners	Low Income	West	Medical Doctors
20	Haematologist	31	34	37	0.03	332,325	Benin	Minimum Estimate	2212 - Specialist medical practitioners	Low Income	West	Medical Doctors
21	Health Promoter/Health Educator	74	81	90	0.07	135,895	Benin	Minimum Estimate	2269 - Health professionals not elsewhere classified	Low Income	West	Other Health Workers
22	Infectious Diseases Specialist	10	11	13	0.01	959,657	Benin	Minimum Estimate	2212 - Specialist medical practitioners	Low Income	West	Medical Doctors
23	Intensive Care Nurse	281	313	347	0.28	35,216	Benin	Minimum Estimate	2221 - Nursing professionals	Low Income	West	Nursing Personnel
24	Medical Laboratory Scientist	1,157	1,288	1,443	1.18	8,450	Benin	Minimum Estimate	3212 - Medical and pathology laboratory technicians	Low Income	West	Other Health Workers
25	Medical Laboratory Technician	2,329	2,643	2,995	2.45	4,078	Benin	Minimum Estimate	3212 - Medical and pathology laboratory technicians	Low Income	West	Other Health Workers
26	Medical Social Worker	393	413	436	0.36	27,965	Benin	Minimum Estimate	1344 - Social welfare managers	Low Income	West	Other Health Workers
27	Mental Health Nurse	401	449	511	0.42	23,864	Benin	Minimum Estimate	2221 - Nursing professionals	Low Income	West	Nursing Personnel
28	Midwife	6,684	7,380	8,151	6.67	1,500	Benin	Minimum Estimate	2222 - Midwifery professionals	Low Income	West	Midwifery Personnel
29	Nephrologist	77	88	103	0.08	117,689	Benin	Minimum Estimate	2212 - Specialist medical practitioners	Low Income	West	Medical Doctors
30	Neuro-Surgeon	39	44	50	0.04	245,024	Benin	Minimum Estimate	2212 - Specialist medical practitioners	Low Income	West	Medical Doctors
31	Nurse Anaesthetist	143	163	186	0.15	65,851	Benin	Minimum Estimate	2221 - Nursing professionals	Low Income	West	Nursing Personnel
32	Nutritionist	1,526	1,628	1,726	1.41	7,082	Benin	Minimum Estimate	2265 - Dieticians and nutritionists	Low Income	West	Other Health Workers
33	Obstetrician & Gynaecologist	600	668	742	0.61	16,458	Benin	Minimum Estimate	2212 - Specialist medical practitioners	Low Income	West	Medical Doctors
34	Occupational Therapist	197	226	258	0.21	47,259	Benin	Minimum Estimate	2269 - Health professionals not elsewhere classified	Low Income	West	Other Health Workers
35	Oncology Nurse	56	63	74	0.06	165,377	Benin	Minimum Estimate	2221 - Nursing professionals	Low Income	West	Nursing Personnel

S/N	Health Professionals	2022	2026	2030	Density per 10,000 population	Required Population ratio (1 professional is to xxx population)	Name of Country	Modelling Scenario	ISCO-08 Match	Income Group Classification	Sub-Regional Grouping	SDG 3c Occupation
36	Operating Theatre Nurse	946	1,065	1,217	1.00	10,005	Benin	Minimum Estimate	2221 - Nursing professionals	Low Income	West	Nursing Personnel
37	Ophthalmic Nurse	100	114	130	0.11	93,936	Benin	Minimum Estimate	2221 - Nursing professionals	Low Income	West	Nursing Personnel
38	Ophthalmologist	26	30	35	0.03	349,697	Benin	Minimum Estimate	2212 - Specialist medical practitioners	Low Income	West	Medical Doctors
39	Optometrist	95	107	121	0.10	100,553	Benin	Minimum Estimate	2267 - Optometrists and ophthalmic opticians	Low Income	West	Other Health Workers
40	Orthopaedic Nurse	100	116	135	0.11	90,685	Benin	Minimum Estimate	2221 - Nursing professionals	Low Income	West	Nursing Personnel
41	Orthopaedic Surgeon	273	313	361	0.30	33,850	Benin	Minimum Estimate	2212 - Specialist medical practitioners	Low Income	West	Medical Doctors
42	Orthopaedic Technologist	309	348	389	0.32	31,406	Benin	Minimum Estimate	3214 - Medical and dental prosthetic technicians	Low Income	West	Other Health Workers
43	Paediatric Nurse	1,059	1,152	1,242	1.02	9,849	Benin	Minimum Estimate	2221 - Nursing professionals	Low Income	West	Nursing Personnel
44	Paediatric Surgeon	34	38	41	0.03	298,914	Benin	Minimum Estimate	2212 - Specialist medical practitioners	Low Income	West	Medical Doctors
45	Paediatrician	388	433	484	0.40	25,225	Benin	Minimum Estimate	2212 - Specialist medical practitioners	Low Income	West	Medical Doctors
46	Pathologist	46	50	56	0.05	217,672	Benin	Minimum Estimate	2212 - Specialist medical practitioners	Low Income	West	Medical Doctors
47	Pharmacist	542	565	591	0.49	20,576	Benin	Minimum Estimate	2262 - Pharmacists	Low Income	West	Pharmacist
48	Pharmacy Technician	892	987	1,093	0.90	11,170	Benin	Minimum Estimate	3213 - Pharmaceutical technicians and assistants	Low Income	West	Other Health Workers
49	Physician	949	1,060	1,187	0.97	10,291	Benin	Minimum Estimate	2212 - Specialist medical practitioners	Low Income	West	Medical Doctors
50	Physiotherapist	224	251	282	0.23	43,361	Benin	Minimum Estimate	2264 - Physiotherapists	Low Income	West	Other Health Workers
51	Plastic Surgeon	55	62	71	0.06	171,939	Benin	Minimum Estimate	2212 - Specialist medical practitioners	Low Income	West	Medical Doctors
52	Psychiatrist	447	495	555	0.45	21,978	Benin	Minimum Estimate	2212 - Specialist medical practitioners	Low Income	West	Medical Doctors
53	Radiation Oncologist	12	14	16	0.01	761,481	Benin	Minimum Estimate	2212 - Specialist medical practitioners	Low Income	West	Medical Doctors
54	Radiographer (Diagnostics and Therapy)	623	700	795	0.65	15,323	Benin	Minimum Estimate	3211 - Medical imaging and therapeutic equipment technicians	Low Income	West	Other Health Workers
55	Radiologist	191	214	243	0.20	50,165	Benin	Minimum Estimate	2212 - Specialist medical practitioners	Low Income	West	Medical Doctors
56	Registered General Nurse / State Certified Nurse	22,373	24,350	26,625	21.85	458	Benin	Minimum Estimate	2221 - Nursing professionals	Low Income	West	Nursing Personnel
57	Renal Nurse	956	1,094	1,279	1.05	9,508	Benin	Minimum Estimate	2221 - Nursing professionals	Low Income	West	Nursing Personnel
58	Respiratory Physician	56	63	71	0.06	171,042	Benin	Minimum Estimate	2212 - Specialist medical practitioners	Low Income	West	Medical Doctors
59	Rheumatologist	25	28	31	0.03	393,216	Benin	Minimum Estimate	2212 - Specialist medical practitioners	Low Income	West	Medical Doctors
60	Speech Therapist	90	100	112	0.09	109,434	Benin	Minimum Estimate	2266 - Audiologists and speech therapists	Low Income	West	Other Health Workers
61	Urologist	8	10	12	0.01	986,609	Benin	Minimum Estimate	2212 - Specialist medical practitioners	Low Income	West	Medical Doctors
1	Anaesthesiologist	22	24	27	0.12	86,772	Botswana	Minimum Estimate	2212 - Specialist medical practitioners	High Income and Upper Middle Income	Southern	Medical Doctors
2	Associate Nurse/Enrolled Nurse/Nursing Assistant	2,612	2,820	3,042	12.86	778	Botswana	Minimum Estimate	3221 - Nursing associate professionals	High Income and Upper Middle Income	Southern	Nursing Personnel
3	Audiologist	6	7	7	0.03	329,917	Botswana	Minimum Estimate	2266 - Audiologists and speech therapists	High Income and Upper Middle Income	Southern	Other Health Workers
4	Cardiologist	11	13	15	0.06	162,243	Botswana	Minimum Estimate	2212 - Specialist medical practitioners	High Income and Upper Middle Income	Southern	Medical Doctors
5	Cardiothoracic Surgeon	3	4	4	0.02	579,114	Botswana	Minimum Estimate	2212 - Specialist medical practitioners	High Income and Upper Middle Income	Southern	Medical Doctors
6	Clinical Officer/Physician Assistant	322	344	370	1.57	6,373	Botswana	Minimum Estimate	3256 - Medical assistants	High Income and Upper Middle Income	Southern	Other Health Workers
7	Clinical Pharmacist	53	59	66	0.28	35,862	Botswana	Minimum Estimate	2262 - Pharmacists	High Income and Upper Middle Income	Southern	Pharmacist
8	Clinical Psychologist	113	122	132	0.56	17,895	Botswana	Minimum Estimate	2634 - Psychologists	High Income and Upper Middle Income	Southern	Other Health Workers
9	Community health worker/Village health worker	1,089	1,134	1,170	4.95	2,020	Botswana	Minimum Estimate	3253 - Community health workers	High Income and Upper Middle Income	Southern	Other Health Workers
10	Dental Surgery Assistant	216	239	264	1.11	8,993	Botswana	Minimum Estimate	3251 - Dental assistants and therapists	High Income and Upper Middle Income	Southern	Other Health Workers
11	Dental Therapist	136	151	166	0.70	14,287	Botswana	Minimum Estimate	3251 - Dental assistants and therapists	High Income and Upper Middle Income	Southern	Other Health Workers
12	Dentist	152	165	178	0.75	13,320	Botswana	Minimum Estimate	2261 - Dentists	High Income and Upper Middle Income	Southern	Dentist
13	Dermatologist	7	8	8	0.03	290,476	Botswana	Minimum Estimate	2212 - Specialist medical practitioners	High Income and Upper Middle Income	Southern	Medical Doctors
14	Endocrinologist	15	18	22	0.09	106,079	Botswana	Minimum Estimate	2212 - Specialist medical practitioners	High Income and Upper Middle Income	Southern	Medical Doctors
15	ENT Surgeon	24	27	31	0.13	77,379	Botswana	Minimum Estimate	2212 - Specialist medical practitioners	High Income and Upper Middle Income	Southern	Medical Doctors
16	Environmental Health Officer	58	62	66	0.28	35,914	Botswana	Minimum Estimate	2263 - Environmental and occupational health and hygiene professionals	High Income and Upper Middle Income	Southern	Other Health Workers
17	Gastroenterologist	13	13	13	0.06	178,361	Botswana	Minimum Estimate	2212 - Specialist medical practitioners	High Income and Upper Middle Income	Southern	Medical Doctors
18	General Medical Practitioner (Generalist Doctor)	787	847	910	3.84	2,602	Botswana	Minimum Estimate	2211 - Generalist medical practitioners	High Income and Upper Middle Income	Southern	Medical Doctors
19	General Surgeon	29	32	36	0.15	64,623	Botswana	Minimum Estimate	2212 - Specialist medical practitioners	High Income and Upper Middle Income	Southern	Medical Doctors
20	Haematologist	4	5	5	0.02	448,607	Botswana	Minimum Estimate	2212 - Specialist medical practitioners	High Income and Upper Middle Income	Southern	Medical Doctors
21	Health Promoter/Health Educator	14	15	16	0.07	145,208	Botswana	Minimum Estimate	2269 - Health professionals not elsewhere classified	High Income and Upper Middle Income	Southern	Other Health Workers
22	Infectious Diseases Specialist	2	3	3	0.01	870,622	Botswana	Minimum Estimate	2212 - Specialist medical practitioners	High Income and Upper Middle Income	Southern	Medical Doctors
23	Intensive Care Nurse	50	54	58	0.24	41,116	Botswana	Minimum Estimate	2221 - Nursing professionals	High Income and Upper Middle Income	Southern	Nursing Personnel
24	Medical Laboratory Scientist	320	348	381	1.62	6,192	Botswana	Minimum Estimate	3212 - Medical and pathology laboratory technicians	High Income and Upper Middle Income	Southern	Other Health Workers
25	Medical Laboratory Technician	261	284	311	1.32	7,604	Botswana	Minimum Estimate	3212 - Medical and pathology laboratory technicians	High Income and Upper Middle Income	Southern	Other Health Workers
26	Medical Social Worker	46	47	48	0.20	49,401	Botswana	Minimum Estimate	1344 - Social welfare managers	High Income and Upper Middle Income	Southern	Other Health Workers
27	Mental Health Nurse	102	110	121	0.51	19,477	Botswana	Minimum Estimate	2221 - Nursing professionals	High Income and Upper Middle Income	Southern	Nursing Personnel
28	Midwife	1,256	1,344	1,425	6.00	1,666	Botswana	Minimum Estimate	2222 - Midwifery professionals	High Income and Upper Middle Income	Southern	Midwifery Personnel
29	Nephrologist	32	37	44	0.19	53,729	Botswana	Minimum Estimate	2212 - Specialist medical practitioners	High Income and Upper Middle Income	Southern	Medical Doctors
30	Neuro-Surgeon	10	12	14	0.06	169,054	Botswana	Minimum Estimate	2212 - Specialist medical practitioners	High Income and Upper Middle Income	Southern	Medical Doctors
31	Nurse Anaesthetist	78	93	111	0.47	21,210	Botswana	Minimum Estimate	2221 - Nursing professionals	High Income and Upper Middle Income	Southern	Nursing Personnel
32	Nutritionist	146	150	152	0.64	15,517	Botswana	Minimum Estimate	2265 - Dieticians and nutritionists	High Income and Upper Middle Income	Southern	Other Health Workers
33	Obstetrician & Gynaecologist	155	166	178	0.75	13,336	Botswana	Minimum Estimate	2212 - Specialist medical practitioners	High Income and Upper Middle Income	Southern	Medical Doctors
34	Occupational Therapist	47	54	63	0.27	37,540	Botswana	Minimum Estimate	2269 - Health professionals not elsewhere classified	High Income and Upper Middle Income	Southern	Other Health Workers
35	Oncology Nurse	27	33	39	0.17	60,273	Botswana	Minimum Estimate	2221 - Nursing professionals	High Income and Upper Middle Income	Southern	Nursing Personnel
36	Operating Theatre Nurse	302	336	383	1.63	6,149	Botswana	Minimum Estimate	2221 - Nursing professionals	High Income and Upper Middle Income	Southern	Nursing Personnel
37	Ophthalmic Nurse	33	37	42	0.18	55,520	Botswana	Minimum Estimate	2221 - Nursing professionals	High Income and Upper Middle Income	Southern	Nursing Personnel
38	Ophthalmologist	12	14	17	0.07	138,766	Botswana	Minimum Estimate	2212 - Specialist medical practitioners	High Income and Upper Middle Income	Southern	Medical Doctors
39	Optometrist	55	64	74	0.31	31,913	Botswana	Minimum Estimate	2267 - Optometrists and ophthalmic opticians	High Income and Upper Middle Income	Southern	Other Health Workers
40	Orthopaedic Nurse	24	29	34	0.14	70,146	Botswana	Minimum Estimate	2221 - Nursing professionals	High Income and Upper Middle Income	Southern	Nursing Personnel
41	Orthopaedic Surgeon	95	108	124	0.52	19,104	Botswana	Minimum Estimate	2212 - Specialist medical practitioners	High Income and Upper Middle Income	Southern	Medical Doctors
42	Orthopaedic Technologist	65	71	78	0.33	30,289	Botswana	Minimum Estimate	3214 - Medical and dental prosthetic technicians	High Income and Upper Middle Income	Southern	Other Health Workers
43	Paediatric Nurse	140	147	151	0.64	15,683	Botswana	Minimum Estimate	2221 - Nursing professionals	High Income and Upper Middle Income	Southern	Nursing Personnel
44	Paediatric Surgeon	4	4	4	0.02	527,956	Botswana	Minimum Estimate	2212 - Specialist medical practitioners	High Income and Upper Middle Income	Southern	Medical Doctors
45	Paediatrician	73	81	91	0.39	25,789	Botswana	Minimum Estimate	2212 - Specialist medical practitioners	High Income and Upper Middle Income	Southern	Medical Doctors
46	Pathologist	24	26	29	0.12	81,742	Botswana	Minimum Estimate	2212 - Specialist medical practitioners	High Income and Upper Middle Income	Southern	Medical Doctors
47	Pharmacist	165	174	185	0.79	12,730	Botswana	Minimum Estimate	2262 - Pharmacists	High Income and Upper Middle Income	Southern	Pharmacist
48	Pharmacy Technician	210	229	249	1.05	9,506	Botswana	Minimum Estimate	3213 - Pharmaceutical technicians and assistants	High Income and Upper Middle Income	Southern	Other Health Workers
49	Physician	203	226	253	1.07	9,343	Botswana	Minimum Estimate	2212 - Specialist medical practitioners	High Income and Upper Middle Income	Southern	Medical Doctors
50	Physiotherapist	51	56	63	0.27	37,548	Botswana	Minimum Estimate	2264 - Physiotherapists	High Income and Upper Middle Income	Southern	Other Health Workers
51	Plastic Surgeon	18	20	22	0.09	106,802	Botswana	Minimum Estimate	2212 - Specialist medical practitioners	High Income and Upper Middle Income	Southern	Medical Doctors
52	Psychiatrist	94	102	111	0.47	21,255	Botswana	Minimum Estimate	2212 - Specialist medical practitioners	High Income and Upper Middle Income	Southern	Medical Doctors
53	Radiation Oncologist	10	13	16	0.07	149,476	Botswana	Minimum Estimate	2212 - Specialist medical practitioners	High Income and Upper Middle Income	Southern	Medical Doctors
54	Radiographer (Diagnostics and Therapy)	189	211	239	1.01	9,876	Botswana	Minimum Estimate	3211 - Medical imaging and therapeutic equipment technicians	High Income and Upper Middle Income	Southern	Other Health Workers
55	Radiologist	57	63	71	0.30	33,465	Botswana	Minimum Estimate	2212 - Specialist medical practitioners	High Income and Upper Middle Income	Southern	Medical Doctors
56	Registered General Nurse / State Certified Nurse	5,105	5,688	6,383	27.09	369	Botswana	Minimum Estimate	2221 - Nursing professionals	High Income and Upper Middle Income	Southern	Nursing Personnel
57	Renal Nurse	397	462	549	2.34	4,279	Botswana	Minimum Estimate	2221 - Nursing professionals	High Income and Upper Middle Income	Southern	Nursing Personnel
58	Respiratory Physician	12	14	15	0.06	155,612	Botswana	Minimum Estimate	2212 - Specialist medical practitioners	High Income and Upper Middle Income	Southern	Medical Doctors

S/N	Health Professionals	2022	2026	2030	Density per 10,000 population	Required Population ratio (1 professional is to xxx population)	Name of Country	Modelling Scenario	ISCO-08 Match	Income Group Classification	Sub-Regional Grouping	SDG 3c Occupation
59	Rheumatologist	7	8	8	0.04	280,450	Botswana	Minimum Estimate	2212 - Specialist medical practitioners	High Income and Upper Middle Income	Southern	Medical Doctors
60	Speech Therapist	20	21	24	0.10	99,193	Botswana	Minimum Estimate	2266 - Audiologists and speech therapists	High Income and Upper Middle Income	Southern	Other Health Workers
61	Urologist	4	5	6	0.03	381,031	Botswana	Minimum Estimate	2212 - Specialist medical practitioners	High Income and Upper Middle Income	Southern	Medical Doctors
1	Anaesthesiologist	172	200	233	0.11	90,315	Burkina Faso	Minimum Estimate	2212 - Specialist medical practitioners	Low Income	West	Medical Doctors
2	Associate Nurse/Enrolled Nurse/Nursing Assistant	21,743	24,075	26,656	12.66	790	Burkina Faso	Minimum Estimate	3221 - Nursing associate professionals	Low Income	West	Nursing Personnel
3	Audiologist	50	55	61	0.03	347,043	Burkina Faso	Minimum Estimate	2266 - Audiologists and speech therapists	Low Income	West	Other Health Workers
4	Cardiologist	52	57	63	0.03	334,586	Burkina Faso	Minimum Estimate	2212 - Specialist medical practitioners	Low Income	West	Medical Doctors
5	Cardiothoracic Surgeon	27	32	37	0.02	566,197	Burkina Faso	Minimum Estimate	2212 - Specialist medical practitioners	Low Income	West	Medical Doctors
6	Clinical Officer/Physician Assistant	1,232	1,287	1,364	0.65	15,324	Burkina Faso	Minimum Estimate	3256 - Medical assistants	Low Income	West	Other Health Workers
7	Clinical Pharmacist	429	472	525	0.25	39,922	Burkina Faso	Minimum Estimate	2262 - Pharmacists	Low Income	West	Pharmacist
8	Clinical Psychologist	807	887	986	0.47	21,294	Burkina Faso	Minimum Estimate	2634 - Psychologists	Low Income	West	Other Health Workers
9	Community health worker/Village health worker	13,500	14,697	16,010	7.62	1,313	Burkina Faso	Minimum Estimate	3253 - Community health workers	Low Income	West	Other Health Workers
10	Dental Surgery Assistant	1,361	1,515	1,695	0.80	12,435	Burkina Faso	Minimum Estimate	3251 - Dental assistants and therapists	Low Income	West	Other Health Workers
11	Dental Therapist	857	955	1,068	0.51	19,725	Burkina Faso	Minimum Estimate	3251 - Dental assistants and therapists	Low Income	West	Other Health Workers
12	Dentist	897	991	1,115	0.53	18,896	Burkina Faso	Minimum Estimate	2261 - Dentists	Low Income	West	Dentist
13	Dermatologist	55	61	68	0.03	310,163	Burkina Faso	Minimum Estimate	2212 - Specialist medical practitioners	Low Income	West	Medical Doctors
14	Endocrinologist	46	53	64	0.03	325,724	Burkina Faso	Minimum Estimate	2212 - Specialist medical practitioners	Low Income	West	Medical Doctors
15	ENT Surgeon	139	155	174	0.08	121,042	Burkina Faso	Minimum Estimate	2212 - Specialist medical practitioners	Low Income	West	Medical Doctors
16	Environmental Health Officer	519	573	629	0.30	33,514	Burkina Faso	Minimum Estimate	2263 - Environmental and occupational health and hygiene professionals	Low Income	West	Other Health Workers
17	Gastroenterologist	214	216	224	0.11	93,333	Burkina Faso	Minimum Estimate	2212 - Specialist medical practitioners	Low Income	West	Medical Doctors
18	General Medical Practitioner (Generalist Doctor)	5,808	6,383	7,025	3.34	2,995	Burkina Faso	Minimum Estimate	2211 - Generalist medical practitioners	Low Income	West	Medical Doctors
19	General Surgeon	138	157	180	0.09	116,508	Burkina Faso	Minimum Estimate	2212 - Specialist medical practitioners	Low Income	West	Medical Doctors
20	Haematologist	83	97	112	0.05	186,876	Burkina Faso	Minimum Estimate	2212 - Specialist medical practitioners	Low Income	West	Medical Doctors
21	Health Promoter/Health Educator	75	78	83	0.04	253,252	Burkina Faso	Minimum Estimate	2269 - Health professionals not elsewhere classified	Low Income	West	Other Health Workers
22	Infectious Diseases Specialist	35	37	40	0.02	525,449	Burkina Faso	Minimum Estimate	2212 - Specialist medical practitioners	Low Income	West	Medical Doctors
23	Intensive Care Nurse	565	621	683	0.32	30,826	Burkina Faso	Minimum Estimate	2221 - Nursing professionals	Low Income	West	Nursing Personnel
24	Medical Laboratory Scientist	1,701	1,862	2,066	0.99	10,141	Burkina Faso	Minimum Estimate	3212 - Medical and pathology laboratory technicians	Low Income	West	Other Health Workers
25	Medical Laboratory Technician	2,471	2,830	3,276	1.57	6,376	Burkina Faso	Minimum Estimate	3212 - Medical and pathology laboratory technicians	Low Income	West	Other Health Workers
26	Medical Social Worker	897	924	956	0.46	21,903	Burkina Faso	Minimum Estimate	1344 - Social welfare managers	Low Income	West	Other Health Workers
27	Mental Health Nurse	681	748	840	0.40	24,954	Burkina Faso	Minimum Estimate	2221 - Nursing professionals	Low Income	West	Nursing Personnel
28	Midwife	14,343	16,377	18,780	8.93	1,119	Burkina Faso	Minimum Estimate	2222 - Midwifery professionals	Low Income	West	Midwifery Personnel
29	Nephrologist	120	136	158	0.08	132,254	Burkina Faso	Minimum Estimate	2212 - Specialist medical practitioners	Low Income	West	Medical Doctors
30	Neuro-Surgeon	58	68	80	0.04	260,883	Burkina Faso	Minimum Estimate	2212 - Specialist medical practitioners	Low Income	West	Medical Doctors
31	Nurse Anaesthetist	299	353	417	0.20	50,368	Burkina Faso	Minimum Estimate	2221 - Nursing professionals	Low Income	West	Nursing Personnel
32	Nutritionist	3,618	3,781	3,940	1.87	5,336	Burkina Faso	Minimum Estimate	2265 - Dieticians and nutritionists	Low Income	West	Other Health Workers
33	Obstetrician & Gynaecologist	1,026	1,142	1,273	0.60	16,533	Burkina Faso	Minimum Estimate	2212 - Specialist medical practitioners	Low Income	West	Medical Doctors
34	Occupational Therapist	347	407	478	0.23	43,869	Burkina Faso	Minimum Estimate	2269 - Health professionals not elsewhere classified	Low Income	West	Other Health Workers
35	Oncology Nurse	96	110	129	0.06	162,729	Burkina Faso	Minimum Estimate	2221 - Nursing professionals	Low Income	West	Nursing Personnel
36	Operating Theatre Nurse	1,593	1,805	2,086	1.00	10,043	Burkina Faso	Minimum Estimate	2221 - Nursing professionals	Low Income	West	Nursing Personnel
37	Ophthalmic Nurse	165	185	208	0.10	100,998	Burkina Faso	Minimum Estimate	2221 - Nursing professionals	Low Income	West	Nursing Personnel
38	Ophthalmologist	44	50	57	0.03	367,597	Burkina Faso	Minimum Estimate	2212 - Specialist medical practitioners	Low Income	West	Medical Doctors
39	Optometrist	171	191	214	0.10	98,559	Burkina Faso	Minimum Estimate	2267 - Optometrists and ophthalmic opticians	Low Income	West	Other Health Workers
40	Orthopaedic Nurse	161	192	227	0.11	92,501	Burkina Faso	Minimum Estimate	2221 - Nursing professionals	Low Income	West	Nursing Personnel
41	Orthopaedic Surgeon	506	580	667	0.32	31,524	Burkina Faso	Minimum Estimate	2212 - Specialist medical practitioners	Low Income	West	Medical Doctors
42	Orthopaedic Technologist	532	599	672	0.32	31,340	Burkina Faso	Minimum Estimate	3214 - Medical and dental prosthetic technicians	Low Income	West	Other Health Workers
43	Paediatric Nurse	2,385	2,645	2,918	1.39	7,213	Burkina Faso	Minimum Estimate	2221 - Nursing professionals	Low Income	West	Nursing Personnel
44	Paediatric Surgeon	64	71	79	0.04	268,867	Burkina Faso	Minimum Estimate	2212 - Specialist medical practitioners	Low Income	West	Medical Doctors
45	Paediatrician	668	742	830	0.39	25,324	Burkina Faso	Minimum Estimate	2212 - Specialist medical practitioners	Low Income	West	Medical Doctors
46	Pathologist	265	287	312	0.15	67,412	Burkina Faso	Minimum Estimate	2212 - Specialist medical practitioners	Low Income	West	Medical Doctors
47	Pharmacist	979	1,015	1,057	0.50	19,819	Burkina Faso	Minimum Estimate	2262 - Pharmacists	Low Income	West	Pharmacist
48	Pharmacy Technician	1,584	1,744	1,924	0.92	10,929	Burkina Faso	Minimum Estimate	3213 - Pharmaceutical technicians and assistants	Low Income	West	Other Health Workers
49	Physician	1,711	1,903	2,129	1.01	9,868	Burkina Faso	Minimum Estimate	2212 - Specialist medical practitioners	Low Income	West	Medical Doctors
50	Physiotherapist	376	422	474	0.22	44,446	Burkina Faso	Minimum Estimate	2264 - Physiotherapists	Low Income	West	Other Health Workers
51	Plastic Surgeon	95	110	127	0.06	166,034	Burkina Faso	Minimum Estimate	2212 - Specialist medical practitioners	Low Income	West	Medical Doctors
52	Psychiatrist	820	888	977	0.47	21,489	Burkina Faso	Minimum Estimate	2212 - Specialist medical practitioners	Low Income	West	Medical Doctors
53	Radiation Oncologist	26	31	37	0.02	562,884	Burkina Faso	Minimum Estimate	2212 - Specialist medical practitioners	Low Income	West	Medical Doctors
54	Radiographer (Diagnostics and Therapy)	1,082	1,207	1,365	0.65	15,368	Burkina Faso	Minimum Estimate	3211 - Medical imaging and therapeutic equipment technicians	Low Income	West	Other Health Workers
55	Radiologist	352	393	444	0.21	47,134	Burkina Faso	Minimum Estimate	2212 - Specialist medical practitioners	Low Income	West	Medical Doctors
56	Registered General Nurse / State Certified Nurse	35,471	39,555	44,248	21.04	475	Burkina Faso	Minimum Estimate	2221 - Nursing professionals	Low Income	West	Nursing Personnel
57	Renal Nurse	1,492	1,687	1,960	0.94	10,673	Burkina Faso	Minimum Estimate	2221 - Nursing professionals	Low Income	West	Nursing Personnel
58	Respiratory Physician	93	105	119	0.06	177,588	Burkina Faso	Minimum Estimate	2212 - Specialist medical practitioners	Low Income	West	Medical Doctors
59	Rheumatologist	42	47	52	0.02	403,752	Burkina Faso	Minimum Estimate	2212 - Specialist medical practitioners	Low Income	West	Medical Doctors
60	Speech Therapist	147	162	179	0.09	117,429	Burkina Faso	Minimum Estimate	2266 - Audiologists and speech therapists	Low Income	West	Other Health Workers
61	Urologist	12	15	18	0.01	1,173,447	Burkina Faso	Minimum Estimate	2212 - Specialist medical practitioners	Low Income	West	Medical Doctors
1	Anaesthesiologist	71	80	89	0.07	134,270	Burundi	Minimum Estimate	2212 - Specialist medical practitioners	Low Income	Central	Medical Doctors
2	Associate Nurse/Enrolled Nurse/Nursing Assistant	10,509	12,017	13,915	11.32	884	Burundi	Minimum Estimate	3221 - Nursing associate professionals	Low Income	Central	Nursing Personnel
3	Audiologist	30	33	37	0.03	324,587	Burundi	Minimum Estimate	2266 - Audiologists and speech therapists	Low Income	Central	Other Health Workers
4	Cardiologist	29	33	37	0.03	327,112	Burundi	Minimum Estimate	2212 - Specialist medical practitioners	Low Income	Central	Medical Doctors
5	Cardiothoracic Surgeon	13	14	16	0.01	762,939	Burundi	Minimum Estimate	2212 - Specialist medical practitioners	Low Income	Central	Medical Doctors
6	Clinical Officer/Physician Assistant	1,929	2,720	3,930	2.91	3,431	Burundi	Minimum Estimate	3256 - Medical assistants	Low Income	Central	Other Health Workers
7	Clinical Pharmacist	188	209	232	0.19	51,364	Burundi	Minimum Estimate	2262 - Pharmacists	Low Income	Central	Pharmacist
8	Clinical Psychologist	536	595	659	0.55	18,130	Burundi	Minimum Estimate	2634 - Psychologists	Low Income	Central	Other Health Workers
9	Community health worker/Village health worker	12,442	17,285	24,554	18.30	546	Burundi	Minimum Estimate	3253 - Community health workers	Low Income	Central	Other Health Workers
10	Dental Surgery Assistant	688	768	863	0.72	13,861	Burundi	Minimum Estimate	3251 - Dental assistants and therapists	Low Income	Central	Other Health Workers
11	Dental Therapist	426	476	534	0.45	22,404	Burundi	Minimum Estimate	3251 - Dental assistants and therapists	Low Income	Central	Other Health Workers
12	Dentist	581	646	731	0.61	16,314	Burundi	Minimum Estimate	2261 - Dentists	Low Income	Central	Dentist
13	Dermatologist	28	31	35	0.03	344,812	Burundi	Minimum Estimate	2212 - Specialist medical practitioners	Low Income	Central	Medical Doctors
14	Endocrinologist	22	25	30	0.03	398,219	Burundi	Minimum Estimate	2212 - Specialist medical practitioners	Low Income	Central	Medical Doctors
15	ENT Surgeon	74	83	95	0.08	125,875	Burundi	Minimum Estimate	2212 - Specialist medical practitioners	Low Income	Central	Medical Doctors
16	Environmental Health Officer	295	328	362	0.30	33,165	Burundi	Minimum Estimate	2263 - Environmental and occupational health and hygiene professionals	Low Income	Central	Other Health Workers
17	Gastroenterologist	45	46	49	0.04	242,848	Burundi	Minimum Estimate	2212 - Specialist medical practitioners	Low Income	Central	Medical Doctors
18	General Medical Practitioner (Generalist Doctor)	3,239	3,903	4,807	3.81	2,622	Burundi	Minimum Estimate	2211 - Generalist medical practitioners	Low Income	Central	Medical Doctors
19	General Surgeon	55	64	75	0.06	158,253	Burundi	Minimum Estimate	2212 - Specialist medical practitioners	Low Income	Central	Medical Doctors
20	Haematologist	22	24	26	0.02	462,981	Burundi	Minimum Estimate	2212 - Specialist medical practitioners	Low Income	Central	Medical Doctors

S/N	Health Professionals	2022	2026	2030	Density per 10,000 population	Required Population ratio (1 professional is to xxx population)	Name of Country	Modelling Scenario	ISCO-08 Match	Income Group Classification	Sub-Regional Grouping	SDG 3c Occupation
21	Health Promoter/Health Educator	90	122	172	0.13	77,712	Burundi	Minimum Estimate	2269 - Health professionals not elsewhere classified	Low Income	Central	Other Health Workers
22	Infectious Diseases Specialist	7	7	8	0.01	1,477,803	Burundi	Minimum Estimate	2212 - Specialist medical practitioners	Low Income	Central	Medical Doctors
23	Intensive Care Nurse	250	279	310	0.26	38,728	Burundi	Minimum Estimate	2221 - Nursing professionals	Low Income	Central	Nursing Personnel
24	Medical Laboratory Scientist	1,150	1,519	2,064	1.58	6,327	Burundi	Minimum Estimate	3212 - Medical and pathology laboratory technicians	Low Income	Central	Other Health Workers
25	Medical Laboratory Technician	2,725	4,197	6,483	4.69	2,130	Burundi	Minimum Estimate	3212 - Medical and pathology laboratory technicians	Low Income	Central	Other Health Workers
26	Medical Social Worker	309	335	362	0.30	32,808	Burundi	Minimum Estimate	1344 - Social welfare managers	Low Income	Central	Other Health Workers
27	Mental Health Nurse	393	439	494	0.42	24,031	Burundi	Minimum Estimate	2221 - Nursing professionals	Low Income	Central	Nursing Personnel
28	Midwife	6,387	7,010	7,740	6.48	1,543	Burundi	Minimum Estimate	2222 - Midwifery professionals	Low Income	Central	Midwifery Personnel
29	Nephrologist	59	67	77	0.06	154,288	Burundi	Minimum Estimate	2212 - Specialist medical practitioners	Low Income	Central	Medical Doctors
30	Neuro-Surgeon	26	29	34	0.03	354,237	Burundi	Minimum Estimate	2212 - Specialist medical practitioners	Low Income	Central	Medical Doctors
31	Nurse Anaesthetist	133	149	166	0.14	72,046	Burundi	Minimum Estimate	2221 - Nursing professionals	Low Income	Central	Nursing Personnel
32	Nutritionist	1,395	1,518	1,639	1.37	7,315	Burundi	Minimum Estimate	2265 - Dieticians and nutritionists	Low Income	Central	Other Health Workers
33	Obstetrician & Gynaecologist	606	677	756	0.63	15,777	Burundi	Minimum Estimate	2212 - Specialist medical practitioners	Low Income	Central	Medical Doctors
34	Occupational Therapist	145	164	184	0.15	64,897	Burundi	Minimum Estimate	2269 - Health professionals not elsewhere classified	Low Income	Central	Other Health Workers
35	Oncology Nurse	47	53	60	0.05	196,356	Burundi	Minimum Estimate	2221 - Nursing professionals	Low Income	Central	Nursing Personnel
36	Operating Theatre Nurse	806	909	1,031	0.87	11,507	Burundi	Minimum Estimate	2221 - Nursing professionals	Low Income	Central	Nursing Personnel
37	Ophthalmic Nurse	57	65	73	0.06	163,455	Burundi	Minimum Estimate	2221 - Nursing professionals	Low Income	Central	Nursing Personnel
38	Ophthalmologist	21	25	28	0.02	423,512	Burundi	Minimum Estimate	2212 - Specialist medical practitioners	Low Income	Central	Medical Doctors
39	Optometrist	98	111	126	0.11	94,969	Burundi	Minimum Estimate	2267 - Optometrists and ophthalmic opticians	Low Income	Central	Other Health Workers
40	Orthopaedic Nurse	74	83	93	0.08	128,320	Burundi	Minimum Estimate	2221 - Nursing professionals	Low Income	Central	Nursing Personnel
41	Orthopaedic Surgeon	106	120	135	0.11	88,371	Burundi	Minimum Estimate	2212 - Specialist medical practitioners	Low Income	Central	Medical Doctors
42	Orthopaedic Technologist	290	323	358	0.30	33,502	Burundi	Minimum Estimate	3214 - Medical and dental prosthetic technicians	Low Income	Central	Other Health Workers
43	Paediatric Nurse	987	1,081	1,162	0.97	10,315	Burundi	Minimum Estimate	2221 - Nursing professionals	Low Income	Central	Nursing Personnel
44	Paediatric Surgeon	35	39	43	0.04	282,621	Burundi	Minimum Estimate	2212 - Specialist medical practitioners	Low Income	Central	Medical Doctors
45	Paediatrician	354	384	414	0.35	28,890	Burundi	Minimum Estimate	2212 - Specialist medical practitioners	Low Income	Central	Medical Doctors
46	Pathologist	59	66	75	0.06	158,587	Burundi	Minimum Estimate	2212 - Specialist medical practitioners	Low Income	Central	Medical Doctors
47	Pharmacist	646	688	731	0.61	16,331	Burundi	Minimum Estimate	2262 - Pharmacists	Low Income	Central	Pharmacist
48	Pharmacy Technician	727	802	882	0.74	13,553	Burundi	Minimum Estimate	3213 - Pharmaceutical technicians and assistants	Low Income	Central	Other Health Workers
49	Physician	742	872	1,044	0.84	11,868	Burundi	Minimum Estimate	2212 - Specialist medical practitioners	Low Income	Central	Medical Doctors
50	Physiotherapist	207	231	257	0.21	46,600	Burundi	Minimum Estimate	2264 - Physiotherapists	Low Income	Central	Other Health Workers
51	Plastic Surgeon	84	95	108	0.09	111,086	Burundi	Minimum Estimate	2212 - Specialist medical practitioners	Low Income	Central	Medical Doctors
52	Psychiatrist	408	452	504	0.42	23,627	Burundi	Minimum Estimate	2212 - Specialist medical practitioners	Low Income	Central	Medical Doctors
53	Radiation Oncologist	13	14	16	0.01	740,970	Burundi	Minimum Estimate	2212 - Specialist medical practitioners	Low Income	Central	Medical Doctors
54	Radiographer (Diagnostics and Therapy)	432	487	552	0.47	21,476	Burundi	Minimum Estimate	3211 - Medical imaging and therapeutic equipment technicians	Low Income	Central	Other Health Workers
55	Radiologist	125	142	163	0.14	71,548	Burundi	Minimum Estimate	2212 - Specialist medical practitioners	Low Income	Central	Medical Doctors
56	Registered General Nurse / State Certified Nurse	21,255	22,827	24,453	20.52	487	Burundi	Minimum Estimate	2221 - Nursing professionals	Low Income	Central	Nursing Personnel
57	Renal Nurse	735	836	957	0.81	12,366	Burundi	Minimum Estimate	2221 - Nursing professionals	Low Income	Central	Nursing Personnel
58	Respiratory Physician	73	78	83	0.07	144,733	Burundi	Minimum Estimate	2212 - Specialist medical practitioners	Low Income	Central	Medical Doctors
59	Rheumatologist	23	26	29	0.02	407,344	Burundi	Minimum Estimate	2212 - Specialist medical practitioners	Low Income	Central	Medical Doctors
60	Speech Therapist	86	96	107	0.09	111,750	Burundi	Minimum Estimate	2266 - Audiologists and speech therapists	Low Income	Central	Other Health Workers
61	Urologist	7	8	10	0.01	1,233,164	Burundi	Minimum Estimate	2212 - Specialist medical practitioners	Low Income	Central	Medical Doctors
1	Anaesthesiologist	6	6	7	0.13	77,776	Cabo Verde	Minimum Estimate	2212 - Specialist medical practitioners	Lower-middle Income	West	Medical Doctors
2	Associate Nurse/Enrolled Nurse/Nursing Assistant	559	594	635	11.36	880	Cabo Verde	Minimum Estimate	3221 - Nursing associate professionals	Lower-middle Income	West	Nursing Personnel
3	Audiologist	2	2	2	0.03	320,480	Cabo Verde	Minimum Estimate	2266 - Audiologists and speech therapists	Lower-middle Income	West	Other Health Workers
4	Cardiologist	4	4	4	0.07	133,509	Cabo Verde	Minimum Estimate	2212 - Specialist medical practitioners	Lower-middle Income	West	Medical Doctors
5	Cardiothoracic Surgeon	1	1	1	0.03	378,073	Cabo Verde	Minimum Estimate	2212 - Specialist medical practitioners	Lower-middle Income	West	Medical Doctors
6	Clinical Officer/Physician Assistant	43	46	50	0.89	11,193	Cabo Verde	Minimum Estimate	3256 - Medical assistants	Lower-middle Income	West	Other Health Workers
7	Clinical Pharmacist	15	16	18	0.32	31,205	Cabo Verde	Minimum Estimate	2262 - Pharmacists	Lower-middle Income	West	Pharmacist
8	Clinical Psychologist	31	33	35	0.62	16,125	Cabo Verde	Minimum Estimate	2634 - Psychologists	Lower-middle Income	West	Other Health Workers
9	Community health worker/Village health worker	217	220	223	4.00	2,499	Cabo Verde	Minimum Estimate	3253 - Community health workers	Lower-middle Income	West	Other Health Workers
10	Dental Surgery Assistant	60	65	70	1.25	8,023	Cabo Verde	Minimum Estimate	3251 - Dental assistants and therapists	Lower-middle Income	West	Other Health Workers
11	Dental Therapist	38	41	44	0.79	12,605	Cabo Verde	Minimum Estimate	3251 - Dental assistants and therapists	Lower-middle Income	West	Other Health Workers
12	Dentist	35	37	38	0.68	14,638	Cabo Verde	Minimum Estimate	2261 - Dentists	Lower-middle Income	West	Dentist
13	Dermatologist	2	2	2	0.04	283,335	Cabo Verde	Minimum Estimate	2212 - Specialist medical practitioners	Lower-middle Income	West	Medical Doctors
14	Endocrinologist	4	5	6	0.10	100,779	Cabo Verde	Minimum Estimate	2212 - Specialist medical practitioners	Lower-middle Income	West	Medical Doctors
15	ENT Surgeon	6	7	8	0.14	70,494	Cabo Verde	Minimum Estimate	2212 - Specialist medical practitioners	Lower-middle Income	West	Medical Doctors
16	Environmental Health Officer	14	14	15	0.27	37,591	Cabo Verde	Minimum Estimate	2263 - Environmental and occupational health and hygiene professionals	Lower-middle Income	West	Other Health Workers
17	Gastroenterologist	5	5	5	0.09	110,232	Cabo Verde	Minimum Estimate	2212 - Specialist medical practitioners	Lower-middle Income	West	Medical Doctors
18	General Medical Practitioner (Generalist Doctor)	168	178	190	3.40	2,938	Cabo Verde	Minimum Estimate	2211 - Generalist medical practitioners	Lower-middle Income	West	Medical Doctors
19	General Surgeon	7	8	10	0.17	58,051	Cabo Verde	Minimum Estimate	2212 - Specialist medical practitioners	Lower-middle Income	West	Medical Doctors
20	Haematologist	1	2	2	0.03	312,246	Cabo Verde	Minimum Estimate	2212 - Specialist medical practitioners	Lower-middle Income	West	Medical Doctors
21	Health Promoter/Health Educator	2	2	2	0.04	261,950	Cabo Verde	Minimum Estimate	2269 - Health professionals not elsewhere classified	Lower-middle Income	West	Other Health Workers
22	Infectious Diseases Specialist	1	1	1	0.01	730,091	Cabo Verde	Minimum Estimate	2212 - Specialist medical practitioners	Lower-middle Income	West	Medical Doctors
23	Intensive Care Nurse	12	12	13	0.24	42,156	Cabo Verde	Minimum Estimate	2221 - Nursing professionals	Lower-middle Income	West	Nursing Personnel
24	Medical Laboratory Scientist	58	63	69	1.23	8,126	Cabo Verde	Minimum Estimate	3212 - Medical and pathology laboratory technicians	Lower-middle Income	West	Other Health Workers
25	Medical Laboratory Technician	46	49	53	0.96	10,435	Cabo Verde	Minimum Estimate	3212 - Medical and pathology laboratory technicians	Lower-middle Income	West	Other Health Workers
26	Medical Social Worker	13	13	13	0.24	42,460	Cabo Verde	Minimum Estimate	1344 - Social welfare managers	Lower-middle Income	West	Other Health Workers
27	Mental Health Nurse	26	28	30	0.53	18,803	Cabo Verde	Minimum Estimate	2221 - Nursing professionals	Lower-middle Income	West	Nursing Personnel
28	Midwife	316	328	338	6.04	1,655	Cabo Verde	Minimum Estimate	2222 - Midwifery professionals	Lower-middle Income	West	Midwifery Personnel
29	Nephrologist	9	10	11	0.19	51,525	Cabo Verde	Minimum Estimate	2212 - Specialist medical practitioners	Lower-middle Income	West	Medical Doctors
30	Neuro-Surgeon	3	3	4	0.07	144,482	Cabo Verde	Minimum Estimate	2212 - Specialist medical practitioners	Lower-middle Income	West	Medical Doctors
31	Nurse Anaesthetist	22	27	34	0.61	16,367	Cabo Verde	Minimum Estimate	2221 - Nursing professionals	Lower-middle Income	West	Nursing Personnel
32	Nutritionist	33	33	34	0.61	16,443	Cabo Verde	Minimum Estimate	2265 - Dieticians and nutritionists	Lower-middle Income	West	Other Health Workers
33	Obstetrician & Gynaecologist	33	35	38	0.67	14,891	Cabo Verde	Minimum Estimate	2212 - Specialist medical practitioners	Lower-middle Income	West	Medical Doctors
34	Occupational Therapist	13	14	17	0.30	33,707	Cabo Verde	Minimum Estimate	2269 - Health professionals not elsewhere classified	Lower-middle Income	West	Other Health Workers
35	Oncology Nurse	8	9	11	0.20	50,246	Cabo Verde	Minimum Estimate	2221 - Nursing professionals	Lower-middle Income	West	Nursing Personnel
36	Operating Theatre Nurse	84	92	105	1.88	5,330	Cabo Verde	Minimum Estimate	2221 - Nursing professionals	Lower-middle Income	West	Nursing Personnel
37	Ophthalmic Nurse	7	8	9	0.16	61,609	Cabo Verde	Minimum Estimate	2221 - Nursing professionals	Lower-middle Income	West	Nursing Personnel
38	Ophthalmologist	3	3	4	0.07	149,945	Cabo Verde	Minimum Estimate	2212 - Specialist medical practitioners	Lower-middle Income	West	Medical Doctors
39	Optometrist	10	11	12	0.21	46,589	Cabo Verde	Minimum Estimate	2267 - Optometrists and ophthalmic opticians	Lower-middle Income	West	Other Health Workers
40	Orthopaedic Nurse	5	6	7	0.12	81,866	Cabo Verde	Minimum Estimate	2221 - Nursing professionals	Lower-middle Income	West	Nursing Personnel
41	Orthopaedic Surgeon	24	27	31	0.55	18,309	Cabo Verde	Minimum Estimate	2212 - Specialist medical practitioners	Lower-middle Income	West	Medical Doctors
42	Orthopaedic Technologist	15	16	18	0.32	31,700	Cabo Verde	Minimum Estimate	3214 - Medical and dental prosthetic technicians	Lower-middle Income	West	Other Health Workers
43	Paediatric Nurse	35	36	36	0.64	15,512	Cabo Verde	Minimum Estimate	2221 - Nursing professionals	Lower-middle Income	West	Nursing Personnel

S/N	Health Professionals	2022	2026	2030	Density per 10,000 population	Required Population ratio (1 professional is to xxx population)	Name of Country	Modelling Scenario	ISCO-08 Match	Income Group Classification	Sub-Regional Grouping	SDG 3c Occupation
44	Paediatric Surgeon	1	1	1	0.02	596,693	Cabo Verde	Minimum Estimate	2212 - Specialist medical practitioners	Lower-middle Income	West	Medical Doctors
45	Paediatrician	18	19	21	0.38	26,502	Cabo Verde	Minimum Estimate	2212 - Specialist medical practitioners	Lower-middle Income	West	Medical Doctors
46	Pathologist	5	6	6	0.11	89,990	Cabo Verde	Minimum Estimate	2212 - Specialist medical practitioners	Lower-middle Income	West	Medical Doctors
47	Pharmacist	28	29	30	0.54	18,420	Cabo Verde	Minimum Estimate	2262 - Pharmacists	Lower-middle Income	West	Pharmacist
48	Pharmacy Technician	50	53	57	1.02	9,782	Cabo Verde	Minimum Estimate	3213 - Pharmaceutical technicians and assistants	Lower-middle Income	West	Other Health Workers
49	Physician	56	61	67	1.19	8,392	Cabo Verde	Minimum Estimate	2212 - Specialist medical practitioners	Lower-middle Income	West	Medical Doctors
50	Physiotherapist	12	13	15	0.27	37,651	Cabo Verde	Minimum Estimate	2264 - Physiotherapists	Lower-middle Income	West	Other Health Workers
51	Plastic Surgeon	4	4	5	0.09	115,183	Cabo Verde	Minimum Estimate	2212 - Specialist medical practitioners	Lower-middle Income	West	Medical Doctors
52	Psychiatrist	26	27	29	0.51	19,438	Cabo Verde	Minimum Estimate	2212 - Specialist medical practitioners	Lower-middle Income	West	Medical Doctors
53	Radiation Oncologist	3	4	6	0.10	98,209	Cabo Verde	Minimum Estimate	2212 - Specialist medical practitioners	Lower-middle Income	West	Medical Doctors
54	Radiographer (Diagnostics and Therapy)	50	54	60	1.08	9,281	Cabo Verde	Minimum Estimate	3211 - Medical imaging and therapeutic equipment technicians	Lower-middle Income	West	Other Health Workers
55	Radiologist	14	16	18	0.31	31,838	Cabo Verde	Minimum Estimate	2212 - Specialist medical practitioners	Lower-middle Income	West	Medical Doctors
56	Registered General Nurse / State Certified Nurse	1,305	1,429	1,588	28.39	352	Cabo Verde	Minimum Estimate	2221 - Nursing professionals	Lower-middle Income	West	Nursing Personnel
57	Renal Nurse	107	120	137	2.45	4,087	Cabo Verde	Minimum Estimate	2221 - Nursing professionals	Lower-middle Income	West	Nursing Personnel
58	Respiratory Physician	3	3	3	0.06	170,052	Cabo Verde	Minimum Estimate	2212 - Specialist medical practitioners	Lower-middle Income	West	Medical Doctors
59	Rheumatologist	2	2	2	0.04	283,748	Cabo Verde	Minimum Estimate	2212 - Specialist medical practitioners	Lower-middle Income	West	Medical Doctors
60	Speech Therapist	5	5	5	0.10	100,205	Cabo Verde	Minimum Estimate	2266 - Audiologists and speech therapists	Lower-middle Income	West	Other Health Workers
61	Urologist	1	1	2	0.03	323,913	Cabo Verde	Minimum Estimate	2212 - Specialist medical practitioners	Lower-middle Income	West	Medical Doctors
1	Anaesthesiologist	253	285	321	0.12	83,380	Cameroon	Minimum Estimate	2212 - Specialist medical practitioners	Lower-middle Income	Central	Medical Doctors
2	Associate Nurse/Enrolled Nurse/Nursing Assistant	23,993	26,767	29,878	11.17	895	Cameroon	Minimum Estimate	3221 - Nursing associate professionals	Lower-middle Income	Central	Nursing Personnel
3	Audiologist	65	72	80	0.03	333,445	Cameroon	Minimum Estimate	2266 - Audiologists and speech therapists	Lower-middle Income	Central	Other Health Workers
4	Cardiologist	75	85	96	0.04	278,820	Cameroon	Minimum Estimate	2212 - Specialist medical practitioners	Lower-middle Income	Central	Medical Doctors
5	Cardiothoracic Surgeon	34	39	43	0.02	615,742	Cameroon	Minimum Estimate	2212 - Specialist medical practitioners	Lower-middle Income	Central	Medical Doctors
6	Clinical Officer/Physician Assistant	2,273	2,493	2,772	1.04	9,593	Cameroon	Minimum Estimate	3256 - Medical assistants	Lower-middle Income	Central	Other Health Workers
7	Clinical Pharmacist	529	595	678	0.25	39,236	Cameroon	Minimum Estimate	2262 - Pharmacists	Lower-middle Income	Central	Pharmacist
8	Clinical Psychologist	1,386	1,553	1,747	0.65	15,303	Cameroon	Minimum Estimate	2634 - Psychologists	Lower-middle Income	Central	Other Health Workers
9	Community health worker/Village health worker	15,259	16,688	18,204	6.81	1,468	Cameroon	Minimum Estimate	3253 - Community health workers	Lower-middle Income	Central	Other Health Workers
10	Dental Surgery Assistant	2,041	2,313	2,628	0.98	10,180	Cameroon	Minimum Estimate	3251 - Dental assistants and therapists	Lower-middle Income	Central	Other Health Workers
11	Dental Therapist	1,284	1,456	1,655	0.62	16,173	Cameroon	Minimum Estimate	3251 - Dental assistants and therapists	Lower-middle Income	Central	Other Health Workers
12	Dentist	1,366	1,537	1,754	0.66	15,242	Cameroon	Minimum Estimate	2261 - Dentists	Lower-middle Income	Central	Dentist
13	Dermatologist	73	83	93	0.03	289,580	Cameroon	Minimum Estimate	2212 - Specialist medical practitioners	Lower-middle Income	Central	Medical Doctors
14	Endocrinologist	65	77	94	0.04	283,117	Cameroon	Minimum Estimate	2212 - Specialist medical practitioners	Lower-middle Income	Central	Medical Doctors
15	ENT Surgeon	193	219	246	0.09	108,427	Cameroon	Minimum Estimate	2212 - Specialist medical practitioners	Lower-middle Income	Central	Medical Doctors
16	Environmental Health Officer	659	730	803	0.30	33,358	Cameroon	Minimum Estimate	2263 - Environmental and occupational health and hygiene professionals	Lower-middle Income	Central	Other Health Workers
17	Gastroenterologist	150	155	164	0.06	161,917	Cameroon	Minimum Estimate	2212 - Specialist medical practitioners	Lower-middle Income	Central	Medical Doctors
18	General Medical Practitioner (Generalist Doctor)	7,291	8,094	8,997	3.37	2,969	Cameroon	Minimum Estimate	2211 - Generalist medical practitioners	Lower-middle Income	Central	Medical Doctors
19	General Surgeon	185	213	248	0.09	107,298	Cameroon	Minimum Estimate	2212 - Specialist medical practitioners	Lower-middle Income	Central	Medical Doctors
20	Haematologist	55	60	64	0.02	415,246	Cameroon	Minimum Estimate	2212 - Specialist medical practitioners	Lower-middle Income	Central	Medical Doctors
21	Health Promoter/Health Educator	116	126	140	0.05	189,080	Cameroon	Minimum Estimate	2269 - Health professionals not elsewhere classified	Lower-middle Income	Central	Other Health Workers
22	Infectious Diseases Specialist	22	23	25	0.01	1,051,747	Cameroon	Minimum Estimate	2212 - Specialist medical practitioners	Lower-middle Income	Central	Medical Doctors
23	Intensive Care Nurse	610	664	720	0.27	37,165	Cameroon	Minimum Estimate	2221 - Nursing professionals	Lower-middle Income	Central	Nursing Personnel
24	Medical Laboratory Scientist	2,250	2,500	2,819	1.06	9,421	Cameroon	Minimum Estimate	3212 - Medical and pathology laboratory technicians	Lower-middle Income	Central	Other Health Workers
25	Medical Laboratory Technician	3,138	3,516	3,962	1.49	6,725	Cameroon	Minimum Estimate	3212 - Medical and pathology laboratory technicians	Lower-middle Income	Central	Other Health Workers
26	Medical Social Worker	596	638	696	0.27	37,688	Cameroon	Minimum Estimate	1344 - Social welfare managers	Lower-middle Income	Central	Other Health Workers
27	Mental Health Nurse	877	985	1,133	0.43	23,486	Cameroon	Minimum Estimate	2221 - Nursing professionals	Lower-middle Income	Central	Nursing Personnel
28	Midwife	14,634	16,075	17,697	6.61	1,513	Cameroon	Minimum Estimate	2222 - Midwifery professionals	Lower-middle Income	Central	Midwifery Personnel
29	Nephrologist	224	260	311	0.12	85,371	Cameroon	Minimum Estimate	2212 - Specialist medical practitioners	Lower-middle Income	Central	Medical Doctors
30	Neuro-Surgeon	109	122	136	0.05	195,905	Cameroon	Minimum Estimate	2212 - Specialist medical practitioners	Lower-middle Income	Central	Medical Doctors
31	Nurse Anaesthetist	417	487	567	0.21	47,122	Cameroon	Minimum Estimate	2221 - Nursing professionals	Lower-middle Income	Central	Nursing Personnel
32	Nutritionist	1,986	2,067	2,140	0.80	12,486	Cameroon	Minimum Estimate	2265 - Dieticians and nutritionists	Lower-middle Income	Central	Other Health Workers
33	Obstetrician & Gynaecologist	1,456	1,646	1,873	0.71	14,183	Cameroon	Minimum Estimate	2212 - Specialist medical practitioners	Lower-middle Income	Central	Medical Doctors
34	Occupational Therapist	590	668	757	0.28	35,279	Cameroon	Minimum Estimate	2269 - Health professionals not elsewhere classified	Lower-middle Income	Central	Other Health Workers
35	Oncology Nurse	161	188	224	0.08	118,656	Cameroon	Minimum Estimate	2221 - Nursing professionals	Lower-middle Income	Central	Nursing Personnel
36	Operating Theatre Nurse	2,452	2,781	3,214	1.21	8,280	Cameroon	Minimum Estimate	2221 - Nursing professionals	Lower-middle Income	Central	Nursing Personnel
37	Ophthalmic Nurse	189	218	254	0.10	105,195	Cameroon	Minimum Estimate	2221 - Nursing professionals	Lower-middle Income	Central	Nursing Personnel
38	Ophthalmologist	58	67	78	0.03	341,932	Cameroon	Minimum Estimate	2212 - Specialist medical practitioners	Lower-middle Income	Central	Medical Doctors
39	Optometrist	213	242	275	0.10	97,263	Cameroon	Minimum Estimate	2267 - Optometrists and ophthalmic opticians	Lower-middle Income	Central	Other Health Workers
40	Orthopaedic Nurse	316	361	410	0.15	65,241	Cameroon	Minimum Estimate	2221 - Nursing professionals	Lower-middle Income	Central	Nursing Personnel
41	Orthopaedic Surgeon	792	916	1,063	0.40	25,120	Cameroon	Minimum Estimate	2212 - Specialist medical practitioners	Lower-middle Income	Central	Medical Doctors
42	Orthopaedic Technologist	739	828	923	0.34	29,020	Cameroon	Minimum Estimate	3214 - Medical and dental prosthetic technicians	Lower-middle Income	Central	Other Health Workers
43	Paediatric Nurse	1,874	2,030	2,172	0.81	12,330	Cameroon	Minimum Estimate	2221 - Nursing professionals	Lower-middle Income	Central	Nursing Personnel
44	Paediatric Surgeon	67	73	78	0.03	344,389	Cameroon	Minimum Estimate	2212 - Specialist medical practitioners	Lower-middle Income	Central	Medical Doctors
45	Paediatrician	734	831	945	0.35	28,251	Cameroon	Minimum Estimate	2212 - Specialist medical practitioners	Lower-middle Income	Central	Medical Doctors
46	Pathologist	156	178	208	0.08	127,740	Cameroon	Minimum Estimate	2212 - Specialist medical practitioners	Lower-middle Income	Central	Medical Doctors
47	Pharmacist	1,116	1,152	1,207	0.46	21,899	Cameroon	Minimum Estimate	2262 - Pharmacists	Lower-middle Income	Central	Pharmacist
48	Pharmacy Technician	2,058	2,292	2,559	0.96	10,437	Cameroon	Minimum Estimate	3213 - Pharmaceutical technicians and assistants	Lower-middle Income	Central	Other Health Workers
49	Physician	1,993	2,249	2,550	0.95	10,474	Cameroon	Minimum Estimate	2212 - Specialist medical practitioners	Lower-middle Income	Central	Medical Doctors
50	Physiotherapist	529	592	662	0.25	40,386	Cameroon	Minimum Estimate	2264 - Physiotherapists	Lower-middle Income	Central	Other Health Workers
51	Plastic Surgeon	135	154	174	0.07	153,621	Cameroon	Minimum Estimate	2212 - Specialist medical practitioners	Lower-middle Income	Central	Medical Doctors
52	Psychiatrist	913	1,017	1,154	0.43	23,091	Cameroon	Minimum Estimate	2212 - Specialist medical practitioners	Lower-middle Income	Central	Medical Doctors
53	Radiation Oncologist	38	45	54	0.02	497,149	Cameroon	Minimum Estimate	2212 - Specialist medical practitioners	Lower-middle Income	Central	Medical Doctors
54	Radiographer (Diagnostics and Therapy)	1,545	1,775	2,072	0.78	12,804	Cameroon	Minimum Estimate	3211 - Medical imaging and therapeutic equipment technicians	Lower-middle Income	Central	Other Health Workers
55	Radiologist	469	551	662	0.25	39,473	Cameroon	Minimum Estimate	2212 - Specialist medical practitioners	Lower-middle Income	Central	Medical Doctors
56	Registered General Nurse / State Certified Nurse	46,774	52,028	58,371	21.91	456	Cameroon	Minimum Estimate	2221 - Nursing professionals	Lower-middle Income	Central	Nursing Personnel
57	Renal Nurse	2,755	3,205	3,829	1.44	6,928	Cameroon	Minimum Estimate	2221 - Nursing professionals	Lower-middle Income	Central	Nursing Personnel
58	Respiratory Physician	114	130	150	0.06	178,687	Cameroon	Minimum Estimate	2212 - Specialist medical practitioners	Lower-middle Income	Central	Medical Doctors
59	Rheumatologist	57	65	73	0.03	368,592	Cameroon	Minimum Estimate	2212 - Specialist medical practitioners	Lower-middle Income	Central	Medical Doctors
60	Speech Therapist	191	211	233	0.09	114,708	Cameroon	Minimum Estimate	2266 - Audiologists and speech therapists	Lower-middle Income	Central	Other Health Workers
61	Urologist	28	37	51	0.02	492,489	Cameroon	Minimum Estimate	2212 - Specialist medical practitioners	Lower-middle Income	Central	Medical Doctors
1	Anaesthesiologist	29	33	38	0.08	126,827	Central African Rep	Minimum Estimate	2212 - Specialist medical practitioners	Low Income	Central	Medical Doctors
2	Associate Nurse/Enrolled Nurse/Nursing Assistant	4,856	5,459	6,213	12.78	783	Central African Rep	Minimum Estimate	3221 - Nursing associate professionals	Low Income	Central	Nursing Personnel
3	Audiologist	12	14	16	0.03	295,795	Central African Rep	Minimum Estimate	2266 - Audiologists and speech therapists	Low Income	Central	Other Health Workers
4	Cardiologist	13	15	17	0.04	283,774	Central African Rep	Minimum Estimate	2212 - Specialist medical practitioners	Low Income	Central	Medical Doctors
5	Cardiothoracic Surgeon	4	5	6	0.01	857,945	Central African Rep	Minimum Estimate	2212 - Specialist medical practitioners	Low Income	Central	Medical Doctors

S/N	Health Professionals	2022	2026	2030	Density per 10,000 population	Required Population ratio (1 professional is to xxx population)	Name of Country	Modelling Scenario	ISCO-08 Match	Income Group Classification	Sub-Regional Grouping	SDG 3c Occupation
6	Clinical Officer/Physician Assistant	624	677	754	1.56	6,416	Central African Rep	Minimum Estimate	3256 - Medical assistants	Low Income	Central	Other Health Workers
7	Clinical Pharmacist	101	113	131	0.27	36,875	Central African Rep	Minimum Estimate	2262 - Pharmacists	Low Income	Central	Pharmacist
8	Clinical Psychologist	261	293	337	0.69	14,389	Central African Rep	Minimum Estimate	2634 - Psychologists	Low Income	Central	Other Health Workers
9	Community health worker/Village health worker	3,106	3,417	3,797	7.80	1,282	Central African Rep	Minimum Estimate	3253 - Community health workers	Low Income	Central	Other Health Workers
10	Dental Surgery Assistant	291	330	385	0.79	12,618	Central African Rep	Minimum Estimate	3251 - Dental assistants and therapists	Low Income	Central	Other Health Workers
11	Dental Therapist	181	205	239	0.49	20,361	Central African Rep	Minimum Estimate	3251 - Dental assistants and therapists	Low Income	Central	Other Health Workers
12	Dentist	239	272	324	0.67	14,979	Central African Rep	Minimum Estimate	2261 - Dentists	Low Income	Central	Dentist
13	Dermatologist	14	16	18	0.04	268,973	Central African Rep	Minimum Estimate	2212 - Specialist medical practitioners	Low Income	Central	Medical Doctors
14	Endocrinologist	16	20	25	0.05	192,191	Central African Rep	Minimum Estimate	2212 - Specialist medical practitioners	Low Income	Central	Medical Doctors
15	ENT Surgeon	32	37	42	0.09	115,319	Central African Rep	Minimum Estimate	2212 - Specialist medical practitioners	Low Income	Central	Medical Doctors
16	Environmental Health Officer	120	135	152	0.31	32,032	Central African Rep	Minimum Estimate	2263 - Environmental and occupational health and hygiene professionals	Low Income	Central	Other Health Workers
17	Gastroenterologist	53	58	66	0.14	73,396	Central African Rep	Minimum Estimate	2212 - Specialist medical practitioners	Low Income	Central	Medical Doctors
18	General Medical Practitioner (Generalist Doctor)	1,482	1,654	1,874	3.85	2,594	Central African Rep	Minimum Estimate	2211 - Generalist medical practitioners	Low Income	Central	Medical Doctors
19	General Surgeon	33	37	44	0.09	111,028	Central African Rep	Minimum Estimate	2212 - Specialist medical practitioners	Low Income	Central	Medical Doctors
20	Haematologist	13	15	17	0.04	283,538	Central African Rep	Minimum Estimate	2212 - Specialist medical practitioners	Low Income	Central	Medical Doctors
21	Health Promoter/Health Educator	33	36	40	0.08	121,648	Central African Rep	Minimum Estimate	2269 - Health professionals not elsewhere classified	Low Income	Central	Other Health Workers
22	Infectious Diseases Specialist	6	6	7	0.01	701,945	Central African Rep	Minimum Estimate	2212 - Specialist medical practitioners	Low Income	Central	Medical Doctors
23	Intensive Care Nurse	132	147	164	0.34	29,706	Central African Rep	Minimum Estimate	2221 - Nursing professionals	Low Income	Central	Nursing Personnel
24	Medical Laboratory Scientist	475	534	618	1.28	7,834	Central African Rep	Minimum Estimate	3212 - Medical and pathology laboratory technicians	Low Income	Central	Other Health Workers
25	Medical Laboratory Technician	523	564	617	1.27	7,855	Central African Rep	Minimum Estimate	3212 - Medical and pathology laboratory technicians	Low Income	Central	Other Health Workers
26	Medical Social Worker	163	177	198	0.41	24,550	Central African Rep	Minimum Estimate	1344 - Social welfare managers	Low Income	Central	Other Health Workers
27	Mental Health Nurse	193	219	263	0.54	18,382	Central African Rep	Minimum Estimate	2221 - Nursing professionals	Low Income	Central	Nursing Personnel
28	Midwife	2,874	3,204	3,659	7.52	1,330	Central African Rep	Minimum Estimate	2222 - Midwifery professionals	Low Income	Central	Midwifery Personnel
29	Nephrologist	26	30	37	0.08	130,672	Central African Rep	Minimum Estimate	2212 - Specialist medical practitioners	Low Income	Central	Medical Doctors
30	Neuro-Surgeon	12	14	15	0.03	315,954	Central African Rep	Minimum Estimate	2212 - Specialist medical practitioners	Low Income	Central	Medical Doctors
31	Nurse Anaesthetist	50	57	65	0.13	74,426	Central African Rep	Minimum Estimate	2221 - Nursing professionals	Low Income	Central	Nursing Personnel
32	Nutritionist	492	536	587	1.20	8,301	Central African Rep	Minimum Estimate	2265 - Dieticians and nutritionists	Low Income	Central	Other Health Workers
33	Obstetrician & Gynaecologist	265	300	344	0.71	14,130	Central African Rep	Minimum Estimate	2212 - Specialist medical practitioners	Low Income	Central	Medical Doctors
34	Occupational Therapist	58	66	77	0.16	62,800	Central African Rep	Minimum Estimate	2269 - Health professionals not elsewhere classified	Low Income	Central	Other Health Workers
35	Oncology Nurse	19	22	27	0.06	177,333	Central African Rep	Minimum Estimate	2221 - Nursing professionals	Low Income	Central	Nursing Personnel
36	Operating Theatre Nurse	365	418	502	1.04	9,610	Central African Rep	Minimum Estimate	2221 - Nursing professionals	Low Income	Central	Nursing Personnel
37	Ophthalmic Nurse	42	49	58	0.12	82,651	Central African Rep	Minimum Estimate	2221 - Nursing professionals	Low Income	Central	Nursing Personnel
38	Ophthalmologist	11	13	17	0.03	291,477	Central African Rep	Minimum Estimate	2212 - Specialist medical practitioners	Low Income	Central	Medical Doctors
39	Optometrist	42	50	60	0.12	81,284	Central African Rep	Minimum Estimate	2267 - Optometrists and ophthalmic opticians	Low Income	Central	Other Health Workers
40	Orthopaedic Nurse	24	27	31	0.06	157,631	Central African Rep	Minimum Estimate	2221 - Nursing professionals	Low Income	Central	Nursing Personnel
41	Orthopaedic Surgeon	80	89	101	0.21	48,234	Central African Rep	Minimum Estimate	2212 - Specialist medical practitioners	Low Income	Central	Medical Doctors
42	Orthopaedic Technologist	111	125	142	0.29	34,418	Central African Rep	Minimum Estimate	3214 - Medical and dental prosthetic technicians	Low Income	Central	Other Health Workers
43	Paediatric Nurse	406	455	514	1.06	9,477	Central African Rep	Minimum Estimate	2221 - Nursing professionals	Low Income	Central	Nursing Personnel
44	Paediatric Surgeon	14	16	17	0.04	280,229	Central African Rep	Minimum Estimate	2212 - Specialist medical practitioners	Low Income	Central	Medical Doctors
45	Paediatrician	144	161	183	0.38	26,564	Central African Rep	Minimum Estimate	2212 - Specialist medical practitioners	Low Income	Central	Medical Doctors
46	Pathologist	32	36	44	0.09	109,522	Central African Rep	Minimum Estimate	2212 - Specialist medical practitioners	Low Income	Central	Medical Doctors
47	Pharmacist	474	520	578	1.19	8,414	Central African Rep	Minimum Estimate	2262 - Pharmacists	Low Income	Central	Pharmacist
48	Pharmacy Technician	408	456	517	1.06	9,402	Central African Rep	Minimum Estimate	3213 - Pharmaceutical technicians and assistants	Low Income	Central	Other Health Workers
49	Physician	352	397	458	0.94	10,587	Central African Rep	Minimum Estimate	2212 - Specialist medical practitioners	Low Income	Central	Medical Doctors
50	Physiotherapist	82	93	106	0.22	45,852	Central African Rep	Minimum Estimate	2264 - Physiotherapists	Low Income	Central	Other Health Workers
51	Plastic Surgeon	17	20	22	0.05	218,017	Central African Rep	Minimum Estimate	2212 - Specialist medical practitioners	Low Income	Central	Medical Doctors
52	Psychiatrist	186	209	245	0.51	19,754	Central African Rep	Minimum Estimate	2212 - Specialist medical practitioners	Low Income	Central	Medical Doctors
53	Radiation Oncologist	5	5	6	0.01	805,249	Central African Rep	Minimum Estimate	2212 - Specialist medical practitioners	Low Income	Central	Medical Doctors
54	Radiographer (Diagnostics and Therapy)	244	278	329	0.68	14,690	Central African Rep	Minimum Estimate	3211 - Medical imaging and therapeutic equipment technicians	Low Income	Central	Other Health Workers
55	Radiologist	79	89	104	0.21	46,626	Central African Rep	Minimum Estimate	2212 - Specialist medical practitioners	Low Income	Central	Medical Doctors
56	Registered General Nurse / State Certified Nurse	9,989	11,224	12,851	26.46	378	Central African Rep	Minimum Estimate	2221 - Nursing professionals	Low Income	Central	Nursing Personnel
57	Renal Nurse	320	373	460	0.96	10,433	Central African Rep	Minimum Estimate	2221 - Nursing professionals	Low Income	Central	Nursing Personnel
58	Respiratory Physician	25	28	31	0.06	156,607	Central African Rep	Minimum Estimate	2212 - Specialist medical practitioners	Low Income	Central	Medical Doctors
59	Rheumatologist	10	11	13	0.03	377,000	Central African Rep	Minimum Estimate	2212 - Specialist medical practitioners	Low Income	Central	Medical Doctors
60	Speech Therapist	34	39	44	0.09	111,166	Central African Rep	Minimum Estimate	2266 - Audiologists and speech therapists	Low Income	Central	Other Health Workers
61	Urologist	3	3	4	0.01	1,210,253	Central African Rep	Minimum Estimate	2212 - Specialist medical practitioners	Low Income	Central	Medical Doctors
1	Anaesthesiologist	96	108	121	0.07	136,600	Chad	Minimum Estimate	2212 - Specialist medical practitioners	Low Income	West	Medical Doctors
2	Associate Nurse/Enrolled Nurse/Nursing Assistant	14,887	16,638	18,569	11.21	892	Chad	Minimum Estimate	3221 - Nursing associate professionals	Low Income	West	Nursing Personnel
3	Audiologist	38	43	48	0.03	345,645	Chad	Minimum Estimate	2266 - Audiologists and speech therapists	Low Income	West	Other Health Workers
4	Cardiologist	35	39	44	0.03	379,422	Chad	Minimum Estimate	2212 - Specialist medical practitioners	Low Income	West	Medical Doctors
5	Cardiothoracic Surgeon	17	19	21	0.01	778,872	Chad	Minimum Estimate	2212 - Specialist medical practitioners	Low Income	West	Medical Doctors
6	Clinical Officer/Physician Assistant	1,228	1,270	1,327	0.81	12,394	Chad	Minimum Estimate	3256 - Medical assistants	Low Income	West	Other Health Workers
7	Clinical Pharmacist	315	351	395	0.24	41,796	Chad	Minimum Estimate	2262 - Pharmacists	Low Income	West	Pharmacist
8	Clinical Psychologist	760	850	959	0.58	17,229	Chad	Minimum Estimate	2634 - Psychologists	Low Income	West	Other Health Workers
9	Community health worker/Village health worker	11,704	12,830	13,997	8.45	1,183	Chad	Minimum Estimate	3253 - Community health workers	Low Income	West	Other Health Workers
10	Dental Surgery Assistant	1,008	1,140	1,293	0.78	12,805	Chad	Minimum Estimate	3251 - Dental assistants and therapists	Low Income	West	Other Health Workers
11	Dental Therapist	632	714	810	0.49	20,459	Chad	Minimum Estimate	3251 - Dental assistants and therapists	Low Income	West	Other Health Workers
12	Dentist	709	802	925	0.56	17,865	Chad	Minimum Estimate	2261 - Dentists	Low Income	West	Dentist
13	Dermatologist	41	47	52	0.03	316,101	Chad	Minimum Estimate	2212 - Specialist medical practitioners	Low Income	West	Medical Doctors
14	Endocrinologist	27	31	37	0.02	447,089	Chad	Minimum Estimate	2212 - Specialist medical practitioners	Low Income	West	Medical Doctors
15	ENT Surgeon	103	116	131	0.08	126,795	Chad	Minimum Estimate	2212 - Specialist medical practitioners	Low Income	West	Medical Doctors
16	Environmental Health Officer	408	460	516	0.31	32,142	Chad	Minimum Estimate	2263 - Environmental and occupational health and hygiene professionals	Low Income	West	Other Health Workers
17	Gastroenterologist	176	188	206	0.13	79,776	Chad	Minimum Estimate	2212 - Specialist medical practitioners	Low Income	West	Medical Doctors
18	General Medical Practitioner (Generalist Doctor)	4,368	4,859	5,401	3.26	3,065	Chad	Minimum Estimate	2211 - Generalist medical practitioners	Low Income	West	Medical Doctors
19	General Surgeon	89	100	114	0.07	144,222	Chad	Minimum Estimate	2212 - Specialist medical practitioners	Low Income	West	Medical Doctors
20	Haematologist	49	56	63	0.04	261,771	Chad	Minimum Estimate	2212 - Specialist medical practitioners	Low Income	West	Medical Doctors
21	Health Promoter/Health Educator	81	84	88	0.05	186,630	Chad	Minimum Estimate	2269 - Health professionals not elsewhere classified	Low Income	West	Other Health Workers
22	Infectious Diseases Specialist	14	16	17	0.01	956,738	Chad	Minimum Estimate	2212 - Specialist medical practitioners	Low Income	West	Medical Doctors
23	Intensive Care Nurse	465	519	575	0.35	28,826	Chad	Minimum Estimate	2221 - Nursing professionals	Low Income	West	Nursing Personnel
24	Medical Laboratory Scientist	1,177	1,306	1,465	0.89	11,271	Chad	Minimum Estimate	3212 - Medical and pathology laboratory technicians	Low Income	West	Other Health Workers
25	Medical Laboratory Technician	1,232	1,328	1,434	0.87	11,523	Chad	Minimum Estimate	3212 - Medical and pathology laboratory technicians	Low Income	West	Other Health Workers
26	Medical Social Worker	809	881	962	0.58	17,196	Chad	Minimum Estimate	1344 - Social welfare managers	Low Income	West	Other Health Workers
27	Mental Health Nurse	560	633	730	0.44	22,568	Chad	Minimum Estimate	2221 - Nursing professionals	Low Income	West	Nursing Personnel
28	Midwife	9,419	10,490	11,733	7.09	1,411	Chad	Minimum Estimate	2222 - Midwifery professionals	Low Income	West	Midwifery Personnel

S/N	Health Professionals	2022	2026	2030	Density per 10,000 population	Required Population ratio (1 professional is to xxx population)	Name of Country	Modelling Scenario	ISCO-08 Match	Income Group Classification	Sub-Regional Grouping	SDG 3c Occupation
29	Nephrologist	76	87	103	0.06	159,603	Chad	Minimum Estimate	2212 - Specialist medical practitioners	Low Income	West	Medical Doctors
30	Neuro-Surgeon	30	34	39	0.02	428,080	Chad	Minimum Estimate	2212 - Specialist medical practitioners	Low Income	West	Medical Doctors
31	Nurse Anaesthetist	160	181	205	0.12	80,946	Chad	Minimum Estimate	2221 - Nursing professionals	Low Income	West	Nursing Personnel
32	Nutritionist	3,375	3,717	4,066	2.45	4,079	Chad	Minimum Estimate	2265 - Dieticians and nutritionists	Low Income	West	Other Health Workers
33	Obstetrician & Gynaecologist	774	875	987	0.60	16,790	Chad	Minimum Estimate	2212 - Specialist medical practitioners	Low Income	West	Medical Doctors
34	Occupational Therapist	204	230	260	0.16	63,515	Chad	Minimum Estimate	2269 - Health professionals not elsewhere classified	Low Income	West	Other Health Workers
35	Oncology Nurse	57	65	75	0.05	218,603	Chad	Minimum Estimate	2221 - Nursing professionals	Low Income	West	Nursing Personnel
36	Operating Theatre Nurse	1,043	1,165	1,331	0.81	12,385	Chad	Minimum Estimate	2221 - Nursing professionals	Low Income	West	Nursing Personnel
37	Ophthalmic Nurse	106	119	134	0.08	123,825	Chad	Minimum Estimate	2221 - Nursing professionals	Low Income	West	Nursing Personnel
38	Ophthalmologist	28	31	35	0.02	465,400	Chad	Minimum Estimate	2212 - Specialist medical practitioners	Low Income	West	Medical Doctors
39	Optometrist	108	119	132	0.08	125,706	Chad	Minimum Estimate	2267 - Optometrists and ophthalmic opticians	Low Income	West	Other Health Workers
40	Orthopaedic Nurse	110	125	141	0.09	117,200	Chad	Minimum Estimate	2221 - Nursing professionals	Low Income	West	Nursing Personnel
41	Orthopaedic Surgeon	271	314	365	0.22	45,274	Chad	Minimum Estimate	2212 - Specialist medical practitioners	Low Income	West	Medical Doctors
42	Orthopaedic Technologist	401	453	509	0.31	32,578	Chad	Minimum Estimate	3214 - Medical and dental prosthetic technicians	Low Income	West	Other Health Workers
43	Paediatric Nurse	1,866	2,092	2,320	1.40	7,155	Chad	Minimum Estimate	2221 - Nursing professionals	Low Income	West	Nursing Personnel
44	Paediatric Surgeon	53	61	68	0.04	242,956	Chad	Minimum Estimate	2212 - Specialist medical practitioners	Low Income	West	Medical Doctors
45	Paediatrician	415	476	545	0.33	30,328	Chad	Minimum Estimate	2212 - Specialist medical practitioners	Low Income	West	Medical Doctors
46	Pathologist	67	74	85	0.05	194,349	Chad	Minimum Estimate	2212 - Specialist medical practitioners	Low Income	West	Medical Doctors
47	Pharmacist	799	825	854	0.52	19,285	Chad	Minimum Estimate	2262 - Pharmacists	Low Income	West	Pharmacist
48	Pharmacy Technician	1,172	1,300	1,445	0.87	11,450	Chad	Minimum Estimate	3213 - Pharmaceutical technicians and assistants	Low Income	West	Other Health Workers
49	Physician	1,172	1,312	1,474	0.89	11,223	Chad	Minimum Estimate	2212 - Specialist medical practitioners	Low Income	West	Medical Doctors
50	Physiotherapist	283	319	359	0.22	46,121	Chad	Minimum Estimate	2264 - Physiotherapists	Low Income	West	Other Health Workers
51	Plastic Surgeon	66	75	85	0.05	196,152	Chad	Minimum Estimate	2212 - Specialist medical practitioners	Low Income	West	Medical Doctors
52	Psychiatrist	690	772	874	0.53	18,892	Chad	Minimum Estimate	2212 - Specialist medical practitioners	Low Income	West	Medical Doctors
53	Radiation Oncologist	14	15	18	0.01	937,148	Chad	Minimum Estimate	2212 - Specialist medical practitioners	Low Income	West	Medical Doctors
54	Radiographer (Diagnostics and Therapy)	737	831	951	0.58	17,355	Chad	Minimum Estimate	3211 - Medical imaging and therapeutic equipment technicians	Low Income	West	Other Health Workers
55	Radiologist	242	272	308	0.19	53,697	Chad	Minimum Estimate	2212 - Specialist medical practitioners	Low Income	West	Medical Doctors
56	Registered General Nurse / State Certified Nurse	39,760	42,192	44,947	27.25	367	Chad	Minimum Estimate	2221 - Nursing professionals	Low Income	West	Nursing Personnel
57	Renal Nurse	937	1,070	1,261	0.77	13,023	Chad	Minimum Estimate	2221 - Nursing professionals	Low Income	West	Nursing Personnel
58	Respiratory Physician	62	72	84	0.05	196,938	Chad	Minimum Estimate	2212 - Specialist medical practitioners	Low Income	West	Medical Doctors
59	Rheumatologist	31	35	40	0.02	418,417	Chad	Minimum Estimate	2212 - Specialist medical practitioners	Low Income	West	Medical Doctors
60	Speech Therapist	117	132	149	0.09	111,367	Chad	Minimum Estimate	2266 - Audiologists and speech therapists	Low Income	West	Other Health Workers
61	Urologist	9	10	12	0.01	1,361,058	Chad	Minimum Estimate	2212 - Specialist medical practitioners	Low Income	West	Medical Doctors
1	Anaesthesiologist	11	13	14	0.16	60,828	Comoros	Minimum Estimate	2212 - Specialist medical practitioners	Lower-middle Income	Southern	Medical Doctors
2	Associate Nurse/Enrolled Nurse/Nursing Assistant	824	890	962	11.00	909	Comoros	Minimum Estimate	3221 - Nursing associate professionals	Lower-middle Income	Southern	Nursing Personnel
3	Audiologist	3	3	3	0.04	284,855	Comoros	Minimum Estimate	2266 - Audiologists and speech therapists	Lower-middle Income	Southern	Other Health Workers
4	Cardiologist	4	5	6	0.06	156,513	Comoros	Minimum Estimate	2212 - Specialist medical practitioners	Lower-middle Income	Southern	Medical Doctors
5	Cardiothoracic Surgeon	2	2	2	0.03	356,565	Comoros	Minimum Estimate	2212 - Specialist medical practitioners	Lower-middle Income	Southern	Medical Doctors
6	Clinical Officer/Physician Assistant	75	81	87	1.00	9,984	Comoros	Minimum Estimate	3256 - Medical assistants	Lower-middle Income	Southern	Other Health Workers
7	Clinical Pharmacist	18	21	23	0.26	37,790	Comoros	Minimum Estimate	2262 - Pharmacists	Lower-middle Income	Southern	Pharmacist
8	Clinical Psychologist	37	41	45	0.52	19,317	Comoros	Minimum Estimate	2634 - Psychologists	Lower-middle Income	Southern	Other Health Workers
9	Community health worker/Village health worker	429	452	473	5.40	1,852	Comoros	Minimum Estimate	3253 - Community health workers	Lower-middle Income	Southern	Other Health Workers
10	Dental Surgery Assistant	68	76	84	0.96	10,418	Comoros	Minimum Estimate	3251 - Dental assistants and therapists	Lower-middle Income	Southern	Other Health Workers
11	Dental Therapist	43	47	53	0.60	16,663	Comoros	Minimum Estimate	3251 - Dental assistants and therapists	Lower-middle Income	Southern	Other Health Workers
12	Dentist	52	57	63	0.72	13,820	Comoros	Minimum Estimate	2261 - Dentists	Lower-middle Income	Southern	Dentist
13	Dermatologist	2	3	3	0.03	297,071	Comoros	Minimum Estimate	2212 - Specialist medical practitioners	Lower-middle Income	Southern	Medical Doctors
14	Endocrinologist	3	3	4	0.04	223,367	Comoros	Minimum Estimate	2212 - Specialist medical practitioners	Lower-middle Income	Southern	Medical Doctors
15	ENT Surgeon	8	9	11	0.12	82,753	Comoros	Minimum Estimate	2212 - Specialist medical practitioners	Lower-middle Income	Southern	Medical Doctors
16	Environmental Health Officer	22	23	25	0.28	35,422	Comoros	Minimum Estimate	2263 - Environmental and occupational health and hygiene professionals	Lower-middle Income	Southern	Other Health Workers
17	Gastroenterologist	5	5	5	0.06	162,065	Comoros	Minimum Estimate	2212 - Specialist medical practitioners	Lower-middle Income	Southern	Medical Doctors
18	General Medical Practitioner (Generalist Doctor)	236	256	278	3.17	3,152	Comoros	Minimum Estimate	2211 - Generalist medical practitioners	Lower-middle Income	Southern	Medical Doctors
19	General Surgeon	6	7	8	0.10	103,054	Comoros	Minimum Estimate	2212 - Specialist medical practitioners	Lower-middle Income	Southern	Medical Doctors
20	Haematologist	3	3	3	0.04	255,354	Comoros	Minimum Estimate	2212 - Specialist medical practitioners	Lower-middle Income	Southern	Medical Doctors
21	Health Promoter/Health Educator	3	4	4	0.05	221,387	Comoros	Minimum Estimate	2269 - Health professionals not elsewhere classified	Lower-middle Income	Southern	Other Health Workers
22	Infectious Diseases Specialist	0	1	1	0.01	1,535,697	Comoros	Minimum Estimate	2212 - Specialist medical practitioners	Lower-middle Income	Southern	Medical Doctors
23	Intensive Care Nurse	22	24	26	0.30	33,191	Comoros	Minimum Estimate	2221 - Nursing professionals	Lower-middle Income	Southern	Nursing Personnel
24	Medical Laboratory Scientist	74	82	91	1.04	9,635	Comoros	Minimum Estimate	3212 - Medical and pathology laboratory technicians	Lower-middle Income	Southern	Other Health Workers
25	Medical Laboratory Technician	56	61	67	0.77	13,027	Comoros	Minimum Estimate	3212 - Medical and pathology laboratory technicians	Lower-middle Income	Southern	Other Health Workers
26	Medical Social Worker	21	21	22	0.25	39,976	Comoros	Minimum Estimate	1344 - Social welfare managers	Lower-middle Income	Southern	Other Health Workers
27	Mental Health Nurse	36	40	44	0.51	19,626	Comoros	Minimum Estimate	2221 - Nursing professionals	Lower-middle Income	Southern	Nursing Personnel
28	Midwife	454	486	520	5.94	1,684	Comoros	Minimum Estimate	2222 - Midwifery professionals	Lower-middle Income	Southern	Midwifery Personnel
29	Nephrologist	8	9	11	0.12	82,355	Comoros	Minimum Estimate	2212 - Specialist medical practitioners	Lower-middle Income	Southern	Medical Doctors
30	Neuro-Surgeon	5	5	6	0.07	138,983	Comoros	Minimum Estimate	2212 - Specialist medical practitioners	Lower-middle Income	Southern	Medical Doctors
31	Nurse Anaesthetist	21	25	30	0.34	29,082	Comoros	Minimum Estimate	2221 - Nursing professionals	Lower-middle Income	Southern	Nursing Personnel
32	Nutritionist	76	77	77	0.88	11,356	Comoros	Minimum Estimate	2265 - Dieticians and nutritionists	Lower-middle Income	Southern	Other Health Workers
33	Obstetrician & Gynaecologist	48	53	58	0.66	15,217	Comoros	Minimum Estimate	2212 - Specialist medical practitioners	Lower-middle Income	Southern	Medical Doctors
34	Occupational Therapist	29	34	39	0.45	22,165	Comoros	Minimum Estimate	2269 - Health professionals not elsewhere classified	Lower-middle Income	Southern	Other Health Workers
35	Oncology Nurse	7	8	9	0.10	96,230	Comoros	Minimum Estimate	2221 - Nursing professionals	Lower-middle Income	Southern	Nursing Personnel
36	Operating Theatre Nurse	104	118	135	1.55	6,438	Comoros	Minimum Estimate	2221 - Nursing professionals	Lower-middle Income	Southern	Nursing Personnel
37	Ophthalmic Nurse	8	9	11	0.12	82,801	Comoros	Minimum Estimate	2221 - Nursing professionals	Lower-middle Income	Southern	Nursing Personnel
38	Ophthalmologist	3	3	4	0.04	228,044	Comoros	Minimum Estimate	2212 - Specialist medical practitioners	Lower-middle Income	Southern	Medical Doctors
39	Optometrist	12	14	16	0.19	53,761	Comoros	Minimum Estimate	2267 - Optometrists and ophthalmic opticians	Lower-middle Income	Southern	Other Health Workers
40	Orthopaedic Nurse	19	22	26	0.29	33,954	Comoros	Minimum Estimate	2221 - Nursing professionals	Lower-middle Income	Southern	Nursing Personnel
41	Orthopaedic Surgeon	30	35	40	0.46	21,778	Comoros	Minimum Estimate	2212 - Specialist medical practitioners	Lower-middle Income	Southern	Medical Doctors
42	Orthopaedic Technologist	32	36	40	0.45	22,011	Comoros	Minimum Estimate	3214 - Medical and dental prosthetic technicians	Lower-middle Income	Southern	Other Health Workers
43	Paediatric Nurse	73	77	81	0.92	10,846	Comoros	Minimum Estimate	2221 - Nursing professionals	Lower-middle Income	Southern	Nursing Personnel
44	Paediatric Surgeon	2	2	2	0.02	508,791	Comoros	Minimum Estimate	2212 - Specialist medical practitioners	Lower-middle Income	Southern	Medical Doctors
45	Paediatrician	32	35	39	0.44	22,680	Comoros	Minimum Estimate	2212 - Specialist medical practitioners	Lower-middle Income	Southern	Medical Doctors
46	Pathologist	7	8	9	0.10	100,710	Comoros	Minimum Estimate	2212 - Specialist medical practitioners	Lower-middle Income	Southern	Medical Doctors
47	Pharmacist	54	57	59	0.68	14,705	Comoros	Minimum Estimate	2262 - Pharmacists	Lower-middle Income	Southern	Pharmacist
48	Pharmacy Technician	74	82	90	1.03	9,676	Comoros	Minimum Estimate	3213 - Pharmaceutical technicians and assistants	Lower-middle Income	Southern	Other Health Workers
49	Physician	61	67	74	0.85	11,779	Comoros	Minimum Estimate	2212 - Specialist medical practitioners	Lower-middle Income	Southern	Medical Doctors
50	Physiotherapist	22	25	28	0.32	31,277	Comoros	Minimum Estimate	2264 - Physiotherapists	Lower-middle Income	Southern	Other Health Workers
51	Plastic Surgeon	13	14	16	0.18	55,175	Comoros	Minimum Estimate	2212 - Specialist medical practitioners	Lower-middle Income	Southern	Medical Doctors

S/N	Health Professionals	2022	2026	2030	Density per 10,000 population	Required Population ratio (1 professional is to xxx population)	Name of Country	Modelling Scenario	ISCO-08 Match	Income Group Classification	Sub-Regional Grouping	SDG 3c Occupation
52	Psychiatrist	34	37	40	0.46	21,625	Comoros	Minimum Estimate	2212 - Specialist medical practitioners	Lower-middle Income	Southern	Medical Doctors
53	Radiation Oncologist	2	2	3	0.03	288,058	Comoros	Minimum Estimate	2212 - Specialist medical practitioners	Lower-middle Income	Southern	Medical Doctors
54	Radiographer (Diagnostics and Therapy)	51	57	65	0.74	13,434	Comoros	Minimum Estimate	3211 - Medical imaging and therapeutic equipment technicians	Lower-middle Income	Southern	Other Health Workers
55	Radiologist	16	18	21	0.24	41,562	Comoros	Minimum Estimate	2212 - Specialist medical practitioners	Lower-middle Income	Southern	Medical Doctors
56	Registered General Nurse / State Certified Nurse	1,808	1,995	2,214	25.35	395	Comoros	Minimum Estimate	2221 - Nursing professionals	Lower-middle Income	Southern	Nursing Personnel
57	Renal Nurse	101	115	133	1.53	6,556	Comoros	Minimum Estimate	2221 - Nursing professionals	Lower-middle Income	Southern	Nursing Personnel
58	Respiratory Physician	6	7	7	0.08	122,818	Comoros	Minimum Estimate	2212 - Specialist medical practitioners	Lower-middle Income	Southern	Medical Doctors
59	Rheumatologist	2	3	3	0.03	315,287	Comoros	Minimum Estimate	2212 - Specialist medical practitioners	Lower-middle Income	Southern	Medical Doctors
60	Speech Therapist	7	8	8	0.10	103,873	Comoros	Minimum Estimate	2266 - Audiologists and speech therapists	Lower-middle Income	Southern	Other Health Workers
61	Urologist	1	1	1	0.02	603,019	Comoros	Minimum Estimate	2212 - Specialist medical practitioners	Lower-middle Income	Southern	Medical Doctors
1	Anaesthesiologist	37	41	47	0.08	118,936	Congo	Minimum Estimate	2212 - Specialist medical practitioners	Lower-middle Income	Central	Medical Doctors
2	Associate Nurse/Enrolled Nurse/Nursing Assistant	5,641	6,209	6,843	12.33	811	Congo	Minimum Estimate	3221 - Nursing associate professionals	Lower-middle Income	Central	Nursing Personnel
3	Audiologist	13	15	16	0.03	344,466	Congo	Minimum Estimate	2266 - Audiologists and speech therapists	Lower-middle Income	Central	Other Health Workers
4	Cardiologist	20	23	26	0.05	209,767	Congo	Minimum Estimate	2212 - Specialist medical practitioners	Lower-middle Income	Central	Medical Doctors
5	Cardiothoracic Surgeon	5	6	6	0.01	859,242	Congo	Minimum Estimate	2212 - Specialist medical practitioners	Lower-middle Income	Central	Medical Doctors
6	Clinical Officer/Physician Assistant	617	685	770	1.39	7,169	Congo	Minimum Estimate	3256 - Medical assistants	Lower-middle Income	Central	Other Health Workers
7	Clinical Pharmacist	119	132	146	0.26	37,792	Congo	Minimum Estimate	2262 - Pharmacists	Lower-middle Income	Central	Pharmacist
8	Clinical Psychologist	325	355	389	0.70	14,260	Congo	Minimum Estimate	2634 - Psychologists	Lower-middle Income	Central	Other Health Workers
9	Community health worker/Village health worker	3,117	3,445	3,814	6.89	1,451	Congo	Minimum Estimate	3253 - Community health workers	Lower-middle Income	Central	Other Health Workers
10	Dental Surgery Assistant	395	441	493	0.89	11,272	Congo	Minimum Estimate	3251 - Dental assistants and therapists	Lower-middle Income	Central	Other Health Workers
11	Dental Therapist	248	277	310	0.56	17,901	Congo	Minimum Estimate	3251 - Dental assistants and therapists	Lower-middle Income	Central	Other Health Workers
12	Dentist	269	297	331	0.60	16,778	Congo	Minimum Estimate	2261 - Dentists	Lower-middle Income	Central	Dentist
13	Dermatologist	16	17	19	0.03	292,574	Congo	Minimum Estimate	2212 - Specialist medical practitioners	Lower-middle Income	Central	Medical Doctors
14	Endocrinologist	25	30	36	0.07	150,805	Congo	Minimum Estimate	2212 - Specialist medical practitioners	Lower-middle Income	Central	Medical Doctors
15	ENT Surgeon	42	48	55	0.10	101,219	Congo	Minimum Estimate	2212 - Specialist medical practitioners	Lower-middle Income	Central	Medical Doctors
16	Environmental Health Officer	137	150	163	0.29	34,079	Congo	Minimum Estimate	2263 - Environmental and occupational health and hygiene professionals	Lower-middle Income	Central	Other Health Workers
17	Gastroenterologist	45	46	48	0.09	115,124	Congo	Minimum Estimate	2212 - Specialist medical practitioners	Lower-middle Income	Central	Medical Doctors
18	General Medical Practitioner (Generalist Doctor)	1,656	1,823	2,011	3.63	2,756	Congo	Minimum Estimate	2211 - Generalist medical practitioners	Lower-middle Income	Central	Medical Doctors
19	General Surgeon	42	49	57	0.10	96,318	Congo	Minimum Estimate	2212 - Specialist medical practitioners	Lower-middle Income	Central	Medical Doctors
20	Haematologist	14	15	16	0.03	338,326	Congo	Minimum Estimate	2212 - Specialist medical practitioners	Lower-middle Income	Central	Medical Doctors
21	Health Promoter/Health Educator	29	32	35	0.06	157,541	Congo	Minimum Estimate	2269 - Health professionals not elsewhere classified	Lower-middle Income	Central	Other Health Workers
22	Infectious Diseases Specialist	5	5	6	0.01	951,358	Congo	Minimum Estimate	2212 - Specialist medical practitioners	Lower-middle Income	Central	Medical Doctors
23	Intensive Care Nurse	125	136	148	0.27	37,463	Congo	Minimum Estimate	2221 - Nursing professionals	Lower-middle Income	Central	Nursing Personnel
24	Medical Laboratory Scientist	555	619	697	1.26	7,924	Congo	Minimum Estimate	3212 - Medical and pathology laboratory technicians	Lower-middle Income	Central	Other Health Workers
25	Medical Laboratory Technician	559	646	752	1.36	7,343	Congo	Minimum Estimate	3212 - Medical and pathology laboratory technicians	Lower-middle Income	Central	Other Health Workers
26	Medical Social Worker	131	136	142	0.26	38,908	Congo	Minimum Estimate	1344 - Social welfare managers	Lower-middle Income	Central	Other Health Workers
27	Mental Health Nurse	215	236	263	0.48	21,011	Congo	Minimum Estimate	2221 - Nursing professionals	Lower-middle Income	Central	Nursing Personnel
28	Midwife	2,900	3,168	3,459	6.23	1,605	Congo	Minimum Estimate	2222 - Midwifery professionals	Lower-middle Income	Central	Midwifery Personnel
29	Nephrologist	42	48	56	0.10	98,051	Congo	Minimum Estimate	2212 - Specialist medical practitioners	Lower-middle Income	Central	Medical Doctors
30	Neuro-Surgeon	17	18	21	0.04	265,547	Congo	Minimum Estimate	2212 - Specialist medical practitioners	Lower-middle Income	Central	Medical Doctors
31	Nurse Anaesthetist	91	105	121	0.22	45,959	Congo	Minimum Estimate	2221 - Nursing professionals	Lower-middle Income	Central	Nursing Personnel
32	Nutritionist	351	362	372	0.67	14,904	Congo	Minimum Estimate	2265 - Dieticians and nutritionists	Lower-middle Income	Central	Other Health Workers
33	Obstetrician & Gynaecologist	324	358	397	0.72	13,967	Congo	Minimum Estimate	2212 - Specialist medical practitioners	Lower-middle Income	Central	Medical Doctors
34	Occupational Therapist	87	99	112	0.20	49,237	Congo	Minimum Estimate	2269 - Health professionals not elsewhere classified	Lower-middle Income	Central	Other Health Workers
35	Oncology Nurse	34	39	46	0.08	120,117	Congo	Minimum Estimate	2221 - Nursing professionals	Lower-middle Income	Central	Nursing Personnel
36	Operating Theatre Nurse	532	604	698	1.26	7,909	Congo	Minimum Estimate	2221 - Nursing professionals	Lower-middle Income	Central	Nursing Personnel
37	Ophthalmic Nurse	80	92	106	0.19	52,401	Congo	Minimum Estimate	2221 - Nursing professionals	Lower-middle Income	Central	Nursing Personnel
38	Ophthalmologist	17	19	23	0.04	240,440	Congo	Minimum Estimate	2212 - Specialist medical practitioners	Lower-middle Income	Central	Medical Doctors
39	Optometrist	57	66	77	0.14	72,267	Congo	Minimum Estimate	2267 - Optometrists and ophthalmic opticians	Lower-middle Income	Central	Other Health Workers
40	Orthopaedic Nurse	45	50	57	0.10	98,240	Congo	Minimum Estimate	2221 - Nursing professionals	Lower-middle Income	Central	Nursing Personnel
41	Orthopaedic Surgeon	147	173	203	0.37	27,228	Congo	Minimum Estimate	2212 - Specialist medical practitioners	Lower-middle Income	Central	Medical Doctors
42	Orthopaedic Technologist	140	154	169	0.30	32,961	Congo	Minimum Estimate	3214 - Medical and dental prosthetic technicians	Lower-middle Income	Central	Other Health Workers
43	Paediatric Nurse	403	433	462	0.83	11,998	Congo	Minimum Estimate	2221 - Nursing professionals	Lower-middle Income	Central	Nursing Personnel
44	Paediatric Surgeon	12	13	14	0.03	399,505	Congo	Minimum Estimate	2212 - Specialist medical practitioners	Lower-middle Income	Central	Medical Doctors
45	Paediatrician	183	202	225	0.41	24,668	Congo	Minimum Estimate	2212 - Specialist medical practitioners	Lower-middle Income	Central	Medical Doctors
46	Pathologist	44	49	55	0.10	99,606	Congo	Minimum Estimate	2212 - Specialist medical practitioners	Lower-middle Income	Central	Medical Doctors
47	Pharmacist	419	443	471	0.85	11,739	Congo	Minimum Estimate	2262 - Pharmacists	Lower-middle Income	Central	Pharmacist
48	Pharmacy Technician	452	494	542	0.98	10,233	Congo	Minimum Estimate	3213 - Pharmaceutical technicians and assistants	Lower-middle Income	Central	Other Health Workers
49	Physician	418	467	526	0.95	10,530	Congo	Minimum Estimate	2212 - Specialist medical practitioners	Lower-middle Income	Central	Medical Doctors
50	Physiotherapist	105	116	130	0.23	42,721	Congo	Minimum Estimate	2264 - Physiotherapists	Lower-middle Income	Central	Other Health Workers
51	Plastic Surgeon	22	25	28	0.05	197,575	Congo	Minimum Estimate	2212 - Specialist medical practitioners	Lower-middle Income	Central	Medical Doctors
52	Psychiatrist	204	222	246	0.44	22,493	Congo	Minimum Estimate	2212 - Specialist medical practitioners	Lower-middle Income	Central	Medical Doctors
53	Radiation Oncologist	11	12	15	0.03	374,200	Congo	Minimum Estimate	2212 - Specialist medical practitioners	Lower-middle Income	Central	Medical Doctors
54	Radiographer (Diagnostics and Therapy)	327	369	421	0.76	13,134	Congo	Minimum Estimate	3211 - Medical imaging and therapeutic equipment technicians	Lower-middle Income	Central	Other Health Workers
55	Radiologist	101	115	132	0.24	41,973	Congo	Minimum Estimate	2212 - Specialist medical practitioners	Lower-middle Income	Central	Medical Doctors
56	Registered General Nurse / State Certified Nurse	9,664	10,732	11,970	21.62	462	Congo	Minimum Estimate	2221 - Nursing professionals	Lower-middle Income	Central	Nursing Personnel
57	Renal Nurse	530	607	707	1.28	7,800	Congo	Minimum Estimate	2221 - Nursing professionals	Lower-middle Income	Central	Nursing Personnel
58	Respiratory Physician	34	38	42	0.08	131,494	Congo	Minimum Estimate	2212 - Specialist medical practitioners	Lower-middle Income	Central	Medical Doctors
59	Rheumatologist	13	15	17	0.03	330,843	Congo	Minimum Estimate	2212 - Specialist medical practitioners	Lower-middle Income	Central	Medical Doctors
60	Speech Therapist	40	44	49	0.09	112,528	Congo	Minimum Estimate	2266 - Audiologists and speech therapists	Lower-middle Income	Central	Other Health Workers
61	Urologist	5	6	7	0.01	778,439	Congo	Minimum Estimate	2212 - Specialist medical practitioners	Lower-middle Income	Central	Medical Doctors
1	Anaesthesiologist	205	235	270	0.10	98,515	Côte d'Ivoire	Minimum Estimate	2212 - Specialist medical practitioners	Lower-middle Income	West	Medical Doctors
2	Associate Nurse/Enrolled Nurse/Nursing Assistant	24,526	27,207	30,232	11.38	879	Côte d'Ivoire	Minimum Estimate	3221 - Nursing associate professionals	Lower-middle Income	West	Nursing Personnel
3	Audiologist	63	70	78	0.03	340,164	Côte d'Ivoire	Minimum Estimate	2266 - Audiologists and speech therapists	Lower-middle Income	West	Other Health Workers
4	Cardiologist	79	92	106	0.04	251,692	Côte d'Ivoire	Minimum Estimate	2212 - Specialist medical practitioners	Lower-middle Income	West	Medical Doctors
5	Cardiothoracic Surgeon	33	38	43	0.02	619,534	Côte d'Ivoire	Minimum Estimate	2212 - Specialist medical practitioners	Lower-middle Income	West	Medical Doctors
6	Clinical Officer/Physician Assistant	2,875	2,920	3,001	1.14	8,779	Côte d'Ivoire	Minimum Estimate	3256 - Medical assistants	Lower-middle Income	West	Other Health Workers
7	Clinical Pharmacist	526	587	661	0.25	40,140	Côte d'Ivoire	Minimum Estimate	2262 - Pharmacists	Lower-middle Income	West	Pharmacist
8	Clinical Psychologist	1,230	1,360	1,515	0.57	17,525	Côte d'Ivoire	Minimum Estimate	2634 - Psychologists	Lower-middle Income	West	Other Health Workers
9	Community health worker/Village health worker	17,850	18,775	19,798	7.48	1,337	Côte d'Ivoire	Minimum Estimate	3253 - Community health workers	Lower-middle Income	West	Other Health Workers
10	Dental Surgery Assistant	2,024	2,275	2,559	0.96	10,387	Côte d'Ivoire	Minimum Estimate	3251 - Dental assistants and therapists	Lower-middle Income	West	Other Health Workers
11	Dental Therapist	1,276	1,435	1,615	0.61	16,459	Côte d'Ivoire	Minimum Estimate	3251 - Dental assistants and therapists	Lower-middle Income	West	Other Health Workers
12	Dentist	1,313	1,457	1,628	0.61	16,314	Côte d'Ivoire	Minimum Estimate	2261 - Dentists	Lower-middle Income	West	Dentist
13	Dermatologist	72	80	89	0.03	298,391	Côte d'Ivoire	Minimum Estimate	2212 - Specialist medical practitioners	Lower-middle Income	West	Medical Doctors

S/N	Health Professionals	2022	2026	2030	Density per 10,000 population	Required Population ratio (1 professional is to xxx population)	Name of Country	Modelling Scenario	ISCO-08 Match	Income Group Classification	Sub-Regional Grouping	SDG 3c Occupation
14	Endocrinologist	71	86	105	0.04	250,042	Côte d'Ivoire	Minimum Estimate	2212 - Specialist medical practitioners	Lower-middle Income	West	Medical Doctors
15	ENT Surgeon	197	224	255	0.10	104,264	Côte d'Ivoire	Minimum Estimate	2212 - Specialist medical practitioners	Lower-middle Income	West	Medical Doctors
16	Environmental Health Officer	655	723	795	0.30	33,479	Côte d'Ivoire	Minimum Estimate	2263 - Environmental and occupational health and hygiene professionals	Lower-middle Income	West	Other Health Workers
17	Gastroenterologist	193	191	195	0.07	135,230	Côte d'Ivoire	Minimum Estimate	2212 - Specialist medical practitioners	Lower-middle Income	West	Medical Doctors
18	General Medical Practitioner (Generalist Doctor)	7,580	8,293	9,094	3.43	2,918	Côte d'Ivoire	Minimum Estimate	2211 - Generalist medical practitioners	Lower-middle Income	West	Medical Doctors
19	General Surgeon	184	211	244	0.09	108,408	Côte d'Ivoire	Minimum Estimate	2212 - Specialist medical practitioners	Lower-middle Income	West	Medical Doctors
20	Haematologist	83	96	110	0.04	241,482	Côte d'Ivoire	Minimum Estimate	2212 - Specialist medical practitioners	Lower-middle Income	West	Medical Doctors
21	Health Promoter/Health Educator	128	130	134	0.05	196,478	Côte d'Ivoire	Minimum Estimate	2269 - Health professionals not elsewhere classified	Lower-middle Income	West	Other Health Workers
22	Infectious Diseases Specialist	23	25	27	0.01	982,570	Côte d'Ivoire	Minimum Estimate	2212 - Specialist medical practitioners	Lower-middle Income	West	Medical Doctors
23	Intensive Care Nurse	601	664	731	0.28	36,347	Côte d'Ivoire	Minimum Estimate	2221 - Nursing professionals	Lower-middle Income	West	Nursing Personnel
24	Medical Laboratory Scientist	2,285	2,480	2,732	1.03	9,680	Côte d'Ivoire	Minimum Estimate	3212 - Medical and pathology laboratory technicians	Lower-middle Income	West	Other Health Workers
25	Medical Laboratory Technician	2,934	2,983	3,067	1.17	8,583	Côte d'Ivoire	Minimum Estimate	3212 - Medical and pathology laboratory technicians	Lower-middle Income	West	Other Health Workers
26	Medical Social Worker	753	783	819	0.31	32,354	Côte d'Ivoire	Minimum Estimate	1344 - Social welfare managers	Lower-middle Income	West	Other Health Workers
27	Mental Health Nurse	858	959	1,091	0.41	24,307	Côte d'Ivoire	Minimum Estimate	2221 - Nursing professionals	Lower-middle Income	West	Nursing Personnel
28	Midwife	14,109	15,492	17,019	6.40	1,562	Côte d'Ivoire	Minimum Estimate	2222 - Midwifery professionals	Lower-middle Income	West	Midwifery Personnel
29	Nephrologist	194	223	261	0.10	101,455	Côte d'Ivoire	Minimum Estimate	2212 - Specialist medical practitioners	Lower-middle Income	West	Medical Doctors
30	Neuro-Surgeon	84	96	110	0.04	240,949	Côte d'Ivoire	Minimum Estimate	2212 - Specialist medical practitioners	Lower-middle Income	West	Medical Doctors
31	Nurse Anaesthetist	346	402	466	0.18	56,911	Côte d'Ivoire	Minimum Estimate	2221 - Nursing professionals	Lower-middle Income	West	Nursing Personnel
32	Nutritionist	2,798	2,976	3,157	1.19	8,416	Côte d'Ivoire	Minimum Estimate	2265 - Dieticians and nutritionists	Lower-middle Income	West	Other Health Workers
33	Obstetrician & Gynaecologist	1,354	1,512	1,690	0.64	15,720	Côte d'Ivoire	Minimum Estimate	2212 - Specialist medical practitioners	Lower-middle Income	West	Medical Doctors
34	Occupational Therapist	460	532	618	0.23	42,941	Côte d'Ivoire	Minimum Estimate	2269 - Health professionals not elsewhere classified	Lower-middle Income	West	Other Health Workers
35	Oncology Nurse	139	160	187	0.07	141,553	Côte d'Ivoire	Minimum Estimate	2221 - Nursing professionals	Lower-middle Income	West	Nursing Personnel
36	Operating Theatre Nurse	2,303	2,625	3,043	1.15	8,711	Côte d'Ivoire	Minimum Estimate	2221 - Nursing professionals	Lower-middle Income	West	Nursing Personnel
37	Ophthalmic Nurse	233	267	306	0.12	86,650	Côte d'Ivoire	Minimum Estimate	2221 - Nursing professionals	Lower-middle Income	West	Nursing Personnel
38	Ophthalmologist	64	75	88	0.03	300,831	Côte d'Ivoire	Minimum Estimate	2212 - Specialist medical practitioners	Lower-middle Income	West	Medical Doctors
39	Optometrist	236	273	317	0.12	83,567	Côte d'Ivoire	Minimum Estimate	2267 - Optometrists and ophthalmic opticians	Lower-middle Income	West	Other Health Workers
40	Orthopaedic Nurse	244	286	333	0.13	79,632	Côte d'Ivoire	Minimum Estimate	2221 - Nursing professionals	Lower-middle Income	West	Nursing Personnel
41	Orthopaedic Surgeon	695	806	938	0.35	28,303	Côte d'Ivoire	Minimum Estimate	2212 - Specialist medical practitioners	Lower-middle Income	West	Medical Doctors
42	Orthopaedic Technologist	702	787	881	0.33	30,166	Côte d'Ivoire	Minimum Estimate	3214 - Medical and dental prosthetic technicians	Lower-middle Income	West	Other Health Workers
43	Paediatric Nurse	2,396	2,658	2,927	1.10	9,091	Côte d'Ivoire	Minimum Estimate	2221 - Nursing professionals	Lower-middle Income	West	Nursing Personnel
44	Paediatric Surgeon	70	76	82	0.03	323,330	Côte d'Ivoire	Minimum Estimate	2212 - Specialist medical practitioners	Lower-middle Income	West	Medical Doctors
45	Paediatrician	843	953	1,081	0.41	24,580	Côte d'Ivoire	Minimum Estimate	2212 - Specialist medical practitioners	Lower-middle Income	West	Medical Doctors
46	Pathologist	125	141	163	0.06	162,573	Côte d'Ivoire	Minimum Estimate	2212 - Specialist medical practitioners	Lower-middle Income	West	Medical Doctors
47	Pharmacist	1,525	1,579	1,644	0.62	16,080	Côte d'Ivoire	Minimum Estimate	2262 - Pharmacists	Lower-middle Income	West	Pharmacist
48	Pharmacy Technician	2,046	2,261	2,504	0.94	10,601	Côte d'Ivoire	Minimum Estimate	3213 - Pharmaceutical technicians and assistants	Lower-middle Income	West	Other Health Workers
49	Physician	2,007	2,229	2,490	0.94	10,658	Côte d'Ivoire	Minimum Estimate	2212 - Specialist medical practitioners	Lower-middle Income	West	Medical Doctors
50	Physiotherapist	506	568	638	0.24	41,650	Côte d'Ivoire	Minimum Estimate	2264 - Physiotherapists	Lower-middle Income	West	Other Health Workers
51	Plastic Surgeon	127	146	167	0.06	159,237	Côte d'Ivoire	Minimum Estimate	2212 - Specialist medical practitioners	Lower-middle Income	West	Medical Doctors
52	Psychiatrist	926	1,025	1,149	0.43	23,092	Côte d'Ivoire	Minimum Estimate	2212 - Specialist medical practitioners	Lower-middle Income	West	Medical Doctors
53	Radiation Oncologist	31	37	43	0.02	614,401	Côte d'Ivoire	Minimum Estimate	2212 - Specialist medical practitioners	Lower-middle Income	West	Medical Doctors
54	Radiographer (Diagnostics and Therapy)	1,479	1,669	1,906	0.72	13,911	Côte d'Ivoire	Minimum Estimate	3211 - Medical imaging and therapeutic equipment technicians	Lower-middle Income	West	Other Health Workers
55	Radiologist	451	511	583	0.22	45,403	Côte d'Ivoire	Minimum Estimate	2212 - Specialist medical practitioners	Lower-middle Income	West	Medical Doctors
56	Registered General Nurse / State Certified Nurse	47,447	52,591	58,771	22.17	451	Côte d'Ivoire	Minimum Estimate	2221 - Nursing professionals	Lower-middle Income	West	Nursing Personnel
57	Renal Nurse	2,410	2,765	3,246	1.23	8,160	Côte d'Ivoire	Minimum Estimate	2221 - Nursing professionals	Lower-middle Income	West	Nursing Personnel
58	Respiratory Physician	139	157	177	0.07	150,289	Côte d'Ivoire	Minimum Estimate	2212 - Specialist medical practitioners	Lower-middle Income	West	Medical Doctors
59	Rheumatologist	58	66	75	0.03	355,857	Côte d'Ivoire	Minimum Estimate	2212 - Specialist medical practitioners	Lower-middle Income	West	Medical Doctors
60	Speech Therapist	195	216	240	0.09	110,977	Côte d'Ivoire	Minimum Estimate	2266 - Audiologists and speech therapists	Lower-middle Income	West	Other Health Workers
61	Urologist	25	30	38	0.01	698,752	Côte d'Ivoire	Minimum Estimate	2212 - Specialist medical practitioners	Lower-middle Income	West	Medical Doctors
1	Anaesthesiologist	504	591	692	0.08	130,118	Democratic Republi	Minimum Estimate	2212 - Specialist medical practitioners	Low Income	Central	Medical Doctors
2	Associate Nurse/Enrolled Nurse/Nursing Assistant	87,878	99,468	112,678	12.50	800	Democratic Republi	Minimum Estimate	3221 - Nursing associate professionals	Low Income	Central	Nursing Personnel
3	Audiologist	217	245	276	0.03	327,346	Democratic Republi	Minimum Estimate	2266 - Audiologists and speech therapists	Low Income	Central	Other Health Workers
4	Cardiologist	234	271	313	0.03	288,097	Democratic Republi	Minimum Estimate	2212 - Specialist medical practitioners	Low Income	Central	Medical Doctors
5	Cardiothoracic Surgeon	82	96	111	0.01	813,490	Democratic Republi	Minimum Estimate	2212 - Specialist medical practitioners	Low Income	Central	Medical Doctors
6	Clinical Officer/Physician Assistant	10,373	10,942	11,708	1.31	7,640	Democratic Republi	Minimum Estimate	3256 - Medical assistants	Low Income	Central	Other Health Workers
7	Clinical Pharmacist	1,729	1,963	2,245	0.25	40,048	Democratic Republi	Minimum Estimate	2262 - Pharmacists	Low Income	Central	Pharmacist
8	Clinical Psychologist	5,118	5,733	6,436	0.71	14,007	Democratic Republi	Minimum Estimate	2634 - Psychologists	Low Income	Central	Other Health Workers
9	Community health worker/Village health worker	60,943	64,633	68,582	7.62	1,312	Democratic Republi	Minimum Estimate	3253 - Community health workers	Low Income	Central	Other Health Workers
10	Dental Surgery Assistant	5,412	6,284	7,336	0.82	12,257	Democratic Republi	Minimum Estimate	3251 - Dental assistants and therapists	Low Income	Central	Other Health Workers
11	Dental Therapist	3,373	3,919	4,577	0.51	19,652	Democratic Republi	Minimum Estimate	3251 - Dental assistants and therapists	Low Income	Central	Other Health Workers
12	Dentist	4,207	4,832	5,646	0.63	15,889	Democratic Republi	Minimum Estimate	2261 - Dentists	Low Income	Central	Dentist
13	Dermatologist	249	283	320	0.04	281,713	Democratic Republi	Minimum Estimate	2212 - Specialist medical practitioners	Low Income	Central	Medical Doctors
14	Endocrinologist	259	314	389	0.04	228,630	Democratic Republi	Minimum Estimate	2212 - Specialist medical practitioners	Low Income	Central	Medical Doctors
15	ENT Surgeon	602	696	805	0.09	111,998	Democratic Republi	Minimum Estimate	2212 - Specialist medical practitioners	Low Income	Central	Medical Doctors
16	Environmental Health Officer	2,224	2,525	2,857	0.32	31,616	Democratic Republi	Minimum Estimate	2263 - Environmental and occupational health and hygiene professionals	Low Income	Central	Other Health Workers
17	Gastroenterologist	461	485	521	0.06	171,710	Democratic Republi	Minimum Estimate	2212 - Specialist medical practitioners	Low Income	Central	Medical Doctors
18	General Medical Practitioner (Generalist Doctor)	26,066	29,283	32,951	3.66	2,733	Democratic Republi	Minimum Estimate	2211 - Generalist medical practitioners	Low Income	Central	Medical Doctors
19	General Surgeon	599	698	824	0.09	108,838	Democratic Republi	Minimum Estimate	2212 - Specialist medical practitioners	Low Income	Central	Medical Doctors
20	Haematologist	233	262	293	0.03	308,519	Democratic Republi	Minimum Estimate	2212 - Specialist medical practitioners	Low Income	Central	Medical Doctors
21	Health Promoter/Health Educator	513	537	569	0.06	157,623	Democratic Republi	Minimum Estimate	2269 - Health professionals not elsewhere classified	Low Income	Central	Other Health Workers
22	Infectious Diseases Specialist	82	92	105	0.01	861,821	Democratic Republi	Minimum Estimate	2212 - Specialist medical practitioners	Low Income	Central	Medical Doctors
23	Intensive Care Nurse	2,263	2,553	2,876	0.32	31,367	Democratic Republi	Minimum Estimate	2221 - Nursing professionals	Low Income	Central	Nursing Personnel
24	Medical Laboratory Scientist	7,768	8,634	9,687	1.08	9,267	Democratic Republi	Minimum Estimate	3212 - Medical and pathology laboratory technicians	Low Income	Central	Other Health Workers
25	Medical Laboratory Technician	10,645	10,849	11,181	1.25	7,990	Democratic Republi	Minimum Estimate	3212 - Medical and pathology laboratory technicians	Low Income	Central	Other Health Workers
26	Medical Social Worker	2,412	2,566	2,717	0.30	33,149	Democratic Republi	Minimum Estimate	1344 - Social welfare managers	Low Income	Central	Other Health Workers
27	Mental Health Nurse	3,225	3,700	4,319	0.48	20,721	Democratic Republi	Minimum Estimate	2221 - Nursing professionals	Low Income	Central	Nursing Personnel
28	Midwife	49,932	55,399	61,921	6.89	1,452	Democratic Republi	Minimum Estimate	2222 - Midwifery professionals	Low Income	Central	Midwifery Personnel
29	Nephrologist	483	571	688	0.08	129,628	Democratic Republi	Minimum Estimate	2212 - Specialist medical practitioners	Low Income	Central	Medical Doctors
30	Neuro-Surgeon	231	272	321	0.04	280,278	Democratic Republi	Minimum Estimate	2212 - Specialist medical practitioners	Low Income	Central	Medical Doctors
31	Nurse Anaesthetist	1,035	1,238	1,478	0.16	60,842	Democratic Republi	Minimum Estimate	2221 - Nursing professionals	Low Income	Central	Nursing Personnel
32	Nutritionist	9,932	10,502	11,058	1.23	8,159	Democratic Republi	Minimum Estimate	2265 - Dieticians and nutritionists	Low Income	Central	Other Health Workers
33	Obstetrician & Gynaecologist	4,906	5,604	6,405	0.71	14,073	Democratic Republi	Minimum Estimate	2212 - Specialist medical practitioners	Low Income	Central	Medical Doctors
34	Occupational Therapist	1,139	1,341	1,584	0.18	56,719	Democratic Republi	Minimum Estimate	2269 - Health professionals not elsewhere classified	Low Income	Central	Other Health Workers
35	Oncology Nurse	378	449	540	0.06	165,468	Democratic Republi	Minimum Estimate	2221 - Nursing professionals	Low Income	Central	Nursing Personnel
36	Operating Theatre Nurse	6,578	7,656	9,028	1.01	9,914	Democratic Republi	Minimum Estimate	2221 - Nursing professionals	Low Income	Central	Nursing Personnel

S/N	Health Professionals	2022	2026	2030	Density per 10,000 population	Required Population ratio (1 professional is to xxx population)	Name of Country	Modelling Scenario	ISCO-08 Match	Income Group Classification	Sub-Regional Grouping	SDG 3c Occupation
37	Ophthalmic Nurse	689	802	939	0.10	95,614	Democratic Republi	Minimum Estimate	2221 - Nursing professionals	Low Income	Central	Nursing Personnel
38	Ophthalmologist	192	227	271	0.03	330,363	Democratic Republi	Minimum Estimate	2212 - Specialist medical practitioners	Low Income	Central	Medical Doctors
39	Optometrist	711	824	959	0.11	93,783	Democratic Republi	Minimum Estimate	2267 - Optometrists and ophthalmic opticians	Low Income	Central	Other Health Workers
40	Orthopaedic Nurse	618	735	871	0.10	103,354	Democratic Republi	Minimum Estimate	2221 - Nursing professionals	Low Income	Central	Nursing Personnel
41	Orthopaedic Surgeon	1,968	2,375	2,872	0.32	31,219	Democratic Republi	Minimum Estimate	2212 - Specialist medical practitioners	Low Income	Central	Medical Doctors
42	Orthopaedic Technologist	2,200	2,528	2,896	0.32	31,166	Democratic Republi	Minimum Estimate	3214 - Medical and dental prosthetic technicians	Low Income	Central	Other Health Workers
43	Paediatric Nurse	7,698	8,528	9,337	1.03	9,677	Democratic Republi	Minimum Estimate	2221 - Nursing professionals	Low Income	Central	Nursing Personnel
44	Paediatric Surgeon	233	257	279	0.03	324,500	Democratic Republi	Minimum Estimate	2212 - Specialist medical practitioners	Low Income	Central	Medical Doctors
45	Paediatrician	2,649	3,016	3,434	0.38	26,231	Democratic Republi	Minimum Estimate	2212 - Specialist medical practitioners	Low Income	Central	Medical Doctors
46	Pathologist	564	654	772	0.09	115,821	Democratic Republi	Minimum Estimate	2212 - Specialist medical practitioners	Low Income	Central	Medical Doctors
47	Pharmacist	6,646	7,230	7,884	0.88	11,427	Democratic Republi	Minimum Estimate	2262 - Pharmacists	Low Income	Central	Pharmacist
48	Pharmacy Technician	6,779	7,695	8,749	0.97	10,299	Democratic Republi	Minimum Estimate	3213 - Pharmaceutical technicians and assistants	Low Income	Central	Other Health Workers
49	Physician	6,263	7,101	8,100	0.90	11,095	Democratic Republi	Minimum Estimate	2212 - Specialist medical practitioners	Low Income	Central	Medical Doctors
50	Physiotherapist	1,589	1,825	2,095	0.23	43,030	Democratic Republi	Minimum Estimate	2264 - Physiotherapists	Low Income	Central	Other Health Workers
51	Plastic Surgeon	329	389	458	0.05	196,532	Democratic Republi	Minimum Estimate	2212 - Specialist medical practitioners	Low Income	Central	Medical Doctors
52	Psychiatrist	3,341	3,782	4,345	0.48	20,635	Democratic Republi	Minimum Estimate	2212 - Specialist medical practitioners	Low Income	Central	Medical Doctors
53	Radiation Oncologist	97	118	144	0.02	620,539	Democratic Republi	Minimum Estimate	2212 - Specialist medical practitioners	Low Income	Central	Medical Doctors
54	Radiographer (Diagnostics and Therapy)	4,145	4,836	5,697	0.64	15,735	Democratic Republi	Minimum Estimate	3211 - Medical imaging and therapeutic equipment technicians	Low Income	Central	Other Health Workers
55	Radiologist	1,300	1,514	1,776	0.20	50,545	Democratic Republi	Minimum Estimate	2212 - Specialist medical practitioners	Low Income	Central	Medical Doctors
56	Registered General Nurse / State Certified Nurse	165,922	181,050	199,564	22.28	449	Democratic Republi	Minimum Estimate	2221 - Nursing professionals	Low Income	Central	Nursing Personnel
57	Renal Nurse	6,028	7,130	8,587	0.96	10,386	Democratic Republi	Minimum Estimate	2221 - Nursing professionals	Low Income	Central	Nursing Personnel
58	Respiratory Physician	442	506	579	0.06	155,649	Democratic Republi	Minimum Estimate	2212 - Specialist medical practitioners	Low Income	Central	Medical Doctors
59	Rheumatologist	183	211	243	0.03	371,519	Democratic Republi	Minimum Estimate	2212 - Specialist medical practitioners	Low Income	Central	Medical Doctors
60	Speech Therapist	631	718	817	0.09	110,393	Democratic Republi	Minimum Estimate	2266 - Audiologists and speech therapists	Low Income	Central	Other Health Workers
61	Urologist	50	60	73	0.01	1,219,604	Democratic Republi	Minimum Estimate	2212 - Specialist medical practitioners	Low Income	Central	Medical Doctors
1	Anaesthesiologist	8	9	10	0.07	137,846	Equatorial Guinea	Minimum Estimate	2212 - Specialist medical practitioners	High Income and Upper Middle Income	West	Medical Doctors
2	Associate Nurse/Enrolled Nurse/Nursing Assistant	1,343	1,499	1,668	11.82	846	Equatorial Guinea	Minimum Estimate	3221 - Nursing associate professionals	High Income and Upper Middle Income	West	Nursing Personnel
3	Audiologist	3	3	4	0.02	400,549	Equatorial Guinea	Minimum Estimate	2266 - Audiologists and speech therapists	High Income and Upper Middle Income	West	Other Health Workers
4	Cardiologist	4	4	5	0.03	311,738	Equatorial Guinea	Minimum Estimate	2212 - Specialist medical practitioners	High Income and Upper Middle Income	West	Medical Doctors
5	Cardiothoracic Surgeon	1	1	1	0.01	1,088,282	Equatorial Guinea	Minimum Estimate	2212 - Specialist medical practitioners	High Income and Upper Middle Income	West	Medical Doctors
6	Clinical Officer/Physician Assistant	195	222	252	1.79	5,583	Equatorial Guinea	Minimum Estimate	3256 - Medical assistants	High Income and Upper Middle Income	West	Other Health Workers
7	Clinical Pharmacist	27	31	34	0.24	41,212	Equatorial Guinea	Minimum Estimate	2262 - Pharmacists	High Income and Upper Middle Income	West	Pharmacist
8	Clinical Psychologist	78	88	98	0.70	14,374	Equatorial Guinea	Minimum Estimate	2634 - Psychologists	High Income and Upper Middle Income	West	Other Health Workers
9	Community health worker/Village health worker	933	1,034	1,144	8.10	1,234	Equatorial Guinea	Minimum Estimate	3253 - Community health workers	High Income and Upper Middle Income	West	Other Health Workers
10	Dental Surgery Assistant	105	120	137	0.97	10,307	Equatorial Guinea	Minimum Estimate	3251 - Dental assistants and therapists	High Income and Upper Middle Income	West	Other Health Workers
11	Dental Therapist	66	75	86	0.61	16,457	Equatorial Guinea	Minimum Estimate	3251 - Dental assistants and therapists	High Income and Upper Middle Income	West	Other Health Workers
12	Dentist	77	87	100	0.71	14,110	Equatorial Guinea	Minimum Estimate	2261 - Dentists	High Income and Upper Middle Income	West	Dentist
13	Dermatologist	4	4	5	0.03	304,025	Equatorial Guinea	Minimum Estimate	2212 - Specialist medical practitioners	High Income and Upper Middle Income	West	Medical Doctors
14	Endocrinologist	5	6	8	0.05	184,828	Equatorial Guinea	Minimum Estimate	2212 - Specialist medical practitioners	High Income and Upper Middle Income	West	Medical Doctors
15	ENT Surgeon	9	10	12	0.08	121,177	Equatorial Guinea	Minimum Estimate	2212 - Specialist medical practitioners	High Income and Upper Middle Income	West	Medical Doctors
16	Environmental Health Officer	35	38	42	0.29	34,079	Equatorial Guinea	Minimum Estimate	2263 - Environmental and occupational health and hygiene professionals	High Income and Upper Middle Income	West	Other Health Workers
17	Gastroenterologist	5	5	6	0.04	254,410	Equatorial Guinea	Minimum Estimate	2212 - Specialist medical practitioners	High Income and Upper Middle Income	West	Medical Doctors
18	General Medical Practitioner (Generalist Doctor)	438	492	552	3.91	2,558	Equatorial Guinea	Minimum Estimate	2211 - Generalist medical practitioners	High Income and Upper Middle Income	West	Medical Doctors
19	General Surgeon	10	12	13	0.09	105,397	Equatorial Guinea	Minimum Estimate	2212 - Specialist medical practitioners	High Income and Upper Middle Income	West	Medical Doctors
20	Haematologist	3	3	3	0.02	475,133	Equatorial Guinea	Minimum Estimate	2212 - Specialist medical practitioners	High Income and Upper Middle Income	West	Medical Doctors
21	Health Promoter/Health Educator	9	10	11	0.08	129,352	Equatorial Guinea	Minimum Estimate	2269 - Health professionals not elsewhere classified	High Income and Upper Middle Income	West	Other Health Workers
22	Infectious Diseases Specialist	1	1	1	0.01	1,124,500	Equatorial Guinea	Minimum Estimate	2212 - Specialist medical practitioners	High Income and Upper Middle Income	West	Medical Doctors
23	Intensive Care Nurse	31	34	37	0.26	38,622	Equatorial Guinea	Minimum Estimate	2221 - Nursing professionals	High Income and Upper Middle Income	West	Nursing Personnel
24	Medical Laboratory Scientist	151	173	200	1.42	7,032	Equatorial Guinea	Minimum Estimate	3212 - Medical and pathology laboratory technicians	High Income and Upper Middle Income	West	Other Health Workers
25	Medical Laboratory Technician	206	238	275	1.95	5,119	Equatorial Guinea	Minimum Estimate	3212 - Medical and pathology laboratory technicians	High Income and Upper Middle Income	West	Other Health Workers
26	Medical Social Worker	23	24	25	0.17	57,154	Equatorial Guinea	Minimum Estimate	1344 - Social welfare managers	High Income and Upper Middle Income	West	Other Health Workers
27	Mental Health Nurse	65	74	83	0.59	16,917	Equatorial Guinea	Minimum Estimate	2221 - Nursing professionals	High Income and Upper Middle Income	West	Nursing Personnel
28	Midwife	757	826	903	6.38	1,567	Equatorial Guinea	Minimum Estimate	2222 - Midwifery professionals	High Income and Upper Middle Income	West	Midwifery Personnel
29	Nephrologist	11	12	14	0.10	98,842	Equatorial Guinea	Minimum Estimate	2212 - Specialist medical practitioners	High Income and Upper Middle Income	West	Medical Doctors
30	Neuro-Surgeon	4	4	4	0.03	325,873	Equatorial Guinea	Minimum Estimate	2212 - Specialist medical practitioners	High Income and Upper Middle Income	West	Medical Doctors
31	Nurse Anaesthetist	16	19	22	0.15	65,739	Equatorial Guinea	Minimum Estimate	2221 - Nursing professionals	High Income and Upper Middle Income	West	Nursing Personnel
32	Nutritionist	85	85	84	0.60	16,702	Equatorial Guinea	Minimum Estimate	2265 - Dieticians and nutritionists	High Income and Upper Middle Income	West	Other Health Workers
33	Obstetrician & Gynaecologist	80	89	98	0.69	14,410	Equatorial Guinea	Minimum Estimate	2212 - Specialist medical practitioners	High Income and Upper Middle Income	West	Medical Doctors
34	Occupational Therapist	20	23	25	0.18	55,508	Equatorial Guinea	Minimum Estimate	2269 - Health professionals not elsewhere classified	High Income and Upper Middle Income	West	Other Health Workers
35	Oncology Nurse	8	9	10	0.07	136,203	Equatorial Guinea	Minimum Estimate	2221 - Nursing professionals	High Income and Upper Middle Income	West	Nursing Personnel
36	Operating Theatre Nurse	127	144	162	1.15	8,668	Equatorial Guinea	Minimum Estimate	2221 - Nursing professionals	High Income and Upper Middle Income	West	Nursing Personnel
37	Ophthalmic Nurse	14	16	18	0.13	77,099	Equatorial Guinea	Minimum Estimate	2221 - Nursing professionals	High Income and Upper Middle Income	West	Nursing Personnel
38	Ophthalmologist	3	4	5	0.03	309,004	Equatorial Guinea	Minimum Estimate	2212 - Specialist medical practitioners	High Income and Upper Middle Income	West	Medical Doctors
39	Optometrist	10	11	12	0.09	113,916	Equatorial Guinea	Minimum Estimate	2267 - Optometrists and ophthalmic opticians	High Income and Upper Middle Income	West	Other Health Workers
40	Orthopaedic Nurse	10	11	12	0.09	114,444	Equatorial Guinea	Minimum Estimate	2221 - Nursing professionals	High Income and Upper Middle Income	West	Nursing Personnel
41	Orthopaedic Surgeon	44	51	60	0.42	23,717	Equatorial Guinea	Minimum Estimate	2212 - Specialist medical practitioners	High Income and Upper Middle Income	West	Medical Doctors
42	Orthopaedic Technologist	34	37	41	0.29	34,834	Equatorial Guinea	Minimum Estimate	3214 - Medical and dental prosthetic technicians	High Income and Upper Middle Income	West	Other Health Workers
43	Paediatric Nurse	93	98	102	0.72	13,831	Equatorial Guinea	Minimum Estimate	2221 - Nursing professionals	High Income and Upper Middle Income	West	Nursing Personnel
44	Paediatric Surgeon	3	3	3	0.02	446,832	Equatorial Guinea	Minimum Estimate	2212 - Specialist medical practitioners	High Income and Upper Middle Income	West	Medical Doctors
45	Paediatrician	43	47	51	0.36	27,529	Equatorial Guinea	Minimum Estimate	2212 - Specialist medical practitioners	High Income and Upper Middle Income	West	Medical Doctors
46	Pathologist	11	13	14	0.10	98,284	Equatorial Guinea	Minimum Estimate	2212 - Specialist medical practitioners	High Income and Upper Middle Income	West	Medical Doctors
47	Pharmacist	95	101	108	0.76	13,086	Equatorial Guinea	Minimum Estimate	2262 - Pharmacists	High Income and Upper Middle Income	West	Pharmacist
48	Pharmacy Technician	110	122	135	0.96	10,441	Equatorial Guinea	Minimum Estimate	3213 - Pharmaceutical technicians and assistants	High Income and Upper Middle Income	West	Other Health Workers
49	Physician	102	116	130	0.92	10,830	Equatorial Guinea	Minimum Estimate	2212 - Specialist medical practitioners	High Income and Upper Middle Income	West	Medical Doctors
50	Physiotherapist	25	28	30	0.22	46,420	Equatorial Guinea	Minimum Estimate	2264 - Physiotherapists	High Income and Upper Middle Income	West	Other Health Workers
51	Plastic Surgeon	5	6	7	0.05	195,751	Equatorial Guinea	Minimum Estimate	2212 - Specialist medical practitioners	High Income and Upper Middle Income	West	Medical Doctors
52	Psychiatrist	61	68	76	0.54	18,507	Equatorial Guinea	Minimum Estimate	2212 - Specialist medical practitioners	High Income and Upper Middle Income	West	Medical Doctors
53	Radiation Oncologist	2	2	3	0.02	551,919	Equatorial Guinea	Minimum Estimate	2212 - Specialist medical practitioners	High Income and Upper Middle Income	West	Medical Doctors
54	Radiographer (Diagnostics and Therapy)	77	88	100	0.71	14,162	Equatorial Guinea	Minimum Estimate	3211 - Medical imaging and therapeutic equipment technicians	High Income and Upper Middle Income	West	Other Health Workers
55	Radiologist	23	26	29	0.21	48,002	Equatorial Guinea	Minimum Estimate	2212 - Specialist medical practitioners	High Income and Upper Middle Income	West	Medical Doctors
56	Registered General Nurse / State Certified Nurse	2,269	2,544	2,836	20.13	497	Equatorial Guinea	Minimum Estimate	2221 - Nursing professionals	High Income and Upper Middle Income	West	Nursing Personnel
57	Renal Nurse	133	154	178	1.27	7,902	Equatorial Guinea	Minimum Estimate	2221 - Nursing professionals	High Income and Upper Middle Income	West	Nursing Personnel
58	Respiratory Physician	7	7	8	0.06	172,657	Equatorial Guinea	Minimum Estimate	2212 - Specialist medical practitioners	High Income and Upper Middle Income	West	Medical Doctors
59	Rheumatologist	3	3	4	0.03	375,768	Equatorial Guinea	Minimum Estimate	2212 - Specialist medical practitioners	High Income and Upper Middle Income	West	Medical Doctors

S/N	Health Professionals	2022	2026	2030	Density per 10,000 population	Required Population ratio (1 professional is to xxx population)	Name of Country	Modelling Scenario	ISCO-08 Match	Income Group Classification	Sub-Regional Grouping	SDG 3c Occupation
60	Speech Therapist	10	11	11	0.08	124,955	Equatorial Guinea	Minimum Estimate	2266 - Audiologists and speech therapists	High Income and Upper Middle Income	West	Other Health Workers
61	Urologist	1	1	1	0.01	1,071,123	Equatorial Guinea	Minimum Estimate	2212 - Specialist medical practitioners	High Income and Upper Middle Income	West	Medical Doctors
1	Anaesthesiologist	25	28	31	0.09	113,453	Eritrea	Minimum Estimate	2212 - Specialist medical practitioners	Low Income	East	Medical Doctors
2	Associate Nurse/Enrolled Nurse/Nursing Assistant	3,070	3,274	3,538	9.97	1,003	Eritrea	Minimum Estimate	3221 - Nursing associate professionals	Low Income	East	Nursing Personnel
3	Audiologist	9	10	11	0.03	322,639	Eritrea	Minimum Estimate	2266 - Audiologists and speech therapists	Low Income	East	Other Health Workers
4	Cardiologist	10	11	12	0.03	287,346	Eritrea	Minimum Estimate	2212 - Specialist medical practitioners	Low Income	East	Medical Doctors
5	Cardiothoracic Surgeon	5	5	6	0.02	626,915	Eritrea	Minimum Estimate	2212 - Specialist medical practitioners	Low Income	East	Medical Doctors
6	Clinical Officer/Physician Assistant	269	279	298	0.85	11,790	Eritrea	Minimum Estimate	3256 - Medical assistants	Low Income	East	Other Health Workers
7	Clinical Pharmacist	68	74	83	0.23	42,768	Eritrea	Minimum Estimate	2262 - Pharmacists	Low Income	East	Pharmacist
8	Clinical Psychologist	186	204	227	0.64	15,637	Eritrea	Minimum Estimate	2634 - Psychologists	Low Income	East	Other Health Workers
9	Community health worker/Village health worker	1,938	2,006	2,094	5.89	1,697	Eritrea	Minimum Estimate	3253 - Community health workers	Low Income	East	Other Health Workers
10	Dental Surgery Assistant	255	284	326	0.92	10,923	Eritrea	Minimum Estimate	3251 - Dental assistants and therapists	Low Income	East	Other Health Workers
11	Dental Therapist	158	175	201	0.57	17,692	Eritrea	Minimum Estimate	3251 - Dental assistants and therapists	Low Income	East	Other Health Workers
12	Dentist	222	246	288	0.81	12,342	Eritrea	Minimum Estimate	2261 - Dentists	Low Income	East	Dentist
13	Dermatologist	9	10	11	0.03	324,681	Eritrea	Minimum Estimate	2212 - Specialist medical practitioners	Low Income	East	Medical Doctors
14	Endocrinologist	9	10	12	0.04	283,718	Eritrea	Minimum Estimate	2212 - Specialist medical practitioners	Low Income	East	Medical Doctors
15	ENT Surgeon	28	32	35	0.10	101,264	Eritrea	Minimum Estimate	2212 - Specialist medical practitioners	Low Income	East	Medical Doctors
16	Environmental Health Officer	88	95	102	0.29	34,912	Eritrea	Minimum Estimate	2263 - Environmental and occupational health and hygiene professionals	Low Income	East	Other Health Workers
17	Gastroenterologist	16	16	17	0.05	204,959	Eritrea	Minimum Estimate	2212 - Specialist medical practitioners	Low Income	East	Medical Doctors
18	General Medical Practitioner (Generalist Doctor)	872	934	1,016	2.87	3,489	Eritrea	Minimum Estimate	2211 - Generalist medical practitioners	Low Income	East	Medical Doctors
19	General Surgeon	17	20	24	0.07	148,781	Eritrea	Minimum Estimate	2212 - Specialist medical practitioners	Low Income	East	Medical Doctors
20	Haematologist	10	11	12	0.03	301,527	Eritrea	Minimum Estimate	2212 - Specialist medical practitioners	Low Income	East	Medical Doctors
21	Health Promoter/Health Educator	15	16	16	0.05	214,324	Eritrea	Minimum Estimate	2269 - Health professionals not elsewhere classified	Low Income	East	Other Health Workers
22	Infectious Diseases Specialist	3	3	3	0.01	1,196,750	Eritrea	Minimum Estimate	2212 - Specialist medical practitioners	Low Income	East	Medical Doctors
23	Intensive Care Nurse	85	90	96	0.27	37,241	Eritrea	Minimum Estimate	2221 - Nursing professionals	Low Income	East	Nursing Personnel
24	Medical Laboratory Scientist	252	273	303	0.86	11,628	Eritrea	Minimum Estimate	3212 - Medical and pathology laboratory technicians	Low Income	East	Other Health Workers
25	Medical Laboratory Technician	204	220	241	0.68	14,689	Eritrea	Minimum Estimate	3212 - Medical and pathology laboratory technicians	Low Income	East	Other Health Workers
26	Medical Social Worker	93	94	95	0.27	37,011	Eritrea	Minimum Estimate	1344 - Social welfare managers	Low Income	East	Other Health Workers
27	Mental Health Nurse	137	150	172	0.49	20,390	Eritrea	Minimum Estimate	2221 - Nursing professionals	Low Income	East	Nursing Personnel
28	Midwife	1,854	1,986	2,186	6.14	1,629	Eritrea	Minimum Estimate	2222 - Midwifery professionals	Low Income	East	Midwifery Personnel
29	Nephrologist	22	25	30	0.09	115,521	Eritrea	Minimum Estimate	2212 - Specialist medical practitioners	Low Income	East	Medical Doctors
30	Neuro-Surgeon	10	12	13	0.04	273,719	Eritrea	Minimum Estimate	2212 - Specialist medical practitioners	Low Income	East	Medical Doctors
31	Nurse Anaesthetist	55	63	74	0.21	48,236	Eritrea	Minimum Estimate	2221 - Nursing professionals	Low Income	East	Nursing Personnel
32	Nutritionist	415	415	417	1.18	8,508	Eritrea	Minimum Estimate	2265 - Dieticians and nutritionists	Low Income	East	Other Health Workers
33	Obstetrician & Gynaecologist	189	206	227	0.64	15,685	Eritrea	Minimum Estimate	2212 - Specialist medical practitioners	Low Income	East	Medical Doctors
34	Occupational Therapist	52	58	66	0.19	53,553	Eritrea	Minimum Estimate	2269 - Health professionals not elsewhere classified	Low Income	East	Other Health Workers
35	Oncology Nurse	18	21	25	0.07	139,914	Eritrea	Minimum Estimate	2221 - Nursing professionals	Low Income	East	Nursing Personnel
36	Operating Theatre Nurse	270	303	352	1.00	9,966	Eritrea	Minimum Estimate	2221 - Nursing professionals	Low Income	East	Nursing Personnel
37	Ophthalmic Nurse	29	33	37	0.10	96,354	Eritrea	Minimum Estimate	2221 - Nursing professionals	Low Income	East	Nursing Personnel
38	Ophthalmologist	9	10	11	0.03	313,619	Eritrea	Minimum Estimate	2212 - Specialist medical practitioners	Low Income	East	Medical Doctors
39	Optometrist	33	38	44	0.12	81,318	Eritrea	Minimum Estimate	2267 - Optometrists and ophthalmic opticians	Low Income	East	Other Health Workers
40	Orthopaedic Nurse	27	31	35	0.10	103,086	Eritrea	Minimum Estimate	2221 - Nursing professionals	Low Income	East	Nursing Personnel
41	Orthopaedic Surgeon	40	48	61	0.18	56,809	Eritrea	Minimum Estimate	2212 - Specialist medical practitioners	Low Income	East	Medical Doctors
42	Orthopaedic Technologist	93	101	111	0.31	32,146	Eritrea	Minimum Estimate	3214 - Medical and dental prosthetic technicians	Low Income	East	Other Health Workers
43	Paediatric Nurse	313	327	343	0.97	10,350	Eritrea	Minimum Estimate	2221 - Nursing professionals	Low Income	East	Nursing Personnel
44	Paediatric Surgeon	8	9	9	0.02	401,155	Eritrea	Minimum Estimate	2212 - Specialist medical practitioners	Low Income	East	Medical Doctors
45	Paediatrician	125	136	148	0.42	23,890	Eritrea	Minimum Estimate	2212 - Specialist medical practitioners	Low Income	East	Medical Doctors
46	Pathologist	20	22	26	0.07	135,379	Eritrea	Minimum Estimate	2212 - Specialist medical practitioners	Low Income	East	Medical Doctors
47	Pharmacist	199	203	211	0.60	16,767	Eritrea	Minimum Estimate	2262 - Pharmacists	Low Income	East	Pharmacist
48	Pharmacy Technician	246	266	292	0.82	12,173	Eritrea	Minimum Estimate	3213 - Pharmaceutical technicians and assistants	Low Income	East	Other Health Workers
49	Physician	222	243	270	0.76	13,089	Eritrea	Minimum Estimate	2212 - Specialist medical practitioners	Low Income	East	Medical Doctors
50	Physiotherapist	67	73	80	0.23	44,350	Eritrea	Minimum Estimate	2264 - Physiotherapists	Low Income	East	Other Health Workers
51	Plastic Surgeon	38	42	46	0.13	76,983	Eritrea	Minimum Estimate	2212 - Specialist medical practitioners	Low Income	East	Medical Doctors
52	Psychiatrist	137	147	165	0.47	21,371	Eritrea	Minimum Estimate	2212 - Specialist medical practitioners	Low Income	East	Medical Doctors
53	Radiation Oncologist	6	7	8	0.02	444,294	Eritrea	Minimum Estimate	2212 - Specialist medical practitioners	Low Income	East	Medical Doctors
54	Radiographer (Diagnostics and Therapy)	157	176	203	0.58	17,313	Eritrea	Minimum Estimate	3211 - Medical imaging and therapeutic equipment technicians	Low Income	East	Other Health Workers
55	Radiologist	47	52	60	0.17	59,286	Eritrea	Minimum Estimate	2212 - Specialist medical practitioners	Low Income	East	Medical Doctors
56	Registered General Nurse / State Certified Nurse	6,899	7,259	7,783	22.09	453	Eritrea	Minimum Estimate	2221 - Nursing professionals	Low Income	East	Nursing Personnel
57	Renal Nurse	275	314	375	1.08	9,277	Eritrea	Minimum Estimate	2221 - Nursing professionals	Low Income	East	Nursing Personnel
58	Respiratory Physician	25	28	30	0.08	118,388	Eritrea	Minimum Estimate	2212 - Specialist medical practitioners	Low Income	East	Medical Doctors
59	Rheumatologist	8	8	9	0.03	380,276	Eritrea	Minimum Estimate	2212 - Specialist medical practitioners	Low Income	East	Medical Doctors
60	Speech Therapist	28	30	32	0.09	110,753	Eritrea	Minimum Estimate	2266 - Audiologists and speech therapists	Low Income	East	Other Health Workers
61	Urologist	2	3	3	0.01	1,142,372	Eritrea	Minimum Estimate	2212 - Specialist medical practitioners	Low Income	East	Medical Doctors
1	Anaesthesiologist	10	10	11	0.10	101,096	Eswatini	Minimum Estimate	2212 - Specialist medical practitioners	Lower-middle Income	Southern	Medical Doctors
2	Associate Nurse/Enrolled Nurse/Nursing Assistant	1,287	1,351	1,443	12.49	801	Eswatini	Minimum Estimate	3221 - Nursing associate professionals	Lower-middle Income	Southern	Nursing Personnel
3	Audiologist	3	3	3	0.03	342,714	Eswatini	Minimum Estimate	2266 - Audiologists and speech therapists	Lower-middle Income	Southern	Other Health Workers
4	Cardiologist	5	6	6	0.05	187,548	Eswatini	Minimum Estimate	2212 - Specialist medical practitioners	Lower-middle Income	Southern	Medical Doctors
5	Cardiothoracic Surgeon	1	1	1	0.01	800,947	Eswatini	Minimum Estimate	2212 - Specialist medical practitioners	Lower-middle Income	Southern	Medical Doctors
6	Clinical Officer/Physician Assistant	164	169	181	1.58	6,348	Eswatini	Minimum Estimate	3256 - Medical assistants	Lower-middle Income	Southern	Other Health Workers
7	Clinical Pharmacist	23	25	27	0.24	42,464	Eswatini	Minimum Estimate	2262 - Pharmacists	Lower-middle Income	Southern	Pharmacist
8	Clinical Psychologist	51	53	57	0.49	20,214	Eswatini	Minimum Estimate	2634 - Psychologists	Lower-middle Income	Southern	Other Health Workers
9	Community health worker/Village health worker	578	587	605	5.24	1,909	Eswatini	Minimum Estimate	3253 - Community health workers	Lower-middle Income	Southern	Other Health Workers
10	Dental Surgery Assistant	105	112	123	1.06	9,439	Eswatini	Minimum Estimate	3251 - Dental assistants and therapists	Lower-middle Income	Southern	Other Health Workers
11	Dental Therapist	65	70	76	0.66	15,210	Eswatini	Minimum Estimate	3251 - Dental assistants and therapists	Lower-middle Income	Southern	Other Health Workers
12	Dentist	88	92	101	0.87	11,473	Eswatini	Minimum Estimate	2261 - Dentists	Lower-middle Income	Southern	Dentist
13	Dermatologist	3	3	4	0.03	311,527	Eswatini	Minimum Estimate	2212 - Specialist medical practitioners	Lower-middle Income	Southern	Medical Doctors
14	Endocrinologist	5	6	7	0.06	169,395	Eswatini	Minimum Estimate	2212 - Specialist medical practitioners	Lower-middle Income	Southern	Medical Doctors
15	ENT Surgeon	11	11	12	0.11	94,260	Eswatini	Minimum Estimate	2212 - Specialist medical practitioners	Lower-middle Income	Southern	Medical Doctors
16	Environmental Health Officer	29	30	31	0.27	37,143	Eswatini	Minimum Estimate	2263 - Environmental and occupational health and hygiene professionals	Lower-middle Income	Southern	Other Health Workers
17	Gastroenterologist	6	6	6	0.05	199,323	Eswatini	Minimum Estimate	2212 - Specialist medical practitioners	Lower-middle Income	Southern	Medical Doctors
18	General Medical Practitioner (Generalist Doctor)	388	406	433	3.75	2,668	Eswatini	Minimum Estimate	2211 - Generalist medical practitioners	Lower-middle Income	Southern	Medical Doctors
19	General Surgeon	11	12	13	0.12	86,394	Eswatini	Minimum Estimate	2212 - Specialist medical practitioners	Lower-middle Income	Southern	Medical Doctors
20	Haematologist	2	2	2	0.02	623,053	Eswatini	Minimum Estimate	2212 - Specialist medical practitioners	Lower-middle Income	Southern	Medical Doctors
21	Health Promoter/Health Educator	8	8	8	0.07	143,269	Eswatini	Minimum Estimate	2269 - Health professionals not elsewhere classified	Lower-middle Income	Southern	Other Health Workers

S/N	Health Professionals	2022	2026	2030	Density per 10,000 population	Required Population ratio (1 professional is to xxx population)	Name of Country	Modelling Scenario	ISCO-08 Match	Income Group Classification	Sub-Regional Grouping	SDG 3c Occupation
22	Infectious Diseases Specialist	1	1	1	0.01	1,034,736	Eswatini	Minimum Estimate	2212 - Specialist medical practitioners	Lower-middle Income	Southern	Medical Doctors
23	Intensive Care Nurse	21	22	24	0.20	49,111	Eswatini	Minimum Estimate	2221 - Nursing professionals	Lower-middle Income	Southern	Nursing Personnel
24	Medical Laboratory Scientist	172	182	199	1.73	5,793	Eswatini	Minimum Estimate	3212 - Medical and pathology laboratory technicians	Lower-middle Income	Southern	Other Health Workers
25	Medical Laboratory Technician	147	156	170	1.48	6,779	Eswatini	Minimum Estimate	3212 - Medical and pathology laboratory technicians	Lower-middle Income	Southern	Other Health Workers
26	Medical Social Worker	18	18	18	0.16	61,965	Eswatini	Minimum Estimate	1344 - Social welfare managers	Lower-middle Income	Southern	Other Health Workers
27	Mental Health Nurse	43	45	51	0.44	22,520	Eswatini	Minimum Estimate	2221 - Nursing professionals	Lower-middle Income	Southern	Nursing Personnel
28	Midwife	624	646	685	5.90	1,695	Eswatini	Minimum Estimate	2222 - Midwifery professionals	Lower-middle Income	Southern	Midwifery Personnel
29	Nephrologist	13	15	18	0.16	64,127	Eswatini	Minimum Estimate	2212 - Specialist medical practitioners	Lower-middle Income	Southern	Medical Doctors
30	Neuro-Surgeon	4	4	5	0.04	239,982	Eswatini	Minimum Estimate	2212 - Specialist medical practitioners	Lower-middle Income	Southern	Medical Doctors
31	Nurse Anaesthetist	24	26	30	0.26	39,102	Eswatini	Minimum Estimate	2221 - Nursing professionals	Lower-middle Income	Southern	Nursing Personnel
32	Nutritionist	46	46	47	0.41	24,597	Eswatini	Minimum Estimate	2265 - Dieticians and nutritionists	Lower-middle Income	Southern	Other Health Workers
33	Obstetrician & Gynaecologist	75	79	86	0.74	13,431	Eswatini	Minimum Estimate	2212 - Specialist medical practitioners	Lower-middle Income	Southern	Medical Doctors
34	Occupational Therapist	18	20	22	0.19	51,749	Eswatini	Minimum Estimate	2269 - Health professionals not elsewhere classified	Lower-middle Income	Southern	Other Health Workers
35	Oncology Nurse	10	11	13	0.11	87,962	Eswatini	Minimum Estimate	2221 - Nursing professionals	Lower-middle Income	Southern	Nursing Personnel
36	Operating Theatre Nurse	119	131	150	1.31	7,660	Eswatini	Minimum Estimate	2221 - Nursing professionals	Lower-middle Income	Southern	Nursing Personnel
37	Ophthalmic Nurse	14	15	17	0.14	69,713	Eswatini	Minimum Estimate	2221 - Nursing professionals	Lower-middle Income	Southern	Nursing Personnel
38	Ophthalmologist	5	5	6	0.05	196,435	Eswatini	Minimum Estimate	2212 - Specialist medical practitioners	Lower-middle Income	Southern	Medical Doctors
39	Optometrist	22	24	27	0.24	42,502	Eswatini	Minimum Estimate	2267 - Optometrists and ophthalmic opticians	Lower-middle Income	Southern	Other Health Workers
40	Orthopaedic Nurse	9	10	11	0.09	106,886	Eswatini	Minimum Estimate	2221 - Nursing professionals	Lower-middle Income	Southern	Nursing Personnel
41	Orthopaedic Surgeon	37	42	48	0.41	24,305	Eswatini	Minimum Estimate	2212 - Specialist medical practitioners	Lower-middle Income	Southern	Medical Doctors
42	Orthopaedic Technologist	29	31	33	0.28	35,406	Eswatini	Minimum Estimate	3214 - Medical and dental prosthetic technicians	Lower-middle Income	Southern	Other Health Workers
43	Paediatric Nurse	65	67	68	0.59	17,062	Eswatini	Minimum Estimate	2221 - Nursing professionals	Lower-middle Income	Southern	Nursing Personnel
44	Paediatric Surgeon	2	2	2	0.02	477,122	Eswatini	Minimum Estimate	2212 - Specialist medical practitioners	Lower-middle Income	Southern	Medical Doctors
45	Paediatrician	41	42	46	0.40	25,144	Eswatini	Minimum Estimate	2212 - Specialist medical practitioners	Lower-middle Income	Southern	Medical Doctors
46	Pathologist	10	11	13	0.11	87,667	Eswatini	Minimum Estimate	2212 - Specialist medical practitioners	Lower-middle Income	Southern	Medical Doctors
47	Pharmacist	82	81	82	0.71	14,003	Eswatini	Minimum Estimate	2262 - Pharmacists	Lower-middle Income	Southern	Pharmacist
48	Pharmacy Technician	96	100	107	0.92	10,834	Eswatini	Minimum Estimate	3213 - Pharmaceutical technicians and assistants	Lower-middle Income	Southern	Other Health Workers
49	Physician	89	95	104	0.90	11,066	Eswatini	Minimum Estimate	2212 - Specialist medical practitioners	Lower-middle Income	Southern	Medical Doctors
50	Physiotherapist	22	24	25	0.22	45,675	Eswatini	Minimum Estimate	2264 - Physiotherapists	Lower-middle Income	Southern	Other Health Workers
51	Plastic Surgeon	7	8	8	0.07	141,847	Eswatini	Minimum Estimate	2212 - Specialist medical practitioners	Lower-middle Income	Southern	Medical Doctors
52	Psychiatrist	40	42	47	0.41	24,597	Eswatini	Minimum Estimate	2212 - Specialist medical practitioners	Lower-middle Income	Southern	Medical Doctors
53	Radiation Oncologist	3	3	3	0.03	336,690	Eswatini	Minimum Estimate	2212 - Specialist medical practitioners	Lower-middle Income	Southern	Medical Doctors
54	Radiographer (Diagnostics and Therapy)	80	87	98	0.86	11,681	Eswatini	Minimum Estimate	3211 - Medical imaging and therapeutic equipment technicians	Lower-middle Income	Southern	Other Health Workers
55	Radiologist	24	26	29	0.25	39,493	Eswatini	Minimum Estimate	2212 - Specialist medical practitioners	Lower-middle Income	Southern	Medical Doctors
56	Registered General Nurse / State Certified Nurse	2,402	2,545	2,799	24.40	410	Eswatini	Minimum Estimate	2221 - Nursing professionals	Lower-middle Income	Southern	Nursing Personnel
57	Renal Nurse	160	182	220	1.93	5,180	Eswatini	Minimum Estimate	2221 - Nursing professionals	Lower-middle Income	Southern	Nursing Personnel
58	Respiratory Physician	8	8	9	0.08	132,369	Eswatini	Minimum Estimate	2212 - Specialist medical practitioners	Lower-middle Income	Southern	Medical Doctors
59	Rheumatologist	3	3	3	0.03	334,989	Eswatini	Minimum Estimate	2212 - Specialist medical practitioners	Lower-middle Income	Southern	Medical Doctors
60	Speech Therapist	9	9	10	0.09	116,683	Eswatini	Minimum Estimate	2266 - Audiologists and speech therapists	Lower-middle Income	Southern	Other Health Workers
61	Urologist	1	1	2	0.01	692,985	Eswatini	Minimum Estimate	2212 - Specialist medical practitioners	Lower-middle Income	Southern	Medical Doctors
1	Anaesthesiologist	612	691	781	0.07	148,565	Ethiopia	Minimum Estimate	2212 - Specialist medical practitioners	Low Income	East	Medical Doctors
2	Associate Nurse/Enrolled Nurse/Nursing Assistant	101,885	112,097	123,411	10.64	940	Ethiopia	Minimum Estimate	3221 - Nursing associate professionals	Low Income	East	Nursing Personnel
3	Audiologist	307	340	374	0.03	309,814	Ethiopia	Minimum Estimate	2266 - Audiologists and speech therapists	Low Income	East	Other Health Workers
4	Cardiologist	295	338	385	0.03	301,336	Ethiopia	Minimum Estimate	2212 - Specialist medical practitioners	Low Income	East	Medical Doctors
5	Cardiothoracic Surgeon	82	90	98	0.01	1,190,604	Ethiopia	Minimum Estimate	2212 - Specialist medical practitioners	Low Income	East	Medical Doctors
6	Clinical Officer/Physician Assistant	19,387	21,978	25,048	2.17	4,619	Ethiopia	Minimum Estimate	3256 - Medical assistants	Low Income	East	Other Health Workers
7	Clinical Pharmacist	1,832	2,030	2,280	0.20	50,782	Ethiopia	Minimum Estimate	2262 - Pharmacists	Low Income	East	Pharmacist
8	Clinical Psychologist	5,083	5,601	6,224	0.54	18,633	Ethiopia	Minimum Estimate	2634 - Psychologists	Low Income	East	Other Health Workers
9	Community health worker/Village health worker	75,141	82,164	89,322	7.70	1,300	Ethiopia	Minimum Estimate	3253 - Community health workers	Low Income	East	Other Health Workers
10	Dental Surgery Assistant	6,711	7,577	8,568	0.74	13,539	Ethiopia	Minimum Estimate	3251 - Dental assistants and therapists	Low Income	East	Other Health Workers
11	Dental Therapist	4,174	4,719	5,339	0.46	21,726	Ethiopia	Minimum Estimate	3251 - Dental assistants and therapists	Low Income	East	Other Health Workers
12	Dentist	5,396	5,996	6,742	0.58	17,185	Ethiopia	Minimum Estimate	2261 - Dentists	Low Income	East	Dentist
13	Dermatologist	287	321	357	0.03	325,707	Ethiopia	Minimum Estimate	2212 - Specialist medical practitioners	Low Income	East	Medical Doctors
14	Endocrinologist	184	213	258	0.02	446,664	Ethiopia	Minimum Estimate	2212 - Specialist medical practitioners	Low Income	East	Medical Doctors
15	ENT Surgeon	918	1,046	1,192	0.10	97,301	Ethiopia	Minimum Estimate	2212 - Specialist medical practitioners	Low Income	East	Medical Doctors
16	Environmental Health Officer	2,855	3,150	3,453	0.30	33,653	Ethiopia	Minimum Estimate	2263 - Environmental and occupational health and hygiene professionals	Low Income	East	Other Health Workers
17	Gastroenterologist	544	573	629	0.05	184,005	Ethiopia	Minimum Estimate	2212 - Specialist medical practitioners	Low Income	East	Medical Doctors
18	General Medical Practitioner (Generalist Doctor)	32,327	36,213	40,574	3.50	2,857	Ethiopia	Minimum Estimate	2211 - Generalist medical practitioners	Low Income	East	Medical Doctors
19	General Surgeon	500	568	654	0.06	176,530	Ethiopia	Minimum Estimate	2212 - Specialist medical practitioners	Low Income	East	Medical Doctors
20	Haematologist	222	240	258	0.02	449,675	Ethiopia	Minimum Estimate	2212 - Specialist medical practitioners	Low Income	East	Medical Doctors
21	Health Promoter/Health Educator	1,099	1,223	1,360	0.12	85,340	Ethiopia	Minimum Estimate	2269 - Health professionals not elsewhere classified	Low Income	East	Other Health Workers
22	Infectious Diseases Specialist	63	68	74	0.01	1,556,090	Ethiopia	Minimum Estimate	2212 - Specialist medical practitioners	Low Income	East	Medical Doctors
23	Intensive Care Nurse	2,419	2,619	2,820	0.24	41,154	Ethiopia	Minimum Estimate	2221 - Nursing professionals	Low Income	East	Nursing Personnel
24	Medical Laboratory Scientist	8,021	9,034	10,329	0.89	11,192	Ethiopia	Minimum Estimate	3212 - Medical and pathology laboratory technicians	Low Income	East	Other Health Workers
25	Medical Laboratory Technician	7,777	8,797	10,037	0.87	11,518	Ethiopia	Minimum Estimate	3212 - Medical and pathology laboratory technicians	Low Income	East	Other Health Workers
26	Medical Social Worker	3,311	3,510	3,726	0.32	31,107	Ethiopia	Minimum Estimate	1344 - Social welfare managers	Low Income	East	Other Health Workers
27	Mental Health Nurse	4,467	5,023	5,833	0.50	19,811	Ethiopia	Minimum Estimate	2221 - Nursing professionals	Low Income	East	Nursing Personnel
28	Midwife	60,354	66,354	73,022	6.29	1,589	Ethiopia	Minimum Estimate	2222 - Midwifery professionals	Low Income	East	Midwifery Personnel
29	Nephrologist	690	800	967	0.08	119,289	Ethiopia	Minimum Estimate	2212 - Specialist medical practitioners	Low Income	East	Medical Doctors
30	Neuro-Surgeon	221	258	304	0.03	378,969	Ethiopia	Minimum Estimate	2212 - Specialist medical practitioners	Low Income	East	Medical Doctors
31	Nurse Anaesthetist	1,323	1,519	1,743	0.15	66,554	Ethiopia	Minimum Estimate	2221 - Nursing professionals	Low Income	East	Nursing Personnel
32	Nutritionist	14,569	15,268	15,891	1.37	7,296	Ethiopia	Minimum Estimate	2265 - Dieticians and nutritionists	Low Income	East	Other Health Workers
33	Obstetrician & Gynaecologist	5,937	6,723	7,657	0.66	15,102	Ethiopia	Minimum Estimate	2212 - Specialist medical practitioners	Low Income	East	Medical Doctors
34	Occupational Therapist	1,247	1,424	1,650	0.14	70,204	Ethiopia	Minimum Estimate	2269 - Health professionals not elsewhere classified	Low Income	East	Other Health Workers
35	Oncology Nurse	547	635	759	0.07	152,169	Ethiopia	Minimum Estimate	2221 - Nursing professionals	Low Income	East	Nursing Personnel
36	Operating Theatre Nurse	8,473	9,721	11,537	1.00	10,012	Ethiopia	Minimum Estimate	2221 - Nursing professionals	Low Income	East	Nursing Personnel
37	Ophthalmic Nurse	984	1,097	1,228	0.11	94,511	Ethiopia	Minimum Estimate	2221 - Nursing professionals	Low Income	East	Nursing Personnel
38	Ophthalmologist	223	252	291	0.03	397,474	Ethiopia	Minimum Estimate	2212 - Specialist medical practitioners	Low Income	East	Medical Doctors
39	Optometrist	673	758	856	0.07	135,592	Ethiopia	Minimum Estimate	2267 - Optometrists and ophthalmic opticians	Low Income	East	Other Health Workers
40	Orthopaedic Nurse	524	604	692	0.06	167,595	Ethiopia	Minimum Estimate	2221 - Nursing professionals	Low Income	East	Nursing Personnel
41	Orthopaedic Surgeon	2,098	2,518	3,053	0.26	37,783	Ethiopia	Minimum Estimate	2212 - Specialist medical practitioners	Low Income	East	Medical Doctors
42	Orthopaedic Technologist	2,603	2,886	3,181	0.27	36,513	Ethiopia	Minimum Estimate	3214 - Medical and dental prosthetic technicians	Low Income	East	Other Health Workers
43	Paediatric Nurse	9,449	10,188	10,841	0.93	10,726	Ethiopia	Minimum Estimate	2221 - Nursing professionals	Low Income	East	Nursing Personnel
44	Paediatric Surgeon	289	317	342	0.03	339,640	Ethiopia	Minimum Estimate	2212 - Specialist medical practitioners	Low Income	East	Medical Doctors

S/N	Health Professionals	2022	2026	2030	Density per 10,000 population	Required Population ratio (1 professional is to xxx population)	Name of Country	Modelling Scenario	ISCO-08 Match	Income Group Classification	Sub-Regional Grouping	SDG 3c Occupation
45	Paediatrician	3,051	3,398	3,812	0.33	30,429	Ethiopia	Minimum Estimate	2212 - Specialist medical practitioners	Low Income	East	Medical Doctors
46	Pathologist	587	738	968	0.09	117,622	Ethiopia	Minimum Estimate	2212 - Specialist medical practitioners	Low Income	East	Medical Doctors
47	Pharmacist	14,739	16,469	18,323	1.58	6,338	Ethiopia	Minimum Estimate	2262 - Pharmacists	Low Income	East	Pharmacist
48	Pharmacy Technician	9,675	10,815	12,064	1.04	9,623	Ethiopia	Minimum Estimate	3213 - Pharmaceutical technicians and assistants	Low Income	East	Other Health Workers
49	Physician	6,070	6,765	7,602	0.66	15,246	Ethiopia	Minimum Estimate	2212 - Specialist medical practitioners	Low Income	East	Medical Doctors
50	Physiotherapist	1,946	2,167	2,416	0.21	48,036	Ethiopia	Minimum Estimate	2264 - Physiotherapists	Low Income	East	Other Health Workers
51	Plastic Surgeon	790	905	1,030	0.09	112,779	Ethiopia	Minimum Estimate	2212 - Specialist medical practitioners	Low Income	East	Medical Doctors
52	Psychiatrist	4,561	5,047	5,718	0.49	20,228	Ethiopia	Minimum Estimate	2212 - Specialist medical practitioners	Low Income	East	Medical Doctors
53	Radiation Oncologist	146	172	205	0.02	561,752	Ethiopia	Minimum Estimate	2212 - Specialist medical practitioners	Low Income	East	Medical Doctors
54	Radiographer (Diagnostics and Therapy)	4,772	5,457	6,379	0.55	18,125	Ethiopia	Minimum Estimate	3211 - Medical imaging and therapeutic equipment technicians	Low Income	East	Other Health Workers
55	Radiologist	1,452	1,668	1,940	0.17	59,505	Ethiopia	Minimum Estimate	2212 - Specialist medical practitioners	Low Income	East	Medical Doctors
56	Registered General Nurse / State Certified Nurse	313,462	342,539	375,272	32.36	309	Ethiopia	Minimum Estimate	2221 - Nursing professionals	Low Income	East	Nursing Personnel
57	Renal Nurse	8,492	9,846	11,913	1.03	9,683	Ethiopia	Minimum Estimate	2221 - Nursing professionals	Low Income	East	Nursing Personnel
58	Respiratory Physician	531	593	660	0.06	175,954	Ethiopia	Minimum Estimate	2212 - Specialist medical practitioners	Low Income	East	Medical Doctors
59	Rheumatologist	242	274	309	0.03	375,896	Ethiopia	Minimum Estimate	2212 - Specialist medical practitioners	Low Income	East	Medical Doctors
60	Speech Therapist	844	939	1,042	0.09	111,365	Ethiopia	Minimum Estimate	2266 - Audiologists and speech therapists	Low Income	East	Other Health Workers
61	Urologist	81	95	116	0.01	991,006	Ethiopia	Minimum Estimate	2212 - Specialist medical practitioners	Low Income	East	Medical Doctors
1	Anaesthesiologist	19	21	23	0.10	97,288	Gabon	Minimum Estimate	2212 - Specialist medical practitioners	High Income and Upper Middle Income	Central	Medical Doctors
2	Associate Nurse/Enrolled Nurse/Nursing Assistant	2,480	2,707	2,940	13.12	762	Gabon	Minimum Estimate	3221 - Nursing associate professionals	High Income and Upper Middle Income	Central	Nursing Personnel
3	Audiologist	5	6	6	0.03	359,885	Gabon	Minimum Estimate	2266 - Audiologists and speech therapists	High Income and Upper Middle Income	Central	Other Health Workers
4	Cardiologist	10	11	12	0.05	184,469	Gabon	Minimum Estimate	2212 - Specialist medical practitioners	High Income and Upper Middle Income	Central	Medical Doctors
5	Cardiothoracic Surgeon	3	3	3	0.02	663,390	Gabon	Minimum Estimate	2212 - Specialist medical practitioners	High Income and Upper Middle Income	Central	Medical Doctors
6	Clinical Officer/Physician Assistant	355	385	418	1.88	5,332	Gabon	Minimum Estimate	3256 - Medical assistants	High Income and Upper Middle Income	Central	Other Health Workers
7	Clinical Pharmacist	54	59	64	0.29	34,641	Gabon	Minimum Estimate	2262 - Pharmacists	High Income and Upper Middle Income	Central	Pharmacist
8	Clinical Psychologist	138	151	165	0.74	13,585	Gabon	Minimum Estimate	2634 - Psychologists	High Income and Upper Middle Income	Central	Other Health Workers
9	Community health worker/Village health worker	1,252	1,364	1,475	6.60	1,514	Gabon	Minimum Estimate	3253 - Community health workers	High Income and Upper Middle Income	Central	Other Health Workers
10	Dental Surgery Assistant	180	200	219	0.98	10,202	Gabon	Minimum Estimate	3251 - Dental assistants and therapists	High Income and Upper Middle Income	Central	Other Health Workers
11	Dental Therapist	114	126	139	0.62	16,139	Gabon	Minimum Estimate	3251 - Dental assistants and therapists	High Income and Upper Middle Income	Central	Other Health Workers
12	Dentist	117	128	138	0.62	16,194	Gabon	Minimum Estimate	2261 - Dentists	High Income and Upper Middle Income	Central	Dentist
13	Dermatologist	6	7	8	0.03	293,833	Gabon	Minimum Estimate	2212 - Specialist medical practitioners	High Income and Upper Middle Income	Central	Medical Doctors
14	Endocrinologist	14	16	18	0.08	121,320	Gabon	Minimum Estimate	2212 - Specialist medical practitioners	High Income and Upper Middle Income	Central	Medical Doctors
15	ENT Surgeon	19	21	24	0.11	94,220	Gabon	Minimum Estimate	2212 - Specialist medical practitioners	High Income and Upper Middle Income	Central	Medical Doctors
16	Environmental Health Officer	55	60	64	0.29	34,964	Gabon	Minimum Estimate	2263 - Environmental and occupational health and hygiene professionals	High Income and Upper Middle Income	Central	Other Health Workers
17	Gastroenterologist	18	18	18	0.08	124,181	Gabon	Minimum Estimate	2212 - Specialist medical practitioners	High Income and Upper Middle Income	Central	Medical Doctors
18	General Medical Practitioner (Generalist Doctor)	756	823	889	3.97	2,516	Gabon	Minimum Estimate	2211 - Generalist medical practitioners	High Income and Upper Middle Income	Central	Medical Doctors
19	General Surgeon	21	23	27	0.12	83,883	Gabon	Minimum Estimate	2212 - Specialist medical practitioners	High Income and Upper Middle Income	Central	Medical Doctors
20	Haematologist	7	7	7	0.03	302,911	Gabon	Minimum Estimate	2212 - Specialist medical practitioners	High Income and Upper Middle Income	Central	Medical Doctors
21	Health Promoter/Health Educator	17	18	19	0.09	116,600	Gabon	Minimum Estimate	2269 - Health professionals not elsewhere classified	High Income and Upper Middle Income	Central	Other Health Workers
22	Infectious Diseases Specialist	2	2	2	0.01	925,547	Gabon	Minimum Estimate	2212 - Specialist medical practitioners	High Income and Upper Middle Income	Central	Medical Doctors
23	Intensive Care Nurse	53	57	61	0.27	36,665	Gabon	Minimum Estimate	2221 - Nursing professionals	High Income and Upper Middle Income	Central	Nursing Personnel
24	Medical Laboratory Scientist	259	286	316	1.42	7,065	Gabon	Minimum Estimate	3212 - Medical and pathology laboratory technicians	High Income and Upper Middle Income	Central	Other Health Workers
25	Medical Laboratory Technician	241	275	313	1.41	7,117	Gabon	Minimum Estimate	3212 - Medical and pathology laboratory technicians	High Income and Upper Middle Income	Central	Other Health Workers
26	Medical Social Worker	50	52	53	0.24	42,038	Gabon	Minimum Estimate	1344 - Social welfare managers	High Income and Upper Middle Income	Central	Other Health Workers
27	Mental Health Nurse	104	114	125	0.56	17,796	Gabon	Minimum Estimate	2221 - Nursing professionals	High Income and Upper Middle Income	Central	Nursing Personnel
28	Midwife	1,171	1,263	1,333	5.96	1,678	Gabon	Minimum Estimate	2222 - Midwifery professionals	High Income and Upper Middle Income	Central	Midwifery Personnel
29	Nephrologist	23	26	29	0.13	75,672	Gabon	Minimum Estimate	2212 - Specialist medical practitioners	High Income and Upper Middle Income	Central	Medical Doctors
30	Neuro-Surgeon	9	10	11	0.05	208,291	Gabon	Minimum Estimate	2212 - Specialist medical practitioners	High Income and Upper Middle Income	Central	Medical Doctors
31	Nurse Anaesthetist	48	56	64	0.29	34,840	Gabon	Minimum Estimate	2221 - Nursing professionals	High Income and Upper Middle Income	Central	Nursing Personnel
32	Nutritionist	123	128	132	0.59	17,010	Gabon	Minimum Estimate	2265 - Dieticians and nutritionists	High Income and Upper Middle Income	Central	Other Health Workers
33	Obstetrician & Gynaecologist	139	153	168	0.75	13,349	Gabon	Minimum Estimate	2212 - Specialist medical practitioners	High Income and Upper Middle Income	Central	Medical Doctors
34	Occupational Therapist	45	50	56	0.25	40,242	Gabon	Minimum Estimate	2269 - Health professionals not elsewhere classified	High Income and Upper Middle Income	Central	Other Health Workers
35	Oncology Nurse	18	21	24	0.11	91,296	Gabon	Minimum Estimate	2221 - Nursing professionals	High Income and Upper Middle Income	Central	Nursing Personnel
36	Operating Theatre Nurse	256	287	320	1.44	6,959	Gabon	Minimum Estimate	2221 - Nursing professionals	High Income and Upper Middle Income	Central	Nursing Personnel
37	Ophthalmic Nurse	36	41	46	0.21	48,777	Gabon	Minimum Estimate	2221 - Nursing professionals	High Income and Upper Middle Income	Central	Nursing Personnel
38	Ophthalmologist	8	9	11	0.05	207,074	Gabon	Minimum Estimate	2212 - Specialist medical practitioners	High Income and Upper Middle Income	Central	Medical Doctors
39	Optometrist	27	30	34	0.15	65,071	Gabon	Minimum Estimate	2267 - Optometrists and ophthalmic opticians	High Income and Upper Middle Income	Central	Other Health Workers
40	Orthopaedic Nurse	23	25	28	0.13	79,717	Gabon	Minimum Estimate	2221 - Nursing professionals	High Income and Upper Middle Income	Central	Nursing Personnel
41	Orthopaedic Surgeon	86	98	112	0.50	19,955	Gabon	Minimum Estimate	2212 - Specialist medical practitioners	High Income and Upper Middle Income	Central	Medical Doctors
42	Orthopaedic Technologist	60	65	71	0.32	31,617	Gabon	Minimum Estimate	3214 - Medical and dental prosthetic technicians	High Income and Upper Middle Income	Central	Other Health Workers
43	Paediatric Nurse	161	171	176	0.79	12,729	Gabon	Minimum Estimate	2221 - Nursing professionals	High Income and Upper Middle Income	Central	Nursing Personnel
44	Paediatric Surgeon	5	5	5	0.02	448,532	Gabon	Minimum Estimate	2212 - Specialist medical practitioners	High Income and Upper Middle Income	Central	Medical Doctors
45	Paediatrician	71	78	84	0.38	26,524	Gabon	Minimum Estimate	2212 - Specialist medical practitioners	High Income and Upper Middle Income	Central	Medical Doctors
46	Pathologist	22	24	27	0.12	82,464	Gabon	Minimum Estimate	2212 - Specialist medical practitioners	High Income and Upper Middle Income	Central	Medical Doctors
47	Pharmacist	236	246	256	1.14	8,741	Gabon	Minimum Estimate	2262 - Pharmacists	High Income and Upper Middle Income	Central	Pharmacist
48	Pharmacy Technician	222	240	257	1.15	8,695	Gabon	Minimum Estimate	3213 - Pharmaceutical technicians and assistants	High Income and Upper Middle Income	Central	Other Health Workers
49	Physician	183	203	225	1.00	9,956	Gabon	Minimum Estimate	2212 - Specialist medical practitioners	High Income and Upper Middle Income	Central	Medical Doctors
50	Physiotherapist	46	50	55	0.25	40,575	Gabon	Minimum Estimate	2264 - Physiotherapists	High Income and Upper Middle Income	Central	Other Health Workers
51	Plastic Surgeon	11	12	13	0.06	167,834	Gabon	Minimum Estimate	2212 - Specialist medical practitioners	High Income and Upper Middle Income	Central	Medical Doctors
52	Psychiatrist	92	101	110	0.50	20,180	Gabon	Minimum Estimate	2212 - Specialist medical practitioners	High Income and Upper Middle Income	Central	Medical Doctors
53	Radiation Oncologist	6	7	8	0.04	262,859	Gabon	Minimum Estimate	2212 - Specialist medical practitioners	High Income and Upper Middle Income	Central	Medical Doctors
54	Radiographer (Diagnostics and Therapy)	157	177	197	0.88	11,312	Gabon	Minimum Estimate	3211 - Medical imaging and therapeutic equipment technicians	High Income and Upper Middle Income	Central	Other Health Workers
55	Radiologist	49	56	62	0.28	35,752	Gabon	Minimum Estimate	2212 - Specialist medical practitioners	High Income and Upper Middle Income	Central	Medical Doctors
56	Registered General Nurse / State Certified Nurse	4,563	5,022	5,477	24.51	408	Gabon	Minimum Estimate	2221 - Nursing professionals	High Income and Upper Middle Income	Central	Nursing Personnel
57	Renal Nurse	285	326	370	1.66	6,015	Gabon	Minimum Estimate	2221 - Nursing professionals	High Income and Upper Middle Income	Central	Nursing Personnel
58	Respiratory Physician	11	12	13	0.06	173,970	Gabon	Minimum Estimate	2212 - Specialist medical practitioners	High Income and Upper Middle Income	Central	Medical Doctors
59	Rheumatologist	6	7	7	0.03	309,207	Gabon	Minimum Estimate	2212 - Specialist medical practitioners	High Income and Upper Middle Income	Central	Medical Doctors
60	Speech Therapist	17	18	20	0.09	112,371	Gabon	Minimum Estimate	2266 - Audiologists and speech therapists	High Income and Upper Middle Income	Central	Other Health Workers
61	Urologist	3	3	4	0.02	559,170	Gabon	Minimum Estimate	2212 - Specialist medical practitioners	High Income and Upper Middle Income	Central	Medical Doctors
1	Anaesthesiologist	16	18	20	0.08	119,689	Gambia	Minimum Estimate	2212 - Specialist medical practitioners	Low Income	West	Medical Doctors
2	Associate Nurse/Enrolled Nurse/Nursing Assistant	2,020	2,235	2,468	10.13	987	Gambia	Minimum Estimate	3221 - Nursing associate professionals	Low Income	West	Nursing Personnel
3	Audiologist	6	7	8	0.03	319,342	Gambia	Minimum Estimate	2266 - Audiologists and speech therapists	Low Income	West	Other Health Workers
4	Cardiologist	8	9	9	0.04	257,161	Gambia	Minimum Estimate	2212 - Specialist medical practitioners	Low Income	West	Medical Doctors
5	Cardiothoracic Surgeon	2	3	3	0.01	809,783	Gambia	Minimum Estimate	2212 - Specialist medical practitioners	Low Income	West	Medical Doctors
6	Clinical Officer/Physician Assistant	122	129	139	0.58	17,348	Gambia	Minimum Estimate	3256 - Medical assistants	Low Income	West	Other Health Workers

S/N	Health Professionals	2022	2026	2030	Density per 10,000 population	Required Population ratio (1 professional is to xxx population)	Name of Country	Modelling Scenario	ISCO-08 Match	Income Group Classification	Sub-Regional Grouping	SDG 3c Occupation
7	Clinical Pharmacist	44	49	55	0.23	44,343	Gambia	Minimum Estimate	2262 - Pharmacists	Low Income	West	Pharmacist
8	Clinical Psychologist	82	93	104	0.43	23,341	Gambia	Minimum Estimate	2634 - Psychologists	Low Income	West	Other Health Workers
9	Community health worker/Village health worker	1,343	1,449	1,552	6.37	1,570	Gambia	Minimum Estimate	3253 - Community health workers	Low Income	West	Other Health Workers
10	Dental Surgery Assistant	209	235	264	1.08	9,221	Gambia	Minimum Estimate	3251 - Dental assistants and therapists	Low Income	West	Other Health Workers
11	Dental Therapist	132	149	167	0.69	14,586	Gambia	Minimum Estimate	3251 - Dental assistants and therapists	Low Income	West	Other Health Workers
12	Dentist	127	143	161	0.66	15,080	Gambia	Minimum Estimate	2261 - Dentists	Low Income	West	Dentist
13	Dermatologist	7	8	8	0.03	292,018	Gambia	Minimum Estimate	2212 - Specialist medical practitioners	Low Income	West	Medical Doctors
14	Endocrinologist	6	7	8	0.03	298,459	Gambia	Minimum Estimate	2212 - Specialist medical practitioners	Low Income	West	Medical Doctors
15	ENT Surgeon	18	20	22	0.09	110,450	Gambia	Minimum Estimate	2212 - Specialist medical practitioners	Low Income	West	Medical Doctors
16	Environmental Health Officer	60	66	72	0.30	33,745	Gambia	Minimum Estimate	2263 - Environmental and occupational health and hygiene professionals	Low Income	West	Other Health Workers
17	Gastroenterologist	11	12	12	0.05	195,443	Gambia	Minimum Estimate	2212 - Specialist medical practitioners	Low Income	West	Medical Doctors
18	General Medical Practitioner (Generalist Doctor)	643	710	782	3.21	3,115	Gambia	Minimum Estimate	2211 - Generalist medical practitioners	Low Income	West	Medical Doctors
19	General Surgeon	15	17	20	0.08	123,370	Gambia	Minimum Estimate	2212 - Specialist medical practitioners	Low Income	West	Medical Doctors
20	Haematologist	9	10	11	0.05	220,234	Gambia	Minimum Estimate	2212 - Specialist medical practitioners	Low Income	West	Medical Doctors
21	Health Promoter/Health Educator	7	7	8	0.03	310,336	Gambia	Minimum Estimate	2269 - Health professionals not elsewhere classified	Low Income	West	Other Health Workers
22	Infectious Diseases Specialist	1	1	1	0.01	1,626,935	Gambia	Minimum Estimate	2212 - Specialist medical practitioners	Low Income	West	Medical Doctors
23	Intensive Care Nurse	46	50	55	0.22	44,701	Gambia	Minimum Estimate	2221 - Nursing professionals	Low Income	West	Nursing Personnel
24	Medical Laboratory Scientist	168	187	209	0.86	11,637	Gambia	Minimum Estimate	3212 - Medical and pathology laboratory technicians	Low Income	West	Other Health Workers
25	Medical Laboratory Technician	161	179	200	0.82	12,170	Gambia	Minimum Estimate	3212 - Medical and pathology laboratory technicians	Low Income	West	Other Health Workers
26	Medical Social Worker	63	66	69	0.28	35,396	Gambia	Minimum Estimate	1344 - Social welfare managers	Low Income	West	Other Health Workers
27	Mental Health Nurse	89	99	112	0.46	21,654	Gambia	Minimum Estimate	2221 - Nursing professionals	Low Income	West	Nursing Personnel
28	Midwife	1,363	1,506	1,660	6.81	1,468	Gambia	Minimum Estimate	2222 - Midwifery professionals	Low Income	West	Midwifery Personnel
29	Nephrologist	17	19	22	0.09	109,263	Gambia	Minimum Estimate	2212 - Specialist medical practitioners	Low Income	West	Medical Doctors
30	Neuro-Surgeon	5	5	6	0.03	392,833	Gambia	Minimum Estimate	2212 - Specialist medical practitioners	Low Income	West	Medical Doctors
31	Nurse Anaesthetist	28	33	38	0.16	64,013	Gambia	Minimum Estimate	2221 - Nursing professionals	Low Income	West	Nursing Personnel
32	Nutritionist	287	299	308	1.27	7,891	Gambia	Minimum Estimate	2265 - Dieticians and nutritionists	Low Income	West	Other Health Workers
33	Obstetrician & Gynaecologist	120	134	148	0.61	16,414	Gambia	Minimum Estimate	2212 - Specialist medical practitioners	Low Income	West	Medical Doctors
34	Occupational Therapist	34	39	44	0.18	55,809	Gambia	Minimum Estimate	2269 - Health professionals not elsewhere classified	Low Income	West	Other Health Workers
35	Oncology Nurse	12	14	16	0.07	152,350	Gambia	Minimum Estimate	2221 - Nursing professionals	Low Income	West	Nursing Personnel
36	Operating Theatre Nurse	187	210	239	0.98	10,164	Gambia	Minimum Estimate	2221 - Nursing professionals	Low Income	West	Nursing Personnel
37	Ophthalmic Nurse	21	23	25	0.10	95,464	Gambia	Minimum Estimate	2221 - Nursing professionals	Low Income	West	Nursing Personnel
38	Ophthalmologist	5	6	7	0.03	339,005	Gambia	Minimum Estimate	2212 - Specialist medical practitioners	Low Income	West	Medical Doctors
39	Optometrist	20	23	25	0.10	96,441	Gambia	Minimum Estimate	2267 - Optometrists and ophthalmic opticians	Low Income	West	Other Health Workers
40	Orthopaedic Nurse	17	19	22	0.09	113,153	Gambia	Minimum Estimate	2221 - Nursing professionals	Low Income	West	Nursing Personnel
41	Orthopaedic Surgeon	57	66	75	0.31	32,389	Gambia	Minimum Estimate	2212 - Specialist medical practitioners	Low Income	West	Medical Doctors
42	Orthopaedic Technologist	60	66	73	0.30	33,353	Gambia	Minimum Estimate	3214 - Medical and dental prosthetic technicians	Low Income	West	Other Health Workers
43	Paediatric Nurse	245	266	287	1.17	8,521	Gambia	Minimum Estimate	2221 - Nursing professionals	Low Income	West	Nursing Personnel
44	Paediatric Surgeon	6	6	7	0.03	368,779	Gambia	Minimum Estimate	2212 - Specialist medical practitioners	Low Income	West	Medical Doctors
45	Paediatrician	74	84	94	0.39	25,925	Gambia	Minimum Estimate	2212 - Specialist medical practitioners	Low Income	West	Medical Doctors
46	Pathologist	10	11	13	0.05	191,573	Gambia	Minimum Estimate	2212 - Specialist medical practitioners	Low Income	West	Medical Doctors
47	Pharmacist	98	102	106	0.44	22,903	Gambia	Minimum Estimate	2262 - Pharmacists	Low Income	West	Pharmacist
48	Pharmacy Technician	175	194	215	0.88	11,336	Gambia	Minimum Estimate	3213 - Pharmaceutical technicians and assistants	Low Income	West	Other Health Workers
49	Physician	178	200	224	0.92	10,859	Gambia	Minimum Estimate	2212 - Specialist medical practitioners	Low Income	West	Medical Doctors
50	Physiotherapist	43	48	53	0.22	45,718	Gambia	Minimum Estimate	2264 - Physiotherapists	Low Income	West	Other Health Workers
51	Plastic Surgeon	11	13	15	0.06	162,083	Gambia	Minimum Estimate	2212 - Specialist medical practitioners	Low Income	West	Medical Doctors
52	Psychiatrist	96	105	117	0.48	20,701	Gambia	Minimum Estimate	2212 - Specialist medical practitioners	Low Income	West	Medical Doctors
53	Radiation Oncologist	2	3	3	0.01	705,002	Gambia	Minimum Estimate	2212 - Specialist medical practitioners	Low Income	West	Medical Doctors
54	Radiographer (Diagnostics and Therapy)	129	146	167	0.69	14,549	Gambia	Minimum Estimate	3211 - Medical imaging and therapeutic equipment technicians	Low Income	West	Other Health Workers
55	Radiologist	38	43	49	0.20	49,750	Gambia	Minimum Estimate	2212 - Specialist medical practitioners	Low Income	West	Medical Doctors
56	Registered General Nurse / State Certified Nurse	3,978	4,386	4,846	19.93	502	Gambia	Minimum Estimate	2221 - Nursing professionals	Low Income	West	Nursing Personnel
57	Renal Nurse	206	236	275	1.13	8,822	Gambia	Minimum Estimate	2221 - Nursing professionals	Low Income	West	Nursing Personnel
58	Respiratory Physician	12	13	15	0.06	162,351	Gambia	Minimum Estimate	2212 - Specialist medical practitioners	Low Income	West	Medical Doctors
59	Rheumatologist	5	6	6	0.03	388,325	Gambia	Minimum Estimate	2212 - Specialist medical practitioners	Low Income	West	Medical Doctors
60	Speech Therapist	17	19	21	0.09	115,318	Gambia	Minimum Estimate	2266 - Audiologists and speech therapists	Low Income	West	Other Health Workers
61	Urologist	2	2	2	0.01	1,000,473	Gambia	Minimum Estimate	2212 - Specialist medical practitioners	Low Income	West	Medical Doctors
1	Anaesthesiologist	307	352	404	0.13	77,362	Ghana	Minimum Estimate	2212 - Specialist medical practitioners	Lower-middle Income	West	Medical Doctors
2	Associate Nurse/Enrolled Nurse/Nursing Assistant	29,827	32,339	35,103	11.22	891	Ghana	Minimum Estimate	3221 - Nursing associate professionals	Lower-middle Income	West	Nursing Personnel
3	Audiologist	77	84	91	0.03	342,339	Ghana	Minimum Estimate	2266 - Audiologists and speech therapists	Lower-middle Income	West	Other Health Workers
4	Cardiologist	112	126	141	0.05	221,923	Ghana	Minimum Estimate	2212 - Specialist medical practitioners	Lower-middle Income	West	Medical Doctors
5	Cardiothoracic Surgeon	58	66	76	0.02	409,521	Ghana	Minimum Estimate	2212 - Specialist medical practitioners	Lower-middle Income	West	Medical Doctors
6	Clinical Officer/Physician Assistant	2,994	3,042	3,164	1.03	9,751	Ghana	Minimum Estimate	3256 - Medical assistants	Lower-middle Income	West	Other Health Workers
7	Clinical Pharmacist	755	827	913	0.29	34,212	Ghana	Minimum Estimate	2262 - Pharmacists	Lower-middle Income	West	Pharmacist
8	Clinical Psychologist	1,803	1,964	2,149	0.69	14,550	Ghana	Minimum Estimate	2634 - Psychologists	Lower-middle Income	West	Other Health Workers
9	Community health worker/Village health worker	17,160	17,760	18,434	5.92	1,688	Ghana	Minimum Estimate	3253 - Community health workers	Lower-middle Income	West	Other Health Workers
10	Dental Surgery Assistant	2,491	2,769	3,069	0.98	10,198	Ghana	Minimum Estimate	3251 - Dental assistants and therapists	Lower-middle Income	West	Other Health Workers
11	Dental Therapist	1,582	1,761	1,953	0.62	16,023	Ghana	Minimum Estimate	3251 - Dental assistants and therapists	Lower-middle Income	West	Other Health Workers
12	Dentist	1,429	1,557	1,693	0.54	18,450	Ghana	Minimum Estimate	2261 - Dentists	Lower-middle Income	West	Dentist
13	Dermatologist	95	104	113	0.04	276,886	Ghana	Minimum Estimate	2212 - Specialist medical practitioners	Lower-middle Income	West	Medical Doctors
14	Endocrinologist	113	127	145	0.05	215,032	Ghana	Minimum Estimate	2212 - Specialist medical practitioners	Lower-middle Income	West	Medical Doctors
15	ENT Surgeon	257	286	322	0.10	96,549	Ghana	Minimum Estimate	2212 - Specialist medical practitioners	Lower-middle Income	West	Medical Doctors
16	Environmental Health Officer	772	832	894	0.29	35,060	Ghana	Minimum Estimate	2263 - Environmental and occupational health and hygiene professionals	Lower-middle Income	West	Other Health Workers
17	Gastroenterologist	327	322	321	0.10	96,460	Ghana	Minimum Estimate	2212 - Specialist medical practitioners	Lower-middle Income	West	Medical Doctors
18	General Medical Practitioner (Generalist Doctor)	9,305	10,002	10,770	3.45	2,899	Ghana	Minimum Estimate	2211 - Generalist medical practitioners	Lower-middle Income	West	Medical Doctors
19	General Surgeon	250	283	323	0.10	96,696	Ghana	Minimum Estimate	2212 - Specialist medical practitioners	Lower-middle Income	West	Medical Doctors
20	Haematologist	102	115	129	0.04	243,082	Ghana	Minimum Estimate	2212 - Specialist medical practitioners	Lower-middle Income	West	Medical Doctors
21	Health Promoter/Health Educator	157	156	158	0.05	195,664	Ghana	Minimum Estimate	2269 - Health professionals not elsewhere classified	Lower-middle Income	West	Other Health Workers
22	Infectious Diseases Specialist	27	29	30	0.01	1,026,824	Ghana	Minimum Estimate	2212 - Specialist medical practitioners	Lower-middle Income	West	Medical Doctors
23	Intensive Care Nurse	668	724	784	0.25	39,900	Ghana	Minimum Estimate	2221 - Nursing professionals	Lower-middle Income	West	Nursing Personnel
24	Medical Laboratory Scientist	2,908	3,117	3,380	1.08	9,223	Ghana	Minimum Estimate	3212 - Medical and pathology laboratory technicians	Lower-middle Income	West	Other Health Workers
25	Medical Laboratory Technician	2,735	2,793	2,899	0.94	10,682	Ghana	Minimum Estimate	3212 - Medical and pathology laboratory technicians	Lower-middle Income	West	Other Health Workers
26	Medical Social Worker	948	950	956	0.31	32,493	Ghana	Minimum Estimate	1344 - Social welfare managers	Lower-middle Income	West	Other Health Workers
27	Mental Health Nurse	1,135	1,243	1,380	0.44	22,647	Ghana	Minimum Estimate	2221 - Nursing professionals	Lower-middle Income	West	Nursing Personnel
28	Midwife	16,759	18,001	19,225	6.15	1,627	Ghana	Minimum Estimate	2222 - Midwifery professionals	Lower-middle Income	West	Midwifery Personnel
29	Nephrologist	287	327	378	0.12	82,575	Ghana	Minimum Estimate	2212 - Specialist medical practitioners	Lower-middle Income	West	Medical Doctors

S/N	Health Professionals	2022	2026	2030	Density per 10,000 population	Required Population ratio (1 professional is to xxx population)	Name of Country	Modelling Scenario	ISCO-08 Match	Income Group Classification	Sub-Regional Grouping	SDG 3c Occupation
30	Neuro-Surgeon	122	140	162	0.05	191,678	Ghana	Minimum Estimate	2212 - Specialist medical practitioners	Lower-middle Income	West	Medical Doctors
31	Nurse Anaesthetist	697	818	956	0.31	32,675	Ghana	Minimum Estimate	2221 - Nursing professionals	Lower-middle Income	West	Nursing Personnel
32	Nutritionist	2,775	2,823	2,863	0.92	10,885	Ghana	Minimum Estimate	2265 - Dieticians and nutritionists	Lower-middle Income	West	Other Health Workers
33	Obstetrician & Gynaecologist	1,728	1,894	2,077	0.66	15,068	Ghana	Minimum Estimate	2212 - Specialist medical practitioners	Lower-middle Income	West	Medical Doctors
34	Occupational Therapist	689	795	922	0.29	33,919	Ghana	Minimum Estimate	2269 - Health professionals not elsewhere classified	Lower-middle Income	West	Other Health Workers
35	Oncology Nurse	229	264	308	0.10	101,442	Ghana	Minimum Estimate	2221 - Nursing professionals	Lower-middle Income	West	Nursing Personnel
36	Operating Theatre Nurse	2,999	3,384	3,875	1.24	8,056	Ghana	Minimum Estimate	2221 - Nursing professionals	Lower-middle Income	West	Nursing Personnel
37	Ophthalmic Nurse	370	404	442	0.14	70,844	Ghana	Minimum Estimate	2221 - Nursing professionals	Lower-middle Income	West	Nursing Personnel
38	Ophthalmologist	90	100	113	0.04	276,826	Ghana	Minimum Estimate	2212 - Specialist medical practitioners	Lower-middle Income	West	Medical Doctors
39	Optometrist	288	315	346	0.11	90,547	Ghana	Minimum Estimate	2267 - Optometrists and ophthalmic opticians	Lower-middle Income	West	Other Health Workers
40	Orthopaedic Nurse	401	468	545	0.17	57,327	Ghana	Minimum Estimate	2221 - Nursing professionals	Lower-middle Income	West	Nursing Personnel
41	Orthopaedic Surgeon	1,016	1,167	1,342	0.43	23,309	Ghana	Minimum Estimate	2212 - Specialist medical practitioners	Lower-middle Income	West	Medical Doctors
42	Orthopaedic Technologist	971	1,082	1,204	0.38	26,006	Ghana	Minimum Estimate	3214 - Medical and dental prosthetic technicians	Lower-middle Income	West	Other Health Workers
43	Paediatric Nurse	2,547	2,748	2,936	0.94	10,660	Ghana	Minimum Estimate	2221 - Nursing professionals	Lower-middle Income	West	Nursing Personnel
44	Paediatric Surgeon	67	71	74	0.02	424,148	Ghana	Minimum Estimate	2212 - Specialist medical practitioners	Lower-middle Income	West	Medical Doctors
45	Paediatrician	953	1,058	1,176	0.38	26,597	Ghana	Minimum Estimate	2212 - Specialist medical practitioners	Lower-middle Income	West	Medical Doctors
46	Pathologist	193	217	246	0.08	127,093	Ghana	Minimum Estimate	2212 - Specialist medical practitioners	Lower-middle Income	West	Medical Doctors
47	Pharmacist	1,991	1,993	2,022	0.65	15,284	Ghana	Minimum Estimate	2262 - Pharmacists	Lower-middle Income	West	Pharmacist
48	Pharmacy Technician	2,785	3,006	3,258	1.04	9,580	Ghana	Minimum Estimate	3213 - Pharmaceutical technicians and assistants	Lower-middle Income	West	Other Health Workers
49	Physician	2,588	2,827	3,101	0.99	10,083	Ghana	Minimum Estimate	2212 - Specialist medical practitioners	Lower-middle Income	West	Medical Doctors
50	Physiotherapist	678	752	840	0.27	37,205	Ghana	Minimum Estimate	2264 - Physiotherapists	Lower-middle Income	West	Other Health Workers
51	Plastic Surgeon	178	203	232	0.07	135,167	Ghana	Minimum Estimate	2212 - Specialist medical practitioners	Lower-middle Income	West	Medical Doctors
52	Psychiatrist	1,196	1,297	1,421	0.45	21,985	Ghana	Minimum Estimate	2212 - Specialist medical practitioners	Lower-middle Income	West	Medical Doctors
53	Radiation Oncologist	64	76	91	0.03	343,526	Ghana	Minimum Estimate	2212 - Specialist medical practitioners	Lower-middle Income	West	Medical Doctors
54	Radiographer (Diagnostics and Therapy)	2,148	2,390	2,681	0.86	11,654	Ghana	Minimum Estimate	3211 - Medical imaging and therapeutic equipment technicians	Lower-middle Income	West	Other Health Workers
55	Radiologist	658	732	819	0.26	38,079	Ghana	Minimum Estimate	2212 - Specialist medical practitioners	Lower-middle Income	West	Medical Doctors
56	Registered General Nurse / State Certified Nurse	58,895	63,748	69,673	22.35	447	Ghana	Minimum Estimate	2221 - Nursing professionals	Lower-middle Income	West	Nursing Personnel
57	Renal Nurse	3,580	4,084	4,728	1.51	6,605	Ghana	Minimum Estimate	2221 - Nursing professionals	Lower-middle Income	West	Nursing Personnel
58	Respiratory Physician	129	145	163	0.05	191,813	Ghana	Minimum Estimate	2212 - Specialist medical practitioners	Lower-middle Income	West	Medical Doctors
59	Rheumatologist	75	83	92	0.03	338,970	Ghana	Minimum Estimate	2212 - Specialist medical practitioners	Lower-middle Income	West	Medical Doctors
60	Speech Therapist	244	265	294	0.09	105,656	Ghana	Minimum Estimate	2266 - Audiologists and speech therapists	Lower-middle Income	West	Other Health Workers
61	Urologist	33	39	47	0.02	664,490	Ghana	Minimum Estimate	2212 - Specialist medical practitioners	Lower-middle Income	West	Medical Doctors
1	Anaesthesiologist	96	106	117	0.09	113,037	Guinea	Minimum Estimate	2212 - Specialist medical practitioners	Low Income	West	Medical Doctors
2	Associate Nurse/Enrolled Nurse/Nursing Assistant	10,916	11,949	13,102	9.89	1,011	Guinea	Minimum Estimate	3221 - Nursing associate professionals	Low Income	West	Nursing Personnel
3	Audiologist	31	34	37	0.03	355,556	Guinea	Minimum Estimate	2266 - Audiologists and speech therapists	Low Income	West	Other Health Workers
4	Cardiologist	33	36	38	0.03	349,983	Guinea	Minimum Estimate	2212 - Specialist medical practitioners	Low Income	West	Medical Doctors
5	Cardiothoracic Surgeon	16	18	19	0.01	693,353	Guinea	Minimum Estimate	2212 - Specialist medical practitioners	Low Income	West	Medical Doctors
6	Clinical Officer/Physician Assistant	1,337	1,372	1,426	1.08	9,223	Guinea	Minimum Estimate	3256 - Medical assistants	Low Income	West	Other Health Workers
7	Clinical Pharmacist	268	292	323	0.24	40,924	Guinea	Minimum Estimate	2262 - Pharmacists	Low Income	West	Pharmacist
8	Clinical Psychologist	576	627	692	0.52	19,111	Guinea	Minimum Estimate	2634 - Psychologists	Low Income	West	Other Health Workers
9	Community health worker/Village health worker	9,840	10,483	11,135	8.41	1,189	Guinea	Minimum Estimate	3253 - Community health workers	Low Income	West	Other Health Workers
10	Dental Surgery Assistant	893	985	1,095	0.83	12,111	Guinea	Minimum Estimate	3251 - Dental assistants and therapists	Low Income	West	Other Health Workers
11	Dental Therapist	560	618	686	0.52	19,338	Guinea	Minimum Estimate	3251 - Dental assistants and therapists	Low Income	West	Other Health Workers
12	Dentist	631	694	784	0.59	16,904	Guinea	Minimum Estimate	2261 - Dentists	Low Income	West	Dentist
13	Dermatologist	35	38	42	0.03	318,690	Guinea	Minimum Estimate	2212 - Specialist medical practitioners	Low Income	West	Medical Doctors
14	Endocrinologist	28	32	39	0.03	336,829	Guinea	Minimum Estimate	2212 - Specialist medical practitioners	Low Income	West	Medical Doctors
15	ENT Surgeon	90	98	106	0.08	124,707	Guinea	Minimum Estimate	2212 - Specialist medical practitioners	Low Income	West	Medical Doctors
16	Environmental Health Officer	326	358	391	0.29	33,927	Guinea	Minimum Estimate	2263 - Environmental and occupational health and hygiene professionals	Low Income	West	Other Health Workers
17	Gastroenterologist	137	140	148	0.11	88,792	Guinea	Minimum Estimate	2212 - Specialist medical practitioners	Low Income	West	Medical Doctors
18	General Medical Practitioner (Generalist Doctor)	3,744	4,055	4,404	3.33	3,005	Guinea	Minimum Estimate	2211 - Generalist medical practitioners	Low Income	West	Medical Doctors
19	General Surgeon	80	89	100	0.08	132,150	Guinea	Minimum Estimate	2212 - Specialist medical practitioners	Low Income	West	Medical Doctors
20	Haematologist	40	45	50	0.04	265,718	Guinea	Minimum Estimate	2212 - Specialist medical practitioners	Low Income	West	Medical Doctors
21	Health Promoter/Health Educator	71	72	74	0.06	176,619	Guinea	Minimum Estimate	2269 - Health professionals not elsewhere classified	Low Income	West	Other Health Workers
22	Infectious Diseases Specialist	10	12	13	0.01	984,356	Guinea	Minimum Estimate	2212 - Specialist medical practitioners	Low Income	West	Medical Doctors
23	Intensive Care Nurse	284	310	338	0.26	39,205	Guinea	Minimum Estimate	2221 - Nursing professionals	Low Income	West	Nursing Personnel
24	Medical Laboratory Scientist	1,184	1,273	1,389	1.05	9,507	Guinea	Minimum Estimate	3212 - Medical and pathology laboratory technicians	Low Income	West	Other Health Workers
25	Medical Laboratory Technician	2,140	2,265	2,399	1.81	5,513	Guinea	Minimum Estimate	3212 - Medical and pathology laboratory technicians	Low Income	West	Other Health Workers
26	Medical Social Worker	453	475	503	0.38	26,266	Guinea	Minimum Estimate	1344 - Social welfare managers	Low Income	West	Other Health Workers
27	Mental Health Nurse	410	451	512	0.39	25,797	Guinea	Minimum Estimate	2221 - Nursing professionals	Low Income	West	Nursing Personnel
28	Midwife	8,117	8,894	9,808	7.40	1,352	Guinea	Minimum Estimate	2222 - Midwifery professionals	Low Income	West	Midwifery Personnel
29	Nephrologist	78	85	98	0.07	134,516	Guinea	Minimum Estimate	2212 - Specialist medical practitioners	Low Income	West	Medical Doctors
30	Neuro-Surgeon	31	35	39	0.03	342,512	Guinea	Minimum Estimate	2212 - Specialist medical practitioners	Low Income	West	Medical Doctors
31	Nurse Anaesthetist	184	203	224	0.17	59,178	Guinea	Minimum Estimate	2221 - Nursing professionals	Low Income	West	Nursing Personnel
32	Nutritionist	1,493	1,596	1,696	1.28	7,811	Guinea	Minimum Estimate	2265 - Dieticians and nutritionists	Low Income	West	Other Health Workers
33	Obstetrician & Gynaecologist	651	718	793	0.60	16,698	Guinea	Minimum Estimate	2212 - Specialist medical practitioners	Low Income	West	Medical Doctors
34	Occupational Therapist	205	227	253	0.19	52,402	Guinea	Minimum Estimate	2269 - Health professionals not elsewhere classified	Low Income	West	Other Health Workers
35	Oncology Nurse	61	67	76	0.06	173,791	Guinea	Minimum Estimate	2221 - Nursing professionals	Low Income	West	Nursing Personnel
36	Operating Theatre Nurse	969	1,062	1,198	0.91	11,033	Guinea	Minimum Estimate	2221 - Nursing professionals	Low Income	West	Nursing Personnel
37	Ophthalmic Nurse	106	116	129	0.10	102,526	Guinea	Minimum Estimate	2221 - Nursing professionals	Low Income	West	Nursing Personnel
38	Ophthalmologist	27	30	34	0.03	391,916	Guinea	Minimum Estimate	2212 - Specialist medical practitioners	Low Income	West	Medical Doctors
39	Optometrist	101	108	116	0.09	113,503	Guinea	Minimum Estimate	2267 - Optometrists and ophthalmic opticians	Low Income	West	Other Health Workers
40	Orthopaedic Nurse	111	124	137	0.10	96,630	Guinea	Minimum Estimate	2221 - Nursing professionals	Low Income	West	Nursing Personnel
41	Orthopaedic Surgeon	283	316	355	0.27	37,258	Guinea	Minimum Estimate	2212 - Specialist medical practitioners	Low Income	West	Medical Doctors
42	Orthopaedic Technologist	342	376	413	0.31	32,123	Guinea	Minimum Estimate	3214 - Medical and dental prosthetic technicians	Low Income	West	Other Health Workers
43	Paediatric Nurse	1,202	1,319	1,436	1.08	9,245	Guinea	Minimum Estimate	2221 - Nursing professionals	Low Income	West	Nursing Personnel
44	Paediatric Surgeon	37	41	44	0.03	304,858	Guinea	Minimum Estimate	2212 - Specialist medical practitioners	Low Income	West	Medical Doctors
45	Paediatrician	435	482	537	0.40	24,693	Guinea	Minimum Estimate	2212 - Specialist medical practitioners	Low Income	West	Medical Doctors
46	Pathologist	56	61	70	0.05	188,171	Guinea	Minimum Estimate	2212 - Specialist medical practitioners	Low Income	West	Medical Doctors
47	Pharmacist	696	697	705	0.54	18,614	Guinea	Minimum Estimate	2262 - Pharmacists	Low Income	West	Pharmacist
48	Pharmacy Technician	1,002	1,083	1,176	0.89	11,250	Guinea	Minimum Estimate	3213 - Pharmaceutical technicians and assistants	Low Income	West	Other Health Workers
49	Physician	1,031	1,130	1,247	0.94	10,617	Guinea	Minimum Estimate	2212 - Specialist medical practitioners	Low Income	West	Medical Doctors
50	Physiotherapist	242	266	292	0.22	45,442	Guinea	Minimum Estimate	2264 - Physiotherapists	Low Income	West	Other Health Workers
51	Plastic Surgeon	62	69	76	0.06	173,479	Guinea	Minimum Estimate	2212 - Specialist medical practitioners	Low Income	West	Medical Doctors
52	Psychiatrist	452	495	554	0.42	23,855	Guinea	Minimum Estimate	2212 - Specialist medical practitioners	Low Income	West	Medical Doctors

S/N	Health Professionals	2022	2026	2030	Density per 10,000 population	Required Population ratio (1 professional is to xxx population)	Name of Country	Modelling Scenario	ISCO-08 Match	Income Group Classification	Sub-Regional Grouping	SDG 3c Occupation
53	Radiation Oncologist	16	18	19	0.01	682,864	Guinea	Minimum Estimate	2212 - Specialist medical practitioners	Low Income	West	Medical Doctors
54	Radiographer (Diagnostics and Therapy)	688	758	853	0.65	15,502	Guinea	Minimum Estimate	3211 - Medical imaging and therapeutic equipment technicians	Low Income	West	Other Health Workers
55	Radiologist	219	241	269	0.20	49,108	Guinea	Minimum Estimate	2212 - Specialist medical practitioners	Low Income	West	Medical Doctors
56	Registered General Nurse / State Certified Nurse	21,362	23,074	25,207	19.07	524	Guinea	Minimum Estimate	2221 - Nursing professionals	Low Income	West	Nursing Personnel
57	Renal Nurse	959	1,055	1,210	0.92	10,902	Guinea	Minimum Estimate	2221 - Nursing professionals	Low Income	West	Nursing Personnel
58	Respiratory Physician	72	80	89	0.07	149,207	Guinea	Minimum Estimate	2212 - Specialist medical practitioners	Low Income	West	Medical Doctors
59	Rheumatologist	26	29	31	0.02	424,527	Guinea	Minimum Estimate	2212 - Specialist medical practitioners	Low Income	West	Medical Doctors
60	Speech Therapist	96	105	115	0.09	115,609	Guinea	Minimum Estimate	2266 - Audiologists and speech therapists	Low Income	West	Other Health Workers
61	Urologist	10	11	13	0.01	1,015,416	Guinea	Minimum Estimate	2212 - Specialist medical practitioners	Low Income	West	Medical Doctors
1	Anaesthesiologist	16	18	20	0.10	98,041	Guinea-Bissau	Minimum Estimate	2212 - Specialist medical practitioners	Low Income	West	Medical Doctors
2	Associate Nurse/Enrolled Nurse/Nursing Assistant	1,830	1,988	2,164	10.93	915	Guinea-Bissau	Minimum Estimate	3221 - Nursing associate professionals	Low Income	West	Nursing Personnel
3	Audiologist	5	5	6	0.03	343,596	Guinea-Bissau	Minimum Estimate	2266 - Audiologists and speech therapists	Low Income	West	Other Health Workers
4	Cardiologist	5	5	6	0.03	349,421	Guinea-Bissau	Minimum Estimate	2212 - Specialist medical practitioners	Low Income	West	Medical Doctors
5	Cardiothoracic Surgeon	3	3	3	0.02	572,703	Guinea-Bissau	Minimum Estimate	2212 - Specialist medical practitioners	Low Income	West	Medical Doctors
6	Clinical Officer/Physician Assistant	198	202	208	1.06	9,427	Guinea-Bissau	Minimum Estimate	3256 - Medical assistants	Low Income	West	Other Health Workers
7	Clinical Pharmacist	40	44	48	0.24	40,854	Guinea-Bissau	Minimum Estimate	2262 - Pharmacists	Low Income	West	Pharmacist
8	Clinical Psychologist	94	102	112	0.56	17,730	Guinea-Bissau	Minimum Estimate	2634 - Psychologists	Low Income	West	Other Health Workers
9	Community health worker/Village health worker	1,139	1,194	1,249	6.32	1,583	Guinea-Bissau	Minimum Estimate	3253 - Community health workers	Low Income	West	Other Health Workers
10	Dental Surgery Assistant	153	170	189	0.95	10,474	Guinea-Bissau	Minimum Estimate	3251 - Dental assistants and therapists	Low Income	West	Other Health Workers
11	Dental Therapist	95	106	118	0.60	16,770	Guinea-Bissau	Minimum Estimate	3251 - Dental assistants and therapists	Low Income	West	Other Health Workers
12	Dentist	115	126	141	0.72	13,976	Guinea-Bissau	Minimum Estimate	2261 - Dentists	Low Income	West	Dentist
13	Dermatologist	5	6	7	0.03	304,777	Guinea-Bissau	Minimum Estimate	2212 - Specialist medical practitioners	Low Income	West	Medical Doctors
14	Endocrinologist	5	6	7	0.04	280,052	Guinea-Bissau	Minimum Estimate	2212 - Specialist medical practitioners	Low Income	West	Medical Doctors
15	ENT Surgeon	14	15	17	0.08	118,358	Guinea-Bissau	Minimum Estimate	2212 - Specialist medical practitioners	Low Income	West	Medical Doctors
16	Environmental Health Officer	49	53	58	0.29	34,448	Guinea-Bissau	Minimum Estimate	2263 - Environmental and occupational health and hygiene professionals	Low Income	West	Other Health Workers
17	Gastroenterologist	19	19	19	0.10	101,918	Guinea-Bissau	Minimum Estimate	2212 - Specialist medical practitioners	Low Income	West	Medical Doctors
18	General Medical Practitioner (Generalist Doctor)	564	610	660	3.33	3,000	Guinea-Bissau	Minimum Estimate	2211 - Generalist medical practitioners	Low Income	West	Medical Doctors
19	General Surgeon	13	14	16	0.08	123,173	Guinea-Bissau	Minimum Estimate	2212 - Specialist medical practitioners	Low Income	West	Medical Doctors
20	Haematologist	6	6	7	0.03	287,875	Guinea-Bissau	Minimum Estimate	2212 - Specialist medical practitioners	Low Income	West	Medical Doctors
21	Health Promoter/Health Educator	12	12	12	0.06	162,452	Guinea-Bissau	Minimum Estimate	2269 - Health professionals not elsewhere classified	Low Income	West	Other Health Workers
22	Infectious Diseases Specialist	1	2	2	0.01	1,220,646	Guinea-Bissau	Minimum Estimate	2212 - Specialist medical practitioners	Low Income	West	Medical Doctors
23	Intensive Care Nurse	42	45	48	0.24	40,924	Guinea-Bissau	Minimum Estimate	2221 - Nursing professionals	Low Income	West	Nursing Personnel
24	Medical Laboratory Scientist	149	162	177	0.90	11,128	Guinea-Bissau	Minimum Estimate	3212 - Medical and pathology laboratory technicians	Low Income	West	Other Health Workers
25	Medical Laboratory Technician	127	135	144	0.73	13,708	Guinea-Bissau	Minimum Estimate	3212 - Medical and pathology laboratory technicians	Low Income	West	Other Health Workers
26	Medical Social Worker	61	62	64	0.32	30,873	Guinea-Bissau	Minimum Estimate	1344 - Social welfare managers	Low Income	West	Other Health Workers
27	Mental Health Nurse	64	70	78	0.40	25,145	Guinea-Bissau	Minimum Estimate	2221 - Nursing professionals	Low Income	West	Nursing Personnel
28	Midwife	1,097	1,185	1,284	6.48	1,543	Guinea-Bissau	Minimum Estimate	2222 - Midwifery professionals	Low Income	West	Midwifery Personnel
29	Nephrologist	14	15	18	0.09	111,359	Guinea-Bissau	Minimum Estimate	2212 - Specialist medical practitioners	Low Income	West	Medical Doctors
30	Neuro-Surgeon	6	6	7	0.04	277,522	Guinea-Bissau	Minimum Estimate	2212 - Specialist medical practitioners	Low Income	West	Medical Doctors
31	Nurse Anaesthetist	29	32	35	0.18	56,247	Guinea-Bissau	Minimum Estimate	2221 - Nursing professionals	Low Income	West	Nursing Personnel
32	Nutritionist	201	210	217	1.10	9,125	Guinea-Bissau	Minimum Estimate	2265 - Dieticians and nutritionists	Low Income	West	Other Health Workers
33	Obstetrician & Gynaecologist	102	112	123	0.62	16,149	Guinea-Bissau	Minimum Estimate	2212 - Specialist medical practitioners	Low Income	West	Medical Doctors
34	Occupational Therapist	36	40	45	0.23	43,913	Guinea-Bissau	Minimum Estimate	2269 - Health professionals not elsewhere classified	Low Income	West	Other Health Workers
35	Oncology Nurse	10	11	13	0.07	153,772	Guinea-Bissau	Minimum Estimate	2221 - Nursing professionals	Low Income	West	Nursing Personnel
36	Operating Theatre Nurse	160	177	201	1.02	9,830	Guinea-Bissau	Minimum Estimate	2221 - Nursing professionals	Low Income	West	Nursing Personnel
37	Ophthalmic Nurse	17	18	21	0.10	95,785	Guinea-Bissau	Minimum Estimate	2221 - Nursing professionals	Low Income	West	Nursing Personnel
38	Ophthalmologist	4	5	6	0.03	347,742	Guinea-Bissau	Minimum Estimate	2212 - Specialist medical practitioners	Low Income	West	Medical Doctors
39	Optometrist	16	18	20	0.10	98,978	Guinea-Bissau	Minimum Estimate	2267 - Optometrists and ophthalmic opticians	Low Income	West	Other Health Workers
40	Orthopaedic Nurse	20	23	25	0.13	78,463	Guinea-Bissau	Minimum Estimate	2221 - Nursing professionals	Low Income	West	Nursing Personnel
41	Orthopaedic Surgeon	49	56	64	0.32	31,059	Guinea-Bissau	Minimum Estimate	2212 - Specialist medical practitioners	Low Income	West	Medical Doctors
42	Orthopaedic Technologist	55	60	66	0.33	30,124	Guinea-Bissau	Minimum Estimate	3214 - Medical and dental prosthetic technicians	Low Income	West	Other Health Workers
43	Paediatric Nurse	174	188	200	1.01	9,904	Guinea-Bissau	Minimum Estimate	2221 - Nursing professionals	Low Income	West	Nursing Personnel
44	Paediatric Surgeon	5	5	6	0.03	352,931	Guinea-Bissau	Minimum Estimate	2212 - Specialist medical practitioners	Low Income	West	Medical Doctors
45	Paediatrician	58	64	71	0.36	27,924	Guinea-Bissau	Minimum Estimate	2212 - Specialist medical practitioners	Low Income	West	Medical Doctors
46	Pathologist	9	10	12	0.06	164,503	Guinea-Bissau	Minimum Estimate	2212 - Specialist medical practitioners	Low Income	West	Medical Doctors
47	Pharmacist	136	139	142	0.72	13,841	Guinea-Bissau	Minimum Estimate	2262 - Pharmacists	Low Income	West	Pharmacist
48	Pharmacy Technician	169	183	198	1.00	9,978	Guinea-Bissau	Minimum Estimate	3213 - Pharmaceutical technicians and assistants	Low Income	West	Other Health Workers
49	Physician	146	161	177	0.90	11,157	Guinea-Bissau	Minimum Estimate	2212 - Specialist medical practitioners	Low Income	West	Medical Doctors
50	Physiotherapist	38	42	46	0.23	42,903	Guinea-Bissau	Minimum Estimate	2264 - Physiotherapists	Low Income	West	Other Health Workers
51	Plastic Surgeon	9	10	12	0.06	171,408	Guinea-Bissau	Minimum Estimate	2212 - Specialist medical practitioners	Low Income	West	Medical Doctors
52	Psychiatrist	69	75	83	0.42	23,644	Guinea-Bissau	Minimum Estimate	2212 - Specialist medical practitioners	Low Income	West	Medical Doctors
53	Radiation Oncologist	2	3	3	0.01	680,915	Guinea-Bissau	Minimum Estimate	2212 - Specialist medical practitioners	Low Income	West	Medical Doctors
54	Radiographer (Diagnostics and Therapy)	111	124	139	0.70	14,190	Guinea-Bissau	Minimum Estimate	3211 - Medical imaging and therapeutic equipment technicians	Low Income	West	Other Health Workers
55	Radiologist	34	38	42	0.21	47,175	Guinea-Bissau	Minimum Estimate	2212 - Specialist medical practitioners	Low Income	West	Medical Doctors
56	Registered General Nurse / State Certified Nurse	5,036	5,196	5,408	27.51	363	Guinea-Bissau	Minimum Estimate	2221 - Nursing professionals	Low Income	West	Nursing Personnel
57	Renal Nurse	167	189	219	1.11	8,984	Guinea-Bissau	Minimum Estimate	2221 - Nursing professionals	Low Income	West	Nursing Personnel
58	Respiratory Physician	10	11	13	0.06	158,131	Guinea-Bissau	Minimum Estimate	2212 - Specialist medical practitioners	Low Income	West	Medical Doctors
59	Rheumatologist	4	4	5	0.02	405,526	Guinea-Bissau	Minimum Estimate	2212 - Specialist medical practitioners	Low Income	West	Medical Doctors
60	Speech Therapist	14	16	17	0.09	117,084	Guinea-Bissau	Minimum Estimate	2266 - Audiologists and speech therapists	Low Income	West	Other Health Workers
61	Urologist	1	1	2	0.01	1,189,617	Guinea-Bissau	Minimum Estimate	2212 - Specialist medical practitioners	Low Income	West	Medical Doctors
1	Anaesthesiologist	345	398	463	0.09	116,619	Kenya	Minimum Estimate	2212 - Specialist medical practitioners	Lower-middle Income	East	Medical Doctors
2	Associate Nurse/Enrolled Nurse/Nursing Assistant	49,688	53,594	58,305	10.79	927	Kenya	Minimum Estimate	3221 - Nursing associate professionals	Lower-middle Income	East	Nursing Personnel
3	Audiologist	148	163	179	0.03	303,425	Kenya	Minimum Estimate	2266 - Audiologists and speech therapists	Lower-middle Income	East	Other Health Workers
4	Cardiologist	190	217	247	0.05	218,975	Kenya	Minimum Estimate	2212 - Specialist medical practitioners	Lower-middle Income	East	Medical Doctors
5	Cardiothoracic Surgeon	55	62	70	0.01	777,401	Kenya	Minimum Estimate	2212 - Specialist medical practitioners	Lower-middle Income	East	Medical Doctors
6	Clinical Officer/Physician Assistant	7,471	8,128	9,004	1.67	5,981	Kenya	Minimum Estimate	3256 - Medical assistants	Lower-middle Income	East	Other Health Workers
7	Clinical Pharmacist	999	1,100	1,238	0.23	43,598	Kenya	Minimum Estimate	2262 - Pharmacists	Lower-middle Income	East	Pharmacist
8	Clinical Psychologist	2,763	2,988	3,270	0.60	16,542	Kenya	Minimum Estimate	2634 - Psychologists	Lower-middle Income	East	Other Health Workers
9	Community health worker/Village health worker	29,732	32,640	36,065	6.68	1,497	Kenya	Minimum Estimate	3253 - Community health workers	Lower-middle Income	East	Other Health Workers
10	Dental Surgery Assistant	3,354	3,731	4,189	0.77	12,958	Kenya	Minimum Estimate	3251 - Dental assistants and therapists	Lower-middle Income	East	Other Health Workers
11	Dental Therapist	2,104	2,345	2,634	0.49	20,605	Kenya	Minimum Estimate	3251 - Dental assistants and therapists	Lower-middle Income	East	Other Health Workers
12	Dentist	2,409	2,623	2,920	0.54	18,630	Kenya	Minimum Estimate	2261 - Dentists	Lower-middle Income	East	Dentist
13	Dermatologist	143	158	174	0.03	311,849	Kenya	Minimum Estimate	2212 - Specialist medical practitioners	Lower-middle Income	East	Medical Doctors
14	Endocrinologist	129	154	191	0.04	279,434	Kenya	Minimum Estimate	2212 - Specialist medical practitioners	Lower-middle Income	East	Medical Doctors

S/N	Health Professionals	2022	2026	2030	Density per 10,000 population	Required Population ratio (1 professional is to xxx population)	Name of Country	Modelling Scenario	ISCO-08 Match	Income Group Classification	Sub-Regional Grouping	SDG 3c Occupation
15	ENT Surgeon	473	532	611	0.11	88,342	Kenya	Minimum Estimate	2212 - Specialist medical practitioners	Lower-middle Income	East	Medical Doctors
16	Environmental Health Officer	1,335	1,444	1,558	0.29	34,791	Kenya	Minimum Estimate	2263 - Environmental and occupational health and hygiene professionals	Lower-middle Income	East	Other Health Workers
17	Gastroenterologist	216	224	245	0.05	219,173	Kenya	Minimum Estimate	2212 - Specialist medical practitioners	Lower-middle Income	East	Medical Doctors
18	General Medical Practitioner (Generalist Doctor)	16,652	18,229	20,088	3.71	2,693	Kenya	Minimum Estimate	2211 - Generalist medical practitioners	Lower-middle Income	East	Medical Doctors
19	General Surgeon	315	360	422	0.08	128,039	Kenya	Minimum Estimate	2212 - Specialist medical practitioners	Lower-middle Income	East	Medical Doctors
20	Haematologist	109	117	126	0.02	427,965	Kenya	Minimum Estimate	2212 - Specialist medical practitioners	Lower-middle Income	East	Medical Doctors
21	Health Promoter/Health Educator	391	421	460	0.09	117,225	Kenya	Minimum Estimate	2269 - Health professionals not elsewhere classified	Lower-middle Income	East	Other Health Workers
22	Infectious Diseases Specialist	49	54	60	0.01	898,299	Kenya	Minimum Estimate	2212 - Specialist medical practitioners	Lower-middle Income	East	Medical Doctors
23	Intensive Care Nurse	1,435	1,546	1,666	0.31	32,501	Kenya	Minimum Estimate	2221 - Nursing professionals	Lower-middle Income	East	Nursing Personnel
24	Medical Laboratory Scientist	4,766	5,285	5,981	1.11	9,006	Kenya	Minimum Estimate	3212 - Medical and pathology laboratory technicians	Lower-middle Income	East	Other Health Workers
25	Medical Laboratory Technician	5,118	5,814	6,715	1.25	7,998	Kenya	Minimum Estimate	3212 - Medical and pathology laboratory technicians	Lower-middle Income	East	Other Health Workers
26	Medical Social Worker	1,214	1,280	1,368	0.25	39,349	Kenya	Minimum Estimate	1344 - Social welfare managers	Lower-middle Income	East	Other Health Workers
27	Mental Health Nurse	2,216	2,446	2,812	0.52	19,185	Kenya	Minimum Estimate	2221 - Nursing professionals	Lower-middle Income	East	Nursing Personnel
28	Midwife	29,022	31,249	33,979	6.26	1,599	Kenya	Minimum Estimate	2222 - Midwifery professionals	Lower-middle Income	East	Midwifery Personnel
29	Nephrologist	397	457	550	0.10	97,641	Kenya	Minimum Estimate	2212 - Specialist medical practitioners	Lower-middle Income	East	Medical Doctors
30	Neuro-Surgeon	119	139	170	0.03	315,199	Kenya	Minimum Estimate	2212 - Specialist medical practitioners	Lower-middle Income	East	Medical Doctors
31	Nurse Anaesthetist	782	915	1,076	0.20	50,213	Kenya	Minimum Estimate	2221 - Nursing professionals	Lower-middle Income	East	Nursing Personnel
32	Nutritionist	5,066	5,264	5,463	1.01	9,903	Kenya	Minimum Estimate	2265 - Dieticians and nutritionists	Lower-middle Income	East	Other Health Workers
33	Obstetrician & Gynaecologist	2,998	3,292	3,650	0.68	14,808	Kenya	Minimum Estimate	2212 - Specialist medical practitioners	Lower-middle Income	East	Medical Doctors
34	Occupational Therapist	717	828	981	0.18	54,894	Kenya	Minimum Estimate	2269 - Health professionals not elsewhere classified	Lower-middle Income	East	Other Health Workers
35	Oncology Nurse	306	355	426	0.08	126,385	Kenya	Minimum Estimate	2221 - Nursing professionals	Lower-middle Income	East	Nursing Personnel
36	Operating Theatre Nurse	4,881	5,531	6,549	1.22	8,208	Kenya	Minimum Estimate	2221 - Nursing professionals	Lower-middle Income	East	Nursing Personnel
37	Ophthalmic Nurse	381	425	484	0.09	111,365	Kenya	Minimum Estimate	2221 - Nursing professionals	Lower-middle Income	East	Nursing Personnel
38	Ophthalmologist	124	143	171	0.03	314,555	Kenya	Minimum Estimate	2212 - Specialist medical practitioners	Lower-middle Income	East	Medical Doctors
39	Optometrist	402	459	531	0.10	101,847	Kenya	Minimum Estimate	2267 - Optometrists and ophthalmic opticians	Lower-middle Income	East	Other Health Workers
40	Orthopaedic Nurse	299	345	400	0.07	135,229	Kenya	Minimum Estimate	2221 - Nursing professionals	Lower-middle Income	East	Nursing Personnel
41	Orthopaedic Surgeon	1,454	1,684	1,973	0.37	27,360	Kenya	Minimum Estimate	2212 - Specialist medical practitioners	Lower-middle Income	East	Medical Doctors
42	Orthopaedic Technologist	1,293	1,417	1,551	0.29	34,925	Kenya	Minimum Estimate	3214 - Medical and dental prosthetic technicians	Lower-middle Income	East	Other Health Workers
43	Paediatric Nurse	3,819	4,063	4,315	0.80	12,568	Kenya	Minimum Estimate	2221 - Nursing professionals	Lower-middle Income	East	Nursing Personnel
44	Paediatric Surgeon	116	121	125	0.02	432,894	Kenya	Minimum Estimate	2212 - Specialist medical practitioners	Lower-middle Income	East	Medical Doctors
45	Paediatrician	1,566	1,717	1,910	0.35	28,294	Kenya	Minimum Estimate	2212 - Specialist medical practitioners	Lower-middle Income	East	Medical Doctors
46	Pathologist	380	427	501	0.09	107,409	Kenya	Minimum Estimate	2212 - Specialist medical practitioners	Lower-middle Income	East	Medical Doctors
47	Pharmacist	5,649	6,041	6,494	1.20	8,324	Kenya	Minimum Estimate	2262 - Pharmacists	Lower-middle Income	East	Pharmacist
48	Pharmacy Technician	4,720	5,144	5,638	1.04	9,596	Kenya	Minimum Estimate	3213 - Pharmaceutical technicians and assistants	Lower-middle Income	East	Other Health Workers
49	Physician	3,578	3,981	4,488	0.83	12,036	Kenya	Minimum Estimate	2212 - Specialist medical practitioners	Lower-middle Income	East	Medical Doctors
50	Physiotherapist	971	1,068	1,190	0.22	45,423	Kenya	Minimum Estimate	2264 - Physiotherapists	Lower-middle Income	East	Other Health Workers
51	Plastic Surgeon	359	418	487	0.09	111,119	Kenya	Minimum Estimate	2212 - Specialist medical practitioners	Lower-middle Income	East	Medical Doctors
52	Psychiatrist	2,172	2,372	2,679	0.50	20,161	Kenya	Minimum Estimate	2212 - Specialist medical practitioners	Lower-middle Income	East	Medical Doctors
53	Radiation Oncologist	86	102	122	0.02	439,789	Kenya	Minimum Estimate	2212 - Specialist medical practitioners	Lower-middle Income	East	Medical Doctors
54	Radiographer (Diagnostics and Therapy)	2,775	3,148	3,672	0.68	14,680	Kenya	Minimum Estimate	3211 - Medical imaging and therapeutic equipment technicians	Lower-middle Income	East	Other Health Workers
55	Radiologist	835	957	1,121	0.21	47,954	Kenya	Minimum Estimate	2212 - Specialist medical practitioners	Lower-middle Income	East	Medical Doctors
56	Registered General Nurse / State Certified Nurse	92,413	102,564	116,138	21.54	464	Kenya	Minimum Estimate	2221 - Nursing professionals	Lower-middle Income	East	Nursing Personnel
57	Renal Nurse	4,894	5,630	6,789	1.26	7,911	Kenya	Minimum Estimate	2221 - Nursing professionals	Lower-middle Income	East	Nursing Personnel
58	Respiratory Physician	257	283	314	0.06	172,325	Kenya	Minimum Estimate	2212 - Specialist medical practitioners	Lower-middle Income	East	Medical Doctors
59	Rheumatologist	125	139	155	0.03	349,207	Kenya	Minimum Estimate	2212 - Specialist medical practitioners	Lower-middle Income	East	Medical Doctors
60	Speech Therapist	391	424	469	0.09	115,060	Kenya	Minimum Estimate	2266 - Audiologists and speech therapists	Lower-middle Income	East	Other Health Workers
61	Urologist	50	60	76	0.01	699,219	Kenya	Minimum Estimate	2212 - Specialist medical practitioners	Lower-middle Income	East	Medical Doctors
1	Anaesthesiologist	20	22	24	0.11	88,702	Lesotho	Minimum Estimate	2212 - Specialist medical practitioners	Lower-middle Income	Southern	Medical Doctors
2	Associate Nurse/Enrolled Nurse/Nursing Assistant	2,494	2,642	2,804	13.01	769	Lesotho	Minimum Estimate	3221 - Nursing associate professionals	Lower-middle Income	Southern	Nursing Personnel
3	Audiologist	6	6	7	0.03	331,178	Lesotho	Minimum Estimate	2266 - Audiologists and speech therapists	Lower-middle Income	Southern	Other Health Workers
4	Cardiologist	10	10	11	0.05	203,617	Lesotho	Minimum Estimate	2212 - Specialist medical practitioners	Lower-middle Income	Southern	Medical Doctors
5	Cardiothoracic Surgeon	3	3	3	0.02	623,906	Lesotho	Minimum Estimate	2212 - Specialist medical practitioners	Lower-middle Income	Southern	Medical Doctors
6	Clinical Officer/Physician Assistant	298	316	338	1.57	6,370	Lesotho	Minimum Estimate	3256 - Medical assistants	Lower-middle Income	Southern	Other Health Workers
7	Clinical Pharmacist	51	55	59	0.28	36,272	Lesotho	Minimum Estimate	2262 - Pharmacists	Lower-middle Income	Southern	Pharmacist
8	Clinical Psychologist	105	111	117	0.54	18,426	Lesotho	Minimum Estimate	2634 - Psychologists	Lower-middle Income	Southern	Other Health Workers
9	Community health worker/Village health worker	1,193	1,234	1,271	5.91	1,691	Lesotho	Minimum Estimate	3253 - Community health workers	Lower-middle Income	Southern	Other Health Workers
10	Dental Surgery Assistant	178	190	203	0.94	10,647	Lesotho	Minimum Estimate	3251 - Dental assistants and therapists	Lower-middle Income	Southern	Other Health Workers
11	Dental Therapist	111	119	127	0.59	17,067	Lesotho	Minimum Estimate	3251 - Dental assistants and therapists	Lower-middle Income	Southern	Other Health Workers
12	Dentist	143	150	158	0.73	13,694	Lesotho	Minimum Estimate	2261 - Dentists	Lower-middle Income	Southern	Dentist
13	Dermatologist	6	7	7	0.03	302,768	Lesotho	Minimum Estimate	2212 - Specialist medical practitioners	Lower-middle Income	Southern	Medical Doctors
14	Endocrinologist	11	13	15	0.07	147,641	Lesotho	Minimum Estimate	2212 - Specialist medical practitioners	Lower-middle Income	Southern	Medical Doctors
15	ENT Surgeon	21	23	25	0.11	87,738	Lesotho	Minimum Estimate	2212 - Specialist medical practitioners	Lower-middle Income	Southern	Medical Doctors
16	Environmental Health Officer	53	56	58	0.27	37,336	Lesotho	Minimum Estimate	2263 - Environmental and occupational health and hygiene professionals	Lower-middle Income	Southern	Other Health Workers
17	Gastroenterologist	17	16	16	0.08	130,555	Lesotho	Minimum Estimate	2212 - Specialist medical practitioners	Lower-middle Income	Southern	Medical Doctors
18	General Medical Practitioner (Generalist Doctor)	751	794	841	3.91	2,561	Lesotho	Minimum Estimate	2211 - Generalist medical practitioners	Lower-middle Income	Southern	Medical Doctors
19	General Surgeon	25	26	28	0.13	75,667	Lesotho	Minimum Estimate	2212 - Specialist medical practitioners	Lower-middle Income	Southern	Medical Doctors
20	Haematologist	4	4	5	0.02	468,768	Lesotho	Minimum Estimate	2212 - Specialist medical practitioners	Lower-middle Income	Southern	Medical Doctors
21	Health Promoter/Health Educator	14	14	15	0.07	140,703	Lesotho	Minimum Estimate	2269 - Health professionals not elsewhere classified	Lower-middle Income	Southern	Other Health Workers
22	Infectious Diseases Specialist	2	2	3	0.01	819,665	Lesotho	Minimum Estimate	2212 - Specialist medical practitioners	Lower-middle Income	Southern	Medical Doctors
23	Intensive Care Nurse	48	50	53	0.25	40,791	Lesotho	Minimum Estimate	2221 - Nursing professionals	Lower-middle Income	Southern	Nursing Personnel
24	Medical Laboratory Scientist	354	382	416	1.93	5,171	Lesotho	Minimum Estimate	3212 - Medical and pathology laboratory technicians	Lower-middle Income	Southern	Other Health Workers
25	Medical Laboratory Technician	292	314	340	1.58	6,325	Lesotho	Minimum Estimate	3212 - Medical and pathology laboratory technicians	Lower-middle Income	Southern	Other Health Workers
26	Medical Social Worker	52	53	56	0.27	37,372	Lesotho	Minimum Estimate	1344 - Social welfare managers	Lower-middle Income	Southern	Other Health Workers
27	Mental Health Nurse	102	109	117	0.54	18,387	Lesotho	Minimum Estimate	2221 - Nursing professionals	Lower-middle Income	Southern	Nursing Personnel
28	Midwife	1,173	1,220	1,260	5.84	1,711	Lesotho	Minimum Estimate	2222 - Midwifery professionals	Lower-middle Income	Southern	Midwifery Personnel
29	Nephrologist	26	29	33	0.15	66,258	Lesotho	Minimum Estimate	2212 - Specialist medical practitioners	Lower-middle Income	Southern	Medical Doctors
30	Neuro-Surgeon	9	10	12	0.05	186,246	Lesotho	Minimum Estimate	2212 - Specialist medical practitioners	Lower-middle Income	Southern	Medical Doctors
31	Nurse Anaesthetist	51	58	65	0.30	33,052	Lesotho	Minimum Estimate	2221 - Nursing professionals	Lower-middle Income	Southern	Nursing Personnel
32	Nutritionist	145	143	140	0.66	15,259	Lesotho	Minimum Estimate	2265 - Dieticians and nutritionists	Lower-middle Income	Southern	Other Health Workers
33	Obstetrician & Gynaecologist	144	155	168	0.79	12,731	Lesotho	Minimum Estimate	2212 - Specialist medical practitioners	Lower-middle Income	Southern	Medical Doctors
34	Occupational Therapist	39	44	49	0.23	43,745	Lesotho	Minimum Estimate	2269 - Health professionals not elsewhere classified	Lower-middle Income	Southern	Other Health Workers
35	Oncology Nurse	20	22	25	0.12	85,195	Lesotho	Minimum Estimate	2221 - Nursing professionals	Lower-middle Income	Southern	Nursing Personnel
36	Operating Theatre Nurse	254	276	305	1.41	7,079	Lesotho	Minimum Estimate	2221 - Nursing professionals	Lower-middle Income	Southern	Nursing Personnel
37	Ophthalmic Nurse	28	30	33	0.15	65,494	Lesotho	Minimum Estimate	2221 - Nursing professionals	Lower-middle Income	Southern	Nursing Personnel

S/N	Health Professionals	2022	2026	2030	Density per 10,000 population	Required Population ratio (1 professional is to xxx population)	Name of Country	Modelling Scenario	ISCO-08 Match	Income Group Classification	Sub-Regional Grouping	SDG 3c Occupation
38	Ophthalmologist	10	11	12	0.06	178,523	Lesotho	Minimum Estimate	2212 - Specialist medical practitioners	Lower-middle Income	Southern	Medical Doctors
39	Optometrist	46	50	54	0.25	40,268	Lesotho	Minimum Estimate	2267 - Optometrists and ophthalmic opticians	Lower-middle Income	Southern	Other Health Workers
40	Orthopaedic Nurse	19	22	25	0.12	85,324	Lesotho	Minimum Estimate	2221 - Nursing professionals	Lower-middle Income	Southern	Nursing Personnel
41	Orthopaedic Surgeon	70	77	86	0.40	25,233	Lesotho	Minimum Estimate	2212 - Specialist medical practitioners	Lower-middle Income	Southern	Medical Doctors
42	Orthopaedic Technologist	58	62	66	0.31	32,547	Lesotho	Minimum Estimate	3214 - Medical and dental prosthetic technicians	Lower-middle Income	Southern	Other Health Workers
43	Paediatric Nurse	134	137	138	0.64	15,545	Lesotho	Minimum Estimate	2221 - Nursing professionals	Lower-middle Income	Southern	Nursing Personnel
44	Paediatric Surgeon	4	4	4	0.02	499,403	Lesotho	Minimum Estimate	2212 - Specialist medical practitioners	Lower-middle Income	Southern	Medical Doctors
45	Paediatrician	53	57	63	0.29	34,508	Lesotho	Minimum Estimate	2212 - Specialist medical practitioners	Lower-middle Income	Southern	Medical Doctors
46	Pathologist	22	24	26	0.12	81,690	Lesotho	Minimum Estimate	2212 - Specialist medical practitioners	Lower-middle Income	Southern	Medical Doctors
47	Pharmacist	151	152	153	0.72	13,924	Lesotho	Minimum Estimate	2262 - Pharmacists	Lower-middle Income	Southern	Pharmacist
48	Pharmacy Technician	183	193	204	0.95	10,529	Lesotho	Minimum Estimate	3213 - Pharmaceutical technicians and assistants	Lower-middle Income	Southern	Other Health Workers
49	Physician	176	189	203	0.94	10,611	Lesotho	Minimum Estimate	2212 - Specialist medical practitioners	Lower-middle Income	Southern	Medical Doctors
50	Physiotherapist	44	47	51	0.24	42,186	Lesotho	Minimum Estimate	2264 - Physiotherapists	Lower-middle Income	Southern	Other Health Workers
51	Plastic Surgeon	17	19	20	0.09	105,844	Lesotho	Minimum Estimate	2212 - Specialist medical practitioners	Lower-middle Income	Southern	Medical Doctors
52	Psychiatrist	96	101	108	0.50	19,956	Lesotho	Minimum Estimate	2212 - Specialist medical practitioners	Lower-middle Income	Southern	Medical Doctors
53	Radiation Oncologist	6	7	8	0.04	284,015	Lesotho	Minimum Estimate	2212 - Specialist medical practitioners	Lower-middle Income	Southern	Medical Doctors
54	Radiographer (Diagnostics and Therapy)	163	177	197	0.92	10,913	Lesotho	Minimum Estimate	3211 - Medical imaging and therapeutic equipment technicians	Lower-middle Income	Southern	Other Health Workers
55	Radiologist	50	56	65	0.31	32,269	Lesotho	Minimum Estimate	2212 - Specialist medical practitioners	Lower-middle Income	Southern	Medical Doctors
56	Registered General Nurse / State Certified Nurse	4,303	4,639	5,041	23.37	428	Lesotho	Minimum Estimate	2221 - Nursing professionals	Lower-middle Income	Southern	Nursing Personnel
57	Renal Nurse	319	356	406	1.88	5,331	Lesotho	Minimum Estimate	2221 - Nursing professionals	Lower-middle Income	Southern	Nursing Personnel
58	Respiratory Physician	8	9	9	0.04	230,855	Lesotho	Minimum Estimate	2212 - Specialist medical practitioners	Lower-middle Income	Southern	Medical Doctors
59	Rheumatologist	6	6	6	0.03	342,264	Lesotho	Minimum Estimate	2212 - Specialist medical practitioners	Lower-middle Income	Southern	Medical Doctors
60	Speech Therapist	17	18	20	0.09	109,994	Lesotho	Minimum Estimate	2266 - Audiologists and speech therapists	Lower-middle Income	Southern	Other Health Workers
61	Urologist	3	3	4	0.02	511,681	Lesotho	Minimum Estimate	2212 - Specialist medical practitioners	Lower-middle Income	Southern	Medical Doctors
1	Anaesthesiologist	35	40	46	0.09	110,126	Liberia	Minimum Estimate	2212 - Specialist medical practitioners	Low Income	West	Medical Doctors
2	Associate Nurse/Enrolled Nurse/Nursing Assistant	4,749	5,266	5,873	11.55	866	Liberia	Minimum Estimate	3221 - Nursing associate professionals	Low Income	West	Nursing Personnel
3	Audiologist	13	14	16	0.03	325,613	Liberia	Minimum Estimate	2266 - Audiologists and speech therapists	Low Income	West	Other Health Workers
4	Cardiologist	16	18	21	0.04	245,586	Liberia	Minimum Estimate	2212 - Specialist medical practitioners	Low Income	West	Medical Doctors
5	Cardiothoracic Surgeon	5	6	7	0.01	773,325	Liberia	Minimum Estimate	2212 - Specialist medical practitioners	Low Income	West	Medical Doctors
6	Clinical Officer/Physician Assistant	966	1,098	1,273	2.53	3,954	Liberia	Minimum Estimate	3256 - Medical assistants	Low Income	West	Other Health Workers
7	Clinical Pharmacist	101	110	123	0.24	41,465	Liberia	Minimum Estimate	2262 - Pharmacists	Low Income	West	Pharmacist
8	Clinical Psychologist	280	304	333	0.65	15,269	Liberia	Minimum Estimate	2634 - Psychologists	Low Income	West	Other Health Workers
9	Community health worker/Village health worker	4,234	4,956	5,858	11.61	861	Liberia	Minimum Estimate	3253 - Community health workers	Low Income	West	Other Health Workers
10	Dental Surgery Assistant	393	441	499	0.98	10,221	Liberia	Minimum Estimate	3251 - Dental assistants and therapists	Low Income	West	Other Health Workers
11	Dental Therapist	247	277	313	0.61	16,272	Liberia	Minimum Estimate	3251 - Dental assistants and therapists	Low Income	West	Other Health Workers
12	Dentist	277	307	347	0.68	14,722	Liberia	Minimum Estimate	2261 - Dentists	Low Income	West	Dentist
13	Dermatologist	14	16	17	0.03	292,921	Liberia	Minimum Estimate	2212 - Specialist medical practitioners	Low Income	West	Medical Doctors
14	Endocrinologist	16	19	23	0.05	219,840	Liberia	Minimum Estimate	2212 - Specialist medical practitioners	Low Income	West	Medical Doctors
15	ENT Surgeon	38	43	49	0.10	103,906	Liberia	Minimum Estimate	2212 - Specialist medical practitioners	Low Income	West	Medical Doctors
16	Environmental Health Officer	126	137	149	0.29	34,311	Liberia	Minimum Estimate	2263 - Environmental and occupational health and hygiene professionals	Low Income	West	Other Health Workers
17	Gastroenterologist	46	46	48	0.09	106,315	Liberia	Minimum Estimate	2212 - Specialist medical practitioners	Low Income	West	Medical Doctors
18	General Medical Practitioner (Generalist Doctor)	1,616	1,789	1,998	3.94	2,540	Liberia	Minimum Estimate	2211 - Generalist medical practitioners	Low Income	West	Medical Doctors
19	General Surgeon	30	35	40	0.08	127,387	Liberia	Minimum Estimate	2212 - Specialist medical practitioners	Low Income	West	Medical Doctors
20	Haematologist	8	8	9	0.02	576,182	Liberia	Minimum Estimate	2212 - Specialist medical practitioners	Low Income	West	Medical Doctors
21	Health Promoter/Health Educator	46	51	57	0.11	87,991	Liberia	Minimum Estimate	2269 - Health professionals not elsewhere classified	Low Income	West	Other Health Workers
22	Infectious Diseases Specialist	5	5	5	0.01	940,714	Liberia	Minimum Estimate	2212 - Specialist medical practitioners	Low Income	West	Medical Doctors
23	Intensive Care Nurse	99	106	115	0.23	44,217	Liberia	Minimum Estimate	2221 - Nursing professionals	Low Income	West	Nursing Personnel
24	Medical Laboratory Scientist	517	591	688	1.36	7,343	Liberia	Minimum Estimate	3212 - Medical and pathology laboratory technicians	Low Income	West	Other Health Workers
25	Medical Laboratory Technician	1,187	1,423	1,721	3.41	2,928	Liberia	Minimum Estimate	3212 - Medical and pathology laboratory technicians	Low Income	West	Other Health Workers
26	Medical Social Worker	132	134	138	0.27	36,555	Liberia	Minimum Estimate	1344 - Social welfare managers	Low Income	West	Other Health Workers
27	Mental Health Nurse	177	194	220	0.43	23,085	Liberia	Minimum Estimate	2221 - Nursing professionals	Low Income	West	Nursing Personnel
28	Midwife	3,260	3,509	3,793	7.44	1,345	Liberia	Minimum Estimate	2222 - Midwifery professionals	Low Income	West	Midwifery Personnel
29	Nephrologist	38	43	50	0.10	100,348	Liberia	Minimum Estimate	2212 - Specialist medical practitioners	Low Income	West	Medical Doctors
30	Neuro-Surgeon	15	17	20	0.04	253,222	Liberia	Minimum Estimate	2212 - Specialist medical practitioners	Low Income	West	Medical Doctors
31	Nurse Anaesthetist	63	73	84	0.17	60,250	Liberia	Minimum Estimate	2221 - Nursing professionals	Low Income	West	Nursing Personnel
32	Nutritionist	343	345	346	0.68	14,659	Liberia	Minimum Estimate	2265 - Dieticians and nutritionists	Low Income	West	Other Health Workers
33	Obstetrician & Gynaecologist	270	298	331	0.65	15,382	Liberia	Minimum Estimate	2212 - Specialist medical practitioners	Low Income	West	Medical Doctors
34	Occupational Therapist	77	89	104	0.20	48,913	Liberia	Minimum Estimate	2269 - Health professionals not elsewhere classified	Low Income	West	Other Health Workers
35	Oncology Nurse	27	31	36	0.07	139,632	Liberia	Minimum Estimate	2221 - Nursing professionals	Low Income	West	Nursing Personnel
36	Operating Theatre Nurse	449	511	597	1.18	8,500	Liberia	Minimum Estimate	2221 - Nursing professionals	Low Income	West	Nursing Personnel
37	Ophthalmic Nurse	45	50	57	0.11	88,796	Liberia	Minimum Estimate	2221 - Nursing professionals	Low Income	West	Nursing Personnel
38	Ophthalmologist	14	16	18	0.04	277,926	Liberia	Minimum Estimate	2212 - Specialist medical practitioners	Low Income	West	Medical Doctors
39	Optometrist	49	56	65	0.13	78,441	Liberia	Minimum Estimate	2267 - Optometrists and ophthalmic opticians	Low Income	West	Other Health Workers
40	Orthopaedic Nurse	36	43	50	0.10	102,410	Liberia	Minimum Estimate	2221 - Nursing professionals	Low Income	West	Nursing Personnel
41	Orthopaedic Surgeon	101	120	145	0.29	34,896	Liberia	Minimum Estimate	2212 - Specialist medical practitioners	Low Income	West	Medical Doctors
42	Orthopaedic Technologist	126	139	154	0.30	32,998	Liberia	Minimum Estimate	3214 - Medical and dental prosthetic technicians	Low Income	West	Other Health Workers
43	Paediatric Nurse	323	341	359	0.70	14,212	Liberia	Minimum Estimate	2221 - Nursing professionals	Low Income	West	Nursing Personnel
44	Paediatric Surgeon	11	11	11	0.02	447,094	Liberia	Minimum Estimate	2212 - Specialist medical practitioners	Low Income	West	Medical Doctors
45	Paediatrician	126	140	156	0.31	32,669	Liberia	Minimum Estimate	2212 - Specialist medical practitioners	Low Income	West	Medical Doctors
46	Pathologist	26	29	34	0.07	147,677	Liberia	Minimum Estimate	2212 - Specialist medical practitioners	Low Income	West	Medical Doctors
47	Pharmacist	465	472	482	0.96	10,470	Liberia	Minimum Estimate	2262 - Pharmacists	Low Income	West	Pharmacist
48	Pharmacy Technician	468	502	544	1.07	9,347	Liberia	Minimum Estimate	3213 - Pharmaceutical technicians and assistants	Low Income	West	Other Health Workers
49	Physician	434	485	547	1.08	9,286	Liberia	Minimum Estimate	2212 - Specialist medical practitioners	Low Income	West	Medical Doctors
50	Physiotherapist	94	104	116	0.23	43,762	Liberia	Minimum Estimate	2264 - Physiotherapists	Low Income	West	Other Health Workers
51	Plastic Surgeon	24	28	33	0.06	155,196	Liberia	Minimum Estimate	2212 - Specialist medical practitioners	Low Income	West	Medical Doctors
52	Psychiatrist	173	188	209	0.41	24,351	Liberia	Minimum Estimate	2212 - Specialist medical practitioners	Low Income	West	Medical Doctors
53	Radiation Oncologist	6	6	8	0.01	673,937	Liberia	Minimum Estimate	2212 - Specialist medical practitioners	Low Income	West	Medical Doctors
54	Radiographer (Diagnostics and Therapy)	297	334	384	0.76	13,234	Liberia	Minimum Estimate	3211 - Medical imaging and therapeutic equipment technicians	Low Income	West	Other Health Workers
55	Radiologist	88	99	114	0.22	44,510	Liberia	Minimum Estimate	2212 - Specialist medical practitioners	Low Income	West	Medical Doctors
56	Registered General Nurse / State Certified Nurse	9,121	9,964	11,070	21.83	458	Liberia	Minimum Estimate	2221 - Nursing professionals	Low Income	West	Nursing Personnel
57	Renal Nurse	469	535	631	1.25	8,031	Liberia	Minimum Estimate	2221 - Nursing professionals	Low Income	West	Nursing Personnel
58	Respiratory Physician	21	23	26	0.05	198,374	Liberia	Minimum Estimate	2212 - Specialist medical practitioners	Low Income	West	Medical Doctors
59	Rheumatologist	11	12	14	0.03	376,272	Liberia	Minimum Estimate	2212 - Specialist medical practitioners	Low Income	West	Medical Doctors
60	Speech Therapist	37	40	44	0.09	115,040	Liberia	Minimum Estimate	2266 - Audiologists and speech therapists	Low Income	West	Other Health Workers

S/N	Health Professionals	2022	2026	2030	Density per 10,000 population	Required Population ratio (1 professional is to xxx population)	Name of Country	Modelling Scenario	ISCO-08 Match	Income Group Classification	Sub-Regional Grouping	SDG 3c Occupation
61	Urologist	5	6	7	0.01	732,433	Liberia	Minimum Estimate	2212 - Specialist medical practitioners	Low Income	West	Medical Doctors
1	Anaesthesiologist	180	204	231	0.08	120,845	Madagascar	Minimum Estimate	2212 - Specialist medical practitioners	Low Income	Southern	Medical Doctors
2	Associate Nurse/Enrolled Nurse/Nursing Assistant	22,974	24,894	27,053	9.70	1,031	Madagascar	Minimum Estimate	3221 - Nursing associate professionals	Low Income	Southern	Nursing Personnel
3	Audiologist	74	82	92	0.03	304,754	Madagascar	Minimum Estimate	2266 - Audiologists and speech therapists	Low Income	Southern	Other Health Workers
4	Cardiologist	88	101	116	0.04	241,565	Madagascar	Minimum Estimate	2212 - Specialist medical practitioners	Low Income	Southern	Medical Doctors
5	Cardiothoracic Surgeon	33	37	41	0.01	676,469	Madagascar	Minimum Estimate	2212 - Specialist medical practitioners	Low Income	Southern	Medical Doctors
6	Clinical Officer/Physician Assistant	2,263	2,461	2,706	0.97	10,292	Madagascar	Minimum Estimate	3256 - Medical assistants	Low Income	Southern	Other Health Workers
7	Clinical Pharmacist	541	601	674	0.24	41,317	Madagascar	Minimum Estimate	2262 - Pharmacists	Low Income	Southern	Pharmacist
8	Clinical Psychologist	1,441	1,609	1,803	0.65	15,475	Madagascar	Minimum Estimate	2634 - Psychologists	Low Income	Southern	Other Health Workers
9	Community health worker/Village health worker	15,673	16,870	18,099	6.48	1,543	Madagascar	Minimum Estimate	3253 - Community health workers	Low Income	Southern	Other Health Workers
10	Dental Surgery Assistant	2,026	2,273	2,552	0.91	10,955	Madagascar	Minimum Estimate	3251 - Dental assistants and therapists	Low Income	Southern	Other Health Workers
11	Dental Therapist	1,249	1,402	1,574	0.56	17,759	Madagascar	Minimum Estimate	3251 - Dental assistants and therapists	Low Income	Southern	Other Health Workers
12	Dentist	1,807	2,018	2,267	0.81	12,333	Madagascar	Minimum Estimate	2261 - Dentists	Low Income	Southern	Dentist
13	Dermatologist	70	79	88	0.03	318,195	Madagascar	Minimum Estimate	2212 - Specialist medical practitioners	Low Income	Southern	Medical Doctors
14	Endocrinologist	62	74	91	0.03	305,556	Madagascar	Minimum Estimate	2212 - Specialist medical practitioners	Low Income	Southern	Medical Doctors
15	ENT Surgeon	214	243	279	0.10	99,907	Madagascar	Minimum Estimate	2212 - Specialist medical practitioners	Low Income	Southern	Medical Doctors
16	Environmental Health Officer	688	755	826	0.30	33,837	Madagascar	Minimum Estimate	2263 - Environmental and occupational health and hygiene professionals	Low Income	Southern	Other Health Workers
17	Gastroenterologist	131	135	142	0.05	194,473	Madagascar	Minimum Estimate	2212 - Specialist medical practitioners	Low Income	Southern	Medical Doctors
18	General Medical Practitioner (Generalist Doctor)	7,225	7,958	8,771	3.14	3,183	Madagascar	Minimum Estimate	2211 - Generalist medical practitioners	Low Income	Southern	Medical Doctors
19	General Surgeon	150	174	203	0.07	136,910	Madagascar	Minimum Estimate	2212 - Specialist medical practitioners	Low Income	Southern	Medical Doctors
20	Haematologist	78	87	97	0.03	289,225	Madagascar	Minimum Estimate	2212 - Specialist medical practitioners	Low Income	Southern	Medical Doctors
21	Health Promoter/Health Educator	113	122	133	0.05	209,536	Madagascar	Minimum Estimate	2269 - Health professionals not elsewhere classified	Low Income	Southern	Other Health Workers
22	Infectious Diseases Specialist	19	21	24	0.01	1,177,126	Madagascar	Minimum Estimate	2212 - Specialist medical practitioners	Low Income	Southern	Medical Doctors
23	Intensive Care Nurse	661	725	793	0.28	35,159	Madagascar	Minimum Estimate	2221 - Nursing professionals	Low Income	Southern	Nursing Personnel
24	Medical Laboratory Scientist	2,063	2,313	2,618	0.94	10,636	Madagascar	Minimum Estimate	3212 - Medical and pathology laboratory technicians	Low Income	Southern	Other Health Workers
25	Medical Laboratory Technician	2,119	2,338	2,592	0.93	10,751	Madagascar	Minimum Estimate	3212 - Medical and pathology laboratory technicians	Low Income	Southern	Other Health Workers
26	Medical Social Worker	742	766	797	0.29	34,777	Madagascar	Minimum Estimate	1344 - Social welfare managers	Low Income	Southern	Other Health Workers
27	Mental Health Nurse	1,126	1,259	1,438	0.52	19,371	Madagascar	Minimum Estimate	2221 - Nursing professionals	Low Income	Southern	Nursing Personnel
28	Midwife	14,848	16,218	17,705	6.33	1,579	Madagascar	Minimum Estimate	2222 - Midwifery professionals	Low Income	Southern	Midwifery Personnel
29	Nephrologist	170	196	231	0.08	120,594	Madagascar	Minimum Estimate	2212 - Specialist medical practitioners	Low Income	Southern	Medical Doctors
30	Neuro-Surgeon	66	76	89	0.03	311,339	Madagascar	Minimum Estimate	2212 - Specialist medical practitioners	Low Income	Southern	Medical Doctors
31	Nurse Anaesthetist	362	415	475	0.17	58,744	Madagascar	Minimum Estimate	2221 - Nursing professionals	Low Income	Southern	Nursing Personnel
32	Nutritionist	3,230	3,280	3,319	1.19	8,376	Madagascar	Minimum Estimate	2265 - Dieticians and nutritionists	Low Income	Southern	Other Health Workers
33	Obstetrician & Gynaecologist	1,498	1,669	1,865	0.67	14,943	Madagascar	Minimum Estimate	2212 - Specialist medical practitioners	Low Income	Southern	Medical Doctors
34	Occupational Therapist	382	433	495	0.18	56,282	Madagascar	Minimum Estimate	2269 - Health professionals not elsewhere classified	Low Income	Southern	Other Health Workers
35	Oncology Nurse	133	153	179	0.06	155,204	Madagascar	Minimum Estimate	2221 - Nursing professionals	Low Income	Southern	Nursing Personnel
36	Operating Theatre Nurse	2,243	2,537	2,938	1.06	9,474	Madagascar	Minimum Estimate	2221 - Nursing professionals	Low Income	Southern	Nursing Personnel
37	Ophthalmic Nurse	223	252	288	0.10	96,918	Madagascar	Minimum Estimate	2221 - Nursing professionals	Low Income	Southern	Nursing Personnel
38	Ophthalmologist	61	71	83	0.03	334,319	Madagascar	Minimum Estimate	2212 - Specialist medical practitioners	Low Income	Southern	Medical Doctors
39	Optometrist	253	292	337	0.12	82,787	Madagascar	Minimum Estimate	2267 - Optometrists and ophthalmic opticians	Low Income	Southern	Other Health Workers
40	Orthopaedic Nurse	191	217	247	0.09	112,984	Madagascar	Minimum Estimate	2221 - Nursing professionals	Low Income	Southern	Nursing Personnel
41	Orthopaedic Surgeon	617	711	824	0.30	33,846	Madagascar	Minimum Estimate	2212 - Specialist medical practitioners	Low Income	Southern	Medical Doctors
42	Orthopaedic Technologist	706	784	867	0.31	32,243	Madagascar	Minimum Estimate	3214 - Medical and dental prosthetic technicians	Low Income	Southern	Other Health Workers
43	Paediatric Nurse	2,529	2,714	2,892	1.03	9,665	Madagascar	Minimum Estimate	2221 - Nursing professionals	Low Income	Southern	Nursing Personnel
44	Paediatric Surgeon	66	71	76	0.03	369,308	Madagascar	Minimum Estimate	2212 - Specialist medical practitioners	Low Income	Southern	Medical Doctors
45	Paediatrician	1,229	1,327	1,436	0.51	19,442	Madagascar	Minimum Estimate	2212 - Specialist medical practitioners	Low Income	Southern	Medical Doctors
46	Pathologist	171	193	225	0.08	124,038	Madagascar	Minimum Estimate	2212 - Specialist medical practitioners	Low Income	Southern	Medical Doctors
47	Pharmacist	1,628	1,721	1,825	0.66	15,252	Madagascar	Minimum Estimate	2262 - Pharmacists	Low Income	Southern	Pharmacist
48	Pharmacy Technician	1,939	2,140	2,366	0.85	11,793	Madagascar	Minimum Estimate	3213 - Pharmaceutical technicians and assistants	Low Income	Southern	Other Health Workers
49	Physician	1,805	2,000	2,228	0.80	12,514	Madagascar	Minimum Estimate	2212 - Specialist medical practitioners	Low Income	Southern	Medical Doctors
50	Physiotherapist	516	573	640	0.23	43,607	Madagascar	Minimum Estimate	2264 - Physiotherapists	Low Income	Southern	Other Health Workers
51	Plastic Surgeon	308	356	410	0.15	68,048	Madagascar	Minimum Estimate	2212 - Specialist medical practitioners	Low Income	Southern	Medical Doctors
52	Psychiatrist	1,099	1,205	1,343	0.48	20,735	Madagascar	Minimum Estimate	2212 - Specialist medical practitioners	Low Income	Southern	Medical Doctors
53	Radiation Oncologist	35	40	47	0.02	590,598	Madagascar	Minimum Estimate	2212 - Specialist medical practitioners	Low Income	Southern	Medical Doctors
54	Radiographer (Diagnostics and Therapy)	1,292	1,470	1,696	0.61	16,404	Madagascar	Minimum Estimate	3211 - Medical imaging and therapeutic equipment technicians	Low Income	Southern	Other Health Workers
55	Radiologist	393	451	524	0.19	52,742	Madagascar	Minimum Estimate	2212 - Specialist medical practitioners	Low Income	Southern	Medical Doctors
56	Registered General Nurse / State Certified Nurse	41,274	45,985	51,592	18.50	540	Madagascar	Minimum Estimate	2221 - Nursing professionals	Low Income	Southern	Nursing Personnel
57	Renal Nurse	2,127	2,446	2,883	1.04	9,651	Madagascar	Minimum Estimate	2221 - Nursing professionals	Low Income	Southern	Nursing Personnel
58	Respiratory Physician	274	292	311	0.11	89,611	Madagascar	Minimum Estimate	2212 - Specialist medical practitioners	Low Income	Southern	Medical Doctors
59	Rheumatologist	59	67	75	0.03	372,507	Madagascar	Minimum Estimate	2212 - Specialist medical practitioners	Low Income	Southern	Medical Doctors
60	Speech Therapist	214	236	263	0.09	105,762	Madagascar	Minimum Estimate	2266 - Audiologists and speech therapists	Low Income	Southern	Other Health Workers
61	Urologist	19	23	29	0.01	933,380	Madagascar	Minimum Estimate	2212 - Specialist medical practitioners	Low Income	Southern	Medical Doctors
1	Anaesthesiologist	106	120	136	0.07	141,504	Malawi	Minimum Estimate	2212 - Specialist medical practitioners	Low Income	Southern	Medical Doctors
2	Associate Nurse/Enrolled Nurse/Nursing Assistant	17,019	18,769	20,819	10.81	925	Malawi	Minimum Estimate	3221 - Nursing associate professionals	Low Income	Southern	Nursing Personnel
3	Audiologist	50	56	63	0.03	307,230	Malawi	Minimum Estimate	2266 - Audiologists and speech therapists	Low Income	Southern	Other Health Workers
4	Cardiologist	56	64	72	0.04	266,913	Malawi	Minimum Estimate	2212 - Specialist medical practitioners	Low Income	Southern	Medical Doctors
5	Cardiothoracic Surgeon	19	21	23	0.01	841,199	Malawi	Minimum Estimate	2212 - Specialist medical practitioners	Low Income	Southern	Medical Doctors
6	Clinical Officer/Physician Assistant	1,443	1,517	1,638	0.86	11,672	Malawi	Minimum Estimate	3256 - Medical assistants	Low Income	Southern	Other Health Workers
7	Clinical Pharmacist	379	424	483	0.25	39,755	Malawi	Minimum Estimate	2262 - Pharmacists	Low Income	Southern	Pharmacist
8	Clinical Psychologist	1,251	1,395	1,570	0.82	12,269	Malawi	Minimum Estimate	2634 - Psychologists	Low Income	Southern	Other Health Workers
9	Community health worker/Village health worker	10,566	11,345	12,263	6.37	1,570	Malawi	Minimum Estimate	3253 - Community health workers	Low Income	Southern	Other Health Workers
10	Dental Surgery Assistant	1,199	1,355	1,559	0.81	12,388	Malawi	Minimum Estimate	3251 - Dental assistants and therapists	Low Income	Southern	Other Health Workers
11	Dental Therapist	741	837	962	0.50	20,071	Malawi	Minimum Estimate	3251 - Dental assistants and therapists	Low Income	Southern	Other Health Workers
12	Dentist	1,049	1,188	1,390	0.72	13,901	Malawi	Minimum Estimate	2261 - Dentists	Low Income	Southern	Dentist
13	Dermatologist	48	55	61	0.03	313,954	Malawi	Minimum Estimate	2212 - Specialist medical practitioners	Low Income	Southern	Medical Doctors
14	Endocrinologist	44	51	62	0.03	309,590	Malawi	Minimum Estimate	2212 - Specialist medical practitioners	Low Income	Southern	Medical Doctors
15	ENT Surgeon	138	157	178	0.09	108,003	Malawi	Minimum Estimate	2212 - Specialist medical practitioners	Low Income	Southern	Medical Doctors
16	Environmental Health Officer	475	526	580	0.30	33,311	Malawi	Minimum Estimate	2263 - Environmental and occupational health and hygiene professionals	Low Income	Southern	Other Health Workers
17	Gastroenterologist	130	132	140	0.07	136,658	Malawi	Minimum Estimate	2212 - Specialist medical practitioners	Low Income	Southern	Medical Doctors
18	General Medical Practitioner (Generalist Doctor)	4,903	5,446	6,095	3.16	3,161	Malawi	Minimum Estimate	2211 - Generalist medical practitioners	Low Income	Southern	Medical Doctors
19	General Surgeon	109	127	152	0.08	126,394	Malawi	Minimum Estimate	2212 - Specialist medical practitioners	Low Income	Southern	Medical Doctors
20	Haematologist	60	69	78	0.04	247,583	Malawi	Minimum Estimate	2212 - Specialist medical practitioners	Low Income	Southern	Medical Doctors
21	Health Promoter/Health Educator	72	77	83	0.04	229,927	Malawi	Minimum Estimate	2269 - Health professionals not elsewhere classified	Low Income	Southern	Other Health Workers
22	Infectious Diseases Specialist	13	14	15	0.01	1,243,462	Malawi	Minimum Estimate	2212 - Specialist medical practitioners	Low Income	Southern	Medical Doctors

S/N	Health Professionals	2022	2026	2030	Density per 10,000 population	Required Population ratio (1 professional is to xxx population)	Name of Country	Modelling Scenario	ISCO-08 Match	Income Group Classification	Sub-Regional Grouping	SDG 3c Occupation
23	Intensive Care Nurse	420	467	518	0.27	37,260	Malawi	Minimum Estimate	2221 - Nursing professionals	Low Income	Southern	Nursing Personnel
24	Medical Laboratory Scientist	1,743	1,912	2,138	1.12	8,967	Malawi	Minimum Estimate	3212 - Medical and pathology laboratory technicians	Low Income	Southern	Other Health Workers
25	Medical Laboratory Technician	1,727	1,811	1,938	1.01	9,852	Malawi	Minimum Estimate	3212 - Medical and pathology laboratory technicians	Low Income	Southern	Other Health Workers
26	Medical Social Worker	458	485	524	0.27	36,492	Malawi	Minimum Estimate	1344 - Social welfare managers	Low Income	Southern	Other Health Workers
27	Mental Health Nurse	655	748	889	0.46	21,518	Malawi	Minimum Estimate	2221 - Nursing professionals	Low Income	Southern	Nursing Personnel
28	Midwife	10,149	11,181	12,492	6.46	1,548	Malawi	Minimum Estimate	2222 - Midwifery professionals	Low Income	Southern	Midwifery Personnel
29	Nephrologist	138	159	192	0.10	99,552	Malawi	Minimum Estimate	2212 - Specialist medical practitioners	Low Income	Southern	Medical Doctors
30	Neuro-Surgeon	37	42	48	0.03	398,066	Malawi	Minimum Estimate	2212 - Specialist medical practitioners	Low Income	Southern	Medical Doctors
31	Nurse Anaesthetist	249	284	325	0.17	59,379	Malawi	Minimum Estimate	2221 - Nursing professionals	Low Income	Southern	Nursing Personnel
32	Nutritionist	1,554	1,670	1,791	0.93	10,774	Malawi	Minimum Estimate	2265 - Dieticians and nutritionists	Low Income	Southern	Other Health Workers
33	Obstetrician & Gynaecologist	1,057	1,181	1,329	0.69	14,468	Malawi	Minimum Estimate	2212 - Specialist medical practitioners	Low Income	Southern	Medical Doctors
34	Occupational Therapist	210	238	275	0.14	69,939	Malawi	Minimum Estimate	2269 - Health professionals not elsewhere classified	Low Income	Southern	Other Health Workers
35	Oncology Nurse	107	123	147	0.08	130,495	Malawi	Minimum Estimate	2221 - Nursing professionals	Low Income	Southern	Nursing Personnel
36	Operating Theatre Nurse	1,370	1,560	1,840	0.96	10,394	Malawi	Minimum Estimate	2221 - Nursing professionals	Low Income	Southern	Nursing Personnel
37	Ophthalmic Nurse	169	189	214	0.11	89,892	Malawi	Minimum Estimate	2221 - Nursing professionals	Low Income	Southern	Nursing Personnel
38	Ophthalmologist	42	47	55	0.03	348,316	Malawi	Minimum Estimate	2212 - Specialist medical practitioners	Low Income	Southern	Medical Doctors
39	Optometrist	164	185	210	0.11	91,603	Malawi	Minimum Estimate	2267 - Optometrists and ophthalmic opticians	Low Income	Southern	Other Health Workers
40	Orthopaedic Nurse	95	108	122	0.06	158,365	Malawi	Minimum Estimate	2221 - Nursing professionals	Low Income	Southern	Nursing Personnel
41	Orthopaedic Surgeon	398	461	538	0.28	35,738	Malawi	Minimum Estimate	2212 - Specialist medical practitioners	Low Income	Southern	Medical Doctors
42	Orthopaedic Technologist	447	497	551	0.29	35,046	Malawi	Minimum Estimate	3214 - Medical and dental prosthetic technicians	Low Income	Southern	Other Health Workers
43	Paediatric Nurse	1,633	1,812	2,010	1.04	9,620	Malawi	Minimum Estimate	2221 - Nursing professionals	Low Income	Southern	Nursing Personnel
44	Paediatric Surgeon	46	50	53	0.03	366,275	Malawi	Minimum Estimate	2212 - Specialist medical practitioners	Low Income	Southern	Medical Doctors
45	Paediatrician	661	750	860	0.45	22,381	Malawi	Minimum Estimate	2212 - Specialist medical practitioners	Low Income	Southern	Medical Doctors
46	Pathologist	116	129	149	0.08	128,191	Malawi	Minimum Estimate	2212 - Specialist medical practitioners	Low Income	Southern	Medical Doctors
47	Pharmacist	718	765	824	0.43	23,286	Malawi	Minimum Estimate	2262 - Pharmacists	Low Income	Southern	Pharmacist
48	Pharmacy Technician	1,181	1,321	1,489	0.77	12,937	Malawi	Minimum Estimate	3213 - Pharmaceutical technicians and assistants	Low Income	Southern	Other Health Workers
49	Physician	1,182	1,329	1,514	0.79	12,702	Malawi	Minimum Estimate	2212 - Specialist medical practitioners	Low Income	Southern	Medical Doctors
50	Physiotherapist	327	365	407	0.21	47,308	Malawi	Minimum Estimate	2264 - Physiotherapists	Low Income	Southern	Other Health Workers
51	Plastic Surgeon	178	209	245	0.13	78,746	Malawi	Minimum Estimate	2212 - Specialist medical practitioners	Low Income	Southern	Medical Doctors
52	Psychiatrist	619	699	815	0.43	23,527	Malawi	Minimum Estimate	2212 - Specialist medical practitioners	Low Income	Southern	Medical Doctors
53	Radiation Oncologist	27	31	35	0.02	545,861	Malawi	Minimum Estimate	2212 - Specialist medical practitioners	Low Income	Southern	Medical Doctors
54	Radiographer (Diagnostics and Therapy)	905	1,032	1,211	0.63	15,813	Malawi	Minimum Estimate	3211 - Medical imaging and therapeutic equipment technicians	Low Income	Southern	Other Health Workers
55	Radiologist	266	304	358	0.19	53,119	Malawi	Minimum Estimate	2212 - Specialist medical practitioners	Low Income	Southern	Medical Doctors
56	Registered General Nurse / State Certified Nurse	30,464	34,148	38,855	20.21	495	Malawi	Minimum Estimate	2221 - Nursing professionals	Low Income	Southern	Nursing Personnel
57	Renal Nurse	1,734	1,996	2,404	1.26	7,937	Malawi	Minimum Estimate	2221 - Nursing professionals	Low Income	Southern	Nursing Personnel
58	Respiratory Physician	119	134	152	0.08	126,540	Malawi	Minimum Estimate	2212 - Specialist medical practitioners	Low Income	Southern	Medical Doctors
59	Rheumatologist	39	44	50	0.03	386,767	Malawi	Minimum Estimate	2212 - Specialist medical practitioners	Low Income	Southern	Medical Doctors
60	Speech Therapist	141	156	173	0.09	111,234	Malawi	Minimum Estimate	2266 - Audiologists and speech therapists	Low Income	Southern	Other Health Workers
61	Urologist	11	14	17	0.01	1,104,929	Malawi	Minimum Estimate	2212 - Specialist medical practitioners	Low Income	Southern	Medical Doctors
1	Anaesthesiologist	118	135	154	0.08	132,426	Mali	Minimum Estimate	2212 - Specialist medical practitioners	Low Income	West	Medical Doctors
2	Associate Nurse/Enrolled Nurse/Nursing Assistant	17,090	19,339	21,900	10.74	931	Mali	Minimum Estimate	3221 - Nursing associate professionals	Low Income	West	Nursing Personnel
3	Audiologist	50	56	63	0.03	322,873	Mali	Minimum Estimate	2266 - Audiologists and speech therapists	Low Income	West	Other Health Workers
4	Cardiologist	50	56	63	0.03	323,896	Mali	Minimum Estimate	2212 - Specialist medical practitioners	Low Income	West	Medical Doctors
5	Cardiothoracic Surgeon	19	21	24	0.01	862,184	Mali	Minimum Estimate	2212 - Specialist medical practitioners	Low Income	West	Medical Doctors
6	Clinical Officer/Physician Assistant	1,102	1,168	1,260	0.62	16,062	Mali	Minimum Estimate	3256 - Medical assistants	Low Income	West	Other Health Workers
7	Clinical Pharmacist	372	422	483	0.24	42,104	Mali	Minimum Estimate	2262 - Pharmacists	Low Income	West	Pharmacist
8	Clinical Psychologist	918	1,023	1,144	0.56	17,800	Mali	Minimum Estimate	2634 - Psychologists	Low Income	West	Other Health Workers
9	Community health worker/Village health worker	13,277	14,953	16,838	8.26	1,210	Mali	Minimum Estimate	3253 - Community health workers	Low Income	West	Other Health Workers
10	Dental Surgery Assistant	1,313	1,486	1,693	0.83	12,049	Mali	Minimum Estimate	3251 - Dental assistants and therapists	Low Income	West	Other Health Workers
11	Dental Therapist	823	932	1,061	0.52	19,234	Mali	Minimum Estimate	3251 - Dental assistants and therapists	Low Income	West	Other Health Workers
12	Dentist	914	1,036	1,199	0.59	16,996	Mali	Minimum Estimate	2261 - Dentists	Low Income	West	Dentist
13	Dermatologist	51	57	65	0.03	316,057	Mali	Minimum Estimate	2212 - Specialist medical practitioners	Low Income	West	Medical Doctors
14	Endocrinologist	37	43	53	0.03	380,297	Mali	Minimum Estimate	2212 - Specialist medical practitioners	Low Income	West	Medical Doctors
15	ENT Surgeon	135	153	172	0.08	118,510	Mali	Minimum Estimate	2212 - Specialist medical practitioners	Low Income	West	Medical Doctors
16	Environmental Health Officer	503	567	637	0.31	32,043	Mali	Minimum Estimate	2263 - Environmental and occupational health and hygiene professionals	Low Income	West	Other Health Workers
17	Gastroenterologist	114	123	136	0.07	148,332	Mali	Minimum Estimate	2212 - Specialist medical practitioners	Low Income	West	Medical Doctors
18	General Medical Practitioner (Generalist Doctor)	5,352	6,034	6,814	3.34	2,991	Mali	Minimum Estimate	2211 - Generalist medical practitioners	Low Income	West	Medical Doctors
19	General Surgeon	125	145	172	0.08	117,679	Mali	Minimum Estimate	2212 - Specialist medical practitioners	Low Income	West	Medical Doctors
20	Haematologist	85	101	120	0.06	169,793	Mali	Minimum Estimate	2212 - Specialist medical practitioners	Low Income	West	Medical Doctors
21	Health Promoter/Health Educator	67	71	76	0.04	266,340	Mali	Minimum Estimate	2269 - Health professionals not elsewhere classified	Low Income	West	Other Health Workers
22	Infectious Diseases Specialist	13	14	16	0.01	1,245,541	Mali	Minimum Estimate	2212 - Specialist medical practitioners	Low Income	West	Medical Doctors
23	Intensive Care Nurse	493	556	625	0.31	32,662	Mali	Minimum Estimate	2221 - Nursing professionals	Low Income	West	Nursing Personnel
24	Medical Laboratory Scientist	1,408	1,592	1,819	0.90	11,158	Mali	Minimum Estimate	3212 - Medical and pathology laboratory technicians	Low Income	West	Other Health Workers
25	Medical Laboratory Technician	2,079	2,387	2,748	1.35	7,404	Mali	Minimum Estimate	3212 - Medical and pathology laboratory technicians	Low Income	West	Other Health Workers
26	Medical Social Worker	730	801	879	0.43	23,197	Mali	Minimum Estimate	1344 - Social welfare managers	Low Income	West	Other Health Workers
27	Mental Health Nurse	567	646	753	0.37	26,892	Mali	Minimum Estimate	2221 - Nursing professionals	Low Income	West	Nursing Personnel
28	Midwife	13,552	15,465	17,718	8.69	1,151	Mali	Minimum Estimate	2222 - Midwifery professionals	Low Income	West	Midwifery Personnel
29	Nephrologist	102	117	137	0.07	147,097	Mali	Minimum Estimate	2212 - Specialist medical practitioners	Low Income	West	Medical Doctors
30	Neuro-Surgeon	37	43	49	0.02	415,757	Mali	Minimum Estimate	2212 - Specialist medical practitioners	Low Income	West	Medical Doctors
31	Nurse Anaesthetist	212	243	277	0.14	73,537	Mali	Minimum Estimate	2221 - Nursing professionals	Low Income	West	Nursing Personnel
32	Nutritionist	3,516	3,891	4,293	2.10	4,757	Mali	Minimum Estimate	2265 - Dieticians and nutritionists	Low Income	West	Other Health Workers
33	Obstetrician & Gynaecologist	1,001	1,130	1,277	0.63	15,965	Mali	Minimum Estimate	2212 - Specialist medical practitioners	Low Income	West	Medical Doctors
34	Occupational Therapist	254	293	339	0.17	59,963	Mali	Minimum Estimate	2269 - Health professionals not elsewhere classified	Low Income	West	Other Health Workers
35	Oncology Nurse	77	88	103	0.05	196,380	Mali	Minimum Estimate	2221 - Nursing professionals	Low Income	West	Nursing Personnel
36	Operating Theatre Nurse	1,266	1,456	1,708	0.84	11,844	Mali	Minimum Estimate	2221 - Nursing professionals	Low Income	West	Nursing Personnel
37	Ophthalmic Nurse	143	162	185	0.09	109,846	Mali	Minimum Estimate	2221 - Nursing professionals	Low Income	West	Nursing Personnel
38	Ophthalmologist	39	44	51	0.02	400,526	Mali	Minimum Estimate	2212 - Specialist medical practitioners	Low Income	West	Medical Doctors
39	Optometrist	148	165	184	0.09	110,707	Mali	Minimum Estimate	2267 - Optometrists and ophthalmic opticians	Low Income	West	Other Health Workers
40	Orthopaedic Nurse	139	161	185	0.09	110,102	Mali	Minimum Estimate	2221 - Nursing professionals	Low Income	West	Nursing Personnel
41	Orthopaedic Surgeon	346	401	468	0.23	43,417	Mali	Minimum Estimate	2212 - Specialist medical practitioners	Low Income	West	Medical Doctors
42	Orthopaedic Technologist	493	558	629	0.31	32,469	Mali	Minimum Estimate	3214 - Medical and dental prosthetic technicians	Low Income	West	Other Health Workers
43	Paediatric Nurse	2,407	2,758	3,143	1.54	6,490	Mali	Minimum Estimate	2221 - Nursing professionals	Low Income	West	Nursing Personnel
44	Paediatric Surgeon	63	71	80	0.04	256,983	Mali	Minimum Estimate	2212 - Specialist medical practitioners	Low Income	West	Medical Doctors
45	Paediatrician	567	660	770	0.38	26,410	Mali	Minimum Estimate	2212 - Specialist medical practitioners	Low Income	West	Medical Doctors

S/N	Health Professionals	2022	2026	2030	Density per 10,000 population	Required Population ratio (1 professional is to xxx population)	Name of Country	Modelling Scenario	ISCO-08 Match	Income Group Classification	Sub-Regional Grouping	SDG 3c Occupation
46	Pathologist	88	98	113	0.06	178,501	Mali	Minimum Estimate	2212 - Specialist medical practitioners	Low Income	West	Medical Doctors
47	Pharmacist	879	920	968	0.48	20,975	Mali	Minimum Estimate	2262 - Pharmacists	Low Income	West	Pharmacist
48	Pharmacy Technician	1,393	1,566	1,763	0.86	11,562	Mali	Minimum Estimate	3213 - Pharmaceutical technicians and assistants	Low Income	West	Other Health Workers
49	Physician	1,503	1,713	1,958	0.96	10,399	Mali	Minimum Estimate	2212 - Specialist medical practitioners	Low Income	West	Medical Doctors
50	Physiotherapist	349	396	448	0.22	45,566	Mali	Minimum Estimate	2264 - Physiotherapists	Low Income	West	Other Health Workers
51	Plastic Surgeon	86	98	112	0.06	181,722	Mali	Minimum Estimate	2212 - Specialist medical practitioners	Low Income	West	Medical Doctors
52	Psychiatrist	723	815	931	0.46	21,811	Mali	Minimum Estimate	2212 - Specialist medical practitioners	Low Income	West	Medical Doctors
53	Radiation Oncologist	19	22	25	0.01	800,105	Mali	Minimum Estimate	2212 - Specialist medical practitioners	Low Income	West	Medical Doctors
54	Radiographer (Diagnostics and Therapy)	901	1,028	1,188	0.58	17,094	Mali	Minimum Estimate	3211 - Medical imaging and therapeutic equipment technicians	Low Income	West	Other Health Workers
55	Radiologist	281	320	368	0.18	55,198	Mali	Minimum Estimate	2212 - Specialist medical practitioners	Low Income	West	Medical Doctors
56	Registered General Nurse / State Certified Nurse	38,619	42,798	47,696	23.46	426	Mali	Minimum Estimate	2221 - Nursing professionals	Low Income	West	Nursing Personnel
57	Renal Nurse	1,261	1,439	1,690	0.84	11,941	Mali	Minimum Estimate	2221 - Nursing professionals	Low Income	West	Nursing Personnel
58	Respiratory Physician	80	94	110	0.05	185,067	Mali	Minimum Estimate	2212 - Specialist medical practitioners	Low Income	West	Medical Doctors
59	Rheumatologist	40	45	51	0.02	402,850	Mali	Minimum Estimate	2212 - Specialist medical practitioners	Low Income	West	Medical Doctors
60	Speech Therapist	143	162	182	0.09	112,173	Mali	Minimum Estimate	2266 - Audiologists and speech therapists	Low Income	West	Other Health Workers
61	Urologist	13	15	18	0.01	1,101,510	Mali	Minimum Estimate	2212 - Specialist medical practitioners	Low Income	West	Medical Doctors
1	Anaesthesiologist	43	48	54	0.12	86,944	Mauritania	Minimum Estimate	2212 - Specialist medical practitioners	Lower-middle Income	West	Medical Doctors
2	Associate Nurse/Enrolled Nurse/Nursing Assistant	4,377	4,894	5,499	11.80	847	Mauritania	Minimum Estimate	3221 - Nursing associate professionals	Lower-middle Income	West	Nursing Personnel
3	Audiologist	12	13	15	0.03	320,608	Mauritania	Minimum Estimate	2266 - Audiologists and speech therapists	Lower-middle Income	West	Other Health Workers
4	Cardiologist	18	20	23	0.05	200,728	Mauritania	Minimum Estimate	2212 - Specialist medical practitioners	Lower-middle Income	West	Medical Doctors
5	Cardiothoracic Surgeon	7	7	8	0.02	566,606	Mauritania	Minimum Estimate	2212 - Specialist medical practitioners	Lower-middle Income	West	Medical Doctors
6	Clinical Officer/Physician Assistant	296	339	419	0.97	10,289	Mauritania	Minimum Estimate	3256 - Medical assistants	Lower-middle Income	West	Other Health Workers
7	Clinical Pharmacist	104	115	128	0.27	36,435	Mauritania	Minimum Estimate	2262 - Pharmacists	Lower-middle Income	West	Pharmacist
8	Clinical Psychologist	245	270	300	0.64	15,618	Mauritania	Minimum Estimate	2634 - Psychologists	Lower-middle Income	West	Other Health Workers
9	Community health worker/Village health worker	2,276	2,569	3,046	6.93	1,443	Mauritania	Minimum Estimate	3253 - Community health workers	Lower-middle Income	West	Other Health Workers
10	Dental Surgery Assistant	367	416	471	1.01	9,941	Mauritania	Minimum Estimate	3251 - Dental assistants and therapists	Lower-middle Income	West	Other Health Workers
11	Dental Therapist	231	262	297	0.63	15,800	Mauritania	Minimum Estimate	3251 - Dental assistants and therapists	Lower-middle Income	West	Other Health Workers
12	Dentist	249	281	318	0.68	14,723	Mauritania	Minimum Estimate	2261 - Dentists	Lower-middle Income	West	Dentist
13	Dermatologist	13	15	17	0.04	279,374	Mauritania	Minimum Estimate	2212 - Specialist medical practitioners	Lower-middle Income	West	Medical Doctors
14	Endocrinologist	11	13	17	0.04	279,648	Mauritania	Minimum Estimate	2212 - Specialist medical practitioners	Lower-middle Income	West	Medical Doctors
15	ENT Surgeon	38	43	49	0.11	94,978	Mauritania	Minimum Estimate	2212 - Specialist medical practitioners	Lower-middle Income	West	Medical Doctors
16	Environmental Health Officer	115	128	142	0.30	33,067	Mauritania	Minimum Estimate	2263 - Environmental and occupational health and hygiene professionals	Lower-middle Income	West	Other Health Workers
17	Gastroenterologist	60	61	63	0.14	73,663	Mauritania	Minimum Estimate	2212 - Specialist medical practitioners	Lower-middle Income	West	Medical Doctors
18	General Medical Practitioner (Generalist Doctor)	1,258	1,406	1,586	3.43	2,917	Mauritania	Minimum Estimate	2211 - Generalist medical practitioners	Lower-middle Income	West	Medical Doctors
19	General Surgeon	33	38	45	0.10	104,790	Mauritania	Minimum Estimate	2212 - Specialist medical practitioners	Lower-middle Income	West	Medical Doctors
20	Haematologist	9	10	11	0.02	440,599	Mauritania	Minimum Estimate	2212 - Specialist medical practitioners	Lower-middle Income	West	Medical Doctors
21	Health Promoter/Health Educator	18	20	23	0.05	190,987	Mauritania	Minimum Estimate	2269 - Health professionals not elsewhere classified	Lower-middle Income	West	Other Health Workers
22	Infectious Diseases Specialist	4	4	4	0.01	1,082,871	Mauritania	Minimum Estimate	2212 - Specialist medical practitioners	Lower-middle Income	West	Medical Doctors
23	Intensive Care Nurse	107	117	128	0.27	36,564	Mauritania	Minimum Estimate	2221 - Nursing professionals	Lower-middle Income	West	Nursing Personnel
24	Medical Laboratory Scientist	353	400	467	1.03	9,719	Mauritania	Minimum Estimate	3212 - Medical and pathology laboratory technicians	Lower-middle Income	West	Other Health Workers
25	Medical Laboratory Technician	330	412	560	1.34	7,482	Mauritania	Minimum Estimate	3212 - Medical and pathology laboratory technicians	Lower-middle Income	West	Other Health Workers
26	Medical Social Worker	175	181	188	0.40	24,906	Mauritania	Minimum Estimate	1344 - Social welfare managers	Lower-middle Income	West	Other Health Workers
27	Mental Health Nurse	151	171	195	0.42	23,894	Mauritania	Minimum Estimate	2221 - Nursing professionals	Lower-middle Income	West	Nursing Personnel
28	Midwife	2,521	2,789	3,075	6.56	1,525	Mauritania	Minimum Estimate	2222 - Midwifery professionals	Lower-middle Income	West	Midwifery Personnel
29	Nephrologist	41	48	57	0.12	82,376	Mauritania	Minimum Estimate	2212 - Specialist medical practitioners	Lower-middle Income	West	Medical Doctors
30	Neuro-Surgeon	16	18	20	0.04	228,992	Mauritania	Minimum Estimate	2212 - Specialist medical practitioners	Lower-middle Income	West	Medical Doctors
31	Nurse Anaesthetist	78	91	105	0.22	44,509	Mauritania	Minimum Estimate	2221 - Nursing professionals	Lower-middle Income	West	Nursing Personnel
32	Nutritionist	510	531	550	1.18	8,506	Mauritania	Minimum Estimate	2265 - Dieticians and nutritionists	Lower-middle Income	West	Other Health Workers
33	Obstetrician & Gynaecologist	242	270	302	0.65	15,500	Mauritania	Minimum Estimate	2212 - Specialist medical practitioners	Lower-middle Income	West	Medical Doctors
34	Occupational Therapist	108	122	138	0.29	34,027	Mauritania	Minimum Estimate	2269 - Health professionals not elsewhere classified	Lower-middle Income	West	Other Health Workers
35	Oncology Nurse	30	35	41	0.09	112,901	Mauritania	Minimum Estimate	2221 - Nursing professionals	Lower-middle Income	West	Nursing Personnel
36	Operating Theatre Nurse	447	507	582	1.25	8,023	Mauritania	Minimum Estimate	2221 - Nursing professionals	Lower-middle Income	West	Nursing Personnel
37	Ophthalmic Nurse	42	48	55	0.12	84,579	Mauritania	Minimum Estimate	2221 - Nursing professionals	Lower-middle Income	West	Nursing Personnel
38	Ophthalmologist	11	13	16	0.03	295,842	Mauritania	Minimum Estimate	2212 - Specialist medical practitioners	Lower-middle Income	West	Medical Doctors
39	Optometrist	44	50	58	0.12	80,965	Mauritania	Minimum Estimate	2267 - Optometrists and ophthalmic opticians	Lower-middle Income	West	Other Health Workers
40	Orthopaedic Nurse	66	74	83	0.18	56,830	Mauritania	Minimum Estimate	2221 - Nursing professionals	Lower-middle Income	West	Nursing Personnel
41	Orthopaedic Surgeon	146	168	195	0.42	24,044	Mauritania	Minimum Estimate	2212 - Specialist medical practitioners	Lower-middle Income	West	Medical Doctors
42	Orthopaedic Technologist	141	157	174	0.37	26,938	Mauritania	Minimum Estimate	3214 - Medical and dental prosthetic technicians	Lower-middle Income	West	Other Health Workers
43	Paediatric Nurse	377	406	434	0.92	10,812	Mauritania	Minimum Estimate	2221 - Nursing professionals	Lower-middle Income	West	Nursing Personnel
44	Paediatric Surgeon	11	11	12	0.03	384,614	Mauritania	Minimum Estimate	2212 - Specialist medical practitioners	Lower-middle Income	West	Medical Doctors
45	Paediatrician	173	194	219	0.47	21,346	Mauritania	Minimum Estimate	2212 - Specialist medical practitioners	Lower-middle Income	West	Medical Doctors
46	Pathologist	24	27	31	0.07	151,119	Mauritania	Minimum Estimate	2212 - Specialist medical practitioners	Lower-middle Income	West	Medical Doctors
47	Pharmacist	201	207	216	0.47	21,484	Mauritania	Minimum Estimate	2262 - Pharmacists	Lower-middle Income	West	Pharmacist
48	Pharmacy Technician	389	431	479	1.02	9,761	Mauritania	Minimum Estimate	3213 - Pharmaceutical technicians and assistants	Lower-middle Income	West	Other Health Workers
49	Physician	379	428	488	1.05	9,528	Mauritania	Minimum Estimate	2212 - Specialist medical practitioners	Lower-middle Income	West	Medical Doctors
50	Physiotherapist	100	112	126	0.27	37,276	Mauritania	Minimum Estimate	2264 - Physiotherapists	Lower-middle Income	West	Other Health Workers
51	Plastic Surgeon	25	29	33	0.07	142,375	Mauritania	Minimum Estimate	2212 - Specialist medical practitioners	Lower-middle Income	West	Medical Doctors
52	Psychiatrist	172	191	215	0.46	21,690	Mauritania	Minimum Estimate	2212 - Specialist medical practitioners	Lower-middle Income	West	Medical Doctors
53	Radiation Oncologist	7	9	10	0.02	455,647	Mauritania	Minimum Estimate	2212 - Specialist medical practitioners	Lower-middle Income	West	Medical Doctors
54	Radiographer (Diagnostics and Therapy)	298	337	386	0.83	12,109	Mauritania	Minimum Estimate	3211 - Medical imaging and therapeutic equipment technicians	Lower-middle Income	West	Other Health Workers
55	Radiologist	92	103	116	0.25	40,252	Mauritania	Minimum Estimate	2212 - Specialist medical practitioners	Lower-middle Income	West	Medical Doctors
56	Registered General Nurse / State Certified Nurse	9,831	10,812	11,983	25.67	390	Mauritania	Minimum Estimate	2221 - Nursing professionals	Lower-middle Income	West	Nursing Personnel
57	Renal Nurse	512	595	702	1.51	6,641	Mauritania	Minimum Estimate	2221 - Nursing professionals	Lower-middle Income	West	Nursing Personnel
58	Respiratory Physician	35	39	45	0.10	104,697	Mauritania	Minimum Estimate	2212 - Specialist medical practitioners	Lower-middle Income	West	Medical Doctors
59	Rheumatologist	11	12	14	0.03	336,210	Mauritania	Minimum Estimate	2212 - Specialist medical practitioners	Lower-middle Income	West	Medical Doctors
60	Speech Therapist	35	39	44	0.09	106,741	Mauritania	Minimum Estimate	2266 - Audiologists and speech therapists	Lower-middle Income	West	Other Health Workers
61	Urologist	5	6	7	0.01	713,138	Mauritania	Minimum Estimate	2212 - Specialist medical practitioners	Lower-middle Income	West	Medical Doctors
1	Anaesthesiologist	28	31	35	0.27	36,783	Mauritius	Minimum Estimate	2212 - Specialist medical practitioners	High Income and Upper Middle Income	Southern	Medical Doctors
2	Associate Nurse/Enrolled Nurse/Nursing Assistant	1,617	1,689	1,771	13.83	723	Mauritius	Minimum Estimate	3221 - Nursing associate professionals	High Income and Upper Middle Income	Southern	Nursing Personnel
3	Audiologist	3	4	4	0.03	337,927	Mauritius	Minimum Estimate	2266 - Audiologists and speech therapists	High Income and Upper Middle Income	Southern	Other Health Workers
4	Cardiologist	21	23	27	0.21	47,913	Mauritius	Minimum Estimate	2212 - Specialist medical practitioners	High Income and Upper Middle Income	Southern	Medical Doctors
5	Cardiothoracic Surgeon	3	3	3	0.02	406,536	Mauritius	Minimum Estimate	2212 - Specialist medical practitioners	High Income and Upper Middle Income	Southern	Medical Doctors
6	Clinical Officer/Physician Assistant	300	314	329	2.56	3,904	Mauritius	Minimum Estimate	3256 - Medical assistants	High Income and Upper Middle Income	Southern	Other Health Workers
7	Clinical Pharmacist	55	59	63	0.49	20,450	Mauritius	Minimum Estimate	2262 - Pharmacists	High Income and Upper Middle Income	Southern	Pharmacist

S/N	Health Professionals	2022	2026	2030	Density per 10,000 population	Required Population ratio (1 professional is to xxx population)	Name of Country	Modelling Scenario	ISCO-08 Match	Income Group Classification	Sub-Regional Grouping	SDG 3c Occupation
8	Clinical Psychologist	65	65	66	0.52	19,336	Mauritius	Minimum Estimate	2634 - Psychologists	High Income and Upper Middle Income	Southern	Other Health Workers
9	Community health worker/Village health worker	576	576	573	4.50	2,224	Mauritius	Minimum Estimate	3253 - Community health workers	High Income and Upper Middle Income	Southern	Other Health Workers
10	Dental Surgery Assistant	71	74	76	0.59	16,915	Mauritius	Minimum Estimate	3251 - Dental assistants and therapists	High Income and Upper Middle Income	Southern	Other Health Workers
11	Dental Therapist	44	46	48	0.37	27,065	Mauritius	Minimum Estimate	3251 - Dental assistants and therapists	High Income and Upper Middle Income	Southern	Other Health Workers
12	Dentist	63	63	62	0.48	20,909	Mauritius	Minimum Estimate	2261 - Dentists	High Income and Upper Middle Income	Southern	Dentist
13	Dermatologist	4	4	4	0.03	318,660	Mauritius	Minimum Estimate	2212 - Specialist medical practitioners	High Income and Upper Middle Income	Southern	Medical Doctors
14	Endocrinologist	35	39	43	0.34	29,784	Mauritius	Minimum Estimate	2212 - Specialist medical practitioners	High Income and Upper Middle Income	Southern	Medical Doctors
15	ENT Surgeon	27	29	35	0.28	36,058	Mauritius	Minimum Estimate	2212 - Specialist medical practitioners	High Income and Upper Middle Income	Southern	Medical Doctors
16	Environmental Health Officer	32	32	32	0.25	40,165	Mauritius	Minimum Estimate	2263 - Environmental and occupational health and hygiene professionals	High Income and Upper Middle Income	Southern	Other Health Workers
17	Gastroenterologist	6	6	6	0.05	216,494	Mauritius	Minimum Estimate	2212 - Specialist medical practitioners	High Income and Upper Middle Income	Southern	Medical Doctors
18	General Medical Practitioner (Generalist Doctor)	582	605	629	4.91	2,036	Mauritius	Minimum Estimate	2211 - Generalist medical practitioners	High Income and Upper Middle Income	Southern	Medical Doctors
19	General Surgeon	28	31	34	0.27	37,450	Mauritius	Minimum Estimate	2212 - Specialist medical practitioners	High Income and Upper Middle Income	Southern	Medical Doctors
20	Haematologist	3	4	4	0.03	335,268	Mauritius	Minimum Estimate	2212 - Specialist medical practitioners	High Income and Upper Middle Income	Southern	Medical Doctors
21	Health Promoter/Health Educator	15	16	16	0.13	79,643	Mauritius	Minimum Estimate	2269 - Health professionals not elsewhere classified	High Income and Upper Middle Income	Southern	Other Health Workers
22	Infectious Diseases Specialist	1	1	1	0.01	1,452,717	Mauritius	Minimum Estimate	2212 - Specialist medical practitioners	High Income and Upper Middle Income	Southern	Medical Doctors
23	Intensive Care Nurse	46	48	51	0.40	24,821	Mauritius	Minimum Estimate	2221 - Nursing professionals	High Income and Upper Middle Income	Southern	Nursing Personnel
24	Medical Laboratory Scientist	282	308	338	2.62	3,815	Mauritius	Minimum Estimate	3212 - Medical and pathology laboratory technicians	High Income and Upper Middle Income	Southern	Other Health Workers
25	Medical Laboratory Technician	217	237	259	2.02	4,962	Mauritius	Minimum Estimate	3212 - Medical and pathology laboratory technicians	High Income and Upper Middle Income	Southern	Other Health Workers
26	Medical Social Worker	29	28	27	0.22	46,385	Mauritius	Minimum Estimate	1344 - Social welfare managers	High Income and Upper Middle Income	Southern	Other Health Workers
27	Mental Health Nurse	72	72	72	0.56	17,894	Mauritius	Minimum Estimate	2221 - Nursing professionals	High Income and Upper Middle Income	Southern	Nursing Personnel
28	Midwife	655	655	648	5.05	1,981	Mauritius	Minimum Estimate	2222 - Midwifery professionals	High Income and Upper Middle Income	Southern	Midwifery Personnel
29	Nephrologist	61	68	75	0.58	17,254	Mauritius	Minimum Estimate	2212 - Specialist medical practitioners	High Income and Upper Middle Income	Southern	Medical Doctors
30	Neuro-Surgeon	15	17	21	0.17	60,436	Mauritius	Minimum Estimate	2212 - Specialist medical practitioners	High Income and Upper Middle Income	Southern	Medical Doctors
31	Nurse Anaesthetist	101	117	135	1.05	9,485	Mauritius	Minimum Estimate	2221 - Nursing professionals	High Income and Upper Middle Income	Southern	Nursing Personnel
32	Nutritionist	123	120	116	0.92	10,915	Mauritius	Minimum Estimate	2265 - Dieticians and nutritionists	High Income and Upper Middle Income	Southern	Other Health Workers
33	Obstetrician & Gynaecologist	72	73	73	0.57	17,433	Mauritius	Minimum Estimate	2212 - Specialist medical practitioners	High Income and Upper Middle Income	Southern	Medical Doctors
34	Occupational Therapist	46	50	54	0.42	23,881	Mauritius	Minimum Estimate	2269 - Health professionals not elsewhere classified	High Income and Upper Middle Income	Southern	Other Health Workers
35	Oncology Nurse	49	55	62	0.48	20,843	Mauritius	Minimum Estimate	2221 - Nursing professionals	High Income and Upper Middle Income	Southern	Nursing Personnel
36	Operating Theatre Nurse	333	360	399	3.12	3,210	Mauritius	Minimum Estimate	2221 - Nursing professionals	High Income and Upper Middle Income	Southern	Nursing Personnel
37	Ophthalmic Nurse	53	57	62	0.48	20,669	Mauritius	Minimum Estimate	2221 - Nursing professionals	High Income and Upper Middle Income	Southern	Nursing Personnel
38	Ophthalmologist	21	23	26	0.21	48,290	Mauritius	Minimum Estimate	2212 - Specialist medical practitioners	High Income and Upper Middle Income	Southern	Medical Doctors
39	Optometrist	43	47	53	0.41	24,416	Mauritius	Minimum Estimate	2267 - Optometrists and ophthalmic opticians	High Income and Upper Middle Income	Southern	Other Health Workers
40	Orthopaedic Nurse	13	13	14	0.11	89,662	Mauritius	Minimum Estimate	2221 - Nursing professionals	High Income and Upper Middle Income	Southern	Nursing Personnel
41	Orthopaedic Surgeon	110	119	128	1.00	9,993	Mauritius	Minimum Estimate	2212 - Specialist medical practitioners	High Income and Upper Middle Income	Southern	Medical Doctors
42	Orthopaedic Technologist	34	35	36	0.28	35,863	Mauritius	Minimum Estimate	3214 - Medical and dental prosthetic technicians	High Income and Upper Middle Income	Southern	Other Health Workers
43	Paediatric Nurse	76	75	73	0.57	17,525	Mauritius	Minimum Estimate	2221 - Nursing professionals	High Income and Upper Middle Income	Southern	Nursing Personnel
44	Paediatric Surgeon	1	1	1	0.01	1,244,683	Mauritius	Minimum Estimate	2212 - Specialist medical practitioners	High Income and Upper Middle Income	Southern	Medical Doctors
45	Paediatrician	77	82	87	0.68	14,774	Mauritius	Minimum Estimate	2212 - Specialist medical practitioners	High Income and Upper Middle Income	Southern	Medical Doctors
46	Pathologist	9	10	10	0.08	130,292	Mauritius	Minimum Estimate	2212 - Specialist medical practitioners	High Income and Upper Middle Income	Southern	Medical Doctors
47	Pharmacist	248	258	270	2.10	4,751	Mauritius	Minimum Estimate	2262 - Pharmacists	High Income and Upper Middle Income	Southern	Pharmacist
48	Pharmacy Technician	194	202	210	1.64	6,108	Mauritius	Minimum Estimate	3213 - Pharmaceutical technicians and assistants	High Income and Upper Middle Income	Southern	Other Health Workers
49	Physician	233	249	267	2.07	4,821	Mauritius	Minimum Estimate	2212 - Specialist medical practitioners	High Income and Upper Middle Income	Southern	Medical Doctors
50	Physiotherapist	41	43	47	0.37	26,878	Mauritius	Minimum Estimate	2264 - Physiotherapists	High Income and Upper Middle Income	Southern	Other Health Workers
51	Plastic Surgeon	16	17	17	0.13	76,823	Mauritius	Minimum Estimate	2212 - Specialist medical practitioners	High Income and Upper Middle Income	Southern	Medical Doctors
52	Psychiatrist	66	66	65	0.51	19,801	Mauritius	Minimum Estimate	2212 - Specialist medical practitioners	High Income and Upper Middle Income	Southern	Medical Doctors
53	Radiation Oncologist	18	21	25	0.19	51,962	Mauritius	Minimum Estimate	2212 - Specialist medical practitioners	High Income and Upper Middle Income	Southern	Medical Doctors
54	Radiographer (Diagnostics and Therapy)	210	228	247	1.92	5,219	Mauritius	Minimum Estimate	3211 - Medical imaging and therapeutic equipment technicians	High Income and Upper Middle Income	Southern	Other Health Workers
55	Radiologist	52	55	60	0.46	21,560	Mauritius	Minimum Estimate	2212 - Specialist medical practitioners	High Income and Upper Middle Income	Southern	Medical Doctors
56	Registered General Nurse / State Certified Nurse	5,472	5,960	6,511	50.54	198	Mauritius	Minimum Estimate	2221 - Nursing professionals	High Income and Upper Middle Income	Southern	Nursing Personnel
57	Renal Nurse	781	865	957	7.39	1,353	Mauritius	Minimum Estimate	2221 - Nursing professionals	High Income and Upper Middle Income	Southern	Nursing Personnel
58	Respiratory Physician	8	8	8	0.06	158,241	Mauritius	Minimum Estimate	2212 - Specialist medical practitioners	High Income and Upper Middle Income	Southern	Medical Doctors
59	Rheumatologist	5	5	5	0.04	236,286	Mauritius	Minimum Estimate	2212 - Specialist medical practitioners	High Income and Upper Middle Income	Southern	Medical Doctors
60	Speech Therapist	19	20	24	0.19	51,916	Mauritius	Minimum Estimate	2266 - Audiologists and speech therapists	High Income and Upper Middle Income	Southern	Other Health Workers
61	Urologist	13	16	19	0.15	68,637	Mauritius	Minimum Estimate	2212 - Specialist medical practitioners	High Income and Upper Middle Income	Southern	Medical Doctors
1	Anaesthesiologist	217	245	276	0.09	114,103	Mozambique	Minimum Estimate	2212 - Specialist medical practitioners	Low Income	Southern	Medical Doctors
2	Associate Nurse/Enrolled Nurse/Nursing Assistant	29,234	32,580	36,366	11.55	866	Mozambique	Minimum Estimate	3221 - Nursing associate professionals	Low Income	Southern	Nursing Personnel
3	Audiologist	78	87	97	0.03	325,798	Mozambique	Minimum Estimate	2266 - Audiologists and speech therapists	Low Income	Southern	Other Health Workers
4	Cardiologist	80	89	98	0.03	321,826	Mozambique	Minimum Estimate	2212 - Specialist medical practitioners	Low Income	Southern	Medical Doctors
5	Cardiothoracic Surgeon	44	49	56	0.02	567,654	Mozambique	Minimum Estimate	2212 - Specialist medical practitioners	Low Income	Southern	Medical Doctors
6	Clinical Officer/Physician Assistant	3,919	4,285	4,746	1.51	6,605	Mozambique	Minimum Estimate	3256 - Medical assistants	Low Income	Southern	Other Health Workers
7	Clinical Pharmacist	563	625	703	0.22	44,708	Mozambique	Minimum Estimate	2262 - Pharmacists	Low Income	Southern	Pharmacist
8	Clinical Psychologist	1,527	1,695	1,896	0.60	16,609	Mozambique	Minimum Estimate	2634 - Psychologists	Low Income	Southern	Other Health Workers
9	Community health worker/Village health worker	23,522	25,799	28,259	8.97	1,115	Mozambique	Minimum Estimate	3253 - Community health workers	Low Income	Southern	Other Health Workers
10	Dental Surgery Assistant	1,838	2,046	2,294	0.73	13,754	Mozambique	Minimum Estimate	3251 - Dental assistants and therapists	Low Income	Southern	Other Health Workers
11	Dental Therapist	1,133	1,261	1,413	0.45	22,327	Mozambique	Minimum Estimate	3251 - Dental assistants and therapists	Low Income	Southern	Other Health Workers
12	Dentist	1,629	1,814	2,059	0.65	15,307	Mozambique	Minimum Estimate	2261 - Dentists	Low Income	Southern	Dentist
13	Dermatologist	73	82	91	0.03	344,930	Mozambique	Minimum Estimate	2212 - Specialist medical practitioners	Low Income	Southern	Medical Doctors
14	Endocrinologist	64	76	92	0.03	340,657	Mozambique	Minimum Estimate	2212 - Specialist medical practitioners	Low Income	Southern	Medical Doctors
15	ENT Surgeon	208	236	265	0.08	118,946	Mozambique	Minimum Estimate	2212 - Specialist medical practitioners	Low Income	Southern	Medical Doctors
16	Environmental Health Officer	776	866	962	0.30	32,825	Mozambique	Minimum Estimate	2263 - Environmental and occupational health and hygiene professionals	Low Income	Southern	Other Health Workers
17	Gastroenterologist	191	192	200	0.06	155,857	Mozambique	Minimum Estimate	2212 - Specialist medical practitioners	Low Income	Southern	Medical Doctors
18	General Medical Practitioner (Generalist Doctor)	8,504	9,518	10,692	3.40	2,943	Mozambique	Minimum Estimate	2211 - Generalist medical practitioners	Low Income	Southern	Medical Doctors
19	General Surgeon	161	187	220	0.07	142,548	Mozambique	Minimum Estimate	2212 - Specialist medical practitioners	Low Income	Southern	Medical Doctors
20	Haematologist	82	90	98	0.03	322,080	Mozambique	Minimum Estimate	2212 - Specialist medical practitioners	Low Income	Southern	Medical Doctors
21	Health Promoter/Health Educator	187	203	223	0.07	140,326	Mozambique	Minimum Estimate	2269 - Health professionals not elsewhere classified	Low Income	Southern	Other Health Workers
22	Infectious Diseases Specialist	21	23	26	0.01	1,215,883	Mozambique	Minimum Estimate	2212 - Specialist medical practitioners	Low Income	Southern	Medical Doctors
23	Intensive Care Nurse	592	668	751	0.24	41,947	Mozambique	Minimum Estimate	2221 - Nursing professionals	Low Income	Southern	Nursing Personnel
24	Medical Laboratory Scientist	3,370	3,847	4,435	1.41	7,074	Mozambique	Minimum Estimate	3212 - Medical and pathology laboratory technicians	Low Income	Southern	Other Health Workers
25	Medical Laboratory Technician	5,311	5,898	6,568	2.09	4,787	Mozambique	Minimum Estimate	3212 - Medical and pathology laboratory technicians	Low Income	Southern	Other Health Workers
26	Medical Social Worker	718	757	808	0.26	38,809	Mozambique	Minimum Estimate	1344 - Social welfare managers	Low Income	Southern	Other Health Workers
27	Mental Health Nurse	1,019	1,148	1,330	0.42	23,583	Mozambique	Minimum Estimate	2221 - Nursing professionals	Low Income	Southern	Nursing Personnel
28	Midwife	18,855	20,769	22,996	7.29	1,372	Mozambique	Minimum Estimate	2222 - Midwifery professionals	Low Income	Southern	Midwifery Personnel
29	Nephrologist	156	178	209	0.07	149,810	Mozambique	Minimum Estimate	2212 - Specialist medical practitioners	Low Income	Southern	Medical Doctors
30	Neuro-Surgeon	82	94	107	0.03	292,754	Mozambique	Minimum Estimate	2212 - Specialist medical practitioners	Low Income	Southern	Medical Doctors

S/N	Health Professionals	2022	2026	2030	Density per 10,000 population	Required Population ratio (1 professional is to xxx population)	Name of Country	Modelling Scenario	ISCO-08 Match	Income Group Classification	Sub-Regional Grouping	SDG 3c Occupation
31	Nurse Anaesthetist	429	494	568	0.18	55,440	Mozambique	Minimum Estimate	2221 - Nursing professionals	Low Income	Southern	Nursing Personnel
32	Nutritionist	2,373	2,511	2,645	0.84	11,919	Mozambique	Minimum Estimate	2265 - Dieticians and nutritionists	Low Income	Southern	Other Health Workers
33	Obstetrician & Gynaecologist	1,661	1,866	2,102	0.67	14,979	Mozambique	Minimum Estimate	2212 - Specialist medical practitioners	Low Income	Southern	Medical Doctors
34	Occupational Therapist	394	448	512	0.16	61,526	Mozambique	Minimum Estimate	2269 - Health professionals not elsewhere classified	Low Income	Southern	Other Health Workers
35	Oncology Nurse	130	148	173	0.06	181,072	Mozambique	Minimum Estimate	2221 - Nursing professionals	Low Income	Southern	Nursing Personnel
36	Operating Theatre Nurse	2,103	2,365	2,719	0.87	11,545	Mozambique	Minimum Estimate	2221 - Nursing professionals	Low Income	Southern	Nursing Personnel
37	Ophthalmic Nurse	219	244	275	0.09	114,220	Mozambique	Minimum Estimate	2221 - Nursing professionals	Low Income	Southern	Nursing Personnel
38	Ophthalmologist	61	69	79	0.03	396,535	Mozambique	Minimum Estimate	2212 - Specialist medical practitioners	Low Income	Southern	Medical Doctors
39	Optometrist	236	261	290	0.09	108,608	Mozambique	Minimum Estimate	2267 - Optometrists and ophthalmic opticians	Low Income	Southern	Other Health Workers
40	Orthopaedic Nurse	189	218	250	0.08	126,129	Mozambique	Minimum Estimate	2221 - Nursing professionals	Low Income	Southern	Nursing Personnel
41	Orthopaedic Surgeon	500	598	722	0.23	43,357	Mozambique	Minimum Estimate	2212 - Specialist medical practitioners	Low Income	Southern	Medical Doctors
42	Orthopaedic Technologist	799	898	1,005	0.32	31,390	Mozambique	Minimum Estimate	3214 - Medical and dental prosthetic technicians	Low Income	Southern	Other Health Workers
43	Paediatric Nurse	2,567	2,820	3,075	0.97	10,278	Mozambique	Minimum Estimate	2221 - Nursing professionals	Low Income	Southern	Nursing Personnel
44	Paediatric Surgeon	88	98	108	0.03	293,972	Mozambique	Minimum Estimate	2212 - Specialist medical practitioners	Low Income	Southern	Medical Doctors
45	Paediatrician	1,078	1,220	1,384	0.44	22,765	Mozambique	Minimum Estimate	2212 - Specialist medical practitioners	Low Income	Southern	Medical Doctors
46	Pathologist	169	189	219	0.07	143,383	Mozambique	Minimum Estimate	2212 - Specialist medical practitioners	Low Income	Southern	Medical Doctors
47	Pharmacist	1,644	1,711	1,793	0.57	17,482	Mozambique	Minimum Estimate	2262 - Pharmacists	Low Income	Southern	Pharmacist
48	Pharmacy Technician	1,949	2,151	2,383	0.76	13,207	Mozambique	Minimum Estimate	3213 - Pharmaceutical technicians and assistants	Low Income	Southern	Other Health Workers
49	Physician	1,881	2,093	2,344	0.74	13,427	Mozambique	Minimum Estimate	2212 - Specialist medical practitioners	Low Income	Southern	Medical Doctors
50	Physiotherapist	559	628	703	0.22	44,857	Mozambique	Minimum Estimate	2264 - Physiotherapists	Low Income	Southern	Other Health Workers
51	Plastic Surgeon	325	371	421	0.13	74,894	Mozambique	Minimum Estimate	2212 - Specialist medical practitioners	Low Income	Southern	Medical Doctors
52	Psychiatrist	965	1,077	1,229	0.39	25,564	Mozambique	Minimum Estimate	2212 - Specialist medical practitioners	Low Income	Southern	Medical Doctors
53	Radiation Oncologist	37	44	51	0.02	614,233	Mozambique	Minimum Estimate	2212 - Specialist medical practitioners	Low Income	Southern	Medical Doctors
54	Radiographer (Diagnostics and Therapy)	1,269	1,435	1,656	0.53	18,949	Mozambique	Minimum Estimate	3211 - Medical imaging and therapeutic equipment technicians	Low Income	Southern	Other Health Workers
55	Radiologist	409	469	548	0.18	57,008	Mozambique	Minimum Estimate	2212 - Specialist medical practitioners	Low Income	Southern	Medical Doctors
56	Registered General Nurse / State Certified Nurse	52,145	56,277	61,307	19.52	512	Mozambique	Minimum Estimate	2221 - Nursing professionals	Low Income	Southern	Nursing Personnel
57	Renal Nurse	1,938	2,203	2,587	0.83	12,112	Mozambique	Minimum Estimate	2221 - Nursing professionals	Low Income	Southern	Nursing Personnel
58	Respiratory Physician	225	257	293	0.09	107,666	Mozambique	Minimum Estimate	2212 - Specialist medical practitioners	Low Income	Southern	Medical Doctors
59	Rheumatologist	61	68	76	0.02	413,966	Mozambique	Minimum Estimate	2212 - Specialist medical practitioners	Low Income	Southern	Medical Doctors
60	Speech Therapist	237	264	293	0.09	107,676	Mozambique	Minimum Estimate	2266 - Audiologists and speech therapists	Low Income	Southern	Other Health Workers
61	Urologist	16	19	24	0.01	1,311,974	Mozambique	Minimum Estimate	2212 - Specialist medical practitioners	Low Income	Southern	Medical Doctors
1	Anaesthesiologist	22	24	27	0.11	95,103	Namibia	Minimum Estimate	2212 - Specialist medical practitioners	High Income and Upper Middle Income	Southern	Medical Doctors
2	Associate Nurse/Enrolled Nurse/Nursing Assistant	2,619	2,805	3,043	12.20	820	Namibia	Minimum Estimate	3221 - Nursing associate professionals	High Income and Upper Middle Income	Southern	Nursing Personnel
3	Audiologist	7	7	8	0.03	323,870	Namibia	Minimum Estimate	2266 - Audiologists and speech therapists	High Income and Upper Middle Income	Southern	Other Health Workers
4	Cardiologist	11	13	14	0.05	183,932	Namibia	Minimum Estimate	2212 - Specialist medical practitioners	High Income and Upper Middle Income	Southern	Medical Doctors
5	Cardiothoracic Surgeon	3	3	3	0.01	731,290	Namibia	Minimum Estimate	2212 - Specialist medical practitioners	High Income and Upper Middle Income	Southern	Medical Doctors
6	Clinical Officer/Physician Assistant	426	458	528	2.41	4,149	Namibia	Minimum Estimate	3256 - Medical assistants	High Income and Upper Middle Income	Southern	Other Health Workers
7	Clinical Pharmacist	49	54	60	0.24	42,278	Namibia	Minimum Estimate	2262 - Pharmacists	High Income and Upper Middle Income	Southern	Pharmacist
8	Clinical Psychologist	100	108	117	0.46	21,869	Namibia	Minimum Estimate	2634 - Psychologists	High Income and Upper Middle Income	Southern	Other Health Workers
9	Community health worker/Village health worker	1,228	1,316	1,593	8.25	1,212	Namibia	Minimum Estimate	3253 - Community health workers	High Income and Upper Middle Income	Southern	Other Health Workers
10	Dental Surgery Assistant	201	220	241	0.94	10,613	Namibia	Minimum Estimate	3251 - Dental assistants and therapists	High Income and Upper Middle Income	Southern	Other Health Workers
11	Dental Therapist	126	138	151	0.59	16,947	Namibia	Minimum Estimate	3251 - Dental assistants and therapists	High Income and Upper Middle Income	Southern	Other Health Workers
12	Dentist	152	163	175	0.69	14,528	Namibia	Minimum Estimate	2261 - Dentists	High Income and Upper Middle Income	Southern	Dentist
13	Dermatologist	7	8	8	0.03	310,655	Namibia	Minimum Estimate	2212 - Specialist medical practitioners	High Income and Upper Middle Income	Southern	Medical Doctors
14	Endocrinologist	11	12	14	0.06	179,302	Namibia	Minimum Estimate	2212 - Specialist medical practitioners	High Income and Upper Middle Income	Southern	Medical Doctors
15	ENT Surgeon	24	26	29	0.11	87,124	Namibia	Minimum Estimate	2212 - Specialist medical practitioners	High Income and Upper Middle Income	Southern	Medical Doctors
16	Environmental Health Officer	63	67	71	0.28	35,840	Namibia	Minimum Estimate	2263 - Environmental and occupational health and hygiene professionals	High Income and Upper Middle Income	Southern	Other Health Workers
17	Gastroenterologist	14	13	13	0.05	190,230	Namibia	Minimum Estimate	2212 - Specialist medical practitioners	High Income and Upper Middle Income	Southern	Medical Doctors
18	General Medical Practitioner (Generalist Doctor)	846	908	995	4.09	2,447	Namibia	Minimum Estimate	2211 - Generalist medical practitioners	High Income and Upper Middle Income	Southern	Medical Doctors
19	General Surgeon	27	30	34	0.13	75,214	Namibia	Minimum Estimate	2212 - Specialist medical practitioners	High Income and Upper Middle Income	Southern	Medical Doctors
20	Haematologist	4	4	5	0.02	523,702	Namibia	Minimum Estimate	2212 - Specialist medical practitioners	High Income and Upper Middle Income	Southern	Medical Doctors
21	Health Promoter/Health Educator	21	23	26	0.12	86,467	Namibia	Minimum Estimate	2269 - Health professionals not elsewhere classified	High Income and Upper Middle Income	Southern	Other Health Workers
22	Infectious Diseases Specialist	3	3	3	0.01	881,690	Namibia	Minimum Estimate	2212 - Specialist medical practitioners	High Income and Upper Middle Income	Southern	Medical Doctors
23	Intensive Care Nurse	58	61	66	0.26	38,887	Namibia	Minimum Estimate	2221 - Nursing professionals	High Income and Upper Middle Income	Southern	Nursing Personnel
24	Medical Laboratory Scientist	282	308	353	1.53	6,534	Namibia	Minimum Estimate	3212 - Medical and pathology laboratory technicians	High Income and Upper Middle Income	Southern	Other Health Workers
25	Medical Laboratory Technician	234	270	373	2.13	4,700	Namibia	Minimum Estimate	3212 - Medical and pathology laboratory technicians	High Income and Upper Middle Income	Southern	Other Health Workers
26	Medical Social Worker	59	62	66	0.27	36,730	Namibia	Minimum Estimate	1344 - Social welfare managers	High Income and Upper Middle Income	Southern	Other Health Workers
27	Mental Health Nurse	98	106	115	0.45	22,033	Namibia	Minimum Estimate	2221 - Nursing professionals	High Income and Upper Middle Income	Southern	Nursing Personnel
28	Midwife	1,375	1,461	1,551	6.07	1,647	Namibia	Minimum Estimate	2222 - Midwifery professionals	High Income and Upper Middle Income	Southern	Midwifery Personnel
29	Nephrologist	27	31	36	0.14	70,991	Namibia	Minimum Estimate	2212 - Specialist medical practitioners	High Income and Upper Middle Income	Southern	Medical Doctors
30	Neuro-Surgeon	9	10	11	0.04	222,329	Namibia	Minimum Estimate	2212 - Specialist medical practitioners	High Income and Upper Middle Income	Southern	Medical Doctors
31	Nurse Anaesthetist	66	79	94	0.37	26,922	Namibia	Minimum Estimate	2221 - Nursing professionals	High Income and Upper Middle Income	Southern	Nursing Personnel
32	Nutritionist	221	225	229	0.90	11,145	Namibia	Minimum Estimate	2265 - Dieticians and nutritionists	High Income and Upper Middle Income	Southern	Other Health Workers
33	Obstetrician & Gynaecologist	163	178	197	0.78	12,746	Namibia	Minimum Estimate	2212 - Specialist medical practitioners	High Income and Upper Middle Income	Southern	Medical Doctors
34	Occupational Therapist	45	51	57	0.22	44,640	Namibia	Minimum Estimate	2269 - Health professionals not elsewhere classified	High Income and Upper Middle Income	Southern	Other Health Workers
35	Oncology Nurse	23	27	32	0.12	80,232	Namibia	Minimum Estimate	2221 - Nursing professionals	High Income and Upper Middle Income	Southern	Nursing Personnel
36	Operating Theatre Nurse	292	324	365	1.43	6,969	Namibia	Minimum Estimate	2221 - Nursing professionals	High Income and Upper Middle Income	Southern	Nursing Personnel
37	Ophthalmic Nurse	30	33	37	0.14	69,576	Namibia	Minimum Estimate	2221 - Nursing professionals	High Income and Upper Middle Income	Southern	Nursing Personnel
38	Ophthalmologist	10	12	13	0.05	196,355	Namibia	Minimum Estimate	2212 - Specialist medical practitioners	High Income and Upper Middle Income	Southern	Medical Doctors
39	Optometrist	50	56	62	0.24	41,118	Namibia	Minimum Estimate	2267 - Optometrists and ophthalmic opticians	High Income and Upper Middle Income	Southern	Other Health Workers
40	Orthopaedic Nurse	23	26	30	0.12	86,488	Namibia	Minimum Estimate	2221 - Nursing professionals	High Income and Upper Middle Income	Southern	Nursing Personnel
41	Orthopaedic Surgeon	87	99	114	0.45	22,341	Namibia	Minimum Estimate	2212 - Specialist medical practitioners	High Income and Upper Middle Income	Southern	Medical Doctors
42	Orthopaedic Technologist	68	73	79	0.31	32,252	Namibia	Minimum Estimate	3214 - Medical and dental prosthetic technicians	High Income and Upper Middle Income	Southern	Other Health Workers
43	Paediatric Nurse	165	171	175	0.69	14,556	Namibia	Minimum Estimate	2221 - Nursing professionals	High Income and Upper Middle Income	Southern	Nursing Personnel
44	Paediatric Surgeon	5	6	6	0.02	429,278	Namibia	Minimum Estimate	2212 - Specialist medical practitioners	High Income and Upper Middle Income	Southern	Medical Doctors
45	Paediatrician	65	71	77	0.30	32,907	Namibia	Minimum Estimate	2212 - Specialist medical practitioners	High Income and Upper Middle Income	Southern	Medical Doctors
46	Pathologist	23	25	28	0.11	91,062	Namibia	Minimum Estimate	2212 - Specialist medical practitioners	High Income and Upper Middle Income	Southern	Medical Doctors
47	Pharmacist	279	291	305	1.20	8,314	Namibia	Minimum Estimate	2262 - Pharmacists	High Income and Upper Middle Income	Southern	Pharmacist
48	Pharmacy Technician	255	273	294	1.15	8,681	Namibia	Minimum Estimate	3213 - Pharmaceutical technicians and assistants	High Income and Upper Middle Income	Southern	Other Health Workers
49	Physician	193	211	233	0.94	10,609	Namibia	Minimum Estimate	2212 - Specialist medical practitioners	High Income and Upper Middle Income	Southern	Medical Doctors
50	Physiotherapist	51	56	61	0.24	42,057	Namibia	Minimum Estimate	2264 - Physiotherapists	High Income and Upper Middle Income	Southern	Other Health Workers
51	Plastic Surgeon	17	18	20	0.08	130,221	Namibia	Minimum Estimate	2212 - Specialist medical practitioners	High Income and Upper Middle Income	Southern	Medical Doctors
52	Psychiatrist	95	102	111	0.43	23,001	Namibia	Minimum Estimate	2212 - Specialist medical practitioners	High Income and Upper Middle Income	Southern	Medical Doctors
53	Radiation Oncologist	7	9	11	0.04	233,625	Namibia	Minimum Estimate	2212 - Specialist medical practitioners	High Income and Upper Middle Income	Southern	Medical Doctors

S/N	Health Professionals	2022	2026	2030	Density per 10,000 population	Required Population ratio (1 professional is to xxx population)	Name of Country	Modelling Scenario	ISCO-08 Match	Income Group Classification	Sub-Regional Grouping	SDG 3c Occupation
54	Radiographer (Diagnostics and Therapy)	175	195	220	0.87	11,451	Namibia	Minimum Estimate	3211 - Medical imaging and therapeutic equipment technicians	High Income and Upper Middle Income	Southern	Other Health Workers
55	Radiologist	54	62	74	0.30	32,818	Namibia	Minimum Estimate	2212 - Specialist medical practitioners	High Income and Upper Middle Income	Southern	Medical Doctors
56	Registered General Nurse / State Certified Nurse	5,174	5,502	5,913	23.29	429	Namibia	Minimum Estimate	2221 - Nursing professionals	High Income and Upper Middle Income	Southern	Nursing Personnel
57	Renal Nurse	339	387	446	1.76	5,688	Namibia	Minimum Estimate	2221 - Nursing professionals	High Income and Upper Middle Income	Southern	Nursing Personnel
58	Respiratory Physician	10	11	12	0.05	209,422	Namibia	Minimum Estimate	2212 - Specialist medical practitioners	High Income and Upper Middle Income	Southern	Medical Doctors
59	Rheumatologist	7	8	8	0.03	309,921	Namibia	Minimum Estimate	2212 - Specialist medical practitioners	High Income and Upper Middle Income	Southern	Medical Doctors
60	Speech Therapist	19	21	22	0.09	114,267	Namibia	Minimum Estimate	2266 - Audiologists and speech therapists	High Income and Upper Middle Income	Southern	Other Health Workers
61	Urologist	3	4	4	0.02	572,626	Namibia	Minimum Estimate	2212 - Specialist medical practitioners	High Income and Upper Middle Income	Southern	Medical Doctors
1	Anaesthesiologist	124	145	168	0.07	144,644	Niger	Minimum Estimate	2212 - Specialist medical practitioners	Low Income	West	Medical Doctors
2	Associate Nurse/Enrolled Nurse/Nursing Assistant	21,125	24,324	28,016	11.50	869	Niger	Minimum Estimate	3221 - Nursing associate professionals	Low Income	West	Nursing Personnel
3	Audiologist	59	69	79	0.03	307,596	Niger	Minimum Estimate	2266 - Audiologists and speech therapists	Low Income	West	Other Health Workers
4	Cardiologist	56	66	77	0.03	314,996	Niger	Minimum Estimate	2212 - Specialist medical practitioners	Low Income	West	Medical Doctors
5	Cardiothoracic Surgeon	22	26	30	0.01	820,513	Niger	Minimum Estimate	2212 - Specialist medical practitioners	Low Income	West	Medical Doctors
6	Clinical Officer/Physician Assistant	1,689	1,806	1,951	0.81	12,418	Niger	Minimum Estimate	3256 - Medical assistants	Low Income	West	Other Health Workers
7	Clinical Pharmacist	425	489	567	0.23	42,794	Niger	Minimum Estimate	2262 - Pharmacists	Low Income	West	Pharmacist
8	Clinical Psychologist	902	1,029	1,181	0.49	20,586	Niger	Minimum Estimate	2634 - Psychologists	Low Income	West	Other Health Workers
9	Community health worker/Village health worker	17,944	20,059	22,406	9.19	1,088	Niger	Minimum Estimate	3253 - Community health workers	Low Income	West	Other Health Workers
10	Dental Surgery Assistant	1,414	1,633	1,896	0.78	12,838	Niger	Minimum Estimate	3251 - Dental assistants and therapists	Low Income	West	Other Health Workers
11	Dental Therapist	883	1,019	1,182	0.49	20,588	Niger	Minimum Estimate	3251 - Dental assistants and therapists	Low Income	West	Other Health Workers
12	Dentist	1,003	1,167	1,382	0.57	17,541	Niger	Minimum Estimate	2261 - Dentists	Low Income	West	Dentist
13	Dermatologist	62	72	83	0.03	292,945	Niger	Minimum Estimate	2212 - Specialist medical practitioners	Low Income	West	Medical Doctors
14	Endocrinologist	33	45	64	0.03	366,291	Niger	Minimum Estimate	2212 - Specialist medical practitioners	Low Income	West	Medical Doctors
15	ENT Surgeon	151	176	204	0.08	119,452	Niger	Minimum Estimate	2212 - Specialist medical practitioners	Low Income	West	Medical Doctors
16	Environmental Health Officer	601	696	804	0.33	30,330	Niger	Minimum Estimate	2263 - Environmental and occupational health and hygiene professionals	Low Income	West	Other Health Workers
17	Gastroenterologist	186	196	210	0.09	115,510	Niger	Minimum Estimate	2212 - Specialist medical practitioners	Low Income	West	Medical Doctors
18	General Medical Practitioner (Generalist Doctor)	6,363	7,264	8,305	3.41	2,932	Niger	Minimum Estimate	2211 - Generalist medical practitioners	Low Income	West	Medical Doctors
19	General Surgeon	136	160	191	0.08	126,665	Niger	Minimum Estimate	2212 - Specialist medical practitioners	Low Income	West	Medical Doctors
20	Haematologist	76	88	103	0.04	236,372	Niger	Minimum Estimate	2212 - Specialist medical practitioners	Low Income	West	Medical Doctors
21	Health Promoter/Health Educator	102	109	118	0.05	205,066	Niger	Minimum Estimate	2269 - Health professionals not elsewhere classified	Low Income	West	Other Health Workers
22	Infectious Diseases Specialist	18	20	23	0.01	1,055,983	Niger	Minimum Estimate	2212 - Specialist medical practitioners	Low Income	West	Medical Doctors
23	Intensive Care Nurse	645	744	857	0.35	28,452	Niger	Minimum Estimate	2221 - Nursing professionals	Low Income	West	Nursing Personnel
24	Medical Laboratory Scientist	1,695	1,931	2,222	0.92	10,902	Niger	Minimum Estimate	3212 - Medical and pathology laboratory technicians	Low Income	West	Other Health Workers
25	Medical Laboratory Technician	2,381	2,554	2,754	1.13	8,822	Niger	Minimum Estimate	3212 - Medical and pathology laboratory technicians	Low Income	West	Other Health Workers
26	Medical Social Worker	1,096	1,219	1,355	0.56	17,987	Niger	Minimum Estimate	1344 - Social welfare managers	Low Income	West	Other Health Workers
27	Mental Health Nurse	716	834	982	0.41	24,668	Niger	Minimum Estimate	2221 - Nursing professionals	Low Income	West	Nursing Personnel
28	Midwife	13,236	15,069	17,292	7.11	1,406	Niger	Minimum Estimate	2222 - Midwifery professionals	Low Income	West	Midwifery Personnel
29	Nephrologist	117	138	165	0.07	146,095	Niger	Minimum Estimate	2212 - Specialist medical practitioners	Low Income	West	Medical Doctors
30	Neuro-Surgeon	36	43	51	0.02	479,826	Niger	Minimum Estimate	2212 - Specialist medical practitioners	Low Income	West	Medical Doctors
31	Nurse Anaesthetist	206	245	292	0.12	83,399	Niger	Minimum Estimate	2221 - Nursing professionals	Low Income	West	Nursing Personnel
32	Nutritionist	5,023	5,671	6,383	2.61	3,825	Niger	Minimum Estimate	2265 - Dieticians and nutritionists	Low Income	West	Other Health Workers
33	Obstetrician & Gynaecologist	1,112	1,288	1,492	0.61	16,324	Niger	Minimum Estimate	2212 - Specialist medical practitioners	Low Income	West	Medical Doctors
34	Occupational Therapist	257	302	353	0.15	68,804	Niger	Minimum Estimate	2269 - Health professionals not elsewhere classified	Low Income	West	Other Health Workers
35	Oncology Nurse	83	98	118	0.05	205,175	Niger	Minimum Estimate	2221 - Nursing professionals	Low Income	West	Nursing Personnel
36	Operating Theatre Nurse	1,343	1,557	1,823	0.75	13,301	Niger	Minimum Estimate	2221 - Nursing professionals	Low Income	West	Nursing Personnel
37	Ophthalmic Nurse	167	203	249	0.10	96,763	Niger	Minimum Estimate	2221 - Nursing professionals	Low Income	West	Nursing Personnel
38	Ophthalmologist	48	59	72	0.03	333,304	Niger	Minimum Estimate	2212 - Specialist medical practitioners	Low Income	West	Medical Doctors
39	Optometrist	258	299	347	0.14	69,988	Niger	Minimum Estimate	2267 - Optometrists and ophthalmic opticians	Low Income	West	Other Health Workers
40	Orthopaedic Nurse	135	159	187	0.08	129,904	Niger	Minimum Estimate	2221 - Nursing professionals	Low Income	West	Nursing Personnel
41	Orthopaedic Surgeon	370	431	503	0.21	48,343	Niger	Minimum Estimate	2212 - Specialist medical practitioners	Low Income	West	Medical Doctors
42	Orthopaedic Technologist	564	656	760	0.31	32,059	Niger	Minimum Estimate	3214 - Medical and dental prosthetic technicians	Low Income	West	Other Health Workers
43	Paediatric Nurse	2,816	3,236	3,694	1.51	6,609	Niger	Minimum Estimate	2221 - Nursing professionals	Low Income	West	Nursing Personnel
44	Paediatric Surgeon	83	98	114	0.05	213,177	Niger	Minimum Estimate	2212 - Specialist medical practitioners	Low Income	West	Medical Doctors
45	Paediatrician	711	828	965	0.40	25,233	Niger	Minimum Estimate	2212 - Specialist medical practitioners	Low Income	West	Medical Doctors
46	Pathologist	81	92	105	0.04	230,263	Niger	Minimum Estimate	2212 - Specialist medical practitioners	Low Income	West	Medical Doctors
47	Pharmacist	1,090	1,180	1,285	0.53	18,896	Niger	Minimum Estimate	2262 - Pharmacists	Low Income	West	Pharmacist
48	Pharmacy Technician	1,632	1,871	2,152	0.88	11,304	Niger	Minimum Estimate	3213 - Pharmaceutical technicians and assistants	Low Income	West	Other Health Workers
49	Physician	1,711	1,972	2,284	0.94	10,632	Niger	Minimum Estimate	2212 - Specialist medical practitioners	Low Income	West	Medical Doctors
50	Physiotherapist	400	465	539	0.22	45,194	Niger	Minimum Estimate	2264 - Physiotherapists	Low Income	West	Other Health Workers
51	Plastic Surgeon	92	108	128	0.05	190,991	Niger	Minimum Estimate	2212 - Specialist medical practitioners	Low Income	West	Medical Doctors
52	Psychiatrist	939	1,081	1,254	0.52	19,383	Niger	Minimum Estimate	2212 - Specialist medical practitioners	Low Income	West	Medical Doctors
53	Radiation Oncologist	18	21	26	0.01	942,793	Niger	Minimum Estimate	2212 - Specialist medical practitioners	Low Income	West	Medical Doctors
54	Radiographer (Diagnostics and Therapy)	1,000	1,164	1,369	0.57	17,696	Niger	Minimum Estimate	3211 - Medical imaging and therapeutic equipment technicians	Low Income	West	Other Health Workers
55	Radiologist	319	371	436	0.18	55,554	Niger	Minimum Estimate	2212 - Specialist medical practitioners	Low Income	West	Medical Doctors
56	Registered General Nurse / State Certified Nurse	42,342	47,268	53,038	21.83	458	Niger	Minimum Estimate	2221 - Nursing professionals	Low Income	West	Nursing Personnel
57	Renal Nurse	1,424	1,680	2,015	0.83	11,984	Niger	Minimum Estimate	2221 - Nursing professionals	Low Income	West	Nursing Personnel
58	Respiratory Physician	105	122	142	0.06	171,799	Niger	Minimum Estimate	2212 - Specialist medical practitioners	Low Income	West	Medical Doctors
59	Rheumatologist	45	52	61	0.02	400,289	Niger	Minimum Estimate	2212 - Specialist medical practitioners	Low Income	West	Medical Doctors
60	Speech Therapist	169	197	228	0.09	106,761	Niger	Minimum Estimate	2266 - Audiologists and speech therapists	Low Income	West	Other Health Workers
61	Urologist	13	16	20	0.01	1,201,349	Niger	Minimum Estimate	2212 - Specialist medical practitioners	Low Income	West	Medical Doctors
1	Anaesthesiologist	1,316	1,478	1,656	0.08	125,506	Nigeria	Minimum Estimate	2212 - Specialist medical practitioners	Lower-middle Income	West	Medical Doctors
2	Associate Nurse/Enrolled Nurse/Nursing Assistant	207,435	224,990	243,809	11.74	852	Nigeria	Minimum Estimate	3221 - Nursing associate professionals	Lower-middle Income	West	Nursing Personnel
3	Audiologist	531	587	645	0.03	322,545	Nigeria	Minimum Estimate	2266 - Audiologists and speech therapists	Lower-middle Income	West	Other Health Workers
4	Cardiologist	655	734	820	0.04	253,771	Nigeria	Minimum Estimate	2212 - Specialist medical practitioners	Lower-middle Income	West	Medical Doctors
5	Cardiothoracic Surgeon	179	201	224	0.01	927,451	Nigeria	Minimum Estimate	2212 - Specialist medical practitioners	Lower-middle Income	West	Medical Doctors
6	Clinical Officer/Physician Assistant	24,983	25,879	26,905	1.30	7,685	Nigeria	Minimum Estimate	3256 - Medical assistants	Lower-middle Income	West	Other Health Workers
7	Clinical Pharmacist	4,253	4,649	5,108	0.25	40,595	Nigeria	Minimum Estimate	2262 - Pharmacists	Lower-middle Income	West	Pharmacist
8	Clinical Psychologist	8,781	9,644	10,651	0.51	19,467	Nigeria	Minimum Estimate	2634 - Psychologists	Lower-middle Income	West	Other Health Workers
9	Community health worker/Village health worker	139,280	147,449	155,877	7.52	1,330	Nigeria	Minimum Estimate	3253 - Community health workers	Lower-middle Income	West	Other Health Workers
10	Dental Surgery Assistant	12,446	13,828	15,368	0.74	13,519	Nigeria	Minimum Estimate	3251 - Dental assistants and therapists	Lower-middle Income	West	Other Health Workers
11	Dental Therapist	7,837	8,703	9,664	0.47	21,502	Nigeria	Minimum Estimate	3251 - Dental assistants and therapists	Lower-middle Income	West	Other Health Workers
12	Dentist	8,268	9,256	10,456	0.50	19,833	Nigeria	Minimum Estimate	2261 - Dentists	Lower-middle Income	West	Dentist
13	Dermatologist	563	618	676	0.03	307,750	Nigeria	Minimum Estimate	2212 - Specialist medical practitioners	Lower-middle Income	West	Medical Doctors
14	Endocrinologist	335	391	463	0.02	445,398	Nigeria	Minimum Estimate	2212 - Specialist medical practitioners	Lower-middle Income	West	Medical Doctors
15	ENT Surgeon	1,681	1,875	2,087	0.10	99,535	Nigeria	Minimum Estimate	2212 - Specialist medical practitioners	Lower-middle Income	West	Medical Doctors

S/N	Health Professionals	2022	2026	2030	Density per 10,000 population	Required Population ratio (1 professional is to xxx population)	Name of Country	Modelling Scenario	ISCO-08 Match	Income Group Classification	Sub-Regional Grouping	SDG 3c Occupation
16	Environmental Health Officer	5,120	5,620	6,145	0.30	33,852	Nigeria	Minimum Estimate	2263 - Environmental and occupational health and hygiene professionals	Lower-middle Income	West	Other Health Workers
17	Gastroenterologist	1,941	1,966	2,022	0.10	101,921	Nigeria	Minimum Estimate	2212 - Specialist medical practitioners	Lower-middle Income	West	Medical Doctors
18	General Medical Practitioner (Generalist Doctor)	65,722	71,577	77,884	3.75	2,666	Nigeria	Minimum Estimate	2211 - Generalist medical practitioners	Lower-middle Income	West	Medical Doctors
19	General Surgeon	1,107	1,221	1,359	0.07	152,504	Nigeria	Minimum Estimate	2212 - Specialist medical practitioners	Lower-middle Income	West	Medical Doctors
20	Haematologist	769	864	967	0.05	215,186	Nigeria	Minimum Estimate	2212 - Specialist medical practitioners	Lower-middle Income	West	Medical Doctors
21	Health Promoter/Health Educator	1,376	1,430	1,490	0.07	138,943	Nigeria	Minimum Estimate	2269 - Health professionals not elsewhere classified	Lower-middle Income	West	Other Health Workers
22	Infectious Diseases Specialist	149	162	176	0.01	1,174,556	Nigeria	Minimum Estimate	2212 - Specialist medical practitioners	Lower-middle Income	West	Medical Doctors
23	Intensive Care Nurse	4,819	5,246	5,693	0.27	36,509	Nigeria	Minimum Estimate	2221 - Nursing professionals	Lower-middle Income	West	Nursing Personnel
24	Medical Laboratory Scientist	17,128	18,423	19,952	0.96	10,376	Nigeria	Minimum Estimate	3212 - Medical and pathology laboratory technicians	Lower-middle Income	West	Other Health Workers
25	Medical Laboratory Technician	24,051	24,417	24,934	1.21	8,266	Nigeria	Minimum Estimate	3212 - Medical and pathology laboratory technicians	Lower-middle Income	West	Other Health Workers
26	Medical Social Worker	7,673	7,967	8,265	0.40	25,064	Nigeria	Minimum Estimate	1344 - Social welfare managers	Lower-middle Income	West	Other Health Workers
27	Mental Health Nurse	6,250	6,695	7,262	0.35	28,484	Nigeria	Minimum Estimate	2221 - Nursing professionals	Lower-middle Income	West	Nursing Personnel
28	Midwife	118,986	128,234	138,459	6.67	1,498	Nigeria	Minimum Estimate	2222 - Midwifery professionals	Lower-middle Income	West	Midwifery Personnel
29	Nephrologist	1,346	1,505	1,709	0.08	120,916	Nigeria	Minimum Estimate	2212 - Specialist medical practitioners	Lower-middle Income	West	Medical Doctors
30	Neuro-Surgeon	411	463	522	0.03	397,456	Nigeria	Minimum Estimate	2212 - Specialist medical practitioners	Lower-middle Income	West	Medical Doctors
31	Nurse Anaesthetist	2,446	2,777	3,146	0.15	66,033	Nigeria	Minimum Estimate	2221 - Nursing professionals	Lower-middle Income	West	Nursing Personnel
32	Nutritionist	29,260	30,870	32,430	1.56	6,403	Nigeria	Minimum Estimate	2265 - Dieticians and nutritionists	Lower-middle Income	West	Other Health Workers
33	Obstetrician & Gynaecologist	10,285	11,361	12,536	0.60	16,579	Nigeria	Minimum Estimate	2212 - Specialist medical practitioners	Lower-middle Income	West	Medical Doctors
34	Occupational Therapist	2,880	3,227	3,629	0.17	57,155	Nigeria	Minimum Estimate	2269 - Health professionals not elsewhere classified	Lower-middle Income	West	Other Health Workers
35	Oncology Nurse	973	1,092	1,241	0.06	166,803	Nigeria	Minimum Estimate	2221 - Nursing professionals	Lower-middle Income	West	Nursing Personnel
36	Operating Theatre Nurse	18,075	20,036	22,478	1.09	9,206	Nigeria	Minimum Estimate	2221 - Nursing professionals	Lower-middle Income	West	Nursing Personnel
37	Ophthalmic Nurse	1,676	1,855	2,057	0.10	100,911	Nigeria	Minimum Estimate	2221 - Nursing professionals	Lower-middle Income	West	Nursing Personnel
38	Ophthalmologist	467	521	585	0.03	354,416	Nigeria	Minimum Estimate	2212 - Specialist medical practitioners	Lower-middle Income	West	Medical Doctors
39	Optometrist	1,752	1,943	2,152	0.10	96,539	Nigeria	Minimum Estimate	2267 - Optometrists and ophthalmic opticians	Lower-middle Income	West	Other Health Workers
40	Orthopaedic Nurse	1,285	1,452	1,635	0.08	127,189	Nigeria	Minimum Estimate	2221 - Nursing professionals	Lower-middle Income	West	Nursing Personnel
41	Orthopaedic Surgeon	5,165	5,797	6,514	0.31	31,873	Nigeria	Minimum Estimate	2212 - Specialist medical practitioners	Lower-middle Income	West	Medical Doctors
42	Orthopaedic Technologist	4,932	5,455	6,011	0.29	34,601	Nigeria	Minimum Estimate	3214 - Medical and dental prosthetic technicians	Lower-middle Income	West	Other Health Workers
43	Paediatric Nurse	21,555	23,568	25,547	1.23	8,138	Nigeria	Minimum Estimate	2221 - Nursing professionals	Lower-middle Income	West	Nursing Personnel
44	Paediatric Surgeon	582	637	687	0.03	302,816	Nigeria	Minimum Estimate	2212 - Specialist medical practitioners	Lower-middle Income	West	Medical Doctors
45	Paediatrician	7,620	8,413	9,282	0.45	22,375	Nigeria	Minimum Estimate	2212 - Specialist medical practitioners	Lower-middle Income	West	Medical Doctors
46	Pathologist	878	976	1,103	0.05	187,460	Nigeria	Minimum Estimate	2212 - Specialist medical practitioners	Lower-middle Income	West	Medical Doctors
47	Pharmacist	17,962	18,731	19,511	0.94	10,623	Nigeria	Minimum Estimate	2262 - Pharmacists	Lower-middle Income	West	Pharmacist
48	Pharmacy Technician	19,248	20,981	22,860	1.10	9,085	Nigeria	Minimum Estimate	3213 - Pharmaceutical technicians and assistants	Lower-middle Income	West	Other Health Workers
49	Physician	16,859	18,448	20,232	0.98	10,255	Nigeria	Minimum Estimate	2212 - Specialist medical practitioners	Lower-middle Income	West	Medical Doctors
50	Physiotherapist	3,666	4,049	4,468	0.22	46,500	Nigeria	Minimum Estimate	2264 - Physiotherapists	Lower-middle Income	West	Other Health Workers
51	Plastic Surgeon	824	937	1,062	0.05	195,692	Nigeria	Minimum Estimate	2212 - Specialist medical practitioners	Lower-middle Income	West	Medical Doctors
52	Psychiatrist	7,473	8,017	8,676	0.42	23,869	Nigeria	Minimum Estimate	2212 - Specialist medical practitioners	Lower-middle Income	West	Medical Doctors
53	Radiation Oncologist	220	250	285	0.01	728,292	Nigeria	Minimum Estimate	2212 - Specialist medical practitioners	Lower-middle Income	West	Medical Doctors
54	Radiographer (Diagnostics and Therapy)	11,322	12,551	14,013	0.68	14,791	Nigeria	Minimum Estimate	3211 - Medical imaging and therapeutic equipment technicians	Lower-middle Income	West	Other Health Workers
55	Radiologist	3,542	3,906	4,324	0.21	47,977	Nigeria	Minimum Estimate	2212 - Specialist medical practitioners	Lower-middle Income	West	Medical Doctors
56	Registered General Nurse / State Certified Nurse	367,917	404,605	444,720	21.42	467	Nigeria	Minimum Estimate	2221 - Nursing professionals	Lower-middle Income	West	Nursing Personnel
57	Renal Nurse	16,341	18,277	20,771	1.01	9,950	Nigeria	Minimum Estimate	2221 - Nursing professionals	Lower-middle Income	West	Nursing Personnel
58	Respiratory Physician	1,096	1,197	1,304	0.06	159,412	Nigeria	Minimum Estimate	2212 - Specialist medical practitioners	Lower-middle Income	West	Medical Doctors
59	Rheumatologist	445	494	546	0.03	380,903	Nigeria	Minimum Estimate	2212 - Specialist medical practitioners	Lower-middle Income	West	Medical Doctors
60	Speech Therapist	1,481	1,633	1,798	0.09	115,556	Nigeria	Minimum Estimate	2266 - Audiologists and speech therapists	Lower-middle Income	West	Other Health Workers
61	Urologist	134	145	159	0.01	1,301,734	Nigeria	Minimum Estimate	2212 - Specialist medical practitioners	Lower-middle Income	West	Medical Doctors
1	Anaesthesiologist	111	126	144	0.11	90,796	Rwanda	Minimum Estimate	2212 - Specialist medical practitioners	Low Income	East	Medical Doctors
2	Associate Nurse/Enrolled Nurse/Nursing Assistant	10,195	11,352	12,783	9.92	1,008	Rwanda	Minimum Estimate	3221 - Nursing associate professionals	Low Income	East	Nursing Personnel
3	Audiologist	33	37	41	0.03	317,817	Rwanda	Minimum Estimate	2266 - Audiologists and speech therapists	Low Income	East	Other Health Workers
4	Cardiologist	47	56	67	0.05	194,765	Rwanda	Minimum Estimate	2212 - Specialist medical practitioners	Low Income	East	Medical Doctors
5	Cardiothoracic Surgeon	19	21	24	0.02	555,479	Rwanda	Minimum Estimate	2212 - Specialist medical practitioners	Low Income	East	Medical Doctors
6	Clinical Officer/Physician Assistant	1,285	1,549	2,041	1.72	5,831	Rwanda	Minimum Estimate	3256 - Medical assistants	Low Income	East	Other Health Workers
7	Clinical Pharmacist	248	273	304	0.23	42,700	Rwanda	Minimum Estimate	2262 - Pharmacists	Low Income	East	Pharmacist
8	Clinical Psychologist	629	673	724	0.56	18,011	Rwanda	Minimum Estimate	2634 - Psychologists	Low Income	East	Other Health Workers
9	Community health worker/Village health worker	6,681	8,338	11,181	9.41	1,063	Rwanda	Minimum Estimate	3253 - Community health workers	Low Income	East	Other Health Workers
10	Dental Surgery Assistant	915	1,034	1,173	0.90	11,135	Rwanda	Minimum Estimate	3251 - Dental assistants and therapists	Low Income	East	Other Health Workers
11	Dental Therapist	570	645	733	0.56	17,828	Rwanda	Minimum Estimate	3251 - Dental assistants and therapists	Low Income	East	Other Health Workers
12	Dentist	723	801	899	0.69	14,543	Rwanda	Minimum Estimate	2261 - Dentists	Low Income	East	Dentist
13	Dermatologist	34	38	42	0.03	312,091	Rwanda	Minimum Estimate	2212 - Specialist medical practitioners	Low Income	East	Medical Doctors
14	Endocrinologist	33	40	49	0.04	263,833	Rwanda	Minimum Estimate	2212 - Specialist medical practitioners	Low Income	East	Medical Doctors
15	ENT Surgeon	105	121	141	0.11	91,883	Rwanda	Minimum Estimate	2212 - Specialist medical practitioners	Low Income	East	Medical Doctors
16	Environmental Health Officer	322	352	382	0.29	34,209	Rwanda	Minimum Estimate	2263 - Environmental and occupational health and hygiene professionals	Low Income	East	Other Health Workers
17	Gastroenterologist	57	58	60	0.05	213,870	Rwanda	Minimum Estimate	2212 - Specialist medical practitioners	Low Income	East	Medical Doctors
18	General Medical Practitioner (Generalist Doctor)	3,272	3,670	4,213	3.31	3,018	Rwanda	Minimum Estimate	2211 - Generalist medical practitioners	Low Income	East	Medical Doctors
19	General Surgeon	73	86	102	0.08	127,055	Rwanda	Minimum Estimate	2212 - Specialist medical practitioners	Low Income	East	Medical Doctors
20	Haematologist	23	25	27	0.02	486,504	Rwanda	Minimum Estimate	2212 - Specialist medical practitioners	Low Income	East	Medical Doctors
21	Health Promoter/Health Educator	62	72	91	0.08	130,882	Rwanda	Minimum Estimate	2269 - Health professionals not elsewhere classified	Low Income	East	Other Health Workers
22	Infectious Diseases Specialist	8	9	10	0.01	1,325,328	Rwanda	Minimum Estimate	2212 - Specialist medical practitioners	Low Income	East	Medical Doctors
23	Intensive Care Nurse	268	293	320	0.25	40,763	Rwanda	Minimum Estimate	2221 - Nursing professionals	Low Income	East	Nursing Personnel
24	Medical Laboratory Scientist	1,030	1,220	1,516	1.23	8,162	Rwanda	Minimum Estimate	3212 - Medical and pathology laboratory technicians	Low Income	East	Other Health Workers
25	Medical Laboratory Technician	1,353	1,869	2,802	2.43	4,115	Rwanda	Minimum Estimate	3212 - Medical and pathology laboratory technicians	Low Income	East	Other Health Workers
26	Medical Social Worker	237	248	260	0.20	48,988	Rwanda	Minimum Estimate	1344 - Social welfare managers	Low Income	East	Other Health Workers
27	Mental Health Nurse	519	585	671	0.52	19,398	Rwanda	Minimum Estimate	2221 - Nursing professionals	Low Income	East	Nursing Personnel
28	Midwife	7,436	8,043	8,707	6.66	1,501	Rwanda	Minimum Estimate	2222 - Midwifery professionals	Low Income	East	Midwifery Personnel
29	Nephrologist	94	109	130	0.10	99,949	Rwanda	Minimum Estimate	2212 - Specialist medical practitioners	Low Income	East	Medical Doctors
30	Neuro-Surgeon	40	46	54	0.04	235,227	Rwanda	Minimum Estimate	2212 - Specialist medical practitioners	Low Income	East	Medical Doctors
31	Nurse Anaesthetist	239	285	339	0.26	38,311	Rwanda	Minimum Estimate	2221 - Nursing professionals	Low Income	East	Nursing Personnel
32	Nutritionist	813	839	860	0.66	15,156	Rwanda	Minimum Estimate	2265 - Dieticians and nutritionists	Low Income	East	Other Health Workers
33	Obstetrician & Gynaecologist	722	795	879	0.68	14,757	Rwanda	Minimum Estimate	2212 - Specialist medical practitioners	Low Income	East	Medical Doctors
34	Occupational Therapist	209	240	278	0.21	46,839	Rwanda	Minimum Estimate	2269 - Health professionals not elsewhere classified	Low Income	East	Other Health Workers
35	Oncology Nurse	79	94	113	0.09	114,605	Rwanda	Minimum Estimate	2221 - Nursing professionals	Low Income	East	Nursing Personnel
36	Operating Theatre Nurse	1,269	1,461	1,716	1.32	7,548	Rwanda	Minimum Estimate	2221 - Nursing professionals	Low Income	East	Nursing Personnel
37	Ophthalmic Nurse	94	108	124	0.10	104,598	Rwanda	Minimum Estimate	2221 - Nursing professionals	Low Income	East	Nursing Personnel
38	Ophthalmologist	32	38	45	0.03	290,043	Rwanda	Minimum Estimate	2212 - Specialist medical practitioners	Low Income	East	Medical Doctors

S/N	Health Professionals	2022	2026	2030	Density per 10,000 population	Required Population ratio (1 professional is to xxx population)	Name of Country	Modelling Scenario	ISCO-08 Match	Income Group Classification	Sub-Regional Grouping	SDG 3c Occupation
39	Optometrist	137	161	189	0.15	68,816	Rwanda	Minimum Estimate	2267 - Optometrists and ophthalmic opticians	Low Income	East	Other Health Workers
40	Orthopaedic Nurse	71	83	97	0.07	133,830	Rwanda	Minimum Estimate	2221 - Nursing professionals	Low Income	East	Nursing Personnel
41	Orthopaedic Surgeon	188	228	281	0.22	45,373	Rwanda	Minimum Estimate	2212 - Specialist medical practitioners	Low Income	East	Medical Doctors
42	Orthopaedic Technologist	322	357	394	0.30	33,173	Rwanda	Minimum Estimate	3214 - Medical and dental prosthetic technicians	Low Income	East	Other Health Workers
43	Paediatric Nurse	892	942	975	0.75	13,398	Rwanda	Minimum Estimate	2221 - Nursing professionals	Low Income	East	Nursing Personnel
44	Paediatric Surgeon	29	31	33	0.03	399,693	Rwanda	Minimum Estimate	2212 - Specialist medical practitioners	Low Income	East	Medical Doctors
45	Paediatrician	577	602	629	0.48	20,670	Rwanda	Minimum Estimate	2212 - Specialist medical practitioners	Low Income	East	Medical Doctors
46	Pathologist	83	93	105	0.08	123,499	Rwanda	Minimum Estimate	2212 - Specialist medical practitioners	Low Income	East	Medical Doctors
47	Pharmacist	785	792	809	0.63	15,930	Rwanda	Minimum Estimate	2262 - Pharmacists	Low Income	East	Pharmacist
48	Pharmacy Technician	926	1,007	1,102	0.85	11,799	Rwanda	Minimum Estimate	3213 - Pharmaceutical technicians and assistants	Low Income	East	Other Health Workers
49	Physician	863	975	1,121	0.87	11,461	Rwanda	Minimum Estimate	2212 - Specialist medical practitioners	Low Income	East	Medical Doctors
50	Physiotherapist	239	267	299	0.23	43,577	Rwanda	Minimum Estimate	2264 - Physiotherapists	Low Income	East	Other Health Workers
51	Plastic Surgeon	136	156	179	0.14	73,066	Rwanda	Minimum Estimate	2212 - Specialist medical practitioners	Low Income	East	Medical Doctors
52	Psychiatrist	478	532	602	0.46	21,620	Rwanda	Minimum Estimate	2212 - Specialist medical practitioners	Low Income	East	Medical Doctors
53	Radiation Oncologist	26	32	39	0.03	328,198	Rwanda	Minimum Estimate	2212 - Specialist medical practitioners	Low Income	East	Medical Doctors
54	Radiographer (Diagnostics and Therapy)	631	722	839	0.65	15,403	Rwanda	Minimum Estimate	3211 - Medical imaging and therapeutic equipment technicians	Low Income	East	Other Health Workers
55	Radiologist	173	200	237	0.19	53,258	Rwanda	Minimum Estimate	2212 - Specialist medical practitioners	Low Income	East	Medical Doctors
56	Registered General Nurse / State Certified Nurse	20,638	22,789	25,294	19.47	514	Rwanda	Minimum Estimate	2221 - Nursing professionals	Low Income	East	Nursing Personnel
57	Renal Nurse	1,171	1,367	1,626	1.25	7,969	Rwanda	Minimum Estimate	2221 - Nursing professionals	Low Income	East	Nursing Personnel
58	Respiratory Physician	136	139	142	0.11	91,557	Rwanda	Minimum Estimate	2212 - Specialist medical practitioners	Low Income	East	Medical Doctors
59	Rheumatologist	31	35	40	0.03	329,660	Rwanda	Minimum Estimate	2212 - Specialist medical practitioners	Low Income	East	Medical Doctors
60	Speech Therapist	97	107	120	0.09	108,452	Rwanda	Minimum Estimate	2266 - Audiologists and speech therapists	Low Income	East	Other Health Workers
61	Urologist	10	13	16	0.01	786,124	Rwanda	Minimum Estimate	2212 - Specialist medical practitioners	Low Income	East	Medical Doctors
1	Anaesthesiologist	2	2	3	0.11	87,509	Sao Tome and Princ	Minimum Estimate	2212 - Specialist medical practitioners	Lower-middle Income	West	Medical Doctors
2	Associate Nurse/Enrolled Nurse/Nursing Assistant	203	222	245	11.14	898	Sao Tome and Princ	Minimum Estimate	3221 - Nursing associate professionals	Lower-middle Income	West	Nursing Personnel
3	Audiologist	1	1	1	0.03	335,785	Sao Tome and Princ	Minimum Estimate	2266 - Audiologists and speech therapists	Lower-middle Income	West	Other Health Workers
4	Cardiologist	1	1	1	0.05	182,194	Sao Tome and Princ	Minimum Estimate	2212 - Specialist medical practitioners	Lower-middle Income	West	Medical Doctors
5	Cardiothoracic Surgeon	0	0	0	0.02	525,467	Sao Tome and Princ	Minimum Estimate	2212 - Specialist medical practitioners	Lower-middle Income	West	Medical Doctors
6	Clinical Officer/Physician Assistant	13	14	15	0.68	14,679	Sao Tome and Princ	Minimum Estimate	3256 - Medical assistants	Lower-middle Income	West	Other Health Workers
7	Clinical Pharmacist	5	5	6	0.27	36,983	Sao Tome and Princ	Minimum Estimate	2262 - Pharmacists	Lower-middle Income	West	Pharmacist
8	Clinical Psychologist	12	13	15	0.67	15,032	Sao Tome and Princ	Minimum Estimate	2634 - Psychologists	Lower-middle Income	West	Other Health Workers
9	Community health worker/Village health worker	94	98	102	4.63	2,160	Sao Tome and Princ	Minimum Estimate	3253 - Community health workers	Lower-middle Income	West	Other Health Workers
10	Dental Surgery Assistant	19	21	24	1.09	9,186	Sao Tome and Princ	Minimum Estimate	3251 - Dental assistants and therapists	Lower-middle Income	West	Other Health Workers
11	Dental Therapist	12	13	15	0.69	14,498	Sao Tome and Princ	Minimum Estimate	3251 - Dental assistants and therapists	Lower-middle Income	West	Other Health Workers
12	Dentist	12	13	14	0.65	15,310	Sao Tome and Princ	Minimum Estimate	2261 - Dentists	Lower-middle Income	West	Dentist
13	Dermatologist	1	1	1	0.04	282,424	Sao Tome and Princ	Minimum Estimate	2212 - Specialist medical practitioners	Lower-middle Income	West	Medical Doctors
14	Endocrinologist	1	1	1	0.04	223,645	Sao Tome and Princ	Minimum Estimate	2212 - Specialist medical practitioners	Lower-middle Income	West	Medical Doctors
15	ENT Surgeon	2	2	2	0.11	95,171	Sao Tome and Princ	Minimum Estimate	2212 - Specialist medical practitioners	Lower-middle Income	West	Medical Doctors
16	Environmental Health Officer	5	6	6	0.29	34,819	Sao Tome and Princ	Minimum Estimate	2263 - Environmental and occupational health and hygiene professionals	Lower-middle Income	West	Other Health Workers
17	Gastroenterologist	2	2	2	0.07	139,773	Sao Tome and Princ	Minimum Estimate	2212 - Specialist medical practitioners	Lower-middle Income	West	Medical Doctors
18	General Medical Practitioner (Generalist Doctor)	60	65	72	3.25	3,073	Sao Tome and Princ	Minimum Estimate	2211 - Generalist medical practitioners	Lower-middle Income	West	Medical Doctors
19	General Surgeon	2	2	2	0.10	102,403	Sao Tome and Princ	Minimum Estimate	2212 - Specialist medical practitioners	Lower-middle Income	West	Medical Doctors
20	Haematologist	1	1	1	0.03	317,236	Sao Tome and Princ	Minimum Estimate	2212 - Specialist medical practitioners	Lower-middle Income	West	Medical Doctors
21	Health Promoter/Health Educator	1	1	1	0.04	278,225	Sao Tome and Princ	Minimum Estimate	2269 - Health professionals not elsewhere classified	Lower-middle Income	West	Other Health Workers
22	Infectious Diseases Specialist	0	0	0	0.01	1,015,487	Sao Tome and Princ	Minimum Estimate	2212 - Specialist medical practitioners	Lower-middle Income	West	Medical Doctors
23	Intensive Care Nurse	4	5	5	0.22	44,528	Sao Tome and Princ	Minimum Estimate	2221 - Nursing professionals	Lower-middle Income	West	Nursing Personnel
24	Medical Laboratory Scientist	17	18	20	0.92	10,868	Sao Tome and Princ	Minimum Estimate	3212 - Medical and pathology laboratory technicians	Lower-middle Income	West	Other Health Workers
25	Medical Laboratory Technician	21	27	36	1.68	5,947	Sao Tome and Princ	Minimum Estimate	3212 - Medical and pathology laboratory technicians	Lower-middle Income	West	Other Health Workers
26	Medical Social Worker	5	5	5	0.23	44,217	Sao Tome and Princ	Minimum Estimate	1344 - Social welfare managers	Lower-middle Income	West	Other Health Workers
27	Mental Health Nurse	7	8	9	0.41	24,284	Sao Tome and Princ	Minimum Estimate	2221 - Nursing professionals	Lower-middle Income	West	Nursing Personnel
28	Midwife	139	162	196	9.11	1,098	Sao Tome and Princ	Minimum Estimate	2222 - Midwifery professionals	Lower-middle Income	West	Midwifery Personnel
29	Nephrologist	2	2	3	0.13	77,270	Sao Tome and Princ	Minimum Estimate	2212 - Specialist medical practitioners	Lower-middle Income	West	Medical Doctors
30	Neuro-Surgeon	1	1	1	0.06	179,396	Sao Tome and Princ	Minimum Estimate	2212 - Specialist medical practitioners	Lower-middle Income	West	Medical Doctors
31	Nurse Anaesthetist	5	6	7	0.30	32,853	Sao Tome and Princ	Minimum Estimate	2221 - Nursing professionals	Lower-middle Income	West	Nursing Personnel
32	Nutritionist	15	15	15	0.68	14,776	Sao Tome and Princ	Minimum Estimate	2265 - Dieticians and nutritionists	Lower-middle Income	West	Other Health Workers
33	Obstetrician & Gynaecologist	12	13	14	0.65	15,272	Sao Tome and Princ	Minimum Estimate	2212 - Specialist medical practitioners	Lower-middle Income	West	Medical Doctors
34	Occupational Therapist	4	5	6	0.27	37,318	Sao Tome and Princ	Minimum Estimate	2269 - Health professionals not elsewhere classified	Lower-middle Income	West	Other Health Workers
35	Oncology Nurse	2	2	2	0.11	93,904	Sao Tome and Princ	Minimum Estimate	2221 - Nursing professionals	Lower-middle Income	West	Nursing Personnel
36	Operating Theatre Nurse	21	23	27	1.26	7,961	Sao Tome and Princ	Minimum Estimate	2221 - Nursing professionals	Lower-middle Income	West	Nursing Personnel
37	Ophthalmic Nurse	2	2	3	0.12	80,005	Sao Tome and Princ	Minimum Estimate	2221 - Nursing professionals	Lower-middle Income	West	Nursing Personnel
38	Ophthalmologist	1	1	1	0.04	266,405	Sao Tome and Princ	Minimum Estimate	2212 - Specialist medical practitioners	Lower-middle Income	West	Medical Doctors
39	Optometrist	2	3	3	0.13	74,362	Sao Tome and Princ	Minimum Estimate	2267 - Optometrists and ophthalmic opticians	Lower-middle Income	West	Other Health Workers
40	Orthopaedic Nurse	2	3	3	0.15	65,848	Sao Tome and Princ	Minimum Estimate	2221 - Nursing professionals	Lower-middle Income	West	Nursing Personnel
41	Orthopaedic Surgeon	7	8	9	0.42	24,038	Sao Tome and Princ	Minimum Estimate	2212 - Specialist medical practitioners	Lower-middle Income	West	Medical Doctors
42	Orthopaedic Technologist	6	7	8	0.34	29,040	Sao Tome and Princ	Minimum Estimate	3214 - Medical and dental prosthetic technicians	Lower-middle Income	West	Other Health Workers
43	Paediatric Nurse	16	17	18	0.82	12,222	Sao Tome and Princ	Minimum Estimate	2221 - Nursing professionals	Lower-middle Income	West	Nursing Personnel
44	Paediatric Surgeon	0	0	0	0.02	464,199	Sao Tome and Princ	Minimum Estimate	2212 - Specialist medical practitioners	Lower-middle Income	West	Medical Doctors
45	Paediatrician	8	9	9	0.43	23,259	Sao Tome and Princ	Minimum Estimate	2212 - Specialist medical practitioners	Lower-middle Income	West	Medical Doctors
46	Pathologist	1	1	2	0.07	136,873	Sao Tome and Princ	Minimum Estimate	2212 - Specialist medical practitioners	Lower-middle Income	West	Medical Doctors
47	Pharmacist	10	10	10	0.48	20,956	Sao Tome and Princ	Minimum Estimate	2262 - Pharmacists	Lower-middle Income	West	Pharmacist
48	Pharmacy Technician	18	20	22	1.00	9,993	Sao Tome and Princ	Minimum Estimate	3213 - Pharmaceutical technicians and assistants	Lower-middle Income	West	Other Health Workers
49	Physician	19	22	25	1.15	8,683	Sao Tome and Princ	Minimum Estimate	2212 - Specialist medical practitioners	Lower-middle Income	West	Medical Doctors
50	Physiotherapist	5	5	6	0.26	39,110	Sao Tome and Princ	Minimum Estimate	2264 - Physiotherapists	Lower-middle Income	West	Other Health Workers
51	Plastic Surgeon	1	2	2	0.09	114,575	Sao Tome and Princ	Minimum Estimate	2212 - Specialist medical practitioners	Lower-middle Income	West	Medical Doctors
52	Psychiatrist	7	8	9	0.42	24,077	Sao Tome and Princ	Minimum Estimate	2212 - Specialist medical practitioners	Lower-middle Income	West	Medical Doctors
53	Radiation Oncologist	1	1	1	0.04	268,350	Sao Tome and Princ	Minimum Estimate	2212 - Specialist medical practitioners	Lower-middle Income	West	Medical Doctors
54	Radiographer (Diagnostics and Therapy)	14	16	19	0.85	11,776	Sao Tome and Princ	Minimum Estimate	3211 - Medical imaging and therapeutic equipment technicians	Lower-middle Income	West	Other Health Workers
55	Radiologist	4	5	5	0.25	40,546	Sao Tome and Princ	Minimum Estimate	2212 - Specialist medical practitioners	Lower-middle Income	West	Medical Doctors
56	Registered General Nurse / State Certified Nurse	412	459	518	23.64	423	Sao Tome and Princ	Minimum Estimate	2221 - Nursing professionals	Lower-middle Income	West	Nursing Personnel
57	Renal Nurse	26	30	35	1.61	6,218	Sao Tome and Princ	Minimum Estimate	2221 - Nursing professionals	Lower-middle Income	West	Nursing Personnel
58	Respiratory Physician	2	2	2	0.08	118,836	Sao Tome and Princ	Minimum Estimate	2212 - Specialist medical practitioners	Lower-middle Income	West	Medical Doctors
59	Rheumatologist	1	1	1	0.03	337,246	Sao Tome and Princ	Minimum Estimate	2212 - Specialist medical practitioners	Lower-middle Income	West	Medical Doctors
60	Speech Therapist	2	2	2	0.10	104,192	Sao Tome and Princ	Minimum Estimate	2266 - Audiologists and speech therapists	Lower-middle Income	West	Other Health Workers
61	Urologist	0	0	0	0.01	740,359	Sao Tome and Princ	Minimum Estimate	2212 - Specialist medical practitioners	Lower-middle Income	West	Medical Doctors

S/N	Health Professionals	2022	2026	2030	Density per 10,000 population	Required Population ratio (1 professional is to xxx population)	Name of Country	Modelling Scenario	ISCO-08 Match	Income Group Classification	Sub-Regional Grouping	SDG 3c Occupation
1	Anaesthesiologist	122	139	159	0.09	105,648	Senegal	Minimum Estimate	2212 - Specialist medical practitioners	Lower-middle Income	West	Medical Doctors
2	Associate Nurse/Enrolled Nurse/Nursing Assistant	17,495	20,224	23,460	13.98	715	Senegal	Minimum Estimate	3221 - Nursing associate professionals	Lower-middle Income	West	Nursing Personnel
3	Audiologist	41	46	52	0.03	326,958	Senegal	Minimum Estimate	2266 - Audiologists and speech therapists	Lower-middle Income	West	Other Health Workers
4	Cardiologist	58	65	74	0.04	228,778	Senegal	Minimum Estimate	2212 - Specialist medical practitioners	Lower-middle Income	West	Medical Doctors
5	Cardiothoracic Surgeon	19	22	25	0.01	684,441	Senegal	Minimum Estimate	2212 - Specialist medical practitioners	Lower-middle Income	West	Medical Doctors
6	Clinical Officer/Physician Assistant	1,096	1,153	1,230	0.73	13,607	Senegal	Minimum Estimate	3256 - Medical assistants	Lower-middle Income	West	Other Health Workers
7	Clinical Pharmacist	321	357	402	0.24	41,781	Senegal	Minimum Estimate	2262 - Pharmacists	Lower-middle Income	West	Pharmacist
8	Clinical Psychologist	688	764	854	0.51	19,690	Senegal	Minimum Estimate	2634 - Psychologists	Lower-middle Income	West	Other Health Workers
9	Community health worker/Village health worker	9,242	9,927	10,624	6.31	1,586	Senegal	Minimum Estimate	3253 - Community health workers	Lower-middle Income	West	Other Health Workers
10	Dental Surgery Assistant	1,371	1,526	1,711	1.02	9,836	Senegal	Minimum Estimate	3251 - Dental assistants and therapists	Lower-middle Income	West	Other Health Workers
11	Dental Therapist	856	954	1,070	0.64	15,731	Senegal	Minimum Estimate	3251 - Dental assistants and therapists	Lower-middle Income	West	Other Health Workers
12	Dentist	1,038	1,141	1,278	0.76	13,142	Senegal	Minimum Estimate	2261 - Dentists	Lower-middle Income	West	Dentist
13	Dermatologist	47	52	58	0.03	288,720	Senegal	Minimum Estimate	2212 - Specialist medical practitioners	Lower-middle Income	West	Medical Doctors
14	Endocrinologist	57	64	75	0.04	224,306	Senegal	Minimum Estimate	2212 - Specialist medical practitioners	Lower-middle Income	West	Medical Doctors
15	ENT Surgeon	129	146	165	0.10	102,285	Senegal	Minimum Estimate	2212 - Specialist medical practitioners	Lower-middle Income	West	Medical Doctors
16	Environmental Health Officer	416	460	506	0.30	33,331	Senegal	Minimum Estimate	2263 - Environmental and occupational health and hygiene professionals	Lower-middle Income	West	Other Health Workers
17	Gastroenterologist	95	96	99	0.06	167,710	Senegal	Minimum Estimate	2212 - Specialist medical practitioners	Lower-middle Income	West	Medical Doctors
18	General Medical Practitioner (Generalist Doctor)	4,509	4,967	5,476	3.25	3,075	Senegal	Minimum Estimate	2211 - Generalist medical practitioners	Lower-middle Income	West	Medical Doctors
19	General Surgeon	128	147	170	0.10	98,566	Senegal	Minimum Estimate	2212 - Specialist medical practitioners	Lower-middle Income	West	Medical Doctors
20	Haematologist	62	69	76	0.04	222,610	Senegal	Minimum Estimate	2212 - Specialist medical practitioners	Lower-middle Income	West	Medical Doctors
21	Health Promoter/Health Educator	64	67	71	0.04	237,148	Senegal	Minimum Estimate	2269 - Health professionals not elsewhere classified	Lower-middle Income	West	Other Health Workers
22	Infectious Diseases Specialist	10	11	13	0.01	1,336,798	Senegal	Minimum Estimate	2212 - Specialist medical practitioners	Lower-middle Income	West	Medical Doctors
23	Intensive Care Nurse	353	392	434	0.26	38,862	Senegal	Minimum Estimate	2221 - Nursing professionals	Lower-middle Income	West	Nursing Personnel
24	Medical Laboratory Scientist	1,257	1,383	1,539	0.92	10,910	Senegal	Minimum Estimate	3212 - Medical and pathology laboratory technicians	Lower-middle Income	West	Other Health Workers
25	Medical Laboratory Technician	1,307	1,405	1,519	0.90	11,060	Senegal	Minimum Estimate	3212 - Medical and pathology laboratory technicians	Lower-middle Income	West	Other Health Workers
26	Medical Social Worker	405	423	440	0.26	38,107	Senegal	Minimum Estimate	1344 - Social welfare managers	Lower-middle Income	West	Other Health Workers
27	Mental Health Nurse	504	566	649	0.39	25,794	Senegal	Minimum Estimate	2221 - Nursing professionals	Lower-middle Income	West	Nursing Personnel
28	Midwife	10,002	10,939	12,007	7.13	1,403	Senegal	Minimum Estimate	2222 - Midwifery professionals	Lower-middle Income	West	Midwifery Personnel
29	Nephrologist	117	134	156	0.09	107,059	Senegal	Minimum Estimate	2212 - Specialist medical practitioners	Lower-middle Income	West	Medical Doctors
30	Neuro-Surgeon	42	49	58	0.03	290,728	Senegal	Minimum Estimate	2212 - Specialist medical practitioners	Lower-middle Income	West	Medical Doctors
31	Nurse Anaesthetist	255	301	355	0.21	47,279	Senegal	Minimum Estimate	2221 - Nursing professionals	Lower-middle Income	West	Nursing Personnel
32	Nutritionist	1,603	1,686	1,766	1.05	9,536	Senegal	Minimum Estimate	2265 - Dieticians and nutritionists	Lower-middle Income	West	Other Health Workers
33	Obstetrician & Gynaecologist	854	951	1,061	0.63	15,889	Senegal	Minimum Estimate	2212 - Specialist medical practitioners	Lower-middle Income	West	Medical Doctors
34	Occupational Therapist	297	345	403	0.24	41,644	Senegal	Minimum Estimate	2269 - Health professionals not elsewhere classified	Lower-middle Income	West	Other Health Workers
35	Oncology Nurse	92	107	126	0.08	133,166	Senegal	Minimum Estimate	2221 - Nursing professionals	Lower-middle Income	West	Nursing Personnel
36	Operating Theatre Nurse	1,386	1,584	1,845	1.10	9,066	Senegal	Minimum Estimate	2221 - Nursing professionals	Lower-middle Income	West	Nursing Personnel
37	Ophthalmic Nurse	158	177	199	0.12	84,564	Senegal	Minimum Estimate	2221 - Nursing professionals	Lower-middle Income	West	Nursing Personnel
38	Ophthalmologist	46	52	60	0.04	281,050	Senegal	Minimum Estimate	2212 - Specialist medical practitioners	Lower-middle Income	West	Medical Doctors
39	Optometrist	171	192	217	0.13	77,428	Senegal	Minimum Estimate	2267 - Optometrists and ophthalmic opticians	Lower-middle Income	West	Other Health Workers
40	Orthopaedic Nurse	176	207	242	0.14	69,717	Senegal	Minimum Estimate	2221 - Nursing professionals	Lower-middle Income	West	Nursing Personnel
41	Orthopaedic Surgeon	428	495	575	0.34	29,244	Senegal	Minimum Estimate	2212 - Specialist medical practitioners	Lower-middle Income	West	Medical Doctors
42	Orthopaedic Technologist	456	512	573	0.34	29,439	Senegal	Minimum Estimate	3214 - Medical and dental prosthetic technicians	Lower-middle Income	West	Other Health Workers
43	Paediatric Nurse	1,532	1,672	1,804	1.07	9,346	Senegal	Minimum Estimate	2221 - Nursing professionals	Lower-middle Income	West	Nursing Personnel
44	Paediatric Surgeon	39	42	45	0.03	374,629	Senegal	Minimum Estimate	2212 - Specialist medical practitioners	Lower-middle Income	West	Medical Doctors
45	Paediatrician	479	542	615	0.37	27,340	Senegal	Minimum Estimate	2212 - Specialist medical practitioners	Lower-middle Income	West	Medical Doctors
46	Pathologist	70	79	91	0.05	183,716	Senegal	Minimum Estimate	2212 - Specialist medical practitioners	Lower-middle Income	West	Medical Doctors
47	Pharmacist	794	822	857	0.51	19,543	Senegal	Minimum Estimate	2262 - Pharmacists	Lower-middle Income	West	Pharmacist
48	Pharmacy Technician	1,285	1,425	1,585	0.94	10,619	Senegal	Minimum Estimate	3213 - Pharmaceutical technicians and assistants	Lower-middle Income	West	Other Health Workers
49	Physician	1,267	1,410	1,576	0.94	10,676	Senegal	Minimum Estimate	2212 - Specialist medical practitioners	Lower-middle Income	West	Medical Doctors
50	Physiotherapist	328	369	415	0.25	40,560	Senegal	Minimum Estimate	2264 - Physiotherapists	Lower-middle Income	West	Other Health Workers
51	Plastic Surgeon	83	96	110	0.07	153,213	Senegal	Minimum Estimate	2212 - Specialist medical practitioners	Lower-middle Income	West	Medical Doctors
52	Psychiatrist	560	622	704	0.42	23,811	Senegal	Minimum Estimate	2212 - Specialist medical practitioners	Lower-middle Income	West	Medical Doctors
53	Radiation Oncologist	26	31	37	0.02	446,108	Senegal	Minimum Estimate	2212 - Specialist medical practitioners	Lower-middle Income	West	Medical Doctors
54	Radiographer (Diagnostics and Therapy)	924	1,040	1,185	0.71	14,166	Senegal	Minimum Estimate	3211 - Medical imaging and therapeutic equipment technicians	Lower-middle Income	West	Other Health Workers
55	Radiologist	284	320	363	0.22	46,255	Senegal	Minimum Estimate	2212 - Specialist medical practitioners	Lower-middle Income	West	Medical Doctors
56	Registered General Nurse / State Certified Nurse	38,506	42,788	47,863	28.54	350	Senegal	Minimum Estimate	2221 - Nursing professionals	Lower-middle Income	West	Nursing Personnel
57	Renal Nurse	1,462	1,667	1,947	1.17	8,578	Senegal	Minimum Estimate	2221 - Nursing professionals	Lower-middle Income	West	Nursing Personnel
58	Respiratory Physician	71	83	96	0.06	176,040	Senegal	Minimum Estimate	2212 - Specialist medical practitioners	Lower-middle Income	West	Medical Doctors
59	Rheumatologist	37	42	47	0.03	356,510	Senegal	Minimum Estimate	2212 - Specialist medical practitioners	Lower-middle Income	West	Medical Doctors
60	Speech Therapist	125	138	153	0.09	109,898	Senegal	Minimum Estimate	2266 - Audiologists and speech therapists	Lower-middle Income	West	Other Health Workers
61	Urologist	16	18	22	0.01	746,187	Senegal	Minimum Estimate	2212 - Specialist medical practitioners	Lower-middle Income	West	Medical Doctors
1	Anaesthesiologist	2	2	2	0.20	49,215	Seychelles	Minimum Estimate	2212 - Specialist medical practitioners	High Income and Upper Middle Income	Southern	Medical Doctors
2	Associate Nurse/Enrolled Nurse/Nursing Assistant	109	113	116	11.78	849	Seychelles	Minimum Estimate	3221 - Nursing associate professionals	High Income and Upper Middle Income	Southern	Nursing Personnel
3	Audiologist	0	0	0	0.03	384,301	Seychelles	Minimum Estimate	2266 - Audiologists and speech therapists	High Income and Upper Middle Income	Southern	Other Health Workers
4	Cardiologist	1	1	1	0.13	79,869	Seychelles	Minimum Estimate	2212 - Specialist medical practitioners	High Income and Upper Middle Income	Southern	Medical Doctors
5	Cardiothoracic Surgeon	0	0	0	0.03	386,125	Seychelles	Minimum Estimate	2212 - Specialist medical practitioners	High Income and Upper Middle Income	Southern	Medical Doctors
6	Clinical Officer/Physician Assistant	14	15	17	1.67	5,972	Seychelles	Minimum Estimate	3256 - Medical assistants	High Income and Upper Middle Income	Southern	Other Health Workers
7	Clinical Pharmacist	4	4	4	0.42	23,540	Seychelles	Minimum Estimate	2262 - Pharmacists	High Income and Upper Middle Income	Southern	Pharmacist
8	Clinical Psychologist	5	5	5	0.51	19,480	Seychelles	Minimum Estimate	2634 - Psychologists	High Income and Upper Middle Income	Southern	Other Health Workers
9	Community health worker/Village health worker	46	47	47	4.79	2,088	Seychelles	Minimum Estimate	3253 - Community health workers	High Income and Upper Middle Income	Southern	Other Health Workers
10	Dental Surgery Assistant	5	6	6	0.58	17,309	Seychelles	Minimum Estimate	3251 - Dental assistants and therapists	High Income and Upper Middle Income	Southern	Other Health Workers
11	Dental Therapist	3	3	4	0.36	27,637	Seychelles	Minimum Estimate	3251 - Dental assistants and therapists	High Income and Upper Middle Income	Southern	Other Health Workers
12	Dentist	5	5	4	0.45	22,192	Seychelles	Minimum Estimate	2261 - Dentists	High Income and Upper Middle Income	Southern	Dentist
13	Dermatologist	0	0	0	0.03	349,040	Seychelles	Minimum Estimate	2212 - Specialist medical practitioners	High Income and Upper Middle Income	Southern	Medical Doctors
14	Endocrinologist	2	3	3	0.30	32,841	Seychelles	Minimum Estimate	2212 - Specialist medical practitioners	High Income and Upper Middle Income	Southern	Medical Doctors
15	ENT Surgeon	1	2	2	0.19	52,896	Seychelles	Minimum Estimate	2212 - Specialist medical practitioners	High Income and Upper Middle Income	Southern	Medical Doctors
16	Environmental Health Officer	2	2	3	0.26	38,901	Seychelles	Minimum Estimate	2263 - Environmental and occupational health and hygiene professionals	High Income and Upper Middle Income	Southern	Other Health Workers
17	Gastroenterologist	1	1	1	0.06	172,517	Seychelles	Minimum Estimate	2212 - Specialist medical practitioners	High Income and Upper Middle Income	Southern	Medical Doctors
18	General Medical Practitioner (Generalist Doctor)	38	40	42	4.26	2,345	Seychelles	Minimum Estimate	2211 - Generalist medical practitioners	High Income and Upper Middle Income	Southern	Medical Doctors
19	General Surgeon	2	2	3	0.26	38,217	Seychelles	Minimum Estimate	2212 - Specialist medical practitioners	High Income and Upper Middle Income	Southern	Medical Doctors
20	Haematologist	0	0	0	0.04	258,669	Seychelles	Minimum Estimate	2212 - Specialist medical practitioners	High Income and Upper Middle Income	Southern	Medical Doctors
21	Health Promoter/Health Educator	1	1	1	0.08	133,284	Seychelles	Minimum Estimate	2269 - Health professionals not elsewhere classified	High Income and Upper Middle Income	Southern	Other Health Workers
22	Infectious Diseases Specialist	0	0	0	0.01	1,492,967	Seychelles	Minimum Estimate	2212 - Specialist medical practitioners	High Income and Upper Middle Income	Southern	Medical Doctors
23	Intensive Care Nurse	3	3	3	0.35	28,642	Seychelles	Minimum Estimate	2221 - Nursing professionals	High Income and Upper Middle Income	Southern	Nursing Personnel

S/N	Health Professionals	2022	2026	2030	Density per 10,000 population	Required Population ratio (1 professional is to xxx population)	Name of Country	Modelling Scenario	ISCO-08 Match	Income Group Classification	Sub-Regional Grouping	SDG 3c Occupation
24	Medical Laboratory Scientist	17	19	21	2.11	4,747	Seychelles	Minimum Estimate	3212 - Medical and pathology laboratory technicians	High Income and Upper Middle Income	Southern	Other Health Workers
25	Medical Laboratory Technician	13	13	14	1.43	7,009	Seychelles	Minimum Estimate	3212 - Medical and pathology laboratory technicians	High Income and Upper Middle Income	Southern	Other Health Workers
26	Medical Social Worker	2	2	2	0.25	39,659	Seychelles	Minimum Estimate	1344 - Social welfare managers	High Income and Upper Middle Income	Southern	Other Health Workers
27	Mental Health Nurse	5	5	5	0.52	19,332	Seychelles	Minimum Estimate	2221 - Nursing professionals	High Income and Upper Middle Income	Southern	Nursing Personnel
28	Midwife	50	51	50	5.12	1,955	Seychelles	Minimum Estimate	2222 - Midwifery professionals	High Income and Upper Middle Income	Southern	Midwifery Personnel
29	Nephrologist	3	3	4	0.36	27,664	Seychelles	Minimum Estimate	2212 - Specialist medical practitioners	High Income and Upper Middle Income	Southern	Medical Doctors
30	Neuro-Surgeon	1	1	1	0.10	96,765	Seychelles	Minimum Estimate	2212 - Specialist medical practitioners	High Income and Upper Middle Income	Southern	Medical Doctors
31	Nurse Anaesthetist	9	10	11	1.13	8,813	Seychelles	Minimum Estimate	2221 - Nursing professionals	High Income and Upper Middle Income	Southern	Nursing Personnel
32	Nutritionist	9	10	10	1.01	9,880	Seychelles	Minimum Estimate	2265 - Dieticians and nutritionists	High Income and Upper Middle Income	Southern	Other Health Workers
33	Obstetrician & Gynaecologist	5	5	6	0.56	17,921	Seychelles	Minimum Estimate	2212 - Specialist medical practitioners	High Income and Upper Middle Income	Southern	Medical Doctors
34	Occupational Therapist	3	3	4	0.38	26,499	Seychelles	Minimum Estimate	2269 - Health professionals not elsewhere classified	High Income and Upper Middle Income	Southern	Other Health Workers
35	Oncology Nurse	3	3	4	0.38	26,237	Seychelles	Minimum Estimate	2221 - Nursing professionals	High Income and Upper Middle Income	Southern	Nursing Personnel
36	Operating Theatre Nurse	21	23	24	2.48	4,034	Seychelles	Minimum Estimate	2221 - Nursing professionals	High Income and Upper Middle Income	Southern	Nursing Personnel
37	Ophthalmic Nurse	3	4	4	0.43	23,132	Seychelles	Minimum Estimate	2221 - Nursing professionals	High Income and Upper Middle Income	Southern	Nursing Personnel
38	Ophthalmologist	1	1	2	0.16	63,607	Seychelles	Minimum Estimate	2212 - Specialist medical practitioners	High Income and Upper Middle Income	Southern	Medical Doctors
39	Optometrist	3	3	3	0.34	29,834	Seychelles	Minimum Estimate	2267 - Optometrists and ophthalmic opticians	High Income and Upper Middle Income	Southern	Other Health Workers
40	Orthopaedic Nurse	1	1	2	0.16	63,658	Seychelles	Minimum Estimate	2221 - Nursing professionals	High Income and Upper Middle Income	Southern	Nursing Personnel
41	Orthopaedic Surgeon	7	8	9	0.87	11,556	Seychelles	Minimum Estimate	2212 - Specialist medical practitioners	High Income and Upper Middle Income	Southern	Medical Doctors
42	Orthopaedic Technologist	3	3	3	0.32	31,370	Seychelles	Minimum Estimate	3214 - Medical and dental prosthetic technicians	High Income and Upper Middle Income	Southern	Other Health Workers
43	Paediatric Nurse	6	6	6	0.65	15,503	Seychelles	Minimum Estimate	2221 - Nursing professionals	High Income and Upper Middle Income	Southern	Nursing Personnel
44	Paediatric Surgeon	0	0	0	0.01	889,479	Seychelles	Minimum Estimate	2212 - Specialist medical practitioners	High Income and Upper Middle Income	Southern	Medical Doctors
45	Paediatrician	5	5	5	0.51	19,470	Seychelles	Minimum Estimate	2212 - Specialist medical practitioners	High Income and Upper Middle Income	Southern	Medical Doctors
46	Pathologist	1	1	1	0.07	143,054	Seychelles	Minimum Estimate	2212 - Specialist medical practitioners	High Income and Upper Middle Income	Southern	Medical Doctors
47	Pharmacist	12	13	14	1.37	7,321	Seychelles	Minimum Estimate	2262 - Pharmacists	High Income and Upper Middle Income	Southern	Pharmacist
48	Pharmacy Technician	11	12	13	1.30	7,677	Seychelles	Minimum Estimate	3213 - Pharmaceutical technicians and assistants	High Income and Upper Middle Income	Southern	Other Health Workers
49	Physician	15	16	18	1.77	5,648	Seychelles	Minimum Estimate	2212 - Specialist medical practitioners	High Income and Upper Middle Income	Southern	Medical Doctors
50	Physiotherapist	3	3	3	0.34	29,453	Seychelles	Minimum Estimate	2264 - Physiotherapists	High Income and Upper Middle Income	Southern	Other Health Workers
51	Plastic Surgeon	1	1	1	0.13	75,968	Seychelles	Minimum Estimate	2212 - Specialist medical practitioners	High Income and Upper Middle Income	Southern	Medical Doctors
52	Psychiatrist	5	5	5	0.48	20,849	Seychelles	Minimum Estimate	2212 - Specialist medical practitioners	High Income and Upper Middle Income	Southern	Medical Doctors
53	Radiation Oncologist	2	2	2	0.22	45,030	Seychelles	Minimum Estimate	2212 - Specialist medical practitioners	High Income and Upper Middle Income	Southern	Medical Doctors
54	Radiographer (Diagnostics and Therapy)	13	14	15	1.49	6,722	Seychelles	Minimum Estimate	3211 - Medical imaging and therapeutic equipment technicians	High Income and Upper Middle Income	Southern	Other Health Workers
55	Radiologist	4	4	4	0.45	22,340	Seychelles	Minimum Estimate	2212 - Specialist medical practitioners	High Income and Upper Middle Income	Southern	Medical Doctors
56	Registered General Nurse / State Certified Nurse	348	374	393	39.70	252	Seychelles	Minimum Estimate	2221 - Nursing professionals	High Income and Upper Middle Income	Southern	Nursing Personnel
57	Renal Nurse	41	44	46	4.61	2,169	Seychelles	Minimum Estimate	2221 - Nursing professionals	High Income and Upper Middle Income	Southern	Nursing Personnel
58	Respiratory Physician	1	1	1	0.07	146,767	Seychelles	Minimum Estimate	2212 - Specialist medical practitioners	High Income and Upper Middle Income	Southern	Medical Doctors
59	Rheumatologist	0	0	0	0.04	266,699	Seychelles	Minimum Estimate	2212 - Specialist medical practitioners	High Income and Upper Middle Income	Southern	Medical Doctors
60	Speech Therapist	1	1	1	0.15	66,716	Seychelles	Minimum Estimate	2266 - Audiologists and speech therapists	High Income and Upper Middle Income	Southern	Other Health Workers
61	Urologist	1	1	1	0.10	98,470	Seychelles	Minimum Estimate	2212 - Specialist medical practitioners	High Income and Upper Middle Income	Southern	Medical Doctors
1	Anaesthesiologist	65	72	80	0.10	101,078	Sierra Leone	Minimum Estimate	2212 - Specialist medical practitioners	Low Income	West	Medical Doctors
2	Associate Nurse/Enrolled Nurse/Nursing Assistant	7,400	8,046	8,756	10.89	918	Sierra Leone	Minimum Estimate	3221 - Nursing associate professionals	Low Income	West	Nursing Personnel
3	Audiologist	20	21	23	0.03	346,657	Sierra Leone	Minimum Estimate	2266 - Audiologists and speech therapists	Low Income	West	Other Health Workers
4	Cardiologist	26	29	32	0.04	253,341	Sierra Leone	Minimum Estimate	2212 - Specialist medical practitioners	Low Income	West	Medical Doctors
5	Cardiothoracic Surgeon	9	10	11	0.01	734,832	Sierra Leone	Minimum Estimate	2212 - Specialist medical practitioners	Low Income	West	Medical Doctors
6	Clinical Officer/Physician Assistant	880	903	935	1.17	8,540	Sierra Leone	Minimum Estimate	3256 - Medical assistants	Low Income	West	Other Health Workers
7	Clinical Pharmacist	165	180	199	0.25	40,305	Sierra Leone	Minimum Estimate	2262 - Pharmacists	Low Income	West	Pharmacist
8	Clinical Psychologist	386	424	469	0.58	17,107	Sierra Leone	Minimum Estimate	2634 - Psychologists	Low Income	West	Other Health Workers
9	Community health worker/Village health worker	6,316	6,569	6,806	8.48	1,179	Sierra Leone	Minimum Estimate	3253 - Community health workers	Low Income	West	Other Health Workers
10	Dental Surgery Assistant	409	450	501	0.62	16,062	Sierra Leone	Minimum Estimate	3251 - Dental assistants and therapists	Low Income	West	Other Health Workers
11	Dental Therapist	249	274	305	0.38	26,368	Sierra Leone	Minimum Estimate	3251 - Dental assistants and therapists	Low Income	West	Other Health Workers
12	Dentist	427	469	527	0.65	15,276	Sierra Leone	Minimum Estimate	2261 - Dentists	Low Income	West	Dentist
13	Dermatologist	22	24	26	0.03	305,137	Sierra Leone	Minimum Estimate	2212 - Specialist medical practitioners	Low Income	West	Medical Doctors
14	Endocrinologist	14	17	21	0.03	386,123	Sierra Leone	Minimum Estimate	2212 - Specialist medical practitioners	Low Income	West	Medical Doctors
15	ENT Surgeon	57	63	70	0.09	115,524	Sierra Leone	Minimum Estimate	2212 - Specialist medical practitioners	Low Income	West	Medical Doctors
16	Environmental Health Officer	198	215	233	0.29	34,625	Sierra Leone	Minimum Estimate	2263 - Environmental and occupational health and hygiene professionals	Low Income	West	Other Health Workers
17	Gastroenterologist	60	58	58	0.07	135,911	Sierra Leone	Minimum Estimate	2212 - Specialist medical practitioners	Low Income	West	Medical Doctors
18	General Medical Practitioner (Generalist Doctor)	2,324	2,507	2,707	3.37	2,968	Sierra Leone	Minimum Estimate	2211 - Generalist medical practitioners	Low Income	West	Medical Doctors
19	General Surgeon	58	66	77	0.10	103,590	Sierra Leone	Minimum Estimate	2212 - Specialist medical practitioners	Low Income	West	Medical Doctors
20	Haematologist	27	30	32	0.04	251,231	Sierra Leone	Minimum Estimate	2212 - Specialist medical practitioners	Low Income	West	Medical Doctors
21	Health Promoter/Health Educator	43	44	45	0.06	175,676	Sierra Leone	Minimum Estimate	2269 - Health professionals not elsewhere classified	Low Income	West	Other Health Workers
22	Infectious Diseases Specialist	5	5	6	0.01	1,405,878	Sierra Leone	Minimum Estimate	2212 - Specialist medical practitioners	Low Income	West	Medical Doctors
23	Intensive Care Nurse	166	181	196	0.24	41,152	Sierra Leone	Minimum Estimate	2221 - Nursing professionals	Low Income	West	Nursing Personnel
24	Medical Laboratory Scientist	750	801	867	1.08	9,235	Sierra Leone	Minimum Estimate	3212 - Medical and pathology laboratory technicians	Low Income	West	Other Health Workers
25	Medical Laboratory Technician	1,333	1,379	1,428	1.78	5,609	Sierra Leone	Minimum Estimate	3212 - Medical and pathology laboratory technicians	Low Income	West	Other Health Workers
26	Medical Social Worker	230	234	239	0.30	33,438	Sierra Leone	Minimum Estimate	1344 - Social welfare managers	Low Income	West	Other Health Workers
27	Mental Health Nurse	291	321	364	0.45	22,010	Sierra Leone	Minimum Estimate	2221 - Nursing professionals	Low Income	West	Nursing Personnel
28	Midwife	4,631	5,004	5,426	6.74	1,484	Sierra Leone	Minimum Estimate	2222 - Midwifery professionals	Low Income	West	Midwifery Personnel
29	Nephrologist	58	66	77	0.10	103,227	Sierra Leone	Minimum Estimate	2212 - Specialist medical practitioners	Low Income	West	Medical Doctors
30	Neuro-Surgeon	23	26	29	0.04	277,322	Sierra Leone	Minimum Estimate	2212 - Specialist medical practitioners	Low Income	West	Medical Doctors
31	Nurse Anaesthetist	108	123	139	0.17	57,668	Sierra Leone	Minimum Estimate	2221 - Nursing professionals	Low Income	West	Nursing Personnel
32	Nutritionist	854	888	917	1.14	8,755	Sierra Leone	Minimum Estimate	2265 - Dieticians and nutritionists	Low Income	West	Other Health Workers
33	Obstetrician & Gynaecologist	415	455	499	0.62	16,118	Sierra Leone	Minimum Estimate	2212 - Specialist medical practitioners	Low Income	West	Medical Doctors
34	Occupational Therapist	149	167	188	0.23	42,666	Sierra Leone	Minimum Estimate	2269 - Health professionals not elsewhere classified	Low Income	West	Other Health Workers
35	Oncology Nurse	42	48	56	0.07	142,778	Sierra Leone	Minimum Estimate	2221 - Nursing professionals	Low Income	West	Nursing Personnel
36	Operating Theatre Nurse	679	751	852	1.06	9,393	Sierra Leone	Minimum Estimate	2221 - Nursing professionals	Low Income	West	Nursing Personnel
37	Ophthalmic Nurse	63	70	77	0.10	103,761	Sierra Leone	Minimum Estimate	2221 - Nursing professionals	Low Income	West	Nursing Personnel
38	Ophthalmologist	16	18	20	0.03	398,167	Sierra Leone	Minimum Estimate	2212 - Specialist medical practitioners	Low Income	West	Medical Doctors
39	Optometrist	65	72	79	0.10	101,185	Sierra Leone	Minimum Estimate	2267 - Optometrists and ophthalmic opticians	Low Income	West	Other Health Workers
40	Orthopaedic Nurse	79	90	102	0.13	79,019	Sierra Leone	Minimum Estimate	2221 - Nursing professionals	Low Income	West	Nursing Personnel
41	Orthopaedic Surgeon	174	201	232	0.29	34,565	Sierra Leone	Minimum Estimate	2212 - Specialist medical practitioners	Low Income	West	Medical Doctors
42	Orthopaedic Technologist	211	233	256	0.32	31,488	Sierra Leone	Minimum Estimate	3214 - Medical and dental prosthetic technicians	Low Income	West	Other Health Workers
43	Paediatric Nurse	720	774	823	1.02	9,789	Sierra Leone	Minimum Estimate	2221 - Nursing professionals	Low Income	West	Nursing Personnel
44	Paediatric Surgeon	19	20	21	0.03	385,421	Sierra Leone	Minimum Estimate	2212 - Specialist medical practitioners	Low Income	West	Medical Doctors
45	Paediatrician	262	288	317	0.39	25,376	Sierra Leone	Minimum Estimate	2212 - Specialist medical practitioners	Low Income	West	Medical Doctors
46	Pathologist	37	41	47	0.06	170,335	Sierra Leone	Minimum Estimate	2212 - Specialist medical practitioners	Low Income	West	Medical Doctors

S/N	Health Professionals	2022	2026	2030	Density per 10,000 population	Required Population ratio (1 professional is to xxx population)	Name of Country	Modelling Scenario	ISCO-08 Match	Income Group Classification	Sub-Regional Grouping	SDG 3c Occupation
47	Pharmacist	383	388	395	0.50	20,187	Sierra Leone	Minimum Estimate	2262 - Pharmacists	Low Income	West	Pharmacist
48	Pharmacy Technician	624	678	739	0.92	10,872	Sierra Leone	Minimum Estimate	3213 - Pharmaceutical technicians and assistants	Low Income	West	Other Health Workers
49	Physician	629	688	756	0.94	10,625	Sierra Leone	Minimum Estimate	2212 - Specialist medical practitioners	Low Income	West	Medical Doctors
50	Physiotherapist	153	169	186	0.23	43,279	Sierra Leone	Minimum Estimate	2264 - Physiotherapists	Low Income	West	Other Health Workers
51	Plastic Surgeon	39	43	48	0.06	167,608	Sierra Leone	Minimum Estimate	2212 - Specialist medical practitioners	Low Income	West	Medical Doctors
52	Psychiatrist	299	325	362	0.45	22,113	Sierra Leone	Minimum Estimate	2212 - Specialist medical practitioners	Low Income	West	Medical Doctors
53	Radiation Oncologist	9	11	13	0.02	627,351	Sierra Leone	Minimum Estimate	2212 - Specialist medical practitioners	Low Income	West	Medical Doctors
54	Radiographer (Diagnostics and Therapy)	436	484	545	0.68	14,687	Sierra Leone	Minimum Estimate	3211 - Medical imaging and therapeutic equipment technicians	Low Income	West	Other Health Workers
55	Radiologist	129	142	159	0.20	50,384	Sierra Leone	Minimum Estimate	2212 - Specialist medical practitioners	Low Income	West	Medical Doctors
56	Registered General Nurse / State Certified Nurse	13,928	15,025	16,400	20.49	488	Sierra Leone	Minimum Estimate	2221 - Nursing professionals	Low Income	West	Nursing Personnel
57	Renal Nurse	721	816	956	1.20	8,349	Sierra Leone	Minimum Estimate	2221 - Nursing professionals	Low Income	West	Nursing Personnel
58	Respiratory Physician	38	42	47	0.06	172,043	Sierra Leone	Minimum Estimate	2212 - Specialist medical practitioners	Low Income	West	Medical Doctors
59	Rheumatologist	17	18	20	0.03	399,006	Sierra Leone	Minimum Estimate	2212 - Specialist medical practitioners	Low Income	West	Medical Doctors
60	Speech Therapist	60	65	70	0.09	114,312	Sierra Leone	Minimum Estimate	2266 - Audiologists and speech therapists	Low Income	West	Other Health Workers
61	Urologist	7	8	10	0.01	836,449	Sierra Leone	Minimum Estimate	2212 - Specialist medical practitioners	Low Income	West	Medical Doctors
1	Anaesthesiologist	604	642	686	0.12	86,910	South Africa	Minimum Estimate	2212 - Specialist medical practitioners	High Income and Upper Middle Income	Southern	Medical Doctors
2	Associate Nurse/Enrolled Nurse/Nursing Assistant	75,646	80,791	86,623	14.54	688	South Africa	Minimum Estimate	3221 - Nursing associate professionals	High Income and Upper Middle Income	Southern	Nursing Personnel
3	Audiologist	200	215	230	0.04	259,827	South Africa	Minimum Estimate	2266 - Audiologists and speech therapists	High Income and Upper Middle Income	Southern	Other Health Workers
4	Cardiologist	381	409	440	0.07	135,754	South Africa	Minimum Estimate	2212 - Specialist medical practitioners	High Income and Upper Middle Income	Southern	Medical Doctors
5	Cardiothoracic Surgeon	80	81	83	0.01	716,527	South Africa	Minimum Estimate	2212 - Specialist medical practitioners	High Income and Upper Middle Income	Southern	Medical Doctors
6	Clinical Officer/Physician Assistant	11,995	12,805	13,746	2.31	4,329	South Africa	Minimum Estimate	3256 - Medical assistants	High Income and Upper Middle Income	Southern	Other Health Workers
7	Clinical Pharmacist	1,489	1,609	1,754	0.30	33,877	South Africa	Minimum Estimate	2262 - Pharmacists	High Income and Upper Middle Income	Southern	Pharmacist
8	Clinical Psychologist	2,591	2,717	2,857	0.48	20,857	South Africa	Minimum Estimate	2634 - Psychologists	High Income and Upper Middle Income	Southern	Other Health Workers
9	Community health worker/Village health worker	34,494	36,130	37,880	6.36	1,572	South Africa	Minimum Estimate	3253 - Community health workers	High Income and Upper Middle Income	Southern	Other Health Workers
10	Dental Surgery Assistant	4,864	5,157	5,461	0.91	10,941	South Africa	Minimum Estimate	3251 - Dental assistants and therapists	High Income and Upper Middle Income	Southern	Other Health Workers
11	Dental Therapist	3,060	3,247	3,440	0.58	17,368	South Africa	Minimum Estimate	3251 - Dental assistants and therapists	High Income and Upper Middle Income	Southern	Other Health Workers
12	Dentist	3,355	3,518	3,685	0.62	16,200	South Africa	Minimum Estimate	2261 - Dentists	High Income and Upper Middle Income	Southern	Dentist
13	Dermatologist	189	199	210	0.04	284,302	South Africa	Minimum Estimate	2212 - Specialist medical practitioners	High Income and Upper Middle Income	Southern	Medical Doctors
14	Endocrinologist	498	560	632	0.11	93,997	South Africa	Minimum Estimate	2212 - Specialist medical practitioners	High Income and Upper Middle Income	Southern	Medical Doctors
15	ENT Surgeon	841	912	1,012	0.17	58,894	South Africa	Minimum Estimate	2212 - Specialist medical practitioners	High Income and Upper Middle Income	Southern	Medical Doctors
16	Environmental Health Officer	1,473	1,531	1,589	0.27	37,556	South Africa	Minimum Estimate	2263 - Environmental and occupational health and hygiene professionals	High Income and Upper Middle Income	Southern	Other Health Workers
17	Gastroenterologist	326	324	323	0.05	182,760	South Africa	Minimum Estimate	2212 - Specialist medical practitioners	High Income and Upper Middle Income	Southern	Medical Doctors
18	General Medical Practitioner (Generalist Doctor)	24,778	26,403	28,197	4.73	2,114	South Africa	Minimum Estimate	2211 - Generalist medical practitioners	High Income and Upper Middle Income	Southern	Medical Doctors
19	General Surgeon	727	782	847	0.14	70,340	South Africa	Minimum Estimate	2212 - Specialist medical practitioners	High Income and Upper Middle Income	Southern	Medical Doctors
20	Haematologist	105	108	112	0.02	532,820	South Africa	Minimum Estimate	2212 - Specialist medical practitioners	High Income and Upper Middle Income	Southern	Medical Doctors
21	Health Promoter/Health Educator	561	592	626	0.11	95,126	South Africa	Minimum Estimate	2269 - Health professionals not elsewhere classified	High Income and Upper Middle Income	Southern	Other Health Workers
22	Infectious Diseases Specialist	59	61	64	0.01	929,395	South Africa	Minimum Estimate	2212 - Specialist medical practitioners	High Income and Upper Middle Income	Southern	Medical Doctors
23	Intensive Care Nurse	1,245	1,286	1,331	0.22	44,774	South Africa	Minimum Estimate	2221 - Nursing professionals	High Income and Upper Middle Income	Southern	Nursing Personnel
24	Medical Laboratory Scientist	10,672	11,652	12,786	2.15	4,658	South Africa	Minimum Estimate	3212 - Medical and pathology laboratory technicians	High Income and Upper Middle Income	Southern	Other Health Workers
25	Medical Laboratory Technician	8,922	9,780	10,771	1.81	5,531	South Africa	Minimum Estimate	3212 - Medical and pathology laboratory technicians	High Income and Upper Middle Income	Southern	Other Health Workers
26	Medical Social Worker	1,151	1,158	1,156	0.19	51,329	South Africa	Minimum Estimate	1344 - Social welfare managers	High Income and Upper Middle Income	Southern	Other Health Workers
27	Mental Health Nurse	3,199	3,369	3,573	0.60	16,636	South Africa	Minimum Estimate	2221 - Nursing professionals	High Income and Upper Middle Income	Southern	Nursing Personnel
28	Midwife	32,059	33,310	34,467	5.78	1,731	South Africa	Minimum Estimate	2222 - Midwifery professionals	High Income and Upper Middle Income	Southern	Midwifery Personnel
29	Nephrologist	1,034	1,159	1,305	0.22	45,554	South Africa	Minimum Estimate	2212 - Specialist medical practitioners	High Income and Upper Middle Income	Southern	Medical Doctors
30	Neuro-Surgeon	263	281	308	0.05	192,952	South Africa	Minimum Estimate	2212 - Specialist medical practitioners	High Income and Upper Middle Income	Southern	Medical Doctors
31	Nurse Anaesthetist	1,982	2,183	2,407	0.40	24,812	South Africa	Minimum Estimate	2221 - Nursing professionals	High Income and Upper Middle Income	Southern	Nursing Personnel
32	Nutritionist	3,543	3,560	3,569	0.60	16,663	South Africa	Minimum Estimate	2265 - Dieticians and nutritionists	High Income and Upper Middle Income	Southern	Other Health Workers
33	Obstetrician & Gynaecologist	4,203	4,406	4,625	0.78	12,897	South Africa	Minimum Estimate	2212 - Specialist medical practitioners	High Income and Upper Middle Income	Southern	Medical Doctors
34	Occupational Therapist	1,234	1,305	1,400	0.24	42,449	South Africa	Minimum Estimate	2269 - Health professionals not elsewhere classified	High Income and Upper Middle Income	Southern	Other Health Workers
35	Oncology Nurse	823	921	1,034	0.17	57,545	South Africa	Minimum Estimate	2221 - Nursing professionals	High Income and Upper Middle Income	Southern	Nursing Personnel
36	Operating Theatre Nurse	8,818	9,492	10,325	1.74	5,760	South Africa	Minimum Estimate	2221 - Nursing professionals	High Income and Upper Middle Income	Southern	Nursing Personnel
37	Ophthalmic Nurse	970	1,060	1,161	0.19	51,325	South Africa	Minimum Estimate	2221 - Nursing professionals	High Income and Upper Middle Income	Southern	Nursing Personnel
38	Ophthalmologist	419	462	514	0.09	116,037	South Africa	Minimum Estimate	2212 - Specialist medical practitioners	High Income and Upper Middle Income	Southern	Medical Doctors
39	Optometrist	1,857	2,045	2,253	0.38	26,517	South Africa	Minimum Estimate	2267 - Optometrists and ophthalmic opticians	High Income and Upper Middle Income	Southern	Other Health Workers
40	Orthopaedic Nurse	535	551	569	0.10	104,793	South Africa	Minimum Estimate	2221 - Nursing professionals	High Income and Upper Middle Income	Southern	Nursing Personnel
41	Orthopaedic Surgeon	2,656	2,936	3,253	0.54	18,357	South Africa	Minimum Estimate	2212 - Specialist medical practitioners	High Income and Upper Middle Income	Southern	Medical Doctors
42	Orthopaedic Technologist	1,570	1,625	1,685	0.28	35,402	South Africa	Minimum Estimate	3214 - Medical and dental prosthetic technicians	High Income and Upper Middle Income	Southern	Other Health Workers
43	Paediatric Nurse	3,514	3,714	3,920	0.66	15,130	South Africa	Minimum Estimate	2221 - Nursing professionals	High Income and Upper Middle Income	Southern	Nursing Personnel
44	Paediatric Surgeon	109	112	115	0.02	518,349	South Africa	Minimum Estimate	2212 - Specialist medical practitioners	High Income and Upper Middle Income	Southern	Medical Doctors
45	Paediatrician	2,704	3,267	3,989	0.68	14,764	South Africa	Minimum Estimate	2212 - Specialist medical practitioners	High Income and Upper Middle Income	Southern	Medical Doctors
46	Pathologist	671	702	737	0.12	80,584	South Africa	Minimum Estimate	2212 - Specialist medical practitioners	High Income and Upper Middle Income	Southern	Medical Doctors
47	Pharmacist	8,216	8,589	9,008	1.51	6,611	South Africa	Minimum Estimate	2262 - Pharmacists	High Income and Upper Middle Income	Southern	Pharmacist
48	Pharmacy Technician	6,467	6,798	7,164	1.20	8,320	South Africa	Minimum Estimate	3213 - Pharmaceutical technicians and assistants	High Income and Upper Middle Income	Southern	Other Health Workers
49	Physician	5,946	6,460	7,058	1.19	8,430	South Africa	Minimum Estimate	2212 - Specialist medical practitioners	High Income and Upper Middle Income	Southern	Medical Doctors
50	Physiotherapist	1,334	1,401	1,492	0.25	39,876	South Africa	Minimum Estimate	2264 - Physiotherapists	High Income and Upper Middle Income	Southern	Other Health Workers
51	Plastic Surgeon	378	381	383	0.06	155,163	South Africa	Minimum Estimate	2212 - Specialist medical practitioners	High Income and Upper Middle Income	Southern	Medical Doctors
52	Psychiatrist	2,761	2,909	3,088	0.52	19,267	South Africa	Minimum Estimate	2212 - Specialist medical practitioners	High Income and Upper Middle Income	Southern	Medical Doctors
53	Radiation Oncologist	273	306	344	0.06	173,359	South Africa	Minimum Estimate	2212 - Specialist medical practitioners	High Income and Upper Middle Income	Southern	Medical Doctors
54	Radiographer (Diagnostics and Therapy)	5,540	6,005	6,537	1.10	9,109	South Africa	Minimum Estimate	3211 - Medical imaging and therapeutic equipment technicians	High Income and Upper Middle Income	Southern	Other Health Workers
55	Radiologist	1,642	1,766	1,906	0.32	31,281	South Africa	Minimum Estimate	2212 - Specialist medical practitioners	High Income and Upper Middle Income	Southern	Medical Doctors
56	Registered General Nurse / State Certified Nurse	156,811	170,093	185,342	31.15	321	South Africa	Minimum Estimate	2221 - Nursing professionals	High Income and Upper Middle Income	Southern	Nursing Personnel
57	Renal Nurse	12,913	14,479	16,315	2.74	3,644	South Africa	Minimum Estimate	2221 - Nursing professionals	High Income and Upper Middle Income	Southern	Nursing Personnel
58	Respiratory Physician	531	676	867	0.15	67,678	South Africa	Minimum Estimate	2212 - Specialist medical practitioners	High Income and Upper Middle Income	Southern	Medical Doctors
59	Rheumatologist	194	209	226	0.04	264,735	South Africa	Minimum Estimate	2212 - Specialist medical practitioners	High Income and Upper Middle Income	Southern	Medical Doctors
60	Speech Therapist	547	573	621	0.10	95,744	South Africa	Minimum Estimate	2266 - Audiologists and speech therapists	High Income and Upper Middle Income	Southern	Other Health Workers
61	Urologist	137	156	178	0.03	333,457	South Africa	Minimum Estimate	2212 - Specialist medical practitioners	High Income and Upper Middle Income	Southern	Medical Doctors
1	Anaesthesiologist	79	88	99	0.09	113,143	South Sudan	Minimum Estimate	2212 - Specialist medical practitioners	Low Income	East	Medical Doctors
2	Associate Nurse/Enrolled Nurse/Nursing Assistant	10,092	10,719	11,529	10.30	971	South Sudan	Minimum Estimate	3221 - Nursing associate professionals	Low Income	East	Nursing Personnel
3	Audiologist	26	28	31	0.03	359,527	South Sudan	Minimum Estimate	2266 - Audiologists and speech therapists	Low Income	East	Other Health Workers
4	Cardiologist	37	44	52	0.05	214,866	South Sudan	Minimum Estimate	2212 - Specialist medical practitioners	Low Income	East	Medical Doctors
5	Cardiothoracic Surgeon	14	16	17	0.02	645,078	South Sudan	Minimum Estimate	2212 - Specialist medical practitioners	Low Income	East	Medical Doctors
6	Clinical Officer/Physician Assistant	1,144	1,176	1,234	1.11	9,031	South Sudan	Minimum Estimate	3256 - Medical assistants	Low Income	East	Other Health Workers
7	Clinical Pharmacist	213	231	255	0.23	43,915	South Sudan	Minimum Estimate	2262 - Pharmacists	Low Income	East	Pharmacist
8	Clinical Psychologist	567	606	658	0.59	17,019	South Sudan	Minimum Estimate	2634 - Psychologists	Low Income	East	Other Health Workers

S/N	Health Professionals	2022	2026	2030	Density per 10,000 population	Required Population ratio (1 professional is to xxx population)	Name of Country	Modelling Scenario	ISCO-08 Match	Income Group Classification	Sub-Regional Grouping	SDG 3c Occupation
9	Community health worker/Village health worker	7,094	7,290	7,558	6.75	1,480	South Sudan	Minimum Estimate	3253 - Community health workers	Low Income	East	Other Health Workers
10	Dental Surgery Assistant	710	770	849	0.76	13,213	South Sudan	Minimum Estimate	3251 - Dental assistants and therapists	Low Income	East	Other Health Workers
11	Dental Therapist	443	482	530	0.47	21,150	South Sudan	Minimum Estimate	3251 - Dental assistants and therapists	Low Income	East	Other Health Workers
12	Dentist	532	576	639	0.57	17,538	South Sudan	Minimum Estimate	2261 - Dentists	Low Income	East	Dentist
13	Dermatologist	27	29	32	0.03	351,637	South Sudan	Minimum Estimate	2212 - Specialist medical practitioners	Low Income	East	Medical Doctors
14	Endocrinologist	26	30	37	0.03	295,428	South Sudan	Minimum Estimate	2212 - Specialist medical practitioners	Low Income	East	Medical Doctors
15	ENT Surgeon	85	94	106	0.09	105,852	South Sudan	Minimum Estimate	2212 - Specialist medical practitioners	Low Income	East	Medical Doctors
16	Environmental Health Officer	278	297	319	0.28	35,169	South Sudan	Minimum Estimate	2263 - Environmental and occupational health and hygiene professionals	Low Income	East	Other Health Workers
17	Gastroenterologist	71	72	75	0.07	148,487	South Sudan	Minimum Estimate	2212 - Specialist medical practitioners	Low Income	East	Medical Doctors
18	General Medical Practitioner (Generalist Doctor)	2,939	3,123	3,360	3.00	3,334	South Sudan	Minimum Estimate	2211 - Generalist medical practitioners	Low Income	East	Medical Doctors
19	General Surgeon	51	57	65	0.06	171,061	South Sudan	Minimum Estimate	2212 - Specialist medical practitioners	Low Income	East	Medical Doctors
20	Haematologist	31	33	36	0.03	309,192	South Sudan	Minimum Estimate	2212 - Specialist medical practitioners	Low Income	East	Medical Doctors
21	Health Promoter/Health Educator	61	62	64	0.06	173,725	South Sudan	Minimum Estimate	2269 - Health professionals not elsewhere classified	Low Income	East	Other Health Workers
22	Infectious Diseases Specialist	7	8	8	0.01	1,390,588	South Sudan	Minimum Estimate	2212 - Specialist medical practitioners	Low Income	East	Medical Doctors
23	Intensive Care Nurse	294	308	326	0.29	34,372	South Sudan	Minimum Estimate	2221 - Nursing professionals	Low Income	East	Nursing Personnel
24	Medical Laboratory Scientist	845	915	1,012	0.91	11,045	South Sudan	Minimum Estimate	3212 - Medical and pathology laboratory technicians	Low Income	East	Other Health Workers
25	Medical Laboratory Technician	755	804	870	0.78	12,850	South Sudan	Minimum Estimate	3212 - Medical and pathology laboratory technicians	Low Income	East	Other Health Workers
26	Medical Social Worker	378	379	384	0.34	29,112	South Sudan	Minimum Estimate	1344 - Social welfare managers	Low Income	East	Other Health Workers
27	Mental Health Nurse	490	528	587	0.53	19,003	South Sudan	Minimum Estimate	2221 - Nursing professionals	Low Income	East	Nursing Personnel
28	Midwife	5,822	6,206	6,710	5.98	1,672	South Sudan	Minimum Estimate	2222 - Midwifery professionals	Low Income	East	Midwifery Personnel
29	Nephrologist	64	72	84	0.07	133,410	South Sudan	Minimum Estimate	2212 - Specialist medical practitioners	Low Income	East	Medical Doctors
30	Neuro-Surgeon	26	30	34	0.03	327,701	South Sudan	Minimum Estimate	2212 - Specialist medical practitioners	Low Income	East	Medical Doctors
31	Nurse Anaesthetist	145	164	188	0.17	59,737	South Sudan	Minimum Estimate	2221 - Nursing professionals	Low Income	East	Nursing Personnel
32	Nutritionist	1,657	1,649	1,652	1.48	6,758	South Sudan	Minimum Estimate	2265 - Dieticians and nutritionists	Low Income	East	Other Health Workers
33	Obstetrician & Gynaecologist	582	627	682	0.61	16,446	South Sudan	Minimum Estimate	2212 - Specialist medical practitioners	Low Income	East	Medical Doctors
34	Occupational Therapist	165	186	212	0.19	52,852	South Sudan	Minimum Estimate	2269 - Health professionals not elsewhere classified	Low Income	East	Other Health Workers
35	Oncology Nurse	50	57	66	0.06	170,025	South Sudan	Minimum Estimate	2221 - Nursing professionals	Low Income	East	Nursing Personnel
36	Operating Theatre Nurse	895	999	1,148	1.03	9,719	South Sudan	Minimum Estimate	2221 - Nursing professionals	Low Income	East	Nursing Personnel
37	Ophthalmic Nurse	85	93	104	0.09	107,358	South Sudan	Minimum Estimate	2221 - Nursing professionals	Low Income	East	Nursing Personnel
38	Ophthalmologist	28	32	38	0.03	291,828	South Sudan	Minimum Estimate	2212 - Specialist medical practitioners	Low Income	East	Medical Doctors
39	Optometrist	114	134	159	0.14	70,455	South Sudan	Minimum Estimate	2267 - Optometrists and ophthalmic opticians	Low Income	East	Other Health Workers
40	Orthopaedic Nurse	83	93	106	0.09	106,156	South Sudan	Minimum Estimate	2221 - Nursing professionals	Low Income	East	Nursing Personnel
41	Orthopaedic Surgeon	172	191	216	0.19	51,763	South Sudan	Minimum Estimate	2212 - Specialist medical practitioners	Low Income	East	Medical Doctors
42	Orthopaedic Technologist	290	314	342	0.31	32,770	South Sudan	Minimum Estimate	3214 - Medical and dental prosthetic technicians	Low Income	East	Other Health Workers
43	Paediatric Nurse	1,082	1,132	1,189	1.06	9,433	South Sudan	Minimum Estimate	2221 - Nursing professionals	Low Income	East	Nursing Personnel
44	Paediatric Surgeon	29	31	32	0.03	348,068	South Sudan	Minimum Estimate	2212 - Specialist medical practitioners	Low Income	East	Medical Doctors
45	Paediatrician	389	424	467	0.42	23,984	South Sudan	Minimum Estimate	2212 - Specialist medical practitioners	Low Income	East	Medical Doctors
46	Pathologist	60	66	74	0.07	150,274	South Sudan	Minimum Estimate	2212 - Specialist medical practitioners	Low Income	East	Medical Doctors
47	Pharmacist	783	812	852	0.76	13,120	South Sudan	Minimum Estimate	2262 - Pharmacists	Low Income	East	Pharmacist
48	Pharmacy Technician	806	863	936	0.84	11,975	South Sudan	Minimum Estimate	3213 - Pharmaceutical technicians and assistants	Low Income	East	Other Health Workers
49	Physician	703	760	837	0.75	13,368	South Sudan	Minimum Estimate	2212 - Specialist medical practitioners	Low Income	East	Medical Doctors
50	Physiotherapist	211	229	252	0.23	44,408	South Sudan	Minimum Estimate	2264 - Physiotherapists	Low Income	East	Other Health Workers
51	Plastic Surgeon	122	135	151	0.13	74,502	South Sudan	Minimum Estimate	2212 - Specialist medical practitioners	Low Income	East	Medical Doctors
52	Psychiatrist	474	501	545	0.49	20,500	South Sudan	Minimum Estimate	2212 - Specialist medical practitioners	Low Income	East	Medical Doctors
53	Radiation Oncologist	14	16	19	0.02	598,789	South Sudan	Minimum Estimate	2212 - Specialist medical practitioners	Low Income	East	Medical Doctors
54	Radiographer (Diagnostics and Therapy)	485	534	603	0.54	18,528	South Sudan	Minimum Estimate	3211 - Medical imaging and therapeutic equipment technicians	Low Income	East	Other Health Workers
55	Radiologist	149	163	182	0.16	61,584	South Sudan	Minimum Estimate	2212 - Specialist medical practitioners	Low Income	East	Medical Doctors
56	Registered General Nurse / State Certified Nurse	25,053	25,819	27,179	24.41	410	South Sudan	Minimum Estimate	2221 - Nursing professionals	Low Income	East	Nursing Personnel
57	Renal Nurse	804	901	1,045	0.94	10,663	South Sudan	Minimum Estimate	2221 - Nursing professionals	Low Income	East	Nursing Personnel
58	Respiratory Physician	76	83	92	0.08	122,011	South Sudan	Minimum Estimate	2212 - Specialist medical practitioners	Low Income	East	Medical Doctors
59	Rheumatologist	26	28	32	0.03	356,006	South Sudan	Minimum Estimate	2212 - Specialist medical practitioners	Low Income	East	Medical Doctors
60	Speech Therapist	87	93	102	0.09	110,225	South Sudan	Minimum Estimate	2266 - Audiologists and speech therapists	Low Income	East	Other Health Workers
61	Urologist	8	9	11	0.01	1,039,718	South Sudan	Minimum Estimate	2212 - Specialist medical practitioners	Low Income	East	Medical Doctors
1	Anaesthesiologist	74	84	96	0.12	86,699	Togo	Minimum Estimate	2212 - Specialist medical practitioners	Low Income	West	Medical Doctors
2	Associate Nurse/Enrolled Nurse/Nursing Assistant	7,963	8,770	9,689	11.64	859	Togo	Minimum Estimate	3221 - Nursing associate professionals	Low Income	West	Nursing Personnel
3	Audiologist	21	24	26	0.03	317,379	Togo	Minimum Estimate	2266 - Audiologists and speech therapists	Low Income	West	Other Health Workers
4	Cardiologist	29	34	39	0.05	213,807	Togo	Minimum Estimate	2212 - Specialist medical practitioners	Low Income	West	Medical Doctors
5	Cardiothoracic Surgeon	12	13	15	0.02	559,162	Togo	Minimum Estimate	2212 - Specialist medical practitioners	Low Income	West	Medical Doctors
6	Clinical Officer/Physician Assistant	992	1,066	1,161	1.40	7,138	Togo	Minimum Estimate	3256 - Medical assistants	Low Income	West	Other Health Workers
7	Clinical Pharmacist	170	190	214	0.26	38,828	Togo	Minimum Estimate	2262 - Pharmacists	Low Income	West	Pharmacist
8	Clinical Psychologist	370	413	464	0.56	17,943	Togo	Minimum Estimate	2634 - Psychologists	Low Income	West	Other Health Workers
9	Community health worker/Village health worker	6,745	7,290	7,879	9.47	1,056	Togo	Minimum Estimate	3253 - Community health workers	Low Income	West	Other Health Workers
10	Dental Surgery Assistant	650	729	821	0.98	10,169	Togo	Minimum Estimate	3251 - Dental assistants and therapists	Low Income	West	Other Health Workers
11	Dental Therapist	409	459	516	0.62	16,165	Togo	Minimum Estimate	3251 - Dental assistants and therapists	Low Income	West	Other Health Workers
12	Dentist	445	494	555	0.66	15,039	Togo	Minimum Estimate	2261 - Dentists	Low Income	West	Dentist
13	Dermatologist	23	26	29	0.03	291,950	Togo	Minimum Estimate	2212 - Specialist medical practitioners	Low Income	West	Medical Doctors
14	Endocrinologist	18	23	29	0.03	286,208	Togo	Minimum Estimate	2212 - Specialist medical practitioners	Low Income	West	Medical Doctors
15	ENT Surgeon	65	73	83	0.10	100,297	Togo	Minimum Estimate	2212 - Specialist medical practitioners	Low Income	West	Medical Doctors
16	Environmental Health Officer	206	225	245	0.29	34,043	Togo	Minimum Estimate	2263 - Environmental and occupational health and hygiene professionals	Low Income	West	Other Health Workers
17	Gastroenterologist	49	50	53	0.06	156,987	Togo	Minimum Estimate	2212 - Specialist medical practitioners	Low Income	West	Medical Doctors
18	General Medical Practitioner (Generalist Doctor)	2,496	2,739	3,011	3.62	2,765	Togo	Minimum Estimate	2211 - Generalist medical practitioners	Low Income	West	Medical Doctors
19	General Surgeon	67	78	93	0.11	89,143	Togo	Minimum Estimate	2212 - Specialist medical practitioners	Low Income	West	Medical Doctors
20	Haematologist	27	29	32	0.04	258,942	Togo	Minimum Estimate	2212 - Specialist medical practitioners	Low Income	West	Medical Doctors
21	Health Promoter/Health Educator	49	52	55	0.07	149,391	Togo	Minimum Estimate	2269 - Health professionals not elsewhere classified	Low Income	West	Other Health Workers
22	Infectious Diseases Specialist	7	8	8	0.01	983,574	Togo	Minimum Estimate	2212 - Specialist medical practitioners	Low Income	West	Medical Doctors
23	Intensive Care Nurse	180	196	214	0.26	38,916	Togo	Minimum Estimate	2221 - Nursing professionals	Low Income	West	Nursing Personnel
24	Medical Laboratory Scientist	796	881	988	1.19	8,406	Togo	Minimum Estimate	3212 - Medical and pathology laboratory technicians	Low Income	West	Other Health Workers
25	Medical Laboratory Technician	1,406	1,561	1,738	2.09	4,791	Togo	Minimum Estimate	3212 - Medical and pathology laboratory technicians	Low Income	West	Other Health Workers
26	Medical Social Worker	193	199	206	0.25	40,301	Togo	Minimum Estimate	1344 - Social welfare managers	Low Income	West	Other Health Workers
27	Mental Health Nurse	296	329	375	0.45	22,160	Togo	Minimum Estimate	2221 - Nursing professionals	Low Income	West	Nursing Personnel
28	Midwife	4,648	5,053	5,510	6.60	1,515	Togo	Minimum Estimate	2222 - Midwifery professionals	Low Income	West	Midwifery Personnel
29	Nephrologist	66	76	91	0.11	91,162	Togo	Minimum Estimate	2212 - Specialist medical practitioners	Low Income	West	Medical Doctors
30	Neuro-Surgeon	32	36	41	0.05	200,302	Togo	Minimum Estimate	2212 - Specialist medical practitioners	Low Income	West	Medical Doctors
31	Nurse Anaesthetist	134	158	186	0.22	44,704	Togo	Minimum Estimate	2221 - Nursing professionals	Low Income	West	Nursing Personnel

S/N	Health Professionals	2022	2026	2030	Density per 10,000 population	Required Population ratio (1 professional is to xxx population)	Name of Country	Modelling Scenario	ISCO-08 Match	Income Group Classification	Sub-Regional Grouping	SDG 3c Occupation
32	Nutritionist	720	739	757	0.91	10,971	Togo	Minimum Estimate	2265 - Dieticians and nutritionists	Low Income	West	Other Health Workers
33	Obstetrician & Gynaecologist	440	488	542	0.65	15,379	Togo	Minimum Estimate	2212 - Specialist medical practitioners	Low Income	West	Medical Doctors
34	Occupational Therapist	165	190	220	0.26	37,841	Togo	Minimum Estimate	2269 - Health professionals not elsewhere classified	Low Income	West	Other Health Workers
35	Oncology Nurse	49	57	69	0.08	120,747	Togo	Minimum Estimate	2221 - Nursing professionals	Low Income	West	Nursing Personnel
36	Operating Theatre Nurse	761	864	1,002	1.21	8,272	Togo	Minimum Estimate	2221 - Nursing professionals	Low Income	West	Nursing Personnel
37	Ophthalmic Nurse	74	84	97	0.12	85,226	Togo	Minimum Estimate	2221 - Nursing professionals	Low Income	West	Nursing Personnel
38	Ophthalmologist	19	23	27	0.03	304,087	Togo	Minimum Estimate	2212 - Specialist medical practitioners	Low Income	West	Medical Doctors
39	Optometrist	77	89	104	0.12	80,128	Togo	Minimum Estimate	2267 - Optometrists and ophthalmic opticians	Low Income	West	Other Health Workers
40	Orthopaedic Nurse	87	101	117	0.14	71,082	Togo	Minimum Estimate	2221 - Nursing professionals	Low Income	West	Nursing Personnel
41	Orthopaedic Surgeon	241	279	324	0.39	25,641	Togo	Minimum Estimate	2212 - Specialist medical practitioners	Low Income	West	Medical Doctors
42	Orthopaedic Technologist	230	256	285	0.34	29,236	Togo	Minimum Estimate	3214 - Medical and dental prosthetic technicians	Low Income	West	Other Health Workers
43	Paediatric Nurse	711	767	822	0.99	10,138	Togo	Minimum Estimate	2221 - Nursing professionals	Low Income	West	Nursing Personnel
44	Paediatric Surgeon	18	19	20	0.02	408,716	Togo	Minimum Estimate	2212 - Specialist medical practitioners	Low Income	West	Medical Doctors
45	Paediatrician	304	339	380	0.46	21,926	Togo	Minimum Estimate	2212 - Specialist medical practitioners	Low Income	West	Medical Doctors
46	Pathologist	44	50	58	0.07	143,716	Togo	Minimum Estimate	2212 - Specialist medical practitioners	Low Income	West	Medical Doctors
47	Pharmacist	379	390	406	0.49	20,342	Togo	Minimum Estimate	2262 - Pharmacists	Low Income	West	Pharmacist
48	Pharmacy Technician	651	718	796	0.96	10,461	Togo	Minimum Estimate	3213 - Pharmaceutical technicians and assistants	Low Income	West	Other Health Workers
49	Physician	661	738	829	1.00	10,033	Togo	Minimum Estimate	2212 - Specialist medical practitioners	Low Income	West	Medical Doctors
50	Physiotherapist	165	185	207	0.25	40,289	Togo	Minimum Estimate	2264 - Physiotherapists	Low Income	West	Other Health Workers
51	Plastic Surgeon	43	49	56	0.07	148,169	Togo	Minimum Estimate	2212 - Specialist medical practitioners	Low Income	West	Medical Doctors
52	Psychiatrist	300	329	369	0.44	22,504	Togo	Minimum Estimate	2212 - Specialist medical practitioners	Low Income	West	Medical Doctors
53	Radiation Oncologist	12	14	18	0.02	472,096	Togo	Minimum Estimate	2212 - Specialist medical practitioners	Low Income	West	Medical Doctors
54	Radiographer (Diagnostics and Therapy)	485	549	632	0.76	13,143	Togo	Minimum Estimate	3211 - Medical imaging and therapeutic equipment technicians	Low Income	West	Other Health Workers
55	Radiologist	142	161	184	0.22	45,051	Togo	Minimum Estimate	2212 - Specialist medical practitioners	Low Income	West	Medical Doctors
56	Registered General Nurse / State Certified Nurse	18,678	19,824	21,378	25.86	387	Togo	Minimum Estimate	2221 - Nursing professionals	Low Income	West	Nursing Personnel
57	Renal Nurse	818	947	1,130	1.37	7,323	Togo	Minimum Estimate	2221 - Nursing professionals	Low Income	West	Nursing Personnel
58	Respiratory Physician	51	57	65	0.08	128,824	Togo	Minimum Estimate	2212 - Specialist medical practitioners	Low Income	West	Medical Doctors
59	Rheumatologist	18	21	23	0.03	358,415	Togo	Minimum Estimate	2212 - Specialist medical practitioners	Low Income	West	Medical Doctors
60	Speech Therapist	62	68	75	0.09	110,561	Togo	Minimum Estimate	2266 - Audiologists and speech therapists	Low Income	West	Other Health Workers
61	Urologist	6	8	9	0.01	874,927	Togo	Minimum Estimate	2212 - Specialist medical practitioners	Low Income	West	Medical Doctors
1	Anaesthesiologist	262	301	345	0.07	133,533	Uganda	Minimum Estimate	2212 - Specialist medical practitioners	Low Income	East	Medical Doctors
2	Associate Nurse/Enrolled Nurse/Nursing Assistant	45,295	50,993	57,453	12.46	803	Uganda	Minimum Estimate	3221 - Nursing associate professionals	Low Income	East	Nursing Personnel
3	Audiologist	112	126	141	0.03	327,696	Uganda	Minimum Estimate	2266 - Audiologists and speech therapists	Low Income	East	Other Health Workers
4	Cardiologist	111	129	148	0.03	311,174	Uganda	Minimum Estimate	2212 - Specialist medical practitioners	Low Income	East	Medical Doctors
5	Cardiothoracic Surgeon	45	51	58	0.01	789,415	Uganda	Minimum Estimate	2212 - Specialist medical practitioners	Low Income	East	Medical Doctors
6	Clinical Officer/Physician Assistant	7,057	7,649	8,348	1.81	5,518	Uganda	Minimum Estimate	3256 - Medical assistants	Low Income	East	Other Health Workers
7	Clinical Pharmacist	801	885	996	0.22	46,061	Uganda	Minimum Estimate	2262 - Pharmacists	Low Income	East	Pharmacist
8	Clinical Psychologist	2,716	2,920	3,161	0.69	14,573	Uganda	Minimum Estimate	2634 - Psychologists	Low Income	East	Other Health Workers
9	Community health worker/Village health worker	39,186	42,832	46,550	10.08	992	Uganda	Minimum Estimate	3253 - Community health workers	Low Income	East	Other Health Workers
10	Dental Surgery Assistant	2,409	2,753	3,172	0.69	14,523	Uganda	Minimum Estimate	3251 - Dental assistants and therapists	Low Income	East	Other Health Workers
11	Dental Therapist	1,503	1,718	1,978	0.43	23,286	Uganda	Minimum Estimate	3251 - Dental assistants and therapists	Low Income	East	Other Health Workers
12	Dentist	1,845	2,095	2,449	0.53	18,754	Uganda	Minimum Estimate	2261 - Dentists	Low Income	East	Dentist
13	Dermatologist	106	120	135	0.03	342,731	Uganda	Minimum Estimate	2212 - Specialist medical practitioners	Low Income	East	Medical Doctors
14	Endocrinologist	94	112	140	0.03	325,700	Uganda	Minimum Estimate	2212 - Specialist medical practitioners	Low Income	East	Medical Doctors
15	ENT Surgeon	298	343	394	0.09	116,850	Uganda	Minimum Estimate	2212 - Specialist medical practitioners	Low Income	East	Medical Doctors
16	Environmental Health Officer	1,136	1,265	1,402	0.30	32,974	Uganda	Minimum Estimate	2263 - Environmental and occupational health and hygiene professionals	Low Income	East	Other Health Workers
17	Gastroenterologist	183	179	182	0.04	249,264	Uganda	Minimum Estimate	2212 - Specialist medical practitioners	Low Income	East	Medical Doctors
18	General Medical Practitioner (Generalist Doctor)	13,148	14,539	16,090	3.49	2,865	Uganda	Minimum Estimate	2211 - Generalist medical practitioners	Low Income	East	Medical Doctors
19	General Surgeon	223	262	316	0.07	145,009	Uganda	Minimum Estimate	2212 - Specialist medical practitioners	Low Income	East	Medical Doctors
20	Haematologist	85	93	101	0.02	454,913	Uganda	Minimum Estimate	2212 - Specialist medical practitioners	Low Income	East	Medical Doctors
21	Health Promoter/Health Educator	345	373	406	0.09	113,306	Uganda	Minimum Estimate	2269 - Health professionals not elsewhere classified	Low Income	East	Other Health Workers
22	Infectious Diseases Specialist	33	35	39	0.01	1,194,603	Uganda	Minimum Estimate	2212 - Specialist medical practitioners	Low Income	East	Medical Doctors
23	Intensive Care Nurse	975	1,061	1,151	0.25	40,127	Uganda	Minimum Estimate	2221 - Nursing professionals	Low Income	East	Nursing Personnel
24	Medical Laboratory Scientist	4,618	5,093	5,683	1.24	8,087	Uganda	Minimum Estimate	3212 - Medical and pathology laboratory technicians	Low Income	East	Other Health Workers
25	Medical Laboratory Technician	8,495	9,452	10,512	2.28	4,389	Uganda	Minimum Estimate	3212 - Medical and pathology laboratory technicians	Low Income	East	Other Health Workers
26	Medical Social Worker	854	901	958	0.21	47,322	Uganda	Minimum Estimate	1344 - Social welfare managers	Low Income	East	Other Health Workers
27	Mental Health Nurse	1,597	1,793	2,105	0.46	21,730	Uganda	Minimum Estimate	2221 - Nursing professionals	Low Income	East	Nursing Personnel
28	Midwife	23,346	26,001	29,158	6.32	1,581	Uganda	Minimum Estimate	2222 - Midwifery professionals	Low Income	East	Midwifery Personnel
29	Nephrologist	212	248	303	0.07	150,211	Uganda	Minimum Estimate	2212 - Specialist medical practitioners	Low Income	East	Medical Doctors
30	Neuro-Surgeon	96	111	129	0.03	354,709	Uganda	Minimum Estimate	2212 - Specialist medical practitioners	Low Income	East	Medical Doctors
31	Nurse Anaesthetist	685	824	990	0.22	46,438	Uganda	Minimum Estimate	2221 - Nursing professionals	Low Income	East	Nursing Personnel
32	Nutritionist	3,048	3,227	3,387	0.73	13,622	Uganda	Minimum Estimate	2265 - Dieticians and nutritionists	Low Income	East	Other Health Workers
33	Obstetrician & Gynaecologist	2,410	2,685	3,012	0.66	15,253	Uganda	Minimum Estimate	2212 - Specialist medical practitioners	Low Income	East	Medical Doctors
34	Occupational Therapist	519	600	702	0.15	65,515	Uganda	Minimum Estimate	2269 - Health professionals not elsewhere classified	Low Income	East	Other Health Workers
35	Oncology Nurse	204	242	296	0.06	154,319	Uganda	Minimum Estimate	2221 - Nursing professionals	Low Income	East	Nursing Personnel
36	Operating Theatre Nurse	2,961	3,407	4,077	0.89	11,203	Uganda	Minimum Estimate	2221 - Nursing professionals	Low Income	East	Nursing Personnel
37	Ophthalmic Nurse	253	293	344	0.07	133,488	Uganda	Minimum Estimate	2221 - Nursing professionals	Low Income	East	Nursing Personnel
38	Ophthalmologist	88	102	121	0.03	378,560	Uganda	Minimum Estimate	2212 - Specialist medical practitioners	Low Income	East	Medical Doctors
39	Optometrist	345	399	463	0.10	99,565	Uganda	Minimum Estimate	2267 - Optometrists and ophthalmic opticians	Low Income	East	Other Health Workers
40	Orthopaedic Nurse	252	294	340	0.07	135,803	Uganda	Minimum Estimate	2221 - Nursing professionals	Low Income	East	Nursing Personnel
41	Orthopaedic Surgeon	809	985	1,211	0.26	37,753	Uganda	Minimum Estimate	2212 - Specialist medical practitioners	Low Income	East	Medical Doctors
42	Orthopaedic Technologist	1,082	1,218	1,363	0.30	33,893	Uganda	Minimum Estimate	3214 - Medical and dental prosthetic technicians	Low Income	East	Other Health Workers
43	Paediatric Nurse	3,256	3,552	3,790	0.82	12,179	Uganda	Minimum Estimate	2221 - Nursing professionals	Low Income	East	Nursing Personnel
44	Paediatric Surgeon	128	141	151	0.03	305,528	Uganda	Minimum Estimate	2212 - Specialist medical practitioners	Low Income	East	Medical Doctors
45	Paediatrician	1,611	1,806	2,030	0.44	22,708	Uganda	Minimum Estimate	2212 - Specialist medical practitioners	Low Income	East	Medical Doctors
46	Pathologist	227	246	281	0.06	163,002	Uganda	Minimum Estimate	2212 - Specialist medical practitioners	Low Income	East	Medical Doctors
47	Pharmacist	3,601	3,832	4,087	0.89	11,262	Uganda	Minimum Estimate	2262 - Pharmacists	Low Income	East	Pharmacist
48	Pharmacy Technician	3,086	3,425	3,814	0.83	12,080	Uganda	Minimum Estimate	3213 - Pharmaceutical technicians and assistants	Low Income	East	Other Health Workers
49	Physician	2,656	3,001	3,421	0.74	13,462	Uganda	Minimum Estimate	2212 - Specialist medical practitioners	Low Income	East	Medical Doctors
50	Physiotherapist	771	868	978	0.21	47,161	Uganda	Minimum Estimate	2264 - Physiotherapists	Low Income	East	Other Health Workers
51	Plastic Surgeon	400	476	564	0.12	81,756	Uganda	Minimum Estimate	2212 - Specialist medical practitioners	Low Income	East	Medical Doctors
52	Psychiatrist	1,488	1,651	1,902	0.41	24,101	Uganda	Minimum Estimate	2212 - Specialist medical practitioners	Low Income	East	Medical Doctors
53	Radiation Oncologist	78	96	118	0.03	385,853	Uganda	Minimum Estimate	2212 - Specialist medical practitioners	Low Income	East	Medical Doctors
54	Radiographer (Diagnostics and Therapy)	1,755	2,025	2,410	0.53	18,933	Uganda	Minimum Estimate	3211 - Medical imaging and therapeutic equipment technicians	Low Income	East	Other Health Workers

S/N	Health Professionals	2022	2026	2030	Density per 10,000 population	Required Population ratio (1 professional is to xxx population)	Name of Country	Modelling Scenario	ISCO-08 Match	Income Group Classification	Sub-Regional Grouping	SDG 3c Occupation
55	Radiologist	552	648	786	0.17	57,196	Uganda	Minimum Estimate	2212 - Specialist medical practitioners	Low Income	East	Medical Doctors
56	Registered General Nurse / State Certified Nurse	74,383	81,350	90,077	19.66	509	Uganda	Minimum Estimate	2221 - Nursing professionals	Low Income	East	Nursing Personnel
57	Renal Nurse	2,644	3,081	3,780	0.83	12,050	Uganda	Minimum Estimate	2221 - Nursing professionals	Low Income	East	Nursing Personnel
58	Respiratory Physician	351	394	442	0.10	104,584	Uganda	Minimum Estimate	2212 - Specialist medical practitioners	Low Income	East	Medical Doctors
59	Rheumatologist	91	104	117	0.03	393,153	Uganda	Minimum Estimate	2212 - Specialist medical practitioners	Low Income	East	Medical Doctors
60	Speech Therapist	322	360	401	0.09	115,059	Uganda	Minimum Estimate	2266 - Audiologists and speech therapists	Low Income	East	Other Health Workers
61	Urologist	22	28	36	0.01	1,232,972	Uganda	Minimum Estimate	2212 - Specialist medical practitioners	Low Income	East	Medical Doctors
1	Anaesthesiologist	377	438	507	0.08	118,608	United Republic of	Minimum Estimate	2212 - Specialist medical practitioners	Lower-middle Income	East	Medical Doctors
2	Associate Nurse/Enrolled Nurse/Nursing Assistant	51,683	57,433	63,749	10.59	944	United Republic of	Minimum Estimate	3221 - Nursing associate professionals	Lower-middle Income	East	Nursing Personnel
3	Audiologist	151	170	191	0.03	315,856	United Republic of	Minimum Estimate	2266 - Audiologists and speech therapists	Lower-middle Income	East	Other Health Workers
4	Cardiologist	197	230	268	0.04	224,928	United Republic of	Minimum Estimate	2212 - Specialist medical practitioners	Lower-middle Income	East	Medical Doctors
5	Cardiothoracic Surgeon	62	71	82	0.01	733,358	United Republic of	Minimum Estimate	2212 - Specialist medical practitioners	Lower-middle Income	East	Medical Doctors
6	Clinical Officer/Physician Assistant	3,902	4,145	4,460	0.75	13,411	United Republic of	Minimum Estimate	3256 - Medical assistants	Lower-middle Income	East	Other Health Workers
7	Clinical Pharmacist	1,094	1,229	1,387	0.23	43,332	United Republic of	Minimum Estimate	2262 - Pharmacists	Lower-middle Income	East	Pharmacist
8	Clinical Psychologist	2,985	3,299	3,651	0.61	16,482	United Republic of	Minimum Estimate	2634 - Psychologists	Lower-middle Income	East	Other Health Workers
9	Community health worker/Village health worker	32,637	35,594	38,698	6.43	1,555	United Republic of	Minimum Estimate	3253 - Community health workers	Lower-middle Income	East	Other Health Workers
10	Dental Surgery Assistant	3,799	4,340	4,973	0.83	12,116	United Republic of	Minimum Estimate	3251 - Dental assistants and therapists	Lower-middle Income	East	Other Health Workers
11	Dental Therapist	2,362	2,700	3,093	0.51	19,479	United Republic of	Minimum Estimate	3251 - Dental assistants and therapists	Lower-middle Income	East	Other Health Workers
12	Dentist	3,123	3,548	4,076	0.68	14,783	United Republic of	Minimum Estimate	2261 - Dentists	Lower-middle Income	East	Dentist
13	Dermatologist	145	165	186	0.03	324,426	United Republic of	Minimum Estimate	2212 - Specialist medical practitioners	Lower-middle Income	East	Medical Doctors
14	Endocrinologist	113	133	161	0.03	371,349	United Republic of	Minimum Estimate	2212 - Specialist medical practitioners	Lower-middle Income	East	Medical Doctors
15	ENT Surgeon	437	502	576	0.10	104,355	United Republic of	Minimum Estimate	2212 - Specialist medical practitioners	Lower-middle Income	East	Medical Doctors
16	Environmental Health Officer	1,484	1,662	1,852	0.31	32,564	United Republic of	Minimum Estimate	2263 - Environmental and occupational health and hygiene professionals	Lower-middle Income	East	Other Health Workers
17	Gastroenterologist	244	252	267	0.04	223,685	United Republic of	Minimum Estimate	2212 - Specialist medical practitioners	Lower-middle Income	East	Medical Doctors
18	General Medical Practitioner (Generalist Doctor)	14,458	16,165	18,052	3.00	3,334	United Republic of	Minimum Estimate	2211 - Generalist medical practitioners	Lower-middle Income	East	Medical Doctors
19	General Surgeon	320	373	439	0.07	136,831	United Republic of	Minimum Estimate	2212 - Specialist medical practitioners	Lower-middle Income	East	Medical Doctors
20	Haematologist	209	238	268	0.04	224,781	United Republic of	Minimum Estimate	2212 - Specialist medical practitioners	Lower-middle Income	East	Medical Doctors
21	Health Promoter/Health Educator	193	203	216	0.04	277,356	United Republic of	Minimum Estimate	2269 - Health professionals not elsewhere classified	Lower-middle Income	East	Other Health Workers
22	Infectious Diseases Specialist	41	46	51	0.01	1,177,882	United Republic of	Minimum Estimate	2212 - Specialist medical practitioners	Lower-middle Income	East	Medical Doctors
23	Intensive Care Nurse	1,293	1,458	1,637	0.27	36,811	United Republic of	Minimum Estimate	2221 - Nursing professionals	Lower-middle Income	East	Nursing Personnel
24	Medical Laboratory Scientist	4,580	5,109	5,732	0.95	10,476	United Republic of	Minimum Estimate	3212 - Medical and pathology laboratory technicians	Lower-middle Income	East	Other Health Workers
25	Medical Laboratory Technician	7,277	8,099	9,011	1.50	6,675	United Republic of	Minimum Estimate	3212 - Medical and pathology laboratory technicians	Lower-middle Income	East	Other Health Workers
26	Medical Social Worker	1,143	1,217	1,293	0.22	46,501	United Republic of	Minimum Estimate	1344 - Social welfare managers	Lower-middle Income	East	Other Health Workers
27	Mental Health Nurse	2,001	2,279	2,643	0.44	22,671	United Republic of	Minimum Estimate	2221 - Nursing professionals	Lower-middle Income	East	Nursing Personnel
28	Midwife	39,652	44,657	50,226	8.33	1,201	United Republic of	Minimum Estimate	2222 - Midwifery professionals	Lower-middle Income	East	Midwifery Personnel
29	Nephrologist	360	420	500	0.08	119,488	United Republic of	Minimum Estimate	2212 - Specialist medical practitioners	Lower-middle Income	East	Medical Doctors
30	Neuro-Surgeon	131	154	184	0.03	325,445	United Republic of	Minimum Estimate	2212 - Specialist medical practitioners	Lower-middle Income	East	Medical Doctors
31	Nurse Anaesthetist	909	1,073	1,262	0.21	47,613	United Republic of	Minimum Estimate	2221 - Nursing professionals	Lower-middle Income	East	Nursing Personnel
32	Nutritionist	4,534	4,879	5,220	0.87	11,541	United Republic of	Minimum Estimate	2265 - Dieticians and nutritionists	Lower-middle Income	East	Other Health Workers
33	Obstetrician & Gynaecologist	3,154	3,549	3,986	0.66	15,110	United Republic of	Minimum Estimate	2212 - Specialist medical practitioners	Lower-middle Income	East	Medical Doctors
34	Occupational Therapist	744	864	1,007	0.17	59,593	United Republic of	Minimum Estimate	2269 - Health professionals not elsewhere classified	Lower-middle Income	East	Other Health Workers
35	Oncology Nurse	309	363	431	0.07	138,926	United Republic of	Minimum Estimate	2221 - Nursing professionals	Lower-middle Income	East	Nursing Personnel
36	Operating Theatre Nurse	4,380	5,018	5,845	0.98	10,243	United Republic of	Minimum Estimate	2221 - Nursing professionals	Lower-middle Income	East	Nursing Personnel
37	Ophthalmic Nurse	428	484	549	0.09	109,610	United Republic of	Minimum Estimate	2221 - Nursing professionals	Lower-middle Income	East	Nursing Personnel
38	Ophthalmologist	170	196	227	0.04	264,441	United Republic of	Minimum Estimate	2212 - Specialist medical practitioners	Lower-middle Income	East	Medical Doctors
39	Optometrist	981	1,125	1,286	0.21	46,818	United Republic of	Minimum Estimate	2267 - Optometrists and ophthalmic opticians	Lower-middle Income	East	Other Health Workers
40	Orthopaedic Nurse	350	412	482	0.08	124,759	United Republic of	Minimum Estimate	2221 - Nursing professionals	Lower-middle Income	East	Nursing Personnel
41	Orthopaedic Surgeon	1,322	1,543	1,802	0.30	33,336	United Republic of	Minimum Estimate	2212 - Specialist medical practitioners	Lower-middle Income	East	Medical Doctors
42	Orthopaedic Technologist	1,448	1,641	1,850	0.31	32,563	United Republic of	Minimum Estimate	3214 - Medical and dental prosthetic technicians	Lower-middle Income	East	Other Health Workers
43	Paediatric Nurse	5,388	6,001	6,621	1.10	9,110	United Republic of	Minimum Estimate	2221 - Nursing professionals	Lower-middle Income	East	Nursing Personnel
44	Paediatric Surgeon	159	176	193	0.03	313,105	United Republic of	Minimum Estimate	2212 - Specialist medical practitioners	Lower-middle Income	East	Medical Doctors
45	Paediatrician	2,567	2,858	3,178	0.53	18,946	United Republic of	Minimum Estimate	2212 - Specialist medical practitioners	Lower-middle Income	East	Medical Doctors
46	Pathologist	304	345	400	0.07	149,794	United Republic of	Minimum Estimate	2212 - Specialist medical practitioners	Lower-middle Income	East	Medical Doctors
47	Pharmacist	2,097	2,211	2,345	0.39	25,532	United Republic of	Minimum Estimate	2262 - Pharmacists	Lower-middle Income	East	Pharmacist
48	Pharmacy Technician	3,570	4,020	4,528	0.75	13,289	United Republic of	Minimum Estimate	3213 - Pharmaceutical technicians and assistants	Lower-middle Income	East	Other Health Workers
49	Physician	3,867	4,389	4,985	0.83	12,064	United Republic of	Minimum Estimate	2212 - Specialist medical practitioners	Lower-middle Income	East	Medical Doctors
50	Physiotherapist	1,055	1,196	1,356	0.23	44,371	United Republic of	Minimum Estimate	2264 - Physiotherapists	Lower-middle Income	East	Other Health Workers
51	Plastic Surgeon	613	725	852	0.14	70,562	United Republic of	Minimum Estimate	2212 - Specialist medical practitioners	Lower-middle Income	East	Medical Doctors
52	Psychiatrist	1,907	2,155	2,472	0.41	24,272	United Republic of	Minimum Estimate	2212 - Specialist medical practitioners	Lower-middle Income	East	Medical Doctors
53	Radiation Oncologist	101	121	145	0.02	412,450	United Republic of	Minimum Estimate	2212 - Specialist medical practitioners	Lower-middle Income	East	Medical Doctors
54	Radiographer (Diagnostics and Therapy)	2,612	3,010	3,501	0.58	17,137	United Republic of	Minimum Estimate	3211 - Medical imaging and therapeutic equipment technicians	Lower-middle Income	East	Other Health Workers
55	Radiologist	795	913	1,053	0.18	57,069	United Republic of	Minimum Estimate	2212 - Specialist medical practitioners	Lower-middle Income	East	Medical Doctors
56	Registered General Nurse / State Certified Nurse	100,915	111,516	124,223	20.73	482	United Republic of	Minimum Estimate	2221 - Nursing professionals	Lower-middle Income	East	Nursing Personnel
57	Renal Nurse	4,470	5,218	6,215	1.04	9,613	United Republic of	Minimum Estimate	2221 - Nursing professionals	Lower-middle Income	East	Nursing Personnel
58	Respiratory Physician	528	581	638	0.11	94,512	United Republic of	Minimum Estimate	2212 - Specialist medical practitioners	Lower-middle Income	East	Medical Doctors
59	Rheumatologist	131	150	172	0.03	351,072	United Republic of	Minimum Estimate	2212 - Specialist medical practitioners	Lower-middle Income	East	Medical Doctors
60	Speech Therapist	451	509	575	0.10	104,568	United Republic of	Minimum Estimate	2266 - Audiologists and speech therapists	Lower-middle Income	East	Other Health Workers
61	Urologist	36	42	50	0.01	1,184,522	United Republic of	Minimum Estimate	2212 - Specialist medical practitioners	Lower-middle Income	East	Medical Doctors
1	Anaesthesiologist	93	107	123	0.07	150,929	Zambia	Minimum Estimate	2212 - Specialist medical practitioners	Lower-middle Income	Southern	Medical Doctors
2	Associate Nurse/Enrolled Nurse/Nursing Assistant	18,181	20,309	22,719	12.26	816	Zambia	Minimum Estimate	3221 - Nursing associate professionals	Lower-middle Income	Southern	Nursing Personnel
3	Audiologist	43	49	55	0.03	338,185	Zambia	Minimum Estimate	2266 - Audiologists and speech therapists	Lower-middle Income	Southern	Other Health Workers
4	Cardiologist	47	54	63	0.03	296,108	Zambia	Minimum Estimate	2212 - Specialist medical practitioners	Lower-middle Income	Southern	Medical Doctors
5	Cardiothoracic Surgeon	16	18	20	0.01	924,560	Zambia	Minimum Estimate	2212 - Specialist medical practitioners	Lower-middle Income	Southern	Medical Doctors
6	Clinical Officer/Physician Assistant	1,813	1,909	2,050	1.11	8,977	Zambia	Minimum Estimate	3256 - Medical assistants	Lower-middle Income	Southern	Other Health Workers
7	Clinical Pharmacist	356	404	466	0.25	39,674	Zambia	Minimum Estimate	2262 - Pharmacists	Lower-middle Income	Southern	Pharmacist
8	Clinical Psychologist	1,087	1,239	1,420	0.77	13,047	Zambia	Minimum Estimate	2634 - Psychologists	Lower-middle Income	Southern	Other Health Workers
9	Community health worker/Village health worker	12,081	13,260	14,537	7.84	1,275	Zambia	Minimum Estimate	3253 - Community health workers	Lower-middle Income	Southern	Other Health Workers
10	Dental Surgery Assistant	1,135	1,300	1,497	0.81	12,395	Zambia	Minimum Estimate	3251 - Dental assistants and therapists	Lower-middle Income	Southern	Other Health Workers
11	Dental Therapist	709	813	936	0.50	19,812	Zambia	Minimum Estimate	3251 - Dental assistants and therapists	Lower-middle Income	Southern	Other Health Workers
12	Dentist	856	967	1,110	0.60	16,721	Zambia	Minimum Estimate	2261 - Dentists	Lower-middle Income	Southern	Dentist
13	Dermatologist	44	50	56	0.03	329,097	Zambia	Minimum Estimate	2212 - Specialist medical practitioners	Lower-middle Income	Southern	Medical Doctors
14	Endocrinologist	39	46	58	0.03	318,349	Zambia	Minimum Estimate	2212 - Specialist medical practitioners	Lower-middle Income	Southern	Medical Doctors
15	ENT Surgeon	127	146	168	0.09	110,032	Zambia	Minimum Estimate	2212 - Specialist medical practitioners	Lower-middle Income	Southern	Medical Doctors
16	Environmental Health Officer	457	508	562	0.30	33,033	Zambia	Minimum Estimate	2263 - Environmental and occupational health and hygiene professionals	Lower-middle Income	Southern	Other Health Workers

S/N	Health Professionals	2022	2026	2030	Density per 10,000 population	Required Population ratio (1 professional is to xxx population)	Name of Country	Modelling Scenario	ISCO-08 Match	Income Group Classification	Sub-Regional Grouping	SDG 3c Occupation
17	Gastroenterologist	111	115	124	0.07	148,236	Zambia	Minimum Estimate	2212 - Specialist medical practitioners	Lower-middle Income	Southern	Medical Doctors
18	General Medical Practitioner (Generalist Doctor)	5,153	5,718	6,367	3.44	2,909	Zambia	Minimum Estimate	2211 - Generalist medical practitioners	Lower-middle Income	Southern	Medical Doctors
19	General Surgeon	97	114	135	0.07	136,800	Zambia	Minimum Estimate	2212 - Specialist medical practitioners	Lower-middle Income	Southern	Medical Doctors
20	Haematologist	80	89	100	0.05	186,061	Zambia	Minimum Estimate	2212 - Specialist medical practitioners	Lower-middle Income	Southern	Medical Doctors
21	Health Promoter/Health Educator	90	95	101	0.06	181,379	Zambia	Minimum Estimate	2269 - Health professionals not elsewhere classified	Lower-middle Income	Southern	Other Health Workers
22	Infectious Diseases Specialist	15	16	18	0.01	1,053,126	Zambia	Minimum Estimate	2212 - Specialist medical practitioners	Lower-middle Income	Southern	Medical Doctors
23	Intensive Care Nurse	421	468	518	0.28	35,796	Zambia	Minimum Estimate	2221 - Nursing professionals	Lower-middle Income	Southern	Nursing Personnel
24	Medical Laboratory Scientist	1,833	2,044	2,304	1.25	8,023	Zambia	Minimum Estimate	3212 - Medical and pathology laboratory technicians	Lower-middle Income	Southern	Other Health Workers
25	Medical Laboratory Technician	1,902	2,133	2,406	1.30	7,684	Zambia	Minimum Estimate	3212 - Medical and pathology laboratory technicians	Lower-middle Income	Southern	Other Health Workers
26	Medical Social Worker	428	459	499	0.27	36,925	Zambia	Minimum Estimate	1344 - Social welfare managers	Lower-middle Income	Southern	Other Health Workers
27	Mental Health Nurse	625	715	848	0.46	21,752	Zambia	Minimum Estimate	2221 - Nursing professionals	Lower-middle Income	Southern	Nursing Personnel
28	Midwife	9,656	10,688	11,900	6.41	1,560	Zambia	Minimum Estimate	2222 - Midwifery professionals	Lower-middle Income	Southern	Midwifery Personnel
29	Nephrologist	109	129	158	0.09	116,343	Zambia	Minimum Estimate	2212 - Specialist medical practitioners	Lower-middle Income	Southern	Medical Doctors
30	Neuro-Surgeon	31	36	42	0.02	435,540	Zambia	Minimum Estimate	2212 - Specialist medical practitioners	Lower-middle Income	Southern	Medical Doctors
31	Nurse Anaesthetist	270	322	383	0.21	48,318	Zambia	Minimum Estimate	2221 - Nursing professionals	Lower-middle Income	Southern	Nursing Personnel
32	Nutritionist	1,612	1,731	1,850	1.00	10,029	Zambia	Minimum Estimate	2265 - Dieticians and nutritionists	Lower-middle Income	Southern	Other Health Workers
33	Obstetrician & Gynaecologist	999	1,121	1,265	0.68	14,626	Zambia	Minimum Estimate	2212 - Specialist medical practitioners	Lower-middle Income	Southern	Medical Doctors
34	Occupational Therapist	176	204	240	0.13	76,810	Zambia	Minimum Estimate	2269 - Health professionals not elsewhere classified	Lower-middle Income	Southern	Other Health Workers
35	Oncology Nurse	95	113	138	0.07	133,750	Zambia	Minimum Estimate	2221 - Nursing professionals	Lower-middle Income	Southern	Nursing Personnel
36	Operating Theatre Nurse	1,281	1,495	1,802	0.98	10,211	Zambia	Minimum Estimate	2221 - Nursing professionals	Lower-middle Income	Southern	Nursing Personnel
37	Ophthalmic Nurse	116	133	153	0.08	121,053	Zambia	Minimum Estimate	2221 - Nursing professionals	Lower-middle Income	Southern	Nursing Personnel
38	Ophthalmologist	37	43	51	0.03	360,859	Zambia	Minimum Estimate	2212 - Specialist medical practitioners	Lower-middle Income	Southern	Medical Doctors
39	Optometrist	141	163	189	0.10	98,143	Zambia	Minimum Estimate	2267 - Optometrists and ophthalmic opticians	Lower-middle Income	Southern	Other Health Workers
40	Orthopaedic Nurse	74	87	100	0.05	184,447	Zambia	Minimum Estimate	2221 - Nursing professionals	Lower-middle Income	Southern	Nursing Personnel
41	Orthopaedic Surgeon	417	498	600	0.33	30,768	Zambia	Minimum Estimate	2212 - Specialist medical practitioners	Lower-middle Income	Southern	Medical Doctors
42	Orthopaedic Technologist	418	469	523	0.28	35,459	Zambia	Minimum Estimate	3214 - Medical and dental prosthetic technicians	Lower-middle Income	Southern	Other Health Workers
43	Paediatric Nurse	1,733	1,914	2,103	1.13	8,832	Zambia	Minimum Estimate	2221 - Nursing professionals	Lower-middle Income	Southern	Nursing Personnel
44	Paediatric Surgeon	44	47	50	0.03	370,146	Zambia	Minimum Estimate	2212 - Specialist medical practitioners	Lower-middle Income	Southern	Medical Doctors
45	Paediatrician	564	654	762	0.41	24,267	Zambia	Minimum Estimate	2212 - Specialist medical practitioners	Lower-middle Income	Southern	Medical Doctors
46	Pathologist	105	115	131	0.07	140,660	Zambia	Minimum Estimate	2212 - Specialist medical practitioners	Lower-middle Income	Southern	Medical Doctors
47	Pharmacist	899	906	927	0.51	19,744	Zambia	Minimum Estimate	2262 - Pharmacists	Lower-middle Income	Southern	Pharmacist
48	Pharmacy Technician	1,197	1,323	1,471	0.80	12,569	Zambia	Minimum Estimate	3213 - Pharmaceutical technicians and assistants	Lower-middle Income	Southern	Other Health Workers
49	Physician	1,108	1,261	1,448	0.78	12,776	Zambia	Minimum Estimate	2212 - Specialist medical practitioners	Lower-middle Income	Southern	Medical Doctors
50	Physiotherapist	305	343	387	0.21	47,910	Zambia	Minimum Estimate	2264 - Physiotherapists	Lower-middle Income	Southern	Other Health Workers
51	Plastic Surgeon	158	182	208	0.11	89,049	Zambia	Minimum Estimate	2212 - Specialist medical practitioners	Lower-middle Income	Southern	Medical Doctors
52	Psychiatrist	595	673	781	0.42	23,643	Zambia	Minimum Estimate	2212 - Specialist medical practitioners	Lower-middle Income	Southern	Medical Doctors
53	Radiation Oncologist	33	40	49	0.03	374,998	Zambia	Minimum Estimate	2212 - Specialist medical practitioners	Lower-middle Income	Southern	Medical Doctors
54	Radiographer (Diagnostics and Therapy)	829	961	1,139	0.62	16,175	Zambia	Minimum Estimate	3211 - Medical imaging and therapeutic equipment technicians	Lower-middle Income	Southern	Other Health Workers
55	Radiologist	255	297	354	0.19	51,706	Zambia	Minimum Estimate	2212 - Specialist medical practitioners	Lower-middle Income	Southern	Medical Doctors
56	Registered General Nurse / State Certified Nurse	32,115	35,373	39,601	21.47	466	Zambia	Minimum Estimate	2221 - Nursing professionals	Lower-middle Income	Southern	Nursing Personnel
57	Renal Nurse	1,351	1,593	1,957	1.06	9,395	Zambia	Minimum Estimate	2221 - Nursing professionals	Lower-middle Income	Southern	Nursing Personnel
58	Respiratory Physician	83	100	120	0.06	154,203	Zambia	Minimum Estimate	2212 - Specialist medical practitioners	Lower-middle Income	Southern	Medical Doctors
59	Rheumatologist	39	45	51	0.03	365,044	Zambia	Minimum Estimate	2212 - Specialist medical practitioners	Lower-middle Income	Southern	Medical Doctors
60	Speech Therapist	132	147	164	0.09	113,042	Zambia	Minimum Estimate	2266 - Audiologists and speech therapists	Lower-middle Income	Southern	Other Health Workers
61	Urologist	10	12	15	0.01	1,180,588	Zambia	Minimum Estimate	2212 - Specialist medical practitioners	Lower-middle Income	Southern	Medical Doctors
1	Anaesthesiologist	102	117	135	0.09	110,235	Zimbabwe	Minimum Estimate	2212 - Specialist medical practitioners	Lower-middle Income	Southern	Medical Doctors
2	Associate Nurse/Enrolled Nurse/Nursing Assistant	14,803	16,183	17,748	11.89	841	Zimbabwe	Minimum Estimate	3221 - Nursing associate professionals	Lower-middle Income	Southern	Nursing Personnel
3	Audiologist	42	47	51	0.03	292,741	Zimbabwe	Minimum Estimate	2266 - Audiologists and speech therapists	Lower-middle Income	Southern	Other Health Workers
4	Cardiologist	59	66	74	0.05	201,529	Zimbabwe	Minimum Estimate	2212 - Specialist medical practitioners	Lower-middle Income	Southern	Medical Doctors
5	Cardiothoracic Surgeon	12	14	16	0.01	932,473	Zimbabwe	Minimum Estimate	2212 - Specialist medical practitioners	Lower-middle Income	Southern	Medical Doctors
6	Clinical Officer/Physician Assistant	1,104	1,167	1,266	0.86	11,676	Zimbabwe	Minimum Estimate	3256 - Medical assistants	Lower-middle Income	Southern	Other Health Workers
7	Clinical Pharmacist	309	338	378	0.26	39,199	Zimbabwe	Minimum Estimate	2262 - Pharmacists	Lower-middle Income	Southern	Pharmacist
8	Clinical Psychologist	893	935	988	0.66	15,071	Zimbabwe	Minimum Estimate	2634 - Psychologists	Lower-middle Income	Southern	Other Health Workers
9	Community health worker/Village health worker	6,636	7,035	7,422	4.98	2,009	Zimbabwe	Minimum Estimate	3253 - Community health workers	Lower-middle Income	Southern	Other Health Workers
10	Dental Surgery Assistant	975	1,087	1,224	0.82	12,180	Zimbabwe	Minimum Estimate	3251 - Dental assistants and therapists	Lower-middle Income	Southern	Other Health Workers
11	Dental Therapist	610	680	766	0.51	19,465	Zimbabwe	Minimum Estimate	3251 - Dental assistants and therapists	Lower-middle Income	Southern	Other Health Workers
12	Dentist	733	805	910	0.61	16,303	Zimbabwe	Minimum Estimate	2261 - Dentists	Lower-middle Income	Southern	Dentist
13	Dermatologist	41	46	50	0.03	298,480	Zimbabwe	Minimum Estimate	2212 - Specialist medical practitioners	Lower-middle Income	Southern	Medical Doctors
14	Endocrinologist	62	73	89	0.06	164,394	Zimbabwe	Minimum Estimate	2212 - Specialist medical practitioners	Lower-middle Income	Southern	Medical Doctors
15	ENT Surgeon	126	141	159	0.11	93,908	Zimbabwe	Minimum Estimate	2212 - Specialist medical practitioners	Lower-middle Income	Southern	Medical Doctors
16	Environmental Health Officer	369	401	433	0.29	34,578	Zimbabwe	Minimum Estimate	2263 - Environmental and occupational health and hygiene professionals	Lower-middle Income	Southern	Other Health Workers
17	Gastroenterologist	115	114	117	0.08	124,966	Zimbabwe	Minimum Estimate	2212 - Specialist medical practitioners	Lower-middle Income	Southern	Medical Doctors
18	General Medical Practitioner (Generalist Doctor)	4,079	4,443	4,857	3.26	3,070	Zimbabwe	Minimum Estimate	2211 - Generalist medical practitioners	Lower-middle Income	Southern	Medical Doctors
19	General Surgeon	164	186	218	0.15	67,961	Zimbabwe	Minimum Estimate	2212 - Specialist medical practitioners	Lower-middle Income	Southern	Medical Doctors
20	Haematologist	33	36	40	0.03	376,607	Zimbabwe	Minimum Estimate	2212 - Specialist medical practitioners	Lower-middle Income	Southern	Medical Doctors
21	Health Promoter/Health Educator	53	56	60	0.04	248,171	Zimbabwe	Minimum Estimate	2269 - Health professionals not elsewhere classified	Lower-middle Income	Southern	Other Health Workers
22	Infectious Diseases Specialist	15	17	18	0.01	821,931	Zimbabwe	Minimum Estimate	2212 - Specialist medical practitioners	Lower-middle Income	Southern	Medical Doctors
23	Intensive Care Nurse	318	353	391	0.26	38,268	Zimbabwe	Minimum Estimate	2221 - Nursing professionals	Lower-middle Income	Southern	Nursing Personnel
24	Medical Laboratory Scientist	1,549	1,686	1,871	1.26	7,928	Zimbabwe	Minimum Estimate	3212 - Medical and pathology laboratory technicians	Lower-middle Income	Southern	Other Health Workers
25	Medical Laboratory Technician	1,337	1,452	1,593	1.07	9,339	Zimbabwe	Minimum Estimate	3212 - Medical and pathology laboratory technicians	Lower-middle Income	Southern	Other Health Workers
26	Medical Social Worker	368	381	397	0.27	37,077	Zimbabwe	Minimum Estimate	1344 - Social welfare managers	Lower-middle Income	Southern	Other Health Workers
27	Mental Health Nurse	447	491	559	0.38	26,410	Zimbabwe	Minimum Estimate	2221 - Nursing professionals	Lower-middle Income	Southern	Nursing Personnel
28	Midwife	8,169	8,863	9,685	6.50	1,539	Zimbabwe	Minimum Estimate	2222 - Midwifery professionals	Lower-middle Income	Southern	Midwifery Personnel
29	Nephrologist	134	157	192	0.13	76,214	Zimbabwe	Minimum Estimate	2212 - Specialist medical practitioners	Lower-middle Income	Southern	Medical Doctors
30	Neuro-Surgeon	40	46	52	0.04	284,926	Zimbabwe	Minimum Estimate	2212 - Specialist medical practitioners	Lower-middle Income	Southern	Medical Doctors
31	Nurse Anaesthetist	309	364	430	0.29	34,730	Zimbabwe	Minimum Estimate	2221 - Nursing professionals	Lower-middle Income	Southern	Nursing Personnel
32	Nutritionist	1,116	1,173	1,226	0.82	12,188	Zimbabwe	Minimum Estimate	2265 - Dieticians and nutritionists	Lower-middle Income	Southern	Other Health Workers
33	Obstetrician & Gynaecologist	939	1,031	1,138	0.76	13,106	Zimbabwe	Minimum Estimate	2212 - Specialist medical practitioners	Lower-middle Income	Southern	Medical Doctors
34	Occupational Therapist	174	202	238	0.16	62,097	Zimbabwe	Minimum Estimate	2269 - Health professionals not elsewhere classified	Lower-middle Income	Southern	Other Health Workers
35	Oncology Nurse	111	131	158	0.11	93,088	Zimbabwe	Minimum Estimate	2221 - Nursing professionals	Lower-middle Income	Southern	Nursing Personnel
36	Operating Theatre Nurse	1,498	1,705	1,995	1.35	7,409	Zimbabwe	Minimum Estimate	2221 - Nursing professionals	Lower-middle Income	Southern	Nursing Personnel
37	Ophthalmic Nurse	184	207	235	0.16	63,237	Zimbabwe	Minimum Estimate	2221 - Nursing professionals	Lower-middle Income	Southern	Nursing Personnel
38	Ophthalmologist	57	65	76	0.05	195,674	Zimbabwe	Minimum Estimate	2212 - Specialist medical practitioners	Lower-middle Income	Southern	Medical Doctors
39	Optometrist	271	309	353	0.24	42,332	Zimbabwe	Minimum Estimate	2267 - Optometrists and ophthalmic opticians	Lower-middle Income	Southern	Other Health Workers

S/N	Health Professionals	2022	2026	2030	Density per 10,000 population	Required Population ratio (1 professional is to xxx population)	Name of Country	Modelling Scenario	ISCO-08 Match	Income Group Classification	Sub-Regional Grouping	SDG 3c Occupation
40	Orthopaedic Nurse	65	77	92	0.06	161,966	Zimbabwe	Minimum Estimate	2221 - Nursing professionals	Lower-middle Income	Southern	Nursing Personnel
41	Orthopaedic Surgeon	423	488	566	0.38	26,315	Zimbabwe	Minimum Estimate	2212 - Specialist medical practitioners	Lower-middle Income	Southern	Medical Doctors
42	Orthopaedic Technologist	334	369	407	0.27	36,757	Zimbabwe	Minimum Estimate	3214 - Medical and dental prosthetic technicians	Lower-middle Income	Southern	Other Health Workers
43	Paediatric Nurse	1,026	1,103	1,165	0.78	12,790	Zimbabwe	Minimum Estimate	2221 - Nursing professionals	Lower-middle Income	Southern	Nursing Personnel
44	Paediatric Surgeon	36	39	42	0.03	359,386	Zimbabwe	Minimum Estimate	2212 - Specialist medical practitioners	Lower-middle Income	Southern	Medical Doctors
45	Paediatrician	378	430	494	0.33	30,027	Zimbabwe	Minimum Estimate	2212 - Specialist medical practitioners	Lower-middle Income	Southern	Medical Doctors
46	Pathologist	108	118	133	0.09	110,919	Zimbabwe	Minimum Estimate	2212 - Specialist medical practitioners	Lower-middle Income	Southern	Medical Doctors
47	Pharmacist	518	539	573	0.39	25,714	Zimbabwe	Minimum Estimate	2262 - Pharmacists	Lower-middle Income	Southern	Pharmacist
48	Pharmacy Technician	1,014	1,113	1,229	0.82	12,130	Zimbabwe	Minimum Estimate	3213 - Pharmaceutical technicians and assistants	Lower-middle Income	Southern	Other Health Workers
49	Physician	1,114	1,241	1,400	0.94	10,630	Zimbabwe	Minimum Estimate	2212 - Specialist medical practitioners	Lower-middle Income	Southern	Medical Doctors
50	Physiotherapist	259	286	318	0.21	46,893	Zimbabwe	Minimum Estimate	2264 - Physiotherapists	Lower-middle Income	Southern	Other Health Workers
51	Plastic Surgeon	87	97	109	0.07	137,700	Zimbabwe	Minimum Estimate	2212 - Specialist medical practitioners	Lower-middle Income	Southern	Medical Doctors
52	Psychiatrist	461	505	570	0.38	25,988	Zimbabwe	Minimum Estimate	2212 - Specialist medical practitioners	Lower-middle Income	Southern	Medical Doctors
53	Radiation Oncologist	37	44	53	0.04	280,775	Zimbabwe	Minimum Estimate	2212 - Specialist medical practitioners	Lower-middle Income	Southern	Medical Doctors
54	Radiographer (Diagnostics and Therapy)	956	1,082	1,254	0.85	11,795	Zimbabwe	Minimum Estimate	3211 - Medical imaging and therapeutic equipment technicians	Lower-middle Income	Southern	Other Health Workers
55	Radiologist	303	344	398	0.27	37,141	Zimbabwe	Minimum Estimate	2212 - Specialist medical practitioners	Lower-middle Income	Southern	Medical Doctors
56	Registered General Nurse / State Certified Nurse	25,530	28,421	32,048	21.62	463	Zimbabwe	Minimum Estimate	2221 - Nursing professionals	Lower-middle Income	Southern	Nursing Personnel
57	Renal Nurse	1,653	1,942	2,373	1.62	6,180	Zimbabwe	Minimum Estimate	2221 - Nursing professionals	Lower-middle Income	Southern	Nursing Personnel
58	Respiratory Physician	57	66	76	0.05	195,941	Zimbabwe	Minimum Estimate	2212 - Specialist medical practitioners	Lower-middle Income	Southern	Medical Doctors
59	Rheumatologist	35	39	44	0.03	343,354	Zimbabwe	Minimum Estimate	2212 - Specialist medical practitioners	Lower-middle Income	Southern	Medical Doctors
60	Speech Therapist	110	121	132	0.09	113,379	Zimbabwe	Minimum Estimate	2266 - Audiologists and speech therapists	Lower-middle Income	Southern	Other Health Workers
61	Urologist	14	16	19	0.01	765,253	Zimbabwe	Minimum Estimate	2212 - Specialist medical practitioners	Lower-middle Income	Southern	Medical Doctors
1	Anaesthesiologist	910	997	1,087	0.25	40,416	Algeria	Maximum Estimate	2212 - Specialist medical practitioners	High Income and Upper Middle Income	West	Medical Doctors
2	Associate Nurse/Enrolled Nurse/Nursing Assistant	79,737	86,676	92,560	21.01	476	Algeria	Maximum Estimate	3221 - Nursing associate professionals	High Income and Upper Middle Income	West	Nursing Personnel
3	Audiologist	283	308	331	0.07	133,925	Algeria	Maximum Estimate	2266 - Audiologists and speech therapists	High Income and Upper Middle Income	West	Other Health Workers
4	Cardiologist	1,148	1,328	1,528	0.35	28,908	Algeria	Maximum Estimate	2212 - Specialist medical practitioners	High Income and Upper Middle Income	West	Medical Doctors
5	Cardiothoracic Surgeon	117	125	131	0.03	336,456	Algeria	Maximum Estimate	2212 - Specialist medical practitioners	High Income and Upper Middle Income	West	Medical Doctors
6	Clinical Officer/Physician Assistant	6,758	7,619	8,407	1.92	5,205	Algeria	Maximum Estimate	3256 - Medical assistants	High Income and Upper Middle Income	West	Other Health Workers
7	Clinical Pharmacist	2,859	3,193	3,489	0.80	12,569	Algeria	Maximum Estimate	2262 - Pharmacists	High Income and Upper Middle Income	West	Pharmacist
8	Clinical Psychologist	4,381	4,612	4,764	1.08	9,238	Algeria	Maximum Estimate	2634 - Psychologists	High Income and Upper Middle Income	West	Other Health Workers
9	Community health worker/Village health worker	33,665	34,976	35,389	8.03	1,245	Algeria	Maximum Estimate	3253 - Community health workers	High Income and Upper Middle Income	West	Other Health Workers
10	Dental Surgery Assistant	7,516	8,210	8,741	1.98	5,048	Algeria	Maximum Estimate	3251 - Dental assistants and therapists	High Income and Upper Middle Income	West	Other Health Workers
11	Dental Therapist	5,045	5,507	5,855	1.33	7,535	Algeria	Maximum Estimate	3251 - Dental assistants and therapists	High Income and Upper Middle Income	West	Other Health Workers
12	Dentist	7,186	7,600	7,742	1.76	5,673	Algeria	Maximum Estimate	2261 - Dentists	High Income and Upper Middle Income	West	Dentist
13	Dermatologist	249	265	280	0.06	158,000	Algeria	Maximum Estimate	2212 - Specialist medical practitioners	High Income and Upper Middle Income	West	Medical Doctors
14	Endocrinologist	1,126	1,395	1,662	0.38	26,187	Algeria	Maximum Estimate	2212 - Specialist medical practitioners	High Income and Upper Middle Income	West	Medical Doctors
15	ENT Surgeon	1,026	1,140	1,292	0.29	33,985	Algeria	Maximum Estimate	2212 - Specialist medical practitioners	High Income and Upper Middle Income	West	Medical Doctors
16	Environmental Health Officer	3,267	3,463	3,630	0.82	12,186	Algeria	Maximum Estimate	2263 - Environmental and occupational health and hygiene professionals	High Income and Upper Middle Income	West	Other Health Workers
17	Gastroenterologist	525	546	549	0.13	79,460	Algeria	Maximum Estimate	2212 - Specialist medical practitioners	High Income and Upper Middle Income	West	Medical Doctors
18	General Medical Practitioner (Generalist Doctor)	26,237	28,509	30,457	6.91	1,447	Algeria	Maximum Estimate	2211 - Generalist medical practitioners	High Income and Upper Middle Income	West	Medical Doctors
19	General Surgeon	1,113	1,315	1,520	0.35	28,873	Algeria	Maximum Estimate	2212 - Specialist medical practitioners	High Income and Upper Middle Income	West	Medical Doctors
20	Haematologist	142	154	168	0.04	262,091	Algeria	Maximum Estimate	2212 - Specialist medical practitioners	High Income and Upper Middle Income	West	Medical Doctors
21	Health Promoter/Health Educator	670	724	771	0.18	56,858	Algeria	Maximum Estimate	2269 - Health professionals not elsewhere classified	High Income and Upper Middle Income	West	Other Health Workers
22	Infectious Diseases Specialist	77	80	83	0.02	531,000	Algeria	Maximum Estimate	2212 - Specialist medical practitioners	High Income and Upper Middle Income	West	Medical Doctors
23	Intensive Care Nurse	2,687	2,877	3,062	0.69	14,420	Algeria	Maximum Estimate	2221 - Nursing professionals	High Income and Upper Middle Income	West	Nursing Personnel
24	Medical Laboratory Scientist	10,412	11,953	13,376	3.06	3,273	Algeria	Maximum Estimate	3212 - Medical and pathology laboratory technicians	High Income and Upper Middle Income	West	Other Health Workers
25	Medical Laboratory Technician	8,030	8,876	9,641	2.20	4,552	Algeria	Maximum Estimate	3212 - Medical and pathology laboratory technicians	High Income and Upper Middle Income	West	Other Health Workers
26	Medical Social Worker	1,981	2,070	2,089	0.48	21,048	Algeria	Maximum Estimate	1344 - Social welfare managers	High Income and Upper Middle Income	West	Other Health Workers
27	Mental Health Nurse	7,354	7,791	7,977	1.82	5,483	Algeria	Maximum Estimate	2221 - Nursing professionals	High Income and Upper Middle Income	West	Nursing Personnel
28	Midwife	44,554	46,721	47,382	10.77	929	Algeria	Maximum Estimate	2222 - Midwifery professionals	High Income and Upper Middle Income	West	Midwifery Personnel
29	Nephrologist	2,005	2,332	2,608	0.60	16,784	Algeria	Maximum Estimate	2212 - Specialist medical practitioners	High Income and Upper Middle Income	West	Medical Doctors
30	Neuro-Surgeon	450	503	610	0.14	70,711	Algeria	Maximum Estimate	2212 - Specialist medical practitioners	High Income and Upper Middle Income	West	Medical Doctors
31	Nurse Anaesthetist	3,253	3,886	4,584	1.04	9,604	Algeria	Maximum Estimate	2221 - Nursing professionals	High Income and Upper Middle Income	West	Nursing Personnel
32	Nutritionist	8,009	8,374	8,608	1.95	5,130	Algeria	Maximum Estimate	2265 - Dieticians and nutritionists	High Income and Upper Middle Income	West	Other Health Workers
33	Obstetrician & Gynaecologist	4,037	4,325	4,570	1.04	9,655	Algeria	Maximum Estimate	2212 - Specialist medical practitioners	High Income and Upper Middle Income	West	Medical Doctors
34	Occupational Therapist	2,006	2,180	2,338	0.53	18,784	Algeria	Maximum Estimate	2269 - Health professionals not elsewhere classified	High Income and Upper Middle Income	West	Other Health Workers
35	Oncology Nurse	1,638	1,939	2,231	0.51	19,661	Algeria	Maximum Estimate	2221 - Nursing professionals	High Income and Upper Middle Income	West	Nursing Personnel
36	Operating Theatre Nurse	13,645	15,088	16,516	3.79	2,642	Algeria	Maximum Estimate	2221 - Nursing professionals	High Income and Upper Middle Income	West	Nursing Personnel
37	Ophthalmic Nurse	2,225	2,539	2,844	0.65	15,431	Algeria	Maximum Estimate	2221 - Nursing professionals	High Income and Upper Middle Income	West	Nursing Personnel
38	Ophthalmologist	642	764	898	0.21	48,541	Algeria	Maximum Estimate	2212 - Specialist medical practitioners	High Income and Upper Middle Income	West	Medical Doctors
39	Optometrist	1,609	1,923	2,239	0.51	19,566	Algeria	Maximum Estimate	2267 - Optometrists and ophthalmic opticians	High Income and Upper Middle Income	West	Other Health Workers
40	Orthopaedic Nurse	2,169	2,327	2,489	0.56	17,733	Algeria	Maximum Estimate	2221 - Nursing professionals	High Income and Upper Middle Income	West	Nursing Personnel
41	Orthopaedic Surgeon	5,124	5,808	6,499	1.47	6,789	Algeria	Maximum Estimate	2212 - Specialist medical practitioners	High Income and Upper Middle Income	West	Medical Doctors
42	Orthopaedic Technologist	2,189	2,332	2,469	0.56	17,892	Algeria	Maximum Estimate	3214 - Medical and dental prosthetic technicians	High Income and Upper Middle Income	West	Other Health Workers
43	Paediatric Nurse	5,807	6,128	6,139	1.39	7,184	Algeria	Maximum Estimate	2221 - Nursing professionals	High Income and Upper Middle Income	West	Nursing Personnel
44	Paediatric Surgeon	261	277	282	0.06	156,760	Algeria	Maximum Estimate	2212 - Specialist medical practitioners	High Income and Upper Middle Income	West	Medical Doctors
45	Paediatrician	3,742	4,212	4,620	1.05	9,526	Algeria	Maximum Estimate	2212 - Specialist medical practitioners	High Income and Upper Middle Income	West	Medical Doctors
46	Pathologist	664	699	717	0.16	60,910	Algeria	Maximum Estimate	2212 - Specialist medical practitioners	High Income and Upper Middle Income	West	Medical Doctors
47	Pharmacist	4,787	5,294	5,772	1.32	7,586	Algeria	Maximum Estimate	2262 - Pharmacists	High Income and Upper Middle Income	West	Pharmacist
48	Pharmacy Technician	8,385	9,059	9,649	2.19	4,566	Algeria	Maximum Estimate	3213 - Pharmaceutical technicians and assistants	High Income and Upper Middle Income	West	Other Health Workers
49	Physician	10,715	12,003	13,171	3.00	3,334	Algeria	Maximum Estimate	2212 - Specialist medical practitioners	High Income and Upper Middle Income	West	Medical Doctors
50	Physiotherapist	2,454	2,668	2,944	0.67	14,879	Algeria	Maximum Estimate	2264 - Physiotherapists	High Income and Upper Middle Income	West	Other Health Workers
51	Plastic Surgeon	419	446	471	0.11	93,978	Algeria	Maximum Estimate	2212 - Specialist medical practitioners	High Income and Upper Middle Income	West	Medical Doctors
52	Psychiatrist	5,435	5,736	5,886	1.34	7,440	Algeria	Maximum Estimate	2212 - Specialist medical practitioners	High Income and Upper Middle Income	West	Medical Doctors
53	Radiation Oncologist	538	657	789	0.18	55,502	Algeria	Maximum Estimate	2212 - Specialist medical practitioners	High Income and Upper Middle Income	West	Medical Doctors
54	Radiographer (Diagnostics and Therapy)	9,886	11,141	12,219	2.79	3,589	Algeria	Maximum Estimate	3211 - Medical imaging and therapeutic equipment technicians	High Income and Upper Middle Income	West	Other Health Workers
55	Radiologist	3,117	3,527	3,929	0.89	11,180	Algeria	Maximum Estimate	2212 - Specialist medical practitioners	High Income and Upper Middle Income	West	Medical Doctors
56	Registered General Nurse / State Certified Nurse	230,989	260,058	284,270	64.70	155	Algeria	Maximum Estimate	2221 - Nursing professionals	High Income and Upper Middle Income	West	Nursing Personnel
57	Renal Nurse	40,115	46,711	52,252	11.94	838	Algeria	Maximum Estimate	2221 - Nursing professionals	High Income and Upper Middle Income	West	Nursing Personnel
58	Respiratory Physician	694	783	870	0.20	50,808	Algeria	Maximum Estimate	2212 - Specialist medical practitioners	High Income and Upper Middle Income	West	Medical Doctors
59	Rheumatologist	259	287	317	0.07	138,954	Algeria	Maximum Estimate	2212 - Specialist medical practitioners	High Income and Upper Middle Income	West	Medical Doctors
60	Speech Therapist	1,030	1,122	1,279	0.29	34,063	Algeria	Maximum Estimate	2266 - Audiologists and speech therapists	High Income and Upper Middle Income	West	Other Health Workers
61	Urologist	242	285	327	0.07	133,488	Algeria	Maximum Estimate	2212 - Specialist medical practitioners	High Income and Upper Middle Income	West	Medical Doctors
1	Anaesthesiologist	441	502	568	0.17	58,292	Angola	Maximum Estimate	2212 - Specialist medical practitioners	Lower-middle Income	Southern	Medical Doctors

S/N	Health Professionals	2022	2026	2030	Density per 10,000 population	Required Population ratio (1 professional is to xxx population)	Name of Country	Modelling Scenario	ISCO-08 Match	Income Group Classification	Sub-Regional Grouping	SDG 3c Occupation
2	Associate Nurse/Enrolled Nurse/Nursing Assistant	59,441	67,085	75,616	22.85	438	Angola	Maximum Estimate	3221 - Nursing associate professionals	Lower-middle Income	Southern	Nursing Personnel
3	Audiologist	190	215	241	0.07	137,746	Angola	Maximum Estimate	2266 - Audiologists and speech therapists	Lower-middle Income	Southern	Other Health Workers
4	Cardiologist	433	507	590	0.18	56,112	Angola	Maximum Estimate	2212 - Specialist medical practitioners	Lower-middle Income	Southern	Medical Doctors
5	Cardiothoracic Surgeon	80	89	97	0.03	340,983	Angola	Maximum Estimate	2212 - Specialist medical practitioners	Lower-middle Income	Southern	Medical Doctors
6	Clinical Officer/Physician Assistant	9,838	10,791	11,927	3.62	2,762	Angola	Maximum Estimate	3256 - Medical assistants	Lower-middle Income	Southern	Other Health Workers
7	Clinical Pharmacist	1,512	1,685	1,889	0.57	17,486	Angola	Maximum Estimate	2262 - Pharmacists	Lower-middle Income	Southern	Pharmacist
8	Clinical Psychologist	3,332	3,723	4,171	1.26	7,930	Angola	Maximum Estimate	2634 - Psychologists	Lower-middle Income	Southern	Other Health Workers
9	Community health worker/Village health worker	47,178	53,194	59,873	18.11	552	Angola	Maximum Estimate	3253 - Community health workers	Lower-middle Income	Southern	Other Health Workers
10	Dental Surgery Assistant	4,613	5,249	5,976	1.80	5,542	Angola	Maximum Estimate	3251 - Dental assistants and therapists	Lower-middle Income	Southern	Other Health Workers
11	Dental Therapist	3,096	3,522	4,009	1.21	8,261	Angola	Maximum Estimate	3251 - Dental assistants and therapists	Lower-middle Income	Southern	Other Health Workers
12	Dentist	3,741	4,241	4,870	1.47	6,787	Angola	Maximum Estimate	2261 - Dentists	Lower-middle Income	Southern	Dentist
13	Dermatologist	166	187	210	0.06	158,289	Angola	Maximum Estimate	2212 - Specialist medical practitioners	Lower-middle Income	Southern	Medical Doctors
14	Endocrinologist	177	212	257	0.08	127,537	Angola	Maximum Estimate	2212 - Specialist medical practitioners	Lower-middle Income	Southern	Medical Doctors
15	ENT Surgeon	507	579	661	0.20	50,087	Angola	Maximum Estimate	2212 - Specialist medical practitioners	Lower-middle Income	Southern	Medical Doctors
16	Environmental Health Officer	2,449	2,756	3,084	0.93	10,758	Angola	Maximum Estimate	2263 - Environmental and occupational health and hygiene professionals	Lower-middle Income	Southern	Other Health Workers
17	Gastroenterologist	812	847	897	0.27	36,649	Angola	Maximum Estimate	2212 - Specialist medical practitioners	Lower-middle Income	Southern	Medical Doctors
18	General Medical Practitioner (Generalist Doctor)	19,561	22,079	24,952	7.55	1,324	Angola	Maximum Estimate	2211 - Generalist medical practitioners	Lower-middle Income	Southern	Medical Doctors
19	General Surgeon	440	521	625	0.19	52,533	Angola	Maximum Estimate	2212 - Specialist medical practitioners	Lower-middle Income	Southern	Medical Doctors
20	Haematologist	147	163	181	0.05	183,539	Angola	Maximum Estimate	2212 - Specialist medical practitioners	Lower-middle Income	Southern	Medical Doctors
21	Health Promoter/Health Educator	886	961	1,050	0.32	31,391	Angola	Maximum Estimate	2269 - Health professionals not elsewhere classified	Lower-middle Income	Southern	Other Health Workers
22	Infectious Diseases Specialist	82	89	97	0.03	342,033	Angola	Maximum Estimate	2212 - Specialist medical practitioners	Lower-middle Income	Southern	Medical Doctors
23	Intensive Care Nurse	1,845	2,043	2,253	0.68	14,715	Angola	Maximum Estimate	2221 - Nursing professionals	Lower-middle Income	Southern	Nursing Personnel
24	Medical Laboratory Scientist	6,291	7,159	8,196	2.49	4,023	Angola	Maximum Estimate	3212 - Medical and pathology laboratory technicians	Lower-middle Income	Southern	Other Health Workers
25	Medical Laboratory Technician	7,849	8,994	10,372	3.15	3,174	Angola	Maximum Estimate	3212 - Medical and pathology laboratory technicians	Lower-middle Income	Southern	Other Health Workers
26	Medical Social Worker	1,979	2,079	2,193	0.66	15,042	Angola	Maximum Estimate	1344 - Social welfare managers	Lower-middle Income	Southern	Other Health Workers
27	Mental Health Nurse	3,258	3,684	4,231	1.28	7,785	Angola	Maximum Estimate	2221 - Nursing professionals	Lower-middle Income	Southern	Nursing Personnel
28	Midwife	34,338	37,802	41,897	12.68	789	Angola	Maximum Estimate	2222 - Midwifery professionals	Lower-middle Income	Southern	Midwifery Personnel
29	Nephrologist	362	423	502	0.15	65,421	Angola	Maximum Estimate	2212 - Specialist medical practitioners	Lower-middle Income	Southern	Medical Doctors
30	Neuro-Surgeon	167	186	208	0.06	158,928	Angola	Maximum Estimate	2212 - Specialist medical practitioners	Lower-middle Income	Southern	Medical Doctors
31	Nurse Anaesthetist	924	1,066	1,226	0.37	27,007	Angola	Maximum Estimate	2221 - Nursing professionals	Lower-middle Income	Southern	Nursing Personnel
32	Nutritionist	5,463	5,735	5,988	1.81	5,527	Angola	Maximum Estimate	2265 - Dieticians and nutritionists	Lower-middle Income	Southern	Other Health Workers
33	Obstetrician & Gynaecologist	3,135	3,553	4,022	1.21	8,233	Angola	Maximum Estimate	2212 - Specialist medical practitioners	Lower-middle Income	Southern	Medical Doctors
34	Occupational Therapist	814	932	1,069	0.32	30,928	Angola	Maximum Estimate	2269 - Health professionals not elsewhere classified	Lower-middle Income	Southern	Other Health Workers
35	Oncology Nurse	310	360	423	0.13	77,911	Angola	Maximum Estimate	2221 - Nursing professionals	Lower-middle Income	Southern	Nursing Personnel
36	Operating Theatre Nurse	4,406	5,049	5,847	1.78	5,633	Angola	Maximum Estimate	2221 - Nursing professionals	Lower-middle Income	Southern	Nursing Personnel
37	Ophthalmic Nurse	653	750	861	0.26	38,418	Angola	Maximum Estimate	2221 - Nursing professionals	Lower-middle Income	Southern	Nursing Personnel
38	Ophthalmologist	168	195	227	0.07	145,556	Angola	Maximum Estimate	2212 - Specialist medical practitioners	Lower-middle Income	Southern	Medical Doctors
39	Optometrist	659	759	873	0.26	37,880	Angola	Maximum Estimate	2267 - Optometrists and ophthalmic opticians	Lower-middle Income	Southern	Other Health Workers
40	Orthopaedic Nurse	981	1,122	1,276	0.38	25,982	Angola	Maximum Estimate	2221 - Nursing professionals	Lower-middle Income	Southern	Nursing Personnel
41	Orthopaedic Surgeon	1,569	2,014	2,597	0.80	12,507	Angola	Maximum Estimate	2212 - Specialist medical practitioners	Lower-middle Income	Southern	Medical Doctors
42	Orthopaedic Technologist	1,365	1,538	1,724	0.52	19,245	Angola	Maximum Estimate	3214 - Medical and dental prosthetic technicians	Lower-middle Income	Southern	Other Health Workers
43	Paediatric Nurse	5,221	5,754	6,261	1.89	5,297	Angola	Maximum Estimate	2221 - Nursing professionals	Lower-middle Income	Southern	Nursing Personnel
44	Paediatric Surgeon	201	220	238	0.07	139,591	Angola	Maximum Estimate	2212 - Specialist medical practitioners	Lower-middle Income	Southern	Medical Doctors
45	Paediatrician	2,036	2,250	2,484	0.75	13,321	Angola	Maximum Estimate	2212 - Specialist medical practitioners	Lower-middle Income	Southern	Medical Doctors
46	Pathologist	411	468	539	0.16	60,999	Angola	Maximum Estimate	2212 - Specialist medical practitioners	Lower-middle Income	Southern	Medical Doctors
47	Pharmacist	5,383	5,662	5,957	1.80	5,544	Angola	Maximum Estimate	2262 - Pharmacists	Lower-middle Income	Southern	Pharmacist
48	Pharmacy Technician	5,747	6,364	7,053	2.13	4,691	Angola	Maximum Estimate	3213 - Pharmaceutical technicians and assistants	Lower-middle Income	Southern	Other Health Workers
49	Physician	5,000	5,623	6,341	1.92	5,211	Angola	Maximum Estimate	2212 - Specialist medical practitioners	Lower-middle Income	Southern	Medical Doctors
50	Physiotherapist	1,191	1,346	1,520	0.46	21,793	Angola	Maximum Estimate	2264 - Physiotherapists	Lower-middle Income	Southern	Other Health Workers
51	Plastic Surgeon	248	280	313	0.09	106,005	Angola	Maximum Estimate	2212 - Specialist medical practitioners	Lower-middle Income	Southern	Medical Doctors
52	Psychiatrist	2,537	2,850	3,247	0.98	10,154	Angola	Maximum Estimate	2212 - Specialist medical practitioners	Lower-middle Income	Southern	Medical Doctors
53	Radiation Oncologist	115	136	161	0.05	204,617	Angola	Maximum Estimate	2212 - Specialist medical practitioners	Lower-middle Income	Southern	Medical Doctors
54	Radiographer (Diagnostics and Therapy)	3,641	4,184	4,854	1.47	6,785	Angola	Maximum Estimate	3211 - Medical imaging and therapeutic equipment technicians	Lower-middle Income	Southern	Other Health Workers
55	Radiologist	1,459	1,681	1,953	0.59	16,864	Angola	Maximum Estimate	2212 - Specialist medical practitioners	Lower-middle Income	Southern	Medical Doctors
56	Registered General Nurse / State Certified Nurse	123,538	134,863	147,733	44.78	223	Angola	Maximum Estimate	2221 - Nursing professionals	Lower-middle Income	Southern	Nursing Personnel
57	Renal Nurse	7,074	8,261	9,796	2.98	3,351	Angola	Maximum Estimate	2221 - Nursing professionals	Lower-middle Income	Southern	Nursing Personnel
58	Respiratory Physician	391	427	464	0.14	71,464	Angola	Maximum Estimate	2212 - Specialist medical practitioners	Lower-middle Income	Southern	Medical Doctors
59	Rheumatologist	126	145	165	0.05	201,041	Angola	Maximum Estimate	2212 - Specialist medical practitioners	Lower-middle Income	Southern	Medical Doctors
60	Speech Therapist	405	456	512	0.15	64,626	Angola	Maximum Estimate	2266 - Audiologists and speech therapists	Lower-middle Income	Southern	Other Health Workers
61	Urologist	53	61	72	0.02	458,808	Angola	Maximum Estimate	2212 - Specialist medical practitioners	Lower-middle Income	Southern	Medical Doctors
1	Anaesthesiologist	203	230	260	0.21	46,966	Benin	Maximum Estimate	2212 - Specialist medical practitioners	Low Income	West	Medical Doctors
2	Associate Nurse/Enrolled Nurse/Nursing Assistant	20,550	22,894	25,493	20.87	479	Benin	Maximum Estimate	3221 - Nursing associate professionals	Low Income	West	Nursing Personnel
3	Audiologist	79	89	99	0.08	123,400	Benin	Maximum Estimate	2266 - Audiologists and speech therapists	Low Income	West	Other Health Workers
4	Cardiologist	159	181	205	0.17	59,786	Benin	Maximum Estimate	2212 - Specialist medical practitioners	Low Income	West	Medical Doctors
5	Cardiothoracic Surgeon	36	41	46	0.04	265,703	Benin	Maximum Estimate	2212 - Specialist medical practitioners	Low Income	West	Medical Doctors
6	Clinical Officer/Physician Assistant	3,090	3,398	3,753	3.08	3,248	Benin	Maximum Estimate	3256 - Medical assistants	Low Income	West	Other Health Workers
7	Clinical Pharmacist	565	627	702	0.58	17,354	Benin	Maximum Estimate	2262 - Pharmacists	Low Income	West	Pharmacist
8	Clinical Psychologist	950	1,049	1,165	0.95	10,477	Benin	Maximum Estimate	2634 - Psychologists	Low Income	West	Other Health Workers
9	Community health worker/Village health worker	22,621	25,072	27,707	22.68	441	Benin	Maximum Estimate	3253 - Community health workers	Low Income	West	Other Health Workers
10	Dental Surgery Assistant	1,879	2,109	2,365	1.93	5,169	Benin	Maximum Estimate	3251 - Dental assistants and therapists	Low Income	West	Other Health Workers
11	Dental Therapist	1,260	1,413	1,584	1.30	7,716	Benin	Maximum Estimate	3251 - Dental assistants and therapists	Low Income	West	Other Health Workers
12	Dentist	1,513	1,685	1,892	1.55	6,454	Benin	Maximum Estimate	2261 - Dentists	Low Income	West	Dentist
13	Dermatologist	60	67	75	0.06	163,386	Benin	Maximum Estimate	2212 - Specialist medical practitioners	Low Income	West	Medical Doctors
14	Endocrinologist	57	66	79	0.07	153,151	Benin	Maximum Estimate	2212 - Specialist medical practitioners	Low Income	West	Medical Doctors
15	ENT Surgeon	212	240	270	0.22	45,137	Benin	Maximum Estimate	2212 - Specialist medical practitioners	Low Income	West	Medical Doctors
16	Environmental Health Officer	903	1,003	1,107	0.91	11,048	Benin	Maximum Estimate	2263 - Environmental and occupational health and hygiene professionals	Low Income	West	Other Health Workers
17	Gastroenterologist	224	226	234	0.19	51,811	Benin	Maximum Estimate	2212 - Specialist medical practitioners	Low Income	West	Medical Doctors
18	General Medical Practitioner (Generalist Doctor)	7,131	7,905	8,757	7.17	1,395	Benin	Maximum Estimate	2211 - Generalist medical practitioners	Low Income	West	Medical Doctors
19	General Surgeon	162	185	214	0.18	56,838	Benin	Maximum Estimate	2212 - Specialist medical practitioners	Low Income	West	Medical Doctors
20	Haematologist	65	71	76	0.06	160,159	Benin	Maximum Estimate	2212 - Specialist medical practitioners	Low Income	West	Medical Doctors
21	Health Promoter/Health Educator	306	335	370	0.30	32,888	Benin	Maximum Estimate	2269 - Health professionals not elsewhere classified	Low Income	West	Other Health Workers
22	Infectious Diseases Specialist	25	28	31	0.03	398,603	Benin	Maximum Estimate	2212 - Specialist medical practitioners	Low Income	West	Medical Doctors
23	Intensive Care Nurse	629	702	781	0.64	15,650	Benin	Maximum Estimate	2221 - Nursing professionals	Low Income	West	Nursing Personnel
24	Medical Laboratory Scientist	2,312	2,578	2,899	2.38	4,198	Benin	Maximum Estimate	3212 - Medical and pathology laboratory technicians	Low Income	West	Other Health Workers

S/N	Health Professionals	2022	2026	2030	Density per 10,000 population	Required Population ratio (1 professional is to xxx population)	Name of Country	Modelling Scenario	ISCO-08 Match	Income Group Classification	Sub-Regional Grouping	SDG 3c Occupation
25	Medical Laboratory Technician	4,319	4,880	5,511	4.52	2,214	Benin	Maximum Estimate	3212 - Medical and pathology laboratory technicians	Low Income	West	Other Health Workers
26	Medical Social Worker	752	800	862	0.71	14,042	Benin	Maximum Estimate	1344 - Social welfare managers	Low Income	West	Other Health Workers
27	Mental Health Nurse	1,136	1,278	1,464	1.20	8,317	Benin	Maximum Estimate	2221 - Nursing professionals	Low Income	West	Nursing Personnel
28	Midwife	13,515	14,851	16,328	13.36	749	Benin	Maximum Estimate	2222 - Midwifery professionals	Low Income	West	Midwifery Personnel
29	Nephrologist	162	185	216	0.18	56,252	Benin	Maximum Estimate	2212 - Specialist medical practitioners	Low Income	West	Medical Doctors
30	Neuro-Surgeon	72	81	91	0.07	134,052	Benin	Maximum Estimate	2212 - Specialist medical practitioners	Low Income	West	Medical Doctors
31	Nurse Anaesthetist	354	403	457	0.37	26,715	Benin	Maximum Estimate	2221 - Nursing professionals	Low Income	West	Nursing Personnel
32	Nutritionist	2,875	3,067	3,253	2.66	3,757	Benin	Maximum Estimate	2265 - Dieticians and nutritionists	Low Income	West	Other Health Workers
33	Obstetrician & Gynaecologist	1,053	1,183	1,335	1.10	9,108	Benin	Maximum Estimate	2212 - Specialist medical practitioners	Low Income	West	Medical Doctors
34	Occupational Therapist	370	423	485	0.40	25,185	Benin	Maximum Estimate	2269 - Health professionals not elsewhere classified	Low Income	West	Other Health Workers
35	Oncology Nurse	123	140	162	0.13	75,327	Benin	Maximum Estimate	2221 - Nursing professionals	Low Income	West	Nursing Personnel
36	Operating Theatre Nurse	1,770	1,994	2,278	1.87	5,348	Benin	Maximum Estimate	2221 - Nursing professionals	Low Income	West	Nursing Personnel
37	Ophthalmic Nurse	193	219	250	0.21	48,735	Benin	Maximum Estimate	2221 - Nursing professionals	Low Income	West	Nursing Personnel
38	Ophthalmologist	66	75	85	0.07	142,672	Benin	Maximum Estimate	2212 - Specialist medical practitioners	Low Income	West	Medical Doctors
39	Optometrist	258	291	328	0.27	37,194	Benin	Maximum Estimate	2267 - Optometrists and ophthalmic opticians	Low Income	West	Other Health Workers
40	Orthopaedic Nurse	422	491	568	0.47	21,503	Benin	Maximum Estimate	2221 - Nursing professionals	Low Income	West	Nursing Personnel
41	Orthopaedic Surgeon	565	649	748	0.61	16,313	Benin	Maximum Estimate	2212 - Specialist medical practitioners	Low Income	West	Medical Doctors
42	Orthopaedic Technologist	534	602	676	0.55	18,089	Benin	Maximum Estimate	3214 - Medical and dental prosthetic technicians	Low Income	West	Other Health Workers
43	Paediatric Nurse	2,151	2,347	2,533	2.07	4,832	Benin	Maximum Estimate	2221 - Nursing professionals	Low Income	West	Nursing Personnel
44	Paediatric Surgeon	73	81	88	0.07	139,200	Benin	Maximum Estimate	2212 - Specialist medical practitioners	Low Income	West	Medical Doctors
45	Paediatrician	863	964	1,077	0.88	11,347	Benin	Maximum Estimate	2212 - Specialist medical practitioners	Low Income	West	Medical Doctors
46	Pathologist	106	116	129	0.11	94,292	Benin	Maximum Estimate	2212 - Specialist medical practitioners	Low Income	West	Medical Doctors
47	Pharmacist	1,121	1,177	1,243	1.02	9,768	Benin	Maximum Estimate	2262 - Pharmacists	Low Income	West	Pharmacist
48	Pharmacy Technician	2,018	2,239	2,486	2.04	4,909	Benin	Maximum Estimate	3213 - Pharmaceutical technicians and assistants	Low Income	West	Other Health Workers
49	Physician	2,128	2,374	2,654	2.17	4,602	Benin	Maximum Estimate	2212 - Specialist medical practitioners	Low Income	West	Medical Doctors
50	Physiotherapist	465	522	587	0.48	20,805	Benin	Maximum Estimate	2264 - Physiotherapists	Low Income	West	Other Health Workers
51	Plastic Surgeon	123	140	160	0.13	76,433	Benin	Maximum Estimate	2212 - Specialist medical practitioners	Low Income	West	Medical Doctors
52	Psychiatrist	977	1,086	1,224	1.00	9,956	Benin	Maximum Estimate	2212 - Specialist medical practitioners	Low Income	West	Medical Doctors
53	Radiation Oncologist	37	43	49	0.04	250,273	Benin	Maximum Estimate	2212 - Specialist medical practitioners	Low Income	West	Medical Doctors
54	Radiographer (Diagnostics and Therapy)	1,417	1,601	1,829	1.50	6,647	Benin	Maximum Estimate	3211 - Medical imaging and therapeutic equipment technicians	Low Income	West	Other Health Workers
55	Radiologist	543	624	728	0.60	16,570	Benin	Maximum Estimate	2212 - Specialist medical practitioners	Low Income	West	Medical Doctors
56	Registered General Nurse / State Certified Nurse	48,767	52,562	56,924	46.74	214	Benin	Maximum Estimate	2221 - Nursing professionals	Low Income	West	Nursing Personnel
57	Renal Nurse	3,114	3,564	4,166	3.43	2,919	Benin	Maximum Estimate	2221 - Nursing professionals	Low Income	West	Nursing Personnel
58	Respiratory Physician	141	160	180	0.15	67,775	Benin	Maximum Estimate	2212 - Specialist medical practitioners	Low Income	West	Medical Doctors
59	Rheumatologist	45	50	56	0.05	218,187	Benin	Maximum Estimate	2212 - Specialist medical practitioners	Low Income	West	Medical Doctors
60	Speech Therapist	162	181	201	0.17	60,593	Benin	Maximum Estimate	2266 - Audiologists and speech therapists	Low Income	West	Other Health Workers
61	Urologist	26	32	40	0.03	297,950	Benin	Maximum Estimate	2212 - Specialist medical practitioners	Low Income	West	Medical Doctors
1	Anaesthesiologist	52	58	65	0.28	36,359	Botswana	Maximum Estimate	2212 - Specialist medical practitioners	High Income and Upper Middle Income	Southern	Medical Doctors
2	Associate Nurse/Enrolled Nurse/Nursing Assistant	4,916	5,310	5,732	24.23	413	Botswana	Maximum Estimate	3221 - Nursing associate professionals	High Income and Upper Middle Income	Southern	Nursing Personnel
3	Audiologist	17	18	19	0.08	122,235	Botswana	Maximum Estimate	2266 - Audiologists and speech therapists	High Income and Upper Middle Income	Southern	Other Health Workers
4	Cardiologist	48	55	62	0.26	38,130	Botswana	Maximum Estimate	2212 - Specialist medical practitioners	High Income and Upper Middle Income	Southern	Medical Doctors
5	Cardiothoracic Surgeon	9	10	12	0.05	205,419	Botswana	Maximum Estimate	2212 - Specialist medical practitioners	High Income and Upper Middle Income	Southern	Medical Doctors
6	Clinical Officer/Physician Assistant	711	759	815	3.45	2,897	Botswana	Maximum Estimate	3256 - Medical assistants	High Income and Upper Middle Income	Southern	Other Health Workers
7	Clinical Pharmacist	127	140	157	0.66	15,063	Botswana	Maximum Estimate	2262 - Pharmacists	High Income and Upper Middle Income	Southern	Pharmacist
8	Clinical Psychologist	209	226	245	1.03	9,666	Botswana	Maximum Estimate	2634 - Psychologists	High Income and Upper Middle Income	Southern	Other Health Workers
9	Community health worker/Village health worker	2,270	2,359	2,427	10.27	973	Botswana	Maximum Estimate	3253 - Community health workers	High Income and Upper Middle Income	Southern	Other Health Workers
10	Dental Surgery Assistant	482	534	590	2.48	4,027	Botswana	Maximum Estimate	3251 - Dental assistants and therapists	High Income and Upper Middle Income	Southern	Other Health Workers
11	Dental Therapist	322	357	394	1.66	6,033	Botswana	Maximum Estimate	3251 - Dental assistants and therapists	High Income and Upper Middle Income	Southern	Other Health Workers
12	Dentist	392	424	459	1.93	5,179	Botswana	Maximum Estimate	2261 - Dentists	High Income and Upper Middle Income	Southern	Dentist
13	Dermatologist	13	15	16	0.07	151,459	Botswana	Maximum Estimate	2212 - Specialist medical practitioners	High Income and Upper Middle Income	Southern	Medical Doctors
14	Endocrinologist	29	35	43	0.18	55,059	Botswana	Maximum Estimate	2212 - Specialist medical practitioners	High Income and Upper Middle Income	Southern	Medical Doctors
15	ENT Surgeon	61	67	76	0.32	31,392	Botswana	Maximum Estimate	2212 - Specialist medical practitioners	High Income and Upper Middle Income	Southern	Medical Doctors
16	Environmental Health Officer	175	187	198	0.84	11,971	Botswana	Maximum Estimate	2263 - Environmental and occupational health and hygiene professionals	High Income and Upper Middle Income	Southern	Other Health Workers
17	Gastroenterologist	32	33	34	0.14	69,507	Botswana	Maximum Estimate	2212 - Specialist medical practitioners	High Income and Upper Middle Income	Southern	Medical Doctors
18	General Medical Practitioner (Generalist Doctor)	1,629	1,757	1,891	7.99	1,252	Botswana	Maximum Estimate	2211 - Generalist medical practitioners	High Income and Upper Middle Income	Southern	Medical Doctors
19	General Surgeon	63	71	80	0.34	29,295	Botswana	Maximum Estimate	2212 - Specialist medical practitioners	High Income and Upper Middle Income	Southern	Medical Doctors
20	Haematologist	9	10	12	0.05	204,910	Botswana	Maximum Estimate	2212 - Specialist medical practitioners	High Income and Upper Middle Income	Southern	Medical Doctors
21	Health Promoter/Health Educator	65	69	73	0.31	32,269	Botswana	Maximum Estimate	2269 - Health professionals not elsewhere classified	High Income and Upper Middle Income	Southern	Other Health Workers
22	Infectious Diseases Specialist	6	7	7	0.03	336,318	Botswana	Maximum Estimate	2212 - Specialist medical practitioners	High Income and Upper Middle Income	Southern	Medical Doctors
23	Intensive Care Nurse	115	124	133	0.56	17,764	Botswana	Maximum Estimate	2221 - Nursing professionals	High Income and Upper Middle Income	Southern	Nursing Personnel
24	Medical Laboratory Scientist	660	718	788	3.34	2,993	Botswana	Maximum Estimate	3212 - Medical and pathology laboratory technicians	High Income and Upper Middle Income	Southern	Other Health Workers
25	Medical Laboratory Technician	518	567	623	2.64	3,794	Botswana	Maximum Estimate	3212 - Medical and pathology laboratory technicians	High Income and Upper Middle Income	Southern	Other Health Workers
26	Medical Social Worker	96	99	101	0.43	23,251	Botswana	Maximum Estimate	1344 - Social welfare managers	High Income and Upper Middle Income	Southern	Other Health Workers
27	Mental Health Nurse	299	325	358	1.52	6,592	Botswana	Maximum Estimate	2221 - Nursing professionals	High Income and Upper Middle Income	Southern	Nursing Personnel
28	Midwife	2,394	2,566	2,728	11.50	870	Botswana	Maximum Estimate	2222 - Midwifery professionals	High Income and Upper Middle Income	Southern	Midwifery Personnel
29	Nephrologist	65	75	89	0.38	26,357	Botswana	Maximum Estimate	2212 - Specialist medical practitioners	High Income and Upper Middle Income	Southern	Medical Doctors
30	Neuro-Surgeon	20	23	27	0.11	88,404	Botswana	Maximum Estimate	2212 - Specialist medical practitioners	High Income and Upper Middle Income	Southern	Medical Doctors
31	Nurse Anaesthetist	214	257	309	1.31	7,629	Botswana	Maximum Estimate	2221 - Nursing professionals	High Income and Upper Middle Income	Southern	Nursing Personnel
32	Nutritionist	292	300	304	1.29	7,769	Botswana	Maximum Estimate	2265 - Dieticians and nutritionists	High Income and Upper Middle Income	Southern	Other Health Workers
33	Obstetrician & Gynaecologist	290	311	333	1.40	7,122	Botswana	Maximum Estimate	2212 - Specialist medical practitioners	High Income and Upper Middle Income	Southern	Medical Doctors
34	Occupational Therapist	90	104	121	0.51	19,458	Botswana	Maximum Estimate	2269 - Health professionals not elsewhere classified	High Income and Upper Middle Income	Southern	Other Health Workers
35	Oncology Nurse	69	83	100	0.42	23,626	Botswana	Maximum Estimate	2221 - Nursing professionals	High Income and Upper Middle Income	Southern	Nursing Personnel
36	Operating Theatre Nurse	578	643	732	3.11	3,218	Botswana	Maximum Estimate	2221 - Nursing professionals	High Income and Upper Middle Income	Southern	Nursing Personnel
37	Ophthalmic Nurse	61	69	79	0.33	29,864	Botswana	Maximum Estimate	2221 - Nursing professionals	High Income and Upper Middle Income	Southern	Nursing Personnel
38	Ophthalmologist	28	33	38	0.16	61,930	Botswana	Maximum Estimate	2212 - Specialist medical practitioners	High Income and Upper Middle Income	Southern	Medical Doctors
39	Optometrist	147	170	197	0.83	12,022	Botswana	Maximum Estimate	2267 - Optometrists and ophthalmic opticians	High Income and Upper Middle Income	Southern	Other Health Workers
40	Orthopaedic Nurse	104	122	143	0.61	16,510	Botswana	Maximum Estimate	2221 - Nursing professionals	High Income and Upper Middle Income	Southern	Nursing Personnel
41	Orthopaedic Surgeon	190	217	249	1.05	9,528	Botswana	Maximum Estimate	2212 - Specialist medical practitioners	High Income and Upper Middle Income	Southern	Medical Doctors
42	Orthopaedic Technologist	114	127	140	0.59	16,945	Botswana	Maximum Estimate	3214 - Medical and dental prosthetic technicians	High Income and Upper Middle Income	Southern	Other Health Workers
43	Paediatric Nurse	283	297	304	1.29	7,769	Botswana	Maximum Estimate	2221 - Nursing professionals	High Income and Upper Middle Income	Southern	Nursing Personnel
44	Paediatric Surgeon	9	9	9	0.04	249,543	Botswana	Maximum Estimate	2212 - Specialist medical practitioners	High Income and Upper Middle Income	Southern	Medical Doctors
45	Paediatrician	151	168	188	0.80	12,535	Botswana	Maximum Estimate	2212 - Specialist medical practitioners	High Income and Upper Middle Income	Southern	Medical Doctors
46	Pathologist	54	59	65	0.28	36,010	Botswana	Maximum Estimate	2212 - Specialist medical practitioners	High Income and Upper Middle Income	Southern	Medical Doctors
47	Pharmacist	370	392	417	1.77	5,651	Botswana	Maximum Estimate	2262 - Pharmacists	High Income and Upper Middle Income	Southern	Pharmacist

S/N	Health Professionals	2022	2026	2030	Density per 10,000 population	Required Population ratio (1 professional is to xxx population)	Name of Country	Modelling Scenario	ISCO-08 Match	Income Group Classification	Sub-Regional Grouping	SDG 3c Occupation
48	Pharmacy Technician	482	525	571	2.41	4,145	Botswana	Maximum Estimate	3213 - Pharmaceutical technicians and assistants	High Income and Upper Middle Income	Southern	Other Health Workers
49	Physician	438	487	544	2.30	4,346	Botswana	Maximum Estimate	2212 - Specialist medical practitioners	High Income and Upper Middle Income	Southern	Medical Doctors
50	Physiotherapist	109	120	135	0.57	17,459	Botswana	Maximum Estimate	2264 - Physiotherapists	High Income and Upper Middle Income	Southern	Other Health Workers
51	Plastic Surgeon	40	44	49	0.21	48,350	Botswana	Maximum Estimate	2212 - Specialist medical practitioners	High Income and Upper Middle Income	Southern	Medical Doctors
52	Psychiatrist	213	230	251	1.06	9,397	Botswana	Maximum Estimate	2212 - Specialist medical practitioners	High Income and Upper Middle Income	Southern	Medical Doctors
53	Radiation Oncologist	34	42	51	0.22	45,691	Botswana	Maximum Estimate	2212 - Specialist medical practitioners	High Income and Upper Middle Income	Southern	Medical Doctors
54	Radiographer (Diagnostics and Therapy)	419	468	527	2.23	4,478	Botswana	Maximum Estimate	3211 - Medical imaging and therapeutic equipment technicians	High Income and Upper Middle Income	Southern	Other Health Workers
55	Radiologist	159	177	198	0.84	11,942	Botswana	Maximum Estimate	2212 - Specialist medical practitioners	High Income and Upper Middle Income	Southern	Medical Doctors
56	Registered General Nurse / State Certified Nurse	10,106	11,165	12,429	52.76	190	Botswana	Maximum Estimate	2221 - Nursing professionals	High Income and Upper Middle Income	Southern	Nursing Personnel
57	Renal Nurse	1,284	1,497	1,778	7.57	1,321	Botswana	Maximum Estimate	2221 - Nursing professionals	High Income and Upper Middle Income	Southern	Nursing Personnel
58	Respiratory Physician	28	32	35	0.15	66,852	Botswana	Maximum Estimate	2212 - Specialist medical practitioners	High Income and Upper Middle Income	Southern	Medical Doctors
59	Rheumatologist	12	14	15	0.07	153,270	Botswana	Maximum Estimate	2212 - Specialist medical practitioners	High Income and Upper Middle Income	Southern	Medical Doctors
60	Speech Therapist	35	38	43	0.18	55,444	Botswana	Maximum Estimate	2266 - Audiologists and speech therapists	High Income and Upper Middle Income	Southern	Other Health Workers
61	Urologist	12	14	17	0.07	134,392	Botswana	Maximum Estimate	2212 - Specialist medical practitioners	High Income and Upper Middle Income	Southern	Medical Doctors
1	Anaesthesiologist	379	441	511	0.24	41,147	Burkina Faso	Maximum Estimate	2212 - Specialist medical practitioners	Low Income	West	Medical Doctors
2	Associate Nurse/Enrolled Nurse/Nursing Assistant	40,861	45,103	49,854	23.69	422	Burkina Faso	Maximum Estimate	3221 - Nursing associate professionals	Low Income	West	Nursing Personnel
3	Audiologist	136	151	166	0.08	126,839	Burkina Faso	Maximum Estimate	2266 - Audiologists and speech therapists	Low Income	West	Other Health Workers
4	Cardiologist	233	258	284	0.13	74,146	Burkina Faso	Maximum Estimate	2212 - Specialist medical practitioners	Low Income	West	Medical Doctors
5	Cardiothoracic Surgeon	72	84	98	0.05	215,389	Burkina Faso	Maximum Estimate	2212 - Specialist medical practitioners	Low Income	West	Medical Doctors
6	Clinical Officer/Physician Assistant	4,023	3,954	3,954	1.91	5,245	Burkina Faso	Maximum Estimate	3256 - Medical assistants	Low Income	West	Other Health Workers
7	Clinical Pharmacist	1,004	1,109	1,245	0.60	16,790	Burkina Faso	Maximum Estimate	2262 - Pharmacists	Low Income	West	Pharmacist
8	Clinical Psychologist	1,462	1,607	1,789	0.85	11,733	Burkina Faso	Maximum Estimate	2634 - Psychologists	Low Income	West	Other Health Workers
9	Community health worker/Village health worker	38,594	40,298	42,442	20.31	492	Burkina Faso	Maximum Estimate	3253 - Community health workers	Low Income	West	Other Health Workers
10	Dental Surgery Assistant	3,103	3,453	3,856	1.83	5,465	Burkina Faso	Maximum Estimate	3251 - Dental assistants and therapists	Low Income	West	Other Health Workers
11	Dental Therapist	2,077	2,310	2,579	1.22	8,170	Burkina Faso	Maximum Estimate	3251 - Dental assistants and therapists	Low Income	West	Other Health Workers
12	Dentist	2,388	2,636	2,958	1.40	7,119	Burkina Faso	Maximum Estimate	2261 - Dentists	Low Income	West	Dentist
13	Dermatologist	104	115	128	0.06	164,888	Burkina Faso	Maximum Estimate	2212 - Specialist medical practitioners	Low Income	West	Medical Doctors
14	Endocrinologist	89	104	125	0.06	166,895	Burkina Faso	Maximum Estimate	2212 - Specialist medical practitioners	Low Income	West	Medical Doctors
15	ENT Surgeon	348	390	436	0.21	48,250	Burkina Faso	Maximum Estimate	2212 - Specialist medical practitioners	Low Income	West	Medical Doctors
16	Environmental Health Officer	1,557	1,719	1,888	0.90	11,171	Burkina Faso	Maximum Estimate	2263 - Environmental and occupational health and hygiene professionals	Low Income	West	Other Health Workers
17	Gastroenterologist	521	526	546	0.26	38,215	Burkina Faso	Maximum Estimate	2212 - Specialist medical practitioners	Low Income	West	Medical Doctors
18	General Medical Practitioner (Generalist Doctor)	12,583	13,679	14,938	7.11	1,406	Burkina Faso	Maximum Estimate	2211 - Generalist medical practitioners	Low Income	West	Medical Doctors
19	General Surgeon	296	336	388	0.18	54,228	Burkina Faso	Maximum Estimate	2212 - Specialist medical practitioners	Low Income	West	Medical Doctors
20	Haematologist	178	206	238	0.11	88,150	Burkina Faso	Maximum Estimate	2212 - Specialist medical practitioners	Low Income	West	Medical Doctors
21	Health Promoter/Health Educator	421	424	437	0.21	47,347	Burkina Faso	Maximum Estimate	2269 - Health professionals not elsewhere classified	Low Income	West	Other Health Workers
22	Infectious Diseases Specialist	87	94	100	0.05	209,571	Burkina Faso	Maximum Estimate	2212 - Specialist medical practitioners	Low Income	West	Medical Doctors
23	Intensive Care Nurse	1,239	1,369	1,511	0.72	13,936	Burkina Faso	Maximum Estimate	2221 - Nursing professionals	Low Income	West	Nursing Personnel
24	Medical Laboratory Scientist	4,128	4,430	4,851	2.33	4,297	Burkina Faso	Maximum Estimate	3212 - Medical and pathology laboratory technicians	Low Income	West	Other Health Workers
25	Medical Laboratory Technician	8,039	8,684	9,597	4.62	2,163	Burkina Faso	Maximum Estimate	3212 - Medical and pathology laboratory technicians	Low Income	West	Other Health Workers
26	Medical Social Worker	1,675	1,749	1,862	0.90	11,144	Burkina Faso	Maximum Estimate	1344 - Social welfare managers	Low Income	West	Other Health Workers
27	Mental Health Nurse	1,852	2,055	2,334	1.11	8,980	Burkina Faso	Maximum Estimate	2221 - Nursing professionals	Low Income	West	Nursing Personnel
28	Midwife	31,899	36,992	43,084	20.52	487	Burkina Faso	Maximum Estimate	2222 - Midwifery professionals	Low Income	West	Midwifery Personnel
29	Nephrologist	252	285	331	0.16	63,267	Burkina Faso	Maximum Estimate	2212 - Specialist medical practitioners	Low Income	West	Medical Doctors
30	Neuro-Surgeon	100	118	139	0.07	150,893	Burkina Faso	Maximum Estimate	2212 - Specialist medical practitioners	Low Income	West	Medical Doctors
31	Nurse Anaesthetist	697	823	970	0.46	21,659	Burkina Faso	Maximum Estimate	2221 - Nursing professionals	Low Income	West	Nursing Personnel
32	Nutritionist	6,800	7,156	7,511	3.57	2,799	Burkina Faso	Maximum Estimate	2265 - Dieticians and nutritionists	Low Income	West	Other Health Workers
33	Obstetrician & Gynaecologist	1,791	2,022	2,306	1.10	9,053	Burkina Faso	Maximum Estimate	2212 - Specialist medical practitioners	Low Income	West	Medical Doctors
34	Occupational Therapist	648	758	890	0.42	23,570	Burkina Faso	Maximum Estimate	2269 - Health professionals not elsewhere classified	Low Income	West	Other Health Workers
35	Oncology Nurse	216	250	293	0.14	71,524	Burkina Faso	Maximum Estimate	2221 - Nursing professionals	Low Income	West	Nursing Personnel
36	Operating Theatre Nurse	2,969	3,367	3,889	1.86	5,389	Burkina Faso	Maximum Estimate	2221 - Nursing professionals	Low Income	West	Nursing Personnel
37	Ophthalmic Nurse	317	355	399	0.19	52,671	Burkina Faso	Maximum Estimate	2221 - Nursing professionals	Low Income	West	Nursing Personnel
38	Ophthalmologist	111	125	142	0.07	147,644	Burkina Faso	Maximum Estimate	2212 - Specialist medical practitioners	Low Income	West	Medical Doctors
39	Optometrist	471	525	586	0.28	35,916	Burkina Faso	Maximum Estimate	2267 - Optometrists and ophthalmic opticians	Low Income	West	Other Health Workers
40	Orthopaedic Nurse	685	815	965	0.46	21,788	Burkina Faso	Maximum Estimate	2221 - Nursing professionals	Low Income	West	Nursing Personnel
41	Orthopaedic Surgeon	1,000	1,147	1,318	0.63	15,958	Burkina Faso	Maximum Estimate	2212 - Specialist medical practitioners	Low Income	West	Medical Doctors
42	Orthopaedic Technologist	917	1,038	1,171	0.56	17,995	Burkina Faso	Maximum Estimate	3214 - Medical and dental prosthetic technicians	Low Income	West	Other Health Workers
43	Paediatric Nurse	4,842	5,385	5,948	2.83	3,539	Burkina Faso	Maximum Estimate	2221 - Nursing professionals	Low Income	West	Nursing Personnel
44	Paediatric Surgeon	141	158	173	0.08	121,949	Burkina Faso	Maximum Estimate	2212 - Specialist medical practitioners	Low Income	West	Medical Doctors
45	Paediatrician	1,522	1,693	1,892	0.90	11,104	Burkina Faso	Maximum Estimate	2212 - Specialist medical practitioners	Low Income	West	Medical Doctors
46	Pathologist	805	867	938	0.45	22,437	Burkina Faso	Maximum Estimate	2212 - Specialist medical practitioners	Low Income	West	Medical Doctors
47	Pharmacist	2,001	2,091	2,202	1.05	9,495	Burkina Faso	Maximum Estimate	2262 - Pharmacists	Low Income	West	Pharmacist
48	Pharmacy Technician	3,556	3,923	4,346	2.07	4,835	Burkina Faso	Maximum Estimate	3213 - Pharmaceutical technicians and assistants	Low Income	West	Other Health Workers
49	Physician	4,042	4,489	5,019	2.39	4,183	Burkina Faso	Maximum Estimate	2212 - Specialist medical practitioners	Low Income	West	Medical Doctors
50	Physiotherapist	780	876	985	0.47	21,373	Burkina Faso	Maximum Estimate	2264 - Physiotherapists	Low Income	West	Other Health Workers
51	Plastic Surgeon	210	244	282	0.13	74,634	Burkina Faso	Maximum Estimate	2212 - Specialist medical practitioners	Low Income	West	Medical Doctors
52	Psychiatrist	1,717	1,875	2,084	0.99	10,068	Burkina Faso	Maximum Estimate	2212 - Specialist medical practitioners	Low Income	West	Medical Doctors
53	Radiation Oncologist	74	88	105	0.05	199,466	Burkina Faso	Maximum Estimate	2212 - Specialist medical practitioners	Low Income	West	Medical Doctors
54	Radiographer (Diagnostics and Therapy)	2,444	2,742	3,133	1.50	6,668	Burkina Faso	Maximum Estimate	3211 - Medical imaging and therapeutic equipment technicians	Low Income	West	Other Health Workers
55	Radiologist	973	1,120	1,323	0.64	15,577	Burkina Faso	Maximum Estimate	2212 - Specialist medical practitioners	Low Income	West	Medical Doctors
56	Registered General Nurse / State Certified Nurse	73,170	81,605	91,243	43.39	230	Burkina Faso	Maximum Estimate	2221 - Nursing professionals	Low Income	West	Nursing Personnel
57	Renal Nurse	4,870	5,508	6,403	3.06	3,268	Burkina Faso	Maximum Estimate	2221 - Nursing professionals	Low Income	West	Nursing Personnel
58	Respiratory Physician	245	277	313	0.15	67,304	Burkina Faso	Maximum Estimate	2212 - Specialist medical practitioners	Low Income	West	Medical Doctors
59	Rheumatologist	76	85	94	0.04	223,504	Burkina Faso	Maximum Estimate	2212 - Specialist medical practitioners	Low Income	West	Medical Doctors
60	Speech Therapist	263	291	322	0.15	65,358	Burkina Faso	Maximum Estimate	2266 - Audiologists and speech therapists	Low Income	West	Other Health Workers
61	Urologist	39	47	59	0.03	349,878	Burkina Faso	Maximum Estimate	2212 - Specialist medical practitioners	Low Income	West	Medical Doctors
1	Anaesthesiologist	161	181	203	0.17	58,907	Burundi	Maximum Estimate	2212 - Specialist medical practitioners	Low Income	Central	Medical Doctors
2	Associate Nurse/Enrolled Nurse/Nursing Assistant	20,206	22,377	24,406	20.40	490	Burundi	Maximum Estimate	3221 - Nursing associate professionals	Low Income	Central	Nursing Personnel
3	Audiologist	81	90	100	0.08	119,416	Burundi	Maximum Estimate	2266 - Audiologists and speech therapists	Low Income	Central	Other Health Workers
4	Cardiologist	131	147	165	0.14	72,691	Burundi	Maximum Estimate	2212 - Specialist medical practitioners	Low Income	Central	Medical Doctors
5	Cardiothoracic Surgeon	35	39	43	0.04	281,068	Burundi	Maximum Estimate	2212 - Specialist medical practitioners	Low Income	Central	Medical Doctors
6	Clinical Officer/Physician Assistant	5,404	6,297	6,870	5.72	1,747	Burundi	Maximum Estimate	3256 - Medical assistants	Low Income	Central	Other Health Workers
7	Clinical Pharmacist	438	489	552	0.47	21,314	Burundi	Maximum Estimate	2262 - Pharmacists	Low Income	Central	Pharmacist
8	Clinical Psychologist	971	1,079	1,198	1.00	9,979	Burundi	Maximum Estimate	2634 - Psychologists	Low Income	Central	Other Health Workers
9	Community health worker/Village health worker	35,088	41,041	44,842	37.30	268	Burundi	Maximum Estimate	3253 - Community health workers	Low Income	Central	Other Health Workers

S/N	Health Professionals	2022	2026	2030	Density per 10,000 population	Required Population ratio (1 professional is to xxx population)	Name of Country	Modelling Scenario	ISCO-08 Match	Income Group Classification	Sub-Regional Grouping	SDG 3c Occupation
10	Dental Surgery Assistant	1,650	1,844	2,068	1.73	5,785	Burundi	Maximum Estimate	3251 - Dental assistants and therapists	Low Income	Central	Other Health Workers
11	Dental Therapist	1,111	1,241	1,392	1.16	8,596	Burundi	Maximum Estimate	3251 - Dental assistants and therapists	Low Income	Central	Other Health Workers
12	Dentist	1,549	1,720	1,946	1.63	6,131	Burundi	Maximum Estimate	2261 - Dentists	Low Income	Central	Dentist
13	Dermatologist	54	60	67	0.06	179,846	Burundi	Maximum Estimate	2212 - Specialist medical practitioners	Low Income	Central	Medical Doctors
14	Endocrinologist	43	51	60	0.05	197,899	Burundi	Maximum Estimate	2212 - Specialist medical practitioners	Low Income	Central	Medical Doctors
15	ENT Surgeon	182	206	236	0.20	50,869	Burundi	Maximum Estimate	2212 - Specialist medical practitioners	Low Income	Central	Medical Doctors
16	Environmental Health Officer	886	983	1,085	0.90	11,055	Burundi	Maximum Estimate	2263 - Environmental and occupational health and hygiene professionals	Low Income	Central	Other Health Workers
17	Gastroenterologist	103	108	115	0.10	103,020	Burundi	Maximum Estimate	2212 - Specialist medical practitioners	Low Income	Central	Medical Doctors
18	General Medical Practitioner (Generalist Doctor)	7,320	8,283	9,106	7.61	1,314	Burundi	Maximum Estimate	2211 - Generalist medical practitioners	Low Income	Central	Medical Doctors
19	General Surgeon	126	146	171	0.14	69,538	Burundi	Maximum Estimate	2212 - Specialist medical practitioners	Low Income	Central	Medical Doctors
20	Haematologist	44	47	51	0.04	232,937	Burundi	Maximum Estimate	2212 - Specialist medical practitioners	Low Income	Central	Medical Doctors
21	Health Promoter/Health Educator	469	541	593	0.50	20,105	Burundi	Maximum Estimate	2269 - Health professionals not elsewhere classified	Low Income	Central	Other Health Workers
22	Infectious Diseases Specialist	16	17	19	0.02	642,230	Burundi	Maximum Estimate	2212 - Specialist medical practitioners	Low Income	Central	Medical Doctors
23	Intensive Care Nurse	556	621	691	0.58	17,335	Burundi	Maximum Estimate	2221 - Nursing professionals	Low Income	Central	Nursing Personnel
24	Medical Laboratory Scientist	2,645	3,077	3,442	2.90	3,450	Burundi	Maximum Estimate	3212 - Medical and pathology laboratory technicians	Low Income	Central	Other Health Workers
25	Medical Laboratory Technician	7,040	8,385	9,223	7.69	1,300	Burundi	Maximum Estimate	3212 - Medical and pathology laboratory technicians	Low Income	Central	Other Health Workers
26	Medical Social Worker	559	624	715	0.63	15,952	Burundi	Maximum Estimate	1344 - Social welfare managers	Low Income	Central	Other Health Workers
27	Mental Health Nurse	1,113	1,247	1,406	1.19	8,434	Burundi	Maximum Estimate	2221 - Nursing professionals	Low Income	Central	Nursing Personnel
28	Midwife	12,432	13,515	14,827	12.43	805	Burundi	Maximum Estimate	2222 - Midwifery professionals	Low Income	Central	Midwifery Personnel
29	Nephrologist	124	140	160	0.14	73,735	Burundi	Maximum Estimate	2212 - Specialist medical practitioners	Low Income	Central	Medical Doctors
30	Neuro-Surgeon	49	54	63	0.05	190,642	Burundi	Maximum Estimate	2212 - Specialist medical practitioners	Low Income	Central	Medical Doctors
31	Nurse Anaesthetist	344	386	431	0.36	27,777	Burundi	Maximum Estimate	2221 - Nursing professionals	Low Income	Central	Nursing Personnel
32	Nutritionist	2,621	2,852	3,079	2.57	3,893	Burundi	Maximum Estimate	2265 - Dieticians and nutritionists	Low Income	Central	Other Health Workers
33	Obstetrician & Gynaecologist	1,057	1,198	1,380	1.18	8,450	Burundi	Maximum Estimate	2212 - Specialist medical practitioners	Low Income	Central	Medical Doctors
34	Occupational Therapist	273	307	345	0.29	34,565	Burundi	Maximum Estimate	2269 - Health professionals not elsewhere classified	Low Income	Central	Other Health Workers
35	Oncology Nurse	114	128	145	0.12	82,159	Burundi	Maximum Estimate	2221 - Nursing professionals	Low Income	Central	Nursing Personnel
36	Operating Theatre Nurse	1,492	1,684	1,912	1.61	6,209	Burundi	Maximum Estimate	2221 - Nursing professionals	Low Income	Central	Nursing Personnel
37	Ophthalmic Nurse	112	126	142	0.12	84,236	Burundi	Maximum Estimate	2221 - Nursing professionals	Low Income	Central	Nursing Personnel
38	Ophthalmologist	59	67	76	0.06	156,681	Burundi	Maximum Estimate	2212 - Specialist medical practitioners	Low Income	Central	Medical Doctors
39	Optometrist	272	309	349	0.29	34,281	Burundi	Maximum Estimate	2267 - Optometrists and ophthalmic opticians	Low Income	Central	Other Health Workers
40	Orthopaedic Nurse	308	347	390	0.33	30,752	Burundi	Maximum Estimate	2221 - Nursing professionals	Low Income	Central	Nursing Personnel
41	Orthopaedic Surgeon	266	295	328	0.28	36,326	Burundi	Maximum Estimate	2212 - Specialist medical practitioners	Low Income	Central	Medical Doctors
42	Orthopaedic Technologist	494	551	610	0.51	19,646	Burundi	Maximum Estimate	3214 - Medical and dental prosthetic technicians	Low Income	Central	Other Health Workers
43	Paediatric Nurse	1,998	2,186	2,342	1.95	5,123	Burundi	Maximum Estimate	2221 - Nursing professionals	Low Income	Central	Nursing Personnel
44	Paediatric Surgeon	76	85	92	0.08	130,660	Burundi	Maximum Estimate	2212 - Specialist medical practitioners	Low Income	Central	Medical Doctors
45	Paediatrician	774	837	901	0.75	13,261	Burundi	Maximum Estimate	2212 - Specialist medical practitioners	Low Income	Central	Medical Doctors
46	Pathologist	135	151	171	0.14	69,278	Burundi	Maximum Estimate	2212 - Specialist medical practitioners	Low Income	Central	Medical Doctors
47	Pharmacist	1,317	1,411	1,514	1.28	7,836	Burundi	Maximum Estimate	2262 - Pharmacists	Low Income	Central	Pharmacist
48	Pharmacy Technician	1,638	1,812	2,006	1.68	5,939	Burundi	Maximum Estimate	3213 - Pharmaceutical technicians and assistants	Low Income	Central	Other Health Workers
49	Physician	1,770	1,989	2,195	1.84	5,448	Burundi	Maximum Estimate	2212 - Specialist medical practitioners	Low Income	Central	Medical Doctors
50	Physiotherapist	428	477	532	0.44	22,496	Burundi	Maximum Estimate	2264 - Physiotherapists	Low Income	Central	Other Health Workers
51	Plastic Surgeon	187	213	241	0.20	49,715	Burundi	Maximum Estimate	2212 - Specialist medical practitioners	Low Income	Central	Medical Doctors
52	Psychiatrist	880	978	1,091	0.92	10,889	Burundi	Maximum Estimate	2212 - Specialist medical practitioners	Low Income	Central	Medical Doctors
53	Radiation Oncologist	42	47	53	0.04	226,344	Burundi	Maximum Estimate	2212 - Specialist medical practitioners	Low Income	Central	Medical Doctors
54	Radiographer (Diagnostics and Therapy)	983	1,120	1,300	1.12	8,954	Burundi	Maximum Estimate	3211 - Medical imaging and therapeutic equipment technicians	Low Income	Central	Other Health Workers
55	Radiologist	362	431	543	0.49	20,254	Burundi	Maximum Estimate	2212 - Specialist medical practitioners	Low Income	Central	Medical Doctors
56	Registered General Nurse / State Certified Nurse	47,461	50,372	53,390	44.86	223	Burundi	Maximum Estimate	2221 - Nursing professionals	Low Income	Central	Nursing Personnel
57	Renal Nurse	2,420	2,753	3,149	2.66	3,757	Burundi	Maximum Estimate	2221 - Nursing professionals	Low Income	Central	Nursing Personnel
58	Respiratory Physician	180	192	204	0.17	58,681	Burundi	Maximum Estimate	2212 - Specialist medical practitioners	Low Income	Central	Medical Doctors
59	Rheumatologist	42	48	53	0.04	224,754	Burundi	Maximum Estimate	2212 - Specialist medical practitioners	Low Income	Central	Medical Doctors
60	Speech Therapist	156	174	195	0.16	61,614	Burundi	Maximum Estimate	2266 - Audiologists and speech therapists	Low Income	Central	Other Health Workers
61	Urologist	19	23	28	0.02	417,092	Burundi	Maximum Estimate	2212 - Specialist medical practitioners	Low Income	Central	Medical Doctors
1	Anaesthesiologist	13	14	16	0.29	34,282	Cabo Verde	Maximum Estimate	2212 - Specialist medical practitioners	Lower-middle Income	West	Medical Doctors
2	Associate Nurse/Enrolled Nurse/Nursing Assistant	1,034	1,100	1,181	21.14	473	Cabo Verde	Maximum Estimate	3221 - Nursing associate professionals	Lower-middle Income	West	Nursing Personnel
3	Audiologist	4	4	5	0.08	119,321	Cabo Verde	Maximum Estimate	2266 - Audiologists and speech therapists	Lower-middle Income	West	Other Health Workers
4	Cardiologist	16	17	18	0.32	30,775	Cabo Verde	Maximum Estimate	2212 - Specialist medical practitioners	Lower-middle Income	West	Medical Doctors
5	Cardiothoracic Surgeon	3	3	4	0.07	147,941	Cabo Verde	Maximum Estimate	2212 - Specialist medical practitioners	Lower-middle Income	West	Medical Doctors
6	Clinical Officer/Physician Assistant	94	103	117	2.11	4,732	Cabo Verde	Maximum Estimate	3256 - Medical assistants	Lower-middle Income	West	Other Health Workers
7	Clinical Pharmacist	35	38	42	0.74	13,464	Cabo Verde	Maximum Estimate	2262 - Pharmacists	Lower-middle Income	West	Pharmacist
8	Clinical Psychologist	57	60	64	1.14	8,785	Cabo Verde	Maximum Estimate	2634 - Psychologists	Lower-middle Income	West	Other Health Workers
9	Community health worker/Village health worker	486	509	543	9.91	1,009	Cabo Verde	Maximum Estimate	3253 - Community health workers	Lower-middle Income	West	Other Health Workers
10	Dental Surgery Assistant	126	136	147	2.62	3,812	Cabo Verde	Maximum Estimate	3251 - Dental assistants and therapists	Lower-middle Income	West	Other Health Workers
11	Dental Therapist	84	91	98	1.75	5,730	Cabo Verde	Maximum Estimate	3251 - Dental assistants and therapists	Lower-middle Income	West	Other Health Workers
12	Dentist	89	93	97	1.73	5,795	Cabo Verde	Maximum Estimate	2261 - Dentists	Lower-middle Income	West	Dentist
13	Dermatologist	3	4	4	0.07	148,919	Cabo Verde	Maximum Estimate	2212 - Specialist medical practitioners	Lower-middle Income	West	Medical Doctors
14	Endocrinologist	8	9	11	0.19	51,949	Cabo Verde	Maximum Estimate	2212 - Specialist medical practitioners	Lower-middle Income	West	Medical Doctors
15	ENT Surgeon	15	17	19	0.34	29,231	Cabo Verde	Maximum Estimate	2212 - Specialist medical practitioners	Lower-middle Income	West	Medical Doctors
16	Environmental Health Officer	41	43	45	0.80	12,530	Cabo Verde	Maximum Estimate	2263 - Environmental and occupational health and hygiene professionals	Lower-middle Income	West	Other Health Workers
17	Gastroenterologist	13	13	13	0.23	42,652	Cabo Verde	Maximum Estimate	2212 - Specialist medical practitioners	Lower-middle Income	West	Medical Doctors
18	General Medical Practitioner (Generalist Doctor)	349	372	400	7.18	1,393	Cabo Verde	Maximum Estimate	2211 - Generalist medical practitioners	Lower-middle Income	West	Medical Doctors
19	General Surgeon	15	17	20	0.36	27,901	Cabo Verde	Maximum Estimate	2212 - Specialist medical practitioners	Lower-middle Income	West	Medical Doctors
20	Haematologist	3	3	3	0.06	169,492	Cabo Verde	Maximum Estimate	2212 - Specialist medical practitioners	Lower-middle Income	West	Medical Doctors
21	Health Promoter/Health Educator	9	9	10	0.19	53,942	Cabo Verde	Maximum Estimate	2269 - Health professionals not elsewhere classified	Lower-middle Income	West	Other Health Workers
22	Infectious Diseases Specialist	2	2	2	0.03	299,323	Cabo Verde	Maximum Estimate	2212 - Specialist medical practitioners	Lower-middle Income	West	Medical Doctors
23	Intensive Care Nurse	27	29	31	0.55	18,241	Cabo Verde	Maximum Estimate	2221 - Nursing professionals	Lower-middle Income	West	Nursing Personnel
24	Medical Laboratory Scientist	125	135	150	2.69	3,721	Cabo Verde	Maximum Estimate	3212 - Medical and pathology laboratory technicians	Lower-middle Income	West	Other Health Workers
25	Medical Laboratory Technician	108	119	136	2.48	4,033	Cabo Verde	Maximum Estimate	3212 - Medical and pathology laboratory technicians	Lower-middle Income	West	Other Health Workers
26	Medical Social Worker	28	28	29	0.52	19,309	Cabo Verde	Maximum Estimate	1344 - Social welfare managers	Lower-middle Income	West	Other Health Workers
27	Mental Health Nurse	76	80	86	1.53	6,523	Cabo Verde	Maximum Estimate	2221 - Nursing professionals	Lower-middle Income	West	Nursing Personnel
28	Midwife	627	651	671	11.99	834	Cabo Verde	Maximum Estimate	2222 - Midwifery professionals	Lower-middle Income	West	Midwifery Personnel
29	Nephrologist	17	19	22	0.39	25,622	Cabo Verde	Maximum Estimate	2212 - Specialist medical practitioners	Lower-middle Income	West	Medical Doctors
30	Neuro-Surgeon	5	6	7	0.12	80,808	Cabo Verde	Maximum Estimate	2212 - Specialist medical practitioners	Lower-middle Income	West	Medical Doctors
31	Nurse Anaesthetist	47	58	72	1.30	7,667	Cabo Verde	Maximum Estimate	2221 - Nursing professionals	Lower-middle Income	West	Nursing Personnel
32	Nutritionist	69	70	71	1.28	7,819	Cabo Verde	Maximum Estimate	2265 - Dieticians and nutritionists	Lower-middle Income	West	Other Health Workers

S/N	Health Professionals	2022	2026	2030	Density per 10,000 population	Required Population ratio (1 professional is to xxx population)	Name of Country	Modelling Scenario	ISCO-08 Match	Income Group Classification	Sub-Regional Grouping	SDG 3c Occupation
33	Obstetrician & Gynaecologist	61	65	69	1.23	8,099	Cabo Verde	Maximum Estimate	2212 - Specialist medical practitioners	Lower-middle Income	West	Medical Doctors
34	Occupational Therapist	25	28	32	0.57	17,601	Cabo Verde	Maximum Estimate	2269 - Health professionals not elsewhere classified	Lower-middle Income	West	Other Health Workers
35	Oncology Nurse	17	20	25	0.45	22,459	Cabo Verde	Maximum Estimate	2221 - Nursing professionals	Lower-middle Income	West	Nursing Personnel
36	Operating Theatre Nurse	154	169	192	3.45	2,897	Cabo Verde	Maximum Estimate	2221 - Nursing professionals	Lower-middle Income	West	Nursing Personnel
37	Ophthalmic Nurse	14	15	17	0.30	33,604	Cabo Verde	Maximum Estimate	2221 - Nursing professionals	Lower-middle Income	West	Nursing Personnel
38	Ophthalmologist	6	7	8	0.14	69,636	Cabo Verde	Maximum Estimate	2212 - Specialist medical practitioners	Lower-middle Income	West	Medical Doctors
39	Optometrist	26	29	32	0.57	17,424	Cabo Verde	Maximum Estimate	2267 - Optometrists and ophthalmic opticians	Lower-middle Income	West	Other Health Workers
40	Orthopaedic Nurse	22	25	29	0.51	19,478	Cabo Verde	Maximum Estimate	2221 - Nursing professionals	Lower-middle Income	West	Nursing Personnel
41	Orthopaedic Surgeon	48	54	61	1.09	9,194	Cabo Verde	Maximum Estimate	2212 - Specialist medical practitioners	Lower-middle Income	West	Medical Doctors
42	Orthopaedic Technologist	27	29	31	0.56	17,843	Cabo Verde	Maximum Estimate	3214 - Medical and dental prosthetic technicians	Lower-middle Income	West	Other Health Workers
43	Paediatric Nurse	70	72	72	1.29	7,771	Cabo Verde	Maximum Estimate	2221 - Nursing professionals	Lower-middle Income	West	Nursing Personnel
44	Paediatric Surgeon	2	2	2	0.04	282,946	Cabo Verde	Maximum Estimate	2212 - Specialist medical practitioners	Lower-middle Income	West	Medical Doctors
45	Paediatrician	38	40	43	0.78	12,887	Cabo Verde	Maximum Estimate	2212 - Specialist medical practitioners	Lower-middle Income	West	Medical Doctors
46	Pathologist	11	12	14	0.25	40,028	Cabo Verde	Maximum Estimate	2212 - Specialist medical practitioners	Lower-middle Income	West	Medical Doctors
47	Pharmacist	58	59	63	1.13	8,875	Cabo Verde	Maximum Estimate	2262 - Pharmacists	Lower-middle Income	West	Pharmacist
48	Pharmacy Technician	112	119	128	2.29	4,371	Cabo Verde	Maximum Estimate	3213 - Pharmaceutical technicians and assistants	Lower-middle Income	West	Other Health Workers
49	Physician	123	133	146	2.61	3,836	Cabo Verde	Maximum Estimate	2212 - Specialist medical practitioners	Lower-middle Income	West	Medical Doctors
50	Physiotherapist	27	29	32	0.57	17,485	Cabo Verde	Maximum Estimate	2264 - Physiotherapists	Lower-middle Income	West	Other Health Workers
51	Plastic Surgeon	9	10	11	0.19	52,466	Cabo Verde	Maximum Estimate	2212 - Specialist medical practitioners	Lower-middle Income	West	Medical Doctors
52	Psychiatrist	57	60	65	1.15	8,667	Cabo Verde	Maximum Estimate	2212 - Specialist medical practitioners	Lower-middle Income	West	Medical Doctors
53	Radiation Oncologist	8	10	14	0.25	39,908	Cabo Verde	Maximum Estimate	2212 - Specialist medical practitioners	Lower-middle Income	West	Medical Doctors
54	Radiographer (Diagnostics and Therapy)	109	119	131	2.35	4,263	Cabo Verde	Maximum Estimate	3211 - Medical imaging and therapeutic equipment technicians	Lower-middle Income	West	Other Health Workers
55	Radiologist	39	43	48	0.86	11,691	Cabo Verde	Maximum Estimate	2212 - Specialist medical practitioners	Lower-middle Income	West	Medical Doctors
56	Registered General Nurse / State Certified Nurse	2,479	2,709	3,004	53.71	186	Cabo Verde	Maximum Estimate	2221 - Nursing professionals	Lower-middle Income	West	Nursing Personnel
57	Renal Nurse	342	382	439	7.81	1,280	Cabo Verde	Maximum Estimate	2221 - Nursing professionals	Lower-middle Income	West	Nursing Personnel
58	Respiratory Physician	7	7	8	0.14	72,208	Cabo Verde	Maximum Estimate	2212 - Specialist medical practitioners	Lower-middle Income	West	Medical Doctors
59	Rheumatologist	3	3	4	0.06	154,968	Cabo Verde	Maximum Estimate	2212 - Specialist medical practitioners	Lower-middle Income	West	Medical Doctors
60	Speech Therapist	9	9	10	0.18	55,460	Cabo Verde	Maximum Estimate	2266 - Audiologists and speech therapists	Lower-middle Income	West	Other Health Workers
61	Urologist	3	4	5	0.09	113,966	Cabo Verde	Maximum Estimate	2212 - Specialist medical practitioners	Lower-middle Income	West	Medical Doctors
1	Anaesthesiologist	563	636	717	0.27	37,335	Cameroon	Maximum Estimate	2212 - Specialist medical practitioners	Lower-middle Income	Central	Medical Doctors
2	Associate Nurse/Enrolled Nurse/Nursing Assistant	45,452	50,796	56,838	21.27	470	Cameroon	Maximum Estimate	3221 - Nursing associate professionals	Lower-middle Income	Central	Nursing Personnel
3	Audiologist	176	197	219	0.08	122,468	Cameroon	Maximum Estimate	2266 - Audiologists and speech therapists	Lower-middle Income	Central	Other Health Workers
4	Cardiologist	344	391	441	0.16	60,682	Cameroon	Maximum Estimate	2212 - Specialist medical practitioners	Lower-middle Income	Central	Medical Doctors
5	Cardiothoracic Surgeon	92	104	117	0.04	229,295	Cameroon	Maximum Estimate	2212 - Specialist medical practitioners	Lower-middle Income	Central	Medical Doctors
6	Clinical Officer/Physician Assistant	6,368	7,089	7,970	2.99	3,341	Cameroon	Maximum Estimate	3256 - Medical assistants	Lower-middle Income	Central	Other Health Workers
7	Clinical Pharmacist	1,254	1,425	1,647	0.62	16,059	Cameroon	Maximum Estimate	2262 - Pharmacists	Lower-middle Income	Central	Pharmacist
8	Clinical Psychologist	2,501	2,807	3,163	1.18	8,452	Cameroon	Maximum Estimate	2634 - Psychologists	Lower-middle Income	Central	Other Health Workers
9	Community health worker/Village health worker	42,555	47,267	52,394	19.60	510	Cameroon	Maximum Estimate	3253 - Community health workers	Lower-middle Income	Central	Other Health Workers
10	Dental Surgery Assistant	4,591	5,201	5,905	2.21	4,532	Cameroon	Maximum Estimate	3251 - Dental assistants and therapists	Lower-middle Income	Central	Other Health Workers
11	Dental Therapist	3,073	3,480	3,950	1.48	6,775	Cameroon	Maximum Estimate	3251 - Dental assistants and therapists	Lower-middle Income	Central	Other Health Workers
12	Dentist	3,691	4,149	4,733	1.77	5,649	Cameroon	Maximum Estimate	2261 - Dentists	Lower-middle Income	Central	Dentist
13	Dermatologist	139	157	176	0.07	152,336	Cameroon	Maximum Estimate	2212 - Specialist medical practitioners	Lower-middle Income	Central	Medical Doctors
14	Endocrinologist	129	153	186	0.07	142,328	Cameroon	Maximum Estimate	2212 - Specialist medical practitioners	Lower-middle Income	Central	Medical Doctors
15	ENT Surgeon	482	546	616	0.23	43,417	Cameroon	Maximum Estimate	2212 - Specialist medical practitioners	Lower-middle Income	Central	Medical Doctors
16	Environmental Health Officer	1,978	2,189	2,410	0.90	11,119	Cameroon	Maximum Estimate	2263 - Environmental and occupational health and hygiene professionals	Lower-middle Income	Central	Other Health Workers
17	Gastroenterologist	340	353	376	0.14	70,613	Cameroon	Maximum Estimate	2212 - Specialist medical practitioners	Lower-middle Income	Central	Medical Doctors
18	General Medical Practitioner (Generalist Doctor)	15,883	17,738	19,853	7.44	1,344	Cameroon	Maximum Estimate	2211 - Generalist medical practitioners	Lower-middle Income	Central	Medical Doctors
19	General Surgeon	404	465	543	0.20	49,093	Cameroon	Maximum Estimate	2212 - Specialist medical practitioners	Lower-middle Income	Central	Medical Doctors
20	Haematologist	115	126	136	0.05	196,234	Cameroon	Maximum Estimate	2212 - Specialist medical practitioners	Lower-middle Income	Central	Medical Doctors
21	Health Promoter/Health Educator	613	686	784	0.30	33,570	Cameroon	Maximum Estimate	2269 - Health professionals not elsewhere classified	Lower-middle Income	Central	Other Health Workers
22	Infectious Diseases Specialist	51	55	59	0.02	449,990	Cameroon	Maximum Estimate	2212 - Specialist medical practitioners	Lower-middle Income	Central	Medical Doctors
23	Intensive Care Nurse	1,400	1,527	1,658	0.62	16,143	Cameroon	Maximum Estimate	2221 - Nursing professionals	Lower-middle Income	Central	Nursing Personnel
24	Medical Laboratory Scientist	5,158	5,826	6,705	2.54	3,938	Cameroon	Maximum Estimate	3212 - Medical and pathology laboratory technicians	Lower-middle Income	Central	Other Health Workers
25	Medical Laboratory Technician	8,898	10,083	11,491	4.32	2,315	Cameroon	Maximum Estimate	3212 - Medical and pathology laboratory technicians	Lower-middle Income	Central	Other Health Workers
26	Medical Social Worker	1,188	1,338	1,582	0.62	16,147	Cameroon	Maximum Estimate	1344 - Social welfare managers	Lower-middle Income	Central	Other Health Workers
27	Mental Health Nurse	2,579	2,908	3,360	1.26	7,918	Cameroon	Maximum Estimate	2221 - Nursing professionals	Lower-middle Income	Central	Nursing Personnel
28	Midwife	29,306	31,971	34,982	13.07	765	Cameroon	Maximum Estimate	2222 - Midwifery professionals	Lower-middle Income	Central	Midwifery Personnel
29	Nephrologist	467	543	649	0.24	40,872	Cameroon	Maximum Estimate	2212 - Specialist medical practitioners	Lower-middle Income	Central	Medical Doctors
30	Neuro-Surgeon	194	217	241	0.09	110,762	Cameroon	Maximum Estimate	2212 - Specialist medical practitioners	Lower-middle Income	Central	Medical Doctors
31	Nurse Anaesthetist	1,070	1,252	1,463	0.55	18,251	Cameroon	Maximum Estimate	2221 - Nursing professionals	Lower-middle Income	Central	Nursing Personnel
32	Nutritionist	3,947	4,114	4,262	1.60	6,269	Cameroon	Maximum Estimate	2265 - Dieticians and nutritionists	Lower-middle Income	Central	Other Health Workers
33	Obstetrician & Gynaecologist	2,660	3,081	3,641	1.39	7,194	Cameroon	Maximum Estimate	2212 - Specialist medical practitioners	Lower-middle Income	Central	Medical Doctors
34	Occupational Therapist	1,104	1,250	1,419	0.53	18,827	Cameroon	Maximum Estimate	2269 - Health professionals not elsewhere classified	Lower-middle Income	Central	Other Health Workers
35	Oncology Nurse	364	426	507	0.19	52,440	Cameroon	Maximum Estimate	2221 - Nursing professionals	Lower-middle Income	Central	Nursing Personnel
36	Operating Theatre Nurse	4,595	5,217	6,027	2.26	4,416	Cameroon	Maximum Estimate	2221 - Nursing professionals	Lower-middle Income	Central	Nursing Personnel
37	Ophthalmic Nurse	367	423	491	0.18	54,364	Cameroon	Maximum Estimate	2221 - Nursing professionals	Lower-middle Income	Central	Nursing Personnel
38	Ophthalmologist	148	169	194	0.07	137,189	Cameroon	Maximum Estimate	2212 - Specialist medical practitioners	Lower-middle Income	Central	Medical Doctors
39	Optometrist	587	665	754	0.28	35,459	Cameroon	Maximum Estimate	2267 - Optometrists and ophthalmic opticians	Lower-middle Income	Central	Other Health Workers
40	Orthopaedic Nurse	1,346	1,537	1,747	0.65	15,325	Cameroon	Maximum Estimate	2221 - Nursing professionals	Lower-middle Income	Central	Nursing Personnel
41	Orthopaedic Surgeon	1,604	1,858	2,157	0.81	12,377	Cameroon	Maximum Estimate	2212 - Specialist medical practitioners	Lower-middle Income	Central	Medical Doctors
42	Orthopaedic Technologist	1,309	1,470	1,643	0.61	16,305	Cameroon	Maximum Estimate	3214 - Medical and dental prosthetic technicians	Lower-middle Income	Central	Other Health Workers
43	Paediatric Nurse	3,885	4,220	4,519	1.69	5,927	Cameroon	Maximum Estimate	2221 - Nursing professionals	Lower-middle Income	Central	Nursing Personnel
44	Paediatric Surgeon	145	157	167	0.06	160,159	Cameroon	Maximum Estimate	2212 - Specialist medical practitioners	Lower-middle Income	Central	Medical Doctors
45	Paediatrician	1,649	1,864	2,116	0.79	12,619	Cameroon	Maximum Estimate	2212 - Specialist medical practitioners	Lower-middle Income	Central	Medical Doctors
46	Pathologist	377	430	502	0.19	52,966	Cameroon	Maximum Estimate	2212 - Specialist medical practitioners	Lower-middle Income	Central	Medical Doctors
47	Pharmacist	2,367	2,486	2,665	1.01	9,861	Cameroon	Maximum Estimate	2262 - Pharmacists	Lower-middle Income	Central	Pharmacist
48	Pharmacy Technician	4,682	5,239	5,885	2.21	4,529	Cameroon	Maximum Estimate	3213 - Pharmaceutical technicians and assistants	Lower-middle Income	Central	Other Health Workers
49	Physician	4,626	5,219	5,908	2.21	4,522	Cameroon	Maximum Estimate	2212 - Specialist medical practitioners	Lower-middle Income	Central	Medical Doctors
50	Physiotherapist	1,108	1,242	1,391	0.52	19,220	Cameroon	Maximum Estimate	2264 - Physiotherapists	Lower-middle Income	Central	Other Health Workers
51	Plastic Surgeon	301	344	390	0.15	68,635	Cameroon	Maximum Estimate	2212 - Specialist medical practitioners	Lower-middle Income	Central	Medical Doctors
52	Psychiatrist	2,089	2,334	2,663	1.00	10,002	Cameroon	Maximum Estimate	2212 - Specialist medical practitioners	Lower-middle Income	Central	Medical Doctors
53	Radiation Oncologist	122	145	173	0.06	153,849	Cameroon	Maximum Estimate	2212 - Specialist medical practitioners	Lower-middle Income	Central	Medical Doctors
54	Radiographer (Diagnostics and Therapy)	3,496	4,063	4,825	1.83	5,458	Cameroon	Maximum Estimate	3211 - Medical imaging and therapeutic equipment technicians	Lower-middle Income	Central	Other Health Workers
55	Radiologist	1,384	1,692	2,150	0.84	11,892	Cameroon	Maximum Estimate	2212 - Specialist medical practitioners	Lower-middle Income	Central	Medical Doctors

S/N	Health Professionals	2022	2026	2030	Density per 10,000 population	Required Population ratio (1 professional is to xxx population)	Name of Country	Modelling Scenario	ISCO-08 Match	Income Group Classification	Sub-Regional Grouping	SDG 3c Occupation
56	Registered General Nurse / State Certified Nurse	93,520	103,250	115,096	43.25	231	Cameroon	Maximum Estimate	2221 - Nursing professionals	Lower-middle Income	Central	Nursing Personnel
57	Renal Nurse	8,925	10,384	12,406	4.68	2,138	Cameroon	Maximum Estimate	2221 - Nursing professionals	Lower-middle Income	Central	Nursing Personnel
58	Respiratory Physician	294	337	386	0.14	69,282	Cameroon	Maximum Estimate	2212 - Specialist medical practitioners	Lower-middle Income	Central	Medical Doctors
59	Rheumatologist	103	117	131	0.05	204,486	Cameroon	Maximum Estimate	2212 - Specialist medical practitioners	Lower-middle Income	Central	Medical Doctors
60	Speech Therapist	341	378	417	0.16	64,174	Cameroon	Maximum Estimate	2266 - Audiologists and speech therapists	Lower-middle Income	Central	Other Health Workers
61	Urologist	93	128	184	0.07	136,718	Cameroon	Maximum Estimate	2212 - Specialist medical practitioners	Lower-middle Income	Central	Medical Doctors
1	Anaesthesiologist	68	77	88	0.18	55,029	Central African Rep	Maximum Estimate	2212 - Specialist medical practitioners	Low Income	Central	Medical Doctors
2	Associate Nurse/Enrolled Nurse/Nursing Assistant	9,314	10,415	11,799	24.27	412	Central African Rep	Maximum Estimate	3221 - Nursing associate professionals	Low Income	Central	Nursing Personnel
3	Audiologist	34	39	45	0.09	108,130	Central African Rep	Maximum Estimate	2266 - Audiologists and speech therapists	Low Income	Central	Other Health Workers
4	Cardiologist	57	65	76	0.16	64,166	Central African Rep	Maximum Estimate	2212 - Specialist medical practitioners	Low Income	Central	Medical Doctors
5	Cardiothoracic Surgeon	13	15	17	0.03	290,546	Central African Rep	Maximum Estimate	2212 - Specialist medical practitioners	Low Income	Central	Medical Doctors
6	Clinical Officer/Physician Assistant	2,008	2,056	2,163	4.49	2,228	Central African Rep	Maximum Estimate	3256 - Medical assistants	Low Income	Central	Other Health Workers
7	Clinical Pharmacist	231	260	302	0.62	16,027	Central African Rep	Maximum Estimate	2262 - Pharmacists	Low Income	Central	Pharmacist
8	Clinical Psychologist	460	518	597	1.23	8,122	Central African Rep	Maximum Estimate	2634 - Psychologists	Low Income	Central	Other Health Workers
9	Community health worker/Village health worker	9,443	9,993	10,713	22.08	453	Central African Rep	Maximum Estimate	3253 - Community health workers	Low Income	Central	Other Health Workers
10	Dental Surgery Assistant	685	777	903	1.86	5,385	Central African Rep	Maximum Estimate	3251 - Dental assistants and therapists	Low Income	Central	Other Health Workers
11	Dental Therapist	460	522	607	1.25	8,014	Central African Rep	Maximum Estimate	3251 - Dental assistants and therapists	Low Income	Central	Other Health Workers
12	Dentist	625	710	846	1.74	5,740	Central African Rep	Maximum Estimate	2261 - Dentists	Low Income	Central	Dentist
13	Dermatologist	26	30	34	0.07	143,561	Central African Rep	Maximum Estimate	2212 - Specialist medical practitioners	Low Income	Central	Medical Doctors
14	Endocrinologist	32	38	48	0.10	98,832	Central African Rep	Maximum Estimate	2212 - Specialist medical practitioners	Low Income	Central	Medical Doctors
15	ENT Surgeon	79	91	104	0.21	46,572	Central African Rep	Maximum Estimate	2212 - Specialist medical practitioners	Low Income	Central	Medical Doctors
16	Environmental Health Officer	360	404	456	0.94	10,677	Central African Rep	Maximum Estimate	2263 - Environmental and occupational health and hygiene professionals	Low Income	Central	Other Health Workers
17	Gastroenterologist	108	117	135	0.28	35,683	Central African Rep	Maximum Estimate	2212 - Specialist medical practitioners	Low Income	Central	Medical Doctors
18	General Medical Practitioner (Generalist Doctor)	3,265	3,597	4,027	8.29	1,206	Central African Rep	Maximum Estimate	2211 - Generalist medical practitioners	Low Income	Central	Medical Doctors
19	General Surgeon	74	83	98	0.20	49,545	Central African Rep	Maximum Estimate	2212 - Specialist medical practitioners	Low Income	Central	Medical Doctors
20	Haematologist	28	32	37	0.08	131,613	Central African Rep	Maximum Estimate	2212 - Specialist medical practitioners	Low Income	Central	Medical Doctors
21	Health Promoter/Health Educator	179	188	202	0.42	23,934	Central African Rep	Maximum Estimate	2269 - Health professionals not elsewhere classified	Low Income	Central	Other Health Workers
22	Infectious Diseases Specialist	13	15	16	0.03	301,074	Central African Rep	Maximum Estimate	2212 - Specialist medical practitioners	Low Income	Central	Medical Doctors
23	Intensive Care Nurse	300	335	375	0.77	12,998	Central African Rep	Maximum Estimate	2221 - Nursing professionals	Low Income	Central	Nursing Personnel
24	Medical Laboratory Scientist	1,098	1,210	1,377	2.85	3,511	Central African Rep	Maximum Estimate	3212 - Medical and pathology laboratory technicians	Low Income	Central	Other Health Workers
25	Medical Laboratory Technician	1,808	1,866	1,957	4.05	2,472	Central African Rep	Maximum Estimate	3212 - Medical and pathology laboratory technicians	Low Income	Central	Other Health Workers
26	Medical Social Worker	291	317	358	0.74	13,549	Central African Rep	Maximum Estimate	1344 - Social welfare managers	Low Income	Central	Other Health Workers
27	Mental Health Nurse	555	633	763	1.58	6,323	Central African Rep	Maximum Estimate	2221 - Nursing professionals	Low Income	Central	Nursing Personnel
28	Midwife	6,097	6,761	7,673	15.76	634	Central African Rep	Maximum Estimate	2222 - Midwifery professionals	Low Income	Central	Midwifery Personnel
29	Nephrologist	54	62	77	0.16	62,385	Central African Rep	Maximum Estimate	2212 - Specialist medical practitioners	Low Income	Central	Medical Doctors
30	Neuro-Surgeon	24	27	30	0.06	163,744	Central African Rep	Maximum Estimate	2212 - Specialist medical practitioners	Low Income	Central	Medical Doctors
31	Nurse Anaesthetist	131	149	171	0.35	28,426	Central African Rep	Maximum Estimate	2221 - Nursing professionals	Low Income	Central	Nursing Personnel
32	Nutritionist	949	1,036	1,139	2.34	4,279	Central African Rep	Maximum Estimate	2265 - Dieticians and nutritionists	Low Income	Central	Other Health Workers
33	Obstetrician & Gynaecologist	469	531	611	1.26	7,948	Central African Rep	Maximum Estimate	2212 - Specialist medical practitioners	Low Income	Central	Medical Doctors
34	Occupational Therapist	111	127	148	0.31	32,755	Central African Rep	Maximum Estimate	2269 - Health professionals not elsewhere classified	Low Income	Central	Other Health Workers
35	Oncology Nurse	46	52	63	0.13	76,921	Central African Rep	Maximum Estimate	2221 - Nursing professionals	Low Income	Central	Nursing Personnel
36	Operating Theatre Nurse	682	781	935	1.94	5,163	Central African Rep	Maximum Estimate	2221 - Nursing professionals	Low Income	Central	Nursing Personnel
37	Ophthalmic Nurse	82	95	113	0.23	42,976	Central African Rep	Maximum Estimate	2221 - Nursing professionals	Low Income	Central	Nursing Personnel
38	Ophthalmologist	28	33	40	0.08	120,848	Central African Rep	Maximum Estimate	2212 - Specialist medical practitioners	Low Income	Central	Medical Doctors
39	Optometrist	118	139	166	0.34	29,186	Central African Rep	Maximum Estimate	2267 - Optometrists and ophthalmic opticians	Low Income	Central	Other Health Workers
40	Orthopaedic Nurse	101	115	132	0.27	36,952	Central African Rep	Maximum Estimate	2221 - Nursing professionals	Low Income	Central	Nursing Personnel
41	Orthopaedic Surgeon	176	193	218	0.45	22,240	Central African Rep	Maximum Estimate	2212 - Specialist medical practitioners	Low Income	Central	Medical Doctors
42	Orthopaedic Technologist	187	211	238	0.49	20,425	Central African Rep	Maximum Estimate	3214 - Medical and dental prosthetic technicians	Low Income	Central	Other Health Workers
43	Paediatric Nurse	843	946	1,067	2.19	4,568	Central African Rep	Maximum Estimate	2221 - Nursing professionals	Low Income	Central	Nursing Personnel
44	Paediatric Surgeon	31	34	38	0.08	127,328	Central African Rep	Maximum Estimate	2212 - Specialist medical practitioners	Low Income	Central	Medical Doctors
45	Paediatrician	313	349	396	0.81	12,272	Central African Rep	Maximum Estimate	2212 - Specialist medical practitioners	Low Income	Central	Medical Doctors
46	Pathologist	72	82	98	0.20	49,146	Central African Rep	Maximum Estimate	2212 - Specialist medical practitioners	Low Income	Central	Medical Doctors
47	Pharmacist	1,039	1,111	1,208	2.49	4,011	Central African Rep	Maximum Estimate	2262 - Pharmacists	Low Income	Central	Pharmacist
48	Pharmacy Technician	913	1,017	1,149	2.37	4,228	Central African Rep	Maximum Estimate	3213 - Pharmaceutical technicians and assistants	Low Income	Central	Other Health Workers
49	Physician	826	924	1,056	2.18	4,594	Central African Rep	Maximum Estimate	2212 - Specialist medical practitioners	Low Income	Central	Medical Doctors
50	Physiotherapist	170	193	220	0.45	22,081	Central African Rep	Maximum Estimate	2264 - Physiotherapists	Low Income	Central	Other Health Workers
51	Plastic Surgeon	37	43	49	0.10	99,656	Central African Rep	Maximum Estimate	2212 - Specialist medical practitioners	Low Income	Central	Medical Doctors
52	Psychiatrist	407	459	541	1.12	8,943	Central African Rep	Maximum Estimate	2212 - Specialist medical practitioners	Low Income	Central	Medical Doctors
53	Radiation Oncologist	16	18	21	0.04	230,188	Central African Rep	Maximum Estimate	2212 - Specialist medical practitioners	Low Income	Central	Medical Doctors
54	Radiographer (Diagnostics and Therapy)	541	616	730	1.51	6,630	Central African Rep	Maximum Estimate	3211 - Medical imaging and therapeutic equipment technicians	Low Income	Central	Other Health Workers
55	Radiologist	213	241	280	0.58	17,303	Central African Rep	Maximum Estimate	2212 - Specialist medical practitioners	Low Income	Central	Medical Doctors
56	Registered General Nurse / State Certified Nurse	22,907	25,671	29,232	60.16	166	Central African Rep	Maximum Estimate	2221 - Nursing professionals	Low Income	Central	Nursing Personnel
57	Renal Nurse	1,057	1,228	1,519	3.16	3,163	Central African Rep	Maximum Estimate	2221 - Nursing professionals	Low Income	Central	Nursing Personnel
58	Respiratory Physician	60	66	73	0.15	66,320	Central African Rep	Maximum Estimate	2212 - Specialist medical practitioners	Low Income	Central	Medical Doctors
59	Rheumatologist	18	21	24	0.05	206,415	Central African Rep	Maximum Estimate	2212 - Specialist medical practitioners	Low Income	Central	Medical Doctors
60	Speech Therapist	61	69	78	0.16	62,394	Central African Rep	Maximum Estimate	2266 - Audiologists and speech therapists	Low Income	Central	Other Health Workers
61	Urologist	9	10	12	0.03	397,562	Central African Rep	Maximum Estimate	2212 - Specialist medical practitioners	Low Income	Central	Medical Doctors
1	Anaesthesiologist	217	245	275	0.17	60,382	Chad	Maximum Estimate	2212 - Specialist medical practitioners	Low Income	West	Medical Doctors
2	Associate Nurse/Enrolled Nurse/Nursing Assistant	28,180	31,415	34,985	21.13	473	Chad	Maximum Estimate	3221 - Nursing associate professionals	Low Income	West	Nursing Personnel
3	Audiologist	104	117	131	0.08	126,413	Chad	Maximum Estimate	2266 - Audiologists and speech therapists	Low Income	West	Other Health Workers
4	Cardiologist	158	178	198	0.12	83,701	Chad	Maximum Estimate	2212 - Specialist medical practitioners	Low Income	West	Medical Doctors
5	Cardiothoracic Surgeon	46	51	57	0.03	290,763	Chad	Maximum Estimate	2212 - Specialist medical practitioners	Low Income	West	Medical Doctors
6	Clinical Officer/Physician Assistant	3,717	3,831	3,981	2.42	4,130	Chad	Maximum Estimate	3256 - Medical assistants	Low Income	West	Other Health Workers
7	Clinical Pharmacist	728	812	915	0.55	18,044	Chad	Maximum Estimate	2262 - Pharmacists	Low Income	West	Pharmacist
8	Clinical Psychologist	1,352	1,512	1,709	1.03	9,663	Chad	Maximum Estimate	2634 - Psychologists	Low Income	West	Other Health Workers
9	Community health worker/Village health worker	29,975	32,702	35,518	21.45	466	Chad	Maximum Estimate	3253 - Community health workers	Low Income	West	Other Health Workers
10	Dental Surgery Assistant	2,300	2,599	2,944	1.78	5,626	Chad	Maximum Estimate	3251 - Dental assistants and therapists	Low Income	West	Other Health Workers
11	Dental Therapist	1,546	1,747	1,978	1.19	8,373	Chad	Maximum Estimate	3251 - Dental assistants and therapists	Low Income	West	Other Health Workers
12	Dentist	1,943	2,197	2,531	1.53	6,530	Chad	Maximum Estimate	2261 - Dentists	Low Income	West	Dentist
13	Dermatologist	78	88	99	0.06	167,769	Chad	Maximum Estimate	2212 - Specialist medical practitioners	Low Income	West	Medical Doctors
14	Endocrinologist	54	62	73	0.04	224,384	Chad	Maximum Estimate	2212 - Specialist medical practitioners	Low Income	West	Medical Doctors
15	ENT Surgeon	258	292	328	0.20	50,480	Chad	Maximum Estimate	2212 - Specialist medical practitioners	Low Income	West	Medical Doctors
16	Environmental Health Officer	1,224	1,380	1,548	0.93	10,714	Chad	Maximum Estimate	2263 - Environmental and occupational health and hygiene professionals	Low Income	West	Other Health Workers
17	Gastroenterologist	443	472	520	0.32	31,625	Chad	Maximum Estimate	2212 - Specialist medical practitioners	Low Income	West	Medical Doctors

S/N	Health Professionals	2022	2026	2030	Density per 10,000 population	Required Population ratio (1 professional is to xxx population)	Name of Country	Modelling Scenario	ISCO-08 Match	Income Group Classification	Sub-Regional Grouping	SDG 3c Occupation
18	General Medical Practitioner (Generalist Doctor)	9,308	10,334	11,469	6.93	1,443	Chad	Maximum Estimate	2211 - Generalist medical practitioners	Low Income	West	Medical Doctors
19	General Surgeon	192	217	248	0.15	66,513	Chad	Maximum Estimate	2212 - Specialist medical practitioners	Low Income	West	Medical Doctors
20	Haematologist	94	106	120	0.07	137,953	Chad	Maximum Estimate	2212 - Specialist medical practitioners	Low Income	West	Medical Doctors
21	Health Promoter/Health Educator	421	437	457	0.28	36,051	Chad	Maximum Estimate	2269 - Health professionals not elsewhere classified	Low Income	West	Other Health Workers
22	Infectious Diseases Specialist	41	45	49	0.03	337,913	Chad	Maximum Estimate	2212 - Specialist medical practitioners	Low Income	West	Medical Doctors
23	Intensive Care Nurse	1,013	1,131	1,255	0.76	13,211	Chad	Maximum Estimate	2221 - Nursing professionals	Low Income	West	Nursing Personnel
24	Medical Laboratory Scientist	2,752	3,041	3,396	2.06	4,861	Chad	Maximum Estimate	3212 - Medical and pathology laboratory technicians	Low Income	West	Other Health Workers
25	Medical Laboratory Technician	4,068	4,358	4,668	2.82	3,543	Chad	Maximum Estimate	3212 - Medical and pathology laboratory technicians	Low Income	West	Other Health Workers
26	Medical Social Worker	1,503	1,634	1,789	1.08	9,242	Chad	Maximum Estimate	1344 - Social welfare managers	Low Income	West	Other Health Workers
27	Mental Health Nurse	1,501	1,708	1,991	1.21	8,265	Chad	Maximum Estimate	2221 - Nursing professionals	Low Income	West	Nursing Personnel
28	Midwife	19,591	21,609	23,955	14.48	691	Chad	Maximum Estimate	2222 - Midwifery professionals	Low Income	West	Midwifery Personnel
29	Nephrologist	162	186	220	0.13	74,729	Chad	Maximum Estimate	2212 - Specialist medical practitioners	Low Income	West	Medical Doctors
30	Neuro-Surgeon	54	61	69	0.04	239,570	Chad	Maximum Estimate	2212 - Specialist medical practitioners	Low Income	West	Medical Doctors
31	Nurse Anaesthetist	370	420	475	0.29	34,880	Chad	Maximum Estimate	2221 - Nursing professionals	Low Income	West	Nursing Personnel
32	Nutritionist	6,193	6,827	7,476	4.51	2,219	Chad	Maximum Estimate	2265 - Dieticians and nutritionists	Low Income	West	Other Health Workers
33	Obstetrician & Gynaecologist	1,323	1,496	1,690	1.02	9,799	Chad	Maximum Estimate	2212 - Specialist medical practitioners	Low Income	West	Medical Doctors
34	Occupational Therapist	384	433	490	0.30	33,781	Chad	Maximum Estimate	2269 - Health professionals not elsewhere classified	Low Income	West	Other Health Workers
35	Oncology Nurse	124	141	164	0.10	100,653	Chad	Maximum Estimate	2221 - Nursing professionals	Low Income	West	Nursing Personnel
36	Operating Theatre Nurse	1,938	2,167	2,472	1.50	6,670	Chad	Maximum Estimate	2221 - Nursing professionals	Low Income	West	Nursing Personnel
37	Ophthalmic Nurse	206	231	260	0.16	63,735	Chad	Maximum Estimate	2221 - Nursing professionals	Low Income	West	Nursing Personnel
38	Ophthalmologist	73	82	92	0.06	179,163	Chad	Maximum Estimate	2212 - Specialist medical practitioners	Low Income	West	Medical Doctors
39	Optometrist	293	324	357	0.22	46,344	Chad	Maximum Estimate	2267 - Optometrists and ophthalmic opticians	Low Income	West	Other Health Workers
40	Orthopaedic Nurse	462	526	595	0.36	27,881	Chad	Maximum Estimate	2221 - Nursing professionals	Low Income	West	Nursing Personnel
41	Orthopaedic Surgeon	596	692	806	0.49	20,489	Chad	Maximum Estimate	2212 - Specialist medical practitioners	Low Income	West	Medical Doctors
42	Orthopaedic Technologist	683	772	868	0.52	19,113	Chad	Maximum Estimate	3214 - Medical and dental prosthetic technicians	Low Income	West	Other Health Workers
43	Paediatric Nurse	3,574	4,016	4,453	2.68	3,728	Chad	Maximum Estimate	2221 - Nursing professionals	Low Income	West	Nursing Personnel
44	Paediatric Surgeon	117	134	150	0.09	110,635	Chad	Maximum Estimate	2212 - Specialist medical practitioners	Low Income	West	Medical Doctors
45	Paediatrician	927	1,064	1,222	0.74	13,534	Chad	Maximum Estimate	2212 - Specialist medical practitioners	Low Income	West	Medical Doctors
46	Pathologist	267	289	317	0.19	51,995	Chad	Maximum Estimate	2212 - Specialist medical practitioners	Low Income	West	Medical Doctors
47	Pharmacist	1,681	1,726	1,781	1.08	9,234	Chad	Maximum Estimate	2262 - Pharmacists	Low Income	West	Pharmacist
48	Pharmacy Technician	2,649	2,940	3,270	1.98	5,059	Chad	Maximum Estimate	3213 - Pharmaceutical technicians and assistants	Low Income	West	Other Health Workers
49	Physician	2,734	3,053	3,419	2.07	4,838	Chad	Maximum Estimate	2212 - Specialist medical practitioners	Low Income	West	Medical Doctors
50	Physiotherapist	583	657	739	0.45	22,406	Chad	Maximum Estimate	2264 - Physiotherapists	Low Income	West	Other Health Workers
51	Plastic Surgeon	148	168	189	0.11	87,905	Chad	Maximum Estimate	2212 - Specialist medical practitioners	Low Income	West	Medical Doctors
52	Psychiatrist	1,423	1,600	1,827	1.11	9,027	Chad	Maximum Estimate	2212 - Specialist medical practitioners	Low Income	West	Medical Doctors
53	Radiation Oncologist	39	44	51	0.03	326,338	Chad	Maximum Estimate	2212 - Specialist medical practitioners	Low Income	West	Medical Doctors
54	Radiographer (Diagnostics and Therapy)	1,694	1,911	2,186	1.32	7,550	Chad	Maximum Estimate	3211 - Medical imaging and therapeutic equipment technicians	Low Income	West	Other Health Workers
55	Radiologist	673	757	859	0.52	19,247	Chad	Maximum Estimate	2212 - Specialist medical practitioners	Low Income	West	Medical Doctors
56	Registered General Nurse / State Certified Nurse	90,069	94,771	100,044	60.68	165	Chad	Maximum Estimate	2221 - Nursing professionals	Low Income	West	Nursing Personnel
57	Renal Nurse	3,077	3,518	4,151	2.53	3,957	Chad	Maximum Estimate	2221 - Nursing professionals	Low Income	West	Nursing Personnel
58	Respiratory Physician	165	191	221	0.13	74,769	Chad	Maximum Estimate	2212 - Specialist medical practitioners	Low Income	West	Medical Doctors
59	Rheumatologist	56	63	71	0.04	232,698	Chad	Maximum Estimate	2212 - Specialist medical practitioners	Low Income	West	Medical Doctors
60	Speech Therapist	211	238	268	0.16	61,795	Chad	Maximum Estimate	2266 - Audiologists and speech therapists	Low Income	West	Other Health Workers
61	Urologist	29	33	38	0.02	433,675	Chad	Maximum Estimate	2212 - Specialist medical practitioners	Low Income	West	Medical Doctors
1	Anaesthesiologist	24	28	32	0.37	27,367	Comoros	Maximum Estimate	2212 - Specialist medical practitioners	Lower-middle Income	Southern	Medical Doctors
2	Associate Nurse/Enrolled Nurse/Nursing Assistant	1,514	1,638	1,775	20.28	493	Comoros	Maximum Estimate	3221 - Nursing associate professionals	Lower-middle Income	Southern	Nursing Personnel
3	Audiologist	7	8	8	0.10	104,996	Comoros	Maximum Estimate	2266 - Audiologists and speech therapists	Lower-middle Income	Southern	Other Health Workers
4	Cardiologist	19	22	25	0.29	35,070	Comoros	Maximum Estimate	2212 - Specialist medical practitioners	Lower-middle Income	Southern	Medical Doctors
5	Cardiothoracic Surgeon	5	6	7	0.07	133,828	Comoros	Maximum Estimate	2212 - Specialist medical practitioners	Lower-middle Income	Southern	Medical Doctors
6	Clinical Officer/Physician Assistant	430	456	484	5.53	1,807	Comoros	Maximum Estimate	3256 - Medical assistants	Lower-middle Income	Southern	Other Health Workers
7	Clinical Pharmacist	43	48	54	0.62	16,119	Comoros	Maximum Estimate	2262 - Pharmacists	Lower-middle Income	Southern	Pharmacist
8	Clinical Psychologist	67	74	83	0.94	10,598	Comoros	Maximum Estimate	2634 - Psychologists	Lower-middle Income	Southern	Other Health Workers
9	Community health worker/Village health worker	1,028	1,084	1,137	12.99	770	Comoros	Maximum Estimate	3253 - Community health workers	Lower-middle Income	Southern	Other Health Workers
10	Dental Surgery Assistant	158	175	194	2.21	4,515	Comoros	Maximum Estimate	3251 - Dental assistants and therapists	Lower-middle Income	Southern	Other Health Workers
11	Dental Therapist	105	117	130	1.48	6,749	Comoros	Maximum Estimate	3251 - Dental assistants and therapists	Lower-middle Income	Southern	Other Health Workers
12	Dentist	135	148	164	1.88	5,326	Comoros	Maximum Estimate	2261 - Dentists	Lower-middle Income	Southern	Dentist
13	Dermatologist	5	5	6	0.06	155,788	Comoros	Maximum Estimate	2212 - Specialist medical practitioners	Lower-middle Income	Southern	Medical Doctors
14	Endocrinologist	5	6	8	0.09	114,568	Comoros	Maximum Estimate	2212 - Specialist medical practitioners	Lower-middle Income	Southern	Medical Doctors
15	ENT Surgeon	21	23	26	0.30	33,371	Comoros	Maximum Estimate	2212 - Specialist medical practitioners	Lower-middle Income	Southern	Medical Doctors
16	Environmental Health Officer	65	70	74	0.85	11,807	Comoros	Maximum Estimate	2263 - Environmental and occupational health and hygiene professionals	Lower-middle Income	Southern	Other Health Workers
17	Gastroenterologist	13	13	14	0.16	62,998	Comoros	Maximum Estimate	2212 - Specialist medical practitioners	Lower-middle Income	Southern	Medical Doctors
18	General Medical Practitioner (Generalist Doctor)	515	559	608	6.94	1,440	Comoros	Maximum Estimate	2211 - Generalist medical practitioners	Lower-middle Income	Southern	Medical Doctors
19	General Surgeon	14	16	19	0.22	46,481	Comoros	Maximum Estimate	2212 - Specialist medical practitioners	Lower-middle Income	Southern	Medical Doctors
20	Haematologist	6	6	7	0.08	126,283	Comoros	Maximum Estimate	2212 - Specialist medical practitioners	Lower-middle Income	Southern	Medical Doctors
21	Health Promoter/Health Educator	23	24	26	0.30	33,745	Comoros	Maximum Estimate	2269 - Health professionals not elsewhere classified	Lower-middle Income	Southern	Other Health Workers
22	Infectious Diseases Specialist	1	1	1	0.01	699,073	Comoros	Maximum Estimate	2212 - Specialist medical practitioners	Lower-middle Income	Southern	Medical Doctors
23	Intensive Care Nurse	51	55	60	0.68	14,681	Comoros	Maximum Estimate	2221 - Nursing professionals	Lower-middle Income	Southern	Nursing Personnel
24	Medical Laboratory Scientist	154	171	191	2.19	4,569	Comoros	Maximum Estimate	3212 - Medical and pathology laboratory technicians	Lower-middle Income	Southern	Other Health Workers
25	Medical Laboratory Technician	118	130	143	1.64	6,097	Comoros	Maximum Estimate	3212 - Medical and pathology laboratory technicians	Lower-middle Income	Southern	Other Health Workers
26	Medical Social Worker	41	43	45	0.52	19,279	Comoros	Maximum Estimate	1344 - Social welfare managers	Lower-middle Income	Southern	Other Health Workers
27	Mental Health Nurse	104	115	130	1.49	6,724	Comoros	Maximum Estimate	2221 - Nursing professionals	Lower-middle Income	Southern	Nursing Personnel
28	Midwife	868	928	991	11.31	884	Comoros	Maximum Estimate	2222 - Midwifery professionals	Lower-middle Income	Southern	Midwifery Personnel
29	Nephrologist	16	19	22	0.25	40,257	Comoros	Maximum Estimate	2212 - Specialist medical practitioners	Lower-middle Income	Southern	Medical Doctors
30	Neuro-Surgeon	8	9	11	0.12	80,669	Comoros	Maximum Estimate	2212 - Specialist medical practitioners	Lower-middle Income	Southern	Medical Doctors
31	Nurse Anaesthetist	52	62	74	0.85	11,800	Comoros	Maximum Estimate	2221 - Nursing professionals	Lower-middle Income	Southern	Nursing Personnel
32	Nutritionist	149	150	151	1.74	5,763	Comoros	Maximum Estimate	2265 - Dieticians and nutritionists	Lower-middle Income	Southern	Other Health Workers
33	Obstetrician & Gynaecologist	87	95	105	1.20	8,325	Comoros	Maximum Estimate	2212 - Specialist medical practitioners	Lower-middle Income	Southern	Medical Doctors
34	Occupational Therapist	55	63	73	0.84	11,934	Comoros	Maximum Estimate	2269 - Health professionals not elsewhere classified	Lower-middle Income	Southern	Other Health Workers
35	Oncology Nurse	16	19	22	0.25	39,597	Comoros	Maximum Estimate	2221 - Nursing professionals	Lower-middle Income	Southern	Nursing Personnel
36	Operating Theatre Nurse	192	217	250	2.86	3,491	Comoros	Maximum Estimate	2221 - Nursing professionals	Lower-middle Income	Southern	Nursing Personnel
37	Ophthalmic Nurse	16	18	20	0.23	43,298	Comoros	Maximum Estimate	2221 - Nursing professionals	Lower-middle Income	Southern	Nursing Personnel
38	Ophthalmologist	7	8	9	0.10	95,384	Comoros	Maximum Estimate	2212 - Specialist medical practitioners	Lower-middle Income	Southern	Medical Doctors
39	Optometrist	34	38	44	0.50	19,985	Comoros	Maximum Estimate	2267 - Optometrists and ophthalmic opticians	Lower-middle Income	Southern	Other Health Workers
40	Orthopaedic Nurse	80	93	109	1.24	8,071	Comoros	Maximum Estimate	2221 - Nursing professionals	Lower-middle Income	Southern	Nursing Personnel

S/N	Health Professionals	2022	2026	2030	Density per 10,000 population	Required Population ratio (1 professional is to xxx population)	Name of Country	Modelling Scenario	ISCO-08 Match	Income Group Classification	Sub-Regional Grouping	SDG 3c Occupation
41	Orthopaedic Surgeon	63	73	84	0.97	10,355	Comoros	Maximum Estimate	2212 - Specialist medical practitioners	Lower-middle Income	Southern	Medical Doctors
42	Orthopaedic Technologist	59	66	75	0.85	11,743	Comoros	Maximum Estimate	3214 - Medical and dental prosthetic technicians	Lower-middle Income	Southern	Other Health Workers
43	Paediatric Nurse	147	157	164	1.87	5,338	Comoros	Maximum Estimate	2221 - Nursing professionals	Lower-middle Income	Southern	Nursing Personnel
44	Paediatric Surgeon	4	4	4	0.04	239,407	Comoros	Maximum Estimate	2212 - Specialist medical practitioners	Lower-middle Income	Southern	Medical Doctors
45	Paediatrician	68	74	81	0.92	10,837	Comoros	Maximum Estimate	2212 - Specialist medical practitioners	Lower-middle Income	Southern	Medical Doctors
46	Pathologist	15	17	20	0.22	44,593	Comoros	Maximum Estimate	2212 - Specialist medical practitioners	Lower-middle Income	Southern	Medical Doctors
47	Pharmacist	211	221	233	2.66	3,756	Comoros	Maximum Estimate	2262 - Pharmacists	Lower-middle Income	Southern	Pharmacist
48	Pharmacy Technician	178	196	216	2.47	4,055	Comoros	Maximum Estimate	3213 - Pharmaceutical technicians and assistants	Lower-middle Income	Southern	Other Health Workers
49	Physician	134	148	163	1.87	5,356	Comoros	Maximum Estimate	2212 - Specialist medical practitioners	Lower-middle Income	Southern	Medical Doctors
50	Physiotherapist	47	53	60	0.68	14,606	Comoros	Maximum Estimate	2264 - Physiotherapists	Lower-middle Income	Southern	Other Health Workers
51	Plastic Surgeon	28	32	35	0.40	24,923	Comoros	Maximum Estimate	2212 - Specialist medical practitioners	Lower-middle Income	Southern	Medical Doctors
52	Psychiatrist	74	81	90	1.03	9,740	Comoros	Maximum Estimate	2212 - Specialist medical practitioners	Lower-middle Income	Southern	Medical Doctors
53	Radiation Oncologist	6	8	9	0.11	93,092	Comoros	Maximum Estimate	2212 - Specialist medical practitioners	Lower-middle Income	Southern	Medical Doctors
54	Radiographer (Diagnostics and Therapy)	114	129	146	1.68	5,963	Comoros	Maximum Estimate	3211 - Medical imaging and therapeutic equipment technicians	Lower-middle Income	Southern	Other Health Workers
55	Radiologist	46	53	61	0.70	14,308	Comoros	Maximum Estimate	2212 - Specialist medical practitioners	Lower-middle Income	Southern	Medical Doctors
56	Registered General Nurse / State Certified Nurse	3,587	3,934	4,341	49.73	201	Comoros	Maximum Estimate	2221 - Nursing professionals	Lower-middle Income	Southern	Nursing Personnel
57	Renal Nurse	327	372	432	4.95	2,019	Comoros	Maximum Estimate	2221 - Nursing professionals	Lower-middle Income	Southern	Nursing Personnel
58	Respiratory Physician	14	16	17	0.19	52,050	Comoros	Maximum Estimate	2212 - Specialist medical practitioners	Lower-middle Income	Southern	Medical Doctors
59	Rheumatologist	4	5	5	0.06	173,716	Comoros	Maximum Estimate	2212 - Specialist medical practitioners	Lower-middle Income	Southern	Medical Doctors
60	Speech Therapist	13	14	15	0.17	57,688	Comoros	Maximum Estimate	2266 - Audiologists and speech therapists	Lower-middle Income	Southern	Other Health Workers
61	Urologist	3	4	4	0.05	206,723	Comoros	Maximum Estimate	2212 - Specialist medical practitioners	Lower-middle Income	Southern	Medical Doctors
1	Anaesthesiologist	85	96	108	0.19	51,286	Congo	Maximum Estimate	2212 - Specialist medical practitioners	Lower-middle Income	Central	Medical Doctors
2	Associate Nurse/Enrolled Nurse/Nursing Assistant	11,109	12,331	13,725	24.75	404	Congo	Maximum Estimate	3221 - Nursing associate professionals	Lower-middle Income	Central	Nursing Personnel
3	Audiologist	35	39	43	0.08	127,833	Congo	Maximum Estimate	2266 - Audiologists and speech therapists	Lower-middle Income	Central	Other Health Workers
4	Cardiologist	89	102	117	0.21	47,622	Congo	Maximum Estimate	2212 - Specialist medical practitioners	Lower-middle Income	Central	Medical Doctors
5	Cardiothoracic Surgeon	16	18	20	0.04	279,519	Congo	Maximum Estimate	2212 - Specialist medical practitioners	Lower-middle Income	Central	Medical Doctors
6	Clinical Officer/Physician Assistant	2,125	2,490	2,950	5.36	1,866	Congo	Maximum Estimate	3256 - Medical assistants	Lower-middle Income	Central	Other Health Workers
7	Clinical Pharmacist	283	312	349	0.63	15,847	Congo	Maximum Estimate	2262 - Pharmacists	Lower-middle Income	Central	Pharmacist
8	Clinical Psychologist	587	640	702	1.27	7,898	Congo	Maximum Estimate	2634 - Psychologists	Lower-middle Income	Central	Other Health Workers
9	Community health worker/Village health worker	12,111	14,284	16,930	30.71	326	Congo	Maximum Estimate	3253 - Community health workers	Lower-middle Income	Central	Other Health Workers
10	Dental Surgery Assistant	921	1,028	1,149	2.07	4,831	Congo	Maximum Estimate	3251 - Dental assistants and therapists	Lower-middle Income	Central	Other Health Workers
11	Dental Therapist	616	688	768	1.38	7,228	Congo	Maximum Estimate	3251 - Dental assistants and therapists	Lower-middle Income	Central	Other Health Workers
12	Dentist	714	787	875	1.58	6,342	Congo	Maximum Estimate	2261 - Dentists	Lower-middle Income	Central	Dentist
13	Dermatologist	30	33	36	0.06	154,190	Congo	Maximum Estimate	2212 - Specialist medical practitioners	Lower-middle Income	Central	Medical Doctors
14	Endocrinologist	49	59	71	0.13	77,340	Congo	Maximum Estimate	2212 - Specialist medical practitioners	Lower-middle Income	Central	Medical Doctors
15	ENT Surgeon	105	118	135	0.24	40,993	Congo	Maximum Estimate	2212 - Specialist medical practitioners	Lower-middle Income	Central	Medical Doctors
16	Environmental Health Officer	411	449	489	0.88	11,360	Congo	Maximum Estimate	2263 - Environmental and occupational health and hygiene professionals	Lower-middle Income	Central	Other Health Workers
17	Gastroenterologist	109	112	118	0.21	46,799	Congo	Maximum Estimate	2212 - Specialist medical practitioners	Lower-middle Income	Central	Medical Doctors
18	General Medical Practitioner (Generalist Doctor)	3,884	4,361	4,918	8.89	1,125	Congo	Maximum Estimate	2211 - Generalist medical practitioners	Lower-middle Income	Central	Medical Doctors
19	General Surgeon	95	110	129	0.23	42,821	Congo	Maximum Estimate	2212 - Specialist medical practitioners	Lower-middle Income	Central	Medical Doctors
20	Haematologist	31	34	36	0.07	152,595	Congo	Maximum Estimate	2212 - Specialist medical practitioners	Lower-middle Income	Central	Medical Doctors
21	Health Promoter/Health Educator	186	214	249	0.45	22,089	Congo	Maximum Estimate	2269 - Health professionals not elsewhere classified	Lower-middle Income	Central	Other Health Workers
22	Infectious Diseases Specialist	13	14	15	0.03	376,379	Congo	Maximum Estimate	2212 - Specialist medical practitioners	Lower-middle Income	Central	Medical Doctors
23	Intensive Care Nurse	287	313	341	0.61	16,270	Congo	Maximum Estimate	2221 - Nursing professionals	Lower-middle Income	Central	Nursing Personnel
24	Medical Laboratory Scientist	1,445	1,657	1,922	3.48	2,870	Congo	Maximum Estimate	3212 - Medical and pathology laboratory technicians	Lower-middle Income	Central	Other Health Workers
25	Medical Laboratory Technician	2,489	3,033	3,709	6.74	1,484	Congo	Maximum Estimate	3212 - Medical and pathology laboratory technicians	Lower-middle Income	Central	Other Health Workers
26	Medical Social Worker	268	282	302	0.55	18,188	Congo	Maximum Estimate	1344 - Social welfare managers	Lower-middle Income	Central	Other Health Workers
27	Mental Health Nurse	621	683	766	1.39	7,220	Congo	Maximum Estimate	2221 - Nursing professionals	Lower-middle Income	Central	Nursing Personnel
28	Midwife	5,620	6,139	6,703	12.07	828	Congo	Maximum Estimate	2222 - Midwifery professionals	Lower-middle Income	Central	Midwifery Personnel
29	Nephrologist	88	100	117	0.21	47,250	Congo	Maximum Estimate	2212 - Specialist medical practitioners	Lower-middle Income	Central	Medical Doctors
30	Neuro-Surgeon	32	36	40	0.07	136,959	Congo	Maximum Estimate	2212 - Specialist medical practitioners	Lower-middle Income	Central	Medical Doctors
31	Nurse Anaesthetist	232	268	310	0.56	17,911	Congo	Maximum Estimate	2221 - Nursing professionals	Lower-middle Income	Central	Nursing Personnel
32	Nutritionist	715	739	762	1.38	7,268	Congo	Maximum Estimate	2265 - Dieticians and nutritionists	Lower-middle Income	Central	Other Health Workers
33	Obstetrician & Gynaecologist	584	651	730	1.32	7,583	Congo	Maximum Estimate	2212 - Specialist medical practitioners	Lower-middle Income	Central	Medical Doctors
34	Occupational Therapist	165	187	214	0.39	25,891	Congo	Maximum Estimate	2269 - Health professionals not elsewhere classified	Lower-middle Income	Central	Other Health Workers
35	Oncology Nurse	80	93	108	0.20	50,976	Congo	Maximum Estimate	2221 - Nursing professionals	Lower-middle Income	Central	Nursing Personnel
36	Operating Theatre Nurse	995	1,130	1,306	2.37	4,223	Congo	Maximum Estimate	2221 - Nursing professionals	Lower-middle Income	Central	Nursing Personnel
37	Ophthalmic Nurse	154	176	201	0.36	27,486	Congo	Maximum Estimate	2221 - Nursing professionals	Lower-middle Income	Central	Nursing Personnel
38	Ophthalmologist	39	45	53	0.10	104,429	Congo	Maximum Estimate	2212 - Specialist medical practitioners	Lower-middle Income	Central	Medical Doctors
39	Optometrist	155	179	208	0.38	26,605	Congo	Maximum Estimate	2267 - Optometrists and ophthalmic opticians	Lower-middle Income	Central	Other Health Workers
40	Orthopaedic Nurse	186	210	236	0.43	23,526	Congo	Maximum Estimate	2221 - Nursing professionals	Lower-middle Income	Central	Nursing Personnel
41	Orthopaedic Surgeon	337	396	467	0.84	11,838	Congo	Maximum Estimate	2212 - Specialist medical practitioners	Lower-middle Income	Central	Medical Doctors
42	Orthopaedic Technologist	240	264	291	0.52	19,120	Congo	Maximum Estimate	3214 - Medical and dental prosthetic technicians	Lower-middle Income	Central	Other Health Workers
43	Paediatric Nurse	863	931	993	1.79	5,586	Congo	Maximum Estimate	2221 - Nursing professionals	Lower-middle Income	Central	Nursing Personnel
44	Paediatric Surgeon	27	29	30	0.05	183,964	Congo	Maximum Estimate	2212 - Specialist medical practitioners	Lower-middle Income	Central	Medical Doctors
45	Paediatrician	403	446	495	0.89	11,206	Congo	Maximum Estimate	2212 - Specialist medical practitioners	Lower-middle Income	Central	Medical Doctors
46	Pathologist	94	105	120	0.22	46,144	Congo	Maximum Estimate	2212 - Specialist medical practitioners	Lower-middle Income	Central	Medical Doctors
47	Pharmacist	851	899	958	1.73	5,770	Congo	Maximum Estimate	2262 - Pharmacists	Lower-middle Income	Central	Pharmacist
48	Pharmacy Technician	1,009	1,103	1,211	2.19	4,576	Congo	Maximum Estimate	3213 - Pharmaceutical technicians and assistants	Lower-middle Income	Central	Other Health Workers
49	Physician	995	1,123	1,274	2.30	4,343	Congo	Maximum Estimate	2212 - Specialist medical practitioners	Lower-middle Income	Central	Medical Doctors
50	Physiotherapist	220	244	272	0.49	20,330	Congo	Maximum Estimate	2264 - Physiotherapists	Lower-middle Income	Central	Other Health Workers
51	Plastic Surgeon	49	55	62	0.11	89,320	Congo	Maximum Estimate	2212 - Specialist medical practitioners	Lower-middle Income	Central	Medical Doctors
52	Psychiatrist	456	499	554	1.00	9,982	Congo	Maximum Estimate	2212 - Specialist medical practitioners	Lower-middle Income	Central	Medical Doctors
53	Radiation Oncologist	33	39	46	0.08	119,826	Congo	Maximum Estimate	2212 - Specialist medical practitioners	Lower-middle Income	Central	Medical Doctors
54	Radiographer (Diagnostics and Therapy)	742	841	963	1.74	5,733	Congo	Maximum Estimate	3211 - Medical imaging and therapeutic equipment technicians	Lower-middle Income	Central	Other Health Workers
55	Radiologist	290	333	388	0.70	14,189	Congo	Maximum Estimate	2212 - Specialist medical practitioners	Lower-middle Income	Central	Medical Doctors
56	Registered General Nurse / State Certified Nurse	19,935	22,089	24,565	44.36	225	Congo	Maximum Estimate	2221 - Nursing professionals	Lower-middle Income	Central	Nursing Personnel
57	Renal Nurse	1,741	1,992	2,320	4.21	2,376	Congo	Maximum Estimate	2221 - Nursing professionals	Lower-middle Income	Central	Nursing Personnel
58	Respiratory Physician	84	93	104	0.19	53,612	Congo	Maximum Estimate	2212 - Specialist medical practitioners	Lower-middle Income	Central	Medical Doctors
59	Rheumatologist	24	27	30	0.05	182,429	Congo	Maximum Estimate	2212 - Specialist medical practitioners	Lower-middle Income	Central	Medical Doctors
60	Speech Therapist	71	78	87	0.16	63,438	Congo	Maximum Estimate	2266 - Audiologists and speech therapists	Lower-middle Income	Central	Other Health Workers
61	Urologist	15	18	22	0.04	250,844	Congo	Maximum Estimate	2212 - Specialist medical practitioners	Lower-middle Income	Central	Medical Doctors
1	Anaesthesiologist	464	533	611	0.23	43,445	Côte d'Ivoire	Maximum Estimate	2212 - Specialist medical practitioners	Lower-middle Income	West	Medical Doctors
2	Associate Nurse/Enrolled Nurse/Nursing Assistant	45,327	50,179	55,678	20.97	477	Côte d'Ivoire	Maximum Estimate	3221 - Nursing associate professionals	Lower-middle Income	West	Nursing Personnel

S/N	Health Professionals	2022	2026	2030	Density per 10,000 population	Required Population ratio (1 professional is to xxx population)	Name of Country	Modelling Scenario	ISCO-08 Match	Income Group Classification	Sub-Regional Grouping	SDG 3c Occupation
3	Audiologist	171	191	212	0.08	125,366	Côte d'Ivoire	Maximum Estimate	2266 - Audiologists and speech therapists	Lower-middle Income	West	Other Health Workers
4	Cardiologist	358	413	474	0.18	56,056	Côte d'Ivoire	Maximum Estimate	2212 - Specialist medical practitioners	Lower-middle Income	West	Medical Doctors
5	Cardiothoracic Surgeon	84	97	110	0.04	241,967	Côte d'Ivoire	Maximum Estimate	2212 - Specialist medical practitioners	Lower-middle Income	West	Medical Doctors
6	Clinical Officer/Physician Assistant	10,146	10,151	10,226	3.88	2,576	Côte d'Ivoire	Maximum Estimate	3256 - Medical assistants	Lower-middle Income	West	Other Health Workers
7	Clinical Pharmacist	1,197	1,344	1,523	0.57	17,400	Côte d'Ivoire	Maximum Estimate	2262 - Pharmacists	Lower-middle Income	West	Pharmacist
8	Clinical Psychologist	2,163	2,404	2,690	1.01	9,869	Côte d'Ivoire	Maximum Estimate	2634 - Psychologists	Lower-middle Income	West	Other Health Workers
9	Community health worker/Village health worker	44,503	46,063	47,877	18.12	552	Côte d'Ivoire	Maximum Estimate	3253 - Community health workers	Lower-middle Income	West	Other Health Workers
10	Dental Surgery Assistant	4,508	5,067	5,696	2.14	4,667	Côte d'Ivoire	Maximum Estimate	3251 - Dental assistants and therapists	Lower-middle Income	West	Other Health Workers
11	Dental Therapist	3,015	3,388	3,808	1.43	6,982	Côte d'Ivoire	Maximum Estimate	3251 - Dental assistants and therapists	Lower-middle Income	West	Other Health Workers
12	Dentist	3,448	3,823	4,269	1.61	6,223	Côte d'Ivoire	Maximum Estimate	2261 - Dentists	Lower-middle Income	West	Dentist
13	Dermatologist	136	152	169	0.06	157,390	Côte d'Ivoire	Maximum Estimate	2212 - Specialist medical practitioners	Lower-middle Income	West	Medical Doctors
14	Endocrinologist	140	167	205	0.08	128,478	Côte d'Ivoire	Maximum Estimate	2212 - Specialist medical practitioners	Lower-middle Income	West	Medical Doctors
15	ENT Surgeon	491	558	634	0.24	41,897	Côte d'Ivoire	Maximum Estimate	2212 - Specialist medical practitioners	Lower-middle Income	West	Medical Doctors
16	Environmental Health Officer	1,965	2,168	2,384	0.90	11,160	Côte d'Ivoire	Maximum Estimate	2263 - Environmental and occupational health and hygiene professionals	Lower-middle Income	West	Other Health Workers
17	Gastroenterologist	388	390	401	0.15	65,693	Côte d'Ivoire	Maximum Estimate	2212 - Specialist medical practitioners	Lower-middle Income	West	Medical Doctors
18	General Medical Practitioner (Generalist Doctor)	16,142	17,574	19,199	7.24	1,381	Côte d'Ivoire	Maximum Estimate	2211 - Generalist medical practitioners	Lower-middle Income	West	Medical Doctors
19	General Surgeon	395	453	524	0.20	50,549	Côte d'Ivoire	Maximum Estimate	2212 - Specialist medical practitioners	Lower-middle Income	West	Medical Doctors
20	Haematologist	160	184	211	0.08	125,847	Côte d'Ivoire	Maximum Estimate	2212 - Specialist medical practitioners	Lower-middle Income	West	Medical Doctors
21	Health Promoter/Health Educator	688	696	712	0.27	36,938	Côte d'Ivoire	Maximum Estimate	2269 - Health professionals not elsewhere classified	Lower-middle Income	West	Other Health Workers
22	Infectious Diseases Specialist	55	59	64	0.02	413,348	Côte d'Ivoire	Maximum Estimate	2212 - Specialist medical practitioners	Lower-middle Income	West	Medical Doctors
23	Intensive Care Nurse	1,361	1,505	1,662	0.63	15,999	Côte d'Ivoire	Maximum Estimate	2221 - Nursing professionals	Lower-middle Income	West	Nursing Personnel
24	Medical Laboratory Scientist	4,831	5,242	5,782	2.19	4,568	Côte d'Ivoire	Maximum Estimate	3212 - Medical and pathology laboratory technicians	Lower-middle Income	West	Other Health Workers
25	Medical Laboratory Technician	7,077	7,119	7,245	2.76	3,628	Côte d'Ivoire	Maximum Estimate	3212 - Medical and pathology laboratory technicians	Lower-middle Income	West	Other Health Workers
26	Medical Social Worker	1,366	1,449	1,555	0.59	16,969	Côte d'Ivoire	Maximum Estimate	1344 - Social welfare managers	Lower-middle Income	West	Other Health Workers
27	Mental Health Nurse	2,473	2,774	3,170	1.20	8,363	Côte d'Ivoire	Maximum Estimate	2221 - Nursing professionals	Lower-middle Income	West	Nursing Personnel
28	Midwife	27,787	30,380	33,248	12.51	800	Côte d'Ivoire	Maximum Estimate	2222 - Midwifery professionals	Lower-middle Income	West	Midwifery Personnel
29	Nephrologist	406	465	545	0.21	48,612	Côte d'Ivoire	Maximum Estimate	2212 - Specialist medical practitioners	Lower-middle Income	West	Medical Doctors
30	Neuro-Surgeon	158	179	204	0.08	129,957	Côte d'Ivoire	Maximum Estimate	2212 - Specialist medical practitioners	Lower-middle Income	West	Medical Doctors
31	Nurse Anaesthetist	855	994	1,154	0.43	22,994	Côte d'Ivoire	Maximum Estimate	2221 - Nursing professionals	Lower-middle Income	West	Nursing Personnel
32	Nutritionist	5,413	5,778	6,149	2.31	4,320	Côte d'Ivoire	Maximum Estimate	2265 - Dieticians and nutritionists	Lower-middle Income	West	Other Health Workers
33	Obstetrician & Gynaecologist	2,425	2,736	3,100	1.17	8,552	Côte d'Ivoire	Maximum Estimate	2212 - Specialist medical practitioners	Lower-middle Income	West	Medical Doctors
34	Occupational Therapist	867	1,003	1,164	0.44	22,781	Côte d'Ivoire	Maximum Estimate	2269 - Health professionals not elsewhere classified	Lower-middle Income	West	Other Health Workers
35	Oncology Nurse	305	352	412	0.16	64,309	Côte d'Ivoire	Maximum Estimate	2221 - Nursing professionals	Lower-middle Income	West	Nursing Personnel
36	Operating Theatre Nurse	4,320	4,929	5,712	2.15	4,641	Côte d'Ivoire	Maximum Estimate	2221 - Nursing professionals	Lower-middle Income	West	Nursing Personnel
37	Ophthalmic Nurse	451	514	588	0.22	45,117	Côte d'Ivoire	Maximum Estimate	2221 - Nursing professionals	Lower-middle Income	West	Nursing Personnel
38	Ophthalmologist	159	183	213	0.08	124,277	Côte d'Ivoire	Maximum Estimate	2212 - Specialist medical practitioners	Lower-middle Income	West	Medical Doctors
39	Optometrist	644	745	864	0.33	30,725	Côte d'Ivoire	Maximum Estimate	2267 - Optometrists and ophthalmic opticians	Lower-middle Income	West	Other Health Workers
40	Orthopaedic Nurse	1,032	1,208	1,410	0.53	18,831	Côte d'Ivoire	Maximum Estimate	2221 - Nursing professionals	Lower-middle Income	West	Nursing Personnel
41	Orthopaedic Surgeon	1,433	1,662	1,933	0.73	13,730	Côte d'Ivoire	Maximum Estimate	2212 - Specialist medical practitioners	Lower-middle Income	West	Medical Doctors
42	Orthopaedic Technologist	1,223	1,378	1,548	0.58	17,168	Côte d'Ivoire	Maximum Estimate	3214 - Medical and dental prosthetic technicians	Lower-middle Income	West	Other Health Workers
43	Paediatric Nurse	4,755	5,280	5,813	2.18	4,578	Côte d'Ivoire	Maximum Estimate	2221 - Nursing professionals	Lower-middle Income	West	Nursing Personnel
44	Paediatric Surgeon	150	164	177	0.07	150,447	Côte d'Ivoire	Maximum Estimate	2212 - Specialist medical practitioners	Lower-middle Income	West	Medical Doctors
45	Paediatrician	1,824	2,060	2,332	0.88	11,391	Côte d'Ivoire	Maximum Estimate	2212 - Specialist medical practitioners	Lower-middle Income	West	Medical Doctors
46	Pathologist	285	323	373	0.14	71,163	Côte d'Ivoire	Maximum Estimate	2212 - Specialist medical practitioners	Lower-middle Income	West	Medical Doctors
47	Pharmacist	4,335	4,459	4,605	1.74	5,737	Côte d'Ivoire	Maximum Estimate	2262 - Pharmacists	Lower-middle Income	West	Pharmacist
48	Pharmacy Technician	4,692	5,185	5,743	2.16	4,622	Côte d'Ivoire	Maximum Estimate	3213 - Pharmaceutical technicians and assistants	Lower-middle Income	West	Other Health Workers
49	Physician	4,493	4,975	5,540	2.09	4,790	Côte d'Ivoire	Maximum Estimate	2212 - Specialist medical practitioners	Lower-middle Income	West	Medical Doctors
50	Physiotherapist	1,059	1,190	1,339	0.50	19,837	Côte d'Ivoire	Maximum Estimate	2264 - Physiotherapists	Lower-middle Income	West	Other Health Workers
51	Plastic Surgeon	286	328	376	0.14	70,721	Côte d'Ivoire	Maximum Estimate	2212 - Specialist medical practitioners	Lower-middle Income	West	Medical Doctors
52	Psychiatrist	2,062	2,291	2,581	0.97	10,276	Côte d'Ivoire	Maximum Estimate	2212 - Specialist medical practitioners	Lower-middle Income	West	Medical Doctors
53	Radiation Oncologist	95	111	131	0.05	202,403	Côte d'Ivoire	Maximum Estimate	2212 - Specialist medical practitioners	Lower-middle Income	West	Medical Doctors
54	Radiographer (Diagnostics and Therapy)	3,300	3,745	4,301	1.62	6,160	Côte d'Ivoire	Maximum Estimate	3211 - Medical imaging and therapeutic equipment technicians	Lower-middle Income	West	Other Health Workers
55	Radiologist	1,272	1,467	1,710	0.65	15,427	Côte d'Ivoire	Maximum Estimate	2212 - Specialist medical practitioners	Lower-middle Income	West	Medical Doctors
56	Registered General Nurse / State Certified Nurse	94,392	103,850	115,293	43.52	230	Côte d'Ivoire	Maximum Estimate	2221 - Nursing professionals	Lower-middle Income	West	Nursing Personnel
57	Renal Nurse	7,875	9,035	10,605	4.00	2,498	Côte d'Ivoire	Maximum Estimate	2221 - Nursing professionals	Lower-middle Income	West	Nursing Personnel
58	Respiratory Physician	343	388	437	0.16	60,833	Côte d'Ivoire	Maximum Estimate	2212 - Specialist medical practitioners	Lower-middle Income	West	Medical Doctors
59	Rheumatologist	105	119	134	0.05	197,658	Côte d'Ivoire	Maximum Estimate	2212 - Specialist medical practitioners	Lower-middle Income	West	Medical Doctors
60	Speech Therapist	351	389	431	0.16	61,712	Côte d'Ivoire	Maximum Estimate	2266 - Audiologists and speech therapists	Lower-middle Income	West	Other Health Workers
61	Urologist	81	99	124	0.05	210,737	Côte d'Ivoire	Maximum Estimate	2212 - Specialist medical practitioners	Lower-middle Income	West	Medical Doctors
1	Anaesthesiologist	1,167	1,369	1,602	0.18	56,190	Democratic Republi	Maximum Estimate	2212 - Specialist medical practitioners	Low Income	Central	Medical Doctors
2	Associate Nurse/Enrolled Nurse/Nursing Assistant	162,912	184,216	208,584	23.15	432	Democratic Republi	Maximum Estimate	3221 - Nursing associate professionals	Low Income	Central	Nursing Personnel
3	Audiologist	592	668	750	0.08	120,505	Democratic Republi	Maximum Estimate	2266 - Audiologists and speech therapists	Low Income	Central	Other Health Workers
4	Cardiologist	1,037	1,203	1,389	0.15	64,943	Democratic Republi	Maximum Estimate	2212 - Specialist medical practitioners	Low Income	Central	Medical Doctors
5	Cardiothoracic Surgeon	251	293	341	0.04	264,230	Democratic Republi	Maximum Estimate	2212 - Specialist medical practitioners	Low Income	Central	Medical Doctors
6	Clinical Officer/Physician Assistant	24,397	25,246	26,521	2.97	3,367	Democratic Republi	Maximum Estimate	3256 - Medical assistants	Low Income	Central	Other Health Workers
7	Clinical Pharmacist	4,023	4,576	5,242	0.58	17,146	Democratic Republi	Maximum Estimate	2262 - Pharmacists	Low Income	Central	Pharmacist
8	Clinical Psychologist	9,148	10,259	11,536	1.28	7,812	Democratic Republi	Maximum Estimate	2634 - Psychologists	Low Income	Central	Other Health Workers
9	Community health worker/Village health worker	139,285	145,926	153,161	17.05	587	Democratic Republi	Maximum Estimate	3253 - Community health workers	Low Income	Central	Other Health Workers
10	Dental Surgery Assistant	12,752	14,821	17,292	1.92	5,202	Democratic Republi	Maximum Estimate	3251 - Dental assistants and therapists	Low Income	Central	Other Health Workers
11	Dental Therapist	8,562	9,945	11,598	1.29	7,756	Democratic Republi	Maximum Estimate	3251 - Dental assistants and therapists	Low Income	Central	Other Health Workers
12	Dentist	10,938	12,553	14,649	1.63	6,124	Democratic Republi	Maximum Estimate	2261 - Dentists	Low Income	Central	Dentist
13	Dermatologist	468	533	606	0.07	148,988	Democratic Republi	Maximum Estimate	2212 - Specialist medical practitioners	Low Income	Central	Medical Doctors
14	Endocrinologist	511	620	766	0.09	116,025	Democratic Republi	Maximum Estimate	2212 - Specialist medical practitioners	Low Income	Central	Medical Doctors
15	ENT Surgeon	1,484	1,715	1,979	0.22	45,543	Democratic Republi	Maximum Estimate	2212 - Specialist medical practitioners	Low Income	Central	Medical Doctors
16	Environmental Health Officer	6,673	7,576	8,572	0.95	10,539	Democratic Republi	Maximum Estimate	2263 - Environmental and occupational health and hygiene professionals	Low Income	Central	Other Health Workers
17	Gastroenterologist	1,136	1,200	1,292	0.14	69,247	Democratic Republi	Maximum Estimate	2212 - Specialist medical practitioners	Low Income	Central	Medical Doctors
18	General Medical Practitioner (Generalist Doctor)	53,752	60,261	67,740	7.52	1,329	Democratic Republi	Maximum Estimate	2211 - Generalist medical practitioners	Low Income	Central	Medical Doctors
19	General Surgeon	1,344	1,566	1,849	0.21	48,492	Democratic Republi	Maximum Estimate	2212 - Specialist medical practitioners	Low Income	Central	Medical Doctors
20	Haematologist	452	508	569	0.06	158,769	Democratic Republi	Maximum Estimate	2212 - Specialist medical practitioners	Low Income	Central	Medical Doctors
21	Health Promoter/Health Educator	2,269	2,361	2,485	0.28	36,020	Democratic Republi	Maximum Estimate	2269 - Health professionals not elsewhere classified	Low Income	Central	Other Health Workers
22	Infectious Diseases Specialist	204	230	260	0.03	346,198	Democratic Republi	Maximum Estimate	2212 - Specialist medical practitioners	Low Income	Central	Medical Doctors
23	Intensive Care Nurse	5,128	5,802	6,554	0.73	13,765	Democratic Republi	Maximum Estimate	2221 - Nursing professionals	Low Income	Central	Nursing Personnel
24	Medical Laboratory Scientist	16,149	17,928	20,112	2.24	4,461	Democratic Republi	Maximum Estimate	3212 - Medical and pathology laboratory technicians	Low Income	Central	Other Health Workers
25	Medical Laboratory Technician	24,090	24,295	24,789	2.78	3,599	Democratic Republi	Maximum Estimate	3212 - Medical and pathology laboratory technicians	Low Income	Central	Other Health Workers

S/N	Health Professionals	2022	2026	2030	Density per 10,000 population	Required Population ratio (1 professional is to xxx population)	Name of Country	Modelling Scenario	ISCO-08 Match	Income Group Classification	Sub-Regional Grouping	SDG 3c Occupation
26	Medical Social Worker	4,505	4,812	5,142	0.57	17,491	Democratic Republi	Maximum Estimate	1344 - Social welfare managers	Low Income	Central	Other Health Workers
27	Mental Health Nurse	9,237	10,662	12,528	1.40	7,141	Democratic Republi	Maximum Estimate	2221 - Nursing professionals	Low Income	Central	Nursing Personnel
28	Midwife	100,358	109,734	121,218	13.50	741	Democratic Republi	Maximum Estimate	2222 - Midwifery professionals	Low Income	Central	Midwifery Personnel
29	Nephrologist	1,011	1,196	1,441	0.16	61,874	Democratic Republi	Maximum Estimate	2212 - Specialist medical practitioners	Low Income	Central	Medical Doctors
30	Neuro-Surgeon	442	519	609	0.07	147,669	Democratic Republi	Maximum Estimate	2212 - Specialist medical practitioners	Low Income	Central	Medical Doctors
31	Nurse Anaesthetist	2,480	2,966	3,543	0.39	25,374	Democratic Republi	Maximum Estimate	2221 - Nursing professionals	Low Income	Central	Nursing Personnel
32	Nutritionist	18,867	19,992	21,092	2.34	4,278	Democratic Republi	Maximum Estimate	2265 - Dieticians and nutritionists	Low Income	Central	Other Health Workers
33	Obstetrician & Gynaecologist	8,625	9,883	11,346	1.26	7,938	Democratic Republi	Maximum Estimate	2212 - Specialist medical practitioners	Low Income	Central	Medical Doctors
34	Occupational Therapist	2,170	2,554	3,014	0.34	29,803	Democratic Republi	Maximum Estimate	2269 - Health professionals not elsewhere classified	Low Income	Central	Other Health Workers
35	Oncology Nurse	855	1,017	1,223	0.14	73,158	Democratic Republi	Maximum Estimate	2221 - Nursing professionals	Low Income	Central	Nursing Personnel
36	Operating Theatre Nurse	12,401	14,445	17,031	1.90	5,257	Democratic Republi	Maximum Estimate	2221 - Nursing professionals	Low Income	Central	Nursing Personnel
37	Ophthalmic Nurse	1,327	1,542	1,800	0.20	49,910	Democratic Republi	Maximum Estimate	2221 - Nursing professionals	Low Income	Central	Nursing Personnel
38	Ophthalmologist	489	573	675	0.08	132,861	Democratic Republi	Maximum Estimate	2212 - Specialist medical practitioners	Low Income	Central	Medical Doctors
39	Optometrist	1,957	2,265	2,627	0.29	34,255	Democratic Republi	Maximum Estimate	2267 - Optometrists and ophthalmic opticians	Low Income	Central	Other Health Workers
40	Orthopaedic Nurse	2,593	3,084	3,655	0.41	24,633	Democratic Republi	Maximum Estimate	2221 - Nursing professionals	Low Income	Central	Nursing Personnel
41	Orthopaedic Surgeon	4,171	5,040	6,101	0.68	14,697	Democratic Republi	Maximum Estimate	2212 - Specialist medical practitioners	Low Income	Central	Medical Doctors
42	Orthopaedic Technologist	3,752	4,324	4,969	0.55	18,161	Democratic Republi	Maximum Estimate	3214 - Medical and dental prosthetic technicians	Low Income	Central	Other Health Workers
43	Paediatric Nurse	15,440	17,151	18,785	2.08	4,812	Democratic Republi	Maximum Estimate	2221 - Nursing professionals	Low Income	Central	Nursing Personnel
44	Paediatric Surgeon	501	553	600	0.07	150,739	Democratic Republi	Maximum Estimate	2212 - Specialist medical practitioners	Low Income	Central	Medical Doctors
45	Paediatrician	5,735	6,525	7,426	0.82	12,133	Democratic Republi	Maximum Estimate	2212 - Specialist medical practitioners	Low Income	Central	Medical Doctors
46	Pathologist	1,310	1,515	1,778	0.20	50,325	Democratic Republi	Maximum Estimate	2212 - Specialist medical practitioners	Low Income	Central	Medical Doctors
47	Pharmacist	13,691	14,812	16,089	1.79	5,593	Democratic Republi	Maximum Estimate	2262 - Pharmacists	Low Income	Central	Pharmacist
48	Pharmacy Technician	15,145	17,195	19,564	2.17	4,604	Democratic Republi	Maximum Estimate	3213 - Pharmaceutical technicians and assistants	Low Income	Central	Other Health Workers
49	Physician	14,202	16,033	18,220	2.03	4,932	Democratic Republi	Maximum Estimate	2212 - Specialist medical practitioners	Low Income	Central	Medical Doctors
50	Physiotherapist	3,292	3,787	4,354	0.48	20,698	Democratic Republi	Maximum Estimate	2264 - Physiotherapists	Low Income	Central	Other Health Workers
51	Plastic Surgeon	732	866	1,021	0.11	88,214	Democratic Republi	Maximum Estimate	2212 - Specialist medical practitioners	Low Income	Central	Medical Doctors
52	Psychiatrist	7,319	8,337	9,647	1.08	9,288	Democratic Republi	Maximum Estimate	2212 - Specialist medical practitioners	Low Income	Central	Medical Doctors
53	Radiation Oncologist	311	378	462	0.05	193,679	Democratic Republi	Maximum Estimate	2212 - Specialist medical practitioners	Low Income	Central	Medical Doctors
54	Radiographer (Diagnostics and Therapy)	9,407	10,984	12,946	1.44	6,925	Democratic Republi	Maximum Estimate	3211 - Medical imaging and therapeutic equipment technicians	Low Income	Central	Other Health Workers
55	Radiologist	3,636	4,260	5,023	0.56	17,867	Democratic Republi	Maximum Estimate	2212 - Specialist medical practitioners	Low Income	Central	Medical Doctors
56	Registered General Nurse / State Certified Nurse	352,843	383,075	420,133	46.90	213	Democratic Republi	Maximum Estimate	2221 - Nursing professionals	Low Income	Central	Nursing Personnel
57	Renal Nurse	19,884	23,527	28,344	3.18	3,146	Democratic Republi	Maximum Estimate	2221 - Nursing professionals	Low Income	Central	Nursing Personnel
58	Respiratory Physician	1,085	1,240	1,417	0.16	63,655	Democratic Republi	Maximum Estimate	2212 - Specialist medical practitioners	Low Income	Central	Medical Doctors
59	Rheumatologist	333	384	442	0.05	204,075	Democratic Republi	Maximum Estimate	2212 - Specialist medical practitioners	Low Income	Central	Medical Doctors
60	Speech Therapist	1,123	1,279	1,456	0.16	61,957	Democratic Republi	Maximum Estimate	2266 - Audiologists and speech therapists	Low Income	Central	Other Health Workers
61	Urologist	151	182	223	0.03	398,692	Democratic Republi	Maximum Estimate	2212 - Specialist medical practitioners	Low Income	Central	Medical Doctors
1	Anaesthesiologist	19	21	24	0.17	59,906	Equatorial Guinea	Maximum Estimate	2212 - Specialist medical practitioners	High Income and Upper Middle Income	West	Medical Doctors
2	Associate Nurse/Enrolled Nurse/Nursing Assistant	2,654	3,007	3,404	24.16	414	Equatorial Guinea	Maximum Estimate	3221 - Nursing associate professionals	High Income and Upper Middle Income	West	Nursing Personnel
3	Audiologist	8	9	10	0.07	146,461	Equatorial Guinea	Maximum Estimate	2266 - Audiologists and speech therapists	High Income and Upper Middle Income	West	Other Health Workers
4	Cardiologist	17	18	20	0.14	69,681	Equatorial Guinea	Maximum Estimate	2212 - Specialist medical practitioners	High Income and Upper Middle Income	West	Medical Doctors
5	Cardiothoracic Surgeon	3	3	4	0.03	369,101	Equatorial Guinea	Maximum Estimate	2212 - Specialist medical practitioners	High Income and Upper Middle Income	West	Medical Doctors
6	Clinical Officer/Physician Assistant	668	771	888	6.31	1,585	Equatorial Guinea	Maximum Estimate	3256 - Medical assistants	High Income and Upper Middle Income	West	Other Health Workers
7	Clinical Pharmacist	67	75	83	0.59	16,969	Equatorial Guinea	Maximum Estimate	2262 - Pharmacists	High Income and Upper Middle Income	West	Pharmacist
8	Clinical Psychologist	144	163	182	1.29	7,755	Equatorial Guinea	Maximum Estimate	2634 - Psychologists	High Income and Upper Middle Income	West	Other Health Workers
9	Community health worker/Village health worker	3,044	3,443	3,888	27.55	363	Equatorial Guinea	Maximum Estimate	3253 - Community health workers	High Income and Upper Middle Income	West	Other Health Workers
10	Dental Surgery Assistant	234	268	306	2.17	4,616	Equatorial Guinea	Maximum Estimate	3251 - Dental assistants and therapists	High Income and Upper Middle Income	West	Other Health Workers
11	Dental Therapist	157	179	205	1.45	6,898	Equatorial Guinea	Maximum Estimate	3251 - Dental assistants and therapists	High Income and Upper Middle Income	West	Other Health Workers
12	Dentist	202	229	263	1.86	5,363	Equatorial Guinea	Maximum Estimate	2261 - Dentists	High Income and Upper Middle Income	West	Dentist
13	Dermatologist	7	8	9	0.06	156,270	Equatorial Guinea	Maximum Estimate	2212 - Specialist medical practitioners	High Income and Upper Middle Income	West	Medical Doctors
14	Endocrinologist	11	13	15	0.11	94,080	Equatorial Guinea	Maximum Estimate	2212 - Specialist medical practitioners	High Income and Upper Middle Income	West	Medical Doctors
15	ENT Surgeon	23	26	29	0.21	47,973	Equatorial Guinea	Maximum Estimate	2212 - Specialist medical practitioners	High Income and Upper Middle Income	West	Medical Doctors
16	Environmental Health Officer	105	115	125	0.88	11,360	Equatorial Guinea	Maximum Estimate	2263 - Environmental and occupational health and hygiene professionals	High Income and Upper Middle Income	West	Other Health Workers
17	Gastroenterologist	12	13	14	0.10	102,978	Equatorial Guinea	Maximum Estimate	2212 - Specialist medical practitioners	High Income and Upper Middle Income	West	Medical Doctors
18	General Medical Practitioner (Generalist Doctor)	1,040	1,181	1,340	9.50	1,053	Equatorial Guinea	Maximum Estimate	2211 - Generalist medical practitioners	High Income and Upper Middle Income	West	Medical Doctors
19	General Surgeon	24	28	31	0.22	44,944	Equatorial Guinea	Maximum Estimate	2212 - Specialist medical practitioners	High Income and Upper Middle Income	West	Medical Doctors
20	Haematologist	7	7	7	0.05	193,306	Equatorial Guinea	Maximum Estimate	2212 - Specialist medical practitioners	High Income and Upper Middle Income	West	Medical Doctors
21	Health Promoter/Health Educator	53	61	70	0.50	20,140	Equatorial Guinea	Maximum Estimate	2269 - Health professionals not elsewhere classified	High Income and Upper Middle Income	West	Other Health Workers
22	Infectious Diseases Specialist	3	3	3	0.02	448,178	Equatorial Guinea	Maximum Estimate	2212 - Specialist medical practitioners	High Income and Upper Middle Income	West	Medical Doctors
23	Intensive Care Nurse	73	79	86	0.60	16,538	Equatorial Guinea	Maximum Estimate	2221 - Nursing professionals	High Income and Upper Middle Income	West	Nursing Personnel
24	Medical Laboratory Scientist	394	462	542	3.87	2,586	Equatorial Guinea	Maximum Estimate	3212 - Medical and pathology laboratory technicians	High Income and Upper Middle Income	West	Other Health Workers
25	Medical Laboratory Technician	660	769	895	6.36	1,573	Equatorial Guinea	Maximum Estimate	3212 - Medical and pathology laboratory technicians	High Income and Upper Middle Income	West	Other Health Workers
26	Medical Social Worker	46	48	49	0.35	28,783	Equatorial Guinea	Maximum Estimate	1344 - Social welfare managers	High Income and Upper Middle Income	West	Other Health Workers
27	Mental Health Nurse	198	225	254	1.81	5,534	Equatorial Guinea	Maximum Estimate	2221 - Nursing professionals	High Income and Upper Middle Income	West	Nursing Personnel
28	Midwife	1,455	1,581	1,722	12.17	822	Equatorial Guinea	Maximum Estimate	2222 - Midwifery professionals	High Income and Upper Middle Income	West	Midwifery Personnel
29	Nephrologist	22	26	30	0.21	46,878	Equatorial Guinea	Maximum Estimate	2212 - Specialist medical practitioners	High Income and Upper Middle Income	West	Medical Doctors
30	Neuro-Surgeon	7	8	8	0.06	166,191	Equatorial Guinea	Maximum Estimate	2212 - Specialist medical practitioners	High Income and Upper Middle Income	West	Medical Doctors
31	Nurse Anaesthetist	50	57	66	0.47	21,492	Equatorial Guinea	Maximum Estimate	2221 - Nursing professionals	High Income and Upper Middle Income	West	Nursing Personnel
32	Nutritionist	169	169	168	1.20	8,365	Equatorial Guinea	Maximum Estimate	2265 - Dieticians and nutritionists	High Income and Upper Middle Income	West	Other Health Workers
33	Obstetrician & Gynaecologist	144	160	177	1.25	7,971	Equatorial Guinea	Maximum Estimate	2212 - Specialist medical practitioners	High Income and Upper Middle Income	West	Medical Doctors
34	Occupational Therapist	38	43	48	0.34	29,215	Equatorial Guinea	Maximum Estimate	2269 - Health professionals not elsewhere classified	High Income and Upper Middle Income	West	Other Health Workers
35	Oncology Nurse	19	22	25	0.18	55,540	Equatorial Guinea	Maximum Estimate	2221 - Nursing professionals	High Income and Upper Middle Income	West	Nursing Personnel
36	Operating Theatre Nurse	237	270	304	2.16	4,623	Equatorial Guinea	Maximum Estimate	2221 - Nursing professionals	High Income and Upper Middle Income	West	Nursing Personnel
37	Ophthalmic Nurse	28	31	35	0.25	40,283	Equatorial Guinea	Maximum Estimate	2221 - Nursing professionals	High Income and Upper Middle Income	West	Nursing Personnel
38	Ophthalmologist	8	10	11	0.08	128,283	Equatorial Guinea	Maximum Estimate	2212 - Specialist medical practitioners	High Income and Upper Middle Income	West	Medical Doctors
39	Optometrist	26	30	33	0.24	42,393	Equatorial Guinea	Maximum Estimate	2267 - Optometrists and ophthalmic opticians	High Income and Upper Middle Income	West	Other Health Workers
40	Orthopaedic Nurse	41	47	52	0.37	27,126	Equatorial Guinea	Maximum Estimate	2221 - Nursing professionals	High Income and Upper Middle Income	West	Nursing Personnel
41	Orthopaedic Surgeon	86	101	117	0.83	12,020	Equatorial Guinea	Maximum Estimate	2212 - Specialist medical practitioners	High Income and Upper Middle Income	West	Medical Doctors
42	Orthopaedic Technologist	57	63	69	0.49	20,389	Equatorial Guinea	Maximum Estimate	3214 - Medical and dental prosthetic technicians	High Income and Upper Middle Income	West	Other Health Workers
43	Paediatric Nurse	201	213	222	1.57	6,370	Equatorial Guinea	Maximum Estimate	2221 - Nursing professionals	High Income and Upper Middle Income	West	Nursing Personnel
44	Paediatric Surgeon	6	7	7	0.05	210,126	Equatorial Guinea	Maximum Estimate	2212 - Specialist medical practitioners	High Income and Upper Middle Income	West	Medical Doctors
45	Paediatrician	100	109	118	0.84	11,972	Equatorial Guinea	Maximum Estimate	2212 - Specialist medical practitioners	High Income and Upper Middle Income	West	Medical Doctors
46	Pathologist	24	28	31	0.22	45,095	Equatorial Guinea	Maximum Estimate	2212 - Specialist medical practitioners	High Income and Upper Middle Income	West	Medical Doctors
47	Pharmacist	239	259	280	1.98	5,052	Equatorial Guinea	Maximum Estimate	2262 - Pharmacists	High Income and Upper Middle Income	West	Pharmacist
48	Pharmacy Technician	261	291	323	2.28	4,381	Equatorial Guinea	Maximum Estimate	3213 - Pharmaceutical technicians and assistants	High Income and Upper Middle Income	West	Other Health Workers

S/N	Health Professionals	2022	2026	2030	Density per 10,000 population	Required Population ratio (1 professional is to xxx population)	Name of Country	Modelling Scenario	ISCO-08 Match	Income Group Classification	Sub-Regional Grouping	SDG 3c Occupation
49	Physician	243	275	310	2.20	4,552	Equatorial Guinea	Maximum Estimate	2212 - Specialist medical practitioners	High Income and Upper Middle Income	West	Medical Doctors
50	Physiotherapist	52	58	63	0.45	22,296	Equatorial Guinea	Maximum Estimate	2264 - Physiotherapists	High Income and Upper Middle Income	West	Other Health Workers
51	Plastic Surgeon	12	14	16	0.11	87,093	Equatorial Guinea	Maximum Estimate	2212 - Specialist medical practitioners	High Income and Upper Middle Income	West	Medical Doctors
52	Psychiatrist	142	160	179	1.27	7,843	Equatorial Guinea	Maximum Estimate	2212 - Specialist medical practitioners	High Income and Upper Middle Income	West	Medical Doctors
53	Radiation Oncologist	7	9	10	0.07	137,968	Equatorial Guinea	Maximum Estimate	2212 - Specialist medical practitioners	High Income and Upper Middle Income	West	Medical Doctors
54	Radiographer (Diagnostics and Therapy)	173	198	224	1.59	6,290	Equatorial Guinea	Maximum Estimate	3211 - Medical imaging and therapeutic equipment technicians	High Income and Upper Middle Income	West	Other Health Workers
55	Radiologist	64	73	83	0.59	16,941	Equatorial Guinea	Maximum Estimate	2212 - Specialist medical practitioners	High Income and Upper Middle Income	West	Medical Doctors
56	Registered General Nurse / State Certified Nurse	4,654	5,191	5,762	40.90	244	Equatorial Guinea	Maximum Estimate	2221 - Nursing professionals	High Income and Upper Middle Income	West	Nursing Personnel
57	Renal Nurse	440	510	587	4.18	2,392	Equatorial Guinea	Maximum Estimate	2221 - Nursing professionals	High Income and Upper Middle Income	West	Nursing Personnel
58	Respiratory Physician	18	20	22	0.15	65,215	Equatorial Guinea	Maximum Estimate	2212 - Specialist medical practitioners	High Income and Upper Middle Income	West	Medical Doctors
59	Rheumatologist	6	6	7	0.05	206,038	Equatorial Guinea	Maximum Estimate	2212 - Specialist medical practitioners	High Income and Upper Middle Income	West	Medical Doctors
60	Speech Therapist	17	19	20	0.14	70,705	Equatorial Guinea	Maximum Estimate	2266 - Audiologists and speech therapists	High Income and Upper Middle Income	West	Other Health Workers
61	Urologist	3	4	5	0.03	307,137	Equatorial Guinea	Maximum Estimate	2212 - Specialist medical practitioners	High Income and Upper Middle Income	West	Medical Doctors
1	Anaesthesiologist	58	64	72	0.20	49,538	Eritrea	Maximum Estimate	2212 - Specialist medical practitioners	Low Income	East	Medical Doctors
2	Associate Nurse/Enrolled Nurse/Nursing Assistant	5,737	6,131	6,648	18.75	533	Eritrea	Maximum Estimate	3221 - Nursing associate professionals	Low Income	East	Nursing Personnel
3	Audiologist	25	28	30	0.09	117,458	Eritrea	Maximum Estimate	2266 - Audiologists and speech therapists	Low Income	East	Other Health Workers
4	Cardiologist	44	50	57	0.16	62,581	Eritrea	Maximum Estimate	2212 - Specialist medical practitioners	Low Income	East	Medical Doctors
5	Cardiothoracic Surgeon	12	14	15	0.04	232,306	Eritrea	Maximum Estimate	2212 - Specialist medical practitioners	Low Income	East	Medical Doctors
6	Clinical Officer/Physician Assistant	644	669	714	2.04	4,911	Eritrea	Maximum Estimate	3256 - Medical assistants	Low Income	East	Other Health Workers
7	Clinical Pharmacist	159	173	194	0.55	18,200	Eritrea	Maximum Estimate	2262 - Pharmacists	Low Income	East	Pharmacist
8	Clinical Psychologist	343	375	418	1.18	8,487	Eritrea	Maximum Estimate	2634 - Psychologists	Low Income	East	Other Health Workers
9	Community health worker/Village health worker	4,303	4,457	4,660	13.12	762	Eritrea	Maximum Estimate	3253 - Community health workers	Low Income	East	Other Health Workers
10	Dental Surgery Assistant	592	658	753	2.12	4,728	Eritrea	Maximum Estimate	3251 - Dental assistants and therapists	Low Income	East	Other Health Workers
11	Dental Therapist	398	442	505	1.42	7,041	Eritrea	Maximum Estimate	3251 - Dental assistants and therapists	Low Income	East	Other Health Workers
12	Dentist	571	633	741	2.08	4,800	Eritrea	Maximum Estimate	2261 - Dentists	Low Income	East	Dentist
13	Dermatologist	18	19	21	0.06	167,718	Eritrea	Maximum Estimate	2212 - Specialist medical practitioners	Low Income	East	Medical Doctors
14	Endocrinologist	17	20	24	0.07	141,747	Eritrea	Maximum Estimate	2212 - Specialist medical practitioners	Low Income	East	Medical Doctors
15	ENT Surgeon	71	79	88	0.25	40,566	Eritrea	Maximum Estimate	2212 - Specialist medical practitioners	Low Income	East	Medical Doctors
16	Environmental Health Officer	264	284	306	0.86	11,637	Eritrea	Maximum Estimate	2263 - Environmental and occupational health and hygiene professionals	Low Income	East	Other Health Workers
17	Gastroenterologist	41	42	44	0.13	79,413	Eritrea	Maximum Estimate	2212 - Specialist medical practitioners	Low Income	East	Medical Doctors
18	General Medical Practitioner (Generalist Doctor)	1,846	2,014	2,244	6.37	1,570	Eritrea	Maximum Estimate	2211 - Generalist medical practitioners	Low Income	East	Medical Doctors
19	General Surgeon	44	53	67	0.19	51,905	Eritrea	Maximum Estimate	2212 - Specialist medical practitioners	Low Income	East	Medical Doctors
20	Haematologist	20	21	23	0.06	156,659	Eritrea	Maximum Estimate	2212 - Specialist medical practitioners	Low Income	East	Medical Doctors
21	Health Promoter/Health Educator	71	73	77	0.22	46,051	Eritrea	Maximum Estimate	2269 - Health professionals not elsewhere classified	Low Income	East	Other Health Workers
22	Infectious Diseases Specialist	6	7	7	0.02	487,122	Eritrea	Maximum Estimate	2212 - Specialist medical practitioners	Low Income	East	Medical Doctors
23	Intensive Care Nurse	192	203	217	0.61	16,425	Eritrea	Maximum Estimate	2221 - Nursing professionals	Low Income	East	Nursing Personnel
24	Medical Laboratory Scientist	531	577	643	1.82	5,482	Eritrea	Maximum Estimate	3212 - Medical and pathology laboratory technicians	Low Income	East	Other Health Workers
25	Medical Laboratory Technician	470	508	559	1.58	6,327	Eritrea	Maximum Estimate	3212 - Medical and pathology laboratory technicians	Low Income	East	Other Health Workers
26	Medical Social Worker	178	180	185	0.52	19,057	Eritrea	Maximum Estimate	1344 - Social welfare managers	Low Income	East	Other Health Workers
27	Mental Health Nurse	397	438	505	1.44	6,933	Eritrea	Maximum Estimate	2221 - Nursing professionals	Low Income	East	Nursing Personnel
28	Midwife	3,567	3,809	4,185	11.76	850	Eritrea	Maximum Estimate	2222 - Midwifery professionals	Low Income	East	Midwifery Personnel
29	Nephrologist	47	53	63	0.18	54,901	Eritrea	Maximum Estimate	2212 - Specialist medical practitioners	Low Income	East	Medical Doctors
30	Neuro-Surgeon	19	21	24	0.07	147,413	Eritrea	Maximum Estimate	2212 - Specialist medical practitioners	Low Income	East	Medical Doctors
31	Nurse Anaesthetist	139	161	188	0.53	18,885	Eritrea	Maximum Estimate	2221 - Nursing professionals	Low Income	East	Nursing Personnel
32	Nutritionist	790	791	797	2.25	4,449	Eritrea	Maximum Estimate	2265 - Dieticians and nutritionists	Low Income	East	Other Health Workers
33	Obstetrician & Gynaecologist	336	367	406	1.14	8,737	Eritrea	Maximum Estimate	2212 - Specialist medical practitioners	Low Income	East	Medical Doctors
34	Occupational Therapist	99	111	127	0.36	27,927	Eritrea	Maximum Estimate	2269 - Health professionals not elsewhere classified	Low Income	East	Other Health Workers
35	Oncology Nurse	44	51	61	0.17	57,524	Eritrea	Maximum Estimate	2221 - Nursing professionals	Low Income	East	Nursing Personnel
36	Operating Theatre Nurse	507	570	659	1.88	5,321	Eritrea	Maximum Estimate	2221 - Nursing professionals	Low Income	East	Nursing Personnel
37	Ophthalmic Nurse	57	63	71	0.20	49,904	Eritrea	Maximum Estimate	2221 - Nursing professionals	Low Income	East	Nursing Personnel
38	Ophthalmologist	22	25	28	0.08	125,593	Eritrea	Maximum Estimate	2212 - Specialist medical practitioners	Low Income	East	Medical Doctors
39	Optometrist	91	103	119	0.33	29,925	Eritrea	Maximum Estimate	2267 - Optometrists and ophthalmic opticians	Low Income	East	Other Health Workers
40	Orthopaedic Nurse	117	131	149	0.42	24,001	Eritrea	Maximum Estimate	2221 - Nursing professionals	Low Income	East	Nursing Personnel
41	Orthopaedic Surgeon	156	218	317	0.95	10,478	Eritrea	Maximum Estimate	2212 - Specialist medical practitioners	Low Income	East	Medical Doctors
42	Orthopaedic Technologist	162	177	195	0.55	18,313	Eritrea	Maximum Estimate	3214 - Medical and dental prosthetic technicians	Low Income	East	Other Health Workers
43	Paediatric Nurse	618	648	679	1.91	5,233	Eritrea	Maximum Estimate	2221 - Nursing professionals	Low Income	East	Nursing Personnel
44	Paediatric Surgeon	17	18	19	0.05	187,729	Eritrea	Maximum Estimate	2212 - Specialist medical practitioners	Low Income	East	Medical Doctors
45	Paediatrician	266	287	314	0.89	11,291	Eritrea	Maximum Estimate	2212 - Specialist medical practitioners	Low Income	East	Medical Doctors
46	Pathologist	51	56	64	0.18	54,552	Eritrea	Maximum Estimate	2212 - Specialist medical practitioners	Low Income	East	Medical Doctors
47	Pharmacist	411	421	438	1.24	8,079	Eritrea	Maximum Estimate	2262 - Pharmacists	Low Income	East	Pharmacist
48	Pharmacy Technician	557	603	663	1.87	5,357	Eritrea	Maximum Estimate	3213 - Pharmaceutical technicians and assistants	Low Income	East	Other Health Workers
49	Physician	503	550	610	1.72	5,801	Eritrea	Maximum Estimate	2212 - Specialist medical practitioners	Low Income	East	Medical Doctors
50	Physiotherapist	140	153	169	0.47	21,054	Eritrea	Maximum Estimate	2264 - Physiotherapists	Low Income	East	Other Health Workers
51	Plastic Surgeon	86	95	106	0.30	33,775	Eritrea	Maximum Estimate	2212 - Specialist medical practitioners	Low Income	East	Medical Doctors
52	Psychiatrist	300	325	366	1.04	9,614	Eritrea	Maximum Estimate	2212 - Specialist medical practitioners	Low Income	East	Medical Doctors
53	Radiation Oncologist	18	21	25	0.07	142,322	Eritrea	Maximum Estimate	2212 - Specialist medical practitioners	Low Income	East	Medical Doctors
54	Radiographer (Diagnostics and Therapy)	370	422	499	1.43	6,998	Eritrea	Maximum Estimate	3211 - Medical imaging and therapeutic equipment technicians	Low Income	East	Other Health Workers
55	Radiologist	144	166	199	0.57	17,511	Eritrea	Maximum Estimate	2212 - Specialist medical practitioners	Low Income	East	Medical Doctors
56	Registered General Nurse / State Certified Nurse	15,637	16,225	17,156	48.73	205	Eritrea	Maximum Estimate	2221 - Nursing professionals	Low Income	East	Nursing Personnel
57	Renal Nurse	910	1,040	1,240	3.57	2,802	Eritrea	Maximum Estimate	2221 - Nursing professionals	Low Income	East	Nursing Personnel
58	Respiratory Physician	60	65	71	0.20	50,096	Eritrea	Maximum Estimate	2212 - Specialist medical practitioners	Low Income	East	Medical Doctors
59	Rheumatologist	14	15	17	0.05	210,480	Eritrea	Maximum Estimate	2212 - Specialist medical practitioners	Low Income	East	Medical Doctors
60	Speech Therapist	50	54	58	0.16	61,272	Eritrea	Maximum Estimate	2266 - Audiologists and speech therapists	Low Income	East	Other Health Workers
61	Urologist	7	8	9	0.03	377,258	Eritrea	Maximum Estimate	2212 - Specialist medical practitioners	Low Income	East	Medical Doctors
1	Anaesthesiologist	23	25	27	0.24	42,481	Eswatini	Maximum Estimate	2212 - Specialist medical practitioners	Lower-middle Income	Southern	Medical Doctors
2	Associate Nurse/Enrolled Nurse/Nursing Assistant	2,406	2,525	2,702	23.39	428	Eswatini	Maximum Estimate	3221 - Nursing associate professionals	Lower-middle Income	Southern	Nursing Personnel
3	Audiologist	8	9	9	0.08	127,020	Eswatini	Maximum Estimate	2266 - Audiologists and speech therapists	Lower-middle Income	Southern	Other Health Workers
4	Cardiologist	21	24	26	0.23	44,117	Eswatini	Maximum Estimate	2212 - Specialist medical practitioners	Lower-middle Income	Southern	Medical Doctors
5	Cardiothoracic Surgeon	3	4	4	0.03	298,387	Eswatini	Maximum Estimate	2212 - Specialist medical practitioners	Lower-middle Income	Southern	Medical Doctors
6	Clinical Officer/Physician Assistant	343	353	376	3.27	3,054	Eswatini	Maximum Estimate	3256 - Medical assistants	Lower-middle Income	Southern	Other Health Workers
7	Clinical Pharmacist	55	59	65	0.57	17,451	Eswatini	Maximum Estimate	2262 - Pharmacists	Lower-middle Income	Southern	Pharmacist
8	Clinical Psychologist	94	98	106	0.92	10,923	Eswatini	Maximum Estimate	2634 - Psychologists	Lower-middle Income	Southern	Other Health Workers
9	Community health worker/Village health worker	1,171	1,185	1,215	10.53	950	Eswatini	Maximum Estimate	3253 - Community health workers	Lower-middle Income	Southern	Other Health Workers
10	Dental Surgery Assistant	232	248	273	2.35	4,257	Eswatini	Maximum Estimate	3251 - Dental assistants and therapists	Lower-middle Income	Southern	Other Health Workers

S/N	Health Professionals	2022	2026	2030	Density per 10,000 population	Required Population ratio (1 professional is to xxx population)	Name of Country	Modelling Scenario	ISCO-08 Match	Income Group Classification	Sub-Regional Grouping	SDG 3c Occupation
11	Dental Therapist	155	167	183	1.57	6,354	Eswatini	Maximum Estimate	3251 - Dental assistants and therapists	Lower-middle Income	Southern	Other Health Workers
12	Dentist	220	231	254	2.19	4,574	Eswatini	Maximum Estimate	2261 - Dentists	Lower-middle Income	Southern	Dentist
13	Dermatologist	6	7	7	0.06	163,064	Eswatini	Maximum Estimate	2212 - Specialist medical practitioners	Lower-middle Income	Southern	Medical Doctors
14	Endocrinologist	10	11	13	0.11	87,827	Eswatini	Maximum Estimate	2212 - Specialist medical practitioners	Lower-middle Income	Southern	Medical Doctors
15	ENT Surgeon	26	28	31	0.26	37,988	Eswatini	Maximum Estimate	2212 - Specialist medical practitioners	Lower-middle Income	Southern	Medical Doctors
16	Environmental Health Officer	86	90	94	0.81	12,381	Eswatini	Maximum Estimate	2263 - Environmental and occupational health and hygiene professionals	Lower-middle Income	Southern	Other Health Workers
17	Gastroenterologist	15	14	14	0.13	79,059	Eswatini	Maximum Estimate	2212 - Specialist medical practitioners	Lower-middle Income	Southern	Medical Doctors
18	General Medical Practitioner (Generalist Doctor)	784	823	880	7.62	1,312	Eswatini	Maximum Estimate	2211 - Generalist medical practitioners	Lower-middle Income	Southern	Medical Doctors
19	General Surgeon	25	27	30	0.26	38,889	Eswatini	Maximum Estimate	2212 - Specialist medical practitioners	Lower-middle Income	Southern	Medical Doctors
20	Haematologist	4	4	4	0.03	290,701	Eswatini	Maximum Estimate	2212 - Specialist medical practitioners	Lower-middle Income	Southern	Medical Doctors
21	Health Promoter/Health Educator	32	33	35	0.30	33,113	Eswatini	Maximum Estimate	2269 - Health professionals not elsewhere classified	Lower-middle Income	Southern	Other Health Workers
22	Infectious Diseases Specialist	3	3	3	0.02	405,448	Eswatini	Maximum Estimate	2212 - Specialist medical practitioners	Lower-middle Income	Southern	Medical Doctors
23	Intensive Care Nurse	50	53	56	0.48	20,769	Eswatini	Maximum Estimate	2221 - Nursing professionals	Lower-middle Income	Southern	Nursing Personnel
24	Medical Laboratory Scientist	334	357	394	3.43	2,912	Eswatini	Maximum Estimate	3212 - Medical and pathology laboratory technicians	Lower-middle Income	Southern	Other Health Workers
25	Medical Laboratory Technician	275	294	323	2.81	3,564	Eswatini	Maximum Estimate	3212 - Medical and pathology laboratory technicians	Lower-middle Income	Southern	Other Health Workers
26	Medical Social Worker	39	41	46	0.42	23,658	Eswatini	Maximum Estimate	1344 - Social welfare managers	Lower-middle Income	Southern	Other Health Workers
27	Mental Health Nurse	129	136	153	1.33	7,513	Eswatini	Maximum Estimate	2221 - Nursing professionals	Lower-middle Income	Southern	Nursing Personnel
28	Midwife	1,194	1,236	1,309	11.28	887	Eswatini	Maximum Estimate	2222 - Midwifery professionals	Lower-middle Income	Southern	Midwifery Personnel
29	Nephrologist	27	30	37	0.32	31,168	Eswatini	Maximum Estimate	2212 - Specialist medical practitioners	Lower-middle Income	Southern	Medical Doctors
30	Neuro-Surgeon	8	9	9	0.08	124,726	Eswatini	Maximum Estimate	2212 - Specialist medical practitioners	Lower-middle Income	Southern	Medical Doctors
31	Nurse Anaesthetist	67	74	84	0.73	13,792	Eswatini	Maximum Estimate	2221 - Nursing professionals	Lower-middle Income	Southern	Nursing Personnel
32	Nutritionist	98	98	99	0.86	11,668	Eswatini	Maximum Estimate	2265 - Dieticians and nutritionists	Lower-middle Income	Southern	Other Health Workers
33	Obstetrician & Gynaecologist	139	150	168	1.47	6,784	Eswatini	Maximum Estimate	2212 - Specialist medical practitioners	Lower-middle Income	Southern	Medical Doctors
34	Occupational Therapist	35	38	43	0.37	26,839	Eswatini	Maximum Estimate	2269 - Health professionals not elsewhere classified	Lower-middle Income	Southern	Other Health Workers
35	Oncology Nurse	23	26	31	0.27	37,277	Eswatini	Maximum Estimate	2221 - Nursing professionals	Lower-middle Income	Southern	Nursing Personnel
36	Operating Theatre Nurse	231	253	290	2.52	3,968	Eswatini	Maximum Estimate	2221 - Nursing professionals	Lower-middle Income	Southern	Nursing Personnel
37	Ophthalmic Nurse	26	28	31	0.27	37,238	Eswatini	Maximum Estimate	2221 - Nursing professionals	Lower-middle Income	Southern	Nursing Personnel
38	Ophthalmologist	11	12	14	0.12	83,526	Eswatini	Maximum Estimate	2212 - Specialist medical practitioners	Lower-middle Income	Southern	Medical Doctors
39	Optometrist	59	65	73	0.63	15,846	Eswatini	Maximum Estimate	2267 - Optometrists and ophthalmic opticians	Lower-middle Income	Southern	Other Health Workers
40	Orthopaedic Nurse	38	42	46	0.40	24,975	Eswatini	Maximum Estimate	2221 - Nursing professionals	Lower-middle Income	Southern	Nursing Personnel
41	Orthopaedic Surgeon	75	84	96	0.83	12,065	Eswatini	Maximum Estimate	2212 - Specialist medical practitioners	Lower-middle Income	Southern	Medical Doctors
42	Orthopaedic Technologist	51	54	57	0.49	20,324	Eswatini	Maximum Estimate	3214 - Medical and dental prosthetic technicians	Lower-middle Income	Southern	Other Health Workers
43	Paediatric Nurse	136	138	139	1.21	8,273	Eswatini	Maximum Estimate	2221 - Nursing professionals	Lower-middle Income	Southern	Nursing Personnel
44	Paediatric Surgeon	5	5	5	0.05	222,211	Eswatini	Maximum Estimate	2212 - Specialist medical practitioners	Lower-middle Income	Southern	Medical Doctors
45	Paediatrician	82	85	92	0.80	12,561	Eswatini	Maximum Estimate	2212 - Specialist medical practitioners	Lower-middle Income	Southern	Medical Doctors
46	Pathologist	23	25	29	0.25	39,245	Eswatini	Maximum Estimate	2212 - Specialist medical practitioners	Lower-middle Income	Southern	Medical Doctors
47	Pharmacist	175	174	178	1.56	6,423	Eswatini	Maximum Estimate	2262 - Pharmacists	Lower-middle Income	Southern	Pharmacist
48	Pharmacy Technician	218	228	243	2.11	4,744	Eswatini	Maximum Estimate	3213 - Pharmaceutical technicians and assistants	Lower-middle Income	Southern	Other Health Workers
49	Physician	194	207	226	1.96	5,101	Eswatini	Maximum Estimate	2212 - Specialist medical practitioners	Lower-middle Income	Southern	Medical Doctors
50	Physiotherapist	47	50	54	0.46	21,597	Eswatini	Maximum Estimate	2264 - Physiotherapists	Lower-middle Income	Southern	Other Health Workers
51	Plastic Surgeon	17	17	18	0.16	63,988	Eswatini	Maximum Estimate	2212 - Specialist medical practitioners	Lower-middle Income	Southern	Medical Doctors
52	Psychiatrist	92	97	108	0.94	10,648	Eswatini	Maximum Estimate	2212 - Specialist medical practitioners	Lower-middle Income	Southern	Medical Doctors
53	Radiation Oncologist	9	10	12	0.10	96,120	Eswatini	Maximum Estimate	2212 - Specialist medical practitioners	Lower-middle Income	Southern	Medical Doctors
54	Radiographer (Diagnostics and Therapy)	178	195	224	1.97	5,086	Eswatini	Maximum Estimate	3211 - Medical imaging and therapeutic equipment technicians	Lower-middle Income	Southern	Other Health Workers
55	Radiologist	67	76	90	0.81	12,418	Eswatini	Maximum Estimate	2212 - Specialist medical practitioners	Lower-middle Income	Southern	Medical Doctors
56	Registered General Nurse / State Certified Nurse	4,829	5,047	5,479	47.83	209	Eswatini	Maximum Estimate	2221 - Nursing professionals	Lower-middle Income	Southern	Nursing Personnel
57	Renal Nurse	517	588	712	6.24	1,601	Eswatini	Maximum Estimate	2221 - Nursing professionals	Lower-middle Income	Southern	Nursing Personnel
58	Respiratory Physician	18	18	19	0.17	60,406	Eswatini	Maximum Estimate	2212 - Specialist medical practitioners	Lower-middle Income	Southern	Medical Doctors
59	Rheumatologist	5	6	6	0.05	184,708	Eswatini	Maximum Estimate	2212 - Specialist medical practitioners	Lower-middle Income	Southern	Medical Doctors
60	Speech Therapist	16	17	18	0.15	65,546	Eswatini	Maximum Estimate	2266 - Audiologists and speech therapists	Lower-middle Income	Southern	Other Health Workers
61	Urologist	3	4	5	0.04	231,124	Eswatini	Maximum Estimate	2212 - Specialist medical practitioners	Lower-middle Income	Southern	Medical Doctors
1	Anaesthesiologist	1,393	1,573	1,778	0.15	65,244	Ethiopia	Maximum Estimate	2212 - Specialist medical practitioners	Low Income	East	Medical Doctors
2	Associate Nurse/Enrolled Nurse/Nursing Assistant	179,205	197,812	218,653	18.86	530	Ethiopia	Maximum Estimate	3221 - Nursing associate professionals	Low Income	East	Nursing Personnel
3	Audiologist	831	921	1,014	0.09	114,347	Ethiopia	Maximum Estimate	2266 - Audiologists and speech therapists	Low Income	East	Other Health Workers
4	Cardiologist	1,296	1,489	1,699	0.15	68,344	Ethiopia	Maximum Estimate	2212 - Specialist medical practitioners	Low Income	East	Medical Doctors
5	Cardiothoracic Surgeon	216	237	257	0.02	452,341	Ethiopia	Maximum Estimate	2212 - Specialist medical practitioners	Low Income	East	Medical Doctors
6	Clinical Officer/Physician Assistant	45,504	51,643	58,911	5.09	1,963	Ethiopia	Maximum Estimate	3256 - Medical assistants	Low Income	East	Other Health Workers
7	Clinical Pharmacist	4,207	4,679	5,284	0.46	21,899	Ethiopia	Maximum Estimate	2262 - Pharmacists	Low Income	East	Pharmacist
8	Clinical Psychologist	9,008	9,937	11,060	0.95	10,484	Ethiopia	Maximum Estimate	2634 - Psychologists	Low Income	East	Other Health Workers
9	Community health worker/Village health worker	170,118	188,013	206,900	17.84	561	Ethiopia	Maximum Estimate	3253 - Community health workers	Low Income	East	Other Health Workers
10	Dental Surgery Assistant	15,791	17,864	20,213	1.74	5,740	Ethiopia	Maximum Estimate	3251 - Dental assistants and therapists	Low Income	East	Other Health Workers
11	Dental Therapist	10,607	11,992	13,560	1.17	8,556	Ethiopia	Maximum Estimate	3251 - Dental assistants and therapists	Low Income	East	Other Health Workers
12	Dentist	14,043	15,598	17,522	1.51	6,613	Ethiopia	Maximum Estimate	2261 - Dentists	Low Income	East	Dentist
13	Dermatologist	552	618	688	0.06	168,840	Ethiopia	Maximum Estimate	2212 - Specialist medical practitioners	Low Income	East	Medical Doctors
14	Endocrinologist	374	433	524	0.05	220,075	Ethiopia	Maximum Estimate	2212 - Specialist medical practitioners	Low Income	East	Medical Doctors
15	ENT Surgeon	2,280	2,602	2,967	0.26	39,099	Ethiopia	Maximum Estimate	2212 - Specialist medical practitioners	Low Income	East	Medical Doctors
16	Environmental Health Officer	8,565	9,449	10,358	0.89	11,218	Ethiopia	Maximum Estimate	2263 - Environmental and occupational health and hygiene professionals	Low Income	East	Other Health Workers
17	Gastroenterologist	1,289	1,362	1,497	0.13	77,249	Ethiopia	Maximum Estimate	2212 - Specialist medical practitioners	Low Income	East	Medical Doctors
18	General Medical Practitioner (Generalist Doctor)	66,113	74,460	83,955	7.25	1,380	Ethiopia	Maximum Estimate	2211 - Generalist medical practitioners	Low Income	East	Medical Doctors
19	General Surgeon	1,177	1,345	1,559	0.14	74,059	Ethiopia	Maximum Estimate	2212 - Specialist medical practitioners	Low Income	East	Medical Doctors
20	Haematologist	382	413	444	0.04	261,430	Ethiopia	Maximum Estimate	2212 - Specialist medical practitioners	Low Income	East	Medical Doctors
21	Health Promoter/Health Educator	4,783	5,345	5,973	0.52	19,412	Ethiopia	Maximum Estimate	2269 - Health professionals not elsewhere classified	Low Income	East	Other Health Workers
22	Infectious Diseases Specialist	146	157	170	0.01	682,320	Ethiopia	Maximum Estimate	2212 - Specialist medical practitioners	Low Income	East	Medical Doctors
23	Intensive Care Nurse	5,390	5,851	6,318	0.54	18,373	Ethiopia	Maximum Estimate	2221 - Nursing professionals	Low Income	East	Nursing Personnel
24	Medical Laboratory Scientist	16,716	18,988	21,936	1.90	5,264	Ethiopia	Maximum Estimate	3212 - Medical and pathology laboratory technicians	Low Income	East	Other Health Workers
25	Medical Laboratory Technician	18,523	21,284	24,688	2.14	4,674	Ethiopia	Maximum Estimate	3212 - Medical and pathology laboratory technicians	Low Income	East	Other Health Workers
26	Medical Social Worker	6,113	6,562	7,108	0.61	16,261	Ethiopia	Maximum Estimate	1344 - Social welfare managers	Low Income	East	Other Health Workers
27	Mental Health Nurse	11,584	13,092	15,318	1.33	7,544	Ethiopia	Maximum Estimate	2221 - Nursing professionals	Low Income	East	Nursing Personnel
28	Midwife	114,605	125,525	137,828	11.88	842	Ethiopia	Maximum Estimate	2222 - Midwifery professionals	Low Income	East	Midwifery Personnel
29	Nephrologist	1,401	1,624	1,963	0.17	58,766	Ethiopia	Maximum Estimate	2212 - Specialist medical practitioners	Low Income	East	Medical Doctors
30	Neuro-Surgeon	405	469	552	0.05	208,692	Ethiopia	Maximum Estimate	2212 - Specialist medical practitioners	Low Income	East	Medical Doctors
31	Nurse Anaesthetist	3,028	3,478	3,997	0.34	29,017	Ethiopia	Maximum Estimate	2221 - Nursing professionals	Low Income	East	Nursing Personnel
32	Nutritionist	26,711	28,015	29,176	2.52	3,974	Ethiopia	Maximum Estimate	2265 - Dieticians and nutritionists	Low Income	East	Other Health Workers
33	Obstetrician & Gynaecologist	10,528	12,062	13,958	1.21	8,267	Ethiopia	Maximum Estimate	2212 - Specialist medical practitioners	Low Income	East	Medical Doctors

S/N	Health Professionals	2022	2026	2030	Density per 10,000 population	Required Population ratio (1 professional is to xxx population)	Name of Country	Modelling Scenario	ISCO-08 Match	Income Group Classification	Sub-Regional Grouping	SDG 3c Occupation
34	Occupational Therapist	2,375	2,713	3,145	0.27	36,817	Ethiopia	Maximum Estimate	2269 - Health professionals not elsewhere classified	Low Income	East	Other Health Workers
35	Oncology Nurse	1,223	1,420	1,690	0.15	68,346	Ethiopia	Maximum Estimate	2221 - Nursing professionals	Low Income	East	Nursing Personnel
36	Operating Theatre Nurse	15,708	18,039	21,397	1.85	5,398	Ethiopia	Maximum Estimate	2221 - Nursing professionals	Low Income	East	Nursing Personnel
37	Ophthalmic Nurse	2,042	2,274	2,540	0.22	45,681	Ethiopia	Maximum Estimate	2221 - Nursing professionals	Low Income	East	Nursing Personnel
38	Ophthalmologist	627	707	806	0.07	143,847	Ethiopia	Maximum Estimate	2212 - Specialist medical practitioners	Low Income	East	Medical Doctors
39	Optometrist	1,875	2,109	2,376	0.20	48,848	Ethiopia	Maximum Estimate	2267 - Optometrists and ophthalmic opticians	Low Income	East	Other Health Workers
40	Orthopaedic Nurse	2,196	2,530	2,899	0.25	40,016	Ethiopia	Maximum Estimate	2221 - Nursing professionals	Low Income	East	Nursing Personnel
41	Orthopaedic Surgeon	4,968	6,004	7,308	0.63	15,779	Ethiopia	Maximum Estimate	2212 - Specialist medical practitioners	Low Income	East	Medical Doctors
42	Orthopaedic Technologist	4,358	4,840	5,345	0.46	21,730	Ethiopia	Maximum Estimate	3214 - Medical and dental prosthetic technicians	Low Income	East	Other Health Workers
43	Paediatric Nurse	18,063	19,535	20,791	1.79	5,596	Ethiopia	Maximum Estimate	2221 - Nursing professionals	Low Income	East	Nursing Personnel
44	Paediatric Surgeon	618	678	733	0.06	158,746	Ethiopia	Maximum Estimate	2212 - Specialist medical practitioners	Low Income	East	Medical Doctors
45	Paediatrician	6,807	7,572	8,477	0.73	13,683	Ethiopia	Maximum Estimate	2212 - Specialist medical practitioners	Low Income	East	Medical Doctors
46	Pathologist	1,393	1,746	2,285	0.20	49,810	Ethiopia	Maximum Estimate	2212 - Specialist medical practitioners	Low Income	East	Medical Doctors
47	Pharmacist	29,395	32,841	36,547	3.15	3,177	Ethiopia	Maximum Estimate	2262 - Pharmacists	Low Income	East	Pharmacist
48	Pharmacy Technician	20,920	23,415	26,163	2.25	4,436	Ethiopia	Maximum Estimate	3213 - Pharmaceutical technicians and assistants	Low Income	East	Other Health Workers
49	Physician	13,460	15,022	16,894	1.46	6,860	Ethiopia	Maximum Estimate	2212 - Specialist medical practitioners	Low Income	East	Medical Doctors
50	Physiotherapist	4,039	4,506	5,037	0.43	23,033	Ethiopia	Maximum Estimate	2264 - Physiotherapists	Low Income	East	Other Health Workers
51	Plastic Surgeon	1,797	2,058	2,342	0.20	49,586	Ethiopia	Maximum Estimate	2212 - Specialist medical practitioners	Low Income	East	Medical Doctors
52	Psychiatrist	9,422	10,460	11,927	1.03	9,696	Ethiopia	Maximum Estimate	2212 - Specialist medical practitioners	Low Income	East	Medical Doctors
53	Radiation Oncologist	404	479	574	0.05	200,792	Ethiopia	Maximum Estimate	2212 - Specialist medical practitioners	Low Income	East	Medical Doctors
54	Radiographer (Diagnostics and Therapy)	10,895	12,532	14,721	1.27	7,847	Ethiopia	Maximum Estimate	3211 - Medical imaging and therapeutic equipment technicians	Low Income	East	Other Health Workers
55	Radiologist	4,170	4,877	5,784	0.50	19,900	Ethiopia	Maximum Estimate	2212 - Specialist medical practitioners	Low Income	East	Medical Doctors
56	Registered General Nurse / State Certified Nurse	708,978	772,703	842,845	72.67	138	Ethiopia	Maximum Estimate	2221 - Nursing professionals	Low Income	East	Nursing Personnel
57	Renal Nurse	27,553	31,947	38,651	3.35	2,985	Ethiopia	Maximum Estimate	2221 - Nursing professionals	Low Income	East	Nursing Personnel
58	Respiratory Physician	1,435	1,602	1,782	0.15	65,179	Ethiopia	Maximum Estimate	2212 - Specialist medical practitioners	Low Income	East	Medical Doctors
59	Rheumatologist	431	488	551	0.05	210,750	Ethiopia	Maximum Estimate	2212 - Specialist medical practitioners	Low Income	East	Medical Doctors
60	Speech Therapist	1,532	1,706	1,899	0.16	61,110	Ethiopia	Maximum Estimate	2266 - Audiologists and speech therapists	Low Income	East	Other Health Workers
61	Urologist	246	296	370	0.03	308,062	Ethiopia	Maximum Estimate	2212 - Specialist medical practitioners	Low Income	East	Medical Doctors
1	Anaesthesiologist	43	47	53	0.24	42,501	Gabon	Maximum Estimate	2212 - Specialist medical practitioners	High Income and Upper Middle Income	Central	Medical Doctors
2	Associate Nurse/Enrolled Nurse/Nursing Assistant	4,750	5,223	5,720	25.55	391	Gabon	Maximum Estimate	3221 - Nursing associate professionals	High Income and Upper Middle Income	Central	Nursing Personnel
3	Audiologist	14	15	17	0.08	132,934	Gabon	Maximum Estimate	2266 - Audiologists and speech therapists	High Income and Upper Middle Income	Central	Other Health Workers
4	Cardiologist	42	47	53	0.24	42,539	Gabon	Maximum Estimate	2212 - Specialist medical practitioners	High Income and Upper Middle Income	Central	Medical Doctors
5	Cardiothoracic Surgeon	8	9	10	0.04	232,344	Gabon	Maximum Estimate	2212 - Specialist medical practitioners	High Income and Upper Middle Income	Central	Medical Doctors
6	Clinical Officer/Physician Assistant	1,204	1,344	1,504	6.75	1,481	Gabon	Maximum Estimate	3256 - Medical assistants	High Income and Upper Middle Income	Central	Other Health Workers
7	Clinical Pharmacist	126	138	152	0.68	14,678	Gabon	Maximum Estimate	2262 - Pharmacists	High Income and Upper Middle Income	Central	Pharmacist
8	Clinical Psychologist	249	273	298	1.33	7,516	Gabon	Maximum Estimate	2634 - Psychologists	High Income and Upper Middle Income	Central	Other Health Workers
9	Community health worker/Village health worker	4,582	5,295	6,118	27.46	364	Gabon	Maximum Estimate	3253 - Community health workers	High Income and Upper Middle Income	Central	Other Health Workers
10	Dental Surgery Assistant	400	444	486	2.17	4,604	Gabon	Maximum Estimate	3251 - Dental assistants and therapists	High Income and Upper Middle Income	Central	Other Health Workers
11	Dental Therapist	267	297	324	1.45	6,898	Gabon	Maximum Estimate	3251 - Dental assistants and therapists	High Income and Upper Middle Income	Central	Other Health Workers
12	Dentist	303	331	355	1.59	6,286	Gabon	Maximum Estimate	2261 - Dentists	High Income and Upper Middle Income	Central	Dentist
13	Dermatologist	12	13	15	0.06	154,579	Gabon	Maximum Estimate	2212 - Specialist medical practitioners	High Income and Upper Middle Income	Central	Medical Doctors
14	Endocrinologist	26	31	36	0.16	62,406	Gabon	Maximum Estimate	2212 - Specialist medical practitioners	High Income and Upper Middle Income	Central	Medical Doctors
15	ENT Surgeon	47	52	58	0.26	38,284	Gabon	Maximum Estimate	2212 - Specialist medical practitioners	High Income and Upper Middle Income	Central	Medical Doctors
16	Environmental Health Officer	166	179	193	0.86	11,655	Gabon	Maximum Estimate	2263 - Environmental and occupational health and hygiene professionals	High Income and Upper Middle Income	Central	Other Health Workers
17	Gastroenterologist	40	41	41	0.19	53,935	Gabon	Maximum Estimate	2212 - Specialist medical practitioners	High Income and Upper Middle Income	Central	Medical Doctors
18	General Medical Practitioner (Generalist Doctor)	1,754	1,935	2,126	9.51	1,052	Gabon	Maximum Estimate	2211 - Generalist medical practitioners	High Income and Upper Middle Income	Central	Medical Doctors
19	General Surgeon	45	52	59	0.26	38,044	Gabon	Maximum Estimate	2212 - Specialist medical practitioners	High Income and Upper Middle Income	Central	Medical Doctors
20	Haematologist	14	15	16	0.07	143,637	Gabon	Maximum Estimate	2212 - Specialist medical practitioners	High Income and Upper Middle Income	Central	Medical Doctors
21	Health Promoter/Health Educator	103	114	126	0.57	17,642	Gabon	Maximum Estimate	2269 - Health professionals not elsewhere classified	High Income and Upper Middle Income	Central	Other Health Workers
22	Infectious Diseases Specialist	6	6	6	0.03	363,237	Gabon	Maximum Estimate	2212 - Specialist medical practitioners	High Income and Upper Middle Income	Central	Medical Doctors
23	Intensive Care Nurse	121	130	140	0.62	16,022	Gabon	Maximum Estimate	2221 - Nursing professionals	High Income and Upper Middle Income	Central	Nursing Personnel
24	Medical Laboratory Scientist	637	720	814	3.65	2,740	Gabon	Maximum Estimate	3212 - Medical and pathology laboratory technicians	High Income and Upper Middle Income	Central	Other Health Workers
25	Medical Laboratory Technician	949	1,135	1,358	6.10	1,639	Gabon	Maximum Estimate	3212 - Medical and pathology laboratory technicians	High Income and Upper Middle Income	Central	Other Health Workers
26	Medical Social Worker	102	108	112	0.51	19,742	Gabon	Maximum Estimate	1344 - Social welfare managers	High Income and Upper Middle Income	Central	Other Health Workers
27	Mental Health Nurse	304	335	367	1.65	6,065	Gabon	Maximum Estimate	2221 - Nursing professionals	High Income and Upper Middle Income	Central	Nursing Personnel
28	Midwife	2,231	2,402	2,527	11.31	885	Gabon	Maximum Estimate	2222 - Midwifery professionals	High Income and Upper Middle Income	Central	Midwifery Personnel
29	Nephrologist	46	53	60	0.27	37,233	Gabon	Maximum Estimate	2212 - Specialist medical practitioners	High Income and Upper Middle Income	Central	Medical Doctors
30	Neuro-Surgeon	17	19	21	0.09	108,666	Gabon	Maximum Estimate	2212 - Specialist medical practitioners	High Income and Upper Middle Income	Central	Medical Doctors
31	Nurse Anaesthetist	120	139	161	0.72	13,939	Gabon	Maximum Estimate	2221 - Nursing professionals	High Income and Upper Middle Income	Central	Nursing Personnel
32	Nutritionist	256	266	273	1.22	8,181	Gabon	Maximum Estimate	2265 - Dieticians and nutritionists	High Income and Upper Middle Income	Central	Other Health Workers
33	Obstetrician & Gynaecologist	255	283	313	1.40	7,136	Gabon	Maximum Estimate	2212 - Specialist medical practitioners	High Income and Upper Middle Income	Central	Medical Doctors
34	Occupational Therapist	84	94	105	0.47	21,320	Gabon	Maximum Estimate	2269 - Health professionals not elsewhere classified	High Income and Upper Middle Income	Central	Other Health Workers
35	Oncology Nurse	42	49	57	0.25	39,443	Gabon	Maximum Estimate	2221 - Nursing professionals	High Income and Upper Middle Income	Central	Nursing Personnel
36	Operating Theatre Nurse	474	532	595	2.67	3,744	Gabon	Maximum Estimate	2221 - Nursing professionals	High Income and Upper Middle Income	Central	Nursing Personnel
37	Ophthalmic Nurse	69	77	86	0.39	25,930	Gabon	Maximum Estimate	2221 - Nursing professionals	High Income and Upper Middle Income	Central	Nursing Personnel
38	Ophthalmologist	19	21	24	0.11	93,036	Gabon	Maximum Estimate	2212 - Specialist medical practitioners	High Income and Upper Middle Income	Central	Medical Doctors
39	Optometrist	72	82	93	0.41	24,165	Gabon	Maximum Estimate	2267 - Optometrists and ophthalmic opticians	High Income and Upper Middle Income	Central	Other Health Workers
40	Orthopaedic Nurse	95	106	118	0.53	18,977	Gabon	Maximum Estimate	2221 - Nursing professionals	High Income and Upper Middle Income	Central	Nursing Personnel
41	Orthopaedic Surgeon	169	194	221	0.99	10,146	Gabon	Maximum Estimate	2212 - Specialist medical practitioners	High Income and Upper Middle Income	Central	Medical Doctors
42	Orthopaedic Technologist	105	114	124	0.55	18,022	Gabon	Maximum Estimate	3214 - Medical and dental prosthetic technicians	High Income and Upper Middle Income	Central	Other Health Workers
43	Paediatric Nurse	339	359	370	1.65	6,060	Gabon	Maximum Estimate	2221 - Nursing professionals	High Income and Upper Middle Income	Central	Nursing Personnel
44	Paediatric Surgeon	10	10	10	0.05	214,978	Gabon	Maximum Estimate	2212 - Specialist medical practitioners	High Income and Upper Middle Income	Central	Medical Doctors
45	Paediatrician	157	171	184	0.82	12,149	Gabon	Maximum Estimate	2212 - Specialist medical practitioners	High Income and Upper Middle Income	Central	Medical Doctors
46	Pathologist	46	52	58	0.26	38,355	Gabon	Maximum Estimate	2212 - Specialist medical practitioners	High Income and Upper Middle Income	Central	Medical Doctors
47	Pharmacist	581	604	626	2.80	3,570	Gabon	Maximum Estimate	2262 - Pharmacists	High Income and Upper Middle Income	Central	Pharmacist
48	Pharmacy Technician	525	565	606	2.71	3,693	Gabon	Maximum Estimate	3213 - Pharmaceutical technicians and assistants	High Income and Upper Middle Income	Central	Other Health Workers
49	Physician	422	472	525	2.35	4,255	Gabon	Maximum Estimate	2212 - Specialist medical practitioners	High Income and Upper Middle Income	Central	Medical Doctors
50	Physiotherapist	97	106	117	0.52	19,175	Gabon	Maximum Estimate	2264 - Physiotherapists	High Income and Upper Middle Income	Central	Other Health Workers
51	Plastic Surgeon	23	26	29	0.13	77,036	Gabon	Maximum Estimate	2212 - Specialist medical practitioners	High Income and Upper Middle Income	Central	Medical Doctors
52	Psychiatrist	209	228	249	1.12	8,945	Gabon	Maximum Estimate	2212 - Specialist medical practitioners	High Income and Upper Middle Income	Central	Medical Doctors
53	Radiation Oncologist	19	22	26	0.12	86,330	Gabon	Maximum Estimate	2212 - Specialist medical practitioners	High Income and Upper Middle Income	Central	Medical Doctors
54	Radiographer (Diagnostics and Therapy)	348	392	439	1.97	5,081	Gabon	Maximum Estimate	3211 - Medical imaging and therapeutic equipment technicians	High Income and Upper Middle Income	Central	Other Health Workers
55	Radiologist	136	155	177	0.79	12,620	Gabon	Maximum Estimate	2212 - Specialist medical practitioners	High Income and Upper Middle Income	Central	Medical Doctors
56	Registered General Nurse / State Certified Nurse	8,893	9,760	10,618	47.53	210	Gabon	Maximum Estimate	2221 - Nursing professionals	High Income and Upper Middle Income	Central	Nursing Personnel

S/N	Health Professionals	2022	2026	2030	Density per 10,000 population	Required Population ratio (1 professional is to xxx population)	Name of Country	Modelling Scenario	ISCO-08 Match	Income Group Classification	Sub-Regional Grouping	SDG 3c Occupation
57	Renal Nurse	917	1,049	1,191	5.35	1,871	Gabon	Maximum Estimate	2221 - Nursing professionals	High Income and Upper Middle Income	Central	Nursing Personnel
58	Respiratory Physician	27	30	32	0.14	69,122	Gabon	Maximum Estimate	2212 - Specialist medical practitioners	High Income and Upper Middle Income	Central	Medical Doctors
59	Rheumatologist	11	12	13	0.06	169,683	Gabon	Maximum Estimate	2212 - Specialist medical practitioners	High Income and Upper Middle Income	Central	Medical Doctors
60	Speech Therapist	30	33	35	0.16	63,463	Gabon	Maximum Estimate	2266 - Audiologists and speech therapists	High Income and Upper Middle Income	Central	Other Health Workers
61	Urologist	9	11	13	0.06	168,913	Gabon	Maximum Estimate	2212 - Specialist medical practitioners	High Income and Upper Middle Income	Central	Medical Doctors
1	Anaesthesiologist	36	41	46	0.19	52,824	Gambia	Maximum Estimate	2212 - Specialist medical practitioners	Low Income	West	Medical Doctors
2	Associate Nurse/Enrolled Nurse/Nursing Assistant	3,738	4,137	4,572	18.77	533	Gambia	Maximum Estimate	3221 - Nursing associate professionals	Low Income	West	Nursing Personnel
3	Audiologist	17	19	21	0.09	116,175	Gambia	Maximum Estimate	2266 - Audiologists and speech therapists	Low Income	West	Other Health Workers
4	Cardiologist	35	39	43	0.18	56,517	Gambia	Maximum Estimate	2212 - Specialist medical practitioners	Low Income	West	Medical Doctors
5	Cardiothoracic Surgeon	6	7	8	0.03	313,463	Gambia	Maximum Estimate	2212 - Specialist medical practitioners	Low Income	West	Medical Doctors
6	Clinical Officer/Physician Assistant	307	324	346	1.43	6,985	Gambia	Maximum Estimate	3256 - Medical assistants	Low Income	West	Other Health Workers
7	Clinical Pharmacist	100	113	127	0.52	19,158	Gambia	Maximum Estimate	2262 - Pharmacists	Low Income	West	Pharmacist
8	Clinical Psychologist	148	167	189	0.78	12,892	Gambia	Maximum Estimate	2634 - Psychologists	Low Income	West	Other Health Workers
9	Community health worker/Village health worker	2,889	3,101	3,315	13.62	734	Gambia	Maximum Estimate	3253 - Community health workers	Low Income	West	Other Health Workers
10	Dental Surgery Assistant	443	497	558	2.29	4,364	Gambia	Maximum Estimate	3251 - Dental assistants and therapists	Low Income	West	Other Health Workers
11	Dental Therapist	296	333	373	1.53	6,530	Gambia	Maximum Estimate	3251 - Dental assistants and therapists	Low Income	West	Other Health Workers
12	Dentist	335	375	423	1.74	5,746	Gambia	Maximum Estimate	2261 - Dentists	Low Income	West	Dentist
13	Dermatologist	13	14	16	0.07	153,603	Gambia	Maximum Estimate	2212 - Specialist medical practitioners	Low Income	West	Medical Doctors
14	Endocrinologist	11	13	16	0.07	151,028	Gambia	Maximum Estimate	2212 - Specialist medical practitioners	Low Income	West	Medical Doctors
15	ENT Surgeon	44	50	55	0.23	43,837	Gambia	Maximum Estimate	2212 - Specialist medical practitioners	Low Income	West	Medical Doctors
16	Environmental Health Officer	180	198	217	0.89	11,248	Gambia	Maximum Estimate	2263 - Environmental and occupational health and hygiene professionals	Low Income	West	Other Health Workers
17	Gastroenterologist	24	25	27	0.11	89,653	Gambia	Maximum Estimate	2212 - Specialist medical practitioners	Low Income	West	Medical Doctors
18	General Medical Practitioner (Generalist Doctor)	1,311	1,450	1,601	6.57	1,521	Gambia	Maximum Estimate	2211 - Generalist medical practitioners	Low Income	West	Medical Doctors
19	General Surgeon	33	37	43	0.18	57,019	Gambia	Maximum Estimate	2212 - Specialist medical practitioners	Low Income	West	Medical Doctors
20	Haematologist	16	18	20	0.08	120,047	Gambia	Maximum Estimate	2212 - Specialist medical practitioners	Low Income	West	Medical Doctors
21	Health Promoter/Health Educator	32	34	36	0.15	66,807	Gambia	Maximum Estimate	2269 - Health professionals not elsewhere classified	Low Income	West	Other Health Workers
22	Infectious Diseases Specialist	3	3	3	0.01	768,534	Gambia	Maximum Estimate	2212 - Specialist medical practitioners	Low Income	West	Medical Doctors
23	Intensive Care Nurse	104	114	124	0.51	19,636	Gambia	Maximum Estimate	2221 - Nursing professionals	Low Income	West	Nursing Personnel
24	Medical Laboratory Scientist	354	394	442	1.82	5,496	Gambia	Maximum Estimate	3212 - Medical and pathology laboratory technicians	Low Income	West	Other Health Workers
25	Medical Laboratory Technician	388	431	481	1.98	5,048	Gambia	Maximum Estimate	3212 - Medical and pathology laboratory technicians	Low Income	West	Other Health Workers
26	Medical Social Worker	116	123	130	0.54	18,662	Gambia	Maximum Estimate	1344 - Social welfare managers	Low Income	West	Other Health Workers
27	Mental Health Nurse	255	286	327	1.35	7,426	Gambia	Maximum Estimate	2221 - Nursing professionals	Low Income	West	Nursing Personnel
28	Midwife	2,794	3,097	3,425	14.06	711	Gambia	Maximum Estimate	2222 - Midwifery professionals	Low Income	West	Midwifery Personnel
29	Nephrologist	35	40	46	0.19	52,453	Gambia	Maximum Estimate	2212 - Specialist medical practitioners	Low Income	West	Medical Doctors
30	Neuro-Surgeon	8	9	11	0.04	228,298	Gambia	Maximum Estimate	2212 - Specialist medical practitioners	Low Income	West	Medical Doctors
31	Nurse Anaesthetist	68	79	92	0.38	26,562	Gambia	Maximum Estimate	2221 - Nursing professionals	Low Income	West	Nursing Personnel
32	Nutritionist	556	579	600	2.47	4,056	Gambia	Maximum Estimate	2265 - Dieticians and nutritionists	Low Income	West	Other Health Workers
33	Obstetrician & Gynaecologist	211	236	265	1.09	9,196	Gambia	Maximum Estimate	2212 - Specialist medical practitioners	Low Income	West	Medical Doctors
34	Occupational Therapist	65	73	83	0.34	29,492	Gambia	Maximum Estimate	2269 - Health professionals not elsewhere classified	Low Income	West	Other Health Workers
35	Oncology Nurse	25	29	34	0.14	70,756	Gambia	Maximum Estimate	2221 - Nursing professionals	Low Income	West	Nursing Personnel
36	Operating Theatre Nurse	349	392	446	1.84	5,447	Gambia	Maximum Estimate	2221 - Nursing professionals	Low Income	West	Nursing Personnel
37	Ophthalmic Nurse	40	44	49	0.20	49,518	Gambia	Maximum Estimate	2221 - Nursing professionals	Low Income	West	Nursing Personnel
38	Ophthalmologist	14	16	18	0.07	137,579	Gambia	Maximum Estimate	2212 - Specialist medical practitioners	Low Income	West	Medical Doctors
39	Optometrist	55	61	69	0.28	35,527	Gambia	Maximum Estimate	2267 - Optometrists and ophthalmic opticians	Low Income	West	Other Health Workers
40	Orthopaedic Nurse	71	81	91	0.37	26,685	Gambia	Maximum Estimate	2221 - Nursing professionals	Low Income	West	Nursing Personnel
41	Orthopaedic Surgeon	117	135	154	0.63	15,776	Gambia	Maximum Estimate	2212 - Specialist medical practitioners	Low Income	West	Medical Doctors
42	Orthopaedic Technologist	102	114	126	0.52	19,394	Gambia	Maximum Estimate	3214 - Medical and dental prosthetic technicians	Low Income	West	Other Health Workers
43	Paediatric Nurse	472	515	554	2.27	4,408	Gambia	Maximum Estimate	2221 - Nursing professionals	Low Income	West	Nursing Personnel
44	Paediatric Surgeon	13	14	14	0.06	171,363	Gambia	Maximum Estimate	2212 - Specialist medical practitioners	Low Income	West	Medical Doctors
45	Paediatrician	160	180	202	0.83	12,031	Gambia	Maximum Estimate	2212 - Specialist medical practitioners	Low Income	West	Medical Doctors
46	Pathologist	22	25	29	0.12	82,663	Gambia	Maximum Estimate	2212 - Specialist medical practitioners	Low Income	West	Medical Doctors
47	Pharmacist	215	224	235	0.97	10,288	Gambia	Maximum Estimate	2262 - Pharmacists	Low Income	West	Pharmacist
48	Pharmacy Technician	396	439	488	2.00	4,995	Gambia	Maximum Estimate	3213 - Pharmaceutical technicians and assistants	Low Income	West	Other Health Workers
49	Physician	402	451	506	2.08	4,811	Gambia	Maximum Estimate	2212 - Specialist medical practitioners	Low Income	West	Medical Doctors
50	Physiotherapist	90	100	111	0.46	21,973	Gambia	Maximum Estimate	2264 - Physiotherapists	Low Income	West	Other Health Workers
51	Plastic Surgeon	26	30	34	0.14	71,833	Gambia	Maximum Estimate	2212 - Specialist medical practitioners	Low Income	West	Medical Doctors
52	Psychiatrist	212	235	264	1.09	9,197	Gambia	Maximum Estimate	2212 - Specialist medical practitioners	Low Income	West	Medical Doctors
53	Radiation Oncologist	7	9	10	0.04	240,651	Gambia	Maximum Estimate	2212 - Specialist medical practitioners	Low Income	West	Medical Doctors
54	Radiographer (Diagnostics and Therapy)	291	331	378	1.56	6,426	Gambia	Maximum Estimate	3211 - Medical imaging and therapeutic equipment technicians	Low Income	West	Other Health Workers
55	Radiologist	108	124	142	0.59	17,039	Gambia	Maximum Estimate	2212 - Specialist medical practitioners	Low Income	West	Medical Doctors
56	Registered General Nurse / State Certified Nurse	8,011	8,777	9,641	39.67	252	Gambia	Maximum Estimate	2221 - Nursing professionals	Low Income	West	Nursing Personnel
57	Renal Nurse	668	766	893	3.69	2,712	Gambia	Maximum Estimate	2221 - Nursing professionals	Low Income	West	Nursing Personnel
58	Respiratory Physician	29	33	37	0.15	65,361	Gambia	Maximum Estimate	2212 - Specialist medical practitioners	Low Income	West	Medical Doctors
59	Rheumatologist	9	10	11	0.05	214,824	Gambia	Maximum Estimate	2212 - Specialist medical practitioners	Low Income	West	Medical Doctors
60	Speech Therapist	31	34	38	0.15	64,613	Gambia	Maximum Estimate	2266 - Audiologists and speech therapists	Low Income	West	Other Health Workers
61	Urologist	6	7	8	0.03	306,692	Gambia	Maximum Estimate	2212 - Specialist medical practitioners	Low Income	West	Medical Doctors
1	Anaesthesiologist	696	798	916	0.29	34,151	Ghana	Maximum Estimate	2212 - Specialist medical practitioners	Lower-middle Income	West	Medical Doctors
2	Associate Nurse/Enrolled Nurse/Nursing Assistant	55,096	59,655	64,712	20.70	483	Ghana	Maximum Estimate	3221 - Nursing associate professionals	Lower-middle Income	West	Nursing Personnel
3	Audiologist	210	230	250	0.08	125,262	Ghana	Maximum Estimate	2266 - Audiologists and speech therapists	Lower-middle Income	West	Other Health Workers
4	Cardiologist	504	565	632	0.20	49,505	Ghana	Maximum Estimate	2212 - Specialist medical practitioners	Lower-middle Income	West	Medical Doctors
5	Cardiothoracic Surgeon	148	171	197	0.06	159,007	Ghana	Maximum Estimate	2212 - Specialist medical practitioners	Lower-middle Income	West	Medical Doctors
6	Clinical Officer/Physician Assistant	9,994	10,724	11,845	3.85	2,600	Ghana	Maximum Estimate	3256 - Medical assistants	Lower-middle Income	West	Other Health Workers
7	Clinical Pharmacist	1,735	1,909	2,118	0.68	14,737	Ghana	Maximum Estimate	2262 - Pharmacists	Lower-middle Income	West	Pharmacist
8	Clinical Psychologist	3,215	3,512	3,855	1.23	8,112	Ghana	Maximum Estimate	2634 - Psychologists	Lower-middle Income	West	Other Health Workers
9	Community health worker/Village health worker	41,020	42,040	43,441	14.01	714	Ghana	Maximum Estimate	3253 - Community health workers	Lower-middle Income	West	Other Health Workers
10	Dental Surgery Assistant	5,537	6,156	6,818	2.18	4,590	Ghana	Maximum Estimate	3251 - Dental assistants and therapists	Lower-middle Income	West	Other Health Workers
11	Dental Therapist	3,694	4,105	4,544	1.45	6,888	Ghana	Maximum Estimate	3251 - Dental assistants and therapists	Lower-middle Income	West	Other Health Workers
12	Dentist	3,799	4,135	4,491	1.44	6,954	Ghana	Maximum Estimate	2261 - Dentists	Lower-middle Income	West	Dentist
13	Dermatologist	180	197	216	0.07	145,276	Ghana	Maximum Estimate	2212 - Specialist medical practitioners	Lower-middle Income	West	Medical Doctors
14	Endocrinologist	221	249	284	0.09	110,066	Ghana	Maximum Estimate	2212 - Specialist medical practitioners	Lower-middle Income	West	Medical Doctors
15	ENT Surgeon	642	713	803	0.26	38,801	Ghana	Maximum Estimate	2212 - Specialist medical practitioners	Lower-middle Income	West	Medical Doctors
16	Environmental Health Officer	2,315	2,496	2,681	0.86	11,687	Ghana	Maximum Estimate	2263 - Environmental and occupational health and hygiene professionals	Lower-middle Income	West	Other Health Workers
17	Gastroenterologist	731	724	728	0.23	42,599	Ghana	Maximum Estimate	2212 - Specialist medical practitioners	Lower-middle Income	West	Medical Doctors
18	General Medical Practitioner (Generalist Doctor)	19,703	21,170	22,841	7.32	1,366	Ghana	Maximum Estimate	2211 - Generalist medical practitioners	Lower-middle Income	West	Medical Doctors

S/N	Health Professionals	2022	2026	2030	Density per 10,000 population	Required Population ratio (1 professional is to xxx population)	Name of Country	Modelling Scenario	ISCO-08 Match	Income Group Classification	Sub-Regional Grouping	SDG 3c Occupation
19	General Surgeon	533	605	691	0.22	45,232	Ghana	Maximum Estimate	2212 - Specialist medical practitioners	Lower-middle Income	West	Medical Doctors
20	Haematologist	202	227	255	0.08	122,844	Ghana	Maximum Estimate	2212 - Specialist medical practitioners	Lower-middle Income	West	Medical Doctors
21	Health Promoter/Health Educator	817	828	863	0.28	35,608	Ghana	Maximum Estimate	2269 - Health professionals not elsewhere classified	Lower-middle Income	West	Other Health Workers
22	Infectious Diseases Specialist	68	71	76	0.02	413,342	Ghana	Maximum Estimate	2212 - Specialist medical practitioners	Lower-middle Income	West	Medical Doctors
23	Intensive Care Nurse	1,525	1,655	1,796	0.57	17,407	Ghana	Maximum Estimate	2221 - Nursing professionals	Lower-middle Income	West	Nursing Personnel
24	Medical Laboratory Scientist	6,191	6,635	7,210	2.32	4,316	Ghana	Maximum Estimate	3212 - Medical and pathology laboratory technicians	Lower-middle Income	West	Other Health Workers
25	Medical Laboratory Technician	6,487	6,502	6,645	2.15	4,642	Ghana	Maximum Estimate	3212 - Medical and pathology laboratory technicians	Lower-middle Income	West	Other Health Workers
26	Medical Social Worker	1,819	1,862	1,930	0.62	16,025	Ghana	Maximum Estimate	1344 - Social welfare managers	Lower-middle Income	West	Other Health Workers
27	Mental Health Nurse	3,303	3,634	4,050	1.30	7,717	Ghana	Maximum Estimate	2221 - Nursing professionals	Lower-middle Income	West	Nursing Personnel
28	Midwife	32,585	34,859	37,092	11.87	843	Ghana	Maximum Estimate	2222 - Midwifery professionals	Lower-middle Income	West	Midwifery Personnel
29	Nephrologist	586	668	773	0.25	40,409	Ghana	Maximum Estimate	2212 - Specialist medical practitioners	Lower-middle Income	West	Medical Doctors
30	Neuro-Surgeon	224	254	294	0.09	105,875	Ghana	Maximum Estimate	2212 - Specialist medical practitioners	Lower-middle Income	West	Medical Doctors
31	Nurse Anaesthetist	1,621	1,900	2,223	0.71	14,058	Ghana	Maximum Estimate	2221 - Nursing professionals	Lower-middle Income	West	Nursing Personnel
32	Nutritionist	5,410	5,531	5,637	1.81	5,530	Ghana	Maximum Estimate	2265 - Dieticians and nutritionists	Lower-middle Income	West	Other Health Workers
33	Obstetrician & Gynaecologist	3,124	3,466	3,861	1.24	8,083	Ghana	Maximum Estimate	2212 - Specialist medical practitioners	Lower-middle Income	West	Medical Doctors
34	Occupational Therapist	1,292	1,490	1,727	0.55	18,094	Ghana	Maximum Estimate	2269 - Health professionals not elsewhere classified	Lower-middle Income	West	Other Health Workers
35	Oncology Nurse	505	584	682	0.22	45,787	Ghana	Maximum Estimate	2221 - Nursing professionals	Lower-middle Income	West	Nursing Personnel
36	Operating Theatre Nurse	5,509	6,221	7,130	2.28	4,378	Ghana	Maximum Estimate	2221 - Nursing professionals	Lower-middle Income	West	Nursing Personnel
37	Ophthalmic Nurse	709	773	845	0.27	37,049	Ghana	Maximum Estimate	2221 - Nursing professionals	Lower-middle Income	West	Nursing Personnel
38	Ophthalmologist	214	237	265	0.08	117,812	Ghana	Maximum Estimate	2212 - Specialist medical practitioners	Lower-middle Income	West	Medical Doctors
39	Optometrist	795	869	952	0.30	32,900	Ghana	Maximum Estimate	2267 - Optometrists and ophthalmic opticians	Lower-middle Income	West	Other Health Workers
40	Orthopaedic Nurse	1,710	1,998	2,327	0.74	13,435	Ghana	Maximum Estimate	2221 - Nursing professionals	Lower-middle Income	West	Nursing Personnel
41	Orthopaedic Surgeon	2,042	2,346	2,697	0.86	11,598	Ghana	Maximum Estimate	2212 - Specialist medical practitioners	Lower-middle Income	West	Medical Doctors
42	Orthopaedic Technologist	1,749	1,961	2,195	0.70	14,256	Ghana	Maximum Estimate	3214 - Medical and dental prosthetic technicians	Lower-middle Income	West	Other Health Workers
43	Paediatric Nurse	5,073	5,483	5,857	1.87	5,346	Ghana	Maximum Estimate	2221 - Nursing professionals	Lower-middle Income	West	Nursing Personnel
44	Paediatric Surgeon	143	150	157	0.05	199,890	Ghana	Maximum Estimate	2212 - Specialist medical practitioners	Lower-middle Income	West	Medical Doctors
45	Paediatrician	2,040	2,260	2,509	0.80	12,471	Ghana	Maximum Estimate	2212 - Specialist medical practitioners	Lower-middle Income	West	Medical Doctors
46	Pathologist	452	505	572	0.18	54,660	Ghana	Maximum Estimate	2212 - Specialist medical practitioners	Lower-middle Income	West	Medical Doctors
47	Pharmacist	5,359	5,639	6,062	1.96	5,089	Ghana	Maximum Estimate	2262 - Pharmacists	Lower-middle Income	West	Pharmacist
48	Pharmacy Technician	6,436	6,964	7,578	2.43	4,115	Ghana	Maximum Estimate	3213 - Pharmaceutical technicians and assistants	Lower-middle Income	West	Other Health Workers
49	Physician	5,719	6,232	6,820	2.18	4,584	Ghana	Maximum Estimate	2212 - Specialist medical practitioners	Lower-middle Income	West	Medical Doctors
50	Physiotherapist	1,428	1,587	1,778	0.57	17,558	Ghana	Maximum Estimate	2264 - Physiotherapists	Lower-middle Income	West	Other Health Workers
51	Plastic Surgeon	395	451	515	0.16	60,837	Ghana	Maximum Estimate	2212 - Specialist medical practitioners	Lower-middle Income	West	Medical Doctors
52	Psychiatrist	2,687	2,925	3,218	1.03	9,709	Ghana	Maximum Estimate	2212 - Specialist medical practitioners	Lower-middle Income	West	Medical Doctors
53	Radiation Oncologist	179	212	253	0.08	123,394	Ghana	Maximum Estimate	2212 - Specialist medical practitioners	Lower-middle Income	West	Medical Doctors
54	Radiographer (Diagnostics and Therapy)	4,751	5,311	5,988	1.92	5,212	Ghana	Maximum Estimate	3211 - Medical imaging and therapeutic equipment technicians	Lower-middle Income	West	Other Health Workers
55	Radiologist	1,821	2,067	2,370	0.76	13,106	Ghana	Maximum Estimate	2212 - Specialist medical practitioners	Lower-middle Income	West	Medical Doctors
56	Registered General Nurse / State Certified Nurse	116,443	124,912	135,525	43.53	230	Ghana	Maximum Estimate	2221 - Nursing professionals	Lower-middle Income	West	Nursing Personnel
57	Renal Nurse	11,523	13,147	15,222	4.87	2,051	Ghana	Maximum Estimate	2221 - Nursing professionals	Lower-middle Income	West	Nursing Personnel
58	Respiratory Physician	314	353	396	0.13	78,998	Ghana	Maximum Estimate	2212 - Specialist medical practitioners	Lower-middle Income	West	Medical Doctors
59	Rheumatologist	135	150	167	0.05	187,918	Ghana	Maximum Estimate	2212 - Specialist medical practitioners	Lower-middle Income	West	Medical Doctors
60	Speech Therapist	438	476	528	0.17	58,810	Ghana	Maximum Estimate	2266 - Audiologists and speech therapists	Lower-middle Income	West	Other Health Workers
61	Urologist	109	130	158	0.05	195,064	Ghana	Maximum Estimate	2212 - Specialist medical practitioners	Lower-middle Income	West	Medical Doctors
1	Anaesthesiologist	214	238	263	0.20	50,478	Guinea	Maximum Estimate	2212 - Specialist medical practitioners	Low Income	West	Medical Doctors
2	Associate Nurse/Enrolled Nurse/Nursing Assistant	20,755	22,699	24,877	18.78	532	Guinea	Maximum Estimate	3221 - Nursing associate professionals	Low Income	West	Nursing Personnel
3	Audiologist	85	93	101	0.08	130,821	Guinea	Maximum Estimate	2266 - Audiologists and speech therapists	Low Income	West	Other Health Workers
4	Cardiologist	153	164	174	0.13	75,927	Guinea	Maximum Estimate	2212 - Specialist medical practitioners	Low Income	West	Medical Doctors
5	Cardiothoracic Surgeon	41	46	50	0.04	267,073	Guinea	Maximum Estimate	2212 - Specialist medical practitioners	Low Income	West	Medical Doctors
6	Clinical Officer/Physician Assistant	3,482	3,531	3,631	2.77	3,613	Guinea	Maximum Estimate	3256 - Medical assistants	Low Income	West	Other Health Workers
7	Clinical Pharmacist	632	690	765	0.58	17,279	Guinea	Maximum Estimate	2262 - Pharmacists	Low Income	West	Pharmacist
8	Clinical Psychologist	1,054	1,147	1,268	0.96	10,429	Guinea	Maximum Estimate	2634 - Psychologists	Low Income	West	Other Health Workers
9	Community health worker/Village health worker	23,871	25,321	26,787	20.25	494	Guinea	Maximum Estimate	3253 - Community health workers	Low Income	West	Other Health Workers
10	Dental Surgery Assistant	2,016	2,222	2,466	1.86	5,377	Guinea	Maximum Estimate	3251 - Dental assistants and therapists	Low Income	West	Other Health Workers
11	Dental Therapist	1,352	1,490	1,654	1.25	8,019	Guinea	Maximum Estimate	3251 - Dental assistants and therapists	Low Income	West	Other Health Workers
12	Dentist	1,665	1,832	2,067	1.56	6,412	Guinea	Maximum Estimate	2261 - Dentists	Low Income	West	Dentist
13	Dermatologist	65	71	78	0.06	169,670	Guinea	Maximum Estimate	2212 - Specialist medical practitioners	Low Income	West	Medical Doctors
14	Endocrinologist	55	63	76	0.06	172,137	Guinea	Maximum Estimate	2212 - Specialist medical practitioners	Low Income	West	Medical Doctors
15	ENT Surgeon	222	243	264	0.20	50,153	Guinea	Maximum Estimate	2212 - Specialist medical practitioners	Low Income	West	Medical Doctors
16	Environmental Health Officer	978	1,074	1,173	0.88	11,309	Guinea	Maximum Estimate	2263 - Environmental and occupational health and hygiene professionals	Low Income	West	Other Health Workers
17	Gastroenterologist	340	347	368	0.28	35,756	Guinea	Maximum Estimate	2212 - Specialist medical practitioners	Low Income	West	Medical Doctors
18	General Medical Practitioner (Generalist Doctor)	7,961	8,606	9,338	7.06	1,416	Guinea	Maximum Estimate	2211 - Generalist medical practitioners	Low Income	West	Medical Doctors
19	General Surgeon	175	194	218	0.16	60,613	Guinea	Maximum Estimate	2212 - Specialist medical practitioners	Low Income	West	Medical Doctors
20	Haematologist	80	89	99	0.07	134,590	Guinea	Maximum Estimate	2212 - Specialist medical practitioners	Low Income	West	Medical Doctors
21	Health Promoter/Health Educator	339	345	357	0.27	36,835	Guinea	Maximum Estimate	2269 - Health professionals not elsewhere classified	Low Income	West	Other Health Workers
22	Infectious Diseases Specialist	28	34	43	0.03	294,115	Guinea	Maximum Estimate	2212 - Specialist medical practitioners	Low Income	West	Medical Doctors
23	Intensive Care Nurse	635	696	759	0.57	17,470	Guinea	Maximum Estimate	2221 - Nursing professionals	Low Income	West	Nursing Personnel
24	Medical Laboratory Scientist	2,606	2,814	3,090	2.34	4,268	Guinea	Maximum Estimate	3212 - Medical and pathology laboratory technicians	Low Income	West	Other Health Workers
25	Medical Laboratory Technician	5,167	5,481	5,818	4.40	2,272	Guinea	Maximum Estimate	3212 - Medical and pathology laboratory technicians	Low Income	West	Other Health Workers
26	Medical Social Worker	894	940	1,008	0.76	13,102	Guinea	Maximum Estimate	1344 - Social welfare managers	Low Income	West	Other Health Workers
27	Mental Health Nurse	1,174	1,296	1,482	1.12	8,908	Guinea	Maximum Estimate	2221 - Nursing professionals	Low Income	West	Nursing Personnel
28	Midwife	17,606	19,273	21,216	16.00	625	Guinea	Maximum Estimate	2222 - Midwifery professionals	Low Income	West	Midwifery Personnel
29	Nephrologist	161	177	204	0.15	64,800	Guinea	Maximum Estimate	2212 - Specialist medical practitioners	Low Income	West	Medical Doctors
30	Neuro-Surgeon	54	60	67	0.05	197,815	Guinea	Maximum Estimate	2212 - Specialist medical practitioners	Low Income	West	Medical Doctors
31	Nurse Anaesthetist	433	477	527	0.40	25,167	Guinea	Maximum Estimate	2221 - Nursing professionals	Low Income	West	Nursing Personnel
32	Nutritionist	2,851	3,054	3,252	2.45	4,075	Guinea	Maximum Estimate	2265 - Dieticians and nutritionists	Low Income	West	Other Health Workers
33	Obstetrician & Gynaecologist	1,146	1,271	1,417	1.07	9,341	Guinea	Maximum Estimate	2212 - Specialist medical practitioners	Low Income	West	Medical Doctors
34	Occupational Therapist	383	424	471	0.36	28,094	Guinea	Maximum Estimate	2269 - Health professionals not elsewhere classified	Low Income	West	Other Health Workers
35	Oncology Nurse	136	150	168	0.13	78,452	Guinea	Maximum Estimate	2221 - Nursing professionals	Low Income	West	Nursing Personnel
36	Operating Theatre Nurse	1,802	1,977	2,227	1.69	5,933	Guinea	Maximum Estimate	2221 - Nursing professionals	Low Income	West	Nursing Personnel
37	Ophthalmic Nurse	203	223	246	0.19	53,736	Guinea	Maximum Estimate	2221 - Nursing professionals	Low Income	West	Nursing Personnel
38	Ophthalmologist	68	75	83	0.06	159,128	Guinea	Maximum Estimate	2212 - Specialist medical practitioners	Low Income	West	Medical Doctors
39	Optometrist	276	296	318	0.24	41,631	Guinea	Maximum Estimate	2267 - Optometrists and ophthalmic opticians	Low Income	West	Other Health Workers
40	Orthopaedic Nurse	465	519	577	0.43	22,998	Guinea	Maximum Estimate	2221 - Nursing professionals	Low Income	West	Nursing Personnel
41	Orthopaedic Surgeon	580	649	730	0.55	18,155	Guinea	Maximum Estimate	2212 - Specialist medical practitioners	Low Income	West	Medical Doctors

S/N	Health Professionals	2022	2026	2030	Density per 10,000 population	Required Population ratio (1 professional is to xxx population)	Name of Country	Modelling Scenario	ISCO-08 Match	Income Group Classification	Sub-Regional Grouping	SDG 3c Occupation
42	Orthopaedic Technologist	591	651	715	0.54	18,543	Guinea	Maximum Estimate	3214 - Medical and dental prosthetic technicians	Low Income	West	Other Health Workers
43	Paediatric Nurse	2,431	2,673	2,907	2.19	4,568	Guinea	Maximum Estimate	2221 - Nursing professionals	Low Income	West	Nursing Personnel
44	Paediatric Surgeon	79	87	93	0.07	142,929	Guinea	Maximum Estimate	2212 - Specialist medical practitioners	Low Income	West	Medical Doctors
45	Paediatrician	969	1,074	1,195	0.90	11,090	Guinea	Maximum Estimate	2212 - Specialist medical practitioners	Low Income	West	Medical Doctors
46	Pathologist	134	147	167	0.13	78,901	Guinea	Maximum Estimate	2212 - Specialist medical practitioners	Low Income	West	Medical Doctors
47	Pharmacist	1,536	1,528	1,539	1.18	8,495	Guinea	Maximum Estimate	2262 - Pharmacists	Low Income	West	Pharmacist
48	Pharmacy Technician	2,297	2,478	2,690	2.04	4,912	Guinea	Maximum Estimate	3213 - Pharmaceutical technicians and assistants	Low Income	West	Other Health Workers
49	Physician	2,411	2,642	2,913	2.20	4,542	Guinea	Maximum Estimate	2212 - Specialist medical practitioners	Low Income	West	Medical Doctors
50	Physiotherapist	500	550	604	0.46	21,935	Guinea	Maximum Estimate	2264 - Physiotherapists	Low Income	West	Other Health Workers
51	Plastic Surgeon	136	152	168	0.13	78,782	Guinea	Maximum Estimate	2212 - Specialist medical practitioners	Low Income	West	Medical Doctors
52	Psychiatrist	996	1,095	1,235	0.93	10,701	Guinea	Maximum Estimate	2212 - Specialist medical practitioners	Low Income	West	Medical Doctors
53	Radiation Oncologist	45	50	55	0.04	241,128	Guinea	Maximum Estimate	2212 - Specialist medical practitioners	Low Income	West	Medical Doctors
54	Radiographer (Diagnostics and Therapy)	1,562	1,725	1,944	1.47	6,800	Guinea	Maximum Estimate	3211 - Medical imaging and therapeutic equipment technicians	Low Income	West	Other Health Workers
55	Radiologist	618	688	777	0.59	17,013	Guinea	Maximum Estimate	2212 - Specialist medical practitioners	Low Income	West	Medical Doctors
56	Registered General Nurse / State Certified Nurse	44,213	47,427	51,487	38.99	256	Guinea	Maximum Estimate	2221 - Nursing professionals	Low Income	West	Nursing Personnel
57	Renal Nurse	3,101	3,412	3,916	2.97	3,369	Guinea	Maximum Estimate	2221 - Nursing professionals	Low Income	West	Nursing Personnel
58	Respiratory Physician	183	203	226	0.17	58,615	Guinea	Maximum Estimate	2212 - Specialist medical practitioners	Low Income	West	Medical Doctors
59	Rheumatologist	47	52	56	0.04	235,525	Guinea	Maximum Estimate	2212 - Specialist medical practitioners	Low Income	West	Medical Doctors
60	Speech Therapist	172	188	206	0.16	64,286	Guinea	Maximum Estimate	2266 - Audiologists and speech therapists	Low Income	West	Other Health Workers
61	Urologist	32	37	43	0.03	303,844	Guinea	Maximum Estimate	2212 - Specialist medical practitioners	Low Income	West	Medical Doctors
1	Anaesthesiologist	38	42	46	0.23	42,829	Guinea-Bissau	Maximum Estimate	2212 - Specialist medical practitioners	Low Income	West	Medical Doctors
2	Associate Nurse/Enrolled Nurse/Nursing Assistant	3,477	3,770	4,094	20.68	484	Guinea-Bissau	Maximum Estimate	3221 - Nursing associate professionals	Low Income	West	Nursing Personnel
3	Audiologist	13	15	16	0.08	124,958	Guinea-Bissau	Maximum Estimate	2266 - Audiologists and speech therapists	Low Income	West	Other Health Workers
4	Cardiologist	20	23	25	0.13	79,283	Guinea-Bissau	Maximum Estimate	2212 - Specialist medical practitioners	Low Income	West	Medical Doctors
5	Cardiothoracic Surgeon	8	9	9	0.05	210,035	Guinea-Bissau	Maximum Estimate	2212 - Specialist medical practitioners	Low Income	West	Medical Doctors
6	Clinical Officer/Physician Assistant	579	570	569	2.91	3,435	Guinea-Bissau	Maximum Estimate	3256 - Medical assistants	Low Income	West	Other Health Workers
7	Clinical Pharmacist	94	103	113	0.57	17,470	Guinea-Bissau	Maximum Estimate	2262 - Pharmacists	Low Income	West	Pharmacist
8	Clinical Psychologist	171	186	203	1.03	9,718	Guinea-Bissau	Maximum Estimate	2634 - Psychologists	Low Income	West	Other Health Workers
9	Community health worker/Village health worker	2,626	2,715	2,809	14.24	702	Guinea-Bissau	Maximum Estimate	3253 - Community health workers	Low Income	West	Other Health Workers
10	Dental Surgery Assistant	345	384	427	2.16	4,636	Guinea-Bissau	Maximum Estimate	3251 - Dental assistants and therapists	Low Income	West	Other Health Workers
11	Dental Therapist	232	257	286	1.45	6,919	Guinea-Bissau	Maximum Estimate	3251 - Dental assistants and therapists	Low Income	West	Other Health Workers
12	Dentist	300	330	369	1.87	5,362	Guinea-Bissau	Maximum Estimate	2261 - Dentists	Low Income	West	Dentist
13	Dermatologist	10	11	12	0.06	160,122	Guinea-Bissau	Maximum Estimate	2212 - Specialist medical practitioners	Low Income	West	Medical Doctors
14	Endocrinologist	10	12	14	0.07	141,734	Guinea-Bissau	Maximum Estimate	2212 - Specialist medical practitioners	Low Income	West	Medical Doctors
15	ENT Surgeon	34	38	42	0.21	47,551	Guinea-Bissau	Maximum Estimate	2212 - Specialist medical practitioners	Low Income	West	Medical Doctors
16	Environmental Health Officer	147	160	173	0.87	11,483	Guinea-Bissau	Maximum Estimate	2263 - Environmental and occupational health and hygiene professionals	Low Income	West	Other Health Workers
17	Gastroenterologist	45	45	46	0.24	42,255	Guinea-Bissau	Maximum Estimate	2212 - Specialist medical practitioners	Low Income	West	Medical Doctors
18	General Medical Practitioner (Generalist Doctor)	1,201	1,294	1,398	7.06	1,416	Guinea-Bissau	Maximum Estimate	2211 - Generalist medical practitioners	Low Income	West	Medical Doctors
19	General Surgeon	29	32	36	0.18	55,557	Guinea-Bissau	Maximum Estimate	2212 - Specialist medical practitioners	Low Income	West	Medical Doctors
20	Haematologist	11	12	13	0.07	151,363	Guinea-Bissau	Maximum Estimate	2212 - Specialist medical practitioners	Low Income	West	Medical Doctors
21	Health Promoter/Health Educator	61	60	61	0.31	32,317	Guinea-Bissau	Maximum Estimate	2269 - Health professionals not elsewhere classified	Low Income	West	Other Health Workers
22	Infectious Diseases Specialist	3	4	4	0.02	510,626	Guinea-Bissau	Maximum Estimate	2212 - Specialist medical practitioners	Low Income	West	Medical Doctors
23	Intensive Care Nurse	96	103	111	0.56	17,866	Guinea-Bissau	Maximum Estimate	2221 - Nursing professionals	Low Income	West	Nursing Personnel
24	Medical Laboratory Scientist	328	354	386	1.96	5,103	Guinea-Bissau	Maximum Estimate	3212 - Medical and pathology laboratory technicians	Low Income	West	Other Health Workers
25	Medical Laboratory Technician	324	335	350	1.78	5,616	Guinea-Bissau	Maximum Estimate	3212 - Medical and pathology laboratory technicians	Low Income	West	Other Health Workers
26	Medical Social Worker	118	121	124	0.63	15,836	Guinea-Bissau	Maximum Estimate	1344 - Social welfare managers	Low Income	West	Other Health Workers
27	Mental Health Nurse	184	203	228	1.16	8,626	Guinea-Bissau	Maximum Estimate	2221 - Nursing professionals	Low Income	West	Nursing Personnel
28	Midwife	2,208	2,370	2,552	12.89	776	Guinea-Bissau	Maximum Estimate	2222 - Midwifery professionals	Low Income	West	Midwifery Personnel
29	Nephrologist	29	32	37	0.19	52,846	Guinea-Bissau	Maximum Estimate	2212 - Specialist medical practitioners	Low Income	West	Medical Doctors
30	Neuro-Surgeon	11	12	13	0.07	153,144	Guinea-Bissau	Maximum Estimate	2212 - Specialist medical practitioners	Low Income	West	Medical Doctors
31	Nurse Anaesthetist	70	78	86	0.44	22,981	Guinea-Bissau	Maximum Estimate	2221 - Nursing professionals	Low Income	West	Nursing Personnel
32	Nutritionist	389	406	421	2.13	4,705	Guinea-Bissau	Maximum Estimate	2265 - Dieticians and nutritionists	Low Income	West	Other Health Workers
33	Obstetrician & Gynaecologist	180	198	219	1.10	9,066	Guinea-Bissau	Maximum Estimate	2212 - Specialist medical practitioners	Low Income	West	Medical Doctors
34	Occupational Therapist	69	77	86	0.44	22,958	Guinea-Bissau	Maximum Estimate	2269 - Health professionals not elsewhere classified	Low Income	West	Other Health Workers
35	Oncology Nurse	22	25	28	0.14	69,380	Guinea-Bissau	Maximum Estimate	2221 - Nursing professionals	Low Income	West	Nursing Personnel
36	Operating Theatre Nurse	301	335	379	1.92	5,205	Guinea-Bissau	Maximum Estimate	2221 - Nursing professionals	Low Income	West	Nursing Personnel
37	Ophthalmic Nurse	32	36	40	0.20	49,758	Guinea-Bissau	Maximum Estimate	2221 - Nursing professionals	Low Income	West	Nursing Personnel
38	Ophthalmologist	11	12	14	0.07	141,117	Guinea-Bissau	Maximum Estimate	2212 - Specialist medical practitioners	Low Income	West	Medical Doctors
39	Optometrist	44	49	54	0.28	36,342	Guinea-Bissau	Maximum Estimate	2267 - Optometrists and ophthalmic opticians	Low Income	West	Other Health Workers
40	Orthopaedic Nurse	88	99	110	0.55	18,019	Guinea-Bissau	Maximum Estimate	2221 - Nursing professionals	Low Income	West	Nursing Personnel
41	Orthopaedic Surgeon	102	116	133	0.67	14,920	Guinea-Bissau	Maximum Estimate	2212 - Specialist medical practitioners	Low Income	West	Medical Doctors
42	Orthopaedic Technologist	98	107	118	0.59	16,856	Guinea-Bissau	Maximum Estimate	3214 - Medical and dental prosthetic technicians	Low Income	West	Other Health Workers
43	Paediatric Nurse	341	369	392	1.98	5,058	Guinea-Bissau	Maximum Estimate	2221 - Nursing professionals	Low Income	West	Nursing Personnel
44	Paediatric Surgeon	11	12	12	0.06	161,945	Guinea-Bissau	Maximum Estimate	2212 - Specialist medical practitioners	Low Income	West	Medical Doctors
45	Paediatrician	123	136	150	0.76	13,216	Guinea-Bissau	Maximum Estimate	2212 - Specialist medical practitioners	Low Income	West	Medical Doctors
46	Pathologist	21	24	27	0.14	71,879	Guinea-Bissau	Maximum Estimate	2212 - Specialist medical practitioners	Low Income	West	Medical Doctors
47	Pharmacist	336	338	343	1.74	5,736	Guinea-Bissau	Maximum Estimate	2262 - Pharmacists	Low Income	West	Pharmacist
48	Pharmacy Technician	397	428	463	2.34	4,274	Guinea-Bissau	Maximum Estimate	3213 - Pharmaceutical technicians and assistants	Low Income	West	Other Health Workers
49	Physician	330	362	398	2.01	4,964	Guinea-Bissau	Maximum Estimate	2212 - Specialist medical practitioners	Low Income	West	Medical Doctors
50	Physiotherapist	81	89	97	0.49	20,361	Guinea-Bissau	Maximum Estimate	2264 - Physiotherapists	Low Income	West	Other Health Workers
51	Plastic Surgeon	21	23	26	0.13	76,353	Guinea-Bissau	Maximum Estimate	2212 - Specialist medical practitioners	Low Income	West	Medical Doctors
52	Psychiatrist	155	170	188	0.96	10,460	Guinea-Bissau	Maximum Estimate	2212 - Specialist medical practitioners	Low Income	West	Medical Doctors
53	Radiation Oncologist	7	8	9	0.05	221,233	Guinea-Bissau	Maximum Estimate	2212 - Specialist medical practitioners	Low Income	West	Medical Doctors
54	Radiographer (Diagnostics and Therapy)	250	278	313	1.58	6,316	Guinea-Bissau	Maximum Estimate	3211 - Medical imaging and therapeutic equipment technicians	Low Income	West	Other Health Workers
55	Radiologist	95	105	118	0.59	16,824	Guinea-Bissau	Maximum Estimate	2212 - Specialist medical practitioners	Low Income	West	Medical Doctors
56	Registered General Nurse / State Certified Nurse	11,267	11,483	11,802	60.10	166	Guinea-Bissau	Maximum Estimate	2221 - Nursing professionals	Low Income	West	Nursing Personnel
57	Renal Nurse	551	623	720	3.67	2,727	Guinea-Bissau	Maximum Estimate	2221 - Nursing professionals	Low Income	West	Nursing Personnel
58	Respiratory Physician	24	27	30	0.15	66,975	Guinea-Bissau	Maximum Estimate	2212 - Specialist medical practitioners	Low Income	West	Medical Doctors
59	Rheumatologist	7	8	9	0.04	224,008	Guinea-Bissau	Maximum Estimate	2212 - Specialist medical practitioners	Low Income	West	Medical Doctors
60	Speech Therapist	26	28	30	0.15	65,611	Guinea-Bissau	Maximum Estimate	2266 - Audiologists and speech therapists	Low Income	West	Other Health Workers
61	Urologist	4	5	5	0.03	378,420	Guinea-Bissau	Maximum Estimate	2212 - Specialist medical practitioners	Low Income	West	Medical Doctors
1	Anaesthesiologist	788	905	1,051	0.19	51,326	Kenya	Maximum Estimate	2212 - Specialist medical practitioners	Lower-middle Income	East	Medical Doctors
2	Associate Nurse/Enrolled Nurse/Nursing Assistant	86,592	94,013	103,069	19.08	524	Kenya	Maximum Estimate	3221 - Nursing associate professionals	Lower-middle Income	East	Nursing Personnel
3	Audiologist	400	439	482	0.09	112,471	Kenya	Maximum Estimate	2266 - Audiologists and speech therapists	Lower-middle Income	East	Other Health Workers

S/N	Health Professionals	2022	2026	2030	Density per 10,000 population	Required Population ratio (1 professional is to xxx population)	Name of Country	Modelling Scenario	ISCO-08 Match	Income Group Classification	Sub-Regional Grouping	SDG 3c Occupation
4	Cardiologist	836	954	1,088	0.20	49,779	Kenya	Maximum Estimate	2212 - Specialist medical practitioners	Lower-middle Income	East	Medical Doctors
5	Cardiothoracic Surgeon	144	163	184	0.03	294,042	Kenya	Maximum Estimate	2212 - Specialist medical practitioners	Lower-middle Income	East	Medical Doctors
6	Clinical Officer/Physician Assistant	16,778	18,559	20,930	3.89	2,569	Kenya	Maximum Estimate	3256 - Medical assistants	Lower-middle Income	East	Other Health Workers
7	Clinical Pharmacist	2,318	2,562	2,898	0.54	18,586	Kenya	Maximum Estimate	2262 - Pharmacists	Lower-middle Income	East	Pharmacist
8	Clinical Psychologist	4,898	5,302	5,809	1.07	9,310	Kenya	Maximum Estimate	2634 - Psychologists	Lower-middle Income	East	Other Health Workers
9	Community health worker/Village health worker	69,581	78,305	88,964	16.52	605	Kenya	Maximum Estimate	3253 - Community health workers	Lower-middle Income	East	Other Health Workers
10	Dental Surgery Assistant	7,875	8,783	9,872	1.82	5,497	Kenya	Maximum Estimate	3251 - Dental assistants and therapists	Lower-middle Income	East	Other Health Workers
11	Dental Therapist	5,273	5,876	6,601	1.22	8,222	Kenya	Maximum Estimate	3251 - Dental assistants and therapists	Lower-middle Income	East	Other Health Workers
12	Dentist	6,263	6,815	7,585	1.39	7,172	Kenya	Maximum Estimate	2261 - Dentists	Lower-middle Income	East	Dentist
13	Dermatologist	276	305	337	0.06	160,664	Kenya	Maximum Estimate	2212 - Specialist medical practitioners	Lower-middle Income	East	Medical Doctors
14	Endocrinologist	260	308	383	0.07	139,517	Kenya	Maximum Estimate	2212 - Specialist medical practitioners	Lower-middle Income	East	Medical Doctors
15	ENT Surgeon	1,183	1,331	1,525	0.28	35,389	Kenya	Maximum Estimate	2212 - Specialist medical practitioners	Lower-middle Income	East	Medical Doctors
16	Environmental Health Officer	4,006	4,332	4,674	0.86	11,597	Kenya	Maximum Estimate	2263 - Environmental and occupational health and hygiene professionals	Lower-middle Income	East	Other Health Workers
17	Gastroenterologist	509	531	583	0.11	91,992	Kenya	Maximum Estimate	2212 - Specialist medical practitioners	Lower-middle Income	East	Medical Doctors
18	General Medical Practitioner (Generalist Doctor)	33,699	37,143	41,263	7.63	1,310	Kenya	Maximum Estimate	2211 - Generalist medical practitioners	Lower-middle Income	East	Medical Doctors
19	General Surgeon	689	787	922	0.17	58,616	Kenya	Maximum Estimate	2212 - Specialist medical practitioners	Lower-middle Income	East	Medical Doctors
20	Haematologist	200	213	229	0.04	236,576	Kenya	Maximum Estimate	2212 - Specialist medical practitioners	Lower-middle Income	East	Medical Doctors
21	Health Promoter/Health Educator	1,674	1,836	2,048	0.38	26,242	Kenya	Maximum Estimate	2269 - Health professionals not elsewhere classified	Lower-middle Income	East	Other Health Workers
22	Infectious Diseases Specialist	112	123	138	0.03	391,162	Kenya	Maximum Estimate	2212 - Specialist medical practitioners	Lower-middle Income	East	Medical Doctors
23	Intensive Care Nurse	3,303	3,559	3,835	0.71	14,117	Kenya	Maximum Estimate	2221 - Nursing professionals	Lower-middle Income	East	Nursing Personnel
24	Medical Laboratory Scientist	10,081	11,311	12,993	2.42	4,136	Kenya	Maximum Estimate	3212 - Medical and pathology laboratory technicians	Lower-middle Income	East	Other Health Workers
25	Medical Laboratory Technician	12,510	14,521	17,155	3.20	3,122	Kenya	Maximum Estimate	3212 - Medical and pathology laboratory technicians	Lower-middle Income	East	Other Health Workers
26	Medical Social Worker	2,402	2,600	2,893	0.54	18,474	Kenya	Maximum Estimate	1344 - Social welfare managers	Lower-middle Income	East	Other Health Workers
27	Mental Health Nurse	5,770	6,384	7,370	1.37	7,318	Kenya	Maximum Estimate	2221 - Nursing professionals	Lower-middle Income	East	Nursing Personnel
28	Midwife	56,239	60,270	65,299	12.03	831	Kenya	Maximum Estimate	2222 - Midwifery professionals	Lower-middle Income	East	Midwifery Personnel
29	Nephrologist	792	910	1,096	0.20	48,987	Kenya	Maximum Estimate	2212 - Specialist medical practitioners	Lower-middle Income	East	Medical Doctors
30	Neuro-Surgeon	223	259	313	0.06	171,048	Kenya	Maximum Estimate	2212 - Specialist medical practitioners	Lower-middle Income	East	Medical Doctors
31	Nurse Anaesthetist	1,772	2,072	2,438	0.45	22,146	Kenya	Maximum Estimate	2221 - Nursing professionals	Lower-middle Income	East	Nursing Personnel
32	Nutritionist	9,991	10,384	10,779	1.99	5,019	Kenya	Maximum Estimate	2265 - Dieticians and nutritionists	Lower-middle Income	East	Other Health Workers
33	Obstetrician & Gynaecologist	5,405	6,015	6,805	1.26	7,914	Kenya	Maximum Estimate	2212 - Specialist medical practitioners	Lower-middle Income	East	Medical Doctors
34	Occupational Therapist	1,363	1,573	1,864	0.35	28,878	Kenya	Maximum Estimate	2269 - Health professionals not elsewhere classified	Lower-middle Income	East	Other Health Workers
35	Oncology Nurse	661	767	919	0.17	58,518	Kenya	Maximum Estimate	2221 - Nursing professionals	Lower-middle Income	East	Nursing Personnel
36	Operating Theatre Nurse	8,966	10,168	12,041	2.24	4,464	Kenya	Maximum Estimate	2221 - Nursing professionals	Lower-middle Income	East	Nursing Personnel
37	Ophthalmic Nurse	745	830	942	0.17	57,269	Kenya	Maximum Estimate	2221 - Nursing professionals	Lower-middle Income	East	Nursing Personnel
38	Ophthalmologist	343	390	454	0.08	118,656	Kenya	Maximum Estimate	2212 - Specialist medical practitioners	Lower-middle Income	East	Medical Doctors
39	Optometrist	1,131	1,290	1,483	0.27	36,454	Kenya	Maximum Estimate	2267 - Optometrists and ophthalmic opticians	Lower-middle Income	East	Other Health Workers
40	Orthopaedic Nurse	1,258	1,454	1,682	0.31	32,156	Kenya	Maximum Estimate	2221 - Nursing professionals	Lower-middle Income	East	Nursing Personnel
41	Orthopaedic Surgeon	2,963	3,430	4,018	0.74	13,441	Kenya	Maximum Estimate	2212 - Specialist medical practitioners	Lower-middle Income	East	Medical Doctors
42	Orthopaedic Technologist	2,199	2,416	2,656	0.49	20,395	Kenya	Maximum Estimate	3214 - Medical and dental prosthetic technicians	Lower-middle Income	East	Other Health Workers
43	Paediatric Nurse	7,622	8,111	8,594	1.58	6,310	Kenya	Maximum Estimate	2221 - Nursing professionals	Lower-middle Income	East	Nursing Personnel
44	Paediatric Surgeon	242	253	262	0.05	206,667	Kenya	Maximum Estimate	2212 - Specialist medical practitioners	Lower-middle Income	East	Medical Doctors
45	Paediatrician	3,466	3,782	4,186	0.77	12,912	Kenya	Maximum Estimate	2212 - Specialist medical practitioners	Lower-middle Income	East	Medical Doctors
46	Pathologist	862	969	1,138	0.21	47,330	Kenya	Maximum Estimate	2212 - Specialist medical practitioners	Lower-middle Income	East	Medical Doctors
47	Pharmacist	11,025	11,835	12,785	2.37	4,226	Kenya	Maximum Estimate	2262 - Pharmacists	Lower-middle Income	East	Pharmacist
48	Pharmacy Technician	10,101	11,042	12,149	2.25	4,451	Kenya	Maximum Estimate	3213 - Pharmaceutical technicians and assistants	Lower-middle Income	East	Other Health Workers
49	Physician	7,893	8,788	9,912	1.84	5,449	Kenya	Maximum Estimate	2212 - Specialist medical practitioners	Lower-middle Income	East	Medical Doctors
50	Physiotherapist	2,029	2,238	2,506	0.46	21,550	Kenya	Maximum Estimate	2264 - Physiotherapists	Lower-middle Income	East	Other Health Workers
51	Plastic Surgeon	819	956	1,113	0.21	48,598	Kenya	Maximum Estimate	2212 - Specialist medical practitioners	Lower-middle Income	East	Medical Doctors
52	Psychiatrist	4,561	4,983	5,641	1.04	9,574	Kenya	Maximum Estimate	2212 - Specialist medical practitioners	Lower-middle Income	East	Medical Doctors
53	Radiation Oncologist	226	267	323	0.06	166,752	Kenya	Maximum Estimate	2212 - Specialist medical practitioners	Lower-middle Income	East	Medical Doctors
54	Radiographer (Diagnostics and Therapy)	6,222	7,104	8,344	1.55	6,448	Kenya	Maximum Estimate	3211 - Medical imaging and therapeutic equipment technicians	Lower-middle Income	East	Other Health Workers
55	Radiologist	2,362	2,773	3,346	0.63	15,954	Kenya	Maximum Estimate	2212 - Specialist medical practitioners	Lower-middle Income	East	Medical Doctors
56	Registered General Nurse / State Certified Nurse	182,784	201,421	226,608	42.06	238	Kenya	Maximum Estimate	2221 - Nursing professionals	Lower-middle Income	East	Nursing Personnel
57	Renal Nurse	15,755	18,122	21,855	4.07	2,457	Kenya	Maximum Estimate	2221 - Nursing professionals	Lower-middle Income	East	Nursing Personnel
58	Respiratory Physician	660	727	804	0.15	67,372	Kenya	Maximum Estimate	2212 - Specialist medical practitioners	Lower-middle Income	East	Medical Doctors
59	Rheumatologist	222	248	277	0.05	195,257	Kenya	Maximum Estimate	2212 - Specialist medical practitioners	Lower-middle Income	East	Medical Doctors
60	Speech Therapist	705	764	847	0.16	63,664	Kenya	Maximum Estimate	2266 - Audiologists and speech therapists	Lower-middle Income	East	Other Health Workers
61	Urologist	147	181	234	0.04	226,698	Kenya	Maximum Estimate	2212 - Specialist medical practitioners	Lower-middle Income	East	Medical Doctors
1	Anaesthesiologist	47	52	58	0.27	37,496	Lesotho	Maximum Estimate	2212 - Specialist medical practitioners	Lower-middle Income	Southern	Medical Doctors
2	Associate Nurse/Enrolled Nurse/Nursing Assistant	4,622	4,905	5,223	24.25	412	Lesotho	Maximum Estimate	3221 - Nursing associate professionals	Lower-middle Income	Southern	Nursing Personnel
3	Audiologist	16	17	18	0.08	121,638	Lesotho	Maximum Estimate	2266 - Audiologists and speech therapists	Lower-middle Income	Southern	Other Health Workers
4	Cardiologist	41	43	45	0.21	47,742	Lesotho	Maximum Estimate	2212 - Specialist medical practitioners	Lower-middle Income	Southern	Medical Doctors
5	Cardiothoracic Surgeon	9	9	10	0.05	210,070	Lesotho	Maximum Estimate	2212 - Specialist medical practitioners	Lower-middle Income	Southern	Medical Doctors
6	Clinical Officer/Physician Assistant	592	626	669	3.11	3,220	Lesotho	Maximum Estimate	3256 - Medical assistants	Lower-middle Income	Southern	Other Health Workers
7	Clinical Pharmacist	122	132	146	0.69	14,594	Lesotho	Maximum Estimate	2262 - Pharmacists	Lower-middle Income	Southern	Pharmacist
8	Clinical Psychologist	195	204	216	1.00	9,979	Lesotho	Maximum Estimate	2634 - Psychologists	Lower-middle Income	Southern	Other Health Workers
9	Community health worker/Village health worker	2,361	2,436	2,502	11.64	859	Lesotho	Maximum Estimate	3253 - Community health workers	Lower-middle Income	Southern	Other Health Workers
10	Dental Surgery Assistant	404	433	462	2.14	4,673	Lesotho	Maximum Estimate	3251 - Dental assistants and therapists	Lower-middle Income	Southern	Other Health Workers
11	Dental Therapist	270	290	309	1.43	6,983	Lesotho	Maximum Estimate	3251 - Dental assistants and therapists	Lower-middle Income	Southern	Other Health Workers
12	Dentist	366	385	404	1.87	5,354	Lesotho	Maximum Estimate	2261 - Dentists	Lower-middle Income	Southern	Dentist
13	Dermatologist	12	13	14	0.06	159,312	Lesotho	Maximum Estimate	2212 - Specialist medical practitioners	Lower-middle Income	Southern	Medical Doctors
14	Endocrinologist	22	25	28	0.13	76,026	Lesotho	Maximum Estimate	2212 - Specialist medical practitioners	Lower-middle Income	Southern	Medical Doctors
15	ENT Surgeon	53	57	61	0.28	35,280	Lesotho	Maximum Estimate	2212 - Specialist medical practitioners	Lower-middle Income	Southern	Medical Doctors
16	Environmental Health Officer	160	167	173	0.80	12,445	Lesotho	Maximum Estimate	2263 - Environmental and occupational health and hygiene professionals	Lower-middle Income	Southern	Other Health Workers
17	Gastroenterologist	43	42	42	0.20	50,807	Lesotho	Maximum Estimate	2212 - Specialist medical practitioners	Lower-middle Income	Southern	Medical Doctors
18	General Medical Practitioner (Generalist Doctor)	1,497	1,587	1,689	7.85	1,273	Lesotho	Maximum Estimate	2211 - Generalist medical practitioners	Lower-middle Income	Southern	Medical Doctors
19	General Surgeon	56	60	64	0.30	33,595	Lesotho	Maximum Estimate	2212 - Specialist medical practitioners	Lower-middle Income	Southern	Medical Doctors
20	Haematologist	8	9	9	0.04	238,018	Lesotho	Maximum Estimate	2212 - Specialist medical practitioners	Lower-middle Income	Southern	Medical Doctors
21	Health Promoter/Health Educator	55	59	65	0.31	32,718	Lesotho	Maximum Estimate	2269 - Health professionals not elsewhere classified	Lower-middle Income	Southern	Other Health Workers
22	Infectious Diseases Specialist	6	6	7	0.03	318,449	Lesotho	Maximum Estimate	2212 - Specialist medical practitioners	Lower-middle Income	Southern	Medical Doctors
23	Intensive Care Nurse	110	116	122	0.57	17,679	Lesotho	Maximum Estimate	2221 - Nursing professionals	Lower-middle Income	Southern	Nursing Personnel
24	Medical Laboratory Scientist	690	751	831	3.89	2,570	Lesotho	Maximum Estimate	3212 - Medical and pathology laboratory technicians	Lower-middle Income	Southern	Other Health Workers
25	Medical Laboratory Technician	546	590	645	3.02	3,311	Lesotho	Maximum Estimate	3212 - Medical and pathology laboratory technicians	Lower-middle Income	Southern	Other Health Workers
26	Medical Social Worker	114	125	146	0.73	13,780	Lesotho	Maximum Estimate	1344 - Social welfare managers	Lower-middle Income	Southern	Other Health Workers

S/N	Health Professionals	2022	2026	2030	Density per 10,000 population	Required Population ratio (1 professional is to xxx population)	Name of Country	Modelling Scenario	ISCO-08 Match	Income Group Classification	Sub-Regional Grouping	SDG 3c Occupation
27	Mental Health Nurse	297	316	342	1.58	6,323	Lesotho	Maximum Estimate	2221 - Nursing professionals	Lower-middle Income	Southern	Nursing Personnel
28	Midwife	2,268	2,353	2,423	11.24	890	Lesotho	Maximum Estimate	2222 - Midwifery professionals	Lower-middle Income	Southern	Midwifery Personnel
29	Nephrologist	52	58	67	0.31	32,509	Lesotho	Maximum Estimate	2212 - Specialist medical practitioners	Lower-middle Income	Southern	Medical Doctors
30	Neuro-Surgeon	18	20	22	0.10	97,209	Lesotho	Maximum Estimate	2212 - Specialist medical practitioners	Lower-middle Income	Southern	Medical Doctors
31	Nurse Anaesthetist	139	158	178	0.83	12,100	Lesotho	Maximum Estimate	2221 - Nursing professionals	Lower-middle Income	Southern	Nursing Personnel
32	Nutritionist	286	283	278	1.30	7,705	Lesotho	Maximum Estimate	2265 - Dieticians and nutritionists	Lower-middle Income	Southern	Other Health Workers
33	Obstetrician & Gynaecologist	273	302	345	1.65	6,075	Lesotho	Maximum Estimate	2212 - Specialist medical practitioners	Lower-middle Income	Southern	Medical Doctors
34	Occupational Therapist	76	84	95	0.44	22,627	Lesotho	Maximum Estimate	2269 - Health professionals not elsewhere classified	Lower-middle Income	Southern	Other Health Workers
35	Oncology Nurse	47	53	61	0.28	35,565	Lesotho	Maximum Estimate	2221 - Nursing professionals	Lower-middle Income	Southern	Nursing Personnel
36	Operating Theatre Nurse	485	527	583	2.70	3,707	Lesotho	Maximum Estimate	2221 - Nursing professionals	Lower-middle Income	Southern	Nursing Personnel
37	Ophthalmic Nurse	54	58	62	0.29	34,805	Lesotho	Maximum Estimate	2221 - Nursing professionals	Lower-middle Income	Southern	Nursing Personnel
38	Ophthalmologist	23	25	28	0.13	78,024	Lesotho	Maximum Estimate	2212 - Specialist medical practitioners	Lower-middle Income	Southern	Medical Doctors
39	Optometrist	122	131	141	0.65	15,332	Lesotho	Maximum Estimate	2267 - Optometrists and ophthalmic opticians	Lower-middle Income	Southern	Other Health Workers
40	Orthopaedic Nurse	82	95	109	0.50	19,806	Lesotho	Maximum Estimate	2221 - Nursing professionals	Lower-middle Income	Southern	Nursing Personnel
41	Orthopaedic Surgeon	144	159	177	0.82	12,234	Lesotho	Maximum Estimate	2212 - Specialist medical practitioners	Lower-middle Income	Southern	Medical Doctors
42	Orthopaedic Technologist	101	109	118	0.55	18,308	Lesotho	Maximum Estimate	3214 - Medical and dental prosthetic technicians	Lower-middle Income	Southern	Other Health Workers
43	Paediatric Nurse	257	264	265	1.23	8,108	Lesotho	Maximum Estimate	2221 - Nursing professionals	Lower-middle Income	Southern	Nursing Personnel
44	Paediatric Surgeon	9	9	9	0.04	234,125	Lesotho	Maximum Estimate	2212 - Specialist medical practitioners	Lower-middle Income	Southern	Medical Doctors
45	Paediatrician	105	113	123	0.57	17,524	Lesotho	Maximum Estimate	2212 - Specialist medical practitioners	Lower-middle Income	Southern	Medical Doctors
46	Pathologist	49	53	59	0.27	36,589	Lesotho	Maximum Estimate	2212 - Specialist medical practitioners	Lower-middle Income	Southern	Medical Doctors
47	Pharmacist	306	311	321	1.51	6,608	Lesotho	Maximum Estimate	2262 - Pharmacists	Lower-middle Income	Southern	Pharmacist
48	Pharmacy Technician	407	431	461	2.15	4,644	Lesotho	Maximum Estimate	3213 - Pharmaceutical technicians and assistants	Lower-middle Income	Southern	Other Health Workers
49	Physician	383	408	439	2.04	4,913	Lesotho	Maximum Estimate	2212 - Specialist medical practitioners	Lower-middle Income	Southern	Medical Doctors
50	Physiotherapist	94	101	109	0.51	19,792	Lesotho	Maximum Estimate	2264 - Physiotherapists	Lower-middle Income	Southern	Other Health Workers
51	Plastic Surgeon	38	41	45	0.21	48,421	Lesotho	Maximum Estimate	2212 - Specialist medical practitioners	Lower-middle Income	Southern	Medical Doctors
52	Psychiatrist	213	225	241	1.12	8,946	Lesotho	Maximum Estimate	2212 - Specialist medical practitioners	Lower-middle Income	Southern	Medical Doctors
53	Radiation Oncologist	19	22	25	0.12	85,479	Lesotho	Maximum Estimate	2212 - Specialist medical practitioners	Lower-middle Income	Southern	Medical Doctors
54	Radiographer (Diagnostics and Therapy)	366	405	460	2.17	4,615	Lesotho	Maximum Estimate	3211 - Medical imaging and therapeutic equipment technicians	Lower-middle Income	Southern	Other Health Workers
55	Radiologist	146	172	215	1.06	9,467	Lesotho	Maximum Estimate	2212 - Specialist medical practitioners	Lower-middle Income	Southern	Medical Doctors
56	Registered General Nurse / State Certified Nurse	8,322	8,956	9,713	45.07	222	Lesotho	Maximum Estimate	2221 - Nursing professionals	Lower-middle Income	Southern	Nursing Personnel
57	Renal Nurse	1,026	1,146	1,308	6.05	1,654	Lesotho	Maximum Estimate	2221 - Nursing professionals	Lower-middle Income	Southern	Nursing Personnel
58	Respiratory Physician	17	18	20	0.09	110,203	Lesotho	Maximum Estimate	2212 - Specialist medical practitioners	Lower-middle Income	Southern	Medical Doctors
59	Rheumatologist	10	11	12	0.05	186,860	Lesotho	Maximum Estimate	2212 - Specialist medical practitioners	Lower-middle Income	Southern	Medical Doctors
60	Speech Therapist	31	33	35	0.16	61,557	Lesotho	Maximum Estimate	2266 - Audiologists and speech therapists	Lower-middle Income	Southern	Other Health Workers
61	Urologist	9	11	13	0.06	158,883	Lesotho	Maximum Estimate	2212 - Specialist medical practitioners	Lower-middle Income	Southern	Medical Doctors
1	Anaesthesiologist	79	91	105	0.21	48,303	Liberia	Maximum Estimate	2212 - Specialist medical practitioners	Low Income	West	Medical Doctors
2	Associate Nurse/Enrolled Nurse/Nursing Assistant	9,211	10,266	11,480	22.37	447	Liberia	Maximum Estimate	3221 - Nursing associate professionals	Low Income	West	Nursing Personnel
3	Audiologist	36	39	43	0.08	118,444	Liberia	Maximum Estimate	2266 - Audiologists and speech therapists	Low Income	West	Other Health Workers
4	Cardiologist	72	82	93	0.18	54,750	Liberia	Maximum Estimate	2212 - Specialist medical practitioners	Low Income	West	Medical Doctors
5	Cardiothoracic Surgeon	13	15	18	0.03	289,902	Liberia	Maximum Estimate	2212 - Specialist medical practitioners	Low Income	West	Medical Doctors
6	Clinical Officer/Physician Assistant	4,063	4,804	5,699	11.00	909	Liberia	Maximum Estimate	3256 - Medical assistants	Low Income	West	Other Health Workers
7	Clinical Pharmacist	240	263	294	0.58	17,277	Liberia	Maximum Estimate	2262 - Pharmacists	Low Income	West	Pharmacist
8	Clinical Psychologist	511	554	609	1.20	8,358	Liberia	Maximum Estimate	2634 - Psychologists	Low Income	West	Other Health Workers
9	Community health worker/Village health worker	12,619	15,157	18,030	33.82	296	Liberia	Maximum Estimate	3253 - Community health workers	Low Income	West	Other Health Workers
10	Dental Surgery Assistant	898	1,008	1,139	2.23	4,477	Liberia	Maximum Estimate	3251 - Dental assistants and therapists	Low Income	West	Other Health Workers
11	Dental Therapist	601	674	761	1.49	6,697	Liberia	Maximum Estimate	3251 - Dental assistants and therapists	Low Income	West	Other Health Workers
12	Dentist	733	812	914	1.79	5,580	Liberia	Maximum Estimate	2261 - Dentists	Low Income	West	Dentist
13	Dermatologist	27	30	33	0.06	154,104	Liberia	Maximum Estimate	2212 - Specialist medical practitioners	Low Income	West	Medical Doctors
14	Endocrinologist	32	38	45	0.09	111,202	Liberia	Maximum Estimate	2212 - Specialist medical practitioners	Low Income	West	Medical Doctors
15	ENT Surgeon	96	108	122	0.24	41,555	Liberia	Maximum Estimate	2212 - Specialist medical practitioners	Low Income	West	Medical Doctors
16	Environmental Health Officer	377	410	446	0.87	11,437	Liberia	Maximum Estimate	2263 - Environmental and occupational health and hygiene professionals	Low Income	West	Other Health Workers
17	Gastroenterologist	114	115	119	0.24	42,544	Liberia	Maximum Estimate	2212 - Specialist medical practitioners	Low Income	West	Medical Doctors
18	General Medical Practitioner (Generalist Doctor)	3,868	4,347	4,904	9.51	1,051	Liberia	Maximum Estimate	2211 - Generalist medical practitioners	Low Income	West	Medical Doctors
19	General Surgeon	73	83	96	0.19	52,709	Liberia	Maximum Estimate	2212 - Specialist medical practitioners	Low Income	West	Medical Doctors
20	Haematologist	18	19	19	0.04	260,867	Liberia	Maximum Estimate	2212 - Specialist medical practitioners	Low Income	West	Medical Doctors
21	Health Promoter/Health Educator	285	323	369	0.71	14,102	Liberia	Maximum Estimate	2269 - Health professionals not elsewhere classified	Low Income	West	Other Health Workers
22	Infectious Diseases Specialist	12	12	14	0.03	376,125	Liberia	Maximum Estimate	2212 - Specialist medical practitioners	Low Income	West	Medical Doctors
23	Intensive Care Nurse	227	246	266	0.52	19,132	Liberia	Maximum Estimate	2221 - Nursing professionals	Low Income	West	Nursing Personnel
24	Medical Laboratory Scientist	1,203	1,393	1,624	3.11	3,213	Liberia	Maximum Estimate	3212 - Medical and pathology laboratory technicians	Low Income	West	Other Health Workers
25	Medical Laboratory Technician	2,960	3,595	4,317	8.07	1,240	Liberia	Maximum Estimate	3212 - Medical and pathology laboratory technicians	Low Income	West	Other Health Workers
26	Medical Social Worker	274	282	298	0.59	16,955	Liberia	Maximum Estimate	1344 - Social welfare managers	Low Income	West	Other Health Workers
27	Mental Health Nurse	525	581	662	1.30	7,665	Liberia	Maximum Estimate	2221 - Nursing professionals	Low Income	West	Nursing Personnel
28	Midwife	6,549	7,026	7,571	14.85	673	Liberia	Maximum Estimate	2222 - Midwifery professionals	Low Income	West	Midwifery Personnel
29	Nephrologist	78	89	105	0.21	48,409	Liberia	Maximum Estimate	2212 - Specialist medical practitioners	Low Income	West	Medical Doctors
30	Neuro-Surgeon	29	33	38	0.07	135,444	Liberia	Maximum Estimate	2212 - Specialist medical practitioners	Low Income	West	Medical Doctors
31	Nurse Anaesthetist	160	184	213	0.42	23,908	Liberia	Maximum Estimate	2221 - Nursing professionals	Low Income	West	Nursing Personnel
32	Nutritionist	680	684	686	1.35	7,390	Liberia	Maximum Estimate	2265 - Dieticians and nutritionists	Low Income	West	Other Health Workers
33	Obstetrician & Gynaecologist	487	544	613	1.21	8,290	Liberia	Maximum Estimate	2212 - Specialist medical practitioners	Low Income	West	Medical Doctors
34	Occupational Therapist	146	168	197	0.39	25,771	Liberia	Maximum Estimate	2269 - Health professionals not elsewhere classified	Low Income	West	Other Health Workers
35	Oncology Nurse	60	68	80	0.16	63,577	Liberia	Maximum Estimate	2221 - Nursing professionals	Low Income	West	Nursing Personnel
36	Operating Theatre Nurse	841	958	1,119	2.21	4,533	Liberia	Maximum Estimate	2221 - Nursing professionals	Low Income	West	Nursing Personnel
37	Ophthalmic Nurse	87	97	110	0.22	46,227	Liberia	Maximum Estimate	2221 - Nursing professionals	Low Income	West	Nursing Personnel
38	Ophthalmologist	33	38	44	0.09	116,540	Liberia	Maximum Estimate	2212 - Specialist medical practitioners	Low Income	West	Medical Doctors
39	Optometrist	134	153	176	0.35	28,975	Liberia	Maximum Estimate	2267 - Optometrists and ophthalmic opticians	Low Income	West	Other Health Workers
40	Orthopaedic Nurse	154	180	210	0.41	24,241	Liberia	Maximum Estimate	2221 - Nursing professionals	Low Income	West	Nursing Personnel
41	Orthopaedic Surgeon	260	314	382	0.75	13,245	Liberia	Maximum Estimate	2212 - Specialist medical practitioners	Low Income	West	Medical Doctors
42	Orthopaedic Technologist	216	240	267	0.52	19,054	Liberia	Maximum Estimate	3214 - Medical and dental prosthetic technicians	Low Income	West	Other Health Workers
43	Paediatric Nurse	675	714	750	1.47	6,795	Liberia	Maximum Estimate	2221 - Nursing professionals	Low Income	West	Nursing Personnel
44	Paediatric Surgeon	23	24	24	0.05	208,397	Liberia	Maximum Estimate	2212 - Specialist medical practitioners	Low Income	West	Medical Doctors
45	Paediatrician	283	312	348	0.68	14,639	Liberia	Maximum Estimate	2212 - Specialist medical practitioners	Low Income	West	Medical Doctors
46	Pathologist	59	67	79	0.16	64,366	Liberia	Maximum Estimate	2212 - Specialist medical practitioners	Low Income	West	Medical Doctors
47	Pharmacist	1,613	1,751	1,928	3.82	2,619	Liberia	Maximum Estimate	2262 - Pharmacists	Low Income	West	Pharmacist
48	Pharmacy Technician	1,177	1,270	1,382	2.72	3,673	Liberia	Maximum Estimate	3213 - Pharmaceutical technicians and assistants	Low Income	West	Other Health Workers
49	Physician	1,007	1,130	1,277	2.49	4,019	Liberia	Maximum Estimate	2212 - Specialist medical practitioners	Low Income	West	Medical Doctors

S/N	Health Professionals	2022	2026	2030	Density per 10,000 population	Required Population ratio (1 professional is to xxx population)	Name of Country	Modelling Scenario	ISCO-08 Match	Income Group Classification	Sub-Regional Grouping	SDG 3c Occupation
50	Physiotherapist	196	218	244	0.48	20,837	Liberia	Maximum Estimate	2264 - Physiotherapists	Low Income	West	Other Health Workers
51	Plastic Surgeon	55	64	75	0.15	67,782	Liberia	Maximum Estimate	2212 - Specialist medical practitioners	Low Income	West	Medical Doctors
52	Psychiatrist	396	432	483	0.95	10,520	Liberia	Maximum Estimate	2212 - Specialist medical practitioners	Low Income	West	Medical Doctors
53	Radiation Oncologist	18	21	24	0.05	211,460	Liberia	Maximum Estimate	2212 - Specialist medical practitioners	Low Income	West	Medical Doctors
54	Radiographer (Diagnostics and Therapy)	683	773	892	1.76	5,691	Liberia	Maximum Estimate	3211 - Medical imaging and therapeutic equipment technicians	Low Income	West	Other Health Workers
55	Radiologist	260	299	350	0.69	14,460	Liberia	Maximum Estimate	2212 - Specialist medical practitioners	Low Income	West	Medical Doctors
56	Registered General Nurse / State Certified Nurse	18,609	20,225	22,353	44.11	227	Liberia	Maximum Estimate	2221 - Nursing professionals	Low Income	West	Nursing Personnel
57	Renal Nurse	1,530	1,744	2,055	4.06	2,466	Liberia	Maximum Estimate	2221 - Nursing professionals	Low Income	West	Nursing Personnel
58	Respiratory Physician	53	59	66	0.13	77,589	Liberia	Maximum Estimate	2212 - Specialist medical practitioners	Low Income	West	Medical Doctors
59	Rheumatologist	20	22	25	0.05	206,109	Liberia	Maximum Estimate	2212 - Specialist medical practitioners	Low Income	West	Medical Doctors
60	Speech Therapist	66	72	79	0.16	64,486	Liberia	Maximum Estimate	2266 - Audiologists and speech therapists	Low Income	West	Other Health Workers
61	Urologist	16	19	23	0.05	214,248	Liberia	Maximum Estimate	2212 - Specialist medical practitioners	Low Income	West	Medical Doctors
1	Anaesthesiologist	415	470	532	0.19	52,465	Madagascar	Maximum Estimate	2212 - Specialist medical practitioners	Low Income	Southern	Medical Doctors
2	Associate Nurse/Enrolled Nurse/Nursing Assistant	42,356	46,018	50,172	18.00	556	Madagascar	Maximum Estimate	3221 - Nursing associate professionals	Low Income	Southern	Nursing Personnel
3	Audiologist	202	226	251	0.09	111,344	Madagascar	Maximum Estimate	2266 - Audiologists and speech therapists	Low Income	Southern	Other Health Workers
4	Cardiologist	415	476	544	0.19	51,329	Madagascar	Maximum Estimate	2212 - Specialist medical practitioners	Low Income	Southern	Medical Doctors
5	Cardiothoracic Surgeon	88	99	111	0.04	250,729	Madagascar	Maximum Estimate	2212 - Specialist medical practitioners	Low Income	Southern	Medical Doctors
6	Clinical Officer/Physician Assistant	5,382	5,826	6,383	2.29	4,358	Madagascar	Maximum Estimate	3256 - Medical assistants	Low Income	Southern	Other Health Workers
7	Clinical Pharmacist	1,245	1,391	1,572	0.57	17,686	Madagascar	Maximum Estimate	2262 - Pharmacists	Low Income	Southern	Pharmacist
8	Clinical Psychologist	2,612	2,920	3,279	1.18	8,511	Madagascar	Maximum Estimate	2634 - Psychologists	Low Income	Southern	Other Health Workers
9	Community health worker/Village health worker	35,935	38,858	41,894	15.00	667	Madagascar	Maximum Estimate	3253 - Community health workers	Low Income	Southern	Other Health Workers
10	Dental Surgery Assistant	4,627	5,194	5,832	2.09	4,794	Madagascar	Maximum Estimate	3251 - Dental assistants and therapists	Low Income	Southern	Other Health Workers
11	Dental Therapist	3,110	3,491	3,918	1.40	7,135	Madagascar	Maximum Estimate	3251 - Dental assistants and therapists	Low Income	Southern	Other Health Workers
12	Dentist	4,572	5,106	5,734	2.05	4,877	Madagascar	Maximum Estimate	2261 - Dentists	Low Income	Southern	Dentist
13	Dermatologist	135	152	169	0.06	165,116	Madagascar	Maximum Estimate	2212 - Specialist medical practitioners	Low Income	Southern	Medical Doctors
14	Endocrinologist	125	149	181	0.07	152,878	Madagascar	Maximum Estimate	2212 - Specialist medical practitioners	Low Income	Southern	Medical Doctors
15	ENT Surgeon	542	615	704	0.25	39,573	Madagascar	Maximum Estimate	2212 - Specialist medical practitioners	Low Income	Southern	Medical Doctors
16	Environmental Health Officer	2,063	2,266	2,478	0.89	11,279	Madagascar	Maximum Estimate	2263 - Environmental and occupational health and hygiene professionals	Low Income	Southern	Other Health Workers
17	Gastroenterologist	303	313	334	0.12	82,967	Madagascar	Maximum Estimate	2212 - Specialist medical practitioners	Low Income	Southern	Medical Doctors
18	General Medical Practitioner (Generalist Doctor)	15,014	16,593	18,362	6.58	1,519	Madagascar	Maximum Estimate	2211 - Generalist medical practitioners	Low Income	Southern	Medical Doctors
19	General Surgeon	329	382	446	0.16	62,423	Madagascar	Maximum Estimate	2212 - Specialist medical practitioners	Low Income	Southern	Medical Doctors
20	Haematologist	150	167	185	0.07	150,724	Madagascar	Maximum Estimate	2212 - Specialist medical practitioners	Low Income	Southern	Medical Doctors
21	Health Promoter/Health Educator	517	565	624	0.22	44,484	Madagascar	Maximum Estimate	2269 - Health professionals not elsewhere classified	Low Income	Southern	Other Health Workers
22	Infectious Diseases Specialist	45	49	55	0.02	504,513	Madagascar	Maximum Estimate	2212 - Specialist medical practitioners	Low Income	Southern	Medical Doctors
23	Intensive Care Nurse	1,499	1,649	1,812	0.65	15,391	Madagascar	Maximum Estimate	2221 - Nursing professionals	Low Income	Southern	Nursing Personnel
24	Medical Laboratory Scientist	4,423	4,995	5,709	2.05	4,867	Madagascar	Maximum Estimate	3212 - Medical and pathology laboratory technicians	Low Income	Southern	Other Health Workers
25	Medical Laboratory Technician	5,204	5,748	6,387	2.30	4,355	Madagascar	Maximum Estimate	3212 - Medical and pathology laboratory technicians	Low Income	Southern	Other Health Workers
26	Medical Social Worker	1,377	1,457	1,576	0.57	17,422	Madagascar	Maximum Estimate	1344 - Social welfare managers	Low Income	Southern	Other Health Workers
27	Mental Health Nurse	3,237	3,646	4,196	1.51	6,639	Madagascar	Maximum Estimate	2221 - Nursing professionals	Low Income	Southern	Nursing Personnel
28	Midwife	28,960	31,444	34,155	12.22	818	Madagascar	Maximum Estimate	2222 - Midwifery professionals	Low Income	Southern	Midwifery Personnel
29	Nephrologist	358	411	484	0.17	57,493	Madagascar	Maximum Estimate	2212 - Specialist medical practitioners	Low Income	Southern	Medical Doctors
30	Neuro-Surgeon	124	141	165	0.06	167,974	Madagascar	Maximum Estimate	2212 - Specialist medical practitioners	Low Income	Southern	Medical Doctors
31	Nurse Anaesthetist	882	1,013	1,162	0.42	24,015	Madagascar	Maximum Estimate	2221 - Nursing professionals	Low Income	Southern	Nursing Personnel
32	Nutritionist	6,116	6,231	6,327	2.28	4,396	Madagascar	Maximum Estimate	2265 - Dieticians and nutritionists	Low Income	Southern	Other Health Workers
33	Obstetrician & Gynaecologist	2,668	3,010	3,426	1.23	8,101	Madagascar	Maximum Estimate	2212 - Specialist medical practitioners	Low Income	Southern	Medical Doctors
34	Occupational Therapist	732	830	950	0.34	29,337	Madagascar	Maximum Estimate	2269 - Health professionals not elsewhere classified	Low Income	Southern	Other Health Workers
35	Oncology Nurse	305	351	409	0.15	68,040	Madagascar	Maximum Estimate	2221 - Nursing professionals	Low Income	Southern	Nursing Personnel
36	Operating Theatre Nurse	4,156	4,705	5,450	1.96	5,106	Madagascar	Maximum Estimate	2221 - Nursing professionals	Low Income	Southern	Nursing Personnel
37	Ophthalmic Nurse	435	493	560	0.20	49,773	Madagascar	Maximum Estimate	2221 - Nursing professionals	Low Income	Southern	Nursing Personnel
38	Ophthalmologist	160	183	212	0.08	131,420	Madagascar	Maximum Estimate	2212 - Specialist medical practitioners	Low Income	Southern	Medical Doctors
39	Optometrist	695	800	920	0.33	30,311	Madagascar	Maximum Estimate	2267 - Optometrists and ophthalmic opticians	Low Income	Southern	Other Health Workers
40	Orthopaedic Nurse	816	929	1,055	0.38	26,470	Madagascar	Maximum Estimate	2221 - Nursing professionals	Low Income	Southern	Nursing Personnel
41	Orthopaedic Surgeon	1,315	1,517	1,758	0.63	15,861	Madagascar	Maximum Estimate	2212 - Specialist medical practitioners	Low Income	Southern	Medical Doctors
42	Orthopaedic Technologist	1,222	1,359	1,507	0.54	18,534	Madagascar	Maximum Estimate	3214 - Medical and dental prosthetic technicians	Low Income	Southern	Other Health Workers
43	Paediatric Nurse	5,061	5,436	5,791	2.07	4,829	Madagascar	Maximum Estimate	2221 - Nursing professionals	Low Income	Southern	Nursing Personnel
44	Paediatric Surgeon	140	151	161	0.06	173,371	Madagascar	Maximum Estimate	2212 - Specialist medical practitioners	Low Income	Southern	Medical Doctors
45	Paediatrician	2,513	2,713	2,933	1.05	9,516	Madagascar	Maximum Estimate	2212 - Specialist medical practitioners	Low Income	Southern	Medical Doctors
46	Pathologist	386	438	508	0.18	54,831	Madagascar	Maximum Estimate	2212 - Specialist medical practitioners	Low Income	Southern	Medical Doctors
47	Pharmacist	3,370	3,566	3,795	1.37	7,320	Madagascar	Maximum Estimate	2262 - Pharmacists	Low Income	Southern	Pharmacist
48	Pharmacy Technician	4,381	4,852	5,385	1.93	5,178	Madagascar	Maximum Estimate	3213 - Pharmaceutical technicians and assistants	Low Income	Southern	Other Health Workers
49	Physician	4,086	4,526	5,039	1.81	5,534	Madagascar	Maximum Estimate	2212 - Specialist medical practitioners	Low Income	Southern	Medical Doctors
50	Physiotherapist	1,079	1,201	1,344	0.48	20,751	Madagascar	Maximum Estimate	2264 - Physiotherapists	Low Income	Southern	Other Health Workers
51	Plastic Surgeon	706	817	941	0.34	29,676	Madagascar	Maximum Estimate	2212 - Specialist medical practitioners	Low Income	Southern	Medical Doctors
52	Psychiatrist	2,398	2,647	2,975	1.07	9,366	Madagascar	Maximum Estimate	2212 - Specialist medical practitioners	Low Income	Southern	Medical Doctors
53	Radiation Oncologist	105	122	143	0.05	195,027	Madagascar	Maximum Estimate	2212 - Specialist medical practitioners	Low Income	Southern	Medical Doctors
54	Radiographer (Diagnostics and Therapy)	2,949	3,378	3,929	1.42	7,064	Madagascar	Maximum Estimate	3211 - Medical imaging and therapeutic equipment technicians	Low Income	Southern	Other Health Workers
55	Radiologist	1,131	1,331	1,598	0.58	17,152	Madagascar	Maximum Estimate	2212 - Specialist medical practitioners	Low Income	Southern	Medical Doctors
56	Registered General Nurse / State Certified Nurse	81,672	90,806	101,650	36.46	274	Madagascar	Maximum Estimate	2221 - Nursing professionals	Low Income	Southern	Nursing Personnel
57	Renal Nurse	7,038	8,094	9,542	3.43	2,916	Madagascar	Maximum Estimate	2221 - Nursing professionals	Low Income	Southern	Nursing Personnel
58	Respiratory Physician	612	652	694	0.25	40,190	Madagascar	Maximum Estimate	2212 - Specialist medical practitioners	Low Income	Southern	Medical Doctors
59	Rheumatologist	107	121	136	0.05	205,097	Madagascar	Maximum Estimate	2212 - Specialist medical practitioners	Low Income	Southern	Medical Doctors
60	Speech Therapist	385	424	473	0.17	58,900	Madagascar	Maximum Estimate	2266 - Audiologists and speech therapists	Low Income	Southern	Other Health Workers
61	Urologist	57	71	90	0.03	303,732	Madagascar	Maximum Estimate	2212 - Specialist medical practitioners	Low Income	Southern	Medical Doctors
1	Anaesthesiologist	246	279	316	0.16	60,977	Malawi	Maximum Estimate	2212 - Specialist medical practitioners	Low Income	Southern	Medical Doctors
2	Associate Nurse/Enrolled Nurse/Nursing Assistant	32,379	35,757	39,784	20.66	484	Malawi	Maximum Estimate	3221 - Nursing associate professionals	Low Income	Southern	Nursing Personnel
3	Audiologist	138	155	173	0.09	111,707	Malawi	Maximum Estimate	2266 - Audiologists and speech therapists	Low Income	Southern	Other Health Workers
4	Cardiologist	250	285	323	0.17	59,724	Malawi	Maximum Estimate	2212 - Specialist medical practitioners	Low Income	Southern	Medical Doctors
5	Cardiothoracic Surgeon	51	57	63	0.03	304,525	Malawi	Maximum Estimate	2212 - Specialist medical practitioners	Low Income	Southern	Medical Doctors
6	Clinical Officer/Physician Assistant	3,640	3,735	3,935	2.06	4,848	Malawi	Maximum Estimate	3256 - Medical assistants	Low Income	Southern	Other Health Workers
7	Clinical Pharmacist	908	1,021	1,175	0.61	16,283	Malawi	Maximum Estimate	2262 - Pharmacists	Low Income	Southern	Pharmacist
8	Clinical Psychologist	2,165	2,415	2,723	1.41	7,071	Malawi	Maximum Estimate	2634 - Psychologists	Low Income	Southern	Other Health Workers
9	Community health worker/Village health worker	23,144	24,676	26,523	13.79	725	Malawi	Maximum Estimate	3253 - Community health workers	Low Income	Southern	Other Health Workers
10	Dental Surgery Assistant	2,822	3,190	3,662	1.90	5,275	Malawi	Maximum Estimate	3251 - Dental assistants and therapists	Low Income	Southern	Other Health Workers
11	Dental Therapist	1,898	2,145	2,462	1.27	7,846	Malawi	Maximum Estimate	3251 - Dental assistants and therapists	Low Income	Southern	Other Health Workers

S/N	Health Professionals	2022	2026	2030	Density per 10,000 population	Required Population ratio (1 professional is to xxx population)	Name of Country	Modelling Scenario	ISCO-08 Match	Income Group Classification	Sub-Regional Grouping	SDG 3c Occupation
12	Dentist	2,763	3,127	3,657	1.89	5,284	Malawi	Maximum Estimate	2261 - Dentists	Low Income	Southern	Dentist
13	Dermatologist	93	105	119	0.06	162,708	Malawi	Maximum Estimate	2212 - Specialist medical practitioners	Low Income	Southern	Medical Doctors
14	Endocrinologist	89	103	124	0.06	154,284	Malawi	Maximum Estimate	2212 - Specialist medical practitioners	Low Income	Southern	Medical Doctors
15	ENT Surgeon	349	398	453	0.24	42,487	Malawi	Maximum Estimate	2212 - Specialist medical practitioners	Low Income	Southern	Medical Doctors
16	Environmental Health Officer	1,425	1,577	1,739	0.90	11,104	Malawi	Maximum Estimate	2263 - Environmental and occupational health and hygiene professionals	Low Income	Southern	Other Health Workers
17	Gastroenterologist	320	325	345	0.18	55,412	Malawi	Maximum Estimate	2212 - Specialist medical practitioners	Low Income	Southern	Medical Doctors
18	General Medical Practitioner (Generalist Doctor)	10,161	11,291	12,654	6.58	1,521	Malawi	Maximum Estimate	2211 - Generalist medical practitioners	Low Income	Southern	Medical Doctors
19	General Surgeon	240	279	333	0.17	57,637	Malawi	Maximum Estimate	2212 - Specialist medical practitioners	Low Income	Southern	Medical Doctors
20	Haematologist	114	129	147	0.08	131,230	Malawi	Maximum Estimate	2212 - Specialist medical practitioners	Low Income	Southern	Medical Doctors
21	Health Promoter/Health Educator	332	353	387	0.20	49,068	Malawi	Maximum Estimate	2269 - Health professionals not elsewhere classified	Low Income	Southern	Other Health Workers
22	Infectious Diseases Specialist	32	34	36	0.02	530,181	Malawi	Maximum Estimate	2212 - Specialist medical practitioners	Low Income	Southern	Medical Doctors
23	Intensive Care Nurse	981	1,091	1,211	0.63	15,939	Malawi	Maximum Estimate	2221 - Nursing professionals	Low Income	Southern	Nursing Personnel
24	Medical Laboratory Scientist	3,694	4,083	4,631	2.42	4,124	Malawi	Maximum Estimate	3212 - Medical and pathology laboratory technicians	Low Income	Southern	Other Health Workers
25	Medical Laboratory Technician	3,803	4,006	4,338	2.28	4,387	Malawi	Maximum Estimate	3212 - Medical and pathology laboratory technicians	Low Income	Southern	Other Health Workers
26	Medical Social Worker	933	1,013	1,146	0.61	16,422	Malawi	Maximum Estimate	1344 - Social welfare managers	Low Income	Southern	Other Health Workers
27	Mental Health Nurse	1,972	2,259	2,698	1.41	7,089	Malawi	Maximum Estimate	2221 - Nursing professionals	Low Income	Southern	Nursing Personnel
28	Midwife	19,706	21,610	24,066	12.45	803	Malawi	Maximum Estimate	2222 - Midwifery professionals	Low Income	Southern	Midwifery Personnel
29	Nephrologist	415	478	576	0.30	33,139	Malawi	Maximum Estimate	2212 - Specialist medical practitioners	Low Income	Southern	Medical Doctors
30	Neuro-Surgeon	70	79	89	0.05	214,789	Malawi	Maximum Estimate	2212 - Specialist medical practitioners	Low Income	Southern	Medical Doctors
31	Nurse Anaesthetist	629	718	822	0.43	23,465	Malawi	Maximum Estimate	2221 - Nursing professionals	Low Income	Southern	Nursing Personnel
32	Nutritionist	3,183	3,427	3,683	1.91	5,240	Malawi	Maximum Estimate	2265 - Dieticians and nutritionists	Low Income	Southern	Other Health Workers
33	Obstetrician & Gynaecologist	1,880	2,131	2,459	1.29	7,756	Malawi	Maximum Estimate	2212 - Specialist medical practitioners	Low Income	Southern	Medical Doctors
34	Occupational Therapist	405	459	530	0.28	36,265	Malawi	Maximum Estimate	2269 - Health professionals not elsewhere classified	Low Income	Southern	Other Health Workers
35	Oncology Nurse	316	363	430	0.22	44,507	Malawi	Maximum Estimate	2221 - Nursing professionals	Low Income	Southern	Nursing Personnel
36	Operating Theatre Nurse	2,553	2,909	3,426	1.79	5,584	Malawi	Maximum Estimate	2221 - Nursing professionals	Low Income	Southern	Nursing Personnel
37	Ophthalmic Nurse	323	362	410	0.21	46,983	Malawi	Maximum Estimate	2221 - Nursing professionals	Low Income	Southern	Nursing Personnel
38	Ophthalmologist	107	121	140	0.07	137,764	Malawi	Maximum Estimate	2212 - Specialist medical practitioners	Low Income	Southern	Medical Doctors
39	Optometrist	446	504	572	0.30	33,699	Malawi	Maximum Estimate	2267 - Optometrists and ophthalmic opticians	Low Income	Southern	Other Health Workers
40	Orthopaedic Nurse	408	463	524	0.27	36,805	Malawi	Maximum Estimate	2221 - Nursing professionals	Low Income	Southern	Nursing Personnel
41	Orthopaedic Surgeon	834	966	1,128	0.59	17,045	Malawi	Maximum Estimate	2212 - Specialist medical practitioners	Low Income	Southern	Medical Doctors
42	Orthopaedic Technologist	759	845	938	0.49	20,580	Malawi	Maximum Estimate	3214 - Medical and dental prosthetic technicians	Low Income	Southern	Other Health Workers
43	Paediatric Nurse	3,298	3,664	4,058	2.10	4,765	Malawi	Maximum Estimate	2221 - Nursing professionals	Low Income	Southern	Nursing Personnel
44	Paediatric Surgeon	99	106	113	0.06	170,638	Malawi	Maximum Estimate	2212 - Specialist medical practitioners	Low Income	Southern	Medical Doctors
45	Paediatrician	1,582	1,798	2,066	1.07	9,310	Malawi	Maximum Estimate	2212 - Specialist medical practitioners	Low Income	Southern	Medical Doctors
46	Pathologist	260	289	335	0.18	57,122	Malawi	Maximum Estimate	2212 - Specialist medical practitioners	Low Income	Southern	Medical Doctors
47	Pharmacist	1,710	1,809	1,944	1.02	9,827	Malawi	Maximum Estimate	2262 - Pharmacists	Low Income	Southern	Pharmacist
48	Pharmacy Technician	2,741	3,073	3,475	1.81	5,535	Malawi	Maximum Estimate	3213 - Pharmaceutical technicians and assistants	Low Income	Southern	Other Health Workers
49	Physician	2,844	3,196	3,640	1.89	5,281	Malawi	Maximum Estimate	2212 - Specialist medical practitioners	Low Income	Southern	Medical Doctors
50	Physiotherapist	679	757	848	0.44	22,717	Malawi	Maximum Estimate	2264 - Physiotherapists	Low Income	Southern	Other Health Workers
51	Plastic Surgeon	426	500	586	0.30	32,881	Malawi	Maximum Estimate	2212 - Specialist medical practitioners	Low Income	Southern	Medical Doctors
52	Psychiatrist	1,432	1,620	1,900	0.99	10,086	Malawi	Maximum Estimate	2212 - Specialist medical practitioners	Low Income	Southern	Medical Doctors
53	Radiation Oncologist	80	92	106	0.06	181,166	Malawi	Maximum Estimate	2212 - Specialist medical practitioners	Low Income	Southern	Medical Doctors
54	Radiographer (Diagnostics and Therapy)	2,243	2,580	3,067	1.61	6,212	Malawi	Maximum Estimate	3211 - Medical imaging and therapeutic equipment technicians	Low Income	Southern	Other Health Workers
55	Radiologist	770	915	1,132	0.61	16,519	Malawi	Maximum Estimate	2212 - Specialist medical practitioners	Low Income	Southern	Medical Doctors
56	Registered General Nurse / State Certified Nurse	65,601	73,541	83,798	43.62	229	Malawi	Maximum Estimate	2221 - Nursing professionals	Low Income	Southern	Nursing Personnel
57	Renal Nurse	8,393	9,663	11,635	6.10	1,640	Malawi	Maximum Estimate	2221 - Nursing professionals	Low Income	Southern	Nursing Personnel
58	Respiratory Physician	309	350	396	0.21	48,743	Malawi	Maximum Estimate	2212 - Specialist medical practitioners	Low Income	Southern	Medical Doctors
59	Rheumatologist	71	80	90	0.05	214,183	Malawi	Maximum Estimate	2212 - Specialist medical practitioners	Low Income	Southern	Medical Doctors
60	Speech Therapist	254	281	312	0.16	61,773	Malawi	Maximum Estimate	2266 - Audiologists and speech therapists	Low Income	Southern	Other Health Workers
61	Urologist	35	43	55	0.03	342,782	Malawi	Maximum Estimate	2212 - Specialist medical practitioners	Low Income	Southern	Medical Doctors
1	Anaesthesiologist	268	306	348	0.17	58,621	Mali	Maximum Estimate	2212 - Specialist medical practitioners	Low Income	West	Medical Doctors
2	Associate Nurse/Enrolled Nurse/Nursing Assistant	32,090	36,220	40,951	20.10	498	Mali	Maximum Estimate	3221 - Nursing associate professionals	Low Income	West	Nursing Personnel
3	Audiologist	136	154	173	0.08	117,786	Mali	Maximum Estimate	2266 - Audiologists and speech therapists	Low Income	West	Other Health Workers
4	Cardiologist	229	258	289	0.14	70,757	Mali	Maximum Estimate	2212 - Specialist medical practitioners	Low Income	West	Medical Doctors
5	Cardiothoracic Surgeon	50	56	63	0.03	326,337	Mali	Maximum Estimate	2212 - Specialist medical practitioners	Low Income	West	Medical Doctors
6	Clinical Officer/Physician Assistant	2,691	2,756	2,890	1.44	6,965	Mali	Maximum Estimate	3256 - Medical assistants	Low Income	West	Other Health Workers
7	Clinical Pharmacist	869	986	1,129	0.56	17,998	Mali	Maximum Estimate	2262 - Pharmacists	Low Income	West	Pharmacist
8	Clinical Psychologist	1,682	1,874	2,101	1.03	9,694	Mali	Maximum Estimate	2634 - Psychologists	Low Income	West	Other Health Workers
9	Community health worker/Village health worker	30,367	33,625	37,470	18.44	542	Mali	Maximum Estimate	3253 - Community health workers	Low Income	West	Other Health Workers
10	Dental Surgery Assistant	2,968	3,359	3,820	1.87	5,342	Mali	Maximum Estimate	3251 - Dental assistants and therapists	Low Income	West	Other Health Workers
11	Dental Therapist	1,992	2,254	2,563	1.26	7,961	Mali	Maximum Estimate	3251 - Dental assistants and therapists	Low Income	West	Other Health Workers
12	Dentist	2,411	2,732	3,159	1.55	6,450	Mali	Maximum Estimate	2261 - Dentists	Low Income	West	Dentist
13	Dermatologist	96	109	123	0.06	165,811	Mali	Maximum Estimate	2212 - Specialist medical practitioners	Low Income	West	Medical Doctors
14	Endocrinologist	72	85	104	0.05	193,493	Mali	Maximum Estimate	2212 - Specialist medical practitioners	Low Income	West	Medical Doctors
15	ENT Surgeon	339	385	434	0.21	46,977	Mali	Maximum Estimate	2212 - Specialist medical practitioners	Low Income	West	Medical Doctors
16	Environmental Health Officer	1,509	1,701	1,912	0.94	10,681	Mali	Maximum Estimate	2263 - Environmental and occupational health and hygiene professionals	Low Income	West	Other Health Workers
17	Gastroenterologist	294	317	352	0.17	57,446	Mali	Maximum Estimate	2212 - Specialist medical practitioners	Low Income	West	Medical Doctors
18	General Medical Practitioner (Generalist Doctor)	10,980	12,327	13,884	6.82	1,467	Mali	Maximum Estimate	2211 - Generalist medical practitioners	Low Income	West	Medical Doctors
19	General Surgeon	271	314	372	0.18	54,596	Mali	Maximum Estimate	2212 - Specialist medical practitioners	Low Income	West	Medical Doctors
20	Haematologist	165	196	232	0.11	87,858	Mali	Maximum Estimate	2212 - Specialist medical practitioners	Low Income	West	Medical Doctors
21	Health Promoter/Health Educator	315	327	346	0.17	58,422	Mali	Maximum Estimate	2269 - Health professionals not elsewhere classified	Low Income	West	Other Health Workers
22	Infectious Diseases Specialist	28	32	36	0.02	566,141	Mali	Maximum Estimate	2212 - Specialist medical practitioners	Low Income	West	Medical Doctors
23	Intensive Care Nurse	1,093	1,235	1,393	0.68	14,659	Mali	Maximum Estimate	2221 - Nursing professionals	Low Income	West	Nursing Personnel
24	Medical Laboratory Scientist	3,020	3,384	3,849	1.90	5,267	Mali	Maximum Estimate	3212 - Medical and pathology laboratory technicians	Low Income	West	Other Health Workers
25	Medical Laboratory Technician	5,378	6,030	6,839	3.37	2,965	Mali	Maximum Estimate	3212 - Medical and pathology laboratory technicians	Low Income	West	Other Health Workers
26	Medical Social Worker	1,358	1,494	1,652	0.81	12,325	Mali	Maximum Estimate	1344 - Social welfare managers	Low Income	West	Other Health Workers
27	Mental Health Nurse	1,553	1,781	2,094	1.04	9,655	Mali	Maximum Estimate	2221 - Nursing professionals	Low Income	West	Nursing Personnel
28	Midwife	30,232	34,690	39,940	19.59	511	Mali	Maximum Estimate	2222 - Midwifery professionals	Low Income	West	Midwifery Personnel
29	Nephrologist	215	245	289	0.14	69,957	Mali	Maximum Estimate	2212 - Specialist medical practitioners	Low Income	West	Medical Doctors
30	Neuro-Surgeon	64	74	85	0.04	241,085	Mali	Maximum Estimate	2212 - Specialist medical practitioners	Low Income	West	Medical Doctors
31	Nurse Anaesthetist	507	580	663	0.33	30,764	Mali	Maximum Estimate	2221 - Nursing professionals	Low Income	West	Nursing Personnel
32	Nutritionist	6,679	7,415	8,206	4.02	2,488	Mali	Maximum Estimate	2265 - Dieticians and nutritionists	Low Income	West	Other Health Workers
33	Obstetrician & Gynaecologist	1,744	1,975	2,244	1.10	9,079	Mali	Maximum Estimate	2212 - Specialist medical practitioners	Low Income	West	Medical Doctors
34	Occupational Therapist	477	549	637	0.31	31,922	Mali	Maximum Estimate	2269 - Health professionals not elsewhere classified	Low Income	West	Other Health Workers

S/N	Health Professionals	2022	2026	2030	Density per 10,000 population	Required Population ratio (1 professional is to xxx population)	Name of Country	Modelling Scenario	ISCO-08 Match	Income Group Classification	Sub-Regional Grouping	SDG 3c Occupation
35	Oncology Nurse	172	196	228	0.11	88,792	Mali	Maximum Estimate	2221 - Nursing professionals	Low Income	West	Nursing Personnel
36	Operating Theatre Nurse	2,360	2,712	3,174	1.57	6,375	Mali	Maximum Estimate	2221 - Nursing professionals	Low Income	West	Nursing Personnel
37	Ophthalmic Nurse	277	314	358	0.18	56,881	Mali	Maximum Estimate	2221 - Nursing professionals	Low Income	West	Nursing Personnel
38	Ophthalmologist	100	113	129	0.06	157,795	Mali	Maximum Estimate	2212 - Specialist medical practitioners	Low Income	West	Medical Doctors
39	Optometrist	410	456	508	0.25	40,101	Mali	Maximum Estimate	2267 - Optometrists and ophthalmic opticians	Low Income	West	Other Health Workers
40	Orthopaedic Nurse	585	676	778	0.38	26,230	Mali	Maximum Estimate	2221 - Nursing professionals	Low Income	West	Nursing Personnel
41	Orthopaedic Surgeon	748	868	1,013	0.50	20,084	Mali	Maximum Estimate	2212 - Specialist medical practitioners	Low Income	West	Medical Doctors
42	Orthopaedic Technologist	839	951	1,073	0.53	19,023	Mali	Maximum Estimate	3214 - Medical and dental prosthetic technicians	Low Income	West	Other Health Workers
43	Paediatric Nurse	4,673	5,363	6,108	2.99	3,341	Mali	Maximum Estimate	2221 - Nursing professionals	Low Income	West	Nursing Personnel
44	Paediatric Surgeon	136	155	173	0.08	117,994	Mali	Maximum Estimate	2212 - Specialist medical practitioners	Low Income	West	Medical Doctors
45	Paediatrician	1,259	1,468	1,714	0.84	11,866	Mali	Maximum Estimate	2212 - Specialist medical practitioners	Low Income	West	Medical Doctors
46	Pathologist	207	232	266	0.13	76,172	Mali	Maximum Estimate	2212 - Specialist medical practitioners	Low Income	West	Medical Doctors
47	Pharmacist	1,803	1,897	2,008	0.99	10,114	Mali	Maximum Estimate	2262 - Pharmacists	Low Income	West	Pharmacist
48	Pharmacy Technician	3,145	3,538	3,990	1.96	5,108	Mali	Maximum Estimate	3213 - Pharmaceutical technicians and assistants	Low Income	West	Other Health Workers
49	Physician	3,493	3,978	4,546	2.23	4,479	Mali	Maximum Estimate	2212 - Specialist medical practitioners	Low Income	West	Medical Doctors
50	Physiotherapist	719	814	922	0.45	22,112	Mali	Maximum Estimate	2264 - Physiotherapists	Low Income	West	Other Health Workers
51	Plastic Surgeon	189	216	247	0.12	82,473	Mali	Maximum Estimate	2212 - Specialist medical practitioners	Low Income	West	Medical Doctors
52	Psychiatrist	1,530	1,732	1,995	0.98	10,170	Mali	Maximum Estimate	2212 - Specialist medical practitioners	Low Income	West	Medical Doctors
53	Radiation Oncologist	55	63	73	0.04	279,925	Mali	Maximum Estimate	2212 - Specialist medical practitioners	Low Income	West	Medical Doctors
54	Radiographer (Diagnostics and Therapy)	2,064	2,357	2,727	1.34	7,445	Mali	Maximum Estimate	3211 - Medical imaging and therapeutic equipment technicians	Low Income	West	Other Health Workers
55	Radiologist	800	920	1,068	0.53	19,029	Mali	Maximum Estimate	2212 - Specialist medical practitioners	Low Income	West	Medical Doctors
56	Registered General Nurse / State Certified Nurse	82,705	90,790	100,298	49.36	203	Mali	Maximum Estimate	2221 - Nursing professionals	Low Income	West	Nursing Personnel
57	Renal Nurse	4,114	4,696	5,519	2.73	3,657	Mali	Maximum Estimate	2221 - Nursing professionals	Low Income	West	Nursing Personnel
58	Respiratory Physician	208	244	287	0.14	71,027	Mali	Maximum Estimate	2212 - Specialist medical practitioners	Low Income	West	Medical Doctors
59	Rheumatologist	71	81	91	0.04	224,163	Mali	Maximum Estimate	2212 - Specialist medical practitioners	Low Income	West	Medical Doctors
60	Speech Therapist	256	290	326	0.16	62,600	Mali	Maximum Estimate	2266 - Audiologists and speech therapists	Low Income	West	Other Health Workers
61	Urologist	43	50	60	0.03	335,275	Mali	Maximum Estimate	2212 - Specialist medical practitioners	Low Income	West	Medical Doctors
1	Anaesthesiologist	96	108	121	0.26	38,598	Mauritania	Maximum Estimate	2212 - Specialist medical practitioners	Lower-middle Income	West	Medical Doctors
2	Associate Nurse/Enrolled Nurse/Nursing Assistant	8,549	10,070	11,267	23.84	420	Mauritania	Maximum Estimate	3221 - Nursing associate professionals	Lower-middle Income	West	Nursing Personnel
3	Audiologist	31	35	40	0.08	118,380	Mauritania	Maximum Estimate	2266 - Audiologists and speech therapists	Lower-middle Income	West	Other Health Workers
4	Cardiologist	78	90	104	0.22	45,254	Mauritania	Maximum Estimate	2212 - Specialist medical practitioners	Lower-middle Income	West	Medical Doctors
5	Cardiothoracic Surgeon	18	20	22	0.05	214,325	Mauritania	Maximum Estimate	2212 - Specialist medical practitioners	Lower-middle Income	West	Medical Doctors
6	Clinical Officer/Physician Assistant	1,363	2,262	2,551	5.15	1,943	Mauritania	Maximum Estimate	3256 - Medical assistants	Lower-middle Income	West	Other Health Workers
7	Clinical Pharmacist	234	260	292	0.62	16,013	Mauritania	Maximum Estimate	2262 - Pharmacists	Lower-middle Income	West	Pharmacist
8	Clinical Psychologist	416	462	515	1.10	9,082	Mauritania	Maximum Estimate	2634 - Psychologists	Lower-middle Income	West	Other Health Workers
9	Community health worker/Village health worker	9,600	15,379	17,206	34.77	288	Mauritania	Maximum Estimate	3253 - Community health workers	Lower-middle Income	West	Other Health Workers
10	Dental Surgery Assistant	810	917	1,037	2.21	4,518	Mauritania	Maximum Estimate	3251 - Dental assistants and therapists	Lower-middle Income	West	Other Health Workers
11	Dental Therapist	542	614	694	1.48	6,755	Mauritania	Maximum Estimate	3251 - Dental assistants and therapists	Lower-middle Income	West	Other Health Workers
12	Dentist	655	740	837	1.79	5,592	Mauritania	Maximum Estimate	2261 - Dentists	Lower-middle Income	West	Dentist
13	Dermatologist	25	28	32	0.07	147,824	Mauritania	Maximum Estimate	2212 - Specialist medical practitioners	Lower-middle Income	West	Medical Doctors
14	Endocrinologist	22	27	33	0.07	139,997	Mauritania	Maximum Estimate	2212 - Specialist medical practitioners	Lower-middle Income	West	Medical Doctors
15	ENT Surgeon	93	106	122	0.26	38,481	Mauritania	Maximum Estimate	2212 - Specialist medical practitioners	Lower-middle Income	West	Medical Doctors
16	Environmental Health Officer	346	385	426	0.91	11,022	Mauritania	Maximum Estimate	2263 - Environmental and occupational health and hygiene professionals	Lower-middle Income	West	Other Health Workers
17	Gastroenterologist	114	117	122	0.26	38,257	Mauritania	Maximum Estimate	2212 - Specialist medical practitioners	Lower-middle Income	West	Medical Doctors
18	General Medical Practitioner (Generalist Doctor)	2,989	3,737	4,183	8.77	1,141	Mauritania	Maximum Estimate	2211 - Generalist medical practitioners	Lower-middle Income	West	Medical Doctors
19	General Surgeon	71	82	96	0.21	48,697	Mauritania	Maximum Estimate	2212 - Specialist medical practitioners	Lower-middle Income	West	Medical Doctors
20	Haematologist	23	25	26	0.06	178,716	Mauritania	Maximum Estimate	2212 - Specialist medical practitioners	Lower-middle Income	West	Medical Doctors
21	Health Promoter/Health Educator	130	198	220	0.45	22,332	Mauritania	Maximum Estimate	2269 - Health professionals not elsewhere classified	Lower-middle Income	West	Other Health Workers
22	Infectious Diseases Specialist	8	8	9	0.02	511,238	Mauritania	Maximum Estimate	2212 - Specialist medical practitioners	Lower-middle Income	West	Medical Doctors
23	Intensive Care Nurse	242	266	291	0.62	16,088	Mauritania	Maximum Estimate	2221 - Nursing professionals	Lower-middle Income	West	Nursing Personnel
24	Medical Laboratory Scientist	969	1,336	1,513	3.13	3,191	Mauritania	Maximum Estimate	3212 - Medical and pathology laboratory technicians	Lower-middle Income	West	Other Health Workers
25	Medical Laboratory Technician	1,863	3,340	3,813	7.64	1,309	Mauritania	Maximum Estimate	3212 - Medical and pathology laboratory technicians	Lower-middle Income	West	Other Health Workers
26	Medical Social Worker	303	315	328	0.70	14,235	Mauritania	Maximum Estimate	1344 - Social welfare managers	Lower-middle Income	West	Other Health Workers
27	Mental Health Nurse	432	491	565	1.21	8,263	Mauritania	Maximum Estimate	2221 - Nursing professionals	Lower-middle Income	West	Nursing Personnel
28	Midwife	5,006	5,517	6,061	12.93	773	Mauritania	Maximum Estimate	2222 - Midwifery professionals	Lower-middle Income	West	Midwifery Personnel
29	Nephrologist	84	98	115	0.25	40,407	Mauritania	Maximum Estimate	2212 - Specialist medical practitioners	Lower-middle Income	West	Medical Doctors
30	Neuro-Surgeon	27	31	35	0.07	133,446	Mauritania	Maximum Estimate	2212 - Specialist medical practitioners	Lower-middle Income	West	Medical Doctors
31	Nurse Anaesthetist	194	226	263	0.56	17,808	Mauritania	Maximum Estimate	2221 - Nursing professionals	Lower-middle Income	West	Nursing Personnel
32	Nutritionist	1,001	1,042	1,081	2.31	4,329	Mauritania	Maximum Estimate	2265 - Dieticians and nutritionists	Lower-middle Income	West	Other Health Workers
33	Obstetrician & Gynaecologist	428	480	538	1.15	8,715	Mauritania	Maximum Estimate	2212 - Specialist medical practitioners	Lower-middle Income	West	Medical Doctors
34	Occupational Therapist	202	228	257	0.55	18,181	Mauritania	Maximum Estimate	2269 - Health professionals not elsewhere classified	Lower-middle Income	West	Other Health Workers
35	Oncology Nurse	66	77	92	0.20	50,951	Mauritania	Maximum Estimate	2221 - Nursing professionals	Lower-middle Income	West	Nursing Personnel
36	Operating Theatre Nurse	828	939	1,079	2.31	4,326	Mauritania	Maximum Estimate	2221 - Nursing professionals	Lower-middle Income	West	Nursing Personnel
37	Ophthalmic Nurse	82	93	107	0.23	43,755	Mauritania	Maximum Estimate	2221 - Nursing professionals	Lower-middle Income	West	Nursing Personnel
38	Ophthalmologist	29	33	39	0.08	121,147	Mauritania	Maximum Estimate	2212 - Specialist medical practitioners	Lower-middle Income	West	Medical Doctors
39	Optometrist	119	137	157	0.34	29,722	Mauritania	Maximum Estimate	2267 - Optometrists and ophthalmic opticians	Lower-middle Income	West	Other Health Workers
40	Orthopaedic Nurse	276	310	347	0.74	13,523	Mauritania	Maximum Estimate	2221 - Nursing professionals	Lower-middle Income	West	Nursing Personnel
41	Orthopaedic Surgeon	293	338	391	0.84	11,975	Mauritania	Maximum Estimate	2212 - Specialist medical practitioners	Lower-middle Income	West	Medical Doctors
42	Orthopaedic Technologist	252	281	312	0.67	15,035	Mauritania	Maximum Estimate	3214 - Medical and dental prosthetic technicians	Lower-middle Income	West	Other Health Workers
43	Paediatric Nurse	819	885	945	2.02	4,961	Mauritania	Maximum Estimate	2221 - Nursing professionals	Lower-middle Income	West	Nursing Personnel
44	Paediatric Surgeon	23	24	26	0.06	179,169	Mauritania	Maximum Estimate	2212 - Specialist medical practitioners	Lower-middle Income	West	Medical Doctors
45	Paediatrician	385	433	487	1.04	9,608	Mauritania	Maximum Estimate	2212 - Specialist medical practitioners	Lower-middle Income	West	Medical Doctors
46	Pathologist	55	62	72	0.15	65,030	Mauritania	Maximum Estimate	2212 - Specialist medical practitioners	Lower-middle Income	West	Medical Doctors
47	Pharmacist	428	442	462	1.00	10,047	Mauritania	Maximum Estimate	2262 - Pharmacists	Lower-middle Income	West	Pharmacist
48	Pharmacy Technician	868	964	1,073	2.29	4,363	Mauritania	Maximum Estimate	3213 - Pharmaceutical technicians and assistants	Lower-middle Income	West	Other Health Workers
49	Physician	893	1,072	1,214	2.57	3,896	Mauritania	Maximum Estimate	2212 - Specialist medical practitioners	Lower-middle Income	West	Medical Doctors
50	Physiotherapist	211	236	265	0.57	17,690	Mauritania	Maximum Estimate	2264 - Physiotherapists	Lower-middle Income	West	Other Health Workers
51	Plastic Surgeon	55	63	73	0.15	64,615	Mauritania	Maximum Estimate	2212 - Specialist medical practitioners	Lower-middle Income	West	Medical Doctors
52	Psychiatrist	381	427	484	1.04	9,651	Mauritania	Maximum Estimate	2212 - Specialist medical practitioners	Lower-middle Income	West	Medical Doctors
53	Radiation Oncologist	22	26	32	0.07	147,645	Mauritania	Maximum Estimate	2212 - Specialist medical practitioners	Lower-middle Income	West	Medical Doctors
54	Radiographer (Diagnostics and Therapy)	647	735	841	1.80	5,555	Mauritania	Maximum Estimate	3211 - Medical imaging and therapeutic equipment technicians	Lower-middle Income	West	Other Health Workers
55	Radiologist	245	276	314	0.67	14,920	Mauritania	Maximum Estimate	2212 - Specialist medical practitioners	Lower-middle Income	West	Medical Doctors
56	Registered General Nurse / State Certified Nurse	20,126	21,876	23,981	51.41	195	Mauritania	Maximum Estimate	2221 - Nursing professionals	Lower-middle Income	West	Nursing Personnel
57	Renal Nurse	1,636	1,900	2,242	4.81	2,080	Mauritania	Maximum Estimate	2221 - Nursing professionals	Lower-middle Income	West	Nursing Personnel

S/N	Health Professionals	2022	2026	2030	Density per 10,000 population	Required Population ratio (1 professional is to xxx population)	Name of Country	Modelling Scenario	ISCO-08 Match	Income Group Classification	Sub-Regional Grouping	SDG 3c Occupation
58	Respiratory Physician	87	99	112	0.24	41,995	Mauritania	Maximum Estimate	2212 - Specialist medical practitioners	Lower-middle Income	West	Medical Doctors
59	Rheumatologist	20	22	25	0.05	186,381	Mauritania	Maximum Estimate	2212 - Specialist medical practitioners	Lower-middle Income	West	Medical Doctors
60	Speech Therapist	62	70	78	0.17	59,775	Mauritania	Maximum Estimate	2266 - Audiologists and speech therapists	Lower-middle Income	West	Other Health Workers
61	Urologist	15	17	20	0.04	233,194	Mauritania	Maximum Estimate	2212 - Specialist medical practitioners	Lower-middle Income	West	Medical Doctors
1	Anaesthesiologist	62	69	78	0.61	16,397	Mauritius	Maximum Estimate	2212 - Specialist medical practitioners	High Income and Upper Middle Income	Southern	Medical Doctors
2	Associate Nurse/Enrolled Nurse/Nursing Assistant	2,956	3,091	3,247	25.34	395	Mauritius	Maximum Estimate	3221 - Nursing associate professionals	High Income and Upper Middle Income	Southern	Nursing Personnel
3	Audiologist	9	10	10	0.08	124,241	Mauritius	Maximum Estimate	2266 - Audiologists and speech therapists	High Income and Upper Middle Income	Southern	Other Health Workers
4	Cardiologist	84	95	109	0.86	11,693	Mauritius	Maximum Estimate	2212 - Specialist medical practitioners	High Income and Upper Middle Income	Southern	Medical Doctors
5	Cardiothoracic Surgeon	7	8	8	0.06	154,049	Mauritius	Maximum Estimate	2212 - Specialist medical practitioners	High Income and Upper Middle Income	Southern	Medical Doctors
6	Clinical Officer/Physician Assistant	718	747	777	6.06	1,650	Mauritius	Maximum Estimate	3256 - Medical assistants	High Income and Upper Middle Income	Southern	Other Health Workers
7	Clinical Pharmacist	122	131	140	1.09	9,174	Mauritius	Maximum Estimate	2262 - Pharmacists	High Income and Upper Middle Income	Southern	Pharmacist
8	Clinical Psychologist	116	117	118	0.92	10,816	Mauritius	Maximum Estimate	2634 - Psychologists	High Income and Upper Middle Income	Southern	Other Health Workers
9	Community health worker/Village health worker	1,214	1,212	1,205	9.46	1,057	Mauritius	Maximum Estimate	3253 - Community health workers	High Income and Upper Middle Income	Southern	Other Health Workers
10	Dental Surgery Assistant	173	181	188	1.46	6,848	Mauritius	Maximum Estimate	3251 - Dental assistants and therapists	High Income and Upper Middle Income	Southern	Other Health Workers
11	Dental Therapist	116	121	126	0.98	10,238	Mauritius	Maximum Estimate	3251 - Dental assistants and therapists	High Income and Upper Middle Income	Southern	Other Health Workers
12	Dentist	160	160	157	1.21	8,240	Mauritius	Maximum Estimate	2261 - Dentists	High Income and Upper Middle Income	Southern	Dentist
13	Dermatologist	8	8	8	0.06	158,130	Mauritius	Maximum Estimate	2212 - Specialist medical practitioners	High Income and Upper Middle Income	Southern	Medical Doctors
14	Endocrinologist	65	72	80	0.61	16,278	Mauritius	Maximum Estimate	2212 - Specialist medical practitioners	High Income and Upper Middle Income	Southern	Medical Doctors
15	ENT Surgeon	63	67	80	0.63	15,847	Mauritius	Maximum Estimate	2212 - Specialist medical practitioners	High Income and Upper Middle Income	Southern	Medical Doctors
16	Environmental Health Officer	95	95	95	0.75	13,388	Mauritius	Maximum Estimate	2263 - Environmental and occupational health and hygiene professionals	High Income and Upper Middle Income	Southern	Other Health Workers
17	Gastroenterologist	15	15	14	0.11	90,193	Mauritius	Maximum Estimate	2212 - Specialist medical practitioners	High Income and Upper Middle Income	Southern	Medical Doctors
18	General Medical Practitioner (Generalist Doctor)	1,236	1,286	1,339	10.46	956	Mauritius	Maximum Estimate	2211 - Generalist medical practitioners	High Income and Upper Middle Income	Southern	Medical Doctors
19	General Surgeon	58	65	72	0.56	17,811	Mauritius	Maximum Estimate	2212 - Specialist medical practitioners	High Income and Upper Middle Income	Southern	Medical Doctors
20	Haematologist	7	7	7	0.06	173,806	Mauritius	Maximum Estimate	2212 - Specialist medical practitioners	High Income and Upper Middle Income	Southern	Medical Doctors
21	Health Promoter/Health Educator	72	74	76	0.59	16,881	Mauritius	Maximum Estimate	2269 - Health professionals not elsewhere classified	High Income and Upper Middle Income	Southern	Other Health Workers
22	Infectious Diseases Specialist	2	2	2	0.02	560,527	Mauritius	Maximum Estimate	2212 - Specialist medical practitioners	High Income and Upper Middle Income	Southern	Medical Doctors
23	Intensive Care Nurse	107	112	121	0.95	10,492	Mauritius	Maximum Estimate	2221 - Nursing professionals	High Income and Upper Middle Income	Southern	Nursing Personnel
24	Medical Laboratory Scientist	577	631	692	5.37	1,861	Mauritius	Maximum Estimate	3212 - Medical and pathology laboratory technicians	High Income and Upper Middle Income	Southern	Other Health Workers
25	Medical Laboratory Technician	446	485	530	4.12	2,427	Mauritius	Maximum Estimate	3212 - Medical and pathology laboratory technicians	High Income and Upper Middle Income	Southern	Other Health Workers
26	Medical Social Worker	60	59	57	0.45	22,415	Mauritius	Maximum Estimate	1344 - Social welfare managers	High Income and Upper Middle Income	Southern	Other Health Workers
27	Mental Health Nurse	207	207	207	1.61	6,206	Mauritius	Maximum Estimate	2221 - Nursing professionals	High Income and Upper Middle Income	Southern	Nursing Personnel
28	Midwife	1,266	1,260	1,240	9.67	1,034	Mauritius	Maximum Estimate	2222 - Midwifery professionals	High Income and Upper Middle Income	Southern	Midwifery Personnel
29	Nephrologist	120	133	147	1.14	8,795	Mauritius	Maximum Estimate	2212 - Specialist medical practitioners	High Income and Upper Middle Income	Southern	Medical Doctors
30	Neuro-Surgeon	27	30	37	0.29	34,320	Mauritius	Maximum Estimate	2212 - Specialist medical practitioners	High Income and Upper Middle Income	Southern	Medical Doctors
31	Nurse Anaesthetist	238	276	319	2.48	4,025	Mauritius	Maximum Estimate	2221 - Nursing professionals	High Income and Upper Middle Income	Southern	Nursing Personnel
32	Nutritionist	264	258	250	1.97	5,072	Mauritius	Maximum Estimate	2265 - Dieticians and nutritionists	High Income and Upper Middle Income	Southern	Other Health Workers
33	Obstetrician & Gynaecologist	129	131	132	1.03	9,675	Mauritius	Maximum Estimate	2212 - Specialist medical practitioners	High Income and Upper Middle Income	Southern	Medical Doctors
34	Occupational Therapist	87	93	101	0.78	12,768	Mauritius	Maximum Estimate	2269 - Health professionals not elsewhere classified	High Income and Upper Middle Income	Southern	Other Health Workers
35	Oncology Nurse	106	120	136	1.06	9,478	Mauritius	Maximum Estimate	2221 - Nursing professionals	High Income and Upper Middle Income	Southern	Nursing Personnel
36	Operating Theatre Nurse	605	655	729	5.69	1,756	Mauritius	Maximum Estimate	2221 - Nursing professionals	High Income and Upper Middle Income	Southern	Nursing Personnel
37	Ophthalmic Nurse	93	100	109	0.84	11,843	Mauritius	Maximum Estimate	2221 - Nursing professionals	High Income and Upper Middle Income	Southern	Nursing Personnel
38	Ophthalmologist	37	41	47	0.37	26,913	Mauritius	Maximum Estimate	2212 - Specialist medical practitioners	High Income and Upper Middle Income	Southern	Medical Doctors
39	Optometrist	107	119	132	1.02	9,772	Mauritius	Maximum Estimate	2267 - Optometrists and ophthalmic opticians	High Income and Upper Middle Income	Southern	Other Health Workers
40	Orthopaedic Nurse	53	55	58	0.45	21,990	Mauritius	Maximum Estimate	2221 - Nursing professionals	High Income and Upper Middle Income	Southern	Nursing Personnel
41	Orthopaedic Surgeon	214	231	249	1.94	5,142	Mauritius	Maximum Estimate	2212 - Specialist medical practitioners	High Income and Upper Middle Income	Southern	Medical Doctors
42	Orthopaedic Technologist	60	61	63	0.49	20,246	Mauritius	Maximum Estimate	3214 - Medical and dental prosthetic technicians	High Income and Upper Middle Income	Southern	Other Health Workers
43	Paediatric Nurse	151	148	144	1.13	8,863	Mauritius	Maximum Estimate	2221 - Nursing professionals	High Income and Upper Middle Income	Southern	Nursing Personnel
44	Paediatric Surgeon	2	2	2	0.02	603,003	Mauritius	Maximum Estimate	2212 - Specialist medical practitioners	High Income and Upper Middle Income	Southern	Medical Doctors
45	Paediatrician	142	151	160	1.24	8,050	Mauritius	Maximum Estimate	2212 - Specialist medical practitioners	High Income and Upper Middle Income	Southern	Medical Doctors
46	Pathologist	21	22	23	0.17	57,160	Mauritius	Maximum Estimate	2212 - Specialist medical practitioners	High Income and Upper Middle Income	Southern	Medical Doctors
47	Pharmacist	570	591	613	4.79	2,089	Mauritius	Maximum Estimate	2262 - Pharmacists	High Income and Upper Middle Income	Southern	Pharmacist
48	Pharmacy Technician	451	466	483	3.77	2,651	Mauritius	Maximum Estimate	3213 - Pharmaceutical technicians and assistants	High Income and Upper Middle Income	Southern	Other Health Workers
49	Physician	469	500	533	4.14	2,414	Mauritius	Maximum Estimate	2212 - Specialist medical practitioners	High Income and Upper Middle Income	Southern	Medical Doctors
50	Physiotherapist	92	97	107	0.84	11,838	Mauritius	Maximum Estimate	2264 - Physiotherapists	High Income and Upper Middle Income	Southern	Other Health Workers
51	Plastic Surgeon	35	35	35	0.28	35,932	Mauritius	Maximum Estimate	2212 - Specialist medical practitioners	High Income and Upper Middle Income	Southern	Medical Doctors
52	Psychiatrist	147	145	143	1.12	8,947	Mauritius	Maximum Estimate	2212 - Specialist medical practitioners	High Income and Upper Middle Income	Southern	Medical Doctors
53	Radiation Oncologist	46	54	64	0.50	19,950	Mauritius	Maximum Estimate	2212 - Specialist medical practitioners	High Income and Upper Middle Income	Southern	Medical Doctors
54	Radiographer (Diagnostics and Therapy)	426	461	500	3.87	2,581	Mauritius	Maximum Estimate	3211 - Medical imaging and therapeutic equipment technicians	High Income and Upper Middle Income	Southern	Other Health Workers
55	Radiologist	130	139	149	1.16	8,593	Mauritius	Maximum Estimate	2212 - Specialist medical practitioners	High Income and Upper Middle Income	Southern	Medical Doctors
56	Registered General Nurse / State Certified Nurse	9,851	10,717	11,700	90.89	110	Mauritius	Maximum Estimate	2221 - Nursing professionals	High Income and Upper Middle Income	Southern	Nursing Personnel
57	Renal Nurse	2,462	2,727	3,016	23.31	429	Mauritius	Maximum Estimate	2221 - Nursing professionals	High Income and Upper Middle Income	Southern	Nursing Personnel
58	Respiratory Physician	16	16	17	0.13	76,731	Mauritius	Maximum Estimate	2212 - Specialist medical practitioners	High Income and Upper Middle Income	Southern	Medical Doctors
59	Rheumatologist	9	10	10	0.08	122,413	Mauritius	Maximum Estimate	2212 - Specialist medical practitioners	High Income and Upper Middle Income	Southern	Medical Doctors
60	Speech Therapist	35	37	44	0.35	28,737	Mauritius	Maximum Estimate	2266 - Audiologists and speech therapists	High Income and Upper Middle Income	Southern	Other Health Workers
61	Urologist	35	41	48	0.38	26,627	Mauritius	Maximum Estimate	2212 - Specialist medical practitioners	High Income and Upper Middle Income	Southern	Medical Doctors
1	Anaesthesiologist	491	555	626	0.20	50,411	Mozambique	Maximum Estimate	2212 - Specialist medical practitioners	Low Income	Southern	Medical Doctors
2	Associate Nurse/Enrolled Nurse/Nursing Assistant	56,413	63,024	70,563	22.42	446	Mozambique	Maximum Estimate	3221 - Nursing associate professionals	Low Income	Southern	Nursing Personnel
3	Audiologist	212	237	264	0.08	119,682	Mozambique	Maximum Estimate	2266 - Audiologists and speech therapists	Low Income	Southern	Other Health Workers
4	Cardiologist	360	401	445	0.14	70,924	Mozambique	Maximum Estimate	2212 - Specialist medical practitioners	Low Income	Southern	Medical Doctors
5	Cardiothoracic Surgeon	115	130	147	0.05	215,369	Mozambique	Maximum Estimate	2212 - Specialist medical practitioners	Low Income	Southern	Medical Doctors
6	Clinical Officer/Physician Assistant	9,902	10,612	11,566	3.70	2,704	Mozambique	Maximum Estimate	3256 - Medical assistants	Low Income	Southern	Other Health Workers
7	Clinical Pharmacist	1,337	1,493	1,689	0.54	18,590	Mozambique	Maximum Estimate	2262 - Pharmacists	Low Income	Southern	Pharmacist
8	Clinical Psychologist	2,788	3,098	3,470	1.10	9,071	Mozambique	Maximum Estimate	2634 - Psychologists	Low Income	Southern	Other Health Workers
9	Community health worker/Village health worker	56,222	61,468	67,136	21.31	469	Mozambique	Maximum Estimate	3253 - Community health workers	Low Income	Southern	Other Health Workers
10	Dental Surgery Assistant	4,303	4,788	5,358	1.70	5,889	Mozambique	Maximum Estimate	3251 - Dental assistants and therapists	Low Income	Southern	Other Health Workers
11	Dental Therapist	2,899	3,226	3,610	1.14	8,741	Mozambique	Maximum Estimate	3251 - Dental assistants and therapists	Low Income	Southern	Other Health Workers
12	Dentist	4,252	4,736	5,370	1.70	5,869	Mozambique	Maximum Estimate	2261 - Dentists	Low Income	Southern	Dentist
13	Dermatologist	140	157	175	0.06	180,091	Mozambique	Maximum Estimate	2212 - Specialist medical practitioners	Low Income	Southern	Medical Doctors
14	Endocrinologist	127	148	180	0.06	173,801	Mozambique	Maximum Estimate	2212 - Specialist medical practitioners	Low Income	Southern	Medical Doctors
15	ENT Surgeon	520	589	663	0.21	47,535	Mozambique	Maximum Estimate	2212 - Specialist medical practitioners	Low Income	Southern	Medical Doctors
16	Environmental Health Officer	2,329	2,598	2,885	0.91	10,942	Mozambique	Maximum Estimate	2263 - Environmental and occupational health and hygiene professionals	Low Income	Southern	Other Health Workers
17	Gastroenterologist	467	471	492	0.16	63,495	Mozambique	Maximum Estimate	2212 - Specialist medical practitioners	Low Income	Southern	Medical Doctors
18	General Medical Practitioner (Generalist Doctor)	18,217	20,404	22,956	7.30	1,370	Mozambique	Maximum Estimate	2211 - Generalist medical practitioners	Low Income	Southern	Medical Doctors
19	General Surgeon	358	418	495	0.16	63,330	Mozambique	Maximum Estimate	2212 - Specialist medical practitioners	Low Income	Southern	Medical Doctors

S/N	Health Professionals	2022	2026	2030	Density per 10,000 population	Required Population ratio (1 professional is to xxx population)	Name of Country	Modelling Scenario	ISCO-08 Match	Income Group Classification	Sub-Regional Grouping	SDG 3c Occupation
20	Haematologist	156	171	187	0.06	168,853	Mozambique	Maximum Estimate	2212 - Specialist medical practitioners	Low Income	Southern	Medical Doctors
21	Health Promoter/Health Educator	889	960	1,052	0.34	29,720	Mozambique	Maximum Estimate	2269 - Health professionals not elsewhere classified	Low Income	Southern	Other Health Workers
22	Infectious Diseases Specialist	51	56	62	0.02	508,598	Mozambique	Maximum Estimate	2212 - Specialist medical practitioners	Low Income	Southern	Medical Doctors
23	Intensive Care Nurse	1,354	1,531	1,726	0.55	18,264	Mozambique	Maximum Estimate	2221 - Nursing professionals	Low Income	Southern	Nursing Personnel
24	Medical Laboratory Scientist	7,145	8,183	9,475	3.02	3,308	Mozambique	Maximum Estimate	3212 - Medical and pathology laboratory technicians	Low Income	Southern	Other Health Workers
25	Medical Laboratory Technician	12,150	13,415	14,859	4.73	2,116	Mozambique	Maximum Estimate	3212 - Medical and pathology laboratory technicians	Low Income	Southern	Other Health Workers
26	Medical Social Worker	1,429	1,535	1,689	0.54	18,436	Mozambique	Maximum Estimate	1344 - Social welfare managers	Low Income	Southern	Other Health Workers
27	Mental Health Nurse	3,008	3,404	3,966	1.26	7,910	Mozambique	Maximum Estimate	2221 - Nursing professionals	Low Income	Southern	Nursing Personnel
28	Midwife	39,745	43,394	47,636	15.11	662	Mozambique	Maximum Estimate	2222 - Midwifery professionals	Low Income	Southern	Midwifery Personnel
29	Nephrologist	331	377	444	0.14	70,607	Mozambique	Maximum Estimate	2212 - Specialist medical practitioners	Low Income	Southern	Medical Doctors
30	Neuro-Surgeon	155	177	200	0.06	157,152	Mozambique	Maximum Estimate	2212 - Specialist medical practitioners	Low Income	Southern	Medical Doctors
31	Nurse Anaesthetist	1,072	1,237	1,425	0.45	22,116	Mozambique	Maximum Estimate	2221 - Nursing professionals	Low Income	Southern	Nursing Personnel
32	Nutritionist	4,635	4,914	5,185	1.64	6,080	Mozambique	Maximum Estimate	2265 - Dieticians and nutritionists	Low Income	Southern	Other Health Workers
33	Obstetrician & Gynaecologist	2,948	3,342	3,821	1.22	8,214	Mozambique	Maximum Estimate	2212 - Specialist medical practitioners	Low Income	Southern	Medical Doctors
34	Occupational Therapist	749	851	972	0.31	32,376	Mozambique	Maximum Estimate	2269 - Health professionals not elsewhere classified	Low Income	Southern	Other Health Workers
35	Oncology Nurse	320	367	428	0.14	73,339	Mozambique	Maximum Estimate	2221 - Nursing professionals	Low Income	Southern	Nursing Personnel
36	Operating Theatre Nurse	3,926	4,419	5,075	1.62	6,186	Mozambique	Maximum Estimate	2221 - Nursing professionals	Low Income	Southern	Nursing Personnel
37	Ophthalmic Nurse	420	469	526	0.17	59,789	Mozambique	Maximum Estimate	2221 - Nursing professionals	Low Income	Southern	Nursing Personnel
38	Ophthalmologist	156	175	199	0.06	158,368	Mozambique	Maximum Estimate	2212 - Specialist medical practitioners	Low Income	Southern	Medical Doctors
39	Optometrist	641	707	783	0.25	40,239	Mozambique	Maximum Estimate	2267 - Optometrists and ophthalmic opticians	Low Income	Southern	Other Health Workers
40	Orthopaedic Nurse	814	938	1,076	0.34	29,309	Mozambique	Maximum Estimate	2221 - Nursing professionals	Low Income	Southern	Nursing Personnel
41	Orthopaedic Surgeon	1,194	1,445	1,761	0.56	17,755	Mozambique	Maximum Estimate	2212 - Specialist medical practitioners	Low Income	Southern	Medical Doctors
42	Orthopaedic Technologist	1,381	1,554	1,743	0.55	18,105	Mozambique	Maximum Estimate	3214 - Medical and dental prosthetic technicians	Low Income	Southern	Other Health Workers
43	Paediatric Nurse	5,238	5,776	6,308	2.00	5,011	Mozambique	Maximum Estimate	2221 - Nursing professionals	Low Income	Southern	Nursing Personnel
44	Paediatric Surgeon	192	214	235	0.07	134,666	Mozambique	Maximum Estimate	2212 - Specialist medical practitioners	Low Income	Southern	Medical Doctors
45	Paediatrician	2,404	2,723	3,091	0.98	10,194	Mozambique	Maximum Estimate	2212 - Specialist medical practitioners	Low Income	Southern	Medical Doctors
46	Pathologist	382	428	496	0.16	63,270	Mozambique	Maximum Estimate	2212 - Specialist medical practitioners	Low Income	Southern	Medical Doctors
47	Pharmacist	3,660	3,763	3,919	1.26	7,966	Mozambique	Maximum Estimate	2262 - Pharmacists	Low Income	Southern	Pharmacist
48	Pharmacy Technician	4,516	4,981	5,525	1.76	5,692	Mozambique	Maximum Estimate	3213 - Pharmaceutical technicians and assistants	Low Income	Southern	Other Health Workers
49	Physician	4,478	4,973	5,551	1.76	5,672	Mozambique	Maximum Estimate	2212 - Specialist medical practitioners	Low Income	Southern	Medical Doctors
50	Physiotherapist	1,163	1,305	1,463	0.46	21,551	Mozambique	Maximum Estimate	2264 - Physiotherapists	Low Income	Southern	Other Health Workers
51	Plastic Surgeon	731	835	949	0.30	33,247	Mozambique	Maximum Estimate	2212 - Specialist medical practitioners	Low Income	Southern	Medical Doctors
52	Psychiatrist	2,190	2,455	2,820	0.90	11,132	Mozambique	Maximum Estimate	2212 - Specialist medical practitioners	Low Income	Southern	Medical Doctors
53	Radiation Oncologist	117	137	161	0.05	194,634	Mozambique	Maximum Estimate	2212 - Specialist medical practitioners	Low Income	Southern	Medical Doctors
54	Radiographer (Diagnostics and Therapy)	2,944	3,359	3,910	1.25	8,006	Mozambique	Maximum Estimate	3211 - Medical imaging and therapeutic equipment technicians	Low Income	Southern	Other Health Workers
55	Radiologist	1,213	1,427	1,717	0.55	18,089	Mozambique	Maximum Estimate	2212 - Specialist medical practitioners	Low Income	Southern	Medical Doctors
56	Registered General Nurse / State Certified Nurse	113,474	120,981	130,300	41.54	241	Mozambique	Maximum Estimate	2221 - Nursing professionals	Low Income	Southern	Nursing Personnel
57	Renal Nurse	6,415	7,296	8,577	2.74	3,654	Mozambique	Maximum Estimate	2221 - Nursing professionals	Low Income	Southern	Nursing Personnel
58	Respiratory Physician	571	653	744	0.24	42,370	Mozambique	Maximum Estimate	2212 - Specialist medical practitioners	Low Income	Southern	Medical Doctors
59	Rheumatologist	109	123	137	0.04	229,536	Mozambique	Maximum Estimate	2212 - Specialist medical practitioners	Low Income	Southern	Medical Doctors
60	Speech Therapist	426	476	527	0.17	59,840	Mozambique	Maximum Estimate	2266 - Audiologists and speech therapists	Low Income	Southern	Other Health Workers
61	Urologist	50	60	76	0.02	408,959	Mozambique	Maximum Estimate	2212 - Specialist medical practitioners	Low Income	Southern	Medical Doctors
1	Anaesthesiologist	51	56	63	0.25	40,745	Namibia	Maximum Estimate	2212 - Specialist medical practitioners	High Income and Upper Middle Income	Southern	Medical Doctors
2	Associate Nurse/Enrolled Nurse/Nursing Assistant	5,283	5,739	6,171	24.19	413	Namibia	Maximum Estimate	3221 - Nursing associate professionals	High Income and Upper Middle Income	Southern	Nursing Personnel
3	Audiologist	18	20	21	0.08	120,371	Namibia	Maximum Estimate	2266 - Audiologists and speech therapists	High Income and Upper Middle Income	Southern	Other Health Workers
4	Cardiologist	49	54	59	0.23	43,361	Namibia	Maximum Estimate	2212 - Specialist medical practitioners	High Income and Upper Middle Income	Southern	Medical Doctors
5	Cardiothoracic Surgeon	7	8	9	0.03	287,293	Namibia	Maximum Estimate	2212 - Specialist medical practitioners	High Income and Upper Middle Income	Southern	Medical Doctors
6	Clinical Officer/Physician Assistant	1,643	1,866	1,984	7.74	1,292	Namibia	Maximum Estimate	3256 - Medical assistants	High Income and Upper Middle Income	Southern	Other Health Workers
7	Clinical Pharmacist	114	127	148	0.60	16,604	Namibia	Maximum Estimate	2262 - Pharmacists	High Income and Upper Middle Income	Southern	Pharmacist
8	Clinical Psychologist	179	193	210	0.82	12,144	Namibia	Maximum Estimate	2634 - Psychologists	High Income and Upper Middle Income	Southern	Other Health Workers
9	Community health worker/Village health worker	6,972	8,127	8,542	33.23	301	Namibia	Maximum Estimate	3253 - Community health workers	High Income and Upper Middle Income	Southern	Other Health Workers
10	Dental Surgery Assistant	451	493	540	2.11	4,735	Namibia	Maximum Estimate	3251 - Dental assistants and therapists	High Income and Upper Middle Income	Southern	Other Health Workers
11	Dental Therapist	302	330	361	1.41	7,078	Namibia	Maximum Estimate	3251 - Dental assistants and therapists	High Income and Upper Middle Income	Southern	Other Health Workers
12	Dentist	386	414	446	1.75	5,715	Namibia	Maximum Estimate	2261 - Dentists	High Income and Upper Middle Income	Southern	Dentist
13	Dermatologist	13	15	16	0.06	163,506	Namibia	Maximum Estimate	2212 - Specialist medical practitioners	High Income and Upper Middle Income	Southern	Medical Doctors
14	Endocrinologist	21	24	28	0.11	91,990	Namibia	Maximum Estimate	2212 - Specialist medical practitioners	High Income and Upper Middle Income	Southern	Medical Doctors
15	ENT Surgeon	60	66	73	0.28	35,139	Namibia	Maximum Estimate	2212 - Specialist medical practitioners	High Income and Upper Middle Income	Southern	Medical Doctors
16	Environmental Health Officer	189	201	214	0.84	11,947	Namibia	Maximum Estimate	2263 - Environmental and occupational health and hygiene professionals	High Income and Upper Middle Income	Southern	Other Health Workers
17	Gastroenterologist	27	27	28	0.11	90,993	Namibia	Maximum Estimate	2212 - Specialist medical practitioners	High Income and Upper Middle Income	Southern	Medical Doctors
18	General Medical Practitioner (Generalist Doctor)	2,109	2,326	2,505	9.83	1,017	Namibia	Maximum Estimate	2211 - Generalist medical practitioners	High Income and Upper Middle Income	Southern	Medical Doctors
19	General Surgeon	59	66	74	0.29	34,296	Namibia	Maximum Estimate	2212 - Specialist medical practitioners	High Income and Upper Middle Income	Southern	Medical Doctors
20	Haematologist	9	10	10	0.04	244,228	Namibia	Maximum Estimate	2212 - Specialist medical practitioners	High Income and Upper Middle Income	Southern	Medical Doctors
21	Health Promoter/Health Educator	146	165	179	0.71	14,086	Namibia	Maximum Estimate	2269 - Health professionals not elsewhere classified	High Income and Upper Middle Income	Southern	Other Health Workers
22	Infectious Diseases Specialist	6	6	7	0.03	375,588	Namibia	Maximum Estimate	2212 - Specialist medical practitioners	High Income and Upper Middle Income	Southern	Medical Doctors
23	Intensive Care Nurse	131	140	150	0.59	17,009	Namibia	Maximum Estimate	2221 - Nursing professionals	High Income and Upper Middle Income	Southern	Nursing Personnel
24	Medical Laboratory Scientist	793	907	1,011	4.03	2,479	Namibia	Maximum Estimate	3212 - Medical and pathology laboratory technicians	High Income and Upper Middle Income	Southern	Other Health Workers
25	Medical Laboratory Technician	1,592	1,916	2,069	8.10	1,235	Namibia	Maximum Estimate	3212 - Medical and pathology laboratory technicians	High Income and Upper Middle Income	Southern	Other Health Workers
26	Medical Social Worker	113	130	170	0.76	13,090	Namibia	Maximum Estimate	1344 - Social welfare managers	High Income and Upper Middle Income	Southern	Other Health Workers
27	Mental Health Nurse	283	306	334	1.31	7,609	Namibia	Maximum Estimate	2221 - Nursing professionals	High Income and Upper Middle Income	Southern	Nursing Personnel
28	Midwife	2,611	2,770	2,936	11.50	869	Namibia	Maximum Estimate	2222 - Midwifery professionals	High Income and Upper Middle Income	Southern	Midwifery Personnel
29	Nephrologist	55	63	73	0.29	34,961	Namibia	Maximum Estimate	2212 - Specialist medical practitioners	High Income and Upper Middle Income	Southern	Medical Doctors
30	Neuro-Surgeon	17	19	21	0.08	120,220	Namibia	Maximum Estimate	2212 - Specialist medical practitioners	High Income and Upper Middle Income	Southern	Medical Doctors
31	Nurse Anaesthetist	175	210	253	1.00	10,025	Namibia	Maximum Estimate	2221 - Nursing professionals	High Income and Upper Middle Income	Southern	Nursing Personnel
32	Nutritionist	431	439	446	1.75	5,717	Namibia	Maximum Estimate	2265 - Dieticians and nutritionists	High Income and Upper Middle Income	Southern	Other Health Workers
33	Obstetrician & Gynaecologist	302	343	412	1.71	5,841	Namibia	Maximum Estimate	2212 - Specialist medical practitioners	High Income and Upper Middle Income	Southern	Medical Doctors
34	Occupational Therapist	87	97	109	0.43	23,293	Namibia	Maximum Estimate	2269 - Health professionals not elsewhere classified	High Income and Upper Middle Income	Southern	Other Health Workers
35	Oncology Nurse	56	66	80	0.31	31,840	Namibia	Maximum Estimate	2221 - Nursing professionals	High Income and Upper Middle Income	Southern	Nursing Personnel
36	Operating Theatre Nurse	553	615	693	2.72	3,671	Namibia	Maximum Estimate	2221 - Nursing professionals	High Income and Upper Middle Income	Southern	Nursing Personnel
37	Ophthalmic Nurse	58	64	70	0.27	36,517	Namibia	Maximum Estimate	2221 - Nursing professionals	High Income and Upper Middle Income	Southern	Nursing Personnel
38	Ophthalmologist	25	28	31	0.12	82,709	Namibia	Maximum Estimate	2212 - Specialist medical practitioners	High Income and Upper Middle Income	Southern	Medical Doctors
39	Optometrist	135	150	167	0.65	15,292	Namibia	Maximum Estimate	2267 - Optometrists and ophthalmic opticians	High Income and Upper Middle Income	Southern	Other Health Workers
40	Orthopaedic Nurse	99	111	125	0.49	20,460	Namibia	Maximum Estimate	2221 - Nursing professionals	High Income and Upper Middle Income	Southern	Nursing Personnel
41	Orthopaedic Surgeon	178	204	235	0.92	10,875	Namibia	Maximum Estimate	2212 - Specialist medical practitioners	High Income and Upper Middle Income	Southern	Medical Doctors
42	Orthopaedic Technologist	118	128	139	0.54	18,387	Namibia	Maximum Estimate	3214 - Medical and dental prosthetic technicians	High Income and Upper Middle Income	Southern	Other Health Workers

S/N	Health Professionals	2022	2026	2030	Density per 10,000 population	Required Population ratio (1 professional is to xxx population)	Name of Country	Modelling Scenario	ISCO-08 Match	Income Group Classification	Sub-Regional Grouping	SDG 3c Occupation
43	Paediatric Nurse	328	341	349	1.37	7,321	Namibia	Maximum Estimate	2221 - Nursing professionals	High Income and Upper Middle Income	Southern	Nursing Personnel
44	Paediatric Surgeon	11	12	13	0.05	201,379	Namibia	Maximum Estimate	2212 - Specialist medical practitioners	High Income and Upper Middle Income	Southern	Medical Doctors
45	Paediatrician	134	145	158	0.62	16,090	Namibia	Maximum Estimate	2212 - Specialist medical practitioners	High Income and Upper Middle Income	Southern	Medical Doctors
46	Pathologist	50	56	62	0.25	40,782	Namibia	Maximum Estimate	2212 - Specialist medical practitioners	High Income and Upper Middle Income	Southern	Medical Doctors
47	Pharmacist	611	643	687	2.73	3,667	Namibia	Maximum Estimate	2262 - Pharmacists	High Income and Upper Middle Income	Southern	Pharmacist
48	Pharmacy Technician	580	624	679	2.68	3,727	Namibia	Maximum Estimate	3213 - Pharmaceutical technicians and assistants	High Income and Upper Middle Income	Southern	Other Health Workers
49	Physician	475	525	571	2.23	4,480	Namibia	Maximum Estimate	2212 - Specialist medical practitioners	High Income and Upper Middle Income	Southern	Medical Doctors
50	Physiotherapist	108	117	128	0.50	19,872	Namibia	Maximum Estimate	2264 - Physiotherapists	High Income and Upper Middle Income	Southern	Other Health Workers
51	Plastic Surgeon	37	40	43	0.17	59,034	Namibia	Maximum Estimate	2212 - Specialist medical practitioners	High Income and Upper Middle Income	Southern	Medical Doctors
52	Psychiatrist	212	227	246	0.97	10,347	Namibia	Maximum Estimate	2212 - Specialist medical practitioners	High Income and Upper Middle Income	Southern	Medical Doctors
53	Radiation Oncologist	22	27	34	0.13	74,922	Namibia	Maximum Estimate	2212 - Specialist medical practitioners	High Income and Upper Middle Income	Southern	Medical Doctors
54	Radiographer (Diagnostics and Therapy)	390	443	525	2.14	4,670	Namibia	Maximum Estimate	3211 - Medical imaging and therapeutic equipment technicians	High Income and Upper Middle Income	Southern	Other Health Workers
55	Radiologist	155	191	263	1.16	8,625	Namibia	Maximum Estimate	2212 - Specialist medical practitioners	High Income and Upper Middle Income	Southern	Medical Doctors
56	Registered General Nurse / State Certified Nurse	10,348	10,910	11,656	46.02	217	Namibia	Maximum Estimate	2221 - Nursing professionals	High Income and Upper Middle Income	Southern	Nursing Personnel
57	Renal Nurse	1,091	1,244	1,436	5.66	1,768	Namibia	Maximum Estimate	2221 - Nursing professionals	High Income and Upper Middle Income	Southern	Nursing Personnel
58	Respiratory Physician	24	26	28	0.11	90,652	Namibia	Maximum Estimate	2212 - Specialist medical practitioners	High Income and Upper Middle Income	Southern	Medical Doctors
59	Rheumatologist	12	14	15	0.06	170,811	Namibia	Maximum Estimate	2212 - Specialist medical practitioners	High Income and Upper Middle Income	Southern	Medical Doctors
60	Speech Therapist	35	37	40	0.16	64,300	Namibia	Maximum Estimate	2266 - Audiologists and speech therapists	High Income and Upper Middle Income	Southern	Other Health Workers
61	Urologist	9	11	13	0.05	183,193	Namibia	Maximum Estimate	2212 - Specialist medical practitioners	High Income and Upper Middle Income	Southern	Medical Doctors
1	Anaesthesiologist	283	331	385	0.16	63,228	Niger	Maximum Estimate	2212 - Specialist medical practitioners	Low Income	West	Medical Doctors
2	Associate Nurse/Enrolled Nurse/Nursing Assistant	39,484	45,304	52,031	21.37	468	Niger	Maximum Estimate	3221 - Nursing associate professionals	Low Income	West	Nursing Personnel
3	Audiologist	162	188	217	0.09	112,504	Niger	Maximum Estimate	2266 - Audiologists and speech therapists	Low Income	West	Other Health Workers
4	Cardiologist	261	306	358	0.15	68,075	Niger	Maximum Estimate	2212 - Specialist medical practitioners	Low Income	West	Medical Doctors
5	Cardiothoracic Surgeon	60	71	82	0.03	296,983	Niger	Maximum Estimate	2212 - Specialist medical practitioners	Low Income	West	Medical Doctors
6	Clinical Officer/Physician Assistant	4,981	5,250	5,575	2.30	4,350	Niger	Maximum Estimate	3256 - Medical assistants	Low Income	West	Other Health Workers
7	Clinical Pharmacist	967	1,118	1,304	0.54	18,591	Niger	Maximum Estimate	2262 - Pharmacists	Low Income	West	Pharmacist
8	Clinical Psychologist	1,580	1,812	2,091	0.86	11,623	Niger	Maximum Estimate	2634 - Psychologists	Low Income	West	Other Health Workers
9	Community health worker/Village health worker	46,609	51,286	56,452	23.17	432	Niger	Maximum Estimate	3253 - Community health workers	Low Income	West	Other Health Workers
10	Dental Surgery Assistant	3,235	3,736	4,333	1.78	5,617	Niger	Maximum Estimate	3251 - Dental assistants and therapists	Low Income	West	Other Health Workers
11	Dental Therapist	2,178	2,515	2,917	1.20	8,344	Niger	Maximum Estimate	3251 - Dental assistants and therapists	Low Income	West	Other Health Workers
12	Dentist	2,700	3,140	3,720	1.53	6,518	Niger	Maximum Estimate	2261 - Dentists	Low Income	West	Dentist
13	Dermatologist	116	135	157	0.06	155,502	Niger	Maximum Estimate	2212 - Specialist medical practitioners	Low Income	West	Medical Doctors
14	Endocrinologist	67	92	128	0.05	181,861	Niger	Maximum Estimate	2212 - Specialist medical practitioners	Low Income	West	Medical Doctors
15	ENT Surgeon	381	444	514	0.21	47,389	Niger	Maximum Estimate	2212 - Specialist medical practitioners	Low Income	West	Medical Doctors
16	Environmental Health Officer	1,804	2,088	2,412	0.99	10,110	Niger	Maximum Estimate	2263 - Environmental and occupational health and hygiene professionals	Low Income	West	Other Health Workers
17	Gastroenterologist	381	404	437	0.18	55,474	Niger	Maximum Estimate	2212 - Specialist medical practitioners	Low Income	West	Medical Doctors
18	General Medical Practitioner (Generalist Doctor)	13,529	15,372	17,503	7.19	1,391	Niger	Maximum Estimate	2211 - Generalist medical practitioners	Low Income	West	Medical Doctors
19	General Surgeon	303	356	425	0.18	56,957	Niger	Maximum Estimate	2212 - Specialist medical practitioners	Low Income	West	Medical Doctors
20	Haematologist	152	177	206	0.08	118,056	Niger	Maximum Estimate	2212 - Specialist medical practitioners	Low Income	West	Medical Doctors
21	Health Promoter/Health Educator	525	560	602	0.25	40,302	Niger	Maximum Estimate	2269 - Health professionals not elsewhere classified	Low Income	West	Other Health Workers
22	Infectious Diseases Specialist	40	45	51	0.02	480,930	Niger	Maximum Estimate	2212 - Specialist medical practitioners	Low Income	West	Medical Doctors
23	Intensive Care Nurse	1,404	1,623	1,873	0.77	13,018	Niger	Maximum Estimate	2221 - Nursing professionals	Low Income	West	Nursing Personnel
24	Medical Laboratory Scientist	3,887	4,402	5,041	2.08	4,803	Niger	Maximum Estimate	3212 - Medical and pathology laboratory technicians	Low Income	West	Other Health Workers
25	Medical Laboratory Technician	7,094	7,491	7,950	3.27	3,055	Niger	Maximum Estimate	3212 - Medical and pathology laboratory technicians	Low Income	West	Other Health Workers
26	Medical Social Worker	1,885	2,108	2,367	0.97	10,271	Niger	Maximum Estimate	1344 - Social welfare managers	Low Income	West	Other Health Workers
27	Mental Health Nurse	1,874	2,200	2,618	1.08	9,242	Niger	Maximum Estimate	2221 - Nursing professionals	Low Income	West	Nursing Personnel
28	Midwife	27,046	30,407	34,517	14.20	704	Niger	Maximum Estimate	2222 - Midwifery professionals	Low Income	West	Midwifery Personnel
29	Nephrologist	259	306	367	0.15	65,770	Niger	Maximum Estimate	2212 - Specialist medical practitioners	Low Income	West	Medical Doctors
30	Neuro-Surgeon	63	74	88	0.04	277,845	Niger	Maximum Estimate	2212 - Specialist medical practitioners	Low Income	West	Medical Doctors
31	Nurse Anaesthetist	517	614	731	0.30	33,266	Niger	Maximum Estimate	2221 - Nursing professionals	Low Income	West	Nursing Personnel
32	Nutritionist	9,320	10,533	11,868	4.86	2,057	Niger	Maximum Estimate	2265 - Dieticians and nutritionists	Low Income	West	Other Health Workers
33	Obstetrician & Gynaecologist	1,900	2,211	2,580	1.06	9,421	Niger	Maximum Estimate	2212 - Specialist medical practitioners	Low Income	West	Medical Doctors
34	Occupational Therapist	488	571	669	0.28	36,332	Niger	Maximum Estimate	2269 - Health professionals not elsewhere classified	Low Income	West	Other Health Workers
35	Oncology Nurse	189	225	269	0.11	89,866	Niger	Maximum Estimate	2221 - Nursing professionals	Low Income	West	Nursing Personnel
36	Operating Theatre Nurse	2,530	2,932	3,429	1.41	7,072	Niger	Maximum Estimate	2221 - Nursing professionals	Low Income	West	Nursing Personnel
37	Ophthalmic Nurse	346	415	503	0.21	47,895	Niger	Maximum Estimate	2221 - Nursing professionals	Low Income	West	Nursing Personnel
38	Ophthalmologist	120	143	172	0.07	139,903	Niger	Maximum Estimate	2212 - Specialist medical practitioners	Low Income	West	Medical Doctors
39	Optometrist	595	689	801	0.33	30,289	Niger	Maximum Estimate	2267 - Optometrists and ophthalmic opticians	Low Income	West	Other Health Workers
40	Orthopaedic Nurse	566	669	788	0.32	30,886	Niger	Maximum Estimate	2221 - Nursing professionals	Low Income	West	Nursing Personnel
41	Orthopaedic Surgeon	776	905	1,056	0.43	23,037	Niger	Maximum Estimate	2212 - Specialist medical practitioners	Low Income	West	Medical Doctors
42	Orthopaedic Technologist	950	1,106	1,285	0.53	18,976	Niger	Maximum Estimate	3214 - Medical and dental prosthetic technicians	Low Income	West	Other Health Workers
43	Paediatric Nurse	5,557	6,397	7,303	2.99	3,344	Niger	Maximum Estimate	2221 - Nursing professionals	Low Income	West	Nursing Personnel
44	Paediatric Surgeon	183	215	250	0.10	97,426	Niger	Maximum Estimate	2212 - Specialist medical practitioners	Low Income	West	Medical Doctors
45	Paediatrician	1,605	1,869	2,178	0.89	11,177	Niger	Maximum Estimate	2212 - Specialist medical practitioners	Low Income	West	Medical Doctors
46	Pathologist	211	237	270	0.11	89,956	Niger	Maximum Estimate	2212 - Specialist medical practitioners	Low Income	West	Medical Doctors
47	Pharmacist	2,227	2,423	2,652	1.09	9,148	Niger	Maximum Estimate	2262 - Pharmacists	Low Income	West	Pharmacist
48	Pharmacy Technician	3,646	4,194	4,838	1.99	5,029	Niger	Maximum Estimate	3213 - Pharmaceutical technicians and assistants	Low Income	West	Other Health Workers
49	Physician	3,948	4,529	5,220	2.15	4,653	Niger	Maximum Estimate	2212 - Specialist medical practitioners	Low Income	West	Medical Doctors
50	Physiotherapist	822	955	1,108	0.45	21,995	Niger	Maximum Estimate	2264 - Physiotherapists	Low Income	West	Other Health Workers
51	Plastic Surgeon	205	241	283	0.12	85,957	Niger	Maximum Estimate	2212 - Specialist medical practitioners	Low Income	West	Medical Doctors
52	Psychiatrist	1,922	2,225	2,599	1.07	9,339	Niger	Maximum Estimate	2212 - Specialist medical practitioners	Low Income	West	Medical Doctors
53	Radiation Oncologist	56	67	81	0.03	299,098	Niger	Maximum Estimate	2212 - Specialist medical practitioners	Low Income	West	Medical Doctors
54	Radiographer (Diagnostics and Therapy)	2,259	2,641	3,120	1.29	7,758	Niger	Maximum Estimate	3211 - Medical imaging and therapeutic equipment technicians	Low Income	West	Other Health Workers
55	Radiologist	878	1,037	1,241	0.51	19,427	Niger	Maximum Estimate	2212 - Specialist medical practitioners	Low Income	West	Medical Doctors
56	Registered General Nurse / State Certified Nurse	91,018	100,467	111,581	45.94	218	Niger	Maximum Estimate	2221 - Nursing professionals	Low Income	West	Nursing Personnel
57	Renal Nurse	4,830	5,702	6,847	2.84	3,527	Niger	Maximum Estimate	2221 - Nursing professionals	Low Income	West	Nursing Personnel
58	Respiratory Physician	276	321	373	0.15	65,336	Niger	Maximum Estimate	2212 - Specialist medical practitioners	Low Income	West	Medical Doctors
59	Rheumatologist	82	95	110	0.05	220,837	Niger	Maximum Estimate	2212 - Specialist medical practitioners	Low Income	West	Medical Doctors
60	Speech Therapist	302	352	409	0.17	59,547	Niger	Maximum Estimate	2266 - Audiologists and speech therapists	Low Income	West	Other Health Workers
61	Urologist	40	50	64	0.03	370,059	Niger	Maximum Estimate	2212 - Specialist medical practitioners	Low Income	West	Medical Doctors
1	Anaesthesiologist	2,975	3,340	3,739	0.18	55,583	Nigeria	Maximum Estimate	2212 - Specialist medical practitioners	Lower-middle Income	West	Medical Doctors
2	Associate Nurse/Enrolled Nurse/Nursing Assistant	352,936	382,923	415,224	20.00	500	Nigeria	Maximum Estimate	3221 - Nursing associate professionals	Lower-middle Income	West	Nursing Personnel
3	Audiologist	1,428	1,577	1,734	0.08	119,964	Nigeria	Maximum Estimate	2266 - Audiologists and speech therapists	Lower-middle Income	West	Other Health Workers
4	Cardiologist	2,902	3,255	3,635	0.17	57,218	Nigeria	Maximum Estimate	2212 - Specialist medical practitioners	Lower-middle Income	West	Medical Doctors

S/N	Health Professionals	2022	2026	2030	Density per 10,000 population	Required Population ratio (1 professional is to xxx population)	Name of Country	Modelling Scenario	ISCO-08 Match	Income Group Classification	Sub-Regional Grouping	SDG 3c Occupation
5	Cardiothoracic Surgeon	475	534	594	0.03	349,924	Nigeria	Maximum Estimate	2212 - Specialist medical practitioners	Lower-middle Income	West	Medical Doctors
6	Clinical Officer/Physician Assistant	61,613	62,975	64,594	3.13	3,197	Nigeria	Maximum Estimate	3256 - Medical assistants	Lower-middle Income	West	Other Health Workers
7	Clinical Pharmacist	9,921	10,850	11,928	0.58	17,383	Nigeria	Maximum Estimate	2262 - Pharmacists	Lower-middle Income	West	Pharmacist
8	Clinical Psychologist	15,722	17,295	19,130	0.92	10,839	Nigeria	Maximum Estimate	2634 - Psychologists	Lower-middle Income	West	Other Health Workers
9	Community health worker/Village health worker	316,786	332,601	349,105	16.86	593	Nigeria	Maximum Estimate	3253 - Community health workers	Lower-middle Income	West	Other Health Workers
10	Dental Surgery Assistant	28,602	31,763	35,246	1.70	5,896	Nigeria	Maximum Estimate	3251 - Dental assistants and therapists	Lower-middle Income	West	Other Health Workers
11	Dental Therapist	19,168	21,288	23,623	1.14	8,796	Nigeria	Maximum Estimate	3251 - Dental assistants and therapists	Lower-middle Income	West	Other Health Workers
12	Dentist	21,803	24,408	27,554	1.33	7,527	Nigeria	Maximum Estimate	2261 - Dentists	Lower-middle Income	West	Dentist
13	Dermatologist	1,068	1,174	1,286	0.06	161,759	Nigeria	Maximum Estimate	2212 - Specialist medical practitioners	Lower-middle Income	West	Medical Doctors
14	Endocrinologist	672	782	926	0.04	222,625	Nigeria	Maximum Estimate	2212 - Specialist medical practitioners	Lower-middle Income	West	Medical Doctors
15	ENT Surgeon	4,198	4,685	5,217	0.25	39,828	Nigeria	Maximum Estimate	2212 - Specialist medical practitioners	Lower-middle Income	West	Medical Doctors
16	Environmental Health Officer	15,359	16,860	18,435	0.89	11,284	Nigeria	Maximum Estimate	2263 - Environmental and occupational health and hygiene professionals	Lower-middle Income	West	Other Health Workers
17	Gastroenterologist	4,459	4,521	4,653	0.23	44,292	Nigeria	Maximum Estimate	2212 - Specialist medical practitioners	Lower-middle Income	West	Medical Doctors
18	General Medical Practitioner (Generalist Doctor)	131,701	143,119	155,491	7.49	1,335	Nigeria	Maximum Estimate	2211 - Generalist medical practitioners	Lower-middle Income	West	Medical Doctors
19	General Surgeon	2,499	2,756	3,067	0.15	67,581	Nigeria	Maximum Estimate	2212 - Specialist medical practitioners	Lower-middle Income	West	Medical Doctors
20	Haematologist	1,548	1,738	1,943	0.09	107,049	Nigeria	Maximum Estimate	2212 - Specialist medical practitioners	Lower-middle Income	West	Medical Doctors
21	Health Promoter/Health Educator	5,875	6,076	6,303	0.30	32,815	Nigeria	Maximum Estimate	2269 - Health professionals not elsewhere classified	Lower-middle Income	West	Other Health Workers
22	Infectious Diseases Specialist	340	366	397	0.02	521,212	Nigeria	Maximum Estimate	2212 - Specialist medical practitioners	Lower-middle Income	West	Medical Doctors
23	Intensive Care Nurse	10,923	11,912	12,952	0.62	16,049	Nigeria	Maximum Estimate	2221 - Nursing professionals	Lower-middle Income	West	Nursing Personnel
24	Medical Laboratory Scientist	36,215	38,910	42,109	2.03	4,916	Nigeria	Maximum Estimate	3212 - Medical and pathology laboratory technicians	Lower-middle Income	West	Other Health Workers
25	Medical Laboratory Technician	53,453	53,957	54,781	2.66	3,760	Nigeria	Maximum Estimate	3212 - Medical and pathology laboratory technicians	Lower-middle Income	West	Other Health Workers
26	Medical Social Worker	14,482	15,023	15,603	0.75	13,271	Nigeria	Maximum Estimate	1344 - Social welfare managers	Lower-middle Income	West	Other Health Workers
27	Mental Health Nurse	15,852	17,059	18,619	0.90	11,107	Nigeria	Maximum Estimate	2221 - Nursing professionals	Lower-middle Income	West	Nursing Personnel
28	Midwife	239,394	255,862	274,210	13.23	756	Nigeria	Maximum Estimate	2222 - Midwifery professionals	Lower-middle Income	West	Midwifery Personnel
29	Nephrologist	2,642	2,954	3,355	0.16	61,610	Nigeria	Maximum Estimate	2212 - Specialist medical practitioners	Lower-middle Income	West	Medical Doctors
30	Neuro-Surgeon	741	829	929	0.04	223,137	Nigeria	Maximum Estimate	2212 - Specialist medical practitioners	Lower-middle Income	West	Medical Doctors
31	Nurse Anaesthetist	6,108	6,934	7,858	0.38	26,437	Nigeria	Maximum Estimate	2221 - Nursing professionals	Lower-middle Income	West	Nursing Personnel
32	Nutritionist	56,923	60,165	63,319	3.05	3,280	Nigeria	Maximum Estimate	2265 - Dieticians and nutritionists	Lower-middle Income	West	Other Health Workers
33	Obstetrician & Gynaecologist	18,029	19,946	22,064	1.06	9,417	Nigeria	Maximum Estimate	2212 - Specialist medical practitioners	Lower-middle Income	West	Medical Doctors
34	Occupational Therapist	5,468	6,125	6,888	0.33	30,109	Nigeria	Maximum Estimate	2269 - Health professionals not elsewhere classified	Lower-middle Income	West	Other Health Workers
35	Oncology Nurse	2,160	2,431	2,760	0.13	75,025	Nigeria	Maximum Estimate	2221 - Nursing professionals	Lower-middle Income	West	Nursing Personnel
36	Operating Theatre Nurse	33,250	36,893	41,401	2.00	4,999	Nigeria	Maximum Estimate	2221 - Nursing professionals	Lower-middle Income	West	Nursing Personnel
37	Ophthalmic Nurse	3,310	3,657	4,046	0.19	51,294	Nigeria	Maximum Estimate	2221 - Nursing professionals	Lower-middle Income	West	Nursing Personnel
38	Ophthalmologist	1,260	1,399	1,557	0.08	133,237	Nigeria	Maximum Estimate	2212 - Specialist medical practitioners	Lower-middle Income	West	Medical Doctors
39	Optometrist	4,817	5,335	5,901	0.28	35,212	Nigeria	Maximum Estimate	2267 - Optometrists and ophthalmic opticians	Lower-middle Income	West	Other Health Workers
40	Orthopaedic Nurse	5,437	6,146	6,917	0.33	30,064	Nigeria	Maximum Estimate	2221 - Nursing professionals	Lower-middle Income	West	Nursing Personnel
41	Orthopaedic Surgeon	10,268	11,525	12,950	0.62	16,034	Nigeria	Maximum Estimate	2212 - Specialist medical practitioners	Lower-middle Income	West	Medical Doctors
42	Orthopaedic Technologist	8,387	9,294	10,262	0.49	20,267	Nigeria	Maximum Estimate	3214 - Medical and dental prosthetic technicians	Lower-middle Income	West	Other Health Workers
43	Paediatric Nurse	43,618	47,730	51,696	2.49	4,023	Nigeria	Maximum Estimate	2221 - Nursing professionals	Lower-middle Income	West	Nursing Personnel
44	Paediatric Surgeon	1,255	1,373	1,483	0.07	140,396	Nigeria	Maximum Estimate	2212 - Specialist medical practitioners	Lower-middle Income	West	Medical Doctors
45	Paediatrician	17,370	19,165	21,129	1.02	9,831	Nigeria	Maximum Estimate	2212 - Specialist medical practitioners	Lower-middle Income	West	Medical Doctors
46	Pathologist	1,970	2,190	2,472	0.12	83,618	Nigeria	Maximum Estimate	2212 - Specialist medical practitioners	Lower-middle Income	West	Medical Doctors
47	Pharmacist	37,841	39,320	40,823	1.97	5,075	Nigeria	Maximum Estimate	2262 - Pharmacists	Lower-middle Income	West	Pharmacist
48	Pharmacy Technician	41,605	45,370	49,459	2.38	4,199	Nigeria	Maximum Estimate	3213 - Pharmaceutical technicians and assistants	Lower-middle Income	West	Other Health Workers
49	Physician	36,978	40,363	44,160	2.13	4,698	Nigeria	Maximum Estimate	2212 - Specialist medical practitioners	Lower-middle Income	West	Medical Doctors
50	Physiotherapist	7,626	8,427	9,309	0.45	22,315	Nigeria	Maximum Estimate	2264 - Physiotherapists	Lower-middle Income	West	Other Health Workers
51	Plastic Surgeon	1,843	2,097	2,378	0.11	87,420	Nigeria	Maximum Estimate	2212 - Specialist medical practitioners	Lower-middle Income	West	Medical Doctors
52	Psychiatrist	15,480	16,689	18,178	0.88	11,389	Nigeria	Maximum Estimate	2212 - Specialist medical practitioners	Lower-middle Income	West	Medical Doctors
53	Radiation Oncologist	665	755	861	0.04	240,737	Nigeria	Maximum Estimate	2212 - Specialist medical practitioners	Lower-middle Income	West	Medical Doctors
54	Radiographer (Diagnostics and Therapy)	24,913	27,620	30,829	1.49	6,724	Nigeria	Maximum Estimate	3211 - Medical imaging and therapeutic equipment technicians	Lower-middle Income	West	Other Health Workers
55	Radiologist	9,613	10,623	11,780	0.57	17,616	Nigeria	Maximum Estimate	2212 - Specialist medical practitioners	Lower-middle Income	West	Medical Doctors
56	Registered General Nurse / State Certified Nurse	717,494	788,649	865,976	41.71	240	Nigeria	Maximum Estimate	2221 - Nursing professionals	Lower-middle Income	West	Nursing Personnel
57	Renal Nurse	51,601	57,714	65,590	3.17	3,151	Nigeria	Maximum Estimate	2221 - Nursing professionals	Lower-middle Income	West	Nursing Personnel
58	Respiratory Physician	2,903	3,170	3,451	0.17	60,245	Nigeria	Maximum Estimate	2212 - Specialist medical practitioners	Lower-middle Income	West	Medical Doctors
59	Rheumatologist	790	876	970	0.05	214,424	Nigeria	Maximum Estimate	2212 - Specialist medical practitioners	Lower-middle Income	West	Medical Doctors
60	Speech Therapist	2,660	2,936	3,237	0.16	64,189	Nigeria	Maximum Estimate	2266 - Audiologists and speech therapists	Lower-middle Income	West	Other Health Workers
61	Urologist	422	457	503	0.02	411,391	Nigeria	Maximum Estimate	2212 - Specialist medical practitioners	Lower-middle Income	West	Medical Doctors
1	Anaesthesiologist	248	282	321	0.25	40,593	Rwanda	Maximum Estimate	2212 - Specialist medical practitioners	Low Income	East	Medical Doctors
2	Associate Nurse/Enrolled Nurse/Nursing Assistant	20,921	23,928	26,346	20.19	495	Rwanda	Maximum Estimate	3221 - Nursing associate professionals	Low Income	East	Nursing Personnel
3	Audiologist	91	102	112	0.09	116,264	Rwanda	Maximum Estimate	2266 - Audiologists and speech therapists	Low Income	East	Other Health Workers
4	Cardiologist	213	253	299	0.23	43,573	Rwanda	Maximum Estimate	2212 - Specialist medical practitioners	Low Income	East	Medical Doctors
5	Cardiothoracic Surgeon	51	57	65	0.05	202,387	Rwanda	Maximum Estimate	2212 - Specialist medical practitioners	Low Income	East	Medical Doctors
6	Clinical Officer/Physician Assistant	5,382	7,050	7,560	5.70	1,753	Rwanda	Maximum Estimate	3256 - Medical assistants	Low Income	East	Other Health Workers
7	Clinical Pharmacist	580	645	739	0.59	16,999	Rwanda	Maximum Estimate	2262 - Pharmacists	Low Income	East	Pharmacist
8	Clinical Psychologist	1,100	1,182	1,275	0.98	10,219	Rwanda	Maximum Estimate	2634 - Psychologists	Low Income	East	Other Health Workers
9	Community health worker/Village health worker	29,867	40,690	43,744	32.78	305	Rwanda	Maximum Estimate	3253 - Community health workers	Low Income	East	Other Health Workers
10	Dental Surgery Assistant	2,158	2,445	2,777	2.13	4,705	Rwanda	Maximum Estimate	3251 - Dental assistants and therapists	Low Income	East	Other Health Workers
11	Dental Therapist	1,446	1,638	1,858	1.42	7,030	Rwanda	Maximum Estimate	3251 - Dental assistants and therapists	Low Income	East	Other Health Workers
12	Dentist	1,887	2,090	2,345	1.79	5,577	Rwanda	Maximum Estimate	2261 - Dentists	Low Income	East	Dentist
13	Dermatologist	65	73	81	0.06	160,708	Rwanda	Maximum Estimate	2212 - Specialist medical practitioners	Low Income	East	Medical Doctors
14	Endocrinologist	65	79	97	0.08	133,065	Rwanda	Maximum Estimate	2212 - Specialist medical practitioners	Low Income	East	Medical Doctors
15	ENT Surgeon	264	303	352	0.27	36,768	Rwanda	Maximum Estimate	2212 - Specialist medical practitioners	Low Income	East	Medical Doctors
16	Environmental Health Officer	965	1,055	1,147	0.88	11,403	Rwanda	Maximum Estimate	2263 - Environmental and occupational health and hygiene professionals	Low Income	East	Other Health Workers
17	Gastroenterologist	144	147	153	0.12	84,422	Rwanda	Maximum Estimate	2212 - Specialist medical practitioners	Low Income	East	Medical Doctors
18	General Medical Practitioner (Generalist Doctor)	8,401	9,990	11,098	8.53	1,172	Rwanda	Maximum Estimate	2211 - Generalist medical practitioners	Low Income	East	Medical Doctors
19	General Surgeon	182	224	282	0.22	45,264	Rwanda	Maximum Estimate	2212 - Specialist medical practitioners	Low Income	East	Medical Doctors
20	Haematologist	49	53	56	0.04	230,727	Rwanda	Maximum Estimate	2212 - Specialist medical practitioners	Low Income	East	Medical Doctors
21	Health Promoter/Health Educator	463	591	640	0.50	20,192	Rwanda	Maximum Estimate	2269 - Health professionals not elsewhere classified	Low Income	East	Other Health Workers
22	Infectious Diseases Specialist	20	22	23	0.02	561,845	Rwanda	Maximum Estimate	2212 - Specialist medical practitioners	Low Income	East	Medical Doctors
23	Intensive Care Nurse	616	676	739	0.57	17,673	Rwanda	Maximum Estimate	2221 - Nursing professionals	Low Income	East	Nursing Personnel
24	Medical Laboratory Scientist	2,939	3,716	4,199	3.27	3,060	Rwanda	Maximum Estimate	3212 - Medical and pathology laboratory technicians	Low Income	East	Other Health Workers
25	Medical Laboratory Technician	6,810	9,583	10,457	7.89	1,268	Rwanda	Maximum Estimate	3212 - Medical and pathology laboratory technicians	Low Income	East	Other Health Workers
26	Medical Social Worker	475	531	663	0.60	16,539	Rwanda	Maximum Estimate	1344 - Social welfare managers	Low Income	East	Other Health Workers
27	Mental Health Nurse	1,546	1,746	2,006	1.54	6,482	Rwanda	Maximum Estimate	2221 - Nursing professionals	Low Income	East	Nursing Personnel

S/N	Health Professionals	2022	2026	2030	Density per 10,000 population	Required Population ratio (1 professional is to xxx population)	Name of Country	Modelling Scenario	ISCO-08 Match	Income Group Classification	Sub-Regional Grouping	SDG 3c Occupation
28	Midwife	14,926	15,980	17,149	13.14	761	Rwanda	Maximum Estimate	2222 - Midwifery professionals	Low Income	East	Midwifery Personnel
29	Nephrologist	195	228	270	0.21	47,994	Rwanda	Maximum Estimate	2212 - Specialist medical practitioners	Low Income	East	Medical Doctors
30	Neuro-Surgeon	72	83	98	0.08	130,490	Rwanda	Maximum Estimate	2212 - Specialist medical practitioners	Low Income	East	Medical Doctors
31	Nurse Anaesthetist	583	698	835	0.64	15,567	Rwanda	Maximum Estimate	2221 - Nursing professionals	Low Income	East	Nursing Personnel
32	Nutritionist	1,617	1,670	1,711	1.31	7,615	Rwanda	Maximum Estimate	2265 - Dieticians and nutritionists	Low Income	East	Other Health Workers
33	Obstetrician & Gynaecologist	1,272	1,439	1,701	1.40	7,135	Rwanda	Maximum Estimate	2212 - Specialist medical practitioners	Low Income	East	Medical Doctors
34	Occupational Therapist	394	453	525	0.40	24,768	Rwanda	Maximum Estimate	2269 - Health professionals not elsewhere classified	Low Income	East	Other Health Workers
35	Oncology Nurse	189	226	273	0.21	47,520	Rwanda	Maximum Estimate	2221 - Nursing professionals	Low Income	East	Nursing Personnel
36	Operating Theatre Nurse	2,334	2,688	3,160	2.44	4,097	Rwanda	Maximum Estimate	2221 - Nursing professionals	Low Income	East	Nursing Personnel
37	Ophthalmic Nurse	180	206	238	0.18	54,773	Rwanda	Maximum Estimate	2221 - Nursing professionals	Low Income	East	Nursing Personnel
38	Ophthalmologist	83	97	114	0.09	114,503	Rwanda	Maximum Estimate	2212 - Specialist medical practitioners	Low Income	East	Medical Doctors
39	Optometrist	387	453	530	0.41	24,574	Rwanda	Maximum Estimate	2267 - Optometrists and ophthalmic opticians	Low Income	East	Other Health Workers
40	Orthopaedic Nurse	303	355	415	0.32	31,413	Rwanda	Maximum Estimate	2221 - Nursing professionals	Low Income	East	Nursing Personnel
41	Orthopaedic Surgeon	711	979	1,378	1.11	8,990	Rwanda	Maximum Estimate	2212 - Specialist medical practitioners	Low Income	East	Medical Doctors
42	Orthopaedic Technologist	553	615	682	0.52	19,166	Rwanda	Maximum Estimate	3214 - Medical and dental prosthetic technicians	Low Income	East	Other Health Workers
43	Paediatric Nurse	1,922	2,022	2,079	1.59	6,281	Rwanda	Maximum Estimate	2221 - Nursing professionals	Low Income	East	Nursing Personnel
44	Paediatric Surgeon	63	67	70	0.05	187,308	Rwanda	Maximum Estimate	2212 - Specialist medical practitioners	Low Income	East	Medical Doctors
45	Paediatrician	1,249	1,300	1,354	1.04	9,604	Rwanda	Maximum Estimate	2212 - Specialist medical practitioners	Low Income	East	Medical Doctors
46	Pathologist	189	211	240	0.18	54,208	Rwanda	Maximum Estimate	2212 - Specialist medical practitioners	Low Income	East	Medical Doctors
47	Pharmacist	1,764	1,771	1,824	1.45	6,912	Rwanda	Maximum Estimate	2262 - Pharmacists	Low Income	East	Pharmacist
48	Pharmacy Technician	2,161	2,348	2,585	2.01	4,971	Rwanda	Maximum Estimate	3213 - Pharmaceutical technicians and assistants	Low Income	East	Other Health Workers
49	Physician	2,181	2,523	2,794	2.14	4,684	Rwanda	Maximum Estimate	2212 - Specialist medical practitioners	Low Income	East	Medical Doctors
50	Physiotherapist	501	560	630	0.48	20,656	Rwanda	Maximum Estimate	2264 - Physiotherapists	Low Income	East	Other Health Workers
51	Plastic Surgeon	315	363	415	0.32	31,457	Rwanda	Maximum Estimate	2212 - Specialist medical practitioners	Low Income	East	Medical Doctors
52	Psychiatrist	1,084	1,210	1,372	1.05	9,486	Rwanda	Maximum Estimate	2212 - Specialist medical practitioners	Low Income	East	Medical Doctors
53	Radiation Oncologist	76	94	118	0.09	109,852	Rwanda	Maximum Estimate	2212 - Specialist medical practitioners	Low Income	East	Medical Doctors
54	Radiographer (Diagnostics and Therapy)	1,488	1,760	2,169	1.76	5,682	Rwanda	Maximum Estimate	3211 - Medical imaging and therapeutic equipment technicians	Low Income	East	Other Health Workers
55	Radiologist	545	694	978	0.89	11,254	Rwanda	Maximum Estimate	2212 - Specialist medical practitioners	Low Income	East	Medical Doctors
56	Registered General Nurse / State Certified Nurse	41,434	45,591	50,543	39.03	256	Rwanda	Maximum Estimate	2221 - Nursing professionals	Low Income	East	Nursing Personnel
57	Renal Nurse	3,857	4,503	5,357	4.13	2,419	Rwanda	Maximum Estimate	2221 - Nursing professionals	Low Income	East	Nursing Personnel
58	Respiratory Physician	327	334	340	0.26	38,233	Rwanda	Maximum Estimate	2212 - Specialist medical practitioners	Low Income	East	Medical Doctors
59	Rheumatologist	55	63	72	0.05	182,461	Rwanda	Maximum Estimate	2212 - Specialist medical practitioners	Low Income	East	Medical Doctors
60	Speech Therapist	175	193	216	0.17	59,910	Rwanda	Maximum Estimate	2266 - Audiologists and speech therapists	Low Income	East	Other Health Workers
61	Urologist	29	37	48	0.04	262,285	Rwanda	Maximum Estimate	2212 - Specialist medical practitioners	Low Income	East	Medical Doctors
1	Anaesthesiologist	4	5	6	0.26	38,662	Sao Tome and Princ	Maximum Estimate	2212 - Specialist medical practitioners	Lower-middle Income	West	Medical Doctors
2	Associate Nurse/Enrolled Nurse/Nursing Assistant	376	413	457	20.80	481	Sao Tome and Princ	Maximum Estimate	3221 - Nursing associate professionals	Lower-middle Income	West	Nursing Personnel
3	Audiologist	1	2	2	0.08	124,001	Sao Tome and Princ	Maximum Estimate	2266 - Audiologists and speech therapists	Lower-middle Income	West	Other Health Workers
4	Cardiologist	5	5	6	0.25	39,261	Sao Tome and Princ	Maximum Estimate	2212 - Specialist medical practitioners	Lower-middle Income	West	Medical Doctors
5	Cardiothoracic Surgeon	1	1	1	0.05	212,805	Sao Tome and Princ	Maximum Estimate	2212 - Specialist medical practitioners	Lower-middle Income	West	Medical Doctors
6	Clinical Officer/Physician Assistant	45	45	46	2.11	4,742	Sao Tome and Princ	Maximum Estimate	3256 - Medical assistants	Lower-middle Income	West	Other Health Workers
7	Clinical Pharmacist	11	12	14	0.64	15,696	Sao Tome and Princ	Maximum Estimate	2262 - Pharmacists	Lower-middle Income	West	Pharmacist
8	Clinical Psychologist	22	24	27	1.21	8,252	Sao Tome and Princ	Maximum Estimate	2634 - Psychologists	Lower-middle Income	West	Other Health Workers
9	Community health worker/Village health worker	209	216	224	10.18	982	Sao Tome and Princ	Maximum Estimate	3253 - Community health workers	Lower-middle Income	West	Other Health Workers
10	Dental Surgery Assistant	42	47	53	2.41	4,158	Sao Tome and Princ	Maximum Estimate	3251 - Dental assistants and therapists	Lower-middle Income	West	Other Health Workers
11	Dental Therapist	28	31	35	1.60	6,235	Sao Tome and Princ	Maximum Estimate	3251 - Dental assistants and therapists	Lower-middle Income	West	Other Health Workers
12	Dentist	31	33	38	1.71	5,849	Sao Tome and Princ	Maximum Estimate	2261 - Dentists	Lower-middle Income	West	Dentist
13	Dermatologist	1	1	1	0.07	148,177	Sao Tome and Princ	Maximum Estimate	2212 - Specialist medical practitioners	Lower-middle Income	West	Medical Doctors
14	Endocrinologist	1	2	2	0.09	112,237	Sao Tome and Princ	Maximum Estimate	2212 - Specialist medical practitioners	Lower-middle Income	West	Medical Doctors
15	ENT Surgeon	5	5	6	0.26	38,361	Sao Tome and Princ	Maximum Estimate	2212 - Specialist medical practitioners	Lower-middle Income	West	Medical Doctors
16	Environmental Health Officer	16	18	19	0.86	11,606	Sao Tome and Princ	Maximum Estimate	2263 - Environmental and occupational health and hygiene professionals	Lower-middle Income	West	Other Health Workers
17	Gastroenterologist	4	4	4	0.18	55,523	Sao Tome and Princ	Maximum Estimate	2212 - Specialist medical practitioners	Lower-middle Income	West	Medical Doctors
18	General Medical Practitioner (Generalist Doctor)	126	137	150	6.84	1,462	Sao Tome and Princ	Maximum Estimate	2211 - Generalist medical practitioners	Lower-middle Income	West	Medical Doctors
19	General Surgeon	4	4	5	0.22	46,483	Sao Tome and Princ	Maximum Estimate	2212 - Specialist medical practitioners	Lower-middle Income	West	Medical Doctors
20	Haematologist	1	1	1	0.06	160,531	Sao Tome and Princ	Maximum Estimate	2212 - Specialist medical practitioners	Lower-middle Income	West	Medical Doctors
21	Health Promoter/Health Educator	4	4	4	0.18	54,738	Sao Tome and Princ	Maximum Estimate	2269 - Health professionals not elsewhere classified	Lower-middle Income	West	Other Health Workers
22	Infectious Diseases Specialist	0	0	1	0.02	416,768	Sao Tome and Princ	Maximum Estimate	2212 - Specialist medical practitioners	Lower-middle Income	West	Medical Doctors
23	Intensive Care Nurse	10	11	11	0.52	19,164	Sao Tome and Princ	Maximum Estimate	2221 - Nursing professionals	Lower-middle Income	West	Nursing Personnel
24	Medical Laboratory Scientist	35	39	43	1.98	5,060	Sao Tome and Princ	Maximum Estimate	3212 - Medical and pathology laboratory technicians	Lower-middle Income	West	Other Health Workers
25	Medical Laboratory Technician	55	72	98	4.66	2,148	Sao Tome and Princ	Maximum Estimate	3212 - Medical and pathology laboratory technicians	Lower-middle Income	West	Other Health Workers
26	Medical Social Worker	10	10	11	0.50	20,192	Sao Tome and Princ	Maximum Estimate	1344 - Social welfare managers	Lower-middle Income	West	Other Health Workers
27	Mental Health Nurse	21	24	27	1.24	8,061	Sao Tome and Princ	Maximum Estimate	2221 - Nursing professionals	Lower-middle Income	West	Nursing Personnel
28	Midwife	313	387	496	23.31	429	Sao Tome and Princ	Maximum Estimate	2222 - Midwifery professionals	Lower-middle Income	West	Midwifery Personnel
29	Nephrologist	4	5	6	0.27	37,709	Sao Tome and Princ	Maximum Estimate	2212 - Specialist medical practitioners	Lower-middle Income	West	Medical Doctors
30	Neuro-Surgeon	2	2	2	0.10	100,034	Sao Tome and Princ	Maximum Estimate	2212 - Specialist medical practitioners	Lower-middle Income	West	Medical Doctors
31	Nurse Anaesthetist	11	13	16	0.72	13,840	Sao Tome and Princ	Maximum Estimate	2221 - Nursing professionals	Lower-middle Income	West	Nursing Personnel
32	Nutritionist	31	30	30	1.36	7,327	Sao Tome and Princ	Maximum Estimate	2265 - Dieticians and nutritionists	Lower-middle Income	West	Other Health Workers
33	Obstetrician & Gynaecologist	21	24	27	1.22	8,229	Sao Tome and Princ	Maximum Estimate	2212 - Specialist medical practitioners	Lower-middle Income	West	Medical Doctors
34	Occupational Therapist	8	9	11	0.51	19,769	Sao Tome and Princ	Maximum Estimate	2269 - Health professionals not elsewhere classified	Lower-middle Income	West	Other Health Workers
35	Oncology Nurse	4	4	5	0.24	41,700	Sao Tome and Princ	Maximum Estimate	2221 - Nursing professionals	Lower-middle Income	West	Nursing Personnel
36	Operating Theatre Nurse	39	44	52	2.37	4,220	Sao Tome and Princ	Maximum Estimate	2221 - Nursing professionals	Lower-middle Income	West	Nursing Personnel
37	Ophthalmic Nurse	4	5	5	0.24	41,368	Sao Tome and Princ	Maximum Estimate	2221 - Nursing professionals	Lower-middle Income	West	Nursing Personnel
38	Ophthalmologist	2	2	2	0.09	110,336	Sao Tome and Princ	Maximum Estimate	2212 - Specialist medical practitioners	Lower-middle Income	West	Medical Doctors
39	Optometrist	6	7	8	0.37	27,018	Sao Tome and Princ	Maximum Estimate	2267 - Optometrists and ophthalmic opticians	Lower-middle Income	West	Other Health Workers
40	Orthopaedic Nurse	10	12	14	0.64	15,518	Sao Tome and Princ	Maximum Estimate	2221 - Nursing professionals	Lower-middle Income	West	Nursing Personnel
41	Orthopaedic Surgeon	14	16	19	0.87	11,556	Sao Tome and Princ	Maximum Estimate	2212 - Specialist medical practitioners	Lower-middle Income	West	Medical Doctors
42	Orthopaedic Technologist	11	12	14	0.62	16,243	Sao Tome and Princ	Maximum Estimate	3214 - Medical and dental prosthetic technicians	Lower-middle Income	West	Other Health Workers
43	Paediatric Nurse	34	35	37	1.68	5,965	Sao Tome and Princ	Maximum Estimate	2221 - Nursing professionals	Lower-middle Income	West	Nursing Personnel
44	Paediatric Surgeon	1	1	1	0.05	216,730	Sao Tome and Princ	Maximum Estimate	2212 - Specialist medical practitioners	Lower-middle Income	West	Medical Doctors
45	Paediatrician	17	18	19	0.88	11,347	Sao Tome and Princ	Maximum Estimate	2212 - Specialist medical practitioners	Lower-middle Income	West	Medical Doctors
46	Pathologist	3	3	4	0.17	60,181	Sao Tome and Princ	Maximum Estimate	2212 - Specialist medical practitioners	Lower-middle Income	West	Medical Doctors
47	Pharmacist	27	27	28	1.27	7,897	Sao Tome and Princ	Maximum Estimate	2262 - Pharmacists	Lower-middle Income	West	Pharmacist
48	Pharmacy Technician	42	46	51	2.32	4,313	Sao Tome and Princ	Maximum Estimate	3213 - Pharmaceutical technicians and assistants	Lower-middle Income	West	Other Health Workers
49	Physician	44	51	59	2.74	3,655	Sao Tome and Princ	Maximum Estimate	2212 - Specialist medical practitioners	Lower-middle Income	West	Medical Doctors
50	Physiotherapist	9	11	12	0.54	18,456	Sao Tome and Princ	Maximum Estimate	2264 - Physiotherapists	Lower-middle Income	West	Other Health Workers

S/N	Health Professionals	2022	2026	2030	Density per 10,000 population	Required Population ratio (1 professional is to xxx population)	Name of Country	Modelling Scenario	ISCO-08 Match	Income Group Classification	Sub-Regional Grouping	SDG 3c Occupation
51	Plastic Surgeon	3	4	4	0.20	50,811	Sao Tome and Princ	Maximum Estimate	2212 - Specialist medical practitioners	Lower-middle Income	West	Medical Doctors
52	Psychiatrist	17	19	21	0.96	10,397	Sao Tome and Princ	Maximum Estimate	2212 - Specialist medical practitioners	Lower-middle Income	West	Medical Doctors
53	Radiation Oncologist	2	2	2	0.11	93,029	Sao Tome and Princ	Maximum Estimate	2212 - Specialist medical practitioners	Lower-middle Income	West	Medical Doctors
54	Radiographer (Diagnostics and Therapy)	32	37	42	1.93	5,191	Sao Tome and Princ	Maximum Estimate	3211 - Medical imaging and therapeutic equipment technicians	Lower-middle Income	West	Other Health Workers
55	Radiologist	12	14	16	0.75	13,384	Sao Tome and Princ	Maximum Estimate	2212 - Specialist medical practitioners	Lower-middle Income	West	Medical Doctors
56	Registered General Nurse / State Certified Nurse	809	901	1,017	46.40	216	Sao Tome and Princ	Maximum Estimate	2221 - Nursing professionals	Lower-middle Income	West	Nursing Personnel
57	Renal Nurse	84	96	113	5.17	1,936	Sao Tome and Princ	Maximum Estimate	2221 - Nursing professionals	Lower-middle Income	West	Nursing Personnel
58	Respiratory Physician	3	4	4	0.19	53,934	Sao Tome and Princ	Maximum Estimate	2212 - Specialist medical practitioners	Lower-middle Income	West	Medical Doctors
59	Rheumatologist	1	1	1	0.05	186,111	Sao Tome and Princ	Maximum Estimate	2212 - Specialist medical practitioners	Lower-middle Income	West	Medical Doctors
60	Speech Therapist	3	3	4	0.17	58,162	Sao Tome and Princ	Maximum Estimate	2266 - Audiologists and speech therapists	Lower-middle Income	West	Other Health Workers
61	Urologist	1	1	1	0.04	236,362	Sao Tome and Princ	Maximum Estimate	2212 - Specialist medical practitioners	Lower-middle Income	West	Medical Doctors
1	Anaesthesiologist	275	315	359	0.21	46,858	Senegal	Maximum Estimate	2212 - Specialist medical practitioners	Lower-middle Income	West	Medical Doctors
2	Associate Nurse/Enrolled Nurse/Nursing Assistant	32,358	37,410	43,439	25.90	386	Senegal	Maximum Estimate	3221 - Nursing associate professionals	Lower-middle Income	West	Nursing Personnel
3	Audiologist	112	126	140	0.08	120,584	Senegal	Maximum Estimate	2266 - Audiologists and speech therapists	Lower-middle Income	West	Other Health Workers
4	Cardiologist	256	289	327	0.19	51,597	Senegal	Maximum Estimate	2212 - Specialist medical practitioners	Lower-middle Income	West	Medical Doctors
5	Cardiothoracic Surgeon	51	58	65	0.04	259,801	Senegal	Maximum Estimate	2212 - Specialist medical practitioners	Lower-middle Income	West	Medical Doctors
6	Clinical Officer/Physician Assistant	2,884	2,954	3,072	1.84	5,434	Senegal	Maximum Estimate	3256 - Medical assistants	Lower-middle Income	West	Other Health Workers
7	Clinical Pharmacist	740	826	932	0.56	18,009	Senegal	Maximum Estimate	2262 - Pharmacists	Lower-middle Income	West	Pharmacist
8	Clinical Psychologist	1,250	1,389	1,555	0.92	10,813	Senegal	Maximum Estimate	2634 - Psychologists	Lower-middle Income	West	Other Health Workers
9	Community health worker/Village health worker	20,422	21,697	23,025	13.69	731	Senegal	Maximum Estimate	3253 - Community health workers	Lower-middle Income	West	Other Health Workers
10	Dental Surgery Assistant	2,967	3,301	3,698	2.20	4,552	Senegal	Maximum Estimate	3251 - Dental assistants and therapists	Lower-middle Income	West	Other Health Workers
11	Dental Therapist	1,987	2,209	2,474	1.47	6,804	Senegal	Maximum Estimate	3251 - Dental assistants and therapists	Lower-middle Income	West	Other Health Workers
12	Dentist	2,582	2,834	3,172	1.89	5,295	Senegal	Maximum Estimate	2261 - Dentists	Lower-middle Income	West	Dentist
13	Dermatologist	88	99	110	0.07	153,031	Senegal	Maximum Estimate	2212 - Specialist medical practitioners	Lower-middle Income	West	Medical Doctors
14	Endocrinologist	110	125	145	0.09	115,341	Senegal	Maximum Estimate	2212 - Specialist medical practitioners	Lower-middle Income	West	Medical Doctors
15	ENT Surgeon	319	361	408	0.24	41,257	Senegal	Maximum Estimate	2212 - Specialist medical practitioners	Lower-middle Income	West	Medical Doctors
16	Environmental Health Officer	1,248	1,379	1,519	0.90	11,110	Senegal	Maximum Estimate	2263 - Environmental and occupational health and hygiene professionals	Lower-middle Income	West	Other Health Workers
17	Gastroenterologist	236	238	247	0.15	67,343	Senegal	Maximum Estimate	2212 - Specialist medical practitioners	Lower-middle Income	West	Medical Doctors
18	General Medical Practitioner (Generalist Doctor)	9,231	10,158	11,200	6.65	1,503	Senegal	Maximum Estimate	2211 - Generalist medical practitioners	Lower-middle Income	West	Medical Doctors
19	General Surgeon	269	308	358	0.21	46,866	Senegal	Maximum Estimate	2212 - Specialist medical practitioners	Lower-middle Income	West	Medical Doctors
20	Haematologist	111	123	137	0.08	123,440	Senegal	Maximum Estimate	2212 - Specialist medical practitioners	Lower-middle Income	West	Medical Doctors
21	Health Promoter/Health Educator	300	312	329	0.20	50,805	Senegal	Maximum Estimate	2269 - Health professionals not elsewhere classified	Lower-middle Income	West	Other Health Workers
22	Infectious Diseases Specialist	23	25	28	0.02	604,252	Senegal	Maximum Estimate	2212 - Specialist medical practitioners	Lower-middle Income	West	Medical Doctors
23	Intensive Care Nurse	807	896	994	0.59	16,966	Senegal	Maximum Estimate	2221 - Nursing professionals	Lower-middle Income	West	Nursing Personnel
24	Medical Laboratory Scientist	2,601	2,860	3,186	1.90	5,264	Senegal	Maximum Estimate	3212 - Medical and pathology laboratory technicians	Lower-middle Income	West	Other Health Workers
25	Medical Laboratory Technician	3,203	3,380	3,599	2.15	4,658	Senegal	Maximum Estimate	3212 - Medical and pathology laboratory technicians	Lower-middle Income	West	Other Health Workers
26	Medical Social Worker	796	838	888	0.53	18,837	Senegal	Maximum Estimate	1344 - Social welfare managers	Lower-middle Income	West	Other Health Workers
27	Mental Health Nurse	1,466	1,651	1,903	1.14	8,792	Senegal	Maximum Estimate	2221 - Nursing professionals	Lower-middle Income	West	Nursing Personnel
28	Midwife	20,985	22,780	24,820	14.74	678	Senegal	Maximum Estimate	2222 - Midwifery professionals	Lower-middle Income	West	Midwifery Personnel
29	Nephrologist	241	275	321	0.19	52,077	Senegal	Maximum Estimate	2212 - Specialist medical practitioners	Lower-middle Income	West	Medical Doctors
30	Neuro-Surgeon	73	85	99	0.06	168,953	Senegal	Maximum Estimate	2212 - Specialist medical practitioners	Lower-middle Income	West	Medical Doctors
31	Nurse Anaesthetist	611	722	854	0.51	19,677	Senegal	Maximum Estimate	2221 - Nursing professionals	Lower-middle Income	West	Nursing Personnel
32	Nutritionist	3,128	3,296	3,458	2.05	4,872	Senegal	Maximum Estimate	2265 - Dieticians and nutritionists	Lower-middle Income	West	Other Health Workers
33	Obstetrician & Gynaecologist	1,502	1,685	1,896	1.13	8,876	Senegal	Maximum Estimate	2212 - Specialist medical practitioners	Lower-middle Income	West	Medical Doctors
34	Occupational Therapist	556	646	755	0.45	22,255	Senegal	Maximum Estimate	2269 - Health professionals not elsewhere classified	Lower-middle Income	West	Other Health Workers
35	Oncology Nurse	205	240	284	0.17	58,931	Senegal	Maximum Estimate	2221 - Nursing professionals	Lower-middle Income	West	Nursing Personnel
36	Operating Theatre Nurse	2,548	2,913	3,389	2.03	4,937	Senegal	Maximum Estimate	2221 - Nursing professionals	Lower-middle Income	West	Nursing Personnel
37	Ophthalmic Nurse	300	335	377	0.22	44,674	Senegal	Maximum Estimate	2221 - Nursing professionals	Lower-middle Income	West	Nursing Personnel
38	Ophthalmologist	111	125	142	0.08	118,378	Senegal	Maximum Estimate	2212 - Specialist medical practitioners	Lower-middle Income	West	Medical Doctors
39	Optometrist	463	521	589	0.35	28,616	Senegal	Maximum Estimate	2267 - Optometrists and ophthalmic opticians	Lower-middle Income	West	Other Health Workers
40	Orthopaedic Nurse	741	869	1,016	0.60	16,578	Senegal	Maximum Estimate	2221 - Nursing professionals	Lower-middle Income	West	Nursing Personnel
41	Orthopaedic Surgeon	870	1,007	1,168	0.69	14,390	Senegal	Maximum Estimate	2212 - Specialist medical practitioners	Lower-middle Income	West	Medical Doctors
42	Orthopaedic Technologist	797	898	1,009	0.60	16,710	Senegal	Maximum Estimate	3214 - Medical and dental prosthetic technicians	Lower-middle Income	West	Other Health Workers
43	Paediatric Nurse	2,947	3,224	3,479	2.06	4,848	Senegal	Maximum Estimate	2221 - Nursing professionals	Lower-middle Income	West	Nursing Personnel
44	Paediatric Surgeon	86	93	99	0.06	170,990	Senegal	Maximum Estimate	2212 - Specialist medical practitioners	Lower-middle Income	West	Medical Doctors
45	Paediatrician	1,023	1,159	1,317	0.78	12,769	Senegal	Maximum Estimate	2212 - Specialist medical practitioners	Lower-middle Income	West	Medical Doctors
46	Pathologist	160	181	210	0.13	79,782	Senegal	Maximum Estimate	2212 - Specialist medical practitioners	Lower-middle Income	West	Medical Doctors
47	Pharmacist	1,760	1,816	1,890	1.13	8,852	Senegal	Maximum Estimate	2262 - Pharmacists	Lower-middle Income	West	Pharmacist
48	Pharmacy Technician	2,885	3,201	3,564	2.12	4,722	Senegal	Maximum Estimate	3213 - Pharmaceutical technicians and assistants	Lower-middle Income	West	Other Health Workers
49	Physician	2,864	3,177	3,541	2.10	4,752	Senegal	Maximum Estimate	2212 - Specialist medical practitioners	Lower-middle Income	West	Medical Doctors
50	Physiotherapist	684	770	868	0.52	19,399	Senegal	Maximum Estimate	2264 - Physiotherapists	Lower-middle Income	West	Other Health Workers
51	Plastic Surgeon	186	214	246	0.15	68,538	Senegal	Maximum Estimate	2212 - Specialist medical practitioners	Lower-middle Income	West	Medical Doctors
52	Psychiatrist	1,262	1,408	1,601	0.96	10,467	Senegal	Maximum Estimate	2212 - Specialist medical practitioners	Lower-middle Income	West	Medical Doctors
53	Radiation Oncologist	73	89	108	0.06	155,502	Senegal	Maximum Estimate	2212 - Specialist medical practitioners	Lower-middle Income	West	Medical Doctors
54	Radiographer (Diagnostics and Therapy)	2,071	2,335	2,665	1.59	6,294	Senegal	Maximum Estimate	3211 - Medical imaging and therapeutic equipment technicians	Lower-middle Income	West	Other Health Workers
55	Radiologist	803	915	1,053	0.63	15,916	Senegal	Maximum Estimate	2212 - Specialist medical practitioners	Lower-middle Income	West	Medical Doctors
56	Registered General Nurse / State Certified Nurse	82,534	91,366	101,817	60.73	165	Senegal	Maximum Estimate	2221 - Nursing professionals	Lower-middle Income	West	Nursing Personnel
57	Renal Nurse	4,715	5,375	6,280	3.76	2,660	Senegal	Maximum Estimate	2221 - Nursing professionals	Lower-middle Income	West	Nursing Personnel
58	Respiratory Physician	179	207	239	0.14	70,354	Senegal	Maximum Estimate	2212 - Specialist medical practitioners	Lower-middle Income	West	Medical Doctors
59	Rheumatologist	67	76	85	0.05	197,863	Senegal	Maximum Estimate	2212 - Specialist medical practitioners	Lower-middle Income	West	Medical Doctors
60	Speech Therapist	223	247	275	0.16	61,324	Senegal	Maximum Estimate	2266 - Audiologists and speech therapists	Lower-middle Income	West	Other Health Workers
61	Urologist	48	58	71	0.04	233,737	Senegal	Maximum Estimate	2212 - Specialist medical practitioners	Lower-middle Income	West	Medical Doctors
1	Anaesthesiologist	4	4	5	0.46	21,742	Seychelles	Maximum Estimate	2212 - Specialist medical practitioners	High Income and Upper Middle Income	Southern	Medical Doctors
2	Associate Nurse/Enrolled Nurse/Nursing Assistant	205	219	238	24.97	401	Seychelles	Maximum Estimate	3221 - Nursing associate professionals	High Income and Upper Middle Income	Southern	Nursing Personnel
3	Audiologist	1	1	1	0.07	143,815	Seychelles	Maximum Estimate	2266 - Audiologists and speech therapists	High Income and Upper Middle Income	Southern	Other Health Workers
4	Cardiologist	4	5	5	0.51	19,684	Seychelles	Maximum Estimate	2212 - Specialist medical practitioners	High Income and Upper Middle Income	Southern	Medical Doctors
5	Cardiothoracic Surgeon	1	1	1	0.06	153,976	Seychelles	Maximum Estimate	2212 - Specialist medical practitioners	High Income and Upper Middle Income	Southern	Medical Doctors
6	Clinical Officer/Physician Assistant	29	32	34	3.49	2,864	Seychelles	Maximum Estimate	3256 - Medical assistants	High Income and Upper Middle Income	Southern	Other Health Workers
7	Clinical Pharmacist	8	9	9	0.96	10,458	Seychelles	Maximum Estimate	2262 - Pharmacists	High Income and Upper Middle Income	Southern	Pharmacist
8	Clinical Psychologist	9	9	9	0.93	10,753	Seychelles	Maximum Estimate	2634 - Psychologists	High Income and Upper Middle Income	Southern	Other Health Workers
9	Community health worker/Village health worker	96	98	100	10.08	992	Seychelles	Maximum Estimate	3253 - Community health workers	High Income and Upper Middle Income	Southern	Other Health Workers
10	Dental Surgery Assistant	13	14	14	1.43	6,985	Seychelles	Maximum Estimate	3251 - Dental assistants and therapists	High Income and Upper Middle Income	Southern	Other Health Workers
11	Dental Therapist	9	9	9	0.96	10,443	Seychelles	Maximum Estimate	3251 - Dental assistants and therapists	High Income and Upper Middle Income	Southern	Other Health Workers
12	Dentist	12	12	11	1.15	8,678	Seychelles	Maximum Estimate	2261 - Dentists	High Income and Upper Middle Income	Southern	Dentist

S/N	Health Professionals	2022	2026	2030	Density per 10,000 population	Required Population ratio (1 professional is to xxx population)	Name of Country	Modelling Scenario	ISCO-08 Match	Income Group Classification	Sub-Regional Grouping	SDG 3c Occupation
13	Dermatologist	1	1	1	0.06	172,266	Seychelles	Maximum Estimate	2212 - Specialist medical practitioners	High Income and Upper Middle Income	Southern	Medical Doctors
14	Endocrinologist	4	5	6	0.57	17,499	Seychelles	Maximum Estimate	2212 - Specialist medical practitioners	High Income and Upper Middle Income	Southern	Medical Doctors
15	ENT Surgeon	3	4	4	0.43	23,110	Seychelles	Maximum Estimate	2212 - Specialist medical practitioners	High Income and Upper Middle Income	Southern	Medical Doctors
16	Environmental Health Officer	7	7	8	0.77	12,967	Seychelles	Maximum Estimate	2263 - Environmental and occupational health and hygiene professionals	High Income and Upper Middle Income	Southern	Other Health Workers
17	Gastroenterologist	1	1	1	0.14	69,920	Seychelles	Maximum Estimate	2212 - Specialist medical practitioners	High Income and Upper Middle Income	Southern	Medical Doctors
18	General Medical Practitioner (Generalist Doctor)	79	85	92	9.56	1,047	Seychelles	Maximum Estimate	2211 - Generalist medical practitioners	High Income and Upper Middle Income	Southern	Medical Doctors
19	General Surgeon	4	5	5	0.53	18,878	Seychelles	Maximum Estimate	2212 - Specialist medical practitioners	High Income and Upper Middle Income	Southern	Medical Doctors
20	Haematologist	1	1	1	0.07	149,351	Seychelles	Maximum Estimate	2212 - Specialist medical practitioners	High Income and Upper Middle Income	Southern	Medical Doctors
21	Health Promoter/Health Educator	3	3	3	0.35	28,773	Seychelles	Maximum Estimate	2269 - Health professionals not elsewhere classified	High Income and Upper Middle Income	Southern	Other Health Workers
22	Infectious Diseases Specialist	0	0	0	0.01	691,342	Seychelles	Maximum Estimate	2212 - Specialist medical practitioners	High Income and Upper Middle Income	Southern	Medical Doctors
23	Intensive Care Nurse	7	8	8	0.82	12,245	Seychelles	Maximum Estimate	2221 - Nursing professionals	High Income and Upper Middle Income	Southern	Nursing Personnel
24	Medical Laboratory Scientist	36	40	45	4.59	2,181	Seychelles	Maximum Estimate	3212 - Medical and pathology laboratory technicians	High Income and Upper Middle Income	Southern	Other Health Workers
25	Medical Laboratory Technician	26	29	31	3.22	3,108	Seychelles	Maximum Estimate	3212 - Medical and pathology laboratory technicians	High Income and Upper Middle Income	Southern	Other Health Workers
26	Medical Social Worker	5	5	5	0.52	19,206	Seychelles	Maximum Estimate	1344 - Social welfare managers	High Income and Upper Middle Income	Southern	Other Health Workers
27	Mental Health Nurse	15	15	15	1.51	6,625	Seychelles	Maximum Estimate	2221 - Nursing professionals	High Income and Upper Middle Income	Southern	Nursing Personnel
28	Midwife	97	99	97	9.90	1,010	Seychelles	Maximum Estimate	2222 - Midwifery professionals	High Income and Upper Middle Income	Southern	Midwifery Personnel
29	Nephrologist	6	7	7	0.71	14,056	Seychelles	Maximum Estimate	2212 - Specialist medical practitioners	High Income and Upper Middle Income	Southern	Medical Doctors
30	Neuro-Surgeon	1	1	2	0.18	55,434	Seychelles	Maximum Estimate	2212 - Specialist medical practitioners	High Income and Upper Middle Income	Southern	Medical Doctors
31	Nurse Anaesthetist	18	20	23	2.34	4,270	Seychelles	Maximum Estimate	2221 - Nursing professionals	High Income and Upper Middle Income	Southern	Nursing Personnel
32	Nutritionist	20	21	21	2.17	4,603	Seychelles	Maximum Estimate	2265 - Dieticians and nutritionists	High Income and Upper Middle Income	Southern	Other Health Workers
33	Obstetrician & Gynaecologist	9	10	10	1.00	10,030	Seychelles	Maximum Estimate	2212 - Specialist medical practitioners	High Income and Upper Middle Income	Southern	Medical Doctors
34	Occupational Therapist	6	7	7	0.72	13,913	Seychelles	Maximum Estimate	2269 - Health professionals not elsewhere classified	High Income and Upper Middle Income	Southern	Other Health Workers
35	Oncology Nurse	7	8	8	0.84	11,937	Seychelles	Maximum Estimate	2221 - Nursing professionals	High Income and Upper Middle Income	Southern	Nursing Personnel
36	Operating Theatre Nurse	38	41	44	4.48	2,234	Seychelles	Maximum Estimate	2221 - Nursing professionals	High Income and Upper Middle Income	Southern	Nursing Personnel
37	Ophthalmic Nurse	6	7	8	0.76	13,102	Seychelles	Maximum Estimate	2221 - Nursing professionals	High Income and Upper Middle Income	Southern	Nursing Personnel
38	Ophthalmologist	2	2	3	0.28	35,206	Seychelles	Maximum Estimate	2212 - Specialist medical practitioners	High Income and Upper Middle Income	Southern	Medical Doctors
39	Optometrist	6	7	8	0.84	11,899	Seychelles	Maximum Estimate	2267 - Optometrists and ophthalmic opticians	High Income and Upper Middle Income	Southern	Other Health Workers
40	Orthopaedic Nurse	5	6	7	0.68	14,699	Seychelles	Maximum Estimate	2221 - Nursing professionals	High Income and Upper Middle Income	Southern	Nursing Personnel
41	Orthopaedic Surgeon	14	16	17	1.68	5,964	Seychelles	Maximum Estimate	2212 - Specialist medical practitioners	High Income and Upper Middle Income	Southern	Medical Doctors
42	Orthopaedic Technologist	5	5	6	0.58	17,144	Seychelles	Maximum Estimate	3214 - Medical and dental prosthetic technicians	High Income and Upper Middle Income	Southern	Other Health Workers
43	Paediatric Nurse	13	13	13	1.30	7,717	Seychelles	Maximum Estimate	2221 - Nursing professionals	High Income and Upper Middle Income	Southern	Nursing Personnel
44	Paediatric Surgeon	0	0	0	0.02	431,899	Seychelles	Maximum Estimate	2212 - Specialist medical practitioners	High Income and Upper Middle Income	Southern	Medical Doctors
45	Paediatrician	9	9	10	0.97	10,270	Seychelles	Maximum Estimate	2212 - Specialist medical practitioners	High Income and Upper Middle Income	Southern	Medical Doctors
46	Pathologist	1	2	2	0.16	62,346	Seychelles	Maximum Estimate	2212 - Specialist medical practitioners	High Income and Upper Middle Income	Southern	Medical Doctors
47	Pharmacist	22	25	27	2.72	3,675	Seychelles	Maximum Estimate	2262 - Pharmacists	High Income and Upper Middle Income	Southern	Pharmacist
48	Pharmacy Technician	25	27	29	3.02	3,316	Seychelles	Maximum Estimate	3213 - Pharmaceutical technicians and assistants	High Income and Upper Middle Income	Southern	Other Health Workers
49	Physician	31	34	36	3.71	2,698	Seychelles	Maximum Estimate	2212 - Specialist medical practitioners	High Income and Upper Middle Income	Southern	Medical Doctors
50	Physiotherapist	6	7	7	0.76	13,084	Seychelles	Maximum Estimate	2264 - Physiotherapists	High Income and Upper Middle Income	Southern	Other Health Workers
51	Plastic Surgeon	2	3	3	0.28	35,605	Seychelles	Maximum Estimate	2212 - Specialist medical practitioners	High Income and Upper Middle Income	Southern	Medical Doctors
52	Psychiatrist	10	11	11	1.07	9,311	Seychelles	Maximum Estimate	2212 - Specialist medical practitioners	High Income and Upper Middle Income	Southern	Medical Doctors
53	Radiation Oncologist	4	4	5	0.51	19,666	Seychelles	Maximum Estimate	2212 - Specialist medical practitioners	High Income and Upper Middle Income	Southern	Medical Doctors
54	Radiographer (Diagnostics and Therapy)	26	28	30	3.05	3,277	Seychelles	Maximum Estimate	3211 - Medical imaging and therapeutic equipment technicians	High Income and Upper Middle Income	Southern	Other Health Workers
55	Radiologist	9	10	11	1.12	8,949	Seychelles	Maximum Estimate	2212 - Specialist medical practitioners	High Income and Upper Middle Income	Southern	Medical Doctors
56	Registered General Nurse / State Certified Nurse	642	692	733	74.33	135	Seychelles	Maximum Estimate	2221 - Nursing professionals	High Income and Upper Middle Income	Southern	Nursing Personnel
57	Renal Nurse	129	139	145	14.59	685	Seychelles	Maximum Estimate	2221 - Nursing professionals	High Income and Upper Middle Income	Southern	Nursing Personnel
58	Respiratory Physician	1	1	1	0.15	68,351	Seychelles	Maximum Estimate	2212 - Specialist medical practitioners	High Income and Upper Middle Income	Southern	Medical Doctors
59	Rheumatologist	1	1	1	0.07	140,977	Seychelles	Maximum Estimate	2212 - Specialist medical practitioners	High Income and Upper Middle Income	Southern	Medical Doctors
60	Speech Therapist	2	2	3	0.27	37,166	Seychelles	Maximum Estimate	2266 - Audiologists and speech therapists	High Income and Upper Middle Income	Southern	Other Health Workers
61	Urologist	2	2	3	0.26	38,546	Seychelles	Maximum Estimate	2212 - Specialist medical practitioners	High Income and Upper Middle Income	Southern	Medical Doctors
1	Anaesthesiologist	144	160	177	0.22	45,394	Sierra Leone	Maximum Estimate	2212 - Specialist medical practitioners	Low Income	West	Medical Doctors
2	Associate Nurse/Enrolled Nurse/Nursing Assistant	13,821	15,025	16,355	20.35	491	Sierra Leone	Maximum Estimate	3221 - Nursing associate professionals	Low Income	West	Nursing Personnel
3	Audiologist	54	59	64	0.08	126,499	Sierra Leone	Maximum Estimate	2266 - Audiologists and speech therapists	Low Income	West	Other Health Workers
4	Cardiologist	118	131	144	0.18	56,050	Sierra Leone	Maximum Estimate	2212 - Specialist medical practitioners	Low Income	West	Medical Doctors
5	Cardiothoracic Surgeon	24	27	30	0.04	269,098	Sierra Leone	Maximum Estimate	2212 - Specialist medical practitioners	Low Income	West	Medical Doctors
6	Clinical Officer/Physician Assistant	3,504	3,680	3,879	4.84	2,066	Sierra Leone	Maximum Estimate	3256 - Medical assistants	Low Income	West	Other Health Workers
7	Clinical Pharmacist	390	426	471	0.59	17,010	Sierra Leone	Maximum Estimate	2262 - Pharmacists	Low Income	West	Pharmacist
8	Clinical Psychologist	712	780	864	1.08	9,281	Sierra Leone	Maximum Estimate	2634 - Psychologists	Low Income	West	Other Health Workers
9	Community health worker/Village health worker	15,085	15,667	16,214	20.21	495	Sierra Leone	Maximum Estimate	3253 - Community health workers	Low Income	West	Other Health Workers
10	Dental Surgery Assistant	1,039	1,145	1,270	1.58	6,336	Sierra Leone	Maximum Estimate	3251 - Dental assistants and therapists	Low Income	West	Other Health Workers
11	Dental Therapist	701	772	857	1.06	9,395	Sierra Leone	Maximum Estimate	3251 - Dental assistants and therapists	Low Income	West	Other Health Workers
12	Dentist	1,146	1,259	1,410	1.75	5,705	Sierra Leone	Maximum Estimate	2261 - Dentists	Low Income	West	Dentist
13	Dermatologist	42	46	50	0.06	160,660	Sierra Leone	Maximum Estimate	2212 - Specialist medical practitioners	Low Income	West	Medical Doctors
14	Endocrinologist	29	34	41	0.05	192,436	Sierra Leone	Maximum Estimate	2212 - Specialist medical practitioners	Low Income	West	Medical Doctors
15	ENT Surgeon	143	157	173	0.22	46,484	Sierra Leone	Maximum Estimate	2212 - Specialist medical practitioners	Low Income	West	Medical Doctors
16	Environmental Health Officer	594	646	698	0.87	11,542	Sierra Leone	Maximum Estimate	2263 - Environmental and occupational health and hygiene professionals	Low Income	West	Other Health Workers
17	Gastroenterologist	153	148	150	0.19	52,907	Sierra Leone	Maximum Estimate	2212 - Specialist medical practitioners	Low Income	West	Medical Doctors
18	General Medical Practitioner (Generalist Doctor)	5,041	5,446	5,890	7.33	1,364	Sierra Leone	Maximum Estimate	2211 - Generalist medical practitioners	Low Income	West	Medical Doctors
19	General Surgeon	129	148	174	0.22	46,113	Sierra Leone	Maximum Estimate	2212 - Specialist medical practitioners	Low Income	West	Medical Doctors
20	Haematologist	53	58	63	0.08	128,786	Sierra Leone	Maximum Estimate	2212 - Specialist medical practitioners	Low Income	West	Medical Doctors
21	Health Promoter/Health Educator	237	246	257	0.32	31,155	Sierra Leone	Maximum Estimate	2269 - Health professionals not elsewhere classified	Low Income	West	Other Health Workers
22	Infectious Diseases Specialist	11	12	13	0.02	611,771	Sierra Leone	Maximum Estimate	2212 - Specialist medical practitioners	Low Income	West	Medical Doctors
23	Intensive Care Nurse	374	407	442	0.55	18,194	Sierra Leone	Maximum Estimate	2221 - Nursing professionals	Low Income	West	Nursing Personnel
24	Medical Laboratory Scientist	1,598	1,713	1,862	2.33	4,298	Sierra Leone	Maximum Estimate	3212 - Medical and pathology laboratory technicians	Low Income	West	Other Health Workers
25	Medical Laboratory Technician	2,896	3,001	3,115	3.89	2,572	Sierra Leone	Maximum Estimate	3212 - Medical and pathology laboratory technicians	Low Income	West	Other Health Workers
26	Medical Social Worker	459	469	486	0.61	16,401	Sierra Leone	Maximum Estimate	1344 - Social welfare managers	Low Income	West	Other Health Workers
27	Mental Health Nurse	850	940	1,073	1.34	7,454	Sierra Leone	Maximum Estimate	2221 - Nursing professionals	Low Income	West	Nursing Personnel
28	Midwife	9,468	10,187	11,001	13.67	732	Sierra Leone	Maximum Estimate	2222 - Midwifery professionals	Low Income	West	Midwifery Personnel
29	Nephrologist	122	138	161	0.20	49,426	Sierra Leone	Maximum Estimate	2212 - Specialist medical practitioners	Low Income	West	Medical Doctors
30	Neuro-Surgeon	40	45	50	0.06	159,702	Sierra Leone	Maximum Estimate	2212 - Specialist medical practitioners	Low Income	West	Medical Doctors
31	Nurse Anaesthetist	271	310	353	0.44	22,775	Sierra Leone	Maximum Estimate	2221 - Nursing professionals	Low Income	West	Nursing Personnel
32	Nutritionist	1,635	1,703	1,763	2.19	4,557	Sierra Leone	Maximum Estimate	2265 - Dieticians and nutritionists	Low Income	West	Other Health Workers
33	Obstetrician & Gynaecologist	739	816	905	1.13	8,883	Sierra Leone	Maximum Estimate	2212 - Specialist medical practitioners	Low Income	West	Medical Doctors
34	Occupational Therapist	280	313	352	0.44	22,793	Sierra Leone	Maximum Estimate	2269 - Health professionals not elsewhere classified	Low Income	West	Other Health Workers
35	Oncology Nurse	95	109	126	0.16	63,432	Sierra Leone	Maximum Estimate	2221 - Nursing professionals	Low Income	West	Nursing Personnel

S/N	Health Professionals	2022	2026	2030	Density per 10,000 population	Required Population ratio (1 professional is to xxx population)	Name of Country	Modelling Scenario	ISCO-08 Match	Income Group Classification	Sub-Regional Grouping	SDG 3c Occupation
36	Operating Theatre Nurse	1,269	1,405	1,591	1.99	5,029	Sierra Leone	Maximum Estimate	2221 - Nursing professionals	Low Income	West	Nursing Personnel
37	Ophthalmic Nurse	122	135	149	0.19	53,784	Sierra Leone	Maximum Estimate	2221 - Nursing professionals	Low Income	West	Nursing Personnel
38	Ophthalmologist	41	46	52	0.06	154,181	Sierra Leone	Maximum Estimate	2212 - Specialist medical practitioners	Low Income	West	Medical Doctors
39	Optometrist	178	196	217	0.27	37,111	Sierra Leone	Maximum Estimate	2267 - Optometrists and ophthalmic opticians	Low Income	West	Other Health Workers
40	Orthopaedic Nurse	335	381	431	0.54	18,689	Sierra Leone	Maximum Estimate	2221 - Nursing professionals	Low Income	West	Nursing Personnel
41	Orthopaedic Surgeon	400	462	537	0.67	14,928	Sierra Leone	Maximum Estimate	2212 - Specialist medical practitioners	Low Income	West	Medical Doctors
42	Orthopaedic Technologist	369	408	449	0.56	17,927	Sierra Leone	Maximum Estimate	3214 - Medical and dental prosthetic technicians	Low Income	West	Other Health Workers
43	Paediatric Nurse	1,428	1,537	1,631	2.02	4,940	Sierra Leone	Maximum Estimate	2221 - Nursing professionals	Low Income	West	Nursing Personnel
44	Paediatric Surgeon	41	44	45	0.06	178,446	Sierra Leone	Maximum Estimate	2212 - Specialist medical practitioners	Low Income	West	Medical Doctors
45	Paediatrician	567	622	683	0.85	11,758	Sierra Leone	Maximum Estimate	2212 - Specialist medical practitioners	Low Income	West	Medical Doctors
46	Pathologist	85	94	108	0.14	73,799	Sierra Leone	Maximum Estimate	2212 - Specialist medical practitioners	Low Income	West	Medical Doctors
47	Pharmacist	1,340	1,403	1,472	1.84	5,442	Sierra Leone	Maximum Estimate	2262 - Pharmacists	Low Income	West	Pharmacist
48	Pharmacy Technician	1,480	1,611	1,758	2.19	4,572	Sierra Leone	Maximum Estimate	3213 - Pharmaceutical technicians and assistants	Low Income	West	Other Health Workers
49	Physician	1,437	1,567	1,716	2.14	4,678	Sierra Leone	Maximum Estimate	2212 - Specialist medical practitioners	Low Income	West	Medical Doctors
50	Physiotherapist	320	353	389	0.48	20,677	Sierra Leone	Maximum Estimate	2264 - Physiotherapists	Low Income	West	Other Health Workers
51	Plastic Surgeon	86	96	107	0.13	75,242	Sierra Leone	Maximum Estimate	2212 - Specialist medical practitioners	Low Income	West	Medical Doctors
52	Psychiatrist	668	731	819	1.02	9,778	Sierra Leone	Maximum Estimate	2212 - Specialist medical practitioners	Low Income	West	Medical Doctors
53	Radiation Oncologist	30	34	40	0.05	199,679	Sierra Leone	Maximum Estimate	2212 - Specialist medical practitioners	Low Income	West	Medical Doctors
54	Radiographer (Diagnostics and Therapy)	1,003	1,114	1,258	1.57	6,367	Sierra Leone	Maximum Estimate	3211 - Medical imaging and therapeutic equipment technicians	Low Income	West	Other Health Workers
55	Radiologist	379	425	482	0.60	16,618	Sierra Leone	Maximum Estimate	2212 - Specialist medical practitioners	Low Income	West	Medical Doctors
56	Registered General Nurse / State Certified Nurse	28,461	30,469	33,020	41.28	242	Sierra Leone	Maximum Estimate	2221 - Nursing professionals	Low Income	West	Nursing Personnel
57	Renal Nurse	2,348	2,658	3,115	3.90	2,562	Sierra Leone	Maximum Estimate	2221 - Nursing professionals	Low Income	West	Nursing Personnel
58	Respiratory Physician	93	103	113	0.14	71,024	Sierra Leone	Maximum Estimate	2212 - Specialist medical practitioners	Low Income	West	Medical Doctors
59	Rheumatologist	30	33	36	0.05	220,910	Sierra Leone	Maximum Estimate	2212 - Specialist medical practitioners	Low Income	West	Medical Doctors
60	Speech Therapist	107	116	126	0.16	63,900	Sierra Leone	Maximum Estimate	2266 - Audiologists and speech therapists	Low Income	West	Other Health Workers
61	Urologist	22	27	32	0.04	245,280	Sierra Leone	Maximum Estimate	2212 - Specialist medical practitioners	Low Income	West	Medical Doctors
1	Anaesthesiologist	1,425	1,515	1,621	0.27	36,778	South Africa	Maximum Estimate	2212 - Specialist medical practitioners	High Income and Upper Middle Income	Southern	Medical Doctors
2	Associate Nurse/Enrolled Nurse/Nursing Assistant	132,823	142,831	154,484	25.98	385	South Africa	Maximum Estimate	3221 - Nursing associate professionals	High Income and Upper Middle Income	Southern	Nursing Personnel
3	Audiologist	535	573	614	0.10	97,309	South Africa	Maximum Estimate	2266 - Audiologists and speech therapists	High Income and Upper Middle Income	Southern	Other Health Workers
4	Cardiologist	1,569	1,687	1,817	0.30	32,883	South Africa	Maximum Estimate	2212 - Specialist medical practitioners	High Income and Upper Middle Income	Southern	Medical Doctors
5	Cardiothoracic Surgeon	196	200	204	0.03	292,236	South Africa	Maximum Estimate	2212 - Specialist medical practitioners	High Income and Upper Middle Income	Southern	Medical Doctors
6	Clinical Officer/Physician Assistant	24,077	25,960	28,453	4.84	2,065	South Africa	Maximum Estimate	3256 - Medical assistants	High Income and Upper Middle Income	Southern	Other Health Workers
7	Clinical Pharmacist	3,409	3,703	4,061	0.68	14,621	South Africa	Maximum Estimate	2262 - Pharmacists	High Income and Upper Middle Income	Southern	Pharmacist
8	Clinical Psychologist	4,544	4,765	5,011	0.84	11,891	South Africa	Maximum Estimate	2634 - Psychologists	High Income and Upper Middle Income	Southern	Other Health Workers
9	Community health worker/Village health worker	68,389	73,071	80,090	13.83	723	South Africa	Maximum Estimate	3253 - Community health workers	High Income and Upper Middle Income	Southern	Other Health Workers
10	Dental Surgery Assistant	10,884	11,549	12,236	2.05	4,883	South Africa	Maximum Estimate	3251 - Dental assistants and therapists	High Income and Upper Middle Income	Southern	Other Health Workers
11	Dental Therapist	7,271	7,713	8,169	1.37	7,314	South Africa	Maximum Estimate	3251 - Dental assistants and therapists	High Income and Upper Middle Income	Southern	Other Health Workers
12	Dentist	8,497	8,907	9,326	1.56	6,401	South Africa	Maximum Estimate	2261 - Dentists	High Income and Upper Middle Income	Southern	Dentist
13	Dermatologist	362	383	404	0.07	147,793	South Africa	Maximum Estimate	2212 - Specialist medical practitioners	High Income and Upper Middle Income	Southern	Medical Doctors
14	Endocrinologist	924	1,038	1,172	0.20	50,699	South Africa	Maximum Estimate	2212 - Specialist medical practitioners	High Income and Upper Middle Income	Southern	Medical Doctors
15	ENT Surgeon	2,065	2,242	2,480	0.42	24,043	South Africa	Maximum Estimate	2212 - Specialist medical practitioners	High Income and Upper Middle Income	Southern	Medical Doctors
16	Environmental Health Officer	4,419	4,592	4,768	0.80	12,519	South Africa	Maximum Estimate	2263 - Environmental and occupational health and hygiene professionals	High Income and Upper Middle Income	Southern	Other Health Workers
17	Gastroenterologist	751	747	746	0.13	79,201	South Africa	Maximum Estimate	2212 - Specialist medical practitioners	High Income and Upper Middle Income	Southern	Medical Doctors
18	General Medical Practitioner (Generalist Doctor)	48,396	51,845	55,862	9.41	1,063	South Africa	Maximum Estimate	2211 - Generalist medical practitioners	High Income and Upper Middle Income	Southern	Medical Doctors
19	General Surgeon	1,517	1,627	1,759	0.30	33,865	South Africa	Maximum Estimate	2212 - Specialist medical practitioners	High Income and Upper Middle Income	Southern	Medical Doctors
20	Haematologist	178	183	188	0.03	316,032	South Africa	Maximum Estimate	2212 - Specialist medical practitioners	High Income and Upper Middle Income	Southern	Medical Doctors
21	Health Promoter/Health Educator	2,224	2,354	2,525	0.43	23,332	South Africa	Maximum Estimate	2269 - Health professionals not elsewhere classified	High Income and Upper Middle Income	Southern	Other Health Workers
22	Infectious Diseases Specialist	143	149	156	0.03	381,415	South Africa	Maximum Estimate	2212 - Specialist medical practitioners	High Income and Upper Middle Income	Southern	Medical Doctors
23	Intensive Care Nurse	2,874	2,969	3,077	0.52	19,368	South Africa	Maximum Estimate	2221 - Nursing professionals	High Income and Upper Middle Income	Southern	Nursing Personnel
24	Medical Laboratory Scientist	20,258	22,170	24,482	4.13	2,421	South Africa	Maximum Estimate	3212 - Medical and pathology laboratory technicians	High Income and Upper Middle Income	Southern	Other Health Workers
25	Medical Laboratory Technician	16,646	18,600	21,326	3.68	2,720	South Africa	Maximum Estimate	3212 - Medical and pathology laboratory technicians	High Income and Upper Middle Income	Southern	Other Health Workers
26	Medical Social Worker	2,359	2,382	2,397	0.40	24,764	South Africa	Maximum Estimate	1344 - Social welfare managers	High Income and Upper Middle Income	Southern	Other Health Workers
27	Mental Health Nurse	8,278	8,706	9,218	1.55	6,448	South Africa	Maximum Estimate	2221 - Nursing professionals	High Income and Upper Middle Income	Southern	Nursing Personnel
28	Midwife	60,891	63,261	65,437	10.97	912	South Africa	Maximum Estimate	2222 - Midwifery professionals	High Income and Upper Middle Income	Southern	Midwifery Personnel
29	Nephrologist	2,015	2,259	2,543	0.43	23,373	South Africa	Maximum Estimate	2212 - Specialist medical practitioners	High Income and Upper Middle Income	Southern	Medical Doctors
30	Neuro-Surgeon	497	531	582	0.10	102,116	South Africa	Maximum Estimate	2212 - Specialist medical practitioners	High Income and Upper Middle Income	Southern	Medical Doctors
31	Nurse Anaesthetist	4,103	4,519	4,984	0.83	11,980	South Africa	Maximum Estimate	2221 - Nursing professionals	High Income and Upper Middle Income	Southern	Nursing Personnel
32	Nutritionist	7,201	7,234	7,248	1.22	8,204	South Africa	Maximum Estimate	2265 - Dieticians and nutritionists	High Income and Upper Middle Income	Southern	Other Health Workers
33	Obstetrician & Gynaecologist	7,810	8,210	8,642	1.45	6,902	South Africa	Maximum Estimate	2212 - Specialist medical practitioners	High Income and Upper Middle Income	Southern	Medical Doctors
34	Occupational Therapist	2,414	2,559	2,755	0.46	21,571	South Africa	Maximum Estimate	2269 - Health professionals not elsewhere classified	High Income and Upper Middle Income	Southern	Other Health Workers
35	Oncology Nurse	1,657	1,855	2,084	0.35	28,568	South Africa	Maximum Estimate	2221 - Nursing professionals	High Income and Upper Middle Income	Southern	Nursing Personnel
36	Operating Theatre Nurse	16,644	17,921	19,518	3.28	3,047	South Africa	Maximum Estimate	2221 - Nursing professionals	High Income and Upper Middle Income	Southern	Nursing Personnel
37	Ophthalmic Nurse	1,769	1,926	2,104	0.35	28,331	South Africa	Maximum Estimate	2221 - Nursing professionals	High Income and Upper Middle Income	Southern	Nursing Personnel
38	Ophthalmologist	921	1,011	1,114	0.19	53,514	South Africa	Maximum Estimate	2212 - Specialist medical practitioners	High Income and Upper Middle Income	Southern	Medical Doctors
39	Optometrist	4,631	5,096	5,609	0.94	10,653	South Africa	Maximum Estimate	2267 - Optometrists and ophthalmic opticians	High Income and Upper Middle Income	Southern	Other Health Workers
40	Orthopaedic Nurse	2,304	2,372	2,447	0.41	24,359	South Africa	Maximum Estimate	2221 - Nursing professionals	High Income and Upper Middle Income	Southern	Nursing Personnel
41	Orthopaedic Surgeon	5,241	5,794	6,421	1.08	9,298	South Africa	Maximum Estimate	2212 - Specialist medical practitioners	High Income and Upper Middle Income	Southern	Medical Doctors
42	Orthopaedic Technologist	2,757	2,852	2,957	0.50	20,163	South Africa	Maximum Estimate	3214 - Medical and dental prosthetic technicians	High Income and Upper Middle Income	Southern	Other Health Workers
43	Paediatric Nurse	7,691	8,426	9,263	1.57	6,386	South Africa	Maximum Estimate	2221 - Nursing professionals	High Income and Upper Middle Income	Southern	Nursing Personnel
44	Paediatric Surgeon	224	231	237	0.04	251,702	South Africa	Maximum Estimate	2212 - Specialist medical practitioners	High Income and Upper Middle Income	Southern	Medical Doctors
45	Paediatrician	6,546	8,061	10,025	1.70	5,866	South Africa	Maximum Estimate	2212 - Specialist medical practitioners	High Income and Upper Middle Income	Southern	Medical Doctors
46	Pathologist	1,426	1,492	1,568	0.26	37,885	South Africa	Maximum Estimate	2212 - Specialist medical practitioners	High Income and Upper Middle Income	Southern	Medical Doctors
47	Pharmacist	15,844	16,635	17,536	2.95	3,394	South Africa	Maximum Estimate	2262 - Pharmacists	High Income and Upper Middle Income	Southern	Pharmacist
48	Pharmacy Technician	13,712	14,457	15,289	2.57	3,897	South Africa	Maximum Estimate	3213 - Pharmaceutical technicians and assistants	High Income and Upper Middle Income	Southern	Other Health Workers
49	Physician	12,527	13,699	15,104	2.55	3,929	South Africa	Maximum Estimate	2212 - Specialist medical practitioners	High Income and Upper Middle Income	Southern	Medical Doctors
50	Physiotherapist	2,891	3,042	3,256	0.55	18,272	South Africa	Maximum Estimate	2264 - Physiotherapists	High Income and Upper Middle Income	Southern	Other Health Workers
51	Plastic Surgeon	844	849	855	0.14	69,611	South Africa	Maximum Estimate	2212 - Specialist medical practitioners	High Income and Upper Middle Income	Southern	Medical Doctors
52	Psychiatrist	5,696	5,984	6,332	1.06	9,392	South Africa	Maximum Estimate	2212 - Specialist medical practitioners	High Income and Upper Middle Income	Southern	Medical Doctors
53	Radiation Oncologist	605	677	763	0.13	78,089	South Africa	Maximum Estimate	2212 - Specialist medical practitioners	High Income and Upper Middle Income	Southern	Medical Doctors
54	Radiographer (Diagnostics and Therapy)	11,839	12,815	13,931	2.34	4,275	South Africa	Maximum Estimate	3211 - Medical imaging and therapeutic equipment technicians	High Income and Upper Middle Income	Southern	Other Health Workers
55	Radiologist	4,300	4,626	4,995	0.84	11,941	South Africa	Maximum Estimate	2212 - Specialist medical practitioners	High Income and Upper Middle Income	Southern	Medical Doctors
56	Registered General Nurse / State Certified Nurse	293,219	317,107	344,905	58.00	172	South Africa	Maximum Estimate	2221 - Nursing professionals	High Income and Upper Middle Income	Southern	Nursing Personnel
57	Renal Nurse	40,561	45,482	51,251	8.62	1,160	South Africa	Maximum Estimate	2221 - Nursing professionals	High Income and Upper Middle Income	Southern	Nursing Personnel
58	Respiratory Physician	1,602	2,068	2,680	0.46	21,873	South Africa	Maximum Estimate	2212 - Specialist medical practitioners	High Income and Upper Middle Income	Southern	Medical Doctors

S/N	Health Professionals	2022	2026	2030	Density per 10,000 population	Required Population ratio (1 professional is to xxx population)	Name of Country	Modelling Scenario	ISCO-08 Match	Income Group Classification	Sub-Regional Grouping	SDG 3c Occupation
59	Rheumatologist	351	378	409	0.07	145,783	South Africa	Maximum Estimate	2212 - Specialist medical practitioners	High Income and Upper Middle Income	Southern	Medical Doctors
60	Speech Therapist	1,000	1,049	1,140	0.19	52,090	South Africa	Maximum Estimate	2266 - Audiologists and speech therapists	High Income and Upper Middle Income	Southern	Other Health Workers
61	Urologist	369	418	476	0.08	124,644	South Africa	Maximum Estimate	2212 - Specialist medical practitioners	High Income and Upper Middle Income	Southern	Medical Doctors
1	Anaesthesiologist	181	202	227	0.20	49,410	South Sudan	Maximum Estimate	2212 - Specialist medical practitioners	Low Income	East	Medical Doctors
2	Associate Nurse/Enrolled Nurse/Nursing Assistant	19,696	20,895	22,456	20.06	498	South Sudan	Maximum Estimate	3221 - Nursing associate professionals	Low Income	East	Nursing Personnel
3	Audiologist	71	77	85	0.08	132,355	South Sudan	Maximum Estimate	2266 - Audiologists and speech therapists	Low Income	East	Other Health Workers
4	Cardiologist	168	196	231	0.21	48,411	South Sudan	Maximum Estimate	2212 - Specialist medical practitioners	Low Income	East	Medical Doctors
5	Cardiothoracic Surgeon	38	42	47	0.04	236,709	South Sudan	Maximum Estimate	2212 - Specialist medical practitioners	Low Income	East	Medical Doctors
6	Clinical Officer/Physician Assistant	3,917	4,000	4,153	3.72	2,686	South Sudan	Maximum Estimate	3256 - Medical assistants	Low Income	East	Other Health Workers
7	Clinical Pharmacist	503	544	601	0.54	18,607	South Sudan	Maximum Estimate	2262 - Pharmacists	Low Income	East	Pharmacist
8	Clinical Psychologist	1,042	1,114	1,211	1.08	9,251	South Sudan	Maximum Estimate	2634 - Psychologists	Low Income	East	Other Health Workers
9	Community health worker/Village health worker	21,090	21,435	21,983	19.67	508	South Sudan	Maximum Estimate	3253 - Community health workers	Low Income	East	Other Health Workers
10	Dental Surgery Assistant	1,627	1,766	1,944	1.73	5,771	South Sudan	Maximum Estimate	3251 - Dental assistants and therapists	Low Income	East	Other Health Workers
11	Dental Therapist	1,092	1,185	1,304	1.16	8,603	South Sudan	Maximum Estimate	3251 - Dental assistants and therapists	Low Income	East	Other Health Workers
12	Dentist	1,407	1,521	1,687	1.51	6,642	South Sudan	Maximum Estimate	2261 - Dentists	Low Income	East	Dentist
13	Dermatologist	52	56	61	0.05	183,180	South Sudan	Maximum Estimate	2212 - Specialist medical practitioners	Low Income	East	Medical Doctors
14	Endocrinologist	51	60	74	0.07	149,413	South Sudan	Maximum Estimate	2212 - Specialist medical practitioners	Low Income	East	Medical Doctors
15	ENT Surgeon	212	236	264	0.24	42,429	South Sudan	Maximum Estimate	2212 - Specialist medical practitioners	Low Income	East	Medical Doctors
16	Environmental Health Officer	834	891	958	0.85	11,723	South Sudan	Maximum Estimate	2263 - Environmental and occupational health and hygiene professionals	Low Income	East	Other Health Workers
17	Gastroenterologist	184	186	195	0.18	57,059	South Sudan	Maximum Estimate	2212 - Specialist medical practitioners	Low Income	East	Medical Doctors
18	General Medical Practitioner (Generalist Doctor)	6,743	7,149	7,677	6.86	1,458	South Sudan	Maximum Estimate	2211 - Generalist medical practitioners	Low Income	East	Medical Doctors
19	General Surgeon	122	136	155	0.14	72,188	South Sudan	Maximum Estimate	2212 - Specialist medical practitioners	Low Income	East	Medical Doctors
20	Haematologist	63	68	74	0.07	151,828	South Sudan	Maximum Estimate	2212 - Specialist medical practitioners	Low Income	East	Medical Doctors
21	Health Promoter/Health Educator	360	364	373	0.33	29,896	South Sudan	Maximum Estimate	2269 - Health professionals not elsewhere classified	Low Income	East	Other Health Workers
22	Infectious Diseases Specialist	16	17	18	0.02	615,272	South Sudan	Maximum Estimate	2212 - Specialist medical practitioners	Low Income	East	Medical Doctors
23	Intensive Care Nurse	653	687	729	0.65	15,376	South Sudan	Maximum Estimate	2221 - Nursing professionals	Low Income	East	Nursing Personnel
24	Medical Laboratory Scientist	2,168	2,328	2,551	2.28	4,379	South Sudan	Maximum Estimate	3212 - Medical and pathology laboratory technicians	Low Income	East	Other Health Workers
25	Medical Laboratory Technician	3,029	3,116	3,253	2.91	3,432	South Sudan	Maximum Estimate	3212 - Medical and pathology laboratory technicians	Low Income	East	Other Health Workers
26	Medical Social Worker	711	714	728	0.65	15,342	South Sudan	Maximum Estimate	1344 - Social welfare managers	Low Income	East	Other Health Workers
27	Mental Health Nurse	1,393	1,511	1,694	1.52	6,592	South Sudan	Maximum Estimate	2221 - Nursing professionals	Low Income	East	Nursing Personnel
28	Midwife	11,121	11,828	12,771	11.39	878	South Sudan	Maximum Estimate	2222 - Midwifery professionals	Low Income	East	Midwifery Personnel
29	Nephrologist	134	150	174	0.16	64,020	South Sudan	Maximum Estimate	2212 - Specialist medical practitioners	Low Income	East	Medical Doctors
30	Neuro-Surgeon	47	53	61	0.05	182,688	South Sudan	Maximum Estimate	2212 - Specialist medical practitioners	Low Income	East	Medical Doctors
31	Nurse Anaesthetist	362	411	472	0.42	23,745	South Sudan	Maximum Estimate	2221 - Nursing professionals	Low Income	East	Nursing Personnel
32	Nutritionist	3,115	3,109	3,124	2.80	3,574	South Sudan	Maximum Estimate	2265 - Dieticians and nutritionists	Low Income	East	Other Health Workers
33	Obstetrician & Gynaecologist	1,019	1,099	1,200	1.07	9,340	South Sudan	Maximum Estimate	2212 - Specialist medical practitioners	Low Income	East	Medical Doctors
34	Occupational Therapist	315	354	404	0.36	27,675	South Sudan	Maximum Estimate	2269 - Health professionals not elsewhere classified	Low Income	East	Other Health Workers
35	Oncology Nurse	118	134	155	0.14	72,230	South Sudan	Maximum Estimate	2221 - Nursing professionals	Low Income	East	Nursing Personnel
36	Operating Theatre Nurse	1,677	1,874	2,155	1.93	5,176	South Sudan	Maximum Estimate	2221 - Nursing professionals	Low Income	East	Nursing Personnel
37	Ophthalmic Nurse	172	188	208	0.19	53,783	South Sudan	Maximum Estimate	2221 - Nursing professionals	Low Income	East	Nursing Personnel
38	Ophthalmologist	70	80	93	0.08	120,037	South Sudan	Maximum Estimate	2212 - Specialist medical practitioners	Low Income	East	Medical Doctors
39	Optometrist	311	365	432	0.39	25,854	South Sudan	Maximum Estimate	2267 - Optometrists and ophthalmic opticians	Low Income	East	Other Health Workers
40	Orthopaedic Nurse	353	397	449	0.40	24,961	South Sudan	Maximum Estimate	2221 - Nursing professionals	Low Income	East	Nursing Personnel
41	Orthopaedic Surgeon	427	473	533	0.48	21,013	South Sudan	Maximum Estimate	2212 - Specialist medical practitioners	Low Income	East	Medical Doctors
42	Orthopaedic Technologist	501	545	597	0.53	18,811	South Sudan	Maximum Estimate	3214 - Medical and dental prosthetic technicians	Low Income	East	Other Health Workers
43	Paediatric Nurse	2,175	2,287	2,409	2.15	4,659	South Sudan	Maximum Estimate	2221 - Nursing professionals	Low Income	East	Nursing Personnel
44	Paediatric Surgeon	62	66	70	0.06	160,360	South Sudan	Maximum Estimate	2212 - Specialist medical practitioners	Low Income	East	Medical Doctors
45	Paediatrician	870	947	1,045	0.93	10,728	South Sudan	Maximum Estimate	2212 - Specialist medical practitioners	Low Income	East	Medical Doctors
46	Pathologist	136	148	168	0.15	66,565	South Sudan	Maximum Estimate	2212 - Specialist medical practitioners	Low Income	East	Medical Doctors
47	Pharmacist	1,825	1,900	2,002	1.79	5,588	South Sudan	Maximum Estimate	2262 - Pharmacists	Low Income	East	Pharmacist
48	Pharmacy Technician	1,882	2,017	2,187	1.95	5,122	South Sudan	Maximum Estimate	3213 - Pharmaceutical technicians and assistants	Low Income	East	Other Health Workers
49	Physician	1,655	1,784	1,956	1.75	5,718	South Sudan	Maximum Estimate	2212 - Specialist medical practitioners	Low Income	East	Medical Doctors
50	Physiotherapist	441	481	531	0.47	21,112	South Sudan	Maximum Estimate	2264 - Physiotherapists	Low Income	East	Other Health Workers
51	Plastic Surgeon	274	305	340	0.30	32,985	South Sudan	Maximum Estimate	2212 - Specialist medical practitioners	Low Income	East	Medical Doctors
52	Psychiatrist	1,018	1,084	1,189	1.06	9,391	South Sudan	Maximum Estimate	2212 - Specialist medical practitioners	Low Income	East	Medical Doctors
53	Radiation Oncologist	46	53	62	0.06	179,422	South Sudan	Maximum Estimate	2212 - Specialist medical practitioners	Low Income	East	Medical Doctors
54	Radiographer (Diagnostics and Therapy)	1,118	1,230	1,387	1.24	8,061	South Sudan	Maximum Estimate	3211 - Medical imaging and therapeutic equipment technicians	Low Income	East	Other Health Workers
55	Radiologist	425	466	520	0.46	21,529	South Sudan	Maximum Estimate	2212 - Specialist medical practitioners	Low Income	East	Medical Doctors
56	Registered General Nurse / State Certified Nurse	55,103	56,190	58,516	52.60	190	South Sudan	Maximum Estimate	2221 - Nursing professionals	Low Income	East	Nursing Personnel
57	Renal Nurse	2,639	2,957	3,430	3.08	3,249	South Sudan	Maximum Estimate	2221 - Nursing professionals	Low Income	East	Nursing Personnel
58	Respiratory Physician	194	212	234	0.21	48,021	South Sudan	Maximum Estimate	2212 - Specialist medical practitioners	Low Income	East	Medical Doctors
59	Rheumatologist	47	52	57	0.05	195,309	South Sudan	Maximum Estimate	2212 - Specialist medical practitioners	Low Income	East	Medical Doctors
60	Speech Therapist	157	169	184	0.16	60,982	South Sudan	Maximum Estimate	2266 - Audiologists and speech therapists	Low Income	East	Other Health Workers
61	Urologist	22	26	30	0.03	364,575	South Sudan	Maximum Estimate	2212 - Specialist medical practitioners	Low Income	East	Medical Doctors
1	Anaesthesiologist	167	191	218	0.26	38,178	Togo	Maximum Estimate	2212 - Specialist medical practitioners	Low Income	West	Medical Doctors
2	Associate Nurse/Enrolled Nurse/Nursing Assistant	14,700	16,176	17,862	21.46	466	Togo	Maximum Estimate	3221 - Nursing associate professionals	Low Income	West	Nursing Personnel
3	Audiologist	58	65	72	0.09	116,291	Togo	Maximum Estimate	2266 - Audiologists and speech therapists	Low Income	West	Other Health Workers
4	Cardiologist	131	152	176	0.21	47,306	Togo	Maximum Estimate	2212 - Specialist medical practitioners	Low Income	West	Medical Doctors
5	Cardiothoracic Surgeon	31	35	40	0.05	208,134	Togo	Maximum Estimate	2212 - Specialist medical practitioners	Low Income	West	Medical Doctors
6	Clinical Officer/Physician Assistant	2,119	2,268	2,458	2.97	3,370	Togo	Maximum Estimate	3256 - Medical assistants	Low Income	West	Other Health Workers
7	Clinical Pharmacist	395	442	500	0.60	16,591	Togo	Maximum Estimate	2262 - Pharmacists	Low Income	West	Pharmacist
8	Clinical Psychologist	673	751	845	1.02	9,846	Togo	Maximum Estimate	2634 - Psychologists	Low Income	West	Other Health Workers
9	Community health worker/Village health worker	14,388	15,539	16,785	20.17	496	Togo	Maximum Estimate	3253 - Community health workers	Low Income	West	Other Health Workers
10	Dental Surgery Assistant	1,465	1,643	1,847	2.21	4,518	Togo	Maximum Estimate	3251 - Dental assistants and therapists	Low Income	West	Other Health Workers
11	Dental Therapist	980	1,099	1,235	1.48	6,757	Togo	Maximum Estimate	3251 - Dental assistants and therapists	Low Income	West	Other Health Workers
12	Dentist	1,170	1,298	1,459	1.75	5,723	Togo	Maximum Estimate	2261 - Dentists	Low Income	West	Dentist
13	Dermatologist	44	49	54	0.06	154,549	Togo	Maximum Estimate	2212 - Specialist medical practitioners	Low Income	West	Medical Doctors
14	Endocrinologist	37	45	57	0.07	143,049	Togo	Maximum Estimate	2212 - Specialist medical practitioners	Low Income	West	Medical Doctors
15	ENT Surgeon	161	182	207	0.25	40,286	Togo	Maximum Estimate	2212 - Specialist medical practitioners	Low Income	West	Medical Doctors
16	Environmental Health Officer	617	675	735	0.88	11,348	Togo	Maximum Estimate	2263 - Environmental and occupational health and hygiene professionals	Low Income	West	Other Health Workers
17	Gastroenterologist	117	119	126	0.15	65,509	Togo	Maximum Estimate	2212 - Specialist medical practitioners	Low Income	West	Medical Doctors
18	General Medical Practitioner (Generalist Doctor)	5,081	5,583	6,151	7.39	1,353	Togo	Maximum Estimate	2211 - Generalist medical practitioners	Low Income	West	Medical Doctors
19	General Surgeon	144	169	201	0.24	41,372	Togo	Maximum Estimate	2212 - Specialist medical practitioners	Low Income	West	Medical Doctors
20	Haematologist	50	55	60	0.07	137,904	Togo	Maximum Estimate	2212 - Specialist medical practitioners	Low Income	West	Medical Doctors

S/N	Health Professionals	2022	2026	2030	Density per 10,000 population	Required Population ratio (1 professional is to xxx population)	Name of Country	Modelling Scenario	ISCO-08 Match	Income Group Classification	Sub-Regional Grouping	SDG 3c Occupation
21	Health Promoter/Health Educator	206	218	234	0.28	35,431	Togo	Maximum Estimate	2269 - Health professionals not elsewhere classified	Low Income	West	Other Health Workers
22	Infectious Diseases Specialist	17	19	20	0.02	408,386	Togo	Maximum Estimate	2212 - Specialist medical practitioners	Low Income	West	Medical Doctors
23	Intensive Care Nurse	413	451	492	0.59	16,923	Togo	Maximum Estimate	2221 - Nursing professionals	Low Income	West	Nursing Personnel
24	Medical Laboratory Scientist	1,591	1,768	1,991	2.40	4,167	Togo	Maximum Estimate	3212 - Medical and pathology laboratory technicians	Low Income	West	Other Health Workers
25	Medical Laboratory Technician	2,678	2,967	3,297	3.96	2,524	Togo	Maximum Estimate	3212 - Medical and pathology laboratory technicians	Low Income	West	Other Health Workers
26	Medical Social Worker	376	393	418	0.51	19,730	Togo	Maximum Estimate	1344 - Social welfare managers	Low Income	West	Other Health Workers
27	Mental Health Nurse	862	960	1,098	1.32	7,557	Togo	Maximum Estimate	2221 - Nursing professionals	Low Income	West	Nursing Personnel
28	Midwife	9,274	10,035	10,896	13.06	766	Togo	Maximum Estimate	2222 - Midwifery professionals	Low Income	West	Midwifery Personnel
29	Nephrologist	136	157	187	0.23	44,195	Togo	Maximum Estimate	2212 - Specialist medical practitioners	Low Income	West	Medical Doctors
30	Neuro-Surgeon	60	67	77	0.09	108,530	Togo	Maximum Estimate	2212 - Specialist medical practitioners	Low Income	West	Medical Doctors
31	Nurse Anaesthetist	325	384	454	0.55	18,302	Togo	Maximum Estimate	2221 - Nursing professionals	Low Income	West	Nursing Personnel
32	Nutritionist	1,408	1,450	1,488	1.79	5,583	Togo	Maximum Estimate	2265 - Dieticians and nutritionists	Low Income	West	Other Health Workers
33	Obstetrician & Gynaecologist	782	876	986	1.19	8,434	Togo	Maximum Estimate	2212 - Specialist medical practitioners	Low Income	West	Medical Doctors
34	Occupational Therapist	313	360	417	0.50	19,925	Togo	Maximum Estimate	2269 - Health professionals not elsewhere classified	Low Income	West	Other Health Workers
35	Oncology Nurse	109	128	153	0.18	54,206	Togo	Maximum Estimate	2221 - Nursing professionals	Low Income	West	Nursing Personnel
36	Operating Theatre Nurse	1,426	1,620	1,880	2.27	4,411	Togo	Maximum Estimate	2221 - Nursing professionals	Low Income	West	Nursing Personnel
37	Ophthalmic Nurse	144	164	189	0.23	43,962	Togo	Maximum Estimate	2221 - Nursing professionals	Low Income	West	Nursing Personnel
38	Ophthalmologist	50	58	68	0.08	122,650	Togo	Maximum Estimate	2212 - Specialist medical practitioners	Low Income	West	Medical Doctors
39	Optometrist	215	248	287	0.35	28,961	Togo	Maximum Estimate	2267 - Optometrists and ophthalmic opticians	Low Income	West	Other Health Workers
40	Orthopaedic Nurse	374	434	503	0.60	16,569	Togo	Maximum Estimate	2221 - Nursing professionals	Low Income	West	Nursing Personnel
41	Orthopaedic Surgeon	489	566	657	0.79	12,651	Togo	Maximum Estimate	2212 - Specialist medical practitioners	Low Income	West	Medical Doctors
42	Orthopaedic Technologist	407	456	510	0.61	16,360	Togo	Maximum Estimate	3214 - Medical and dental prosthetic technicians	Low Income	West	Other Health Workers
43	Paediatric Nurse	1,422	1,537	1,649	1.98	5,056	Togo	Maximum Estimate	2221 - Nursing professionals	Low Income	West	Nursing Personnel
44	Paediatric Surgeon	39	42	44	0.05	190,747	Togo	Maximum Estimate	2212 - Specialist medical practitioners	Low Income	West	Medical Doctors
45	Paediatrician	653	727	814	0.98	10,228	Togo	Maximum Estimate	2212 - Specialist medical practitioners	Low Income	West	Medical Doctors
46	Pathologist	100	113	132	0.16	62,990	Togo	Maximum Estimate	2212 - Specialist medical practitioners	Low Income	West	Medical Doctors
47	Pharmacist	789	819	861	1.04	9,602	Togo	Maximum Estimate	2262 - Pharmacists	Low Income	West	Pharmacist
48	Pharmacy Technician	1,467	1,624	1,803	2.17	4,616	Togo	Maximum Estimate	3213 - Pharmaceutical technicians and assistants	Low Income	West	Other Health Workers
49	Physician	1,475	1,644	1,842	2.21	4,515	Togo	Maximum Estimate	2212 - Specialist medical practitioners	Low Income	West	Medical Doctors
50	Physiotherapist	348	389	437	0.52	19,049	Togo	Maximum Estimate	2264 - Physiotherapists	Low Income	West	Other Health Workers
51	Plastic Surgeon	96	110	126	0.15	66,340	Togo	Maximum Estimate	2212 - Specialist medical practitioners	Low Income	West	Medical Doctors
52	Psychiatrist	676	745	839	1.01	9,897	Togo	Maximum Estimate	2212 - Specialist medical practitioners	Low Income	West	Medical Doctors
53	Radiation Oncologist	36	43	53	0.06	157,110	Togo	Maximum Estimate	2212 - Specialist medical practitioners	Low Income	West	Medical Doctors
54	Radiographer (Diagnostics and Therapy)	1,088	1,235	1,424	1.72	5,827	Togo	Maximum Estimate	3211 - Medical imaging and therapeutic equipment technicians	Low Income	West	Other Health Workers
55	Radiologist	405	466	542	0.66	15,263	Togo	Maximum Estimate	2212 - Specialist medical practitioners	Low Income	West	Medical Doctors
56	Registered General Nurse / State Certified Nurse	39,555	41,457	44,148	53.45	187	Togo	Maximum Estimate	2221 - Nursing professionals	Low Income	West	Nursing Personnel
57	Renal Nurse	2,651	3,070	3,662	4.43	2,259	Togo	Maximum Estimate	2221 - Nursing professionals	Low Income	West	Nursing Personnel
58	Respiratory Physician	124	140	158	0.19	52,672	Togo	Maximum Estimate	2212 - Specialist medical practitioners	Low Income	West	Medical Doctors
59	Rheumatologist	33	37	42	0.05	197,754	Togo	Maximum Estimate	2212 - Specialist medical practitioners	Low Income	West	Medical Doctors
60	Speech Therapist	111	122	135	0.16	61,559	Togo	Maximum Estimate	2266 - Audiologists and speech therapists	Low Income	West	Other Health Workers
61	Urologist	20	24	30	0.04	276,923	Togo	Maximum Estimate	2212 - Specialist medical practitioners	Low Income	West	Medical Doctors
1	Anaesthesiologist	599	688	790	0.17	58,322	Uganda	Maximum Estimate	2212 - Specialist medical practitioners	Low Income	East	Medical Doctors
2	Associate Nurse/Enrolled Nurse/Nursing Assistant	84,163	94,899	107,169	23.25	430	Uganda	Maximum Estimate	3221 - Nursing associate professionals	Low Income	East	Nursing Personnel
3	Audiologist	304	343	384	0.08	120,297	Uganda	Maximum Estimate	2266 - Audiologists and speech therapists	Low Income	East	Other Health Workers
4	Cardiologist	503	583	671	0.15	68,766	Uganda	Maximum Estimate	2212 - Specialist medical practitioners	Low Income	East	Medical Doctors
5	Cardiothoracic Surgeon	119	137	156	0.03	295,653	Uganda	Maximum Estimate	2212 - Specialist medical practitioners	Low Income	East	Medical Doctors
6	Clinical Officer/Physician Assistant	15,185	16,462	17,973	3.90	2,562	Uganda	Maximum Estimate	3256 - Medical assistants	Low Income	East	Other Health Workers
7	Clinical Pharmacist	1,899	2,115	2,412	0.53	18,905	Uganda	Maximum Estimate	2262 - Pharmacists	Low Income	East	Pharmacist
8	Clinical Psychologist	4,866	5,246	5,701	1.24	8,079	Uganda	Maximum Estimate	2634 - Psychologists	Low Income	East	Other Health Workers
9	Community health worker/Village health worker	86,569	94,634	102,918	22.30	448	Uganda	Maximum Estimate	3253 - Community health workers	Low Income	East	Other Health Workers
10	Dental Surgery Assistant	5,865	6,706	7,708	1.67	5,978	Uganda	Maximum Estimate	3251 - Dental assistants and therapists	Low Income	East	Other Health Workers
11	Dental Therapist	3,943	4,506	5,177	1.12	8,900	Uganda	Maximum Estimate	3251 - Dental assistants and therapists	Low Income	East	Other Health Workers
12	Dentist	5,007	5,682	6,632	1.44	6,927	Uganda	Maximum Estimate	2261 - Dentists	Low Income	East	Dentist
13	Dermatologist	205	232	262	0.06	176,640	Uganda	Maximum Estimate	2212 - Specialist medical practitioners	Low Income	East	Medical Doctors
14	Endocrinologist	188	222	277	0.06	164,178	Uganda	Maximum Estimate	2212 - Specialist medical practitioners	Low Income	East	Medical Doctors
15	ENT Surgeon	750	865	996	0.22	46,295	Uganda	Maximum Estimate	2212 - Specialist medical practitioners	Low Income	East	Medical Doctors
16	Environmental Health Officer	3,408	3,796	4,206	0.91	10,991	Uganda	Maximum Estimate	2263 - Environmental and occupational health and hygiene professionals	Low Income	East	Other Health Workers
17	Gastroenterologist	404	396	407	0.09	111,878	Uganda	Maximum Estimate	2212 - Specialist medical practitioners	Low Income	East	Medical Doctors
18	General Medical Practitioner (Generalist Doctor)	27,259	30,267	33,679	7.31	1,368	Uganda	Maximum Estimate	2211 - Generalist medical practitioners	Low Income	East	Medical Doctors
19	General Surgeon	501	593	716	0.16	63,948	Uganda	Maximum Estimate	2212 - Specialist medical practitioners	Low Income	East	Medical Doctors
20	Haematologist	170	185	202	0.04	228,164	Uganda	Maximum Estimate	2212 - Specialist medical practitioners	Low Income	East	Medical Doctors
21	Health Promoter/Health Educator	1,427	1,551	1,707	0.37	26,788	Uganda	Maximum Estimate	2269 - Health professionals not elsewhere classified	Low Income	East	Other Health Workers
22	Infectious Diseases Specialist	76	82	89	0.02	515,821	Uganda	Maximum Estimate	2212 - Specialist medical practitioners	Low Income	East	Medical Doctors
23	Intensive Care Nurse	2,264	2,468	2,679	0.58	17,238	Uganda	Maximum Estimate	2221 - Nursing professionals	Low Income	East	Nursing Personnel
24	Medical Laboratory Scientist	9,165	10,187	11,524	2.52	3,966	Uganda	Maximum Estimate	3212 - Medical and pathology laboratory technicians	Low Income	East	Other Health Workers
25	Medical Laboratory Technician	16,267	18,162	20,323	4.41	2,265	Uganda	Maximum Estimate	3212 - Medical and pathology laboratory technicians	Low Income	East	Other Health Workers
26	Medical Social Worker	1,604	1,761	2,024	0.46	21,687	Uganda	Maximum Estimate	1344 - Social welfare managers	Low Income	East	Other Health Workers
27	Mental Health Nurse	4,714	5,318	6,289	1.38	7,267	Uganda	Maximum Estimate	2221 - Nursing professionals	Low Income	East	Nursing Personnel
28	Midwife	43,800	48,771	54,730	11.87	842	Uganda	Maximum Estimate	2222 - Midwifery professionals	Low Income	East	Midwifery Personnel
29	Nephrologist	450	525	643	0.14	70,869	Uganda	Maximum Estimate	2212 - Specialist medical practitioners	Low Income	East	Medical Doctors
30	Neuro-Surgeon	183	210	244	0.05	187,811	Uganda	Maximum Estimate	2212 - Specialist medical practitioners	Low Income	East	Medical Doctors
31	Nurse Anaesthetist	1,637	1,971	2,371	0.52	19,383	Uganda	Maximum Estimate	2221 - Nursing professionals	Low Income	East	Nursing Personnel
32	Nutritionist	5,952	6,305	6,618	1.43	6,972	Uganda	Maximum Estimate	2265 - Dieticians and nutritionists	Low Income	East	Other Health Workers
33	Obstetrician & Gynaecologist	4,257	4,810	5,547	1.22	8,170	Uganda	Maximum Estimate	2212 - Specialist medical practitioners	Low Income	East	Medical Doctors
34	Occupational Therapist	994	1,149	1,344	0.29	34,176	Uganda	Maximum Estimate	2269 - Health professionals not elsewhere classified	Low Income	East	Other Health Workers
35	Oncology Nurse	500	598	730	0.16	62,652	Uganda	Maximum Estimate	2221 - Nursing professionals	Low Income	East	Nursing Personnel
36	Operating Theatre Nurse	5,537	6,373	7,615	1.67	6,000	Uganda	Maximum Estimate	2221 - Nursing professionals	Low Income	East	Nursing Personnel
37	Ophthalmic Nurse	490	565	661	0.14	69,458	Uganda	Maximum Estimate	2221 - Nursing professionals	Low Income	East	Nursing Personnel
38	Ophthalmologist	229	264	309	0.07	148,458	Uganda	Maximum Estimate	2212 - Specialist medical practitioners	Low Income	East	Medical Doctors
39	Optometrist	943	1,087	1,258	0.27	36,624	Uganda	Maximum Estimate	2267 - Optometrists and ophthalmic opticians	Low Income	East	Other Health Workers
40	Orthopaedic Nurse	1,093	1,271	1,470	0.32	31,398	Uganda	Maximum Estimate	2221 - Nursing professionals	Low Income	East	Nursing Personnel
41	Orthopaedic Surgeon	1,878	2,300	2,840	0.62	16,100	Uganda	Maximum Estimate	2212 - Specialist medical practitioners	Low Income	East	Medical Doctors
42	Orthopaedic Technologist	1,844	2,081	2,336	0.51	19,779	Uganda	Maximum Estimate	3214 - Medical and dental prosthetic technicians	Low Income	East	Other Health Workers
43	Paediatric Nurse	6,974	7,630	8,138	1.76	5,675	Uganda	Maximum Estimate	2221 - Nursing professionals	Low Income	East	Nursing Personnel

S/N	Health Professionals	2022	2026	2030	Density per 10,000 population	Required Population ratio (1 professional is to xxx population)	Name of Country	Modelling Scenario	ISCO-08 Match	Income Group Classification	Sub-Regional Grouping	SDG 3c Occupation
44	Paediatric Surgeon	276	304	327	0.07	141,370	Uganda	Maximum Estimate	2212 - Specialist medical practitioners	Low Income	East	Medical Doctors
45	Paediatrician	3,758	4,211	4,730	1.03	9,749	Uganda	Maximum Estimate	2212 - Specialist medical practitioners	Low Income	East	Medical Doctors
46	Pathologist	516	561	640	0.14	71,511	Uganda	Maximum Estimate	2212 - Specialist medical practitioners	Low Income	East	Medical Doctors
47	Pharmacist	7,045	7,546	8,122	1.77	5,650	Uganda	Maximum Estimate	2262 - Pharmacists	Low Income	East	Pharmacist
48	Pharmacy Technician	6,953	7,758	8,698	1.89	5,287	Uganda	Maximum Estimate	3213 - Pharmaceutical technicians and assistants	Low Income	East	Other Health Workers
49	Physician	6,125	6,925	7,884	1.71	5,843	Uganda	Maximum Estimate	2212 - Specialist medical practitioners	Low Income	East	Medical Doctors
50	Physiotherapist	1,595	1,798	2,031	0.44	22,700	Uganda	Maximum Estimate	2264 - Physiotherapists	Low Income	East	Other Health Workers
51	Plastic Surgeon	945	1,125	1,333	0.29	34,554	Uganda	Maximum Estimate	2212 - Specialist medical practitioners	Low Income	East	Medical Doctors
52	Psychiatrist	3,387	3,775	4,379	0.96	10,459	Uganda	Maximum Estimate	2212 - Specialist medical practitioners	Low Income	East	Medical Doctors
53	Radiation Oncologist	217	266	329	0.07	138,949	Uganda	Maximum Estimate	2212 - Specialist medical practitioners	Low Income	East	Medical Doctors
54	Radiographer (Diagnostics and Therapy)	4,067	4,753	5,759	1.27	7,850	Uganda	Maximum Estimate	3211 - Medical imaging and therapeutic equipment technicians	Low Income	East	Other Health Workers
55	Radiologist	1,624	1,990	2,565	0.59	17,055	Uganda	Maximum Estimate	2212 - Specialist medical practitioners	Low Income	East	Medical Doctors
56	Registered General Nurse / State Certified Nurse	159,277	172,974	190,326	41.59	240	Uganda	Maximum Estimate	2221 - Nursing professionals	Low Income	East	Nursing Personnel
57	Renal Nurse	8,802	10,260	12,586	2.76	3,619	Uganda	Maximum Estimate	2221 - Nursing professionals	Low Income	East	Nursing Personnel
58	Respiratory Physician	940	1,056	1,182	0.26	39,099	Uganda	Maximum Estimate	2212 - Specialist medical practitioners	Low Income	East	Medical Doctors
59	Rheumatologist	163	186	211	0.05	219,046	Uganda	Maximum Estimate	2212 - Specialist medical practitioners	Low Income	East	Medical Doctors
60	Speech Therapist	579	646	721	0.16	63,975	Uganda	Maximum Estimate	2266 - Audiologists and speech therapists	Low Income	East	Other Health Workers
61	Urologist	66	84	114	0.03	387,524	Uganda	Maximum Estimate	2212 - Specialist medical practitioners	Low Income	East	Medical Doctors
1	Anaesthesiologist	859	997	1,154	0.19	52,102	United Republic of	Maximum Estimate	2212 - Specialist medical practitioners	Lower-middle Income	East	Medical Doctors
2	Associate Nurse/Enrolled Nurse/Nursing Assistant	95,366	105,961	117,637	19.55	512	United Republic of	Maximum Estimate	3221 - Nursing associate professionals	Lower-middle Income	East	Nursing Personnel
3	Audiologist	408	460	515	0.09	116,926	United Republic of	Maximum Estimate	2266 - Audiologists and speech therapists	Lower-middle Income	East	Other Health Workers
4	Cardiologist	874	1,023	1,189	0.20	50,588	United Republic of	Maximum Estimate	2212 - Specialist medical practitioners	Lower-middle Income	East	Medical Doctors
5	Cardiothoracic Surgeon	173	201	231	0.04	260,592	United Republic of	Maximum Estimate	2212 - Specialist medical practitioners	Lower-middle Income	East	Medical Doctors
6	Clinical Officer/Physician Assistant	8,274	8,775	9,436	1.58	6,336	United Republic of	Maximum Estimate	3256 - Medical assistants	Lower-middle Income	East	Other Health Workers
7	Clinical Pharmacist	2,492	2,805	3,174	0.53	18,939	United Republic of	Maximum Estimate	2262 - Pharmacists	Lower-middle Income	East	Pharmacist
8	Clinical Psychologist	4,977	5,520	6,135	1.02	9,806	United Republic of	Maximum Estimate	2634 - Psychologists	Lower-middle Income	East	Other Health Workers
9	Community health worker/Village health worker	68,772	75,024	81,621	13.56	737	United Republic of	Maximum Estimate	3253 - Community health workers	Lower-middle Income	East	Other Health Workers
10	Dental Surgery Assistant	8,928	10,199	11,673	1.94	5,162	United Republic of	Maximum Estimate	3251 - Dental assistants and therapists	Lower-middle Income	East	Other Health Workers
11	Dental Therapist	5,991	6,841	7,828	1.30	7,697	United Republic of	Maximum Estimate	3251 - Dental assistants and therapists	Lower-middle Income	East	Other Health Workers
12	Dentist	8,267	9,385	10,774	1.79	5,593	United Republic of	Maximum Estimate	2261 - Dentists	Lower-middle Income	East	Dentist
13	Dermatologist	279	317	358	0.06	168,524	United Republic of	Maximum Estimate	2212 - Specialist medical practitioners	Lower-middle Income	East	Medical Doctors
14	Endocrinologist	225	265	319	0.05	186,802	United Republic of	Maximum Estimate	2212 - Specialist medical practitioners	Lower-middle Income	East	Medical Doctors
15	ENT Surgeon	1,093	1,255	1,441	0.24	41,741	United Republic of	Maximum Estimate	2212 - Specialist medical practitioners	Lower-middle Income	East	Medical Doctors
16	Environmental Health Officer	4,451	4,987	5,555	0.92	10,855	United Republic of	Maximum Estimate	2263 - Environmental and occupational health and hygiene professionals	Lower-middle Income	East	Other Health Workers
17	Gastroenterologist	516	540	579	0.10	103,009	United Republic of	Maximum Estimate	2212 - Specialist medical practitioners	Lower-middle Income	East	Medical Doctors
18	General Medical Practitioner (Generalist Doctor)	29,398	32,927	36,846	6.12	1,633	United Republic of	Maximum Estimate	2211 - Generalist medical practitioners	Lower-middle Income	East	Medical Doctors
19	General Surgeon	701	818	962	0.16	62,434	United Republic of	Maximum Estimate	2212 - Specialist medical practitioners	Lower-middle Income	East	Medical Doctors
20	Haematologist	397	450	508	0.08	118,595	United Republic of	Maximum Estimate	2212 - Specialist medical practitioners	Lower-middle Income	East	Medical Doctors
21	Health Promoter/Health Educator	804	849	907	0.15	65,966	United Republic of	Maximum Estimate	2269 - Health professionals not elsewhere classified	Lower-middle Income	East	Other Health Workers
22	Infectious Diseases Specialist	89	99	111	0.02	542,332	United Republic of	Maximum Estimate	2212 - Specialist medical practitioners	Lower-middle Income	East	Medical Doctors
23	Intensive Care Nurse	2,970	3,355	3,771	0.63	15,973	United Republic of	Maximum Estimate	2221 - Nursing professionals	Lower-middle Income	East	Nursing Personnel
24	Medical Laboratory Scientist	9,170	10,268	11,567	1.93	5,191	United Republic of	Maximum Estimate	3212 - Medical and pathology laboratory technicians	Lower-middle Income	East	Other Health Workers
25	Medical Laboratory Technician	15,524	17,408	19,506	3.24	3,084	United Republic of	Maximum Estimate	3212 - Medical and pathology laboratory technicians	Lower-middle Income	East	Other Health Workers
26	Medical Social Worker	2,099	2,251	2,414	0.40	24,898	United Republic of	Maximum Estimate	1344 - Social welfare managers	Lower-middle Income	East	Other Health Workers
27	Mental Health Nurse	5,935	6,774	7,878	1.32	7,604	United Republic of	Maximum Estimate	2221 - Nursing professionals	Lower-middle Income	East	Nursing Personnel
28	Midwife	84,961	95,889	108,050	17.92	558	United Republic of	Maximum Estimate	2222 - Midwifery professionals	Lower-middle Income	East	Midwifery Personnel
29	Nephrologist	746	871	1,038	0.17	57,588	United Republic of	Maximum Estimate	2212 - Specialist medical practitioners	Lower-middle Income	East	Medical Doctors
30	Neuro-Surgeon	235	277	329	0.05	181,892	United Republic of	Maximum Estimate	2212 - Specialist medical practitioners	Lower-middle Income	East	Medical Doctors
31	Nurse Anaesthetist	2,192	2,589	3,047	0.51	19,720	United Republic of	Maximum Estimate	2221 - Nursing professionals	Lower-middle Income	East	Nursing Personnel
32	Nutritionist	8,933	9,642	10,347	1.72	5,822	United Republic of	Maximum Estimate	2265 - Dieticians and nutritionists	Lower-middle Income	East	Other Health Workers
33	Obstetrician & Gynaecologist	5,506	6,208	6,998	1.16	8,603	United Republic of	Maximum Estimate	2212 - Specialist medical practitioners	Lower-middle Income	East	Medical Doctors
34	Occupational Therapist	1,412	1,639	1,912	0.32	31,387	United Republic of	Maximum Estimate	2269 - Health professionals not elsewhere classified	Lower-middle Income	East	Other Health Workers
35	Oncology Nurse	733	861	1,022	0.17	58,592	United Republic of	Maximum Estimate	2221 - Nursing professionals	Lower-middle Income	East	Nursing Personnel
36	Operating Theatre Nurse	8,111	9,306	10,848	1.81	5,519	United Republic of	Maximum Estimate	2221 - Nursing professionals	Lower-middle Income	East	Nursing Personnel
37	Ophthalmic Nurse	828	935	1,057	0.18	56,865	United Republic of	Maximum Estimate	2221 - Nursing professionals	Lower-middle Income	East	Nursing Personnel
38	Ophthalmologist	420	483	556	0.09	108,185	United Republic of	Maximum Estimate	2212 - Specialist medical practitioners	Lower-middle Income	East	Medical Doctors
39	Optometrist	2,402	2,755	3,147	0.52	19,134	United Republic of	Maximum Estimate	2267 - Optometrists and ophthalmic opticians	Lower-middle Income	East	Other Health Workers
40	Orthopaedic Nurse	1,487	1,747	2,043	0.34	29,438	United Republic of	Maximum Estimate	2221 - Nursing professionals	Lower-middle Income	East	Nursing Personnel
41	Orthopaedic Surgeon	2,753	3,214	3,754	0.62	16,005	United Republic of	Maximum Estimate	2212 - Specialist medical practitioners	Lower-middle Income	East	Medical Doctors
42	Orthopaedic Technologist	2,467	2,804	3,172	0.53	18,996	United Republic of	Maximum Estimate	3214 - Medical and dental prosthetic technicians	Lower-middle Income	East	Other Health Workers
43	Paediatric Nurse	11,103	12,359	13,606	2.26	4,434	United Republic of	Maximum Estimate	2221 - Nursing professionals	Lower-middle Income	East	Nursing Personnel
44	Paediatric Surgeon	342	379	414	0.07	145,713	United Republic of	Maximum Estimate	2212 - Specialist medical practitioners	Lower-middle Income	East	Medical Doctors
45	Paediatrician	5,764	6,406	7,107	1.18	8,472	United Republic of	Maximum Estimate	2212 - Specialist medical practitioners	Lower-middle Income	East	Medical Doctors
46	Pathologist	690	786	911	0.15	65,751	United Republic of	Maximum Estimate	2212 - Specialist medical practitioners	Lower-middle Income	East	Medical Doctors
47	Pharmacist	4,428	4,699	5,016	0.84	11,943	United Republic of	Maximum Estimate	2262 - Pharmacists	Lower-middle Income	East	Pharmacist
48	Pharmacy Technician	8,119	9,165	10,343	1.72	5,818	United Republic of	Maximum Estimate	3213 - Pharmaceutical technicians and assistants	Lower-middle Income	East	Other Health Workers
49	Physician	8,963	10,180	11,561	1.92	5,203	United Republic of	Maximum Estimate	2212 - Specialist medical practitioners	Lower-middle Income	East	Medical Doctors
50	Physiotherapist	2,189	2,485	2,823	0.47	21,305	United Republic of	Maximum Estimate	2264 - Physiotherapists	Lower-middle Income	East	Other Health Workers
51	Plastic Surgeon	1,437	1,700	2,000	0.33	30,065	United Republic of	Maximum Estimate	2212 - Specialist medical practitioners	Lower-middle Income	East	Medical Doctors
52	Psychiatrist	4,318	4,896	5,639	0.94	10,637	United Republic of	Maximum Estimate	2212 - Specialist medical practitioners	Lower-middle Income	East	Medical Doctors
53	Radiation Oncologist	296	354	425	0.07	140,932	United Republic of	Maximum Estimate	2212 - Specialist medical practitioners	Lower-middle Income	East	Medical Doctors
54	Radiographer (Diagnostics and Therapy)	5,891	6,798	7,911	1.32	7,586	United Republic of	Maximum Estimate	3211 - Medical imaging and therapeutic equipment technicians	Lower-middle Income	East	Other Health Workers
55	Radiologist	2,248	2,592	2,997	0.50	20,055	United Republic of	Maximum Estimate	2212 - Specialist medical practitioners	Lower-middle Income	East	Medical Doctors
56	Registered General Nurse / State Certified Nurse	206,476	225,945	249,581	41.69	240	United Republic of	Maximum Estimate	2221 - Nursing professionals	Lower-middle Income	East	Nursing Personnel
57	Renal Nurse	14,608	17,058	20,321	3.40	2,940	United Republic of	Maximum Estimate	2221 - Nursing professionals	Lower-middle Income	East	Nursing Personnel
58	Respiratory Physician	1,366	1,504	1,648	0.27	36,581	United Republic of	Maximum Estimate	2212 - Specialist medical practitioners	Lower-middle Income	East	Medical Doctors
59	Rheumatologist	236	271	309	0.05	194,667	United Republic of	Maximum Estimate	2212 - Specialist medical practitioners	Lower-middle Income	East	Medical Doctors
60	Speech Therapist	811	916	1,037	0.17	57,968	United Republic of	Maximum Estimate	2266 - Audiologists and speech therapists	Lower-middle Income	East	Other Health Workers
61	Urologist	104	123	147	0.02	406,807	United Republic of	Maximum Estimate	2212 - Specialist medical practitioners	Lower-middle Income	East	Medical Doctors
1	Anaesthesiologist	217	249	286	0.15	64,615	Zambia	Maximum Estimate	2212 - Specialist medical practitioners	Lower-middle Income	Southern	Medical Doctors
2	Associate Nurse/Enrolled Nurse/Nursing Assistant	34,047	38,035	42,584	22.99	435	Zambia	Maximum Estimate	3221 - Nursing associate professionals	Lower-middle Income	Southern	Nursing Personnel
3	Audiologist	119	134	150	0.08	123,899	Zambia	Maximum Estimate	2266 - Audiologists and speech therapists	Lower-middle Income	Southern	Other Health Workers
4	Cardiologist	213	247	285	0.15	65,043	Zambia	Maximum Estimate	2212 - Specialist medical practitioners	Lower-middle Income	Southern	Medical Doctors
5	Cardiothoracic Surgeon	43	49	56	0.03	333,484	Zambia	Maximum Estimate	2212 - Specialist medical practitioners	Lower-middle Income	Southern	Medical Doctors

S/N	Health Professionals	2022	2026	2030	Density per 10,000 population	Required Population ratio (1 professional is to xxx population)	Name of Country	Modelling Scenario	ISCO-08 Match	Income Group Classification	Sub-Regional Grouping	SDG 3c Occupation
6	Clinical Officer/Physician Assistant	5,335	5,539	5,854	3.19	3,139	Zambia	Maximum Estimate	3256 - Medical assistants	Lower-middle Income	Southern	Other Health Workers
7	Clinical Pharmacist	828	946	1,100	0.60	16,715	Zambia	Maximum Estimate	2262 - Pharmacists	Lower-middle Income	Southern	Pharmacist
8	Clinical Psychologist	1,867	2,125	2,438	1.32	7,596	Zambia	Maximum Estimate	2634 - Psychologists	Lower-middle Income	Southern	Other Health Workers
9	Community health worker/Village health worker	29,253	32,277	35,584	19.20	521	Zambia	Maximum Estimate	3253 - Community health workers	Lower-middle Income	Southern	Other Health Workers
10	Dental Surgery Assistant	2,715	3,112	3,583	1.93	5,177	Zambia	Maximum Estimate	3251 - Dental assistants and therapists	Lower-middle Income	Southern	Other Health Workers
11	Dental Therapist	1,822	2,087	2,401	1.29	7,725	Zambia	Maximum Estimate	3251 - Dental assistants and therapists	Lower-middle Income	Southern	Other Health Workers
12	Dentist	2,321	2,619	3,006	1.62	6,177	Zambia	Maximum Estimate	2261 - Dentists	Lower-middle Income	Southern	Dentist
13	Dermatologist	86	97	109	0.06	169,712	Zambia	Maximum Estimate	2212 - Specialist medical practitioners	Lower-middle Income	Southern	Medical Doctors
14	Endocrinologist	78	93	115	0.06	159,448	Zambia	Maximum Estimate	2212 - Specialist medical practitioners	Lower-middle Income	Southern	Medical Doctors
15	ENT Surgeon	321	369	426	0.23	43,486	Zambia	Maximum Estimate	2212 - Specialist medical practitioners	Lower-middle Income	Southern	Medical Doctors
16	Environmental Health Officer	1,370	1,524	1,686	0.91	11,011	Zambia	Maximum Estimate	2263 - Environmental and occupational health and hygiene professionals	Lower-middle Income	Southern	Other Health Workers
17	Gastroenterologist	282	293	318	0.17	58,081	Zambia	Maximum Estimate	2212 - Specialist medical practitioners	Lower-middle Income	Southern	Medical Doctors
18	General Medical Practitioner (Generalist Doctor)	10,914	12,118	13,519	7.31	1,368	Zambia	Maximum Estimate	2211 - Generalist medical practitioners	Lower-middle Income	Southern	Medical Doctors
19	General Surgeon	215	251	299	0.16	61,847	Zambia	Maximum Estimate	2212 - Specialist medical practitioners	Lower-middle Income	Southern	Medical Doctors
20	Haematologist	145	162	181	0.10	102,707	Zambia	Maximum Estimate	2212 - Specialist medical practitioners	Lower-middle Income	Southern	Medical Doctors
21	Health Promoter/Health Educator	466	491	529	0.29	34,539	Zambia	Maximum Estimate	2269 - Health professionals not elsewhere classified	Lower-middle Income	Southern	Other Health Workers
22	Infectious Diseases Specialist	35	38	41	0.02	446,436	Zambia	Maximum Estimate	2212 - Specialist medical practitioners	Lower-middle Income	Southern	Medical Doctors
23	Intensive Care Nurse	949	1,057	1,172	0.63	15,832	Zambia	Maximum Estimate	2221 - Nursing professionals	Lower-middle Income	Southern	Nursing Personnel
24	Medical Laboratory Scientist	3,820	4,293	4,899	2.66	3,759	Zambia	Maximum Estimate	3212 - Medical and pathology laboratory technicians	Lower-middle Income	Southern	Other Health Workers
25	Medical Laboratory Technician	4,526	5,115	5,826	3.16	3,166	Zambia	Maximum Estimate	3212 - Medical and pathology laboratory technicians	Lower-middle Income	Southern	Other Health Workers
26	Medical Social Worker	856	944	1,080	0.60	16,730	Zambia	Maximum Estimate	1344 - Social welfare managers	Lower-middle Income	Southern	Other Health Workers
27	Mental Health Nurse	1,851	2,126	2,532	1.37	7,283	Zambia	Maximum Estimate	2221 - Nursing professionals	Lower-middle Income	Southern	Nursing Personnel
28	Midwife	18,450	20,313	22,544	12.15	823	Zambia	Maximum Estimate	2222 - Midwifery professionals	Lower-middle Income	Southern	Midwifery Personnel
29	Nephrologist	230	271	333	0.18	55,248	Zambia	Maximum Estimate	2212 - Specialist medical practitioners	Lower-middle Income	Southern	Medical Doctors
30	Neuro-Surgeon	61	70	81	0.04	226,506	Zambia	Maximum Estimate	2212 - Specialist medical practitioners	Lower-middle Income	Southern	Medical Doctors
31	Nurse Anaesthetist	662	790	941	0.51	19,642	Zambia	Maximum Estimate	2221 - Nursing professionals	Lower-middle Income	Southern	Nursing Personnel
32	Nutritionist	3,133	3,368	3,606	1.94	5,145	Zambia	Maximum Estimate	2265 - Dieticians and nutritionists	Lower-middle Income	Southern	Other Health Workers
33	Obstetrician & Gynaecologist	1,767	2,015	2,333	1.27	7,852	Zambia	Maximum Estimate	2212 - Specialist medical practitioners	Lower-middle Income	Southern	Medical Doctors
34	Occupational Therapist	340	395	466	0.25	39,567	Zambia	Maximum Estimate	2269 - Health professionals not elsewhere classified	Lower-middle Income	Southern	Other Health Workers
35	Oncology Nurse	228	272	331	0.18	55,665	Zambia	Maximum Estimate	2221 - Nursing professionals	Lower-middle Income	Southern	Nursing Personnel
36	Operating Theatre Nurse	2,406	2,809	3,385	1.84	5,437	Zambia	Maximum Estimate	2221 - Nursing professionals	Lower-middle Income	Southern	Nursing Personnel
37	Ophthalmic Nurse	226	257	295	0.16	62,591	Zambia	Maximum Estimate	2221 - Nursing professionals	Lower-middle Income	Southern	Nursing Personnel
38	Ophthalmologist	97	112	131	0.07	140,939	Zambia	Maximum Estimate	2212 - Specialist medical practitioners	Lower-middle Income	Southern	Medical Doctors
39	Optometrist	393	453	523	0.28	35,378	Zambia	Maximum Estimate	2267 - Optometrists and ophthalmic opticians	Lower-middle Income	Southern	Other Health Workers
40	Orthopaedic Nurse	318	370	429	0.23	43,194	Zambia	Maximum Estimate	2221 - Nursing professionals	Lower-middle Income	Southern	Nursing Personnel
41	Orthopaedic Surgeon	868	1,039	1,250	0.68	14,760	Zambia	Maximum Estimate	2212 - Specialist medical practitioners	Lower-middle Income	Southern	Medical Doctors
42	Orthopaedic Technologist	702	789	883	0.48	21,000	Zambia	Maximum Estimate	3214 - Medical and dental prosthetic technicians	Lower-middle Income	Southern	Other Health Workers
43	Paediatric Nurse	3,355	3,719	4,096	2.21	4,535	Zambia	Maximum Estimate	2221 - Nursing professionals	Lower-middle Income	Southern	Nursing Personnel
44	Paediatric Surgeon	95	102	109	0.06	170,978	Zambia	Maximum Estimate	2212 - Specialist medical practitioners	Lower-middle Income	Southern	Medical Doctors
45	Paediatrician	1,268	1,470	1,716	0.93	10,778	Zambia	Maximum Estimate	2212 - Specialist medical practitioners	Lower-middle Income	Southern	Medical Doctors
46	Pathologist	237	260	297	0.16	62,199	Zambia	Maximum Estimate	2212 - Specialist medical practitioners	Lower-middle Income	Southern	Medical Doctors
47	Pharmacist	2,321	2,319	2,358	1.29	7,732	Zambia	Maximum Estimate	2262 - Pharmacists	Lower-middle Income	Southern	Pharmacist
48	Pharmacy Technician	2,847	3,137	3,489	1.89	5,288	Zambia	Maximum Estimate	3213 - Pharmaceutical technicians and assistants	Lower-middle Income	Southern	Other Health Workers
49	Physician	2,576	2,930	3,359	1.82	5,508	Zambia	Maximum Estimate	2212 - Specialist medical practitioners	Lower-middle Income	Southern	Medical Doctors
50	Physiotherapist	630	710	803	0.43	23,048	Zambia	Maximum Estimate	2264 - Physiotherapists	Lower-middle Income	Southern	Other Health Workers
51	Plastic Surgeon	367	424	486	0.26	38,164	Zambia	Maximum Estimate	2212 - Specialist medical practitioners	Lower-middle Income	Southern	Medical Doctors
52	Psychiatrist	1,352	1,532	1,790	0.97	10,319	Zambia	Maximum Estimate	2212 - Specialist medical practitioners	Lower-middle Income	Southern	Medical Doctors
53	Radiation Oncologist	97	118	145	0.08	126,316	Zambia	Maximum Estimate	2212 - Specialist medical practitioners	Lower-middle Income	Southern	Medical Doctors
54	Radiographer (Diagnostics and Therapy)	1,914	2,238	2,686	1.47	6,818	Zambia	Maximum Estimate	3211 - Medical imaging and therapeutic equipment technicians	Lower-middle Income	Southern	Other Health Workers
55	Radiologist	743	898	1,125	0.63	15,921	Zambia	Maximum Estimate	2212 - Specialist medical practitioners	Lower-middle Income	Southern	Medical Doctors
56	Registered General Nurse / State Certified Nurse	68,050	73,999	81,881	44.45	225	Zambia	Maximum Estimate	2221 - Nursing professionals	Lower-middle Income	Southern	Nursing Personnel
57	Renal Nurse	4,458	5,258	6,461	3.51	2,845	Zambia	Maximum Estimate	2221 - Nursing professionals	Lower-middle Income	Southern	Nursing Personnel
58	Respiratory Physician	227	272	326	0.18	56,745	Zambia	Maximum Estimate	2212 - Specialist medical practitioners	Lower-middle Income	Southern	Medical Doctors
59	Rheumatologist	70	80	92	0.05	202,328	Zambia	Maximum Estimate	2212 - Specialist medical practitioners	Lower-middle Income	Southern	Medical Doctors
60	Speech Therapist	237	264	295	0.16	62,741	Zambia	Maximum Estimate	2266 - Audiologists and speech therapists	Lower-middle Income	Southern	Other Health Workers
61	Urologist	31	39	52	0.03	348,408	Zambia	Maximum Estimate	2212 - Specialist medical practitioners	Lower-middle Income	Southern	Medical Doctors
1	Anaesthesiologist	241	277	319	0.21	46,702	Zimbabwe	Maximum Estimate	2212 - Specialist medical practitioners	Lower-middle Income	Southern	Medical Doctors
2	Associate Nurse/Enrolled Nurse/Nursing Assistant	27,107	29,652	32,561	21.82	458	Zimbabwe	Maximum Estimate	3221 - Nursing associate professionals	Lower-middle Income	Southern	Nursing Personnel
3	Audiologist	114	126	139	0.09	108,035	Zimbabwe	Maximum Estimate	2266 - Audiologists and speech therapists	Lower-middle Income	Southern	Other Health Workers
4	Cardiologist	260	292	327	0.22	45,776	Zimbabwe	Maximum Estimate	2212 - Specialist medical practitioners	Lower-middle Income	Southern	Medical Doctors
5	Cardiothoracic Surgeon	34	39	45	0.03	333,559	Zimbabwe	Maximum Estimate	2212 - Specialist medical practitioners	Lower-middle Income	Southern	Medical Doctors
6	Clinical Officer/Physician Assistant	2,582	2,655	2,810	1.91	5,245	Zimbabwe	Maximum Estimate	3256 - Medical assistants	Lower-middle Income	Southern	Other Health Workers
7	Clinical Pharmacist	697	771	873	0.59	16,901	Zimbabwe	Maximum Estimate	2262 - Pharmacists	Lower-middle Income	Southern	Pharmacist
8	Clinical Psychologist	1,491	1,568	1,664	1.12	8,947	Zimbabwe	Maximum Estimate	2634 - Psychologists	Lower-middle Income	Southern	Other Health Workers
9	Community health worker/Village health worker	14,108	14,831	15,553	10.44	958	Zimbabwe	Maximum Estimate	3253 - Community health workers	Lower-middle Income	Southern	Other Health Workers
10	Dental Surgery Assistant	2,321	2,591	2,915	1.95	5,116	Zimbabwe	Maximum Estimate	3251 - Dental assistants and therapists	Lower-middle Income	Southern	Other Health Workers
11	Dental Therapist	1,555	1,736	1,952	1.31	7,640	Zimbabwe	Maximum Estimate	3251 - Dental assistants and therapists	Lower-middle Income	Southern	Other Health Workers
12	Dentist	1,940	2,130	2,405	1.62	6,167	Zimbabwe	Maximum Estimate	2261 - Dentists	Lower-middle Income	Southern	Dentist
13	Dermatologist	79	87	96	0.06	156,501	Zimbabwe	Maximum Estimate	2212 - Specialist medical practitioners	Lower-middle Income	Southern	Medical Doctors
14	Endocrinologist	122	143	174	0.12	84,163	Zimbabwe	Maximum Estimate	2212 - Specialist medical practitioners	Lower-middle Income	Southern	Medical Doctors
15	ENT Surgeon	317	356	401	0.27	37,341	Zimbabwe	Maximum Estimate	2212 - Specialist medical practitioners	Lower-middle Income	Southern	Medical Doctors
16	Environmental Health Officer	1,107	1,202	1,300	0.87	11,526	Zimbabwe	Maximum Estimate	2263 - Environmental and occupational health and hygiene professionals	Lower-middle Income	Southern	Other Health Workers
17	Gastroenterologist	232	232	241	0.16	60,897	Zimbabwe	Maximum Estimate	2212 - Specialist medical practitioners	Lower-middle Income	Southern	Medical Doctors
18	General Medical Practitioner (Generalist Doctor)	8,243	8,989	9,853	6.61	1,513	Zimbabwe	Maximum Estimate	2211 - Generalist medical practitioners	Lower-middle Income	Southern	Medical Doctors
19	General Surgeon	371	422	493	0.33	30,030	Zimbabwe	Maximum Estimate	2212 - Specialist medical practitioners	Lower-middle Income	Southern	Medical Doctors
20	Haematologist	63	69	75	0.05	198,719	Zimbabwe	Maximum Estimate	2212 - Specialist medical practitioners	Lower-middle Income	Southern	Medical Doctors
21	Health Promoter/Health Educator	228	240	261	0.18	56,187	Zimbabwe	Maximum Estimate	2269 - Health professionals not elsewhere classified	Lower-middle Income	Southern	Other Health Workers
22	Infectious Diseases Specialist	37	40	43	0.03	344,807	Zimbabwe	Maximum Estimate	2212 - Specialist medical practitioners	Lower-middle Income	Southern	Medical Doctors
23	Intensive Care Nurse	732	815	904	0.60	16,554	Zimbabwe	Maximum Estimate	2221 - Nursing professionals	Lower-middle Income	Southern	Nursing Personnel
24	Medical Laboratory Scientist	3,086	3,387	3,806	2.58	3,880	Zimbabwe	Maximum Estimate	3212 - Medical and pathology laboratory technicians	Lower-middle Income	Southern	Other Health Workers
25	Medical Laboratory Technician	2,850	3,093	3,409	2.30	4,347	Zimbabwe	Maximum Estimate	3212 - Medical and pathology laboratory technicians	Lower-middle Income	Southern	Other Health Workers
26	Medical Social Worker	688	738	821	0.57	17,612	Zimbabwe	Maximum Estimate	1344 - Social welfare managers	Lower-middle Income	Southern	Other Health Workers
27	Mental Health Nurse	1,310	1,441	1,646	1.12	8,969	Zimbabwe	Maximum Estimate	2221 - Nursing professionals	Lower-middle Income	Southern	Nursing Personnel
28	Midwife	16,264	17,634	19,258	12.93	774	Zimbabwe	Maximum Estimate	2222 - Midwifery professionals	Lower-middle Income	Southern	Midwifery Personnel

S/N	Health Professionals	2022	2026	2030	Density per 10,000 population	Required Population ratio (1 professional is to xxx population)	Name of Country	Modelling Scenario	ISCO-08 Match	Income Group Classification	Sub-Regional Grouping	SDG 3c Occupation
29	Nephrologist	285	335	410	0.28	35,774	Zimbabwe	Maximum Estimate	2212 - Specialist medical practitioners	Lower-middle Income	Southern	Medical Doctors
30	Neuro-Surgeon	76	87	99	0.07	150,896	Zimbabwe	Maximum Estimate	2212 - Specialist medical practitioners	Lower-middle Income	Southern	Medical Doctors
31	Nurse Anaesthetist	757	893	1,054	0.71	14,160	Zimbabwe	Maximum Estimate	2221 - Nursing professionals	Lower-middle Income	Southern	Nursing Personnel
32	Nutritionist	2,229	2,345	2,452	1.64	6,095	Zimbabwe	Maximum Estimate	2265 - Dieticians and nutritionists	Lower-middle Income	Southern	Other Health Workers
33	Obstetrician & Gynaecologist	1,726	1,922	2,174	1.47	6,798	Zimbabwe	Maximum Estimate	2212 - Specialist medical practitioners	Lower-middle Income	Southern	Medical Doctors
34	Occupational Therapist	333	386	457	0.31	32,400	Zimbabwe	Maximum Estimate	2269 - Health professionals not elsewhere classified	Lower-middle Income	Southern	Other Health Workers
35	Oncology Nurse	259	306	368	0.25	40,130	Zimbabwe	Maximum Estimate	2221 - Nursing professionals	Lower-middle Income	Southern	Nursing Personnel
36	Operating Theatre Nurse	2,835	3,230	3,776	2.55	3,917	Zimbabwe	Maximum Estimate	2221 - Nursing professionals	Lower-middle Income	Southern	Nursing Personnel
37	Ophthalmic Nurse	350	393	445	0.30	33,479	Zimbabwe	Maximum Estimate	2221 - Nursing professionals	Lower-middle Income	Southern	Nursing Personnel
38	Ophthalmologist	135	154	177	0.12	83,853	Zimbabwe	Maximum Estimate	2212 - Specialist medical practitioners	Lower-middle Income	Southern	Medical Doctors
39	Optometrist	716	814	928	0.62	16,093	Zimbabwe	Maximum Estimate	2267 - Optometrists and ophthalmic opticians	Lower-middle Income	Southern	Other Health Workers
40	Orthopaedic Nurse	271	325	387	0.26	38,559	Zimbabwe	Maximum Estimate	2221 - Nursing professionals	Lower-middle Income	Southern	Nursing Personnel
41	Orthopaedic Surgeon	841	970	1,126	0.76	13,240	Zimbabwe	Maximum Estimate	2212 - Specialist medical practitioners	Lower-middle Income	Southern	Medical Doctors
42	Orthopaedic Technologist	558	619	686	0.46	21,821	Zimbabwe	Maximum Estimate	3214 - Medical and dental prosthetic technicians	Lower-middle Income	Southern	Other Health Workers
43	Paediatric Nurse	2,029	2,187	2,306	1.55	6,461	Zimbabwe	Maximum Estimate	2221 - Nursing professionals	Lower-middle Income	Southern	Nursing Personnel
44	Paediatric Surgeon	77	84	89	0.06	168,441	Zimbabwe	Maximum Estimate	2212 - Specialist medical practitioners	Lower-middle Income	Southern	Medical Doctors
45	Paediatrician	791	897	1,031	0.69	14,410	Zimbabwe	Maximum Estimate	2212 - Specialist medical practitioners	Lower-middle Income	Southern	Medical Doctors
46	Pathologist	240	262	297	0.20	49,797	Zimbabwe	Maximum Estimate	2212 - Specialist medical practitioners	Lower-middle Income	Southern	Medical Doctors
47	Pharmacist	1,192	1,234	1,312	0.89	11,188	Zimbabwe	Maximum Estimate	2262 - Pharmacists	Lower-middle Income	Southern	Pharmacist
48	Pharmacy Technician	2,273	2,502	2,776	1.86	5,364	Zimbabwe	Maximum Estimate	3213 - Pharmaceutical technicians and assistants	Lower-middle Income	Southern	Other Health Workers
49	Physician	2,460	2,738	3,081	2.07	4,833	Zimbabwe	Maximum Estimate	2212 - Specialist medical practitioners	Lower-middle Income	Southern	Medical Doctors
50	Physiotherapist	537	595	664	0.45	22,461	Zimbabwe	Maximum Estimate	2264 - Physiotherapists	Lower-middle Income	Southern	Other Health Workers
51	Plastic Surgeon	192	215	240	0.16	62,369	Zimbabwe	Maximum Estimate	2212 - Specialist medical practitioners	Lower-middle Income	Southern	Medical Doctors
52	Psychiatrist	1,043	1,144	1,294	0.87	11,432	Zimbabwe	Maximum Estimate	2212 - Specialist medical practitioners	Lower-middle Income	Southern	Medical Doctors
53	Radiation Oncologist	106	126	151	0.10	98,446	Zimbabwe	Maximum Estimate	2212 - Specialist medical practitioners	Lower-middle Income	Southern	Medical Doctors
54	Radiographer (Diagnostics and Therapy)	2,142	2,445	2,864	1.94	5,146	Zimbabwe	Maximum Estimate	3211 - Medical imaging and therapeutic equipment technicians	Lower-middle Income	Southern	Other Health Workers
55	Radiologist	832	974	1,177	0.81	12,359	Zimbabwe	Maximum Estimate	2212 - Specialist medical practitioners	Lower-middle Income	Southern	Medical Doctors
56	Registered General Nurse / State Certified Nurse	50,098	55,550	62,360	42.07	238	Zimbabwe	Maximum Estimate	2221 - Nursing professionals	Lower-middle Income	Southern	Nursing Personnel
57	Renal Nurse	5,475	6,438	7,871	5.37	1,863	Zimbabwe	Maximum Estimate	2221 - Nursing professionals	Lower-middle Income	Southern	Nursing Personnel
58	Respiratory Physician	133	153	177	0.12	84,394	Zimbabwe	Maximum Estimate	2212 - Specialist medical practitioners	Lower-middle Income	Southern	Medical Doctors
59	Rheumatologist	64	71	79	0.05	188,702	Zimbabwe	Maximum Estimate	2212 - Specialist medical practitioners	Lower-middle Income	Southern	Medical Doctors
60	Speech Therapist	196	215	235	0.16	63,637	Zimbabwe	Maximum Estimate	2266 - Audiologists and speech therapists	Lower-middle Income	Southern	Other Health Workers
61	Urologist	39	46	57	0.04	256,506	Zimbabwe	Maximum Estimate	2212 - Specialist medical practitioners	Lower-middle Income	Southern	Medical Doctors
