# Supplementary material for: Projected health workforce requirements and shortage for addressing the disease burden in the WHO Africa Region, 2022–2030: a needs-based modelling study
Source: BMJ Glob Health. 2024 Oct 22;7(Suppl 1):e015972. doi: 10.1136/bmjgh-2024-015972 (PMC11789529; doi:10.1136/bmjgh-2024-015972)
Supplement: online supplemental material 4 [file bmjgh-7-Suppl_1-s004.pdf]

21/02/24 7:55:08 PM Stepwise Forward Regression [For Tableua.xlsx]
Dependent variable: Rate of Change!J:J
Independent variables: Rate of Change!I:I;Rate of Change!K:M

Stepwise Forward Regression

Dependent variable	**Total HWF Required_2022**
N	47

Correlation Coefficients

Variable	Population (2022)	NCDs_2019	CD_2019	Injuries_2019	Total HWF Required_2022
Population (2022)	1.	0.99077	0.93645	0.98532	0.99601
NCDs_2019	0.99077	1	0.93393	0.98956	0.99195
CD_2019	0.93645	0.93393	1	0.9428	0.91582
Injuries_2019	0.98532	0.98956	0.9428	1	0.9842
Total HWF Required_2022	0.99601	0.99195	0.91582	0.9842	1.

Descriptive Statistics

Independent variable	*Mean*	*Variance*	*Standard Deviation*
**Population (2022)**	23,228.93617	1,242,196,157.10453	35,244.80326
**NCDs_2019**	3,550.57448	28,024,128.25734	5,293.78204
**CD_2019**	6,774.5946	182,505,131.57534	13,509.44601
**Injuries_2019**	1,069.88749	2,531,809.57336	1,591.1661
**Total HWF Required_2022**	207,417.07044	9.99793E+10	316,195.0743

Step 1

R	*R-Squared*	*Adjusted R-Squared*	*S*	*F*	*p-value*
0.99601	0.99203	0.99185	28,541.21945	5,600.76741	0

VAR	*Coefficients*	*Standard Error*	*Beta*	*t*	*p-value > t*	*VIF*	*TOL*
**Population (2022)**	8.93557	0.1194	0.99601	74.83828	0	1.	1.
**Intercept**	-146.72702						

Next step entry candidates

**Variable**	**Partial**	**F**	**p-value**	**% Contribution**
**NCDs_2019**	0.42387	9.63672	0.00333	37%
**CD_2019**	0.53922	18.03853	0.00011	47%
**Injuries_2019**	0.18455	1.5514	0.21952	16%

Adding CD_2019 variable

Step 2

R	*R-Squared*	*Adjusted R-Squared*	*S*	*F*	*p-value*
0.99717	0.99435	0.99409	24,307.93288	3,869.72352	0

VAR	*Coefficients*	*Standard Error*	*Beta*	*t*	*p-value > t*	*VIF*	*TOL*
**Population (2022)**	10.08848	0.28987	1.12452	34.80292	0	8.12591	0.12306
**CD_2019**	-3.21195	0.75625	-0.13723	-4.24718	0.00011	8.12591	0.12306
**Intercept**	-5,167.93333						

Next step entry candidates

Variable	Partial	F	p-value
**NCDs_2019**	0.59069	23.04407	0.00002
**Injuries_2019**	0.46068	11.58448	0.00145

Adding NCDs_2019 variable

Step 3

R	*R-Squared*	*Adjusted R-Squared*	*S*	*F*	*p-value*
0.99816	0.99632	0.99606	19,840.72083	3,879.98891	0

VAR	*Coefficients*	*Standard Error*	*Beta*	*t*	*p-value > t*	*VIF*	*TOL*
**Population (2022)**	7.28928	0.62929	0.8125	11.58338	0.	57.48201	0.0174
**CD_2019**	-3.59678	0.62246	-0.15367	-5.77836	7.67494E-7	8.26297	0.12102
**NCDs_2019**	19.73818	4.11176	0.33046	4.80042	0.00002	55.36431	0.01806
**Intercept**	-7,620.3701						

Next step entry candidates

Variable	Partial	F	p-value
**Injuries_2019**	0.18679	1.51835	0.22472

No more variables meet the criterion.

Final Step

R	*R-Squared*	*Adjusted R-Squared*	*S*	*F*	*p-value*
0.99816	0.99632	0.99606	19,840.72083	3,879.98891	0

VAR	*Coefficients*	*Standard Error*	*Beta*	*t*	*p-value > t*	*VIF*	*TOL*
**Population (2022)**	7.28928	0.62929	0.8125	11.58338	0.	57.48201	0.0174
**CD_2019**	-3.59678	0.62246	-0.15367	-5.77836	7.67494E-7	8.26297	0.12102
**NCDs_2019**	19.73818	4.11176	0.33046	4.80042	0.00002	55.36431	0.01806
**Intercept**	-7,620.3701						
