# Supplementary material for: Projected health workforce requirements and shortage for addressing the disease burden in the WHO Africa Region, 2022–2030: a needs-based modelling study
Source: BMJ Glob Health. 2024 Oct 22;7(Suppl 1):e015972. doi: 10.1136/bmjgh-2024-015972 (PMC11789529; doi:10.1136/bmjgh-2024-015972)
Supplement: online supplemental material 5 [file bmjgh-7-Suppl_1-s005.pdf]

Supplementary material 6: Country-by-country stock projections

Country	Country code	income level	Occupation	Baseline 2022	Projected 2026	Projected 2030
Algeria	DZA	Lower-Middle-Income Economies	Medical Doctors	75,512	76,939	84,499
Angola	AGO	Lower-Middle-Income Economies	Medical Doctors	8,693	9,942	11,251
Benin	BEN	Lower-Middle-Income Economies	Medical Doctors	2,608	4,659	6,635
Botswana	BWA	High/Upper-Middle-Income Economies	Medical Doctors	992	1,126	1,265
Burkina Faso	BFA	Low-Income Economies	Medical Doctors	3,323	7,466	11,582
Burundi	BDI	Low-Income Economies	Medical Doctors	1,015	1,879	2,736
Cameroon	CMR	Lower-Middle-Income Economies	Medical Doctors	3,759	5,843	7,886
Cabo Verde	CPV	Lower-Middle-Income Economies	Medical Doctors	2,644	2,868	3,156
Central African Republic	CAF	Low-Income Economies	Medical Doctors	146	189	232
Chad	TCD	Low-Income Economies	Medical Doctors	1,507	2,400	3,268
Comoros	COM	Lower-Middle-Income Economies	Medical Doctors	354	404	457
Congo	COG	Lower-Middle-Income Economies	Medical Doctors	1,042	1,109	1,167
Côte d'Ivoire	CIV	Lower-Middle-Income Economies	Medical Doctors	6,328	6,702	7,015
Democratic Republic of the Congo	COD	Low-Income Economies	Medical Doctors	21,290	37,475	53,541

Country	Country code	income level	Occupation	Baseline 2022	Projected 2026	Projected 2030
Equatorial Guinea	GNQ	High/Upper-Middle-Income Economies	Medical Doctors	275	2,354	4,332
Eritrea	ERI	Low-Income Economies	Medical Doctors	299	465	628
Eswatini	SWZ	Lower-Middle-Income Economies	Medical Doctors	687	815	944
Ethiopia	ETH	Low-Income Economies	Medical Doctors	23,741	31,680	39,654
Gabon	GAB	High/Upper-Middle-Income Economies	Medical Doctors	1,265	1,194	1,134
Gambia	GMB	Low-Income Economies	Medical Doctors	274	329	385
Ghana	GHA	Lower-Middle-Income Economies	Medical Doctors	4,726	8,660	12,511
Guinea	GIN	Low-Income Economies	Medical Doctors	2,945	4,983	7,008
Guinea-Bissau	GNB	Low-Income Economies	Medical Doctors	531	822	1,111
Kenya	KEN	Lower-Middle-Income Economies	Medical Doctors	12,792	14,080	15,303
Lesotho	LSO	Lower-Middle-Income Economies	Medical Doctors	537	535	540
Liberia	LBR	Low-Income Economies	Medical Doctors	954	1,000	1,052
Madagascar	MDG	Low-Income Economies	Medical Doctors	5,230	5,865	6,527
Malawi	MWI	Low-Income Economies	Medical Doctors	1,104	1,391	1,686
Mali	MLI	Low-Income Economies	Medical Doctors	4,402	6,783	9,155
Mauritania	MRT	Lower-Middle-Income Economies	Medical Doctors	1,273	1,457	1,649

Country	Country code	income level	Occupation	Baseline 2022	Projected 2026	Projected 2030
Mauritius	MUS	High/Upper-Middle-Income Economies	Medical Doctors	1,834	2,825	3,835
Mozambique	MOZ	Low-Income Economies	Medical Doctors	5,719	7,711	9,711
Namibia	NAM	High/Upper-Middle-Income Economies	Medical Doctors	1,597	2,752	3,907
Niger	NER	Low-Income Economies	Medical Doctors	706	1,706	2,694
Nigeria	NGA	Lower-Middle-Income Economies	Medical Doctors	86,181	92,962	100,715
Rwanda	RWA	Low-Income Economies	Medical Doctors	1,648	1,171	713
Sao Tome and Principe	STP	Lower-Middle-Income Economies	Medical Doctors	105	120	135
Senegal	SEN	Lower-Middle-Income Economies	Medical Doctors	2,561	3,624	4,659
Seychelles	SYC	High/Upper-Middle-Income Economies	Medical Doctors	828	718	699
Sierra Leone	SLE	Low-Income Economies	Medical Doctors	1,106	1,704	2,302
South Africa	ZAF	High/Upper-Middle-Income Economies	Medical Doctors	48,021	44,602	43,222
South Sudan	SSD	Low-Income Economies	Medical Doctors	456	705	954
United Republic of Tanzania	TZA	Lower-Middle-Income Economies	Medical Doctors	8,693	10,754	12,848
Togo	TGO	Low-Income Economies	Medical Doctors	730	846	979
Uganda	UGA	Low-Income Economies	Medical Doctors	9,052	14,321	19,566
Zambia	ZMB	Lower-Middle-Income Economies	Medical Doctors	6,531	6,250	6,275

Country	Country code	income level	Occupation	Baseline 2022	Projected 2026	Projected 2030
Zimbabwe	ZWE	Lower-Middle-Income Economies	Medical Doctors	3,132	3,748	4,365
Algeria	DZA	Lower-Middle-Income Economies	Nursing Personnel	104,776	124,888	144,020
Angola	AGO	Lower-Middle-Income Economies	Nursing Personnel	66,717	72,904	78,938
Benin	BEN	Lower-Middle-Income Economies	Nursing Personnel	5,893	6,469	7,046
Botswana	BWA	High/Upper-Middle-Income Economies	Nursing Personnel	8,028	9,099	10,110
Burkina Faso	BFA	Low-Income Economies	Nursing Personnel	13,961	16,984	19,934
Burundi	BDI	Low-Income Economies	Nursing Personnel	9,372	13,290	17,166
Cameroon	CMR	Lower-Middle-Income Economies	Nursing Personnel	16,473	22,073	27,570
Cabo Verde	CPV	Lower-Middle-Income Economies	Nursing Personnel	941	1,012	1,113
Central African Republic	CAF	Low-Income Economies	Nursing Personnel	648	930	1,207
Chad	TCD	Low-Income Economies	Nursing Personnel	3,387	11,407	19,204
Comoros	COM	Lower-Middle-Income Economies	Nursing Personnel	785	873	958
Congo	COG	Lower-Middle-Income Economies	Nursing Personnel	5,573	5,478	5,326
Côte d'Ivoire	CIV	Lower-Middle-Income Economies	Nursing Personnel	19,225	21,407	23,343
Democratic Republic of the Congo	COD	Low-Income Economies	Nursing Personnel	115,316	180,446	244,282
Equatorial Guinea	GNQ	High/Upper-Middle-Income Economies	Nursing Personnel	1,375	3,907	6,330

Country	Country code	income level	Occupation	Baseline 2022	Projected 2026	Projected 2030
Eritrea	ERI	Low-Income Economies	Nursing Personnel	5,007	5,707	6,374
Eswatini	SWZ	Lower-Middle-Income Economies	Nursing Personnel	5,242	5,494	5,724
Ethiopia	ETH	Low-Income Economies	Nursing Personnel	131,262	145,487	159,264
Gabon	GAB	High/Upper-Middle-Income Economies	Nursing Personnel	4,752	4,837	4,869
Gambia	GMB	Low-Income Economies	Nursing Personnel	1,429	1,708	1,979
Ghana	GHA	Lower-Middle-Income Economies	Nursing Personnel	124,204	180,889	237,297
Guinea	GIN	Low-Income Economies	Nursing Personnel	5,019	7,558	10,046
Guinea-Bissau	GNB	Low-Income Economies	Nursing Personnel	1,879	2,658	3,416
Kenya	KEN	Lower-Middle-Income Economies	Nursing Personnel	109,659	106,231	102,025
Lesotho	LSO	Lower-Middle-Income Economies	Nursing Personnel	1,235	2,222	3,181
Liberia	LBR	Low-Income Economies	Nursing Personnel	4,424	3,399	2,384
Madagascar	MDG	Low-Income Economies	Nursing Personnel	4,560	11,555	18,423
Malawi	MWI	Low-Income Economies	Nursing Personnel	9,592	10,566	11,511
Mali	MLI	Low-Income Economies	Nursing Personnel	6,327	9,070	11,756
Mauritania	MRT	Lower-Middle-Income Economies	Nursing Personnel	6,503	6,622	6,740
Mauritius	MUS	High/Upper-Middle-Income Economies	Nursing Personnel	4,599	4,640	4,773

Country	Country code	income level	Occupation	Baseline 2022	Projected 2026	Projected 2030
Mozambique	MOZ	Low-Income Economies	Nursing Personnel	17,168	18,509	19,800
Namibia	NAM	High/Upper-Middle-Income Economies	Nursing Personnel	15,558	14,922	14,643
Niger	NER	Low-Income Economies	Nursing Personnel	4,565	5,328	6,071
Nigeria	NGA	Lower-Middle-Income Economies	Nursing Personnel	220,681	239,161	256,251
Rwanda	RWA	Low-Income Economies	Nursing Personnel	13,501	14,262	14,989
Sao Tome and Principe	STP	Lower-Middle-Income Economies	Nursing Personnel	440	526	609
Senegal	SEN	Lower-Middle-Income Economies	Nursing Personnel	4,737	6,168	7,583
Seychelles	SYC	High/Upper-Middle-Income Economies	Nursing Personnel	1,277	1,205	1,218
Sierra Leone	SLE	Low-Income Economies	Nursing Personnel	15,666	19,123	22,496
South Africa	ZAF	High/Upper-Middle-Income Economies	Nursing Personnel	397,261	362,051	342,223
South Sudan	SSD	Low-Income Economies	Nursing Personnel	4,530	5,104	5,661
United Republic of Tanzania	TZA	Lower-Middle-Income Economies	Nursing Personnel	31,940	38,883	45,637
Togo	TGO	Low-Income Economies	Nursing Personnel	2,630	3,028	3,408
Uganda	UGA	Low-Income Economies	Nursing Personnel	74,197	112,694	151,305
Zambia	ZMB	Lower-Middle-Income Economies	Nursing Personnel	49,975	63,868	77,790
Zimbabwe	ZWE	Lower-Middle-Income Economies	Nursing Personnel	46,537	46,718	46,913

Country	Country code	income level	Occupation	Baseline 2022	Projected 2026	Projected 2030
Algeria	DZA	Lower-Middle-Income Economies	Midwifery Personnel	9,070	11,260	13,350
Angola	AGO	Lower-Middle-Income Economies	Midwifery Personnel	33	199	360
Benin	BEN	Lower-Middle-Income Economies	Midwifery Personnel	2,055	2,693	3,322
Botswana	BWA	High/Upper-Middle-Income Economies	Midwifery Personnel	-	100	197
Burkina Faso	BFA	Low-Income Economies	Midwifery Personnel	9,294	12,395	15,428
Burundi	BDI	Low-Income Economies	Midwifery Personnel	179	721	1,256
Cameroon	CMR	Lower-Middle-Income Economies	Midwifery Personnel	1,674	1,685	1,693
Cabo Verde	CPV	Lower-Middle-Income Economies	Midwifery Personnel	27	29	32
Central African Republic	CAF	Low-Income Economies	Midwifery Personnel	537	771	999
Chad	TCD	Low-Income Economies	Midwifery Personnel	904	6,866	12,671
Comoros	COM	Lower-Middle-Income Economies	Midwifery Personnel	586	649	710
Congo	COG	Lower-Middle-Income Economies	Midwifery Personnel	1,042	1,400	1,728
Côte d'Ivoire	CIV	Lower-Middle-Income Economies	Midwifery Personnel	7,425	8,053	8,595
Democratic Republic of the Congo	COD	Low-Income Economies	Midwifery Personnel	3,039	14,503	25,765
Equatorial Guinea	GNQ	High/Upper-Middle-Income Economies	Midwifery Personnel	26	2,627	5,087
Eritrea	ERI	Low-Income Economies	Midwifery Personnel	67	85	103

Country	Country code	income level	Occupation	Baseline 2022	Projected 2026	Projected 2030
Eswatini	SWZ	Lower-Middle-Income Economies	Midwifery Personnel	14	235	448
Ethiopia	ETH	Low-Income Economies	Midwifery Personnel	34,541	40,462	46,227
Gabon	GAB	High/Upper-Middle-Income Economies	Midwifery Personnel	920	980	1,027
Gambia	GMB	Low-Income Economies	Midwifery Personnel	379	487	593
Ghana	GHA	Lower-Middle-Income Economies	Midwifery Personnel	23,882	36,843	49,743
Guinea	GIN	Low-Income Economies	Midwifery Personnel	893	2,236	3,555
Guinea-Bissau	GNB	Low-Income Economies	Midwifery Personnel	197	279	358
Kenya	KEN	Lower-Middle-Income Economies	Midwifery Personnel	137	145	151
Lesotho	LSO	Lower-Middle-Income Economies	Midwifery Personnel	2,779	2,848	2,915
Liberia	LBR	Low-Income Economies	Midwifery Personnel	989	1,074	1,156
Madagascar	MDG	Low-Income Economies	Midwifery Personnel	3,994	10,811	17,506
Malawi	MWI	Low-Income Economies	Midwifery Personnel	616	3,877	7,109
Mali	MLI	Low-Income Economies	Midwifery Personnel	1,920	3,345	4,743
Mauritania	MRT	Lower-Middle-Income Economies	Midwifery Personnel	1,340	1,456	1,568
Mauritius	MUS	High/Upper-Middle-Income Economies	Midwifery Personnel	567	623	687
Mozambique	MOZ	Low-Income Economies	Midwifery Personnel	13,384	13,561	13,714

Country	Country code	income level	Occupation	Baseline 2022	Projected 2026	Projected 2030
Namibia	NAM	High/Upper-Middle-Income Economies	Midwifery Personnel	17	3,893	7,558
Niger	NER	Low-Income Economies	Midwifery Personnel	825	1,193	1,553
Nigeria	NGA	Lower-Middle-Income Economies	Midwifery Personnel	146,787	136,595	126,438
Rwanda	RWA	Low-Income Economies	Midwifery Personnel	1,990	2,446	2,892
Sao Tome and Principe	STP	Lower-Middle-Income Economies	Midwifery Personnel	25	36	46
Senegal	SEN	Lower-Middle-Income Economies	Midwifery Personnel	3,281	3,498	3,718
Seychelles	SYC	High/Upper-Middle-Income Economies	Midwifery Personnel	179	181	194
Sierra Leone	SLE	Low-Income Economies	Midwifery Personnel	2,271	2,398	2,520
South Africa	ZAF	High/Upper-Middle-Income Economies	Midwifery Personnel	-	-	-
South Sudan	SSD	Low-Income Economies	Midwifery Personnel	3,020	3,810	4,582
United Republic of Tanzania	TZA	Lower-Middle-Income Economies	Midwifery Personnel	-	-	-
Togo	TGO	Low-Income Economies	Midwifery Personnel	1,864	2,035	2,195
Uganda	UGA	Low-Income Economies	Midwifery Personnel	32,957	57,805	82,676
Zambia	ZMB	Lower-Middle-Income Economies	Midwifery Personnel	9,202	14,464	19,713
Zimbabwe	ZWE	Lower-Middle-Income Economies	Midwifery Personnel	9,601	9,758	9,914
Algeria	DZA	Lower-Middle-Income Economies	Dentist	16,222	15,263	15,418

Country	Country code	income level	Occupation	Baseline 2022	Projected 2026	Projected 2030
Angola	AGO	Lower-Middle-Income Economies	Dentist	254	1,978	2,297
Benin	BEN	Lower-Middle-Income Economies	Dentist	50	89	126
Botswana	BWA	High/Upper-Middle-Income Economies	Dentist	99	116	131
Burkina Faso	BFA	Low-Income Economies	Dentist	40	189	286
Burundi	BDI	Low-Income Economies	Dentist	14	25	36
Cameroon	CMR	Lower-Middle-Income Economies	Dentist	317	502	684
Cabo Verde	CPV	Lower-Middle-Income Economies	Dentist	12	93	104
Central African Republic	CAF	Low-Income Economies	Dentist	3	13	15
Chad	TCD	Low-Income Economies	Dentist	21	32	43
Comoros	COM	Lower-Middle-Income Economies	Dentist	32	37	41
Congo	COG	Lower-Middle-Income Economies	Dentist	10	30	33
Côte d'Ivoire	CIV	Lower-Middle-Income Economies	Dentist	536	553	561
Democratic Republic of the Congo	COD	Low-Income Economies	Dentist	451	603	753
Equatorial Guinea	GNQ	High/Upper-Middle-Income Economies	Dentist	-	-	-
Eritrea	ERI	Low-Income Economies	Dentist	67	293	401
Eswatini	SWZ	Lower-Middle-Income Economies	Dentist	50	84	93

Country	Country code	income level	Occupation	Baseline 2022	Projected 2026	Projected 2030
Ethiopia	ETH	Low-Income Economies	Dentist	3,522	3,811	4,132
Gabon	GAB	High/Upper-Middle-Income Economies	Dentist	44	41	39
Gambia	GMB	Low-Income Economies	Dentist	9	10	12
Ghana	GHA	Lower-Middle-Income Economies	Dentist	244	1,333	1,929
Guinea	GIN	Low-Income Economies	Dentist	39	309	534
Guinea-Bissau	GNB	Low-Income Economies	Dentist	20	32	43
Kenya	KEN	Lower-Middle-Income Economies	Dentist	1,344	1,425	1,498
Lesotho	LSO	Lower-Middle-Income Economies	Dentist	84	164	169
Liberia	LBR	Low-Income Economies	Dentist	8	14	14
Madagascar	MDG	Low-Income Economies	Dentist	251	389	411
Malawi	MWI	Low-Income Economies	Dentist	101	115	133
Mali	MLI	Low-Income Economies	Dentist	30	45	60
Mauritania	MRT	Lower-Middle-Income Economies	Dentist	149	170	191
Mauritius	MUS	High/Upper-Middle-Income Economies	Dentist	414	635	837
Mozambique	MOZ	Low-Income Economies	Dentist	348	795	1,239
Namibia	NAM	High/Upper-Middle-Income Economies	Dentist	224	381	524

Country	Country code	income level	Occupation	Baseline 2022	Projected 2026	Projected 2030
Niger	NER	Low-Income Economies	Dentist	28	65	102
Nigeria	NGA	Lower-Middle-Income Economies	Dentist	5,048	6,072	7,139
Rwanda	RWA	Low-Income Economies	Dentist	236	225	218
Sao Tome and Principe	STP	Lower-Middle-Income Economies	Dentist	6	7	8
Senegal	SEN	Lower-Middle-Income Economies	Dentist	223	389	540
Seychelles	SYC	High/Upper-Middle-Income Economies	Dentist	44	41	39
Sierra Leone	SLE	Low-Income Economies	Dentist	15	22	29
South Africa	ZAF	High/Upper-Middle-Income Economies	Dentist	1,976	7,954	9,362
South Sudan	SSD	Low-Income Economies	Dentist	32	48	64
United Republic of Tanzania	TZA	Lower-Middle-Income Economies	Dentist	755	942	1,124
Togo	TGO	Low-Income Economies	Dentist	22	26	29
Uganda	UGA	Low-Income Economies	Dentist	504	584	666
Zambia	ZMB	Lower-Middle-Income Economies	Dentist	306	296	301
Zimbabwe	ZWE	Lower-Middle-Income Economies	Dentist	201	425	649
Algeria	DZA	Lower-Middle-Income Economies	Pharmacist	13,642	23,449	32,607
Angola	AGO	Lower-Middle-Income Economies	Pharmacist	1,434	3,146	3,956

Country	Country code	income level	Occupation	Baseline 2022	Projected 2026	Projected 2030
Benin	BEN	Lower-Middle-Income Economies	Pharmacist	382	773	1,158
Botswana	BWA	High/Upper-Middle-Income Economies	Pharmacist	144	378	597
Burkina Faso	BFA	Low-Income Economies	Pharmacist	591	2,246	3,887
Burundi	BDI	Low-Income Economies	Pharmacist	124	230	336
Cameroon	CMR	Lower-Middle-Income Economies	Pharmacist	602	971	1,340
Cabo Verde	CPV	Lower-Middle-Income Economies	Pharmacist	29	271	367
Central African Republic	CAF	Low-Income Economies	Pharmacist	3	64	91
Chad	TCD	Low-Income Economies	Pharmacist	188	479	689
Comoros	COM	Lower-Middle-Income Economies	Pharmacist	40	63	86
Congo	COG	Lower-Middle-Income Economies	Pharmacist	32	236	303
Côte d'Ivoire	CIV	Lower-Middle-Income Economies	Pharmacist	1,491	3,401	4,300
Democratic Republic of the Congo	COD	Low-Income Economies	Pharmacist	928	2,157	2,624
Equatorial Guinea	GNQ	High/Upper-Middle-Income Economies	Pharmacist	14	23	32
Eritrea	ERI	Low-Income Economies	Pharmacist	229	806	1,160
Eswatini	SWZ	Lower-Middle-Income Economies	Pharmacist	60	394	503
Ethiopia	ETH	Low-Income Economies	Pharmacist	22,344	23,943	25,575

Country	Country code	income level	Occupation	Baseline 2022	Projected 2026	Projected 2030
Gabon	GAB	High/Upper-Middle-Income Economies	Pharmacist	187	176	170
Gambia	GMB	Low-Income Economies	Pharmacist	4	138	197
Ghana	GHA	Lower-Middle-Income Economies	Pharmacist	5,736	6,535	7,259
Guinea	GIN	Low-Income Economies	Pharmacist	255	566	873
Guinea-Bissau	GNB	Low-Income Economies	Pharmacist	2	23	33
Kenya	KEN	Lower-Middle-Income Economies	Pharmacist	1,337	2,391	3,439
Lesotho	LSO	Lower-Middle-Income Economies	Pharmacist	257	445	490
Liberia	LBR	Low-Income Economies	Pharmacist	170	229	287
Madagascar	MDG	Low-Income Economies	Pharmacist	30	565	808
Malawi	MWI	Low-Income Economies	Pharmacist	123	686	977
Mali	MLI	Low-Income Economies	Pharmacist	1,721	3,045	4,357
Mauritania	MRT	Lower-Middle-Income Economies	Pharmacist	97	153	206
Mauritius	MUS	High/Upper-Middle-Income Economies	Pharmacist	41	1,229	1,696
Mozambique	MOZ	Low-Income Economies	Pharmacist	498	884	1,267
Namibia	NAM	High/Upper-Middle-Income Economies	Pharmacist	655	1,248	1,814
Niger	NER	Low-Income Economies	Pharmacist	49	87	125

Country	Country code	income level	Occupation	Baseline 2022	Projected 2026	Projected 2030
Nigeria	NGA	Lower-Middle-Income Economies	Pharmacist	21,006	33,130	44,797
Rwanda	RWA	Low-Income Economies	Pharmacist	886	1,137	1,387
Sao Tome and Principe	STP	Lower-Middle-Income Economies	Pharmacist	37	58	79
Senegal	SEN	Lower-Middle-Income Economies	Pharmacist	352	811	1,244
Seychelles	SYC	High/Upper-Middle-Income Economies	Pharmacist	70	163	203
Sierra Leone	SLE	Low-Income Economies	Pharmacist	193	382	569
South Africa	ZAF	High/Upper-Middle-Income Economies	Pharmacist	17,703	19,127	21,040
South Sudan	SSD	Low-Income Economies	Pharmacist	160	637	911
United Republic of Tanzania	TZA	Lower-Middle-Income Economies	Pharmacist	2,262	3,568	4,824
Togo	TGO	Low-Income Economies	Pharmacist	255	418	580
Uganda	UGA	Low-Income Economies	Pharmacist	1,712	2,020	2,327
Zambia	ZMB	Lower-Middle-Income Economies	Pharmacist	1,425	2,265	3,097
Zimbabwe	ZWE	Lower-Middle-Income Economies	Pharmacist	1,902	2,336	2,770
Algeria	DZA	Lower-Middle-Income Economies	Other Health Workers	366,964	437,404	504,409
Angola	AGO	Lower-Middle-Income Economies	Other Health Workers	58,067	63,452	68,704
Benin	BEN	Lower-Middle-Income Economies	Other Health Workers	13,973	15,338	16,707

Country	Country code	income level	Occupation	Baseline 2022	Projected 2026	Projected 2030
Botswana	BWA	High/Upper-Middle-Income Economies	Other Health Workers	8,210	9,306	10,340
Burkina Faso	BFA	Low-Income Economies	Other Health Workers	10,576	12,867	15,102
Burundi	BDI	Low-Income Economies	Other Health Workers	42,120	59,730	77,149
Cameroon	CMR	Lower-Middle-Income Economies	Other Health Workers	8,354	11,194	13,982
Cabo Verde	CPV	Lower-Middle-Income Economies	Other Health Workers	2,476	2,662	2,930
Central African Republic	CAF	Low-Income Economies	Other Health Workers	4,354	6,247	8,101
Chad	TCD	Low-Income Economies	Other Health Workers	33,924	114,250	192,332
Comoros	COM	Lower-Middle-Income Economies	Other Health Workers	754	838	920
Congo	COG	Lower-Middle-Income Economies	Other Health Workers	4,346	4,272	4,154
Côte d'Ivoire	CIV	Lower-Middle-Income Economies	Other Health Workers	37,741	42,023	45,824
Democratic Republic of the Congo	COD	Low-Income Economies	Other Health Workers	15,905	24,889	33,694
Equatorial Guinea	GNQ	High/Upper-Middle-Income Economies	Other Health Workers	2,902	8,247	13,360
Eritrea	ERI	Low-Income Economies	Other Health Workers	7,859	8,958	10,005
Eswatini	SWZ	Lower-Middle-Income Economies	Other Health Workers	10,642	11,153	11,620
Ethiopia	ETH	Low-Income Economies	Other Health Workers	188,294	208,700	228,463
Gabon	GAB	High/Upper-Middle-Income Economies	Other Health Workers	6,195	6,307	6,349

Country	Country code	income level	Occupation	Baseline 2022	Projected 2026	Projected 2030
Gambia	GMB	Low-Income Economies	Other Health Workers	7,047	8,420	9,758
Ghana	GHA	Lower-Middle-Income Economies	Other Health Workers	77,240	112,491	147,569
Guinea	GIN	Low-Income Economies	Other Health Workers	18,091	27,244	36,211
Guinea-Bissau	GNB	Low-Income Economies	Other Health Workers	6,479	9,166	11,779
Kenya	KEN	Lower-Middle-Income Economies	Other Health Workers	153,550	148,750	142,860
Lesotho	LSO	Lower-Middle-Income Economies	Other Health Workers	23,691	42,614	60,997
Liberia	LBR	Low-Income Economies	Other Health Workers	23,098	17,746	12,450
Madagascar	MDG	Low-Income Economies	Other Health Workers	50,968	129,150	205,915
Malawi	MWI	Low-Income Economies	Other Health Workers	26,224	28,887	31,470
Mali	MLI	Low-Income Economies	Other Health Workers	23,767	34,070	44,160
Mauritania	MRT	Lower-Middle-Income Economies	Other Health Workers	11,463	11,672	11,880
Mauritius	MUS	High/Upper-Middle-Income Economies	Other Health Workers	7,398	7,464	7,679
Mozambique	MOZ	Low-Income Economies	Other Health Workers	139,367	150,250	160,730
Namibia	NAM	High/Upper-Middle-Income Economies	Other Health Workers	8,448	8,103	7,951
Niger	NER	Low-Income Economies	Other Health Workers	6,818	7,958	9,068
Nigeria	NGA	Lower-Middle-Income Economies	Other Health Workers	303,917	329,368	352,903

Country	Country code	income level	Occupation	Baseline 2022	Projected 2026	Projected 2030
Rwanda	RWA	Low-Income Economies	Other Health Workers	63,680	67,269	70,698
Sao Tome and Principe	STP	Lower-Middle-Income Economies	Other Health Workers	828	989	1,146
Senegal	SEN	Lower-Middle-Income Economies	Other Health Workers	29,940	38,983	47,926
Seychelles	SYC	High/Upper-Middle-Income Economies	Other Health Workers	594	561	567
Sierra Leone	SLE	Low-Income Economies	Other Health Workers	18,899	23,069	27,139
South Africa	ZAF	High/Upper-Middle-Income Economies	Other Health Workers	191,863	174,857	165,281
South Sudan	SSD	Low-Income Economies	Other Health Workers	12,908	14,544	16,132
United Republic of Tanzania	TZA	Lower-Middle-Income Economies	Other Health Workers	138,070	168,084	197,280
Togo	TGO	Low-Income Economies	Other Health Workers	21,202	24,414	27,472
Uganda	UGA	Low-Income Economies	Other Health Workers	215,144	326,772	438,729
Zambia	ZMB	Lower-Middle-Income Economies	Other Health Workers	42,024	53,707	65,413
Zimbabwe	ZWE	Lower-Middle-Income Economies	Other Health Workers	42,581	42,747	42,925
