# Supplementary material for: Projected health workforce requirements and shortage for addressing the disease burden in the WHO Africa Region, 2022–2030: a needs-based modelling study
Source: BMJ Glob Health. 2024 Oct 22;7(Suppl 1):e015972. doi: 10.1136/bmjgh-2024-015972 (PMC11789529; doi:10.1136/bmjgh-2024-015972)
Supplement: online supplemental material 6 [file bmjgh-7-Suppl_1-s006.pdf]

Country	Density per 10,000 population (All Occupations)_Base Scenario in 2022	Neonates (0-28 days)	Under five years	Older Children (5 - 14)	Older Children (5 - 9)	Older Children (10 - 14)	Adolecents (15 - 19)	Adults (20 - 64)	Adolecents and adults (15 - 64)	Adolecents and adults (15 - 49)	Adults (50+)	Aged (65+)
Madagascar	89.64	2.0%	14.8%	25.2%	13.2%	12.0%	10.9%	46.0%	56.8%	49.4%	10.6%	3.1%
Gambia	91.34	2.0%	16.9%	27.0%	14.6%	12.4%	10.6%	42.9%	53.5%	47.3%	8.7%	2.5%
Eritrea	93.73	2.0%	14.0%	27.2%	13.4%	13.7%	10.5%	43.8%	54.4%	47.6%	11.3%	4.5%
Malawi	94.59	2.0%	15.3%	27.7%	14.3%	13.4%	11.4%	43.0%	54.4%	48.5%	8.5%	2.6%
Zimbabwe	95.51	2.0%	14.1%	27.8%	14.9%	12.9%	11.0%	44.0%	55.1%	48.8%	9.3%	3.0%
United Republic of Tanzania	95.62	2.0%	16.3%	27.3%	14.4%	12.8%	10.8%	43.0%	53.8%	47.3%	9.1%	2.6%
Guinea	97.16	2.0%	16.0%	27.1%	14.2%	12.9%	11.5%	42.5%	54.0%	47.6%	9.4%	3.0%
Kenya	98.36	2.0%	13.1%	25.5%	13.0%	12.5%	11.2%	47.7%	58.9%	52.0%	9.4%	2.5%
Nigeria	98.99	2.0%	16.5%	27.0%	14.5%	12.5%	10.6%	43.1%	53.8%	46.8%	9.7%	2.7%
Ghana	99.19	2.0%	13.4%	23.7%	12.5%	11.2%	10.1%	49.6%	59.7%	50.7%	12.2%	3.1%
Sierra Leone	99.74	2.0%	14.5%	25.8%	13.4%	12.4%	11.0%	45.8%	56.7%	49.8%	9.9%	2.9%
Angola	100.82	2.0%	17.6%	28.8%	15.5%	13.2%	10.7%	40.7%	51.4%	45.5%	8.1%	2.2%
Côte d'Ivoire	100.85	2.0%	15.7%	25.8%	13.7%	12.1%	11.0%	44.6%	55.6%	48.7%	9.8%	2.9%
South Sudan	101.34	2.0%	15.3%	26.1%	13.7%	12.3%	10.7%	44.6%	55.3%	48.2%	10.5%	3.4%
Zambia	101.45	2.0%	16.0%	28.0%	14.8%	13.2%	11.5%	42.3%	53.9%	48.4%	7.6%	2.1%
Uganda	101.67	2.0%	17.0%	29.0%	15.4%	13.5%	11.5%	40.5%	52.0%	46.7%	7.3%	2.0%
Mozambique	101.79	2.0%	16.5%	27.6%	14.6%	13.0%	11.3%	41.7%	53.1%	47.1%	8.9%	2.9%
Comoros	102.63	2.0%	14.2%	24.8%	13.1%	11.7%	10.3%	47.6%	57.9%	50.0%	11.0%	3.1%
Cameroon	102.66	2.0%	15.5%	26.6%	14.1%	12.5%	10.8%	44.5%	55.2%	48.8%	9.2%	2.7%
Eswatini	103.58	2.0%	12.4%	25.1%	12.3%	12.7%	11.1%	47.4%	58.5%	52.4%	10.2%	4.0%
Democratic Republic of the Congo	103.69	2.0%	17.7%	28.1%	15.3%	12.9%	10.6%	40.6%	51.2%	44.7%	9.5%	3.0%
Sao Tome and Principe	104.41	2.0%	14.5%	27.3%	14.0%	13.3%	11.4%	43.8%	55.2%	47.4%	10.8%	3.0%
Niger	105.13	2.0%	19.8%	29.9%	16.4%	13.5%	10.9%	36.8%	47.7%	41.9%	8.4%	2.6%
Lesotho	105.45	2.0%	11.8%	20.4%	10.5%	9.9%	10.1%	52.7%	62.8%	53.7%	14.1%	4.9%
Mali	105.66	2.0%	17.8%	29.2%	15.6%	13.6%	11.1%	39.4%	50.5%	44.9%	8.1%	2.5%
Guinea-Bissau	105.79	2.0%	15.5%	26.4%	14.1%	12.3%	10.5%	44.7%	55.2%	48.8%	9.3%	2.9%
Benin	106.53	2.0%	15.7%	26.2%	13.9%	12.3%	10.7%	44.1%	54.8%	47.6%	10.4%	3.3%
Burkina Faso	107.60	2.0%	16.6%	27.8%	14.7%	13.0%	11.0%	42.2%	53.2%	47.0%	8.6%	2.4%
Cabo Verde	107.84	2.0%	9.3%	18.7%	9.6%	9.2%	8.8%	58.3%	67.1%	55.7%	16.2%	4.8%
Congo	109.03	2.0%	14.9%	26.4%	14.0%	12.4%	10.4%	45.6%	56.0%	48.3%	10.5%	2.8%
Equatorial Guinea	109.70	2.0%	14.2%	22.5%	12.3%	10.3%	8.6%	52.2%	60.8%	55.0%	8.2%	2.4%
Chad	110.56	2.0%	17.8%	28.6%	15.4%	13.3%	11.3%	39.7%	51.0%	45.6%	7.9%	2.5%
Senegal	110.79	2.0%	15.6%	27.0%	14.4%	12.5%	10.5%	43.8%	54.3%	47.6%	9.8%	3.1%
Togo	111.52	2.0%	14.7%	25.9%	13.5%	12.4%	10.8%	45.7%	56.5%	49.2%	10.1%	2.9%
Botswana	111.89	2.0%	11.6%	21.8%	11.4%	10.5%	9.6%	52.5%	62.1%	53.3%	13.3%	4.5%
Rwanda	112.45	2.0%	14.6%	24.9%	12.9%	12.0%	10.5%	47.0%	57.4%	49.8%	10.7%	3.1%
Ethiopia	115.31	2.0%	14.6%	25.3%	13.3%	12.1%	11.3%	45.3%	56.5%	49.8%	10.3%	3.5%
Gabon	116.68	2.0%	14.4%	22.9%	12.6%	10.3%	8.7%	50.5%	59.2%	51.4%	11.4%	3.5%
Central African Republic	117.46	2.0%	15.3%	28.3%	14.4%	13.8%	12.4%	41.3%	53.7%	47.2%	9.3%	2.8%
Burundi	119.04	2.0%	17.3%	28.0%	15.3%	12.7%	10.2%	42.1%	52.4%	46.5%	8.2%	2.4%
Algeria	119.09	2.0%	11.5%	19.3%	10.6%	8.7%	6.6%	55.8%	62.5%	50.6%	18.6%	6.7%
Liberia	121.28	2.0%	14.6%	25.7%	13.4%	12.3%	10.9%	45.4%	56.3%	48.8%	10.9%	3.3%
South Africa	125.13	2.0%	9.7%	19.1%	9.8%	9.3%	8.3%	57.4%	65.7%	54.4%	16.8%	5.5%
Mauritania	126.02	2.0%	14.8%	24.8%	13.3%	11.6%	10.1%	47.0%	57.1%	49.5%	10.8%	3.2%
Namibia	127.23	2.0%	13.2%	23.6%	12.5%	11.1%	9.6%	49.9%	59.6%	51.6%	11.5%	3.6%
Seychelles	137.97	2.0%	8.1%	15.7%	8.1%	7.6%	6.5%	61.6%	68.2%	50.0%	26.2%	8.1%
Mauritius	166.03	2.0%	5.0%	11.7%	5.4%	6.3%	7.4%	63.3%	70.7%	51.0%	32.2%	12.5%
Regional Average	108.30	2.0%	14.6%	25.3%	13.3%	12.0%	10.4%	46.3%	56.6%	49.0%	11.1%	3.5%
Lowest	89.64	2.0%	5.0%	11.7%	5.4%	6.3%	6.5%	36.8%	47.7%	41.9%	7.3%	2.0%
Highest	166.03	2.0%	19.8%	29.9%	16.4%	13.8%	12.4%	63.3%	70.7%	55.7%	32.2%	12.5%
